# Sugar-sweetened beverages coverage in the British media: an analysis of public health advocacy versus pro-industry messaging

**DOI:** 10.1136/bmjopen-2016-011295

**Published:** 2016-07-19

**Authors:** Alex Elliott-Green, Lirije Hyseni, Ffion Lloyd-Williams, Helen Bromley, Simon Capewell

**Affiliations:** University of Liverpool, Liverpool, Merseyside, UK

**Keywords:** PUBLIC HEALTH, Advocacy, Sugar Sweetened Beverages, Media, Food Industry, Soda

## Abstract

**Objectives:**

To assess the extent of media-based public health advocacy versus pro-industry messaging regarding sugar-sweetened beverages (SSBs).

**Design:**

We conducted a systematic analysis to identify and examine all articles regarding SSBs published in all mainstream British print newspapers and their online news websites from 1 January 2014 to 31 December 2014. We initially conducted a brief literature search to develop appropriate search terms and categorisations for grouping and analysing the articles. Articles were then coded according to the publishing newspaper, article type, topic, prominence and slant (pro-SSB or anti-SSB). A contextual analysis was undertaken to examine key messages in the articles.

**Results:**

We identified 374 articles published during 2014. The majority of articles (81%) suggested that SSBs are unhealthy. Messaging from experts, campaign groups and health organisations was fairly consistent about the detrimental effects of SSB on health. However, relatively few articles assessed any approaches or solutions to potentially combat the problems associated with SSBs. Only one-quarter (24%) suggested any policy change. Meanwhile, articles concerning the food industry produced consistent messages emphasising consumer choice and individual responsibility for making choices regarding SSB consumption, and promoting and advertising their products. The food industry thus often managed to avoid association with the negative press that their products were receiving.

**Conclusions:**

SSBs were frequently published in mainstream British print newspapers and their online news websites during 2014. Public health media advocacy was prominent throughout, with a growing consensus that sugary drinks are bad for people's health. However, the challenge for public health will be to mobilise supportive public opinion to help implement effective regulatory policies. Only then will our population's excess consumption of SSBs come under control.

**Strengths and limitations of this study**

- Systematic analysis of media coverage relating to sugar-sweetened beverages for one calendar year.
- Inclusion of online newspapers as well as print versions.
- Only examined media articles in mainstream national newspapers.
- Study only comprised coverage from January to December 2014; further analysis of developments in sugar policy during 2015 and 2016 is recommended.

## Introduction

One of the main causes for increasing obesity rates is excessive consumption of sugar. The UK Scientific Advisory Committee on Nutrition (SACN) concluded that drinking high-sugar beverages results in weight gain and increased body mass index in teenagers and children and increases the risk of developing type 2 diabetes independently of obesity.^1^

UK and global populations are consuming increasing amounts of sugary drinks and junk foods (high in salt, sugar and saturated fats).^2^ The UK has been progressing towards a tax on sugar-sweetened beverages (SSBs). Evidence from all recent scientific reports supports substantial reductions in sugar consumption.^1^ ^3–6^ On the basis of this evidence, Public Health England, SACN and the WHO recently recommended reducing the intake of free sugars, initially to <10% of total energy intake in adults and children, and then to <5% of total energy intake.^1^ ^3^ ^6^ On 6 March 2016, the Chancellor included in his Budget an announcement of a levy on sugary drinks manufacturers equivalent to a 10% excise tax. However, implementation would be delayed until 2018 to allow the industry time to respond and reformulate.^7^

SSBs provide a substantial proportion of the UK population's sugar intake, particularly in children and adolescents.^8^ ^9^ SSBs thus contribute importantly to obesity, diabetes and cardiovascular disease (CVD) and therefore represent an increasing public health concern.^10–13^ The persistent high consumption rates of free sugars in SSBs highlight a pressing need to evaluate which factors determine and promote their use.^12^ Public health has had a chequered career in highlighting the potential harms of sugar, particularly the free sugars in SSBs. The first notable communicator, John Yudkin, published ‘Pure White and Deadly’ in 1972.^14^ Several decades of low-level advocacy, mostly by dentists then followed. However, this started to change from 2009 onwards, after Robert Lustig's student lecture on fructose became a YouTube hit. Subsequent advocacy and activism in the USA and UK initially increased slowly, but then accelerated.^1^ However, effective policies to reduce SSBs remain limited.

One possible explanation for this may be multinational corporations' concerns that negative publicity regarding the health harms of sugar may threaten profits. Thus, the food industry, for instance, has made considerable efforts to try to neutralise any media coverage of adverse research. These strategies show remarkable similarities to the tactics previously used by Big Tobacco corporations.^15–18^ They include creating doubt regarding scientific studies or scientists who criticise the industry, political lobbying, criticising potential policies as ‘nanny state’ or a threat to personal choice, emphasising personal responsibility and emphasising physical activity rather than curtailing consumption as a remedy for obesity.^15^ ^19^

The media potentially play an important role in communicating such information to the wider public. This messaging will thus be subject to targeting by industry and by health groups. The public health community, therefore, have a potentially big role in agenda-setting (the theory that what the public think is set by the media)^20^ and subsequent policy development for population health.^21–24^ It is hence important to analyse how public health advocacy and food industry messages are framed (defining something as a problem, identifying causes, assignment responsibility and suggesting/endorsing solutions)^25^ in the media. This study therefore aims to examine coverage of SSBs in British print and online media, and assess the extent of public health advocacy versus pro-industry messaging.^26^ The findings may help to inform future strategies aiming to reduce SSB intake.

## Methods

We conducted a systematic analysis of news articles focusing on SSBs published in the major national print and web editions of British newspapers.

### Piloting and selection of search terms

Search terms were originally identified by examining a small sample of newspaper articles and evaluating terminology and phrases that were commonly employed with regard to this SSB. These search terms were then piloted over a 1-month period and the results were analysed for material and contents. As a result of the pilot study, key terms were identified and search terms were purposefully kept broad to ensure the inclusion of a large variety of articles covering this topic.

Articles were sourced via the Nexis database using the following search terms: Sugar* W/5 beverage* (Sugar* and followed by beverage maximum of five words later); Sugar* W/p soft drink* (Sugar* within the same paragraph soft drink*); Fizz* Drink*; Sugar* drink*.

Other synonyms were piloted such as ‘soda’, ‘sugar-sweetened beverages’ and ‘pop’. However, it was noted that ‘soda’ and ‘pop’ were highly unspecific towards the topic and articles in which these terms were used. Conversely, ‘sugar-sweetened beverages’ was too specific and many articles were being overlooked using the search term. Using the search term ‘Sugar* W/5 beverage*’ ensured a large yield of relevant articles.

### Study design and search strategy

**Figure 1:**
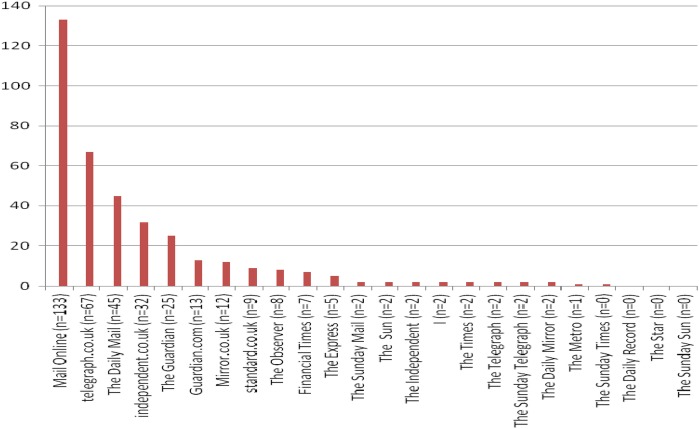
Number of articles on SSBs published in UK. (SSB, sugar-sweetened beverage).

We conducted a systematic analysis of articles focusing on SSBs in the major national print and online websites of British newspapers (figure 1). Articles produced between 1 January 2014 and 31 December 2014 were considered for inclusion (table 1). Flexible search terms were used to include all potentially relevant articles relating to SSBs including fruit juices, sugar or a synonym of SSBs, for example, fizzy drinks, and the industry.

**Table 1:** Key themes covered by the British press and the association between slant, cause and proposed solutions

		Slant on sugar			Slant on industry			Suggested causes			Proposed solutions		
Themes	No.	Good	Bad	Neutral	Good	Bad	Neutral	Individual	Policy	Industry	Individuals	Policy	Industry
Health effects	94	2	93	1	1	6	87	15	1	6	36	31	
Obesity	22		22			2	22	8			9	7	3
Advertisement and promotion	34		13	21	11	9	14	2	2	5	6	4	6
Food industry/companies	57		20	37	9	15	33			3	3	1	5
Youth consumption	29		29			3	26	8			9	7	3
Product consumption	57		54	3		6	51	5	4		24	7	10
Regulation	64		60	4	5	2	57	5	1	6	2	46	7
Other	17		14	3		8	9	2	1	3	3	4	2

Ovid, Embase and MEDLINE databases were also searched to provide supplementary data and related articles to the topic which informed study design and strategy.

The time period for this study was discussed with other experts and academics in the field who felt that it was necessary to assess articles over a 1-year period to gather a wide scope of media representation on this topic. The period 1 January 2014 to 31 December 2014 was selected as this was seen as a crucial time with regard to public health advocacy on sugary drinks. There was increasing debate and support for a sugary drinks tax, reflected in an increase in media coverage.^27^ ^28^ This enabled the tracking of movement and response by the food industry.

### Article selection and inclusion criteria

Articles were screened for eligibility using the following specific inclusion criteria: (1) SSBs or a synonym and (2) have at least one paragraph relating to the regulation of sugar or the food industry. Articles were excluded if they focused on artificial sweetener or represented unpublished articles and non-UK editions.

The inclusion criterion was kept specifically broad to ensure that all relevant articles were evaluated and included. The articles had to relate to the core subject that was being evaluated: SSBs and the relation of these to public health in terms of regulation, controls or guidance, or the role of sugar within beverages, or the food industry opinion or rhetoric with regard to these products in terms of advertisements.

Articles were double screened and coded by two researchers. One researcher was completely blinded and had no information on the newspapers included and where the articles were published, to reduce potential bias.

### Coding framework and analysis

A coding framework was developed to analyse and categorise the articles obtained.

All articles meeting these criteria were then coded in terms of the newspaper in which they were published, article type and topic focus. Articles were categorised into: News Article; Editorial—Opinion pieces; Letters/Comments written in by the public published; Question and Answer; Other.

Studies were excluded if they were: unpublished editorials, duplicates, a non-British publication, articles that did not discuss sugar, food industry or regulation and articles that focused on artificial sweeteners.

Article prominence was assessed using an adaptation of Pollock's scoring classification, adapted to include analysis of online prominence and weighted articles with more views.^29^ There is currently no consensus within the literature of how to monitor online prominence as online articles have fluidity in terms of presentation. Therefore, online articles were coded by the applicable measures used in Pollock's prominence score. However, as not all of the domains were relevant to online print media, they were not given a score. Online articles were coded for the word count of the article, the number of words in the headline and the initial placement of the article on the website in terms of home page and other.

Slant was measured using framework for coding and analysing media articles on tobacco.^30^ Slant was considered from three perspectives: the article's perspective on sugar and on the food industry; the cause of problems relating to sugar, be it individual, industry or public policy; and what solutions the article presented relating to sugar. A pro-sugar slant was defined as an article which predominantly promoted the benefits of sugar. An antisugar slant was defined as an article which predominantly outlined the detrimental effects of sugar. Articles were coded as neutral if there was a mixed or no opinion on the effects of sugar.

Article slant (pro-SSB or anti-SSB) was analysed to understand some of the complex strategies used by the food industry. Separating the analysis into these three areas provided a more detailed overview of the content and the message of the article. This was independently reviewed by two separate researchers and any discrepancies were resolved by discussion.

#### Contextual analysis

In addition to the coding framework, a contextual analysis was conducted to provide additional information with regard to underlying themes and messages framed in the British press. This method is based on a structure for content analysis applied when reviewing tobacco in the media.^31–34^ Key themes (table 1) relating to media advocacy (the strategic use of mass media to advance public policy initiatives) and food industry opposition were identified and then analysed with regard to their frequency during this period. Many qualitative themes relating to SSBs were extracted, demonstrating the predominant sentiments expressed by actors from public health and the food industry.

## Results

Between January and December 2014, 374 articles from 25 national newspapers met the inclusion criteria (figure 1 and see online supplementary appendix 1). The majority of articles (72%) were from online newspapers, 28% in print. The most common article type was News (57%), followed by Editorials (41%) and Letters (2%). *The Daily Mail* and *Mail Online* provided the most extensive coverage of SSBs, accounting for 36% of all articles analysed (figure 1).

10.1136/bmjopen-2016-011295.supp1 Supplementary appendix

### Topics

SSBs were associated with a large variety of topics and subtopics, most frequently health effects (23% of the total articles analysed) (table 1). Of those articles discussing the health impact of SSBs, the most prominent subtopic was the consumption of SSBs by the youth (18.5%). In contrast, articles that concentrated on food industry issues often featured the recurrent subtopic of advertising and product promotion.

### Slant

**Figure 2:**
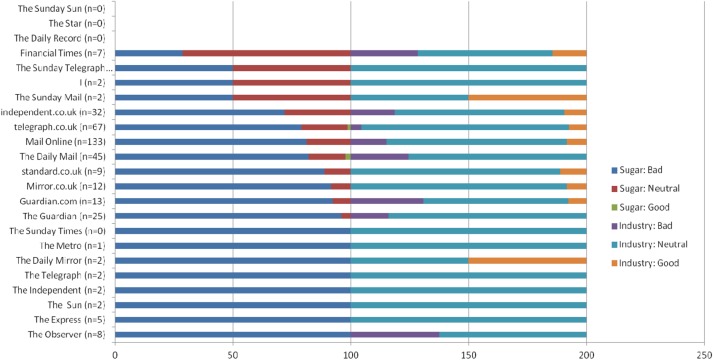
Newspapers with slant on sugar compared to slant on industry (%). (*X*-axis depicts newspapers’ slant on sugar in % 0–100. It also depicts the newspapers’ slant of industry shown as % 100–200).

Sugar was mostly framed as a negative substance across tabloids and broadsheet journals. Irrespective of the main topic, the majority of articles suggested that sugar is ‘bad’ (81%) (eg, *telegraph.co.uk, 22.01.2014 “Banning ‘energy drinks’ from schools: Why not Coke, Sprite and Fanta too?…”; MailOnline, 16.09.2014 “Put sugar tax on unhealthy products to prevent obesity and tooth decay, say scientists”*). Only 14% of articles in British press negatively portrayed the food industry and its influence on promoting sugar, most often the Guardian (21%) or Observer (37%). Most articles portrayed the food industry as neutral (79%) (figure 2).

### Proposed approaches to SSBs

**Figure 3:**
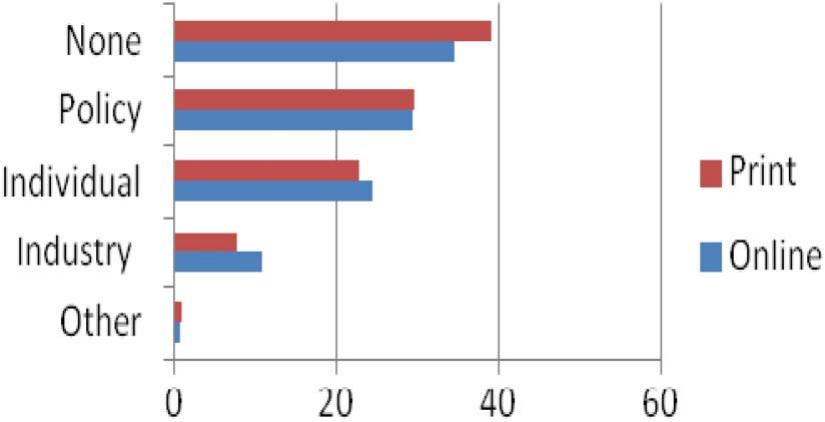
Comparison between online and print newspapers in proposing solutions for SSBs (%). (SSB, sugar-sweetened beverage).

Online newspapers emphasised individual responsibility (ie, the predominant solution presented is for consumers to change their behaviour. For example, *Independent.co.uk, 25/06/14 Drink water to cut obesity, health experts say*) more than print newspapers (12% online vs 9% print). However, online and print newspapers promoted policy changes as a coherent response to the detrimental effect of SSBs to similar extents (31% compared to 30% of print newspapers, figure 3).

**Figure 4:**
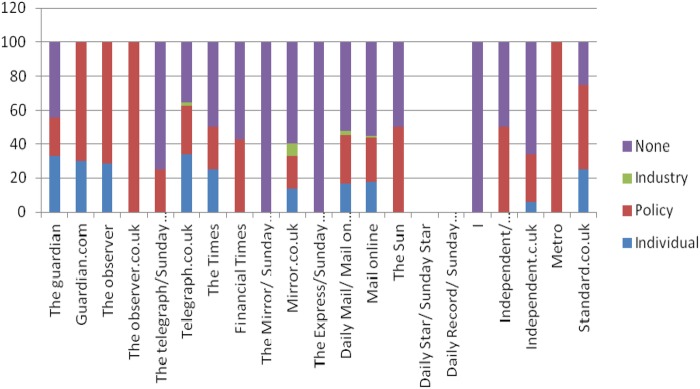
Proposed approaches to SSBs across different newspapers in %. (SSB, sugar-sweetened beverage).

The British press displayed marked heterogeneity regarding perceived causes and solutions to the detrimental impacts of sugar. Although 24% of articles suggested policy changes as a viable solution, 31% placed the responsibility for combating consumption of sugar on individuals and 36% offered no solutions, merely highlighting the problems associated with overconsumption of SSBs (figure 3). Broadsheet newspapers were more likely than tabloid newspapers to propose policy changes besides highlighting individual responsibility (figure 4).

### Contextual analysis

#### Public health media advocacy—health promotion

Three principal ways were established in which SSBs were depicted as detrimental to health. First, obesity was the most discussed health effect throughout the year, particularly in relation to children. Newspapers repeatedly quoted that ‘1 in 3 children in the UK are overweight or obese’. Recommendations and guidance about a reduced dietary intake of sugar were also common. Health organisations, such as WHO and the SACN, reinforced these sentiments throughout this period in online and print newspapers, highlighting the three major effects of SSBs on health.

Second, the link between diabetes and SSBs was often reiterated by experts, for example, by Professor Sattar (Institute of Cardiovascular and Medical Sciences, University of Glasgow) who stated: ‘fruit juices may increase your risk of diabetes’. Campaign groups also emphasised this message throughout 2014, stating ‘Scientific studies have shown that consuming even one sugary drink a day is associated with a significantly increased risk of developing type 2 diabetes even in normal weight people’. Third, the increased risk of CVD is associated with consuming SSBs: ‘one fizzy drink a day was enough to increase the chances of dying from cardiovascular disease by almost a third’. This story was covered by several newspapers and repeated throughout the year.

#### Public health media advocacy—agenda-setting

There was some heterogeneity in proposed actions to tackle SSB-related problems; some newspapers did not cover the issue (figure 4). When solutions were discussed, three main policy options were explored: reformulation of SSBs, the introduction of an SSB tax and changes to labelling or other information provided on SSBs.

Throughout this period, the ‘Responsibility Deal’ and efforts from industry were quoted as a positive development, with an emphasis on voluntarily reducing SSB sugar content. A proportion of articles in early 2014 (n=25) commented on ‘hidden sugar’ within food, with experts and agencies advocating a 30% reduction in sugar intake.

A duty on SSBs, dubbed the ‘sugar tax’, was widely covered throughout this period. Public health experts were already introducing the idea in January 2014; ‘if the responsibility deal is not effective, sugar tax is the next step’. This was initially met by some resistance from newspapers: ‘Taxing sugar would be illiberal and ineffective’. However, as the year progressed, the frequency of sugar tax articles increased. For example, the *telegraph.co.uk (12.07.14)* published an article stating the need for taxation: ‘a sugar tax might not be right but it is necessary and perhaps nanny does know best’. Newspapers also covered wider international progress in the introduction of sugar taxes, for example, in California, describing it as a positive and successful solution.

Although articles covering SSBs describing ‘health warning style labels’ were presented as an alternative to a sugar tax in early 2014, they then largely disappeared.

#### Industry-friendly messaging

Throughout the period of analysis, the British press displayed four industry-friendly approaches in their coverage of SSBs: promoting a positive lifestyle, increasing physical activity, emphasising a balanced diet and promoting socially responsible brands. In the majority of cases (11 of 13), this was promoted by the British press themselves.

First, the promotion of a positive lifestyle was demonstrated by product promotion and advertising conveyed as being exciting and desirable. The promotion of ‘Coca-Cola Life’ illustrated this during its launch with newspaper articles describing female fashion models' dress and sense of style. This brand also used celebrities denoted as ‘sexy’ to advertise their product.

Second, SSB brands tended to emphasise physical activity. Thus, one company was praised for its ‘clever and inventive advert’ suggesting that exercise can ‘burn off’ a can of soft drink in 30 min. The same company promoted an antiobesity campaign across the UK aiming to get families to partake in free exercise in parks. However, these efforts were largely criticised within the British p as being hypocritical and self-promoting.

Third, the food industry tended to repeat messages focused on promoting ‘a healthy balanced diet’. For example, ‘*sugar consumed as part of balanced diets is not a cause of obesity’ (eg, Daily Mail, 09.01.14 “Sugar is the new tobacco”; The Observer, 12.01.14 “DIET—Politicians should stand up to sugar lobby”)*. Other food industry representatives stated ‘*this is about calories in and calories out and getting the balance right*’ *(Daily Mail, 27.05.14 “Health Chiefs slam Coca Cola's £20m Anti-Obesity Stunt”; The Telegraph. 26.06.14 “Coca-Cola in controversy over £20m anti-obesity drive”)*. There was also a denial that SSBs have detrimental health effects, with one claiming ‘*I don't think any of our products are unhealthy’ (Mail Online, 30.08.14/Mail on Sunday, 31.08.14 “Coke Isn't unhealthy, we've spent £15m cutting calories”)*.

Finally, the industry emphasised their positive roles, for example, via their corporate social responsibility strategies. For instance, Tesco launched a programme to reduce childhood obesity by educating children about how food is sourced. Some press framed this as a positive and constructive solution *(Mirror.co.uk 27.01.2014 Children set for ‘food lessons’ as many believe cheese grows on trees and fish fingers contain CHICKEN… Tesco is pumping £15m into a project…)*. Supermarkets also proposed to remove SSBs from premium store locations, including aisle ends and checkouts. These acts were portrayed as positive actions by an industry taking responsibility to safeguard the health of their customers.

## Discussion

This study of recent British press coverage of SSBs highlights three key issues.

First, sugar has recently become a prominent topic in UK media. The British press now largely agrees that sugar is bad for health, particularly in children. This represents a successful communication of public health evidence across the British press.^10^ ^12^ ^35^ A coordinated approach by multiple public health agencies, experts, campaign groups and health organisations has generated coherent messages on the health impacts of SSBs, notably by emphasising the emerging evidence increasingly linking SSBs with obesity, diabetes and CVD and highlighting the specific vulnerability of children. Public health media advocacy has a central role in policy-setting and agenda-setting;^36–38^ this can be fortified by consistent messaging to the British media.

Second, the contested issue of potential solutions and whether policy changes such as labelling, mandatory reformulation and taxation are needed to reduce SSB consumption. The ‘Responsibility Deal’ was launched by the UK government in March 2011. The aim was for the food industry, including food manufacturers, to improve public health by pledging to change products by reducing fat, salt and sugar content.^39^

By June 2014, it was clear that these voluntary agreements had largely failed to reduce sugar content in key products. The concept of reformulation was then largely abandoned and replaced by the concept of an SSB tax. Thus throughout the year, tax had sustained media coverage as a result of public health media advocacy, resulting in increasing prominence, public support and a growing acceptance by the British press.

Although a largely unified approach presented from Action on Sugar, WHO and public health officials was presented to the media, this largely failed to penetrate large sections of the British press, with most articles neglecting to recommend any strategies to control SSBs. Yet the concept of taxation clearly gained momentum throughout this period. Tabloids tended to focus on individual responsibility, whereas broadsheets more often considered the evidence-based approaches of SSB taxation and regulation.^40^ ^41^ Furthermore, as the year progressed, an SSB tax became increasingly accepted as necessary by the British press, following limited progress by the food industry voluntary reformulation.

Finally, Brownell and Warner's^15^ seminal US analysis and, more recently, Nixon *et al*^42^ closely predicted the response of the UK food industry, with industry employing many of the denial tactics previously used by Big Tobacco. The food industry representatives thus tended to promote physical activity, emphasise individual responsibility, advertising ‘light’ or ‘healthier’ products and highlight their companies' social responsibility.^9^ One successful approach was for the industry to generally agree that large amounts of sugar are bad for health, but to simultaneously disassociate their company's specific product from detrimental health effects.

The food industry have tried to deflect the harmful effects of SSBs by focusing on promoting consumption of their products as part of ‘a healthy balanced diet’.^43^ ^44^ However, increasing evidence indicates that SSBs increase the risk of obesity, diabetes and heart disease.^45–48^ However, food companies are able to employ the best marketing agencies and buy multiple and advantageous advertising spots. In contrast, healthy foods can be more expensive and less ubiquitous. Furthermore, healthy foods lack the financial capacity to compete with the marketing strategies of junk food and SSB companies.^49^ ^50^

Junk food and soda taxes are supported by increasing evidence from empirical and modelling studies. The evidence is very strong for a SSB tax. There are empirical data from a number of countries now showing a rapid and substantial benefit (eg, the USA, Norway, Samoa, Fiji, Finland, Hungary and France).^27^ For example, in France, a soda tax was implemented in January 2012 which saw a price increase on drinks with added sugar or sweetener, and consequently sales dropped.^51^ More recently in Mexico, the 10% soda tax implemented in January 2014 has shown considerable positive effects. By December 2014, soda sales were down 12% from December 2013 and sales of bottled water were up by 4%. The drop was greatest among the poorest Mexicans—buying 17% less sweetened soda than the year before.^52^

A systematic review and meta-analysis of the impact of food pricing on dietary consumption indicates significant and positive outcomes. Elasticities are reassuringly consistent: if price is increased by 10% for SSBs, there is a fall in purchasing and consumption by 7% (3–10%). Furthermore, for each 10% decrease in price (subsidy) for healthier food such as fruits or vegetables, consumption is increased by some 14% (11–17%).^53^ Thus, price interventions targeting food and sugary drinks (soda), as proven in other countries, can be powerfully effective.

### Strengths and weaknesses

Our study provides a unique and contemporary analysis looking specifically at the recent framing of SSB issues within the British press. We used a systematic approach to identify, extract and code all relevant newspaper articles besides conducting a contextual analysis. Screening and coding was undertaken by two independent researchers, with one of them blinded to the newspapers. Inclusion of online newspapers was essential, given recent massive cultural changes in how the public access news stories.

It is important to understand how today’s media are framing issues around the growing rate of obesity and the major health impact of SSBs. We believe that this article will inform future action and assist in promoting population health. Such research will also allow us to better understand how the media might influence views and policies on other topics.

This study also has limitations. First, despite efforts to minimise article exclusion, it was not possible to review all major online newspapers within the search undertaken of media articles covering SSBs: the *Sun online*, *Times online* and *BBC.co.uk* were not accessible via Nexis and were therefore not included. While we made substantial effort through other databases and through public relations agents to gain archive access to these three publications, we were unable to obtain consistent, complete and original articles from these online media outlets. One obstacle with regard to *The Sun* and *The Times* was the subscription fee for these papers. In addition, when able to access original published articles, they are not reliably stored within the websites. For instance, many online articles get reshaped several times a day in which the focus and sometime the slant are changed. Without having access to the sequence, it is not possible to evaluate the articles on an equitable basis.

Second, this study examined only media articles in mainstream national newspapers and did not include broadcast media, social media or commercial advertisements.

Third, the inclusion of regulation in the sampling strategy may have influenced the final sample. Stories about, for example, the health benefits of fruit juice were possibly under-represented and stories including public health advocacy were possibly over-represented. This is due to calls for regulation possibly not being included in all articles about SSBs and is more likely to emanate from public health advocates.

Finally, the study comprised coverage only from January to December 2014. With the UK government announcing in their March 2016 budget that a tax on sugary drinks will be implemented in 2018,^7^ a further analysis will clearly be indicated to analyse developments in sugar policy during 2015 and 2016, including the SACN recommendation that adults slash their sugar consumption to just seven teaspoons of sugar per day in men (five in women).^1^

## Conclusions

Public health media advocacy has significant potential to influence policy and practice, particularly around nutrition and healthy lifestyles. This study demonstrates consistent messages about the negative health effects of SSBs being reinforced across the British press, as well as increasing support for policy change and the introduction of a tax on SSBs. Since this research was conducted, the Chancellor for the UK government included in his Budget (16 March 2016) an announcement of a levy on sugary drinks manufacturers equivalent to a 10% excise tax to be implemented in April 2018.^54^ ^55^

However, the UK and global populations face an increase in preventable non-communicable disease attributable to unhealthy diet. This represents a major challenge for public health and healthcare. Further studies are thus needed into the complex relationships between media, public health, government and those industries marketing disease-promoting commodities such as sugary drinks.

Better understanding of effective public health advocacy and effective regulation of food industries will therefore be essential. However, this will happen only when public opinion translates into support for effective policies that reduce the UK consumption of SSBs, especially in children and young people.
