# Supplementary material for: Sugar-sweetened beverages coverage in the British media: an analysis of public health advocacy versus pro-industry messaging
Source: BMJ Open. 2016 Jul 19;6(7):e011295. doi: 10.1136/bmjopen-2016-011295 (PMC4964256; doi:10.1136/bmjopen-2016-011295)
Supplement: Supplementary appendix [file bmjopen-2016-011295supp_appendix.pdf]

FINANCIAL TIMES
Financial Times (London, England)

January 27, 2014 Monday
London Edition 1

Supermarkets join drive against obesity

BYLINE: Scheherazade Daneshkhu, Consumer Industries Editor

SECTION: NATIONAL NEWS; Pg. 2

LENGTH: 516 words

They have been blamed for contributing to a growing obesity problem but supermarkets are falling over one another to brandish their health credentials.

Tesco, the UK's biggest grocer by sales, will announce today a scheme to take primary schoolchildren on outings to farms, factories - and supermarkets - to show them where food comes from and how it is made.

The supermarket chain aims to take 1m schoolchildren - equivalent to one in five of the primary-age school population - on its Farm-to-Fork plan in its first year. The initiative is the first in its Eat Happy Project, which will cost £15m in the first year.

Lidl, the high street discounter, removed chocolates and sweets from its checkout counters this month, saying it was responding to parents' concerns about getting their children to eat healthily when faced with sweets at the checkout.

The supermarket initiatives follow a mounting campaign to try to tackle the spread of obesity in the UK - especially among children.

Action on **Sugar**, a public health campaign launched by medics and health professionals this month, set its aim on getting the food and **soft drinks** industry to reduce **sugar** by 30 per cent in its products gradually over the next three to five years.

The number of obese adults in the UK has doubled over the past 25 years to 26 per cent of the population. Child obesity has also risen sharply to 20 per cent of boys and 17 per cent of girls aged 10-11, though the rate of increase has slowed since 2004.

Packaged and junk foods and more sedentary lifestyles have been blamed for the rise, as well as a lack of knowledge about food.

Though 90 per cent of children said they knew which foods were healthy, according to Future Foundation, the consumer research group, fewer than 10 per cent ate five portions of fruit and vegetables a day.

More than half thought potatoes counted towards the total and one in 10 cited carrot cake as eligible.

Two-thirds of adults surveyed said they thought their children ate much more convenience food than they did and 80 per cent believed their children were less healthy than they themselves used to be as children.

Citing these findings as the basis for its initiative, Tesco said its aim was to improve "children's relationship with food".

Chris Bush, managing-director of Tesco UK, said: "We know parents are concerned that kids don't always understand how food is made and where it comes from, which is important to developing a strong, positive, life-long relationship with food."

The initiative has the support of the National Farmers Union and the Primary Heads Association.

Diabetes UK - one of Tesco's National Charity Partners - said the initiative would help children to "understand the importance of eating a healthy balanced diet".

Most of Britain's largest companies have corporate social responsibility programmes focusing on health, education and community projects.

Tesco's Computers for Schools initiative launched two decades ago won awards but was also a successful commercial promotion. Analysts at the time said that it had had a noticeable effect on Tesco's sales volumes.

LOAD-DATE: January 26, 2014

LANGUAGE: ENGLISH

PUBLICATION-TYPE: Newspaper

Copyright 2014 The Financial Times Ltd.
All Rights Reserved
Please do not cut and paste FT articles and redistribute by email or post to the web.

Mirror

mirror.co.uk

January 27, 2014 Monday 5:39 PM GMT

Children set for 'food lessons' as many believe cheese grows on trees and fish fingers contain CHICKEN; Tesco is pumping £15m into a project to take a million primary school kids around farms and factories and show them where products come from

BYLINE: By Ruki Sayid

SECTION: NEWS,UK NEWS

LENGTH: 480 words

Supermarket giant Tescois to teach children about food after a study found a third of under 11s think cheese grows on trees.

Britain's biggest grocer is pumping £15 million into a "field to fork" project to take a million primary school kids around farms and factories and show them where meat, produce and dairy products come from.

The back to basics Eat Happy project comes as research by the Future Foundation revealed a fifth of five-to-11-year-olds think carrot cake or tomato ketchup count as one of their five-a-day.

Worryingly, one in five believe chicken is the main ingredient in fish fingers yet nine out of 10 think they know which food is good for them.

Launching the scheme in a blog today, Tesco managing director Chris Bush said: "There is a gap, not just in the knowledge our children carry with them but in the fundamental relationship they have with food."

Supermarkets and the food industry have been lambasted by health campaigners for failing to help combat childhood obesity and pressure group Action on Sugar wants to see a 30% sugar reduction in **fizzy drinks,** treats and staples like cereals and soup.

Figures from the National Child Measurement Programme (NCMP) show that 18.9% of ten-to-11-year-olds were obese and a further 14.4% were overweight.

And of those aged four to five-years-old, 9.3% were obese and another 13% were overweight.

Mr Bush said Eat Happy is "a contribution, not a magic bullet".

He added: "I am also realistic that some people will challenge us to have a more abrupt, immediate impact by stopping selling unhealthy alternatives which children find appealing.

"I don't think that is a lasting solution. We will create the healthy habits of a lifetime by making the carrot more appealing not relying on the stick."

Eat Happy will take children behind the scenes to see how veg is grown, bread is baked, cheese and butter made as well as seeing cows being milked.

Schools who are unable to take children on farm, factory or store outings will be able to enjoy a virtual field trip using Google's Connected Classroom technology which will let them talk to UK farmers as well as growers around the world.

Mr Bush said: "This work will take a generation but if we get it right, and I believe we are, we will be helping to break habits formed over several generations and create a healthier future."

The scheme was welcomed by charity The Children's Food Trust which campaigns for healthy food for all kids.

Chief executive Linda Cregan said: "Parents, schools and food retailers and manufacturers all have a responsibility to make sure our children are eating healthy, nutritious food.

"If our children grow up with an understanding and interest in both cooking and eating healthy food, they have the best opportunity to reach their full potential.

"Being overweight or obese from an early age puts our children at a massive disadvantage from the word go."

LOAD-DATE: September 1, 2014

LANGUAGE: ENGLISH

PUBLICATION-TYPE: Newspaper; Web Publication

JOURNAL-CODE: WEBDMI

Copyright 2014 Trinity Mirror, Plc.
All Rights Reserved

THE SUNDAY TIMES
The Sunday Times (London)

January 26, 2014 Sunday
Edition 1;
Ireland

Arguments against fat tax thin on the ground; Subsidies on vegetables and fruit would counterbalance higher prices on unhealthy foods, argues David Madden

BYLINE: David Madden

SECTION: NEWS; Pg. 15

LENGTH: 817 words

The World Health Organisation recognises obesity as a disease, and it is also a substantial risk factor for high blood pressure, heart disease, type 2 diabetes, gall bladder problems and various forms of cancer. Obesity is also a public health issue in Ireland. In 2005, a report produced by the National Taskforce on Obesity provided an extensive list of recommendations. In 2009, because the original recommendations were only partially implemented, a Special Action Group was set up. Among the statistics motivating these concerns were the results of the National Adult Nutrition Survey which indicated that 24% of adults were obese and 37% were overweight.

A notable feature of obesity is the socio-economic gradient, with levels highest among low-income groups. This link may be observed because of the relationship between dietary energy density and dietary cost. Refined grains, added sugars and added fats are among the cheapest sources of dietary energy. Typically, the more nutrientdense foods such as lean meats, fish, fresh vegetables and fruit are more expensive in terms of per calorie intake. Thus, low-income consumers with limited resources are more likely to select diets with high contents of refined grains, added sugars and added fats as a cost-effective way to meet daily calorie requirements.

At a basic level, rising obesity may be explained by increased calorie intake and/or reduced calorie expenditure. Factors such as the fat or carbohydrate composition of food may also affect weight patterns. This suggests measures to combat obesity should address the amount people eat, the composition of that food, and exercise levels.

My research has concentrated on the first two, by examining the role of taxation on people's diets. Among the measures suggested to combat obesity are increased consumption of fruit and vegetables, and lower consumption of fatty and sugary foods. Presuming consumption of these products follows the standard laws of demand, taxation policy can play a role. Tax increases on certain food products should lead to a reduction in their consumption. Moreover, a revenue-neutral reform - comprising higher taxes on fatty and sugary food accompanied by lower taxes or subsidies on fruit and vegetables - should alter the composition of people's diets and may have a beneficial impact in terms of obesity.

A tax on saturated fat was imposed in Denmark in 2011 but removed the following year owing to difficulties in implementation and a belief it had encouraged cross-border shopping. In 2011, Hungary also introduced a tax on foods with high fat, sugar and salt content, whereas France introduced a "soda tax" on **sugar**-sweetened **beverages** (SSBs) in 2012. In Ireland, consideration has been given to the introduction of a tax on SSBs.

One of the principal arguments against a fat tax is that it would be regressive, because consumption of fatty/sugary foods is concentrated among low-income groups. It was on this basis a National Task Force on Obesity in the UK rejected its introduction. My research, using the most recent Irish Household Budget Survey, confirms this conjecture. Pretty much any food-based tax will have a disproportionate effect upon the poor. However, a revenue-neutral combination of a tax on fatty and sugary foods along with a subsidy on fresh fruit and vegetables would be neutral in its effect upon poverty. The same could be said for a combination of a tax on SSBs with a subsidy on fruit and veg. Even in the absence of a subsidy, the amount required to compensate poor families for the effects of the fat tax would be relatively modest.

It can be argued that weight levels are a matter of personal choice and do not constitute a market failure sufficient to merit intervention, unlike alcohol and tobacco, where drink driving and secondhand smoking warrant high taxes. If the degree of market failure regarding obesity is relatively mild, then a fat tax may be unduly paternalistic, and aside from providing information on the fat and sugar content of foods, policymakers should leave individuals free to choose their own diet.

Nor should we underestimate the practical difficulties of implementing such a tax, in particular the choice of foods to be taxed, with representatives of various food interests lobbying for their products to be excluded.

In terms of the regressive effects of such taxes, however, my research indicates they can be almost completely mitigated.

My research concentrated solely on the economic effects of the tax and ignored the potential health benefits. Clearly, if a fat or SSB tax leads to changes in diet and substitution towards healthier food, then benefits will follow in the medium term.

David Madden is a lecturer in the School of Economics at University College Dublin. This is an edited extract from The Poverty Effects of a 'Fat Tax' in Ireland, to be published in full in the journal Health Economics

LOAD-DATE: January 26, 2014

LANGUAGE: ENGLISH

PUBLICATION-TYPE: Newspaper

JOURNAL-CODE: STS

Copyright 2014 Times Newspapers Limited
All Rights Reserved

THE SUNDAY TIMES
The Sunday Times (London)

January 26, 2014 Sunday
Edition 1;
Scotland

'Healthy' foods give off whiff of hype

BYLINE: Kate Mansey

SECTION: NEWS; Pg. 18

LENGTH: 766 words

SOME of the biggest food firms are funding scientific research to establish health claims about their products, ranging from increasing brain power to improving driving skills.

The work, much of which is conducted by academics at universities that have seen their research funding slashed in recent years, has raised concerns about the influence of the food industry on scientific integrity.

A study funded by Mars identified a nutrient in cocoa that could reduce the risk of heart disease, stroke, cancer and diabetes, while research subsidised by Nestlé claimed a daily bar of chocolate could reduce stress for women.

Research at Indiana University, funded by the state's dairy and nutrition council, an industry group, showed chocolate milk helped with muscle recovery in athletes, although subsequent studies found they would recover better from having a full, balanced meal. Research funded by the Austrian firm Red Bull found its energy drink "significantly improves driving performance", a claim later challenged by the International Society of Sports Nutrition, which said it was the caffeine and carbohydrates, rather than the other ingredients in the drink, that contributed to such an effect.

A report published last month said scientific research paid for wholly or in part by the food and drink industry was five times more likely to conclude there was no link between the consumption of **sugary drinks** and weight gain.

Danone, the French food giant, part-funded a study that suggested yoghurt could reduce the risk of heart disease and another that claimed it could increase brain function.

Emeran Mayer, professor of medicine and psychology at University of California, Los Angeles, who conducted research for the second study, said Danone had given him full independence. However, he likened the increasing reliance by academics on funding from the food industry to the influence of the tobacco industry, which spent decades paying for studies attempting to disprove a link between smoking and lung cancer.

"I have been on several peer reviews and I realise that in the review process the company did a lot of arm twisting to keep a paragraph in the manuscript that the reviewers did not want to be in there, so that convinces me that some companies are heavily engaged in influencing the message," said Mayer, who claimed Danone had acted entirely properly. "If you're a young investigator and need the funding, I think you're more easily influenced by what the company wants."

Danone did not respond to requests for comment.

As well as paying for specific academic studies, the food industry is bankrolling the building of laboratories and handing grants to universities across Britain. Figures obtained under the Freedom of Information Act show UK universities have received more than £6m from the food industry over the past three years, including £1m paid to Sheffield Hallam University by food industry sponsors to help build a new food engineering centre.

Jim Mann, professor of human nutrition at Otago University in New Zealand and a critic of the links between academics and the food industry, said: "Let's say I get money from Coca-Cola or Mars. I know if I publish something that says Mars bars are terrible or Coca-Cola is disastrous, I know I'm never going to get money again. So there must surely be a concern at the back of your mind that this industry that has funded your laboratory could pull their support."

Others argue, however, that without funding from the food industry the research would not take place. Lona Sandon, assistant professor of clinical nutrition at the University of Texas, said: "Bias is a concern in all types of research but I believe industry and researchers do need to work together in order to improve the food supply available to the public."

Norman Hollenberg, of Harvard medical school, who conducted the Mars-funded research into cocoa nutrients, said the firm had no input in the final report.

Mars said any research it funded was conducted by respected scientists whose work was peer reviewed.

Nestlé said: "We strongly refute any accusation of bias in Nestlé's research publications. We publish over 200 scientific papers in peer-reviewed journals every year."

Red Bull said: "Our research is in conjunction with internationally recognised scientific organisations."

Feeding a line Red Bull Significantly improves driving performance Chocolate A daily bar can decrease stress for women Cocoa A nutrient in cocoa could lead to decreased risk of heart disease, stroke, cancer and diabetes Yoghurt Eating yoghurt is linked to increase brain function

LOAD-DATE: January 26, 2014

LANGUAGE: ENGLISH

PUBLICATION-TYPE: Newspaper

JOURNAL-CODE: STSscot

Copyright 2014 Times Newspapers Limited
All Rights Reserved

Sunday Express

January 26, 2014
Edition 1;
National Edition

SUGAR VS FAT;
Identical twins set out to discover which of our treats is to blame for the obesity crisis DANNY BUCKLAND meets identical twins who tested the real impact of food

BYLINE: DANNY BUCKLAND

SECTION: NEWS; Pg. 11

LENGTH: 1102 words

THE RACE to demonise sugar in the fight against Britain's soaring obesity levels could be heading in the wrong direction, according to a unique experiment. The "white stuff" is being targeted as public health enemy No. 1 as the nation struggles to cope with personal misery and an annual £5billion bill for treating illness and disease caused by overeating.

Our dimensions are disturbing: 25 per cent of adults are obese and the figures are expected to climb to 60 per cent of men and 50 per cent of women by 2050. Three in 10 children aged between two and 15 are overweight or obese.

Yet as public frenzy builds around the status of sugar, two doctors have conducted a fascinating experiment to see who is the most to blame in the heavyweight fight: Fat or Sugar? Chris and Xand van Tulleken, who have pushed themselves to extremes on expeditions to the Arctic and the jungle, devised an innovative personal challenge to find the answer.

As identical twins with identical genes, they are ideally placed to test the impact of high fat and sugar diets. Xand, director of the Institute of Humanitarian Affairs at Fordham University in New York, went on a high-fat diet with just 5 per cent carbs while Chris had a super low-fat, high-carb regime with no food containing higher than 5g per 100g. Their gruelling month-long quest, detailing the rival metabolic impacts on their minds and bodies, sees them both lose weight with a worrying conclusion that will be revealed on BBC2's Horizon programme on Wednesday.

After duelling through a cycle challenge and a cognitive test trading shares on a virtual stock market however, the verdict surprised both. Neither fat nor sugar was completely culpable and the real villain, a potent combination of high fat and high sugar in processed foods, had escaped the dock and was sunning itself on a metaphoric extradition treaty-free beach.

"We should not vilify a single nutrient. We will be demonising protein next after we have done with fat and sugar," says Chris, an infection doctor at University College Hospital, London, and a Medical Research Council fellow at University College London. "It is too easy to demonise fat or sugar but that enables you to let yourself off the hook in other ways. The enemy is right in front of us in the shape of processed foods."

Research by Professor Paul Kenny, of the Scripps Institute, provides sobering evidence of what is happening a little way down the evolutionary scale.

Feeding laboratory rats with either high-fat and high-sugar diets did little to change their daily habits or health but supply them with chocolate, biscuits and cheesecake (a near 50-50 fat and sugar split) and behaviour changed radically.

They ignored other foods for the cheesecake, going back to it regularly rather than gorging, and put on weight. Their self-regulation system, that naturally stopped them eating too much fat or sugar, effectively switched off.

"It became their main source of calories," Kenny tells Horizon. "They gained massive amounts of weight, became sedentary, slept a lot and did not move around."

He found that the allure of processed food was overriding the body's natural hormones that regulate intake by alerting the brain that the body has enough calories.

It is the same faculty that is impaired in drug addicts whose On-Off mechanisms are degraded by the release of pleasure hormones in the brain's hedonic system, he says.

SUSAN JEBB, Professor of Diet and Population Health at the University of Oxford, has studied the impact of fat and sugar on diets for a decade and believes it is difficult to pin guilt simply on either.

"Processed foods pack calories in and are unbelievably attractive and delicious," she says. "They are temptations for all of us and it is astonishing that any of us stay slim.

"In Britain, where we are surrounded by pretty delicious, relatively affordable and palatable foods, you have to exert quite a level of dietary restraint if you are not going to sleepwalk into obesity."

She adds that no one food is "saint or sinner" while modest lifestyle changes and balanced diet are the way to health.

Chris agrees: "Research over 10 years gives us great confidence to say that no single macro-nutrient diet reduction is the answer. Cutting out a single thing will not solve your problem. Faddish diets simply do not work very well.

"It is about building an environment in your life where you could easily eat a cheap and healthy diet and get enough exercise. It is amazing that we are not all fat and I come away with a sense that I know enough about diet and nutrition and I should be reducing the calories and building an environment where I can do that rather than looking for one toxic ingredient."

The statistics that have lead to a rush to condemn sugar include a tin of tomato soup containing four teaspoons of sugar, the same as a serving of Kellogg's Frosties with semi-skimmed milk, while a can ofCoca-Cola has nine teaspoons of sugar and a 51g Mars Bar, eight.

With the typical Briton consuming 12 teaspoons of sugar a day, experts want that reduced to five. The newly formed Action on Sugar is calling for the food industry to reduce refined sugar content by 20 to 30 per cent in the next five years. "This is not an anti-sugar programme," says Chris. "There is no question that drinking **sugary drinks** will make you fat but whether it is the sugar that is toxic or it is just the calories is not so clear.

"People will be surprised by what happened to our bodies during the experiment. The important element that emerges is that we need to teach a bit of critical thinking so we do not take every sensational new diet or piece of health advice at face value.

"If you find yourself worshiping one nutritional god you may find you need to change churches in a few months as you rise and fall on the tide. Processed foods are delicious and we eat a lot of them so it may be a painful conclusion that we have to remove a bit of deliciousness from our lives but you can find healthy stuff that is delicious."

The 35-year-old twins put themselves through their Angels and Demons dietary mangle in the name of science and both emerged with a greater understanding of how the body performs and how to help it survive.

"It is about creating an environment at home and work where you can have something delicious without every meal being packed with processed foods. Our goal is how we help people make changes to have healthy lives without being totalitarian about their diets."

Sugar v Fat, Horizon, BBC2, Wednesday, 9pm.

'The real villain - processed foods - has escaped the dock'

'Our goal is to help people be healthy'

LOAD-DATE: January 26, 2014

LANGUAGE: ENGLISH

GRAPHIC: ANGELS AND DEMONS: Twins Xand and Chris van Tulleken tested out opposing diets

PUBLICATION-TYPE: Newspaper

JOURNAL-CODE: SXP

Copyright 2014 Express Newspapers

All Rights Reserved

DAILY MAIL (London)

January 24, 2014 Friday

FRESH HEALTH FEARS OVER COLOURING IN FIZZY DRINKS

BYLINE: BY SEAN POULTER CONSUMER AFFAIRS EDITOR

LENGTH: 392 words

HEALTH authorities have launched a fresh inquiry into the safety of a caramel colouring used in soft drinks such as Coca-Cola and Pepsi.

The decision has been taken by the American Food and Drug Administration (FDA) in response to research showing varying levels of 4-methylimidazole (4-MI) in 12 brands of soft drink.

The agency said it is also reviewing new data on the safety of 4-MI in other products.

In the past the FDA, as well as food watchdogs in Britain and Europe, ruled that the chemical poses no threat to the health of consumers.

However, health authorities in California include the substance on a list of cancer-causing agents. As a result drinks companies are required to put a cancer warning on products containing raised levels of 4-MI.

In response to the law, Coke, Pepsi and other manufacturers directed their caramel-colour suppliers to reduce the levels of 4-MI.

Coca-Cola subsequently promised to reduce levels of 4-MI in drinks sold around the world.

However, the same safeguard was not applied by Pepsi in a decision which outraged food campaigners in the UK.

American campaigning group Consumer Reports tested Sprite, Diet Coke, Coca-Cola, Coke Zero, Dr Pepper, Dr Snap, Brisk Iced Tea, A&W Root Beer, Pepsi, Diet Pepsi, Pepsi One and Malta Goya.

The tests found that single servings of two products purchased in California - Pepsi One and Malta Goya - exceeded the level of 4-MI which should have required a cancer warning on the label. No significant level was found in Sprite, and consistently low levels were found in Coke products.

Toxicologist Dr Urvashi Rangan, who led the Consumer Reports investigation, said: There is no reason why consumers need to be exposed to this avoidable and unnecessary risk that can stem from colouring food and beverages brown.'

However, Pepsico has challenged the accuracy of the test results.

Aurora Gonzalez, a spokesman for the firm, said the company is extremely concerned' about the reliability of the study.

She said: PepsiCo abides by the law everywhere we do business.'

The UK arm of the drinks business has rejected claims that 4-MI poses any risk.

Last year, the company said: There is no scientific evidence that 4-MI in foods and beverages is a threat to human health.

Current regulation in the EU states that caramel colouring (4-MI) poses no health risks.'

s.poulter@dailymail.co.uk

© Daily Mail

LOAD-DATE: January 23, 2014

LANGUAGE: ENGLISH

PUBLICATION-TYPE: Papers

Copyright 2014 Associated Newspapers Ltd.
All Rights Reserved

MailOnline

January 24, 2014 Friday 12:31 AM GMT

Fresh health fears over caramel colouring used in fizzy drinks, including Coca Cola: Company vows to reduce amount of chemicals that may cause cancer

BYLINE: SEAN POULTER

SECTION: NEWS

LENGTH: 438 words

- Chemical 4-MI is to face a fresh inquiry into whether or not it causes cancer
- Authorities in California list the controversial additive as a carcinogen
- But European food watchdogs have long argued that the product is safe
- Coca-Cola and other companies have vowed to reduce levels of 4-MI

Health authorities have launched a fresh inquiry into the safety of a caramel colouring used in soft drinks such as Coca-Cola and Pepsi.

The decision has been taken by the American Food and Drug Administration (FDA) in response to research showing varying levels of 4-methylimidazole (4-MI) in 12 brands of soft drink.

The agency said it is also reviewing new data on the safety of 4-MI in other products.

In the past the FDA, as well as food watchdogs in Britain and Europe, ruled that the chemical poses no threat to the health of consumers.

However, health authorities in California include the substance on a list of cancer-causing agents.

As a result drinks companies are required to put a cancer warning on products containing raised levels of 4-MI.

In response to the law, Coke, Pepsi and other manufacturers directed their caramel-colour suppliers to reduce the levels of 4-MI.

Coca-Cola subsequently promised to reduce levels of 4-MI in drinks sold around the world.

However, the same safeguard was not applied by Pepsi in a decision which outraged food campaigners in the UK.

American campaigning group Consumer Reports tested Sprite, Diet Coke, Coca-Cola, Coke Zero, Dr Pepper, Dr Snap, Brisk Iced Tea, A&W Root Beer, Pepsi, Diet Pepsi, Pepsi One and Malta Goya.

The tests found that single servings of two products purchased in California - Pepsi One and Malta Goya - exceeded the level of 4-MI which should have required a cancer warning on the label. No significant level was found in Sprite, and consistently low levels were found in Coke products.

Toxicologist Dr Urvashi Rangan, who led the Consumer Reports investigation in the US, said: 'There is no reason why consumers need to be exposed to this avoid-able and unnecessary risk that can stem from colouring food and beverages brown.'

However, PepsiCo has challenged the accuracy of the test results.

Aurora Gonzalez, a spokesman for the firm, said the company was 'extremely concerned' about the reliability of the study.

She said: 'PepsiCo abides by the law everywhere we do business.'

The UK arm of the drinks business has rejected claims that 4-MI poses any risk.

Last year, the company said: 'There is no scientific evidence that 4-MI in foods and beverages is a threat to human health.

'Current regulation in the EU states that caramel colouring (4-MI) poses no health risks.'

LOAD-DATE: January 24, 2014

LANGUAGE: ENGLISH

PUBLICATION-TYPE: Web Publication

JOURNAL-CODE: WEBDM

The Sun (England)

January 23, 2014 Thursday
Edition 2;
Scotland

What would you like with your [...];
SCOTLAND'S SUGAR DIET SHOCKER

BYLINE: NIAMH ANDERSON

SECTION: FEATURES; Pg. 6

LENGTH: 680 words

SCOTS are eating DAMAGING amounts of sugar every day, experts have warned.

Just adding a dollop of tomato ketchup to your square sausage butty for breakfast would be like taking two teaspoons of the sweet stuff with it.

We've put together a daily menu, bottom, featuring some of our nation's best-loved dishes and snacks and found it contained THIRTY teaspooons of sugar - SIX TIMES the recommended daily amount. While it's all fine grub if eaten as part of a healthy diet and in moderation, here we provide a warning from a nutritional expert and some top tips on cutting back on your daily intake of sugar ...

NUTRITIONAL expert Dr Chris Fenn, 56, of Aberdeen, reckons our sugar rush will only get worse unless food giants and the Scottish Government step up to the plate. She said: 6 Sugar is a huge problem in Scotland. We are conditioned to like it.

Back in the Stone Age cavemen spat out bitter berries and preferred sweeter ones. We have evolved with that liking for sugar.

Food manufacturers know that so they put truck-loads of it in food. The sweeter it is, the more we like it and the more we buy it. They're on a winner and they don't care about our health.

Traditionally typical Scottish foods are white bread, Irn-Bru, fried foods and tablet.

When you've been brought up on that, it's difficult to change and that's going to be a big challenge.

What people don't realise is that they are eating a lot of hidden sugars. So-called savoury foods can actually be very bad for you. White bread and morning rolls are full of sugar, for example.

The main message the Government has been feeding us for the last 30 years is 'eat low-fat foods' so manufacturers have responded by making low-fat products which are loaded with sugar.

'More energy, less headaches' In that case, I'd first try to identify where your sugar is coming from if you're trying to cut down. Check labels on yoghurts, breads, soups and anything you're unsure about.

Some people need to be weaned off sugar, just like drugs. It is an addiction.

It's not just about the longterm affects like diabetes and obesity - both of which are a huge problem in Scotland. But it's also about the shortterm problems it causes. If people cut down their sugar intake, they'd soon realise that they have more energy, less headaches, a better immune system and fewer mood swings.

It is great news the Government is making an issue of this but they should go further and subsidise low-sugar food so that it is affordable to eat healthily.

But the manufacturers are part of the story too. They should realise, 'Hey our sugar levels can be cut down'. It's a huge business opportunity for companies to launch new foods and change their image.

There's a big gap in the market for low-sugar snacks and they could monopolise on that if they're smart enough. 7 Q Dr Chris Fenn's book Forget The Fear of Food: The Essential Guide is available for £9.99. Contact www.chrisfenn.com.

NHS tips on cutting down your intake Q Instead of **fizzy drinks** and juice drinks, go for water or unsweetened fruit juice. Q Swap cakes or biscuits for a currant bun, scone or some malt loaf with low-fat spread. QGRADUALLY reduce the amount of sugar in tea until you can cut it out altogether. QTRY a low-fat spread, banana or low-fat cheese on toast of jam. Check nutritional labels to you pick the foods with addedsugar. Try halving the sugar you in recipes - it works for things except jam, and ice cream. Choose tins of fruit in rather than syrup.

DAiLY MENU PACKED WiTH OUR NATiON'S FAVE GRUB iS SWEET BUT LEAVES SOUR TASTE MORNING SNACK LUNCH AFTERNOON SNACK BREAKFAST DINNER 2x white rolls 2 TEASPOONS 2x

square sausage Greggs pasty (Cheese &Onion) Tunnock's Teacake 0.5 TEASPOON Mince & tatties 1.5 TEASPOONS 1 TEASPOON 10 TEASPOONS 1x square of tablet 0.5 TEASPOONS One serving ketchup Coffee with 1 serving sugar Scotch Pie 2 TEASPOONS Mackie's ice cream 1 TEASPOON 0.5 TEASPOON 1 TEASPOON Coffee with 1 serving sugar 4 TEASPOONS 1 TEASPOON Coffee with 1 serving sugar 5 TEASPOONS Can of irn-Bru TOTAL AMOUNT: 30 TEASPOONS RECOMMENDED DAILY AMOUNT: 5 TEASPOONS

LOAD-DATE: January 23, 2014

LANGUAGE: ENGLISH

PUBLICATION-TYPE: Newspaper

JOURNAL-CODE: SUNscot

Copyright 2014 NEWS GROUP NEWSPAPERS LTD
All Rights Reserved

102 of 341 DOCUMENTS

FINANCIAL TIMES

Financial Times (London, England)

January 22, 2014 Wednesday
USA Edition 1

Peltz joins Mondelez board after Pepsi push; FOOD PRODUCERS

BYLINE: Neil Munshi and Shannon Bond

SECTION: COMPANIES - ROUND-UP; Pg. 14

LENGTH: 267 words

Nelson Peltz, the activist investor, will drop his campaign for PepsiCo to buy Mondelez International, the snacks business behind Oreo cookies, Cadbury chocolate and Trident gum, after Mondelez added him to its board yesterday.

The move saves the company from more agitation in the public campaign Mr Peltz launched last year urging PepsiCo to merge its snacks division with Mondelez. Mr Peltz's Trian Fund Management has billiondollar stakes in both companies.

"Now that Nelson Peltz is a director [at Mondelez], as long as Trian is a significant shareholder of Pepsi-Co, Mr Peltz will recuse himself from discussions pertaining to PepsiCo," said a spokeswoman for Trian.

As PepsiCo has made clear that it is not interested in pursuing a merger with Mondelez, Mr Peltz will instead encourage Pepsi-Co to separate its snacks and beverages divisions, she added.

Indra Nooyi, PepsiCo chief executive, has argued that the company's drinks and snacks brands complement each other and has resisted Mr Peltz's calls for a split.

PepsiCo is nevertheless considering restructuring its underperforming North American beverage business, which has suffered from lower US demand for **fizzy drinks**. It will update shareholders in February.

Trian is Mondelez's fourth-largest shareholder, with a 2.3 per cent stake, according to Thomson Reuters data. Mr Peltz will stand for election to the board, which will expand to 12 members with his addition, at the annual meeting later this year.

Mr Peltz said his focus would be "driving growth, improving margins and increasing value for all shareholders".

Neil Munshi and Shannon Bond

LOAD-DATE: January 21, 2014

LANGUAGE: ENGLISH

PUBLICATION-TYPE: Newspaper

Copyright 2014 The Financial Times Ltd.
All Rights Reserved
Please do not cut and paste FT articles and redistribute by email or post to the web.

MailOnline

MailOnline

January 22, 2014 Wednesday 1:05 PM GMT

SUGAR DETOX DIET... and you can STILL eat cake! Low sugar treats that you'll never notice are healthy

BYLINE: BROOKE ALPERT & DR PATRICIA FARRIS

SECTION: FEMAIL

LENGTH: 1045 words

- We eat far too many **sugar**-laden **soft drinks** and foods, such as cakes
- With diets of refined carbohydrates, we end up with constant flow of **sugar**
- Triglycerides spill into blood, clogging arteries, increasing heart attack risk
- Body can get used to pumping out insulin, cells become 'insulin resistant'
- Over the past few weeks, the Mail revealed a ground-breaking **sugar** detox
- Experts Dr Brooke Alpert & Dr Patricia Farris now explain how you can still eat **sugar** and be healthy

So, can you ever eat sugar and be healthy? If you've been reading the Mail's ground-breaking Sugar Detox over the past few days, this is a question you're bound to be asking yourself right now.

As we've shown, sugar is highly addictive, and can take its toll on your waistline and your skin.

And what makes it so pernicious is that it's very difficult to avoid, as manufacturers have hidden it in so many foods to make their products more appealing. It's in soups, pasta sauces, salad dressings and bread, as well as cakes and biscuits.

But don't worry. There is a way to enjoy cakes, biscuits and other treats without jeopardising your health or appearance.

Just follow our Detox guide, with its simple dietary rules for a three-day blitz that will give your body a complete break from all forms of sugar, and end your cravings, before slowly reintroducing otherwise healthy foods, such as wholegrains, that form sugar when digested (missed the plan? Go to dailymail.co.uk/quitsugar).

After our four-week Sugar Detox, you should be free from cravings, energy dips and mood swings. And, even better news, you can then resume a normal, healthy diet - with cakes, treats and puddings - as long as you follow one easy rule each time.

The key to living in harmony with sugar in your life without risk of addiction is understanding how foods interact in your body. You can then minimise the impact that sugar might have on your body and your skin.

Say you haven't eaten in more than three hours and you grab a piece of fruit to keep you going. As it's been so long since you had a proper meal, your system is unable to break down the fruit and utilise the best parts of it - its antioxidants, fibre, vitamin C, and more. Instead, your body is much more likely to say 'feed me!' and go straight for the sugar contained in the fruit.

But this process can be stopped if you make a conscious effort to only ever eat sugar (whether in the form of pastries, or fruit or refined carbohydrates like bread) in combination with protein foods (such as meat, fish or vegetarian forms of protein like beans and lentils) and/or healthy fats (nuts, seeds, olive oil).

Eating something sugary, whether it's a clementine or a chocolate bar, on an empty stomach with no protein or fats to slow its absorption, means that the sugar will be swept up into your bloodstream very quickly.

This hit of sugar then tells your pancreas to release insulin to help absorb it, thereby telling your body to convert the extra sugar to fat.

So beware. If you're eating anything containing sugar, refined flour or even starchy vegetables on an empty stomach, it will almost immediately be converted into fat.

So by all means enjoy berries for breakfast, but make sure you have eggs and yogurt, too. Grabbing an apple? Then take a handful of nuts as well. It's a simple rule that could change your health - and waistline - for good.

How to keep to your Sugar Detox for ever

Once the four-week sugar detox is over, you can happily return to a healthy diet, as long as you remember to combine sugar (whether in the form of biscuits, potatoes or pasta) with protein and healthy fats whenever you can to minimise its negative impact on your body.

But if you love how the Sugar Detox makes you feel - and how your skin now looks - you can continue on our specially designed maintenance plan. This allows flexibility for special occasions, while building on the astonishing effect of your initial detox.

The plan below limits fruit choices and serving sizes, while optimising the health benefits of fruit without letting your sugar intake get out of hand.

Here's what you can eat

PROTEIN: Minimum of 3 servings per day (1 protein serving is 3 eggs or 6oz/170g lean meat, fish - equivalent to a small chicken breast).

DAIRY: 2 servings, plus the milk in coffee or tea (1 serving of dairy is equivalent to 1oz/30g cheese, 5floz/150ml yogurt or cottage cheese, or 4 floz/100ml milk).

VEGETABLES: Unlimited quantities of approved low-carbohydrate vegetables.

FRUIT: 1 apple plus 1.5 portions of other low-carb fruit.

fat: Minimum of 3 servings of healthy fats (such as 1 oz/30g nuts or seeds or a tablespoon of coconut/olive oil, 1/2 avocado or a handful of olives).

CARBOHYDRATES: 2 servings per day of approved carbohydrates plus 2 x high-fibre crackers (a serving = 1 slice of bread, 2oz/ 60g barley, buckwheat, oatmeal, uncooked wholemeal pasta, quinoa or brown rice).

WINE: Up to a maximum of 5 glasses of red wine per week.

DESSERT: 1oz/30g dark chocolate daily (or same portion of any other chocolate, but only twice a week)

During this maintenance plan, it is ok to throw caution to the wind and enjoy a dessert up to twice a week.

Simply use one of your carbohydrate portion allowances for the extra dessert. So if you know there's a birthday in the office and you'll be offered a slice of cake, plan ahead and forgo that multigrain roll with your lunchtime salad.

Or, if it comes upon you as a surprise - say, an impossible to resist pudding trolley at a celebration dinner - just skip one carbohydrate portion during the next 24 hours.

- If you have a medical problem or blood sugar problem such as hypoglycemia, insulin resistance or diabetes, see your GP before starting any diet. The plan is not suitable for people who exercise intensively.

Extracted from The Sugar Detox: Lose Weight, Feel Great And Look Years Younger by Brooke Alpert and Dr Patricia Farris, published by Bantam Press at £8.99. To order a copy for £7.99 (incl P&P) call 0844 472 4157. © Brooke Alpert and Patricia Farris.

LOAD-DATE: January 23, 2014

LANGUAGE: ENGLISH

PUBLICATION-TYPE: Web Publication

JOURNAL-CODE: WEBDM

Copyright 2014 Associated Newspapers Ltd.
All Rights Reserved

MailOnline

MailOnline

January 22, 2014 Wednesday 12:01 AM GMT

Cameron Diaz exclusive: Don't fall for sugar's sweet talk

BYLINE: CAMERON DIAZ

SECTION: YOU MAG

LENGTH: 594 words

There's a biological reason why most people are seduced by sweet foods, but it's a relationship you should consider ending - immediately!

Do you love sweet foods? If you're like most people, the answer is yes. And it turns out, there's a biological reason for that: sweet foods aren't poisonous. Our ancestors knew that if a food was sweet, it was safe to eat. Sweetness was a sign that a plant was edible (most plants that are poisonous to humans taste bitter). Sweetness is also an indication that the plant is high in glucose, which meant that it would offer us lots of energy.

So yes, humans love sweets, and that love is natural and pure - when you're talking apricots and cherries and watermelons. The sugar contained in fruit, fructose, is a healthy sugar when you're eating the whole fruit and getting all the fibre, vitamins and minerals of the fruit.

But when sugar is extracted from sweet plants and added to another food (such as bread or cereal), you're not getting any nutritional benefits. That sugar is nothing but an added sugar and it offers nothing but empty calories. Your relationship with sugar is one that you should reflect upon. I think that you should consider breaking up with sugar. Immediately.

● Here are some strategies to help combat your sugar habit:

● Cut back on the sugar you add. Stop pouring sugar on to the foods you eat, such as cereal, oatmeal, coffee and tea.

● Do not drink **sugar**-sweetened **beverages.** A 590ml sports drink or sweetened flavoured water contains around seven teaspoons of added sugar.

● Spice it up. Learn to use spices and flavours such as cinnamon, nutmeg, vanilla and cardamom to add sweetness and appeal, so you don't need the sugar. An apple sliced and sprinkled with cardamom is a surprisingly rich-tasting snack.

● Choose fruit over sweets. Have you ever noticed that most sweets come in fruit colours and fruit flavours? A sweet is basically fake fruit that wishes it was fruit and is doing its best to smell and taste like it. An orange in season is as sweet as a sweet, and has even more orange flavour than that lollipop, because it's actually an orange.

● Be on the lookout for common added sugar culprits. Just because they don't taste sweet doesn't mean they aren't spiked with corn syrup or other added sugars. Foods such as ketchup, pretzels, cereal, instant oatmeal, yoghurt, salad dressing and granola.

So how much sugar is too much sugar? The American Heart Association suggests that women shouldn't eat more than six teaspoons of added sugar each day. Let's put that into perspective: one 590ml bottle of **fizzy drink** contains nearly 20 teaspoons of sugar. My personal guideline? I'll share a dessert with friends once in a while. But on a daily basis, fruit is my sweet treat.

MY SHOCK CONFESSION

I've got a confession: I don't like sugar. Sugary things do not make me go back for more. All my life, people have looked at me incredulously when I gently decline their shared treats. 'Not even this amazing sweet?' No, sorry, not into sweets. 'How about this delicious raspberry jam?' No, thank you.

The more I learn about the dangers of sugar, the more grateful I am that I'm not drawn to it. Because the sugar habit is a bad one - I've seen it hurt friends, contributing to anything from food addiction to diabetes to that extra 15 pounds around the middle. The more I learn about all these things, the more I realise that my natural aversion to sugar is probably one of the pillars of my health.

LOAD-DATE: January 22, 2014

LANGUAGE: ENGLISH

PUBLICATION-TYPE: Web Publication

JOURNAL-CODE: WEBDM

Copyright 2014 Associated Newspapers Ltd.
All Rights Reserved

The Telegraph
telegraph.co.uk

January 22, 2014 Wednesday 11:03 AM GMT

Banning 'energy drinks' from schools: Why not Coke, Sprite and Fanta too?;
As a government adviser says schools should ban the sale of energy drinks to children - after a report found they contained 13 teaspoons of sugar each - mother of three Samantha Washington asks why stop there: fizzy drinks are just as bad

BYLINE: By Samantha Washington Sky News Business Presenter

LENGTH: 930 words

With names like Red Bull, Monster and Relentless, they sound more like films on the adult channel to me, than things that could or should be available to children.

They are in fact energy drinks, the market for which is growing fast. Since 2006, the UK's consumption of these beverages has more than doubled to 475 million litres. That's around 7.6 litres per person, and coins in £1.4bn for the producers of the newly declared public enemy.

When I hear a government adviser stating that these energy drinks, with their 13 teaspoons of sugar and 160mg of caffeine, are as harmful as drugs and that they should be banned from schools, I find myself nodding in violent agreement. It sounds like a no-brainer. I have three young children, and the thought of needing to spend money to supplement their energy levels feels like some kind of underworld punishment. Nevermind the potentially harmful effects on their health.

Energy drinks are distinct from sports drinks. The Red Bulls, Monsters and Relentlesses of the world are glucose-based drinks that claim a particular stimulus, or energy from caffeine and often some combination of things called guarana, taurine and ginseng. A sports drink on the other hand replaces fluids and electrolytes lost through sweat and provides a carbohydrate boost.

So this government adviser, John Vincent, says these drinks are "effectively another form of drugs". Pupils themselves report feeling dizzy, sick and hyperactive. The Food Standards Agency acknowledges that consuming energy drinks like these "could potentially lead to short-term effects such as increased excitability, irritability, nervousness or anxiety." It's enough for me, I won't be buying this stuff for my children.

But what we have is not a law, like we do with alcohol or cigarettes, but a "code of practice" as laid down by the British Soft Drinks Association. It says these drinks should not be sold in schools and insists that the cans display a label warning of unsuitability for children and pregnant women. But some schools sell them, and kids still go to supermarkets to buy them. So, this code of practice is about as useful as a chocolate teapot. It's down to the individual school or supermarket to put in place it's own policy on this.

Coming from a business background, however, and having an embarrassing love of interrogating the numbers, I had a good look at the British **Soft Drinks** Association's annual report. It confirms the energy drink market is going gangbusters. But, compared to other categories in the **soft drink** market, it's a minnow swimming in a **sugary** sea.

Carbonates, as they're known to **soft drinks** aficionados, are the **fizzy drinks** I declare hatred of: Coke, Sprite, Fanta (and these are said to contain around eight teaspoons of **sugar** per can). My children take an unsurprisingly different view, and while I don't routinely buy these products, I do allow my children to have a **fizzy drink** on occasion when we go out to dinner as a family. I need to admit that after years of zero tolerance, the syrupy waves have slowly eroded my unwavering wall of resistance.

Sticking clumsily with the oceanic metaphor, it is these "carbonates" which are the big blue whales of the bunch, compromising more than 45 per cent of the soft drink market. Energy drinks represent a paltry 3.3 per cent.

On a per person basis, compared to the seven or so litres we each poured down our gullets of energy drinks last year, we each sloshed 102 litres of the fizzy stuff through our systems. One hundred and two litres! But, as investigators always say: follow the money. The value of sales in the UK of this carbonated "rubbish" is £8.7bn. The equivalent figure for the energy drink is £1.4bn.

But, what I find really interesting here is that if you work out how much money these big drinks companies make per litre, you might start to understand why the big boys could be worried. The value per litre of an energy drink is £2.9. This dwarfs the kerching sound heard by the Coca-Colas of the world who rake in £1.3 per litre for their main products. Coupled with the fact that sales of the fizzy stuff are slowing and purchases of the energy drinks are taking off, it might be white-knuckle time at Carbonate HQ.

Some of the big boys do have their own energy drink brands. In keeping with adult-movie theme, Coca Cola's are called Full Throttle, Power Play and Mother. But these are a tiny part of its overall franchise. When you remember the profit margins of the different categories of drink, it wouldn't be beyond imagination to see some takeovers in this industry in the near future.

But until then, I speculate as to whether the attack on the energy drink sector is also convenient in a business sense for some of the big fish. It's further developed in the United States, where some of fast growing energy drink companies, like Monster, have seen their bottom lines hit by challenges to the safety records of their products.

Closer to home and as a parent, I feel that these energy drinks shouldn't be sold in school cafeterias. But, if you follow the logic, why should this not also apply to squash and **fizzy drinks** too. They might not be "as bad", but it's a volume game, and kids drink more of them.

Sometimes I think we should pause for a moment and look at whether, in the desire to pick fights against new enemies, we have dulled our senses and declared resistance futile to the things we used to hate.

Samantha Washington is a presenter on Sky News Business and BBC 5Live's Wake up to Money.

LOAD-DATE: January 22, 2014

LANGUAGE: ENGLISH

PUBLICATION-TYPE: Web Publication

JOURNAL-CODE: WEBDTNS

Copyright 2014 Telegraph Media Group Limited
All Rights Reserved

Daily Mirror

January 21, 2014 Tuesday
Edition 1;
Northern Ireland

MY BATTLE TO DITCH SUGAR IN MY KIDS' DIET;
THE SWEET TRUTH: DAY 2 For a week Mirror writer Amanda Killelea struggled to reduce the amount of sugar her two sweet-toothed daughters ate... but after tears over chocolate cake came a breakthrough

BYLINE: Amanda Killelea

SECTION: FEATURES; Pg. 30,31

LENGTH: 1688 words

Two little sugar addicts live in my house - my daughters. Over the years Gracie, nine, and Lola, seven, have both developed a terribly sweet tooth - and to be honest I blame myself.

I suspect my children consume way too much sugar than they need to and I often wonder if, aside from the health issues, that it will affect their performance at school and give them 'sugar rush' mood swings as some experts are warning.

The Government's sugar GDA (Guideline Daily Amount) for five to 10-year-olds is 85g - and yet the average child's diet would struggle to stay within this limit.

Even so-called health foods are full of the stuff - a pot of fat-free yoghurt can contain the equivalent of five teaspoons of sugar, whereas a tin of tomato soup has four teaspoons.

Manufacturers add sugar to everything from bread to pasta sauces to baked beans to add flavour and increase the shelf-life of the foods, making it harder for us to know exactly how much sugar we are actually eating.

And sugar labelling on packaging is a minefield for parents, as sugar comes in many guises and is hidden in different wording.

Every time you see the words hydrolysed starch, fructose, glucose, modified starch, starch, carbohydrate, honey, molasses, corn syrup, maltose, sucrose, treacle, fruit syrup or maple syrup, this is in fact sugar.

My family aren't the only ones with a sugar addiction spiralling out of control.

The typical Brit eats or drinks around 12 teaspoons of sugar each day, with some of us putting away up to 46 teaspoons of the stuff.

When Gracie and Lola were born I resolved they would be the healthiest kids around. The health visitor put the fear of God into me about sugar - pointing out that many of the baby foods on the market contained hidden sugar that my children just didn't need.

So I became a mum on a healthy mission making everything from scratch, boiling and pureeing fruit, vegetables and healthy meals to make sure they got the best start in life. But once they started nursery and school it became harder to stick to my healthy regime.

Where once it was rice cakes, carrot sticks and fruit, soon they were tucking into biscuits, chocolate and cakes and they got a taste for it.

Milk and water was replaced by juice, cordial and **fizzy drinks** which made them run round the house like loonies after just a few sips.

It is no wonder that the last time we went to the dentist Gracie had to have a filling.

Now Prime Minister David Cameron has said he will quit **sugary drinks** and sweets one day this week to highlight the Government's concerns about obesity.

But how easy is it to cut down - and more to the point - how would children react with less sugar in their diet? For one week I resolved to drastically reduce the amount of processed sugar in their diet, letting them only eat sugar that naturally occurs in fruit and vegetables. Here is how I got on:

DAY 1: SUNDAY

When I announced my plans Gracie and Lola were devastated. Lola in particular is a little chocoholic, so a week without her beloved Cadbury's Marvellous Creations popping candy chocolate bar - which contains a whopping 58.5g of sugars per 100g - seemed impossible.

I am ashamed to say my daughters normally start their sugar eating at breakfast. Toast with chocolate spread, crumpets with jam and Weetabix with honey all feature on our menu, washed down with a glass of orange juice. So instead, the girls have toast with butter, some fresh raspberries and a cup of hot milk. So far so good.

Lunch is chicken dippers, oven chips and frozen peas. I check the ingredients and there is no sugar added to any of these convenience foods which is a nice surprise. The girls drink water instead of cordial.

We ate out for dinner. Normally I would let the girls have a **fizzy drink** as a "treat". I realise now I am not doing them any favours by describing sugar-laden drinks and sweets as treats. Instead they have water with their spaghetti bolognese.

DAY 2: MONDAY

Gracie has Weetabix without her usual dollop of honey. She scans the packet and finds even Weetabix has sugar added to it, but it is by far the healthiest cereal we have in the house.

Lola opts for fruit and buttered toast.

The girls head off to school with apples and chopped carrots for snacks - I try to give them a Fruit Factory Fruit Strings which are allowed in school and on the box it says they are "bursting with fruit juice concentrate" and have "no artificial colours or flavours". Yet on closer inspection the ingredients list includes glucose syrup, sugar, and fructose syrup and each one contains 9.6g of sugars.

For lunch Gracie has a turkey sandwich, Lola has jacket potato and cheese and they both have crackers and butter for dessert. I feel guilty that they turned down the ginger sponge cake that was on the menu in the canteen.

Dinner is their favourite - roast chicken with lots of veg. Normally the girls want some chocolate or biscuits after tea. Instead it's strawberries and grapes.

DAY 3: TUESDAY

For breakfast Gracie opts for crumpets with butter. Normally she would have jam too but after inspecting the label she declares it has more sugar than fruit.

The Tesco own-brand crumpets contain sugar syrup and their own-brand pancakes Lola chooses also list sugar as an ingredient. I don't use sugar when we do home-made pancakes so I am assuming the supermarkets add sugar to increase shelf-life and add flavour. Both girls have water to drink instead of their cordial.

School dinner was spaghetti and meatballs with fruit for dessert. For tea we have fish, roast potatoes and veg and the girls go happily to bed for once.

DAY 4: WEDNESDAY

We have sausages that need using up so I do a cooked breakfast. But the Morrisons own-brand sausages contain sugar.

I am realising most supermarket foods have sugar added. Unless supermarkets ditch the sugar our only way of avoiding it is to make all our meals - even bread and crumpets - from scratch. But who has time to make bake bread every day?

Dinner is chicken wrapped in bacon with lots of veg again. But that evening Lola's resolve begins to slip - she was literally begging me for a custard cream biscuit. I manage to persuade her to make do with some hummus and carrot sticks instead. She's lost her sense of humour a bit by this point.

DAY 5: THURSDAY

Breakfast is Weetabix washed down with orange juice. I reason that although fruit juice is high in sugar, it is sugar that occurs naturally, and it contains vitamins and minerals the girls' bodies need. At school the girls have chicken pie and fruit.

Thursday night is always a big rush in our house - I usually work late and the girls have after-school activities so we normally have a convenience tea. Tonight it is pizza from Tesco - which sadly has sugar added to the pizza base.

A friend pops round and brings bags of sweets and chocolates for the girls and they are literally salivating over them but interestingly don't ask for any. Instead they have raspberries. Maybe their tastebuds are changing.

DAY 6: FRIDAY

I have noticed the girls have been much calmer this week. Maybe the "sugar rush" theory that sweet stuff affects kids' behaviour is true.

But today I ease off the no-sugar rules and let Lola have a chocolate pancake for breakfast, while Gracie has syrup on hers. Then at lunchtime I get a call off Lola's teacher saying she was in floods of tears at lunchtime because the chocolate cake for dessert had sugar in it.

I don't want to be the weird healthfreak mum who sends her children to parties with a tub of fruit instead of letting them tuck into the fairy cakes. Gwyneth Paltrow I am not.

As a mum this is so hard. You want to encourage healthy choices, but you don't want them to be the odd one out, or to develop a complex about food.

VERDICT

Overall the no-sugar week has been good. It has made us all think more about what we eat and it has certainly made me read food labels much more carefully.

I've been shocked at how many foods contain added sugar when they really don't need it, and also at the big differences in the amounts of sugars added to foods by different brands and retailers.

This makes it really hard for parents to keep tabs on the amount of sugars their kids consume. Hopefully when the new traffic light labelling system comes into full force this year with red, amber and green labels reflecting high to low levels of fat, salt and sugar, it will make life a lot easier.

I realise that we had slipped into bad habits and I wasn't keeping track of the amount of sugar and empty calories my children were consuming.

By cutting back on the cakes, biscuits and sweets I have definitely noticed an improvement in the girls' moods.

They have been less argumentative and a lot calmer, but I'm not sure whether that is a result of eating less sugar or less of the additives that sugary snacks tend to contain.

Eating a diet completely free from added sugar is impossible. I don't want my children to be crying at school over a chocolate cake or have hang-ups about food.

I think a little bit of what you fancy is fine but we will try to steer clear of sugary snacks during the week and save them for weekends and special occasions.

HOW MUCH HIDDEN SUGAR?

Supermarkets and food companies don't make it easy for parents to keep track of how much sugar their children are eating and Amanda found the amounts in similar kids favourites from different brands can vary wildly, as these examples show:

BAKED BEANS

Morrisons - Nu Me - 3g of sugars per 100g

Tesco Healthy Living - 3.8g of sugars per 100g

Asda Chosen by You - 4.1g sugars per 100g

Tesco Everyday Value - 4.5g of sugars per 100g

FROMAGE FRAIS YOGHURTS

Tesco Everyday Value - 1.1g of sugars per 100g

Aldi Everyday Essentials - 11.8g of sugars per 100g

Yoplait Petit Filous - 12.2g of sugars per 100g

Munch Bunch - 12.5g of sugars per 100g

CRUMPETS

Asda Chosen by Kids Yummy Rocket crumpets - 1.1g of sugars per 100g

Warburtons crumpets - 2g of sugars per 100g

Morrisons - 3.3g of sugars per 100g

Tesco - 3.8g of sugars per 100g

I realise now I'm not doing my girls any favours by describing sugar-laden drinks and sweets as treats

They've argued less but I'm not sure if that's due to less sugar or less additives that snacks contain

LOAD-DATE: January 21, 2014

LANGUAGE: ENGLISH

GRAPHIC: switch Gracie, left, and Lola with a sugar-free menu
SUGAR RUSH Amanda shows her girls healthy and not-so healthy treats

PUBLICATION-TYPE: Newspaper

JOURNAL-CODE: DMR

MailOnline

January 21, 2014 Tuesday 2:43 PM GMT

The sugar tsars 'in bed' with confectionery giants: Five members of committee tasked with battling obesity epidemic have 'worryingly close' ties

BYLINE: ARTHUR MARTIN

SECTION: NEWS

LENGTH: 535 words

- Experts under fire for being paid to advise firms like Coca Cola and Mars
- Food giants being urged to cut sugar in products as it is 'the new tobacco'
- Doctors and academics say levels must drop by at least 30 per cent

Experts who advise the Government on sugar consumption were under fire last night after it was revealed they receive funding from confectionery giants.

Five out of eight members of a committee tasked with helping to tackle Britain's obesity epidemic have 'worryingly close' ties with the food industry, it was claimed.

They include chairman Professor Ian Macdonald - one of the country's leading nutritionists - who works as a paid advisor for Coca-Cola and Mars.

Yesterday critics said those who sat on the so-called 'sugar committee' could not be trusted because many of them are 'in bed' with food manufacturers.

The row comes as food giants are being urged to cut sugar in products amid fears it has become the 'new tobacco'.

Doctors and academics say sugar levels must be reduced by at least 30 per cent to halt a wave of disease and death.

The typical Briton consumes 12 teaspoons of sugar a day - and there are nine in a can of Coca-Cola and eight in a 51g Mars Bar.

Professor Macdonald is paid £6,100 to sit on two advisory boards for Coca-Cola and also receives a larger payment for advising Mars.

But in 2009 he faced concerns over a potential conflict of interest and stood down until 2012.

His research at Nottingham University has now received more than £1million in the past three years from the food industry, including £300,000 from Mars.

Funding also comes from Unilever - the world's largest ice cream manufacturer.

The disclosures yesterday prompted calls for the scientist to resign from the panel because of an 'unacceptable' conflict of interests.

Simon Capewell, from campaign group Action on Sugar, said: 'It's like putting Dracula in charge of a blood bank.

If Ian Macdonald doesn't step down, there will be real concerns that their recommendations will be prejudiced by commercial factors rather than scientific public health priorities.'

Cardiologist Aseem Malhotra added: 'I don't think that anyone who is in bed with the food industry should be advising the Government.'

Other committee members with ties to the sugar industry include Ian Johnson, a consultant for Swiss chocolate multinational Barry Callebaut.

Professor Ian Young and Professor Julie Lovegrove have also received funding from the sugar industry, while David Mela is an employee and a shareholder of Unilever. All eight members are paid for their work on the committee.

In an interview with Channel 4's Dispatches programme, Professor Macdonald denied that he is too close to food companies.

He claimed it was important to confer with the industry and said he never discussed any of his government work with Coca-Cola or Mars.

Last night Public Health England said advisers must declare any potential conflicts of interest and be 'independent and professionally impartial'.

A spokesman said there were processes in place to ensure 'transparency and integrity'.

A report to be published later this year by the committee on the health impact of sugar consumption could have a massive impact on the food industry.

LOAD-DATE: January 22, 2014

LANGUAGE: ENGLISH

PUBLICATION-TYPE: Web Publication

JOURNAL-CODE: WEBDM

Copyright 2014 Associated Newspapers Ltd.
All Rights Reserved

MailOnline

January 21, 2014 Tuesday 1:27 PM GMT

SUGAR DETOX DIET: Beware the bagel... not to mention rice, dried fruit and crayfish salad: They're ALL secretly sugar laden - with toxic results for your body

BYLINE: BROOKE ALPERT & DR PATRICIA FARRIS

SECTION: HEALTH

LENGTH: 1901 words

- We eat far too many **sugar**-laden **soft drinks** and foods, such as cakes
- With diets of refined carbohydrates, we end up with constant flow of **sugar**
- Triglycerides spill into blood, clogging arteries, increasing heart attack risk
- Body can get used to pumping out insulin, cells become 'insulin resistant'

For decades, it's been drummed into us that saturated fat is the greatest dietary evil, so it seems hard to believe that sugar is actually worse.

But there's a growing body of expert opinion that, in fact, it's sugar that's to blame for so many deadly illnesses, from heart disease and type 2 diabetes to Alzheimer's and some cancers.

Sugar is also now known to be highly addictive.

What makes it so pernicious is that it's very difficult to avoid, as manufacturers have hidden it in so many foods in order to make their products more appealing. It's in soups, pasta sauces, salad dressings and bread, as well as cakes and biscuits.

The good news is that you can beat your addiction - and prevent the long-term health risks of that addiction - with our Sugar Detox.

In Saturday's Mail, we set out the simple dietary rules for a three-day blitz that will give your body a complete break from all forms of sugar, and end your addiction.

Then yesterday we explained the best way to slowly re-introduce otherwise healthy foods, such as wholegrains, that form sugar when digested.

This is to increase your options and nutrient intake without triggering addiction. Today, we show you how easy it is to consume large amounts of sugar from even 'healthy' foods.

So why is cutting down on sugar so important to health?

We are all consuming far too many **sugar**-laden **soft drinks** and foods, such as cakes, biscuits, white bread and pastries.

Normally the protein, fibre and healthy fats (from foods such as olive oil, avocados and oily fish) in our food would slow down the speed with which our bodies metabolise sugar. This is because these healthy foods take longer to process and make their way through our system.

But because our modern diets are often mostly made up of refined carbohydrates, we end up with a pretty constant flow of sugar through the bloodstream.

This, in turn, triggers the pancreas to release the hormone insulin.

Insulin plays 'good cop' and 'bad cop' in your body. It's good when you eat something with sugar, because it jumps in to control the sugar. But it is also bad because it speeds up sugar's conversion into fat, depositing it in places where you don't want it, such as around your belly.

This is because insulin tries to control the sugar overload by telling the liver to convert some of this sugar into glycogen (so it can be stored for later in the liver).

There are also glycogen stores in the muscles, but once the muscle and liver stores are full, the liver will start to turn excess sugar into fats called triglycerides.

This fat is stored in the liver, but also in fat cells throughout the body, particularly the abdomen. So the more sugar we eat, the more insulin we release - and the more fat we store.

Furthermore, these triglycerides spill out into the bloodstream, clogging up arteries and increasing your risk of heart attacks.

Another problem is that your body can get so accustomed to pumping out quantities of insulin that your cells become 'insulin resistant', which means they are almost numb to insulin and don't respond quickly or effectively to the hormone.

As a result, you can end up with even more sugar in the bloodstream. What happens next is not entirely clear, but it's thought these sugars left unchecked in the bloodstream end up grabbing onto protein molecules throughout the body.

The resulting protein-sugar combinations (or to give them their technical name, advanced glycation end products), can end up in organs all around the body, where they appear to trigger an inflammatory response, causing tissue damage and premature ageing.

Hidden sugar in virtuous food

Virtuous food

Teaspoons of sugar

Sugar per portion (g)

Naked Green Machine anti-oxidant smoothie (per 450ml bottle)

Innocent Thai Vegetable Curry pot (serves 1)

Kellogg's Nutrigrain crunchy oat granola and cinnamon bar (40g)

Marks & Spencer fragrant crayfish and mango salad (serves 1)

Yeo Valley 0 per cent fat natural yogurt, per 160ml serving

Copella Cloudy apple juice (200ml serving)

51.75 12.5

9 14.8

12.8 20.6

13 3

2 4

3 5

These protein-sugar combinations are implicated in a host of age-related illnesses, including cardiovascular disease, kidney disease, Alzheimer's disease and the eye condition macular degeneration.

This all starts with insulin resistance. Worryingly, people with insulin resistance do not realise that they are sick because there are usually no symptoms at all.

Insulin resistance is very tough to fix. Exercise can help, and losing weight is considered to be the best possible move you can make. But it's not easy because insulin resistance very often triggers overeating.

It's thought this is because it makes your body resistant to the 'satiety' appetite hormone, leptin, which tells your brain to stop eating - in this way, sugar can also hijack your brain chemistry.

Another part of the problem is that sugar comes in many disguises, as we reveal here. The secret to preventing your sugar addiction returning is to limit your intake of these foods and drinks.

White flour

The refining process turns any grain into a form of sugar. When the bran and the germ are stripped from wheat, what remains is a simple carbohydrate that's rapidly absorbed by your body, just as a sugar molecule is.

With this quick absorption also comes a tendency to eat more, because the sugar floods straight into your bloodstream, meaning you don't have the same feeling of fullness as you might if you eat something containing fibre or protein (which take longer to digest, so slowing down the digestive process).

So we recommend avoiding - as much as possible - all refined white carbohydrates, especially white pasta, bagels, biscuits, wraps and bread.

Wheat contains a type of carbohydrate called amylopectin A, which is extra potent when the grain has been refined, because the lack of fibre means it converts to blood sugar more easily than any other carbohydrate.

Modern white flour (compared with the flour our ancestors ate) contains a type of protein called gliadin, which triggers a feel-good effect in the brain (it binds to opiate receptors there) and stimulates appetite. This is one of the reasons white-flour foods, such as pasta, are high on the list of comfort foods.

Another one to avoid is the white flour bagel. A plain, average-size bagel (which contains approximately 7g of sugar) is the equivalent in calories and sugar of five slices of white bread and sends your system into a sugar overload - setting you up to crash afterwards and reach for another unhealthy option.

White rice

The refining process takes wholegrain rice and turns it into sugar.

While the glycaemic index (the speed with which it is metabolised by the body) of rice does vary, based on the rice variety and cooking time, all forms of white rice will cause a sugar spike when you eat it. A study in the British Medical Journal in 2012 showed an 11 per cent increase in diabetes risk with each daily serving of white rice.

Opt instead for brown rice or other high-fibre grains, such as quinoa, which slow digestion and make you feel fuller more quickly.

Starchy vegetables

Some vegetables - such as sweetcorn, potatoes, sweet potatoes and butternut squash - are carbohydrates in disguise. The body processes carbohydrates into a form of sugar, so while starchy vegetables aren't as bad as refined carbohydrates, they do raise blood sugar levels.

However, the vitamins and minerals they contain make them better options than desserts, so don't forgo them in favour of cake. Just enjoy them in moderation.

Some fruit

While all fruit has health benefits, some - such as bananas, pineapple and watermelon - are very high in fruit sugars which, though natural, have the same impact on blood sugar levels as table sugar.

It is best to enjoy low-sugar fruits (apples or berries) instead, because just one taste can be enough to start sugar cravings.

Also avoid dried fruit, especially dates, cranberries, raisins and prunes.

The drying process concentrates the sugars and many dried fruits have added sugar, which will increase the jump in your blood sugar levels more quickly.

Fruit juice

Because juice has no protein or fibre, it's a super-quick way to spike your blood sugar levels. A piece of low-sugar fruit gives you the chew factor (which is more satisfying), plus the fibre that will help delay any sugar rush.

Fizzy drinks

All **fizzy drinks** - full sugar or diet versions - have no nutritional value. Most are merely a cocktail of chemicals and artificial colours in a steady stream of liquid sugar or more chemicals.

Although you may think diet **fizzy drinks** will sate your sugar cravings, they do the opposite.

Most are much sweeter than regular sugar and cause an imbalance in your tastebud sensitivity that prevents you from perceiving normal sweetness. This may make you want more sweet food to compensate.

One ten-year study, published in the journal Diabetes Care, found that the waistlines of people who consumed diet drinks expanded by 70 per cent more than non-drinkers' waistlines.

Those who drank more than two diet drinks a day were almost five times more likely to gain weight than those who didn't.

Artificial sweeteners

New evidence suggests artificial sweeteners may be as bad as real sugar when it comes to insulin and blood sugar levels. When sugar receptors (in the mouth, gut and pancreas) get tripped, they signal the brain to get ready for a sugar blast.

The body reacts in turn by absorbing more real sugar, triggering insulin production and turning sugar into fat.

Artificial sweeteners stimulate the same receptors that real sugar does, and with the same results - they may actually cause you to absorb more sugars according to a study at Purdue University in the U.S., published in the Quarterly Journal of Experimental Psychology. Our Sugar Detox has helped thousands of patients kick the sugar habit and reverse this destructive chemical cascade. It can work for you, too.

If you have a medical problem or blood sugar problem such as hypoglycemia, insulin resistance or diabetes, see your GP before starting any diet. The plan is not suitable for people who exercise intensively. Extracted from The Sugar Detox: Lose weight, Feel Great And Look Years Younger by Brooke Alpert and Dr Patricia Farris, published by Bantam Press at £8.99. To order a copy for £7.99 (incl P&P) call 0844 472 4157. © Brooke Alpert and Patricia Farris.

LOAD-DATE: January 22, 2014

LANGUAGE: ENGLISH

PUBLICATION-TYPE: Web Publication

JOURNAL-CODE: WEBDM

Copyright 2014 Associated Newspapers Ltd.
All Rights Reserved

Daily Mirror

January 20, 2014 Monday
Edition 1;
Northern Ireland

Cut down on sugar and BOOST YOUR KID'S BRAIN!; How reducing the huge amounts of sweet stuff in your child's diet could help them do better at school and transform their behaviour. By Amanda Killelea

BYLINE: Amanda Killelea

SECTION: FEATURES; Pg. 30,31

LENGTH: 1579 words

DOES your child throw tantrums and struggle to concentrate? Are they underperforming at school? It could be down to what's being dubbed the sweet white poison - sugar.

Most parents know that "E" numbers in food can affect behaviour. But experts now believe sugars added to the foods we eat every day could not only be contributing to the country's obesity epidemic, but are also linked to bad behaviour and poor performance at school.

We do need some sugar every day. It is a type of carbohydrate that provides energy for the body in the form of glucose, something the brain needs to function and the muscles need during exercise.

However, the World Health Organisation recommends that sugar forms less than 10% of a child's diet, yet many of the foods we buy at the supermarket for our children far exceed that recommended level. Some breakfast cereals contain as much as 25% sugar and yoghurts 13%.

Teenagers in particular can consume up to three times the recommended daily allowance of processed sugars just from sports drinks and fizzy pop alone.

As well as the health implications of eating so much sugar - an increased risk of cancer, diabetes and heart disease - some experts are warning that performance at school could be hampered by poor concentration, hyperactivity and even depressive symptoms. Nutritionist Jane Clarke says: "Sugar can have an enormous influence on behaviour. Children can be very sensitive to blood sugar, and if you take out those rapid blood sugar changes they become calmer.

"If children go to school on an empty stomach they don't perform as well and lack concentration. And if they are wired on processed sugars, then some can't get their head around concentration."

The biggest concern is that sugar is replacing other valuable nutrients that are needed for optimum academic performance.

Studying 5,200 high school students, researchers at the Department of Community Health in Halifax, Canada, found that the worse the nutritional quality of the diet, the worse the academic performance - regardless of socioeconomic factors and gender.

So what can parents do?

"It is much better for children to go to school on an egg, so an egg and soldiers or eggy bread - or toast with peanut butter - are much better than sugary cereals," says Jane.

"At lunch they definitely need protein, so a sandwich using wholegrain bread is ideal.

"If they have packed lunches they could take a flask with a casserole, soup, a pasta dish, or chunks of cheese with wholegrain bread. You need to steer clear of ready meals that are high in sugar.

"As much as you can, and when it is realistic for parents to do it, go back to cooking with really simple, unprocessed foods."

Action group Sustain has launched the Children's Food Campaign to improve the health of young people and protect kids from junk food marketing through good food and education in schools.

It has been lobbying supermarkets to remove sweets and chocolate from check-outs and last week Lidl became the first big store to sign up.

Campaign co-ordinator Malcolm Clark says: "There have been many studies looking at how a poor diet has significant effects on a child's behaviour, concentration and mood.

"Children with diets lacking in essential vitamins, minerals and essential fatty acids tend to perform worse academically, cannot concentrate and are more aggressive.

"Sugary foods, especially snacks, often have little nutritional benefit and mean that you are less likely to be consuming foods that will provide you with those micronutrients.

"Eating sugary foods often overrides the bodies satiety signals so you can't tell whether you are full or not. This is how a sweet tooth develops, the taste for sugary foods stays with you and the body thinks you need more food or sugar. There is some evidence that children performing badly in school and who are eating

sugary foods are much less likely to have the proper intake of fruit and vegetables and slower energy-releasing foods. And if they are drinking **sugary drinks** instead of water or milk they are not getting the nutrition and hydration needed to help them concentrate throughout the day."

The big challenge, according to Malcolm, is the fact that sugar comes in many forms, and it doesn't have to be labelled in that way.

He says: "On cereal bars and things like that the manufacturers put on the label that they are high in fibre or a good source of vitamins, but they will often have added sugar. Manufacturers don't splash that across the label.

"They are allowed to put health claims on because they are legitimate, but it doesn't help if you are giving your child something that you think is a healthier snack when actually they may not be as healthy as you think.

"Traffic light labelling is being introduced this year. This will help us to see if something is high in sugar.

"There is no doubt that sugar has an impact on the body.

"It can be like an addiction, and it's hard for parents because the healthy choice isn't always the easy choice."

TOMORROW: The diary of a mum who fed her kids low-sugar foods for a week

THE IMPORTANCE OF WATER

The amount of fluid your child consumes throughout the day is hugely important as dehydration leads to fatigue, irritability and poor concentration. Water is best, so avoid **sugary drinks** and fruit juices, unless diluted. Fruit juice can be diluted by half with water but a small glass (200ml) is adequate. Also, use a squash that has no added sugar or artificial flavours. Fluid intake can also include milk, tea and coffee.

BRAIN-BOOSTING FOODS

Try to include these healthy foods into your child's diet...

Tuna, salmon, halibut, mackerel, trout, walnuts, peanuts and almonds. These contain essential fatty acids which may slow the brain's ageing process and enhance problemsolving, concentration and memory.

Eggs.It's very important to try to include one egg a day in their diet, unless there are weight issues or high cholesterol. Eggs also contain choline, folate, iron, vitamins A and D and lecithin.

Extra virgin olive oil, nuts, seeds and avocado for a daily dose of healthy mono-unsaturated fats.

Wholegrain cereals, spinach, broccoli, oranges and green peas, which all contain folic acid.

Oatmeal and beans for glucose.

Berries, aubergine, cherries, apples, apricots, onions, broccoli, cabbage, tomatoes and grapes all contain antioxidants.

Limit refined foods, such as white bread, white pasta, biscuits and cakes. This will help to balance the body's blood sugar levels and reduce the "midday crash", which affects mood and energy levels.

Avoid food additives and food colourings, which have been shown to increase hyperactivity in children suffering from ADHD.

Avoid cereal bars that are loaded with added sugar. Instead, opt for a brand like Nakd Bar. They are made from raw ingredients and do not contain any added sugar.

If possible, avoid fruit juices that are full of sugar or dilute 50:50 with water. Also, Tropicana now has a 50:50 juice ready to drink. Having a glass of water or milk may be more beneficial.

Healthy meal plan...to boost your child's performance

With so much confusing information out there, it's hard for busy mums and dads to know what is a healthy diet for their children - and what isn't.

Nutritionist Wendy Griffin (Xpressnutrition@aol.co.uk) has come up with suggestions for feeding your kids a low sugar, but highly nutritious diet which could help boost their memory and concentration and avoid the mood swings often caused by sugar overload.

Breakfast

Skipping breakfast has been shown to have a negative effect on performance during the day. Studies have found that eating breakfast improves short-term memory and attention.

Try Weetabix, no added sugar cereals such as blue box Alpen, porridge oats and Shredded Wheat, or a poached or scrambled egg on wholemeal toast.

A banana and a glass of milk is OK if time is short.

Adding blueberries to the muesli or porridge may improve learning, thinking and memory skills.

Mid-morning snack

One or two oatcakes with some peanut butter.

One or two rice cakes with cream cheese or peanut butter.

A banana.

Snack-size packet of dried fruit.

Snack-size portion of mixed nuts and seeds.

Apple and three walnuts.

Apple slices with peanut butter.

Carrot sticks and hummus.

Celery and cream cheese.

Kiwi fruit.

Hard-boiled egg.

Lunch

Wholemeal bread roll, wholemeal sandwich or a wholemeal pitta.

Choose salad and a filling with plenty of protein such as cheese, tuna, chicken or turkey.

Include a piece of fruit or a plain low-fat yoghurt with blueberries, nuts or seeds.

Tuna pasta salad (spring onions, green pepper, cherry tomatoes).

Chicken drumsticks with sweetcorn and tomato salad.

A flask of homemade vegetable soup or stew.

Evening meal

Eat red meat no more than twice a week. Eat more chicken and turkey and introduce oily fish once a week.

Salmon or tuna fishcakes made with fresh fish and sweet potatoes, served with a green salad or broccoli spinach and green peas.

Poached salmon with a jacket potato and roasted peppers and courgetts.

Grilled chicken breast with sweet potato wedges and salad.

Spaghetti bolognese served with wholemeal pasta.

Omelette made with onions, tomatoes and mushrooms and any other preferred vegetables. Serve with a green avocado salad.

Snacks before bed

Oatcake with peanut butter or cream cheese. Small glass of milk.

A banana.

Peach, warmed in the microwave and topped with a little milk.

Wholegrain toast with a mashed banana on top.

Wholegrain toast with some melted cheese.

A poor diet can also affect their concentration and mood

LOAD-DATE: January 20, 2014

LANGUAGE: ENGLISH

PUBLICATION-TYPE: Newspaper

JOURNAL-CODE: DMR

Copyright 2014 MGN Ltd.
All Rights Reserved

MailOnline

January 20, 2014 Monday 2:03 AM GMT

PETER MCKAY: Prince Charles and a royal takeover by stealth

BYLINE: PETER MCKAY

SECTION: RIGHTMINDS

LENGTH: 1612 words

The Queen, who is 87, and the Prince of Wales, 65, are to 'job-share', we're told.

Charles will accompany his mother to Normandy this summer for the 70th anniversary of the wartime landings.

'We have been told this will probably be the Queen's last official foreign visit,' a French official involved in the D-Day ceremonies is quoted as saying.

HM's and the Prince of Wales's press offices are to be merged into a single communications team.

When the Queen's private secretary, Sir Christopher Geidt, was knighted (his second) in the New Year Honours List, his citation mentioned 'a new approach to constitutional matters . . . [and] the preparation for the transition to a change of reign'.

Like most Palace announcements, the tone is: 'Move along now, there's nothing to see here.' But behind the scenes, efforts are being made to manage public expectations.

Charles has good reason for wanting to seem more involved in the monarch's day-to-day life: the notion among some subjects that the crown should skip a generation next time, going to Prince William, now 31.

Allowed to flourish, this idea would sour the eventual succession of Charles, our longest-serving heir apparent.

For many, William and Kate are a more exciting prospect than Charles and Camilla. They come with less 'baggage' and the promise of a longer reign.

This isn't going to happen, though. God willing, Charles will succeed his mother. But his reign would be ruined if there was a public clamour for William and Kate.

There's less chance of this happening if he is seen to be shadowing the Queen, taking on her more gruelling engagements, his key staff merging with those of HM. Often, in the past, they have seemed at odds.

'For many, William and Kate are a more exciting prospect than Charles and Camilla'

The desired outcome of these changes is that Charles will be seen as a patient, deserving heir apparent. He'll have waited almost an entire natural life span, if not longer, to fulfil his destiny.

William will spend far less time as Prince of Wales. And he is likely to enjoy a far longer reign than Charles.

The question is: will taking on more of the Queen's roles enhance Charles's public standing, or otherwise? His public approval ratings should rise, all things being equal. Who could disapprove of a son helping his mother?

But all things are rarely equal. Charles and Camilla might be compared unfavourably with the Queen and the Duke of Edinburgh.

Charles is not a young 65. Neither does the Duchess of Cornwall seem a sprightly 66. In the wake of the 'job-share' arrangements, how will their May visit to Manitoba, Nova Scotia and Prince Edward Island in Canada be perceived?

Will their performance - and their reception by Canadians - be compared favourably, or unfavourably, with the April visit to Australia by the Duke and Duchess of Cambridge?

The delicacy of Charles's position arises from two factors. His historically long spell as heir apparent - and the dissolution of his first marriage, to Diana Spencer.

Some still believe he should be denied the throne because he married his mistress.

Those who think this feel that having to relinquish it to his elder son by Diana would be poetic justice.

But this can't happen unless Charles himself wants to remove himself from the succession. And there's no sign of that.

The problem would have been solved if the Queen had abdicated, as Queen Beatrix of the Netherlands did, aged 75, last year.

But if HM had abdicated at 75 - i.e., in 2001 - would Charles's succession have been easier?

At that point, his mistress had not yet become his second wife. There was then considerable ill-feeling against Camilla. Her relationship with the rest of the Royal Family didn't appear to be as close as it does now.

So why not simply abdicate now, when Charles and Camilla seem to be restored in public esteem?

The Queen said at her Coronation she would serve until she died, and she has given no indication since that this was not meant to be taken literally.

Why doesn't Charles take on most of the Queen's duties? 'Any formal announcement would be tantamount to declaring a Regency, and that would raise all kinds of difficult constitutional issues,' according to a royal official quoted yesterday.

But if Charles is taking on a substantial proportion of duties, isn't it a de facto Regency?

Yes, but to survive the monarchy must appear to be above rash decisions, or sudden swerves of direction. Instead, there must be a constant process of anticipating public moods, fancies and expectations - some of which will materialise, others not. It might seem casual, but it's anything but.

As the late sage Malcolm Muggeridge wrote in 1961 about the mutual machinations between monarchy and its subjects which keep the great vehicle running: 'The actual flywheels and pistons are discerned beneath the machine's quiet, reassuring hum.'

Ed Miliband's big speech last week got a lukewarm reception.

Talking about the next election, he said: 'It is about who we are as a country. And who we want to recover to be.'

And who we want to recover to be? Clumsy words suggest muddled thoughts.

Sofia Helin, who plays detective Saga Noren in The Bridge - the fashionable murder mystery series set in Denmark and Sweden, shown to 1.5 million viewers on BBC4 - acts the part of an Asperger's sufferer.

When a man smiles at her in a bar, in series one, she asks if he'd like sex.

This made a big impression on viewers here, but few found it surprising in Sweden, she says.

What they also might find odd is our fascination with the Seventies Porsche coupe, pictured, she drives in the show.

There's a lively online petrolheads' debate in progress over the exact model it is, and its engine capacity.

Feminists have a line of their own, too.

'The choice of a powerful car shows that women are entering the male arena as in all other aspects of life,' says Karen Pine, a professor of psychology at Hertfordshire University.

Which sounds like an utterance from always-state-the-obvious Saga, bless her Swedish socks.

Sugar is the new evil. Food manufacturers use it to bulk out their products and make them taste more tempting. The result: an epidemic of obesity.

But mostly it's common sense, isn't it? We limit the amount of sugar we give our children by withholding **fizzy drinks** and processed (as opposed to fresh) food. Or we should do.

Because some don't, there's talk about legislating against sugar. We might as well make laws against stupidity.

Sweets were scarce in my own youth.

But we were allowed occasionally to heat a poker in the fire until it was white hot and stick it in sugar, making our own candy. Delicious!

Ooh la la! Another Francois scandal

The mistress-swopping antics of France's President Francois Hollande is a story that just keeps on giving.

But all French presidents are said to see themselves as modern-day monarchs, despite their 1789 revolution. So having mistresses is in order.

'A court without women is like a year without springtime, like springtime without roses,' said King Francois I, who reigned from 1515 to 1547.

Married twice, Francois kept two official mistresses - Francoise de Foix, Countess of Chateaubriand; then her replacement, Anne de Pisseleu, Duchess of Etampes.

He once staged a beauty contest and, although etiquette demanded that his new queen, Eleanor of Austria, take the prize, he gave it to Anne.

Even today's scooter-borne, skirt-chasing Francois hasn't gone that far.

Don't let bosses off the hook...

The latest mis-selling scandal involves HomeServe, which provides insurance for burst pipes, broken boilers, blocked drains, pest infestations and other household emergencies.

For instance, linked up with the water companies, HomeServe sold insurance to cover burst pipes.

But 19 out of the 25 home insurers already cover this. To avoid prosecution, it agreed to pay a £34.5 million fine.

Why aren't the chief executives of all mis-selling companies and banks prosecuted, as well as being required to pay monster fines?

Naming and shaming might discourage this kind of fraud.

The BBC has launched an investigation into 'bullying' by a presenter and a producer working at an arts unit that makes Radio 4's Front Row and Radio 3's Night Waves.

Isn't it interesting how 'bullying' - usually considered a childhood problem - has now become a legitimate grievance in the adult world?

Either bullying has got worse generally, or those who enter employment in high-pressure occupations are more childish.

This week, Her Majesty's Chief Inspector of Constabulary Tom Winsor publishes his first report into the efficiency and effectiveness of the police.

He claims some citizens 'born under other skies' are likely to be committing murders that are not reported to the police. Honour killings being an extreme example.

'There are cities in the Midlands where police never go because they are never called . . . they just have their own form of community justice,' he says.

I hope he has some proposals for dealing with the problem. Or do we accept some areas of the UK reverting to medieval conditions if it satisfies some notional need for 'diversity'?

LOAD-DATE: January 20, 2014

LANGUAGE: ENGLISH

PUBLICATION-TYPE: Web Publication

JOURNAL-CODE: WEBDM

Copyright 2014 Associated Newspapers Ltd.
All Rights Reserved

The Sun (England)

January 20, 2014 Monday
Edition 1;
Ireland

Teacher ends pop culture at school;
EXCLUSIVE ; FIZZY DRINK BAN

BYLINE: MICHAEL DOYLE

SECTION: NEWS; Pg. 11

LENGTH: 239 words

A SECONDARY school has BANNED **fizzy drinks** in an on-going fight against flab.

Coolmine Community School in Blanchardstown, Dublin, has also introduced fitness classes to help pupils battle the bulge.

Principal Patricia McPhillips said they have introduced an initial two-week programme of new low-fat and nutritional meals in their canteen but they plan to extend it.

She told the Irish Sun: "We just feel that young people should be afforded the choice of healthier food options.

"It's very important for their personal development. We started it this week and the healthy living programme will itself run for two weeks.

"We are concentrating on food alternatives, with more nutrition and no salt meals being offered. We have removed **fizzy drinks** from the canteen and the school vending machine and that move is permanent.

"Our vending machines will have water, yoghurts and drinks such as chocolate milk."

Social Protection Minister Joan Burton was on hand to launch the initiative.

The school, which has 1,100 pupils, has also introduced a 15-minute discussion on healthy living every day and exercise will also play a key part.

Ms McPhillips said: "A fitness class has been offered to all students regardless of their level of fitness each Monday from 4 to 5pm and Wednesday from 1 to 2pm."

She added: "I think we're one of the first schools in the country to go down this road and we're quite sure it will be a success."

michael.doyle@the-sun.ie

LOAD-DATE: January 20, 2014

LANGUAGE: ENGLISH

GRAPHIC: Bin bad habits ... McPhillips
Let's eat the right things ... Minister Burton and pupils

PUBLICATION-TYPE: Newspaper

JOURNAL-CODE: SUN

Copyright 2014 NEWS GROUP NEWSPAPERS LTD
All Rights Reserved

MailOnline

MailOnline

January 19, 2014 Sunday 11:58 PM GMT

The sugar tsars 'in bed' with confectionery giants: Five members of committee tasked with battling obesity epidemic have 'worryingly close' ties

BYLINE: ARTHUR MARTIN

SECTION: NEWS

LENGTH: 535 words

- Experts under fire for being paid to advise firms like Coca Cola and Mars
- Food giants being urged to cut sugar in products as it is 'the new tobacco'
- Doctors and academics say levels must drop by at least 30 per cent

Experts who advise the Government on sugar consumption were under fire last night after it was revealed they receive funding from confectionery giants.

Five out of eight members of a committee tasked with helping to tackle Britain's obesity epidemic have 'worryingly close' ties with the food industry, it was claimed.

They include chairman Professor Ian Macdonald - one of the country's leading nutritionists - who works as a paid advisor for Coca-Cola and Mars.

Yesterday critics said those who sat on the so-called 'sugar committee' could not be trusted because many of them are 'in bed' with food manufacturers.

The row comes as food giants are being urged to cut sugar in products amid fears it has become the 'new tobacco'.

Doctors and academics say sugar levels must be reduced by at least 30 per cent to halt a wave of disease and death.

The typical Briton consumes 12 teaspoons of sugar a day - and there are nine in a can of Coca-Cola and eight in a 51g Mars Bar.

Professor Macdonald is paid £6,100 to sit on two advisory boards for Coca-Cola and also receives a larger payment for advising Mars.

But in 2009 he faced concerns over a potential conflict of interest and stood down until 2012.

His research at Nottingham University has now received more than £1million in the past three years from the food industry, including £300,000 from Mars.

Funding also comes from Unilever - the world's largest ice cream manufacturer.

The disclosures yesterday prompted calls for the scientist to resign from the panel because of an 'unacceptable' conflict of interests.

Simon Capewell, from campaign group Action on Sugar, said: 'It's like putting Dracula in charge of a blood bank.

If Ian Macdonald doesn't step down, there will be real concerns that their recommendations will be prejudiced by commercial factors rather than scientific public health priorities.'

Cardiologist Aseem Malhotra added: 'I don't think that anyone who is in bed with the food industry should be advising the Government.'

Other committee members with ties to the sugar industry include Ian Johnson, a consultant for Swiss chocolate multinational Barry Callebaut.

Professor Ian Young and Professor Julie Lovegrove have also received funding from the sugar industry, while David Mela is an employee and a shareholder of Unilever. All eight members are paid for their work on the committee.

In an interview with Channel 4's Dispatches programme, Professor Macdonald denied that he is too close to food companies.

He claimed it was important to confer with the industry and said he never discussed any of his government work with Coca-Cola or Mars.

Last night Public Health England said advisers must declare any potential conflicts of interest and be 'independent and professionally impartial'.

A spokesman said there were processes in place to ensure 'transparency and integrity'.

A report to be published later this year by the committee on the health impact of sugar consumption could have a massive impact on the food industry.

LOAD-DATE: January 20, 2014

LANGUAGE: ENGLISH

PUBLICATION-TYPE: Web Publication

JOURNAL-CODE: WEBDM

Copyright 2014 Associated Newspapers Ltd.
All Rights Reserved

THE SUNDAY TIMES

The Sunday Times (London)

January 19, 2014 Sunday
Edition 1;
National Edition

Sugar advisers have their cake and eat it

BYLINE: Jon Ungoed-Thomas; Kate Mansey

SECTION: NEWS; Pg. 12

LENGTH: 1188 words

IN THREE months' time, some of the country's leading nutrition experts will assemble for a crucial meeting to deliberate one of the most contentious food issues of recent times: how much sugar should we be eating?

On one side of the debate is a new and vociferous health lobby - Action on Sugar - which claims that sugar is "the new tobacco" and its threat has been underestimated for years.

On the other is the food industry. A presentation by the World Sugar Research Organisation, an industry group, obtained by Channel 4 Dispatches, says it will fiercely resist proposals for any drastic reductions in sugar intake.

It blames the attack on sugar on health campaigners and the "anti-capitalist camp" who want "state control" of food corporations.

It advises an attack on the "bogus" science and warns: "The reputation of sugar as a food is being attacked."

In the middle of this battle is the tortuously named carbohydrates working group of the Scientific Advisory Committee on Nutrition (SACN), the panel of experts that is advising the government on the health impact of sugar consumption. Its recommendation on whether the government should advise people to eat less sugar could have a massive impact on the food industry.

Yet it has now emerged that five members of the eightstrong panel have links with the food industry.

Professor Ian Macdonald, the chairman, has paid advisory roles with Coca-Cola and Mars. David Mela is a senior scientist for Unilever, which owns Wall's and Ben & Jerry's ice cream.

Ian Johnson is a consultant for Barry Callebaut, a Swiss multinational cocoa and chocolate company. Two other members, Professor Ian Young and Professor Julie Lovegrove, have received funding from the sugar industry.

Public Health England, which oversees the work of SACN, says any recommendation on sugar will be rigorously independent.

There are, however, growing concerns about the influence industry has on public health issues. Ministers were accused this month of caving in to the powerful drinks lobby over plans to impose a minimum price for alcohol. An investigation by the British Medical Journal found that, since the coalition took power in May 2010, the Department of Health has had 130 meetings with representative of the alcohol industry.

The government was also criticised after putting plans to introduce plain cigarette packaging in England on hold after a lobbying campaign by the tobacco industry amid concerns about the role of Lynton Crosby, a Tory adviser whose company has worked for tobacco firms. After an outcry from health experts and medical organisations, the government was forced to launch a fresh review of the packaging.

Under World Health Organisation (WHO) rules, ministers are obliged to protect public health policies from the influence of cigarette companies. It means there are very few meetings with the tobacco industry. There are now demands for stricter controls on the influence of the food industry in the decision-making process.

Aseem Malhotra, a cardiologist and science director of Action on Sugar, said: "We must remove conflicted scientists and organisations from determining our dietary advice." He said poor diet contributed to more disease than smoking, physical inactivity and alcohol combined and it was time to put diet "at the centre of preventative health policy". Concerns about whether policy makers are receiving independent advice on sugar intake go much wider than Macdonald's panel. Albert Flynn, an Irish academic, looked at the links between sugar and obesity for an EU food safety body, the European Food Safety Agency (Efsa).

Yet at the same time Flynn was advising Kraft, owner of Cadbury, and working for the International Life Sciences Institute (Ilsi) Europe, whose members include Coca-Cola, Mars and Kellogg's. The 2010 report by Flynn's panel concluded there was not sufficient evidence to propose a recommended limit for sugar consumption and there was no evidence of a "positive correlation" between total sugar intake and obesity.

Efsa said Flynn had correctly declared his interests and the agency had a robust system to ensure "the impartiality of its scientific advice".

Suspicion about the food industry's links to key nutritional scientists dates back to 1998, when the WHO and the Food and Agriculture Organisation convened a meeting of scientists to look at carbohydrate in the diet.

The meeting set no limit for sugar consumption, but it later emerged that it had been partly funded by Ilsi, the industry research group, and the World Sugar Research Organisation (WSRO), a sugar industry body.

Jim Mann, one of the experts at the meeting, said: "When we arrived [we were] told very clearly that it would be inappropriate to say anything bad about sugar."

WHO nutrition experts have since been very sensitive to allegations of conflicts of interest. The Sunday Times revealed last month that leading WHO scientists now want to halve daily sugar intake from 10% to 5% of energy consumption.

Macdonald said be believed it was right to engage with industry. He said he provided a wide range of advice to both Mars and Coca-Cola on nutrition and human health. He added that he also provided specialised advice on pet care to Mars, which also manufactures pet food. Johnson said his advice to Barry Callebaut involved plant chemicals and was not connected to sugar.

Ian Young said his disclosed research funding was "several years ago" and he currently received no research grants from the food industry.

Barbara Gallani, director of regulation, science and health for the Food and Drink Federation, said: "As long as you have a robust system of declaring interests, then I think we can let the best scientists in their field address health issues."

Dispatches: Are You Addicted to Sugar? will be broadcast at 8pm on Channel 4 tomorrow

Sour taste: how firms fund top nutritionists Source: Declaration of interests in 2012 annual report of Scientific Advisory Committee on Nutrition

IAN MACDONALD

Professor of metabolic physiology at Nottingham University Disclosed interests: Adviser to Mars and Coca-Cola. Research funding from Unilever

DAVID MELA

Senior scientist, nutrition and health, Unilever Disclosed interests: Employee of Unilever and shareholder

IAN JOHNSON

Research leader at the Institute of Food Research Disclosed interests: Consultant to Barry Callebaut, Swiss chocolate manufacturer

IAN YOUNG

Director of Centre for Public Health at Queen's University, Belfast Disclosed interests: Has received research funding from Unilever and the Sugar Bureau

JULIE LOVEGROVE

Professor of metabolic nutrition at Reading University Disclosed interests: Has received research funding from Unilever and Sugar Nutrition UK

Yes 49% No 40% Modest risk 45% Severe risk 43% How much of a health risk does a diet high in sugar pose? Do you think labels clearly show sugar levels in food? Don't know 3% Don't know 11% Minor or no risk 9% Support 38% Oppose 49% Do you eat too much sugar? Probably eat too much 48% Probably eat about the right amount 46% Would you support new taxes on sugary products? YouGov questioned 1,957 adults, January 16-17 Don't know 13% Don't know 6%

LOAD-DATE: January 19, 2014

LANGUAGE: ENGLISH

GRAPHIC: Sugar consumption should be cut dramatically, according to the World Health Organisation GETTY/EMMA KIM

PUBLICATION-TYPE: Newspaper

JOURNAL-CODE: STS

Copyright 2014 Times Newspapers Limited
All Rights Reserved

theguardian

The Guardian - Final Edition

January 18, 2014 Saturday

Saturday: Is this the end for fruit juice?: For decades fruit juice has been seen as a healthy option. Then this week a primary school in

Dagenham banned it after claims that it's as bad for you as Coca-Cola. So why is it still recommended as one of our five a day?

BYLINE: Emine Saner

SECTION: GUARDIAN SATURDAY COMMENT PAGES; Pg. 30

LENGTH: 1307 words

This week, it looked as if fruit juice might finally lose its claim to healthiness and be put into the same category as **fizzy drinks**. It emerged that a headteacher, Elizabeth Chaplin, who runs Valence primary school in Dagenham, wrote to parents about a new rule to confiscate juice cartons from children's lunch boxes. Instead, pupils would only be allowed to drink water.

Days earlier, Susan Jebb, a government advisor and head of the diet and obesity research group at the Medical Research Council's Human Nutrition Research unit at Cambridge University, told the Sunday Times that the government's official advice that a glass counts towards your recommended minimum five-a-day servings of fruit and vegetables should be changed.

"Fruit juice isn't the same as intact fruit and it has as much sugar as many classical **sugar drinks**," said Jebb, who has stopped drinking juice. "It is also absorbed very fast, so by the time it gets to your stomach your body doesn't know whether it's Coca-Cola or orange juice, frankly. I have to say it is a relatively easy thing to give up. Swap it and have a piece of real fruit. If you are going to drink it, you should dilute it."

This comes on top of a year or so of stories about the high sugar content of fruit juice. The same US scientists who warned about the use of high-fructose corn syrup in **fizzy drinks** have now turned their attention to juice. "Fruit juice and smoothies are the new danger," Barry Popkin, professor of nutrition at the University of North Carolina, told the Guardian in September. Work by Dr Robert Lustig - whose book Fat Chance: the Bitter Truth about Sugar received much attention last year - and studies such as one published in the British Medical Journal in the summer, which found fruit juice is associated with an increased risk of type 2 diabetes, are starting to make people realise that fruit juice may not be as wholesome as they once believed.

So why is fruit juice still being pushed as a healthy option? "You can't trust government health advice," says Joanna Blythman, author of What to Eat. "They have the same advice that they've been recycling for 50 years and rarely change it. It's embarrassing to admit they've made a mistake."

Does she drink juice? "I don't, really - not in any great quantity," she says. At one point, she says, in the late 1980s and early 90s, she was "a very enthusiastic orange juicer. I remember coming back from the States, where everyone juices like mad, and I got a juicer. Then over the last couple of years there has been more and more evidence that sweet juices are basically just fructose, and have a similar effect on the body to fizzy and **soft drinks** in terms of **sugar**."

The juice industry has long enjoyed a healthy image. Anything to do with fruit, says Blythman, "has always been used to put a halo of health around dubious products that don't merit it. That's business as usual for the food industry."

For all their reliance on phrases such as "100% pure" and "pure squeezed", many of the big commercial orange juice manufacturers make a processed product, as detailed by Alissa Hamilton in her 2009 book Squeezed: What You Don't Know about Orange Juice.

In the early 20th century, juice was mainly sold in cans. During the second world war, the US government commissioned scientists to develop a product that would supply vitamin C to soldiers overseas. "That's when research into developing a frozen concentrate that people would actually like started," says Hamilton. Until then, it had been fairly tasteless - the concentrating process removed the water, but also the natural chemicals that gave orange juice its taste. "They started adding fresh juice to the concentrate and that made it taste good. The discovery was too late for the war, but after the war that's when orange juice started to become really popular."

However, as the market grew, it was becoming too expensive to use fresh juice to add flavour back to concentrate. "They developed the technology around the 1960s to capture and break down the essences and oils that were lost when the juice was concentrated, and came up with these things called flavour packs."

Producers of pasteurised orange juice began storing their juice in vast tanks. In order to keep it "fresh", the product had to be stripped of oxygen. Once this had been done, the juice could be stored for up to a year. The only problem was that this process also removed much of the taste. "You need flavour packs to make it taste like anything we know as orange juice," says Hamilton.

So, does she still drink juice? "I actually never did," she says. "I try to eat the whole thing. If I have an orange, I don't even stop at the fruit - I eat the pith, the peel. Juicing anything would not be my choice."

For most of us it is, though, and it is not obvious that any of the sugar scare stories are affecting the fruit juice market yet. In its latest report, the research company Mintel found that 83% of people drink fruit juice, a juice drink or smoothie at least once a week. It also estimates that the market will grow by 13% by 2018. It found 34% of consumers were concerned about the amount of sugar, but "a striking 76%" believed juice and smoothies to be healthy.

As part of its end-of-year "top products" survey, the retail trade journal the Grocer found a mixed picture for juice brands. The leading brand, Tropicana, experienced a downturn in sales of 5.4% throughout 2013, though sales of Innocent smoothies, owned by Coca-Cola, were up more than 7%. However, Innocent was one of the brands highlighted last year as containing high levels of sugar: a 250ml serving of its pomegranate, blueberry and acai smoothie contains 34g of sugar, around the same as a 330ml can of Coke.

"I think the current coverage about fruit juice and sugar will have an influence on consumers," says Heidi Lanschutzer, food and drink research analyst at Mintel. "The question is whether it's a short- or long-term impact." She says this will depend on how ongoing the coverage is, and whether more schools ban juice, though the biggest impact will be if the government takes Susan Jebb's advice and removes it from the five-a-day list. This, she says,"is one of the market's biggest selling points - if the market is not allowed to use that any more, that will definitely have an impact."

Not everybody is racing to demonise juice just yet. "It's about portion size. 150ml of fruit juice is perfectly acceptable as one of your five-a-day," says Azmina Govindji, dietitian and spokesperson for the British Dietetic Association. "But we would suggest you have it with a meal so it doesn't make your blood sugar go up too quickly. I think the difficulty comes when people think of fruit juice as being a really healthy drink and having half a pint, or having it throughout the day, or where children are being brought up on large amounts.

"The key message is that small amounts - a 150ml glass is quite small - as part of a healthy varied diet is fine. You get fluid and vitamin C but you need to be aware that it does contain sugar. If you can, always choose fresh fruit and veg (over juice). You're going to get fibre, more nutrients and you're likely to have fewer calories."

Does she think the advice on juice being part of the five-a-day will change? "I think what needs to change is advice on portion size."

Blythman, meanwhile, understands that the mixed messages about juice are perplexing for consumers. "People are thoroughly confused," she says. "But I think (growing awareness of sugar levels) will have an effect. The simplest way to put it is: eat whole fruit, don't drink juice."

Captions:

Not so innocent

Fruit juice sales are still strong, but supermarkets and producers will be watching to see if recent negative coverage will change people's attitudes - and government advice

Photograph: Deborah Pendell

LOAD-DATE: January 18, 2014

LANGUAGE: ENGLISH

PUBLICATION-TYPE: Newspaper

Copyright 2014 Guardian Newspapers Limited
All Rights Reserved

DAILY MAIL (London)

January 17, 2014 Friday

I'LL QUIT SUGAR FOR A DAY SAYS PM...WITH MY WIFE'S HELP

BYLINE: BY DAILY MAIL REPORTER

LENGTH: 189 words

DAVID Cameron has promised to give up sweet treats for a day - with the support of his wife - to back the campaign against obesity.

He said he will try to take up the challenge to have no sugar and no **sugary drinks** on one day this week.

The Prime Minister acknowledged that diabetes and obesity were major health concerns'.

Senior Labour MP Keith Vaz attempted to enlist Mr Cameron's support in the war on sugar'.

He warned voluntary measures to ensure manufacturers cut the sugar content in food and drinks by up to 30 per cent had not worked, adding that obesity and type 2 diabetes were twin epidemics'. After Mr Vaz asked him at Prime Minister's Questions to take up the sugar-free challenge, Mr Cameron replied: I'm sure that last proposal would have the strong support of Mrs Cameron so I will take that up if I possibly can.

Can I commend you for... speaking out on the issues of diabetes and obesity with such consistency, because they are major health concerns for our country.

We are taking them very seriously. We are rolling out the NHS health check programme to identify all those between 40 and 74 at risk of diabetes.'

© Daily Mail

LOAD-DATE: January 16, 2014

LANGUAGE: ENGLISH

PUBLICATION-TYPE: Papers

Copyright 2014 Associated Newspapers Ltd.
All Rights Reserved

The Daily Telegraph (London)

January 16, 2014 Thursday
Edition 1;
Scotland

Sugar ban would please PM's wife

SECTION: NEWS; Pg. 12

LENGTH: 172 words

David Cameron said his wife would "strongly support" him giving up sugar for a day to raise awareness about obesity and type 2 diabetes.

The Prime Minister said he was keen to accept a challenge to have no sugar and no **sugary drinks** on one day this week, and that his wife Samantha would back him.

Keith Vaz, a Labour MP, attempted to gain his support in the "war on sugar" during Prime Minister's Questions in the Commons yesterday.

Referring to a new scheme aiming to reduce the sugar content of food and drinks, Mr Vaz asked: "Will you meet with a delegation of health experts to discuss this issue and can we enlist your support in the war on sugar by asking you to give up sugar and **sugary drinks** for one day this week?"

Mr Cameron replied: "I'm sure that last proposal would have the strong support of Mrs Cameron so I will take that up if I possibly can. Can I commend you for raising this issue, for speaking out on the issues of diabetes and obesity with such consistency, because they are major health concerns for our country."

LOAD-DATE: January 16, 2014

LANGUAGE: ENGLISH

PUBLICATION-TYPE: Newspaper

JOURNAL-CODE: DTLscot

Copyright 2014 Telegraph Media Group Limited
All Rights Reserved

The Telegraph
telegraph.co.uk

January 15, 2014 Wednesday 2:40 PM GMT

Primary school angers parents by banning fruit juice;

Pupils at Valence Primary School in Dagenham, Essex - some as young as five - are only being allowed to drink water and dinner ladies are confiscating any juice

BYLINE: By James Edgar and agencies

SECTION: NEWS

LENGTH: 780 words

A primary school has angered parents by banning children from drinking fruit juice as part of a new health drive.

Pupils at Valence Primary School in Dagenham, Essex - some as young as five - are only being allowed to drink water and dinner ladies are confiscating any juice.

The ban comes days after Professor Susan Jebb, the government's leading adviser on obesity, warned people should stop drinking orange juice because it contains as much sugar as Coca-Cola.

In a letter to parents, headteacher Elizabeth Chaplin said the new rule was part of a campaign to win a London Healthy Schools Award.

She wrote: "As part of the school's action plan it was decided that our first step would be to ensure equality for all the children and that everyone would be offered water to drink at lunch time.

"You will no longer have the worry about providing a drink as we will supply the water.

"Please do not put any drink other than water in your child's packed lunch box as they will not be able to drink it at school."

But some angry parents said children who do not like water are being forced to go the whole six-hour school day without a drink.

Carly Nunn, 26, claimed her five-year-old daughter, Teagan, had her drinks snatched by a dinner lady.

She said: "She put a straw in the carton and took one sip and it was taken off her and put in the bin.

"The next day, I put it in a bottle, but it was tipped onto the table to check the colour."

Ricky Biggs, 31, has kept his son Ronnie, four, off school because of the ban.

He said: "He doesn't drink water and when he's been in hospital they've given him squash. How can a hospital say squash is healthy, but a school say it isn't?

"We spoke to the head about it and she just offered us a transfer form."

Ronnie's mother, Kelly Debenam, added: "The letter makes it sound like they're doing the parents a favour, but they're not.

"Other schools don't have this, it's not compulsory, it's just them trying to get this award."

Head Mrs Chaplin said children questioned over their drinks were "more than happy to confirm or demonstrate the water content".

She said: "Regrettably our attempts to promote a healthier lifestyle have been received negatively by a very small minority.

"We are undertaking a review of the whole lunchtime provision and the review group will include representatives from the parents, pupils, staff and governors."

Earlier this year American obesity expert Robert Lustig said sugary fruit juice was worse for health than **fizzy drinks**.

The London Healthy Schools Award scheme is sponsored by London Mayor Boris Johnson.

Schools will be graded bronze, silver or gold for improvements made to pupils' health.

Here is the letter in full:

Dear Mums, Dads and Carers

Happy New Year!

I hope you all had an enjoyable Christmas break and that you are prepared for a very eventful year ahead whatever the weather.

I just wanted to ensure the message that went home in the December newsletter regarding drinks with packed lunches was read by all parents.

We have always promoted and encouraged our children to adopt healthy lifestyles.

We held the healthy schools award for many years.

The government and the Mayor of London are urging all schools to take the lead in reinforcing the benefits of eating and drinking healthily.

They are going as far as providing the funding for all children in reception, year one and year two to have free school meals from September if parents wish to take up this offer.

Our school dinner menu is nutritionally balanced and the only drink available to the children is water.

As part of our application for the London Healthy Schools Award, we have been asked to consider how we are reinforcing the message for those children who bring in a packed lunch from home.

As part of the school's action plan it was decided that our first step would be to ensure equality for all the children and that everyone would be offered water to drink at lunch time.

You will no longer have the worry about providing a drink as we will supply the water.

Please do not put any drink other than water in your child's packed lunch box as they will not be able to drink it at school.

In addition, we are recycling all our food waste from the kitchen and school dinners and are paying for this service.

In order for you to check what your child has eaten and to reduce our recycling we are sending all the waste from packed lunches home.

All children have access to water at all times throughout the day and we encourage children to drink regularly.

A healthy child is a happy child and we hope that by working together we can achieve our aims.

Thank you for your continued support.

Kind regards

Mrs Chaplin

Headteacher

LOAD-DATE: January 16, 2014

LANGUAGE: ENGLISH

PUBLICATION-TYPE: Web Publication

JOURNAL-CODE: WEBDTNS

Copyright 2014 Telegraph Media Group Limited
All Rights Reserved

THE TIMES
The Times (London)

January 15, 2014 Wednesday
Edition 1;
National Edition

Pass the OJ - I'm panic fatigued

BYLINE: Carol Midgley

SECTION: T2;FEATURES; Pg. 2

LENGTH: 167 words

Until this week I'd been feeling secretly smug about this sugar panic. My daughter, aged 9, has never had a **fizzy drink**. Ha, take that Perfect Parents. She tasted it once, spat it out saying bubbles felt "wrong" and drinks nothing but fresh orange Juice. Which turns out to be the Devil's broth. It's full of sugar, says the obesity czar, which is the "new alcohol" and the "new tobacco". I knew "made from concentrate" was rubbish but, come on, do I at least get smug points for the "freshly squeezed" Juice I buy? No. It can contain five teaspoons of sugar a glass. Sigh.

Just a thought, but does the Government ever worry about creating panic fatigue? I know sugar's evil and everything pleasurable should be banned but I struggle to take fresh OJ seriously as The Enemy. I drank little but Vimto for the first 14 years of my life and last time I checked still seemed to be alive. I tell myself that orange Juice is as dangerous as a ciggie but, sorry, can't keep a straight face. I'll try again tomorrow.

LOAD-DATE: January 15, 2014

LANGUAGE: ENGLISH

PUBLICATION-TYPE: Newspaper

JOURNAL-CODE: TIM

Daily Mirror

January 14, 2014 Tuesday
Edition 1;
Northern Ireland

CHOC-OUT;
Supermarket to ban sweet displays at tills to combat kids' pester power

BYLINE: RUKI SAYID

SECTION: NEWS; Pg. 18

LENGTH: 304 words

LIDL will today axe sweets from the checkout at all 600 stores and replace them with healthy snacks such as fruit and nuts.

The discount supermarket said it made the decision after 70% of parents revealed they were pestered for sugary treats as they queued with their shopping.

Two-thirds admitted to caving in and buying them, while 25% said their children enjoyed healthier alternatives if they were available.

Ronny Gottschlich, managing director of Lidl UK, said: "We know how difficult it can be to say no to pester power, so by removing sweets and chocolates from our tills we can make it easier to reward children in healthier ways."

Lidl's move came after new pressure group Action on Sugar called for sugar in food to be cut by 30% to tackle the UK's obesity epidemic.

Malcolm Clark, of the Children's Food Campaign, said Lidl was showing how supermarkets could also help to make us healthier.

BURDEN He said: "The onus is now on other supermarkets and retailers to follow suit and we and the British Dietetic Association will keep up the pressure for them to do so."

Last week Action on Sugar expert Prof Simon Capewell described sugar as "the new tobacco".

He added: "**Sugary drinks** and junk foods are pressed on unsuspecting parents and children by a cynical industry focussed on profit not health.

"The obesity epidemic is generating a huge burden of disease and death."

Figures show 25% of adults in England are obese. That is set to rise to 60% of men, 50% of women and 25% of children by 2050.

The Department of Health said 38 food giants had already pledged to cut calories in their products.

Coca-Cola said it aimed to slash calories in its products by 30% while Tesco made a similar commitment on its own-brand drinks. Mars has reduced its single chocolate portions to no more than 250 calories.

ruki.sayid@mirror.co.uk

LOAD-DATE: January 14, 2014

LANGUAGE: ENGLISH

GRAPHIC: FRESH Apples at tills
healthy choice Lidl's tills will be sweet-free

PUBLICATION-TYPE: Newspaper

JOURNAL-CODE: DMR

The Guardian

January 14, 2014 Tuesday

G2: Life without sugar: Many of us are addicted to sugar. Want to break the habit and get those no-good empty calories out of your life? Zoe Williams explains how to conquer your cravings in 11 easy steps - even if you really, really fancy a Mars bar

BYLINE: Zoe Williams

SECTION: GUARDIAN FEATURES PAGES; Pg. 6

LENGTH: 1657 words

1Know thine enemy

It is droll to observe nutritional advice at the public health level; governments and their agencies always approach obesity as though it were a problem of information or - in the popular phrasing - "awareness". If people only knew how much sugar there was in a Twix, they would simply eat something else.

This knowledge deficit doesn't exist: you won't meet anybody on Earth more intricately apprised of calorie content than someone who is obese. The only people who genuinely don't know shit from sherbet are the authorities themselves, who conflate the problem behaviour - in this case, excess sugar - with the people they perceive as causing them a problem. People, for instance, who drink **fizzy drinks** (except prosecco). So they'll preach two behaviours that are near identical, nutritionally speaking, as the opposing pillars of good and evil. "Drink a fruit juice; do not drink a Lilt. Drink a smoothie; do not drink a McDonald's milkshake." Finally, some exasperated nutritionist will pop up and say: "This is all sugar that doesn't fill you up and doesn't even slake your thirst particularly well." And everybody pounces on them and calls them a quack, even though they are right.

It is all sugar; it all does the same thing to your bloodstream, and it all begets an appetite for more of itself,

as do fags and booze. Leaving aside the thumping idiocies of the Department of Health's Change4Life campaign, the only real fault line is: do you think of it as an addiction or not? If you merely think of it as something you like a bit too much, there is no more a need to excise it from your diet than there is to stop using Twitter just because it drains your time and means you'll never amount to anything.

There is only one step necessary for you, the step of "less".

If you do see it as an addiction, then cutting down won't be enough, and I refer you to steps two through 11.

2Cold turkey

"But what if," I said to Frankie from Pure Package, a company that sends perfectly balanced meals, daily, to people with money, "you just really, really fancy a Mars bar?" I have been calling diet people (for work!) since

Atkins was fashionable. There will be those among you who don't even remember the outbreak of war against wheat, who weren't even alive in a time before bread was the enemy. Think on that.

Anyway, what always charms me is their preposterous alternatives.

So you might say: "What I really love is a buttered crumpet," and they'll go: "That's easy! You can grind some cashew nuts into a sort of makeshift butter and spread it on some kale." That was my motivation in putting the Mars bar question to Frankie, but she wasn't biting. "The only way to stop sugar cravings is to treat it like an addiction and go cold turkey. There's nothing to soften that blow."

3Beware of fruit

Frankie again: "Fruit has been given a halo so we end up eating too much of it." In fact, there's nothing inherently great about fructose. Sure, fructose is better than glucose because it comes accompanied by fibre and vitamins. But in and of itself, it is not better, and "should" (still Frankie), "be accompanied by seeds or nuts. The effect would be to slow down the insulin spike that the fruit brought to the bloodstream. Overall, it should be, not limited, but not seen as something you can eat all the time in any quantity." Generally, the higher the water content, the less the sugar hit, so oranges are better than bananas. Oranges are also better than mangoes. Oranges, it turns out, actually are the only fruit.

4Also beware of (some) naturopaths

Some definitions: "dietitian" is the only term that is subject to professional requirements. Anyone can be a nutritionist. "Naturopath" is what nutritionists call themselves when they want to sound a bit more new-age than they already do. The middle term attracts the most scepticism, based on the presumption that just because your field isn't professionally accredited, you do not know anything and you can't process information. People make it about journalists quite a lot as well; this presumption is mistaken.

That said, I interviewed lifestyle guru Carole Caplin (right) once, and she asked me to do something the next day, and I said: "Unfortunately, tonight I'm going to get completely drunk, so I most probably won't want to do Pilates/circuit training/zumba tomorrow." She fixed me with a beady eye and said: "I try not to eat too much chocolate, but sometimes I go mad. The other day, I ate something like eight squares of Green & Black's. And afterwards I felt terrible, I had a headache, the shivers, I couldn't get out of bed. Whereas if I'd only had two squares, I'm sure my body would have coped with it."

Here's the thing: I'm not convinced that really happened. I think she was using chocolate as a metaphor for booze, in an attempt to find some joint language that we would both understand.

5Give up alcohol

Many drinkers think they don't have a sweet tooth; indeed, they are faintly derisive of people who do. In fact, they get all their sugar from alcohol and if they ever gave it a rest for even two days, they would realise they have an incredibly sweet tooth.

6Gary Barlow

You know that joke, "how do you know when someone has an iPad? Because they tell you"? This adapts very well to the Take That tax avoider. How do you know how Gary Barlow lost five stone? Because he tells you. In precis, he realised, after years of trial and error, "that he doesn't have the kind of body that allows him to eat whatever he likes" and thereafter, cut out sugar, alcohol, any solids at all after 2pm, and refined carbohydrates. I know! As if he couldn't get any more charismatic.

The point is that Barlow is now at the dead centre of the

sugar-free, wheat-free eating crowd, and if you ever want to know how to make a cake out of hemp, Google "Gary Barlow" + "cake out of hemp".

7Grain differentiation

The whole issue of carbohydrates and sugars has been maybe irredeemably muddied by people such as Sarah Ferguson eating spelt, and then going: "I went wheat-free and the weight fell off me," and everybody going: "Wow. That's some strange ju-jitsu, considering spelt is just a variety of wheat."

Almost all carbohydrate converts to glucose, except fibre; the less fibre there is, the more will be converted, until you get, like, a Greggs bap that's basically just a glucose tablet without the mysterious wet-dryness.

If you are unsure whether a carbohydrate is refined or unrefined, ask yourself - have I ever thought:

"I could murder an X"? Sausage roll, yes. Pearl barley risotto, no. Buttered crumpet, yes. Kale spread with cashew butter, no.

The intensity of your desire is an index of the glucose it will deliver. This means a) all refined carbohydrates should be treated as sugars, in your sugar detox, and b) to avoid sugars, you simply avoid all the things you really want.

8A life without sugar

What sugar brings is not, as you might think, sweetness, but texture. So if you have a cake that is wheat-free and sugar-free, it is possible to find alternatives, replacing the wheat with nuts and the sugar with fruit, coconut oil, agave, combinations thereof. The nuts bring clagginess and the fruit is too wet, so the result is soggy and mushy with a mouth-coating trace of clay, a sort of repulsive pabulum whose problem is not its flavour but its mouthfeel. It is better not to replicate your old life, in other words, but to find new hobbies,

such as reading.

9Paleo eating

The best catch-all diet to remove sugar without contravening the copyright of the Atkins diet, this involves eating like our ancestors - very little fruit, almost no grains, a lot of meat and a lot of exercise as you pound away at your treadmill, imagining yourself the predator of the steak you will later eat. Adherents point to the fact that our stone-age ancestors were much healthier than us, having no problems with obesity, cancer or any other diseases that beset our modern age. Pedants point out that the posthumous diagnosis of cancer was pretty patchy until the discovery of the disease in circa 1600BC (some time after the Paleolithic era); and, furthermore, that many ancestors were cut off in their prime by other factors (dinosaurs!), and it is impossible to tell how fat they would have become had they lived to our great age.

I mistook this for Palio eating, and thought it meant eating like a jockey, which would be a mixture of chips, power bars and Viagra.

10Sugar-free alternatives

Basically, the trajectory of a sugar alternative goes like this: is discovered; is lauded by all; becomes available in Holland & Barrett; there are suggestions that it is not as wonderful as it was cracked up to be; is abandoned in favour of something else, which has conveniently come along in the meantime.

11Just stop eating it. What are you, a baby?

Or, more diplomatically put by Ashy-Boyd: "It's all about making sure you're eating a balanced diet, so you never get into a place where your blood sugar has dropped." This involves ceaseless snacking of foodstuff with a low glycaemic load, mainly hummus or things that remind you of hummus or things that are called "hummus" but aren't, in an attempt to appeal to people who only eat hummus (butterbean hummus. Seriously. How is that hummus?). You combine this with an oatcake, or something containing pumpernickel (note: not a German Christmas tree biscuit), and you ignore all the people who are looking at you and thinking: "I wish she would just eat properly and not like some kind of idiot koala."

"That's one way of protecting yourself," Ashy-Boyd continues. "The other thing is, you have to be conscientious about it. Maybe allow yourself a couple of days to go without it. And then once it's out of your bloodstream, it's so much easier to combat that desire."

Cold turkey, see? It's all about the cold turkey.

Captions:

Above: sugar-free chewing gum. Left: the low-sugar Paleo diet

Clockwise from top left: hummus; spelt; oatcakes and nuts

LOAD-DATE: January 13, 2014

LANGUAGE: ENGLISH

PUBLICATION-TYPE: Newspaper

Copyright 2014 Guardian Newspapers Limited
All Rights Reserved

theguardian

Guardian.com.

January 14, 2014 Tuesday

Sugar: a guide to the sweet stuff in all its forms

BYLINE: Alex Rentontheguardian.com

LENGTH: 491 words

ABSTRACT

Alex Renton: Learn how to tell one sweetener from another with our essential guide

FULL TEXT

Sugar is sugar, most science now agrees: a simple carbohydrate that almost all of us in the rich world eat to excess. The same goes for "natural" sweeteners such as honey or maple syrup - but they may have other elements that are good for you, or help you absorb the sugar that travels with them in a more healthy way.

Here's a guide to the terms and types of sweetener in the great sugar debate.

Sucrose

Pure sugar: one molecule of glucose and one of fructose - the latter accounts for the taste we call "sweet". We eat three times as much of it as our grandparents did.

Fructose

The new bad guy in the sugar debate. Fruit juices contain higher fructose levels, and, if the juice has no fibre in it, the fructose may damage the liver and other organs. American endocrinologist Robert Lustig, the scientist behind the "sugar is poison" furore, claims excessive fructose is the key factor in soaring levels of diabetes and obesity and also stimulates over-eating. Labelling legislation doesn't yet require manufacturers to list fructose levels.

High-fructose corn syrup

A cheap sugar made from maize in the US, commonly used in **fizzy drinks** and cheap processed foods. Fructose levels may be as high as 55%.

Table sugar

White sugar is 50% fructose, 50% glucose and made from cane or beet. Demerara, and other brown sugars are essentially the same, but they have molasses, a by-product of the sugar cane refining process, for colour and flavour. The molasses will contain some minerals including iron, calcium, magnesium, and potassium.

'No added sugar'

Often seen on "healthy" fruit juices, five-a-day drinks and fruit snacks. A loophole in labelling regulation means that sweet, concentrated fruit juice can be used - which is, of course, a high-fructose sugar. Naturally sweet fruit juice with the fruit fibre removed is now thought to be as harmful (or worse) than a cola - but the "with bits in" juice with the fruit fibre retained is better for you.

Agave nectar, maple syrup, honey

Natural sugars are still sugar - claims of "unrefined" don't alter the basic fact that they are all largely the same simple chemical. In some plant-derived sugars, like agave, fructose levels can be very high - but the label doesn't tell you how high. Beneficial trace elements and minerals can be in the mix, too; maple syrup is claimed to contain antioxidants that may help with heart disease.

Artificial sweeteners

Some, like "plant-derived" stevia, saccharin, sorbitol and xylitol (used in chewing gum) are chemical relations of sugar's saccharides, and so they act on the body in the same way. But because they are many times sweeter than sugar, fewer calories are involved.

Non-saccharide sweeteners such as aspartame are the basis of Nutrasweet and many low-cal soft drinks. Questions have been raised about their health benefits, but aspartame is currently judged safe.

LOAD-DATE: January 14, 2014

LANGUAGE: ENGLISH

PUBLICATION-TYPE: Newspaper

193 of 341 DOCUMENTS

THE TIMES

The Times (London)

January 14, 2014 Tuesday
Edition 1;
National Edition

Time for a splash of star quality

BYLINE: Deirdre Hipwell

SECTION: BUSINESS; Pg. 37

LENGTH: 90 words

As a way of ensuring your bad news is lost amid the good, SodaStream's weekend unveiling of its "first ever global ambassador" is as textbook as it gets (Deirdre Hipwell writes). Forget the profit warning that followed yesterday, meet Scarlett Johansson. The actress is, it seems, "beyond thrilled" to share her enthusiasm for the **fizzy drinks** machines. Investors need more than that to be enthused; SodaStream said it had missed profit targets for the fourth quarter and the shares sank rather than bubbled, losing 24 per cent in New York.

LOAD-DATE: January 14, 2014

LANGUAGE: ENGLISH

GRAPHIC: HO

PUBLICATION-TYPE: Newspaper

JOURNAL-CODE: TIM

Copyright 2014 Times Newspapers Limited
All Rights Reserved

DAILY MAIL (London)

January 13, 2014 Monday

FRUIT JUICE 'SHOULDN'T COUNT IN YOUR 5 A DAY'

LENGTH: 517 words

SOME BRANDS HAVE MORE SUGAR THAN COLA SAYS OBESITY TSAR

BY SOPHIE BORLAND HEALTH REPORTER

FRUIT juice is so high in sugar it should not count as part of a healthy five-a-day diet, the Government's obesity tsar has warned.

The public should even start watering it down to wean themselves off it, said Oxford professor Dr Susan Jebb.

Some brands of orange juice contain as much sugar as cola and should be taxed because of their potential effect on the nation's health, she claimed.

Experts now say shoppers are now getting confusing messages about food, with a huge range of products saying they count towards a five-a-day diet. Tinned fruit, children's drinks and even spaghetti hoops are all claiming health benefits - even though they are said to contain potentially unhealthy levels of sugar and salt.

Juice drinks have been singled out as a particular concern - lacking fibre and other nutrients as well as being high in calories. Even pure fruit juice is said to contain a large amount of naturally-occurring sugar - but people end up drinking too much of it because they do not see it as unhealthy.

Dr Jebb, a professor of diet and population health at Oxford University, said: I would support taking it [fruit juice] out of the five-a-day guidance.

Fruit juice isn't the same as intact fruit and it has got as much sugar as many classical **sugar drinks**.

It is also absorbed very fast, so by the time it gets to your stomach your body doesn't know whether it's Coca-Cola or orange juice, frankly. I have to say it is a relatively easy thing to give up.

Swap it and have a piece of real fruit. If you are going to drink it, you should dilute it.'

A small carton of orange juice contains about two and a half teaspoons of sugar, while a large glass has five. A whole orange has just two teaspoons, but also contains far more fibre - about 3 grams compared to none in juice.

Fruit also makes people feel fuller, helping to cut down on the need for other snacks. Dr Jebb's comments come after health experts last week urged firms to cut the amount of sugar added to food over concerns it is becoming the new tobacco'.

In 2011, researchers from Leeds University and the University of Bangor also called for fruit juice to be discounted from a healthy diet.

They said it gives the public a sweet tooth' and discourages them from eating fruit pieces that are far better for health.

The five a day' campaign was launched by the Department of Health in 2003, with the aim of encouraging Britons to eat five portions of fruit and vegetables every day. Its guidance says smoothies and juices, plus tins or fruit and veg, can count as up to two portions. Oxford researchers have claimed that following the guidelines could prevent 15,000 deaths a year from cancer, strokes and heart disease.

But surveys have found that as little as a fifth of adults and one in ten children meet the target.

A poll of 2,000 adults by the World Cancer Research Fund last year found that an average of 22 per cent of adults in England meet the five a day target. Figures ranged from 17 per cent in the North to 26 per cent in the South.

© Daily Mail

LOAD-DATE: January 12, 2014

LANGUAGE: ENGLISH

PUBLICATION-TYPE: Papers

Copyright 2014 Associated Newspapers Ltd.
All Rights Reserved

The Daily Telegraph

The Daily Telegraph (London)

January 13, 2014 Monday
Edition 1;
National Edition

Give up orange juice or dilute it, says obesity tsar

BYLINE: Alice Philipson

SECTION: NEWS; Pg. 8

LENGTH: 454 words

PEOPLE should stop drinking orange juice because it contains as much sugar as Coca-Cola, the Government's leading adviser on obesity has warned, as she calls for a tax on all fruit juice.

Prof Susan Jebb said she had given up orange juice and urged others to "wean" themselves off it or dilute it.

Prof Jebb is in charge of the Government's responsibility deal, a series of voluntary pledges by industry aimed at tackling health problems. She warned that fruit juice should not be counted as one of your five-a-day portions of fruit or vegetables because of its high sugar content.

"I would support taking it out of the five-a-day guidance," she told The Sunday Times.

"Fruit juice isn't the same as intact fruit and it has got as much sugar as many classical **sugar drinks**.

"It is also absorbed very fast so by the time it gets to your stomach your body doesn't know whether it's Coca-Cola or orange juice, frankly. I have to say it is a relatively easy thing to give up. Swap it and have a piece of real fruit.

"If you are going to drink it, you should dilute it."

Her comments come after health experts warned last week that sugar had become as dangerous as alcohol or tobacco, urging the food industry to cut the amount it put into food by 30 per cent.

The group of academics, who came together to launch "Action on Sugar", claim the reduction could shave 100 calories from each person's daily intake and reverse Britain's growing obesity epidemic.

They are also asking companies to stop advertising **sugary drinks** and snacks to children, claiming sugar has become "the alcohol of childhood". Simon Capewell, Professor of Clinical Epidemiology at the University of Liverpool, described sugar as "the new tobacco".

A study carried out in 2011 at Bangor University found that even freshly squeezed fruit juices can contain as much as five teaspoons of sugar per glass because the squeezing process concentrates the sweetness.

This is about two thirds of the amount found in a can of soda and can contribute to obesity and also disturb blood sugar levels and the body's natural metabolism, the study found. Whole fruits and vegetables have far more nutrients per calorie, the researchers said.

They also advised that if people did drink juice, one part of fruit juice should be diluted with four parts of water in order to make it more healthy.

Dried fruit, on the other hand, is considered a good source of fibre, vitamins and minerals and can help fight cancer, metabolic disease and heart problems.

Researchers have found that dried fruits contain just as many antioxidants, polyphenols and nutrients as fresh fruit.

"Fruit juice isn't the same as intact fruit and it has got as much sugar as many classical **sugar drinks**. '

LOAD-DATE: January 13, 2014

LANGUAGE: ENGLISH

PUBLICATION-TYPE: Newspaper

JOURNAL-CODE: DTL

Copyright 2014 Telegraph Media Group Limited
All Rights Reserved

The Express

January 13, 2014 Monday

'Don't Drink juice'

SECTION: NEWS; Pg. 17

LENGTH: 84 words

PEOPLE should stop drinking orange juice as it contains as much sugar as Coca-Cola, warns the Government's obesity tsar.

Professor Susan Jebb has given up fruit juice and urged others to "wean" themselves off it or dilute it.

She said it should not count as one of your five-a-day portions of fruit, adding: "Juice isn't the same as intact fruit and it has got as much sugar as many classical **sugar drinks.**

"By the time it gets to your stomach your body doesn't know whether it's Coca-Cola or orange juice."

LOAD-DATE: January 13, 2014

LANGUAGE: ENGLISH

PUBLICATION-TYPE: Newspaper

JOURNAL-CODE: DXP

Copyright 2014 Express Newspapers
All Rights Reserved

MailOnline

MailOnline

January 13, 2014 Monday 8:37 AM GMT

Fruit juice 'shouldn't count in your 5 a day': Some brands have more sugar than cola says obesity tsar

BYLINE: SOPHIE BORLAND

SECTION: HEALTH

LENGTH: 545 words

- Dr Susan Jebb said public should wean themselves off certain products
- Some brands of orange juice contain as much sugar as cola
- Oxford professor claims they should be taxed for impacting health
- Even pure fruit juice is said to contain naturally-occurring sugar

Fruit juice is so high in sugar it should not count as part of a healthy five-a-day diet, the Government's obesity tsar has warned.

The public should even start watering it down to wean themselves off it, said Oxford professor Dr Susan Jebb.

Some brands of orange juice contain as much sugar as cola and should be taxed because of their potential effect on the nation's health, she claimed.

Experts say shoppers are now getting confusing messages about food, with a huge range of products saying they count towards a five-a-day diet.

Tinned fruit, children's drinks and even spaghetti hoops are all claiming health benefits - even though they are said to contain potentially unhealthy levels of sugar and salt.

Juice drinks have been singled out as a particular concern - lacking fibre and other nutrients as well as being high in calories. Even pure fruit juice is said to contain a large amount of naturally-occurring sugar - but people end up drinking too much of it because they do not see it as unhealthy.

Dr Jebb, a professor of diet and population health at Oxford University, said: 'I would support taking it [fruit juice] out of the five-a-day guidance.

'Fruit juice isn't the same as intact fruit and it has got as much sugar as many classical **sugar drinks**.

'It is also absorbed very fast, so by the time it gets to your stomach your body doesn't know whether it's Coca-Cola or orange juice, frankly. I have to say it is a relatively easy thing to give up.

'Swap it and have a piece of real fruit. If you are going to drink it, you should dilute it.'

A small carton of orange juice contains about two and a half teaspoons of sugar, while a large glass has five. A whole orange has just two teaspoons, but also contains far more fibre - about 3 grams compared to none in juice.

Fruit also makes people feel fuller, helping to cut down on the need for other snacks. Dr Jebb's comments come after health experts last week urged firms to cut the amount of sugar added to food over concerns it is becoming the 'new tobacco'.

In 2011, researchers from Leeds University and the University of Bangor also called for fruit juice to be discounted from a healthy diet.

They said it gives the public a 'sweet tooth' and discourages them from eating fruit pieces that are far better for health.

The 'five a day' campaign was launched by the Department of Health in 2003, with the aim of encouraging Britons to eat five portions of fruit and vegetables every day. Its guidance says smoothies and juices, plus tins of fruit and veg, can count as up to two portions. Oxford researchers have claimed that following the guidelines could prevent 15,000 deaths a year from cancer, strokes and heart disease.

But surveys have found that as little as a fifth of adults and one in ten children meet the target.

A poll of 2,000 adults by the World Cancer Research Fund last year found that an average of 22 per cent of adults in England meet the five a day target. Figures ranged from 17 per cent in the North to 26 per cent in the South.

LOAD-DATE: January 13, 2014

LANGUAGE: ENGLISH

PUBLICATION-TYPE: Web Publication

JOURNAL-CODE: WEBDM

Copyright 2014 Associated Newspapers Ltd.
All Rights Reserved

Mirror

mirror.co.uk

January 13, 2014 Monday 6:42 PM GMT

Lidl bans sweets and chocolates at checkouts after sugar branded "the new tobacco";
The discount supermarket said it made the decision after 70% of parents revealed they were pestered for sugary treats as they queued

BYLINE: By Ruki Sayid

SECTION: LIFESTYLE,HEALTH

LENGTH: 303 words

Lidl will today axe sweets from the checkout at all 600 stores and replace them with healthy snacks like fruit and nuts.

The discount supermarket said it made the decision after 70% of parents revealed they were pestered for sugary treats as they queued with their shopping.

Two thirds admitted to caving in and buying them, while 25% said their children enjoyed healthier alternatives if they were available.

Ronny Gottschlich, managing director of Lidl UK, said: "We know how difficult it can be to say no to pester power, so by removing sweets and chocolates from our tills we can make it easier to reward children in healthier ways."

Lidl's move came after new pressure group Action on Sugar called for sugar in food to be cut by 30% to tackle Britain's obesity epidemic.

Malcolm Clark, of the Children's Food Campaign, said Lidl was showing how supermarkets could also help to make us healthier.

He said: "The onus is now on other supermarkets and retailers to follow suit and we and the British Dietetic Association will keep up the pressure for them to do so."

Last week Action on Sugar expert Prof Simon Capewell described sugar as "the new tobacco".

He added: "**Sugary drinks** and junk foods are pressed on unsuspecting parents and children by a cynical industry focused on profit not health.

"The obesity epidemic is generating a huge burden of disease and death."

Figures show 25% of adults in England is obese. That is set to rise to 60% of men, 50% of women and 25% of children by 2050.

The Department of Health said 38 food giants had already pledged to cut calories in their products.

Coca Cola said it aimed to slash calories in its products by 30% while Tesco made a similar commitment on its own-brand drinks. Mars has reduced its single chocolate portions to no more than 250 calories.

Obesity crisis "underestimated"

LOAD-DATE: September 1, 2014

LANGUAGE: ENGLISH

PUBLICATION-TYPE: Newspaper; Web Publication

JOURNAL-CODE: WEBDMI

Copyright 2014 Trinity Mirror, Plc.
All Rights Reserved

Daily Record & Sunday Mail

January 12, 2014 Sunday
Edition 1;
National Edition

Fruit juice? I'd have been as well giving my kids ten B&H; Jackie Bird BEHIND THE HEADLINES

BYLINE: Jackie Bird

SECTION: FEATURES; Pg. 17

LENGTH: 502 words

As far as feeding my children is concerned, I used to think I was a pretty good mother - not for them a packet of crisps and a **fizzy drink** for breakfast.

They never left the house without a healthy cereal with low-fat milk and a glass of fruit juice. What this smug parent didn't realise was that, as far as their general wellbeing was concerned, I might as well have given them 10 Benson & Hedges in their schoolbags.

On the face of it, the warning this week from a group of medics and scientists that sugar is the new tobacco seems to be a dramatic overreaction to grab some headlines. But is it? When you discover that a glass of orange juice naturally contains as much sugar as a can of non-diet coke but is marketed as being healthy, and that so-called nutritional breakfast cereals have generous amounts of sugar added, you begin to realise the extent of how we're all being conned by the food industry.

Not only has it been quietly upping the sugar content in products but often foods with the most sugar are promoted as being low in fat.

As far as obesity is concerned, I've not been the most sympathetic.

Flying home from holiday last year, I watched with concern as a large lady attempted to wedge herself into the airline seat. She then spent the entire flight stuffing herself full of crisps and sweets.

Yes, there are people with medical conditions that mean weight gain is out of their control but there are plenty of others who are simply greedy.

But a look at our high streets and the sheer number of people who are struggling with their weight tells you there must be another factor. Now it appears, blaming forces beyond our control isn't the cop-out it seems. If you are going out of your way to buy low-fat products and the makers are insidiously cramming them full of a substance that is, at worst, contributing to diabetes and heart disease or at best offering empty calories, then how can weight gain be your fault? Unless you're a food scientist, if the label on a fruit smoothie is plastered with claims it's healthy, how are you to know otherwise? I'd like to think I'm fairly intelligent but I really had no idea the small pot of low-fat yogurt I regularly snack on contains five - yes, five - spoonfuls of sugar.

Sugar overloads the liver and any the body doesn't need is converted into fat. Something truly terrible has been happening to our diets and not only is it making us ill but it's sucking billions out of the health service.

Medical experts are now calling for the food industry to be forced by government to reduce the amount of sugar it uses.

In 1953, when tobacco firms in America were faced with growing evidence of a link between smoking and cancer, they got together at a meeting in New York.

The minutes of their meeting contained this call to arms: "The industry should not engage in a merely defensive campaign ... They should sponsor a public relations campaign, which is positive in nature and entirely 'pro-cigarettes'."

What is the food industry going to do now? Watch this space.

LOAD-DATE: January 12, 2014

LANGUAGE: ENGLISH

GRAPHIC: DRIVE NHS's Change4Life ad urging people to cut down on sugar
TAKE YOUR PIC The Voice judge Kylie with fans last week and below, with Tom Jones in the new promo

PUBLICATION-TYPE: Newspaper

JOURNAL-CODE: SML

Copyright 2014 Scottish Daily Record & Sunday Mail Ltd
All Rights Reserved

theguardian

Guardian.com.

January 12, 2014 Sunday

Fruit juice should not be part of your five a day, says government adviser

BYLINE: theguardian.com

LENGTH: 402 words

ABSTRACT

Fruit juice has as much **sugar** as many **soft drinks** and should be removed from five-a-day guidance, says obesity expert

FULL TEXT

An adviser on obesity to the government has called for fruit juice to be removed from the recommended list of five-a-day portions of fruit or vegetables, saying it contains as much **sugar** as many **soft drinks**.

Susan Jebb, head of diet and obesity research at the Medical Research Council's Human Nutrition Research unit in Cambridge, told the Sunday Times she had herself stopped drinking orange juice and advised others to do so, or at least drink it diluted. The paper quoted her as saying she would support a wider tax on sugar-heavy drinks.

Jebb works closely with the government on diet and obesity issues, and leads the government's so-called health responsibility deal, which oversees voluntary pledges by the food and drink industry to improve public health.

Jebb told the Sunday Times she did not see juice as a healthy option: "I would support taking it out of the five-a-day guidance," she said. "Fruit juice isn't the same as intact fruit and it has got as much sugar as many classical **sugar drinks**. It is also absorbed very fast so by the time it gets to your stomach your body doesn't know whether it's Coca-Cola or orange juice, frankly.

"I have to say it is a relatively easy thing to give up. Swap it and have a piece of real fruit. If you are going to drink it, you should dilute it."

The paper said Jebb, who is also professor of diet and population health at the University of Oxford, backs a tax on **sugary drinks** but does not think ministers would support this.

Her comments follow a similar warning in September by two US scientists, Barry Popkin and George Bray, who exposed the health risks of fructose corn syrup in soft drinks in 2004.

Popkin, a professor of nutrition at the University of North Carolina, told the Guardian that fruit juices and fruit smoothies were "the new danger".

He said: "Think of eating one orange or two and getting filled. Now think of drinking a smoothie with six oranges and two hours later it does not affect how much you eat. The entire literature shows that we feel full from drinking beverages like smoothies but it does not affect our overall food intake, whereas eating an orange does. So pulped-up smoothies do nothing good for us but do give us the same amount of sugar as four to six oranges or a large coke. It is deceiving."

LOAD-DATE: January 12, 2014

LANGUAGE: ENGLISH

PUBLICATION-TYPE: Newspaper

212 of 341 DOCUMENTS

The Observer (England)

January 12, 2014

Comment: DIET: Politicians should stand up to the sugar lobby

BYLINE: Observer editorial

SECTION: OBSERVER NEW COMMENT PAGES; Pg. 40

LENGTH: 1086 words

This is National Obesity Awareness Week, focusing on the excess kilos that are presenting a year-round major challenge. Around two-thirds of adults in this country are overweight and a third of year six children are clinically obese, a condition connected to heart disease, strokes and diabetes - the most common causes of death and disability in the country that also drain £ 5bn a year from the public purse. It is predicted that by 2050, half the population could be obese at 10 times the current cost. At a reception tomorrow, Professor Graham MacGregor, head of Action on Sugar, a campaign group supported by clinicians in both the UK and US, launched last week, will endeavour to meet the public health minister Jane Ellison. This has to happen informally since, astonishingly, requests for an official encounter have so far been refused - although the evidence that we are all consuming far too many spoonfuls of sugar a day is incontrovertible.

The Conservative part of the coalition has a well-documented position on this issue. Consumption is the responsibility of the individual, with the lightest of touches exercised on the food, drink and retail sectors, which pursue their interests by deploying every available tactic including marketing, branding, packaging, advertising, sports sponsorship and political lobbying. As a result, the soaring sales of highly addictive calorie-dense snacks, "added value" processed foods and **sugar**-suffused **soft drinks** continues apace.

Definitive research tells us that this state of affairs is far too toxic to be dismissed as sweet nothings. So why is this government so reluctant to take effective action?

One reason might be that the coalition finds the lobbyists persuasive. They are certainly pervasive. Barbara Gallani, of the Food and Drink Federation, for instance, said last week: "Sugars . . . consumed as part of a varied and balanced diet are not a cause of obesity. . ." On successive mornings last week representatives of the alcohol, gambling and sugar industries had a good run in a series of media appearances as they did their best to suggest that, really, there isn't much of a problem. Except that there is, and it goes far beyond the nation's health.

The influence these industries wield goes to the heart of how politics plays out, and how it is seen to be gamed by powerful lobby groups. The Observer, among others, has highlighted the connections that Lynton Crosby's firm - Cameron's strategist - has as advisers in Australia to the alcohol and tobacco industries.

Meanwhile an investigation by the British Medical Journal (BMJ) revealed that health officials and ministers had 130 meetings with alcohol and supermarket lobbyists while they were considering imposing a minimum price per unit of alcohol. The proposal was dropped in July, allegedly because of a lack of "concrete evidence".

Doctors have rightly accused the government of "dancing to the tune of the drinks industry". It takes a David to present serious challenges to the Goliaths that are the multimillion £ industries, with their easy access to

power. Fortunately, such Davids do exist. But it is surely wrong that on the issue of our national diet, the profit motive and the demands of shareholders appear to take precedence over the public's health?

The protection of profit and the political connections between the Conservative party and the food, drink and retail sectors has a long history. In 1979, Margaret Thatcher set up the National Advisory Committee on Nutrition Education (NACNE), chaired by Professor Philip James, a powerhouse in the drive to improve diet. It produced a seminal report, suppressed until it was leaked in 1983, that warned the British diet was connected to the major diseases of our time. Its targets to reduce sugar, fat and salt were ignored.

Just over a decade later, in 1994, the Committee on Medical Aspects of Food and Nutrition Policy (Coma) again recommended that people should reduce salt intake by a third. Incredibly, this advice was first endorsed and then rejected by government because, according to the BMJ, members of the food industry threatened to withdraw funding from the Conservative party. Angered by the lack of government action, Professor Graham MacGregor, a cardiovascular specialist, set up a campaigning group, Consensus Action on Salt & Health (Cash). In 2003, Professor MacGregor co-published a study that predicted reducing an individual's salt intake from 12g a day to 3g would prevent a third of strokes and a quarter of heart disease cases. Under a Labour government, Cash went on to have considerable success. Professor MacGregor and colleagues now aim to see the same reversal in the consumption of sugar - a cut of 20% to 30%. So why is the government reluctant to engage with specialist campaigners, who have the best interests of the electorate at heart?

The new campaign warns of the dangers of "hidden sugars"; there are nine teaspoons in a can of Coke. Coca-Cola and Mars, among others, have taken steps to reformulate some products but progress is patchy. Take refined (cheap) sugar and salt out of ingredients and the profit margin shrinks. Last week, Andrew Lansley, the former health secretary, proposed that the food and drinks industries should take responsibility for a voluntary approach and incremental targets. As a response to a national crisis, that makes no sense.

Instead, a regulatory organisation is required, guided by evidence that sets targets, monitors progress and dispenses punishments. The Food Standards Agency, set up in 2001, showed its teeth, for instance, pushing for stricter rules on TV advertising to children of junk foods. It was rapidly neutered. Government then published a "call for action", a bizarre "new national ambition" to collectively reduce our calorie intake by 5 billion a day. Who is counting? The food and drinks industry applauded. Jamie Oliver called it "worthless, regurgitated, patronising rubbish". Now, we have a voluntary agreement and a reputation as the fattest nation in Europe.

It took a protracted battle with the tobacco industry to establish that smoking is lethal. We cannot wait another 30 years war before sugar and fat are brought under control. Of course, individuals have a responsibility to eat healthily but when advertising, marketing, addictive tastes and low prices combine so seductively that we are unaware how effectively a "normal" diet is killing us then that requires profit to take a back seat and government action to come to the fore.

LOAD-DATE: January 12, 2014

LANGUAGE: ENGLISH

PUBLICATION-TYPE: Newspaper

Copyright 2014 Guardian Newspapers Limited
All Rights Reserved

THE SUNDAY TIMES
The Sunday Times (London)

January 12, 2014 Sunday
Edition 1;
Scotland

'Five-a-day' foods packed with sugar

BYLINE: Kate Mansey

SECTION: NEWS; Pg. 8

LENGTH: 864 words

FOOD companies are using a government-backed campaign designed to persuade us to eat more healthily to sell products laden with sugar, a Sunday Times investigation reveals.

A McDonald's **fizzy drink**, a cocoa bar, Heinz spaghetti hoops and a packet of "fruit flakes" made with sugar and vegetable fat claim to provide one of the "five-a day" portions of fruit and vegetables recommended for a healthy diet.

The five-a-day promotion is based on advice from the World Health Organisation, which recommends eating a minimum of 400g (14oz) of fruit and vegetables, or five portions of 80g each, a day to lower the risk of health problems such as heart disease, stroke, type 2 diabetes and obesity.

Yet some products claiming to be part of your "five-a-day" were more than three-quarters sugar, excessive consumption of which has been linked to some of these conditions.

Some contained added sugar, while others were sweetened with white grape juice or concentrated apple juice, which provide few nutrients.

A packet of Kiddylicious Strawberry Fruit Wriggles was found to be 78% sugar, mainly derived from concentrated apple juice: only 6% of the product was strawberry.

Raspberry Fruit Flakes contained 65% sugar, including fructose-glucose syrup, vegetable fat and concentrated fruit juices. Yu! mango pieces were 83% sugar while Bear Yo Yo raspberry fruit rolls were 49% sugar. Robinsons My-5 fruit shoot drinks, which are aimed at children, were 19% sugar.

A McDonald's Fruitizz **fizzy drink** contained 10% sugar and a Nakd cocoa delight bar made with nuts and raisins also carried the five-a-day claim, despite having 42% sugar.

Health guidelines say no matter how much of one type of fruit you eat, it should be classed as only one of your fivea-day. However, Princes peach slices claimed to provide two portions and Baxters Italian tomato soup also claimed to count as two portions - it also contained added salt, sugar and vegetable oil. Heinz said its spaghetti hoops carried the fivea-day label because of the tomato content of its product, although it also contains added sugar and salt.

When the Department of Health launched the scheme, it said manufacturers could not use the official five-a-day logo on products aimed at children or on anything containing added sugar or salt.

But there is nothing to stop manufacturers putting their own five-a-day labels on foods containing high levels of sugar, salt and few nutrients. The scientist leading a new campaign to reduce sugar in our food by 30% over the next five years described the Sunday Times findings as "scandalous".

Graham Macgregor, of Action on **Sugar**, said: "It is scandalous to label products with high levels of **sugar** as one of your five-a-day. Many fruit juices have the same amount of **sugar** as **soft drinks**. But the food industry still counts them as one of your five-a-day.

"Mothers are being deceived into thinking they are not giving sugar to their children when in fact they are because apple juice concentrate is the same as sugar. It is ridiculous to tell people it is a portion of their five-a-day. Why isn't the Department of Health doing anything about it?" Bear, the manufacturer of Yo Yo rolls,

said it liaised with the health department before adding the claim to its packaging and its products included the entire pulp of the fruit.

Yu! said its mango product was "100% fruit". Baxters used its own five-a-day logo on soups because it wanted customers "to understand the contribution" vegetables make to their diets.

Nakd, McDonald's and Robinsons said the products contained only "natural" fruit sugars. Princes said it was "committed to helping people live healthier lifestyles".

The government has spent more than £4m on its fivea-day scheme but only 25% of people achieve the full five portions of fruit and vegetables a day. Public Health England said it did not "police" manufacturers' five-a-day claims but added: "If someone thought this was misleading they could refer the product to their local trading standards office."

Barbara Gallani, director of regulation at the Food and Drink Federation, which represents manufacturers, defended the right of companies to make the claims. "The nutritional benefits of fruit and vegetables are the same whether they are eaten on their own or as part of a composite product," she said.

"The Department of Health encourages manufacturers to use more fruit and vegetables in their recipes to help people towards their five-a-day, although there is no government advice on how to use a five-a-day label on composite products. For this reason, manufacturers look to guidance from the IGD [industry body], which includes nutritional standards, as a guide how to label their products responsibly."

Products claiming to deliver one of your five-a day

83%sugar Yu! Jus Fruit mango pieces Contain concentrated apple juice

78% Kiddylicious Strawberry Fruit Wriggles Contain concentrated apple juice

65% Fruit bowl Raspberry Fruit Flakes Contain added sugar

42% Nakd Cocoa Delight, raw fruit and nut wholefood bars

19% Robinsons Fruit Shoot My-5

11.8%Princes peach slices with juice Two of fivea- day target

10%McDonalds Fruitizz fizzy fruit drink

3.9%Heinz spaghetti hoops Contain salt and sugar

LOAD-DATE: January 12, 2014

LANGUAGE: ENGLISH

GRAPHIC: PAUL VOZDIC

PUBLICATION-TYPE: Newspaper

JOURNAL-CODE: STSscot

The Sunday Times (London)

January 12, 2014 Sunday
Edition 1;

Northern Ireland

Obesity tsar calls for tax on juice

BYLINE: Kate Mansey

SECTION: NEWS; FRONT PAGE; Pg. 1

LENGTH: 214 words

FRUIT juice contains so much sugar it should be taxed and should not be counted as one of your "fivea-day" portions of fruit, the government's leading adviser on obesity has warned.

Susan Jebb said she had stopped drinking orange juice and warned others to dilute it or "wean" themselves off it.

Jebb oversees the government's responsibility deal, a series of voluntary pledges by industry aimed at tackling health problems such as obesity and alcohol abuse.

She said many orange juices had as much sugar as Coca-Cola and should not be included as one of the "healthy" five-a-day fruit or vegetable portions recommended in health department guidelines.

"I would support taking it out of the five-a-day guidance," she said. "Fruit juice isn't the same as intact fruit and it has got as much sugar as many classical **sugar drinks**. It is also absorbed very fast so by the time it gets to your stomach your body doesn't know whether it's Coca-Cola or orange juice, frankly. I have to say it is a relatively easy thing to give up. Swap it and have a piece of real fruit. If you are going to drink it, you should dilute it."

Jebb said she was in favour of taxing **sugary drinks** but that ministers feared that would be unpopular with the public.

'Five-a-day' foods packed with sugar, page 5 @katemansey

LOAD-DATE: January 12, 2014

LANGUAGE: ENGLISH

PUBLICATION-TYPE: Newspaper

JOURNAL-CODE: STS

Copyright 2014 Times Newspapers Limited
All Rights Reserved

The Telegraph
telegraph.co.uk

January 12, 2014 Sunday 2:47 PM GMT

Wean yourself off orange juice, says government health tsar;

Professor Susan Jebb urges people to give up orange juice because it has 'as much sugar as Coca-Cola'

BYLINE: By Alice Philipson

SECTION: NEWS

LENGTH: 290 words

People should stop drinking orange juice because it contains as much sugar as Coca-Cola, the government's leading adviser on obesity has warned, as she calls for a tax on all fruit juice.

Professor Susan Jebb said she had given up orange juice and urged others to "wean" themselves off it or dilute it.

Professor Jebb is in charge of the government's responsibility deal, a series of voluntary pledges by industry aimed at tackling health problems such as obesity and alcohol abuse.

She warned that fruit juice should not be counted as one of your five-a-day portions of fruit as many juices because of its high sugar content.

"I would support taking it out of the five-a-day guidance," she told The Sunday Times .

"Fruit juice isn't the same as intact fruit and it has got as much sugar as many classical **sugar drinks**. It is also absorbed very fast so by the time it gets to your stomach your body doesn't know whether it's Coca-Cola or orange juice, frankly.

"I have to say it is a relatively easy thing to give up. Swap it and have a piece of real fruit. If you are going to drink it, you should dilute it."

It comes after health experts warned sugar has become as dangerous as alcohol or tobacco, urging the food industry to cut 30 per cent from processed in Britons' cupboards.

The group of academics, who came together to launch 'Action on Sugar', claim the reduction could shave 100 calories from each person's daily intake and reverse the UK's growing obesity epidemic.

They are also asking companies to stop advertising **sugary drinks** and snacks to children claiming sugar has become 'the alcohol of childhood.'

Professor of Clinical Epidemiology at the University of Liverpool, UK, Simon Capewell described sugar as "the new tobacco".

LOAD-DATE: January 13, 2014

LANGUAGE: ENGLISH

PUBLICATION-TYPE: Web Publication

JOURNAL-CODE: WEBDTNS

Copyright 2014 Telegraph Media Group Limited
All Rights Reserved

The Times (London)

January 11, 2014 Saturday
Edition 1;
National Edition

We are too sweet on sugar to give it up easily;
The way to kick the habit is to cut down on the stuff hidden in pasta sauce, ready meals and fizzy drinks

BYLINE: Janice Turner

SECTION: EDITORIAL; OPINION, COLUMNS; Pg. 23

LENGTH: 891 words

Last week my husband spotted an unmissable bargain in Waitrose: two whole pounds of its ownbrand chocolates reduced to 69p. He was heading for the till in triumph when I grabbed it from his hands. Would you buy a carton of Silk Cut if I was trying to give up smoking? Would you stock an alcoholic's house with cheap gin? Sugar is my crack. If sweets or chocolate, cake or biscuits are in the house, I will eat them. And as I work from home, with 24/7 secret snacking opportunities, I don't buy them. When my mother arrives from Yorkshire, suitcase bulging with chocolate teacakes, Tunnock's wafers and Kit Kats, I fling them on top of the highest cupboard. Which means that a week later I will be found teetering on a chair, trying to dislodge a family pack of Maltesers with a ladle.

Christmas brought so many sugary gifts that I considered sneaking them off to a food bank. But, as I have bred a family of fellow addicts, by New Year's Day only the crystallised ginger was left. It seems Type 2 diabetes, which my father developed late in life, will be our shared destiny.

The scientists who this week launched a campaign to persuade consumers that sugar is "the new tobacco" and that companies should reduce the sugar content of food by 30 per cent, have a hard sell. Their battles against saturated fat and salt have been largely won but that is no indicator of likely success.

It is hard to see as an enemy something so intricately bound up with love. As far as I know "my salt" is no endearment and "fatty" certainly is not. But "my sugar", "my sweet", "ah honey, honey, you are my candy girl ..." How can a lover's heart-shaped box, a mother's special cake, the mint from Grandpa's pocket, the centrepiece of birthdays and feast days, the cargo of Santa and the Easter Bunny be poison? Are we to believe The Great British Bake Off is a pushers' convention? When the friends in Sex and the City sat outside the Magnolia Bakery picking at colourful frosting, it seemed that the cupcake had replaced the cigarette: a shared moment, a communal rebellion. Certainly as tobacco has waned, the cupcake has risen, a symbol of both female domestic artistry and a curious kind of empowerment: sod the diet, sod being "good", this sickly little sucker is for me.

Once we were satisfied with a smear on top of a sticky bun; now we demand the icing be higher than the cake. With greater affluence weekly treats become daily; my grandmother would bring me a small chocolate bar, my kids receive a Toblerone the size of a roof beam. Little bags of sweets have been replaced by huge "shareable" ones you can reseal, but never do because without thinking you shovel in the lot.

What fun-suckers could resist this surfeit of sweetness, this abundance of love. This week Gwyneth Paltrow and her daughter Apple were photographed outside her Los Angeles home running a stall selling home-made lemonade. "No sugar added", said the colourful crayoned sign; that sour, selfdenying brew embodying all the smug purity on which Gwynnie is building her retail wing.

Yet in one sense she is right - we have a right to know when our food contains sugar. Over the years, manufacturers have pumped it into savoury dishes. Why is Heinz tomato soup every child's favourite? Because it contains more than a teaspoonful per 100g. "Sports drinks" such as Lucozade market themselves as promoting fitness, when if drunk instead of water they will more than replace every calorie sweated off on the pitch.

Pizza, white bread and low-fat yoghurts are suffused with sugar because it enhances taste and makes you eat more, as it suppresses production of the hormone leptin, which tells your brain you are full. Sugar gives you a pleasurable hit to the brain, a rush akin to the thrill of gambling or drugs or alcohol. The writer Simon

Gray told me that when he gave up booze he found relief from his cravings by drinking melted-down bars of Green & Black's.

And sugar only makes you crave more sugar. My inability to resist the chocolate in my house is not just my pathetic weak will, but chemistry. Eating too much makes me shaky with a roiling brain; giving up leaves me headachey, light-headed and exhausted. Anyone who has taken small children to a birthday party will know the cycle of rush, crash and, next day, cranky sugar "hangover".

It is a powerful drug and manufacturers use it cynically. In super-sweet breakfast cereals and "health" bars they are breeding the next generation of sugar junkies and contributing to the devastation of our collective health.

As Jeremy Hunt, the Health Secretary, remarked this week, 64 per cent of British adults being overweight and a third of children obese should be a cause for "national soul searching". Yet his predecessor, Andrew Lansley, immediately rubbished the call for manufacturers to cut added sugar - people will find the taste "unacceptable",* he said.

Food businesses will resist any threat to sales, but what can we do when obesity costs the NHS £5 billion a year and public health campaigns make no difference? If we eat sugar it should be done consciously. Scientists calculate that if we removed only the stuff shoved stealthily into pasta sauce and ready meals or glugged in **fizzy drinks**, the crisis would ease. Without denying us "sweets for my sweet, sugar for my honey".

'Nobody uses 'my salt' or 'fatty' as a term of endearment

LOAD-DATE: January 11, 2014

LANGUAGE: ENGLISH

PUBLICATION-TYPE: Newspaper

JOURNAL-CODE: TIM

Copyright 2014 Times Newspapers Limited
All Rights Reserved

The Express

January 10, 2014 Friday
Edition 1;
National Edition

THE SHOCKING TRUTH ABOUT SUGAR;
It's in everything from diet foods to ready-made sauces but is this hidden ingredient really as dangerous as experts claim?

BYLINE: Adrian Lee

SECTION: FEATURES; Pg. 27

LENGTH: 1087 words

FOOD companies were yesterday accused of ruining the nation's health by adding too much sugar to our foods. It's blamed for fuelling the obesity crisis, which is linked to a host of killer diseases, and one expert claims sugar is "the new tobacco". We reveal the shocking facts about the white stuff.

Sugar was first imported to Britain in the 14th century from plantations in Madeira. Later it began to arrive from colonies in the Caribbean and was sold in loaf or cone form. Imports really took off in the 18th century but sugar remained an expensive luxury, costing the equivalent today of £50 a pound. But as more plantations were opened prices fell and sugar became a source of energy for the poor, replacing honey as a sweetener.

The average Briton consumes 150lb of sugar every year. That's equivalent to about 34 teaspoons a day and is thought to be 20 times more than in the 1700s.

Most of us don't realise how much sugar we're eating or where it comes from because it takes so many different forms and is present in so many everyday foods. Sugar occurs naturally in fruit but is added to biscuits, cakes, fi zzy drinks and sweets during the manufacturing process. All types of sugar can make us fat but we're encouraged to eat fruit because it also contains other important nutrients. Added sugars give us energy but have no nutrients. Experts say any more than 15 teaspoons of all types of sugar a day is unhealthy.

Drinks often push up our sugar levels. A can of Pepsi or Coca Cola contains 9 teaspoons of sugar, while a tall Starbucks caramel frappuccino with whipped cream contains a whopping 11. Bottled teas and yogurt drinks are notoriously sugary.

So-called "hidden sugars" are added to many savoury products to balance out added salt or make them taste better. A 300g tin of tomato soup typically contains about 4 teaspoons of sugar while a Pot Noodle has 2. A 15ml serving of salad cream contains just under a teaspoonful, while you'll find half a teaspoon of sugar in two slices of bread. A 15g serving of ketchup has a teaspoonful of sugar and there are 3 in a 200g serving of a typical pasta sauce. A can of Heinz baked beans contains about 3 teaspoons of sugar. Sharwood's sweet and sour chicken with rice contains 5 teaspoons.

Studies show that having a high sugar diet (and consequently becoming obese) is linked to Type 2 diabetes and heart disease. It's thought that one in 20 cancers are linked to being overweight. In the UK nearly two-thirds of men and more than half of women are either overweight or obese.

All fruit contains sugar but not in equal amounts. The most sugary are raisins and other dried fruits, then grapes and bananas. A tiny packet (14g) of raisins contains 2 teaspoons of sugar, while an average size banana contains the equivalent of 3 teaspoons. Strawberries (1.3 teaspoons per serving), raspberries (1 teaspoon) and blueberries (1.7 teaspoons) are relatively low in sugar. Best of all is humble rhubarb but not many people can tolerate the tart taste without adding extra sugar. A tomato contains 0.7 teaspoons of sugar.

Sugar has many names. Table sugar is properly known as sucrose but other forms of sugar include dextrose, fructose, glucose, inverted sugar syrup, corn syrup, honey, nectar, lactose and maltose. You might see a label claiming that a product contains "natural sugar" but it will have just the same effect on your weight. Sugar is still sugar under any name.

Confectionery is a major source of sugar. A Mars Bar has 8 teaspoons so it's easy to see how it stacks up. A chocolate muffin contains about 5 teaspoons and a single Jelly Baby has 1 teaspoon. Sweets, cakes and biscuits also contain lots of fat.

Some experts claim it's possible to become addicted to sugar. One study by scientists in France found that mice which were addicted to cocaine chose sugar when given a choice. It's claimed receptors in our brain have become so conditioned to sugar that it has become an addiction which must be fed regularly.

Campaigners claim we're being hoodwinked into consuming sugar packed into apparently healthy products. A study found that a Kellogg's Nutri-Grain Elevenses Raisin Bake bar contained nearly 4 teaspoons. All the leading cereal bars were high in sugar. Parents often opt for fruit juice for children instead of **fizzy drinks**, mistakenly believing it is less sugary but a carton of apple juice and a can of cola have about the same amount. A 250ml serving of white grape juice contains as much sugar as four Krispy Kreme doughnuts. A

250ml can of Red Bull energy drink has more than 7 teaspoons, as do fruit smoothies . A 150g carton of Yeo Valley Zero Fat Family Farm Vanilla fl avoured yogurt contains 5 teaspoons.

You'll often see athletes guzzling **sugary drinks** or eating bananas. The reason they don't get fat is because they burn off so many calories. The rest of us aren't usually so active so if our sugary diets contribute to us taking in too many calories we will put on weight. If you have a sweet tooth regular exercise will help but a growing number of experts believe diet is the main factor in weight gain, not our sedentary lives.

By simply eating an excess of 50 calories per day - just half a biscuit - you could gain 5½lb or 2½kg in a year. So in five years you could be 2 stone or 12½kg heavier.

On food packs look at the "carbs as sugar" label. This includes both added and natural sugars. Less than 5g per 100g is low and more than 15g per 100g is high.

Australian Sarah Wilson, whose book I Quit Sugar has become a best-seller, claims she transformed her health and mood by giving up all forms of sugar, including fruit. She advocates giving up all sugars for a while, then gradually reintroducing small amounts (equivalent to about two pieces of fruit a day). Some experts insist it's just the latest fad and there's no need to go to this extreme.

Breakfast cereals are often very sugary. These are the amounts of sugar for a 100g serving. Cocoa Puffs: 9 teaspoons, Raisin Bran: 7 teaspoons, Alpen: 5 teaspoons, Special K: 3 teaspoons, Corn Flakes: 2.5 teaspoons. In contrast Shredded Wheat contains a 10th of a teaspoon.

Many manufacturers have cut sugar in their products over time without consumers noticing. However they say they have now gone as far as they can without compromising taste. Artificial sweeteners are an alternative but some people complain that they don't like the flavour. Some experts believe that eating or drinking products containing artificial sweeteners merely encourages us to seek out more sugary products.

LOAD-DATE: January 10, 2014

LANGUAGE: ENGLISH

GRAPHIC: SWEET DRUG: Some scientists claim we are all addicted
Pictures: ALAMY

PUBLICATION-TYPE: Newspaper

JOURNAL-CODE: DXP

Copyright 2014 Express Newspapers
All Rights Reserved

theguardian

The Guardian - Final Edition

January 10, 2014 Friday

Comparing sugar to tobacco wrong, says ex-health secretary

BYLINE: Rowena Mason, Political correspondent

SECTION: GUARDIAN HOME PAGES; Pg. 8

LENGTH: 471 words

Health experts are wrong to claim that sugar is as dangerous as smoking, the former health secretary Andrew Lansley said yesterday, as he clashed with one of his old advisers on obesity.

The senior Conservative, now leader of the house, said people would not accept a rapid reduction in the sugar content of familiar foods, as he rejected calls from the Action on Sugar group for a 20% to 30% drop in the amount added to products.

He also criticised "inaccurate analogies" from doctors leading the campaign, after several compared the harmful effects of sugar to drinking alcohol and smoking.

One of those sounding the alarm was Prof Simon Capewell, an expert in clinical epidemiology at the University of Liverpool who sat on Lansley's public health commission when the Tories were in opposition. He argued the government approach of trying to persuade companies to voluntarily reduce the sugar content of food did not go far enough.

"Sugar is the new tobacco. Everywhere, **sugary drinks** and junk foods are now pressed on unsuspecting parents and children by a cynical industry focused on profit not health," Capewell said.

"The obesity epidemic is already generating a huge burden of disease and death."

Dr Aseem Malhotra, science director of the campaign, said sugar could even be thought of as a worse problem than tobacco as its presence in processed food removed the ability to "exercise personal responsibility".

The new campaign is warning of "hidden sugars" in flavoured water, sports drinks, yoghurts, ketchup, ready meals and even bread, particularly those targeted at children.

Studies have found obesity and diabetes already cost the UK more than £5bn a year and this could rise to £50bn by 2050. In that same time period the number of obese adults is set to rise from one in four to more than one in two. Action on Sugar said a 30% reduction in the sugar content of food could help reverse Britain's trend of rising obesity within five years.

Speaking in the House of Commons Lansley praised the food industry's voluntary work to reduce salt content.

"We have had significant success in the reduction of salt in food but it has to be understood that this can only be achieved working with the industry on a voluntary basis . . . and it can only be done on an incremental basis," he said.

"You can't simply slash the sugar in food otherwise people simply won't accept it. That is what they are looking for. I don't think it is helped by what I think are inaccurate analogies. I just don't think the analogy between sugar and tobacco is an appropriate one. I think we have to understand that sugar is an essential component of food. It's just that sugar in excess is an inappropriate and unhelpful diet."

Captions:

Sugary drinks and junk foods are pressed on parents and children by a cynical industry, said a former health adviser

LOAD-DATE: January 10, 2014

LANGUAGE: ENGLISH

PUBLICATION-TYPE: Newspaper

Copyright 2014 Guardian Newspapers Limited

All Rights Reserved

The Sun (England)

January 10, 2014 Friday
Correction Appended
Edition 1;
Scotland

HIDE & SWEET;
SHOCK TRUTH ABOUT OUR FOOD

BYLINE: LYNSEY HOPE

SECTION: NEWS; Pg. 8

LENGTH: 648 words

DAMAGING levels of sugar are hidden in everyday foods including bread, yogurt and tinned soup, a new campaign group warns.

Experts believe we must reduce the amount of sugar we eat by up to 40 per cent to ward off health problems including obesity, diabetes, heart attack and stroke.

Now Action On Sugar is highlighting the massive levels of sugar in some surprising foods.

It is no surprise that there are nine teaspoons of sugar in a can of Coke and eight in a Mars bar.

But even a tin of Heinz Tomato Soup contains four teaspoons.

Dr Aseem Malhotra, cardiologist and Science Director of Action On Sugar, said: "What we want to do is to make people more aware of the sugar hidden in these foods."

Action On Sugar says children are particularly vulnerable, as they are often targeted in clever marketing and advertising campaigns.

Dr Malhotra added: "The message we want to get across is that added sugar has no nutritional value whatsoever and causes no feeling of satiety.

"The body gets no benefit from carbohydrates as added sugar.

"Sugar can damage your health independent of weight gain. Even if you're a normal, healthy weight and you exercise, eating too much sugar puts you at risk of type 2 diabetes."

Only last month experts at the World Health Organisation said we should reduce the sugar we eat from ten to five teaspoons per day.

The new campaign group has been set up by a group of experts including Dr Malhotra and another Campaign group called CASH - Consensus Action on Salt and Health.

Dr Malhotra said: "CASH has been successful in getting the food industry to gradually reduce salt in foods.

"Essentially, what we want to do is exactly the same thing with sugar."

Below we list popular foods and how many teaspoons of sugar they contain - one teaspoon of sugar is 4g.

How many spoonfuls of sugar in...

Parents beware - this 500ml bottle of Dr Pepper is, of course full of sugar, but did you realise just how much? 13

The same amount of Lucozade Energy drink, often used by sportsmen, has only marginally less sugar 11

Go ahead and have a 100g Go Ahead Yogurt Break bar - if you don't mind the ten spoonfuls 10

This 250ml glass of cranberry juice seems like a healthy option but drinking it regularly could be damaging 9

A 250ml bottle of Innocent Special Fruit Smoothie may have loads of fruit but it's loaded with high levels of sugar, too

8 1/2

Will a 51g Mars help you work, rest and play? Eat lots and you could get fat 8

This 250ml can of Red Bull energy drink will give you wings all right ... but they could be bingo wings 7

Parents use these small bags of Cadbury Dairy Milk Buttons to keep toddlers quiet - but may not realise the sugar content 4 1/2

A 30g serving of Kellogg's Frosties can start your day with a tasty crunch but there's a price to pay in terms of sugar intake 4

What better a winter warmer than a 300g tin of Heinz Tomato Soup? There's more than just tomatoes in it, though ... 4

A 45g serving of Jordans Fruit & Nut Muesli might make you feel healthy but it carries a sugary punch 4

This 49g Cadbury Dairy Milk Fruit And Nut bar has 31/2 spoons 3 1/2

A 150g serving of Yeo Valley 0% Fat Natural Yogurt has no fat ... but don't be fooled into thinking it's sugar-free too 3 1/4

Two McVitie's Milk Chocolate Hobnobs will nobble you with a fair bit of sugar

A 200g portion of Heinz Baked Beans on toast is one of the nation's favourites - but what's in that velvety sauce? 2 1/2

A 150g pot of Activia 0% fat Raspberry Yogurt? How healthy ... oh, apart from the two spoonfuls 2

Even a 15ml serving of Heinz Tomato Ketchup on the side of your plate carries a payload of sugar 1No escape. Even a slice of Hovis Wholemeal has a sweet touch 1/2

Even if it's celery you dip into your 50ml serving of Doritos Hot Salsa, you're still scoffing some sweet stuff 1/2

This 25g bag of Walkers salt and vinegar crisps is a savoury deight ... but there is still half a spoonful of sugar in it 1/2

LOAD-DATE: January 10, 2014

LANGUAGE: ENGLISH

CORRECTION-DATE: January 18, 2013

CORRECTION: Correction: IN an article "Hide and Sweet" (10 January), we stated: "This 25g bag of Walkers salt and vinegar crisps is a savoury delight... but there is still half a spoonful of sugar in it." In fact, there is only one twelfth of a teaspoon. We are happy to set the record straight and apologise for the error.

PUBLICATION-TYPE: Newspaper

JOURNAL-CODE: SUNscot

Copyright 2014 NEWS GROUP NEWSPAPERS LTD
All Rights Reserved

DAILY MAIL (London)

January 9, 2014 Thursday

SUGAR IS THE NEW TOBACCO'

LENGTH: 879 words

HEALTH CHIEFS TELL FOOD GIANTS TO SLASH LEVELS

A THIRD

BY SEAN POULTER CONSUMER AFFAIRS EDITOR

FOOD giants are being told to cut the amount of sugar they use because it has become the new tobacco'.

Doctors and academics say levels must be reduced by up to 30 per cent to halt a wave of disease and death.

They found that even zero-fat yoghurts can contain five teaspoons of sugar, while a can of Heinz tomato soup has four.

The equivalent of 11 teaspoons are found in a small Starbucks caramel Frappuccino with whipped cream. A Mars bar has eight. Sugar is the new tobacco,' said Simon Capewell, professor of clinical epidemiology at the University of Liverpool.

Everywhere, **sugary drinks** and junk foods are now pressed on unsuspecting parents and children by a cynical industry focused on profit not health. The obesity epidemic is generating a huge burden of disease and death. Obesity and diabetes already cost the UK over £5billion a year. Without regulation, these costs will exceed £50billion by 2050.'

Professor Capewell is part of a new

US-UK campaign group - Action on Sugar - that says asking firms to make voluntary changes has failed.

The typical Briton consumes 12 teaspoons of sugar a day and some adults consume as many as 46.

The maximum intake recommended by the World Health Organisation is ten, although this guideline is likely to be halved.The UN agency says there is overwhelming evidence coming out about **sugar**-sweetened **beverages** and other **sugar** consumption' being linked to obesity, diabetes and cardiovascular disease.

A study by Action on Sugar found surprisingly high levels of sugar in many foods, including savoury products and healthy options.

The Pret a Manger Very Berry Latte with milk has 26.9g of sugar - the equivalent of seven teaspoons. Yeo Valley Family Farm 0% Fat Vanilla Yogurt has five. Even Glaceau Vitamin Water, which is owned by Coca-Cola, has the equivalent of four teaspoons of sugar in a 500ml bottle. Action of Sugar said food firms should be able to reduce the amount of sugar they add to products by 20 to 30 per cent within three to five years,

taking 100 calories a day out of the typical diet. This would be enough to halt or even reverse rising levels of obesity and associated ill-health, it claimed.

Graham MacGregor, a professor at the Wolfson Institute of Preventive Medicine in London and chairman of Action on Sugar, said: We must now tackle the obesity epidemic both in the UK and worldwide.

We must start a coherent and structured plan to slowly reduce the amount of calories people consume by slowly taking out added **sugar** from foods and **soft drinks**.

This is a simple plan which gives a level playing field to the food industry, and must be adopted by the Department of Health to reduce the completely unnecessary and very large amounts of **sugar** the food and **soft drink** industry is adding to our foods.'

Dr Aseem , the group's science director, said: Added sugar has no nutritional value whatsoever, and causes no feeling of satiety.

Aside from being a major cause of obesity, there is increasing evidence that added sugar increases the risk of developing type 2 diabetes, metabolic syndrome and fatty liver.

We must particularly protect children from this public health hazard and the food industry needs to immediately reduce the amount of **sugar** that they are adding, particularly to children's foods, and stop targeting children with massive advertising for high calorie snacks and **soft drinks**.'

But sugar manufacturers rejected the claims of the health experts saying they were not supported by the consensus of scientific evidence.

Sugar Nutrition UK said the World Health Organisation published a review last year that found that any link between diabetes and body weight was due to overconsumption of calories and was not specific to sugar.

It said: There have also been numerous studies, which have investigated potential links between sugar and diabetes, with experts from the British Dietetic Association, European Food Safety Authority, and Institute of Medicine being very clear that diabetes is not caused by eating sugar. Respected expert committees have reviewed the evidence over many years and all have concluded that the balance of available evidence does not implicate sugar in any of the so-called lifestyle diseases.'

And Barbara Gallani, of the Food and Drink Federation, an industry group, also denied sugar was responsible for obesity.

She said the industry already provided clear information on sugar levels to consumers, using figures and colour-coded labels.

Sugars, or any other nutrient for that matter, consumed as part of a varied and balanced diet are not a cause of obesity, to which there is no simple or single solution,' she added.

Professor Shrinath Reddy, a cardiologist at the Harvard School of Public Health and member of the WHO panel of experts, disputed this conclusion.

He said there was overwhelming evidence coming out about **sugar**-sweetened **beverages** and other **sugar** consumption links to obesity, diabetes and even cardiovascular disease'.

Yoni Freedhoff, assistant professor of medicine at the University of Ottawa, said sugar needed again to become an occasional treat rather than a regular crutch'.

He said that added sugar had found its way into virtually everything we eat.

© Daily Mail

LOAD-DATE: January 8, 2014

LANGUAGE: ENGLISH

PUBLICATION-TYPE: Papers

Daily Mirror

January 9, 2014 Thursday
Edition 2;
National Edition

Britain's top docs declare war on sugar

BYLINE: ANDREW GREGORY

SECTION: NEWS; Pg. 11

LENGTH: 116 words

HEALTH campaigners will today launch a campaign to slash the amount of **sugar** in food and **soft drinks.**

Action on Sugar, set up by leading health experts, aims to tackle Britain's obesity crisis by encouraging food firms to cut "hidden sugars" in their products.

Chairman Prof Graham MacGregor said: "We must start a coherent and structured plan to slowly reduce the amount of calories people consume by slowly taking out added **sugar** from food and **soft drinks.**"

Cardiologist Dr Aseem Malholtra, the campaign's science director, also urged the food industry to "stop targeting children with massive advertising for highcalorie snacks".

In the UK, 64% of adults are overweight with a body mass index above 25.

LOAD-DATE: January 9, 2014

LANGUAGE: ENGLISH

GRAPHIC: CRISIS Obesity is a big issue

PUBLICATION-TYPE: Newspaper

JOURNAL-CODE: DMR

Copyright 2014 MGN Ltd.
All Rights Reserved

Group Limited
All Rights Reserved

The Daily Telegraph

The Daily Telegraph (London)

January 9, 2014 Thursday

Edition 2;
National Edition

Sugar perilous as tobacco, say health experts

BYLINE: Sarah Knapton

SECTION: NEWS; Pg. 10

LENGTH: 371 words

THE obesity crisis could be reversed within five years if the food industry cut sugar in processed foods by 30 per cent, say academics who warned that the natural sweetener has become as dangerous as alcohol or tobacco.

A group of medical professionals and academics has announced a campaign, Action on Sugar, which calls on food producers to reduce levels of sugar in everyday products significantly.

They are also asking companies to stop advertising **sugary drinks** and snacks to children, claiming that sugar has become "the alcohol of childhood".

They want the Government to fine those who do not meet reduction targets, or to impose a sugar tax.

The academics calculated that cutting sugar in processed foods by 20 to 30 per cent over the next three to five years may remove 100 calories from daily diets, enough to halt or reverse the crisis.

"Sugar is the new tobacco," said Simon Capewell, a professor of clinical epidemiology at the University of Liverpool.

"Everywhere, **sugary drinks** and junk foods are now pressed on unsuspecting parents and children by a cynical industry focused on profit not health. The obesity epidemic is already generating a huge burden of disease and death."

One in four adults in England is obese, while three in 10 children aged between two and 15 are overweight or obese. By 2050, 60 per cent of men, 50 per cent of women and 25 per cent of children are expected to be obese.

Diabetes and obesity cost Britain more than £5billion annually, which is likely to rise to £50billion in the next 36 years.

Sugary drinks are known to be a problem, but scientists say many people are unaware that flavoured waters, soups, ketchup and ready meals contain large amounts of sugar.

A can of Heinz tomato soup contains the equivalent of four teaspoons of sugar, while a mug of Cadbury's drinking chocolate holds six teaspoonfuls.

Many companies have signed up to a government initiative to cut sugar by 10 per cent from their products, but academics said that it does not go far enough.

Prof Robert Lustig, of the University of California, said children were "the primary targets" of marketing campaigns and the "least able to resist the messaging", which made **sugary drinks** "like the alcohol of childhood".

LOAD-DATE: January 9, 2014

LANGUAGE: ENGLISH

PUBLICATION-TYPE: Newspaper

JOURNAL-CODE: DTL

Copyright 2014 Telegraph Media Group Limited
All Rights Reserved

The Express

January 9, 2014 Thursday

Sugar is new tobacco say health crusaders

BYLINE: Jo Willey

SECTION: NEWS; Pg. 23

LENGTH: 244 words

WAGING a war on sugar - which has become the new tobacco - is the only way to beat Britain's obesity epidemic, experts claim.

They have now set up Action on Sugar in a bid to force manufacturers to slash levels.

The group wants to encourage the public to shun products "full of hidden sugars".

Professor of Clinical Epidemiology at the University of Liverpool, Simon Capewell, said: "Sugar is the new tobacco.

"Everywhere, **sugary drinks** and junk foods are now pressed on unsuspecting parents and children by a cynical industry focused on profit not health."

The group says there is no reason why the food industry cannot easily achieve a 20 per cent to 30 per cent reduction in the amount of sugar added to products in three to five years.

It says the reduction could reverse or halt the obesity epidemic and would also have a significant impact in reducing chronic disease. The campaign group says flavoured water, sports drinks, yogurts, ready meals and even bread are among the everyday foods that are affected.

Dr Aseem Malhotra, a cardiologist and the science director of Action on Sugar, said: "We must particularly protect children from this public health hazard and the food industry needs to immediately reduce the amount of sugar they are adding."

But Barbara Gallani, director of food science and safety at the Food and Drink Federation, which represents UK manufacturers, said: "Sugars consumed as part of a varied and balanced diet are not a cause of obesity."

LOAD-DATE: January 9, 2014

LANGUAGE: ENGLISH

PUBLICATION-TYPE: Newspaper

JOURNAL-CODE: DXP

Copyright 2014 Express Newspapers
All Rights Reserved

theguardian
The Guardian - Final Edition

January 9, 2014 Thursday

Obesity experts launch campaign to cut sugar in food by 30%

BYLINE: Sarah Boseley, Health editor

SECTION: GUARDIAN HOME PAGES; Pg. 10

LENGTH: 526 words

Obesity experts are launching a campaign to put pressure on the government and industry to cut the sugar content of food and drinks by up to 30%.

The high-profile scientists and doctors behind Action on **Sugar** say that gradual cuts in the amount of **sugar** in ready meals, cereals, sweets and **soft drinks** will not be noticed by the public, but will result in a reduction in the calories we all consume.

A 20-30% reduction in sugar over time will cut our calorie intake by about 100kcal a day. That is enough to halt or even reverse the obesity epidemic and reduce the toll of diabetes and other disease, say the doctors, who include Robert Lustig, author of Fat Chance: The Bitter Truth About Sugar, and Professors John Wass, academic vice-president of the Royal College of Physicians, Philip James of the International Association for the Study of Obesity and Sir Nicholas Wald of the Wolfson Institute of Preventive Medicine.

Action on Sugar aims to do what a similar campaign launched in the 1990s called Cash (Consensus Action on Salt and Health) successfully did for salt levels in our food. It is chaired by Professor Graham MacGregor, who also heads Cash.

"Provided the sugar reductions are done slowly, people won't notice," he said. "In most products in the supermarkets, the salt has come down by between 25% and 40%." People had not noticed the difference. Kellogg's Cornflakes contain 60% less salt than they used to.

The government's strategy against obesity has been to agree voluntary curbs on marketing to children and calorie reduction through a public health "responsibility deal". But MacGregor and others say it is not working and has had no effect on calorie intake. "We must start to slowly reduce the amount of calories people consume by slowly taking out added **sugar** from foods and **soft drinks**," he said.

The industry argues that we should cut calories by eating less, but there is no specific reason to target sugar. "Sugars, or any other nutrient for that matter, consumed as part of a varied and balanced diet are not a cause of obesity, to which there is no simple or single solution," said the Food and Drink Federation. "That's why the food industry has been working on a range of initiatives with other players to tackle obesity and diet-related diseases."

The campaign is worried about hidden sugars in processed foods, such as the nine teaspoons in a standard 330ml can of Coke and the six in a 375g portion of Sharwood's sweet and sour chicken with rice.

Simon Capewell, professor of clinical epidemiology at Liverpool University, called sugar the new tobacco. "Everywhere, **sugary drinks** and junk foods are now pressed on unsuspecting parents and children by a cynical industry focused on profit not health," he said.

The Department of Health said: "Helping people eat fewer calories, including sugar, is a key part of the responsibility deal and our efforts to reduce obesity. There are 38 businesses signed up to reduce calories, but we want to go further still, and are discussing this with the food industry."

Captions:

Action on Sugar aims to do for sugar in food what a similar campaign has done for salt, which has fallen by up to 40%

LOAD-DATE: January 9, 2014

LANGUAGE: ENGLISH

PUBLICATION-TYPE: Newspaper

Copyright 2014 Guardian Newspapers Limited
All Rights Reserved

242 of 341 DOCUMENTS

i-Independent Print Ltd

January 9, 2014
First Edition

Sugar is the new tobacco, warns top doctor; HEALTH

BYLINE: Charlie Cooper HEALTH REPORTER

SECTION: NEWS; Pg. 6

LENGTH: 437 words

The growing obesity epidemic could be "halted or reversed" in less than five years if the food industry makes cuts the amount of "hidden sugar" in our food, leading doctors have said.

Sugar is a major cause of obesity and also increases the risk of type 2 diabetes. Leading experts today launched a new campaign group, Action on Sugar, to alert the public to the high levels of sugar in their food and lobby the government and the food industry to reduce its use of "unnecessary" sugar.

The group, which brings together doctors from the UK, the US and Canada, aims to emulate the reduction in salt levels in our diet. Intake of salt dropped by 15 per cent between 2001 and 2011, leading to a minimum of 6,000 fewer strokes and heart attack deaths per year, saving £1.5bn.

Experts said that if major manufacturers reduced the amount of sugar in their products, adding up to a 20 to 30 per cent decrease in sugar content in three to five years, the obesity epidemic could be stopped in its

tracks. Graham McGregor, professor of cardiovascular medicine at the Wolfson Institute of Preventative Medicine and chairman of the new group, said that the Government's "Responsibility Deals" with the food industry had failed and a new approach was needed.

"This is a simple plan which gives a level playing field to the food industry, and must be adopted by the Department of Health to reduce the completely unnecessary and very large amounts of **sugar** the food and **soft drink** industry is currently adding to our foods," he said.

Children were particularly at risk from high **sugar** foods and **soft drinks**, said Simon Capewell, Professor of Clinical Epidemiology at the University of Liverpool. "**Sugar** is the new tobacco," he said. "Everywhere, **sugary drinks** and junk foods are now pressed on unsuspecting parents and children by a cynical industry focused on profit not health." The obesity epidemic is costing the UK over £5bn a year, he said, estimating that costs could rise to £50bn by 2050. Nearly two thirds of adults and more than a quarter of children in England are overweight.

A Department of Health spokesperson said it wanted to sign up more company to its Responsibility Deals, which have seen 38 food and drink companies volunteer to improve people's diets.

Sugar rush Product (portion) Teaspoons of sugar Pret a Manger Very Berry latte (295g) 7 Sharwood"s Sweet & Sour Chicken With Rice (375g) 6 Yeo Valley Family Farm 0% Fat Vanilla Yogurt (150g) 5 Heinz Classic Tomato Soup (300g) 4 Glaceau Vitamin Water, Defence (500ml) 4 Ragu Tomato and Basil Pasta Sauce (200g) 3 1 Heinz Tomato Ketchup (per portion) SOURCE: ACTION ON SUGAR

LOAD-DATE: January 8, 2014

LANGUAGE: ENGLISH

PUBLICATION-TYPE: Newspaper

JOURNAL-CODE: III

Copyright 2014 Independent Print Limited
All Rights Reserved

The INDEPENDENT

Independent.co.uk

January 9, 2014 Thursday 7:34 AM GMT

'Sugar is the new tobacco': Cuts to amounts hidden in food could halt obesity epidemic, claim doctors;
Experts aim to emulate the reduction in salt levels in our diet

BYLINE: Charlie Cooper

SECTION: FRONTPAGE

LENGTH: 378 words

The growing obesity epidemic could be "halted or reversed" in less than five years if the food industry makes cuts the amount of "hidden sugar" in our food, leading doctors have said.

Sugar is a major cause of obesity and also increases the risk of type 2 diabetes. Leading experts today launched a new campaign group, Action on Sugar, to alert the public to the high levels of sugar in their food and lobby the government and the food industry to reduce its use of "unnecessary" sugar.

The group, which brings together doctors from the UK, the US and Canada, aims to emulate the reduction in salt levels in our diet. Intake of salt dropped by 15 per cent between 2001 and 2011, leading to a minimum of 6,000 fewer strokes and heart attack deaths per year, saving £1.5bn.

Experts said that if major manufacturers reduced the amount of sugar in their products, adding up to a 20 to 30 per cent decrease in sugar content in three to five years, the obesity epidemic could be stopped in its tracks. Graham McGregor, professor of cardiovascular medicine at the Wolfson Institute of Preventative Medicine and chairman of the new group, said that the Government's "Responsibility Deals" with the food industry had failed and a new approach was needed.

"This is a simple plan which gives a level playing field to the food industry, and must be adopted by the Department of Health to reduce the completely unnecessary and very large amounts of **sugar** the food and **soft drink** industry is currently adding to our foods," he said.

Children were particularly at risk from high **sugar** foods and **soft drinks**, said Simon Capewell, Professor of Clinical Epidemiology at the University of Liverpool. "**Sugar** is the new tobacco," he said. "Everywhere, **sugary drinks** and junk foods are now pressed on unsuspecting parents and children by a cynical industry focused on profit not health."

The obesity epidemic is costing the UK over £5bn a year, he said, estimating that costs could rise to £50bn by 2050. Nearly two thirds of adults and more than a quarter of children in England are overweight.

A Department of Health spokesperson said it wanted to sign up more company to its Responsibility Deals, which have seen 38 food and drink companies volunteer to improve people's diets.

UK News in Pictures

LOAD-DATE: January 9, 2014

LANGUAGE: ENGLISH

PUBLICATION-TYPE: Web Publication

JOURNAL-CODE: WEBI

Copyright 2014 Independent Digital News and Media Limited
All Rights Reserved

Copyright 2014 Independent Digital News and Media Limited
All Rights Reserved

MailOnline

January 9, 2014 Thursday 4:32 PM GMT

Start SPREADING the news: Butter consumption at a 40 year high thanks to trans fats backlash

BYLINE: SARA MALM

SECTION: NEWS

LENGTH: 418 words

- Americans consume 5.6lbs of butter per person and year
- Fear of trans fats, found in processed margarine, reason for increase

Americans are eating more butter than they have in decades after a fear of trans fats and clean-food trends put the spread back on the menu.

As a result, butter consumption is now at a 40-year-high with U.S. citizens eating 5.6lbs a year of the yellow stuff.

This is an increase by 25 per cent in the last decade, after hitting a low of 4.1lbs per person in 1997.

In comparison, sales of margarine have dropped as consumers try to avoid trans fats, found in processed foods or products where the qualities of natural fats have been mimicked.

Trans fats increase cholesterol levels in the blood which can lead to heart attacks, strokes and heart disease.

As the health hazards that follow high consumption of trans-fats have become common knowledge, demand for natural dairy products has increased.

'Consumers are changing their perception of food and looking for healthier alternatives.

'They're moving away from highly processed foods and artificial ingredients,' Anuja Miner, executive director of the American Butter Institute, told the LA Times.

Butter, being high in vitamins, beneficial saturated fats, and 'good cholesterol' that is vital for brain and nervous system development, is increasingly becoming the first choice for cooking and sandwiches.

'Everything tastes better with butter,' David Riemersma, president of the American Butter Institute and head of Butterball Farms in Grand Rapids, Michigan told the paper.

'Consumers also want real, natural wholesome products. They want to understand all the things on an ingredient list. Butter fits perfectly. It's either just cream or cream and salt.'

Recent guidelines from the Food and Drug Administration, have all but banned trans fats, and many U.S. companies, such as Unilever, has followed suit and removed it from their products.

Meanwhile academics in Britain are comparing sugar to cigarettes, saying consumption levels must be reduced by 30 per cent to save public health.

'Sugar is the new tobacco,' said Simon Capewell, professor of clinical epidemiology at the University of Liverpool. 'Everywhere, **sugary drinks** and junk foods are now pressed on unsuspecting parents and children by a cynical industry focused on profit not health.

Reducing added sugar to products by 20-30 per cent would be enough to halt or even reverse rising levels of obesity and associated ill-health, US-UK campaign group - Action on Sugar claimed.

LOAD-DATE: January 10, 2014

LANGUAGE: ENGLISH

PUBLICATION-TYPE: Web Publication

JOURNAL-CODE: WEBDM

Copyright 2014 Associated Newspapers Ltd.
All Rights Reserved

MailOnline

January 9, 2014 Thursday 3:47 PM GMT

Start spread-ing the news: Butter consumption at a 40 year high thanks to trans fats backlash

BYLINE: SARA MALM

SECTION: NEWS

LENGTH: 418 words

- Americans consume 5.6lbs of butter per person and year
- Fear of trans fats, found in processed margarine, reason for increase

Americans are eating more butter than they have in decades after a fear of trans fats and clean-food trends put the spread back on the menu.

As a result, butter consumption is now at a 40-year-high with U.S. citizens eating 5.6lbs a year of the yellow stuff.

This is an increase by 25 per cent in the last ten years, after hitting a low of 4.1lbs per person in 1997.

In comparison, sales of margarine has dropped as consumers try to avoid trans fats, found in processed foods or products where the qualities of natural fats have been mimicked.

Trans fats increase cholesterol levels in the blood which can lead to heart attacks, strokes and heart disease.

As the health hazards that follow high consumption of trans-fats have become common knowledge, demand for natural dairy products has increased.

'Consumers are changing their perception of food and looking for healthier alternatives.

'They're moving away from highly processed foods and artificial ingredients,' Anuja Miner, executive director of the American Butter Institute, told the LA Times.

Butter, being high in vitamins, beneficial saturated fats, and 'good cholesterol' that is vital for brain and nervous system development, is increasingly becoming the first choice for cooking and sandwiches.

'Everything tastes better with butter,' David Riemersma, president of the American Butter Institute and head of Butterball Farms in Grand Rapids, Michigan told the paper.

'Consumers also want real, natural wholesome products. They want to understand all the things on an ingredient list. Butter fits perfectly. It's either just cream or cream and salt.'

Recent guidelines from the Food and Drug Administration, have all but banned trans fats, and many U.S. companies, such as Unilever, has followed suit and removed it from their products.

Meanwhile academics in Britain are comparing sugar to cigarettes, saying consumption levels must be reduced by 30 per cent to save public health.

'Sugar is the new tobacco,' said Simon Capewell, professor of clinical epidemiology at the University of Liverpool. 'Everywhere, **sugary drinks** and junk foods are now pressed on unsuspecting parents and children by a cynical industry focused on profit not health.

Reducing added sugar to products by 20-30 per cent would be enough to halt or even reverse rising levels of obesity and associated ill-health, US-UK campaign group - Action on Sugar claimed.

LOAD-DATE: January 10, 2014

LANGUAGE: ENGLISH

PUBLICATION-TYPE: Web Publication

JOURNAL-CODE: WEBDM

Copyright 2014 Associated Newspapers Ltd.
All Rights Reserved

London Evening Standard
standard.co.uk

January 9, 2014 Thursday 2:47 PM GMT

'It's wrong to compare dangers of sugar with smoking', says former Health Secretary Andrew Lansley

BYLINE: Staff/Agency

SECTION: HOME

LENGTH: 237 words

Claims that a sugary diet is as dangerous for you as smoking are inaccurate, former health secretary Andrew Lansley has said.

The Commons Leader made his remarks after one professor described sugar as "the new tobacco".

Speaking during his weekly question and answer session in the Commons, Mr Lansley said slashing the amount of sugar in consumers' diets was also not a wise move.

He said instead the food industry should be allowed to reduce the level incrementally, otherwise people would not accept it.

He said: "You can't simply slash the sugar in food otherwise people simply won't accept it.

"That is what they are looking for. I don't think it is helped by what I think are inaccurate analogies.

"I just don't think the analogy between sugar and tobacco is an appropriate one.

"I think we have to understand that sugar is an essential component of food, it's just that sugar in excess is an inappropriate and unhelpful diet."

His comments were in response to comparisons between sugar and smoking by Professor Simon Capewell, who is leading the Action on Sugar campaign to encourage food manufacturers to reduce the levels of sugar in their products.

Prof Capewell said: "Sugar is the new tobacco. Everywhere, **sugary drinks** and junk foods are now pressed on unsuspecting parents and children by a cynical industry focused on profit not health."

He added the costs of obesity and diabetes in UK could reach £50 billion by 2050.

LOAD-DATE: January 10, 2014

LANGUAGE: ENGLISH

PUBLICATION-TYPE: Newspaper; Web Publication

JOURNAL-CODE: WEBES

Copyright 2014 Evening Standard Limited
All Rights Reserved

London Evening Standard

standard.co.uk

January 9, 2014 Thursday 2:40 PM GMT

Lansley backs food sector on sugar

SECTION: PA NEWS FEEDS

LENGTH: 723 words

It is inaccurate to claim a sugary diet is as dangerous as smoking, former health secretary Andrew Lansley has said.

Mr Lansley said that instead of slashing the amount of sugar consumers' diets, the food industry should be allowed to reduce the level incrementally, otherwise people would not accept it.

The Commons Leader - who was health secretary until he lost the job in 2012 - said the analogy between sugar and tobacco was not appropriate, telling MPs the food industry had already reduced the amount of salt in food.

His comments came as a group of doctors likened the danger posed by a sweet foods to smoking tobacco as they launched a campaign to cut the amount of sugar in consumers' diet.

Speaking during his weekly question and answer session in the Commons, Mr Lansley said: "We have had significant success in the reduction of salt in food but i t has to be understood that this can only be achieved working with the industry on a voluntary basis... and it can only be done on an incremental basis.

"You can't simply slash the sugar in food otherwise people simply won't accept it. That is what they are looking for. I don't think it is helped by what I think are inaccurate analogies. I just don't think the analogy between sugar and tobacco is an appropriate one.

"I think we have to understand that sugar is an essential component of food, it's just that sugar in excess in an inappropriate and unhelpful diet."

Mr Lansley made his remarks as a group of health experts launched a campaign to reduce the amount of **sugar** added to food and **soft drinks** as part of an effort to reverse the UK's obesity and diabetes crisis.

Action on Sugar - modelled on the successful Consensus Action on Salt and Health (Cash) - aims to help the public avoid products "full of hidden sugars" and encourage manufacturers to reduce the ingredient over time.

It says children are a particularly vulnerable group who are targeted by marketers of calorie-dense snacks and **sugar**-sweetened **soft drinks**.

Earlier, Professor S imon Capewell, an expert in clinical epidemiology at the University of Liverpool who is leading the campaign, said obesity and diabetes were already costing the UK more than £5 billion a year.

He said: "Sugar is the new tobacco. Everywhere, **sugary drinks** and junk foods are now pressed on unsuspecting parents and children by a cynical industry focussed on profit not health.

"The obesity epidemic is already generating a huge burden of disease and death. Obesity and diabetes already costs the UK over £5 billion every year. Without regulation, these costs will exceed £50 billion by 2050."

Like Cash, Action on Sugar will set targets for the food industry to add less sugar to products over time so that consumers do not notice the difference in taste.

It claims that the food industry would easily achieve a 20% to 30% reduction in the amount of sugar added to products, which it says would result in a reduction of approximately 100kcal per day or more in those who are particularly prone to obesity.

It says the reduction could reverse or halt the obesity epidemic and would also have a significant impact in reducing chronic disease and claims the programme "is practical, will work and will cost very little".

The group listed flavoured water, sports drinks, yoghurts, ketchup, ready meals and even bread as just a few everyday foods that contain large amounts of sugar.

Advisers to the group include Professor Robert Lustig, of the paediatric endocrinology department at the University Of California, and Assistant Professor Yoni Freedhoff from the University of Ottawa.

Earlier this week, a British Medical Journal investigation criticised ministers for backing the alcohol industry, instead of health experts, on minimum unit pricing.

The Department of Health held regular meetings with representatives from the drinks industry, including on two occasions after a public consultation on the measure had ended, according to the probe.

In a letter to the Daily Telegraph, a group of 22 health professionals including Sir Ian Gilmore, special adviser on alcohol at the Royal College of Physicians, accused the Government of " dancing to the tune of the drinks industry".

The Department of Health insists no decision has been made on minimum pricing, although the proposal is widely believed to have been shelved.

LOAD-DATE: January 10, 2014

LANGUAGE: ENGLISH

PUBLICATION-TYPE: Newspaper; Web Publication

JOURNAL-CODE: WEBES

Copyright 2014 Evening Standard Limited
All Rights Reserved

London Evening Standard
standard.co.uk

January 9, 2014 Thursday 2:40 AM GMT

Health experts launch sugar drive

SECTION: PA NEWS FEEDS

LENGTH: 875 words

A group of health experts are campaigning to reduce the amount of **sugar** added to food and **soft drinks** as part of an effort to reverse the UK's obesity and diabetes crisis.

Action on Sugar - modelled on the successful Consensus Action on Salt and Health (Cash) - aims to help the public avoid products "full of hidden sugars" and encourage manufacturers to reduce the ingredient over time.

It says children are a particularly vulnerable group who are targeted by marketers of calorie-dense snacks and **sugar**-sweetened **soft drinks**.

Like Cash, Action on Sugar will set targets for the food industry to add less sugar to products over time so that consumers do not notice the difference in taste.

It claims that the food industry would easily achieve a 20% to 30% reduction in the amount of sugar added to products, which it says would result in a reduction of approximately 100kcal per day or more in those who are particularly prone to obesity.

It says the reduction could reverse or halt the obesity epidemic and would also have a significant impact in reducing chronic disease and claims the programme "is practical, will work and will cost very little".

The group listed flavoured water, sports drinks, yoghurts, ketchup, ready meals and even bread as just a few everyday foods that contain large amounts of sugar.

The chairman of Action on Sugar, Graham MacGregor, who is professor of cardiovascular medicine at the Wolfson Institute of Preventive Medicine and set up Cash in 1996, said: "We must now tackle the obesity epidemic both in the UK and worldwide.

"The present Government and Department of Health Responsibility Deal has been shown to have had no effect on calorie intake and we must start a coherent and structured plan to slowly reduce the amount of calories people consume by slowly taking out added **sugar** from foods and **soft drinks**.

"This is a simple plan which gives a level playing field to the food industry, and must be adopted by the Department of Health to reduce the completely unnecessary and very large amounts of **sugar** the food and **soft drink** industry is currently adding to our foods."

Dr Aseem Malhotra, cardiologist and science director of Action on Sugar, said: "Added sugar has no nutritional value whatsoever and causes no feeling of satiety. Aside from being a major cause of obesity, there is increasing evidence that added sugar increases the risk of developing type 2 diabetes, metabolic syndrome and fatty liver.

"We must particularly protect children from this public health hazard and the food industry needs to immediately reduce the amount of **sugar** that they are adding, particularly to children's foods, and stop targeting children with massive advertising for high-calorie snacks and **soft drinks**."

Advisers to the group include Professor Robert Lustig of the paediatric endocrinology department at the University Of California, Assistant Professor Yoni Freedhoff from the University of Ottawa and professor of clinical epidemiology Simon Capewell from Liverpool University.

Royal College of Physicians registrar Dr Andrew Goddard said: " We welcome this concerted and collaborative action to tackle the damage to health caused by consuming too much sugar. It is widely acknowledged that sugar is a major factor in both obesity and diabetes, and with many foods, everyday foods such as bread and breakfast cereals, containing high levels of added sugar, it can be difficult for consumers to make healthier choices.

"We strongly support Action on Sugar's campaign for clearer nutritional labelling of food and drink, and welcome their call for evidence-based government action to improve the public's health by reducing the amount of sugar added to food and drink by manufacturers."

Diabetes UK chief executive Barbara Young said: "We fully support any efforts to raise awareness that many foods contain more sugar than people might realise and to call on the food industry to reduce added sugar in our food and drink. This could make a real difference in helping tackle the obesity epidemic that is fuelling rates of type 2 diabetes and other chronic conditions.

"But it is important to be clear that we want to reduce sugar consumption because having too much can easily lead to weight gain, as is true with foods high in fat. So reducing the amount of sugar in our diets is not all that we need to do to reduce our risk of type 2 diabetes. The evidence that sugar has a specific further role in causing type 2 diabetes, other than by increasing our weight, is not clear. We look forward to the conclusions of the Scientific Advisory Committee on Nutrition, which is due to report this year."

A Department of Health spokeswoman said: " Helping people eat fewer calories, including sugar, is a key part of the Responsibility Deal and our efforts to reduce obesity. There are 38 businesses signed up to reduce calories, but we want to go further still, and are discussing this with the food industry.

"As part of the Responsibility Deal calorie reduction pledge, Coca Cola has reduced calories in some of its soft drinks brands by at least 30%, Mars has reduced its single chocolate portions to no more than 250 calories and Tesco has reduced the number of calories sold in its own brand soft drinks by over one billion."

LOAD-DATE: January 9, 2014

LANGUAGE: ENGLISH

PUBLICATION-TYPE: Newspaper; Web Publication

JOURNAL-CODE: WEBES

Copyright 2014 Evening Standard Limited
All Rights Reserved

The Telegraph
telegraph.co.uk

January 9, 2014 Thursday 4:00 PM GMT

Andrew Lansley: analogy between tobacco and sugar 'inaccurate'; Former health secretary Andrew Lansley suggests it is "inaccurate" to claim a sugary diet is as dangerous as smoking, while Action on Sugar chairman Graham MacGregor calls on the food industry to reduce the amount of sugar in their products.

LENGTH: 344 words

It is inaccurate to claim a sugary diet is as dangerous as smoking, former health secretary Andrew Lansleyhas said.

Mr Lansley said that instead of slashing the amount of sugar consumers' diets, the food industry should be allowed to reduce the level incrementally, otherwise people would not accept it.

The Commons Leader - who was health secretary until he lost the job in 2012 - said the analogy between sugar and tobacco was not appropriate, telling MPs the food industry had already reduced the amount of salt in food.

His comments came as a group of doctors likened the danger posed by a sweet foods to smoking tobacco as they launched a campaign to cut the amount of sugar in consumers' diet.

Speaking during his weekly question and answer session in the Commons, Mr Lansley said: "We have had significant success in the reduction of salt in food but it has to be understood that this can only be achieved working with the industry on a voluntary basis ... and it can only be done on an incremental basis.

"You can't simply slash the sugar in food otherwise people simply won't accept it. That is what they are looking for. I don't think it is helped by what I think are inaccurate analogies. I just don't think the analogy between sugar and tobacco is an appropriate one.

"I think we have to understand that sugar is an essential component of food, it's just that sugar in excess in an inappropriate and unhelpful diet."

Mr Lansley made his remarks as a group of health experts launched a campaign to reduce the amount of **sugar** added to food and **soft drinks** as part of an effort to reverse the UK's obesity and diabetes crisis.

Action on Sugar - modelled on the successful Consensus Action on Salt and Health (Cash) - aims to help the public avoid products "full of hidden sugars" and encourage manufacturers to reduce the ingredient over time.

It says children are a particularly vulnerable group who are targeted by marketers of calorie-dense snacks and **sugar**-sweetened **soft drinks**.

Sources: ITN/news agencies

LOAD-DATE: January 10, 2014

LANGUAGE: ENGLISH

PUBLICATION-TYPE: Web Publication

JOURNAL-CODE: WEBDTNS

Copyright 2014 Telegraph Media Group Limited
All Rights Reserved

The Telegraph
telegraph.co.uk

January 9, 2014 Thursday 3:51 PM GMT

Lansley: analogy between tobacco and sugar 'inaccurate'; Former health secretary Andrew Lansley suggests it is "inaccurate" to claim a sugary diet is as dangerous as smoking, while Action on Sugar chairman Graham MacGregor calls on the food industry to reduce the amount of sugar in their products.

LENGTH: 344 words

It is inaccurate to claim a sugary diet is as dangerous as smoking, former health secretary Andrew Lansleyhas said.

Mr Lansley said that instead of slashing the amount of sugar consumers' diets, the food industry should be allowed to reduce the level incrementally, otherwise people would not accept it.

The Commons Leader - who was health secretary until he lost the job in 2012 - said the analogy between sugar and tobacco was not appropriate, telling MPs the food industry had already reduced the amount of salt in food.

His comments came as a group of doctors likened the danger posed by a sweet foods to smoking tobacco as they launched a campaign to cut the amount of sugar in consumers' diet.

Speaking during his weekly question and answer session in the Commons, Mr Lansley said: "We have had significant success in the reduction of salt in food but it has to be understood that this can only be achieved working with the industry on a voluntary basis ... and it can only be done on an incremental basis.

"You can't simply slash the sugar in food otherwise people simply won't accept it. That is what they are looking for. I don't think it is helped by what I think are inaccurate analogies. I just don't think the analogy between sugar and tobacco is an appropriate one.

"I think we have to understand that sugar is an essential component of food, it's just that sugar in excess in an inappropriate and unhelpful diet."

Mr Lansley made his remarks as a group of health experts launched a campaign to reduce the amount of **sugar** added to food and **soft drinks** as part of an effort to reverse the UK's obesity and diabetes crisis.

Action on Sugar - modelled on the successful Consensus Action on Salt and Health (Cash) - aims to help the public avoid products "full of hidden sugars" and encourage manufacturers to reduce the ingredient over time.

It says children are a particularly vulnerable group who are targeted by marketers of calorie-dense snacks and **sugar**-sweetened **soft drinks**.

Sources: ITN/news agencies

LOAD-DATE: January 10, 2014

LANGUAGE: ENGLISH

PUBLICATION-TYPE: Web Publication

JOURNAL-CODE: WEBDTNS

Copyright 2014 Telegraph Media Group Limited
All Rights Reserved

The Telegraph
telegraph.co.uk

January 9, 2014 Thursday 3:45 PM GMT

Comparing sugar to tobacco is alarmist and misleading, claim health experts;
Health experts at Action for Sugar have warned that sugar is as dangerous as tobacco but Dr Victoria Burley, a senior lecturer in Nutrional Epidemiology at Leeds University, says their claims are alarmist and misleading

SECTION: NEWS

LENGTH: 625 words

"It is nuts to claim that sugar is as dangerous as alcohol. It's total hyperbole, quite crazy," said Dr Burley.

"The epidemiology for smoking causing cancer is strong. You can look at figures and see that one quarter of cancer deaths are linked to smoking, that's something like 43,000 deaths a year.

"There is certainly evidence that obesity is linked to cancer and coronary heart disease but there is little evidence that there is a causal link between sugar and obesity.

"So you can't say with any certainty that sugar is a cause of death. It's very hard to work out how much sugar people are eating for one thing. Many people aren't aware of their sugar intake or lie about it.

"Much of the evidence linking sugar to obesity comes from US where they use high fructose corn syrup and the UK food industry hasn't gone down that route. My feeling is that the risk from sugar has been over-exaggerated.

"We have seen evidence suggesting a link between **sugary drinks** and diabetes. A study showed that each extra can each day is associated with an 18 per cent rise in the risk of Type 2 diabetes. But there was a similar rise for arfiticially sweetened drinks so clearly something else is going on as well.

"Sugar may also be useful for people who are very physically active or if you are trying to gain weight. You may need the extra energy. And actually are consumption of sugar has been decreasing steadily since the 1960s. Sales of sweets, jams and preserves have all gone down.

"I think everyone should relax about it. What is a problem is the over-consumption of calories. The moderation line may seem boring but it is the best way to avoid the risks."

Naveed Sattar, Professor of Metabolic Medicine at Glasgow University also agrees.

"The truth is that sugar on its own is not necessarily bad if overall calorie intake matches calorie burn and individuals are normal weight / healthy - however, when the diet leads to overconsumption of calories - here excess fat or sugar can both be stored as excess fat in important body organs such as the liver or muscle increasing health risks such as diabetes, liver disease and associated conditions.

"Many individuals who eat too many calories do so because either their intake of refined sugar or fat are excessive as these things taste so nice and the trick is to retrain the palate over time to like better quality foods.

"Everyone can achieve this but perseverance is needed. Ideally, if better quality foods are available generally together with a parallel reduction in sugar and fat rich foods, such changes would inevitably help individuals make healthier choices. This is where we need to go and the foods and drinks industry must be "cajoled" to helping with this direction of travel

"The evidence that the obesity epidemic is much more to do with over consumption of calories than altered activity levels in now clear, which is why lessening the nations calorie intake is critical to reverse obesity levels.

"It's also true that excess sugar is now pervasive in many foods and drinks so that changes in food formulations towards a gradual lowering of both refined sugar (and fat) content could be a major way to address obesity levels.

"In the meantime, individuals who wish to lose weight or stop weight gain can cut their refined sugar intake by replacing **sugary drinks** by either water or diet drinks. Whilst some may find this hard initially, the palate can adapt to new tastes over a few weeks so that individuals fine this new taste equally enjoyable.

"The same is also true for other sweet or fatty foods; gradual changes in diet towards healthier foods is possible for all and the concept of retaining the palate is something that needs wider publicity"

LOAD-DATE: January 10, 2014

LANGUAGE: ENGLISH

PUBLICATION-TYPE: Web Publication

JOURNAL-CODE: WEBDTNS

Copyright 2014 Telegraph Media Group Limited
All Rights Reserved

The Telegraph
telegraph.co.uk

January 9, 2014 Thursday 7:00 AM GMT

Sugar is as dangerous as alcohol and tobacco, warn health experts; Britain's obesity crisis could be reversed within five years if food companies reduced sugar in products by 30 per cent, health experts claim as they launch a new campaign to cut intake

BYLINE: By Sarah Knapton Science Correspondent

SECTION: NEWS

LENGTH: 619 words

Sugar has become as dangerous as alcohol or tobacco, academics have said as they call on the food industry to cut 30 per cent from processed in Britons' cupboards.

Health experts claim the reduction could shave 100 calories from each person's daily intake and reverse the UK's growing obesity epidemic.

Today a group of health experts and academics come together to launch 'Action on Sugar', a campaign which is calling on the food producers to dramatically reduce levels of sugar in everyday products.

They are also asking companies to stop advertising **sugary drinks** and snacks to children claiming sugar has become 'the alcohol of childhood.'

And they are calling on the government to fine those who do not meet reduction targets or impose a Sugar Tax.

Professor of Clinical Epidemiology at the University of Liverpool, UK, Simon Capewell says, "Sugar is the new tobacco.

"Everywhere, **sugary drinks** and junk foods are now pressed on unsuspecting parents and children by a cynical industry focussed on profit not health.

"The obesity epidemic is already generating a huge burden of disease and death."

One in four adults in England is obese and these figures are set to climb to 60 per cent of men, 50 per cent of women, and 25 per cent of children by 2050. Three in every 10 children aged between two and 15 are overweight or obese.

Obesity and diabetes already costs the UK over £5billion every year which is likely to rise to £50 billion in the next 36 years.

Although **sugary drinks** are known to be a problem scientists say many people are unaware that flavoured waters, soups, ketchup and ready meals also contain large amounts of hidden sugars.

A can of Heinz tomato soup contains the equivalent of four teaspoons of sugar while a mug of Cadbury's drinking chocolate holds six teaspoonfuls. A Yeo Valley vanilla yoghurt contains five teaspoons of sugar.

Last year a study by Oxford University suggested that a 12p tax on **fizzy drinks** would cut consumption by 15 per cent and mean 180,000 fewer obese adults.

The experts have calculated that reducing sugar in processed foods by between 20 and 30 per cent over the next three to five years they could remove 100 calories a day from diets, enough to halt or reverse the obesity epidemic.

They highlight children as a particularly vulnerable group because of the heavy marketing of sweets and **sugary drinks** towards youngsters.

Assistant Professor of Medicine at the University of Ottawa, Canada, Yoni Freedhoff; "Not only has added sugar found its way into virtually everything we eat, but worse still, the use of sugar as a means to pacify, entertain and reward children has become normalized to the point that questioning our current sugary status quo often inspires anger and outrage.

"We need to re-relegate sugar to the role of occasional treat rather than its current role of everyday, anytime, crutch".

Professor of Paediatric Endocrinology at the University Of California, San Francisco Robert Lustig added: "Children are the primary targets of marketing campaigns, and the least able to resist the messaging.

"That makes **sugary drinks** like the "alcohol of childhood", which makes them obese."

Many companies have already signed up to the government's Responsibility Deal to cut sugar by 10 per cent from their products but academics say it does not go far enough.

"We must now tackle the obesity epidemic both in the UK and worldwide," said Professor Graham MacGregor,

"The present government and Department of Health Responsibility Deal has been shown to have had no effect on calorie intake and we must start a coherent and structured plan to slowly reduce the amount of calories people consume by slowly taking out added **sugar** from foods and **soft drinks**."

LOAD-DATE: January 9, 2014

LANGUAGE: ENGLISH

PUBLICATION-TYPE: Web Publication

JOURNAL-CODE: WEBDTNS

Copyright 2014 Telegraph Media Group Limited
All Rights Reserved

THE TIMES
The Times (London)

January 9, 2014 Thursday
Edition 1;
National Edition

Sugar is the new tobacco, say doctors

BYLINE: Chris Smyth

SECTION: NEWS; Pg. 7

LENGTH: 441 words

Sugar is the "new tobacco" and companies need to cut the amount they add to food by 30 per cent to help to stem the obesity epidemic, a new campaigning group of doctors says.

Action on Sugar, which launches today, says that better labelling and more action by the food industry is urgently needed to reduce our consumption of sugar.

Sugar added to food has little nutritional value, does not make people feel full and is dangerous beyond merely the number of calories it contains, the experts say. The sugar we add to food ourselves is dwarfed by the amounts added by food companies, they say.

Action on Sugar models itself on Consensus Action on Salt and Health, which has helped to reduce salt intake by 15 per cent over the past decade. Supermarkets taking salt out of their products has been a big cause of that fall and the group says that the same thing must happen with sugar.

Graham MacGregor, the chairman of Action on **Sugar**, said: "We must start a coherent and structured plan to slowly reduce the amount of calories people consume by slowly taking out added **sugar** from foods and **soft drinks**.

"This is a simple plan which gives a level playing field to the food industry and must be adopted by the Department of Health to reduce the completely unnecessary and very large amounts of **sugar** the food and **soft drink** industry is adding to our foods."

Aseem Malhotra, a cardiologist at Croydon University Hospital, said: "Added sugar has no nutritional value whatsoever and causes no feeling of satiety. Aside from being a major cause of obesity, there is increasing evidence that added sugar increases the risk of developing type 2 diabetes, metabolic syndrome and fatty liver."

Simon Capewell, a professor of clinical epidemiology at the University of Liverpool, said: "Sugar is the new tobacco.

Everywhere, **sugary drinks** and junk foods are now pressed on unsuspecting parents and children by a cynical industry focused on profit, not health." Robert Lustig, a paediatric endocrinologist at the University of California, San Francisco, said: "Sugar is dangerous, exclusive of its calories, just like alcohol." Andrew Goddard, of the Royal College of Physicians, said: "Sugar is a major factor in obesity and diabetes, and with many everyday foods, such as bread and breakfast cereals, containing high levels of added sugar, it can be difficult for consumers to make healthier choices."

Barbara Gallani, of the Food and Drink Federation, said: "Sugars, or any other nutrient for that matter, consumed as part of a varied and balanced diet are not a cause of obesity, to which there is no simple or single solution." Stop children getting caned, Times2

LOAD-DATE: January 9, 2014

LANGUAGE: ENGLISH

PUBLICATION-TYPE: Newspaper

JOURNAL-CODE: TIM

Copyright 2014 Times Newspapers Limited
All Rights Reserved

The Daily Telegraph
The Daily Telegraph (London)

January 8, 2014 Wednesday
Edition 1;
National Edition

America is turning tide on obesity while we do nothing, warns Hunt

BYLINE: Matthew Holehouse

SECTION: NEWS; Pg. 6

LENGTH: 509 words

AMERICA is making better progress than Britain at tackling obesity, the Health Secretary claimed yesterday.

The US has started to "turn the tide" on the problem, but the process has not begun in Britain, Jeremy Hunt said.

Mr Hunt called for some "national soul-searching" about the rising number of overweight people and said they could not look to the Government alone for help. They must start to take responsibility for themselves by changing their unhealthy lifestyles, he said.

Sixty-four per cent of British adults are classed as overweight, meaning their body mass index (BMI) - calculated by dividing a person's weight by their height - is greater than 25, and 26 per cent are classed as being obese, meaning their BMI is over 30.

Obesity has increased significantly in recent decades. Before 1980, fewer than 7 per cent of adults were classed as obese. But by 2050, 60 per cent of men, half of women and a quarter of children are forecast to be obese, according to Public Health England.

In the US, more than 70 per cent are overweight and 31 per cent are obese, and Mr Hunt said Britain must "wake up" to its problem before it got worse.

"America has an even bigger obesity problem than us, but they have actually started to turn the tide on obesity in the US; we haven't started to do that here and I think we've got to do some real national soul-searching about whether or not we're going to grip this problem," he said. "It's not just government; the Government should do more, but it's also about choices that people make over their own lifestyles."

NHS chiefs have ordered the first survey into older and fatter women giving birth, following evidence that maternity units are facing increasing pressure from complex pregnancies.

Some maternity units have recorded a doubling in the number of obese mothers giving birth in just two years, according to research by Sky News.

Obese women are more at risk of high blood pressure and premature delivery. They are also at risk of deep vein thrombosis and blood clots because they are less mobile.

NHS England is to conduct the first audit of complex births, including those to obese mothers, to try to improve patient safety.

The UK has the second highest rates of obesity in Europe, behind only Hungary, which last year introduced taxes on salt, **sugar** and the ingredients in **soft drinks.**

This year the US lost its position as the world's fattest nation, as Mexico's obesity rate rose to more than 32 per cent.

Growth in obesity rates has been slowing in the US since 2005. In 2008, obesity increased in 37 states, but by 2011 this was just 16 states.

The campaign against obesity has been led by Michelle Obama, the First Lady, who has worked particularly to get children living healthier lives.

Mr Hunt also said it was necessary to "reinvent" the NHS to handle financial pressures and the cost of an ageing population. Doctors and nurses are working under "a lot of pressure", he said.

64 pc Proportion of British adults who are classed as being overweight, with a body mass index greater than 25

LOAD-DATE: January 8, 2014

LANGUAGE: ENGLISH

The Time and The Telegraph – covered this story too.

MailOnline

MailOnline

January 8, 2014 Wednesday 11:47 PM GMT

Sugar is 'the new tobacco': Health chiefs tell food giants to slash levels by a third

BYLINE: SEAN POULTER

SECTION: HEALTH

LENGTH: 911 words

- Doctors and academics say levels must be reduced by up to 30 per cent
- They found that even zero-fat yoghurts can contain five teaspoons of sugar
- Heinz tomato soup has four while a Mars bar has eight teaspoons of sugar
- Obesity and diabetes already cost the UK over £5billion a year

Food giants are being told to cut the amount of sugar they use because it has become the 'new tobacco'.

Doctors and academics say levels must be reduced by up to 30 per cent to halt a wave of disease and death.

They found that even zero-fat yoghurts can contain five teaspoons of sugar, while a can of Heinz tomato soup has four.

The equivalent of 11 teaspoons are found in a small Starbucks caramel Frappuccino with whipped cream. A Mars bar has eight.

'Sugar is the new tobacco,' said Simon Capewell, professor of clinical epidemiology at the University of Liverpool.

'Everywhere, **sugary drinks** and junk foods are now pressed on unsuspecting parents and children by a cynical industry focused on profit not health.

The obesity epidemic is generating a huge burden of disease and death.

Obesity and diabetes already cost the UK over £5billion a year. Without regulation, these costs will exceed £50billion by 2050.'

Professor Capewell is part of a new US-UK campaign group - Action on Sugar - that says asking firms to make voluntary changes has failed.

The typical Briton consumes 12 teaspoons of sugar a day and some adults consume as many as 46.

The maximum intake recommended by the World Health Organisation is ten, although this guideline is likely to be halved.

The UN agency says there is 'overwhelming evidence coming out about **sugar**-sweetened **beverages** and other **sugar** consumption' being linked to obesity, diabetes and cardiovascular disease.

A study by Action on Sugar found surprisingly high levels of sugar in many foods, including savoury products and healthy options.

The Pret a Manger Very Berry Latte with milk has 26.9g of sugar - the equivalent of seven teaspoons. Yeo Valley Family Farm 0% Fat Vanilla Yogurt has five.

Even Glaceau Vitamin Water, which is owned by Coca-Cola, has the equivalent of four teaspoons of sugar in a 500ml bottle.

Action of Sugar said food firms should be able to reduce the amount of sugar they add to products by 20 to 30 per cent within three to five years, taking 100 calories a day out of the typical diet.

This would be enough to halt or even reverse rising levels of obesity and associated ill-health, it claimed.

Graham MacGregor, a professor at the Wolfson Institute of Preventive Medicine in London and chairman of Action on Sugar, said: 'We must now tackle the obesity epidemic both in the UK and worldwide.

'We must start a coherent and structured plan to slowly reduce the amount of calories people consume by slowly taking out added **sugar** from foods and **soft drinks**.

'This is a simple plan which gives a level playing field to the food industry, and must be adopted by the Department of Health to reduce the completely unnecessary and very large amounts of **sugar** the food and **soft drink** industry is adding to our foods.'

Dr Aseem , the group's science director, said: 'Added sugar has no nutritional value whatsoever, and causes no feeling of satiety.

'Aside from being a major cause of obesity, there is increasing evidence that added sugar increases the risk of developing type 2 diabetes, metabolic syndrome and fatty liver.

'We must particularly protect children from this public health hazard and the food industry needs to immediately reduce the amount of **sugar** that they are adding, particularly to children's foods, and stop targeting children with massive advertising for high calorie snacks and **soft drinks**.'

But sugar manufacturers rejected the claims of the health experts saying they were not supported by the consensus of scientific evidence.

Sugar Nutrition UK said the World Health Organisation published a review last year that found that any link between diabetes and body weight was due to overconsumption of calories and was not specific to sugar.

It said: 'There have also been numerous studies, which have investigated potential links between sugar and diabetes, with experts from the British Dietetic Association, European Food Safety Authority, and Institute of Medicine being very clear that diabetes is not caused by eating sugar.

Respected expert committees have reviewed the evidence over many years and all have concluded that the balance of available evidence does not implicate sugar in any of the so-called lifestyle diseases.'

And Barbara Gallani, of the Food and Drink Federation, an industry group, also denied sugar was responsible for obesity.

She said the industry already provided clear information on sugar levels to consumers, using figures and colour-coded labels.

'Sugars, or any other nutrient for that matter, consumed as part of a varied and balanced diet are not a cause of obesity, to which there is no simple or single solution,' she added.

Professor Shrinath Reddy, a cardiologist at the Harvard School of Public Health and member of the WHO panel of experts, disputed this conclusion.

He said there was 'overwhelming evidence coming out about **sugar**-sweetened **beverages** and other **sugar** consumption links to obesity, diabetes and even cardiovascular disease'.

Yoni Freedhoff, assistant professor of medicine at the University of Ottawa, said sugar needed again to become an occasional treat rather than a regular 'crutch'.

He said that added sugar had found its way into virtually everything we eat.

LOAD-DATE: January 9, 2014

LANGUAGE: ENGLISH

PUBLICATION-TYPE: Web Publication

JOURNAL-CODE: WEBDM

Copyright 2014 Associated Newspapers Ltd.
All Rights Reserved

MailOnline

January 8, 2014 Wednesday 5:41 PM GMT

FTSE CLOSE: Mothercare sheds weight after shock profits warning; Footsie slips as investors await US Fed minutes

BYLINE: THIS IS MONEY REPORTERS

SECTION: MARKETS

LENGTH: 2876 words

17.30 (CLOSE): A shock profits warning sent Mothercare shares plunging by nearly a third today as Sainsbury's also scaled back sales guidance after a competitive Christmas for retailers.

The babycare products chain fell 31 per cent after it said a 9.9 per cent drop in UK sales would see profits fall short of City expectations, while supermarket Sainsbury's dropped 2 per cent in the FTSE 100 after like-for-like quarterly sales growth nudged up just 0.2 per cent.

Overall, the FTSE 100 Index slipped 33.7 points to 6721.8 as investors awaited the minutes of the US Federal Reserve's latest meeting, which should offer more guidance about its tapering plans.

The Dow Jones Industrial Average fell nearly 60 points in early trading on Wall Street, despite survey figures showing US businesses added the most jobs in a year in December.

The jobs data fuelled hopes of a pick-up in economic growth worldwide, spurring on the pound, which rose to 1.65 US dollars and 1.21 euros.

Among stocks, Mothercare shares sank 128.5p to 291.5p after confidence in its turnaround plan was hit by today's profits warning.

Quarterly sales in its loss-making UK business were lower because of fierce competition over Christmas and the impact of recent store closures on year-on-year comparisons.

Currency deflation also impacted its overseas business.

It is the second high street retailer to warn over profits after Debenhams issued its alert on New Year's Eve.

The department store chain was also lower today, down 0.6p at 76.3p.

Sainsbury's was likewise in the red in the FTSE 100 as its admission that full-year sales were now likely to miss targets took the shine off a better-than-expected festive sales performance.

It maintained its nine year track record of consecutive like-for-like sales growth in the 14 weeks to January 4, but offset this by confirming it no longer expects to achieve an annual sales increase of 1 per cent to 1.5 per cent.

Analysts at Barclays said 0.7 per cent to 0.8 per cent growth was now more likely as they also shaved their forecast for annual pre-tax profits.

Sainsbury's shares were 8.9p lower at 360p, while Tesco slipped 3.5p to 328.3p ahead of its own trading update tomorrow.

Morrisons and Marks & Spencer bucked the trend with a rises of 0.6p to 254.2p and 3.8p to 444.9p respectively.

M&S is expected to announce a 1.5 per cent drop in clothing sales tomorrow, although this is likely to have been offset by much better trading in its food aisles.

Elsewhere, shares in RSA Insurance continued their recovery on hopes that an independent report expected on Thursday will rule out the need for any more write-downs in its troubled Irish business.

Shares were 3p higher at 100.7p, a rise of 3 per cent.

The biggest FTSE 100 risers were RSA Insurance 3p higher at 100.7p, Royal Bank of Scotland up 7.5p to 357.9p, Glencore Xstrata 6p ahead at 314.8p and Lloyds Banking Group 1.3p stronger at 83.8p.

The biggest FTSE 100 fallers were Aberdeen Asset Management down 17.7p to 473.3p, Tate & Lyle off 27.5p to 773p, Imperial Tobacco 67p lower at 2247p and Aggreko 50p weaker at 1690p.

15.30: London shares US stocks fell on Wednesday as investors took profits from the S&P 500's biggest daily gain in three weeks, and following a better-than-expected private sector jobs report.

As the last hour of trading loomed, the FTSE 100 index was down 28.1 points at 6,727.4. In New York, the Dow Jones Industrial Average fell 65.7 points to 16,465.2.

The better-than-expected jobs data strengthened the case for further tapering of the Fed's bond-buying programme, which has boosted equities strongly over the past year.

Investors were awaiting minutes from the Federal Reserve's December meeting, when the US central bank shaved $10billion off its bond-buying programme to $75billion a month, which are due to be released later today.

In London, **sugar** group Tate & Lyle was the top blue chip faller, down 24.0p to 776.5p after a group of health experts launched a campaign to reduce the amount of **sugar** added to food and **soft drinks** as part of an effort to reverse the UK's obesity and diabetes crisis.

Action on **Sugar** aims to help the public avoid products "full of hidden **sugars**" and encourage manufacturers to reduce the ingredient over time. It says children are a particularly vulnerable group who are targeted by marketers of calorie-dense snacks and **sugar**-sweetened **soft drinks.**

Blue chip housebuilder Persimmon was higher though, up 1.8p to 1,295.8p as Britain's property market recovery helped the firm deliver a better-than-expected rise in annual revenues after strong demand sent home sales up by a quarter in the final six months of 2013.

The Charles Church and Westbury Partnerships builder said it was on track for strong growth in underlying profits for 2013 and signalled another robust year ahead with forward sales up 41 per cent.

House prices last month fell for the first time in almost a year but continued to rise annually, according to the latest Halifax survey published today. Despite a 0.6 per cent drop in December - the first decrease since January 2013 - house prices grew by 7.5 per cent year-on-year, remaining close to a six-year high of 7.7 per cent recorded in November.

And the availability of mortgages is expected to significantly increase in the first-quarter of 2014, a Bank of England report said today, as interest rates stay low adding to worries about another UK housing market bubble.

'The driving factor in this surge has been attributed to promotions by lenders and the governments Help to Buy schemes. With house pricing rising 5.5 per cent across the UK for the year to October compared to 12 per cent in the capital has stoked investor's fear that a bubble is forming in the capital,' said Alex Conroy, financials trader at Spreadex.

Banks continued their recent strength on further evidence of a lending recovery in the UK, with Royal Bank of Scotland rising 8.3p to 358.7p, and Lloyds Banking Group up 1.4p to 83.8p.

Blue chip miners were higher too with Glencore Xstrata the best performer, ahead 6.7p to 315.6p following a report it plans to restart production at its Pasar copper smelter in the Philippines, shut since early November following typhoon damage. A bullish sector note from JP Morgan also helped Glencore, with the bank repeating an overweight stance on the firm.

Lower down the food chain, pizza delivery firm Domino's Pizza was also in demand, ahead 11.0p at 514.0p as it revealed faster growth in the UK today as it stepped up the search for a successor to departing boss Lance Batchelor.

The pizza delivery chain saw like-for-like sales rise 10.9 per cent over the 13 weeks to December 29, partly driven by more online marketing and the timing of Christmas. It means full-year profits will be ahead of forecasts for the UK and Ireland, although losses in the group's German arm mean they are likely to remain as expected overall.

But one of the day's biggest fallers was International Personal Finance (IPF), down 26.3p to 483.7p as the emerging markets credit company was hit by a £2.4million fine from Polish authorities at the end of 2013. Panmure Gordon cut its rating for IPF to sell from buy on the news.

13.15: Falls by supermarket groups and weakness in tobacco firms weighed on the Footsie at lunchtime as traders awaited some US data to provide fresh direction.

Overall, the FTSE 100 index was down 30.5 points to 6,725.1 as investors awaited the minutes of the Federal Reserve's December policy meeting, which should offer more guidance about its economic stimulus plans. Last month the US central bank trimmed its bond-buying programme by $10billion to $75billion a month.

Traders will also eye a US private employers jobs report due out today for clues to Friday's important monthly non-farm payrolls (NFP) data.

'ADP jobs data (NFP warm-up) could rekindle optimism should it meet 200K expectations and boost hopes that NFP prints the same on Friday reinforcing recovery hopes and optimism and supporting the Fed's decision that one half of its mandate is on the mend and warrants stimulus reduction,' said Mike van Dulken, Head of Research at Accendo Markets.

In London, Imperial Tobacco and British American Tobacco fell, with traders citing a report Hong Kong's South China Morning Post newspaper that health authorities in China aim to roll out a nationwide smoking ban in public places by the end of this year.

'With China, there's a lot of concern over smog and air pollution. The doors have been flung open with regards to reform, and with that will come a change in standards with regards to healthcare,' said Alastair McCaig, analyst at IG.

'It will increasingly become the focus, and that's a battle that tobacco are going to have to face in the coming years.'

Imperial Tobacco shares were down 54.9p at 2,259.1, with BAT off 28.5p at 3,157.0p.

Supermarket groups also occupied places on the FTSE 100 fallers board after Sainsbury's said it will no longer achieve a full year like-for-like sales increase of 1-1.5 per cent as it saw its sales edge 0.2 per cent higher in the 14 weeks to January 4.

Analysts at Barclays said 0.7-0.8 per cent growth was now more likely for Sainsbury's as they also shaved their forecast for annual pre-tax profits.

Sainsbury's shares were 7.2p lower at 361.7p, while Tesco slipped 4.9p to 326.8p ahead of its own trading update tomorrow. Morrisons, which updates the market next week, fell 2.7p to 250.9p.

Outside the top flight, Mothercare shares slumped 115.5p to 303.5p after confidence in its turnaround plan was hit by today's profits warning.

Debenhams, which issued a profits alert on New Year's Eve, fell 0.5p to 76.3p.

In other retail news, wine merchant Majestic recorded like-for-like UK store sales growth of 2.8 per cent for the 10 weeks to January 6, boosted by purchases of wine bottles costing more than £20. While analysts said the figures were in line with recent trends, shares still fell 13.0p to 535.0p.

But Marks & Spencer bucked the dull retail trend with a rise of 3.8p to 444.9p. It is expected to announce a 1.5 per cent drop in clothing sales tomorrow, although this is likely to have been offset by much better trading in its food aisles.

And shares in RSA Insurance continued their recovery on hopes that an independent report expected on Thursday will rule out the need for any more write-downs in its troubled Irish business. RSA shares were 3.9p higher at 101.6p, having gained 6 per cent on Tuesday, helped also be some bid speculation.

11.25: London stocks have fallen deeper into the red as the session progresses - the FTSE 100 was down 27.2 points at 6,728.3 in late morning trading.

'In a morning full of surprises the FTSE has given back ground in early trade, lagging its European peers as the retailers endured a tough session,' said Toby Morris of CMC Markets.

'The major story of the morning was an unexpected about turn from the major supermarkets after Sainsbury's and Waitrose released numbers.

'The session started off in good spirits, with both Sainsbury's and Waitrose reporting bumper Christmas sales, with the former extending a record 35 consecutive quarters of growth after having been tipped to end the run by some analysts.

'All the supermarkets, including Tesco and Morrisons whose Christmas updates are yet to come, dovetailed initially only to spectacularly reverse after comments from Sainsbury's in a morning conference call.

'Chief financial officer John Rogers revised full year guidance lower and warned of a tough financial fourth quarter as consumers rein in their spending after a loose Christmas and by mid-morning the news had everyone back firmly in the red again.

'Staying with retail, It was a bit of bloodbath for Mothercare this morning, down over 30 per cent after being forced to issue a full-year profit warning and throwing a dummy to a market caught off guard after a strong start to January prior to the update.'

David Madden of IG said investors were cautious ahead of the US Federal Reserve minutes due to be released tonight.

'Traders are playing it safe as they await the update from the Fed at 7pm London time. Tonight's minutes will give us an insight into why the US central bank decided to trim its bond-buying scheme, and we are likely to see low volumes and volatility until then.'

9.30:

The FTSE 100 slid 16.2 points to 6,739.3 as traders digested the latest round of Christmas updates from the retail sector.

Fallers included Sainsbury's as a better-than-expected 0.2 per cent rise in underlying sales for the Christmas quarter was offset by its warning that like-for-like sales for the whole year will be lower than hoped.

Shares initially rose 3 per cent but were later 5.5p lower at 363.3p. Read more here.

Rival Tesco, which reports figures tomorrow, fell 3.3p to 328.45p and Debenhams dropped 1p to 75.85p following its profits warning last week.

Marks & Spencer bucked the trend as its shares rose 3.5p to 444.65p on the eve of its eagerly-awaited trading figures.

Outside the top Footsie index, Mothercare shares took a hammering as it became the second major retailer to warn that it will miss profit expectations.

The babycare products chain said UK quarterly sales were 9.9 per cent lower because of fierce competition over Christmas and the impact of recent store closures on year-on-year comparisons.

With currency deflation also impacting its overseas business, shares slid by 31 per cent or 131p to 289p as it warned that full-year profits were likely to be below the current range of City expectations. Read more here.

Elsewhere, shares in RSA Insurance continued their recovery on hopes that an independent report expected on Thursday will rule out the need for any more write-downs in its troubled Irish business. Shares were 2.3p higher at 100.1p.

8.25:

The FTSE 100 has opened down 2 points at 6,753.4, as investors wait for fresh signs that key economies are picking up before committing more money to stocks.

Recent strong economic figures from Germany have raised market expectations before the release of its trade balance and industrial orders data, both forecast to show an increase in November.

The eurozone unemployment rate and US private-sector hiring data are also due out today, while the US Federal Reserve will release minutes of its last meeting after the close tonight.

The Bank of England and European Central Bank's first interest rate decisions of the year are scheduled tomorrow

But probably most influential for markets will be the the US non-farm payrolls jobs report for December, due this Friday.

Craig Erlam of Alpari said: 'We have seen a pickup in activity over the last couple of days, as traders return to their desks following the winter break, but trading volumes have still been a little lower than normal due to the large number of high volatility events scheduled for the end of the week.

'Well things should pick up more today, with a number of important pieces of economic data due to be released, followed this evening by the FOMC [Federal Open Market Committee] minutes.

The FTSE 100 closed 24.72 points higher at 6,755.45 yesterday, helped by gains in financial stocks.

Brent crude held its ground above $107 per barrel, supported by new worries over Libyan supplies and expectations of another drop in US crude inventories. Gold is at $1,226.30 an ounce.

Stocks to watch today include:

J SAINSBURY: The supermarket posted a small rise in underlying sales in its Christmas quarter, though its rate of growth slowed.

LLOYDS BANKING GROUP: A mass sell-off of the government's 33 per cent stake in Lloyds Banking Group has moved a step closer.

The management of the bailed out bank was asked by UK Financial Investments, which looks after taxpayers' shares, to draw up the detailed information needed for a sale, the Guardian newspaper and Sky News reported.

SSE: The energy supplier will cut its prices in response to a government pledge to remove some taxes and social charges from bills by the end of the current financial year, later than most of its rivals.

ASTRAZENECA: The drugmaker has signed a deal with private biotech company Immunocore to boost its experimental cancer drug pipeline as it seeks to find new medicines to replace those going off patent.

GLENCORE XSTRATA: The company's Pasar copper smelter in the Philippines plans to restart as soon as January 15, after being damaged by Typhoon Haiyan in November, two sources said.

MOTHERCARE: The mother and baby products retailer warned that annual profit would be below current market forecasts, hit by Christmas discounting in Britain, plus weak economic conditions and currency deflation overseas.

PERSIMMON: The housebuilder said full year revenues jumped 21 percent.

BAE SYSTEMS: The company chose Jerry DeMuro to head its US business, pinning its hopes on the former General Dynamics executive to steer the weapons maker through military cuts that are expected to bite over the next decade.

FERREXPO: The Ukrainian iron ore miner said its iron ore pellet output rose to a record high for the company in 2013 even as production fell slightly in the last quarter of the year.

LOAD-DATE: January 9, 2014

LANGUAGE: ENGLISH

PUBLICATION-TYPE: Web Publication

JOURNAL-CODE: WEBDM

Copyright 2014 Associated Newspapers Ltd.
All Rights Reserved

THE SUNDAY TIMES
The Sunday Times (London)

January 5, 2014 Sunday
Edition 1;
National Edition

THE PLAN;
ELIMINATING SUGAR FROM YOUR DIET IS NO EASY TASK, SO TAKE IT SLOWLY. HERE'S HOW

SECTION: STYLE;FEATURES; Pg. 34,35

LENGTH: 1341 words

STEP ONE

If you were trying to quit drinking or smoking, the first thing you would do is throw away the cigarettes and give your alcohol to a friend. We need to strip your kitchen of as many unmistakable **sugar** sources as possible, followed by a special-ops mission to search out all the hidden **sugars** that are undoubtedly residing on your shelves. Use this first week slowly but systematically to eliminate the bad stuff. We're starting with four categories: sauces, cereals, **soft drinks** and sweets.

SAUCES

Without even looking you can throw away ketchup, barbecue sauce and any other "brown" sauce you may be partial to. Then move on to salad dressings, which I can pretty much guarantee will be loaded with sugar. Out they go. Next, look at the pasta sauces I am sure you have for last-minute Italian nights, and you will notice that most tomato-based varieties (even the organic ones) are crammed with sugar.

CEREALS

They get a bad rap for a good reason. You will be hard-pressed to find a conventional cereal brand without sugar, often as one of the first three ingredients. This is not a productive way to start your or your children's day. Finish off the ones you have (if you can't stand the waste) and then commit to buying only cereals without sugars. However, even those with low sugar, such as Weetabix and Shredded Wheat, aren't great for weight loss since they are made of wheat and so have a high GI. Try to limit cereal to a few times a week, because there are far more balanced and nutrient-rich meals with which to start your day. Try some of my easy breakfast alternatives, which don't take much longer to whip up, but are packed with good fats and lasting energy.

SOFT DRINKS

The nutritionist Libby Limon says: "It is thought that our hunger mechanisms do not register the calories from diluted sugars in the same way that we do from whole foods, which means that you can add hundreds of extra calories to your diet without even noticing." So, if you like **fizzy drinks** (regular or diet), start limiting your intake. If you are a four-cans-a-day person (yikes), then cut it to two, and if you drink five a week, limit it to two. And don't be fooled into thinking iced teas, flavoured waters, lemonades, Red Bull and "vitamin" waters are sugar-free options, because they are not. The diet versions of the above are obviously lower in calories, but experts agree that artificial sweeteners taste a lot sweeter than regular sugar, which conditions people to crave more sweet foods. They also trick our metabolisms into thinking sugar is on the way, which causes surges of insulin and more fat. The nutrition researcher David L Katz says: "We refer to a 'sweet tooth', not a 'sugar tooth', and I think that is absolutely right. Our tastebuds don't really differentiate between

sweet in sugar and sweet from, say, aspartame." He adds: "What I have seen in my patients is that those who drink diet soda are more vulnerable to stealth sugars like those added to processed foods that don't taste sweet, such as crackers, breads and pasta sauce."

SWEETS

This is a pretty self-explanatory, but just to be clear: I mean everything from Percy Pigs, Jelly Babies and chocolate bars to homemade brownies, ice cream and doughnuts. Even if you make a habit of sucking throat lozenges, those have to go, too. Think of this as the "easy" category to give up, because it's so straightforward. Nobody can pretend eating a bag of Minstrels is a good thing.

STEP TWO

Keep a sugar diary. It doesn't have to be scientific or precise, but at the end of the day or the week, you will have an honest and comprehensive view of how much sugar you are really taking in. Write down the obvious sugary indulgences and when you eat them, but also record all the hidden sugars you find. It's far easier to make changes when you know exactly what you are up against. If you notice that afternoons or late nights are your biggest struggle, then those times should be your primary focus. Don't worry about having a little jam on your porridge if you are bingeing on chocolate every afternoon at 3pm - focus on that. One battle at a time.

STEP THREE

You now know what you need to eliminate, but what should you add to fill the gaping sugar holes? Let's start with some small but powerful swaps.

BREAKFAST

It is all right to treat yourself to coffee first thing, but, please, no sugar. If you're not ready to give up toast, try topping it with coconut oil, almond butter or sliced avocado instead of jam or marmalade. Once you are off toast altogether, start eating an omelette with spinach (use frozen spinach, it takes no time at all) or porridge made out of rolled oats, quinoa flakes and millet, topped with coconut flakes and sliced almonds. If you are devoted to fruit yoghurt, start buying whole-fat plain yoghurt or coconut yoghurt instead (remember, eating fat doesn't make us fat), and top it with a handful of raspberries and walnuts. I am a huge believer in green smoothies - start making them. I have them for breakfast, but also as snacks and even as a late-night treat if I'm really craving something sweet.

LUNCH AND DINNER

Focus on combining lots of green vegetables with good fats and proteins such as salmon, lean chicken, eggs, tofu, feta cheese, goat's cheese and avocado. Just remember to leave off any ready-made salad dressings, sauces and marinades. Use lemon, olive oil, nut oils, tamari, spices or mustards as flavour enhancers instead. And if you crave something sweet in the afternoon, or after dinner, be prepared with a low-sugar alternative. I often cut up an apple and eat it with a few teaspoons of almond butter, or I have a bowl of pure coconut or whole-fat yoghurt topped with a tablespoon of chia seeds, blueberries or raspberries or walnuts. Anything coconut-based is good for curbing a sugar craving. Buy whole coconuts and chop them up into chunks; or toasted coconut flakes are a good takeaway option. Some nutritionists even suggest a spoonful of coconut oil takes the edge off a craving.

SNACKS A plate of chopped raw vegetables with hummus, pesto or guacamole makes a great snack. As do nuts of every variety, sunflower and pumpkin seeds, kale chips, a handful of coconut flakes, a bowl of dark berries, or celery sticks topped with nut butter. Olives, flax crackers and rye toast are good, as are hard-boiled eggs, sticks of cheese or half an avocado. And for refreshments, make your own herbal and green teas (hot and cold), and try a whole lemon squeezed into a large glass of water as an alternative to sweet drinks. Good luck. ? v ocado. een d et Stay in touch throughout the week and tell us how your adventure eliminating sugar is going. Tweet us at @THESTSTYLE and @calgaryavansino with the hashtag #byebyesugar, or instagram us at @TheStStyle and @calgaryavansino www.calgaryavansino.com NEXT WEEK Lots more nutritious recipes and alternatives for a sugar-free existence. Plus, learn more about why we are addicted to sugar Don't miss Dr Robert Lustig's video explaining the trouble with sugar at www.thesundaytimes.co.uk/style

YOUR SUGAR-FREE SHOPPING LIST

Breakfast Coconut oil, almond and peanut butter, apples, avocado, eggs, frozen spinach, plant-based protein powder, gluten-free bread, rolled oats, quinoa flakes, millet, coconut flakes, sliced almonds, plain full-fat and coconut yoghurts, unsweetened coconut and almond milks, raspberries and walnuts Lunch and dinner Green vegetables, lean protein (such as chicken, salmon, eggs, tofu, feta and goat's cheese) Snacks Raw veg, hummus, pesto, guacamole, cheese, roast chickpeas, fresh coconut, coconut flakes, mixed nuts, kale chips, celery sticks, olives, flax crackers and rye toast Drinks Green and herbal teas

CALGARY'S GREEN SMOOTHIE

2 cups bottled water

1½ cups coconut water

2 handfuls of frozen or fresh fruit: blueberries, raspberries, kiwi and lime are all low in sugar and high in fibre

Handful of frozen kale

Half an avocado

1 tbsp almond butter

1 tbsp chia seeds

Protein powder - not whey- but plant-based. My favourite is Sunwarrior

1 tsp lucuma powder (a natural sweetener)

LOAD-DATE: January 5, 2014

LANGUAGE: ENGLISH

GRAPHIC: GETTY

PUBLICATION-TYPE: Newspaper

JOURNAL-CODE: STS

Copyright 2014 Times Newspapers Limited
All Rights Reserved

THE SUNDAY TIMES
The Sunday Times (London)

January 5, 2014 Sunday
Edition 1;
National Edition

THE SUGAR TRAP;
PART ONE FORGET DIETS: THE REAL REASON WE ARE PUTTING ON WEIGHT IS ALL THE SWEET STUFF HIDDEN IN OUR FOOD. CUT IT OUT AND WATCH THE POUNDS SLIP AWAY. CALGARY AVANSINO EXPLAINS HOW

BYLINE: FLOSSIE SAUNDERS

SECTION: STYLE;FEATURES; Pg. 30,31,32,33

LENGTH: 2370 words

The number one new year's resolution is to lose weight, and yet we are failing spectacularly. Despite the growing list of diets - from Atkins and Caveman to the fasting or 5:2 diet - none of them ever seems to work, or work long term. Instead, we are getting fatter. There are now 1bn overweight adults worldwide, and 300m of them are clinically obese. In Britain, a 2012 NHS survey found that more than a quarter of all adults in England are obese - rates that have risen threefold since 1980. Increasingly, however, experts here and in America are beginning to wise up to the real culprit behind our ever-increasing girths. Rather than fat, as was originally thought, it is sugar that is the biggest threat to our health. As our sugar consumption has increased, so has our weight, and the more we eat, the more unwell and overweight we become.

The NHS reckons the average person in Britain now consumes about 700g of sugar a week - that's 140 teaspoons. Experts say our bodies are designed to handle only half that or less a week. If you really want to look and feel better in 2014, then forget about following a diet: make quitting sugar your new year's resolution. So what exactly is this socially acceptable drug; why is it making us increasingly overweight and unwell; and why can't we stop eating it? Any ingredient that ends in "ose" is a sugar, and there's a mighty long list of them: glucose, fructose, dextrose, maltose, sucrose, lactose and high-fructose corn syrup. The white granulated sugar you put in tea is harvested and refined from sugar beets and sugar cane and, like all other sugars, it has absolutely no nutritional value - no proteins, no essential fats, no vitamins or minerals. These "oses" are the emptiest of empty calories. It's just pure, refined energy. It contains a whole bunch of calories and nothing else.

When we eat any form of sugar, the body deals with it in one of two ways. Either we burn it off as energy - but, given the amount of sugar the average person now consumes, it is impossible to expend it through activity unless you are Mo Farah (and I guarantee you he limits his sugar intake) - or, if it isn't burnt off, it is converted into fat by the liver and stored directly in the fat cells.

The nutritionist Amelia Freer says: "If the amount of glucose in the bloodstream is above the body's comfort zone of about 1Åtsp-2 tsp at any one time - one regular can of Coke has 9 tsp - then the hormone insulin gets produced to chauffeur the excess glucose out of the blood and store it as fat. Elevated levels of insulin circulating in our bodies can be detrimental to our long-term health. Our cells can become less responsive to the presence of insulin, meaning our bodies keep needing to produce more and more insulin to get the same reaction. Eventually the cells stop responding at all. This is type 2 diabetes."

Dr Robert Lustig, author of Fat Chance, says: "In 2011, there were 366m diabetics in the world - more than double the number in 1980." Furthermore, the Center for Science in the Public Interest in America reports that "sugar consumption has increased by 28% since 1983, with many individual foods providing large fractions of the US Department of Agriculture's recommended sugar limits".

HOW MUCHSUGARSHOULDWEEAT?

Most health organisations recommend that people limit themselves to 10 tsp (40g) of added sugars a day, but many researchers say it should be as low as 6 tsp for women and 8 tsp for men. Teaspoons are a much easier measurement to visualise than grams, so lock this easy equation in your head: divide the number of grams by four to get the number of teaspoons. To put that in perspective: a regular Snickers bar contains 27g or about 7 tsp sugar, a 330ml can of Coke has 35g or 9 tsp of sugar, three Oreos have 14g or 3Å tsp, and a chocolate-glazed Krispy Kreme doughnut has 26g or 6½ tsp of sugar.

SUGAR PURE,RENER GYCONTA WHOLE OF CALAND NO

We shouldn't eat manufactured sugar bombs like that, full stop. Any food with sugar in the first three ingredients is a bad idea. Sadly, it's not as simple as cutting out foods that you know are packed with sugar. Lustig (whose YouTube video Sugar: The Bitter Truth is well worth watching) says: "The food industry has contaminated the food supply with added sugar to sell more products and increase profits. Of the 600,000

food items in American grocery stores, 80% have been spiked with added sugar; and the industry uses 56 other names for sugar on the labels. They know E L when they add sugar, you buy more. And because you do not know you're buying it, you buy even more."

So, are we actually physically addicted to the sweet stuff? Most nutritionists respond to that question with: "Try giving it up and then tell me what you think."

The chairman of the Functional Medicine Institute, Dr Mark Hyman, believes we are. "The slick combinations of sugar, fat and salt in junk and processed food have hijacked our tastebuds, brain chemistry and metabolism. These foods are biologically addictive.

S JUST FINED THAT

We are held hostage by the food industry and yet we blame ourselves for not having willpower," he says. "One animal study found that sugar is more addictive than cocaine. When rats were given the choice between cocaine or sweetened water, scientists found that most rats preferred the sweetened water. Even the rats who initially preferred cocaine switched over."

IN A UNCHORIES THING

ESugar improves our mood by prompting the brain to release the "happy" hormone serotonin, which is exactly why we turn to it when we are happy and celebrating, but also when we are sad, lonely or tired. The problem is that what goes up must come down, and those inevitable sugar crashes just make us crave more sugar and encourage a cycle of binge eating that makes us increasingly overweight and www.unwell.BE WARE THE HIDDEN SUGAR

The nutritionist and naturopath Rhian Stephenson tells her clients to check all labels of canned vegetables, breads, sauces, preprepared foods and so-called "health" foods carefully. "If sugar, or a sugar pseudonym, is one of the first three ingredients, steer clear." Even though it is a long list, it is important that you acquaint yourself with the vocabulary, much of which is made to sound healthy, organic and pure. The most common terms are: barley malt syrup, beet sugar, brown rice syrup, rice syrup, cane crystals, coconut sugar, corn sweetener, corn syrup, crystalline fructose, dextrin, evaporated cane juice, fruit juice concentrate, fruit purée, fruit pulp, agave, molasses, organic evaporated cane juice, palm sugar, raw sugar, saccharose, sorghum, treacle, turbinado sugar and xylose.

Sugar is often present in foods you don't even associate with sweetness: pasta sauces, canned salmon, breaded fishfingers, porridge, fruit yoghurt and bouillon cubes. I could scarcely find a breakfast cereal, a deli meat or an Asian cooking sauce that wasn't loaded with added sugar. And that's before I got to the plethora of "healthy", "organic" and "light" products that are boosted with sugar to compensate for the lack of fat. The American dietician Susan Burke-March warns: "Just because a food is labelled 'low fat' or 'fat-free' does not make it calorie-free; manufacturers add sugar to increase the texture and bulk lost by removing fat."

HOW CALS CONS

Jenna Zoe, author of Super Healthy Snacks and Treats, says: "Craving sugary foods doesn't make you a weak human being. We are programmed to opt for sweet foods because, in nature, sweetness is a sign that foods are safe to eat; it meant that early man chose juicy fruit over poisonous plants that are bitter in taste. The problem arises with processed foods, because sweeteners are used in conjunction with junky fats or hydrogenated oils. This is where the addictiveness is created. In nature, sugars and fats are not often found in the same foods."

ALCOHOL

The sugar content of alcoholic drinks varies greatly. Dry white wine and red wine have a relatively low fructose content, while dessert wine and champagne contain more. Stay away from mixed drinks, which are usually laced with sugary syrups and sodas. If you must indulge in spirits, choose a "clean" mixer such as sparkling water or fresh lemon juice. Most health recommend themselves added sugar researchers 6 tsp for for men. easier to grams, so easy equation: number of the number So, a regular contains of sugar, a Coke has **sugar**. We **drink** bombs, full with sugar ingredients Avoid foods 10g of sugar The author David Gillespie says in his book The Sweet Poison Quit Plan: "Alcoholic drinks are OK for the recovering sugarholic as long as they don't taste sweet and are not mixed with other drinks that contain sugar. You can keep the dry wines,

beers and spirits, but you need to toss out the dessert remember: drink ssert wines, ports, sweet sherries, liqueurs and mixers." But member: all alcohol is calorie dense, so if you want to lose weight, ink as little alcohol as possible.

CARBOHYDRATESARESUGARTO

O arches and carbohydrates are more of the same, I'm afraid.

Starches Our refined an more says: down pancreas cells. typically anything cereals; r bodies process certain types of carbohydrates (the white, ned kinds) in a similar way to pure sugar, and they create equally powerful endorphin response, making us want them re and more. The author of Grain Brain, Dr David Perlmutter, ays: "During the course of digestion, carbohydrates are broken wn and sugar is liberated into the bloodstream, causing the ncreas to increase its output of insulin so glucose can penetrate lls. The carbs that trigger the biggest surge in blood sugar are pically the most fattening, for that very reason. They include ything made with refined flour such as breads and reals; starches such as rice, potatoes and corn; and liquid carbs such as soda and fruit juice."

O&ATEARTION

The scientists at Harvard School of Public Health explain: "The glycemic index ranks carbohydrates on a scale from 0 to 100, based on how quickly and by how much they raise blood-sugar levels after eating. Foods with a high glycemic index, such as white bread, are rapidly digested and cause substantial fluctuations in blood sugar. Foods with a low glycemic index, such as whole oats, are digested more slowly, prompting a more gradual rise in blood sugar."

ions e ld organisations people limit tsp (40g) of but many should be and 8 tsp are than this get the divide by four. Eating too many high-glycemic foods may lead to an increased risk of type 2 diabetes, heart disease, some forms of ovulatory infertility and colorectal cancer.

A piece of toast is no longer just a piece of toast. Carbs such as white bread, white rice, pretzels, crackers and bagels are high on the glycemic index, while rye bread, pumpernickel bread, rolled oats, barley and quinoa fall in the low range. So although all carbohydrates are converted into sugar when digested, some are converted into more sugar than others. However, this doesn't mean a bread-free life for ever. Try experimenting with new flours such as almond flour, coconut flour, quinoa flour and flaxmeal. bar about 7 tsp can of tsp of eat or sugar Any food first three idea. more than portion.

During the initial sugar-free week of our plan, however, try to resist carbohydrates as much as humanly possible. If you do feel the need for carbs, choose the good ones: brown rice, rolled oats, millet, quinoa, buckwheat, wild rice, bulgur and rye.

THE TRUTHABOUTFRUIT

Fruit is not fundamentally bad for us, but the amount we are eating can be detrimental. In The Sweet Poison Quit Plan, the author David Gillespie recommends that adults eat only two pieces of fruit a day and children only one. Fruit containing higher amounts of fibre and lower quantities of fructose such as kiwis, apples, grapefruit, blackberries, pears, strawberries, blueberries, raspberries and lemons are the best choices, while bananas, watermelon, pineapple, mangoes, papayas and grapes should be avoided.

None of this matters when it comes to fruit juice, as it's all bad. When fruit is juiced, any positives are squeezed out and all that's left is sugar, water and a bit of vitamin C. Dried fruit is even worse and often contains nearly 70% sugar.

The author of Grain Brain, Dr David Perlmutter, says: "Our caveman ancestors did eat fruit, but not every day of the year. A medium-size apple contains 44 calories of sugar in a fibre-rich blend thanks to the pectin. If you juice several apples and concentrate the liquid down, you get a blast of 85 sugar calories."

If you want to think about it in terms of grams (as a benchmark a 330ml can of Coca-Cola has 35g of sugar), the average glass of orange juice has 21g of sugar, apple juice 28g, cranberry juice 37g and grape juice 38g, and many bottled fruit smoothies contain between 20g and 35g of sugar. All of which says we shouldn't be starting our mornings or hydrating our kids with fruit juice.

However, the Plenish Cleanse founder, Kara Rosen, reassures us that not all juice is evil. "The new juice taking the market by storm is cold-pressed vegetable juice, particularly green juices made up of ingredients such as cucumber, spinach, kale, broccoli and lettuce and low-glycemicindex fruit such as pears. The sugar content is lower than conventional juices, and due to the cold-press juice extraction method, they have other nutritional benefits." So, go green or go water.

HOW TO CALCULATE SUGAR CONSUMPTION

Most health organisations recommend that people limit themselves to 10 tsp (40g) of added sugar a day, but many researchers say it should be 6 tsp for women and 8 tsp for men. Teaspoons are easier to visualise than grams, so remember this easy equation: to get the number of teaspoons, divide the number of grams by four. So, a regular Snickers bar contains 30g or about 7 tsp of sugar, a 330ml can of Coke has 35g or 9 tsp of sugar. We shouldn't eat or drink manufactured sugar bombs, full stop. Any food with sugar in the first three ingredients is a bad idea. Avoid foods with more than 10g of sugar per portion.

PUBLICATION-TYPE: Newspaper

JOURNAL-CODE: STS

Copyright 2014 Times Newspapers Limited
All Rights Reserved

The Daily Telegraph

The Daily Telegraph (London)

January 4, 2014 Saturday
Edition 1;
National Edition

'Fat tax' isn't fair but it might be right

BYLINE: Judith Woods

SECTION: FEATURES; Pg. 26

LENGTH: 231 words

How dispiriting to discover that people in developing countries have seen their waistlines expand along with their economies, leading to a quadrupling of obesity rates.

According to the Overseas Development Institute, one in three people worldwide are now overweight due to changing diets.

The shift from cereals and grains to the fats, sugar and oils present in processed foods, which are regarded as aspirational, has led to one billion people in the world's poorest nations being classified as overweight or obese.

Grotesquely, obesity and under-nourishment are often present in the same households, as more calories does not automatically equate with more vitamins. The result is a health time-bomb.

So what to do? Researchers are calling on overseas governments to introduce a "fat tax" to push up the price of **fizzy drinks,** confectionery, fast food and other products containing high levels of sugar, fat and salt.

Here in Britain, where 64 per cent of us are overweight and cost the NHS £5 billion annually, there is a marked reluctance to accept any such meddling in our democratic right to eat microwave chips.

Lobbying for an imposition of a punitive tax on emerging nations might smack of the worst do-as-I-say-not-as-I-do paternalism.

It may be hard to swallow but sometimes, as with smoking bans, unpalatable decisions have to be made in order to save people from themselves.

LOAD-DATE: January 4, 2014

LANGUAGE: ENGLISH

PUBLICATION-TYPE: Newspaper

JOURNAL-CODE: DTL

Copyright 2014 Telegraph Media Group Limited
All Rights Reserved

The Telegraph

telegraph.co.uk

January 3, 2014 Friday 11:21 PM GMT

Surely Mary Berry could see off the East Europeans;
As PJ Harvey edits the Today programme, why aren't celebrities in charge of everything?

BYLINE: By Judith Woods

LENGTH: 1492 words

Unidentified object in the bagging area. I repeat, unidentified object of derision in the bagging area! Will someone please remove Keith Vaz from the Luton luggage carousel and send him packing, back to Leicester East?

Quite why the self-publicist MP would think it was a good idea to form a one-man welcoming party for incoming Romanians is a mystery. It also reveals him to be woefully out of touch.

Not only had he failed to get the memo regrettably informing him that the Transylvanian-born Cheeky Girls have been resident in the UK since 2002, but he clearly didn't know that the meet-and-greet gig has already been taken by reality-TV royalty.

Great British Bake Off queen Mary Berry is forthwith to be posted at arrivals to stem the Eastern European hordes with well-bred politesse, a plate of Eccles cakes and a gimlet eye for a soggy bottom.

Her hatchet-faced wingman, Lord Sugar - prime-time's answer to Vlad the Impaler - will be at her side, dispensing his own inimitable advice to any Romanians asking about benefits: "It's sink or swim and you've most probably picked up by now that I don't do life jackets."

Yes, I know it sounds crazy, but you know, it just might work. Following in the wake of singer PJ Harvey's magnificently calamitous stint guest-editing the Today programme in the style of Kim Jong-un, the nation's celebrities are being charged with the task of taking over and turning round the rest of Britain's ailing institutions.

Imagine it; how much livelier, not to say orchidaceously florid, the debate on the HS2 rail project would be if orchestrated by motormouth savant Russell Brand?

After the scandal of "crystal Methodist" and former Co-op Bank chair Paul Flowers, financial regulation is crying out for a decentralisation of power and a system of checks and balances; Ant and Dec, step forward please.

As the sector, like the Australian jungle, is beset with nests of vipers, and remains a forum where a load of balls is regularly served up for public consumption, the pair are perfectly suited to the macroeconomic challenges ahead.

Other vexing issues for politicos this year include the European elections (maybe it's time for those Cheeky Girls to hit the hustings and earn their keep again?) and the referendum on Scottish independence.

Needless to say, any Celt who manages to coax the spotlight off unspeakably smug Alex Salmond is to be commended, but I think we all know that the future of the United Kingdom or indeed a New Caledonia really ought to rest with David Tennant, so we'll be happy with whatever he decides, as long as he says it in his beguiling Scots burr. And appears in the second series of Broadchurch. With Olivia Colman.

You see, we've got a great pool of talent out there just waiting to be deployed, if only we can start thinking outside the Tardis.

Let's take the Home Secretary, Theresa May, who has taken to dressing so like bushy-browed Brit model Cara Delevingne (same Black Watch tartan suit, matching sexy thigh boots), that she has been forced to issue an "I'm not the new Cara Delevingne" statement. Which obviously means that Cara is the new Theresa May. What the 21-year-old fashion icon thinks of current counter-terrorism policy can only be guessed at, but I really do think she could make a fair fist of the Home Office if only she'd stop sticking her tongue out quite so often. Especially when addressing the Association of Chief Police Officers.

Other Cabinet posts could quite easily be filled with One Direction, larking about in dropped-crotch skinnies; Harry Styles would make a top-drawer foreign secretary, as he's got more tattoos than a merchant seaman and, as far as the post of prime minister goes, first dibs must go to Kirstie Allsopp.

There was something rather marvellous about the way she called on the Spirit of the Blitz during the floods, and complained about lily-livered householders having the temerity to expect compensation. Then it emerged that not one of her three homes has been affected by floodwater. Oh dear. Still, someone so blithely unconcerned about a poor press deserves to be In Charge of Everything.

Meanwhile, what of our elected representatives? How should our politicians fill their days? They could, like John Hemming MP, drunkenly text Mumsnet from the naughty step. Or hang around coach stations to tell anyone who will listen (and can speak English) that Britain is open for business. Because, I suspect, left to Ant and Dec and Mary and Cara, it soon could be.

The best 101st birthday present

What to give a man on his 101st birthday? Last year my esteemed father-in-law, the artist Derek Clarke, received a retrospective exhibition at the Royal Scottish Academy and a signed card from the Queen. Now that can't be bested, can it?

Oh yes it can! With an MBE.

By pure coincidence, publication of the New Year's Honours List came on Derek's 101st birthday. And so, as the extended family gathered in Edinburgh for the milestone birthday, we glowed at the royal recognition of a lifetime passionately devoted to art.

Derek is, quite simply, a one-off. As fully present as ever he was, any frailties of body have not diminished his fiercely enquiring mind.

To my 11-year-old daughter he dispensed the sort of wise advice she wouldn't countenance from me, but, coming from her much-loved, much-lived grandfather, she treasured it as a precious gift.

He laughed with requisite delight as my five-year-old put on an acrobatic show in her new Rapunzel princess frock. He solicitously enquired after work and holiday plans. And then, amid the clinking of champagne flutes, he raised a hand and we paused expectantly while he addressed us.

Was he about to tell one of his fabulous anecdotes? Talk about the 1943 Tunisian Campaign? Reflect on the vicissitudes of a century?

No, he looked at each one of us; from his devoted wife, Pat, to his youngest grandchild in her scratchy nylon Disney dress and said two little words more resonant, humbling and revealing than any encomium; thank you.

Brownies - couldn't you just eat one?

Who doesn't love a Brownie? Pretty much impossible to resist, aren't they?

No, I'm not talking about Nigella's comfort eating but the guiding organisation set up in 1914 to prove that girls were made of much stronger stuff than sugar, spice and all things nice.

It's the Brownies' centenary this year and I don't mind saying I go all misty-eyed at the sight of them, despite the troubling trendification of the uniform, with gilets and hoodies and yellow baseball caps.

I was never allowed to be a Brownie, which had less to do with their militaristic founding father Lord Baden-Powell's secret talks to twin the Scouts with the Hitler Youth (yes, really) than the fact my mother couldn't be bothered driving me to meetings.

Shame I inherited her laziness when it came to my own daughters, because you can't argue with the merits of a Brownie badge scheme that teaches crucial life skills such as reading bus timetables, painting a circus clown's face and constructing a life-sized suspension bridge using marshmallows and drinking straws.

Brownies is open to those aged between seven to 10 and represents a jolly good start for any girl, whatever her ambitions.

Those not into knots can gain a badge in dancing or designing clothes or hostess skills; it's a little known but rather delicious fact that the acronym of the World Association of Girl Guides and Girl Scouts is WAGGGS.

'Fat tax' isn't fair but it might be right

How dispiriting to discover that people in developing countries have seen their waistlines expand along with their economies, leading to a quadrupling of obesity rates.

According to the Overseas Development Institute, one in three people worldwide are now overweight due to changing diets.

The shift from cereals and grains to the fats, sugar and oils present in processed foods, which are regarded as aspirational, has led to one billion people in the world's poorest nations being classified as overweight or obese.

Grotesquely, obesity and under-nourishment are often present in the same households, as more calories does not automatically equate with more vitamins. The result is a health time-bomb.

So what to do? Researchers are calling on overseas governments to introduce a "fat tax" to push up the price of **fizzy drinks,** confectionery, fast food and other products containing high levels of sugar, fat and salt.

Here in Britain, where 64 per cent of us are overweight and cost the NHS £5 billion annually, there is a marked reluctance to accept any such meddling in our democratic right to eat microwave chips.

Lobbying for an imposition of a punitive tax on emerging nations might smack of the worst do-as-I-say-not-as-I-do paternalism.

It may be hard to swallow but sometimes, as with smoking bans, unpalatable decisions have to be made in order to save people from themselves.

LOAD-DATE: January 4, 2014

LANGUAGE: ENGLISH

PUBLICATION-TYPE: Web Publication

JOURNAL-CODE: WEBDTNS

Copyright 2014 Telegraph Media Group Limited
All Rights Reserved

The Express

January 2, 2014 Thursday
Edition 1;
National Edition

'ditch fizzy drinks tO slash sugar intake'

BYLINE: Sarah O'Grady

SECTION: NEWS; Pg. 7

LENGTH: 221 words

FAMILIES are being urged to "swap while they shop" to cut down on saturated fat and sugar in their diets.

Giving up fizzy drinks for healthier alternatives could cut the average family's sugar intake by 750g a month, according to a Government campaign.

And changing whole milk for semi-skimmed could reduce their fat intake by a third of a pint.

The campaign, run by Public Health England, also encourages people to buy reduced fat cheese, swap butter for low-fat alternatives and to steer clear of sugary cereals.

Professor Kevin Fenton, director of health and wellbeing at PHE, said. "Swapping like-for-like food in our diet could help cut out surprising levels of saturated fat, sugar and ultimately calories.

"We all eat too much saturated fat and sugar. This increases our risk of obesity, cardiovascular disease, diabetes and some cancers."

Professor Fenton said hundreds of thousands of pounds worth of money-off vouchers will be given to those who sign up to Change4Life's Smart Swap campaign.

Public Health Minister Jane Ellison added: "With over 60 per cent of adults and a third of 10 and 11-year-olds overweight or obese, it is important that we keep helping people make better choices about their diet."

The British Soft Drinks Association said soft drinks provide just two per cent of calories in the average adult diet.

LOAD-DATE: January 2, 2014

LANGUAGE: ENGLISH

PUBLICATION-TYPE: Newspaper

JOURNAL-CODE: DXP

Copyright 2014 Express Newspapers

The Guardian - Final Edition

January 2, 2014 Thursday

Health blitz urges public to junk the fat and fizz: Healthier options in diet mean 'easy calorie cut': Drinks body resists Public Health England drive

BYLINE: Josh Halliday

SECTION: GUARDIAN HOME PAGES; Pg. 6

LENGTH: 399 words

Families are being urged to ditch **sugary drinks** and cut down on saturated fat in the latest advertising blitz by England's public health watchdog.

Public Health England said a family of four could reduce their sugar intake by three-quarters of a 1kg bag of sugar in just one month by swapping **fizzy drinks** for healthier alternatives.

Changing whole milk for semi-skimmed milk could mean the average family cutting down their fat intake by a third of a pint over four weeks.

The advertising campaign, Smart Swaps, is seeking to capitalise on the millions of Britons who begin the new year with health-conscious resolutions.

"Swapping like-for-like food in our diet could help cut out surprising levels of saturated fat, sugar and ultimately calories without having to give up the kinds of food we like," said Professor Kevin Fenton, director of health and wellbeing at Public Health England.

He added: "We all eat too much saturated fat and sugar. Together this increases our risk of obesity, cardiovascular disease, diabetes and some cancers."

Families will be offered vouchers to encourage them to avoid sugary cereals and swap butter and certain cheeses for reduced-fat alternatives.

However, the move brought a backlash from the soft drinks industry. The British Soft Drinks Association (BSDA) claimed its products were being shown in a misleading and "deliberately negative" way.

Gavin Partington, director general of the BSDA, said: "It is frustrating for an industry which has been working with the Department of Health to promote healthier behaviours, reformulate products so they are lower in calories, make available smaller pack sizes and focus more of its marketing investment on low- and no-calorie options." He took issue with the depiction in the adverts of a two-litre bottle of pop, claimed to contain the equivalent of 52 **sugar** cubes. "That bottle is not intended to be consumed by an individual, certainly not by one child. Such an extreme depiction . . . undermines the key message of the campaign, namely that it's very easy to make a smart swap to a no-calorie, diet **soft drink**."

The Children's Food Campaign welcomed the initiative but said it would be undermined unless supermarkets made healthier foods more affordable.

Captions:

Smart Swaps says replacing **fizzy drinks** with heathier ones would cut families' sugar intake by 75% of a 1kg sugar bag in a month

Photograph: Change4Life

LOAD-DATE: January 2, 2014

LANGUAGE: ENGLISH

PUBLICATION-TYPE: Newspaper

Copyright 2014 Guardian Newspapers Limited
All Rights Reserved

MailOnline

January 2, 2014 Thursday 10:46 AM GMT

Ditch fizzy drinks and the average family will cut out almost a bag of sugar a month say experts as Government launches public health campaign

BYLINE: HUGO GYE

SECTION: NEWS

LENGTH: 599 words

- Change4Life launches 'Smart Swaps' initiative for the New Year
- Families could cut down on calories and fat intake by switching to sugar-free drinks, plain cereal and semi-skimmed milk

Families have been urged to boost their health in the New Year by abandoning fatty and sugary foods in favour of slimline alternatives.

A new Government campaign claims that if the average family replaces **fizzy drinks** with sugar-free alternatives, it will cut sugar consumption by three quarters of a bag in just a month.

And drinking semi-skimmed instead of whole milk can reduce fat intake by the equivalent of a third of a pint.

The Change4Life 'Smart Swaps' campaign, launched to coincide with the start of 2014, also encourages people to use low-fat alternatives to cheese and butter.

Breakfast cereal is another target of the initiative, with families urged to cut out sugary treats in the morning and replace them with healthier options.

Public Health England, which is behind the campaign, insists that eating healthily need not be more expensive, and is offering vouchers for low-fat and sugar-free foods to encourage families to change their habits.

'Swapping like-for-like food in our diet could help cut out surprising levels of saturated fat, sugar and ultimately calories without having to give up the kinds of food we like,' said Professor Kevin Fenton, director of health and wellbeing at PHE.

'We all eat too much saturated fat and sugar which can increase our calorie intake. Together this increases our risk of obesity, cardiovascular disease, diabetes and some cancers.

'We're committed to doing as much as possible to support families to make these swaps, which is why I'm pleased to announce that hundreds of thousands of pounds worth of offers will be available to those who sign up to Smart Swaps and in store across hundreds of shops in England.'

Public health minister Jane Ellison added: 'We know how difficult it can be to make big changes to your diet which is why this new Change4Life campaign suggests small changes as a step in the right direction.

'With over 60 per cent of adults and a third of 10- and 11-year-olds overweight or obese, it is really important that we keep helping people make better choices about their diet.'

However, the **fizzy drinks** industry has criticised the new campaign, claiming it gives a 'deliberately negative' and distorted picture of their products.

'We are disappointed that for the second year in succession this campaign heavily targets soft drinks which provide just two per cent of the calories in the average adult diet in the UK,' said Gavin Partington of the British Soft Drinks Association.

SIX 'SMART SWAPS' THAT COULD REVOLUTIONISE YOUR FAMILY'S HEALTH

Sugary drinks to sugar-free drinks, milk or water

The average family will reduce sugar intake by three quarters of a bag per month

Sugary cereal to plain cereal

Switching to porridge or whole-wheat cereal

Whole milk to semi-skimmed milk

Semi-skimmed has 70 fewer calories per pint

Semi-skimmed milk to 1% fat or skimmed milk

Skimmed milk is even healthier than semi-skimmed due to the low fat content

Butter to lower-fat butter or spreads

There is 120g more fat in a tub of regular butter than in healthier spreads

Cheese to reduced-fat cheese

Consumers can save more than 250 calories for every block of cheese

LOAD-DATE: January 2, 2014

LANGUAGE: ENGLISH

PUBLICATION-TYPE: Web Publication

JOURNAL-CODE: WEBDM

Copyright 2014 Associated Newspapers Ltd.
All Rights Reserved

London Evening Standard
standard.co.uk

January 2, 2014 Thursday 11:05 AM GMT

Exclusive SodaStream Offer;
Fresh sparkling drinks on tap this Christmas

SECTION: OFFERS

LENGTH: 205 words

THIS PROMOTION HAS NOW CLOSED.

Whether you're looking for the perfect gift or want to show off your cocktail making skills this festive season, SodaStream is the ultimate must-have kitchen appliance for all the family. SodaStream allows you to turn tap water into sparkling water in an instant so you can create homemade **fizzy drinks,** mixers and sparkling water.

SodaStream has teamed up with the Evening Standard to allow readers to purchase their own SodaStream Genesis drinksmaker starter kit for just £39.99 plus £6.99 p&p. Choose from a range of five colours to co-ordinate with your kitchen. Offer available until 31 December 2013 while stocks last.

For Christmas delivery please order by Monday 16 December 2013

Terms and Conditions: Promotion closes at 23:59 on 31 December 2013. P&p £6.99. Offer is redeemable only through www.sodastream.co.uk/standard. No other offers or coupon codes can be used in conjunction with this offer. Delivery will take up to 5 workings days. Subject to availability. Usual promotion rules apply, see www.standard.co.uk/rules. For further information, please write to Customer Care, Evening Standard Limited, Northcliffe House, 2 Derry Street, London, W8 5TT or call 0800 839 173.Promoter: SodaStream

LOAD-DATE: January 2, 2014

LANGUAGE: ENGLISH

PUBLICATION-TYPE: Newspaper; Web Publication

JOURNAL-CODE: WEBES

Copyright 2014 Evening Standard Limited
All Rights Reserved

London Evening Standard

standard.co.uk

January 2, 2014 Thursday 12:30 AM GMT

Campaign urges healthy alternatives

SECTION: PA NEWS FEEDS

LENGTH: 402 words

Families are being urged to "swap while they shop" in a bid to cut down on saturated fat and sugar in their diets.

Switching **fizzy drinks** for sugar-free alternatives could mean the average family would reduce their sugar intake by three quarters of a bag of sugar over just one month, according to the latest Change4Life campaign.

And changing whole milk for semi-skimmed could see the average family cut down their fat intake by a third of a pint over four weeks.

The campaign, run by Public Health England (PHE), also encourages people to swap cheese to reduced fat cheese, butter to a low-fat alternative and to steer clear of sugary cereals and opting for healthier breakfast options.

"Swapping like-for-like food in our diet could help cut out surprising levels of saturated fat, sugar and ultimately calories without having to give up the kinds of food we like," said Professor Kevin Fenton, director of health and wellbeing at PHE.

"We all eat too much saturated fat and sugar which can increase our calorie intake. Together this increases our risk of obesity, cardiovascular disease, diabetes and some cancers.

"We're committed to doing as much as possible to support families to make these swaps, which is why I'm pleased to announce that hundreds of thousands of pounds worth of offers will be available to those who sign up to Smart Swaps and in store across hundreds of shops in England."

Public Health Minister Jane Ellison added: "We know how difficult it can be to make big changes to your diet which is why this new Change4Life campaign suggests small changes as a step in the right direction.

"With over 60% of adults and a third of 10- and 11-year-olds overweight or obese, it is really important that we keep helping people make better choices about their diet."

As part of the new campaign, a series of adverts will run highlighting the " shocking" amounts of sugar in everyday food and drinks, a PHE spokeswoman said.

But the British Soft Drinks Association criticised the campaign for depicting soft drinks in a " deliberately negative" way.

"Whilst it's reassuring to see a recognition that diet soft drinks can play a role in reducing calorie intake, we are disappointed that for the second year in succession this campaign heavily targets soft drinks which provide just 2% of the calories in the average adult diet in the UK," said the Association's director general Gavin Partington.

LOAD-DATE: January 2, 2014

LANGUAGE: ENGLISH

PUBLICATION-TYPE: Newspaper; Web Publication

JOURNAL-CODE: WEBES

Copyright 2014 Evening Standard Limited
All Rights Reserved

The Sun (England)

January 2, 2014 Thursday
Edition 1;
National Edition

FAT FIGHT FIZZY BAN

SECTION: NEWS; Pg. 12

LENGTH: 96 words

FAMILIES are being urged to ditch **fizzy drinks** that can add almost a bag of sugar to their monthly diet.

Switching to sugar-free, diet alternatives would cut out thousands of "empty" calories in the average household, according to health campaigners. Change4Life, run by Public Health England, urges families to swap whole milk for semi-skimmed, opt for low fat cheeses and ditch butter for diet alternatives. Public Health Minister Jane Ellison said: "We eat too much saturated fat and sugar. This increases our risk of obesity, cardiovascular disease, diabetes and some cancers."

LOAD-DATE: January 2, 2014

LANGUAGE: ENGLISH

PUBLICATION-TYPE: Newspaper

JOURNAL-CODE: SUN

Copyright 2014 NEWS GROUP NEWSPAPERS LTD
All Rights Reserved

MailOnline

February 28, 2014 Friday 1:54 PM GMT

Why are we fatter and sicker than ever? The graphs that explain how sugar, fruit juice and margarine are to blame

BYLINE: ANNA HODGEKISS

SECTION: HEALTH

LENGTH: 1799 words

- The graphs were gathered by Kris Gunnars, anutrition researcher
- He says the obesity epidemic correlates with the time we were told to adopt a low fat diet and eat plenty of carbohydrates
- Graphs show that our sugar consumption has rocketed yet consumption of eggs has fallen - and we have swapped butter for margarine
- Calorie intake has also gone up by around 400 calories a day
- Mr Gunnars says our diets filled with processed food are making us ill

We have more access to food than ever - yet we are also fatter and sicker than we have ever been.

How can this be in the 21st Century?

The answer, it is claimed, lies in the series of graphs below, which paint a stark picture of our food and drink consumption over the past few decades.

Compiled by Kris Gunnars, anutrition researcher, medical student and personal trainer, of AuthorityNutrition.com, they appear to show a startling correlation between a change in the Western diet in line with the soaring obesity epidemic.

In a nutshell, Mr Gunnars says that sugar intake has 'skyrocketed in the past 160 years', we have changed the type of fat we eat and are consuming more processed food than ever.

There are more intricate reasons, too, he believes. Eating fewer eggs - proven to keep hunger pangs at bay - and swapping butter for margarine.

On his website, he says: 'There is an immense amount of evidence I've found that runs completely contradictory to what the governments and dietitians around the world are recommending.

'The modern diet is the main reason why people all over the world are fatter and sicker than ever before.

'Everywhere modern processed foods go, chronic diseases like obesity, type 2 diabetes and heart disease soon follow.

'The studies are clear on this... when people abandon their traditional foods in favor of modern processed foods high in sugar, refined flour and vegetable oils, they get sick.

'Of course, there are many things that can contribute to these health problems, but changes in the diet are the most important factor.'

Mr Gunnars adds: 'The first dietary guidelines for Americans were published in the year 1977, almost at the exact same time the obesity epidemic started.' (In the UK, it was 1983.)

'Of course, this doesn't prove anything (correlation does not equal causation), but it makes sense that this could be more than just a mere coincidence.

'The anti-fat message essentially put the blame on saturated fat and cholesterol (harmless), while giving sugar and refined carbs (very unhealthy) a free pass.

'Since the guidelines were published, many massive studies have been conducted on the low-fat diet. It is no better at preventing heart disease, obesity or cancer than the standard Western diet, which is as unhealthy as a diet can get

'For some very strange reason, we are still being advised to follow this type of diet, despite the studies showing it to be completely ineffective.'

Below, Mr Gunnars explains each graph...

SUGAR

'People in Western countries are consuming massive amounts of refined sugars, reaching about 150 lbs (67 kg) per year in some countries. This amounts to over 500 calories of sugar per day.

'The sources vary on the exact figures, but it is very clear that we are consuming way more sugar than our bodies are equipped to handle.

'Controlled human studies show that large amounts of sugar can lead to severe metabolic problems, including insulin resistance, metabolic syndrome, elevated cholesterol and triglycerides - to name a few.

'Added sugar is believed to be one of the main drivers of diseases like obesity, type 2 diabetes, heart disease and even cancer.'

UK-based nutritionist Zoe Harcombe, author of The Obesity Epidemic: What Caused It? How Can We Stop It, agrees.

She told MailOnline: 'The UK chart looks very similar to this chart. Just over 200 years ago we used to take 4-5lb of sugar a year; by the middle of the 19th century, this had increased five-fold to about 25lb a year; we now take about 100lb per year.'

FRUIT JUICE CONSUMPTION

'Of all the **sugar** sources in the diet, **sugar**-sweetened **beverages** are the worst. Fruit juice is actually no better - it contains a similar amount of **sugar** as most **soft drinks.**

'Getting sugar in liquid form is particularly harmful. The studies show that the brain doesn't "register" liquid sugar calories the in the same way as calories from solid foods, which dramatically increases total calorie intake.

'One study found that in children, each daily serving of **sugar**-sweetened **beverages** is linked to a 60 per cent increased risk of obesity.'

Zoe Harcombe adds: 'It's a similar picture in the UK. Figures show that **sugar**-sweetened **beverages** have increased in consumption from 512 ml per person per week in 1975 to 1,142 ml per person per week in 2005.'

CALORIE INTAKE

'Although sources vary on the exact figures, it is clear that calorie intake has increased dramatically in the past few decades.

'There are many complicated reasons for this, including increased processed food and sugar consumption, increased food availability, more aggressive marketing towards children, etc.'

However the picture is different in the UK. Zoe Harcombe says: 'In 1975 we were eating 2,290 calories per person per day - but by 1999, this had fallen to 1,690 calories per person per day.

'Yet there are an increasing number of people who are overweight or obese.'

ADDED FATS

'When health professionals started blaming saturated fat for heart disease, people abandoned traditional fats like butter, lard and coconut oil in favor of processed vegetable oils.

'These oils are very high in Omega-6 fatty acids, which can contribute to inflammation and various problems when consumed in excess.

'These oils are often hydrogenated, which makes them high in trans fats. Many studies have shown that these fats and oils actually increase the risk of heart disease, even if they aren't hydrogenated.

'Therefore, the misguided advice to avoid saturated fat and choose vegetable oils instead may have actually fueled the heart disease epidemic.'

BUTTER AND MARGARINE CONSUMPTION

'Another side effect of the "war" on saturated fat was an increase in margarine consumption.

'Margarine was traditionally made with hydrogenated oils, which are high in trans fats. Many studies show that trans fats increase the risk of heart disease.

'Grass-fed butter actually contains nutrients that are protective against heart disease (like Vitamin K2), therefore the advice to replace heart-healthy butter with trans-fat laden margarine may have done a lot of damage.

SOYBEAN HAS BECOME A MAJOR SOURCE OF CALORIES

'The most commonly consumed vegetable oil in the U.S. is soybean oil. Soybean oil actually provided 7 per cent of calories in the U.S. diet in the year 1999, which is huge.

'However, most people don't have a clue they're eating this much soybean oil. They're actually getting most of it from processed foods, which often have soybean oil added to them because it is cheap.

'The best way to avoid soybean oil (and other nasty ingredients) is to avoid processed foods.'

EGG CONSUMPTION

'Eggs are among the most nutritious foods on the planet. Despite being high in cholesterol, eggs don't raise the bad cholesterol in the blood.

'For some reason, the health authorities have recommended that we cut back on eggs, even though there is no evidence that they contribute to heart disease.

'Since the year 1950, we have decreased our consumption of this highly nutritious food from 375 to 250 eggs per year, a decrease of 33 per cent.

'This has contributed to a deficiency in important nutrients like Choline, which about 90% of Americans aren't getting enough of.'

Zoe Harcombe adds: Eggs are fantastically nutritious - containing virtually every vitamin and mineral needed by humans (except vitamin C - so have egg and grapefruit for breakfast!)'

LINOLEIC ACID

'Most of the Omega-6 fats that people are eating is a fatty acid called linoleic acid.

'Studies show that this fatty acid actually gets incorporated into our cell membranes and body fat stores. These fats are prone to oxidation, which damages molecules (like DNA) in the body and may be increasing our risk of cancer.

'In other words, the increased consumption of processed vegetable oils has lead to actual harmful structural changes in our bodies. That's a scary thought.'

BUT WHAT DO OTHER EXPERTS THINK?

UK-based nutritionist Zoe Harcombe, author of The Obesity Epidemic: What Caused It? How Can We Stop It, agrees.

She told MailOnline: 'We changed our dietary advice in 1977 in the USA and 1983 in the UK.

'The advice changed from a belief that starchy foods were fattening to advice to base our meals on starchy foods. Obesity increased from 2.7 per cent in the UK in 1972 to around 25 per cent by 1999. Coincidence? I think not.

'We have demonised fat generally, and saturated fat specifically, but our ignorance about food has led to bad advice.

'Our governments (US and UK) call biscuits, cakes, pastries, ice cream, savoury snacks, confectionery etc "saturated fats". They are 1) processed food and 2) predominantly carbohydrate.

'Then governments call meat saturated fat - meat has more unsaturated than saturated fat

They call eggs saturated fat - eggs have more unsaturated than saturated fat. Lard has more unsaturated than saturated fat.

'Only dairy products have more saturated than unsaturated fat and they are a terrific source of calcium, vitamin D and bone nutrients.

'We need to go back to eating food - this means eating meat, eggs and dairy products from pasture living animals; fish; vegetables; nuts & seeds; fruits in season and whole grains (not wheat, but brown rice, oats, quinoa etc) in moderation if you are overweight/diabetic.

'Eating real food means eating food in the form that nature provides it. Oranges grow on trees - orange juice doesn't.

'Until we go back to eating what we evolved to eat - we will continue this horrific experiment of evolution where we manage to weigh multiple times more than baby elephants simply because we've lost touch with what food is.

'The most recent Family Food Survey discovered that 80 per cent of the UK food intake comes from processed foods: **sugars**, jams, oven chips, processed meats, plastic cheese, processed vegetables, processed fruit, bread, cakes, buns, pastries, biscuits, cereals, cereal products, **soft drinks,** confectionery, sauces, dressings etc.And we wonder why we're fat and sick!'

LOAD-DATE: February 28, 2014

LANGUAGE: ENGLISH

PUBLICATION-TYPE: Web Publication

JOURNAL-CODE: WEBDM

Copyright 2014 Associated Newspapers Ltd.
All Rights Reserved

MailOnline

February 27, 2014 Thursday 7:26 AM GMT

Cut out junk food ads in schools, government says

BYLINE: ASSOCIATED PRESS

SECTION: AP

LENGTH: 955 words

WASHINGTON (AP) - It's not just about what America's kids are getting in the lunch line.

The Obama administration is moving to phase out junk food advertising on football scoreboards and elsewhere on school grounds - part of a broad effort to combat child obesity and create what Michelle Obama calls "a new norm" for today's schoolchildren and future generations.

"This new approach to eating and activity is not just a fad," Mrs. Obama said Tuesday as she described the proposed rules at the White House.

Scroll down for video

Promotion of **sugary drinks** and junk foods around campuses during the school day would be phased out under the Agriculture Department rules, which are intended to ensure that marketing is brought in line with health standards that already apply to food served by public schools.

That means a scoreboard at a high school football or basketball game eventually wouldn't be allowed to advertise Coca-Cola, for example, though it could advertise Diet Coke or Dasani water, also owned by Coca-Cola Co. Same with the front of a vending machine. Cups, posters and menu boards that promote foods that don't meet federal standards would also be phased out.

Ninety-three percent of such marketing in schools is related to beverages. And many soda companies already have started to transition their sales and advertising in schools from sugary sodas and sports drinks to other products they produce. Companies are spending $149 million a year on marketing to kids in schools, according to the Agriculture Department.

The announcement at the White House was part of a week of events marking the fourth anniversary of the first lady's "Let's Move" program. Mrs. Obama also traveled to Miami Tuesday to announce that the Boys & Girls Clubs of America and the National Recreation and Park Association will serve more fruits and vegetables at after-school programs and ensure kids get 30-60 minutes of physical activity a day. NBC's "Parks and Recreation" star Amy Poehler introduced the first lady.

The proposed school marketing rules come on the heels of federal regulations that now require food in school lunch lines to be more healthful than in the past.

Separate rules, which are to go into effect in September, will cover other food around school as well, including in vending machines and "a la carte" lines in the lunch room. Calorie, fat, sugar and sodium limits now will have to be met on almost every food and beverage sold during the school day, as mandated by a 2010 child nutrition law.

Even though diet sodas would be allowed in high schools under the proposed rules announced Tuesday, the rules don't address the question raised by some as to whether those drinks are actually healthful alternatives to sugary soda.

Some healthful-food rules have come under fire from conservatives who say the government shouldn't dictate what kids eat - and from some students who don't like the new alternatives.

Mrs. Obama defended herself against critics, saying that "I didn't create this issue." She said kids will eventually get used to the changes.

"That's our job as parents, to hold steady through the whining," she said.

Aware of the backlash, the Agriculture Department is allowing schools to make some of their own decisions on what constitutes marketing and is asking for comments on some options. For example, the proposal asks

for comments on initiatives like Pizza Hut's "Book It" program, which coordinates with schools to reward kids with pizza for reading.

Rules for other school fundraisers, like bake sales and marketing for those events, would be left up to schools or states.

Off-campus fundraisers, like an event at a local fast-food outlet that benefits a school, still would be permitted. But posters advertising the fast food may not be allowed in school hallways. An email to parents - with or without the advertising - would have to suffice. The idea is to market to the parents, not the kids.

The rules also make allowances for major infrastructure costs - that scoreboard advertising Coca-Cola, for example, wouldn't have to be immediately torn down. But the school would have to get one with a different message or product the next time it was replaced.

Schools that don't want to comply could leave the National School Lunch Program, which allows schools to collect government reimbursements for free and low-cost lunches for needy students in exchange for following certain standards. Very few schools choose to give up those government dollars, though.

The beverage industry - led by Coca-Cola Co., Dr. Pepper Snapple Group and PepsiCo - is on board with the new rules. American Beverage Association President and CEO Susan Neely said in a statement that aligning signage with the more healthful drinks that will be offered in schools is the "logical next step."

The public will have 60 days to comment on the proposed rules, which also would allow more children access to free lunches and ensure that schools have wellness policies in place.

The 2010 child nutrition law expanded food programs for hungry students. The rules being proposed Tuesday would increase that even further by allowing the highest-poverty schools to serve lunch and breakfast to all students for free, with the cost shared between the federal government and the schools. According to the Agriculture Department and the White House, that initiative would allow 9 million children in 22,000 schools to receive free lunches.

The department already has tested the program in 11 states.

___

Associated Press writer Kelli Kennedy in Miami contributed to this report.

__

Follow Mary Clare Jalonick on Twitter at http://twitter.com/mcjalonick and Darlene Superville at http://www.twitter.com/dsupervilleap

LOAD-DATE: February 27, 2014

LANGUAGE: ENGLISH

PUBLICATION-TYPE: Web Publication

JOURNAL-CODE: WEBDM

Copyright 2014 Associated Newspapers Ltd.
All Rights Reserved

theguardian
Guardian.com.

February 26, 2014 Wednesday

Almost 11,000 admissions to hospital for obesity recorded in 2012-13

BYLINE: Haroon Siddiquetheguardian.com

LENGTH: 424 words

ABSTRACT

Although number of admissions is slightly down on previous 12 months it is still alarmingly high, obesity expert saysReality check: we are we really overweight?

FULL TEXT

There were almost 11,000 admissions to NHS hospitals in England for obesity in 2012-13, official figures have revealed.

Although the number is down on the previous 12 months - the first time there has been a year-on-year decrease since the dataset begins in 2002/03 - it is more than eight times the amount 10 years earlier and still alarmingly high, according to an obesity expert.

Dr Aseem Malhotra, who sits on the Academy of Medical Royal Colleges obesity steering group, said: "These figures show that the obesity epidemic is still a major public health crisis. While there may be a minor decrease from last year, they are still alarmingly high. It's concerning that one year after the Academy of Medical Royal Colleges (AoMRC) - which represents almost every doctor in the UK - published a list of 10 recommendations on obesity, not a single one has been adopted. Until this happens, this problem is only going to get worse. "

The AoMRC recommendations include a tax on **sugary soft drinks,** public funding to extend weight management services, action to reduce the proximity of fast food outlets to schools and a unified system of traffic light food labelling for supermarkets and food manufacturers.

The Health and Social Care Information Centre statistics showed that there were admissions to hospital with a primary diagnosis of obesity in 2012/13, compared with 11,736 over the previous 12 months and 1,275 in 2002/03. Despite the overall year-on-year fall, there was an increase in admissions among both the youngest and oldest age groups. The number of admissions among under-16s rose 12% to 556 and the number of admissions among those aged 65 and over rose 6% to 594.

There was also 10% increase last year from 2011/12 in the total number of admissions with a primary or secondary diagnosis (when it is deemed relevant to care but not the first diagnosis listed) of obesity to 292,404.

Alison Tedstone, director of nutrition and diet at Public Health England, said: "PHE are committed to tackling obesity as people who are overweight or obese have an increased risk of developing type 2 diabetes, heart disease and certain cancers. There is no silver bullet to reducing obesity; it is a complex issue that requires action at individual, family, local and national levels. We can all play our part in this by eating a healthy, balanced diet and being more active."

LOAD-DATE: February 26, 2014

LANGUAGE: ENGLISH

PUBLICATION-TYPE: Newspaper

Copyright 2014 Guardian News and Media Limited or its affiliated companies
All Rights Reserved

Registered in England and Wales. No. 908396
Registered office: PO Box 68164, Kings Place, 90 York Way, London N1P 2AP

MailOnline

February 26, 2014 Wednesday 11:33 AM GMT

Firm behind Haagen-Dazs and Yoplait falls to bottom of Oxfam ranking of food companies because of poor green record

BYLINE: TARA BRADY

SECTION: NEWS

LENGTH: 1109 words

- General Mills also sells the Old El Paso and Betty Crocker brands
- Only top 10 food and drink companies in the world which has not improved
- Oxfam's Behind the Brands campaign measures sourcing policies
- The top three performers were Nestle, Unilever and Coca-Cola

The company that sells Haagen-Dazs, Green Giant and Yoplait in the UK has fallen to the bottom of an Oxfam ranking after failing to improve its social and environmental policies, the charity said.

General Mills, which also sells the Old El Paso and Betty Crocker brands, was the only one of the top 10 food and drink companies in the world that had not improved its sourcing policies, Oxfam said.

The charity's 'Behind the Brands' campaign measures the sourcing policies of the 10 biggest food and drink companies, looking at transparency, women's rights and rights of workers, farmers, land, water and climate.

Oxfam said that while progress had been made by some of the companies on land, women's rights and carbon emissions and some leading companies had made 'major strides', overall the 'big 10' had moved too slowly as a group.

It said that before the campaign launched a year ago, none of the 10 companies had policies to ensure their ingredients were not grown on land that had been taken from communities.

Now, six had policies to help ensure their suppliers were not involved in land grabs, seven had committed to improving conditions for women affected by their operations and eight had improved their policies on climate, mainly through better disclosing their emissions and risks related to climate change.

SCROLL DOWN FOR VIDEO

OXFAM FOOD COMPANY RANKINGS

The rankings as listed by Oxfam for this year, showing a total score adding up points relating to land, women, farmers, workers, climate, transparency and water, are:

1. Nestle 45/70

2. Unilever 44/70

3. Coca-Cola 38/70

4. Mondelez 23/70

4. PepsiCo 23/70

6. Danone 22/70

6. Mars 22/70

8. Kellogg's 20/70

9. Associated British Foods 19/70

10. General Mills 15/70

Oxfam's campaigns and policy director, Ben Phillips, said: 'Consumer power and investors are pushing most of the top 10 food and drink companies in the right direction and we are beginning to see what this can mean for poor and vulnerable people.

'These companies, with so much power and influence, can do so much more and while some are showing leadership, others are clinging to a business model that is outdated and fails to respect human rights.

'Pressure from consumers is what pushed companies to be more responsible and so much more is possible if people continue to add their voice.'

The top three performers - Nestle, Unilever and Coca-Cola - all improved their scores by the biggest margins, Oxfam said.

Greenpeace report slams Head & Shoulders for using suppliers which are 'helping to wipe the orangutan'

A Greenpeace report has blamed the company which makes Head & Shoulders shampoo for helping to wipe out orangutans.

Proctor and Gamble, which makes the anti-dandruff shampoo, has been accused of failing to follow other companies by not using suppliers which are clearing parts of the Indonesian rainforest for palm oil.

A year long investigation by the charity claims to have traced palm oil in Head & Shoulders shampoo and its shaving cream to suppliers accused of destroying the orangutans' habitat.

The suppliers - BW Plantation, KLK and Musim Mas - were all slammed in the report.

Palm oil is an ingredient of so many day to day products.

In too many cases rainforest is illegally destroyed to make way for palm oil plantations.

Of approximately 11 million hectares of oil palm plantations globally, about six million hectares are found in Indonesia.

But in many places, these plantations are taking over rainforests, the natural habitat of endangered species such as orangutans.

The Sumatran tiger is also under threat according to Greenpeace.

Proctor and Gamble, whose Head & Shoulders advert stars England goalkeeper Joe Hart, said it is continuing its pledge that all of its palm oil will be 'sustainably sourced' by next year.

A spokesman said: 'P&G is committed to the sustainable sourcing of palm oil. We have made a public commitment that, by 2015, 100 per cent of all palm oil purchases we make will be sustainably sourced and we are working with our suppliers to ensure we deliver on this commitment.

'We are strongly opposed to irresponsible deforestation practices and take any allegation of impropriety by our suppliers very seriously.

'We will investigate any allegations made to ensure that our policy on the responsible and sustainable sourcing of palm oil have been, and will continue to be, adhered to.

'P&G is a member of the Roundtable on Sustainable Palm Oil (RSPO), we support the RSPO criteria established for sustainable palm oil, and we are part of the working group on sustainable sourcing of palm oil derivatives.'

SCARLETT JOHANSSON ALSO FELL FOUL OF OXFAM'S STRICT POLICIES - SHE WAS DROPPED AS AN AMBASSADOR AFTER ADVERTISING SODASTREAM

She quit her role as Oxfam ambassador in a row over her controversial Super Bowl advert for SodaStream - and chose to keep her links with the Israeli **fizzy drink** firm.

Now speaking for the first time since she severed her ties with the humanitarian group, Scarlett Johansson insists she never saw herself as a role model in the first place.

In an interview with Dazed magazine, Johansson did not directly address the row with Oxfam, but said: 'I don't see myself as being a role model; I never wanted to step into those shoes.

'How could I wake up every day and be a normal person if I was completely aware that my image was being manipulated on a global platform. How could I sleep?

'You have to have peace of mind. You've got to be able to protect those things. How else could you exist? You'd go crazy, anybody would go crazy.'

The row erupted after Johansson featured in an advert for the **fizzy drinks** firm which featured in the SuperBowl earlier this month.

The 29-year-old actress said she had a 'fundamental difference of opinion' with the charity after it said it opposed all trade from Israeli settlements because they say it is illegal and denies Palestinian rights.

SodaStream has a large factory in an Israeli settlement in the West Bank - a territory captured by Israel in 1967 and claimed by the Palestinians.

Johansson instead chose to keep her role as brand ambassador for SodaStream, saying she supports trade and 'social interaction between a democratic Israel and Palestine'.

LOAD-DATE: February 26, 2014

LANGUAGE: ENGLISH

PUBLICATION-TYPE: Web Publication

JOURNAL-CODE: WEBDM

Copyright 2014 Associated Newspapers Ltd.
All Rights Reserved

The Telegraph
telegraph.co.uk

February 26, 2014 Wednesday 5:50 PM GMT

Obesity among young children in America falls significantly; Experts say America has "turned a corner" in the relentless rise in obesity and staying away from sugary drinks for kids is key

BYLINE: By Joanna Walters New York

SECTION: NEWS

LENGTH: 384 words

Obesity among young children has declined significantly in America over the last decade and appears to have "turned a corner" after rising relentlessly since the 1980s, according to a new national report.

The proportion of abnormally fat two-to-five year olds has fallen by 43 percent since 2004 and US federal authorities are cautiously optimistic this is part of a healthy trend that is gaining momentum.

Experts say the message is getting through to parents not to give **sugary drinks,** in particular, to young children, as well as to rely more on fresh foods and move away from junk food and fattening snacks. The research showed that public-health policies to encourage more breast-feeding for longer and more exercise for children were also getting through.

"This is the first time we've seen any indication of any significant decrease in any group," said Cynthia Ogden, a researcher at the US federal health monitoring body, the Centres for Disease Control and Prevention, which produced the report.

It found that eight percent of children in the US aged between two and five were obese in 2012, down from 14 percent in 2004.

Problems remain for older Americans. A total of 35 percent of adults are now sufficiently overweight to be regarded as obese, compared with 32 percent 10 years ago, and 16.9 percent of all children aged 2 to 19 are obese - a reduction of just 0.2 percent since 2004, the report found.

But the results for young children are regarded as promising because of previous research showing that obesity in the under-fives makes it five times more likely that they will grow up to be fat adults.

There has been more focus in the last decade at the national and, in some cases, local level on providing more exercise and healthy foods to children attending play groups as well as public health campaigns - some spearheaded by Michelle Obama, the wife of President Barack Obama.

"The message is getting through to many parents. And while government policies to promote healthy eating are controversial, many food companies have a commercial policy to persuade people - through marketing and pricing - to eat as much unhealthy food as possible, it's just not publicly stated," said Margo Wootan, director of nutrition policy at the Centre for Science in the Public Interest, a lobbying group.

LOAD-DATE: February 27, 2014

LANGUAGE: ENGLISH

PUBLICATION-TYPE: Newspaper; Web Publication

JOURNAL-CODE: WEBDTNS

Copyright 2014 Telegraph Media Group Limited
All Rights Reserved

theguardian

Guardian.com.

February 25, 2014 Tuesday

Brands continue to target fast food marketing at kids

BYLINE: Liza Ramraykatheguardian.com

LENGTH: 991 words

ABSTRACT

Companies are ploughing more and more money into marketing unhealthy foods to children. What will turn the tide?

FULL TEXT

Each year, the world's food and beverage companies spend billions on marketing and advertising their products to children and teenagers. The overwhelming majority of these products are high in calories, added sugar, saturated fat and sodium - fast food, **fizzy drinks,** sweets and chocolate to name just a few. Ask your child to recall a food advert and chances are that it won't be one for apples or broccoli.

US fast food restaurants alone spent $4.6bn on advertising to children and teens in 2012. According to Fast Food Facts 2013, children under six saw almost three adverts for fast foods every day, while 12-17-year-olds saw almost five adverts a day.

Between 2010 and 2013, the number of kids' meals at fast-food restaurants increased by 54%. But the percentage of items that qualified as healthy - less than 1% - remained stagnant.

Report lead author Jennifer Harris, director of marketing at the Yale Rudd Center for Food Policy & Obesity, is concerned that many companies are shifting their focus to increase reach into markets not currently covered by the current system of voluntary self regulation.

"A lot of companies have switched their marketing target to the 12-14 [age] group. This is a really vulnerable time for kids; they are seeing more media and making more decisions on their own," Harris says.

Around one in three children in the US - and in the UK - is overweight or obese. A study published this month by Roberto De Vogli of UC Davis in California found that fast food purchases were independent predictors of increases in the average body mass index (BMI) in the US and 24 other wealthy nations between 1999 to 2008.

So what is business doing?

Encouraging food and drink companies to rethink their messages is the aim of the first White House convening on food marketing to children. Launching the meeting last September, US First Lady Michelle Obama called on the private sector to "move faster" to market responsibly to children.

In January 2014, Subway became the first quick service chain to join Partnership for a Healthy America, a campaign endorsed by Obama to bring together business, non-profits and health advisers to tackle childhood obesity. A three-year commitment worth $41m will see it market healthier options and promote fruit and veg consumption.

Disney has pledged that by 2015, all food and beverage products advertised, sponsored, or promoted on Disney-owned media channels, online destinations and theme parks will be required to meet nutritional guidelines that align with federal standards to promote fruit and vegetables and limit calories, sugar, sodium, and saturated fat.

Earlier this year, Lidl became the first supermarket group in the UK to remove unhealthy products from all tills across its stores, with no seasonal exceptions for Christmas or Easter confectionery. Lidl is replacing these products with healthier options including fresh and dried fruits, nuts and bottled water.

Should regulation be playing a bigger role?

In the UK, regulation exists to prevent adverts for unhealthy foods from being broadcast during or around programmes specifically made for children. But the Children's Food Campaign (CFC) argues that the popularity of family entertainment shows like The X-Factor means later bedtimes for many children - and advertisers are taking advantage by promoting unhealthy foods at these times.

In a joint campaign with the British Heart Foundation, the CFC will next month call for a 9pm watershed for fast food and drinks ads and clearer definition of 'healthy' and 'unhealthy' foods, to close existing loopholes.

But the ISBA, which represents British advertisers, argues that the causal link between the ads that viewers watch, and the food choices they make, is "nominal", and ad prohibitions are currently viewed at the "silver bullet" for tackling a complex public health issue.

Ian Twinn, ISBA's director of public affairs says: "Encouraging people to change their lifestyle rather than slapping bans on ads is what will make a difference.

"There are plenty of good examples of big brands changing their messages to ensure they stay relevant to their consumers but support the overall message for a healthier lifestyle. Coca-Cola, for instance, only advertises its low calorie or sugar-free products."

The UC Davis study suggests that if governments take action to control food industries, they can help prevent obesity and its serious health consequences, including cardiovascular disease and diabetes. This echoes calls in the UK and US for more robust, government-led regulation of the industry, rather than voluntary self-regulation.

In the UK, the CFC hopes the government's food promotion pledge - expected late spring as part of its public health responsibility deal - will target point-of-sale, product packaging, digital marketing and in-school promotion.

Beyond the supermarket

The CFC's Junk Free Checkouts campaign, launched last September, challenged supermarkets to act on consumer concerns about "pester power" and remove unhealthy snacks from checkouts and queuing areas. Shoppers were urged to hand in pass or fail cards to store managers, and name and shame supermarkets via a dedicated website.

Malcolm Clark, CFC co-ordinator, says: "The government's responsibility pledge covers supermarkets, but WH Smith and Boots have chocolate at the checkout, so the question will be whether they engage with the other companies."

Dr Emma Boyland, a psychologist at the University of Liverpool who specialises in the effects of food marketing on children's diets, says the next challenge is to tackle promotion to children via advergaming and social media.

"The cross-border nature of this [area] means that government can only tackle .co.uk. A little progress has been made with TV but advertising has moved to the Internet.. and into another sphere."

LOAD-DATE: February 25, 2014

LANGUAGE: ENGLISH

PUBLICATION-TYPE: Newspaper

Copyright 2014 Guardian News and Media Limited or its affiliated companies
All Rights Reserved
Registered in England and Wales. No. 908396

MailOnline

February 25, 2014 Tuesday 8:34 PM GMT

'We love cookies but they're not sufficient, we need to eat veggies to make our bodies efficient': Michelle performs cringeworthy rap as she announces new rules to ban junk food marketing in schools

BYLINE: ASSOCIATED PRESS

SECTION: NEWS

LENGTH: 1055 words

- First Lady performed a rap song written by school kids designed to promote healthy eating
- Under the proposed rules, companies would be limited in what products they could advertise on signs, vending machines, cups and menu boards on school grounds
- Food industry spent nearly $150 million in 2009 on marketing in schools, 93 percent was promoting drinks
- Schools would require drink companies to promote their diet sodas or water on signs and scoreboards, rather than full-calorie options like Coca-Cola and Pepsi
- Michelle Obama's Let's Move program is now in its fourth year

First Lady Michelle Obama attempted to connect with schoolchildren across the country by rapping about healthy eating habits as she announced new rules that would limit the marketing of unhealthy foods in schools.

The rules would phase out the advertising of **sugary drinks** and junk foods around campuses during the school day and ensure that other promotions in schools were in line with health standards that already apply to school foods.

That means a scoreboard at a high school football or basketball game eventually wouldn't be allowed to advertise Coca-Cola, for example, but it could advertise Diet Coke or Dasani water, which is also owned by Coca-Cola Co.

Scroll down for video...

Same with the front of a vending machine. Cups, posters and menu boards which promote foods that don't meet the standards would also be phased out.

Ninety percent of such marketing in schools is related to beverages, and many soda companies already have started to transition their sales and advertising in schools from sugary sodas and sports drinks to their own healthier products.

Mrs Obama performed some lyrics to a rap song that were written by kids at a school in Virginia.

The jam shows all the benefits of healthy eating, including a healthy brain.

MICHELLE OBAMA'S 'WRAP'

'If I'm going to help my brain come to fruition,

I'm going to feed it quality nutrition.

We love the cookies but they're not sufficient,

We need to eat veggies to make our bodies efficient.

Roll my chicken in a wrap, don't jam it in a nugget.

Get hyped for healthy snacks, fresh food, we love it!'

Source: Students of Marshall High School, Virginia

The proposed rules are part of first lady Michelle Obama's Let's Move initiative to combat child obesity, which is celebrating its fourth anniversary this week.

Mrs. Obama and Agriculture Secretary Tom Vilsack announced the new rules at a White House event.

'The idea here is simple - our classrooms should be healthy places where kids aren't bombarded with ads for junk food,' the first lady said in a statement released before the announcement.

"Because when parents are working hard to teach their kids healthy habits at home, their work shouldn't be undone by unhealthy messages at school.'

The rules also would allow more children access to free lunches and ensure that schools have wellness policies in place.

The proposed rules come on the heels of USDA regulations that are now requiring foods in the school lunch line to be healthier.

Rules set to go into effect next school year will make other foods around school healthier as well, including in vending machines and separate 'a la carte' lines in the lunch room. Calorie, fat, sugar and sodium limits will have to be met on almost every food and beverage sold during the school day at 100,000 schools. Concessions sold at afterschool sports games would be exempt.

The healthier food rules have come under fire from conservatives who think the government shouldn't dictate what kids eat - and from some students who don't like the healthier foods.

Aware of the backlash, the USDA is allowing schools to make some of their own decisions on what constitutes marketing and asking for comments on some options. For example, the proposal asks for comments on initiatives like Pizza Hut's 'Book It' program, which coordinates with schools to reward kids with pizza for reading.

Rules for other school fundraisers, like bake sales and marketing for those events, would be left up to schools or states.

Off-campus fundraisers, like an event at a local fast-food outlet that benefits a school, still would be permitted.

But posters advertising the fast food may not be allowed in school hallways. An email to parents - with or without the advertising - would have to suffice. The idea is to market to the parents, not the kids.

The rule also makes allowances for major infrastructure costs - that scoreboard advertising Coca-Cola, for example, wouldn't have to be immediately torn down. But the school would have to get one with a healthier message the next time it was replaced.

The beverage industry - led by Coca-Cola Co., Dr. Pepper Snapple Group and PepsiCo - is on board with the move. American Beverage Association President and CEO Susan Neely said in a statement that aligning signage with the healthier drinks that will be offered in schools is the "logical next step."

"Mrs. Obama's efforts to continue to strengthen school wellness make sense for the well-being of our schoolchildren," Neely said.

Although Mrs. Obama lobbied Congress to pass the school nutrition bill in 2010, most of her efforts in recent years have been focused on the private sector, building partnerships with food companies and retailers to sell healthier foods.

The child nutrition law also expanded feeding programs for hungry students. The rules being proposed Tuesday would increase that even further by allowing the highest-poverty schools to serve lunch and breakfast to all students for free.

According to the USDA and the White House, that initiative would allow 9 million children in 22,000 schools to receive free lunches.

The USDA has already tested the program, which is designed to increase participation for students and reduce paperwork and applications for schools, in 11 states.

In addition, the Obama administration will announce new guidelines for school wellness policies. Schools have been required to have general wellness policies that set their own general standards for foods, physical activity and other wellness activities since 2004.

But the new rules would require parents and others in the school community to be involved in those decisions.

LOAD-DATE: February 25, 2014

LANGUAGE: ENGLISH

PUBLICATION-TYPE: Web Publication

JOURNAL-CODE: WEBDM

Copyright 2014 Associated Newspapers Ltd.
All Rights Reserved
red office: PO Box 68164, Kings Place, 90 York Way, London N1P 2AP

MailOnline

February 25, 2014 Tuesday 11:48 AM GMT

How much hidden sugar is in YOUR diet? Study reveals that a bowl of tomato soup or natural yogurt has as much sugar as a bowl of Frosties

BYLINE: ANNA HODGEKISS

SECTION: HEALTH

LENGTH: 629 words

- Leading professor has compiled a list of everyday 'hidden sugar' offenders
- These include flavoured water, yogurts, canned soup, ready meals and bread
- Graham MacGregor says food industry is adding more sugar to food
- Says many people fall for the 'low fat' trick - not realising food is packed with sugar instead to give it flavour

We are constantly told how much sugar there is in **fizzy drinks** and cakes. But what about the hidden sugar in so-called healthy foods?

A leading professor has spoken out about the dangers of 'hidden sugar' in food.

He also cautions against opting for low fat foods that are often full of sugar instead to give them flavour.

Graham MacGregor, Professor of Cardiovascular Medicine at the Wolfson Institute of Preventive Medicine at Barts and The London Hospital, says the food industry is adding more and more sugar to food, which consumers are largely unaware of, as it is mostly hidden.

Writing for The Conversation, he said: 'Added sugar in our diet is a very recent phenomenon and only occurred when sugar, obtained from sugar cane, beet and corn, became very cheap to produce.

'It's a completely unnecessary part of our calorie intake: it has no nutritional value, gives no feeling of fullness and is acknowledged to be a major factor in causing obesity and diabetes both in the UK and worldwide.'

Professor MacGregor has compiled a table of some of the most common culprits.

He says: 'While it may not be surprising that a can of Coca Cola has a staggering nine teaspoons of sugar (35g), similar amounts can be found in the most unlikely of foods.'

These include flavoured water, yogurts, canned soup, ready meals and even bread.

As Professor MacGregor expains: 'You might opt for 0 per cent fat in your yoghurt, but what if it also comes with five teaspoons of sugar? A bowl of Frosties with semi-skimmed milk only has four.'

At the other end of his scale, a Starbucks caramel Frappuccino with whipped cream - made with skimmed milk - contains 11 teaspoons of sugar. A can of regular Coke has nine, a Mars bar eight.

When it comes to food, a Sharwood's Sweet and Sour Chicken with Rice contains six teaspoons - two more than a 25g bag of Butterkist toffee popcorn.

'It's clear this sugar plays a part in soaring levels of obesity and diabetes. To this end, leading health experts from across the globe have united to tackle - and to unmask hidden sugar so consumers can make informed decisions about what they eat and drink.'

He adds that sugar in our diets should now be gradually reduced, just as salt has been over the past few years.

'Salt content in food products in the supermarkets have now been reduced by 20-40 per cent and as a result, salt intake has fallen in the UK by 15 per cent (between 2001-2011), the lowest known figure of any developed country.'

This this will have reduced stroke and heart attack deaths by a minimum of 9,000 per year, with a saving in health care costs of at least £1.5bn a year, according to the National Institute for Health and Care Excellence (NICE).

Professor MacGregor says that a similar move could be taken with sugar.

'Like salt, most of the **sugar** we consume is hidden in processed food and **soft drinks**.

'There are also specific taste receptors for sugar, which if sugar intake is gradually reduced, become more sensitive. So over time we don't notice that sugar levels have gone down.

'If we can persuade the Department of Health that this programme is very likely to help considerably with the obesity epidemic - and in particular to reduce childhood obesity - while also reducing the incidence of dental disease, and (very likely) the number of people developing Type 2 diabetes, it should have a good chance of success. '

Revealed: How much sugar other foods contain

LOAD-DATE: February 25, 2014

LANGUAGE: ENGLISH

PUBLICATION-TYPE: Web Publication

JOURNAL-CODE: WEBDM

Copyright 2014 Associated Newspapers Ltd.
All Rights Reserved

THE SUNDAY TIMES
The Sunday Times (London)

February 23, 2014 Sunday
Edition 1;
Ireland

'Ban sugary snacks from school, now'; Time's up: bad food should not be in vending machines for kids. By Siobhán Maguire

BYLINE: Siobhán Maguire

SECTION: NEWS; Pg. 6

LENGTH: 651 words

CONFECTIONERY, crisps and soft drinks should not be sold in secondary schools, according to 70% of the Irish public. Seven out of 10 people oppose the sale of "junk food" from vending machines in schools, with 69% against the sale of soft drinks.

The survey, compiled for The Sunday Times by Behaviour &Attitudes (B&A), found that most people are unhappy with the sale of sugary snacks in schools even when the profits generated are used to pay for necessary services.

Some 61% were against the sale of soft drinks in those circumstances. Just over half, 58%, did not want sweets and crisps sold even if the proceeds raised valuable revenue for schools.

Respondents were in favour of the sale of healthier options in secondary schools, with 69% favouring the sale of flavoured bottle water and 63% approving of the sale of "breakfast" or cereal bars in vending machines.

The Department of Education, which plans to instruct schools on how best to stock vending machines, said the survey shows the vast majority are in favour of healthier options. "The findings seem to be in keeping with what we are hoping to do through the advice to schools on how to encourage healthy eating through their vending machines or tuck shops," it said.

"We believe that if schools implement a suggested 60/40 split between healthy and other food options, and if we give young people the right information so they can make informed choices, then a culture of healthy eating will be created. Young people will be able to carry that knowledge into their homes and lives outside, and continue to make informed choices on food.

"There is nothing wrong with a bit of chocolate and we shouldn't be demonising certain foodstuffs. Rather we need to have open discussions and information on the right amount of any type of food, and encourage healthy lifestyles."

Two weeks ago, the Sunday Times launched a Junk the Junk campaign which aims to persuade the managers of Ireland's post-primary schools to cut back on the proportion of sugary foods and drinks stocked in their vending machines and replace them with healthier options.

Ian McShane, the managing director of B&A, said one of the more interesting aspects of the results was how definite people were in their attitude to junk food in school, with only one percent of those surveyed unsure of how to answer.

Last week, the European Commission's in-house science service, the Joint Research Centre, confirmed it is mapping school-food policies across all EU member states, plus Norway and Switzerland. The research, due to be completed this summer, will show how Ireland compares with its European peers on school food policy. Early indications are it is behind several countries, which have restrictions on what kind of food and beverages are allowed in vending machines. In 2008, Lithuania banned the sale of crisps, lollipops and **fizzy drinks** in schools. Cyprus and Hungary have imposed similar limitations.

France, Denmark and Slovenia ban vending machines on school premises. About twothirds of the policies collated in 30 countries define if, and to what extent, sweet treats are allowed at lunch, while 79% of policies deal with other meal times. Two-thirds of European policies specify limitations on the availability of soft drinks during the school day.

Support for the Junk the Junk campaign continued to build last week with the chefs Darina Allen and Derry Clarke backing the initiative. Clarke believes there is even a need to educate children on what junk food actually is, because snacks purporting to be "healthy" sometimes are not.

"I saw a British chef tweet last week that the only junk food you should eat is the junk food you cook for yourself, which I thought was clever," he said. "There's a lot of junk food out there that we don't know or think of as junk food, such as cereals or cereal bars.

"I'd love to see more people cooking. It should be taught in all schools."

LOAD-DATE: February 23, 2014

LANGUAGE: ENGLISH

GRAPHIC: Chefs Allen and Clarke, inset, back The Sunday Times's anti-junk food campaign

PUBLICATION-TYPE: Newspaper

JOURNAL-CODE: STS

Copyright 2014 Times Newspapers Limited

All Rights Reserved

Financial Times (London, England)

February 21, 2014 Friday
London Edition 3

Peltz to escalate case to split Pepsi; BEVERAGES

BYLINE: Shannon Bond in New York

SECTION: COMPANIES; Pg. 21

LENGTH: 581 words

Nelson Peltz will take his campaign to split PepsiCo directly to fellow investors, less than a week after the US drinks and snacks company maintained that it would keep the businesses under one roof .

Mr Peltz's Trian Partners, which owns a $1.2bn stake in the maker of Pepsi soft drinks and Doritos crisps, said it was "highly disappointed" with Pepsi's decision not to spin off its slow-growing beverage division as Mr Peltz has advocated.

A split would "create two leaner and more entrepreneurial companies", accelerating the food unit's sales and margins and returning more cash to shareholders of a standalone drinks business, Trian argued in a letter and 31-page paper sent to Pepsi's board on Wednesday.

But Pepsi stuck to its argument that snacks and beverages compliment each other.

"We've looked at this from every single angle and we're absolutely convinced that this is the best route for our company," said Brian Cornell, head of the company's Americas food division, at an industry conference in Florida.

He noted the drinks division's link with Taco Bell had led to the snacks segment working with the US fast-food chain to form the popular Doritos Locos Tacos.

Some analysts and investors remain concerned about how Pepsi and its peers are grappling with declining sales of **fizzy drinks** in developed markets, including the US, where consumers are turning away from sugar and artificial sweeteners.

Pepsi's latest results showed North American beverage sales volumes slipped 3 per cent in 2013 after falling 1 per cent in 2012. Global food sales rose 3 per cent last year, while worldwide drink sales rose 1 per cent.

"We believe PepsiCo's business has enormous opportunity to improve," said Ali Dibadj, analyst at Bernstein. "They can probably improve as one company, but they simply haven't yet. Focus often helps, so perhaps a break-up would make capturing the improvements even easier."

Pepsi's shares closed up 1.2 per cent at $78.01 on Thursday.

Mr Peltz has been meeting Pepsi's board over several months to press his case for separation, although in January he dropped a proposal for Pepsi to merge with Mondelez after joining the board of the Oreo maker.

Last week, Pepsi said that after an "exhaustive" review it had concluded that to keep food and drink together was the best way to maximise shareholder value. It announced plans to boost shareholder capital returns by higher dividends and more buybacks.

Trian replied on Wednesday that it "does not agree with the outcome of PepsiCo's strategic review, particularly following another quarter of uninspiring performance and weak 2014 guidance".

Mr Peltz has successfully agitated for the break-up of large food companies, including Cadbury, which spun off drinks maker Dr Pepper Snapple in 2008, and Kraft, whose split in 2012 created Mondelez.

Trian said it "remained open to continued dialogue" with Pepsi's management and board, but that it would now take its case directly to investors, including possibly holding public forums.

"Trian believes the decision is one for shareholders, and it will immediately begin to engage fellow shareholders in a public dialogue with the goal of creating a groundswell of support for a separation of snacks and beverages."

Pepsi said the management and board were "fully aligned" in opposition to a split. "Our focus is on delivering results for our shareholders, not new, costly distractions that will harm shareholder interests."

Additional reporting by Neil Munshi in Boca Raton

See Lex

LOAD-DATE: February 20, 2014

LANGUAGE: ENGLISH

PUBLICATION-TYPE: Newspaper

Copyright 2014 The Financial Times Ltd.
All Rights Reserved
Please do not cut and paste FT articles and redistribute by email or post to the web.

i-Independent Print Ltd

February 21, 2014
First Edition

The 30 Second Briefing

BYLINE: PEPSICO

SECTION: BUSINESS; Pg. 48

LENGTH: 232 words

Are you a Pepsi or a Coca-Cola fan? We'd rather not say but certainly Pepsi has left us fizzing with excitement in the past 24 hours, whetting our appetite for a boardroom showdown.

How so? The **fizzy drinks** owner, PepsiCo, has faced the wrath of an activist investor who wanted to split the company in two, breaking apart the drinks bit from the snacks bit. Nelson Peltz, who owns less than 1 per cent through his Trian Fund Management, wrote a 37-page letter to bosses outlining his proposals. Alas, PepsiCo turned it down.

I bet he wasn't happy. Quite. Mr Peltz said he was "highly disappointed", adding: "It is clear we have vastly different views on the best path forward for PepsiCo. It appears that PepsiCo views structural change as a sign of weakness, an admission of failure and an untenable break with past traditions. Trian views structural change as the best path forward to generate sustainable increases in shareholder value."

Ouch. What did PepsiCo say? Executive vice-president Jim Wilkinson said: "We have engaged constructively with Trian and invested a large amount of management time and significant financial resources analysing Trian's proposals."

What happens next? The war of words will continue, so sit back with a pack of PepsiCo's Walkers Crisps, Doritos, or bowl of Quaker Oats, with a glass of Tropicana or 7UP, and watch it unfold as neither side is likely to back down.

LOAD-DATE: February 20, 2014

LANGUAGE: ENGLISH

PUBLICATION-TYPE: Newspaper

JOURNAL-CODE: III

Copyright 2014 Independent Print Limited
All Rights Reserved

The Daily Telegraph

The Daily Telegraph (London)

February 19, 2014 Wednesday
Edition 2;
National Edition

Coke loses favour among health savvy Americans

BYLINE: Katherine Rushton

SECTION: BUSINESS; Pg. 1

LENGTH: 324 words

COCA-COLA appears to be losing some of its fizz, as Americans swap sugary, high-calorie sparkling drinks for healthier options.

The US drinks maker saw its revenues fall 3.6pc to $11bn in the fourth quarter, after a 4.2pc decline in sales in the North American market, which accounts for nearly half of its revenues worldwide.

The company, which will also cut a further $1bn of costs by 2016, blamed weak economic conditions, but analysts also pointed to an increase in the number of health-conscious consumers trying to cut back on calories and sweeteners.

Fizzy drinks have long been blamed as one of the factors driving a rise in obesity in America, and were thrust further into the spotlight last year, as Michael Bloomberg, the former mayor of New York, waged a controversial battle to ban supersized, **sugary drinks** from sale in the city's restaurants and entertainment venues.

The ruling was ultimately blocked by a judge, who claimed Mr Bloomberg was asserting more power than he was entitled to, but the former mayor's successor, Bill De Blasio, is expected to resume the battle.

Coca-Cola has spent the past decade trying to offset the trend away from **sugary drinks** by introducing more low and zerocalorie versions of its beverages. However, many consumers are now trying to cut back on the artificial sweeteners used to flavour those alternatives as well, amid fears that they could have other adverse effects on their health.

Coca Cola's profits fell 8.4pc to $1.7bn in the fourth quarter, in line with analysts' forecasts, while revenues missed expectations by nearly $300m.

Shares in the drinks maker closed down 3.7pc at $37.47.

Muhtar Kent, chief executive, said: "A rising middle class, greater urbanisation and increasing personal consumption expenditures in markets around the world will continue to drive greater demand for our beverages."

Last week PepsiCo said sales of its carbonated drinks had fallen by "mid-single digits".

LOAD-DATE: February 19, 2014

LANGUAGE: ENGLISH

GRAPHIC: An advertisement from the 1940s shows Coke was consumed in far more modest quantities than in recent years
THE ADVERTISING ARCHIVES

PUBLICATION-TYPE: Newspaper

JOURNAL-CODE: DTL

Copyright 2014 Telegraph Media Group Limited
All Rights Reserved

FINANCIAL TIMES

Financial Times (London, England)

February 19, 2014 Wednesday
London Edition 3

Coca-Cola eyes $1bn cuts as profit falls;
BEVERAGES

BYLINE: Shannon Bond in New York

SECTION: COMPANIES; Pg. 21

LENGTH: 380 words

HIGHLIGHT: Earnings hit by slower sales growth

Company hit 'speed bump' in 2013

Coca-Cola said it would cut another $1bn in costs by 2016 as it reported an 8.4 per cent drop in fourth-quarter profit.

Earnings at the world's largest drinks company were hit in its latest quarter and in 2013 by the spin-off of its bottling operations in the Philippines and Brazil and by slowing sales growth.

Last year "was marked by ongoing global macroeconomic challenges in many markets around the world," said Muhtar Kent, chief executive. Consumer demand has been sluggish in emerging economies, including China, India and Mexico, and in southern Europe.

Coke is also facing weakness in the US, its largest market, where consumers are drinking fewer sweet carbonated beverages amid health concerns.

Global sales volume rose 2 per cent last year compared with 4 per cent in 2012. The company said this was slower than it had expected and below its long-term target.

During the quarter, sales volume rose 1 per cent worldwide. That was worse than the 3 per cent that Wall Street was looking for. The shares fell 3.7 per cent to $37.47 yesterday.

Mr Kent said the company had hit "a speed bump" in 2013 but pledged to "work to restore momentum" this year. Coke said it would save $1bn by 2016 by paring expenses in data, information technology and its supply chain system and revamping its marketing strategy.

The cost cuts will be crucial to growth in profit this year, as the company expects foreign exchange rates to shave 7 per cent from full-year operating income.

In the quarter, net income fell to $1.71bn, or 38 cents a share, from $1.87bn, or 41 cents a share, a year ago. Stripping out some items - including restructuring costs - earnings were 46 cents a share, in line with analysts' estimates.

Revenue dipped 3.6 per cent to $11.04bn from $11.46bn for the quarter and was lower than the $11.31bn Wall Street had forecast.

For the full year, net income fell 4.8 per cent to $8.6bn, or $1.90 a share, from $9bn, or $1.97, in 2012. Full-year earnings per share were $2.08, excluding one-off items. Revenue was down 2.4 per cent to $46.85bn.

In North America, drink sales dropped 1 per cent in the quarter and were flat over the year. Mr Kent said the quarterly decline was mainly due to weak sales of Diet Coke, which has been hit by rising concerns over artificial sweeteners.

LOAD-DATE: February 18, 2014

LANGUAGE: ENGLISH

PUBLICATION-TYPE: Newspaper

Copyright 2014 The Financial Times Ltd.

All Rights Reserved
Please do not cut and paste FT articles and redistribute by email or post to the web.

The Telegraph
telegraph.co.uk

February 18, 2014 Tuesday 6:47 PM GMT

Coca-Cola sales fall as consumers switch to healthier drinks; Coca-Cola's profits fell 8.4pc to $1.7bn in the fourth quarter, while revenues missed expectations by nearly $300m

BYLINE: By Katherine Rushton US Business Editor

SECTION: FINANCE

LENGTH: 366 words

Coca-Cola appears to be losing some of its fizz, as Americans swap sugary, high-calorie, sparkling drinks for healthier options.

The US drinks maker saw its revenues fall 3.6pc to $11bn in the fourth quarter, after a 4.2pc decline in sales in the North American market, which accounts for nearly half of its revenues worldwide.

The company blamed weak economic conditions, but analysts also pointed to an increase in the number of health-conscious consumers trying to cut back on calories and sweeteners.

Fizzy drinks have long been blamed as one of the factors driving a rise in obesity in America, and were thrust further into the spotlight last year when Michael Bloomberg, the former mayor of New York, waged a controversial battle to ban supersized, **sugary drinks** from sale in the city's restaurants and entertainment venues.

The ruling was ultimately blocked by a judge, who claimed Mr Bloomberg was asserting more power than he was entitled to, but the former mayor's successor, Bill De Blasio, is expected to resume the battle.

Coca-Cola has spent the past decade trying to offset the trend away from **sugary drinks** by introducing more low and zero-calorie versions of its beverages. However, many consumers are now trying to cut back on the artificial sweeteners used to flavour those alternatives as well, amid fears that they could have other adverse effects on their health.

Coca-Cola's profits fell 8.4pc to $1.7bn in the fourth quarter, in line with analysts' forecasts, while revenues missed expectations by nearly $300m. Shares in the drinks maker fell 3.4pc to $37.60 in morning trading in New York.

Muhtar Kent, chief executive, said: "While we move forward in what remains an uncertain global economy, the long-term fundamentals driving our business and industry have not changed. A rising middle class, greater urbanisation and increasing personal consumption expenditures in markets around the world will continue to drive greater demand for our beverages."

Coca-Cola is not the only **fizzy drinks** maker to suffer from Americans losing their appetite for **sugary drinks**. Last week, its biggest rival, PepsiCo, said sales of its carbonated drinks had fallen by "mid-single digits".

LOAD-DATE: February 19, 2014

LANGUAGE: ENGLISH

PUBLICATION-TYPE: Newspaper

JOURNAL-CODE: WEBDTNS

Copyright 2014 Telegraph Media Group Limited
All Rights Reserved

The Telegraph
telegraph.co.uk

February 18, 2014 Tuesday 7:00 AM GMT

Life coach: are all **fizzy drinks** bad for you?; Our health experts answer your questions. This week: **fizzy drinks,** anxiety and paralysis and yeast allergies

SECTION: LIFESTYLE

LENGTH: 1237 words

Q: I have recently read about the effects of **fizzy drinks** on health, and the association between sugar-laden 'pop' and death rates from cardiovascular disease. Would swapping sugary **fizzy drinks** for their diet equivalents make a difference, or are artificial sweeteners as bad for you?

Corinna Young, London

A: Sara Stanner writes

As well as heart disease, being overweight increases the risk of Type 2 diabetes, some cancers and osteoarthritis, and sugar consumption has hardly been out of the headlines this year.

So many people are struggling with their weight and need to cut back on calories, and sugar-containing drinks are certainly an area to consider - particularly for children, who we know are consuming too much added sugar. More than a quarter of this comes from sugar-containing drinks like non-diet **fizzy drinks**, squash and sweetened juices. The calories from these drinks don't seem to satisfy us in the same way as calories from food would, so we end up consuming more without feeling full.

Diet drinks contain sweeteners like aspartame and Stevia, which provide a similar sweet taste with few or no calories. Opting for a diet version of a soft drink instead of a standard one can save you around 100 calories, so even if you are consuming two standard soft drinks a day that is a significant calorie reduction.

Some consumers have concerns about additives because they see long, unfamiliar names or E numbers and think of them as 'unnatural', but an E number is a reference number given to a food additive that has been approved for use throughout the EU. Those permitted for use in the UK have been extensively tested and their safety is regularly reviewed. However, people with a rare condition called phenylketonuria must avoid all products containing aspartame.

While water and milk are good choices as alternatives to **sugary drinks**, studies have shown that diet drinks can be beneficial for people trying to manage their weight by improving the palatability of their diet. Fruit

juices and smoothies naturally contain sugars and provide important nutrients such as vitamin C, folate and potassium, but they are also a source of calories and should be kept to meal times to protect the teeth.

Paralysed with fear

Q: When my daughter, 25, becomes stressed or anxious there are occasions when her hands become paralysed. It happened last year when we travelled in a light aircraft in rough weather conditions. A neurologist suggested it was connected to migraine and nothing more sinister like MS or Parkinson's disease. I have had PD for the past six years.

A: Dr Dan Rutherford writes

I'm glad to agree that this is not serious and is in fact quite common. When some people become acutely anxious they tend to breathe too rapidly (hyperventilate), which causes more carbon dioxide to be lost in the breath than usual. This alters the acidity of the blood, which in turn does odd things to the way the nerves and muscles work.

Typically the fingers and thumbs go into spasm, as can all the toes. Pins and needles sensations, often around the mouth, may accompany this seeming paralysis and the person usually feels very lightheaded and even more anxious.

The solution is to strongly reassure your daughter, and get her to deliberately focus on slowing down her rate of breathing. If she can't manage that then re-breathing for a short time into a paper bag does the trick, by making her reabsorb carbon dioxide. The whole process is actually reversible. As soon as hyperventilation stops, the blood rapidly returns to its normal acidity and the symptoms disappear.

Yeast-free living

Q: My daughter who was 40 last year has been treated for a yeast allergy condition for 18 months. She saw a doctor last week and was told the condition was not improving, even though she has been on a strict diet. The regime is very different from the dietary needs of her husband and children so I admire her for being able to cope with these demands on her lifestyle. I would be grateful for any advice to help her, please. Does exercise help?

A: Sara Stanner writes

Yeast fermentation is an important, natural and "traditional" process used in the brewing of beer, production of wine and bread, and in the manufacture of cheese and yogurt. A form of yeast can also be naturally present on ripe fruit and vegetables and pre-cooked foods.

Avoiding all sources of yeast is particularly challenging as it is often present in everyday foods such as bread, rolls, cakes and other cereal products, stock, gravy and salad dressings, dried fruit, dairy products, some processed foods, as well as many alcoholic drinks. Some foods that have been opened and stored for a long time, such as jam, can also develop yeasts on the surface.

If your daughter is still experiencing symptoms she might benefit from starting a food and symptom diary, where she records everything she eats and drinks over a week, as well as how she is feeling. This could help her identify anything that might be causing a problem for her. It is also important that she checks the ingredient list on packaged foods, as there is the possibility that the recipe of certain items may have changed and now contains yeast.

Although a yeast-free diet can be limiting, it is still possible for your daughter to consume a healthy diet that contains a variety of yeast-free foods and drinks. Although she can't eat many cereal products, soda, pitta and naan breads are usually yeast-free, as are most crispbreads and rice cakes - although it is always wise to check the labels.

Bear in mind, though, that persisting with a very restricted diet, which may limit intake of important nutrients, can cause problems of its own. So if your daughter's symptoms continue, she should visit her GP to make sure that they are due to yeast intolerance and ask to see an allergy dietitian who can give her advice tailored to her individual needs and requirements.

A: Tony Gallagher writes

Yeast intolerance, as opposed to a yeast allergy, can be affected by exercise. A good supply of oxygen helps the body to kill off germs, bacteria, viruses and some types of fungus. Medical studies have shown that exercising for 30 minutes each day, five days per week, could help reduce yeast infection symptoms. This should be moderate exercise, which will improve immune function, as opposed to more extensive exercise, which could suppress the immune system, making a person even more prone to yeast infections.

Perspiration can create the conditions in which yeast thrives. Yeast micro-organisms love warm, moist places, usually occurring in skin folds and areas where you sweat a lot. It is best to wear clothing that wicks away moisture to keep the skin clean and dry. Showering, and drying off thoroughly, immediately after exercise is advised.

Read more: Life coach: what is a good diet for Crohn's disease?

Read more: Life coach: is my unborn baby getting enough food?

Send your questions to lifecoach@telegraph.co.uk, or to 111 Buckingham Palace Road, London SW1W 0DT

LOAD-DATE: February 18, 2014

LANGUAGE: ENGLISH

PUBLICATION-TYPE: Newspaper

JOURNAL-CODE: WEBDTNS

Copyright 2014 Telegraph Media Group Limited
All Rights Reserved

DAILY MAIL (London)

February 17, 2014 Monday

MUM WHO BECAME HOOKED ON SUGAR

LENGTH: 640 words

ADDICT ATE 50 SPOONFULS A DAY BEFORE KICKING HABIT

BY ANDY DOLAN

A MOTHER who was so addicted to sugar she consumed the equivalent of 50 teaspoons a day has told how kicking the habit has transformed her life.

Nikki Oakley ate chocolate biscuits for breakfast, substituted supper with puddings and raided the sweet cupboard during years of overindulgence to satisfy her cravings.

Yesterday, the 45-year-old said that while her sugar habit did not cause her weight to balloon, it was an emotional crutch' that she turned to for a pick-me-up or even because she was bored.

The mother-of-two said her addiction was so severe that when she quit sugar she would often burst into tears and was short-tempered, listless and depressed. She also suffered severe headaches.

It took a fortnight for those feelings to subside,' she said. Everywhere I looked there were people eating bags of sweets, ice-creams and drinking **fizzy drinks**. Shopping was torture. I would walk up the aisles looking at the biscuits, feeling like crying, I wanted them so much.

It was a cycle - I ate sugary foods as a pick-me-up, but after the high came a low and I would need more.

But once I'd got through those first few weeks I started to feel so much better - calmer but more alert and with more energy.'

In the old days, if Mrs Oakley did have proper meals they consisted of sugar-laden cereal bars for breakfast, sandwiches with crisps and cake for lunch and processed ready meals for dinner with dessert.

Occasionally she would cook a curry or sweet and sour dish but the sauce would be from a jar and therefore high in sugar. Throughout the day she would snack on cookies, pies, sweets and chocolate bars. She would also sip on **fizzy drinks** all day.

Now, however, she has porridge for breakfast, chicken or ham salad for lunch and snacks on oatcakes with low-fat hummus, berries and natural yoghurt sprinkled with nuts or raisins. Instead of relying on microwaveable meals for dinner, she cooks curries, stir-fries and spaghetti bolognese from scratch without sauces from jars.

And Mrs Oakley, who works as a childminder and lives in Redditch, Worcestershire, with electrician husband Glyn, also 45, has cut out puddings completely. She had always jogged and now she is running faster than ever. Her weight has also dropped from 9st 11lb to 8st 7lb.

To help her change her ways, Mrs Oakley contacted The Healthy Employee, which works with companies to educate workers on diet and wellbeing to improve health and cut absenteeism. Its specialists deemed her a sugar addict.

Anna Mason, who runs the company, warned yesterday that sugar could be more powerful than any opiates' and said Britain is hooked on processed food'. She added: Many people on an apparently healthy diet can be consuming 40 to 50 teaspoons of sugar a day eating things like granola bars - which are held together with sugary syrup - or baked beans, which have sugar in the sauce.'

Mrs Oakley has been on a no-sugar diet since last September because even the smallest amount can reignite her cravings.

However, she admitted that she has occasionally been unable to resist temptation and recently had a gingerbread biscuit when baking with the children she cares for.

She said of her addiction: It takes hold after the first few mouthfuls of intense pleasure - I would start putting food into my mouth, even though I don't really want it.

Your head is telling you to stop, but you just can't.'

The recommended daily sugar intake for a woman is six teaspoons, according to the World Health Organisation, but the typical Briton will consume double that.

At a time when obesity and diabetes cost the UK more than £5billion a year, campaign group Action on Sugar is calling on food manufacturers to cut the sugar in their products by 20 to 30 per cent within the next three to five years.

a.dolan@dailymail.co.uk

© Daily Mail

LOAD-DATE: February 16, 2014

LANGUAGE: ENGLISH

PUBLICATION-TYPE: Papers

MailOnline

February 17, 2014 Monday 1:26 AM GMT

'I used to cry in the supermarket biscuit aisle because I wanted sugar so badly!' Woman addicted to the white stuff says too many sweets left her depressed

BYLINE: ANDY DOLAN

SECTION: FEMAIL

LENGTH: 672 words

- Nikki Oakley, 45, from Redditch, was eating a huge 50tsp of sugar a day
- Mound of sweets left her depressed, listless and plagued by headaches
- Has been diagnosed with sugar addiction and banned from eating it
- Cries in supermarket aisles because she wants biscuits so badly

A mother who was so addicted to sugar she consumed the equivalent of 50 teaspoons a day has told how kicking the habit has transformed her life.

Nikki Oakley ate chocolate biscuits for breakfast, substituted supper with puddings and raided the sweet cupboard during years of overindulgence to satisfy her cravings.

Yesterday, the 45-year-old said that while her sugar habit did not cause her weight to balloon, it was an 'emotional crutch' that she turned to for a pick-me-up or even because she was bored.

The mother-of-two said her addiction was so severe that when she quit sugar she would often burst into tears and was short-tempered, listless and depressed. She also suffered severe headaches.

'It took a fortnight for those feelings to subside,' she said. 'Everywhere I looked there were people eating bags of sweets, ice-creams and drinking **fizzy drinks**. Shopping was torture. I would walk up the aisles looking at the biscuits, feeling like crying, I wanted them so much.

'It was a cycle - I ate sugary foods as a pick-me-up, but after the high came a low and I would need more.

'But once I'd got through those first few weeks I started to feel so much better - calmer but more alert and with more energy.'

In the old days, if Mrs Oakley did have proper meals they consisted of sugar-laden cereal bars for breakfast, sandwiches with crisps and cake for lunch and processed ready meals for dinner with dessert.

Occasionally she would cook a curry or sweet and sour dish but the sauce would be from a jar and therefore high in sugar. Throughout the day she would snack on cookies, pies, sweets and chocolate bars. She would also sip on **fizzy drinks** all day.

Now, however, she has porridge for breakfast, chicken or ham salad for lunch and snacks on oatcakes with low-fat hummus, berries and natural yoghurt sprinkled with nuts or raisins. Instead of relying on microwaveable meals for dinner, she cooks curries, stir-fries and spaghetti bolognese from scratch without sauces from jars.

And Mrs Oakley, who works as a childminder and lives in Redditch, Worcestershire, with electrician husband Glyn, also 45, has cut out puddings completely. She had always jogged and now she is running faster than ever. Her weight has also dropped from 9st 11lb to 8st 7lb.

To help her change her ways, Mrs Oakley contacted The Healthy Employee, which works with companies to educate workers on diet and wellbeing to improve health and cut absenteeism. Its specialists deemed her a sugar addict.

Anna Mason, who runs the company, warned yesterday that sugar could be 'more powerful than any opiates' and said Britain is 'hooked on processed food'. She added: 'Many people on an apparently healthy diet can be consuming 40 to 50 teaspoons of sugar a day eating things like granola bars - which are held together with sugary syrup - or baked beans, which have sugar in the sauce.'

Mrs Oakley has been on a no-sugar diet since last September because even the smallest amount can reignite her cravings.

However, she admitted that she has occasionally been unable to resist temptation and recently had a gingerbread biscuit when baking with the children she cares for.

She said of her addiction: 'It takes hold after the first few mouthfuls of intense pleasure - I would start putting food into my mouth, even though I don't really want it.

'Your head is telling you to stop, but you just can't.'

The recommended daily sugar intake for a woman is six teaspoons, according to the World Health Organisation, but the typical Briton will consume double that.

At a time when obesity and diabetes cost the UK more than £5billion a year, campaign group Action on Sugar is calling on food manufacturers to cut the sugar in their products by 20 to 30 per cent within the next three to five years.

LOAD-DATE: February 17, 2014

LANGUAGE: ENGLISH

PUBLICATION-TYPE: Web Publication

JOURNAL-CODE: WEBDM

Copyright 2014 Associated Newspapers Ltd.
All Rights Reserved

FT
FINANCIAL TIMES
Financial Times (London, England)

February 14, 2014 Friday
London Edition 3

Pepsi hit by refusal to break up;
FOOD PRODUCERS

BYLINE: Shannon Bond in New York

SECTION: COMPANIES; Pg. 20

LENGTH: 514 words

HIGHLIGHT: Calls for drinks spin-off rebuffed

Outlook adds to fall in share price

PepsiCo shares fell after it said it would not split off its drinks business despite a declining US soda market and activist investor pressure.

Its announcement that it would lift shareholder capital returns 35 per cent to $8.7bn this year and extend its cost-cutting plan failed to shore up confidence, with Pepsi shares falling 2.2 per cent to $79.69 by the close in New York trading.

The world's second-largest soft drink maker by market share delivered another rebuff to the call from activist investor Nelson Peltz to divide its drinks and snacks businesses, saying it would "maximise shareholder value" by keeping the units together.

While the decision had been widely anticipated, the share price fall might reflect disappointment with Pepsi's insistence on its "power of one" strategy, said Tom Mullarkey, analyst at Morningstar.

Ali Dibadj of Bernstein Research said the main issue was "a broader industry concern" as drinks makers grapple with changing consumer tastes.

Analysts also pointed to the company's 2014 core earnings forecast of $4.50 - well below Wall Street's consensus forecast of $4.69 - as a source of investor disappointment.

Pepsi said unfavourable foreign exchange rates would trim 4 percentage points from core profit growth this year, and gave a forecast of a 7 per cent rise this year - at the low end of its long-term "high single-digits" target.

Mr Peltz has been pressing Pepsi to split off the sluggish North American beverage business to allow its food brands, including Doritos and Quaker Oats, to grow faster.

North American drink sales volume dropped 3 per cent in 2013 after falling 1 per cent in 2012, as US consumers turned away from both high-calorie sodas and artificially sweetened diet drinks.

But yesterday Indra Nooyi, chief executive, said that after an "exhaustive" review, the company concluded "our portfolio is truly better together . . . our presence in beverages contributes to our snacks growth."

"We are encouraged with the progress we made in North American beverages with a category that remains challenged," Ms Nooyi said.

While she said demand for colas had been particularly affected, she added: "It's too big a business and too profitable a business not to let the transformation play out."

Pepsi said it planned to test drinks sweetened with a mix of sugar and stevia, a calorie-free sweetener, in the US this year. Rivals Coca-Cola and Dr Pepper Snapple have similar plans.

Ms Nooyi also suggested a foray into so-called "at-home" drinks - such as SodaStream or Green Mountain's Keurig system - could be in Pepsi's future. It had been testing single-serve home delivery products, she said.

The news came as Pepsi reported a 9 per cent rise in 2013 net income to $6.74bn, or $4.32 a share, from $6.18bn, or $3.92 a share, in 2012. Full-year revenue was up 1 per cent to $66.4bn from $65.5bn, in line with Wall Street's forecast.

Global food sales volume rose 3 per cent last year while drink sales were up 1 per cent. Declining drink sales in North America and Europe were offset by growing demand for snacks and emerging markets growth.

LOAD-DATE: February 13, 2014

LANGUAGE: ENGLISH

PUBLICATION-TYPE: Newspaper

Copyright 2014 The Financial Times Ltd.
All Rights Reserved
Please do not cut and paste FT articles and redistribute by email or post to the web.

DAILY MAIL (London)

February 13, 2014 Thursday

OBESITY UK: IT'S WORSE THAN FEARED

BYLINE: BY SOPHIE BORLAND HEALTH REPORTER

LENGTH: 771 words

DIRE warnings that half of Britons will be obese by 2050 are an underestimate', a shock report warns today.

The obesity epidemic could be far worse than predicted because experts did not factor in how much fatter we are getting as we age, it says.

The report blames junk food firms for confusing the public about healthy eating, and says ministers and GPs are failing to get a grip of the problem, with NHS systems not fit for purpose'.

More than a quarter of adults (26 per cent) are obese, up from just 8 per cent in 1980.

In 2007, an alarming government review warned that by 2050, obesity would affect half of all adults and cost the economy £50billion a year. Yet a report by the National Obesity Forum says the predictions were optimistic' and underestimated the true scale of the problem'.

The forum - which comprises doctors and other experts - says the Government is focusing too much on prevention rather than

helping those who are obese lose weight. It also describes a reward system for GPs, which pays them to record whether patients are too fat without having to offer any dietary advice, as being unfit for purpose'.

Dr Aseem Malhotra, a cardiologist at Croydon University Hospitals, South London, said obesity was the greatest threat to health worldwide'.

He said poor diet was contributing to more disease than physical inactivity, smoking and alcohol combined, and accused junk food firms of adopting strategies of denial' and trying to confuse the public to ensure they carried on buying unhealthy products.

Dr Malhotra said the companies were using the same tactics as those used by the tobacco industry 50 years ago when evidence first emerged that smoking caused lung cancer.

Junk food companies sponsor sporting events and athletes endorse **sugary drinks**, with advertising that targets the most vulnerable members of society, including children,' he said.

Professor David Haslam, chairman of the forum, said that if anything, levels of obesity had worsened since the 2007 report which influenced several government policies.

He said: Not only is the situation not improving, but the doomsday scenario set out in that report might underestimate the true scale of the problem. There needs to be concerted action. We've seen hard-hitting campaigns against smoking and it's time to back up the work that's already being done with a similar approach for obesity.'

The Government has come under fire for failing to tackle obesity and campaigners including TV chef Jamie Oliver have condemned its plans as inadequate' and a cop-out'.

There is also widespread scepticism about its flagship responsibility deal' policy whereby food and drinks firms promise to make products healthier without facing any penalties if they don't. Campaigners have accused ministers are cosying up' to the manufacturers and putting Dracula in charge of the blood bank' by allowing them to set their own rules.

Today's report also criticises the Government's Change 4 Life drive - which uses adverts to encourage the public to eat healthily and take exercise for not having enough of an impact. The initiative has cost the taxpayer £75million since it was launched in January 2009 but the report says a harder-hitting campaign is needed.

It also urges GPs to regularly offer dieting and exercise advise for obese patients whenever they come in for appointments. Doctors shouldn't worry about insulting patients by implying they are fat', the report states.

One reason the scale of obesity may have previously been underestimated is that experts had not accounted for the fact that most of us get gradually fatter with age.

Research by Glasgow University in November showed that 85 per cent of men and 77 per cent of women are either obese or overweight when they reach 65.

A damning report by the World Health Organisation in October warned that the UK had the worst obesity rates in Europe and said the problem was a ticking time bomb waiting to explode'.

A Department of Health spokesman said: England has one of the highest rates of obesity in the Western world and it causes dangerous and life-limiting health conditions such as Type 2 diabetes and heart disease.

But this is not just a matter for government - we look to industry, health professionals and voluntary groups to work jointly to help individuals improve their diet and lifestyles.'

Professor Kevin Fenton, director of health and wellbeing at Public Health England - the government body tasked with tackling obesity - said: Obesity is an international problem. It is a complex issue that requires action at national, local, family and individual level.'

s.borland@dailymail.co.uk

© Daily Mail

LOAD-DATE: February 12, 2014

LANGUAGE: ENGLISH

PUBLICATION-TYPE: Papers

Copyright 2014 Associated Newspapers Ltd.
All Rights Reserved

i-Independent Print Ltd

February 13, 2014
First Edition

Dr Pepper loses its fizz as sales fall;
From the business pages The Wall Street Journal

SECTION: BUSINESS; Pg. 41

LENGTH: 69 words

The US drinks giant Dr Pepper Snapple Group saw sales and profit fall in the fourth quarter as the global appetite for **fizzy drinks** continued to decline. Sales fell by 1.4 per cent to $1.46bn (£880m), while profits fell to $156m (£94.3m), from $170m (£102.8m) a year earlier. The soft drink and juice maker said it expected sales to be flat this year and that it planned to buy back up to $400m (£241.9m) in stock.

LOAD-DATE: February 12, 2014

LANGUAGE: ENGLISH

PUBLICATION-TYPE: Newspaper

JOURNAL-CODE: III

Copyright 2014 Independent Print Limited
All Rights Reserved

MailOnline

MailOnline

February 13, 2014 Thursday 10:47 PM GMT

California bill seeks warnings on **sugary drinks**

BYLINE: ASSOCIATED PRESS

SECTION: AP

LENGTH: 325 words

SACRAMENTO, Calif. (AP) - California would become the first state to require warning labels on sodas and other **sugary drinks** under a proposal a state lawmaker announced Thursday.

SB1000 would require the warning on the front of all beverage containers with added sweeteners that have 75 or more calories in every 12 ounces. The label would read: "STATE OF CALIFORNIA SAFETY WARNING: Drinking **beverages** with added **sugar**(s) contributes to obesity, diabetes, and tooth decay."

Democratic Sen. William Monning, who proposed the bill, said there is overwhelming research showing the link between **sugary drinks** and those health problems, adding that the wording was developed by a national panel of nutrition and public health experts. The bill has the backing of the California Medical Association and the California Center for Public Health Advocacy.

"The goal of the warning quite simply is to give consumers the right to know what are well-established medical impacts from consuming these beverages," Monning, from Carmel, said in a telephone interview. "We're talking about a public health epidemic that will take more lives than gun violence."

The Latino Coalition for a Healthy California and the California Black Health Network also are sponsoring the legislation, citing the heavy consumption of **sugary drinks** and associated health problems among minorities.

A bill similar to Monning's was introduced last year in Vermont, but it has been held in the Committee on Human Services since April. The Vermont bill would require manufacturers to put warning labels on **beverages** that contain **sugar** or other artificial additives.

CalBev, the California arm of the Washington, D.C.-based American Beverage Association, noted that the industry already posts calorie counts on the front of many beverage containers as part of its "Clear on Calories" campaign that began in 2010. Also, drink bottles already have detailed ingredient lists and nutritional information.

LOAD-DATE: February 13, 2014

LANGUAGE: ENGLISH

PUBLICATION-TYPE: Web Publication

JOURNAL-CODE: WEBDM

Copyright 2014 Associated Newspapers Ltd.
All Rights Reserved

The Daily Telegraph

The Daily Telegraph (London)

February 12, 2014 Wednesday
Edition 1;
National Edition

Business needs to fight back against these unfair attacks from politicians; Viewpoint

BYLINE: Allister Heath

SECTION: BUSINESS; Pg. 2

LENGTH: 1201 words

Politics has become the greatest threat to Britain's recovery. The UK needs the private sector to hire and invest if we are to achieve genuine, sustainable growth; and for that a predictable, reassuring and supportive political climate is required to give companies the confidence to spend.

Instead, the political classes, in their wisdom, have decided to ramp up their attacks on business, floating ever more extreme proposals. Barely a day goes by without another proposal from either the Coalition or Labour to interfere, to tax, to regulate, to micro-manage, to punish or to shame.

Many businesses were already nervous ahead of the Scottish referendum and general election; but the chutzpah implicit in the latest threats to nationalise and break up energy companies, at the very same time that they are being asked to invest vast amounts in the country's crumbling infrastructure, has rattled many boardrooms.

To many wealth creators and captains of industry, it feels as if the UK is slowly falling out of love with business, and that the climate will worsen considerably over the next few years with yet more, usually irrational, government intervention, higher tax rates on income and perhaps even Britain's first ever wealth tax.

The British establishment, they increasingly fear, no longer gets commerce. France may have started to understand that relentless assaults on capitalism don't work; but the British are sleepwalking down a road first taken by Francois Hollande, and are in urgent need of being jolted out of their stupor.

The only answer is for companies to fight back far more effectively, to defend themselves from unfair attacks and to highlight the good that they do. It was excellent to see the Confederation of British Industry speak out on Monday, warning that all of this politicking risked being a "mood killer" for investment. Even more importantly, companies need to throw caution to the wind and join in directly, and those firms that are facing the worst attacks need to learn to hold their heads high and to speak up for themselves more forcibly.

They need to explain that careless talk costs jobs, and demonstrate how knee-jerk legislation will be lethal for our economic prospects. Take finance, that most demonised of sectors: Britain used to be the world's largest exporter of financial services, helping to pay for many of our exports of manufactured goods. But United Nations Conference on Trade and Development (UNCTAD) figures show that the UK's financial services exports have fallen by 20pc since their peak seven years ago. America's, by contrast, have continued to grow despite the crisis and have now overtaken ours for the first time.

The corporate sector as a whole needs encouraging, not bashing.

Britain's top 100 firms and their staff across all sectors of the economy paid £77.6bn in tax the last financial year, up from £77.1bn in the previous 12 months and equivalent to 15pc of the UK's total tax take, according to PwC. It is in all of our interest for companies to grow and expand, not to retrench.

Of course, the banking sector in particular was in need of dramatic reforms in 2008; but most of the changes required are now either in place or well under way. The forex market scandal still needs to be dealt with severely, and the resolution mechanisms to allow troubled banks to go bust and be wound down safely, avoiding the outrage of taxpayer bail-outs, need to be finalised. But we are almost there; the next challenge is to make sure that the City can start growing again, creating jobs, wealth and tax receipts.

Yet many still seek to refight yesterday's battles, or to pretend that the sector's pay practices haven't been drastically transformed. Take the manufactured outrage at Barclays, which has just suffered a bad drop in profits while simultaneously boosting pay.

The firm has stumbled and deserves to be held to account. But does anybody really believe that Barclays wants to pay its staff more than it has to, given market forces, or that it is not already well aware that it needs to keep costs under control? Its CEO's wealth is closely aligned to the value of the share price, which fell sharply yesterday when it was announced that compensation went up as a share of revenues. Clearly, Antony Jenkins must do more - but he doesn't need politicians or activists to tell him that.

It is strange how the anti-business Left, which usually accuses the private sector of being too stingy towards staff, believes banks to be uniquely different and somehow willing to minimise profits to boost bonuses. The only workers the banker-bashers don't want to help are those in wholesale financial services; the only capitalists they feel sorry for are those who own bank shares.

If you are an energy investor, of course, then it's fine to see your wealth destroyed by mindless threats, which is exactly what has happened to energy company shareholders in recent months. The latest attack

comes from Ed Davey, the Secretary of State for Energy: he feels that Centrica's UK gas supply margins are too high.

Davey may well be right that the UK energy market is insufficiently competitive - but his letter doesn't even remotely begin to show that, and his analysis is shockingly amateurish and superficial. One cannot assess an industry merely by considering market share, or by looking in isolation at profit margins for one sub-set of operations for the past few years, ignoring fluctuations in global supply and demand, other structural changes during that time, the company's return on capital, regulatory-imposed spending obligations, the degree of market contestability or comparable figures from other industries and countries. It just doesn't make sense.

While the energy market is in need of reform, politicians of all parties still find it easier to blame supposedly exploitative businesses, while asking them to underwrite their giant experiment with ultra-costly renewable energy, one of the most damaging policy blunders of recent years.

Food and drink companies are also in the politicians' sights for making money from **sugary drinks** and sweets; retailers will undoubtedly be next. Yet people should have the right to eat and drink what they want. Companies should make the contents of what they are selling clear, but ultimately individuals need to exercise responsibility. If people want to consume too much sugar and not exercise enough, they should take the blame - not the maker of chocolate bars or the retailers.

Business needs to fight back in other areas, too. Competition and open markets, not quotas, are the best way to promote meritocracy and diversity in the workplace, and the best companies are in the vanguard of social progress. Women aged between 22 and 29 in full-time work now earn 0.3pc more than their male counterparts, and between the ages of 30 and 39, men earn just 1pc more - for these groups, the gender pay gap has effectively been eliminated.

Plenty of huge and often intractable problems remain but businesses need to learn to boast about what they are getting right.

Allister Heath is editor of City AM

'Companies need to speak up for themselves more forcibly and explain that careless talk costs jobs'

LOAD-DATE: February 12, 2014

LANGUAGE: ENGLISH

GRAPHIC: David Cameron at the CBI conference. The industry body has warned that politicking risks being a 'mood killer' for investment
PETER MACDIARMID/GETTY IMAGES

PUBLICATION-TYPE: Newspaper

JOURNAL-CODE: DTL

Copyright 2014 Telegraph Media Group Limited
All Rights Reserved

The Telegraph
telegraph.co.uk

February 11, 2014 Tuesday 8:54 PM GMT

Business needs to fight back against these unfair attacks from politicians;
To many wealth creators and captains of industry, it feels as if the UK is slowly falling out of love with business, and that the climate will worsen considerably over the next few years with yet more, usually irrational, government intervention

BYLINE: By Allister Heath

SECTION: FINANCE

LENGTH: 1189 words

Politics has become the greatest threat to Britain's recovery. The UK needs the private sector to hire and invest if we are to achieve genuine, sustainable growth; and for that a predictable, reassuring and supportive political climate is required to give companies the confidence to spend.

Instead, the political classes, in their wisdom, have decided to ramp up their attacks on business, floating ever more extreme proposals. Barely a day goes by without another proposal from either the Coalition or Labour to interfere, to tax, to regulate, to micro-manage, to punish or to shame.

Many businesses were already nervous ahead of the Scottish referendum and general election; but the chutzpah implicit in the latest threats to nationalise and break up energy companies, at the very same time that they are being asked to invest vast amounts in the country's crumbling infrastructure, has rattled many boardrooms.

To many wealth creators and captains of industry, it feels as if the UK is slowly falling out of love with business, and that the climate will worsen considerably over the next few years with yet more, usually irrational, government intervention, higher tax rates on income and perhaps even Britain's first ever wealth tax.

The British establishment, they increasingly fear, no longer gets commerce. France may have started to understand that relentless assaults on capitalism don't work; but the British are sleepwalking down a road first taken by Francois Hollande, and are in urgent need of being jolted out of their stupor.

The only answer is for companies to fight back far more effectively, to defend themselves from unfair attacks and to highlight the good that they do. It was excellent to see the Confederation of British Industry speak out on Monday, warning that all of this politicking risked being a "mood killer" for investment.

Even more importantly, companies need to throw caution to the wind and join in directly, and those firms that are facing the worst attacks need to learn to hold their heads high and to speak up for themselves more forcibly.

They need to explain that careless talk costs jobs, and demonstrate how knee-jerk legislation will be lethal for our economic prospects. Take finance, that most demonised of sectors: Britain used to be the world's largest exporter of financial services, helping to pay for many of our exports of manufactured goods.

But United Nations Conference on Trade and Development (UNCTAD) figures show that the UK's financial services exports have fallen by 20pc since their peak seven years ago. America's, by contrast, have continued to grow despite the crisis and have now overtaken ours for the first time.

The corporate sector as a whole needs encouraging, not bashing.

Business needs to fight back against these unfair attacks from politicians; To many wealth creators and captains of industry, it feels as if the UK is slowly falling out of love with business, and that the climate will worsen considerably over the next few years with yet more, usually irrational, government intervention

telegraph.co.uk February 11, 2014 Tuesday 8:54 PM GMT

Britain's top 100 firms and their staff across all sectors of the economy paid £77.6bn in tax the last financial year, up from £77.1bn in the previous 12 months and equivalent to 15pc of the UK's total tax take, according to PwC. It is in all of our interest for companies to grow and expand, not to retrench.

Of course, the banking sector in particular was in need of dramatic reforms in 2008; but most of the changes required are now either in place or well under way.

The forex market scandal still needs to be dealt with severely, and the resolution mechanisms to allow troubled banks to go bust and be wound down safely, avoiding the outrage of taxpayer bail-outs, need to be finalised. But we are almost there; the next challenge is to make sure that the City can start growing again, creating jobs, wealth and tax receipts.

Yet many still seek to refight yesterday's battles, or to pretend that the sector's pay practices haven't been drastically transformed. Take the manufactured outrage at Barclays, which has just suffered a bad drop in profits while simultaneously boosting pay.

The firm has stumbled and deserves to be held to account. But does anybody really believe that Barclays wants to pay its staff more than it has to, given market forces, or that it is not already well aware that it needs to keep costs under control?

Its CEO's wealth is closely aligned to the value of the share price, which fell sharply yesterday when it was announced that compensation went up as a share of revenues.

Clearly, Antony Jenkins must do more - but he doesn't need politicians or activists to tell him that.

It is strange how the anti-business Left, which usually accuses the private sector of being too stingy towards staff, believes banks to be uniquely different and somehow willing to minimise profits to boost bonuses.

The only workers the banker-bashers don't want to help are those in wholesale financial services; the only capitalists they feel sorry for are those who own bank shares.

If you are an energy investor, of course, then it's fine to see your wealth destroyed by mindless threats, which is exactly what has happened to energy company shareholders in recent months.

The latest attack comes from Ed Davey, the Secretary of State for Energy: he feels that Centrica's UK gas supply margins are too high.

Davey may well be right that the UK energy market is insufficiently competitive - but his letter doesn't even remotely begin to show that, and his analysis is shockingly amateurish and superficial.

One cannot assess an industry merely by considering market share, or by looking in isolation at profit margins for one sub-set of operations for the past few years, ignoring fluctuations in global supply and demand, other structural changes during that time, the company's return on capital, regulatory-imposed spending obligations, the degree of market contestability or comparable figures from other industries and countries. It just doesn't make sense.

While the energy market is in need of reform, politicians of all parties still find it easier to blame supposedly exploitative businesses, while asking them to underwrite their giant experiment with ultra-costly renewable energy, one of the most damaging policy blunders of recent years.

Food and drink companies are also in the politicians' sights for making money from **sugary drinks** and sweets; retailers will undoubtedly be next.

Yet people should have the right to eat and drink what they want. Companies should make the contents of what they are selling clear, but ultimately individuals need to exercise responsibility. If people want to consume too much sugar and not exercise enough, they should take the blame - not the maker of chocolate bars or the retailers.

Business needs to fight back in other areas, too. Competition and open markets, not quotas, are the best way to promote meritocracy and diversity in the workplace, and the best companies are in the vanguard of social progress.

Business needs to fight back against these unfair attacks from politicians; To many wealth creators and captains of industry, it feels as if the UK is slowly falling out of love with business, and that the climate will worsen considerably over the next few years with yet more, usually irrational, government intervention
telegraph.co.uk February 11, 2014 Tuesday 8:54 PM GMT

Women aged between 22 and 29 in full-time work now earn 0.3pc more than their male counterparts, and between the ages of 30 and 39, men earn just 1pc more - for these groups, the gender pay gap has effectively been eliminated.

Plenty of huge and often intractable problems remain but businesses need to learn to boast about what they are getting right.

Allister Heath is editor of City A.M.

LOAD-DATE: February 12, 2014

LANGUAGE: ENGLISH

PUBLICATION-TYPE: Web Publication

JOURNAL-CODE: WEBDTNS

Copyright 2014 Telegraph Media Group Limited
All Rights Reserved

The Daily Telegraph

The Daily Telegraph (London)

February 10, 2014 Monday
Edition 1;
National Edition

There can't be many, if [...]; Doctor's Diary

BYLINE: James Le Fanu

SECTION: FEATURES; OPINION, COLUMN; Pg. 21

LENGTH: 192 words

There can't be many, if any, readers of this paper who consume three cans of **fizzy drinks** a day, so the latest scare story - that such a level of consumption triples the risk of heart disease - will surely not apply.

However, this is just the latest scientific finding in a campaign to blame sugar for practically everything, particularly the so-called epidemic of diabetes and obesity. It is not necessarily that people are consuming more sugar, but rather, it is claimed by Californian endocrinologist and prominent anti-sugar evangelist Robert Lustig, the wrong sort: the cheap, high-fructose corn syrup often found in carbonated drinks that has replaced one third of our total sugar intake over the past 30 years.

The fact remains that sugar in any form has never been demonstrated to cause either obesity or diabetes as an independent factor distinct from total calorie consumption - that is, people eating more than they should. Meanwhile, as the main constituent of oral rehydration therapy for children with severe diarrhoeal illnesses, sugar might be considered the most health-giving of all foods, saving an estimated two million lives a year.

LOAD-DATE: February 10, 2014

LANGUAGE: ENGLISH

PUBLICATION-TYPE: Newspaper

JOURNAL-CODE: DTL

Copyright 2014 Telegraph Media Group Limited
All Rights Reserved

MailOnline

February 10, 2014 Monday 1:22 PM GMT

Fruit juice should NOT count in our five-a-day because some versions 'contain as much sugar as **fizzy drinks'**

BYLINE: RACHEL WATSON and ANNA HODGEKISS

SECTION: HEALTH

LENGTH: 1028 words

- Juice is potentially 'just as bad' as sugary, sweetened drinks, say doctors
- 250ml of apple juice typically contains 110 calories and 26g of sugar
- 250ml of cola typically contains 105 calories and 26.5g of sugar
- People who drank 500ml of grape juice every day for three months had increased insulin resistance and a larger waist circumference
- Experts say the recommended amount should be no more than 150ml a day

Fruit juice should not be classed as one of our five-a-day because some versions contain almost as much sugar as **fizzy drinks**, scientists have warned.

Researchers from Glasgow University say that drinking fruit juice is potentially 'just as bad for you' as sugary, sweetened drinks.

They have asked the UK government to change its guidelines and have also recommended that labels should be placed on fruit juice containers telling people not to drink more than 150ml (¼pt) a day.

A 250ml serving of orange juice contains 115 calories - and many people drink more than this, with many 500ml servings available in high street cafes.

Even pure fruit juice is said to contain a large amount of naturally-occurring sugar - but people end up drinking too much of it because they do not see it as unhealthy.

Professor Naveed Sattar and Dr Jason Gill, from the university's Institute of Cardiovascular and Medical Sciences, say viewing juice as a fruit equivalent was 'probably counter-productive' as it 'fuels the perception that drinking fruit juice is good for health'.

Writing in The Lancet Diabetes and Endocrinology journal, Professor Sattar, professor of metabolic medicine, and Dr Gill identified a possible link between high fruit juice intake and an increased risk of diabetes.

The researchers believe cutting fruit juice intake could have 'major health benefits' such as a fall in obesity and heart problems.

They conducted a trial which concluded that drinking 500ml of grape juice per day for three months increased insulin resistance - which can lead to Type 2 diabetes - as well as the waist circumference in overweight adults.

Although fruit juices do contain vitamins and minerals, which sugar-sweetened drinks do not, Dr Gill argued that this 'might not be sufficient to offset the adverse metabolic consequences of excessive fruit juice consumption'.

HOW SOFT DRINKS COMPARE

Fruit juice has a similar energy density and sugar content to other **sugary drinks**.

For example, 250ml (almost ½pt) of apple juice typically contains 110 calories and 26g (0.91oz) of sugar.

250ml of cola typically contains 105 calories and 26.5g (0.93oz) of sugar.

An apple, on the other hand, contains around 50 calories - and you get the benefit of fibre, creating a feeling of fullness and aiding digestion.

The research also highlighted that a glass of fruit juice contains 'substantially' more sugar than a piece of fruit, and that much of the 'goodness' in fruit, such as fibre, is often not found in fruit juice.

In a test measuring public awareness of sugar content, 48 per cent of those who took the poll underestimated the sugar content of fruit juices and smoothies, while 12 per cent overestimated the sugar content of carbonated drinks.

More than 2,000 adults took part in the survey, in which they were shown pictures of full containers of different non-alcoholic drinks and were asked to estimate the number of teaspoons of sugar in each.

While there have been calls to ban children from drinking fruit juice in the US, the researchers did not recommend this for the UK, and also stated that a fruit juice tax should not be introduced.

Dr Gill said that despite popular belief, fruit juices are not as healthy as they seem.

He added: 'Contrary to the general perception of the public, and of many healthcare professionals, that drinking fruit juice is a positive health behaviour, their consumption might not be substantially different in health terms than drinking other **sugary drinks**.'

Professor Sattar said: 'Fruit juice has a similar energy density and sugar content to other **sugary drinks**. For example, 250ml (almost ½pt) of apple juice typically contains 110 calories and 26g (0.91oz) of sugar; and 250ml of cola typically contains 105 calories and 26.5g (0.93oz) of sugar.'

An apple, on the other hand, contains around 50 calories - and you get the benefit of fibre, creating a feeling of fullness and aiding digestion.

Professor Sattar added: 'Additionally, by contrast with the evidence for solid fruit intake, for which high consumption is generally associated with reduced or neutral risk of diabetes, current evidence suggests high fruit juice intake is associated with increased risk of diabetes.

'We have known for years about the dangers of excess saturated fat intake, an observation which led the food industry to replace unhealthy fats with presumed "healthier" sugars in many food products.

'Helping individuals cut not only their excessive fat intake, but also refined sugar intake, could have major health benefits including lessening obesity and heart attacks.

'There needs to be a refocus to develop foods which not only limit saturated fat intake but simultaneously limit refined sugar content.'

He added: 'In the broader context of public health policy, it is important that debate about **sugar**-sweetened **beverage** reduction should include fruit juice.'

Commenting on the research, Gavin Partington, Director General of the British Soft Drinks Assocation, said: 'The majority of adults and children in the UK do not meet the minimum recommendation of 5 portions of fruit and vegetables a day.

'Fruit juice does contain naturally occurring sugar, but also provides essential vitamins and nutrients which is why Government has recommended that it can count as one of your 5 a day.

'It's worth remembering that fruit juice accounts for just 1 per cent of the calories in the average British diet but like all food and drink it should be consumed as part of a balanced diet and active lifestyle.'

LOAD-DATE: February 10, 2014

LANGUAGE: ENGLISH

PUBLICATION-TYPE: Web Publication

JOURNAL-CODE: WEBDM

Copyright 2014 Associated Newspapers Ltd.
All Rights Reserved

The Telegraph
telegraph.co.uk

February 10, 2014 Monday 7:15 AM GMT

Doctor's Diary on sugar: Scaremongers fail to mention that it also saves millions of lives;
For some seriously ill children, sugar might be considered the most health-giving of all foods

LENGTH: 643 words

There can't be many, if any, readers of this paper who consume three cans of **fizzy drinks** a day, so the latest scare story - that such a level of consumption triples the risk of heart disease - will surely not apply.

However, this is just the latest scientific finding in a campaign to blame sugar for practically everything, particularly the so-called epidemic of diabetes and obesity. It is not necessarily that people are consuming more sugar, but rather, it is claimed by Californian endocrinologist and prominent anti-sugar evangelist Robert Lustig, the wrong sort: the cheap, high-fructose corn syrup often found in carbonated drinks that has replaced one third of our total sugar intake over the past 30 years.

The fact remains that sugar in any form has never been demonstrated to cause either obesity or diabetes as an independent factor distinct from total calorie consumption - that is, people eating more than they should. Meanwhile, as the main constituent of oral rehydration therapy for children with severe diarrhoeal illnesses, sugar might be considered the most health-giving of all foods, saving an estimated two million lives a year.

The recent report from the Department of Energy that, on average, our homes are seven degrees Fahrenheit warmer than in the 1970s is scarcely surprising, as 40 years ago, two thirds did not have central heating. This has made us perhaps a less hardy nation - unlike elsewhere, as I learnt from a Russian patient who,

with her husband and three children, celebrated the Orthodox Feast of the Epiphany on January 19 with a swim in an outdoor pool in south London.

This chilly ritual has been taken up with great enthusiasm since the fall of communism. The spiritual benefits are paralleled by the physical and mental, as suggested by a study of 49 winter swimmers carried out by Prof Pirrko Huttinen of the University of Oslo, published in the International Journal of Circumpolar Health. Over a period of four months, they reported an improvement in memory and mood, less tension and fatigue, and reduced symptoms of rheumatism, asthma and fibromyalgia.

The young woman featured last week with a range of distressing neurological symptoms following her return from a backpacking holiday in West Africa has elicited some helpful observations. First, an RAF Group Captain, now retired, reports the same pattern of events following a sailing holiday on Minorca in 2007, starting with a severe sore throat, leading to deafness, loss of balance, facial palsy, difficulty in swallowing and double vision. It took 16 months to recover, and even now he is still quite deaf, his balance is poor and, bizarrely, his left eye closes at the breakfast table when eating and reading The Daily Telegraph at the same time.

This pattern of symptoms, observes neurologist Dr John Boughey, is strongly suggestive of an inflammatory process in the brain stem, perhaps caused by the herpes simplex virus - though there are several other possible candidates. The symptoms should continue to improve, albeit slowly, though the vertigo and clicking in the ear warrant a further assessment from an ENT specialist.

Finally, my thanks to a reader for passing on a most useful tip for those troubled by recurrent cystitis - as she has been for 15 years. Her GP arranged for her to have an ultrasound of the bladder, which demonstrated some residual urine. She was advised to walk around the bathroom for a couple of minutes after passing urine and to then try again. The result of this practice has been "astounding": she has not had cystitis since.

Email medical questions confidentially to Dr James LeFanu at drjames@telegraph.co.uk
Answers will be published on the Telegraph website every Friday, at telegraph.co.uk/health

LOAD-DATE: February 10, 2014

LANGUAGE: ENGLISH

PUBLICATION-TYPE: Web Publication

JOURNAL-CODE: WEBDTNS

Copyright 2014 Telegraph Media Group Limited
All Rights Reserved

The Express

February 6, 2014 Thursday
Edition 1;
National Edition

Obesity's a matter of self-indulgence - it's not a disease

BYLINE: Leo McKinstry

SECTION: EDITORIAL; OPINION, COLUMNS; Pg. 12

LENGTH: 887 words

....

IUSED to wonder which milestone of 40 I would reach first: my girth or my age. In the end there was no contest. Increasing corpulence meant that my waistline was the easy winner. The years since then have seen no reversal in the widening middle-age spread. The collar sizes keep rising along with the disapproval of doctors and the bills for furniture broken by my weight, including a once-robust iron bed.

But my experience is hardly unique. It seems that I am in step - if that is not too energetic a term - with the majority of British people. According to the latest alarming reports we are becoming a nation of dedicated artery cloggers and couch potatoes. Yesterday the Government's public health agency published new data which claimed that two-thirds of all adults in the country are overweight or obese.

In some areas such as Copeland in Cumbria and Doncaster in Yorkshire, 75 per cent of the population have joined the heavyweight division.

These findings reinforce the idea that Britain is in the midst of a full-blown obesity crisis, causing severe damage not only to the nation's pavements but even to the health service and the wider economy.

Estimates of the annual costs of obesity to the NHS range from £5billion to £8billion. There have also been claims that two million people are now eligible for expensive bariatric surgery, such as the fitting of gastric bands to reduce appetite, because their weight problems are so severe.

DR Tam Fry, head of the National Obesity Forum pressure group, described yesterday's figures as "almost criminal". Indeed his own forum argued last month that on current trends more than half the entire British population will be obese by 2050.

What is so striking about the institutional hysteria over obesity is the portrayal of we gutbuckets as the passive victims of forces beyond our control. The fault lies not with our own failure to exercise selfdiscipline but with uncaring political and corporate interests.

So the Government is attacked for cutting public services such as leisure centres, for failing to promote healthy eating programmes and for refusing to impose heavy regulations on the food industry. Similarly the big retailers and manufacturers are portrayed as cynical manipulators forcefeeding us sugar and junk food to maximise profits.

One politically correct narrative holds that obesity is the result of mass poverty, first because the disadvantaged suffer from low self-esteem which compels them to indulge in comfort eating and second because healthy foods are allegedly more expensive.

This focus on the victimhood of the overweight can be seen in programmes dreamed up to tackle obesity which all emphasise not personal responsibility but state intervention.

So in February 2013 the medical profession issued a 10-point plan that included a 20 per cent tax on **fizzy drinks,** a ban on junk food TV advertising before the watershed of 9pm, more stringent food labelling, restrictions on fast-food outlets near schools and the expenditure of at least £100million a year on "weight management services", similar to smoking cessation programmes. But these demands for a frenzy of taxpayer-funded activity are misplaced.

The blame for obesity lies not with the wicked Tories or greedy companies but with the fatties. We should face the consequences of our own selfindulgence.

I am overweight because I consume too much and take no exercise. The only time I ever break into a sweat is if I find there is no lager in the fridge.

Obesity is a lifestyle choice not a social condition or disease that can be caught. We do not need labels or taxes or awareness programmes or counselling or surgery. We just need to change our habits.

Public-health campaigners and Left-wing politicians do not see it like that. Just as in their great global warming scare they want to create a climate of doom-laden crisis so they can extend the influence of the state while also fuelling their self-important vanity as crusading saviours.

For the puritan public health zealots, never happier than when nosing through children's lunchboxes or raging against the iniquities of hamburgers, the major food companies are an enticing new target now that they have successfully turned the big tobacco companies into pariahs.

THIS eagerness for power and control must be challenged. The obesity crisis is artificial. In fact contrary to all the hysteria the latest figures from official health surveys shows that obesity has been on the decline since 2005.

The fat panic is further contradicted by the growth of food banks, which this year are predicted to supply almost one million users. In line with the concern about families not eating properly the National Childbirth Trust claimed that 55 per cent of parents of young children face a choice between "buying food or keeping their babies and toddlers warm". Well the campaigners cannot have it both ways. It is absurd to pretend that vast swathes of modern Britain are gripped by both hunger and obesity.

In truth both of these manufactured visions play to the sentimental cult of victimhood, where people can be rescued only by the big, high-taxing, interventionist state.

Ironically by crushing the concept of personal responsibility such an approach is likely to worsen unhealthy lifestyles.

'Face the consequences of lifestyle choices'

LOAD-DATE: February 6, 2014

LANGUAGE: ENGLISH

GRAPHIC: BAD DIET: Two-thirds of adults are overweight or obese
Picture: ALAMY

PUBLICATION-TYPE: Newspaper

JOURNAL-CODE: DXP

Copyright 2014 Express Newspapers
All Rights Reserved

Financial Times (London, England)

February 6, 2014 Thursday
London Edition 3

Coke takes 10% stake in Green Mountain; BEVERAGES

BYLINE: Shannon Bond in New York

SECTION: COMPANIES; Pg. 19

LENGTH: 391 words

HIGHLIGHT: Coffee machines to supply **fizzy drinks**

Coca-Cola is served by the glass, can, bottle - and soon, the capsule.

The world's largest beverage company by sales has taken a 10 per cent stake in Green Mountain Coffee Roasters, maker of the Keurig single-serve coffee brewer, for $1.25bn.

Coke will buy about 16.7m shares priced at $74.98 each, representing the volume-weighted average price over the last 50 days. Green Mountain shares surged 58 per cent in after-hours trading to $128.

The companies also signed a 10-year deal that will see Coke's beverages, which include Coca-Cola, Diet Coke, Sprite and Fanta, available in Green Mountain's forthcoming cold beverage machine.

The device, which is expected to launch next year will allow consumers to make cold drinks at home using single-serve capsules, or pods. It will compete with SodaStream, the Israeli company that sells soda flavourings and a machine that carbonates water.

"Keurig can do for cold beverages what has been done with hot coffee and tea at home," said Brian Kelley, the Green Mountain chief executive who formerly worked for Coca-Cola. "We believe there is significant opportunity to premiumise and accelerate growth in the cold beverage category."

The machine will make both carbonated and still drinks, meaning that it can also be used for sports drinks, juices and teas. Mr Kelley said the deal would give Green Mountain access to Coke's global distribution system as well as to its range of drinks.

Muhtar Kent, Coca-Cola chief executive, said the deal would be an "enhancement" to the company's bottling system , under which a number of independent companies distribute and in some cases produce its drinks. He said the bottlers would play a "complementary" role in the Green Mountain agreement.

"This is not a zero-sum game, it just provides more opportunity for our brands," he said.

Green Mountain estimates that each of the US's 120m households drinks 14 beverages a day.

"By tapping into these beverage occasions, Green Mountain hopes to become a major player, with established retail distribution, in the $98bn retail soft drinks market," said Jonas Feliciano, an analyst at Euro-monitor International.

The deal with Coke has "potential to be a game changer," Mr Feliciano said. "SodaStream has to be concerned."

Coca-Cola shares rose 1 per cent in after-hours trade to $37.99. SodaStream was down 7.8 per cent to $33.

LOAD-DATE: February 5, 2014

LANGUAGE: ENGLISH

PUBLICATION-TYPE: Newspaper

The Daily Telegraph

The Daily Telegraph (London)

February 5, 2014 Wednesday
Edition 1;
National Edition

The sugar-slashers are on the warpath

BYLINE: ANJANA AHUJA

SECTION: EDITORIAL; OPINION, COLUMNS; Pg. 18

LENGTH: 821 words

The jaunty slogan on the metallic green tin is meant to convey the carefree essence of childhood: "Don't Grow Up. 7 Up." When viewed through the lens of recent headlines, however, the phrase acquires a more sinister interpretation. According to an American study published this week, three cans of carbonated drinks contain enough calories to triple a person's risk of developing heart disease. The suggestion is that if you spend your childhood knocking back gallons of sugary liquid, you may not get the opportunity to grow up at all.

The World Health Organisation was, coincidentally, ploughing a similar furrow yesterday. Its new World Cancer Report notes that half of cancers could be prevented via lifestyle changes - singling out alcohol, obesity and sugar as aggravating factors. Whichever way you cut it, health officials are beginning to fall out of love with the sweet stuff.

So why has our affair with sugar suddenly turned bitter? Well, partly because we needed a new target. We've had the war on tobacco, and on fat. Salt is another recent adversary, with a group called the Consensus Action on Salt and Health, or CASH, spearheading a British campaign to lower salt in foods. The strategy is to isolate an unhealthy product and bash it relentlessly. And for the past half-century, it's been brilliantly successful. When it came to salt, the pressure forced food manufacturers to sign up to reduce the amount in products such as bread and crisps. As a result, we consume around 15 per cent less than a decade ago.

With salt slain, it was time to move on. And so, last month, some of the people behind CASH decided to set up Action on Sugar, with some ambitious, well-meaning aims: to cut the amount of refined added sugar to the point that it constitutes no more than 5 per cent of a product's calories; to persuade food firms to recognise added sugar as a health problem; to see children recognised as a "vulnerable group" whose developing bodies can be particularly harmed by sugar; to improve labelling; and, if all else fails, to push for a sugar tax.

Action on Sugar is chaired, as was CASH, by Graham MacGregor, Professor of Cardiovascular Medicine at Queen Mary, University of London. But it is the name of its science director, Aseem Malhotra, that might be more familiar: he wrote a controversial article in the British Medical Journal last year, arguing that saturated fat had been unfairly demonised as a cause of heart disease. Although widely aired, this view was not wholly endorsed by the British Heart Foundation: it insisted that people with the highest cholesterol were still at the greatest risk of a heart attack.

And that - in a sugar-coated nutshell - is the problem with campaigns like this.

Quantifying lifestyle factors is not always straightforward. Take the **fizzy drinks** survey. It did find a correlation between estimated added sugar intake and cardiovascular disease. But the groups being compared were at opposite extremes of the consumption spectrum: either heavy or light consumers. As pointed out by David Spiegelhalter, Winton Professor of the Public Understanding of Risk at Cambridge University, "the increased hazard for moderately different groups is not that great". Cutting down on added sugar would have substantial benefits for overall public health - but the average person might not benefit that much.

These are complex nutritional messages to digest, and any anti-**sugar** lobby needs to be careful. Food and **beverage** firms will seize on the smallest uncertainty and inconsistency in the scientific evidence to preserve the profitable, teeth-rotting status quo. Campaigners will only be trusted if they play it absolutely straight.

That uncertainty also gives weak-willed consumers an excuse to keep high-sugar foods in the shopping basket - especially when it can hide behind a shocking multitude of euphemisms, such as fructose, glucose, sucrose, corn syrup, molasses and maple syrup. Remember, nature is on the side of the sugar-pushers: it is cheap to produce, seductive to the palate - which has been honed through evolution to seek out energy-rich foods - and extremely hard to replace without consumers noticing. Artificial sweeteners, in any case, may not be a risk-free alternative: there is evidence that they do not always trigger the body's satiety circuits. (In his BMJ piece, Dr Malhotra usefully revealed that low-fat yoghurts were packed with sugars.)

The campaigners' central message is sound: it is virtually certain that deriving more than 10 per cent of your calorie intake from added sugars increases the risk of obesity, diabetes and heart disease - as well as self-loathing. But it's only one of many facets of our lifestyle that we should be reflecting on. Plus, there's a cheering, bittersweet irony: added sugar is only killing us because we are not dying of something else first.

Comment on Anjana Ahuja's view at www.telegraph.co.uk/personalview

LOAD-DATE: February 5, 2014

LANGUAGE: ENGLISH

PUBLICATION-TYPE: Newspaper

JOURNAL-CODE: DTL

FREE METRO

Metro (UK)

February 5, 2014 Wednesday
Edition 1;
National Edition

Live well, die bored

SECTION: FEATURES; Pg. 14

LENGTH: 123 words

So drinking one can of **sugary drink** a day can increase the risk of heart disease (Metro, Mon). We should eat nothing but fruit and vegetables, drink boiled water and maybe live to a ripe old age.

But how many of us would want to live like that? When I was a child, we rarely had **fizzy drinks,** burgers or ice cream because they were considered treats.

We did, however, eat fried ¦breakfasts, fish and chips and roast dinners, and, from a young age, we were allowed small amounts of watereddown alcohol. I'm still active in my late-fifties and there are people older than me who I'm sure have a similar childhood background.

If we use a little common sense, we can enjoy the 'bad' foods as long as we don't overdo them.

Alan Wilton, via Facebook

LOAD-DATE: February 5, 2014

LANGUAGE: ENGLISH

PUBLICATION-TYPE: Newspaper

JOURNAL-CODE: MTR

Copyright 2014 Associated Newspapers Ltd.
All Rights Reserved

The Telegraph

telegraph.co.uk

February 5, 2014 Wednesday 10:39 AM GMT

The sugar-slashers are on the warpath;
First fat, then salt - now the sweet stuff is being targeted for removal from the British diet

BYLINE: By Anjana Ahuja

LENGTH: 809 words

The jaunty slogan on the metallic green tin is meant to convey the carefree essence of childhood: "Don't Grow Up. 7 Up." When viewed through the lens of recent headlines, however, the phrase acquires a more sinister interpretation. According to an American study published this week, three cans of carbonated drinks contain enough calories to triple a person's risk of developing heart disease. The suggestion is that if you spend your childhood knocking back gallons of sugary liquid, you may not get the opportunity to grow up at all.

The World Health Organisation was, coincidentally, ploughing a similar furrow yesterday. Its new World Cancer Report notes that half of cancers could be prevented via lifestyle changes - singling out alcohol, obesity and sugar as aggravating factors. Whichever way you cut it, health officials are beginning to fall out of love with the sweet stuff.

So why has our affair with sugar suddenly turned bitter? Well, partly because we needed a new target. We've had the war on tobacco, and on fat. Salt is another recent adversary, with a group called the Consensus Action on Salt and Health, or CASH, spearheading a British campaign to lower salt in foods. The strategy is to isolate an unhealthy product and bash it relentlessly. And for the past half-century, it's been brilliantly successful. When it came to salt, the pressure forced food manufacturers to sign up to reduce the amount in products such as bread and crisps. As a result, we consume around 15 per cent less than a decade ago.

With salt slain, it was time to move on. And so, last month, some of the people behind CASH decided to set up Action on Sugar, with some ambitious, well-meaning aims: to cut the amount of refined added sugar to

the point that it constitutes no more than 5 per cent of a product's calories; to persuade food firms to recognise added sugar as a health problem; to see children recognised as a "vulnerable group" whose developing bodies can be particularly harmed by sugar; to improve labelling; and, if all else fails, to push for a sugar tax.

Action on Sugar is chaired, as was CASH, by Graham MacGregor, Professor of Cardiovascular Medicine at Queen Mary, University of London. But it is the name of its science director, Aseem Malhotra, that might be more familiar: he wrote a controversial article in the British Medical Journal last year, arguing that saturated fat had been unfairly demonised as a cause of heart disease. Although widely aired, this view was not wholly endorsed by the British Heart Foundation: it insisted that people with the highest cholesterol were still at the greatest risk of a heart attack.

And that - in a sugar-coated nutshell - is the problem with campaigns like this. Quantifying lifestyle factors is not always straightforward. Take the **fizzy drinks** survey. It did find a correlation between estimated added sugar intake and cardiovascular disease. But the groups being compared were at opposite extremes of the consumption spectrum: either heavy or light consumers. As pointed out by David Spiegelhalter, Winton Professor of the Public Understanding of Risk at Cambridge University, "the increased hazard for moderately different groups is not that great". Cutting down on added sugar would have substantial benefits for overall public health - but the average person might not benefit that much.

These are complex nutritional messages to digest, and any anti-**sugar** lobby needs to be careful. Food and **beverage** firms will seize on the smallest uncertainty and inconsistency in the scientific evidence to preserve the profitable, teeth-rotting status quo. Campaigners will only be trusted if they play it absolutely straight.

That uncertainty also gives weak-willed consumers an excuse to keep high-sugar foods in the shopping basket - especially when it can hide behind a shocking multitude of euphemisms, such as fructose, glucose, sucrose, corn syrup, molasses and maple syrup. Remember, nature is on the side of the sugar-pushers: it is cheap to produce, seductive to the palate - which has been honed through evolution to seek out energy-rich foods - and extremely hard to replace without consumers noticing. Artificial sweeteners, in any case, may not be a risk-free alternative: there is evidence that they do not always trigger the body's satiety circuits. (In his BMJ piece, Dr Malhotra usefully revealed that low-fat yoghurts were packed with sugars.)

The campaigners' central message is sound: it is virtually certain that deriving more than 10 per cent of your calorie intake from added sugars increases the risk of obesity, diabetes and heart disease - as well as self-loathing. But it's only one of many facets of our lifestyle that we should be reflecting on. Plus, there's a cheering, bittersweet irony: added sugar is only killing us because we are not dying of something else first.

LOAD-DATE: February 6, 2014

LANGUAGE: ENGLISH

PUBLICATION-TYPE: Web Publication

JOURNAL-CODE: WEBDTNS

Copyright 2014 Telegraph Media Group Limited
All Rights Reserved

DAILY MAIL (London)

February 4, 2014 Tuesday

ONE FIZZY DRINK PER DAY RAISES HEART RISK

BYLINE: BY JENNY HOPE MEDICAL CORRESPONDENT

LENGTH: 547 words

TOO much sugar can double your risk of dying from heart disease, warn researchers.

Scientists have discovered an alarming link between excessive consumption of sugar found in **fizzy drinks** or processed food and heart-related deaths.

They found that even one **fizzy drink** a day was enough to increase the chances of dying from cardiovascular disease (CVD) by almost a third.

And for those consuming a quarter of their daily calories from sugar, the risk of heart-related death doubled.

Added sugar is that which is added in the processing of food products, rather than coming from natural sources such as fruit.

Dietary guidelines from the World Health Organisation recommend that added sugar should account for less than 10 per cent of calorie intake.

But British campaigners are calling for an upper limit of 5 per cent, along with a **sugary drinks** tax because they say sugar is the new tobacco'.

Professor Graham MacGregor, chairman of Action On Sugar, said: This is an important study.

It clearly shows a high sugar intake is associated with an increased risk of stroke and heart attacks, highlighting the need for much more focus on reducing sugar to reduce obesity and cardiovascular risk.

Not only is added sugar an unnecessary cause of calories and a cause of tooth decay, but also predisposes to strokes and heart attacks. We need to take action now.'

The study, led by Dr Quanhe Yang, from the Centers for Disease Control and Prevention in Atlanta, used US national health survey data to determine how much added sugar people were consuming.

Between 2005 and 2010

, added sugar accounted for at least 10 per cent of the calories consumed by more than 70 per cent of the US population. Around a tenth of adults got a quarter or more of their calories from added sugar.

The data was matched against heart disease mortality over a period of 14.6 years, during which 831 CVD deaths were recorded, says a report in JAMA Internal Medicine.

The risk of heart-related death was 38 per cent higher for people who consumed 17 to 21 per cent of daily calories from sugar compared with those who were under 10 per cent. It was four times higher for those getting one-third or more of calories from added sugar.

One can of sugar-sweetened drink every day increased the risk of CVD death by 29 per cent compared with drinking one can or less a week.

A single 360ml can of **fizzy drink** can contain eight teaspoons of sugar. The researchers say the extra risk is not simply because people consuming more sugar are more likely to be overweight or obese, which makes heart problems more likely.

They claim excessive sugar has an independent effect on the body which is not yet understood. It may push up blood pressure and have adverse effects on blood fats and inflammation.

The typical Briton consumes 12 teaspoons of sugar a day, and some consume as many as 46. The current WHO maximum is the equivalent of ten.

A spokesman for the sugar industry disputed that sugar causes heart disease.

Dr Glenys Jones, of Sugar Nutrition UK, said: Experts across the globe, including the World Health Organisation and UK Department of Health, have reviewed the scientific evidence and have clearly stated that the consensus of research shows that eating a diet containing added sugar does not cause heart disease.'

© Daily Mail

LOAD-DATE: February 3, 2014

LANGUAGE: ENGLISH

PUBLICATION-TYPE: Papers

Copyright 2014 Associated Newspapers Ltd.
All Rights Reserved

The Daily Telegraph

The Daily Telegraph (London)

February 4, 2014 Tuesday
Edition 2;
National Edition

Three cans of pop a day could triple risk of heart disease; Added sugars can raise heart disease risk

BYLINE: Laura Donnelly and James Edgar

SECTION: NEWS; FRONT PAGE; Pg. 1

LENGTH: 317 words

CONSUMING three cans of **fizzy drinks** every day could triple the chance of developing heart disease, a study has suggested.

Scientists in America found a strong association between the proportion of daily calories from foods laden with added sugars - - rather than those that occur naturally - - and death rates from cardiovascular disease.

For people who take a quarter of their daily calories from the sugars common in **fizzy drinks**, sweets and desserts, the researchers found the risk tripled compared with those for whom the contribution was less than 10 per cent.

The World Health Organisation recommends that added sugar should make up less than a 10th of total calorie intake. In Britain, the average adult aged between 19 and 65 consumes 1,882 calories per day, according to the Health and Social Care Information Centre. three cans of **fizzy drinks** each containing a typical 140 calories of added sugar would amount to a quarter of this .

Using US national health survey data, the study, published in the journal JAMA Internal Medicine, found that latest figures showed that about a 10th of adults had obtained a quarter or more of their calories from added sugar.

The information was matched against heart disease mortality over a typical period of 14.6 years, during which 831 cardiovascular deaths were recorded.

Prof Naveed Satta, from the British Heart Foundation Glasgow Cardiovascular Research Centre, said the study suggests that those whose diet is high in added sugars may also have an increased risk of heart attack. "Of course, sugar per se is not harmful - we need it for the body's energy needs - but when consumed in excess it will contribute to weight gain and, in turn, may accelerate heart disease."

Dr Nita Forouhi, from the Medical Research Council Epidemiology Unit at Cambridge University, suggested a health warning on **soft drinks** with high **sugar** content should be considered.

LOAD-DATE: February 4, 2014

LANGUAGE: ENGLISH

PUBLICATION-TYPE: Newspaper

JOURNAL-CODE: DTL

Copyright 2014 Telegraph Media Group Limited
All Rights Reserved

The Express

February 4, 2014 Tuesday
Edition 3;
National Edition

Sugary treats can triple heart disease death risk

BYLINE: Louise Sassoon

SECTION: NEWS; Pg. 25

LENGTH: 265 words

TOO many sugar-laden sweets, desserts and drinks can triple your chances of dying from a heart condition, a study reveals.

Scientists have discovered a significant link between the two, with just one sugar-sweetened drink a day increasing the risk of cardiovascular disease.

The latest research, led by Dr Quanhe Yang from the Centres for Disease Control and Prevention in Atlanta, used United States national health survey data to determine how much added sugar people were consuming.

Between 2005 and 2010, added sugar accounted for at least 10 per cent of the calories consumed by more than 70 per cent of the US population.

Around one tenth of adults obtained a quarter or more of their calories from added sugar.

The figures were matched against heart disease mortality over a typical period of 14.6 years.

For people obtaining a quarter of their calories from added sugar, the risk tripled compared with those whose sugar contribution was less than 10 per cent.

The authors, writing in the journal JAMA Internal Medicine, concluded: "A higher percentage of calories from added sugar is associated with significantly increased risk of CVD mortality."

Dietary guidelines from the World Health Organisation recommend added sugar should make up less than 10 per cent of calorie intake.

But many processed foods and **beverages** are packed with **sugar**.

A single can of **fizzy drink**, for example, may contain 35 grams of sugar providing 140 calories.

British expert Dr Nita Forouhi, from Cambridge University, called for "clear front of pack labelling of sugar content" to help consumers buying food products.

LOAD-DATE: February 4, 2014

LANGUAGE: ENGLISH

GRAPHIC: Food laden with sugar can reduce life expectancy

PUBLICATION-TYPE: Newspaper

JOURNAL-CODE: DXP

Copyright 2014 Express Newspapers
All Rights Reserved

Metro (UK)

February 4, 2014 Tuesday
Edition 1;
Scotland

Kick the can or kick the bucket?

BYLINE: AIDAN RADNEDGE

SECTION: NEWS; FRONT PAGE, TEASERS; Pg. 1

LENGTH: 278 words

MILLIONS of people put their lives on the line by drinking as little as one can of **sugary drink** a day, researchers are warning.

They fail to realise they are risking their health because of the amount of added **sugar** used to sweeten **soft drinks**.

Just one can contains as much as the recommended daily intake and is assoc iated with an increased chance of having a deadly heart attack.

Yet the researchers have found most people were getting more than a tenth of their daily calories from added sugar and one in ten was getting a quarter or more.

Experts in Britain said the findings strengthened calls for prominent health warnings on packaging and consideration of new taxes on highsugar drinks. Dr Nita Forouhi, from the University of Cambridge, said the study added 'to the mounting evidence for the many health risks of the high consumption of added **sugars'**. She added: '**Sugar** sweetened **beverages** and fruit drinks represent the "low hanging fruit" for first wave of pub lic and policy action.' The researchers found the average American gets 14.9 per cent of their calo ries each day from added sugar, half as much again as the World Health Organi sation guidelines suggest.

The risk of dying from cardiovascular disease notably rises when intake is above 15 per cent - equivalent to drinking one can of **fizzy drink** in a 2,000calorie daily diet, the US team noted.

Here, the **soft drinks** industry scrambled to refute the claims last night, insisting there was no proven link between added **sugar** and heart disease.

British **Soft Drinks** Association direc torgeneral Gavin Partington said: '**Soft drinks** manufacturers have led the way in reducing **sugar** in their products.'

LOAD-DATE: February 4, 2014

LANGUAGE: ENGLISH

PUBLICATION-TYPE: Newspaper

London Evening Standard
standard.co.uk

February 4, 2014 Tuesday 10:40 AM GMT

Sugar 'raises heart attack risk'

SECTION: PA NEWS FEEDS

LENGTH: 784 words

Consuming too many sugary sweets, desserts and drinks can triple your chances of dying from heart disease, a study has shown.

Scientists in the US found a striking association between the proportion of daily calories supplied by sugar-laden foods and heart disease death rates.

One **sugar**-sweetened **beverage** a day was enough to increase the risk of dying from cardiovascular disease (CVD) affecting the heart and arteries.

For people obtaining a quarter of their calories from added sugar, the risk tripled compared with those whose sugar contribution was less than 10%.

Sugar consumption in the top fifth of the range studied doubled the likelihood of death from heart disease.

The researchers specifically focused on added sugar in the diet - that is, sugar added in the processing or preparing of food, rather than natural sources.

Dietary guidelines from the World Health Organisation recommend that added sugar should make up less than 10% of total calorie intake.

Yet many processed foods and **beverages** are packed with **sugar**. A single can of **fizzy drink**, for instance, may contain 35 grams of sugar providing 140 calories.

The new study, led by Dr Quanhe Yang, from the Centres for Disease Control and Prevention in Atlanta, used US national health survey data to determine how much added sugar people were consuming.

Between 2005 and 2010, added sugar accounted for at least 10% of the calories consumed by more than 70% of the US population, the research showed.

Around a tenth of adults obtained a quarter or more of their calories from added sugar.

The data were matched against heart disease mortality over a typical period of 14.6 years, during which a total of 831 CVD deaths were recorded.

Writing in the journal JAMA Internal Medicine, the authors concluded: "Our findings indicate that most US adults consume more added sugar than is recommended for a healthy diet.

"A higher percentage of calories from added sugar is associated with significantly increased risk of CVD mortality."

Commenting on the results in the journal, Dr Laura Schmidt from the University of California at San Francisco, wrote: "We are in the midst of a paradigm shift in research on the health effects of sugar, one fuelled by extremely high rates of added sugar overconsumption in the American public.

"In sum, the study by Yang et al contributes a range of new findings to the growing body of research on sugar as an independent risk factor in chronic disease."

British expert Dr Nita Forouhi, from the Medical Research Council Epidemiology Unit at Cambridge University, called for "clear front of pack labelling of sugar content" to help consumers buying food products.

She added: "While policy makers deliberate on the pros and cons of a **sugary drinks** tax, there is a public health action less talked about: a health warning on **soft drinks** with high **sugar** content, recommending to limit consumption as part of a healthy diet."

Professor Naveed Satta, from the British Heart Foundation Glasgow Cardiovascular Research Centre at the University of Glasgow, said: " We have known for years about the dangers of excess saturated fat intake, an observation which led the food industry to replace unhealthy fats with presumed 'healthier' sugars in many food products.

"However, the present study, perhaps more strongly than previous ones, suggests that those whose diet is high in added sugars may also have an increased risk of heart attack. Of course, sugar per se is not harmful - we need it for the body's energy needs - but when consumed in excess it will contribute to weight gain and, in turn, may accelerate heart disease."

He added: "Helping individuals cut not only their excessive fat intake, but also refined sugar intake, could have major health benefits including lessening obesity and heart attacks. The first target, now taken up by an increasing number of countries, is to tax sugar-rich drinks."

The study showed that the average proportion of daily calories obtained from added sugar rose from 15.7% in 1988-1994 to 16.8% in 1999-2004. It decreased to 14.9% in 2005-2010.

Gavin Partington, director general of the British **Soft Drinks** Association, said: "**Soft drinks** manufacturers have led the way in reducing **sugar** in their products - companies with market share of around 60% are now signed up to the Responsibility Deal calorie reduction pledge.

"Furthermore, soft drinks manufacturers are committed to providing on-pack nutritional information, wider availability of smaller portion sizes and have also substantially increased their advertising and marketing expenditure on low and zero calorie drinks.

"It is important to remember that soft drinks contribute just 2% of calories to the average British diet."

LOAD-DATE: February 4, 2014

LANGUAGE: ENGLISH

PUBLICATION-TYPE: Newspaper; Web Publication

JOURNAL-CODE: WEBES

Copyright 2014 Evening Standard Limited
All Rights Reserved

THE TIMES

The Times (London)

February 4, 2014 Tuesday
Edition 1;
National Edition

Sugary drinks linked to increased risk of fatal heart attack

BYLINE: Chris Smyth

SECTION: NEWS; Pg. 14

LENGTH: 127 words

People who have a **sugary drink** every day are 30 per cent more likely to suffer a fatal heart attack than those who drink one a week **Fizzy drinks** account for 37 per cent of added sugar in American diets, researchers from the Centres for Disease Control in Atlanta, Georgia, said. British experts said the study added to evidence of the risks of a high-sugar diet, suggesting such drinks should be first in line for taxes or health warnings. The World Health Organisation advises people to get no more than 10 per cent of their calories from added sugar, but 71 per cent of those studied consumed more. Professor Naveed Sattar, of the University of Glasgow, said "To ignore the mounting evidence for the adverse health effects of excess sugar intake would seem unwise."

LOAD-DATE: February 4, 2014

LANGUAGE: ENGLISH

PUBLICATION-TYPE: Newspaper

JOURNAL-CODE: TIM

Copyright 2014 Times Newspapers Limited
All Rights Reserved

DAILY MAIL (London)

February 3, 2014 Monday

HOW FOOD GIANTS WOO MINISTERS

BYLINE: BY DANIEL MARTIN

LENGTH: 787 words

THE food industry lobby has been given unprecedented access to the heart of government, a Daily Mail investigation has found.

Fast food companies, supermarkets, restaurant chains and chocolate and **fizzy drinks** firms have had dozens of meetings with ministers.

Yet health campaigners say they have been shunned - at a time when the Coalition has been resisting calls for tough laws to restrict the amount of sugar in food.

McDonald's, Mars, Pepsi, Nando's and Tesco are among firms invited to ministerial meetings since the 2010 election.

The Food and Drink Federation, the lobbyist for the food industry which has led opposition to calls for anti-obesity legislation, has had 16 meetings with ministers and 99 meetings with officials since the Coalition took over, official figures reveal.

Last night public health experts said the extraordinary access showed that the Government was keener on listening to the food industry than to those with the interest of people's health at heart.

Tam Fry, of the National Obesity Forum, said: These meetings are an example of how the industry has a charmed route into the corridors of power that is denied to everyone else. The fear of people like us is that

the Government secretly sews things up on the industry's behalf. Perhaps if the Government weren't so influenced by

them, we would have a maximum sugar level in foods by now.'

Campaigners say that without tough action to tackle obesity, which can lead to diabetes, heart disease, stroke and cancer, the NHS could go bankrupt.

The revelation comes weeks after it emerged that drinks industry representatives met dozens of ministers and officials before the Government's decision to drop plans for a minimum alcohol pricing.

Today a study published in the Bulletin of the World Health Organisation says there is a correlation between countries with few regulations on food production and those with consumption of unhealthy fast foods.

Researchers say that if governments take action, such as stopping the clustering of fast food outlets on high streets or taxing **fizzy drinks**, they can help turn the tide against obesity.

Campaign group Action On Sugar, which wants laws to force manufacturers to cut the sugar content of their products by up to 30 per cent, will tonight have its first meeting with Health Secretary Jeremy Hunt, but hopes of a breakthrough are not high.

Its chairman Graham MacGregor, a professor of cardiovascular medicine who has also led anti-salt campaigns, said: We rarely get access to ministers - they don't want to see us. My impression is that if the food industry want to see them, they get in. The food industry is riding all over us. It's a scandal.

I don't know what Jeremy Hunt will say, but I don't think it will be positive.' Last month Action On Sugar unveiled research showing that zero-fat yoghurts can contain five teaspoons of sugar, while a can of Heinz tomato soup has four.

The Academy of Medical Royal Colleges has called for a 20 per cent tax on food and drink which has high sugar levels.

But ministers have so far rejected all calls for restrictions or taxes on sugar content.

The Coalition instead wants companies to agree a voluntary cut sugar in levels. The Department of Health has published details of ministerial meetings between April 2011 and September 2013 on its website but for unknown reasons was unable to do so for the period from May 2010 to March 2011.

The details show that big food firms met ministers on average once a month.

Labour's public health spokesman Luciana Berger said: People will rightly ask why ministers have spent so much time meeting with the fast food and drink industry. Ministers caved in to vested interests. David Cameron has no plan to tackle the obesity epidemic.'

Public health campaigners also accuse the Coalition of hampering action to target obesity by its decision to strip responsibility for nutrition from the Food Standards Agency and place it under the political control of the Department of Health.

Last night the Department of Health said the meetings with the food industry were about trying to persuade it to improve the healthiness of products.

Improving the public's health is a priority,' a spokesman said. We are not giving business, big or small, power over public health policy but food businesses have a big role to play in helping people to lead a healthier life.'

Terry Jones, spokesman for the Food and Drink Federation, said: The significant commitments developed at those meetings to eliminate artificial trans fats and reduce the saturated fat, salt and calorie content of a wide range of foods as well as providing clear nutrition labelling, have empowered consumers to make dietary choices appropriate to their lifestyle.'

© Daily Mail

LOAD-DATE: February 2, 2014

LANGUAGE: ENGLISH

PUBLICATION-TYPE: Papers

Copyright 2014 Associated Newspapers Ltd.
All Rights Reserved

239 of 277 DOCUMENTS

theguardian
Guardian.com.

February 3, 2014 Monday

Coca-Cola's America Is Beautiful ad: why liberals should be upset

BYLINE: Jill Filipovictheguardian.com

LENGTH: 1349 words

ABSTRACT

Jill Filipovic: Coke's Super Bowl ad tugged at our heartstrings with its diverse US portrait, but the goal is to get minorities hooked on soda

FULL TEXT

My favorite Super Bowl commercial last night was Coca-Cola's #AmericaIsBeautiful. The spot, shot with an Instragram-filter aesthetic, featured a multi-lingual rendition of "America the Beautiful" sung by children and illustrated with a diversity of New Americana scenes: a cowboy riding his horse, kids at the movies, teenagers on surfboards at dawn and breakdancing at dusk, headscarf-wearing young women buying food from a cart in Chinatown, two men in yarmulkes looking upon the newly-built Freedom Tower, a same-sex couple roller skating and hugging their daughter. I admit it, I teared up a bit. And I braced myself for the predictable right-wing outrage. But perhaps those of us who care about inequality and racism should be angry, too.

Coca-Cola's diversity ad wasn't purposed just to celebrate the reality of a multi-ethnic America. It was to sell soda to rapidly-expanding but vulnerable populations, even if that means contributing to serious health problems, exploiting divides in class and education, and exacerbating racial inequality. The genius of the Coca-Cola company is that they made the racial aspect of soda marketing work in their favor with this ad. Conservative indignation came immediately, and Twitter exploded with objections to the spot. "We speak ENGLISH here, IDIOTS" pretty much sums up the complaints. Twitter, of course, is a great democratizer, and it's easy to find some idiot saying just about anything. But right wing politicians and media got on board, too. Former GOP Congressman Allen West called the commercial "truly disturbing", and opined:

If we cannot be proud enough as a country to sing American the Beautiful in English in a commercial during the Super Bowl, by a company as American as they come - doggone we are on the road to perdition.

One writer on the conservative website Breitbart.com said Coke used an iconic song to "push multiculturalism down our throats", promoting a scenario in which "the United States of America is no longer a nation ruled by the Constitution and American traditions in which English is the language of government." It's easy to laugh at conservative bigotry and historical ignorance. The United States has always been a multi-lingual country. English itself was an important from colonizers. Spanish has long been the predominant language in many parts of the United States, and many Spanish-speaking folks became

Americans because the borders moved, not because they did. Most Americans today are the descendants of immigrants who certainly did not speak the languages native to the piece of land on which we now reside. And when you see someone get mad at multi-lingualism, it's obvious you're watching a racist fly their flag. Coca-Cola knew exactly what it was doing with this commercial. It knew it would inflame white conservatives, but, more importantly, it knew the commercial would align Coke with Latinos and other quickly-growing groups in the United States. So Coke expands its market share and promotes its product while endorsing a vision of a diverse, multi-cultural America. What's the harm? Unfortunately, the harm lands squarely on the bodies of kids and families with few resources. Educated, affluent white Americans are drinking less soda than they were a few years ago, and **soft drink** makers now rely largely on "heavy users" - those who drink several sodas every day - to keep their businesses booming. Heavy users tend to be in lower-income areas - places New Orleans, Louisiana and Rome, Georgia. Coke is trying to expand that model. Long dominant in Latin America - that region is Coke's second-largest market - the company has been trying to capture the Latino market in the United States through target marketing. That is, of course, how businesses operate. But Coca-Cola's model depends on consumers who drink significantly more soda than average - a habit that comes with a series of serious health consequences - and on targeting children, who will (ideally) be life-long Coke drinkers. Expanding populations mean new consumers. American soda companies expanded abroad decades ago, and Coke has been especially aggressive at marketing its products to lower-income consumers who have enough extra cash to spend on a **sugary** indulgence. A crisis of conscience at his role in expanding Coke into impoverished Brazilian *favelas*caused one Coca-Cola executiveto try and reign in the company's practices; he was fired for his efforts. Coke has long been successful in Mexico, where it operates its largest independent bottling plant. That country is not only the second-highest soda consumer in the world, right behind the United States, but now has the world's highest obesity rates (sinking the US to number two). In response to serious public health issues driven by soda consumption, Mexico recentlyimplemented a plan to tax soda. Soda companies have launched a large-scale offensive against both the tax and any criticisms of soda. In the United States, efforts at securing more "heavy users" are especially pernicious when directed at Latino communities. One in four Latino households in the US is food insecure, compared to one in 10 white households. Of the top 10 US counties with the highest rates of food insecurity, nine are predominantly Latino. Malnutrition rates aretwice as high among Hispanic children as non-Hispanic children in the United States. Hispanic children are also more likely to be overweight or obese. Nearly 12% of Hispanic adults have diagnosed diabetes - by comparison, only 7% of non-Hispanic white Americans have diagnosed diabetes. Within Mexican-American and Puerto Rican populations, diabetes rates climb above 13%. African Americans, also target "heavy user" consumers for soda companies, have diabetes rates that hover around 12%. Perhaps most disturbingly, younger Latinos face higher rates of developing diabetes than any other group: Latina girls born in 2000 have a more than 50% chance of developing the disease in their lifetime. Marketing to low-income and of-color populations works. In one study focused on New York City, researchers found that the proportion of African-Americans, Puerto Ricans, Mexicans and Mexican-Americans who drank more than one soda every day was more than twice the proportion of whites. People living in households with income 200% of the poverty line or below were more likely to be regular soda drinkers than people in wealthier households. African-American New Yorkers were more than three times as likely as whites to drink soda frequently; Mexican-Americans were 2.9 times as likely, and Puerto Ricans were 2.4. It makes sense: Soda is a cheap treat that also provides energy and calories. It's accessible just about anywhere. You can drink it just about anywhere. A low-wage worker needing to fill their belly or a kid looking for something tasty or a mom looking to treat her kids and feed herself on the run don't have to go further than the drive-thru or corner bodega, and doesn't have to invest much, in picking up a **soft drink**. But that doesn't mean we should be applauding soda companies, even if their ads tug at our heartstrings and our liberal values. It means consumers should have more affordable options, corporate advertising of unhealthy food should be regulated more tightly and Americans should be collectively enraged at our obscenely low wages and lack of a comprehensive social safety net - the things that create unhealthy, perverse incentives for consumers. It means we should cast a critical eye when soda companies fly the flag of diversity, when, in fact, their product contributes tostark racial inequalities. Coke's targeting of Latino and other immigrant populations is about as progressive as RJ Reynolds marketing menthol cigarettes to African-Americans or Phillip Morris hawking Virginia Slims to women - that is, not very. Before we applaud Coke's advertising diversity, we should ask: do we really want Coke to diversify?

LOAD-DATE: February 3, 2014

LANGUAGE: ENGLISH

PUBLICATION-TYPE: Newspaper

Copyright 2014 Guardian News and Media Limited or its affiliated companies
All Rights Reserved
Registered in England and Wales. No. 908396
Registered office: PO Box 68164, Kings Place, 90 York Way, London N245 of 277 DOCUMENTS

MailOnline

February 3, 2014 Monday 11:04 PM GMT

One fizzy drink per day raises heart risk: And sugary diet could double the chance of death

BYLINE: JENNY HOPE

SECTION: HEALTH

LENGTH: 595 words

- One **fizzy drink** a day increases chance of dying from cardiovascular disease by a third, scientists say
- Those whose diet is 25 per cent sugar double change of heart-related death
- Comes as campaigners compare sugar to tobacco and call for a sugar tax

Too much sugar can double your risk of dying from heart disease, warn researchers.

Scientists have discovered an alarming link between excessive consumption of sugar found in **fizzy drinks** or processed food and heart-related deaths.

They found that even one **fizzy drink** a day was enough to increase the chances of dying from cardiovascular disease (CVD) by almost a third.

And for those consuming a quarter of their daily calories from sugar, the risk of heart-related death doubled.

Added sugar is that which is introduced to the processing of food products, rather than coming from natural sources such as fruit.

Dietary guidelines from the World Health Organisation recommend that added sugar should account for less than 10 per cent of calorie intake.

But British campaigners are calling for an upper limit of 5 per cent, along with a **sugary drinks** tax because they say sugar is the 'new tobacco'.

Professor Graham MacGregor, chairman of Action On Sugar, said: 'This is an important study.

'It clearly shows a high sugar intake is associated with an increased risk of stroke and heart attacks, highlighting the need for much more focus on reducing sugar to reduce obesity and cardiovascular risk.

'Not only is added sugar an unnecessary cause of calories and a cause of tooth decay, but also predisposes to strokes and heart attacks. We need to take action now.'

The study, led by Dr Quanhe Yang, from the Centers for Disease Control and Prevention in Atlanta, used US national health survey data to determine how much added sugar people were consuming.

Between 2005 and 2010, added sugar accounted for at least 10 per cent of the calories consumed by more than 70 per cent of the US population. bout a tenth of adults got a quarter or more of their calories from added sugar, says a report in JAMA Internal Medicine.

The data was matched against heart disease mortality over a period of 14.6 years, during which 831 CVD deaths were recorded in the study group which was representative of the population.

The risk of heart-related death was 38 per cent higher for people who consumed 17 to 21 per cent of daily calories from sugar compared with those who were under 10 per cent. It was four times higher for those getting one-third or more of calories from added sugar.

One can of sugar-sweetened drink every day increased the risk of CVD death by 29 per cent compared with drinking one can or less a week. A 360ml can of **fizzy drink** can contain eight teaspoons of sugar.

The researchers say the extra risk is not simply because people consuming more sugar are more likely to be overweight or obese, which makes heart problems more likely.

They claim excessive sugar has an independent effect on the body which is not yet understood. It may push up blood pressure and have adverse effects on blood fats and inflammation.

The typical Briton consumes 12 teaspoons of sugar a day, and some consume as many as 46. The current WHO maximum is the equivalent of ten.

A sugar industry spokesman disputed the heart disease claim. Dr Glenys Jones, of Sugar Nutrition UK, said: 'Experts across the globe, including the World Health Organisation and UK Department of Health, have reviewed the scientific evidence and have clearly stated that the consensus of research shows that eating a diet containing added sugar does not cause heart disease.'

LOAD-DATE: February 3, 2014

LANGUAGE: ENGLISH

PUBLICATION-TYPE: Web Publication

JOURNAL-CODE: WEBDM

Copyright 2014 Associated Newspapers Ltd.
All Rights Reserved

1P 2AP

MailOnline

February 3, 2014 Monday 12:18 AM GMT

How food giants sweet talk ministers: Sugar campaigners' fears over 'secret stitch-up' meetings

BYLINE: DANIEL MARTIN

SECTION: NEWS

LENGTH: 834 words

- Daily Mail investigation finds food industry let in to the heart of government
- Meanwhile health campaigners say they have been shunned by the Coalition
- McDonald's, Pepsi, Mars, Nando's and Subway met with ministers
- Critics say the relationship is getting in the way of health legislation

The food industry lobby has been given unprecedented access to the heart of government, a Daily Mail investigation has found.

Fast food companies, supermarkets, restaurant chains and chocolate and **fizzy drinks** firms have had dozens of meetings with ministers.

Yet health campaigners say they have been shunned - at a time when the Coalition has been resisting calls for tough laws to restrict the amount of sugar in food.

McDonald's, Mars, Pepsi, Nando's and Tesco are among firms invited to ministerial meetings since the 2010 election.

The Food and Drink Federation, the lobbyist for the food industry which has led opposition to calls for anti-obesity legislation, has had 16 meetings with ministers and 99 meetings with officials since the Coalition took over, official figures reveal.

Last night public health experts said the extraordinary access showed that the Government was keener on listening to the food industry than to those with the interest of people's health at heart.

Tam Fry, of the National Obesity Forum, said: 'These meetings are an example of how the industry has a charmed route into the corridors of power that is denied to everyone else.

'The fear of people like us is that the Government secretly sews things up on the industry's behalf. Perhaps if the Government weren't so influenced by them, we would have a maximum sugar level in foods by now.'

Campaigners say that without tough action to tackle obesity, which can lead to diabetes, heart disease, stroke and cancer, the NHS could go bankrupt.

The revelation comes weeks after it emerged that drinks industry representatives met dozens of ministers and officials before the Government's decision to drop plans for a minimum alcohol pricing.

Today a study published in the Bulletin of the World Health Organisation says there is a correlation between countries with few regulations on food production and those with consumption of unhealthy fast foods.

Researchers say that if governments take action, such as stopping the clustering of fast food outlets on high streets or taxing **fizzy drinks**, they can help turn the tide against obesity.

Campaign group Action On Sugar, which wants laws to force manufacturers to cut the sugar content of their products by up to 30 per cent, will tonight have its first meeting with Health Secretary Jeremy Hunt, but hopes of a breakthrough are not high.

Its chairman Graham MacGregor, a professor of cardiovascular medicine who has also led anti-salt campaigns, said: 'We rarely get access to ministers - they don't want to see us.

'My impression is that if the food industry want to see them, they get in. The food industry is riding all over us. It's a scandal.

'I don't know what Jeremy Hunt will say, but I don't think it will be positive.' Last month Action On Sugar unveiled research showing that zero-fat yoghurts can contain five teaspoons of sugar, while a can of Heinz tomato soup has four.

The Academy of Medical Royal Colleges has called for a 20 per cent tax on food and drink which has high sugar levels.

But ministers have so far rejected all calls for restrictions or taxes on sugar content.

The Coalition instead wants companies to agree a voluntary cut sugar in levels. The Department of Health has published details of ministerial meetings between April 2011 and September 2013 on its website but for unknown reasons was unable to do so for the period from May 2010 to March 2011.

The details show that big food firms met ministers on average once a month.

Labour's public health spokesman Luciana Berger said: 'People will rightly ask why ministers have spent so much time meeting with the fast food and drink industry. Ministers caved in to vested interests. David Cameron has no plan to tackle the obesity epidemic.'

Public health campaigners also accuse the Coalition of hampering action to target obesity by its decision to strip responsibility for nutrition from the Food Standards Agency and place it under the political control of the Department of Health.

Last night the Department of Health said the meetings with the food industry were about trying to persuade it to improve the healthiness of products.

'Improving the public's health is a priority,' a spokesman said. 'We are not giving business, big or small, power over public health policy but food businesses have a big role to play in helping people to lead a healthier life.'

Terry Jones, spokesman for the Food and Drink Federation, said: 'The significant commitments developed at those meetings to eliminate artificial trans fats and reduce the saturated fat, salt and calorie content of a wide range of foods as well as providing clear nutrition labelling, have empowered consumers to make dietary choices appropriate to their lifestyle.'

LOAD-DATE: February 3, 2014

LANGUAGE: ENGLISH

PUBLICATION-TYPE: Web Publication

JOURNAL-CODE: WEBDM

Copyright 2014 Associated Newspapers Ltd.
All Rights Reserved

The Telegraph
telegraph.co.uk

February 3, 2014 Monday 9:00 PM GMT

Three fizzy drinks per day could triple chance of heart disease; Strong association between the proportion of daily calories from sugar-laden foods and death rates from cardiovascular disease, report finds

BYLINE: By James Edgar

SECTION: NEWS

LENGTH: 484 words

Drinking just three cans of fizzy pop each day could triple your chance of developing heart disease, a study has suggested.

Scientists in America found a strong association between the proportion of daily calories from foods laden with added sugars - rather than those that occur naturally - and death rates from cardiovascular disease.

For people who take on a quarter of their calories each day from the sugars common in sticky drinks, sweets and desserts, the researchers found the risk tripled compared to those whose sugar contribution was less than 10 per cent.

Dietary guidelines from the World Health Organisation recommend added sugar should make up less than a tenth of total calorie intake, yet many processed foods and **beverages** are packed with **sugar**.

In Britain the average adult between 19 and 65 takes on 1,882 calories per day, according to the The Health and Social Care Information Centre, so three cans of **fizzy drinks** each containing 140 of added sugar would amount to around a quarter.

The new study, published in the journal JAMA Internal Medicine, used American national health survey data to determine how much added sugar people were consuming. Between 2005 and 2010 it accounted for at least 10 per cent of the calories consumed by more than 70 per cent of the US population, the research showed.

Around a tenth of adults obtained a quarter or more of their calories from added sugar.

The information was matched against heart disease mortality over a typical period of 14.6 years, during which a total of 831 cardiovascular deaths were recorded.

Professor Naveed Satta from the British Heart Foundation Glasgow Cardiovascular Research Centre at the University of Glasgow said: "We have known for years about the dangers of excess saturated fat intake, an observation which led the food industry to replace unhealthy fats with presumed 'healthier' sugars in many food products.

"However, the present study, perhaps more strongly than previous ones, suggests that those whose diet is high in added sugars may also have an increased risk of heart attack. Of course, sugar per se is not harmful - we need it for the body's energy needs - but when consumed in excess it will contribute to weight gain and, in turn, may accelerate heart disease."

Dr Nita Forouhi from the Medical Research Council Epidemiology Unit at Cambridge University, called for "clear front of pack labelling of sugar content" to help consumers buying food products.

"While policy makers deliberate on the pros and cons of a **sugary drinks** tax, there is a public health action less talked about: a health warning on **soft drinks** with high **sugar** content, recommending to limit consumption as part of a healthy diet," she said.

The study showed that the average proportion of daily calories obtained from added sugar rose from 15.7 per cent in 1988-1994 to 16.8 per cent in 1999-2004. It decreased to 14.9 per cent in 2005-2010.

LOAD-DATE: February 4, 2014

LANGUAGE: ENGLISH

PUBLICATION-TYPE: Web Publication

JOURNAL-CODE: WEBDTNS

Copyright 2014 Telegraph Media Group Limited
All Rights Reserved

The Observer (England)

February 2, 2014

Food crusaders' new challenge: cut sugar to save NHS £ 50bn a year: The campaigners who helped reduce salt intake in British diets by 15% in seven years have a fresh target in sight. But they fear this struggle will be much tougher - and talks with the health secretary this week could be crucial: THE DOLCE METER: SUGAR LEVELS IN EVERYDAY FOODS

BYLINE: Barry Neild

SECTION: OBSERVER HOME NEWS PAGES; Pg. 20

LENGTH: 999 words

For a group of academics who spent over a decade pushing food manufacturers to use less salt, success has been so sweet they have decided to take on a new challenge: sugar.

But by opening a new front in their campaign to prevent a health crisis, they say they face a bitter struggle. This time an unappetising blend of government reluctance, vested business interests and fragmented industry regulation lies in their way.

Action on Sugar was launched last month. At a time when many people were defaulting on new year pledges to improve their diets, the organisation said the burden of cutting our sugar intake by up to 40% over the next four years should instead be placed on Britain's food and drink manufacturers. The group will meet Jeremy Hunt, the health secretary, this week to try to persuade him to sign up to their cause.

The health and nutrition experts behind the campaign say that unless Hunt agrees, rising levels of obesity and type 2 diabetes could cost the country up to £ 50bn a year - more than half of NHS England's current budget.

"It is very difficult to argue with what we're saying," says Graham MacGregor, a professor of cardiovascular medicine and the chairman of Action on Sugar. "Human beings don't need to eat added sugar. It was never a feature of a mammalian diet, because we couldn't get it."

Some academics have controversially likened sugar to addictive drugs such as tobacco or cocaine and accuse the food industry of cynically hooking children and parents on junk food to maximise profits.

Action on Sugar has avoided similar comparisons, instead publishing data that shows how much sugar is ladled into our diets. A tall Starbucks caramel Frappuccino contains 11 teaspoons of sugar - barely two spoons under the recommended daily intake for women. Coca-Cola or Pepsi contains nine per can and a bowl of Kellogg's Frosties has four.

In raising the issue, MacGregor and his team want to replicate the achievements of their earlier campaign, Consensus Action on Salt and Health (Cash). Largely thanks to Cash, our salt intake in foods such as bread, cereals and meat has fallen by 15% in the last seven years, contributing to a reduction in strokes, heart attacks and cases of high blood pressure.

This cutback, done incrementally so that few noticed any difference in taste, was championed by the Food Standards Agency, the government's food safety body. It drew up reduction timetables and even funded research into how bakers could reduce salt levels without ruining their loaves.

Action on **Sugar** argues that the same gradual reductions could be applied to added **sugar** with little impact on taste. It says consumers would benefit from improved diets, the government would curb future healthcare costs and manufacturers would save on ingredient costs. "Reformulation is a win-win," says nutritionist Katharine Jenner, the group's campaign director. She says recent pledges by Tesco to cut **sugar** in its **soft drinks** and a reduction in the size of Mars and Snickers bars (albeit without a corresponding price drop), shows it can happen.

But, she says, though many food producers may be willing in principle, in reality they are loth to sign up to a voluntary "responsibility deal" on calorie reduction introduced in 2012 by Andrew Lansley, the Conservative former health secretary, because it could damage their market position.

At the root of this problem, says MacGregor, is a 2012 decision to strip responsibility for nutrition from the Food Standards Agency, placing it instead under the political control of the Department of Health under Lansley's successor, Hunt.

MacGregor is scornful of the Conservatives' commitment to reducing sugar and believes changes in the political landscape since the 1990s launch of the anti-salt campaign mean his new project could face strong resistance, much of it driven by a powerful food industry "sugar lobby". "Lansley's responsibility deal is completely bonkers," he says. "Making the food industry responsible for policing itself is a joke."

He dismisses Tesco's promise as having negligible impact. "Tesco's soft drinks account for about 0.5% of the market. What we want is that reduction applied across every single sweet and soft drink that is marketed in the UK, and then we'll see a reduction.

"It's got to be Coca-Cola, Schweppes, every supermarket, everything. And if you do it slowly, and people get used to it and they prefer the drink with less sugar, then you can do it again. This is how the salt thing worked. Do it slowly: incremental reductions, incremental targets."

The Department of Health says it is continuing to discuss the issue with the food industry and is "willing to go further still", but its policy is aimed at fighting obesity by helping people eat fewer calories, of which sugar is only one source. It says 38 firms have signed up to its deal.

Sugar Nutrition UK, a research body funded by sugar manufacturers, disputes Action on Sugar's medical claims. It says sugar consumption is not a cause of diabetes and cannot solely be blamed for obesity. "It is simply not right to say that reducing the amount of sugar in foods will always result in a reduction of calories,"

it said. "In most cases the sugar will need to be replaced by another ingredient and the reformulated recipes can contain more calories than the original."

Barbara Gallani of the Food and Drink Federation, which represents manufacturers, says more research is needed into how consumers react to new recipes that mean changes to taste, texture and ingredients.

Despite the resistance, MacGregor says Action on Sugar will achieve its goals, even if it has to wait for a change of government. His group has already planned talks with Labour and the Liberal Democrats to persuade them to consider a possible "sugar tax" to enforce blanket reductions.

"If Jeremy Hunt agrees to do this, which is very unlikely, then obviously we'll put them on the back burner. But we do get things done. We will get sugar down the way we've got salt down."

LOAD-DATE: February 1, 2014

LANGUAGE: ENGLISH

PUBLICATION-TYPE: Newspaper

Copyright 2014 Guardian Newspapers Limited
All Rights Reserved

Sunday Express

February 2, 2014
Edition 1;
National Edition

End this diet of state nannying

BYLINE: Kirsty Buchanan

SECTION: EDITORIAL; OPINION, COLUMNS; Pg. 38

LENGTH: 885 words

MY COUNCIL seems to spend a good deal of its time and my money policing the contents of my son's school lunchbox. Under the tax payerfunded Healthy Bor ough initiative, which aims to cut obesity levels among residents of Tower Hamlets, East London, school staff are obliged to open pupils' lunchboxes for inspection.

To be treated like a healthy food halfwit who would stuff my son full of **fizzy drinks,** crisps and chocolate were it not for the paternalistic auspices of my meddlesome council would be irritating enough but the programme makes no sense.

Buttery croissants are fine and stodgy sausage rolls pass muster but salted popcorn is a nono.

Crisps are banned but jammy dodgers or sugartopped jam roll are fine. Pure orange juice is forbidden but children's yogurts, which are 15 per cent sugar, are applauded.

Ultimately it is the sheer poundwasting pointlessness of it all that bothers me. Unless the council wants to start kicking in the doors of parents' homes to see what its young citizens are getting fed for breakfast and dinner, a couple of carrot sticks at school is a tickbox exercise in futility. While New Labour was hellbent in inter vening in every part of our lives, David Cameron's "small state, big society" mantra carried an expectation of more personal responsibility and less "nanny state" meddling. Yet despite endless exhortations for us to take more res ponsibility, Nanny State Britain is alive and www.well.No winter trip on

public transport would be complete without an automated voice warning us to mind slippery surfaces, even though we managed to negotiate the slip pery walk to the station without a pratfall. When it is hot messages on trains remind us to carry water and our doctors' surgeries and libraries are stuffed with leaflets urging us to take more exercise and eat more fruit and veg.

IF THERE is a person alive who does not know that an apple is better for them than a chocolate cake I have yet to meet them but knowledge and choice are not the same thing. Time and again the responsible must be patronised or punished along with the stupid or stubborn... and it is not just councils who are at it.

In this Parliament ministers have considered bringing in a minimum unit price for alcohol to combat binge-drinking, so penalising the moderate imbiber along with the antisocial gutter hugger.

Proposals to slap "fat taxes" on food surface regularly, even though a similar scheme in Norway was ditched after its sole result was to push up food prices for all struggling families.

Meanwhile a Uturn on plain packaging on cigarettes means this too is now being seriously considered despite concerns it will be a blessing for criminal gangs cashing in with counterfeit cigarettes. There are many reasons why youngsters take up smoking but I doubt any of them include the lure of a shiny fag packet.

Now comes the Government's decision to allow MPs a free vote on banning smoking in cars carrying children. The Lords last week backed a Labour amend ment to the Children and Families Bill to protect youngsters with this ban and the free vote on the amendment will take place in the Commons next month. If passed it will empower, though not compel, the Gov ernment to make it a criminal offence for drivers to fail to prevent smoking in their cars when children are present.

Labour has already promised to include this impossibletoenforce measure in its manifesto if it does not become law by May, www.2015.No one disputes smoking in a con fined space with children present is a dis gustingly selfish and harmful thing to do but if you want adults to behave like grown ups it's best not to treat them like children. Will a ban on smoking in the home be next? Will parentpolice launch nighttime raids on homes, wrenching televisions from children's bedrooms and binning crisps and sweets found in the cupboard? A day after the Government said it would not oppose Labour's nanny state car smok ing ban it refused to support a Conserva tive amendment to the Immigration Bill. Respected backbencher Dominic Raab, backed by almost 100 Tory colleagues and former Labour home secretary David Blunkett, wanted to stop foreign criminals abusing the European Convention of Human Rights as they fight deportation.

ALMOST 200 foreign felons every year cite Article 8, the "right to a family life", when facing an im migration tribunal and possible deportation but Raab's amend ment would have removed that right for anyone convicted of a serious offence who served more than a year in jail. Only proof that returning to their home country would result in their torture or death would have spared them.

Every year violent offenders, drug dealers and rapists use Article 8 to stay in a country with a rule of law they do not respect. Some are not married, childless or being chased for child maintenance.

Yet ministers argued Raab's amend ment is not necessary because the current appeals process is being cut down from 17 to four. Four? Most Britons would consider one appeal for a violent foreign criminal overly generous but four? Perhaps MPs should worry less about the stupidity of generally lawabiding Britons and more about the cunning of foreign criminals living in our country.

'Time and again the responsible must be punished along with the stupid or stubborn'

LOAD-DATE: February 2, 2014

LANGUAGE: ENGLISH

GRAPHIC: FRESH OUT OF IDEAS: Pupils' lunchboxes are being inspected by tutors
Picture: LEANNE TEMME/Getty

PUBLICATION-TYPE: Newspaper

JOURNAL-CODE: SXP

Copyright 2014 Express Newspapers
All Rights Reserved

Food crusaders' new challenge: cut sugar to save NHS £ 50bn a year: The campaigners who helped reduce salt intake in British diets by 15% in seven years have a fresh target in sight. But they fear this struggle will be much tougher - and talks with the health secretary this week could be crucial: THE DOLCE METER: SUGAR LEVELS IN EVERYDAY FOODS The Observer (England) February 2, 2014

JOURNAL-CODE: MTRscot

Copyright 2014 Associated Newspapers Ltd.
All Rights Reserved

London Evening Standard
standard.co.uk

April 1, 2014 Tuesday 5:08 PM GMT

Society 'normalising overweight'

SECTION: PA NEWS FEEDS

LENGTH: 1149 words

Obesity has become too normal in society and a "sugar tax" may be necessary in the long run, England's chief medical officer has said.

Dame Sally Davies used her annual report on the state of the nation's health to say too many people - and parents - may be ignoring the growing problem of obesity.

She said she had long been concerned that the fashion industry presented being underweight as an "ideal" aim.

"Yet I am increasingly concerned that society may be normalising overweight," she added.

"For example: larger mannequins are being introduced into clothes shops; 'size inflation' means that clothes with the same size label have become larger in recent decades; and news stories about overweight often feature pictures of severely obese people, which are unrepresentative of the majority of the overweight population."

Dame Sally said many people did not recognise they had a weight problem, with data showing that 52% of overweight men and 30% of overweight women think they are about the right weight.

Some 11% of obese men and 6% of obese women think the same, while 77% of parents of overweight children do not recognise their child is heavier than they should be.

And she said figures showed that between 2006/07 and 2012/13, some 27,860 children every year were found to be severely obese - on or above the 99.6th centile for weight.

This was putting them at increased risk of illness, including diabetes, asthma, musculoskeletal problems and heart disease.

Dame Sally said adults and children consumed more sugar than they should, with a third of added sugar in the diet of 11 to 18-year-olds coming from **fizzy drinks**, smoothies, and fruit juices with added sugar.

"This is an alarming proportion; **soft drinks** are easily avoidable sources of added **sugar**," she said, adding that clear labelling would help increase public understanding.

And Dame Sally reiterated her view that a "sugar tax" may be needed - something she told MPs earlier this month when she said she believed "research will find sugar is addictive".

Her views clash with those of the Government, which has taken the approach of asking manufacturers and food giants to sign up to voluntary codes with the aim of avoiding legislation on the issue.

In her report, Dame Sally said: " I call on manufacturers to ramp up reformulation of products to use less added sugar.

"If voluntary efforts fail to deliver then we, as a society, may need to consider the public health benefits that could be derived from regulation such as a 'sugar tax'."

Dame Sally also said far too few people did enough exercise, with recommendations being a minimum of 150 minutes (2.5 hours) of moderate intensity activity per week.

Furthermore, evidence has found that adults watched an average of 1,648 minutes (27.5 hours) of television per week in 2013.

Figures for England showed that almost two-thirds of adults and one-third of children aged two to 15 were overweight or obese.

In 1980, around 7% of adults were obese compared with around 25% today.

Dame Sally also used her study to call for more research into a potential link between deafness and blindness and dementia.

Data suggested a greater prevalence of dementia, including Alzheimer's disease, in those with severe vision loss or severe hearing impairment.

She also said England should explore the impact of minimum unit pricing for alcohol if Scotland introduced it.

Simon Gillespie, chief executive of the British Heart Foundation, said: "This report is yet another reminder that we can no longer turn a blind eye to the numbers on the scales.

"It's particularly troubling that around a third of children in the UK are now overweight or obese. Parents, schools and local communities all have a part to play to help kids reduce their risk of heart disease by eating well and keeping active.

"But the Government also needs to protect young hearts by stopping children being bombarded with junk food marketing. By placing a 9pm watershed on TV ads for junk food and regulating marketing tactics used to target kids online they can make it easier for families to eat healthily."

Professor Kevin Fenton, national director of health and wellbeing at Public Health England, said: "PHE welcome the Chief Medical Officer's report and we share her concerns on the normalisation of obesity and excessive alcohol intake.

"Overweight and obesity costs the NHS over £5 billion each year and is entirely preventable. PHE are committed to helping to tackle obesity through a range of approaches that support action on the local environment to make eating less and being more physically active, easier."

Tam Fry, from the Child Growth Foundation and the National Obesity Forum, said: " Having recently stated to the House of Commons health committee that she believes research will show that sugar is addictive and that taxing it may be required, the report lets the food and beverage industries off the hook.

"It makes no mention of sugar being addictive - which it is - and trots out the off-repeated Whitehall mantra that Government will legislate if excess sugar is not cut out of their products.

"It gives industry no deadline by which to show improvement with the likely result that her words will be quite ignored.

"How distressing."

Dame Sally pointed out that besides its other effects, alcohol was very calorific and she was concerned that Budget changes to alcohol duty - including a penny off a pint a beer - could encourage more drinking.

"Once I discovered the calories in alcohol, as well as the bad side effects, I drank much less," she told BBC Radio 4's Today.

"I worry that people will drink more but in fact at the moment alcohol consumption has not recently gone up, probably because of austerity."

People also needed to be made more aware that fruit juice was not a healthy option in large quantities," she said.

"When I used to sit in my clinic, mothers would bring their children in drinking vast amounts of orange juice and they thought this was healthy.

"Well one glass of orange juice in the day is but actually orange juice, and other juices, have a lot of sugar, natural sugar, and many calories.

"People need to think about this."

Shadow public health minister Luciana Berger said: "This report clearly illustrates the Government's failure to get to grips with the alarming rate of obesity.

"The Government should be on the side of parents in tackling this problem but they're failing. Their voluntary Responsibility Deal is weak and simply not working.

"Nothing is more important to any parent than protecting their child's health, yet the system is stacked against parents who want to make the right decisions for their children.

"The scale of the obesity challenge demands bold leadership. That is why Labour is consulting with parents and experts about whether a cap is needed on the amount of sugar, fat and salt in food marketed to children."

LOAD-DATE: April 2, 2014

LANGUAGE: ENGLISH

PUBLICATION-TYPE: Newspaper; Web Publication

JOURNAL-CODE: WEBES

Copyright 2014 Evening Standard Limited
All Rights Reserved

MailOnline

March 31, 2014 Monday 10:49 PM GMT

Fizzy drinks fall flat as soda sales decline in U.S. even faster than expected

BYLINE: ASSOCIATED PRESS

SECTION: NEWS

LENGTH: 433 words

- Total sales fell 3 percent in 2013 to 8.9 billion cases
- It was the ninth straight year of decline and lowest since 1995
- Soda sales in the United States grew throughout most of the 1990s, before beginning to slow in 1999

- Coca-Cola Co's share of the U.S. soft drinks market rose 0.4 percentage points, while PepsiCo Inc's market share shrank by the same percentage

Americans cut back on soda at an accelerated pace last year, underscoring the difficulties Coca-Cola and PepsiCo face in winning back customers.

U.S. sales volume of carbonated soft drinks fell 3 percent in 2013, extending a streak of declines that began nearly a decade ago.

It also represents a steeper drop than the 1.2 percent decline in 2012 and the 1 percent drop in 2011, according to an annual report by Beverage Digest, an industry tracker.

Carbonated soft drinks still represent the biggest category in the beverage industry. But the popularity of longtime favorites like Coke, Pepsi and Dr Pepper is waning as a growing number of alternatives like flavored waters and energy drinks pop up in beverage aisles. Soda has also been under fire from public health advocates for fueling weight gain.

Even diet sodas are suffering. Last year, for instance, Diet Coke's sales volume declined 6.8 percent, compared to a 0.5 percent drop for regular Coke, according to Beverage Digest. Diet Pepsi declined 6.9 percent, compared to a 3.6 percent decline for regular Pepsi.

Industry executives blame the trend in diet sodas on worries people have about artificial sweeteners. But diet sodas are also facing intensifying competition from the proliferation of lower-calorie alternatives, many of which are made with artificial sweeteners as well.

Sparkling Ice, a small brand owned by TalkingRain, for instance, last year saw sales more than double, according to IRI, a Chicago-based market research firm.

Overall, Coca-Cola, which also owns Sprite and Fanta, saw its soda volume fall 2.2 percent.

PepsiCo, which makes Mountain Dew, saw volume fall 4.4 percent. That was despite the company's stepped up marketing for its flagship soda, including sponsorship of the Super Bowl halftime show for the past two years.

Coca-Cola and PepsiCo both make an array of other beverages, including bottled water, orange juice and sports drinks. But sodas still account for a large and lucrative portion of their businesses, and executives have expressed determination in getting sales volume back on the path to growth.

Dan Schafer, a spokesman for Coca-Cola, said the Atlanta company was 'committed to returning our overall sparkling business to growth in the U.S.'

LOAD-DATE: March 31, 2014

LANGUAGE: ENGLISH

PUBLICATION-TYPE: Web Publication

JOURNAL-CODE: WEBDM

Copyright 2014 Associated Newspapers Ltd.
All Rights Reserved

The INDEPENDENT

The Independent (London)

March 28, 2014 Friday
First Edition

Obese is becoming the new normal, warns Chief Medical Officer

BYLINE: OLIVER WRIGHT WHITEHALL EDITOR

SECTION: NEWS; Pg. 4

LENGTH: 348 words

More than half of adults in Britain are now overweight or obese, but many don't realise it because society has "normalised" being fat, the country's Chief Medical Officer has warned.

Dame Sally Davies said stealth increases in clothes sizes and bigger shop mannequins were misleading people into thinking they were a normal weight when in fact they were fat.

She pointed to official figures for England which show almost two-thirds of adults and one-third of children aged two to 15 are overweight or obese. In 1980, about 7 per cent of adults were obese compared with about 25 per cent today. Surveys show that 52 per cent of overweight men and 30 per cent of overweight women think they are about the right weight.

"I am increasingly concerned that society may be normalising overweight," she said. "For example: larger mannequins are being introduced into clothes shops; 'size inflation' means that clothes with the same size label have become larger in recent decades; and news stories about being overweight often feature pictures of severely obese people, which are unrepresentative of the majority of the overweight population."

Dame Sally reiterated her view that a "sugar tax" may become necessary, saying a third of added sugar in the diet of 11- to 18-year-olds comes from **fizzy drinks,** smoothies or fruit juices with added sugar.

Her views clash with those of the Government, which has taken the approach of asking manufacturers and food giants to sign up to voluntary codes with the aim of avoiding legislation on the issue.

"I call on manufacturers to ramp up reformulation of products to use less added sugar," she said. "If voluntary efforts fail to deliver, then we, as a society, may need to consider the public health benefits that could be derived from regulation such as a 'sugar tax'."

Dame Sally also said far too few people did enough exercise, with recommendations being a minimum of 150 minutes (2.5 hours) of moderate-intensity activity per week.

Furthermore, evidence has found that adults watched an average of 1,648 minutes (27.5 hours) of television per week in 2013.

LOAD-DATE: March 28, 2014

LANGUAGE: ENGLISH

PUBLICATION-TYPE: Newspaper

JOURNAL-CODE: IA

Copyright 2014 Independent Print Ltd
All Rights Reserved

Metro (UK)

March 28, 2014 Friday
Edition 1;
National Edition

Sugary drinks 'are worse than pizzas or chocolate'

BYLINE: NICOLE LE MARIE

SECTION: NEWS; Pg. 51

LENGTH: 227 words

SUGARY drinks are causing more teenage obesity than junk food, new research suggests.

Cola, pop and other soft drinks are worse than pizza, chips, sweets and chocolate for weight gain.

Schools where fizzy beverages are available have students who drink them more often. They are also more likely to be obese when their body mass index is measured.

By contrast, pupils at schools with healthy eating guidelines consume 60 per cent fewer sugary drinks.

Health campaigner Tam Fry has demanded such soft drinks be banned from every British school.

He said: 'Children do not need anything other than water to refresh themselves. We have seen a huge number of children having teeth out before primary school.' The study of 11,000 pupils was by Prof Louise Masse, from Canada's University of British Columbia.

Unlike the US, Canada does not have school breakfasts or lunches subsidised by the government.

Mr Fry, chairman of the Child Growth Foundation, believes the obesity crisis has been made worse by fizzy drinks manufacturers. He accused them of encouraging children to pick up the habit as infants. He added: 'The Chief Medical Officer should be taxing sugar but the soft drinks industry is very powerful and in league with the government.

'It is the taxpayers that have to pick up the bill for all the harm soft drinks cause. Obesity costs the country £6billion a year.'

LOAD-DATE: March 28, 2014

LANGUAGE: ENGLISH

PUBLICATION-TYPE: Newspaper

JOURNAL-CODE: MTR

Copyright 2014 Associated Newspapers Ltd.
All Rights Reserved

THE TIMES
The Times (London)

March 28, 2014 Friday
Edition 1;
National Edition

Now it's normal to be fat, warns Britain's health chief

BYLINE: Chris Smyth

SECTION: NEWS; Pg. 3

LENGTH: 604 words

Being fat has come to be seen as normal in a Britain where most people are overweight, the Chief Medical Officer has warned. In her annual report, Dame Sally Davies said that obesity is now so common that society is "normalising being overweight". Professor Davies hit out at "size inflation" in clothing and said pictures of extremely fat people in the media are distorting our sense of the normal. She said a "sugar tax" on **fizzy drinks** needs to be considered, and took a swipe at pubs and supermarkets in a call for fresh restrictions on alcohol sales.

"I have long been concerned that being underweight is often portrayed as the ideal weight, particularly in the fashion industry. Yet I am increasingly concerned that society may be normalising being overweight," Professor Davies said. "Larger mannequins are being introduced into clothes shops, size inflation means that clothes with the same size label have become larger in recent decades, and news stories about weight often feature pictures of severely obese people, which are unrepresentative of the majority of overweight people." Last year Debenhams became the first British retailer to use size 16 mannequins in shops, saying this was the size of the average British woman and urging other shops to follow suit. Jo Swinson, the Equalities Minister, said that fashion ought to embrace "a broader range of body shapes".

Two thirds of adults and a third of children are now overweight or obese, and Professor Davies said people cannot spot when they have a problem. She pointed to a study that found three quarters of parents of overweight children did not recognise that their offspring were too fat. Half of overweight men and a third of overweight women think they are "about the right weight" she said, as do one in nine obese men and one 16 obese women.

The average man is 5ft 9in tall and weighs 13st 3lb while the average woman is 5ft 4in and 11st, making both overweight. A man of this height should lose at least 1st 4lb and a woman at least 11lb.

Professor Davies said this represented "a profound change in the health of the nation over a relatively short period of time". She urged food and drink manufacturers to cut the amount of sugar in their products and said that if such efforts fail "then we, as a society, may need to consider the public health benefits that could be derived from regulation such as a sugar tax". Cities must improve road safety to encourage more people to cycle, she said.

Figures this week showed a 40 per cent rise in liver disease in the past decade, but Professor Davies said a further "huge increase" was still to come, because the disease takes decades to appear. Britain now drinks about twice as much per person as 50 years ago.

She criticised films and television for glamorising drunkenness. "Drinking to excess is not normal behaviour, and portraying it as such is irresponsible," said Professor Davies She said that a minimum unit alcohol price is likely to cut drinking and said that England should copy Scotland if minimum pricing there proves successful.

In an attack on pubs and supermarkets, she added: "I deplore the methods which retailers use to entice consumers to purchase ever greater quantities of alcohol. For example, supermarkets promote multi-buy offers and sell alcohol below cost price. Licensed premises have redefined small glasses of wine, and omitted from menus the 125ml measure, which they are legally obliged to offer."

Simon Gillespie, chief executive of the British Heart Foundation, said: "This report is yet another reminder that we can no longer turn a blind eye to the numbers on the scales."

LOAD-DATE: March 28, 2014

LANGUAGE: ENGLISH

PUBLICATION-TYPE: Newspaper

JOURNAL-CODE: TIM

Copyright 2014 Times Newspapers Limited
All Rights Reserved

The INDEPENDENT

Independent.co.uk

March 27, 2014 Thursday 7:06 PM GMT

Obesity now seen as normal by society, warns Chief Medical Officer; In her annual report, Dame Sally added that many people did not recognise when they are overweight

BYLINE: Kashmira Gander

SECTION: FRONTPAGE

LENGTH: 626 words

Obesity is becoming a norm in society, England's Chief Medical Officer has warned in her annual report on the state of the nation's health.

Dame Sally Davies said that too many people ignore the problem of obesity, and claimed that a "sugar tax" may be necessary to encourage people to have healthier diets - advice she gave MPs earlier this month when she said she believed "research will find sugar is addictive".

"I am increasingly concerned that society may be normalising overweight," she wrote, citing: "larger mannequins are being introduced into clothes shops; 'size inflation' means that clothes with the same size label have become larger in recent decades; and news stories about overweight often feature pictures of severely obese people, which are unrepresentative of the majority of the overweight population."

She also claimed that people do not recognise that they have weight problems, with 52 per cent of overweight men and 30 per cent of overweight women believing they are a healthy size, she warned.

Dame Sally added that children are being being put at risk of developing illnesses including diabetes, asthma, musculoskeletal problems and heart disease.

She cited figures showing that between 2006/2007 and 2012/13, 27,860 children every year were found to be severely obese - on or above the 99.6th centile for weight.

The prevalence of the condition is being exacerbated by adults and children consuming more sugar than they should, she said, and explained that a third of added sugar in the diet of 11 to 18-year-olds comes from "easily avoidable sources" such as **fizzy drinks**, smoothies and fruit juices with added sugar.

Her proposal to introduce a sugar tax clashes with the Government's approach which involves asking manufacturers and food giants to sign up to voluntary codes with the aim of avoiding legislation on the issue.

"If voluntary efforts fail to deliver then we, as a society, may need to consider the public health benefits that could be derived from regulation such as a 'sugar tax'," she advised in her report.

She also pointed to statistics showing that most people fail to meet the 2.5 hours recommended level of exercise, but found that adults watched an average of 1,648 minutes (27.5 hours) of television per week in 2013.

Simon Gillespie, Chief Executive of the British Heart Foundation, said: "This report is yet another reminder that we can no longer turn a blind eye to the numbers on the scales.

"It's particularly troubling that around a third of children in the UK are now overweight or obese. Parents, schools and local communities all have a part to play to help kids reduce their risk of heart disease by eating well and keeping active.

Read more:DNA tests show Elvis Presley was prone to obesity and diseaseDentists call for ban on **sugary drinks** in schools to combat tooth decayChildhood obesity partly caused by strict parenting, say scientistsEating fried food is more likely to make you fat if you have 'obesity genes'

"But the Government also needs to protect young hearts by stopping children being bombarded with junk food marketing. By placing a 9pm watershed on TV ads for junk food and regulating marketing tactics used to target kids online they can make it easier for families to eat healthily."

Professor Kevin Fenton, national director of health and wellbeing at Public Health England, said: "PHE welcome the Chief Medical Officer's report and we share her concerns on the normalisation of obesity.

"Overweight and obesity costs the NHS over £5 billion each year and is entirely preventable. PHE are committed to helping to tackle obesity through a range of approaches that support action on the local environment to make eating less and being more physically active, easier."

Additional reporting by PA

LOAD-DATE: March 28, 2014

LANGUAGE: ENGLISH

PUBLICATION-TYPE: Newspaper; Web Publication

JOURNAL-CODE: WEBI

Copyright 2014 Independent Digital News and Media Limited

All Rights Reserved

MailOnline

March 27, 2014 Thursday 4:19 PM GMT

Bigger is not better: Larger mannequins have made being overweight 'normal', says nation's health chief

BYLINE: LUKE GARRATT

SECTION: HEALTH

LENGTH: 745 words

- Dame Sally Davies says plus-sized mannequins normalising obesity
- Chief medical officer's report suggests 'size inflation' is also to blame
- 25 per cent of adults in the UK are obese, compared with 7 per cent in 1980
- Dame Davies also suggested the introduction of a 'sugar tax'
- Claims companies needed to reduce the amount of added sugar in drinks

Stores using larger sized mannequins have been accused of 'normalising' Britain's obesity crisis, according to a report by England's chief medical officer.

Dame Sally Davies has warned that larger mannequins are making people think being overweight is acceptable.

She also said that many people do not recognise they have a weight problem, with data showing that 52 per cent of overweight men and 30 per cent of overweight women think they are the right weight.

Dame Davies blamed larger mannequins, combined with retailers' recent tendency to carry out 'size inflation' with clothes - when clothes with the same size label become larger - for undermining the health risks that come with obesity.

The fashion industry has long been accused of presenting 'underweight' as an ideal goal.

However, Dame Davies believes that in a recent trend, society has been complicit in normalising being 'overweight'.

She said: 'Larger mannequins are being introduced into clothes shops.

'"Size inflation" means that clothes with the same size label have become larger in recent decades.

'News stories about overweight (issues) often feature pictures of severely obese people, which are under-representative of the overweight population.'

While the average woman is a size 16, most clothes stores in Britain use mannequins that are between size 8 and 10.

Debenhams was the first department store to start using size 16 mannequins, in a push to try and get all high street stores to display larger models.

Many stores did not follow suit, with Marks & Spencer saying they took a 'responsible approach to visual merchandising'.

The issue of mannequins was a subject touched upon in Dame Davies' larger warning on the British obesity crisis.

She presented data that said around 11 per cent of obese men and 6 per cent of obese women think they are the right weight, while 77 per cent of parents of overweight children do not recognise their child is heavier than they should be.

Estimates suggest that almost two thirds of adults and one third of children under 18 are obese or overweight - around 33 million adults and 3 million children.

Around 25 per cent of adults in the UK are obese, compared with only 7 per cent of adults in 1980, an increase of 18 per cent over the last three decades.

In her report, Dame Davies also suggested a radical 'sugar tax'.

She said adults and children consumed too much, with a third of added sugar in the diet of children aged between 11 and 18 coming from **fizzy drinks**, smoothies and fruit juices.

She said: 'This is an alarming proportion.

'**Soft drinks** are easily avoidable sources of added **sugar**.

'I call on manufacturers to ramp up reformulation of products to use less sugar.

'If voluntary efforts fail to deliver then we, as a society, may need to consider the public health benefits that could be derived from regulation such as a "sugar tax".'

Dame Davies' report also pointed out that research found that adults watched an average of 1,648 minutes (27.5 hours) of television per week in 2013.

Dame Davies also spoke about the effects of alcohol on obesity, and suggested that changes to the Budget, such as a penny off a pint of beer, could encourage drinking.

This increased drinking could lead to rapid weight gain in the UK.

She said: 'Once I discovered the calories in alcohol, as well as the bad side effects, I drank much less,' she told BBC Radio 4.

'I worry that people will drink more but in fact at the moment alcohol consumption has not recently gone up, probably because of austerity.'

'People also needed to be made more aware that fruit juice was not a healthy option in large quantities,' she said.

'When I used to sit in my clinic, mothers would bring their children in drinking vast amounts of orange juice and they thought this was healthy.

She also used her study to call for more research into a potential link between deafness and blindness and dementia.

Data suggested a greater prevalence of dementia, including Alzheimer's disease, in those with severe vision loss or severe hearing impairment.

She also said England should explore the impact of minimum unit pricing for alcohol if Scotland introduced it.

LOAD-DATE: March 27, 2014

LANGUAGE: ENGLISH

PUBLICATION-TYPE: Web Publication

JOURNAL-CODE: WEBDM

Copyright 2014 Associated Newspapers Ltd.
All Rights Reserved

London Evening Standard
standard.co.uk

March 27, 2014 Thursday 4:11 PM GMT

Being overweight 'now considered normal' Chief Medical Officer says

SECTION: HOME

LENGTH: 396 words

Being overweight is now widely regarded as the "norm", the Government's Chief Medical Officer warned today.

Professor Dame Sally Davies said the average person in England is overweight but millions, particularly men, are convincing themselves that they are "about the right weight".

Three quarters of parents with overweight children do not recognise that they are too fat. "I am increasingly concerned that society may be normalising being overweight," Prof Davies said in her annual public health report.

"Larger mannequins are being introduced into clothes shops, ' size inflation' means that clothes with the same size label have become larger in recent decades, and news stories about weight often feature pictures of severely obese people, which are unrepresentative of the majority of overweight people."

Many obese people should get off the sofa, she suggested, and do more exercise rather than watch television. Adults should do a minimum of 2.5 hours of moderate intensity activity per week, she recommended, but around 40 per cent are not achieving this.

She added that this compared with adults watching an average of 27.5 hours of television a week, according to research.

A "sugar tax" should also be considered if the drink and food industry refused to take "voluntary" steps to cut the amount in products, she added.

For children aged 11 to 18, almost a third of the added **sugar** in their diet comes from **soft drinks**, the report highlighted, calling for manufacturers to reformulate and re-size products to use less **sugar** where possible.

Prof Davies warned that the "relative risks" for cyclists and pedestrians are "unacceptably high" and had to be reduced to encourage more people to walk and use a bike. Almost two thirds of adults and one third of children under 18 are overweight or obese, according to estimates.

The study of public health in 2012 found that in England, the average man weighs around 84kg and is around 175cm tall, while the average woman weighs around 70kg, and is about 162cm tall.

"Both the average man and the average woman are overweight," it added. "Overweight is associated with an increased risk of heart disease, type 2 diabetes, and cancer. Yet evidence shows that 52 per cent of overweight men and 30 per cent of overweight women think they are 'about the right weight', along with 11 per cent of obese men and six per cent of obese women."

LOAD-DATE: March 28, 2014

LANGUAGE: ENGLISH

PUBLICATION-TYPE: Newspaper; Web Publication

JOURNAL-CODE: WEBES

Copyright 2014 Evening Standard Limited
All Rights Reserved

The Telegraph
telegraph.co.uk

March 27, 2014 Thursday 2:35 PM GMT

Size 16 mannequins make being fat 'normal'; Chief medical officer says large fashion mannequins "normalise" being overweight

BYLINE: By Laura Donnelly Health Editor

SECTION: NEWS

LENGTH: 692 words

Large mannequins in shops are "normalising" being overweight and making increasing numbers of people feel that obesity is acceptable, the Chief Medical Officer has warned.

Retailers recently began using size 16 models in shop windows in an effort to reflect the shape of "real women" in Britain.

But in her annual report, Dame Sally Davies said the use of larger mannequins, combined with "size inflation" in many shops - meaning clothes with the same label have become larger - have normalised being overweight, blinding people to the health risks of obesity.

Dame Sally said: "I have long been concerned that being underweight is often portrayed as the ideal weight, particularly in the fashion industry.

"Yet I am increasingly concerned that society may be normalising being overweight.

"Larger mannequins are being introduced into clothes shops, 'size inflation' means that clothes with the same size label have become larger in recent decades, and news stories about weight often feature pictures of severely obese people, which are unrepresentative of the majority of overweight people."

In December Debenhams became the first department store to display size 16 mannequins in its 179 stores, and urged rival high street shops to follow its lead.

Earlier in the year equalities minister Jo Swinson called for fashion stores to promote a more diverse range of women, saying it was "as if there's only one way of being beautiful".

Nearly all clothes shops in Britain use size 10 or size 8 mannequins although the average British woman is a size 16.

At the time a spokeswoman for the British Retail Consortium said it was down to each individual store whether to display plus-size models.

"We won't be issuing guidance in the short term but it's an issue UK retailers take very seriously," she said.

A spokesman for Marks & Spencer said it takes a "responsible approach to visual merchandising" and pointed out that it uses size 10 models, which is above the size 8 market average.

Latest estimates suggest that almost two thirds of adults and one third of children under 18 are overweight or obese.

Dame Sally said she was concerned by studies which showed that many people who are overweight believe they are "about the right weight" and highlighted one piece of research which found 77 per cent of parents of overweight children did not recognise that their child was overweight.

The report also reaffirms the CMO's previous views on added sugar in drinks and alcohol minimum pricing.

The report highlights the fact that in children aged 11-18, almost a third of the added **sugar** in their average diet comes from **soft drinks.** The CMO called on manufacturers to reformulate and resize products to use less **sugar** where possible.

In the report, she also says that if voluntary efforts fail, then we may need to consider the benefits of regulation such as a 'sugar tax'. She also said we should explore the impact of minimum unit pricing for alcohol in Scotland, if it is introduced there.

Dame Sally also called for improvements in safety for pedestrians and cyclists to encourage people to walk and cycle more and reap the associated health benefits.

The chief medical officer said that the relative risks of walking and cycling have become unacceptably high, with the risk of serious injury for each kilometre travelled on a bike is 21 times higher than by car.

The report suggests that soap operas encourage bad habits in viewers.

It says: "In popular culture, drinking alcohol to excess is sometimes portrayed as normal behaviour. An analysis of six weeks of soap operas in the UK in 2010 found 162 instances of characters drinking to excess, with negative consequences rarely shown. In fact, 75 per cent of the population does not consume excessive quantities of alcohol, and the proportion of the population which abstains from alcohol is increasing."

The report also says research is suggesting an increasing prevalence of dementia, including Alzheimer's disease, in those with severe vision loss or severe hearing impairment.

Dame Sally said more data was needed to investigate the association between such impairment and the causes of dementia.

LOAD-DATE: March 28, 2014

LANGUAGE: ENGLISH

PUBLICATION-TYPE: Newspaper; Web Publication

JOURNAL-CODE: WEBDTNS

Copyright 2014 Telegraph Media Group Limited
All Rights Reserved

The Express

March 25, 2014 Tuesday
Edition 1;
National Edition

Water 'will outsell cola'

SECTION: NEWS; Pg. 17

LENGTH: 141 words

HEALTHY Britons are drinking so much bottled water it is set to overtake sales of **fizzy drinks**.

We spent £343million on 210 million gallons of water last year, boosting sales by 10.5 per cent.

Health experts have been calling for a tax on **sugary drinks**, which raise the risk of obesity, diabetes and tooth decay.

A spokesman for The Grocer said: "As temperatures spiked last July, so did sales of bottled water. But experts believe the sustained growth that followed points to a more significant sea change in consumer behaviour."

Bottled water now has a 60 per cent share of the low-calorie drinks market compared to 30 per cent in 1994, and it is expected to overtake drinks like cola by 2017.

Tam Fry, of the National Obesity Forum, said: "Water may not cure obesity, but if you drink a glass of water before a meal you are less inclined to overeat."

LOAD-DATE: March 25, 2014

LANGUAGE: ENGLISH

PUBLICATION-TYPE: Newspaper

JOURNAL-CODE: DXP

Copyright 2014 Express Newspapers
All Rights Reserved

Metro (UK)

March 24, 2014 Monday
Edition 1;
Scotland

On day six, a miracle happened;

Sugar is addictive - just like alcohol, nicotine and heroin. So what happens when you go cold turkey? Amy Grier finds out

BYLINE: Amy Grier

SECTION: FEATURES; Pg. 42

LENGTH: 1107 words

IT'S not as if I was eating Mars bars for breakfast. But two months ago, after keeping a food diary for a week, I was shocked at how much sugar I was consuming.

I already knew the perils of **fizzy drinks** and cereal bars but the amount in foods I had thought relatively harmless was an eye-opener.

Take the Pret A Manger porridge and honey I'd grab for breakfast - that's 33g. A large glass of white wine? That's up to 8g, while a tin of shop-bought soup is at least 10g. Something had to change.

The sugar debate has been raging since 1972, when British Professor John Yudkin went against the grain to suggest it was sugar, not fat, that was to blame for our inflated waistlines. He went as far as saying it was also bad for our health.

Contested by food manufacturers and sugar lobbies vested in feeding our addiction, Yudkin's work has now been accepted as prophesy, predicting all too accurately the way over-consumption of sugar would create an obesity epidemic.

According to the Overseas Development Institute, 64 per cent of us Brits are now obese. What's more, a record three million of us have diabetes, say Diabetes UK.

'Sugar used to be a condiment,' says Dr Robert Lustig, author of Fat Chance and the man responsible for the renewed focus on Yudkin's work. 'Now, it's a staple. Just as with alcohol, we have a limited capacity to metabolise it before it does damage.'

Our bodies can handle sugar in small doses, using insulin to break it down and use it as energy. But beyond a certain amount, it is converted into fat. This has two important effects. Firstly, you get fat. And secondly, after the initial hit, your blood sugar level falls lower than it was to start with, meaning you need a bigger hit to satisfy you next time. Over the past year, there's been a mass of revelations about the way sugar consumption impacts on our health. It causes more obesity than saturated fat, it's the root of that worldwide diabetes epidemic, it creates a hospitable environment for cancer, anxiety, poor concentration, and premature www.ageing.No wonder England's chief medical officer Dame Sally Davies has suggested the government should implement a sugar tax.

With this in mind, I began my quest. The first step was to throw out everything that would feed the addiction. Next, I owned up to the two main reasons I used sugar as a crutch: stress and laziness.

I started in earnest after a consultation with nutritionist Vicki Edgson.

'Sugar doesn't just mean Jelly Babies,' she says. 'A regular yoghurt can contain as much as eight teaspoons of sugar.'

SO I banished white carbs and booze (they both convert into sugar in your bloodstream), processed foods, fruit yoghurts and fizzy or flavoured drinks (which pack in excess sugar by the teaspoon-load). I replaced them with protein and good fats - eggs, avocado, full-fat natural yoghurt, spinach, oily fish, nuts, small amounts of berries.

The carbs I did eat (lentils, quinoa, chickpeas, butternut squash) were for lunch and I filled Tupperware boxes with sugar-free snacks: boiled eggs, cooked prawns, celery and humous, nuts, smoked chicken or feta and beetroot.

The first few days were rough. I felt tired, relied more heavily on caffeine, which only increased the withdrawal headaches, and my skin flared up. Limiting my fruit intake was also tough. Fructose is a natural sugar but a sugar all the same.

'Generally, the higher the water content, the less the sugar hit,' says Edgson. 'Eat fruits whole rather than juicing them, as juicing removes the fibre, which slows down absorption of sugar into the blood stream.'

But on day six something miraculous happened. My alarm went off at 6.30am and, for the first time, I didn't feel tired.

'In studies on rats, sugar affected the dopamine and endorphin receptors, which are part of the reward system,' says nutritionist Patrick Holford. 'The more sugar they had, the more those receptors shut down. It's a process called down regulation, which means you need more and more sugar to get the same effect.

'When you give up sugar, it takes up to a week for your body to re-sensitise to your natural dopamine and for your blood sugar levels to even out. That's when your energy starts to come back.'

My mission was labour intensive but, slowly, it worked. After two weeks, I'd lost half a stone. Unfortunately, the jury is still out on how long it really takes to kick the habit. Lustig says three weeks. 'Just like any other drug, after three weeks your palette will be re-sensitised,' he says.

But at week three I hit a wall. Standing outside a Sainsbury's store inhaling the smell of its bakery was a definite low point. So I found new ways to eat sweet.

'Cinnamon tastes sweet and contains cinnulin, which could help to lower blood sugar,' says Holford. I followed his advice and added it to my coffee, yoghurt and quinoa porridge. Vanilla, star anise and coconut oil also helped dupe my unsuspecting palette.

By week four, not indulging meant the 'need' stayed in my head. And at the end of six weeks I'd released myself from the substance I'd unwittingly been addicted to since I first clutched a chocolate bar aged four.

I can't say I've stayed entirely on the wagon but cutting it out even briefly is a good way to make you re-evaluate how, when and why you let it into your life, if at all.

SWEET SWAP THE ALTERNATIVES

Knowing your maple from your molasses is crucial if you're to kick the sugar habit. Here are the natural substitutes.

Xylitol

This is extracted from woody plants such as birch and its low glycemic index (GI) has little effect on blood sugar levels. It therefore doesn't create insulin resistance. It's not as sweet as sugar but is great in baking, tea and coffee.

Agave The goji berry of the sugar world, this syrup from the inside of the cactus-like agave plant (pictured) was all the rage two years ago. It's up to 90 per cent fructose, which means it has a high GI and will raise blood sugar levels. It can also be very highly processed.

Stevia Its icing sugar-like texture makes this plant derivative perfect for baking and, with zero calories, it's almost too good to be true. Perhaps it is - at 300 times sweeter than sugar, it could cause the kind of addiction you're trying to kick in the first place.

Brown rice syrup This is made by boiling down brown rice with various cultures and enzymes. It's not as sweet as honey or sugar and has no notable nutritional benefits so use sparingly.

Coconut blossom nectar The latest wonder ingredient, this is the sap of the coconut tree and less highly processed than other syrups. It doesn't cause spikes in blood sugar levels and, unlike coconut oil, it doesn't have a strong coconut flavour.

LOAD-DATE: March 24, 2014

LANGUAGE: ENGLISH

GRAPHIC: SHUTTERSTOCK
Kicking the habit: How hard can it be to cut out sugar? Nutritionist Vicki Edgson (below right) says more foods contain the sweet stuff than you might think

PUBLICATION-TYPE: Newspaper

JOURNAL-CODE: MTRscot

Copyright 2014 Associated Newspapers Ltd.
All Rights Reserved

The Daily Telegraph

The Daily Telegraph (London)

March 22, 2014 Saturday
Edition 1;
National Edition

Stick up for sweetness;
Baking Club Each week, Rose Prince shows you the way to better baking. Today: Flapjacks

BYLINE: Rose Prince

SECTION: WEEKEND;FEATURES; Pg. 10

LENGTH: 664 words

The bitter truth about sugar sits uneasily amid the baking craze. In fact, the baking fraternity has been rather quiet while sugar takes the rap for being the cause of every killer illness from obesity to cancer. For bakers, campaigning against adding sugar to their cakes is a little like a bank campaigning against taking cash deposits. It is a necessary evil in the trade, with few palatable or practical alternatives.

There is no point trying to defend the nutritional negatives about sugar, though I have felt at times a certain unpleasant tone in the activism. I and fellow weak-willed humans know full well that, no matter how scary the headlines, we are going to continue to enjoy something sweet all our lives - and no nutritional police force will stop that.

I am in favour, however, of the food industry taking responsibility, even of a tax on processed foods that are stuffed full of sugar and fat, but in all the jawing on about the iniquities of sugar, I have not heard anyone mention that - before the fridge freezer - sugar played an important role in nourishment.

Sure, sugar contains so-called empty calories with no known dietary benefit, but it is a preservative. At one time, before low-priced imported fruit, the only source of vitamin C available to a child in winter was the jam on his toast. The same child's parents may have allowed him only a teaspoon or two of jam a day, which is very different to the frenzied consumption of **sugary drinks** and foods that health authorities worry about now.

It is not just what we eat but how we eat it, and home bakers can justifiably celebrate sugar as a sublimely enjoyable ingredient, so long as they are not stuffing 10 cakes a day into their children.

When I was little, flapjacks were regarded as a healthy option (all those sustaining oats.) Now that we have discovered granola and agave syrup, the flapjack has become the Dunkin' Donut of the genre. The rebel in me, irritated (in a low-blood-sugar sort of way) at the pious sugar police, decided that flapjacks should be the order of the day, and this week's recipe. I had forgotten how delightful it is to chew on a toffee-glued cluster of grains. There is something good in there, somewhere...

Equipment

Baking tray (25-38cm/10-15in, about 2-3cm/1-1½in depth), lined with baking parchment

Ingredients

350g (12oz) butter 1 tbsp/15ml golden syrup 225g/8oz golden caster sugar 450g/1lb jumbo oats

Preheat the oven to 180C/350F/ Gas 4. Melt the butter and add the golden syrup and sugar. Bring slowly to boiling point and then remove from the heat. Add the oats and stir well.

Pour or spoon the mixture into the baking tray, then lightly spread until even.

Bake for about 25 minutes until the top of the flapjacks turns gold. Remove from the oven and allow to cool for a minute. Cut into squares before the flapjacks cool down.

Leave in the pan for another 15 minutes, then separate them.

Your letters

Cheryl Arden is a coconut fan without a source of fresh coconut.

She says: "I love to make cakes and biscuits with coconut - is there a source of prepared fresh coconut?"

The only decent tool I have ever seen that effectively removes the flesh from the nut was in Sri Lanka. It looks like an oblong boot jack, and the cook kneels on it and works the mechanism to dig out the contents of the coconut.

But you can buy frozen grated coconut in Asian supermarkets. The online Asian food specialists www.planet-asia.com sells the Sumeru brand, £1.99 for 454g.

Next week: Baked pastries with feta and herbs. You will need filo pastry sheets, feta cheese, mint, thyme, parsley, chilli flakes and butter.

Tomorrow at 12.30pm, The BBC Radio 4 Food Programme tells the story of Rose Prince and her son Jack's Pocket Bakery.

Online

'She championed butter, pooh-poohed the health police and positively encouraged the pursuit of anything that was wild and edible. And she knew what she was on about' - read Rose Prince's tribute to Clarissa Dickson Wright on www.telegraph.co.uk/food

LOAD-DATE: March 22, 2014

LANGUAGE: ENGLISH

GRAPHIC: ANDREW CROWLEY

PUBLICATION-TYPE: Newspaper

JOURNAL-CODE: DTL

Copyright 2014 Telegraph Media Group Limited
All Rights Reserved

i-Independent Print Ltd

March 21, 2014
First Edition

Dentists urge ban on sugary drinks; HEALTH

BYLINE: Charlie Cooper HEALTH REPORTER

SECTION: NEWS; Pg. 23

LENGTH: 242 words

All **sugary drinks** - including energy drinks - should be banned in schools to tackle tooth decay, which affects one in four children starting school, leading dentists have said.

While crisps, confectionery and **fizzy drinks** have already been banned from council-run schools, energy drinks, which are often very high in sugar, have only been banned at two schools in the north of England, the British Dental Health Foundation (BDHF) said, adding that their example should be taken up by the Government.

The move comes amid growing pressure for action on high levels of sugar in our diet, which doctors say is a leading cause of obesity, but provides next to no nutritional value. Dr Nigel Carter, chief executive of the BDHF said that an increase in consumption of **sugary drinks** was also one of the leading causes of dental decay, particularly in children.

"It is very refreshing to see these schools taking their own initiative and banning **sugary drinks**," he said. "Proposals such as the introduction of a duty on **sugary drinks**, and brands reducing the amount of **sugar** in their **soft drinks**, have both been mooted in the last 12 months. If we can build on these foundations, there will be an inevitable reduction in consumption and benefits for both general and dental health."

The Department for Education is currently consulting on "revised school food standards to make sure children are always served healthy, nutritional meals at school", a spokesman said.

LOAD-DATE: March 21, 2014

LANGUAGE: ENGLISH

PUBLICATION-TYPE: Newspaper

JOURNAL-CODE: III

Copyright 2014 Independent Print Limited
All Rights Reserved

The Sun (England)

March 21, 2014 Friday
Edition 1;
National Edition

'BAN PUPIL POP AND SAVE TEETH'

BYLINE: NICK McDERMOTT

SECTION: NEWS; Pg. 32

LENGTH: 164 words

DENTISTS have called for **sugary drinks** to be banned in schools - to stop kids' teeth rotting.

They say ministers must act to help protect the oral health of children who guzzle cans of pop.

The British Dental Health Foundation said a ban would also help in the fight against obesity. The organisation has praised Burnage Media College in Manchester and William Howard School in Brampton, Cumbria, for independently banning energy drinks.

Chief executive Dr Nigel Carter urged the Government to roll out the idea across the country. He said: "Drinks packed with sugar have long been problematic to oral health. The Foundation has supported policies designed to curb **sugary drinks** consumption from a number of health initiatives, and still the Government has not acted."

The BDHF wants energy and **sugary drinks** banned in schools but does not oppose sugar-free ones.

CAMPAIGNERS Action on Junk Food Marketing have called for a ban on unhealthy grub and drink ads during family TV shows before 9pm.

LOAD-DATE: March 21, 2014

LANGUAGE: ENGLISH

GRAPHIC: Fizz ... full of sugar

PUBLICATION-TYPE: Newspaper

JOURNAL-CODE: SUN

Copyright 2014 NEWS GROUP NEWSPAPERS LTD
All Rights Reserved

The INDEPENDENT
Independent.co.uk

March 20, 2014 Thursday 8:28 PM GMT

Dentists call for ban on sugary drinks in schools to combat tooth decay; Crisps, confectionery and fizzy drinks have already been banned from council-run schools

BYLINE: Charlie Cooper

SECTION: FRONTPAGE

LENGTH: 242 words

All **sugary drinks** - including energy drinks - should be banned in schools to tackle tooth decay which affects one in four children starting school, leading dentists have said.

While crisps, confectionery and **fizzy drinks** have already been banned from council-run schools, energy drinks, which are often very high in sugar, have only been banned at two schools in the north of England, the British Dental Health Foundation (BDHF) said, adding that their example should be taken up by the Government.

The move comes amid growing pressure for action on high levels of sugar in our diet, which doctors say is a leading cause of obesity, but provides next to no nutritional value.

Dr Nigel Carter, chief executive of the BDHF said that an increase in consumption of **sugary drinks** was also one of the leading causes of dental decay, particularly in children.

"It is very refreshing to see these schools taking their own initiative and banning **sugary drinks**..." he said. "Proposals such as the introduction of a duty on **sugary drinks** and brands reducing the amount of **sugar** in their **soft drinks** have both been mooted in the last 12 months. If we can build on these foundations, there will be an inevitable reduction in consumption and benefits for both general and dental health."

The Department for Education is currently consulting on a "revised school food standards to make sure children are always served healthy, nutritional meals at school," a spokesperson said.

LOAD-DATE: March 21, 2014

LANGUAGE: ENGLISH

PUBLICATION-TYPE: Newspaper; Web Publication

JOURNAL-CODE: WEBI

Copyright 2014 Independent Digital News and Media Limited
All Rights Reserved

The Observer
The Observer (England)

March 16, 2014

Sugar is now at the heart of the debate on food and health. But how easy would it be for an ordinary family to cut it out for 30 days? Louise Carpenter and her children take up the challenge

BYLINE: PHOTOGRAPH Richard Saker

SECTION: OBSERVER FOOD MONTHLY; Pg. 40

LENGTH: 2414 words

As a mother of four I am not sure how I am supposed to feel about sugar. If I believe the anti-sugar lobby, it's "the new tobacco". Sugar rather than fat, the argument goes, is responsible for ever-rising levels of obesity. "Sugar is not addictive like tobacco," explains Professor Graham MacGregor, chairman of the campaign group Action on Sugar, "but it causes just as much harm in other ways. It is an unnecessary source of calories and a major cause of obesity, thereby causing many deaths and diabetes."

The more sugar you eat or drink, the more the body stores it as fat. Hence the links to obesity. But what is emerging is just how much of what we eat is stuffed with "hidden" sugar, not just in **fizzy drinks** and doughnuts, but sauces, cereals, fruit juices, even fruit itself. This month Britain's chief medical officer Dame Sally Davies said "we may need to introduce a sugar tax" to help reduce the amount manufacturers put in their products.

This can be confusing for the average person who is just trying to feed their kids. And that's before addressing the counter-argument that sugar is taking too much of the blame and that overall nutrition, as well as exercise, are important too.

To try to make sense of it all, I go cold turkey for 30 days, dragging my family along for the ride. If I cut out sugar, would I feel better? Being the kind of person who steams my children's vegetables while allowing them a sticky bun at the weekend, I am a pretty good guinea pig. How much hidden sugar is really in my children's diet? And how much of life without sugar can I tolerate?

Week One

The mood of this first week free from sugar is, to quote Thomas the Tank's Fat Controller, "confusion and delay".

I've done a lot of reading: Michael Moss's bestseller Salt Sugar Fat; the blog Kate Quit Sugar; the NHS Choices website; endless press coverage; James Duigan's Clean & Lean Diet. I've watched Dr Robert Lustig's convincing lecture, Sugar: the Bitter Truth, on YouTube.

Here's what goes in the bin: Cheerios, Fruit and Fibre, Petits Filous yoghurts (my son has been known to eat three on the trot), baked beans, tomato sauce, tomato and mascarpone pasta sauce and the children's Saturday evening "treat": pizzas. Also on the way out are jam, honey and anything, frankly, that kids find tastes nice.

Where I am confused is on the issue of fruit and moderation. Fruit is laden with sugar (fructose). If, as Lustig says, fructose is "poisonous", what is moderation? Smoothies and fruit juices, if you believe some research, are as bad as Coke (35g of sugars - nearly 9 teaspoons per can). Get rid of fruit in my children's lunchboxes? They have two pieces every day and often fruit for pudding. But a banana can have 7 tsp, grapes 1 1/2 , and a melon 12 tsp - all their favourites.

The NHS is less hardline, more sane, advising a "balanced diet" and so too is Kate Quit Sugar: "I eat fruit because it is delicious. The whole fruit includes the fibre of the flesh and also the naturally occurring fruit sugar. People have a million opinions on fruit . . . make up your own mind!"

But when it comes to mass-market fruit juices, even some of those sold as having relatively lower sugar content, everybody is pretty much united. They are bad. Spawns of the devil.

I decide that the fruit stays for the kids but not bananas, and no fruit for me. I supplement bananas with kiwis and lower fructose fruit such as berries, and switch to raw vegetables such as peppers and sugar snaps, which the children welcome (thank God). Brown pasta and granary bread also stay. The children are small, and I'm not prepared to experiment with them in the way I can with myself.

There's some grumbling about the cereals (20.9g of sugars - 5 tsp - per 100g); "why do they tell us on the box they are healthy?" asks my eight-year-old. A quick life lesson there. But when I hide the chocolate biscuits (two each after school, normally: 1 tsp of sugar each) there is a riot. "You're lying! You're lying!" It's a routine for them. I relent.

As for me, the diet is a drastic change, not from cutting out sweet stuff (I'm not big on biscuits and chocolate) but from eating no carbohydrates at all (all sugar in the end). By day two, I have no energy. I have to go to bed straight after the children at 8.30. The running I started a few months ago - which brought me such mental relaxation and quick weight loss - is off the cards. I feel cross and resentful.

I seem to live on boiled eggs, almonds, coconut flakes, protein in various forms, avocado and kale. I go to the health food shop in my country town and spend more than £ 40 on chia seeds, quinoa, flaxseed, more coconut flakes, hazelnuts, coconut water (hideously expensive) and more kale. I'm like a crazed celebrity. By Sherborne standards, I've spent so much in one hit the shopkeeper throws in protein shake samples for free, for my planned smoothies. In Sainsbury's I buy expensive, unsweetened almond milk and a small jar of coconut oil that costs £ 6. Six £! That's the price of a chicken for the kids.

Breakfast is my main problem. The low glycemic index granola from the health shop, bought at vast expense, disappears in one sitting. It's all very well for Hollywood stars to whip up smoothies of avocado, kale, blueberries and chia seeds, but you try doing that without a housekeeper or a nanny when you've got four kids, a job and lunchboxes to pack.

So I'll leave you with an image at the end of this first week: determined to make said smoothie but not owning a smoothie maker, following a recipe, I throw into the food processor the frozen berries (low sugar), kale, chia seeds, coconut water. It all explodes over the top and onto the floor. The dog laps it up and is sick. The little one is banging his spoon on the table singing at the top of his voice "No no no sugar, never never never!" I am in so much pain with my back - which I later find out is severe constipation - that I can hardly move. I cry out in such anguish that my husband looks worried rather than bemused.

But by day seven, everybody is eating full fat Greek yoghurt sweetened with berries and topped with protein-packed nuts. Perhaps this is a new definition of good mothering? Never mind if the mother can't move.

Week Two

The backache has eased. I make a string of rather joyless suppers with quinoa and various leaves (no balsamic in the dressing) and spiced-up protein or smoked salmon. The husband confesses that one day he is so starving he eats two hot lunches at work.

The NHS recommends that we try to limit ourselves to 10tsp (40g) of added sugars a day, but some have said this should be 6 tsp for women and 8tsp for men. The World Health Organisation now recommends just 6tsp (25g) for adults. Roughly, you divide the grams by four to get the teaspoons.

I am a sad woman in the supermarket, squinting at the "carbohydrates (of which sugars)" labels. (A free smartphone app, FoodSwitch, scans labels - measuring total fat, saturated fat, sugars and salt - and offers healthier alternatives.) Anyway, according to the NHS high sugar is more than 22.5g of total sugars per 100g and low is 5g or less per 100g. A lot of innocent-looking stuff - like a boeuf bourguignon or coq au vin packet flavouring - is out for being way too high.

I must be on about 1 tsp a day now, allowing for the odd oat cake and glass of red wine (half a teaspoon), which, paradoxically, the stress of the whole project makes necessary. I realise that a significant amount of my previous sugar intake came from bread and rice, and white wine. When I reintroduce a fruit yoghurt to my son as an experiment, he goes nuts with the sugar rush.

This week's progress is shaped by a visit to London to see Dr John Briffa, author of the weight loss guide Escape the Diet Trap, who makes sense of these subtle changes in my life. The low energy is my body recalibrating its metabolism, switching its system of fuel from carbs to fats and proteins. "Hang in there," he advises. "You are going to start feeling a lot better very soon."

Eat fats to fill up, he says: "Historically, we've had this focus on fat but it appears that fat is not inherently fattening. Insulin plays a key role in fat storage and the more insulin you secrete, the more you are likely to become insulin-resistant." Basically, you eat a lot of sugar, you store a lot of weight.

Briffa is hardline on carbs, even porridge: "just a big bowl of starch". My constipation is due to a lack of water and vegetables. "I've seen hundreds of clients on this kind of diet and none of them suffer from constipation . . . People weren't eating granary bread and porridge two million years ago." (When I quote this back to my husband, he retorts "People didn't live long two million years ago".)

I sense my need for clear answers is irritating Briffa. I basically want him to tell me what to feed my kids. Is it not absurd for a middle-class mother, committed to fresh food, to be stressing about a chocolate biscuit and worse, fruit, in itself full of soluble fibre and goodness?

"Look," he says, "if you have normal kids who exercise, with no weight problems and no history of diabetes, a rule of thumb would be that natural sugars from fruits are OK. But if you brought an obese kid in here, I would certainly be telling you to take the fruit out of the lunchbox. And the granary roll is OK, but only as a vehicle for getting a healthier filling inside them. I can't tell you that a biscuit as a snack is good. It has no nutritional value at all.'

On Valentine's Day, my son gives me a shortbread heart biscuit covered in pink icing that he has made at playgroup. I eat it in an instant because it was made and given with such love. It's delicious. What could matter more than this?

Week Three

Two people tell me I look "fresh". I haven't been "fresh" for years. I'm waking up refreshed, which I've been craving for the last 10 years. Briffa had predicted this: my blood sugars have stabilised.

The children seem to have forgotten about cereal and fruit juice. We make our own pizza on Saturday night. Working mostly from home means I'm around at teatime, making it easier to control and plan the menu.

I've abandoned kale smoothies, which even Briffa said were hardcore, and we've settled into a scrambled egg/granola/yoghurt breakfast routine, with a bit of Weetabix for them too (shoot me). I'm working out that I resent hidden sugars more than the obvious sugars. In other words, yoghurts, sauces and cereals are worse than biscuits for me because I consciously choose to allow the biscuits in moderation.

Being the mother of three girls is a factor. Perhaps an important change in women of my generation is that none of us wants to create in our daughters food/body issues from things having been "forbidden", hence the biscuits in moderation. But my school-age girls surprise me: they have already learnt about traffic light food labelling in class. I never got any of this as a child.

With a history of mild dieting (and teenage years of pretty extreme dieting), I am resisting the urge to get on the scales. This is not about being on a diet. I have to keep reminding myself of James Duigan, personal trainer to Elle Macpherson, who in his own books agrees with Briffa: "Sugar is a nuclear fat bomb exploding all over your body," he says. The magic formula is to fill up on good fats and proteins and stabilise your blood sugar. So I continue to eat and snack on (mostly) good fats: nuts, avocado and a bit of cheese. I relax about bacon. After three decades of anti-fat programming, this feels like I'm breaking some kind of diet law.

The week closes with a spectacular display of bad behaviour. During a dinner party, I knock back a lot of prosecco (the worst), red wine and potato gratin. At the end of the evening I throw up in our bathroom. Classy. This has to be the sugar since I've drunk more before and not been such a wreck. I feel poisoned.

Week Four

I go running four times! A miracle given how I felt in week one.

The children and I agree to photographs in our home with all the food and drink we have cut out. Piles of it are laid on the table. My kids fall upon the sugar stuffs like locusts, clinging the packets to their chests and shouting requests for smoothies, chocolate bars and jelly babies - stuff they had supposedly forgotten about. They are slightly out of control and it panics me. This, is what happens when food is forbidden. I resolve to undo this psychology of the forbidden food by calibrating them ever so slightly in the opposite direction next week, when it's over.

As the week draws to a close, I feel relief like a convict waiting by the prison gates. On day 31, I wake up and the girls present me with chocolate cup cakes they have made in secret to celebrate. I eat one because my eldest daughter wants me to. I go downstairs and find a box of Belgian chocolates. I taste one. You know what? I don't even like it. Only Gwyneth Paltrow could be more annoying than that.

Postscript

Two weeks after my 30-day diet ends, it pains me to say that I continue to eat in the same way. I thought I'd be liberated - free from the tyranny - but my palate has been retrained. I find bread heavy now. I don't want rice or chocolate biscuits or pasta. Protein fills me up and keeps me going. I don't buy juice, smoothies, yoghurt or Cheerios for the children and I carry on putting raw vegetables and nuts alongside fruit in their lunchboxes. I'm certainly not going to be a militant anti-sugar mother, but I cannot find it in my heart to allow them a can of Coke, however much it's "a treat".

They continue to have their two chocolate biscuits after school, though, along with their treats on Saturday, whether it's popcorn, pizza or an iced bun, but I am now more conscious of their daily tally. Two biscuits, with juice, combined with a banana and grapes and perhaps a pasta sauce and a bowl of porridge with honey? That's too much sugar for my children in one day.

I don't believe that the levels of sugar my children eat compromise their health. They are fit and slim. But equally, I'm not prepared to be hoodwinked by products stuffed with hidden sugars. Thirty days of being on this diet has, ultimately, made me sugar-aware rather than permanently sugar-free.

And only now can I say it: if I carry on, I'll drop a jean size too, although for the first time in my life, that's really not the point. OFM

Captions:

Louise Carpenter at home in Dorset with her children - and some of the foods that were essential to their low-sugar diet.

LOAD-DATE: March 15, 2014

LANGUAGE: ENGLISH

PUBLICATION-TYPE: Newspaper

Copyright 2014 Guardian Newspapers Limited
All Rights Reserved

The Observer (England)

March 16, 2014

Big Food is in wilful denial about the harm sugar does to our children: Only by improving processed foods can we tackle obesity among the young. Just beware hysteria about fruit

BYLINE: Aseem Malhotra

SECTION: OBSERVER NEW COMMENT PAGES; Pg. 37

LENGTH: 1159 words

I don't believe that anyone chooses to be fat. The Gallup International survey of 57,000 adults revealed that health is what mattered most in life, followed by a happy family environment. So how can this be reconciled with the statistic that 60% of UK adults and one in three children are overweight or obese?

It is partly because a fundamental misunderstanding among the public - and the scientific community - has interfered with our collective ability to curb this epidemic. The belief that we make our food choices deliberately, and that they reflect our true desires, sustains the status quo and obscures the reality that decisions about the food we consume are often difficult to control.

As Professor Theresa Marteau, director of the behaviour and health research unit at the University of Cambridge points out: "We are heavily influenced by automatic behaviours. . . where the desire for instant gratification far outweighs less assured and more distant rewards."

It is this instinctive behaviour that has been exploited by a food industry that has handsomely profited from ensuring cheap junk food is available to anyone, anywhere, at any time. The further manipulations have been rightly put under scrutiny in recent months, with the exposure of hidden sugars in foods not normally associated as being junk, such as salad dressings and even "low fat" or "healthy" cereals.

A group of interested parties, including myself, launched Action on Sugar this year. Our aim is to press the food industry into reducing added sugar in processed foods. We are not telling you - despite some of the screaming "sugar is toxic" headlines - that bananas and oranges are evil.

The food industry's classic response is to say that the ingredients are listed on the label, knowing full well that many consumers will purchase a food item based upon the way it's promoted, not its nutritional content. The fact that many products in a single portion come close to, if not exceed, the recent World Health Organisation limits of six teaspoons of non-milk extrinsic sugar (NMES) is as clear an indication as any that this issue is way beyond one of simple personal responsibility. Regulation to protect consumers and children is clearly needed.

NMES includes any sugar added to food in processing, as well as fruit juice and smoothies. Fruit juice lacks the fibre of whole fruit, which slows the absorption of sugar into the blood stream and makes you feel full more easily. You can easily down a glass of juice made up from six apples and still feel hungry, but try eating more than three whole apples and you'll struggle.

In addition to overwhelming scientific consensus that added sugar is a source of unnecessary calories, there is mounting evidence that the effects of excess consumption are independent of body weight. A study published in JAMA Internal Medicine last month revealed that those who consume more than 25% of calories from added sugar trebled their risk of cardiovascular death compared with those who consumed less than 10% from added sugar, even among the non-obese.

However, the industry remains in denial. On BBC Newsnight recently, the president of Coca-Cola Europe said his company's nine sugar-lump laden drink had the same number of calories as half a croissant or a cappuccino. Coca-Cola says that it's OK to consume its "happy" calories as long as you exercise, but this is not in keeping with independent scientific evidence. It matters where the calories are coming from.

The erroneous message that has promoted calorie restriction over good nutrition has proved to be the lottery win for diet companies. In the United States, the weight-loss industry generates a staggering $58bn in revenue annually, despite long-term studies revealing that the majority of individuals regain virtually all of the weight lost during treatment, irrespective of whether they maintain their diet or exercise programme. And such fad dieting that encourages rapid weight loss followed by regain is detrimental to health.

With all the attention on sugar, we mustn't neglect the other dietary villains. The excess consumption of refined carbohydrates found in white bread, pizza bases, burger buns and chips, combined with the excess salt and trans-fats in processed meat, continues to have awful consequences. On Friday, a study revealed that greater access to takeaway shops was associated with a greater body mass index and higher odds of obesity.

The traditional Greek/Mediterranean diet of oily fish, extra virgin olive oil, nuts, vegetables and yes, plenty of whole fruit (and, happily, a moderate intake of red wine) has the strongest evidence base for reducing heart attacks, strokes, dementia and cancer. Such a diet, also low in refined carbohydrates, may even be the best for athletic performance, according to one of the most respected sports medicine scientists in the world, Professor Timothy Noakes, of South Africa. Prof Noakes discusses its merits in Cereal Killers, a documentary that has its UK premiere in London tomorrow. It has been adopted by the Australian cricket team under the guidance of their physician, Dr Peter Brukner, and it clearly hasn't done them much harm in their recent performances.

Meanwhile, Big Food continues to peddle pathology with impunity, spending billions targeting children with junk food advertisements and even co-opting "respected" scientific bodies. Last week, Professor Ian McDonald, chair of the UK's Scientific Advisory Committee on nutrition, who has admitted to receiving substantial amounts in research funding from Coca-Cola and Mars, declared that his board will ignore the new guidance from the WHO on sugar limits. How he can continue in his current role when his credibility has been so badly compromised is beyond me.

When I met health secretary Jeremy Hunt a few weeks ago, he said there was no silver bullet to tackling the obesity epidemic and wanted a plan of action. But he already has one. It's been just over a year since Britain's doctors submitted a 10-point plan, following a one-year review of the evidence on policies to tackle the obesity epidemic. These included a tax on **sugary drinks,** banning junk food advertising to children, restrictions on fast food outlets near schools and compulsory nutritional standards in hospitals.

Not a single proposal has been implemented. Political ideology continues to trump scientific evidence and the interests of Big Food continue to take precedence over our children's health. According to Britain's chief medical officer, Dame Sally Davies, our continued failure to act may result in the first generation of children that will be outlived by their parents. That is a truly chilling prospect that can no longer be ignored.

Aseem Malhotra is a cardiologist and science

director of Action on Sugartrebeled

Captions:

A study last month revealed that those who consume more than 25% of calories from added sugar trebled their risk of cardiovascular death.

Alamy

LOAD-DATE: March 15, 2014

LANGUAGE: ENGLISH

DAILY MAIL (London)

March 15, 2014 Saturday

END OF AISLE BOOZE DISPLAYS FACE BAN TO BEAT OBESITY

BYLINE: BY SEAN POULTER CONSUMER AFFAIRS EDITOR

LENGTH: 447 words

SUPERMARKETS could be blocked from putting **sugary drinks** and alcohol in eye-catching positions in a bid to curb consumption.

Researchers found that sales of **fizzy drinks** soared by 52 per cent when displayed at the end of aisles. Similarly, sales of drink rose by up to 46 per cent when placed at the end rather than in the middle of the aisle.

The study, which was funded by the Department of Health, will inform decisions by ministers who are under fierce pressure to tackle obesity and the health problems associated with sugar and alcohol.

Dame Sally Davies, the chief medical officer, recently suggested it may be necessary to impose a sugar tax on **fizzy drinks** and other foods to curb consumption. Separately, David Cameron is under pressure from the medical profession to impose a minimum price on alcohol amid concerns over binge drinking.

However, the new research suggests that a meaningful reduction in consumption could be delivered by simply putting controls on where products are placed. At the same time, a decision by stores to put healthy foods, such as fruit, in premium positions could boost sales and improve the nation's diet.

The researchers from the Behaviour and Health Research Unit (BHRU) based at Cambridge University used sales data for beer, wine and spirits as well as three non-alcoholic options - **fizzy drinks**, coffee and tea - from a branch of a major supermarket.

After taking into account prices, promotions and a number of display locations, they found that sales of **fizzy drinks** soared by 51.7 per cent when placed at the end of an aisle. The rise for coffee was even higher at 73.5 per cent, while tea sales rose 113.8 per cent.

For alcohol, sales of beer went up by 23.2 per cent, wine by 33.6 per cent and spirits by 46.1 per cent.

Supermarkets have long been aware that certain positions generate stronger sales. However, this is the first time that independent scientific research has quantified the remarkable impact on products linked to diet and health. Dr Ryota Nakamura, from the BHRU, whose research was published in the journal Social Sciences and Medicine, said controls on where products are placed in stores may be necessary.

Our study shows, for the first time, that these types of displays dramatically influence people's decisions to purchase alcohol and carbonated drinks,' he said. Prohibiting or limiting this marketing tactic for less healthy options, or utilising this for healthier ones, holds the promising possibility of encouraging healthier lifestyle choices.'

A spokesman for the British Retail Consortium said retailers lead the way in clear labelling and advice to customers to help them eat and drink responsibly'.

© Daily Mail

LOAD-DATE: March 14, 2014

LANGUAGE: ENGLISH

PUBLICATION-TYPE: Papers

Copyright 2014 Associated Newspapers Ltd.
All Rights Reserved

The Times (London)

March 15, 2014 Saturday
Edition 1;
National Edition

News on sugar delivered without a sweetener

BYLINE: Rose Wild Feedback

SECTION: NEWS; OPINION, COLUMNS; Pg. 27

LENGTH: 462 words

Readers of a pedantic bent - oh yes, we have a few - were provoked to much mirth by our recent headline, "Too much sleep bad for you". Francis Harvey of Bristol summed up the general mood by quoting Stephen Fry, from the BBC TV series A Bit of Fry and Laurie: "Well of course too much is bad for you. That's what 'too much' means, you blithering t***."

But how do we know how much is too much? Our efforts to set out guidelines for healthy sugar consumption seem only to have added to the confusion of the nation, according to Carolyn Livesey from Crail, Anstruther.

"I won't be sticking your 'Good Sugar Guide' to my fridge," she writes. "The foods you recommend as healthy do not take into account overall nutritional content, particularly the children's food. You recommend a 'light' cheese, although growing children should not be fed low-fat options unless they have an obesity problem. Sugar-free strawberry jelly is nutrition-free and full of artificial sweeteners. Salt and vinegar Ryvita minis? High salt is just as bad as high sugar. By the way, I challenge you to eat rhubarb without about double its weight in sugar."

Bleugh, I can feel the fur on my tongue just reading about it.

Professor Simon Smail from Cardiff says we should have made clear the real dangers lie in added sugar. "The draft World Health Organisation guidance applies to all monosaccharides (such as glucose, fructose) and disaccharides (such as sucrose or table sugar) added to food during preparation, as well as sugars that are naturally present in honey, syrups, fruit juices and fruit concentrates. It does not include the sugar in whole foods, such as fruit."

So what about that guilty banana in the red alert section of the chart? "Your guide treats sugar in whole fruit as equivalent to the sugar added to food items (biscuits, drinks, etc). The effect on the body of sugar in whole fruit, eg, a banana, is much less damaging than consuming the same amount of added sugar in a **fizzy drink** or a fruit drink. Most dietitians regard the banana as an excellent food, and it is one of the fruits that children will eat happily, so to denigrate it is a real shame."

Ursula Arens, who is features editor of Network Health Dietitians magazine, agrees on the fizzy pop: "Cutting out sugar-sweetened drinks and juices from the typical UK diet would cut added sugar intakes by 24-40 per cent (adults v teenage diet), which would easily achieve the WHO guideline." That's good news for me, since I scarcely touch the stuff. Unless, hang on, how much sugar is there in Australian shiraz? In the end it comes down to common sense, doesn't it? I'm with Deborah Fuller of Holloway, London: "The answer is surely to eat a good mixed diet with some raw foods and avoid overly processed food whenever possible."

LOAD-DATE: March 15, 2014

LANGUAGE: ENGLISH

PUBLICATION-TYPE: Newspaper

JOURNAL-CODE: TIM

Copyright 2014 Times Newspapers Limited
All Rights Reserved

THE TIMES
The Times (London)

March 15, 2014 Saturday
Edition 1;
National Edition

Coke is confronting the hard truths about soft drinks, starting from zero;
Calls for a sugar tax don't dismay Jon Woods who warns that it won't solve obesity problems, writes Dominic Walsh

BYLINE: Dominic Walsh

SECTION: NEWS; Pg. 53

LENGTH: 1386 words

Not so long ago, the issue of the number of calories in its **fizzy drinks** would have sent Coca-Cola executives reaching for their hard hats. The rising tide of obesity, particularly among children, was an uncomfortable subject that the world's biggest soft drinks maker struggled to deal with.

Not any more. These days, even fresh calls for a **sugar** tax in Britain, this time from Dame Sally Davies, the chief medical officer, do not throw Jon Woods, head of the **soft drinks** giant's British operations. Indeed, he is all too eager to talk about it.

Brandishing a can of Coke Zero, the company's fast-growing zero-calorie cola, Mr Woods declares confidently: "Clearly there's a lot of concern around calories and obesity, particularly in the UK. Coke Zero is one of the ways we can work to address it."

The Northern Irishman's sales and marketing background, honed during spells with Cadbury Schweppes and what is now the brewing giant Anheuser-Busch InBev, has left him well equipped to deal with the issue head-on as general manager of Coca-Cola GB and Ireland.

Does he understand the calls for a sugar tax on food and drink companies?

"I understand the concern. There's an obesity issue. The numbers are quite startling - 60 per cent of adults are overweight or obese and 30 per cent of children are overweight or obese.

"What I believe is causing it is a fundamental imbalance between the calories people are consuming and the calories they are expending. But that's quite a broad issue. It's not going to be tackled by a single initiative. You talk about a soft drinks tax, but that's not going to tackle obesity."

He quotes statistics suggesting that soft drinks account for 2 per cent of an average adult's diet. "To put it into context, alcohol accounts for 8 per cent. So I think it's hard to lay the blame for obesity at the door of just soft drinks. Over the last ten years consumption of regular soft drinks has declined by 9 per cent, yet obesity rates keep going up. So I think it's hard to draw a direct comparison."

Nevertheless, Mr Woods is adamant that Coca-Cola will play its part in the war on obesity. Under the Government's responsibility deal, Coca-Cola GB has signed up to a commitment to reduce the calories per litre in its **fizzy drinks** - it also owns brands such as Fanta and Sprite - by 5 per cent by the end of this year. To achieve that, it is spending £15 million reformulating some of its products. Last year, it relaunched Sprite with 30 per cent fewer calories after switching to using a natural zero-calorie sweetener made from the stevia plant.

Could it use stevia in its core Coke drink?

"I don't think we need to, because we've got two great variants of Coke which are already zero calorie. On Coke, we're seeing most of our growth coming from Diet Coke and Coke Zero, which account for 40 per cent of our cola volumes, so we're seeing a gradual reduction in calories per litre as the mix changes within the three Cokes."

There's also the issue of taste to consider. "Ultimately, if you want a growing business, which I do in the UK, we have to produce products that taste great. Because if they don't taste great, nobody buys them."

Under the responsible marketing code, the company has committed not to promote or advertise its brands directly to children, for whom **fizzy drinks** account for 4 per cent of calorie intake. Above all, the company is making sure consumers who do want to buy its more **sugary drinks** know exactly what they're putting in their shopping baskets. "We put clear information on what's in our drinks and the guideline daily amounts front and centre on the pack."

Last year it launched an advertising campaign on mainstream television focusing openly on the 139 calories in a standard can of Coke. "Nobody had ever done that before. It was probably quite brave. I think it was the first time we really felt comfortable about going out and talking about the calories in our product."

Hand-in-glove with its investment in reducing calories in its drinks, Coke GB is also accelerating its focus on the other side of the equation - "calories-out" - by encouraging people to burn more calories by leading more active lives. Today it is announcing plans to invest £20 million over the next five years to help get a million people to become more active through "the delivery of inspiring and engaging community based physical activity programmes".

Mr Woods, a keen mountain-biker, says that the statistics for physical exercise in the UK make for "pretty uncomfortable" reading. "It's not for my business to preach to my consumers that they need to be more physically active. But if you have a brand that can connect with people, and you have the marketing skill we have, you can encourage people to do more."

Coke's long-standing links with sport through its sponsorship of the Olympic Games and the World Cup have given it a huge leg-up in this respect and this week Coke GB has been closely involved in organising the British leg of the Fifa World Cup Trophy Tour, which will see the trophy visit 90 countries before returning to Brazil in time for the tournament this summer.

Despite their American ownership, both Coca-Cola GB and its British bottling franchisee, the separately quoted Coca-Cola Enterprises, see themselves as playing a key role in the British economy. The statistics are certainly compelling. Between them, the two organisations employ more than 4,000 people, most at CCE's six manufacturing plants, including Europe's biggest soft drinks plant, in Wakefield.

Ninety-seven per cent of the products sold by Coke in Britain are made in the country and 95 per cent of its suppliers are based in Britain. It has almost a quarter of the British soft drinks market, selling about 500 million unit cases and delivering annual turnover of about £1.5 billion, in turn contributing £2.4 billion of value to the British economy. All of which makes it easily one of the Atlanta-based global giant's top ten markets.

Mr Woods says that the business - comprising 21 brands, including Innocent smoothies, Ocean Spray cranberry juice and Abbey Well water - has continued to grow during recent recessionary times, increasing total volumes by 3.5 per cent last year. Coke Zero grew by 26 per cent and is predicted to grow by a double-digit percentage this year on the back of heavy marketing investment, while it sold 22 million of the new 250ml Coca-Cola cans, sold at 49p compared with about 65p for the standard 330ml can.

To keep ahead of what Mr Woods admits is a very competitive market, Coke GB spent £33 million on media last year, much of its focused on its "lights" like Coke Zero, and he expects that to rise by another 5 per cent this year. He says he is "cautiously positive" on consumer confidence, although he admits that keeping on top of the obesity issue is just as critical to Coca-Cola in Britain.

Some experts have suggested that the big multinationals such as Coke have been used as scapegoats in the obesity debate, even demonised, although Mr Woods is happy to tackle the issue head on. "We're the biggest brand so we tend to get quite a lot of attention, that's for sure, but I don't feel picked on. I think it's up to me and my business to earn the trust of the people who consume our drinks every day."

Q&A

Who is your mentor?

I don't have one specific mentor, but I am fortunate to have learnt from many great business leaders, all of whom I am still in touch with today Does money motivate you?

Yes it does, but if I didn't enjoy how I spend my days, I would find something that I do enjoy

What was the most important event in your working life?No single moment, but starting at Cadbury's as a shift manager at the Crunchie plant provided me with a great insight into managing people What does leadership mean to you?

Building a great team and delivering results

How do you relax?

Mountain biking

What gadget must you have?

Has to be my bike - a Ghost RT Lector What is your favourite television programme?

Top Gear - it's the one show where I laugh at the same things as my 15-yearold son

CV

Age 45

Education Campbell College, Belfast; Loughborough University Career 1990-97 Cadbury Schweppes; 1997-2004 Anheuser-Busch InBev; 2005-present Coca-Cola GB & Ireland, business development director, then general manager from 2010 Family married, with a son and a daughter

LOAD-DATE: March 15, 2014

LANGUAGE: ENGLISH

GRAPHIC: Jon Woods shares the public's concern about rising obesity levels, and the Coca-Cola GB boss wants his company to play its part in addressing the problem
TIMES PHOTOGRAPHER, PAUL ROGERS

PUBLICATION-TYPE: Newspaper

JOURNAL-CODE: TIM

Copyright 2014 Times Newspapers Limited

All Rights Reserved

Daily Record & Sunday Mail

March 14, 2014 Friday
Edition 1;
National Edition

Sweet and sour talks

SECTION: FEATURES; Pg. 17

LENGTH: 109 words

CLEARLY the government are taking the need to reduce our sugar intake very, very seriously.

So seriously, they have a panel of eight eminent experts looking at the issue.

I'm sure it's helpful that six of them, including chairman Professor Ian MacDonald, have strong connections to the food and fizzy drink industries.

And that their decision to ignore the World Health Organisation's advice to halve the amount we consume has nothing whatsoever to do with their links to those who shove most of the sugary stuff down our throats.

Nor will the fact that the prof's research funding has come from companies such as Wall's ice cream, influence him. The very idea.

LOAD-DATE: March 14, 2014

LANGUAGE: ENGLISH

PUBLICATION-TYPE: Newspaper

JOURNAL-CODE: DRC

Copyright 2014 Scottish Daily Record & Sunday Mail Ltd
All Rights Reserved

MailOnline

MailOnline

March 14, 2014 Friday 4:12 PM GMT

Putting alcohol and fizzy drinks at the end of supermarket aisles DOES make us buy more - so could moving them reduce obesity?

BYLINE: SEAN POULTER, CONSUMER AFFAIRS EDITOR

SECTION: HEALTH

LENGTH: 774 words

- Sales of **fizzy drinks** increase 52% when they are put at the end of aisles
- Sales of alcohol increase 46% when it is placed at the end of aisles
- Coffee sales soar 74% when it is put at the end of the aisle
- As a result, supermarkets could be banned from putting alcohol and **fizzy drinks** in these premium locations in a bid to reduce consumption

Supermarkets could be blocked from putting **sugary drinks** and alcohol in premium eye-catching positions in a bid to curb consumption.

New research found that sales of **fizzy drinks** like Coca-Cola, Pepsi and Fanta soar by 52 per cent when placed at the end of aisles.

Similarly, sales of alcohol rise by up to 46 per cent when placed at the end of the aisle rather than in the middle.

The study, which was funded by the Department of Health, will inform decisions by ministers who are under fierce pressure to tackle obesity and the health problems associated with sugar and alcohol.

The researchers involved were from the Behaviour and Health Research Unit (BHRU), which is a collaboration between the University of Cambridge, the University of East Anglia, and MRC Human Nutrition Research.

The government's chief medical officer, Dame Sally Davies, recently suggested it may be necessary to impose a sugar tax on **fizzy drinks** and other foods to curb consumption.

Separately, David Cameron is under fierce pressure from the medical profession to impose a minimum price on alcohol to remove the cheapest beer and spirits from sale amid concerns about binge drinking.

However, the BHRU research suggests a meaningful reduction in consumption could be delivered by simply putting controls on where products are placed.

At the same time, a decision by stores to put healthy foods, such as fruit, in these premium positions could boost sales and improve the nation's diet.

The researchers used sales data for beer, wine and spirits as well as three non-alcoholic options - **fizzy drinks**, coffee and tea - from a branch of a major high street supermarket.

After taking into account prices, promotions and a number of display locations, they found that sales of **fizzy drinks** soared by 51.7 per cent when placed at the end of an aisle. The rise for coffee was even higher at 73.5 per cent, while tea sales rose 113.8 per cent.

In terms of drink, sales of beer went up by 23.2 per cent, wine by 33.6 per cent and spirits by 46.1 per cent.

Supermarkets have long been aware that certain shelves generate stronger sales and many charge big brands a fee to ensure their products are seen at eye level.

However, this is the first time that independent scientific research has been able to quantify the remarkable impact on products linked to diet and health.

Dr Ryota Nakamura, from the BHRU and the University of East Anglia, said controls on where products are placed in stores may be necessary.

'Our study shows, for the first time, that these types of displays dramatically influence people's decisions to purchase alcohol and carbonated drinks,' he said.

'Prohibiting or limiting this marketing tactic for less healthy options, or utilising this for healthier ones, holds the promising possibility of encouraging healthier lifestyle choices.'

A debate about where products are promoted in stores is already raging around the placing of sweets, drinks and snacks that are high in sugar at the check-outs, where they capture the attention of children.

Former public health minister, Anna Soubry, made efforts to get rid of what she called 'guilt aisles' while in post. David Cameron has also been critical of chains like WH Smith for offering chocolate oranges at checkouts.

Research published by the 'Junk Free Checkouts' campaign last year found eight in ten parents were unhappy that stores continue to fuel obesity and pester power by putting sweets at the tills.

The BHRU research team also found that price does have an impact on sales. For example, for every one per cent reduction in the price of beer there was a 5.6 per cent rise in sales.

Similarly, there was a 5.2 per cent rise in sales of wine and five per cent for spirits.

Lower prices were a smaller factor for non-alcoholic drinks. A one per cent price drop saw a 2.3 per cent rise in sales of **fizzy drinks**, two per cent for coffee and 1.8 per cent for tea.

The director of the BHRU, Professor Theresa Marteau, from Cambridge, said: 'Although we often assume price is the biggest factor in purchase choices, end-of-aisle displays may play a far greater role.

'It would therefore make sense that any intervention to curb the consumption of alcohol and sugar-sweetened drinks takes this into consideration.'

The research is published in the journal Social Sciences and Medicine.

LOAD-DATE: March 14, 2014

LANGUAGE: ENGLISH

PUBLICATION-TYPE: Web Publication

JOURNAL-CODE: WEBDM

Copyright 2014 Associated Newspapers Ltd.
All Rights Reserved

MailOnline

March 14, 2014 Friday 3:22 PM GMT

Americans believe marijuana is LESS harmful than sugar, says new survey

BYLINE: DAILY MAIL REPORTER

SECTION: NEWS

LENGTH: 420 words

- Sugar ranked as more harmful than pot but less harmful than tobacco and alcohol
- Survey questioned 1,000 people on their opinions
- Latest hit to sugar as officials blame it for public health epidemic of obesity

Americans now believe marijuana is so harmless that they ranked sugar as more harmful to a person's health in a recent poll.

The survey questioned 1,000 adults on a wide variety of topics ranging from health to politics to religion.

One of the questions asked which substance 'is more harmful to a person's overall health:' marijuana, sugar, tobacco or alcohol?

Scroll down for video

According to the results, tallied by NBC News and the Wall Street Journal, 49 per cent ranked tobacco as most harmful followed by alcohol at 24 per cent, sugar at 15 per cent, marijuana at 8 per cent, all at 3 per cent, and not sure at 1 per cent.

The full poll can be read here.

The results are just the latest evidence of sugar's image problem, with the sweet carb being called the 'new tobacco' by doctors earlier this year.

Food giants are being told to cut the amount of sugar they use because it has become the 'new tobacco'.

Doctors and academics say levels must be reduced by up to 30 per cent to halt a wave of disease and death.

They found that even zero-fat yoghurts can contain five teaspoons of sugar, while a can of Heinz tomato soup has four.

The equivalent of 11 teaspoons are found in a small Starbucks caramel Frappuccino with whipped cream. A Mars bar has eight.

'Sugar is the new tobacco,' said Simon Capewell, professor of clinical epidemiology at the University of Liverpool.

'Everywhere, **sugary drinks** and junk foods are now pressed on unsuspecting parents and children by a cynical industry focused on profit not health.

The obesity epidemic is generating a huge burden of disease and death.

However marijuana is not without health risks, as use can lead to early onset psychosis and the effects of use on the developing brain.

But there have been no documented deaths from marijuana overdose and there are medicinal applications for pot.

'Anyone who takes a truly objective look at the evidence surrounding these substances could not possibly arrive at any other conclusion,' Mason Tvert of the Marijuana Policy Project, told The Huffington Post.

'The public's understanding of marijuana is more in line with the facts than ever before. Marijuana is not entirely harmless, but there is no longer any doubt that it poses far less harm to the consumer than many of the legal products engrained in American culture.'

LOAD-DATE: March 14, 2014

LANGUAGE: ENGLISH

PUBLICATION-TYPE: Web Publication

JOURNAL-CODE: WEBDM

Copyright 2014 Associated Newspapers Ltd.
All Rights Reserved

MailOnline

March 12, 2014 Wednesday 2:51 PM GMT

Sugar adviser linked to Coca-Cola 'will not act' on UN guidance: Doctors and MPs accuse officials of 'immense arrogance' for ignoring suggested limits

BYLINE: SOPHIE BORLAND

SECTION: NEWS

LENGTH: 561 words

- World Health Organisation urging adults and children to half sugar intake
- Head of Government science panel Professor Ian MacDonald, who used to work for Coca Cola, will 'take note' of advice
- Critics claim revelation shows Government too close to big businesses

A radical UN recommendation to halve sugar intake will not be implemented in Britain says a Whitehall adviser on nutrition who has worked for Mars and Coca-Cola.

Professor Ian MacDonald, head of a panel of health experts in charge of drawing up guidelines on sugar, said it will 'not act' on the World Health Organisation's proposal.

The move led to fury yesterday as senior doctors and MPs accused officials of 'immense arrogance' for ignoring the suggested limit of six teaspoons a day, in the face of an obesity crisis that threatens to overwhelm the NHS.

And campaigners last night accused Professor MacDonald - who only recently left the pay of the two fast food giants - of being 'in the pocket' of the sugar industry.

He is one of six scientists on the panel of eight who have links to manufacturers of sugary foods, including the world's largest chocolate maker and **fizzy drinks** producers.

The row comes amid growing concern over the high levels of sugar in everyday foods, which experts believe is a major contributor to obesity, heart disease and diabetes.

Around a quarter of adults in Britain are obese but this is predicted to soar to more than half the population by 2050 and cost the economy £50billion a year.

An average adult eats between 11 and 12 teaspoons of sugar a day - double the new recommendation - while children consume as many as 15.

But referring to the WHO guidelines, Professor MacDonald said: 'The position, I've been informed by the officials, is that actually we would take note of it but we would not act on it.'

He added that the Government would take the recommendations of his own panel, the Carbohydrate Working Group of the Scientific Advisory Committee on Nutrition, rather than those of the WHO.

Graham MacGregor, of the group Action on Sugar and professor of cardiology at the Wolfson Institute of Preventive Medicine, accused Professor MacDonald of 'arrogance' about the extent of the health crisis posed by sugar.

He said: 'To say that he is going to ignore the WHO has an immense amount of arrogance about it.

'Given the fact we are facing an obesity crisis, what is his plan? ... it's a real timebomb.

'Obesity and diabetes are going to overwhelm the health service in terms of cost. We have to do something.'

Tam Fry, of the National Obesity Forum, said: 'Ian MacDonald is a leading figure in his discipline.

'The problem is his research money is so dependent on these firms that you have to ask the question, is he in their pocket?'

Luciana Berger MP, Labour's shadow public health minister, accused the Government of being 'too close to big businesses to do what's best for our nation's health'.

The SACN is expected to produce its own sugar guidelines in June.

- An earlier version of this article stated that Professor Ian Young had received funding from Sugar Nutrition UK. In fact, Professor Young received funding in the years up to and including 2008 from the Sugar Bureau, which renamed itself as Sugar Nutrition UK in January 2012. We are happy to make clear that Professor Young did not receive funding from the Sugar Bureau or Sugar Nutrition UK after 2008.

LOAD-DATE: March 12, 2014

LANGUAGE: ENGLISH

PUBLICATION-TYPE: Web Publication

JOURNAL-CODE: WEBDM

Copyright 2014 Associated Newspapers Ltd.
All Rights Reserved

DAILY MAIL (London)

March 10, 2014 Monday

SUGAR ADVISER LINKED TO COCA-COLA WILL NOT ACT' ON UN GUIDANCE

BYLINE: BY SOPHIE BORLAND HEALTH REPORTER

LENGTH: 450 words

THE radical UN recommendation to halve sugar intake will not be implemented in Britain says a Whitehall adviser on nutrition who has worked for Mars and Coca-Cola.

Professor Ian MacDonald, head of a panel of health experts in charge of drawing up guidelines on sugar, said it will not act' on the World Health Organisation's proposal.

The move led to fury yesterday as senior doctors and MPs accused officials of immense arrogance' for ignoring the suggested limit of six teaspoons a day, in the face of an obesity crisis that threatens to overwhelm the NHS.

And campaigners last night accused Professor MacDonald - who only recently left the pay of the two fast food giants - of being in the pocket' of the sugar industry.

He is one of six scientists on the panel of eight who have links to manufacturers of sugary foods, including the world's largest chocolate maker and **fizzy drinks** producers.

The row comes amid growing concern over the high levels of sugar in everyday foods, which experts believe is a major contributor to obesity, heart disease and diabetes.

Around a quarter of adults in Britain are obese but this is predicted to soar to more than half the population by 2050 and cost the economy £50billion a year.

An average adult eats between 11 and 12 teaspoons of sugar a day - double the new recommendation - while children consume as many as 15. But referring to the WHO guidelines, Professor MacDonald said: The position, I've been informed by the officials, is that actually we would take note of it but we would not act on it.'

He added that the Government would take the recommendations of his own panel, the Carbohydrate Working Group of the Scientific Advisory Committee on Nutrition, rather than those of the WHO.

Graham MacGregor, of the group Action on Sugar and professor of cardiology at the Wolfson Institute of Preventive Medicine, accused Professor MacDonald of arrogance' about the extent of the health crisis posed by sugar. He said: To say that he is going to ignore the WHO has an immense amount of arrogance about it. Given the fact we are facing an obesity crisis, what is his plan??...?it's a real timebomb?...? Obesity and diabetes are going to overwhelm the health service in terms of cost. We have to do something.'

Tam Fry, of the National Obesity Forum, said: Ian MacDonald is a leading figure in his discipline. The problem is his research money is so dependent on these firms that you have to ask the question, is he in their pocket?'

Luciana Berger MP, Labour's shadow public health minister, accused the Government of being too close to big businesses to do what's best for our nation's health'.

The SACN is expected to produce its own sugar guidelines in June.

© Daily Mail

LOAD-DATE: March 9, 2014

LANGUAGE: ENGLISH

PUBLICATION-TYPE: Papers

Copyright 2014 Associated Newspapers Ltd.
All Rights Reserved

THE SUNDAY TIMES
The Sunday Times (London)

March 9, 2014 Sunday
Edition 1;
National Edition

Ignore the warring food police and trust your gut instinct

BYLINE: INDIA KNIGHT

SECTION: FEATURES; Pg. 22

LENGTH: 803 words

Abaffling and alarming week of headlines for people who eat food - which is to say, er, everyone. The big news was that high-protein, low-carb diets were "as bad for you as smoking" - a patently ludicrous assertion, given that nobody dies from eating sea bass and broccoli.

A couple of days later came a new headline, this time screaming that low-fat diets do not curb heart disease or help you live longer and are a complete waste of time because the real culprits are ... carbohydrates and sugar.

You can see the impasse: a low-carb diet - restricting carbohydrates, shunning sugar and embracing "good fats" such as olive oil - is as bad for you as smoking, but eating carbs and sugar does nothing for you either.

By the end of the week people up and down the country were gazing at their breakfast, lunch and dinner in despair. The mere act of eating had become a minefield.

A few years ago, having lost a fairly dramatic amount of weight quickly and painlessly, I wrote a low-carb diet book - so, yes, I'm biased. Speak, as I did, to any doctor, any nutritionist, and a number of things become crystal clear.

One, sugar isn't anybody's friend, whether it's in **fizzy drinks** or natural produce (organic fruit juice can still rot your teeth). Two, stodge makes you fat. Three, it's not a good idea to eat Frankenfoods that have been so manipulated that they bear no resemblance to anything you might find in nature. If you can't pronounce, let alone recognise, the 22 ingredients in your "healthy" snack, best put it back. Four, vegetables, the greener and leafier the better, are good for you. Five, fake sugar is worse than real sugar. Six, "diet" anything is worse than the real version.

These are hardly earthshattering aperçus: they are how people used to eat. If your granny felt a bit tubby, she would dump the cakes, biscuits and potatoes for a bit and lose weight. In France women who are watching their weight don't eat croissants for breakfast but they do eat steak, fish and vegetables for lunch and have cheese for pudding. Not all carbohydrates are created equal: complex carbs - brown things, basically - are fine, whereas processed carbs, or white things, are not.

The matter has become complicated because people have melodramatic views of lowcarb diets. They think a typical meal might involve a steak fried in butter, with a creambased sauce, a fried egg on top and a side of chicken wings with a mayonnaise dip - as opposed to, say, a giant spinach salad and a piece of fish. This presupposes that people have no common sense: surely by now everyone knows that ramming cheap, antiobiotic-pumped (and the rest) red meat down your gullet three times a day isn't a brilliant idea.

The American writer Michael Pollan, a vocal critic of US food policies, put it neatly back in 2006 when he wrote: "Eat food, not too much, mostly plants."

"Food" here refers to real, recognisable food, meat and fish included. (Pollan is also good on "the French paradox". He wrote: "They have better heart health than we do despite being a cheese-eating, wine-swilling, foie grasgobbling people. The American paradox is we are a people who worry unreasonably about dietary health yet have the worst diet in the world.") Pollan aside, I really hate these mad food edicts that pop up all over the headlines three times a year. What I hate even more is that we have become so anxious about the food we eat, so obsessed with health and weight control, that we have thrown all common sense out of the window.

Deep down we all know how to eat. We know that a home-made wholemeal loaf is better than plastic white bread. We know that giving a child a boiled egg is healthier than giving them a bowl of cereal (up to six teaspoons of sugar a serving. Yes, even the "healthy" ones). We know that "convenience" food is likely to inconvenience our health in the long term.

What I hate most of all about this is that food, one of life's greatest pleasures and joys, has become medicalised. You know what? We all die in the end. Your stupid chia seeds aren't going to save you and neither is your batty, joy-denying extreme diet, which, by the way, is likely to give you brittle bones in late middle age.

We all know how to eat - or, at least, we used to. When you feel battered by every headline, every health scare, every "healthy eating guideline" - eggs are the devil! Oh no they're not. Red wine will kill you! Oops, sorry, reverse that - there's only one thing to do: go with your instinct. Eat food you trust. Have the odd blowout and wake up face-down in a tray of doughnuts, by all means. But eat sensibly overall.

When you don't know who or what to trust, the answer is staring you in the face: trust yourself.

@indiaknight

IN FRANCE WOMEN WHO ARE WATCHING THEIR WEIGHT DON'T EAT CROISSANTS BUT THEY DO EAT STEAK AND HAVE CHEESE FOR PUDDING

LOAD-DATE: March 11, 2014

LANGUAGE: ENGLISH

GRAPHIC: Food is one of life's great pleasures - eating what you like, in moderation, is not going to kill you

PUBLICATION-TYPE: Newspaper

JOURNAL-CODE: STS

Copyright 2014 Times Newspapers Limited
All Rights Reserved

194 of 280 DOCUMENTS

DAILY MAIL (London)

March 7, 2014 Friday

A LETHALLY SWEET RELATIONSHIP

LENGTH: 1356 words

AN EXCORIATING ATTACK

A CARDIOLOGIST ON THE COSY LINKS BETWEEN POLITICIANS AND THE FOOD GIANTS, WHOSE REFUSAL TO CUT SUGAR LEVELS IS CAUSING COUNTLESS DEATHS

BY DR ASEEM MALHOTRA

On paper, you would think it had been a good week for those with serious concerns about the amounts of sugar that so many of us are consuming.

On Wednesday, for instance, no less an authority than the UN's World Health Organisation came out with the firm recommendation that we should all be aiming to cut our sugar intake by half and that children should not be given **fizzy drinks** at all.

That came just one day after Dame Sally Davies, the Government's Chief Medical Officer, proposed that a sugar tax needed to be introduced if we wanted to cut sugar intake and reduce obesity.

And it came on the same day that an eminent New York cardiovascular research scientist warned that the long-running demonisation of fats, and saturated fats in particular, could be entirely misplaced.

The real killer, according to Dr James DaNicolantio, particularly when it comes to heart disease and Type 2 diabetes those two scourges of the modern age is sugar.

After these three dramatic interventions, surely it is game, set and match for those of us who would like to see sugar consumption seriously reduced? Well, as a cardiologist and science director of the campaigning health group, Action On Sugar, I can tell you that we're certainly not dancing in the streets just yet.

For despite the mounting evidence of the damage to health done by sugar evidence that many working in the field would now describe as overwhelming this was the response from the Prime Minister's official spokesman on Wednesday: What we are doing is working with the industry. You have already seen commitments from retailers and food manufacturers to reduce levels of salt, to remove some artificial fats, to reduce calorie content and improve labelling.'

It's the first sentence what we are doing is working with the industry' that gives the game away because it's absolutely true.

The links between Government ministers and food manufacturers, and indeed between scientists who are supposed to advise the Government and the food manufacturers, are nothing short of astonishing. For these are the same food manufacturers who have been adding extra sugar to processed foods, confectionery and **fizzy drinks** for decades.

It is the closeness of those links that are widely blamed for a compulsory food traffic-light system an idea once enthusiastically championed by the Food Standards Agency and designed to give shoppers an idea of the nutritional value (or not) of the item they were about to buy quietly dying a death soon after the Coalition came to power.

But then what do we expect when the London Olympic Games, the biggest celebration of fitness and health in this country for decades, was sponsored by Coca-Cola and McDonald's?

And what do we expect when, as this newspaper revealed only last month, fast-food companies, supermarkets and restaurant chains have had dozens of meetings with ministers since the last election?

Nando's, Tesco, Pepsi, Mars and, almost inevitably, the ubiquitous McDonald's are among those who have had the chance to bend the ministerial ear since 2010

.

Government ministers hate their close links to the food industry being exposed by the media. But these links exist and are one reason, I believe, for the Government's lax approach.

Too often, when a minister pronounces on dietary matters, I wonder whether he is just saying what his chums in the food industry have told him to say. Meanwhile, rates of obesity, Type 2 diabetes, heart disease, stroke and cancer all conditions with increasingly strong links to sugar intake continue to rise.

The Food and Drink Federation, the industry lobby group, for instance, has had 16 meeting with ministers since the last election and an astonishing 99 with Government officials. By contrast, we in Action On Sugar have had just one, although a second, we're told, is in the pipeline.

There's no way of knowing who has met whom, but there is also no hiding the conflicts of interest.

Bill Morgan, special adviser to former Health Secretary Andrew Lansley, for instance, came from a PR firm that has Coca-Cola, Kraft Foods and Tesco on its books, although the PR company has always denied that Mr Morgan worked on two of those accounts.

But its website did highlight his potential for linking the interests of its clients with Government policy.

Even Mr Lansley, himself, architect of the Government's Responsibility Deal', which effectively handed responsibility for the levels of fats, salt and sugar in food to the food manufacturers themselves, has past links with the industry. In 2009, while still Shadow Health Secretary, he was director of a marketing firm where clients included Pepsi, Mars, Pizza Hut and Diageo's Guinness.

Earlier this year, Channel 4's Dispatches programme revealed that five of the eight members of the Government's Scientific Advisory Committee of Nutrition (SACN) received substantial funding from large confectionary companies.

These included the committee's chairman, Professor Ian Macdonald, who receives money not only from Unilever, the world's biggest ice-cream maker, but also from Coca-Cola and Mars.

His committee colleague, Professor Tom Sanders, one of the Government's most trusted advisers when it comes to diet, sugar and heart disease, has had his research funded to the tune of £4.5 million by the sugar giant Tate & Lyle.

There is, of course, no evidence or indeed, any suggestion that members of the SACN have ever put the interests of the companies that sponsor them first.

But the committee has gone against prevailing scientific opinion as rates of obesity and diet-related diabetes have grown and its reports are said to be one of the main reasons why official health advice on the amounts of sugar we eat haven't changed in 11 years.

This advice suggests that an individual can consume a staggering 22 teaspoons of sugar a day as part of a guideline daily amount. On Wednesday, the World Health Organisation suggested that figure should be just six teaspoons. The difference is huge and, in the face of the dramatic intervention by the WHO, I wonder if the SACN's committee's recommendations will suddenly be changing now?

On Wednesday, I was due to go on Channel 4 News to discuss the WHO's new recommendations but the debate was cancelled when the Food and Drink Federation (FDF) withdrew, apparently after discovering that I would be up against them.

Had they turned up, I might have pointed out that the relentless drive for profit among food companies had contributed to unnecessary suffering for millions of people and thousands of deaths that might otherwise have been prevented.

Moreover, there is growing evidence that not all calories are the same (contrary to what the food companies will tell you). Research from Stanford University last year showed that for every 150 sugar calories (from a can of Coke, for example) consumed above the recommended daily calorie intake, there is an 11-fold increase in the prevalence of Type 2 diabetes compared with 150 excess' calories consumed from a non-sugar source.

That increase is completely independent of body weight and levels of exercise: in other words, we are all vulnerable.

Yet still the Government does nothing. And as long as influential organisations such as the British Nutrition Foundation have a membership that reads like the Who's Who of the sugar industry Coca-Cola, Kellogg's McDonald's, Nestle and Pepsi among them and the British Dietetic Association can promote an Eat Well plate that includes a full-sugar can of cola and fruit juice as part of a balanced diet, I fear that won't change.

But change it must, for our health, and particularly the health of our children, depends upon it.

The WHO is absolutely right, we must cut down on our consumption of dietary sugar and it's high-time the Government cast aside its cosy relationship with the food industry, faced up to the unpalatable scientific facts and did the right thing.

? Dr Aseem Malhotra is Science Director of Action On Sugar

A leading cardiologist

© Daily Mail

LOAD-DATE: March 6, 2014

LANGUAGE: ENGLISH

PUBLICATION-TYPE: Papers

Copyright 2014 Associated Newspapers Ltd.
All Rights Reserved

195 of 280 DOCUMENTS

DAILY MAIL (London)

March 7, 2014 Friday

HOURS AFTER HEALTH EXPERTS TELL US TO HALVE OUR SUGAR INTAKE...

BYLINE: BY LAURA CLARK AND DANIEL MARTIN

LENGTH: 773 words

A STRICT limit on sugar in pupils' lunches is to be scrapped in a major overhaul of school meals, it emerged yesterday.

The decision, which provoked an outcry from health and obesity experts, comes less than a day after World Health Organisation chiefs demanded a sharp cut in children's sugar intake.

Critics warned ministers against ignoring the WHO advice and wasting a chance to slash sugar intake. One MP described the school lunch proposals, which are almost certain to be implemented, as puzzling to say the least'. Prescribed levels of fat and salt - as well as vitamins and minerals such as iron and calcium - are also set to be abandoned under the Department for Education plans.

Separate rules which dictate how many times certain items such as chips can be served during the week will remain in place. Those rules will be expanded and updated, for example to specify that wholegrain food must be offered each week to provide fibre to children. But more than a quarter of primary schools which trialled the proposed new system exceeded the old sugar limit - of four teaspoons per meal - by more than 10 per cent.

Nutritionists said robust' action is needed to meet a new target from the WHO, a subsidiary of the UN, to reduce adults' sugar intake to 12 teaspoons per day and children to six.

On Wednesday UN health chiefs went even further, and said adults should aim to halve this to six teaspoons and children less than six.

Unveiling its draft advice, WHO officials said sugar may pose the same threat to health as tobacco. It is increasingly implicated in obesity, heart disease and a range of other serious illnesses, they warned.

A single **fizzy drink** serving could exceed the six-teaspoon daily limit. At present, schools in England must comply with two sets of catering standards: one covering the frequency that specific items must be served during the week and another prescribing the levels of 14 nutrients in school lunches.

These nutrient standards stipulate that an average lunch in a primary must contain no more than 15.5g of sugar - equivalent to just under four teaspoons - and 18.9g for secondaries, or four-and-a-half teaspoons.

Maximum limits are also set for fat, saturated fat and sodium, and minimums for key nutrients such as iron, fibre, zinc, protein and fibre.

The Department for Education, which commissioned chefs Henry Dimbleby and John Vincent to review school food, said caterers find current rules over-complicated'.

A consultation paper published yesterday said they were difficult to understand' and led to schools paying £20 per recipe to be privately analysed. The nutrient-based standards can restrict cooks from being creative,' it added.

Under the plans, schools will no longer be required to prove they meet nutrient standards and will instead use new food-based standards to devise menus. These state, for example, that desserts, cakes and biscuits must be served only at lunchtime.

But in a trial of this approach, half of secondaries and a third of primaries failed to stay within the maximum sugar limit.

There would be strengthened practical guidance on portion sizes for desserts', in addition to existing limits on the quantity of fruit juices children can drink. Schools remain barred from offering **fizzy drinks**.

Professor Susan Jebb, an Oxford University nutritionist who helped draw up the new rules, said: If a school meets all of those food-based standards, it ought to be pretty close to meeting the nutrient standards we previously had. Schools can be confident they will be close to them.'

She said sugar consumption was already higher than the limit in some schools since cooks found the nutrient rules difficult to implement.

Newly-established academy schools will also be required to comply.

Tam Fry, from the National Obesity Forum, said: These guidelines come just days after the Chief Medical Officer [Sally Davies] confirmed sugar is the real disaster. The WHO report was our signpost to crack down on sugar.'

Katharine Jenner, a nutritionist at Queen Mary, University of London and Action for Sugar's campaign director, said the WHO's target was such a long way from where we are'.

She added: Challenging taste preferences in childhood is vital because it affects the rest of their lives.

We would want to look at this in detail and whether a child's diet at school will be worse than it was before.'

Labour MP Simon Danczuk said: I believe parents will be extremely disappointed by this decision.

Why the Government would want to reduce the healthiness of school meals is puzzling to say the least. The Government is going in the wrong direction.'

A sweet relationship - Page 15

© Daily Mail

LOAD-DATE: March 6, 2014

LANGUAGE: ENGLISH

PUBLICATION-TYPE: Papers

Copyright 2014 Associated Newspapers Ltd.
All Rights Reserved

The INDEPENDENT
Independent.co.uk

March 7, 2014 Friday 9:09 AM GMT

Mark Steel: Worried about what all the sugar in supermarket foods is doing to you? Don't be. At least it's not beef dripping; Sugar is in everything. Soon tap water will be flavoured with melted Yorkie bars

BYLINE: Mark Steel

SECTION: FRONTPAGE

LENGTH: 888 words

The World Health Organisation has said sugar is the "new tobacco", making us fat and useless, so now the experts are wondering how this could possibly have happened.

But one cause that may have contributed is that sugar is now in everything. Bread is full of sugar and soup is full of sugar and soon carrots will be full of ice cream and tap water will be flavoured with melted Yorkie bars, and new cereals are launched with adverts that go: "Everyone's asking why new Sweetypops are so pop-pop-popular. They're solid sugar lumps deep fried in treacle fat and glazed with concentrated Jaffa cakes then coated with five kinds of maple syrup and a doughnut. Scientists have said one bowl could contain up to 73 per cent of daily recommended goodness particles, and Sweetypops now come in new raspberry flavour, so it even counts towards your five a day."

The staff at coffee shop chains are instructed to ask everyone "would you like a muffin with that?", with a sorrow that makes you feel they'll be whipped if you don't have at least seven.

And everyone offers you chocolate. At one point, if you bought a Daily Telegraph in WH Smiths, you were told this entitled you to a half-price giant-size Toblerone, so whole villages in Surrey must have flooded as nutty dribbles oozed out of angry mouths spluttering about the need to bring back National Service. It's everywhere. I expect if you go to B&Q now you're asked: "Would you like a cinnamon bun with your shelving brackets?"

And the pressure on children is even greater. Supermarkets pack the checkouts with crisps and sweets and signs saying: "Hey kids, if your parents really love you they'll buy you our special offer life-size chocolate baboon - eight stone of pure creamy ape happiness. If they don't let you have one, it means you were adopted."

The executive of Tesco interviewed on the news agreed that something must be done about this impending sugar disaster, as if the supermarkets have played no part in causing it. Maybe someone sneaks into branches of Tesco at night, and every morning the manager arrives and says: "Oh not again. There are three aisles of extra-sugary-super-sweet flagons of Coke. When I left yesterday they were full of fresh fish."

Asked whether he thought there should be legislation to resolve the issue, the Tesco executive said they were already dealing with it voluntarily. Of course they are. They might make billions out of promoting food packed with unnecessary sugar but for Tesco the priority is keeping children healthy. Then at the shareholders' AGM the chief executive will announce: "I have excellent news. This year we've sold hardly any biscuits or **fizzy drinks,** so there are no dividends to any of you, but now kids can jump around in sandpits instead of collapsing in a wheezy diabetic fit because their body is 60 per cent cream soda" and everyone will cheer.

Tomorrow there will be an item on the news in which a crack dealer assures us he's doing all he can to reduce the crack content of crack, as his main concern is the health and well-being of his customers.

Some experts dispute the concerns altogether. In particular, a research body called Sugar Nutrition UK claims there's no evidence that added sugar does us harm or is linked to obesity, and it must be coincidence that this group is funded by the manufacturers of sugar.

Their impartial opinion is that if added sugar in food such as pasta sauce is reduced, "the sugar will need to be replaced by another ingredient that may contain more calories than the sugar". This is clearly true, if the item you replace the sugar with is a bucket of beef dripping.

The same goes for ready-made chicken kievs. It may contain six spoons of sugar, but if you took that out and stuffed it with a tray of used oil from a chip shop's deep fat frier it could be even more unhealthy, so it's best to leave it as it is.

One possibility could be to replace the added sugar with nothing, with a similar technique to that used by people who give up having sugar in tea. Usually, when someone does this, they cope with nothing in their tea, and don't feel the need to replace the sugar with something else such as a bucket of chicken nuggets.

Sugar Nutrition UK may object that insufficient research has been carried out on the effects of nothing, with philosophers still uncertain as to what nothing actually is, which means there could be harmful side effects worse than the relatively benign epidemic of heart disease their product is responsible for.

The free market being what it is, they'll probably get their way, though you might wonder if it's a coincidence that these bodies with scientific-sounding names that are funded by companies that make things always conclude that the things they make are excellent and safe and don't need to be curtailed at all. There's probably an organisation called Breaking and Entering Research UK, funded by the nation's burglars, that will produce a report that goes: "There is no evidence that having your belongings stolen makes you any poorer, or reduces the number of belongings you have in any way, as long as you get robbed as part of an overall balanced diet, which of course we fully recommend."

Read more:A sugar tax won't lead to a sweet sigh of relief on obesityWhy I'm giving up sugar for LentTaxing sugar would be illiberal and ineffective

LOAD-DATE: March 7, 2014

LANGUAGE: ENGLISH

PUBLICATION-TYPE: Newspaper; Web Publication

JOURNAL-CODE: WEBI

Copyright 2014 Independent Digital News and Media Limited
All Rights Reserved

The Independent (London)

March 7, 2014 Friday
First Edition

Worried about what sugar is doing to you? There's no need, because supermarkets, like crack dealers, have your health as their top priority

BYLINE: Mark Steel

SECTION: COMMENT; Pg. 27

LENGTH: 866 words

The World Health Organisation has said sugar is the "new tobacco", making us fat and useless, so now the experts are wondering how this could possibly have happened.

But one cause that may have contributed is that sugar is now in everything. Bread is full of sugar and soup is full of sugar and soon carrots will be full of ice cream and tap water will be flavoured with melted Yorkie bars, and new cereals are launched with adverts that go: "Everyone's asking why new Sweetypops are so pop-pop-popular. They're solid sugar lumps deep fried in treacle fat and glazed with concentrated Jaffa cakes then coated with five kinds of maple syrup and a doughnut. Scientists have said one bowl could contain up to 73 per cent of daily recommended goodness particles, and Sweetypops now come in new raspberry flavour, so it even counts towards your five a day."

The staff at coffee shop chains are instructed to ask everyone "would you like a muffin with that?", with a sorrow that makes you feel they'll be whipped if you don't have at least seven.

And everyone offers you chocolate. At one point, if you bought a Daily Telegraph in WH Smiths, you were told this entitled you to a half-price giant-size Toblerone, so whole villages in Surrey must have flooded as nutty dribbles oozed out of angry mouths spluttering about the need to bring back National Service. It's everywhere. I expect if you go to B&Q now you're asked: "Would you like a cinnamon bun with your shelving brackets?"

And the pressure on children is even greater. Supermarkets pack the checkouts with crisps and sweets and signs saying: "Hey kids, if your parents really love you they'll buy you our special offer life-size chocolate baboon - eight stone of pure creamy ape happiness. If they don't let you have one, it means you were adopted."

The executive of Tesco interviewed on the news agreed that something must be done about this impending sugar disaster, as if the supermarkets have played no part in causing it. Maybe someone sneaks into branches of Tesco at night, and every morning the manager arrives and says: "Oh not again. There are three aisles of extra-sugary-super-sweet flagons of Coke. When I left yesterday they were full of fresh fish."

Asked whether he thought there should be legislation to resolve the issue, the Tesco executive said they were already dealing with it voluntarily. Of course they are. They might make billions out of promoting food packed with unnecessary sugar but for Tesco the priority is keeping children healthy. Then at the shareholders' AGM the chief executive will announce: "I have excellent news. This year we've sold hardly any biscuits or **fizzy drinks,** so there are no dividends to any of you, but now kids can jump around in sandpits instead of collapsing in a wheezy diabetic fit because their body is 60 per cent cream soda" and everyone will cheer.

Tomorrow there will be an item on the news in which a crack dealer assures us he's doing all he can to reduce the crack content of crack, as his main concern is the health and well-being of his customers.

Some experts dispute the concerns altogether. In particular, a research body called Sugar Nutrition UK claims there's no evidence that added sugar does us harm or is linked to obesity, and it must be coincidence that this group is funded by the manufacturers of sugar.

Their impartial opinion is that if added sugar in food such as pasta sauce is reduced, "the sugar will need to be replaced by another ingredient that may contain more calories than the sugar". This is clearly true, if the item you replace the sugar with is a bucket of beef dripping.

The same goes for ready-made chicken kievs. It may contain six spoons of sugar, but if you took that out and stuffed it with a tray of used oil from a chip shop's deep fat frier it could be even more unhealthy, so it's best to leave it as it is.

One possibility could be to replace the added sugar with nothing, with a similar technique to that used by people who give up having sugar in tea. Usually, when someone does this, they cope with nothing in their tea, and don't feel the need to replace the sugar with something else such as a bucket of chicken nuggets.

Sugar Nutrition UK may object that insufficient research has been carried out on the effects of nothing, with philosophers still uncertain as to what nothing actually is, which means there could be harmful side effects worse than the relatively benign epidemic of heart disease their product is responsible for.

The free market being what it is, they'll probably get their way, though you might wonder if it's a coincidence that these bodies with scientific-sounding names that are funded by companies that make things always conclude that the things they make are excellent and safe and don't need to be curtailed at all. There's probably an organisation called Breaking and Entering Research UK, funded by the nation's burglars, that will produce a report that goes: "There is no evidence that having your belongings stolen makes you any poorer, or reduces the number of belongings you have in any way, as long as you get robbed as part of an overall balanced diet, which of course we fully recommend."

Twitter: @mrmarksteel

LOAD-DATE: March 6, 2014

LANGUAGE: ENGLISH

PUBLICATION-TYPE: Newspaper

JOURNAL-CODE: IA

Copyright 2014 Independent Print Ltd
All Rights Reserved

MailOnline

MailOnline

March 7, 2014 Friday 2:30 PM GMT

The sugary drinks and snacks that you can't even have ONE of without breaching new guidelines on daily intake

BYLINE: MARTIN ROBINSON

SECTION: HEALTH

LENGTH: 964 words

- Experts say maximum of six teaspoons of sugar per day would be ideal figure for adults who want to stay healthy
- World Health Organisation recommendation slashed in half from equivalent of about 12 level teaspoons a day
- This guideline amount has been cut to tackle obesity and heart disease amid fears sugar is as deadly as tobacco
- Action on Sugar compile list showing single items broken down by the number of teaspoons of sugar they contain

These drinks, meals and snacks contain up to double the safe amount of sugar that experts believe adults should consume per day.

A single Starbucks caramel frappuccino has 11 teaspoons of sugar and a can of Coca-Cola or Pepsi has nine teaspoons, researchers have said.

This week the World Health Organisation recommended people should have no more than six teaspoons of sugar in 24 hours.

Doctors say this rule is key to avoiding obesity, heart disease and other serious illnesses because they fear sugar is as dangerous as tobacco.

Experts have backed the advice but said today it is extremely hard to calculate how much a sugar people are eating or drinking, especially because of unclear labelling.

Pressure group Action on Sugar has produced its own table of foods to make it clearer for consumers.

It also revealed that many single products take up the new recommended allowance.

For example a 51g Mars Bar has eight teaspoons, a can of Red Bull has seven, a Muller strawberry shortcake Crunch Corner has six teaspoons and so does an Innocent smoothie.

Action on Sugar science director, Aseem Malhotra, who wrote for the Mail on the subject today, told the Guardian: 'I agree with the WHO recommendation, but it has to be translated into something meaningful for the consumer.'

The debate over the danger of sugar is raging and UN health chiefs have warned children should not be given **fizzy drinks** at all because they have dangerous sugar levels.

Adults have been told should halve their average intake to six teaspoons a day - slashed dramatically amid fears that sugar poses the same threat to health as tobacco.

Experts blame it for millions of premature deaths across the world every year.

Graham MacGregor, a London cardiologist and health campaigner, said: 'Added sugar is a completely unnecessary part of our diets, contributing to obesity, type II diabetes and tooth decay.

'We have known about the health risks of sugar for years and yet nothing substantial has been done.

'The new recommendations will be a wakeup call to the Department of Health and the Government to take action by forcing the food industry to slowly reduce the huge amount of sugar added across the board.'

Chief medical officer Sally Davies has already said a tax may be put on calorie-laden food and drink to curb soaring levels of obesity.

Labour suggested last night it would impose a maximum limit on sugar, fat and salt in products marketed at children.

The number of obese British adults is expected to double from one in four to one in two by 2050 - at a cost to the economy of £50billion a year.

The UN's World Health Organisation said the crisis was being fuelled by hidden sugar in processed food and drink such as yoghurts, muesli, sauces, **fizzy drinks**, juice and smoothies.

Last night it published the draft guidelines urging adults to eat no more than 12 teaspoons of sugar a day and to aim for six.

And it said children should try for less than six teaspoons and avoid cans of **fizzy drink** such as Coke, which contains seven spoons.

Francesco Branca, director for nutrition for health and development at WHO, said: 'Obesity affects half a billion people in the world and it is on the rise.

'Sugar along with other risk factors might certainly become the new tobacco in terms of public health action. The consumption of a single serving of sugar sweetened soda might actually already exceed the limit for a child. So certainly the consumption of **sugar**-sweetened **beverages** should be done with great care.

'It actually is one of the elements that has been more constantly associated to increase weight gain particularly in children.'

The guidelines will now be discussed by academics and medical experts before a final version is published. But Dr Branca said food and drinks manufacturers should drastically alter their products.

A bowl of muesli contains two and a half teaspoons of sugar, a latte has five, a chocolate bar six or seven while some ready-meals have more than eight.

Labour's health spokesman Andy Burnham said his party was considering setting a legal maximum on the amount of sugar, fat and salt in foods aimed at children.

'We have a big ambitious health policy coming out,' he added.

'We feel the Government has lost its way completely on public health, there's no leadership at all now.'

It is understood the policy would cover products such as Kellogg's Frosties, which is 37 per cent sugar.

On Monday, Dame Sally told MPs that being overweight had become 'normalised'.

But David Cameron's official spokesman yesterday played down the need for a sugar tax and said ministers would rather encourage food and drinks firms to voluntarily make products healthier.

He added: 'What we are doing is working with the industry. You have already seen commitments from retailers and food manufacturers to reduce levels of salt, to remove some artificial fats, to reduce calorie content and improve labelling, as well as public health campaigns by local authorities and the NHS.'

The draft guidelines do not address the health effects of sugar substitutes or chemical sweeteners.

Andrew Percy, a Tory MP on the Commons health select committee, raised fears of 'nanny state' meddling.

He said: 'What we need to do is educate people about food, and proper labelling of food is important in that. But, in the end, people must have the discretion to make their own choices.'

LOAD-DATE: March 7, 2014

LANGUAGE: ENGLISH

PUBLICATION-TYPE: Web Publication

JOURNAL-CODE: WEBDM

Copyright 2014 Associated Newspapers Ltd.
All Rights Reserved

MailOnline

MailOnline

March 7, 2014 Friday 12:18 AM GMT

Anger as schools scrap limit on sugar in meals hours after health experts tell us to halve our intake

BYLINE: LAURA CLARK and DANIEL MARTIN

SECTION: NEWS

LENGTH: 821 words

- WHO experts says children should have just six teaspoons of sugar a day
- Current rules says school lunches can have no more than four teaspoons
- But prescribed levels of sugar, fat and salt are now set to be abandoned
- New guidelines will instead stipulate how often different foods can be served

A strict limit on sugar in pupils' lunches is to be scrapped in a major overhaul of school meals, it emerged yesterday.

The decision, which provoked an outcry from health and obesity experts, comes less than a day after World Health Organisation chiefs demanded a sharp cut in children's sugar intake.

Critics warned ministers against ignoring the WHO advice and wasting a chance to slash sugar intake.

One MP described the school lunch proposals, which are almost certain to be implemented, as 'puzzling to say the least'. Prescribed levels of fat and salt - as well as vitamins and minerals such as iron and calcium - are also set to be abandoned under the Department for Education plans.

Separate rules which dictate how many times certain items such as chips can be served during the week will remain in place.

Those rules will be expanded and updated, for example to specify that wholegrain food must be offered each week to provide fibre to children.

But more than a quarter of primary schools which trialled the proposed new system exceeded the old sugar limit - of four teaspoons per meal - by more than 10 per cent.

Nutritionists said 'robust' action is needed to meet a new target from the WHO, a subsidiary of the UN, to reduce adults' sugar intake to 12 teaspoons per day and children's to six.

On Wednesday UN health chiefs went even further, and said adults should aim to halve this to six teaspoons and children to less than six.

Unveiling its draft advice, WHO officials said sugar may pose the same threat to health as tobacco. It is increasingly implicated in obesity, heart disease and a range of other serious illnesses, they warned.

A single **fizzy drink** serving could exceed the six-teaspoon daily limit. At present, schools in England must comply with two sets of catering standards: one covering the frequency that specific items must be served during the week and another prescribing the levels of 14 nutrients in school lunches.

These nutrient standards stipulate that an average lunch in a primary must contain no more than 15.5g of sugar - equivalent to just under four teaspoons. In secondaries the limit is 18.9g, or four-and-a-half teaspoons.

Maximum limits are also set for fat, saturated fat and sodium, and minimums for key nutrients such as iron, fibre, zinc, protein and fibre.

The Department for Education, which commissioned chefs Henry Dimbleby and John Vincent to review school food, said caterers find current rules 'over-complicated'.

A consultation paper published yesterday said they were 'difficult to understand' and led to schools paying £20 for each recipe to be privately analysed. 'The nutrient-based standards can restrict cooks from being creative,' it added.

Under the plans, schools will no longer be required to prove they meet nutrient standards and will instead use new food-based standards to devise menus. These state, for example, that desserts, cakes and biscuits must be served only at lunchtime.

But in a trial of this approach, half of secondaries and a third of primaries failed to stay within the maximum sugar limit.

There would be 'strengthened practical guidance on portion sizes for desserts', in addition to existing limits on the quantity of fruit juices children can drink. Schools remain barred from offering **fizzy drinks**.

Professor Susan Jebb, an Oxford University nutritionist who helped draw up the new rules, said: 'If a school meets all of those food-based standards, it ought to be pretty close to meeting the nutrient standards we previously had. Schools can be confident they will be close to them.'

She said sugar consumption was already higher than the limit in some schools since cooks found the nutrient rules difficult to implement.

Newly-established academy schools will also be required to comply.

Tam Fry, from the National Obesity Forum, said: 'These guidelines come just days after the Chief Medical Officer [Sally Davies] confirmed sugar is the real disaster. The WHO report was our signpost to crack down on sugar.'

Katharine Jenner, a nutritionist at Queen Mary, University of London and Action for Sugar's campaign director, said the WHO's target was 'such a long way from where we are'.

She added: 'Challenging taste preferences in childhood is vital because it affects the rest of their lives. We would want to look at this in detail and whether a child's diet at school will be worse than it was before.'

Labour MP Simon Danczuk said: 'I believe parents will be extremely disappointed by this decision.

'Why the Government would want to reduce the healthiness of school meals is puzzling to say the least. The Government is going in the wrong direction.'

LOAD-DATE: March 7, 2014

LANGUAGE: ENGLISH

PUBLICATION-TYPE: Web Publication

JOURNAL-CODE: WEBDM

Copyright 2014 Associated Newspapers Ltd.
All Rights Reserved

The Telegraph
telegraph.co.uk

March 7, 2014 Friday 2:00 PM GMT

Taxing sugar probably will work, and it's not an attack on our 'freedom'

BYLINE: By Martha Gill

SECTION: BLOG

LENGTH: 516 words

We, the British, are a nation of fat people - increasingly, of fat children. But if there's one thing that we, the British, really take exception to, it's being treated like fat children.

This week the chief medical officer Dame Sally Davies endorsed a tax on sugar. The idea is that a tax will nudge us all into cutting our sugar intake, and help curb obesity rates. My fellow bloggers Brendan O'Neill and Jacob Rees-Mogg are amongst many who take issue with this - calling it an intrusive, infantilising, nanny-state-ing idea that will dangerously impose on our freedoms and will only lead to the poor scrimping on the rest of their shopping so they can still buy chocolate bars. I mean, that's a bit of a glib summary. You can read their excellent pieces here and here. But I don't agree with either of them.

I'll address the most basic concern first, that the tax simply won't work. There's actually a good chance that it will - effectively reducing sugar consumption, particularly amongst the young.

Let's look at a near-ish analogy, the cigarette tax. Raising the price of cigarettes has been shown to consistently reduce consumption. A 10 per cent increase in price causes a 3 to 5 per cent drop in demand. And there's a more striking effect on the young: a 10 per cent price rise causes a 3.5 per cent reduction in teen smoking, and a 6-7 per cent drop in kids who smoke. It's a good analogy, as it shows a tax doesn't just cause shoppers to economise on other stuff, even with an addictive product like tobacco.

And there's a recent Stanford/Cornell economics study that applies this directly to sugar. The authors looked at millions of food and drink transactions to look at how people react to increases in price. They found that a 20 per cent price rise in **sugary drinks** lead to people spending 4 per cent less (their household calorie intake dropping by about 5 per cent). All good signs the tax might work.

Second, let's look at the idea that the tax will curb our freedoms. There is an assumption here. It's that right now, we're free - frolicking un-nudged in a world of infinite, perfectly informed choice. We're not, really. We

are being nudged by food companies, who know that the best way to sell their products is to make them taste nice (pump them full of sugar). Tempting packaging nudges us, supermarket placement nudges us, and we are nudged constantly, wherever we are, by adverts.

Of course, the information is on the packets, and we can make an informed choice, should we wish, about how many calories we wish to consume. But which of us has the time to think such a decision through each time we buy a snack? Who has the energy to maintain a fight against marketing tactics every day? Exercising freedom of choice involves bothering to concentrate. When it comes to a decision as small as this, many of us simply don't.

Willpower, as I wrote earlier this week, is a limited resource. It's important we save ours for the choices that really matter. Let's not use it all up resisting Mars bars.

Follow @TelegraphBlogs

LOAD-DATE: March 8, 2014

LANGUAGE: ENGLISH

PUBLICATION-TYPE: Newspaper; Web Publication

JOURNAL-CODE: WEBDTB

Copyright 2014 Telegraph Media Group Limited
All Rights Reserved

DAILY MAIL (London)

March 6, 2014 Thursday

CUT YOUR SUGAR INTAKE

LENGTH: 707 words

HALF

BY SOPHIE BORLAND AND DANIEL MARTIN

CHILDREN should not be given **fizzy drinks** because they contain dangerous amounts of sugar, UN health chiefs said yesterday.

They also warned adults should halve their average intake to six teaspoons a day to avoid obesity, heart disease and other serious illnesses.

The guideline amount has been slashed dramatically amid fears that sugar poses the same threat to health as tobacco.

Experts blame it for millions of premature deaths across the world every year.

Graham MacGregor, a London cardiologist and health campaigner, said: Added sugar is a completely unnecessary part of our diets, contributing to obesity, type II diabetes and tooth decay.

We have known about the health risks of sugar for years and yet nothing substantial has been done.

The new recommendations will be a wakeup call to the Department of Health and the Government to take action by forcing the food industry to slowly reduce the huge amount of sugar added across the board.'

Chief medical officer Sally Davies has already said a tax may be put on calorie-laden food and drink to curb soaring levels of obesity. Labour suggested last

night it would impose a maximum limit on sugar, fat and salt in products marketed at children.

The number of obese British adults is expected to double from one in four to one in two by 2050 - at a cost to the economy of £50billion a year.

The UN's World Health Organisation said the crisis was being fuelled by hidden sugar in processed food and drink such as yoghurts, muesli, sauces, **fizzy drinks**, juice and smoothies.

Last night it published the draft guidelines urging adults to eat no more than 12 teaspoons of sugar a day and to aim for six.

And it said children should try for less than six teaspoons and avoid cans of **fizzy drink** such as Coke, which contains seven spoons.

Francesco Branca, director for nutrition for health and development at WHO, said: Obesity affects half a billion people in the world and it is on the rise.

Sugar along with other risk factors might certainly become the new tobacco in terms of public health action. The consumption of a single serving of sugar sweetened soda might actually already exceed the limit for a child. So certainly the consumption of **sugar**-sweetened **beverages** should be done with great care.

It actually is one of the elements that has been more constantly associated to increase weight gain particularly in children.'

The guidelines will now be discussed by academics and medical experts before a final version is published. But Dr Branca said food and drinks manufacturers should drastically alter their products.

A bowl of muesli contains two and a half teaspoons of sugar, a latte has five, a chocolate bar six or seven while some ready-meals have more than eight.

Labour's health spokesman Andy Burnham said his party was considering setting a legal maximum on the amount of sugar, fat and salt in foods aimed at children. We have a big ambitious health policy coming out,' he added.

We feel the Government has lost its way completely on public health, there's no leadership at all now.'

It is understood the policy would cover products such as Kellogg's Frosties, which is 37 per cent sugar.

On Monday, Dame Sally told MPs that being overweight had become normalised'.

But David Cameron's official spokesman yesterday played down the need for a sugar tax and said ministers would rather encourage food and drinks firms to voluntarily make products healthier.

He added: What we are doing is working with the industry. You have already seen commitments from retailers and food manufacturers to reduce levels of salt, to remove some artificial fats, to reduce calorie content and improve labelling, as well as public health campaigns by local authorities and the NHS.'

The draft guidelines do not address the health effects of sugar substitutes or chemical sweeteners.

Andrew Percy, a Tory MP on the Commons health select committee, raised fears of nanny state' meddling.

He said: What we need to do is educate people about food, and proper labelling of food is important in that. But, in the end, people must have the discretion to make their own choices.'

Professor MacGregor is a cardiologist at Barts and chairman of the group Action on Sugar.

© Daily Mail

LOAD-DATE: March 5, 2014

LANGUAGE: ENGLISH

PUBLICATION-TYPE: Papers

Copyright 2014 Associated Newspapers Ltd.
All Rights Reserved

The Daily Telegraph (London)

March 6, 2014 Thursday
Edition 2;
National Edition

Halve sugar intake, say health experts

BYLINE: Edward Malnick

SECTION: NEWS; FRONT PAGE; Pg. 1

LENGTH: 468 words

THE daily allowance for a person's sugar intake should be halved to six teaspoons, the World Health Organisation (WHO) said yesterday.

Draft guidance published by the international body advises a dramatic reduction in sugar consumption to help avoid mounting health problems including obesity and tooth decay.

The WHO is proposing current formal recommendation that no more than 10 per cent of individual's calories should come from sugar - the equivalent of "level" teaspoons a for the average adult.

However, its draft guidelines state that further reduction to 5 cent "would have additional benefits". lower limit amounts around six teaspoons - less than the levels sugar in a 50g Mars Bar.

The move comes amid growing evidence sugar contributes to range of chronic It follows calls scientists for the recommendation to be halved.

The WHO's announcement comes after a study by the University of Southern California, disclosed yesterday, found that eating much protein could be as dangerous smoking for middle-aged people.

Separately a leading heart scientist warned that NHS guidelines advising people to cut down on high-fat foods may be putting the public at risk. Dr James DiNicolantonio warns that more focus needed on the "harms" of consuming high levels of carbohydrates and sugar.

The WHO limits apply to all "free" sugar, which is sugar that is added to foods by manufacturer, plus that naturally present in honey, syrups, and fruit juices.

The announcement came after Dame Sally Davies, Britain's chief medical officer, suggested a sugar tax to curb obesity rates.

Dr Francesco Branca, director for nutrition for health and development at WHO, warned that obesity already affected half a billion people. The recommendation for less than 5 per cent important because it told countries that reductions to "below 5 per cent even better", he said.

The WHO warned much of the sugars consumed today were "hidden" in processed foods "that are not usually seen as sweets".

Dr Branca said a single can of **fizzy drink** could exceed the amount of sugar that children should have in a under the current limits, which are reflected NHS guidance.

The new draft guidelines, which are now to consultation, were published after the WHO considered a report scientists at Newcastle University which suggested the limit should be halved to reduce tooth decay.

The Action on Sugar campaign group, which also backs the move as a way helping to tackle obesity and chronic diseases such as diabetes, said the WHO's proposals should have gone further halved the 10 per cent recommendation.

The food and drink industry denies claims over the effect of sugar on health.

High-fat food mistake: Page 9

Editorial Comment: Page 23

MATT 'When I hired you to kill my husband I didn' t expect you to come and cook him steak every day'

LOAD-DATE: March 6, 2014

LANGUAGE: ENGLISH

PUBLICATION-TYPE: Newspaper

JOURNAL-CODE: DTL

Copyright 2014 Telegraph Media Group Limited
All Rights Reserved

theguardian

The Guardian - Final Edition

March 6, 2014 Thursday

A can of Coke a day is too much sugar, warns WHO: Ideal for adults is 5% of calories, says global body: 'Need for action' over sugar-sweetened drinks

BYLINE: Sarah Boseley, Health editor

SECTION: GUARDIAN HOME PAGES; Pg. 7

LENGTH: 638 words

Adults in Britain should aim to cut their sugar intake to 5% of daily calories, according to the World Health Organisation - less than the amount, for an average person, in a single can of Coca-Cola.

In a new draft guideline, the WHO said all people should try to reduce the amount of sugar they consume. It reiterated its 2003 guidance that countries should set a limit of 10% of daily calories from sugar - but said the ideal level would be 5%.

For an adult of average bodyweight, with an intake of about 2,000 calories a day, 5% would equate to 100 calories - which at four calories in a gram would be 25g of sugar, said Dr Francesco Branca, the WHO's director of nutrition for health and development. A standard 330ml can of cola contains 35g of sugar.

Even at a 10% limit, said Branca, a can of sugar-sweetened drink "approaches the amount that is acceptable for an adult. For a child, since a child has a lower energy requirement, that could be a lot less. Consumption of a single serving of sugar-sweetened soda might actually exceed the limit of 10% for a child."

Branca added that soft drink consumption "is one of the elements that has been more constantly associated with increased weight gain, particularly in children. This is an area where more intense action needs to be taken if this guideline is to be implemented."

The WHO's intervention is triggered by concern over the obesity epidemic, as well as tooth decay. There are particular anxieties about sugar-sweetened drinks such as colas, lemonades and sports drinks, which give "empty calories", devoid of the nutrients found in some other foods.

The WHO's nutrition guidance expert advisory group (NUGAG) has been mulling over the evidence for nearly two years and commissioned scientific reviews of the risks posed to health by "free sugars" - those added to food and drinks rather than the intrinsic sugars in fruit and vegetables.

The evidence is clearest on dental caries, the report says. Studies show an increase in tooth decay in children who get more than 10% of their calories from sugar. There was also evidence that children consuming less than that - about 5% of their calorie intake - also developed dental caries, although at lower levels.

"Because dental caries are the result of lifelong exposure to the dietary risk factor (ie, sugars), even small reductions in risk of dental caries in childhood is of significance in later life," says the document.

The link with obesity and diseases for which it is a risk factor, such as strokes, diabetes, heart disease and some cancers, is less clear cut. However, analyses of all available well-conducted trials suggested that people who cut down on sugar also managed to reduce weight - and those who ate more sugar put on weight.

Studies also showed that children who drank a lot of **sugar**-sweetened **beverages,** such as colas, were more likely to be overweight than those who rarely drank them.

The new guideline is likely to be strongly opposed by the food and drink industry and their supporters, who argue that no one food or type of food is a problem - all food and drinks are fine in moderation, they say.

Public Health England said it would "carefully look" at the recommendation to reduce sugar consumption to less than 5%. Average intake for adults in the UK is 11.6% and for children it is 15.2% - both above current government advice, which is for a maximum 10%.

"PHE welcome the new WHO draft guidelines to reduce sugar intake to 10% of total daily calorie intake and will carefully consider the suggestion that a further reduction of sugar to below 5% of total energy intake per day would have additional benefits," said Alison Tedstone, director of nutrition and diet at PHE.

Captions:

35g

Amount of sugar in a standard 330ml can of cola. The WHO's 5% target for an adult's daily sugar intake equates to 25g

LOAD-DATE: March 6, 2014

LANGUAGE: ENGLISH

PUBLICATION-TYPE: Newspaper

Copyright 2014 Guardian Newspapers Limited
All Rights Reserved

i-Independent Print Ltd

March 6, 2014
First Edition

I'm so glad Dame Sally has Lent me a good reason to become healthier

BYLINE: Simon Kelner KELNER'S VIEW

SECTION: FEATURES; Pg. 16

LENGTH: 492 words

As a pleasure seeker who understands the need to be conscious of my own mortality, I'm always looking for a reason to give something up, and Lent seems as good a reason as any. I'm not a Roman Cath-olic, but I do like the idea of a period of self-denial at this time of year, for health reasons if not for spiritual or religious purification. I don't lack for willpower, and last year I gave up alcohol for 11 months, an experiment in abstinence which came to an end in December with the combined assault of my birthday, a number of parties and, of course, Christmas. (I have since realised that my alcohol intake over the month probably equated to what most people drink in the course of a year.) I don't smoke or eat red meat, so I was struggling over what to give up this Lent until I heard a radio phonein responding to the proposal by the Chief Medical Officer, Dame Sally Davies (right), that the Government should introduce a sugar tax to curb growing rates of obesity.

It was hard to keep up with the random litany of statistics, but they all added up, in my view, to supporting Dame Sally's opinion. The UK consumes 5,727 million litres of **sugary soft drinks** every year. There are four teaspoons of **sugar** in a can of peas, and five teaspoons in a low-fat yoghurt. If 20p tax were added to a litre of **soft drink**, more than £1bn could be raised to be spent on health initiatives. Smoothies are the short cut to obesity. About 60 per cent of Britons are considered overweight or obese. And so on.

I resolved that, this Lent, it was sugar that was going to get the boot. I have eaten at enough restaurants in my time - and have the figure to prove it - to know that if something tastes good, it's usually for one of four, unadvertised, reasons - butter, salt, cream or sugar. So I realise my mission is going to be really tough, because sugar is the hidden persuader, and, according to Dame Sally, will be proved to be addictive.

She says that a tax may be the most effective way of changing our eating and drinking regime. "We have normalised being overweight," she said. Others in the medical profession say sugar is a dangerous, life-threatening drug.

The arguments against a tax on sugary products are familiar enough. For instance, this is a tax on the poorest people, and while there is a class dimension to this debate - the middle-class person who asks for two sugars in a cup of tea is regarded with as much astonishment as the one who strikes up a Marlboro at a

dinner - this is where the Government comes in, legislating to change behaviour for the general betterment of society (treating diseases caused by unhealthy lifestyles costs the NHS £6bn a year).

The food and drink industry believes in education not legislation. People should be free to make their own choices, say libertarians.

Well, I've made my choice. I'm not going to wait until the Nanny State comes knocking. Here goes. Expect some bad-tempered columns over the next month.

LOAD-DATE: March 5, 2014

LANGUAGE: ENGLISH

PUBLICATION-TYPE: Newspaper

JOURNAL-CODE: III

Copyright 2014 Independent Print Limited
All Rights Reserved

The INDEPENDENT
Independent.co.uk

March 6, 2014 Thursday 12:11 AM GMT

Is sugar the new evil? Arguments for and against the grain; Lobbyists say taxing added sugar can achieve results, but international bodies haven't jumped on the bandwagon yet

BYLINE: Charlie Cooper

SECTION: FRONTPAGE

LENGTH: 1217 words

Not very long ago, the question would have seemed absurd. Now it is on the lips of respected scientists; MPs are beginning to talk of "a war on sugar", and this week even England's chief medical officer - one of the most esteemed voices in the public debate on health - has said sugar may have to be, like the old enemy tobacco, taxed in order to protect the nation's health.

There came a time in the evolution of public attitudes to smoking, when the doctors had been shouting for long enough that the public was broadly aware of the risks and the only question left for Government was: what do we about it?

Some believe we are now at the same point in our attitudes to sugar. Others - largely but not exclusively representatives of the food and drink industry - say the entire debate has been skewed by scaremongering.

More or less everyone agrees that eating too much sugar is bad for you. There is also no doubt obesity is a growing problem which is putting a significant, avoidable burden on the NHS by increasing the rates of diabetes, heart disease and other long-term conditions.

But to what extent is sugar - rather than saturated fats, or salt, carbohydrates or proteins, or any of the other bogey-men of modern diets - the cause of obesity and how much should we worry about it?

Yesterday, hopes of achieving anything resembling clarity from health authorities were confounded once again. The World Health Organisation (WHO), widely expected to halve the recommended sugar intake in new draft guidance, instead said it would continue to recommend that sugar make up no more than 10 per cent of the energy we consume (or about 50g daily), while adding that cutting this to five per cent would have "additional benefits". The decision will now go out to public consultation.

Simon Capewell, professor of clinical epidemiology at the University of Liverpool and the man who made headlines by comparing sugar to tobacco earlier this year, said that he suspected "dirty work" on the part of food and drinks companies might lie behind the WHO's less than resounding message.

Professor Capewell, a leading figure in the new medical pressure group Action on Sugar said that, while it was not known exactly how much of our current obesity epidemic - a third of children and two thirds of UK adults overweight or obese - was caused by sugar, there was now no doubt the white stuff makes an "important contribution" and one that could be easily prevented.

Public Health England (PHE) figures show that 11.6 per cent of the calories consumed by an average adult in the UK, and 15.2 per cent of those consumed by children, come from sugar - that can be from sugar added to food, but also from sugars naturally occurring in things like fruit juices and honey.

It's the added **sugar**, or refined **sugars**, in everything from cooking sauces to ready meals, to canned soup that Action against **Sugar** has a problem with - these and the huge amounts of **sugar** encountered in many popular **soft drinks.**

They say that added sugar is, like tobacco, "completely unnecessary" and, like tobacco, has to go.

"[Sugar] is of equal importance to tobacco in terms of representing a cause of major disease and death which is completely preventable," Professor Capewell told The Independent. "In the same way tobacco was around for decades and people slowly came to understand the harms, we're probably now with sugar where we were with tobacco in the 1980s - we know it's a bad thing, we know we ought to do something."

"Refined sugars represent empty calories, they have zero nutritional value. Until about 200 years ago sugar simply didn't exist in the UK food market. Going further back, our forebears were hunter-gatherers. That's what our bodies are built for - wandering round eating nuts and berries and the occasional antelope. Sugar? Maybe raiding a bees nest once a year perhaps. Our bodies are not used to it, and it's not natural."

The food industry contends that singling out sugar is scientifically unsound and will harm wider health messages about achieving a balanced diet.

Responding to the chief medical officer Dame Sally Davies' suggestion that the UK may have to consider a sugar tax in the future, the Food and Drink Federation (FDF) said earlier this week that "there is no single or simple solution" to the obesity problem, adding that, through the Government's voluntary Responsibility Deals, they are already working to cut sugar, just as they have done with salt and saturated fats.

The FDF added that "any additional taxation of food will hit the poorest families hardest at a time they can least afford it."

Changing advice on diets has certainly created a hugely complicated web of sometimes conflicting advice. This week alone academics at the University of Southern California have suggested that eating too much animal protein - from things like meat and eggs - can be as bad for us as smoking, while US cardiovascular scientist claims today [Thursday] that carbohydrates, not saturated fats are responsible for the obesity epidemic in his country.

Action on Sugar that focusing on added sugar, while not being a silver bullet, would bring achievable reductions in obesity without confusing consumers.

They claim that if major manufacturers committed to gradually reduce the amount of sugar in their products, adding up to a 20 to 30 per cent decrease in sugar content within three to five years, the obesity epidemic could be "halted or reversed".

"The food industry say Government has no business interfering in families, we must protect personal choice," Professor Capewell said. "We say there is no personal choice. At the moment, a mother can walk into a supermarket with a choice of four tomato soups - with three, or four, or five teaspoons of sugar in them. She has a choice of thousands of ready meals - with five, or six, or even nine teaspoons of sugar in.

"We think the Government should stand up and protect children, we think that responsible companies should stand up and protect children."

Killer foods: The current hitlist

Salt

While chefs may champion its cause, Britain's salt intake has been dramatically reduced in recent years. Its links to high blood pressure are well documented and the WHO has said salt reduction is of equal importance to stopping smoking. Anchovies, bacon, cheese and chicken nuggets are among the worst salt offenders.

Saturated fats

They raise cholesterol in the blood, increasing the risk of heart disease. The NHS says that most Britons eat too much of the fats - found in butter, lard, pies, cakes, meats, cheese and cream. Doctors recommend eating oily fish, nuts seeds, fruit and vegetables as a source of unsaturated fats.

Carbohydrates

They provide the majority of calories consumed in a day (between 45 and 65 per cent) but when consumed in large amounts can have health impacts. Yesterday, a US expert argued that carbohydrates were to blame for a surge in diabetes and obesity.

Protein

A new study revealed that middle-aged people who eat protein-rich food were four times more likely to die of cancer than someone who only eats a little. Researchers said proteins derived from animals were "nearly as bad as smoking". A high-protein diet was defined as one in which 20 per cent of calories came from protein. They recommended 0.8g of protein per kilogram of body weight.

LOAD-DATE: March 6, 2014

LANGUAGE: ENGLISH

PUBLICATION-TYPE: Newspaper; Web Publication

JOURNAL-CODE: WEBI

Copyright 2014 Independent Digital News and Media Limited
All Rights Reserved

The INDEPENDENT

The Independent (London)

March 6, 2014 Thursday
First Edition

Sweet temptation;
Editorials Taxing sugar would be illiberal and ineffective

SECTION: NEWS; Pg. 2

LENGTH: 337 words

Sugar is a killer. Despite recent increases in the world price of this commodity, it remains an easy way for the food industry to generate profits. The sugar content of much fast food and takeaways, in particular, is too high, and we are taking the sweet road to mass obesity.

It costs the NHS dear, it shortens lifespans and destroys the quality of life of individuals and families after strokes, heart attacks and the onset of diabetes. In many ways, it is the "new tobacco".

And yet taxing it in the same way we tax tobacco and alcohol may not be the best answer. Much could be achieved through voluntary action by the supermarkets, the fast-food chains and other interested parties.

So-called "meal deals" and other clever marketing encourage us to pile on the calories. The supermarkets could take more care about promoting chocolate, biscuits and **fizzy drinks**.

The manufacturers should reduce the size of chocolate bars and bags of sweets. (The same goes for the modern, inflated "grab bag" of crisps, about twice the size of the conventional pack of say 20 years ago).

Such voluntary action, and improved labelling, would be more effective than tax, because it removes temptation in the first place. What's more, a sugar tax will only work if it is set at a deterrent level, as with cigarettes. Unless that is done, the main impact will be to drive up the cost of the weekly shop, hitting poorer families hardest - but without reducing consumption.

Indeed, having to make extra room in the budget for sweet drinks and cookies would mean less cash for fresh fruit. A truly deterrent level of taxation - pricing cola as if it were whisky - would be politically unacceptable.

Besides, with special taxes on carrier bags, the possibility of minimum pricing for alcohol, varying duties on different types of booze and tobacco and different VAT rates on warm/hot snacks from the restaurant, our supermarkets will soon come to resemble fiscal adventure playgrounds, and to no great purpose.

Tax is not the answer to every social ill.

LOAD-DATE: March 5, 2014

LANGUAGE: ENGLISH

PUBLICATION-TYPE: Newspaper

JOURNAL-CODE: IA

Copyright 2014 Independent Print Ltd
All Rights Reserved

The INDEPENDENT
The Independent (London)

March 6, 2014 Thursday
First Edition

A spoonful of sugar... makes the medical experts go round; Is our sweetest food the deadliest? CHARLIE COOPER tries to find the truth in a health war being waged by doctors, public health bodies and big business

BYLINE: CHARLIE COOPER

SECTION: NEWS; Pg. 20

LENGTH: 1083 words

Is sugar the new tobacco? Not very long ago, this question would have seemed absurd. Now it is on the lips of respected scientists; MPs are beginning to talk of "a war on sugar", and this week even England's Chief Medical Officer - one of the most esteemed voices in the public debate on health - has said sugar may have to be, like the old enemy tobacco, taxed to protect the nation's health.

There came a time in the evolution of public attitudes to smoking when doctors had been shouting for long enough that the public was broadly aware of the risks, and the only question left for Government was: what do we about it? Some believe we are now at the same point with sugar. Others - largely but not exclusively representatives of the food and drink industry - say the entire debate has been skewed by scaremongering.

More or less everyone agrees that eating too much sugar is bad for you. There is also no doubt that obesity is a growing problem which is putting a significant, avoidable burden on the NHS by increasing the rates of diabetes, heart disease and other long-term conditions. But to what extent is sugar - rather than saturated fats, or salt, carbohydrates or proteins, or any of the other bogey-men of modern diets - the cause of obesity, and how much should we worry about it?

Yesterday, hopes of achieving clarity from health authorities were confounded once again. The World Health Organisation (WHO), widely expected to halve the recommended sugar intake in new draft guidance, instead said it would continue to recommend that sugar make up no more than 10 per cent of the energy we consume (or about 50g daily), while adding that cutting this to 5 per cent would have "additional benefits". The decision will now go out to public consultation.

Simon Capewell, professor of clinical epidemiology at the University of Liverpool and the man who made headlines by comparing sugar to tobacco earlier this year, said he suspected "dirty work" on the part of food and drinks companies might lie behind the WHO's message.

Professor Capewell, a leading figure in the new medical pressure group Action on Sugar, said that while it was not known exactly how much of our current obesity epidemic - a third of children and two thirds of UK adults overweight or obese - was caused by sugar, there was now no doubt that it makes an "important contribution".

Public Health England (PHE) figures show that 11.6 per cent of the calories consumed by an average adult in the UK, and 15.2 per cent of those consumed by children, come from sugar - that can be from sugar added to food, but also from sugars naturally occurring in things like fruit juices and honey.

It's the added **sugar**, or refined **sugars**, in everything from cooking sauces to ready meals to canned soup that Action Against **Sugar** has a problem with - these and the huge amounts of **sugar** in many popular **soft drinks.** They say that added **sugar** is, like tobacco, "completely unnecessary" and has to go.

"[Sugar] is of equal importance to tobacco in terms of representing a cause of major disease and death which is completely preventable," Professor Capewell told The Independent. "In the same way tobacco was around for decades and people slowly came to understand the harms, we're probably now with sugar where we were with tobacco in the 1980s - we know it's a bad thing, we know we ought to do something.

"Refined sugars represent empty calories, they have zero nutritional value. Until about 200 years ago sugar simply didn't exist in the UK food market. Going further back, our forebears were hunter-gatherers. That's what our bodies are built for - wandering around eating nuts and berries and the occasional antelope. Sugar? Maybe raiding a bees' nest once a year. Our bodies are not used to it, and it's not natural."

The food industry contends that singling out sugar is scientifically unsound and will harm wider health messages about achieving a balanced diet.

Responding to Dame Sally Davies' suggestion that the UK may have to consider a sugar tax in the future, the Food and Drink Federation (FDF) said earlier this week that "there is no single or simple solution" to the obesity problem, adding that, through the Government's voluntary Responsibility Deals, they are already working to cut sugar.

It added that "any additional taxation of food will hit the poorest families hardest at a time they can least afford it".

Advice can sometimes be conflicting. This week, academics at the University of Southern California suggested that eating too much animal protein - from things like meat and eggs - can be as bad as smoking, while a US cardiovascular scientist claims today that carbohydrates are responsible for the obesity epidemic.

But Action on Sugar says that focusing on added sugar would bring achievable reductions in obesity without confusing consumers. "The food industry say Government has no business interfering in families, we must protect personal choice," Professor Capewell said.

"We say there is no personal choice. At the moment, a mother can walk into a supermarket with a choice of four soups - with three, or four, or five teaspoons of sugar in them. The Government should stand up to protect children."

KILLER FOODS

THE CURRENT HITLIST

Salt

While chefs may champion its cause, Britain's salt intake has been dramatically reduced in recent years. Its links to high blood pressure are well documented and the WHO has said salt reduction is of equal importance to stopping smoking. Anchovies, bacon, cheese and chicken nuggets are among the worst salt offenders.

Saturated fats

They raise cholesterol in the blood, increasing the risk of heart disease. The NHS says that most Britons eat too much of the fats - found in butter, lard, pies, cakes, meats, cheese and cream. Doctors recommend eating oily fish, nuts seeds, fruit and vegetables as a source of unsaturated fats.

Carbohydrates

They provide the majority of calories consumed in a day (between 45 and 65 per cent) but when consumed in large amounts can have health impacts. Yesterday, a US expert argued that carbohydrates were to blame for a surge in diabetes and obesity.

Protein

A new study revealed that middle-aged people who eat protein-rich food were four times more likely to die of cancer than someone who only eats a little. Researchers said proteins derived from animals were "nearly as bad as smoking". A high-protein diet was defined as one in which 20 per cent of calories came from protein. They recommended 0.8g of protein per kilogram of body weight.

LOAD-DATE: March 5, 2014

LANGUAGE: ENGLISH

PUBLICATION-TYPE: Newspaper

JOURNAL-CODE: IA

Copyright 2014 Independent Print Ltd
All Rights Reserved

MailOnline

March 6, 2014 Thursday 6:47 PM GMT

People should cut their sugar intake to just six teaspoons a day, says World Health Organisation

BYLINE: SOPHIE BORLAND and DANIEL MARTIN

SECTION: HEALTH

LENGTH: 785 words

- The WHO recommends sugar makes up less than 10% of energy intake
- This is the equivalent of about 12 level teaspoons a day
- Now, experts say halving this could have additional health benefits
- Guideline amount slashed amid fears sugar poses same threat as tobacco
- They say 5%, or six teaspoons, is the ideal figure that people should aim for
- Labour suggest it would impose a maximum limit on sugar, fat and salt in products marketed at children
- Crisis is fuelled by hidden sugar in juice and processed foods, says WHO

Children should not be given **fizzy drinks** because they contain dangerous amounts of sugar, UN health chiefs said yesterday.

They also warned adults should halve their average intake to six teaspoons a day to avoid obesity, heart disease and other serious illnesses.

The guideline amount has been slashed dramatically amid fears that sugar poses the same threat to health as tobacco.

Experts blame it for millions of premature deaths across the world every year.

Graham MacGregor, a London cardiologist and health campaigner, said: 'Added sugar is a completely unnecessary part of our diets, contributing to obesity, type II diabetes and tooth decay.

'We have known about the health risks of sugar for years and yet nothing substantial has been done.

'The new recommendations will be a wakeup call to the Department of Health and the Government to take action by forcing the food industry to slowly reduce the huge amount of sugar added across the board.'

Chief medical officer Sally Davies has already said a tax may be put on calorie-laden food and drink to curb soaring levels of obesity.

Labour suggested last night it would impose a maximum limit on sugar, fat and salt in products marketed at children.

The number of obese British adults is expected to double from one in four to one in two by 2050 - at a cost to the economy of £50billion a year.

The UN's World Health Organisation said the crisis was being fuelled by hidden sugar in processed food and drink such as yoghurts, muesli, sauces, **fizzy drinks**, juice and smoothies.

Last night it published the draft guidelines urging adults to eat no more than 12 teaspoons of sugar a day and to aim for six.

And it said children should try for less than six teaspoons and avoid cans of **fizzy drink** such as Coke, which contains seven spoons.

Francesco Branca, director for nutrition for health and development at WHO, said: 'Obesity affects half a billion people in the world and it is on the rise.

'Sugar along with other risk factors might certainly become the new tobacco in terms of public health action. The consumption of a single serving of sugar sweetened soda might actually already exceed the limit for a child. So certainly the consumption of **sugar**-sweetened **beverages** should be done with great care.

'It actually is one of the elements that has been more constantly associated to increase weight gain particularly in children.'

The guidelines will now be discussed by academics and medical experts before a final version is published. But Dr Branca said food and drinks manufacturers should drastically alter their products.

A bowl of muesli contains two and a half teaspoons of sugar, a latte has five, a chocolate bar six or seven while some ready-meals have more than eight.

Labour's health spokesman Andy Burnham said his party was considering setting a legal maximum on the amount of sugar, fat and salt in foods aimed at children.

'We have a big ambitious health policy coming out,' he added.

'We feel the Government has lost its way completely on public health, there's no leadership at all now.'

It is understood the policy would cover products such as Kellogg's Frosties, which is 37 per cent sugar.

On Monday, Dame Sally told MPs that being overweight had become 'normalised'.

But David Cameron's official spokesman yesterday played down the need for a sugar tax and said ministers would rather encourage food and drinks firms to voluntarily make products healthier.

He added: 'What we are doing is working with the industry. You have already seen commitments from retailers and food manufacturers to reduce levels of salt, to remove some artificial fats, to reduce calorie content and improve labelling, as well as public health campaigns by local authorities and the NHS.'

The draft guidelines do not address the health effects of sugar substitutes or chemical sweeteners.

Andrew Percy, a Tory MP on the Commons health select committee, raised fears of 'nanny state' meddling.

He said: 'What we need to do is educate people about food, and proper labelling of food is important in that. But, in the end, people must have the discretion to make their own choices.'

Professor MacGregor is a cardiologist at Barts and chairman of the group Action on Sugar.

LOAD-DATE: March 6, 2014

LANGUAGE: ENGLISH

PUBLICATION-TYPE: Web Publication

JOURNAL-CODE: WEBDM

Copyright 2014 Associated Newspapers Ltd.
All Rights Reserved

Mirror

mirror.co.uk

March 6, 2014 Thursday 1:48 AM GMT

Sugar warning: People should halve the amount in their diet, experts say;
The World Health Organisation has advised we should go from a maximum 12 teaspoons a day to just six

BYLINE: By Andrew Gregory

SECTION: LIFESTYLE,HEALTH

LENGTH: 366 words

The World Health Organisation yesterday said people should halve the amount of sugar in their diet.

Officials advised we should go from a maximum 12 teaspoons a day to just six.

This would mean just one can of Coca Cola would exceed a person's daily limit.

WHO guidelines say sugars should make up less than 10% of total energy intake per day for adults and children, but experts believe slashing this further to just 5% is more "ideal".

Dr Francesco Branca, of WHO, said "Five per cent is the ideal one and the 10% is the more realistic one.

"We have few countries (hitting) below 10%. But, yes, we should aim for 5% if we can."

He added obesity affects half a billion people around the world and is on the rise among all age groups.

He said taxes on **sugary drinks** were working in the USA, France, Hungary and Finland.

Simon Capewell, professor of clinical epidemiology at the University of Liverpool, UK, said: "Brits currently consume about three times too much sugar, 15% not 5%.

"This is because of the huge amounts of added sugars hidden in most processed foods: soups, yoghurts, ready meals, **sugary drinks**, fruit juices and smoothies."

WHO criticised the UK Government's responsibility to deal with the food and drinks industry, which sees firms signing up to voluntary codes.

But David Cameron's official spokesman yesterday insisted that was the "right way".

Meanwhile, Labour announced it could ban children's food that is high in fat, salt and sugar.

Shadow Health Secretary Andy Burnham said: "We are going to have quite bold new thinking around children's diets."

Luciana Berger, Labour's Shadow Public Health Minister, last night confirmed Labour's plans.

She said: "Labour shares the World Health Organisation's concern about the amount of sugar in people's diets. We believe there are too many products on shop shelves which are presented as healthier options but in fact contain high levels of sugar.

"Parents need to have confidence that the food they buy is healthy - especially cereal and fruit drinks marketed at children.

"This issue demands strong leadership. Labour is asking the public and experts about our proposals to introduce clear limits for sugar, fats and salts in children's food."

LOAD-DATE: September 1, 2014

LANGUAGE: ENGLISH

PUBLICATION-TYPE: Newspaper; Web Publication

JOURNAL-CODE: WEBDMI

Copyright 2014 Trinity Mirror, Plc.
All Rights Reserved

The Telegraph
telegraph.co.uk

March 6, 2014 Thursday 5:11 PM GMT

New sugar limits: 26 'mini health time bombs'; Following the World Health Organisation's warning that adults should halve their daily sugar intake to six teaspoons, here are 26 food and drink products described by Action on Sugar, a campaign group, as 'mini health time bombs'

BYLINE: By Edward Malnick

SECTION: NEWS

LENGTH: 662 words

The World Health Organisation has warned that the average adult's daily sugar consumption should be halved to six teaspoons of "free" sugar to help reduce health problems such as obesity and tooth decay. The WHO suggests children should consume even less than six teaspoons.

Here we outline a series of popular food and drink products with total sugar levels amounting to five teaspoons or above and which are described by Dr Aseem Malhotra, a cardiologist and science director of Action on Sugar, a campaign group, as "mini health time bombs".

"The amount of sugar in some of these products is really quite staggering, with many exceeding the safe recommended limits advised by the World Health Organisation," he said. "Perhaps now it is time for health warnings on food similar to what exists on cigarette packets, to help consumers make more informed choices."

New sugar limits: 26 'mini health time bombs'; Following the World Health Organisation's warning that adults should halve their daily sugar intake to six teaspoons, here are 26 food and drink products described by Action on Sugar, a campaign group, as 'mini health time bombs' telegraph.co.uk March 6, 2014 Thursday 5:11 PM GMT

Click here for an explanation of the limits in a Q&A

Starbucks caramel frappuccino with whipped cream (with milk) Contains the equivalent of 11 teaspoons of sugar*

Coca Cola original 330ml 9 teaspoons

Mars Refuel milk drink 350 ml 11 teaspoons

Pepsi regular cola 330ml 9 teaspoons

ASDA dark Belgian chocolate pudding 100g serving 9 teaspoons

Tesco sticky toffee sponge pudding 100g serving 9 teaspoons

Mars chocolate bar 51g 8 teaspoons

Fanta Fruit Twist 330ml 8 teaspoons

Waitrose lemon drizzle pudding 115g serving 8 teaspoons

Red Bull energy drink 250ml 7 teaspoons

Tesco Juice Bar Super Fruita smoothie 250ml 7 teaspoons

Sainsbury's strawberry and banana smoothie 250ml 7 teaspoons

Heinz treacle sponge pudding 75g serving 7 teaspoons

Muller Crunch Corner strawberry shortcake yoghurt 135g 6 teaspoons

Twix bar 50g 6 teaspoons

Innocent Smoothie, mango and passion fruit 200ml serving 6 teaspoons

Sharwood's sweet and sour chicken with rice 375g 6 teaspoons

Cadbury hot drinking chocolate (with semi-skimmed milk) 200ml 6 teaspoons

Crunchie bar 40g 6 teaspoons

Tropicana smooth orange juice 200ml serving 5 teaspoons

Sainsbury's 100 per cent pressed apple juice 200ml serving 5 teaspoons

Capri-Sun orange juice 200ml 5 teaspoons

Sprite sparkling lemon and lime drink 330ml 5 teaspoons

Snickers bar 48g 5 teaspoons

Don Simon clementine juice 200ml serving 5 teaspoons

Hartleys strawberry jelly, per serving 5 teaspoons

*based on 4g of sugar equating to one teaspoon

Some of the products contain milk, whole fruit and vegetables which have naturally occurring sugar that is not included in the World Health Organisation's limits. The six teaspoon limit includes any sugar that is added to foods by the manufacturer, plus that naturally present in honey, syrups, and fruit juices.

Sources: Action on Sugar (survey carried out in January 2014); nutritional values provided for individual products

LOAD-DATE: March 7, 2014

LANGUAGE: ENGLISH

New sugar limits: 26 'mini health time bombs'; Following the World Health Organisation's warning that adults should halve their daily sugar intake to six teaspoons, here are 26 food and drink products described by Action on Sugar, a campaign group, as 'mini health time bombs' telegraph.co.uk March 6, 2014 Thursday 5:11 PM GMT

PUBLICATION-TYPE: Newspaper; Web Publication

JOURNAL-CODE: WEBDTNS

Copyright 2014 Telegraph Media Group Limited
All Rights Reserved

THE TIMES

The Times (London)

March 6, 2014 Thursday
Edition 1;
Ireland

Now we are being urged to halve our sugar intake

BYLINE: Chris Smyth

SECTION: NEWS; Pg. 7

LENGTH: 386 words

People should be encouraged to halve the amount of sugar in their diet, the World Health Organisation says.

Guidance issued yesterday stopped short of changing WHO's recommen dation that sugar make up no more than ten per cent of an adult's daily cal orie intake, but it added fresh advice that cutting sugar to five per cent of our diet would bring additional benefits, mainly by reducing the risk of tooth decay.

The guidance came a day after Pro fessor Dame Sally Davies, the Chief Medical Officer, called for a sugar tax. Experts are considering whether NHS guidance needs to be revised and will report in the summer.

"There is increasing concern that consumption of free sugars, particular ly in the form of sugarsweetened bev erages, may result in both reduced in take of foods containing more nutri tionally adequate calories and an increase in total caloric intake, leading to an unhealthy diet, weight gain and increased risk of non-communicable diseases," the WHO said in a draft re port issued for consultation. "No harm is associated with reducing the intake of free sugars to less than five per cent of total energy, particularly when consid ering the risk of dental caries."

The five per cent limit is roughly equivalent to six teaspoons of sugar a day, while a **fizzy drink** contains about ten teaspoons. The recommendations apply to all sugar added to food as well as those naturally present in honey and fruit Juice, although not those in fruit.

The report, which has been debated by experts for years, says there is strong evidence for a ten per cent limit, but greater uncertainty over the benefits of cutting to five per cent. This sets the scene for furious lobbying before a deci sion.

Ten years ago, the US sugar indus try called for America to cut funding to the WHO after it adopted the ten per cent recommendation.

Alison Tedstone, the director of nutrition and diet at Public Health England, said she would "carefully con sider" the five per cent limit, adding: "Our surveys show that the UK popula tion should reduce their sugar

intake, as average intake for adults is 11.6 per cent, and 15.2 per cent for children ... Too many calories can lead to being over weight or obese which can increase the risk of cardiovascular disease, type 2 diabetes and some cancers."

Matthew Syed, page 18

LOAD-DATE: March 6, 2014

LANGUAGE: ENGLISH

PUBLICATION-TYPE: Newspaper

JOURNAL-CODE: TIM

Copyright 2014 Times Newspapers Limited
All Rights Reserved

DAILY MAIL (London)

March 5, 2014 Wednesday

SLAP A SUGAR TAX ON FIZZY DRINKS AND JUNK FOOD

BYLINE: BY SOPHIE BORLAND HEALTH REPORTER

LENGTH: 627 words

A SUGAR tax may have to be imposed on calorie-laden food and drink to tackle the nation's obesity epidemic, the chief medical officer warned yesterday.

Dame Sally Davies said that increasing the prices of certain products, particularly fruit juice and **fizzy drinks,** could be the only way of changing the public's poor diet.

Being overweight had become normalised', she added, with shops altering the dimensions of their clothes so that people do not realise they are getting fatter.

Dame Sally said she thought that research will find that sugar is addictive', and warned that the crisis is now so severe that today's children will possibly have a lower life expectancy than their parents' generation.

The idea that sugar, rather than fat, causes both obesity and diabetes is gaining support among diet and health experts.

In January an alliance of doctors and academics described sugar as the new tobacco', blaming it for a range of health problems and early death.

The typical Briton consumes 12 teaspoons of sugar a day and some adults consume as many as 46.

Currently just over a quarter of adults are obese, up from only 8 per cent in 1980.

But experts say this figure is expected to more than double by 2050, and there is particular concern about the numbers of obese children.

Dame Sally did not specify how much tax would be added to sweet food and drinks, but experts have previously suggested the rate should be 20 per cent.

This would mean a Mars Bar would go up from 60p to 72p, a can of coke from 70p to 84p and a carton of fruit juice from £1.50 to £1.80

She also did not go into details about which products would be taxed, but experts have previously stated that a sugar content of 15 per cent is too high.

Addressing MPs on the health select committee, Dame Sally, the chief medical officer for England, said: What we are comes off our plate - it is calories. So we will need to continue to explore how we can help people to reduce their calories and that gets you in to culture.

I think the science is going such that we will find that sugar is addictive. I don't think we've managed to get over to the public how calorie-packed fruit juices, smoothies and sodas are.

We need to think about reformulation [changing what's in products] but we also need to have a re-education so that the public know that one's fine but not lots of them. So we may need to move towards some form of sugar tax, but I hope we don't have to.'

She also said clothing manufacturers had changed their dress sizes so the public would not realise they were getting fatter. A woman who fitted in to size 14 clothes now would actually have been a size 16 in the 1970s.

She said: I worry that we have resized women's dress sizes, so we have normalised being overweight.

We have to look at a way not of ostracizing people who are obese and making them feel bad about themselves but somehow making them understand and realise this is pathological and will cause them harm.' She also said that today's children could be the first to have lower life expectancies than their parents.

The average life expectancy has increased over the generations due to better standards of living and medical advances but obesity threatens to reverse this trend, she said.

We have generation of children who because of their weight and lack of activity will not live as long as my generation.

It will be the first generation that live less. That is of great concern.'

In January a report by the National Obesity Forum warned that dire predictions that half the nation will be too fat by 2050 were in fact an underestimate.

It said the obesity epidemic could be far worse than predicted because experts did not factor in how much fatter we are getting as we age - and the extent of the problem in children.

© Daily Mail

LOAD-DATE: March 4, 2014

LANGUAGE: ENGLISH

PUBLICATION-TYPE: Papers

Copyright 2014 Associated Newspapers Ltd.
All Rights Reserved

Daily Mirror

March 5, 2014 Wednesday
Edition 1;

Northern Ireland

'tax sugar to save kids from obesity'

BYLINE: PIERS EADY

SECTION: NEWS; Pg. 14

LENGTH: 140 words

A TAX on sugar may have to be intro duced to halt the obesity epidemic, MPs were warned yesterday.

Dame Sally Davies, England's chief medical officer, said research could prove sugar was addictive.

But she said food and drinks manufacturers were unlikely to re-size sugary products without strong government action.

She told the health select committee: "We may need to introduce a sugar tax. We have normalised being overweight. I do fear that this gener ation of children will live for fewer years than my parents' generation."

Earlier this year doctors called for a **soft drinks** tax to reduce **sugar** intake.

The Department of Health already has a "responsibility deal", a series of voluntary pledges made by food and drink manufacturers designed to tackle obesity. But most of these pledges involved reducing salt and calorie counts in products.

LOAD-DATE: March 5, 2014

LANGUAGE: ENGLISH

GRAPHIC: WARNING Dame Sally Davies

PUBLICATION-TYPE: Newspaper

JOURNAL-CODE: DMR

Copyright 2014 MGN Ltd.
All Rights Reserved

241 of 280 DOCUMENTS

242 of 280 DOCUMENTS

The Express

March 5, 2014 Wednesday

'Tax on sugar' will help fight war on obesity

BYLINE: Rebecca Johnson

SECTION: NEWS; Pg. 18

LENGTH: 338 words

A TAX on sugar may be introduced to curb child and adult obesity, the chief medical officer for England warned yesterday.

Professor Dame Sally Davies told the Commons Health Select Committee being overweight had become "normalised" in Britain.

She claimed the Government should regulate the food and drinks industry to protect people against excess calorie intake.

Dame Sally said: "We need to be both strong and prepared to regulate. I think that the science will find sugar is addictive.

"We haven't managed to get over to the public how calorie packed fruit juices are, smoothies are, colas and carbonated drinks are.

"We need to have a big education to know that one is fine but not lots of them. We may need to move to some sort of sugar tax - but I hope we don't have to."

In January, a group of health experts established the Action on **Sugar** campaign group, which works to reduce the amount of **sugar** added to food and **soft drinks** and educate the public about "hidden **sugars**".

Flavoured water, sports drinks, yoghurts, ketchup, ready meals and even bread have been identified by the group as some everyday foods that contain large amounts of sugar.

Dame Sally added that promoting physical education alone would not solve Britain's obesity crisis.

She said: "I worry that we have re-sized a women's dress size. We have normalised being overweight. We have to find a new way, not of ostracising people who are obese and making them feel bad about themselves, but somehow helping them to understand this is pathological and will cause them harm.

"We have a generation of children who because they are overweight and lack activity may not live as long as my generation. They will be the first generation who will live less."

A National Obesity Forum report warned in January that predictions that half the nation will be too fat by 2050 were, in fact, an underestimate.

It said the obesity epidemic could be far worse because experts did not factor in the extent to which we put on weight as we age or the scale of the problem in children.

LOAD-DATE: March 5, 2014

LANGUAGE: ENGLISH

GRAPHIC: Dame Sally wants education on weight

PUBLICATION-TYPE: Newspaper

JOURNAL-CODE: DXP

Copyright 2014 Express Newspapers
All Rights Reserved

The Guardian - Final Edition

March 5, 2014 Wednesday

Sugar may be addictive and could be taxed, says chief medical officer

BYLINE: Sarah Boseley, Health editor

SECTION: GUARDIAN HOME PAGES; Pg. 4

LENGTH: 494 words

Sugar could be addictive and the government may need to introduce a tax on it in order to combat obesity, England's chief medical officer said yesterday.

Dame Sally Davies made the comments to the Commons health select committee a day ahead of an announcement from the World Health Organisation on new proposed limits for sugar consumption.

Davies told the committee she thought that "research will find sugar is addictive" - a position that has been advanced by some in the US on the back of research by addiction scientists working for the US government's National Institutes of Health. The possibility is strongly disputed by the food industry. "We may need to introduce a sugar tax," she added.

UK groups such as the Children's Food Campaign have been lobbying for a tax on sugar in food and drinks. Doctors also supported the call for a 20% tax on **sugary drinks** in a report from the Academy of Medical Royal Colleges in February. The government, however, has appeared to be disinclined to look at food taxes as a way to tackle the unhealthy eating habits that lead to obesity, heart disease, strokes, diabetes and some forms of cancer.

Davies said she did not believe that food and drink manufacturers would re-size their products - to offer smaller portions of food products containing high levels of sugar, salt and fat - without strong government action.

The food and drink industry is a partner with the government in the public health Responsibility Deal, which asks companies to make pledges on improving the health of their products. There have been pledges on salt and saturated fat reduction and on calorie cuts, but as yet sugar has not been explicitly part of the programme.

Sugar content is on the front of pack labelling that ministers have been promoting, and products with high levels should get a red traffic-light symbol, but the scheme is voluntary and not all companies are compliant.

Davies explained her concern to the committee: "We have normalised being overweight. I do fear that this generation of children will live (for fewer years) than my parents' generation."

Guidance from the World Health Organisation at present suggests that sugar should not make up more than 10% of anybody's calorie intake. It has been revising the guideline over more than a year, however, and will put out new guidance for consultation today.

The WHO commissioned two studies to inform the process: one on the damage that sugar does to teeth, and the other on its effect on obesity.

The latter study, by Jim Mann and colleagues from New Zealand, published in the British Medical Journal last year, found that sugar did not directly cause obesity. However, those who consumed a lot of it, particularly in sweetened drinks, tended to put on weight as it is calorie-dense and the drinks did not make people feel full, so they continued to eat.

Captions:

20%

The level of tax on **sugary drinks** that was proposed by doctors at the Academy of Medical Royal Colleges last month

LOAD-DATE: March 5, 2014

LANGUAGE: ENGLISH

PUBLICATION-TYPE: Newspaper

Copyright 2014 Guardian Newspapers Limited
All Rights Reserved

i-Independent Print Ltd

March 5, 2014
First Edition

Food taxes are punitive - let's make 'good' foods cheaper; Comment

SECTION: EDITORIAL; Pg. 5

LENGTH: 262 words

Dame Sally Davies' timing is impeccable. Today the World Health Organisation is expected to announce new, lower recommendations for sugar consumption. Taxing the stuff is, in principle, one good way to reduce people's intake.

Dame Sally is in good company. Hers is the seventh proposal for food taxes in Britain by prestigious doctors and academics in the past two years.

None have been adopted. Her proposal is unlikely to fare better, for two reasons. Such taxes are economically ineffective and politically unacceptable.

Recent research has shown the limited effect taxes would have on **soft drinks**, the major source of **sugar** in the UK diet.

A 10 per cent tax would reduce average consumption by 7.5ml a day, less than a sip. A 20 per cent tax would cut daily sugar intake by one gram, four calories. More important are the political obstacles. Two years ago consumers rebelled against "the pasty tax". The Chancellor George Osborne quickly backed off.

Since then, in the US, referenda have consistently rejected soft drinks taxes in New York, Washington, Philadelphia and California.

Even in Denmark, one of the most tax-tolerant nations on earth, the government introduced, then rapidly repealed taxes on fat and sugar.

After those experiences, no rational democratic politician will touch food taxes for years.

Food taxes are punitive, especially on the poor. Instead of raising the cost "bad" foods, we need to make "good" foods cheaper. It is feasible, but that's another, longer story.

Professor Jack Winkler, former Professor of Nutrition Policy, London Metropolitan University

LOAD-DATE: March 4, 2014

LANGUAGE: ENGLISH

PUBLICATION-TYPE: Newspaper

JOURNAL-CODE: III

Copyright 2014 Independent Print Limited
All Rights Reserved

The INDEPENDENT
Independent.co.uk

March 5, 2014 Wednesday 7:35 PM GMT

Taxing sugar would be illiberal and ineffective;
Instead, manufacturers should reduce the size of chocolate bars and bags of sweets

BYLINE: Editorial

SECTION: VOICES

LENGTH: 338 words

Sugar is a killer. Despite recent increases in the world price of this commodity, it remains an easy way for the food industry to generate profits. The sugar content of much fast food and takeaways, in particular, is too high, and we are taking the sweet road to mass obesity.

It costs the NHS dear, it shortens lifespans and destroys the quality of life of individuals and families after strokes, heart attacks and the onset of diabetes. In many ways, it is the "new tobacco".

And yet taxing it in the same way we tax tobacco and alcohol may not be the best answer. Much could be achieved through voluntary action by the supermarkets, the fast-food chains and other interested parties.

So-called "meal deals" and other clever marketing encourage us to pile on the calories. The supermarkets could take more care about promoting chocolate, biscuits and **fizzy drinks**.

The manufacturers should reduce the size of chocolate bars and bags of sweets. (The same goes for the modern, inflated "grab bag" of crisps, about twice the size of the conventional pack of say 20 years ago).

Such voluntary action, and improved labelling, would be more effective than tax, because it removes temptation in the first place. What's more, a sugar tax will only work if it is set at a deterrent level, as with

cigarettes. Unless that is done, the main impact will be to drive up the cost of the weekly shop, hitting poorer families hardest - but without reducing consumption.

Indeed, having to make extra room in the budget for sweet drinks and cookies would mean less cash for fresh fruit. A truly deterrent level of taxation - pricing cola as if it were whisky - would be politically unacceptable.

Besides, with special taxes on carrier bags, the possibility of minimum pricing for alcohol, varying duties on different types of booze and tobacco and different VAT rates on warm/hot snacks from the restaurant, our supermarkets will soon come to resemble fiscal adventure playgrounds, and to no great purpose.

Tax is not the answer to every social ill.

LOAD-DATE: March 6, 2014

LANGUAGE: ENGLISH

PUBLICATION-TYPE: Newspaper; Web Publication

JOURNAL-CODE: WEBI

Copyright 2014 Independent Digital News and Media Limited
All Rights Reserved

The INDEPENDENT
Independent.co.uk

March 5, 2014 Wednesday 5:44 PM GMT

I'm so glad Dame Sally has 'Lent' me a good reason to become healthier;
It's sugar that's going to get the boot this month

BYLINE: Simon Kelner

SECTION: COMMENT

LENGTH: 492 words

As a pleasure seeker who understands the need to be conscious of my own mortality, I'm always looking for a reason to give something up, and Lent seems as good a reason as any.

I'm not a Roman Catholic, but I do like the idea of a period of self-denial at this time of year, for health reasons if not for spiritual or religious purification. I don't lack for willpower, and last year I gave up alcohol for 11 months, an experiment in abstinence which came to an end in December with the combined assault of my birthday, a number of parties and, of course, Christmas. (I have since realised that my alcohol intake over the month probably equated to what most people drink in the course of a year.)

I don't smoke or eat red meat, so I was struggling over what to give up this Lent until I heard a radio phone-in responding to the proposal by the Chief Medical Officer, Dame Sally Davies, that the government should introduce a **sugar** tax to curb growing rates of obesity. It was hard to keep up with the random litany of statistics, but they all added up, in my view, to supporting Dame Sally's opinion. The UK consumes 5,727 million litres of **sugary soft drinks** every year. There are four teaspoons of **sugar** in a can of peas, and five

teaspoons in a low-fat yoghurt. If 20p tax were added to a litre of **soft drink**, more than £1bn could be raised to be spent on health initiatives. Smoothies are the short cut to obesity. About 60 per cent of Britons are considered overweight or obese. And so on. I resolved that, this Lent, it was **sugar** that was going to get the boot.

I have eaten at enough restaurants in my time - and have the figure to prove it - to know that if something tastes good, it's usually for one of four, unadvertised, reasons - butter, salt, cream or sugar. So I realise my mission is going to be really tough, because sugar is the hidden persuader, and, according to Dame Sally, will be proven to be addictive. She says that a tax may be the most effective way of changing our eating and drinking regime. "We have normalised being overweight," she said, while others in the medical profession say sugar is a dangerous, life-threatening drug.

The arguments against a tax on sugary products are familiar enough. For instance, this is a tax on the poorest people, and while there is a class dimension to this debate - the middle-class person who asks for two sugars in a cup of tea is regarded with as much astonishment as the one who strikes up a Marlboro at a dinner - this is where the Government comes in, legislating to change behaviour for the general betterment of society (treating diseases caused by unhealthy lifestyles costs the NHS £6 billion a year). The food and drink industry believes in education not legislation. People should be free to make their own choices, say libertarians.

Well, I've made my choice. I'm not going to wait until the Nanny State comes knocking. Here goes. Expect some bad-tempered columns over the next month.

LOAD-DATE: March 6, 2014

LANGUAGE: ENGLISH

PUBLICATION-TYPE: Newspaper; Web Publication

JOURNAL-CODE: WEBI

Copyright 2014 Independent Digital News and Media Limited
All Rights Reserved

The INDEPENDENT
Independent.co.uk

March 5, 2014 Wednesday 12:58 AM GMT

Tax 'addictive' sugar to combat growing levels of obesity, says chief medical officer;
Dame Sally Davies believes levy may be needed to reduce intake, but critics claim move would 'hit poorest hardest'

BYLINE: Ian Johnston, Paul Bignell

SECTION: FRONTPAGE

LENGTH: 601 words

The Government could be forced to bring in a tax on sugar to help combat growing levels of obesity, the Chief Medical Officer for England has warned.

Tax 'addictive' sugar to combat growing levels of obesity, says chief medical officer; Dame Sally Davies believes levy may be needed to reduce intake, but critics claim move would 'hit poorest hardest'
Independent.co.uk March 5, 2014 Wednesday 12:58 AM GMT

Dame Sally Davies told the Health Select Committee she expected ongoing research to establish that sugar is addictive. And she said that being overweight had become "normalised" in the UK and feared that today's children would live shorter lives than her parents' generation.

Responding to Dame Sally's remarks, a leading food industry body insisted sugar was not a cause of obesity - when eaten as part of a balanced diet - and said a tax would hit "the poorest families hardest".

She was speaking ahead of an expected announcement by the World Health Organisation today that the recommended level of sugar in people's diet be reduced dramatically. A well-placed source told The Independent that the current recommended figure of 10 per cent of total energy intake from "free sugars" - mainly refined and fruit sugars - would be cut in half to 5 per cent.

A WHO spokesman declined to comment.

Speaking to the committee, Dame Sally said that she believed that "research will find sugar is addictive".

She said: "We may need to move toward some kind of sugar tax, but I hope we don't have to. We have normalised being overweight. I do fear this generation of children will live less than my parents' generation."

About 64 per cent of adults in the UK are considered to be overweight or obese. Obesity is a risk factor for diabetes, heart disease and other serious medical conditions and costs the NHS billions every year.

In September, sugar was described as "the most dangerous drug of the times" by Paul van der Velpen, head of Amsterdam's health service.

Professor Terence Stephenson, chairman of the Academy of Medical Royal Colleges, which produced a report last year calling for a tax on **sugary drinks**, welcomed the idea of a more general tax.

"We would be entirely supportive of the principle," he said.

He said sugar gave people a "rapid high" which then drops while they are still producing insulin, resulting in a "craving" for more.

Professor Stephenson said concern particularly centred on processed foods where sugar and corn syrup were added to "sweeten the food and give children and adults a palate that likes sweet things".

He rejected suggestions that a sugar tax would represent too high a level of state interference. "The nanny state can be a caring state," he said, adding that government intervention had brought about things beneficial things like seat belts and drink-drive limits.

However Terry Jones, of the Food and Drink Federation, said many foods were taxed at the VAT rate of 20 per cent and "any additional taxation of food will hit the poorest families hardest at a time when they can least afford it".

"We need all parties to act together to empower more appropriate choices by consumers," he said.

"Sugars, or any other nutrient for that matter, consumed as part of a varied and balanced diet are not a cause of obesity, to which there is no simple or single solution.

"That's why the food industry has been working on a range of initiatives with other players to tackle obesity and diet related diseases through a number of interventions."

Labelling on food enabled consumers to find out the sugar content of the food that they buy, Mr Jones said, adding that the industry had taken action to reduce sugar along with salt and saturated far.

"Delivering on these commitments will require considerable research and investment as well as consumer acceptance of new recipes that can result in changes in taste, texture and ingredients," he added.

LOAD-DATE: March 5, 2014

LANGUAGE: ENGLISH

PUBLICATION-TYPE: Newspaper; Web Publication

JOURNAL-CODE: WEBI

Copyright 2014 Independent Digital News and Media Limited
All Rights Reserved

The INDEPENDENT

Independent.co.uk

March 5, 2014 Wednesday 12:01 AM GMT

i Deputy Editor's Letter: Sugar tax

BYLINE: Rhodri Jones

SECTION: FRONTPAGE

LENGTH: 274 words

"Another tax? Aren't we hit with enough taxes already!" I hear you say as you turn the page. Our cover story, which adds another voice to the growing campaign against sugar and the damage it is doing to our health, won't be popular among chocoholics.

Speaking to the Health Select Committee yesterday, the Chief Medical Officer Dame Sally Davies said that "sugar has become addictive" and that we have "normalised being overweight". But is taxing the answer? If it really is addictive, surely it will just hit the consumer in the pocket, topping up the Treasury coffers in the process.

The food industry says that it has been working hard to reduce sugar in products - something which can't really be disputed, even if much of it has been accidental. Manufacturers have long been raising prices while quietly shrinking the size of their chocolate bars. But, of course, this isn't just about chocolate.

When consumers look for more healthy options, we often focus on reducing our salt and calorie intake, paying no more than casual attention to sugar content. You don't need to have many cakes, biscuits, **fizzy drinks** or ready meals to quickly reach the recommended daily amount of 70g for men, or 50g for women. Sugary foods and drinks not only cause tooth decay, they contribute to your risk of diabetes and being overweight or obese.

So therein lies the answer. While causing financial pain may be a short-term answer, education provides a more sustainable solution. Do the two have to work in conjunction? The choices are out there - the incentive of living a longer, healthier life should be enough.

i@independent.co.uk

Twitter.com: @jonesrhodri

LOAD-DATE: March 5, 2014

LANGUAGE: ENGLISH

PUBLICATION-TYPE: Newspaper; Web Publication

JOURNAL-CODE: WEBI

Copyright 2014 Independent Digital News and Media Limited
All Rights Reserved

London Evening Standard
standard.co.uk

March 5, 2014 Wednesday 5:50 PM GMT

Halve sugar intake for health - WHO

SECTION: PA NEWS FEEDS

LENGTH: 998 words

People should cut their sugar intake in half if they want to reap health benefits, the World Health Organisation (WHO) has said.

Current WHO guidelines say sugars should make up less than 10% of total energy intake per day for adults and children but experts believe slashing this to 5% is more "ideal".

In new draft guidelines, which are subject to consultation, the WHO maintains its original advice that sugars should be less than 10% of total energy intake per day.

But it argues that cutting this intake to less than 5% would bring "additional health benefits" and is the figure people should aim for.

For adults of a normal weight, this would mean cutting intake from around 50g - about 12 level teaspoons - of sugar per day to less than 25g.

Health experts backed the move but called on WHO to make the 5% an official recommendation.

They also criticised the UK Government's responsibility deal with the food and drinks industry, which sees companies such as chocolate manufacturers signing up to voluntary codes.

The WHO's limits on intake of sugars apply to all monosaccharides (such as glucose, fructose) and disaccharides (such as sucrose or table sugar).

These are added to food by the manufacturer, the cook or the consumer, and are also sugars that are naturally present in honey, syrups, fruit juices and fruit concentrates.

The WHO said much of the sugars consumed today are "hidden" in processed foods such as sweets, with sugary **fizzy drinks** having about 10 teaspoons of sugar.

The new guidelines follow several studies on the impact of sugar on obesity and dental cavities.

Dr Francesco Branca, director for nutrition for health and development at the WHO, told a news conference that the 10% target was a "strong recommendation" while the 5% target was "conditional", based on the evidence.

He added: "Five percent is the ideal one and the 10% is the more realistic one.

"We have few countries (hitting) below 10%. But, yes, we should aim for 5% if we can."

Dr Branca said obesity affects half a billion people around the world and is on the rise among all age groups.

He said the recommendation for less than 5% was important because it told countries that reductions to "below 5% are even better".

Announcing the revisions, the WHO said in a statement: "WHO's current recommendation, from 2002, is that sugars should make up less than 10% of total energy intake per day.

"The new draft guideline also proposes that sugars should be less than 10% of total energy intake per day.

"It further suggests that a reduction to below 5% of total energy intake per day would have additional benefits."

The news comes after England's chief medical officer, Professor Dame Sally Davies, told MPs yesterday that a "sugar tax" may have to be introduced to curb child and adult obesity.

She said being overweight had become "normalised" in Britain and the Government should regulate the food and drinks industry to protect people against the dangers of excess calorie consumption.

"We need to be both strong and prepared to regulate. I think that the science is going such that that we will find sugar is addictive," she said.

"We haven't managed to get over to the public how calorie packed fruit juices are, smoothies are, colas and carbonated drinks. We need to have a big education to know one is fine, but not lots of them.

"We may need to move to some sort of sugar tax, but I hope we don't have to."

Asked if the Prime Minister was ready to consider introducing new laws or taxes to cut sugar consumption, David Cameron's official spokesman said yesterday: "The Prime Minister's view is that the significant things the Government is doing through the responsibility deal with industry is the right way to be going about this, because at the heart of this is ensuring people have the public health information."

Today, Action on Sugar - a group of experts and academics in obesity, health and nutrition - called on the WHO to make its official recommendation less than 5%.

The NHS currently recommends added sugars make up no more than 10% of calorie intake from food and drink each day.

Action on Sugar chairman Professor Graham MacGregor; said: "Added sugar is a completely unnecessary part of our diets, contributing to obesity, Type 2 diabetes and tooth decay.

"We strongly urge the WHO to recommend reducing sugar intakes to below 5% daily calories, as this will have the biggest impact on our health.

"We have known about the health risks of sugar for years and yet nothing substantial has been done - new recommendations will be a wakeup call to the Department of Health and the Government to take action on sugar now by forcing the industry to slowly reduce the huge amount of sugar added by the food industry across the board.

"Setting targets for sugar reduction will not rely on the industry-determined responsibility deal calorie pledge, which has had no measurable effect on calorie intake.

"Unless they act now, obesity and diabetes are going to completely overwhelm the NHS."

Simon Capewell, professor of clinical epidemiology at the University of Liverpool, UK, said: "A 5% target would reflect extensive scientific evidence linking excess sugars with obesity, diabetes, heart disease and common cancers (and rotten teeth in kids).

"Brits currently consume about three times too much sugar, 15% not 5%.

"This is because of the huge amounts of added sugars hidden in most processed foods: soups, yoghurts, ready meals, **sugary drinks**, fruit juices and smoothies.

"The UK Government and the industry have a clear duty to slash the sugars hidden in these foods, to protect our kids."

He said taxes on **sugary drinks** were working in the USA, France, Hungary and Finland.

Science director of Action on Sugar, cardiologist Dr Aseem Malhotra said: "I fully welcome the calls for the WHO to recommend intakes to be reduced to 5%, but this needs to be translated into something meaningful.

"In particular, consumers need to know how much sugar is being added to processed foods, which currently isn't the case."

LOAD-DATE: March 6, 2014

LANGUAGE: ENGLISH

PUBLICATION-TYPE: Newspaper; Web Publication

JOURNAL-CODE: WEBES

Copyright 2014 Evening Standard Limited
All Rights Reserved

The Sun (England)

March 5, 2014 Wednesday
Edition 2;
National Edition

'Put a tax on sugar'

SECTION: NEWS; Pg. 2

LENGTH: 86 words

SWEETS and **sugary drinks** could be TAXED to tackle obesity.

Dame Sally Davies, chief medical officer for England, told MPs yesterday: "We have normalised being overweight. We may need to introduce a sugar tax."

Campaigners have dubbed sugar "the alcohol of childhood". Nearly a third of kids and a quarter of adults are overweight or obese. Dame Sally added: "I fear this generation of children will live less than my parents' generation."

Many food firms have vowed to cut calories and salt.

Fry-up cancer risk - Page 24

LOAD-DATE: March 5, 2014

LANGUAGE: ENGLISH

PUBLICATION-TYPE: Newspaper

JOURNAL-CODE: SUN

Copyright 2014 NEWS GROUP NEWSPAPERS LTD
All Rights Reserved

The Telegraph
telegraph.co.uk

March 5, 2014 Wednesday 6:17 PM GMT

World Health Organisation advises halving sugar intake;
The World Health Organisation advises halving the amount of sugar that people consume daily, after Britain's chief medical officer Dame Sally Davies said a sugar tax may be needed to curb obesity rates

BYLINE: By Edward Malnick

SECTION: NEWS

LENGTH: 929 words

Sugar consumption should be halved to help reduce health problems such as obesity and tooth decay, the World Health Organisation (WHO) has warned.

The WHO set out draft guidance advising a dramatic reduction in sugar intake amid growing evidence that it contributes to chronic diseases.

The move follows calls by some leading scientists and campaigners for the current recommended daily limits on sugar intake to be halved to 5 per cent of an individual's overall calorie consumption - the equivalent of six "level" teaspoons a day for the average adult.

The new guidance proposed by the WHO retains the current recommended limit of 10 per cent - around 12 teaspoons of sugar.

However, the WHO states in the draft guidelines that a further reduction to 5 per cent "would have additional benefits", with a senior official at the health body describing the lower figure as the "ideal" limit for which people should now aim.

The limits apply to all "free" sugar, which is sugar that is added to foods by the manufacturer, plus that naturally present in honey, syrups, and fruit juices.

The move comes a day after Britain's chief medical officer suggested that a sugar tax should be introduced in order to curb obesity rates, saying research was likely to find that the ingredient is "addictive".

Dr Francesco Branca, director for nutrition for health and development at the WHO, said that the 10 per cent target was a "strong recommendation" while the 5 per cent target was "conditional", based on evidence considered by the health body.

He added: "Five percent is the ideal one and the 10 per cent is the more realistic one.

"We have few countries (hitting) below 10 per cent. But, yes, we should aim for 5 per cent if we can."

Dr Branca said obesity affects half a billion people around the world and is on the rise among all age groups.

He said the recommendation for less than 5 per cent was important because it told countries that reductions to "below 5% are even better".

The WHO warned that much of the sugars consumed today are "hidden" in processed foods "that are not usually seen as sweets".

Dr Branca said a single can of **fizzy drink** could exceed the amount of sugar that children should have in whole day under the current 10 per cent limit.

The new draft guidelines, which are now out to consultation, were published after the WHO's experts considered two studies which analysed the impact of sugar on dental cavities on obesity.

The report on tooth decay, by scientists at Newcastle University suggested that the limit should be halved to 5 per cent.

The move was also backed by campaign groups such as Action on Sugar, which warns that current levels of sugar intake are "unsustainable" and need to be radically reduced to help tackle obesity and chronic diseases such as diabetes. The group's advisors include Prof David Haslam, chairman of the National Institute for Health and Care Excellence, the NHS rationing body.

However, campaigners will be disappointed that the WHO has not gone further and formally halved the 10 per cent recommendation.

Dr Aseem Malhotra, science director of Action on Sugar, said: "It is a step in the right direction because they have acknowledged it should be less than 5 per cent, but it needs to be translated into something meaningful for the consumer."

Simon Capewell, professor of clinical epidemiology at the University of Liverpool said: "A 5 per cent target would reflect extensive scientific evidence linking excess sugars with obesity, diabetes, heart disease and common cancers."

The industry denies the claims over the effect of sugar on health, saying expert committees had found that the "balance of evidence" exonerated the substance from contributing to "lifestyle diseases".

However the WHO warned of "increasing concern" that consumption of free sugars can contribute towards diseases such as diabetes and tooth decay.

The proposals come a day after Dame Sally Davies, the chief medical officer for the UK, said she believed "research will find sugar is addictive" and that "we may need to introduce a sugar tax".

Addressing MPs on the Commons health select committee, she said: ""We have a generation of children who, because they're overweight and their lack of activity, may well not live as long as my generation.

"They will be the first generation that live less, and that is of great concern."

Alison Tedstone, Director of Nutrition and Diet at Public Health England (PHE), the official advisory body, said: "PHE welcome the new WHO draft guidelines to reduce sugar intake to 10 per cent of total daily calorie intake and will carefully consider the suggestion that a further reduction of sugar to below 5 per cent of total energy intake per day would have additional benefits."

A Department of Health spokesman added: "We are already making real progress in getting food and drink manufacturers to cut the amount of calories, including sugar, in their foods through the public health Responsibility Deal."

Barbara Gallani, director of regulation, science and health at the Food and Drink Federation, an industry body, said manufacturers were already working to reduce overall calories.

She added: "Where a conditional recommendation of a further reduction of sugars intake to below 5 per cent of total energy is made, the report cautions that there is greater uncertainty about the quality of the underpinning science base. WHO emphasise the need for 'substantial debate and involvement of stakeholders before this recommendation can be adopted as policy'."

John Yudkin: the man who tried to warn us about sugar

World Health Organisation advises halving sugar intake; The World Health Organisation advises halving the amount of sugar that people consume daily, after Britain's chief medical officer Dame Sally Davies said a sugar tax may be needed to curb obesity rates telegraph.co.uk March 5, 2014 Wednesday 6:17 PM GMT

LOAD-DATE: March 6, 2014

LANGUAGE: ENGLISH

PUBLICATION-TYPE: Newspaper; Web Publication

JOURNAL-CODE: WEBDTNS

Copyright 2014 Telegraph Media Group Limited
All Rights Reserved

THE TIMES

The Times (London)

March 5, 2014 Wednesday
Edition 1;
Scotland

Sugar is addictive, warns top doctor

BYLINE: Rosemary Bennett

SECTION: NEWS; Pg. 4

LENGTH: 234 words

Sugar is an addictive substance, research is likely to prove, and some form of sugar tax may be needed to alert the public to its dangers, the country's most senior doctor has warned (Rosemary Bennett writes).

Dame Sally Davies, the Chief Medical Officer, said people did not realise that fruit Juice, smoothies, and **fizzy drinks**, were "highly calorific" and they should cut down.

She criticised as inadequate the Government's voluntary approach with food companies, and urged ministers to regulate them.

Companies were not going to re-size and reformulate their products unless forced to, she said.

Dame Sally made clear her support for a sugar tax to a committee of MPs. "I think with the science we will find sugar is addictive," she said. "We need a big education [programme] there and may need to move to some form of sugar tax."

Ministers needed put more pressure on food companies to make the food in the shops healthier, she said. "They have to be strong and be prepared to regulate," she said.

The Academy of Medical Royal Colleges, an umbrella group for the entire medical profession, has called for a 20 per cent tax to reduce obesity.

Research by Oxford and Reading universities said that a 20 per cent tax would reduce the number of obese adults in Britain by 180,000.

Britain consumes more than 5,727 million litres of **sugary drinks** a year, so a 20p tax per litre would raise more than £1.1 billion.

LOAD-DATE: March 5, 2014

LANGUAGE: ENGLISH

PUBLICATION-TYPE: Newspaper

JOURNAL-CODE: TIMscot

Copyright 2014 Times Newspapers Limited
All Rights Reserved

MailOnline

March 4, 2014 Tuesday 11:43 PM GMT

Slap a sugar tax on fizzy drinks and junk food: Shock call by chief medical officer

BYLINE: SOPHIE BORLAND

SECTION: HEALTH

LENGTH: 838 words

- Dame Sally Davies said price of some products should be increased
- She said obesity had become 'normalised' in British society
- Dame Sally said research 'will find that sugar is addictive'

A sugar tax may have to be imposed on calorie-laden food and drink to tackle the nation's obesity epidemic, the chief medical officer warned yesterday.

Dame Sally Davies said that increasing the prices of certain products, particularly fruit juice and **fizzy drinks**, could be the only way to change the public's poor diet.

Being overweight had become 'normalised', with shops altering the dimensions of their clothes so that people do not realise they are getting fatter.

Dame Sally said she thought that research 'will find that sugar is addictive', and warned that the crisis is now so severe that today's children will possibly have a lower life expectancy than their parents' generation.

The idea that sugar, rather than fat, causes both obesity and diabetes is gaining support among diet and health experts.

In January an alliance of doctors and academics described sugar as 'the new tobacco', blaming it for a range of health problems and early death.

The typical Briton consumes 12 teaspoons of sugar a day and some adults consume as many as 46.

Currently just over a quarter of adults are obese, up from only 8 per cent in 1980.

But experts say this figure is expected to more than double by 2050, and there is particular concern about the number of obese children.

Dame Sally did not specify how much tax would be added to sweet food and drinks, but experts have previously suggested the rate should be 20 per cent.

This would mean a Mars Bar would go up from 60p to 72p, a can of coke from 70p to 84p and a carton of fruit juice from £1.50 to £1.80

She also did not go into details about which products would be taxed, but experts have previously stated that a sugar content of 15 per cent is too high.

Addressing MPs on the health select committee, Dame Sally, the chief medical officer for England, said: 'What we are comes off our plate - it is calories. So we will need to continue to explore how we can help people to reduce their calories and that gets you in to culture.

I WAS ON 50 TEASPOONS A DAY

Nikki Oakley became so addicted to sugar that she consumed the equivalent of 50 teaspoons a day - but she has transformed her life by kicking the habit.

The 45-year-old gorged on chocolate biscuits or sugar-laden cereal bars for breakfast, followed by sandwiches, crisps and cakes for lunch and ready meals followed by a pudding in the evening. She shunned tea and coffee during the day in favour of sugary **fizzy drinks**.

Mrs Oakley said her addiction was so severe that she was left in tears, short-tempered, listless and depressed and suffering from headaches after quitting the 'white stuff' last September.

'It took a fortnight for those feelings to subside,' said the mother of two, from Redditch, Worcestershire.

'It was a cycle - I ate sugary foods as a pick-me-up, but after the high came a low and I would need more. But once I'd got through those first few weeks, I started to feel so much better.'

The keen jogger said she was running faster than ever and had lost 18lb.

'I think the science is going such that we will find that sugar is addictive. I don't think we've managed to get over to the public how calorie-packed fruit juices, smoothies and sodas are.

'We need to think about reformulation [changing what's in products], but we also need to have a re-education so that the public know that one's fine but not lots of them. So we may need to move towards some form of sugar tax, but I hope we don't have to.'

She also said clothing manufacturers had changed their dress sizes so the public would not realise they were getting fatter. A woman who fitted in to size 14 clothes now would actually have been a size 16 in the 1970s. 'I worry that we have resized women's dress sizes, so we have normalised being overweight,' she said.

'We have to look at a way not of ostracizing people who are obese and making them feel bad about themselves but somehow making them understand and realise this is pathological and will cause them harm.'

She also said that today's children could be the first to have lower life expectancies than their parents.

The average life expectancy has increased over the generations due to better standards of living and medical advances but obesity threatens to reverse this trend, she said.

'We have generation of children who because of their weight and lack of activity will not live as long as my generation,' she added. 'It will be the first generation that live less. That is of great concern.'

In January a report by the National Obesity Forum warned that dire predictions that half the nation will be too fat by 2050 were in fact an underestimate.

It said the obesity epidemic could be far worse than predicted because experts did not factor in how much fatter we are getting as we age - and the extent of the problem in children.

LOAD-DATE: March 4, 2014

LANGUAGE: ENGLISH

PUBLICATION-TYPE: Web Publication

JOURNAL-CODE: WEBDM

Copyright 2014 Associated Newspapers Ltd.
All Rights Reserved

Mirror

mirror.co.uk

March 4, 2014 Tuesday 8:54 PM GMT

Medical chief calls for tax on SUGAR to halt obesity epidemic; England's chief medical officer Dame Sally Davies says sugar could be addictive - and fears children today may not live as long as their grandparents' generation

BYLINE: By Piers Eady

SECTION: NEWS,UK NEWS

LENGTH: 208 words

England's top doctor has said a tax on sugar may be needed to halt the country's growing obesity epidemic.

Dame Sally Davies, England's chief medical officer, told MPs today that research could prove sugar is addictive.

And she warned that children today may not live as long as their grandparents' generation because of the amount of sugar in their diet.

Dame Sally said: "We may need to introduce a sugar tax.

"We have normalised being overweight. I do fear that this generation of children will live for fewer years than my parents' generation."

Davies said she did not believe food and drink manufacturers would re-size sugary products without strong government action.

Earlier this year doctors called for a **soft drinks** tax to reduce **sugar** intake.

However, the food industry says it has been working to reduce sugar in its products.

The Department of Health already has a 'responsibility deal' - a series of voluntary pledges made by food and drink manufacturers designed to tackle obesity.

But most of these pledges involved reducing salt and calorie count rather than sugar content.

Ministers are also pursuing plans for voluntary labels on the front of food packaging to include information about sugar.

Sugar's Connection to Obesity and Heart Disease

LOAD-DATE: September 1, 2014

LANGUAGE: ENGLISH

PUBLICATION-TYPE: Newspaper; Web Publication

JOURNAL-CODE: WEBDMI

Copyright 2014 Trinity Mirror, Plc.
All Rights Reserved

MailOnline

March 3, 2014 Monday 3:08 PM GMT

Special report: Sugar - the bitter truth?

BYLINE: ANNA PURSGLOVE

SECTION: YOU MAG

LENGTH: 1401 words

For four decades we've been led to believe that fat is the ultimate food enemy, but we've been fed a lie: the real danger is sweet, addictive - and found in almost everything we eat

So what do you know about eating and getting fat? If you're the average British person then it's probably something along these lines: eating too much fat will make me obese, clog up my arteries and lead to a heart attack, so I should follow a low-fat diet and eat lots of fruit and vegetables.

Wrong. While you were busy fretting over your saturated fats and dietary cholesterols, there was a far more potent food nasty lurking in your kitchen: sugar. The amount of sugar we eat is now being blamed not just for the obesity epidemic but for heart disease, type 2 diabetes and soaring cancer rates. It's not just the excess calories we're consuming; the problem lies in the way we metabolise sugar.

'We have been sold an absolute lie about food and health,' says Zoë Harcombe, nutritionist and author of The Obesity Epidemic. 'It has been put about since the 1970s that fat was the bad guy, yet the only fats we

know to be harmful are trans fats, and these are almost exclusively man-made. If the fat occurs naturally then it's fine - no exceptions. Sugar, on the other hand, when added to food, is almost uniformly bad.'

So why was this information hidden from us? 'Because,' says Harcombe, 'the commercial food producers, who rely on sugar, represent a huge and powerful lobby. It's not just the obvious brands, such as **fizzy drinks** manufacturers, that would suffer if sugar were removed from our diets. Sugar is added to just about everything you buy ready-made: bread, sauces, ready meals, drinks, tinned foods... The list is endless.' Even baked beans can contain two and a half teaspoons of sugar in just half a tin. Furthermore, say campaigners, the low-fat industry (now worth billions) is absolutely reliant on sugar because the only way to stop low-fat food tasting like cardboard is to replace fat with sugar.

FRUCTOSE: THE FACTS

- Fructose is found in fruit, sucrose (table sugar) and in high fructose corn syrup. It is a simple sugar and is sweeter than glucose.
- Sucrose (the sugar that we commonly add to food) is made up of 50 per cent glucose and 50 per cent fructose.
- High fructose corn syrup (containing more fructose than glucose) commonly replaces sucrose in the US food industry because it is both sweeter and cheaper. In the UK, it can also be labelled as 'glucose-fructose syrup' or 'HFCS'.
- Because it causes a lower blood sugar spike than sucrose or glucose, and therefore has a low glycaemic index, manufacturers are allowed to claim that fructose is 'healthier' than the other two.

Robert Lustig MD is a paediatric endocrinologist and childhood obesity expert at the University of California and one of the most vocal of the anti-sugar campaigners. Lustig's 90-minute lecture 'Sugar: The Bitter Truth' (viewed over four million times on YouTube) is uncompromising in its condemnation of sugar as the cause of the obesity epidemic and its assertion that governments (under pressure from powerful food producers) have kept this fact hidden.

Lustig makes the point that we have been trying the low-fat approach for 40 years and it has failed to make us slimmer. In fact we've got fatter - and sicker (six per cent of adults in the UK are now registered diabetic).

While the percentage of our daily calorie intake accounted for by fat has dropped steadily, the incidence of obesity and related illnesses, including type 2 diabetes, has rocketed. 'Sugar is the problem,' states Lustig, 'and yet public health officials are still advising us to follow a low-fat diet. It's Albert Einstein's definition of insanity - doing the same thing over and over again and expecting different results.'

It might all have been different if, 40 years ago, we had listened to John Yudkin, a British physiologist and nutritionist. His 1972 book Pure, White and Deadly argued that we were massively overeating sugar, which was not only making us fat, but also causing liver damage, heart disease and cancer. However, these beliefs earned him some powerful enemies in the sugar industry. In 1979 the World Sugar Research Organisation rubbished his work as 'science fiction', while the food industry got squarely behind the theory that saturated fat was the dietary devil.

Yudkin's problem, adds Lustig, was that he could see the correlation between sugar and disease but couldn't quite prove it. Fast forward to 2014 and the anti-sugar lobby now has science on its side. We now know, for example, that carbohydrate (of which sugar is the most dangerous form), not natural fats, is the driver of the type of 'bad' LDL cholesterol that leads to heart disease. And numerous studies have linked both overproduction of insulin (which stimulates cell growth) and obesity to increased risks of various cancers including that of the breast and liver.

What Lustig is gunning for in particular is fructose, a fruit sugar that makes up 50 per cent of the refined sugar found in virtually everything you buy pre-made. The problem with fructose is twofold. Firstly, there is no hormone to remove fructose from our bloodstream (unlike glucose, which stimulates insulin production).

It is therefore left to the liver to remove it and when the liver is overwhelmed it converts fructose to liver fat, which ups our chances of developing insulin resistance (a precursor to diabetes), hardened arteries and heart disease. Secondly, fructose suppresses the hormone leptin, which tells you when you're full. In other words, your brain lets you consume it without limit.

It's going to be hard to turn back the sugar clock, says Lustig. But a good place to start would be drinks, because it's particularly bad to take on sugar in liquid form. 'Think of it this way,' says David Gillespie, an Australian lawyer, father of six and campaigning author of Sweet Poison. 'If you sit your kids down in front of a fruit bowl they are only going to eat one orange, because an orange contains fibre, which makes you full. There is no problem there - my kids think of fruit as nature's dessert. Now take out all the fibre [by turning it into a juice] and suddenly that child can consume way more than one orange in a sitting.'

The amount of fructose lurking in your juice carton also depends on whether the manufacturer has added extra sugar. However, to give an idea of why anti-sugar campaigners are so worried about fruit juice, 12fl oz of unsweetened apple juice contain nine teaspoons of sugar (36 grams) - even more than a can of cola, which has about eight and a quarter teaspoons.

Public health bodies are starting to listen. The World Health Organization (WHO) now says that sugar restrictions must be considered in order to stem the global 'tidal wave' of cancer. WHO is thought to be on the brink of revising its guidelines on manufacturer-added sugar (including the sugar from honey, syrups and fruit juices), down from ten per cent to five per cent of our daily calorie intake - this means around six teaspoons a day for women.

We can only guess what John Yudkin, who died in 1995, would have made of the wide acceptance of his ideas. His book Pure, White and Deadly is back in print - this time with an introduction by Robert Lustig. 'I think he would have been pleased,' says his biochemist son, Professor Michael Yudkin. 'Not to say, "I told you so", but because my father's great passion was public health and he saw the world being harmed by something he thought was preventable.'

David Gillespie believes that we are on the brink of a public health revolution - similar to what's happened with tobacco. 'But this is not going to take anywhere near as long,' he predicts. 'The early anti-tobacco campaigners didn't have the internet and social media. A few powerful people had a stranglehold on the data but that isn't the case any more. In ten years' time, parents are going to be looking back and saying, "Can you believe that they used to feed fruit juice to children?"'

Pure, White and Deadly by John Yudkin is available for £8.99 (Penguin). To order a copy for £7.99 with free p&p, contact the YOU Bookshop on 0844 472 4157, you-bookshop.co.uk

FAT CHANCE by Dr Robert Lustig, published by 4th Estate £8.99

LOAD-DATE: March 3, 2014

LANGUAGE: ENGLISH

PUBLICATION-TYPE: Web Publication

JOURNAL-CODE: WEBDM

Copyright 2014 Associated Newspapers Ltd.
All Rights Reserved

THE SUNDAY TIMES
The Sunday Times (London)

March 2, 2014 Sunday
Edition 1;
Ireland

Hospitals told to operate healthy snack machines

SECTION: NEWS; Pg. 5

LENGTH: 335 words

THE HSE is to introduce a national policy on vending machines in all medical settings, writes Siobhán Maguire.

The policy will govern the supply, installation and stocking of vending machines in hospitals, nursing homes, clinics and medical centres.

All public health settings will have to follow strict guidelines in order to keep a vending machine on site.

Managers will have to ask an HSE health expert for permission to install a vending machine and comply with rules that ensure 60% of all products in the machine are healthy.

Machines will operate under three classifications: "healthy", "other", and "cannot be dispensed".

The "cannot be dispensed" category is understood to include sugary and **fizzy drinks** and highly calorific sweets and chocolate. The "other" category will permit snacks such as smaller, mini or "fun-size" chocolate bars and low-calorie or diet drinks.

There will be further restrictions around the salt, sugar and fat content of products and where they can be placed in a machine.

The rules have been drawn up by an HSE expert group on obesity called the vending expert group. Full details of the plan will be unveiled this week but it is understood that hospitals will have no wriggle room on how machines will be supplied, installed or stocked.

The announcement comes four weeks after The Sunday Times launched its Junk the Junk campaign, which aims to persuade the management of Ireland's post-primary schools to cut back on sugary foods and drinks stocked in vending machines.

In response Ruairi Quinn, the education minister, has revealed his own plans to encourage schools to have a 60% mix of healthy food options in vending machines.

The HSE group does not believe this is satisfactory and plans to meet the minister to discuss a new code of conduct for vending machines in secondary schools.

Last week Ireland endorsed the European Commission's Action Plan on Obesity. The plan, while voluntary, endeavours to introduce a range of healthy eating options for children and adults by 2020.

LOAD-DATE: March 2, 2014

LANGUAGE: ENGLISH

PUBLICATION-TYPE: Newspaper

JOURNAL-CODE: STS

Copyright 2014 Times Newspapers Limited
All Rights Reserved

The Telegraph
telegraph.co.uk

May 1, 2014 Thursday 5:36 PM GMT

Pepsi's challenge: get healthy and split business; Kevin Dreyer, portfolio manager at Gabelli Funds, discusses pressure felt by Pepsico to deliver healthier products and the case for separating the company's snack and beverage business

SECTION: FINANCE

LENGTH: 141 words

Sales of some low calorie **soft drinks** have nose-dived since their **sugar** content was cut, it was revealed yesterday/Monday.

Artificial sweetener stevia was added to the recipe for Sprite in March 2013 in a bid to turn round falling sales, but the move has backfired, according to industry experts who say it shows that despite health concerns over sugar levels in drinks, consumers still prefer them over low-calorie versions.

Five of the 10 best selling carbonates, including supermarket own-label brands, are now zero or low-sugar brands.

They have achieved a combined growth of 2.5 per cent in the past year, but the leading full-sugar brands, such as Coke, Pepsi, Fanta and Dr Pepper, were up 4.3 per cent.

Sales of full-sugar Coke are up £19 million, while demand for Pepsi is also up.

Video: Bloomberg

LOAD-DATE: May 2, 2014

LANGUAGE: ENGLISH

PUBLICATION-TYPE: Newspaper; Web Publication

JOURNAL-CODE: WEBDTNS

Copyright 2014 Telegraph Media Group Limited
All Rights Reserved

April 29, 2014 Tuesday 12:17 PM GMT

Never mind five a day - one in four don't eat ANY fruit or veg, and half can't be bothered to keep fit

BYLINE: JOHN HALL

SECTION: HEALTH

LENGTH: 458 words

- Many British adults choose to live on junk food instead of a healthy diet
- Quarter never eat fruit or vegetables, while 50 per cent do not exercise
- Despite eating badly, half of Britons are unhappy with their appearance
- Most blame a busy lifestyle and lack of money for their unhealthy diet

Half of British adults cannot be bothered to exercise, while one in four never eat fruit or vegetables, according to a shocking new study.

Instead of sticking to the so-called 'five-a-day' rule and ensuring they are physically fit, many British adults choose to live on a junk food and **sugary drink** diet while doing little to burn off the calories.

Despite a lackadaisical attitude towards their health, half of Britons say they are unhappy with their appearance, with most of those blaming a busy lifestyle and lack of money for their unhealthy diet.

The shocking figures were revealed in Aviva's Health Check UK report, which was released today.

The study found that one in three 25 to 34-year-olds were officially overweight, with that figure rising to 50 per cent of all 35 to 44-year-olds.

That figure improves slightly in the over-55 age range, where only a quarter are overweight.

Despite a rising level of concern surrounding obesity in Britain, a third of those who fall into the category insist they were happy with their weight and feel fine physically.

The lack of interest in taking care of ones health stems from people prioritising their work and social life over exercise and a good diet, according to Aviva UK Health medical director Dr Doug Wright.

'The nation is falling woefully short of hitting the five-a-day target of fruit and veg and too many people are failing to fit proper exercise into their lives,' he told the Mirror.

'It's about a subtle change in habits like swapping chocolate for an apple... Being overweight brings with it the potential for serious health conditions, yet people are living in denial by claiming they are in very good health,' he added.

'The risks are well known with conditions including type 2 diabetes, heart disease, stroke and even some types of cancer,' Dr Wright said.

Last week it was revealed that super-sized graves dedicated solely for obese people are set to be installed at a cemetery amid fears of a growing 'obesity crisis'.

Plans for 30 super-sized plots - which will be near the road so undertakers don't have far to carry the body - have been drawn up by council workers after larger graves were requested by funeral directors struggling to haul coffins over long distances in Sutton Bridge, Lincolnshire.

Sutton Bridge and Wingland Parish Council hopes to open a new three-acre burial ground next year because space is running out at the cemetery in the local church - where plots measure 9ft by 4ft.

LOAD-DATE: May 2, 2014

LANGUAGE: ENGLISH

PUBLICATION-TYPE: Web Publication

JOURNAL-CODE: WEBDM

Copyright 2014 Associated Newspapers Ltd.
All Rights Reserved

Metro (UK)

April 29, 2014 Tuesday
Edition 1;
National Edition

Low-cal drinks sales turn sour as sugar is cut

BYLINE: HAYDEN SMITH

SECTION: NEWS; Pg. 24

LENGTH: 201 words

SALES of some low-calorie **soft drinks** have nosedived since their **sugar** content was cut.

Sprite has suffered a 9.4 per cent fall in the past year since sugar levels were reduced by a third and artificial sweetener added.

Its maker Coca-Cola has been under pressure from British health lobbyists. Despite an advertising campaign, Sprite revenues fell to £55million in the past 12 months as sweet-toothed drinkers in Britain shunned it.

Artificial sweetener stevia was added to the recipe in March 2013 but the move has backfired.

Caroline Cater, of Coca-Cola Enterprises, told The Grocer magazine: 'It's a very competitive category.' The switch to stevia attracted new consumers who 'don't drink as heavily', she said.

Diet Coke has also suffered, with sales down 2.4 per cent to £465million, while Vimto sales are down more than five per cent to £21million.

Five of the ten best-selling carbonates, including supermarket own-label brands, are now zero or low-sugar products.

However, sales of full-sugar Coca-Cola are up £19million, while demand for normal Pepsi has also risen.

Last month, chief medical officer Dame Sally Davies called for an education programme on the risks relating to 'calorie-packed soft drinks'.

LOAD-DATE: April 29, 2014

LANGUAGE: ENGLISH

PUBLICATION-TYPE: Newspaper

JOURNAL-CODE: MTR

Copyright 2014 Associated Newspapers Ltd.
All Rights Reserved

49 of 143 DOCUMENTS

MailOnline

April 24, 2014 Thursday 9:09 PM GMT

Mexico food labeling rules draw fire on sugar

BYLINE: ASSOCIATED PRESS

SECTION: AP

LENGTH: 424 words

MEXICO CITY (AP) - Mexico's new food labeling rules were supposed to help fight an obesity epidemic, but activists and experts said Monday they may actually encourage the public to consume high levels of sugar.

The debate over sugar has grown bitter, in a country with one of the highest obesity rates in the Western Hemisphere.

The new label rules unveiled last week list the amount of sugar and other contents as a percent of recommended daily intakes. The new labels will no longer list the weights of the ingredients, instead simply listing them as calories and percentages of recommended daily intake.

But the labels assume that an average acceptable daily consumption of sugar is about 360 calories, equivalent to about 90 grams of sugar.

The World Health Organization has proposed a sugar intake of as little as 100 calories or about 25 grams per day.

Almost three dozen public health and nutrition experts published a full-page ad in Mexican newspapers Monday saying the new rules "increase the risk of obesity and diabetes."

It said the labeling system "is difficult to understand and represents a serious risk to the health of Mexicans," according to the ad.

The government health agency responsible for publishing the new rules last week did not immediately answer calls for comment.

"This is terrible, because some people are going to see this label... and they're going to say, 'well, I'll drink this Coca Cola, because it is 70 percent of my sugar requirement, and I can drink another 6 ½-ounce one, to get 100 percent of what they recommend I get of sugar,'" said Alejandro Calvillo, head of the Consumer Power activist group.

Calvillo said the decision to stop listing the weight of sugar in products was a mistake, in part because his group had some success in educating the public that 90 grams of sugar are equal to about 6 tablespoons - about two-thirds of a cup.

Mexico is among the fattest countries in the world. Just under one-third of adults are obese, according to the U.N. Food and Agriculture Organization. Seven out of 10 Mexicans are overweight and the country has surpassed the U.S. in obesity rates, according to a United Nations report, mostly due to a diet of fatty foods and sugary sodas.

Last year, Mexico's lawmakers approved a new tax on junk food as part of the government's campaign to fight obesity. The move came a little over a day after legislators agreed to tax soft drinks. Mexicans drink an average of 163 liters (43 gallons) of soft drinks annually, also among the highest soda consumption rates in the world.

LOAD-DATE: April 30, 2014

LANGUAGE: ENGLISH

PUBLICATION-TYPE: Web Publication

JOURNAL-CODE: WEBDM

Copyright 2014 Associated Newspapers Ltd.
All Rights Reserved

MailOnline

April 24, 2014 Thursday 9:09 PM GMT

Mexico food labeling rules draw fire on sugar

BYLINE: ASSOCIATED PRESS

SECTION: AP

LENGTH: 424 words

MEXICO CITY (AP) - Mexico's new food labeling rules were supposed to help fight an obesity epidemic, but activists and experts said Monday they may actually encourage the public to consume high levels of sugar.

The debate over sugar has grown bitter, in a country with one of the highest obesity rates in the Western Hemisphere.

The new label rules unveiled last week list the amount of sugar and other contents as a percent of recommended daily intakes. The new labels will no longer list the weights of the ingredients, instead simply listing them as calories and percentages of recommended daily intake.

But the labels assume that an average acceptable daily consumption of sugar is about 360 calories, equivalent to about 90 grams of sugar.

The World Health Organization has proposed a sugar intake of as little as 100 calories or about 25 grams per day.

Almost three dozen public health and nutrition experts published a full-page ad in Mexican newspapers Monday saying the new rules "increase the risk of obesity and diabetes."

It said the labeling system "is difficult to understand and represents a serious risk to the health of Mexicans," according to the ad.

The government health agency responsible for publishing the new rules last week did not immediately answer calls for comment.

"This is terrible, because some people are going to see this label... and they're going to say, 'well, I'll drink this Coca Cola, because it is 70 percent of my sugar requirement, and I can drink another 6 ½-ounce one, to get 100 percent of what they recommend I get of sugar,'" said Alejandro Calvillo, head of the Consumer Power activist group.

Calvillo said the decision to stop listing the weight of sugar in products was a mistake, in part because his group had some success in educating the public that 90 grams of sugar are equal to about 6 tablespoons - about two-thirds of a cup.

Mexico is among the fattest countries in the world. Just under one-third of adults are obese, according to the U.N. Food and Agriculture Organization. Seven out of 10 Mexicans are overweight and the country has surpassed the U.S. in obesity rates, according to a United Nations report, mostly due to a diet of fatty foods and sugary sodas.

Last year, Mexico's lawmakers approved a new tax on junk food as part of the government's campaign to fight obesity. The move came a little over a day after legislators agreed to tax soft drinks. Mexicans drink an average of 163 liters (43 gallons) of soft drinks annually, also among the highest soda consumption rates in the world.

LOAD-DATE: April 30, 2014

LANGUAGE: ENGLISH

PUBLICATION-TYPE: Web Publication

JOURNAL-CODE: WEBDM

Copyright 2014 Associated Newspapers Ltd.
All Rights Reserved

MailOnline

April 24, 2014 Thursday 9:09 PM GMT

Mexico food labeling rules draw fire on sugar

BYLINE: ASSOCIATED PRESS

SECTION: AP

LENGTH: 424 words

MEXICO CITY (AP) - Mexico's new food labeling rules were supposed to help fight an obesity epidemic, but activists and experts said Monday they may actually encourage the public to consume high levels of sugar.

The debate over sugar has grown bitter, in a country with one of the highest obesity rates in the Western Hemisphere.

The new label rules unveiled last week list the amount of sugar and other contents as a percent of recommended daily intakes. The new labels will no longer list the weights of the ingredients, instead simply listing them as calories and percentages of recommended daily intake.

But the labels assume that an average acceptable daily consumption of sugar is about 360 calories, equivalent to about 90 grams of sugar.

The World Health Organization has proposed a sugar intake of as little as 100 calories or about 25 grams per day.

Almost three dozen public health and nutrition experts published a full-page ad in Mexican newspapers Monday saying the new rules "increase the risk of obesity and diabetes."

It said the labeling system "is difficult to understand and represents a serious risk to the health of Mexicans," according to the ad.

The government health agency responsible for publishing the new rules last week did not immediately answer calls for comment.

"This is terrible, because some people are going to see this label... and they're going to say, 'well, I'll drink this Coca Cola, because it is 70 percent of my sugar requirement, and I can drink another 6 ½-ounce one, to get 100 percent of what they recommend I get of sugar,'" said Alejandro Calvillo, head of the Consumer Power activist group.

Calvillo said the decision to stop listing the weight of sugar in products was a mistake, in part because his group had some success in educating the public that 90 grams of sugar are equal to about 6 tablespoons - about two-thirds of a cup.

Mexico is among the fattest countries in the world. Just under one-third of adults are obese, according to the U.N. Food and Agriculture Organization. Seven out of 10 Mexicans are overweight and the country has surpassed the U.S. in obesity rates, according to a United Nations report, mostly due to a diet of fatty foods and sugary sodas.

Last year, Mexico's lawmakers approved a new tax on junk food as part of the government's campaign to fight obesity. The move came a little over a day after legislators agreed to tax soft drinks. Mexicans drink an average of 163 liters (43 gallons) of soft drinks annually, also among the highest soda consumption rates in the world.

LOAD-DATE: May 2, 2014

LANGUAGE: ENGLISH

PUBLICATION-TYPE: Web Publication

JOURNAL-CODE: WEBDM

Copyright 2014 Associated Newspapers Ltd.
All Rights Reserved

MailOnline

April 24, 2014 Thursday 6:18 PM GMT

Eating greens will 'not make children happy': Government funded survey finds fizzy drinks and sweets are 'better for the wellbeing' of seven-year-olds

BYLINE: AMANDA WILLIAMS

SECTION: NEWS

LENGTH: 807 words

- NHS report found children who eat sweets and watch TV in moderation are happier than those who are banned from the treats
- But it concluded that the consumption of fruit and vegetables has little effect on children's reported well-being

To the cheers of schoolchildren everywhere, a Government backed study has found that eating your greens is not as good for emotional 'wellbeing' as sweets.

The Department of Health funded report has found that children who eat sweets, drink **fizzy drinks** and watch television in moderation are actually enjoying a more rounded childhood than those who are banned from the treats.

It concluded that the consumption of fruit and vegetables has little effect on children's reported wellbeing.

In fact they found eating greens might even make children less happy in general.

The report stated: 'Being happier and lacking worry does not mean never having sweets, snacks and television.

'In fact, there was some indication in the results that higher well-being was more likely when these were enjoyed in moderation.

'For example, well-being was lower among children who never watched television and among those who watched it the most, higher among those who watched television, but for less than an hour a day.'

It went on to say: 'Eating fewer than the recommended five-a-day portions of fruit and vegetables was not significantly related to wellbeing.

'Likewise, those young people who had crisps, sweets or **fizzy drinks** on a daily or near-daily basis did not differ in their reported levels of wellbeing from those who had these types of snacks more rarely.'

The report was based on data gathered from almost 13,000 children, and was carried out by analysts at NatCen Social Research, on figures provided by the Millennium Cohort Study.

The study tracks the lives of a cohort of children born in 2000.

The report also found that children taking part in PE at school are likely to be more happy in general than those who don't.

A sense of wellbeing can also be handed down through parents. If couples are have a higher wellbeing - their children are likely to have higher wellbeing, the report stated.

Children who were allowed to watch five hours of television a day and children allowed to watch none at all, both scored lower for well-being than those who watched around an hour a day.

Scroll down for video

The report also noted that almost half of children who said they liked PE 'a lot in the questionnaire, also said that they were happy all the time'.

The report's lead author Jenny Chanfreau, senior researcher at NatCen, said: 'Our findings suggest that healthy eating does not enhance wellbeing during childhood.

'In fact, children who regularly ate snacks such as biscuits and cakes were as likely to report being happy as children who did not report eating such snacks.

'There are, however, some other strong arguments for promoting healthy behaviours among children.

'Being happier and lacking worry does not mean never having sweets, snacks and television. In fact, there was some indication in the results that higher well-being was more likely when these were enjoyed in moderation'

- NatCen Social Research report

'Not least that behaviours learnt early in life may continue into adulthood and, unlike among children, adults who eat more fruit and vegetables do have higher levels of wellbeing.'

A Department of Health spokesman said: 'Our advice continues to be that we should aim to reduce intake of foods that are high in fat, salt and sugar where possible, especially for children.

'It is important for children to have a healthy balanced diet, to get into healthy habits for life.

'Improving the nation's health is one of our top priorities, especially when it comes to diet and nutrition. Through initiatives such as our Change4Life and Healthy Start Programmes, we encourage people to adopt healthier habits for themselves and their families.'

Ms Chanfreau also today told the British Sociological Association's annual conference, that children living with a step-parent or a lone parent are as happy as those living with two biological parents.

She said: 'We found that the family type had no significant effect on the happiness of the seven-year-olds or the 11 to 15-year-olds.

'It's the quality of the relationships in the home that matters - not the family composition. Getting on well with siblings, having fun with the family at weekends, and having a parent who reported rarely or never shouting when the child was naughty, were all linked with a higher likelihood of being happy all the time among seven-year-olds.

'Pupil relations at school are also important - being bullied at school or being "horrible" to others was strongly associated with lower happiness in the seven-year-olds, for instance.'

LOAD-DATE: April 30, 2014

LANGUAGE: ENGLISH

PUBLICATION-TYPE: Web Publication

JOURNAL-CODE: WEBDM

Copyright 2014 Associated Newspapers Ltd.
All Rights Reserved

MailOnline

April 24, 2014 Thursday 6:18 PM GMT

Eating greens will 'not make children happy': Government funded survey finds fizzy drinks and sweets are 'better for the wellbeing' of seven-year-olds

BYLINE: AMANDA WILLIAMS

SECTION: NEWS

LENGTH: 807 words

- NHS report found children who eat sweets and watch TV in moderation are happier than those who are banned from the treats
- But it concluded that the consumption of fruit and vegetables has little effect on children's reported well-being

To the cheers of schoolchildren everywhere, a Government backed study has found that eating your greens is not as good for emotional 'wellbeing' as sweets.

The Department of Health funded report has found that children who eat sweets, drink **fizzy drinks** and watch television in moderation are actually enjoying a more rounded childhood than those who are banned from the treats.

It concluded that the consumption of fruit and vegetables has little effect on children's reported wellbeing.

In fact they found eating greens might even make children less happy in general.

The report stated: 'Being happier and lacking worry does not mean never having sweets, snacks and television.

'In fact, there was some indication in the results that higher well-being was more likely when these were enjoyed in moderation.

'For example, well-being was lower among children who never watched television and among those who watched it the most, higher among those who watched television, but for less than an hour a day.'

It went on to say: 'Eating fewer than the recommended five-a-day portions of fruit and vegetables was not significantly related to wellbeing.

'Likewise, those young people who had crisps, sweets or **fizzy drinks** on a daily or near-daily basis did not differ in their reported levels of wellbeing from those who had these types of snacks more rarely.'

The report was based on data gathered from almost 13,000 children, and was carried out by analysts at NatCen Social Research, on figures provided by the Millennium Cohort Study.

The study tracks the lives of a cohort of children born in 2000.

The report also found that children taking part in PE at school are likely to be more happy in general than those who don't.

A sense of wellbeing can also be handed down through parents. If couples are have a higher wellbeing - their children are likely to have higher wellbeing, the report stated.

Children who were allowed to watch five hours of television a day and children allowed to watch none at all, both scored lower for well-being than those who watched around an hour a day.

Scroll down for video

The report also noted that almost half of children who said they liked PE 'a lot in the questionnaire, also said that they were happy all the time'.

The report's lead author Jenny Chanfreau, senior researcher at NatCen, said: 'Our findings suggest that healthy eating does not enhance wellbeing during childhood.

'In fact, children who regularly ate snacks such as biscuits and cakes were as likely to report being happy as children who did not report eating such snacks.

'There are, however, some other strong arguments for promoting healthy behaviours among children.

'Being happier and lacking worry does not mean never having sweets, snacks and television. In fact, there was some indication in the results that higher well-being was more likely when these were enjoyed in moderation'

- NatCen Social Research report

'Not least that behaviours learnt early in life may continue into adulthood and, unlike among children, adults who eat more fruit and vegetables do have higher levels of wellbeing.'

A Department of Health spokesman said: 'Our advice continues to be that we should aim to reduce intake of foods that are high in fat, salt and sugar where possible, especially for children.

'It is important for children to have a healthy balanced diet, to get into healthy habits for life.

'Improving the nation's health is one of our top priorities, especially when it comes to diet and nutrition. Through initiatives such as our Change4Life and Healthy Start Programmes, we encourage people to adopt healthier habits for themselves and their families.'

Ms Chanfreau also today told the British Sociological Association's annual conference, that children living with a step-parent or a lone parent are as happy as those living with two biological parents.

She said: 'We found that the family type had no significant effect on the happiness of the seven-year-olds or the 11 to 15-year-olds.

'It's the quality of the relationships in the home that matters - not the family composition. Getting on well with siblings, having fun with the family at weekends, and having a parent who reported rarely or never shouting when the child was naughty, were all linked with a higher likelihood of being happy all the time among seven-year-olds.

'Pupil relations at school are also important - being bullied at school or being "horrible" to others was strongly associated with lower happiness in the seven-year-olds, for instance.'

LOAD-DATE: April 30, 2014

LANGUAGE: ENGLISH

PUBLICATION-TYPE: Web Publication

JOURNAL-CODE: WEBDM

Copyright 2014 Associated Newspapers Ltd.
All Rights Reserved

MailOnline

April 24, 2014 Thursday 9:09 PM GMT

Mexico food labeling rules draw fire on sugar

BYLINE: ASSOCIATED PRESS

SECTION: AP

LENGTH: 424 words

MEXICO CITY (AP) - Mexico's new food labeling rules were supposed to help fight an obesity epidemic, but activists and experts said Monday they may actually encourage the public to consume high levels of sugar.

The debate over sugar has grown bitter, in a country with one of the highest obesity rates in the Western Hemisphere.

The new label rules unveiled last week list the amount of sugar and other contents as a percent of recommended daily intakes. The new labels will no longer list the weights of the ingredients, instead simply listing them as calories and percentages of recommended daily intake.

But the labels assume that an average acceptable daily consumption of sugar is about 360 calories, equivalent to about 90 grams of sugar.

The World Health Organization has proposed a sugar intake of as little as 100 calories or about 25 grams per day.

Almost three dozen public health and nutrition experts published a full-page ad in Mexican newspapers Monday saying the new rules "increase the risk of obesity and diabetes."

It said the labeling system "is difficult to understand and represents a serious risk to the health of Mexicans," according to the ad.

The government health agency responsible for publishing the new rules last week did not immediately answer calls for comment.

"This is terrible, because some people are going to see this label... and they're going to say, 'well, I'll drink this Coca Cola, because it is 70 percent of my sugar requirement, and I can drink another 6 ½-ounce one, to get 100 percent of what they recommend I get of sugar,'" said Alejandro Calvillo, head of the Consumer Power activist group.

Calvillo said the decision to stop listing the weight of sugar in products was a mistake, in part because his group had some success in educating the public that 90 grams of sugar are equal to about 6 tablespoons - about two-thirds of a cup.

Mexico is among the fattest countries in the world. Just under one-third of adults are obese, according to the U.N. Food and Agriculture Organization. Seven out of 10 Mexicans are overweight and the country has surpassed the U.S. in obesity rates, according to a United Nations report, mostly due to a diet of fatty foods and sugary sodas.

Last year, Mexico's lawmakers approved a new tax on junk food as part of the government's campaign to fight obesity. The move came a little over a day after legislators agreed to tax soft drinks. Mexicans drink an average of 163 liters (43 gallons) of soft drinks annually, also among the highest soda consumption rates in the world.

LOAD-DATE: April 30, 2014

LANGUAGE: ENGLISH

PUBLICATION-TYPE: Web Publication

JOURNAL-CODE: WEBDM

Copyright 2014 Associated Newspapers Ltd.
All Rights Reserved

MailOnline

MailOnline

April 24, 2014 Thursday 6:18 PM GMT

Eating greens will 'not make children happy': Government funded survey finds fizzy drinks and sweets are 'better for the wellbeing' of seven-year-olds

BYLINE: AMANDA WILLIAMS

SECTION: NEWS

LENGTH: 807 words

- NHS report found children who eat sweets and watch TV in moderation are happier than those who are banned from the treats
- But it concluded that the consumption of fruit and vegetables has little effect on children's reported well-being

To the cheers of schoolchildren everywhere, a Government backed study has found that eating your greens is not as good for emotional 'wellbeing' as sweets.

The Department of Health funded report has found that children who eat sweets, drink **fizzy drinks** and watch television in moderation are actually enjoying a more rounded childhood than those who are banned from the treats.

It concluded that the consumption of fruit and vegetables has little effect on children's reported wellbeing.

In fact they found eating greens might even make children less happy in general.

The report stated: 'Being happier and lacking worry does not mean never having sweets, snacks and television.

'In fact, there was some indication in the results that higher well-being was more likely when these were enjoyed in moderation.

'For example, well-being was lower among children who never watched television and among those who watched it the most, higher among those who watched television, but for less than an hour a day.'

It went on to say: 'Eating fewer than the recommended five-a-day portions of fruit and vegetables was not significantly related to wellbeing.

'Likewise, those young people who had crisps, sweets or **fizzy drinks** on a daily or near-daily basis did not differ in their reported levels of wellbeing from those who had these types of snacks more rarely.'

The report was based on data gathered from almost 13,000 children, and was carried out by analysts at NatCen Social Research, on figures provided by the Millennium Cohort Study.

The study tracks the lives of a cohort of children born in 2000.

The report also found that children taking part in PE at school are likely to be more happy in general than those who don't.

A sense of wellbeing can also be handed down through parents. If couples are have a higher wellbeing - their children are likely to have higher wellbeing, the report stated.

Children who were allowed to watch five hours of television a day and children allowed to watch none at all, both scored lower for well-being than those who watched around an hour a day.

Scroll down for video

The report also noted that almost half of children who said they liked PE 'a lot in the questionnaire, also said that they were happy all the time'.

The report's lead author Jenny Chanfreau, senior researcher at NatCen, said: 'Our findings suggest that healthy eating does not enhance wellbeing during childhood.

'In fact, children who regularly ate snacks such as biscuits and cakes were as likely to report being happy as children who did not report eating such snacks.

'There are, however, some other strong arguments for promoting healthy behaviours among children.

'Being happier and lacking worry does not mean never having sweets, snacks and television. In fact, there was some indication in the results that higher well-being was more likely when these were enjoyed in moderation'

- NatCen Social Research report

'Not least that behaviours learnt early in life may continue into adulthood and, unlike among children, adults who eat more fruit and vegetables do have higher levels of wellbeing.'

A Department of Health spokesman said: 'Our advice continues to be that we should aim to reduce intake of foods that are high in fat, salt and sugar where possible, especially for children.

'It is important for children to have a healthy balanced diet, to get into healthy habits for life.

'Improving the nation's health is one of our top priorities, especially when it comes to diet and nutrition. Through initiatives such as our Change4Life and Healthy Start Programmes, we encourage people to adopt healthier habits for themselves and their families.'

Ms Chanfreau also today told the British Sociological Association's annual conference, that children living with a step-parent or a lone parent are as happy as those living with two biological parents.

She said: 'We found that the family type had no significant effect on the happiness of the seven-year-olds or the 11 to 15-year-olds.

'It's the quality of the relationships in the home that matters - not the family composition. Getting on well with siblings, having fun with the family at weekends, and having a parent who reported rarely or never shouting when the child was naughty, were all linked with a higher likelihood of being happy all the time among seven-year-olds.

'Pupil relations at school are also important - being bullied at school or being "horrible" to others was strongly associated with lower happiness in the seven-year-olds, for instance.'

LOAD-DATE: April 30, 2014

LANGUAGE: ENGLISH

PUBLICATION-TYPE: Web Publication

JOURNAL-CODE: WEBDM

Copyright 2014 Associated Newspapers Ltd.
All Rights Reserved

MailOnline

April 24, 2014 Thursday 11:28 AM GMT

Sugary drinks increase the risk of high blood pressure by 70% - even in teenagers

BYLINE: EMMA INNES

SECTION: HEALTH

LENGTH: 380 words

- **Sugary drinks** can even cause high blood pressure in teenagers
- They could cause high blood pressure by lowering the body's levels of nitric oxide which causes the blood vessels to constrict

Everyone knows that drinking **sugary drinks**, such as **fizzy drinks** and some fruit juices, can cause them to pile on the pounds.

But new research suggests they can also lead to an increase in blood pressure.

A review published in The American Journal of Cardiology revealed there is a strong correlation between drinking sugar-sweetened drinks and elevated blood pressure.

Dr Aaqib Habib Malik, from the Griffin Hospital in Derby, Connecticut, and his colleagues reviewed 12 studies to establish if there is a link between sweet drinks and high blood pressure, Health Day reports.

The studies they looked at included almost 410,000 people.

They discovered that all of the studies showed a correlation between consuming sugar-sweetened drinks and high blood pressure.

The studies revealed that people who drink **sugary drinks** are between 26 and 70 per cent more likely to develop high blood pressure than people who do not drink them, Daily RX reports.

They also showed that teenagers who drink three or more sugar-sweetened drinks a day are 87 per cent more likely to develop high blood pressure.

The researchers made a number of suggestions as to how the drinks might increase blood pressure.

They say they can lower the levels of nitric oxide in the body which causes the blood vessels to constrict and, therefore, the blood pressure to increase.

Alternatively, they suggest that the increase in blood pressure could be a response to the salt in some of these drinks.

Medical Xpress reports that the authors wrote: 'In conclusion, our systematic review shows that the consumption of [**sugar**-sweetened **beverages**] is associated with higher [blood pressure], leading to increased incidence of hypertension.

'Restriction on [**sugar**-sweetened **beverage**] consumption should be incorporated in the recommendations of lifestyle modifications for the treatment of hypertension.

'Interventions to reduce intake of [**sugar**-sweetened **beverage**] should be an integral part of public health strategy to reduce the incidence of hypertension.'

Sugary drinks have already been associated with diabetes, obesity and heart disease.

LOAD-DATE: April 30, 2014

LANGUAGE: ENGLISH

PUBLICATION-TYPE: Web Publication

JOURNAL-CODE: WEBDM

Copyright 2014 Associated Newspapers Ltd.
All Rights Reserved

The Telegraph
telegraph.co.uk

April 24, 2014 Thursday 6:01 AM GMT

Eating your greens will not make you happy, finds Government-backed study;
Children allowed to eat sweets and watch television in moderation score higher for "well-being" than those not allowed to at all, study funded by Department of Health concludes

BYLINE: By John Bingham Social Affairs Editor

SECTION: NEWS

LENGTH: 622 words

It is something generations of reluctant schoolboy have long suspected - eating your greens might be good for you but it does not make you happy.

A major Government-funded study of the factors which improve children's happiness and reduce worry found that, although some healthy activities such as sport are linked to higher levels of general well-being, eating the recommended portions of fruit and vegetables is not.

The report, based on data gathered from almost 13,000 children, also found evidence that those who enjoy "unhealthy" things such as sweets, **fizzy drinks** or television, in moderation are enjoying a happier and more rounded childhood than those who are not allowed them at all.

The study, carried out by analysts at NatCen Social Research and funded by the Department of Health, concluded that deprivation, family discord and problems at school or with friends can all have a negative impact on the general well-being of seven year-olds.

The report, being presented at the conference of the British Sociological Association, shows that those who enjoy PE at school are markedly more likely to report being happy in general and children whose parents enjoy good health also report higher levels of well-being in their own daily lives.

But when it comes to eating, the findings are less likely to please health chiefs.

The findings suggest that eating fruit and vegetables has little effect on children's reported well-being - a measure which incorporates happiness and factors such as worry - possibly even making them slightly less happy overall.

Meanwhile those allowed to eat sweets and sugary snacks in moderation also emerged as having higher levels of well-being than those for whom they are forbidden.

While children left to watch more than five hours a day of television scored lower for well-being than those who watched around an hour a day, so too did those who did not watch any.

The report, based on data provided by the Millennium Cohort Study, which tracks the lives of thousands of children born in 2000, highlighted how 40 per cent of those who said they liked PE "a lot", separately said that they were happy "all the time".

Strikingly, less than a quarter of those who said they did not enjoy PE spoke of being always happy - suggesting links between health and happiness.

But when the children's assessment of their happiness was compared with their diets a more complicated picture emerged.

There was no significant difference in well-being between those allowed crisps, cakes, biscuits, sweets, chocolate or **sugary drinks** between meals and those who were not.

Meanwhile 38 per cent of those who said they were happy "all the time" ate no fruit and vegetables. Only 36 per cent of the happiest children had one or two portions and only 35 per cent had three or more.

Jenny Chanfreau, the lead author, said the difference was not statistically significant but showed that it cannot be claimed that eating greens will make children happier overall.

"This is not to say that eating healthily isn't good for you," she said.

"It is just not linked with happiness as reported by seven year-olds."

She said one of the most striking findings was that moderation with unhealthy activities rather than complete prohibition was linked with higher general well-being.

"Being happier and lacking worry does not mean never having sweets, snacks and television," the report concludes.

"In fact, there was some indication in the results that higher well-being was more likely when these were enjoyed in moderation.

"For example, well-being was lower among children who never watched television and among those who watched it the most, higher among those who watched television, but for less than an hour a day."

LOAD-DATE: April 24, 2014

LANGUAGE: ENGLISH

PUBLICATION-TYPE: Newspaper; Web Publication

JOURNAL-CODE: WEBDTNS

Copyright 2014 Telegraph Media Group Limited

All Rights Reserved

MailOnline

MailOnline

April 22, 2014 Tuesday 11:50 PM GMT

Advocates vow to revive Navajo junk-food tax

BYLINE: ASSOCIATED PRESS

SECTION: AP

LENGTH: 837 words

FLAGSTAFF, Ariz. (AP) - Facing a high prevalence of diabetes, many American Indian tribes are returning to their roots with community and home gardens, cooking classes that incorporate traditional foods, and running programs to encourage healthy lifestyles.

The latest effort on the Navajo Nation, the country's largest reservation, is to use the tax system to spur people to ditch junk food.

A proposed 2 percent sales tax on chips, cookies and sodas failed Tuesday in a Tribal Council vote. But the measure still has widespread support, and advocates plan to revive it, with the hope of making the tribe one of the first governments to enact a junk-food tax.

Elected officials across the U.S. have taken aim at **sugary drinks** with proposed bans, size limits, tax hikes and warning labels, though their efforts have not gained widespread traction. In Mexico, lawmakers approved a junk food tax and a tax on **soft drinks** last year as part of that government's campaign to fight obesity.

Navajo President Ben Shelly earlier this year vetoed measures to establish a junk-food tax and eliminate the tax on fresh fruit and vegetables. At Tuesday's meeting, tribal lawmakers overturned the veto on the tax cut, but a vote to secure the junk-food tax fell short. Lawmakers voted 13-7 in favor it, but the tax needed 16 votes to pass.

The Dine Community Advocacy Alliance, which led the effort, said it plans to revise the proposal and bring it before lawmakers again during the summer legislative session.

"We're going to keep moving on it," group member Gloria Begay said. "It's not so much the tax money - it's the message. The message being, 'Let's look at our health and make healthier choices.' We have to go out and do more education awareness."

Shelly said he supports the proposal's intent but questioned how the higher tax on snacks high in fat, sugar and salt would be enacted and regulated. Supporters say the tax is another tool in their fight for the health of the people.

"If we can encourage our people to make healthier choices and work on the prevention side, we increase the life span of our children, we improve their quality of life," said professional golfer Notah Begay III, who is among supporters.

American Indians and Alaska Natives as a whole have the highest age-adjusted prevalence of diabetes among U.S. racial and ethnic groups, according to the American Diabetes Association. They are more than twice as likely as non-Hispanic whites to have the disease that was the fourth leading cause of death in the Navajo area from 2003 to 2005, according to the Indian Health Service.

Native children ages 10 to 19 are nine times as likely to be diagnosed with Type 2 diabetes, the IHS said.

The proposed Navajo Nation tax wouldn't have added significantly to the price of junk food, but buying food on the reservation presents obstacles that don't exist in most of urban America. The reservation is a vast 27,000 square miles with few grocery stores and a population with an unemployment rate of around 50 percent. Thousands of people live without electricity and have no way of storing perishable food items for too long.

"They have a tendency to purchase what's available, and it's not always the best food," said Leslie Wheelock, director of tribal relations for the U.S. Department of Agriculture.

Wheelock said the diabetes issue in tribal communities is one that has been overlooked in the past or not taken as seriously as it could be. It has roots in the federal government taking over American Indian lands and introducing food that tribal members weren't used to, she said.

To help remedy that, the USDA runs a program that distributes nutritional food to 276 tribes. Grants from the agency have gone toward gardening lessons for children within the Seneca Nation of Indians in New York, culturally relevant exercise programs for the Spirit Lake Tribe in North Dakota and food demonstrations using fresh fruit and vegetables on the Zuni reservations in New Mexico.

The Dine Community Advocacy Alliance estimated a junk-food tax would result in at least $1 million a year in revenue that could go toward wellness centers, community parks, walking trails and picnic grounds in tribal communities in Utah, New Mexico and Arizona. It would have expired at the end of 2018.

No other sales tax on the Navajo Nation specifically targets the spending habits of consumers. Alcohol is sold in a few places on the reservation but isn't taxed. Retailers and distributors pay a tobacco tax.

Opponents of the junk food tax argued it would burden customers and drive revenue off the reservation. Mike Gardner, executive director of the Arizona Beverage Association, said the lack of specifics in the legislation as to what exactly would be taxed could mean fruit juice and nutritional shakes would be lumped in the same category as sodas.

"I don't think they mean that, but that's what will happen," Gardner said. "It's a little loose, a little vague. It's going to create problems for retailers and... it doesn't solve the problem."

LOAD-DATE: May 2, 2014

LANGUAGE: ENGLISH

PUBLICATION-TYPE: Web Publication

JOURNAL-CODE: WEBDM

Copyright 2014 Associated Newspapers Ltd.
All Rights Reserved

DAILY MAIL (London)

April 19, 2014 Saturday

SWEETS AT THE CHECKOUT WILL STAY AS STORES REBEL OVER BAN

BYLINE: BY SEAN POULTER CONSUMER AFFAIRS EDITOR

LENGTH: 586 words

PLANS to clamp down on the promotion of junk food in supermarkets have been scrapped after major stores refused to sign up.

Under the scheme, so-called guilt lanes' - where sweets and chocolates are prominently displayed next to checkouts - would have been banned.

But the retailers have effectively killed off the Government's Responsibility Deal' to tackle rising obesity levels by refusing to sign up.

Health campaigners last night accused the supermarkets of putting profit before their customers' health, and said ministers should introduce laws on junk food rather than relying on voluntary schemes.

As well as action on guilt lanes, under the plan there would have been a ban on promoting **sugary drinks** and snacks in high-profile positions on the end of shelves after a Government-backed study found this led to surge in sales.

The supermarkets have also rejected the idea of setting up a scheme that would have given customers rewards for buying healthy food such as fruit and vegetables.

The collapse of the initiatives is a huge blow to the Government's efforts to tackle rising obesity and associated ill-health, which is costing the NHS £5billion a year to treat.

The Government's chief medical officer, Dame Sally Davies, is so concerned about unhealthy food that she has suggested it may be necessary to impose sugar tax to curb consumption.

And it will raise further questions over the influence that supermarkets and other food giants are able to exert on government policy.

The Daily Mail revealed in February how the food industry lobby had been given unprecedented access to the heart of Government.

Supermarkets, fast food chains and chocolate and **fizzy drinks** firms have had dozens of meetings with ministers since the 2010 election, while health campaigners had been kept at arms length.

Tam Fry, spokesman for the National Obesity Forum and chairman of the Child Growth Foundation, said the collapse of the scheme was evidence that voluntary deals with the retail industry would not work.

He said: Pester power at the checkouts, where children demand sweets and chocolates, is enormously powerful.

The fact that the supermarkets refuse to tackle this is a disaster.

The government has been told by many people that voluntary deals do not work and here is the proof. The reality is that money comes first and health second.'

She added: It is time for the Government to take a firm line. Obesity is a totally preventable problem. If the supermarkets lose a little money for public good that is something they should be able to shoulder.'

It was hoped the public health Responsibility Deal would have seen all the major supermarkets promise to voluntarily ban junk food - high in fat, salt and sugar - from guilt lanes' and prominent positions at the end of aisles.

Retailers would also have agreed that more than 30 per cent of food promotions, such as buy-one-get-one-free, would be skewed towards healthy products such as fruit and vegetables.

There would also have been a ban on the use of popular children's characters, such as movie superheroes or pop stars, from being used to promote unhealthy food and **fizzy drinks**.

However, trade magazine The Grocer said the supermarkets had refused to sign up to the wide-ranging measures as they had failed to reach a consensus and claimed a voluntary pledge would not work on such a commercially sensitive issue'.

The Department of Health has conceded that it will have to leave it up to individual retailers as to whether they want to stop promoting junk food.

© Daily Mail

LOAD-DATE: April 18, 2014

LANGUAGE: ENGLISH

PUBLICATION-TYPE: Papers

Copyright 2014 Associated Newspapers Ltd.
All Rights Reserved

MailOnline

April 19, 2014 Saturday 1:29 AM GMT

Sweets at the checkout will stay as stores rebel over ban: Supermarkets resist call from health campaigners to remove temptation from the tills

BYLINE: SEAN POULTER, CONSUMER AFFAIRS EDITOR

SECTION: NEWS

LENGTH: 615 words

- Under scheme 'guilt lanes' would have been banned
- Retailers have killed Government's 'Responsibility Deal'
- Aimed to tackle rising obesity levels by banning sweets from checkouts

Plans to clamp down on the promotion of junk food in supermarkets have been scrapped after major stores refused to sign up.

Under the scheme, so-called 'guilt lanes' - where sweets and chocolates are prominently displayed next to checkouts - would have been banned.

But the retailers have effectively killed off the Government's 'Responsibility Deal' to tackle rising obesity levels by refusing to sign up.

Health campaigners last night accused the supermarkets of putting profit before their customers' health, and said ministers should introduce laws on junk food rather than relying on voluntary schemes.

As well as action on guilt lanes, under the plan there would have been a ban on promoting **sugary drinks** and snacks in high-profile positions on the end of shelves after a Government-backed study found this led to a surge in sales.

The supermarkets have also rejected the idea of setting up a scheme that would have given customers rewards for buying healthy food such as fruit and vegetables.

The collapse of the initiatives is a huge blow to the Government's efforts to tackle rising obesity and associated ill-health, which is costing the NHS £5billion a year to treat.

The Government's chief medical officer, Dame Sally Davies, is so concerned about unhealthy food that she has suggested it may be necessary to impose sugar tax to curb consumption.

And it will raise further questions over the influence that supermarkets and other food giants are able to exert on government policy.

The Daily Mail revealed in February how the food industry lobby had been given unprecedented access to the heart of Government.

Supermarkets, fast food chains and chocolate and **fizzy drinks** firms have had dozens of meetings with ministers since the 2010 election, while health campaigners had been kept at arms length.

Tam Fry, spokesman for the National Obesity Forum and chairman of the Child Growth Foundation, said the collapse of the scheme was evidence that voluntary deals with the retail industry would not work.

He said: 'Pester power at the checkouts, where children demand sweets and chocolates, is enormously powerful.

'The fact that the supermarkets refuse to tackle this is a disaster.

'The Government has been told by many people that voluntary deals do not work and here is the proof.

'The reality is that money comes first and health second.'

She added: 'It is time for the Government to take a firm line. Obesity is a totally preventable problem.

'If the supermarkets lose a little money for public good that is something they should be able to shoulder.'

It was hoped the public health Responsibility Deal would have seen all the major supermarkets promise to voluntarily ban junk food - high in fat, salt and sugar - from 'guilt lanes' and prominent positions at the end of aisles.

Retailers would also have agreed that more than 30 per cent of food promotions, such as buy-one-get-one-free, would be skewed towards healthy products such as fruit and vegetables.

There would also have been a ban on the use of popular children's characters, such as movie superheroes or pop stars, to promote unhealthy food and **fizzy drinks**.

However, trade magazine The Grocer said the supermarkets had refused to sign up to the wide-ranging measures as they 'had failed to reach a consensus and claimed a voluntary pledge would not work on such a commercially sensitive issue'.

The Department of Health has conceded that it will have to leave it up to individual retailers as to whether they want to stop promoting junk food.

LOAD-DATE: April 21, 2014

LANGUAGE: ENGLISH

PUBLICATION-TYPE: Web Publication

JOURNAL-CODE: WEBDM

Copyright 2014 Associated Newspapers Ltd.
All Rights Reserved

80 of 143 DOCUMENTS

THE TIMES
The Times (London)

April 18, 2014 Friday
Edition 1;
National Edition

PepsiCo's added bite;
IN BUSINESS

SECTION: BUSINESS; Pg. 49

LENGTH: 48 words

PepsiCo reported a strongerthan-expected first-quarter profit, thanks to strong snack sales and price rises. As it struggles with falls in its **fizzy drink** sales, it reported global snack volume rose 2 per cent. Net income was $1.22 billion, up from $1.08 billion in the same period last year.

LOAD-DATE: April 18, 2014

LANGUAGE: ENGLISH

PUBLICATION-TYPE: Newspaper

JOURNAL-CODE: TIM

Copyright 2014 Times Newspapers Limited
All Rights Reserved

83 of 143 DOCUMENTS

The Daily Telegraph
The Daily Telegraph (London)

April 16, 2014 Wednesday
Edition 2;
National Edition

Coke rediscovers its pop thanks to Russian Games

BYLINE: Katherine Rushton

SECTION: BUSINESS; Pg. 3

LENGTH: 299 words

COCA-COLA reassured investors it is getting its fizz back as the company steadied its decline and beat analysts' forecasts with its first-quarter results. Profits at the drinks giant fell 8pc to $1.62bn, while revenues slipped 4pc to $10.58bn.

However, the slowdown was not as bad as feared, after a surge in sales in China and Russia.

The company does not break out revenues for those countries, but said that the volume of drinks sold in China rose by 12pc, helped by heavy advertising around Chinese New Year. A marketing blitz around the Sochi Winter Olympics also helped fuel a 9pc increase in volume sales in Russia.

Its performance in developing markets helped to offset a 4pc drop in Europe, where customers are losing their appetite for high-sugar, **fizzy drinks**. Sales in America, which have been in decline for the last few quarters, started to plateau.

"We're beginning to get our momentum back here the beginning of 2014," Muhtar Kent, chairman and chief executive of the company told investors. "All of us at The Coca-Cola Company remain confident in our ability to deliver on our strategies while further strengthening our foundation for profitable and sustainable long-term growth."

Coca-Cola and other drinks manufacturers have suffered in developed markets, as more consumers swap carbonated drinks for healthier options. The drinks industry has spent the past decade trying to offset that trend by introducing more low and zero-calorie versions of its beverages, but many consumers are now trying to cut back on those as well.

However, Coca-Cola said falling US sales had steadied, as the company took more market shares from competitors. "Our North America Group delivered even volume versus the prior year quarter while gaining value share and maintaining volume share," it said.

LOAD-DATE: April 16, 2014

LANGUAGE: ENGLISH

GRAPHIC: The soft drinks manufacturer has suffered as consumers switch to healthier options
CRAIG LOVELL / EAGLE VISIONS PHO

PUBLICATION-TYPE: Newspaper

JOURNAL-CODE: DTL

Copyright 2014 Telegraph Media Group Limited
All Rights Reserved

Financial Times (London, England)

April 16, 2014 Wednesday
London Edition 3

Losing sparkle: Coca-Cola hits near 15-year low for global sales of fizzy drinks

BYLINE: Shannon Bond in New York

SECTION: COMPANIES; Pg. 19

LENGTH: 179 words

Coca-Cola sold fewer **fizzy drinks** around the world in the first quarter for the first time in nearly 15 years and quarterly profit fell 7.5 per cent from a year ago, **writesShannon Bond** .

But the world's largest drinks company still sold more beverages in the first three months of the year as higher sales in China, Russia, India and Latin America helped offset the effects of US and European consumers turning away from carbonated drinks.

First-quarter net income fell to $1.62bn, or 36 cents a share, from $1.75bn, or 39 cents a share, a year ago. Revenue declined 4 per cent to $10.58bn, in line with forecasts.

The company said results were hit by the sale of its bottling operations in Brazil and the Philippines last year, the stronger dollar and the devaluation of Venezuela's currency.

Worldwide, sales of still drinks, such as Minute Maid juice, Dasani water and Powerade sports beverages, were up 8 per cent, but **fizzy drinks** volumes fell 1 per cent. That was the first decline since the second quarter of 1999.

Coca-Cola's shares closed yesterday up 3.7 per cent at $40.18.

LOAD-DATE: April 15, 2014

LANGUAGE: ENGLISH

PUBLICATION-TYPE: Newspaper

Copyright 2014 The Financial Times Ltd.
All Rights Reserved
Please do not cut and paste FT articles and redistribute by email or post to the web.

The Independent (London)

April 16, 2014 Wednesday
First Edition

Coke loses its fizz in the UK as bottles get smaller; NEWS IN BRIEF DRINKS

SECTION: BUSINESS; Pg. 51

LENGTH: 56 words

Coca-Cola's global sales of fizzy pop fell for the first time in 15 years partly due to a fall of more than 10 per cent in sales in the UK, where it froze prices but reduced the size of bottles. The UK decline was higher than the 5 per cent European average fall over the last quarter. Better sales of non-**fizzy drinks** outweighed the fall.

LOAD-DATE: April 15, 2014

LANGUAGE: ENGLISH

PUBLICATION-TYPE: Newspaper

JOURNAL-CODE: IA

Copyright 2014 Independent Print Ltd
All Rights Reserved

mirror.co.uk

April 15, 2014 Tuesday 6:53 PM GMT

10 retro food rules of the Mad Men diet; Just as Mad Men returns to our TVs, research shows that retro eating habits could keep us slim and healthy...

BYLINE: By Caroline Jones

SECTION: LIFESTYLE,DIETING

LENGTH: 1257 words

Everything we thought we knew about the dangers of eating too much fat has been turned on its head.

In a U-turn on advice we've been given for the last 40 years, a review of 72,000 studies found there was no evidence that saturated fats - found in butter, meat and full-fat milk - caused heart disease.

And Government diet adviser Dr Susan Jebb has backed plans to stop fruit juice - previously seen as a super-healthy drink - being counted as one of your five a day because it's so packed with sugar.

In fact sugar, we're now told, is the real baddie, not fat after all. Confused?

It seems much of what we've thought of as healthy for nearly half a century probably isn't so great and many of the unfashionable foods our grandparents tucked into could actually be good for us and our waistlines!

Nutritionist Linda Foster says: "A diet based on relatively old-fashioned foods, such as eggs for breakfast and plenty of home-cooked meals, including those containing some lean red meat and butter, can be pretty healthy and slimming, especially if you eat good amounts of seasonal fruit and veg as we would have done in the past."

Cooking your own meals as families used to do, rather than relying on processed convenience foods, is the real key, according to award-winning food writer Michael Pollan. His latest book - Cooked - blames our expanding girths on the fact we no longer cook real food. He points out the decline in home cooking "closely parallels the rise in obesity".

So perhaps we need to go back 50 years - when TV's Mad Men is set - or even 100 years to when being overweight was rare for some no-nonsense tips.

One 1914 poster from the American health board sums up advice that still holds true today: "Food: Buy it with thought. Cook it with care. Use less wheat and meat. Buy local foods. Serve just enough. Use what is left. Don't waste it."

Here's our guide, based on the good food sense of our grandmothers...1. Butter your bread and drink milk

Your gran probably guzzled a pint of full-fat milk a day and spread butter thickly on her bread.

But from the mid-1970s we were told it was clogging our arteries and we should switch to margarines, sunflower oils and skimmed milk.

Experts now say saturated fat may not be as bad as we thought, while other studies link some man-made spreads to health concerns of their own.

"So you're probably safer with good old-fashioned butter in moderation," advises Linda.

Evidence also suggests whole milk could be better than skimmed as it contains only around 4% fat and is more nutritious because of vitamins A, D, E and K in the cream.2. Tuck into (lean) red meat twice a week

In the past 40 years many of us have eaten less red meat because of worries that it's fattening or unhealthy. But it seems grandma knew best when she insisted the family eat liver once a week and hearty roast beef on Sundays.

Latest research suggests eating lean beef, liver, lamb or pork twice a week (as opposed to processed meats such as bacon and sausages) is highly nutritious and may help us slim.

The British Nutrition Foundation said there was no evidence to suggest moderate amounts of lean red meat were unhealthy and that it may help fight obesity, its high protein improving the feeling of fullness.3. "Go to work on an egg"

This old advice died out in the 70s when it was reported eggs increased cholesterol levels. We now eat less than half the number we did in the 60s, switching to breakfast cereals - heavily processed grains with lots of sugar.

Recent studies show eggs don't raise cholesterol and are more nutritious than cereals, naturally packed with protein, vitamins A, E and D and zinc. One US study even found that when dieters ate two eggs for breakfast for five days a week they lost 65% more weight than those who ate only a bagel.4. Have fish on a Friday - and during the week

For years it was traditional in the UK to eat fish on a Friday, with sardines on toast a common lunch and kippers making a hearty breakfast. Today fish consumption is at an all-time low in Britain. We should aim for two portions a week, one of them oily fish - salmon, mackerel or fresh tuna - but nearly 60% of us don't hit this.

Which is madness when you consider that fish is a dream diet food, with white varieties a low-calorie source of protein and oily fish rich in the Omega-3 fats linked to better heart health.

A study published in the International Journal of Obesity found that when dieters ate fish a few times a week, they lost more weight than non-fish eaters.5. Cook your own meals

Unless you're a celebrity who can afford a private chef, letting other people cook for you means supermarket convenience food, handing over control of what you consume in the process - everything from portion size to ingredients.

Many of these meals contain chemicals like ethoxylated diglycerides or xantham gum. Granny didn't keep those in her pantry and the long-term effects are unknown. Vitabiotics nutritionist Anita Ellis says: "Cooking meals from scratch means you know exactly what goes into them and is far healthier and much cheaper on today's tight budgets." Little wonder that research consistently shows that people who cook regularly are slimmer than those who don't.6. Drink tap water or tea

Forget expensive fruit juices, smoothies and mineral waters. Tap water or tea are cheaper and healthier.

Scientists have found sugar-based calories like those found in sweet drinks are more likely to cause tummy fat than calories from other sources, with fruit juice no better than **fizzy drinks**.

The Government's Susan Jebb points out: "Fruit juice is high in sugar. Your stomach doesn't know whether it's Coca-Cola or orange juice."

Tap water is free, contains no sugar, fat or calories and studies suggest antioxidants in tea could protect against disease, including diabetes.7. Stop snacking

Granny would ban snacks between meals, saying "You'll ruin your appetite". She had a point.

Eating a nutritious three square meals a day was seen as common sense but we've become a nation of grazers, nibbling all the wrong high-calorie foods without ever having a properly balanced meal of protein, carbs and veg.8. Avoid foods with 'low-fat or 'lite' on the label

These labels disguise the fact manufacturers have often added extra sugar to make up for the lack of fat so the item still tastes appetising. And research now suggests diet foods don't make us feel as full as normal foods, so we're likely to over-eat in the long-run.9. Bake your own treats

In the past, cakes were home-made for special occasions - rather than a daily snack from the coffee shop. Chips were a once-a week-treat. Rather than banning such indulgent foods, writer Michael Pollan suggests simply cooking them yourself.

Whether it's chips, pies or ice cream, making these from scratch is pretty time-consuming and it's only because food factories have made them instantly available that we can consume them so often.

"Enjoy these treats as often as you're willing to prepare them," he says. "Chances are it won't be every day."10. Eat at the table

The dinner table was once the heart of family life. But TV dinners and longer working hours have made the dining ritual much less common.

Research has long maintained eating in front of a screen encourages mindless munching, which leads to the consumption of more calories.

Other studies show that families who eat together, especially around a table, stay slimmer together. Communal eating, with its interaction and chatter, slows the pace of meals, regulating appetite and preventing over-eating.

Food planner

LOAD-DATE: September 4, 2014

LANGUAGE: ENGLISH

PUBLICATION-TYPE: Newspaper; Web Publication

JOURNAL-CODE: WEBDMI

The Telegraph
telegraph.co.uk

April 15, 2014 Tuesday 5:23 PM GMT

Coca-Cola gets fizz back as sales decline slows; Drinks maker says falling sales in US have steadied as it took more market share from competitors

BYLINE: By Katherine Rushton US Business Editor

SECTION: FINANCE

LENGTH: 318 words

Coca-Cola reassured investors it is getting its fizz back on Tuesday, as the company steadied its decline and beat analysts' forecasts with its first quarter results.

Profits at the drinks giant fell 8pc to $1.62bn, whilst revenues slipped 4pc to $10.58bn. However, the slowdown was not as bad as feared, after a surge in sales in China and Russia.

The company does not break out revenues for those countries, but said that the volume of drinks sold in China rose by 12pc, helped by heavy advertising around Chinese New Year. A marketing blitz around the Sochi Winter Olympics also helped fuel a 9pc increase in volume sales in Russia.

Its performance in developing markets helped to offset a 4pc drop in Europe, where customers are losing their appetite for high-sugar, **fizzy drinks**. Sales in America, which have been in decline for the last few quarters, started to plateau.

"We're beginning to get our momentum back here the beginning of 2014," Muhtar Kent, chairman and chief executive of the company told investors. "All of us at The Coca-Cola Company remain confident in our ability to deliver on our strategies while further strengthening our foundation for profitable and sustainable long-term growth."

Coca-Cola and other drinks manufacturers have suffered in developed markets, as more consumers swap carbonated drinks for healthier options. The drinks industry has spent the past decade trying to offset that trend by introducing more low and zero-calorie versions of its beverages, but many consumers are now trying to cut back on those as well.

However, Coca-Cola said falling US sales had steadied, as the company took more market shares from competitors. "Our North America Group delivered even volume versus the prior year quarter while gaining value share and maintaining volume share," it said.

Shares in the company rose 3pc in morning trading in New York, as investors digested the brighter outlook.

LOAD-DATE: April 18, 2014

LANGUAGE: ENGLISH

PUBLICATION-TYPE: Newspaper; Web Publication

JOURNAL-CODE: WEBDTNS

Copyright 2014 Telegraph Media Group Limited
All Rights Reserved

The Telegraph
telegraph.co.uk

April 15, 2014 Tuesday 5:23 PM GMT

Coca-Cola gets fizz back as sales decline slows; Drinks maker says falling sales in US have steadied as it took more market share from competitors

BYLINE: By Katherine Rushton US Business Editor

SECTION: FINANCE

LENGTH: 318 words

Coca-Cola reassured investors it is getting its fizz back on Tuesday, as the company steadied its decline and beat analysts' forecasts with its first quarter results.

Profits at the drinks giant fell 8pc to $1.62bn, whilst revenues slipped 4pc to $10.58bn. However, the slowdown was not as bad as feared, after a surge in sales in China and Russia.

The company does not break out revenues for those countries, but said that the volume of drinks sold in China rose by 12pc, helped by heavy advertising around Chinese New Year. A marketing blitz around the Sochi Winter Olympics also helped fuel a 9pc increase in volume sales in Russia.

Its performance in developing markets helped to offset a 4pc drop in Europe, where customers are losing their appetite for high-sugar, **fizzy drinks**. Sales in America, which have been in decline for the last few quarters, started to plateau.

"We're beginning to get our momentum back here the beginning of 2014," Muhtar Kent, chairman and chief executive of the company told investors. "All of us at The Coca-Cola Company remain confident in our ability to deliver on our strategies while further strengthening our foundation for profitable and sustainable long-term growth."

Coca-Cola and other drinks manufacturers have suffered in developed markets, as more consumers swap carbonated drinks for healthier options. The drinks industry has spent the past decade trying to offset that

trend by introducing more low and zero-calorie versions of its beverages, but many consumers are now trying to cut back on those as well.

However, Coca-Cola said falling US sales had steadied, as the company took more market shares from competitors. "Our North America Group delivered even volume versus the prior year quarter while gaining value share and maintaining volume share," it said.

Shares in the company rose 3pc in morning trading in New York, as investors digested the brighter outlook.

LOAD-DATE: April 20, 2014

LANGUAGE: ENGLISH

PUBLICATION-TYPE: Newspaper; Web Publication

JOURNAL-CODE: WEBDTNS

Copyright 2014 Telegraph Media Group Limited
All Rights Reserved

The Telegraph

telegraph.co.uk

April 15, 2014 Tuesday 5:23 PM GMT

Coca-Cola gets fizz back as sales decline slows;
Drinks maker says falling sales in US have steadied as it took more market share from competitors

BYLINE: By Katherine Rushton US Business Editor

SECTION: FINANCE

LENGTH: 318 words

Coca-Cola reassured investors it is getting its fizz back on Tuesday, as the company steadied its decline and beat analysts' forecasts with its first quarter results.

Profits at the drinks giant fell 8pc to $1.62bn, whilst revenues slipped 4pc to $10.58bn. However, the slowdown was not as bad as feared, after a surge in sales in China and Russia.

The company does not break out revenues for those countries, but said that the volume of drinks sold in China rose by 12pc, helped by heavy advertising around Chinese New Year. A marketing blitz around the Sochi Winter Olympics also helped fuel a 9pc increase in volume sales in Russia.

Its performance in developing markets helped to offset a 4pc drop in Europe, where customers are losing their appetite for high-sugar, **fizzy drinks**. Sales in America, which have been in decline for the last few quarters, started to plateau.

"We're beginning to get our momentum back here the beginning of 2014," Muhtar Kent, chairman and chief executive of the company told investors. "All of us at The Coca-Cola Company remain confident in our ability to deliver on our strategies while further strengthening our foundation for profitable and sustainable long-term growth."

Coca-Cola and other drinks manufacturers have suffered in developed markets, as more consumers swap carbonated drinks for healthier options. The drinks industry has spent the past decade trying to offset that trend by introducing more low and zero-calorie versions of its beverages, but many consumers are now trying to cut back on those as well.

However, Coca-Cola said falling US sales had steadied, as the company took more market shares from competitors. "Our North America Group delivered even volume versus the prior year quarter while gaining value share and maintaining volume share," it said.

Shares in the company rose 3pc in morning trading in New York, as investors digested the brighter outlook.

LOAD-DATE: April 21, 2014

LANGUAGE: ENGLISH

PUBLICATION-TYPE: Newspaper; Web Publication

JOURNAL-CODE: WEBDTNS

Copyright 2014 Telegraph Media Group Limited
All Rights Reserved

Daily Mirror

April 14, 2014 Monday
Edition 1;
Northern Ireland

EAT MEAT AND BUTTER AND STILL LOSE WEIGHT;
The Mad Men Diet: Just as Mad Men returns to our TVs, research shows that retro eating habits could keep us slim and healthy...

BYLINE: Caroline Jones

SECTION: FEATURES; Pg. 26,27

LENGTH: 1257 words

Everything we thought we knew about the dangers of eating too much fat has been turned on its head. In a U-turn on advice we've been given for the last 40 years, a review of 72,000 studies found there was no evidence that saturated fats - found in butter, meat and full-fat milk - caused heart disease.

And Government diet adviser Dr Susan Jebb has backed plans to stop fruit juice - previously seen as a super-healthy drink - being counted as one of your five a day because it's so packed with sugar.

In fact sugar, we're now told, is the real baddie, not fat after all. Confused?

It seems much of what we've thought of as healthy for nearly half a century probably isn't so great and many of the unfashionable foods our grandparents tucked into could actually be good for us and our waistlines!

Nutritionist Linda Foster says: "A diet based on relatively old-fashioned foods, such as eggs for breakfast and plenty of home-cooked meals, including those containing some lean red meat and butter, can be pretty healthy and slimming, especially if you eat good amounts of seasonal fruit and veg as we would have done in the past."

Cooking your own meals as families used to do, rather than relying on processed convenience foods, is the real key, according to award-winning food writer Michael Pollan. His latest book - Cooked - blames our expanding girths on the fact we no longer cook real food. He points out the decline in home cooking "closely parallels the rise in obesity".

So perhaps we need to go back 50 years - when TV's Mad Men is set - or even 100 years to when being overweight was rare for some no-nonsense tips.

One 1914 poster from the American health board sums up advice that still holds true today: "Food: Buy it with thought. Cook it with care. Use less wheat and meat. Buy local foods. Serve just enough. Use what is left. Don't waste it."

Here's our guide, based on the good food sense of our grandmothers...

The 10 retro food rules

1 Butter your bread and drink milk

Your gran probably guzzled a pint of full-fat milk a day and spread butter thickly on her bread. But from the mid-1970s we were told it was clogging our arteries and we should switch to margarines, sunflower oils and skimmed milk.

Experts now say saturated fat may not be as bad as we thought, while other studies link some man-made spreads to health concerns of their own. "So you're probably safer with good old-fashioned butter in moderation," advises Linda.

Evidence also suggests whole milk could be better than skimmed as it contains only around 4% fat and is more nutritious because of vitamins A, D, E and K in the cream.

2 Tuck into (lean) red meat twice a week

In the past 40 years many of us have eaten less red meat because of worries that it's fattening or unhealthy. But it seems grandma knew best when she insisted the family eat liver once a week and hearty roast beef on Sundays.

Latest research suggests eating lean beef, liver, lamb or pork twice a week (as opposed to processed meats such as bacon and sausages) is highly nutritious and may help us slim.

The British Nutrition Foundation said there was no evidence to suggest moderate amounts of lean red meat were unhealthy and that it may help fight obesity, its high protein improving the feeling of fullness.

3 "Go to work on an egg"

This old advice died out in the 70s when it was reported eggs increased cholesterol levels. We now eat less than half the number we did in the 60s, switching to breakfast cereals - heavily processed grains with lots of sugar.

Recent studies show eggs don't raise cholesterol and are more nutritious than cereals, naturally packed with protein, vitamins A, E and D and zinc. One US study even found that when dieters ate two eggs for breakfast for five days a week they lost 65% more weight than those who ate only a bagel.

4 Have fish on a Friday - and during the week

For years it was traditional in the UK to eat fish on a Friday, with sardines on toast a common lunch and kippers making a hearty breakfast. Today fish consumption is at an all-time low in Britain. We should aim for

two portions a week, one of them oily fish - salmon, mackerel or fresh tuna - but nearly 60% of us don't hit this.

Which is madness when you consider that fish is a dream diet food, with white varieties a low-calorie source of protein and oily fish rich in the Omega-3 fats linked to better heart health.

A study published in the International Journal of Obesity found that when dieters ate fish a few times a week, they lost more weight than non-fish eaters.

5 Cook your own meals

Unless you're a celebrity who can afford a private chef, letting other people cook for you means supermarket convenience food, handing over control of what you consume in the process - everything from portion size to ingredients.

Many of these meals contain chemicals like ethoxylated diglycerides or xantham gum. Granny didn't keep those in her pantry and the long-term effects are unknown. Vitabiotics nutritionist Anita Ellis says: "Cooking meals from scratch means you know exactly what goes into them and is far healthier and much cheaper on today's tight budgets." Little wonder that research consistently shows that people who cook regularly are slimmer than those who don't.

6 Drink tap water or tea

Forget expensive fruit juices, smoothies and mineral waters. Tap water or tea are cheaper and healthier.

Scientists have found sugar-based calories like those found in sweet drinks are more likely to cause tummy fat than calories from other sources, with fruit juice no better than **fizzy drinks**.

The Government's Susan Jebb points out: "Fruit juice is high in sugar. Your stomach doesn't know whether it's Coca-Cola or orange juice."

Tap water is free, contains no sugar, fat or calories and studies suggest antioxidants in tea could protect against disease, including diabetes.

7 Stop snacking

Granny would ban snacks between meals, saying "You'll ruin your appetite". She had a point. Eating a nutritious three square meals a day was seen as common sense but we've become a nation of grazers, nibbling all the wrong high-calorie foods without ever having a properly balanced meal of protein, carbs and veg.

8 Avoid foods with 'low-fat or 'lite' on the label

These labels disguise the fact manufacturers have often added extra sugar to make up for the lack of fat so the item still tastes appetising. And research now suggests diet foods don't make us feel as full as normal foods, so we're likely to over-eat in the long-run.

9 Bake your own treats

In the past, cakes were home-made for special occasions - rather than a daily snack from the coffee shop. Chips were a once-a week-treat. Rather than banning such indulgent foods, writer Michael Pollan suggests simply cooking them yourself.

Whether it's chips, pies or ice cream, making these from scratch is pretty time-consuming and it's only because food factories have made them instantly available that we can consume them so often.

"Enjoy these treats as often as you're willing to prepare them," he says. "Chances are it won't be every day."

10 Eat at the table

The dinner table was once the heart of family life. But TV dinners and longer working hours have made the dining ritual much less common.

Research has long maintained eating in front of a screen encourages mindless munching, which leads to the consumption of more calories.

Other studies show that families who eat together, especially around a table, stay slimmer together. Communal eating, with its interaction and chatter, slows the pace of meals, regulating appetite and preventing over-eating.

LOAD-DATE: April 14, 2014

LANGUAGE: ENGLISH

GRAPHIC: WISE Six sensible tips from the US health board back in 1914
RETRO The cast of the final series of Mad Men, which returns to our screens this Wednesday

PUBLICATION-TYPE: Newspaper

JOURNAL-CODE: DMR

MailOnline

MailOnline

April 6, 2014 Sunday 11:27 AM GMT

Low fat foods contain an average 20% more sugar than full fat equivalents and can increase risk of weight gain and diabetes, health experts warn

BYLINE: MARK DUELL

SECTION: NEWS

LENGTH: 543 words

- Experts label some of sugar levels found in a probe as dangerous
- Eating 'skinny' version of certain products could lead to health risk
- Channel 4's Dispatches tests some of UK's best-known food brands

'Low fat' foods contain 20 per cent more sugar on average than their full fat equivalents, a study has claimed.

Experts labelled some of the sugar levels found in an investigation as dangerous, adding that eating the 'skinny' version of certain products could lead to a higher risk of diabetes and weight gain.

Investigators tested some of Britain's best-known food brands and suggested there are inaccuracies in the labelling of fat content - and that consumers often struggle to understand food labels.

The probe by reporter Antony Barnett for Channel 4's Dispatches: The Truth about Low Fat Food looks at how many people wade through confusing messages about what they should eat.

The programme also sees one couple eat nothing but low-fat foods for seven days - and examines the effect this has on their intake of the ingredients many people try their best to avoid.

It found one of Waitrose's own-brand low-fat vinaigrette had ten times the sugar content of the standard version - 15.6g compared to 1.58g, reported the Sunday Mirror 's Francesca Cookney.

In addition, Tesco allegedly puts almost three times as much sugar into its '25 per cent less fat' peanut butter than in its ordinary spread - 15.2g compared to 5.5g.

And Morrisons frozen chicken tikka masala meal contains 7.4g of sugar - less than half what is in the NuMe chicken tikka masala meal of 16.3g. The recommended daily limit of sugar for adults is 90g.

According to the Sunday Mirror, dietician Nicole Berberian said: 'Some of the products we looked at had extremely high levels of sugar.

'Without fat, sugar is absorbed rapidly into the body giving you a large intake of energy which you may not actually need. If you're not an athlete who's burning it off, that's a bad idea.

'The sugar will convert into fat and you'll also find you feel hungry again quicker and end up eating more. Continuously high sugar levels contribute to obesity and diabetes.'

'Without fat, sugar is absorbed rapidly into the body giving you a large intake of energy which you may not actually need'

Nicole Berberian, dietician

A Waitrose spokesman told MailOnline: 'The nutritional content of our products is a high priority and we're progressively reducing sugar content across our ranges.

'For example, we have just revamped our chilled fruit juices - taking out 7.1 tonnes of sugar over the next year.'

And a Tesco spokesman said: 'Helping our customers to make healthy choices is a key priority for us and all our products are clearly labelled with nutritional information on the front of the pack.

'We always act in accordance with the strict rules around the use of words such as low fat or light. We are also working hard to reduce **sugar** across our ranges. For example, we have reduced the **sugar** content in our **soft drinks** by 30 per cent.'

The press offices of Tesco and Morrisons have been contacted for comment by MailOnline. Dispatches: The Truth About Low Fat Food will be broadcast on Channel 4 tomorrow night at 8pm.

LOAD-DATE: April 6, 2014

LANGUAGE: ENGLISH

PUBLICATION-TYPE: Web Publication

JOURNAL-CODE: WEBDM

Copyright 2014 Associated Newspapers Ltd.
All Rights Reserved

The Sunday Telegraph

The Sunday Telegraph (London)

April 6, 2014
Edition 1;
National Edition

Fruit drinks four times over the limit;

Study by Telegraph finds juice sold in supermarkets and coffee shops contains more than 20 teaspoons of sugar: world health guidelines are six teaspoons a day

BYLINE: EDWARD MALNICK

SECTION: NEWS; Pg. 16

LENGTH: 1171 words

SOME FRUIT juices and smoothies contain four times the amount of sugar the World Health Organisation recommends an average person should consume in a day, a Sunday Telegraph analysis shows.

A survey of 50 products from supermarkets, coffee shops and food outlets found that more than half contained at least six teaspoons of sugar, which is the recommended daily limit.

Two of the items - large fruit drinks from Costa, the coffee-shop chain - contained at least 23 teaspoons in a single serving.

Experts and campaigners yesterday described the amounts as "horrifying". They believe that high levels of sugar are contributing to health conditions such as diabetes, heart disease, tooth decay and cancer.

The findings follow a warning from experts that a healthy diet should include 10 portions of fruit and vegetables a day, doubling the current five-a-day official advice.

Much of the sugar found in fruit juices is naturally occurring rather than added. However, the NHS advises that fruit juice, restricted to a 150ml serving, should only make up one of the recommended five daily portions.

It warns that such drinks can damage teeth because sugar that would otherwise be contained within the structure of whole fruit is released when the fruit is juiced or blended.

Action on Sugar, a campaign group, said ministers should act to remove fruit juices and smoothies from the recommendation, relegating **sugary drinks** to an "occasional treat".

Prof Susan Jebb, a government obesity adviser, warned earlier this year that people should "swap" juice for a piece of fruit, or at least dilute it.

Aseem Malhotra, a cardiologist and Action on Sugar's science director, said: "The level of sugar in some of these drinks is horrifying, with many containing well above the upper limit of six teaspoons of sugar consumption recommended by the World Health Organisation.

"In my view **sugary drinks** such as these should be consumed as an occasional treat, perhaps once a month.

"Scientific studies have shown that consuming even one **sugary drink** a day is associated with a significantly increased risk of developing type 2 diabetes even in normal weight people.

"The Department of Health and the Food Standards Agency must act immediately to remove fruit juice and smoothies from the 'five a day' recommendations and must emphasise that these drinks should not be recommended to young children as part of a healthy diet."

Prof Paula Moynihan, professor of nutrition at the University of Newcastle, who advised the WHO on its sugar limits, added that many of the drinks seemed "unnecessarily large". Some of the drinks served by food outlets amounted to almost two-thirds of a litre.

Waitrose, said it was introducing a new range of fruit juices with reduced sugar levels - as well as replacing existing drinks - as the supermarket acknowledged they could contain "high levels" of naturally occurring sugar.

Fruit drinks four times over the limit; Study by Telegraph finds juice sold in supermarkets and coffee shops contains more than 20 teaspoons of sugar: world health guidelines are six teaspoons a day The Sunday Telegraph (London) April 6, 2014

The Sunday Telegraph carried out a survey of 50 fruit juices, smoothies and other fruit-based drinks on sale at a series of supermarkets, high-street shops and food outlets.

Of the items surveyed, 21 contained more than six teaspoons of sugar - which equates to around 24g. Ten drinks contained 12 or more teaspoons.

Costa's "red berry cooler" had the highest amount of sugar of the products examined, with a "massimo" (610ml) takeaway cup containing 97.1g, or around 24 teaspoons - four times the daily limit now advised by the WHO.

Costa's tropical fruit and mango and passion fruit drinks contained 23 and 17 teaspoons of sugar respectively in a massimo serving.

Other drinks containing 12 or more teaspoons of sugar included CaffÈ Nero's 655ml raspberry, orange and green tea "fruit booster", which had 17 teaspoons; Starbucks' "venti" (591ml) raspberry blackcurrant frappuccino contained 14 teaspoons; a large (500ml) strawberry and banana iced fruit smoothie from McDonald's had 13 teaspoons, and Pret a Manger's "beet beautiful juice" contained 12 teaspoons in a 400ml bottle.

Many of the drinks from coffee shops and food outlets came in much larger sizes than the bottles sold in supermarkets, or the recommended serving - usually between 150ml and 250ml - listed on larger cartons of drinks. However, of the 29 drinks containing six or more teaspoons of sugar, 14 were from supermarkets.

They included fruit juices and smoothies produced by Tesco, Sainsbury's, Waitrose and Asda. Forty-one of the drinks examined - the vast majority - contained five or more teaspoons of sugar in a single serving.

Last week the WHO closed a consultation on new draft guidance, which retains its current formal recommendation that no more than 10 per cent of an individual's daily calories should come from "free" sugar - the equivalent of 12 "level" teaspoons - for the average adult (one level teaspoon equates to around 4g).

"Free" sugar is added sugar, plus that naturally present in honey, syrups and fruit juices.

However, its proposed guidelines state that a further reduction to 5 per cent - around six teaspoons - "would have additional benefits".

A senior official from the WHO described the target as the "ideal" limit for which people should now aim.

Retailers insisted that clear labelling, or nutritional information detailed on their websites, enabled consumers to make "informed" choices.

A spokesman for Costa said: "Costa seeks to provide customers with a choice of products across its drink and food ranges, allowing them to choose lower-calorie options if they so wish as well as a more indulgent occasional treat."

A spokesman for Pret A Manger said: "We believe that natural sugars extracted from fresh fruit and vegetables are a better alternative to artificial sugars such as aspartame and fructose-glucose syrups, which are so commonly found today."

SPOONFULS OF SUGAR...

Costa red berry cooler 610ml cup 24tsp

Costa tropical fruit 610ml cup 23tsp

Costa mango and passion fruit 610ml cup 17tsp

CaffÈ Nero fruit booster raspberry, orange and green tea 655ml cup 17tsp

Starbucks raspberry blackcurrant frappuccino 591ml cup 14tsp

Starbucks mango passion fruit frappuccino 591ml cup 13tsp

McDonald's strawberry and banana iced fruit smoothie 500ml 13tsp

McDonald's mango and pineapple iced fruit smoothie 500ml cup 13tsp

Pret A Manger beet beautiful juice 400ml bottle 12tsp

McDonald's Fruiti 500ml cup 12tsp

Caf fÈ Nero fruit booster mango and passion fruit 500ml cup lltsp

Pure Pret apple drink 330ml 9tsp

Pret A Manger mango smoothie 250ml 8tsp

Jammin Tropical juice drink 250ml serving 8tsp

Welch's white grape, pear and apple juice drink 250ml serving 7tsp

Tesco red berries smoothie 250ml serving 7tsp

Tesco Juice Bar Super Fruita smoothie 250ml bottle 7tsp

Tesco apple juice I 250ml serving 7 tsp

Tesco apple and mango juice 250ml serving 7 tsp

Pret A Manger super smoothie 400ml bottle 7 tsp

Asda exotic juice 250ml carton 7 tsp

Waitrose pure pressed red grape juice 1150ml serving 16 tsp

I Tesco everyday value I orange juice I 250ml serving 6 tsp

I Sainsbury's cranberry juice drink 200ml 6 tsp

Sainsbury's apple and I raspberry juice drink I 200ml serving 6 tsp

LOAD-DATE: April 6, 2014

LANGUAGE: ENGLISH

GRAPHIC: The vast majority of fruit drinks had five or more teaspoons of sugar

PUBLICATION-TYPE: Newspaper

JOURNAL-CODE: STL

Copyright 2014 Telegraph Media Group Limited
All Rights Reserved

The Telegraph
telegraph.co.uk

April 5, 2014 Saturday 8:16 PM GMT

Fruit juices and smoothies contain 'horrifying' sugar levels;

Telegraph analysis shows that many fruit juices and smoothies contain more sugar than the World Health Organisation recommends an average person should consume in a day

BYLINE: By Edward Malnick

SECTION: NEWS

LENGTH: 964 words

Some fruit juices and smoothies contain four times the amount of sugar the World Health Organisation recommends an average person should consume in a day, a Telegraph analysis shows.

A survey of 50 products from supermarkets, coffee shops and food outlets found that more than half contained at least six teaspoons of sugar, which is the recommended daily limit.

Two of the items - large fruit drinks from Costa, the coffee-shop chain - contained at least 23 teaspoons in a single serving.

Experts and campaigners described the amounts as "horrifying". They believe that high levels of sugar are contributing to health conditions such as diabetes, heart disease, tooth decay and cancer.

The findings follow a warning from experts that a healthy diet should include 10 portions of fruit and vegetables a day, doubling the current five-a-day official advice.

Much of the sugar found in fruit juices is naturally occurring rather than added. However, the NHS advises that fruit juice, restricted to a 150ml serving, should only make up one of the recommended five daily portions.

It warns that such drinks can damage teeth because sugar that would otherwise be contained within the structure of whole fruit is released when the fruit is juiced or blended.

Datatable: Sugar levels in fruit juices and smoothies

Action on Sugar, a campaign group, said ministers should act to remove fruit juices and smoothies from the recommendation, relegating **sugary drinks** to an "occasional treat".

Prof Susan Jebb, a government obesity adviser, warned earlier this year that people should "swap" juice for a piece of fruit, or at least dilute it.

Aseem Malhotra, a cardiologist and Action on Sugar's science director, said: "The level of sugar in some of these drinks is horrifying, with many containing well above the upper limit of six

teaspoons of sugar consumption recommended by the World Health Organisation.

"In my view **sugary drinks** such as these should be consumed as an occasional treat, perhaps once a month.

"Scientific studies have shown that consuming even one **sugary drink** a day is associated with a significantly increased risk of developing type 2 diabetes even in normal weight people.

"The Department of Health and the Food Standards Agency must act immediately to remove fruit juice and smoothies from the 'five a day' recommendations and must emphasise that these drinks should not be recommended to young children as part of a healthy diet."

Prof Paula Moynihan, professor of nutrition at the University of Newcastle, who advised the WHO on its sugar limits, added that many of the drinks seemed "unnecessarily large". Some of the drinks served by food outlets amounted to almost two-thirds of a litre.

Waitrose said it was introducing a new range of fruit juices with reduced sugar levels - as well as replacing existing drinks - as the supermarket acknowledged they could contain "high levels" of naturally occurring sugar.

The Telegraph carried out a survey of 50 fruit juices, smoothies and other fruit-based drinks on sale at a series of supermarkets, high-street shops and food outlets.

Of the items surveyed, 21 contained more than six teaspoons of sugar - which equates to around 24g. Ten drinks contained 12 or more teaspoons.

Costa's "red berry cooler" had the highest amount of sugar of the products examined, with a "massimo" (610ml) takeaway cup containing 97.1g, or around 24 teaspoons - four times the daily limit now advised by the WHO.

Costa's tropical fruit and mango and passion fruit drinks contained 23 and 17 teaspoons of sugar respectively in a massimo serving.

Other drinks containing 12 or more teaspoons of sugar included Caffè Nero's 655ml raspberry, orange and green tea "fruit booster", which had 17 teaspoons; Starbucks' "venti" (591ml) raspberry blackcurrant frappuccino contained 14 teaspoons; a large (500ml) strawberry and banana iced fruit smoothie from McDonald's had 13 teaspoons, and Pret a Manger's "beet beautiful juice" contained 12 teaspoons in a 400ml bottle.

Many of the drinks from coffee shops and food outlets came in much larger sizes than the bottles sold in supermarkets, or the recommended serving - usually between 150ml and 250ml - listed on larger cartons of drinks. However, of the 29 drinks containing six or more teaspoons of sugar, 14 were from supermarkets.

They included fruit juices and smoothies produced by Tesco, Sainsbury's, Waitrose and Asda. Forty-one of the drinks examined - the vast majority - contained five or more teaspoons of sugar in a single serving.

Last week the WHO closed a consultation on new draft guidance, which retains its current formal recommendation that no more than 10 per cent of an individual's daily calories should come from "free" sugar - the equivalent of 12 "level" teaspoons - for the average adult (one level teaspoon equates to around 4g).

"Free" sugar is added sugar, plus that naturally present in honey, syrups and fruit juices.

However, its proposed guidelines state that a further reduction to 5 per cent - around six teaspoons - "would have additional benefits".

A senior official from the WHO described the target as the "ideal" limit for which people should now aim.

Retailers insisted that clear labelling, or nutritional information detailed on their websites, enabled consumers to make "informed" choices.

A spokesman for Costa said: "Costa seeks to provide customers with a choice of products across its drink and food ranges, allowing them to choose lower-calorie options if they so wish as well as a more indulgent occasional treat."

A spokesman for Pret A Manger said: "We believe that natural sugars extracted from fresh fruit and vegetables are a better alternative to artificial sugars such as aspartame and fructose-glucose syrups, which are so commonly found today."

LOAD-DATE: April 6, 2014

LANGUAGE: ENGLISH

PUBLICATION-TYPE: Newspaper; Web Publication

JOURNAL-CODE: WEBDTNS

Copyright 2014 Telegraph Media Group Limited
All Rights Reserved

Daily Mirror

April 4, 2014 Friday
Edition 1;
Northern Ireland

THEY THINK IT'S ALL COLA...;
CITY DESK

BYLINE: GRAHAM HISCOTT

SECTION: Pg. 44

LENGTH: 91 words

Fizzy drinks giants Coca Cola and Pepsi are locked in a World Cup clash.

Coca Cola, an official World Cup sponsor since 1978, has kicked off its biggest-ever marketing campaign to tie in with the tournament in Brazil.

Using the slogan "The World's Cup", the firm is planning an advertising blitz to boost sales.

Meanwhile, arch rival Pepsi has signed-up a galaxy of footie stars, including Arsenal's Jack Wilshere and Barcelona legend Lionel Messi, to ruin Coke's party.

Pepsi used the same day as Coca Cola to launch its all-star 'Live for Now' advert.

LOAD-DATE: April 4, 2014

LANGUAGE: ENGLISH

GRAPHIC: PEPSI MAN Wilshere

PUBLICATION-TYPE: Newspaper

JOURNAL-CODE: DMR

Copyright 2014 MGN Ltd.
All Rights Reserved

MailOnline

MailOnline

June 1, 2014 Sunday 9:14 PM GMT

The bitter truth about pledges to cut sugar: Food giants including Coca-Cola and Nestle accused of side-stepping promises to make products less fattening

BYLINE: BEN SPENCER

SECTION: SCIENCE

LENGTH: 814 words

- Producers pledged to reduce sugar following a 2011 deal with Government
- However, some of the world's biggest food producers have failed to act
- 37 firm signed the 2011 pledge including Coca Cola, Mars, Nestle and PepsiCo
- Coca Cola has reduced sugar in Sprite but refused to change the recipe of Coke
- A 330ml can of Coke contains 35g or nine teaspoons of sugar

Food giants which promised to make products healthier under the Government's trumpeted 'responsibility deal' have simply side-stepped their pledges, it has emerged.

Some of the world's biggest firms have failed to reduce calories or sugar content in their most popular brands - including Coca Cola, Magnum ice cream and Kit Kat chocolate.

The Public Health Responsibility Deal, introduced by the Government in 2011, has been much criticised by health campaigners, who say it gives the food and drink industries carte blanche to regulate themselves.

Some 663 organisations are 'partners' in the responsibility deal, under which they have made variety of pledges covering marketing, labelling and workplace practices.

Thirty seven firms, including Coca Cola, Mars, Nestlé and PepsiCo, signed up to a 'calorie reduction' pledge.

Their promise included 'supporting and enabling' customers to eat and drink fewer calories through actions such as product/menu reformulation, reviewing portion sizes, education and information, and actions to shift the marketing mix towards lower calorie options'.

But it has emerged that some of the companies have not touched their main brands - instead focusing on reduced sugar in lesser-known products or altering labelling.

Campaigners called on ministers to abandon the voluntary system and instead introduce sugar reduction targets similar to those in place for salt.

Coca Cola has reduced the calories in its Sprite drink - but has refused to change its flagship product.

Unilever, which makes Wall's and Ben & Jerry's ice creams has reduced the number of calories in children's ice creams but not in its normal range.

Nestlé has cut the calories in Aero and white Kit Kat Chunky bars but not in ordinary milk chocolate Kit Kats.

Keith Vaz MP, chairman of the Commons home affairs select committee, told the Sunday Telegraph: 'The problem with the Responsibility Deal is that it is all voluntary.

'As far as Coke is concerned it remains the same product it was 10 years ago - there doesn't seem to have been any change at all.

'We need targets. Unless we get the target we have no benchmark.'

Health experts have become increasingly concerned at the population's intake of sugar - comparing it to tobacco in terms of its public health impact.

Simon Capewell, professor of clinical epidemiology at the University of Liverpool, said in January: 'Sugar is the new tobacco.

'Everywhere, **sugary drinks** and junk foods are now pressed on unsuspecting parents and children by a cynical industry focused on profit not health.

'Obesity and diabetes already cost the UK over £5billion a year. Without regulation, these costs will exceed £50billion by 2050.'

As part of its commitment to the calorie pledge, Coca Cola has cut the amount of sugar and calories in Glacéau vitaminwater and Sprite by 30 per cent.

But a 330ml can of Coca Cola still contains 35g - or nine teaspoons - of sugar.

A spokesman for Coca Cola GB said: 'We have no plans to change Coca Cola. We know that people love it and we provide two great-tasting sugar-free, no calorie options in Diet Coke and Coca-Cola Zero, which together comprise more than 40 per cent of the cola we sell in the UK.'

Nestlé has reduced the number of calories in Aero and white KitKat Chunky bars by 5 per cent.

However, it has not reduced the number of calories in milk chocolate KitKat bars and other popular products such as Lions and Rolos.

Unilever, which owns Wall's and Ben & Jerry's, pledged to cut the number of calories in children's ice creams, which now contain 110 or fewer calories per serving.

It is also offering 'smaller and lower calorie alternative choices' such as mini Magnums. But ordinary Magnum bars and ice cream tubs from Ben & Jerry's have not been changed.

A Unilever spokesman said: 'Sugar plays an important role in ice cream, not only for taste but also for texture and structure. However, we have a programme in place that aims to gradually reduce sugar in our products whilst maintaining our high standards of product quality.'

A Department of Health spokesman said: 'Many manufacturers and retailers are already taking sugar, fat and salt out of their products as a result of our Responsibility Deal, which is based on working collaboratively with industry rather than imposing unrealistic targets.

'The Scientific Advisory Committee on Nutrition is currently undertaking a review of carbohydrates, and they are looking at sugar as part of this. Their draft report which is due to be published later this year will inform our future thinking.'

LOAD-DATE: June 1, 2014

LANGUAGE: ENGLISH

PUBLICATION-TYPE: Web Publication

JOURNAL-CODE: WEBDM

Copyright 2014 Associated Newspapers Ltd.
All Rights Reserved

MailOnline

June 1, 2014 Sunday 11:03 AM GMT

FUNDSMITH EQUITY: Can fizzy drinks put sparkle in new emerging markets trust from star Terry Smith?

BYLINE: JEFF PRESTRIDGE, FINANCIAL MAIL ON SUNDAY

SECTION: INVESTING

LENGTH: 392 words

Neil Woodford's Equity Income Fund, which will be launched tomorrow, is not the only new fund in town.

Terry Smith, chief executive of investment house Fundsmith, hopes to raise £250 million from the current launch of an investment trust focusing on emerging markets.

Like Woodford, who enjoyed incredible success at Invesco Perpetual, managing its Income and High Income funds before going it alone with Woodford Investment Management, Smith has an enviable record - albeit over a shorter period.

His Fundsmith Equity Fund is among the top performing in its global equity sector, delivering a return of 67 per cent since its launch three and a half years ago.

Smith knows his mind. For a start, he prefers to hold cash generative businesses that are consumer focused - from food and drink manufacturers to hotel groups.

Among the equity fund's top holdings are American drinks giant Doctor Pepper Snapple - the producer of Canada Dry, 7 Up and Sunkist - and Domino's Pizza. He has no truck with banks, chemical firms or airlines - all too risky.

He also likes to run concentrated portfolios and stick with holdings through thick and thin. The result is that the fund has only 25 stocks.

He says: 'With a portfolio of 25 stocks, I have all the diversity I need as a fund manager. It means the fund is concentrated on the world's greatest companies.'

Other holdings include Imperial Tobacco, Microsoft and Unilever.

Unsurprisingly these investment principles will underpin the new trust. So the 35 to 55 stocks will be consumer focused with an ability to generate cash. The difference is that the companies he buys will have a big slice of their operations in developing economies. Smith says independent research shows that consumption in emerging markets will rise from $12 trillion (£7 trillion) in 2010 to $30 trillion in 2025, an increase of 150 per cent.

For developed markets, the increase will be just 30 per cent.

The trust's launch could be well timed. Hugh Young, managing director of Aberdeen Asset Management's Asian operations, believes that emerging markets 'appear to be turning a corner' after years of underperformance.

Further details on the new trust can be obtained at feetplc.co.uk. The minimum investment during the offer period, which closes at noon on June 20, is £1,000. Details on the Woodford Equity Income Fund can be found at hl.co.uk.

LOAD-DATE: June 1, 2014

LANGUAGE: ENGLISH

PUBLICATION-TYPE: Web Publication

JOURNAL-CODE: WEBDM

Copyright 2014 Associated Newspapers Ltd.
All Rights Reserved

The Sunday Telegraph

The Sunday Telegraph (London)

June 1, 2014
Edition 1;
National Edition

Food firms sidestep pledge on sugar;
Firms fail to cut sugar in top brands

BYLINE: EDWARD MALNICK

SECTION: NEWS; Pg. 1,2

LENGTH: 1067 words

FOOD COMPANIES signed up to the Government's flagship "healthy eating" pledge have failed to reduce the amount of sugar in some of their best-known brands.

The sugar and overall calorie levels in products such as Coca-Cola and Magnum ice creams have remained the same despite their manufacturers promising to help customers to "eat and drink fewer calories".

Instead, the companies have reduced sugar in lesserknown products and introduced measures such as improved labelling and promotion of sugar-free alternatives.

Last night, MPs and campaigners said it showed that the "calorie pledge" in the Government's Responsibility Deal, under which food manufacturers pledged to encourage healthier eating, was failing.

They called on ministers to introduce specific sugar reduction targets, similar to those in place for salt since 2006, to tackle rising levels of obesity and health problems such as type 2 diabetes. Researchers said in April that reduced consumption of salt had played an "important role" in the falling number of deaths from heart disease.

After the Department of Health published its annual update on the Responsibility Deal last week, Jane Ellison, the public health minister, congratulated the food companies involved on their "successes". But The Sunday Telegraph's survey of action taken under the deal found that Coca-Cola has reduced the amount of sugar in drinks such as Sprite but left its flagship drink untouched.

The firm behind Wall's and Ben & Jerry's ice creams has reduced the number of calories in "children's ice creams" but not in ordinary ranges; Nestlé has cut the calories in Aero and white KitKat Chunky bars but not in ordinary milk chocolate KitKats.

Keith Vaz, the Labour chairman of the Commons home affairs select committee, said: "The problem with the Responsibility Deal is that it is all voluntary.

"As far as Coke is concerned it remains the same product it was 10 years ago - there doesn't seem to have been any change at all."

Warning that consumption of sugar was at "crisis levels", Mr Vaz, who has type 2 diabetes, added: "We need targets. Unless we get the target we have no benchmark."

Earlier this month Mr Vaz led a parliamentary debate calling for food labels to include the number of teaspoons in each product. He was backed by Action on Sugar, which claimed the move would help to ensure that firms reduce calories "across the board".

Kawther Hashem, a nutritionist for Action on Sugar said: "Examples such as these show the Responsibility Deal's calorie pledge is completely ineffectual.

"It is time for the Department of Health to introduce sugar reduction targets, to encourage companies to reduce sugar and calorie content of all their products, across the board, creating the muchneeded level playing field."

In total 663 organisations are "partners" in the responsibility deal and made a variety of pledges.

Thirty-seven firms, including Coca Cola, Mars, Nestlé and PepsiCo, signed up to the "calorie reduction" pledge to "support and enable our customers to eat and drink fewer calories through actions such as product/menu reformulation, reviewing portion sizes, education and information, and actions to shift the marketing mix towards lower calorie options."

As part of its commitment to the calorie pledge, Coca Cola has cut the amount of sugar in Glacéau vitaminwater by 30 per cent and the number of overall calories in Sprite by the same amount. It has also introduced a 250ml can as a "slimline" alternative to the normal 330ml helping. However, the amount of sugar and overall number of calories in a 330ml can of Coca-Cola original remains at 139, including 35g, or nine level teaspoons, of sugar.

The firm said it had no plans to reduce the sugar or overall calorie levels in the drink "as we know our consumers love its great taste".

AG Barr, the Scottish firm which makes Irn Bru, made a pledge under the deal to reduce the average calorific content per 100ml of its drinks by 5 per cent by 2016.

However, original Irn Bru, which contains 107 calories, including 6.5 teaspoons of sugar, in a 250ml serving, has not been changed.

Instead the firm aims to increase sales of "**sugar** free" Irn Bru, which accounts for 32 per cent of total Irn Bru sales. AG Barr has also reduced **sugar** in lesser-known **soft drinks**, including Barr Cola, Tizer and Red Kola, by between 2 per cent and 46 per cent.

Nestlé has reduced the number of calories in Aero and white KitKat Chunky bars by 5 per cent. It has also increased the volume of items with fewer than 110 calories per serving and produced new lowercalorie products. However, it has not reduced the number of calories in milk chocolate KitKat bars and other popular products such as Lions and Rolos.

Unilever, which owns Wall's and Ben & Jerry's, pledged to cut the number of calories in "children's ice creams", which now contain 110 or fewer calories per serving. It is also offering "smaller and lower calorie alternative choices" such as "mini" Magnums. However, ordinary Magnum bars, as well as ice cream tubs from Ben & Jerry's, have not been affected.

A spokesman for Coca-Cola GB said: "We have no plans to change Coca-Cola. We know that people love it and we provide two greattasting sugar-free, no calorie options in Diet Coke and Coca-Cola Zero, which together comprise more than 40 per cent of the cola we sell in the UK."

A spokesman for AG Barr said: "The Irn-Bru recipe delivers a unique taste and flavour which is recognised and loved by the brand's many loyal consumers, who we know will not accept any change."

A Nestlé spokesman said: "We continue to seek opportunities across our business to improve the nutritional profile of our products without compromising the taste and quality of the products our consumers love."

The firm had "reformulated" its Kit Kat bar to remove the equivalent of 3,800 tons of saturated fat, the spokesman added.

A Unilever spokesman said: "Sugar plays an important role in ice cream, not only for taste but also for texture and structure. However, we have a programme in place that aims to gradually reduce sugar in our products whilst maintaining our high standards of product quality."

A Department of Health spokesman said: "Many manufacturers and retailers are already taking sugar, fat and salt out of their products as a result of our Responsibility Deal, which is based on working collaboratively with industry rather than imposing unrealistic targets."

LOAD-DATE: June 1, 2014

LANGUAGE: ENGLISH

GRAPHIC: Eva Longoria advertises Magnums

PUBLICATION-TYPE: Newspaper

JOURNAL-CODE: STL

Copyright 2014 Telegraph Media Group Limited
All Rights Reserved

THE SUNDAY TIMES
The Sunday Times (London)

June 1, 2014 Sunday
Edition 1;
Scotland

Food firms 'have too much power'

BYLINE: Mark Macaskill

SECTION: NEWS; Pg. 14

LENGTH: 467 words

A SCOTTISH academic recruited by the World Health Organisation (WHO) to help fight obesity said weakening the influence of the food industry over governments is a first step towards improving diets.

Gerard Hastings, professor of social marketing at Stirling and the Open universities, expressed concern about the "creeping commercialisation" of society, which sees vested interests such as the food industry wielding "significant influence" over government policy.

Hastings, who will travel to Geneva shortly for the inaugural meeting of the WHO's global commission on obesity, argues that the influence of big business on government food policy warrants close scrutiny.

"Transparency is really important. The job of business is to increase sales, bolster profits and maximise returns to shareholders. Its role in the policy arena should be carefully red-lined; it has a role in implementing policy, but not in forming it.

Government is forever making the mistake of thinking that businesses can be a partner in improving public health. They need to be involved, but not as a partner."

Hastings warned that the availability of unhealthy food is out of control. He stops short of calling for certain foods to be banned but suggests radical changes are required to tackle the sale of sugary foods. "It's the Wild West as far as business and marketing is concerned. If you come up with a product, by and large, no one says hang on, is this a good idea for the consumer - or indeed the planet? There's very little constraint. The assumption is that you have a God-given right to make something and sell it; but is there really a need for yet another **sugary drink** or fatty confection?" The UK has higher levels of obesity and overweight people than anywhere in western Europe except for Iceland and Malta, according to a recent study in the Lancet medical journal. It found 67% of men and 57% of women are overweight or obese. More than a quarter of children are also overweight or obese - 26% of boys and 29% of girls.

Among the big businesses seeking to tackle obesity are Coca-Cola Great Britain, which is investing £20m in physical activity programmes to get 1m people active by 2020. It will offer a range of free activities including badminton and tai chi in Birmingham, Newcastle and London this summer with expansion planned across the UK. Tesco recently announced sweets and chocolate are to be banned from store checkouts after a survey of parents found about two-thirds believed removing confectionery near the checkout would help them make healthier choices for their children.

Hastings welcomed their efforts but said they were not driven by altruism. He cited a colleague who, upon hearing of Coca-Cola's plans, remarked that is "like the fox saying it will pay for self-defence classes for the chickens".

LOAD-DATE: June 1, 2014

LANGUAGE: ENGLISH

PUBLICATION-TYPE: Newspaper

JOURNAL-CODE: STSscot

Copyright 2014 Times Newspapers Limited
All Rights Reserved

MailOnline

telegraph.co.uk

May 31, 2014 Saturday 10:40 PM GMT

Manufacturers fail to reduce sugar despite 'healthy eating' pledge, Telegraph finds;
Telegraph finds that sugar and overall calorie levels in items such as Coca-Cola and Magnum ice creams have remained the same despite their manufacturers promising to lower them

BYLINE: By Edward Malnick

SECTION: NEWS

LENGTH: 1223 words

Food companies signed up to the Government's flagship "healthy eating" pledge have failed to reduce the amount of sugar in some of their best-known brands.

The sugar and overall calorie levels in products such as Coca-Cola and Magnum ice creams have remained the same despite their manufacturers promising to help customers to "eat and drink fewer calories".

Instead, the companies have reduced sugar in lesser-known products and introduced measures such as improved labelling and promotion of sugar-free alternatives.

MPs and campaigners said it showed that the "calorie pledge" in the Government's Responsibility Deal, under which food manufacturers pledged to encourage healthier eating, was failing.

They called on ministers to introduce specific sugar reduction targets, similar to those in place for salt since 2006, to tackle rising levels of obesity and health problems such as type 2 diabetes. Researchers said in April that reduced consumption of salt had played an "important role" in the falling number of deaths from heart disease.

After the Department of Health published its annual update on the Responsibility Deal last week, Jane Ellison, the public health minister, congratulated the food companies involved on their "successes".

But The Telegraph's survey of action taken under the deal found that Coca-Cola has reduced the amount of sugar in drinks such as Sprite but left its flagship drink untouched.

The firm behind Wall's and Ben & Jerry's ice creams has reduced the number of calories in "children's ice creams" but not in ordinary ranges; Nestlé has cut the calories in Aero and white KitKat Chunky bars but not in ordinary milk chocolate KitKats.

Keith Vaz, the Labour chairman of the Commons home affairs select committee, said: "The problem with the Responsibility Deal is that it is all voluntary.

"As far as Coke is concerned it remains the same product it was 10 years ago - there doesn't seem to have been any change at all."

Warning that consumption of sugar was at "crisis levels", Mr Vaz, who has type 2 diabetes, added: "We need targets. Unless we get the target we have no benchmark."

Earlier this month Mr Vaz led a parliamentary debate calling for food labels to include the number of teaspoons in each product. He was backed by Action on Sugar, which claimed the move would help to ensure that firms reduce calories "across the board".

Kawther Hashem, a nutritionist for Action on Sugar said: "Examples such as these show the Responsibility Deal's calorie pledge is completely ineffectual.

"It is time for the Department of Health to introduce sugar reduction targets, to encourage companies to reduce sugar and calorie content of all their products, across the board, creating the much-needed level playing field."

Dr Aseem Malhotra, a cardiologist and Action on Sugar's science director, said the findings suggested food firms was simply paying "lip service" to the government by signing up to the calorie pledge.

"This investigation by the Telegraph has exposed what a farce the responsibility deal really is and further substantiates why we cannot allow the food industry to have a say in health policy," he added.

In total 663 organisations are "partners" in the responsibility deal and made a variety of pledges.

Thirty-seven firms, including Coca Cola, Mars, Nestlé and PepsiCo, signed up to the "calorie reduction" pledge to "support and enable our customers to eat and drink fewer calories through actions such as product/menu reformulation, reviewing portion sizes, education and information, and actions to shift the marketing mix towards lower calorie options."

As part of its commitment to the calorie pledge, Coca Cola has cut the amount of sugar in Glacéau vitaminwater by 30 per cent and the number of overall calories in Sprite by the same amount.

It has also introduced a 250ml can as a "slimline" alternative to the normal 330ml helping. However, the amount of sugar and overall number of calories in a 330ml can of Coca-Cola original remains at 139, including 35g, or nine level teaspoons, of sugar.

The firm said it had no plans to reduce the sugar or overall calorie levels in the drink "as we know our consumers love its great taste".

AG Barr, the Scottish firm which makes Irn Bru, made a pledge under the deal to reduce the average calorific content per 100ml of its drinks by 5 per cent by 2016.

However, original Irn Bru, which contains 107 calories, including 6.5 teaspoons of sugar, in a 250ml serving, has not been changed.

Instead the firm aims to increase sales of "**sugar** free" Irn Bru, which accounts for 32 per cent of total Irn Bru sales. AG Barr has also reduced **sugar** in lesser-known **soft drinks**, including Barr Cola, Tizer and Red Kola, by between 2 per cent and 46 per cent.

Nestlé has reduced the number of calories in Aero and white KitKat Chunky bars by 5 per cent. It has also increased the volume of items with fewer than 110 calories per serving and produced new lower-calorie products. However, it has not reduced the number of calories in milk chocolate KitKat bars and other popular products such as Lions and Rolos.

Unilever, which owns Wall's and Ben & Jerry's, pledged to cut the number of calories in "children's ice creams", which now contain 110 or fewer calories per serving. It is also offering "smaller and lower calorie alternative choices" such as "mini" Magnums. However, ordinary Magnum bars, as well as ice cream tubs from Ben & Jerry's, have not been affected.

A spokesman for Coca-Cola GB said: "We have no plans to change Coca-Cola. We know that people love it and we provide two great-tasting sugar-free, no calorie options in Diet Coke and Coca-Cola Zero, which together comprise more than 40 per cent of the cola we sell in the UK."

A spokesman for AG Barr said: "The Irn-Bru recipe delivers a unique taste and flavour which is recognised and loved by the brand's many loyal consumers, who we know will not accept any change."

A Nestlé spokesman said: "We continue to seek opportunities across our business to improve the nutritional profile of our products without compromising the taste and quality of the products our consumers love."

The firm had "reformulated" its Kit Kat bar to remove the equivalent of 3,800 tons of saturated fat, the spokesman added.

A Unilever spokesman said: "Sugar plays an important role in ice cream, not only for taste but also for texture and structure. However, we have a programme in place that aims to gradually reduce sugar in our products whilst maintaining our high standards of product quality."

An Food and Drink Federation spokesman said: "Britain's food and drink manufacturers recognise the worldwide health burden of obesity and diet-related diseases and have been working alongside government, health organisations, NGOs and other stakeholders to drive change.

"There is no single solution to a complex, multifactorial problem like obesity, which is why food manufacturers are taking a range of actions under the Responsibility Deal calorie reduction pledge to help consumers achieve healthier diets and become more physically active."

A Department of Health spokesman said: "Many manufacturers and retailers are already taking sugar, fat and salt out of their products as a result of our Responsibility Deal, which is based on working collaboratively with industry rather than imposing unrealistic targets."

LOAD-DATE: June 1, 2014

LANGUAGE: ENGLISH

PUBLICATION-TYPE: Newspaper; Web Publication

JOURNAL-CODE: WEBDTNS

Copyright 2014 Telegraph Media Group Limited

The Telegraph

telegraph.co.uk

May 30, 2014 Friday 3:40 PM GMT

The Kitchen Thinker: the smoothie fightback; His smoothies have been compared to fizzy drinks and doughnuts. Now, the founder of Innocent is fighting back

BYLINE: By Bee Wilson

LENGTH: 534 words

Richard Reed does not need my sympathy. The multimillionaire Cambridge graduate, co-founder of Innocent Smoothies(now sold in 16 European countries), is a celebrated businessman, often on television. He lives in Holland Park in west London, goes rock-climbing for fun and works in an office called Fruit Towers.

Yet I do feel a bit sorry for Reed. Until very recently, smoothies and juice were seen as an utterly benign way to make money (even if the halo slipped when Coca-Cola took more than a 90 per cent share in Innocent last year). Reed's initial inspiration came from his mother, a nurse, whose advice when he left his Yorkshire home was, "Promise me you'll have a piece of fruit every day." The company's mantra is "tastes good, does good".

So it must have been a nasty shock when Robert Lustig's polemic, Fat Chance, was published in January 2013, sparking headlines along the lines of SMOOTHIES WORSE FOR KIDS THAN CRACK COCAINE! (I exaggerate. But only slightly.) Lustig's thesis is that sugar is the root cause of the obesity crisis. For Lustig, it doesn't matter whether the sugar takes the form of a **fizzy drink** or a holier-than-thou pomegranate smoothie. Susan Jebb, who advises the Government on obesity, has also spoken against fruit juice, saying that the body can't tell the difference between orange juice and sugary pop.

It's a sign of how rattled Innocent is that it summoned me for a personal meeting at Fruit Towers with Reed, plus the CEO Douglas Lamont and tech adviser Simon Allison. A copy of Fat Chance is sitting prominently on the table ("required reading!" says Allison, a tad nervously). Innocent wants to put its side of the story. "It

feels a bit unfair!" says Lamont, that Innocent doesn't get credit for having "absolutely zero" added sugar. Reed is outraged that people have compared his smoothies to something as unhealthy as a doughnut.

They are fighting back. One of the most startling details in Lustig's book was the assertion that the "shearing action" of a blender destroys the insoluble fibre in the fruit. Innocent commissioned research from Oxford Brookes University, contradicting this. It seems that the "fibrous structure" of fruit is not totally destroyed by being puréed into a smoothie.

Innocent is surely right that some of the anti-juice talk has gone too far. Large numbers of adolescent girls are anaemic, one of the great neglected scandals in our diet. At breakfast, teenage girls might do better to have a small glass of juice - whose vitamin C helps iron absorption - than tea and coffee, which inhibit iron. Juice diluted with sparkling water and a wedge of lime can also be a good prop for those cutting down on **fizzy drinks** or wine.

But we have come a long way since the time - not so long ago - when fruit juice was seen as one of the most righteous things you could put in your body. Even Reed - who is now diversifying into green vegetable juices - freely admits that a piece of fresh fruit is a better option than one of his drinks. He says that "you'll never hear us say, 'Have a smoothie instead of eating a piece of fruit'." In which case, I'll take the fruit.

Follow @StellaMagazine on Twitter

LOAD-DATE: May 31, 2014

LANGUAGE: ENGLISH

PUBLICATION-TYPE: Newspaper; Web Publication

JOURNAL-CODE: WEBDTNS

Copyright 2014 Telegraph Media Group Limited
All Rights Reserved

38 of 215 DOCUMENTS

The INDEPENDENT
Independent.co.uk

May 29, 2014 Thursday 11:17 AM GMT

Obesity levels will put great strain on healthcare systems around the world. Policy-makers need to be well prepared; US food labels are soon to receive their first major makeover in 20 years

BYLINE: Editorial

SECTION: VOICES

LENGTH: 578 words

Obesity is altering the shape of the average human body. With every passing year, the waistline of this mannequin expands - or it would, if somebody sculpted the data into physical form. New research makes

clear the bulge. So great has the surge in obesity been over the past 33 years, a Lancet report reveals, that 2.1 billion people are now classed as overweight. An OECD investigation provides a lens on the shorter term: overall, the world is putting on weight at quite a clip, even though the rate of increase in the past decade slowed in some developed countries, including the UK.

The implications of this for global health are obvious and worrying: fat - as it bulks up the human frame - is linked to a rise in non-communicable diseases, from diabetes, to strokes, and some forms of cancer. And as we get fatter, so too we are living longer - global life expectancy has risen by over a decade since 1970. The combination of these two trends will strain healthcare services worldwide, as more of the population falls chronically ill. By 2025, the UN aims to have halted obesity's rise. If there is to be a chance of success - and the juggernaut will take some slowing - three kinds of regulation will be needed.

First, individuals should be further encouraged to make sensible dietary choices, or self-regulate. Education on the risks of unhealthy eating has grown across the developed world in recent years - notably led, in America, by the First Lady, Michelle Obama. Governments can help to inform citizens by promoting dietary lessons in school, and refining packaging: US food labels are soon to receive their first major makeover in 20 years - making them less obfuscatory - and the UK is in the process of rolling out a consistent "traffic-light" system to warn shoppers of high calorie counts (although the scheme is a voluntary one, and has been side-stepped altogether by 40 per cent of the market). On its own, however, better information will not suffice. For one, poorer communities may find healthy eating incompatible with household budgets (and 61 per cent of the world's obese population now lives in the developing world - evidence of the bond between poverty and calorie-rich, nutrition-poor diets).

The second form of regulation requires voluntary corporate buy-in. With the rise of health consciousness, makers and sellers of food have an economic incentive to show concern for the health of their customers. This can be openly professed, such as in Tesco's recent decision to remove sweets from the checkout. Or it can be subtle. Hi-tech is increasingly being brought to Big Food, with the development of smarter fat substitutes, for example, that allow for less of a compromise between health and taste. Such measures hold promise, and may do more for obesity rates than simply haranguing people to avoid the Golden Arches altogether.

Public health bodies remain sceptical of the food industry's capacity to self-regulate. A 2013 study in The Lancet concluded that there was "no evidence for benefit...concerning public health". Ultimately, the responsibility for combating obesity lies with governments. Since many bear at least part of the cost of healthcare, they have ample reason to act. (In the UK, the bill for obesity-related illnesses reaches £5.1bn a year.) Sterner intervention - up to and including taxes on **sugary drinks** - ought not to be off the menu. Big Food is fighting such change, from New York to Mexico. We hope it loses.

LOAD-DATE: May 29, 2014

LANGUAGE: ENGLISH

PUBLICATION-TYPE: Newspaper; Web Publication

JOURNAL-CODE: WEBI

Copyright 2014 Independent Digital News and Media Limited
All Rights Reserved

The INDEPENDENT

The Independent (London)

May 29, 2014 Thursday
First Edition

Tummy trouble;
Editorials Obesity levels will put great strain on healthcare systems around the world. Policy-makers need to be well prepared

SECTION: NEWS; Pg. 2

LENGTH: 578 words

Obesity is altering the shape of the average human body. With every passing year, the waistline of this mannequin expands - or it would, if somebody sculpted the data into physical form. New research makes clear the bulge. So great has the surge in obesity been over the past 33 years, a Lancet report reveals, that 2.1 billion people are now classed as overweight. An OECD investigation provides a lens on the shorter term: overall, the world is putting on weight at quite a clip, even though the rate of increase in the past decade slowed in some developed countries, including the UK.

The implications of this for global health are obvious and worrying: fat - as it bulks up the human frame - is linked to a rise in non-communicable diseases, from diabetes, to strokes, and some forms of cancer. And as we get fatter, so too we are living longer - global life expectancy has risen by over a decade since 1970. The combination of these two trends will strain healthcare services worldwide, as more of the population falls chronically ill. By 2025, the UN aims to have halted obesity's rise. If there is to be a chance of success - and the juggernaut will take some slowing - three kinds of regulation will be needed.

First, individuals should be further encouraged to make sensible dietary choices, or self-regulate. Education on the risks of unhealthy eating has grown across the developed world in recent years - notably led, in America, by the First Lady, Michelle Obama. Governments can help to inform citizens by promoting dietary lessons in school, and refining packaging: US food labels are soon to receive their first major makeover in 20 years - making them less obfuscatory - and the UK is in the process of rolling out a consistent "traffic-light" system to warn shoppers of high calorie counts (although the scheme is a voluntary one, and has been side-stepped altogether by 40 per cent of the market). On its own, however, better information will not suffice. For one, poorer communities may find healthy eating incompatible with household budgets (and 61 per cent of the world's obese population now lives in the developing world - evidence of the bond between poverty and calorie-rich, nutrition-poor diets).

The second form of regulation requires voluntary corporate buy-in. With the rise of health consciousness, makers and sellers of food have an economic incentive to show concern for the health of their customers. This can be openly professed, such as in Tesco's recent decision to remove sweets from the checkout. Or it can be subtle. Hi-tech is increasingly being brought to Big Food, with the development of smarter fat substitutes, for example, that allow for less of a compromise between health and taste. Such measures hold promise, and may do more for obesity rates than simply haranguing people to avoid the Golden Arches altogether.

Public health bodies remain sceptical of the food industry's capacity to self-regulate. A 2013 study in The Lancet concluded that there was "no evidence for benefit ??? concerning public health". Ultimately, the responsibility for combating obesity lies with governments. Since many bear at least part of the cost of healthcare, they have ample reason to act. (In the UK, the bill for obesity-related illnesses reaches £5.1bn a year.) Sterner intervention - up to and including taxes on **sugary drinks** - ought not to be off the menu. Big Food is fighting such change, from New York to Mexico. We hope it loses.

LOAD-DATE: May 28, 2014

LANGUAGE: ENGLISH

PUBLICATION-TYPE: Newspaper

JOURNAL-CODE: IA

Copyright 2014 Independent Print Ltd
All Rights Reserved

DAILY MAIL (London)

May 28, 2014 Wednesday

PUT HEALTH WARNINGS ON SUGARY DRINKS'

BYLINE: BY JENNY HOPE MEDICAL CORRESPONDENT

LENGTH: 204 words

SUGARY drinks should carry health warnings like cigarettes, according to a public health expert.

Professor Simon Capewell, who has described sugar as the new tobacco', wants the Government to follow the example of California, which is set to introduce drinks labels warning of links to obesity, diabetes and tooth decay.

The University of Liverpool academic said a third of Britain's children, and two-thirds of adults, are now overweight or obese - but halving children's intake of sugar-sweetened drinks could arrest or even reverse current trends.

In a personal view published on bmj.com, the British Medical Journal's website, Professor Capewell said: Many other potentially harmful products already carry effective health warnings ... cigarettes have gone from being socially acceptable to quite unacceptable after warning labels were implemented.'

He said changing drinks' packaging would be an interesting experiment' that may offer an effective new strategy'.

However, Professor Tom Sanders, head of King's College London's diabetes division, said: Sugar is not like tobacco: it is not addictive and does not cause cardiovascular disease and cancer.

A warning label on soft drinks suggests a lack of perspective.'

© Daily Mail

LOAD-DATE: May 27, 2014

LANGUAGE: ENGLISH

PUBLICATION-TYPE: Papers

Copyright 2014 Associated Newspapers Ltd.
All Rights Reserved

The Daily Telegraph
The Daily Telegraph (London)

May 28, 2014 Wednesday
Edition 1;
Scotland

Fizzy drinks 'should carry warnings like cigarettes'

SECTION: NEWS; Pg. 6

LENGTH: 394 words

SUGARY drinks should carry health warnings to make them as socially unacceptable as cigarettes, an expert in public health has suggested.

Prof Simon Capewell, a professor of public health at the University of Liverpool, called for legislation that would require manufacturers to warn consumers about links between fizzy drinks and obesity, diabetes and tooth decay.

Such legislation is under consideration in California and he suggested the Government should follow that example.

Writing in the British Medical Journal, Prof Capewell said a third of children and two thirds of adults are overweight or obese in the UK.

Halving children's consumption of sugar-sweetened beverages could perhaps arrest or even reverse the current increases in obesity, he said.

Prof Capewell highlighted a recent European study which showed that adults who drank more than one can of sugary fizzy drink a day had a 22 per cent higher risk of developing Type 2 diabetes than those who drank less than one can a month.

He said there was public support for health warnings about added sugar as the food stuff was being "progressively demonised".

"Many other potentially harmful products already carry effective health warnings," said Prof Capewell.

"For example, insecticides and other toxic products have long carried labels warning users to take extreme care.

"Similarly, cigarettes have gone from being socially acceptable to quite unacceptable after warning labels were implemented. The effectiveness of tobacco warnings and plain packaging is now accepted by almost everyone not linked to the industry."

He said warning labels for sugary drinks could be particularly effective when combined with proposals such as marketing bans and a duty on the drinks.

Research from the University of Glasgow, published earlier this year, showed people were underestimating sugar levels in drinks which are sometimes perceived to be "healthy" options.

Gavin Partington, the director general of the British Soft Drinks Association, said trying to blame one set of products for the "complex" problem of obesity was "misguided". He said soft drinks have full nutrition labelling, including calorie content, printed on the packaging.

"Manufacturers have been taking steps to reduce the calorie content of their drinks over many years - more than 60 per cent of drinks now contain no added sugar," he said.

LOAD-DATE: May 28, 2014

LANGUAGE: ENGLISH

PUBLICATION-TYPE: Newspaper

JOURNAL-CODE: DTLscot

Copyright 2014 Telegraph Media Group Limited
All Rights Reserved

The Express

May 28, 2014 Wednesday
Edition 1;
National Edition

Fizzy drinks 'should have a big health warning like cigarettes'

BYLINE: Jo Willey

SECTION: NEWS; Pg. 4

LENGTH: 422 words

SUGAR-PACKED fruit juice and **fizzy drinks** should carry health warnings just like cigarettes and poisons, a leading expert has claimed.

The harm caused by calorieladen drinks is not being taken seriously enough even though they are directly fuelling an obesity epidemic, says Professor Simon Capewell.

Experts already warn that fruit juice, seen by many as a healthy option, should be drunk no more than once a day.

Prof Capewell, a public health expert from the University of Liverpool, is calling on the Government to follow the example of legislation under consideration in California.

That proposes warnings to consumers about the contribution of **fizzy drinks** to obesity, diabetes and tooth decay.

Obese Prof Capewell said **sugary drinks** should become as socially unacceptable as cigarettes.

He added: "Cigarettes have gone from being socially acceptable to quite unacceptable because of warning labels.

"The effectiveness of tobacco warnings and plain packaging is now accepted by almost everyone not linked to the industry." In a personal view published online in the British Medical Journal's www.bmj.com, Prof Capewell said a third of children and two-thirds of adults are overweight or obese in Britain.

Halving US and UK children's **sugar**-sweetened **beverage** consumption could arrest or even reverse that trend, he said.

He highlighted a European study showing adults who drank more than one can of sugary **fizzy drinks** a day had a 22 per cent higher risk of developing Type 2 diabetes than those who drank less than a can a month.

Prof Capewell said: "A BBC survey found 60 per cent of adults would support health warnings on food packaging similar to those on cigarettes.

"Around 70 per cent supported banning **sugary drinks** in schools or limiting sugar allowed in certain foods. Almost half would support a tax on **sugary drinks**."

Leading cardiologist Aseem Malhotra said: "**Sugary drinks** represent a particular health hazard. What is especially concerning is that recent scientific studies have confirmed sugar intake is associated with an increased risk of heart disease, high blood pressure and Type 2 diabetes independent of body weight, revealing that we are all vulnerable.

"These chronic diseases contribute to considerable suffering and unsustainable health care costs. It's high time health warnings were placed on these 'mini health time bombs'."

The British Soft Drinks Association said: "Obesity is a far more complex problem than Professor Capewell's simplistic approach implies and trying to blame one set of products is misguided."

LOAD-DATE: May 28, 2014

LANGUAGE: ENGLISH

GRAPHIC: Think before you drink any calorie-laden fizz or fruit juice

PUBLICATION-TYPE: Newspaper

JOURNAL-CODE: DXP

Copyright 2014 Express Newspapers
All Rights Reserved

53 of 215 DOCUMENTS

i-Independent Print Ltd

May 28, 2014
First Edition

Expert wants health warnings on fizzy drinks; DIET

BYLINE: Martha Linden

SECTION: NEWS; Pg. 17

LENGTH: 172 words

Health warnings should be added to **sugary drinks** in an attempt to make them as socially unacceptable as cigarettes, a leading expert has said.

Simon Capewell, a professor of public health at the University of Liverpool, said the Government should follow the example of legislators in California, who have proposed warning consumers about the contribution of **fizzy drinks** to obesity, diabetes and tooth decay.

In a personal view published on the British Medical Journal's website, Prof Capewell said a third of UK children and two-thirds of adults were now overweight or obese.

Halving the amount of sugarsweetened drinks consumed by US and British children could mean a reduction in energy intake of 50 to 100 kilocalories a day, perhaps arresting or even reversing current increases in obesity, he said. He highlighted a recent European study which showed that adults who drank more than one can of sugary carbonated drink per day had a 22 per cent higher risk of developing type 2 diabetes than those who drank under one can a month.

LOAD-DATE: May 27, 2014

LANGUAGE: ENGLISH

PUBLICATION-TYPE: Newspaper

JOURNAL-CODE: III

Copyright 2014 Independent Print Limited
All Rights Reserved

54 of 215 DOCUMENTS

MailOnline

May 28, 2014 Wednesday 9:16 AM GMT

'Put health warnings on sugary drinks': Experts say soft drinks should be treated like cigarettes

BYLINE: JENNY HOPE

SECTION: HEALTH

LENGTH: 778 words

- Professor Simon Capewell urges Government to follow California
- US state considering placing warning labels about links to obesity
- University of Liverpool academic says current policies have no effect

Sugary drinks should be labelled with health warnings to make them as socially unacceptable as cigarettes because of their contribution to Britain's obesity epidemic, says a leading public health doctor.

Professor Simon Capewell of the University of Liverpool caused controversy earlier this year by declaring 'sugar is the new tobacco' and claiming the food industry was focused on 'profit not health'.

Now he is calling on the Government to follow the example of California which is considering placing warning labels on all sweetened drinks about the links to obesity, diabetes and tooth decay.

He said current policies are failing to reverse the obesity epidemic in which sugar is 'increasingly implicated as a specific causal factor'.

And he highlighted a recent European study showing adults who drank more than one can of sugary **fizzy drinks** a day had a 22 per cent higher risk of developing Type 2 diabetes than those who drank less than a can a month.

In a personal view published on bmj.com (must credit as stated), Prof Capewell said a third of children and two-thirds of adults are now overweight or obese in the UK.

Halving US and UK children's **sugar**-sweetened **beverage** consumption could mean a 50 to 100 kcal reduction in energy intake a day, perhaps arresting or even reversing the current increases in obesity, he said.

He said surveys showed 60 per cent of Britons would support warnings on food packaging, which may herald a 'tipping point in public attitudes and political feasibilities.'

The Government's chief medical officer Dame Sally Davies warned in March that the Government may soon have to slap a sugar tax on junk food and **fizzy drinks** to tackle obesity.

Professor Capewell is part of a new US-UK campaign group - Action on Sugar - that says asking firms to make voluntary changes has failed.

He caused controversy in January by declaring: 'Sugar is the new tobacco'.

The typical Briton consumes 12 teaspoons of sugar a day and some adults consume as many as 46.

The maximum intake recommended by the World Health Organisation is ten, although this guideline is likely to be halved.

Prof Capewell sugar was being 'progressively demonised'.

'Many other potentially harmful products already carry effective health warnings. For example, insecticides and other toxic products have long carried labels warning users to take extreme care' he said.

'Similarly, cigarettes have gone from being socially acceptable to quite unacceptable after warning labels were implemented.

'The effectiveness of tobacco warnings and plain packaging is now accepted by almost everyone not linked to the industry' he said.

Prof Capewell said warning labels represented an 'interesting natural experiment' that 'may offer an effective new strategy to complement existing, potentially powerful interventions like marketing bans and **sugary drinks** duties'.

Prof Naveed Sattar, Professor of Metabolic Medicine, BHF Glasgow Cardiovascular Research Centre, University of Glasgow, said there was mounting evidence for **sugary drinks** contributing to excess energy intake, obesity and, in turn, higher diabetes risks.

'Furthermore, as Prof Capewell points out, public support for an obesity warning on these drinks appears strong' he added.

Dr Nita Forouhi, MRC Programme Leader and Public Health Physician at MRC Epidemiology Unit, University of Cambridge, said 'Big problems need bold solutions.

If the California Bill, currently being considered for health warning labels on **sugary drinks**, goes ahead, it will set an important precedent.

'There is a consistent body of evidence for the adverse health effects of **sugary drinks**.'

However, Professor Tom Sanders, Head of Diabetes and Nutritional Sciences Division, School of Medicine, King's College London, said 'Excessive intake of **sugary drinks** contributes to unhealthy weight gain in children.

'But sugar is not like tobacco: it is not addictive and does not cause cardiovascular disease and cancer.

'Oral rehydration solutions, which contain sugar, have prevented millions of deaths.

'The risks to young people's health presented by smoking, alcohol, drugs, unsafe sex, tattooing and body piercing are far greater - a warning label on soft drinks suggests a lack of perspective.'

California is considering a law that would require all cans and bottles of **sugary beverages** to have this warning label: 'Drinking **beverages** with added **sugars** contributes to obesity, diabetes and tooth decay.'

It could be voted on by the state senate next week.

LOAD-DATE: May 28, 2014

LANGUAGE: ENGLISH

PUBLICATION-TYPE: Web Publication

JOURNAL-CODE: WEBDM

Copyright 2014 Associated Newspapers Ltd.
All Rights Reserved

The Times (London)

May 28, 2014 Wednesday
Edition 2;
National Edition

GPs get new weapon to fight obesity: the prescription diet

BYLINE: Oliver Moody

SECTION: NEWS; Pg. 14

LENGTH: 564 words

Millions of overweight and obese people should be offered weight management courses such as Weight Watchers and Slimming World, a government health watchdog has said.

All GPs should be able to commission or recommend slimming programmes that can cost more than £100 per patient and last at least three months, according to new guidance released today by the National Institute for Health and Care Excellence.

NICE is urging people to lose a small fraction of their weight for the sake of their long-term health.

GPs have already been able to arrange slimming courses for obese patients, but the new guidelines are an attempt to standardise the service across England as the country faces one of the worst national weight crises in the developed world.

"The number of people who are overweight or obese in England is rising," said Mike Kelly, director of the centre for public health at NICE. "More than a quarter of adults are now classified as obese and a further 42 per cent of men and a third of women are overweight.

"It not only damages their health but dealing with the long-term consequences of obesity costs the NHS around £5.1 billion each year."

NICE sought to make clear that the weight-loss courses should not be seen as a "magic bullet" that would guarantee long-term results. It identified Rosemary Conlon, a chain of diet and fitness clubs, alongside Slimming World and Weight Watchers on a list of providers suitable for courses lasting more than a year.

In their response to NICE's consultation on the new guidance, Weight Watchers welcomed the move. "The draft guidance holds a wealth of practical pointers to maximise the effectiveness of these types of lifestyle services," s apokesman said.

Alarm bells have been ringing across Europe in recent weeks as pressure grows on governments to act on **sugar** and obesity. The new guidance from NICE coincides with a call from a leading medical academic for **sugary soft drinks** to carry health warnings similar to those imposed on tobacco.

Simon Capewell, professor of public health and policy at the University of Liverpool, claims that halving the amount of sweetened drinks consumed by British children could cut their calorie intake by up to 100 calories a day.

"Warning labels for refined sugars hidden in sweetened drinks and processed foods represent an interesting natural experiment," he writes on the BMJ's website today.

"They may offer an effective new strategy for complementing existing, potentially powerful interventions, such as duties on **sugary drinks** or banning marketing to children."

Plans to force all **sugary soft drinks** manufacturers to label products with warnings are already afoot in California, where state senators could vote as early as today on the bill.

Professor Capewell points to a poll showing that three quarters of Californian voters supported compulsory labelling, while another survey found that 60 per cent of Britons would back a similar move.

"Both UK and US policies are failing to reverse trends in obesity," he writes. "Most obesity treatments offered to people, such as advice or preventative medicines, are weak and poorly sustained. More effective policies are therefore urgently needed." Responding to Professor Capewell's warning, the government said that it was currently satisfied with its policy of encouraging companies to be more forthcoming about the nutritional value of their products.

LOAD-DATE: May 28, 2014

LANGUAGE: ENGLISH

GRAPHIC: Children's taste for **sugary drinks** is a concern

PUBLICATION-TYPE: Newspaper

JOURNAL-CODE: TIM

Copyright 2014 Times Newspapers Limited
All Rights Reserved

DAILY MAIL (London)

May 27, 2014 Tuesday

HEALTH CHIEFS SLAM COCA COLA'S £20M ANTI-OBESITY 'STUNT'

BYLINE: BY SIAN BOYLE

LENGTH: 471 words

COCA-COLA has come under fire over a £20million anti-obesity campaign it is running in 70 parks this summer.

Thousands of families will be urged to take part in free sports sessions such as tennis, rounders and Zumba from Friday as part of the Coca-Cola Zero ParkLives .

But nutrition campaigners claim it is an obscene' attempt to distract attention from the company's part in fuelling Britain's obesity epidemic.

Earlier this month, public health officials warned that children are consuming around 40 per cent more sugar than is recommended.

Public Health England said **soft drinks** were the chief culprit, providing the largest source of **sugar** in the diets of those aged four to 18.

Drinks such as Coca-Cola, with nine teaspoons of sugar per can, accounted for 30 per cent of added sugar intake for those aged 11 to 18, the National Diet and Nutrition Survey found.

Yesterday Dr Aseem Malhotra, cardiologist and science director for campaign group Action on Sugar, branded Coca-Cola's parks scheme a really disingenuous stunt'.

Dr Malhotra said: They are trying to deflect attention from their own part in creating an obesity epidemic, which has been fuelled almost entirely by rising calorie consumption.'

He added that obscene' attempts by firms to associate themselves with active lifestyles could encourage the public to consume more unhealthy products, wrongly assuming that small amounts of activity would be enough to balance it out.

Wayne and Coleen Rooney helped to launch Coca-Cola Zero in 2006. The parks project bearing its name will see activities such as basketball, hula-hooping and t'ai chi in London, Birmingham and Newcastle, with plans to expand it to more cities in the future.

The Coca-Cola Zero ParkLives project is the latest offshoot of the controversial Public Health Responsibility Deal', under which the food and drink industries can effectively regulate themselves.

The Daily Mail revealed in February how the food industry lobby had been granted dozens of meetings with ministers since 2010

, while health campaigners have been kept at arm's length.

Coca-Cola has stated that in accordance with its marketing code, the fitness programme will not be marketed at children under 12. It will offer free sports sessions and coaching for families and young adults.

Jon Woods, general manager of Coca-Cola Great Britain, said: We know that companies get to grow in a market if they stay close to their customers. Obesity is a great concern for our consumers - 61 per cent of adults are overweight and obese, as are 33 per cent of children.

We want to play a more productive role in finding solutions to obesity.

Historically, we would have shied away from this but we are taking a more proactive approach. This is about calories in and calories out and getting the energy balance right.'

s.boyle@dailymail.co.uk

© Daily Mail

LOAD-DATE: May 26, 2014

LANGUAGE: ENGLISH

PUBLICATION-TYPE: Papers

Copyright 2014 Associated Newspapers Ltd.
All Rights Reserved
69 of 215 DOCUMENTS

The Telegraph
telegraph.co.uk

May 27, 2014 Tuesday 5:29 PM GMT

Questor share tip: AG Barr still a core holding; Scottish based fizzy drinks maker enjoys strong start to the year, says Questor

BYLINE: By John Ficenec

SECTION: FINANCE

LENGTH: 620 words

AG Barr639p+16½Questor says HOLD

SOFT drinks group AG Barr said yesterday that it would focus on increasing sales of its brands, which include Irn-Bru, Tizer and Rubicon fruit juices, rather than looking for big acquisitions.

The **fizzy drinks** maker said revenue was up 5.2pc during the first 15 weeks ended May 11; this is a steady performance and only marginally behind sales growth of 5.5pc reported for the final three months ended January. When compared to the wider drinks market, where sales increased by 1.9pc during the same 15-week period, it shows AG Barr is gaining a bigger share of the soft drinks market.

Encouragingly, the company isn't cutting prices or spending heavily on advertising to achieve this. Roger White, chief executive, said that prices had held firm or risen in line with inflation and marketing and advertising spend was in line with expectations. This meant that profit margins at AG Barr had held firm.

That is good news for investors because, as sales increase, then profits should also rise when the profit margin is steady.

Mr White said the company has seen strong sales from its water products from the start of the new financial year; the Strathmore bottled water range has expanded its range by offering twist fruit flavours. Traditional brands, such as Irn-Bru, have also increased their range by moving into the ice cream market.

AG Barr is operating with a strong balance sheet. The company said at the start of the year that it would end January with lower debt levels than expected. In its full-year results, the company reported net debt of just £2.1m and house broker Investec is forecasting that it will be in a net cash position within the next eight months, which creates some interesting scenarios for investors.

When a company becomes debt free and continues to grow profits then cash builds up. This can either be used to expand or returned to shareholders. AG Barr has said it will use a portion of this cash to improve its bottling facilities, which means capital expenditure will increase to about £19m, from £13m last year.

Investec estimates the drinks company will still generate free cash flow of £24m in the coming year, and that covers the full-year dividend payments of about £13m, or 11.8p per share, about 2 times. That leaves plenty of room for the dividend to rise at a rate above inflation during the coming years.

Alternatively, AG Barr could go shopping for more brands. The company is now in a stronger position than it was two years ago when it launched the ambitious, but ultimately ill-fated £1.4bn bid to merge with rival Britvic. The drinks company will have a tough trading period ahead of it. A scorching summer in the UK last year was behind strong sales; even if the fickle British summer obliges with a repeat of last year's warm weather - and you wouldn't bet on it right now - AG Barr would be doing well to hit double-digit sales growth. If we have a washout summer it will make trading all the more challenging. For the long-term investor, whether the sun shines or not is hardly important, but in the short term it does bring into question buying at this price.

Shares in the FTSE 250-listed drinks group have not run away during the past 12 months, having risen 10pc, exactly in line with the wider index. However, they are by no means cheap and trade on 22 times forecast earnings, a premium to the wider soft drinks sector.

While not a buy at these prices, the company remains a solid investment to hold. The attractive points are the strong balance sheet and cash generation that provide the potential for the dividend to accelerate. The gains from here should be steady and the shares remain a hold.

LOAD-DATE: May 28, 2014

LANGUAGE: ENGLISH

PUBLICATION-TYPE: Newspaper; Web Publication

JOURNAL-CODE: WEBDTNS

Copyright 2014 Telegraph Media Group Limited

The Daily Telegraph

The Daily Telegraph (London)

May 26, 2014 Monday
Edition 1;
Scotland

Coca-Cola accused of a public relations stunt as it funds anti-obesity campaign

BYLINE: Laura Donnelly

SECTION: NEWS; Pg. 4

LENGTH: 274 words

COCA-COLA prompted controversy yesterday after it announced plans to fund a £20million anti-obesity fitness drive in parks around the country.

The company said the new fitness initiative would mean thousands of free sessions and coaching for families, in activities such as tennis, basketball, hula-hooping, Zumba and rounders.

But nutrition campaigners immediately accused the company of an "obscene" attempt to divert attention from its own part in Britain's obesity epidemic. Earlier this month, public health officials warned that children and teenagers were consuming about 40 per cent more sugar than recommended.

Public Health England said **soft drinks** and fruit juices were the chief culprits. **Soft drinks** such as Coca-Cola, which has nine teaspoons of **sugar** per can, amounted to 30 per cent of the added **sugar** intake for those aged 11 to 18.

Dr Aseem Malhotra, a cardiologist and science director for campaign group Action on Sugar, said of Coca-Cola's fitness campaign: "They are trying to deflect attention from their own part in creating an obesity epidemic, which has been fuelled almost entirely by rising calorie consumption."

Under Coca-Cola Zero ParkLives, 70 parks in London, Birmingham and Newcastle will offer free activities. The scheme will be marketed by Coca-Cola Zero - one of its zero-calorie products. The company said it was

making major strides to help tackle obesity in Britain, with 40 per cent of its sales now involving "zero calorie" versions.

Jon Woods, the general manager of Coca-Cola Great Britain, said: "We don't think all the concerns about obesity can be laid at our door - we want healthy, happy consumers."

LOAD-DATE: May 26, 2014

LANGUAGE: ENGLISH

PUBLICATION-TYPE: Newspaper

JOURNAL-CODE: DTLscot

Copyright 2014 Telegraph Media Group Limited
All Rights Reserved

The INDEPENDENT

Independent.co.uk

May 26, 2014 Monday 10:11 AM GMT

Coca-Cola accused of "obscene" hypocrisy in £20m 'anti-obesity' drive; The drinks giant has come under fire from health campaigners for its decision to bring free fitness classes to 70 parks in Britain

BYLINE: Tom Payne

SECTION: HOME NEWS

LENGTH: 381 words

Coca-Cola has been accused of using a £20 million anti-obesity drive to distract attention from its contribution to Britain's obesity epidemic.

The drinks giant plans to pour millions into fitness programme called Coca-Cola Zero ParkLives, offering thousands of free sessions and coaching for families across 70 parks in England.

But the announcement immediately attracted criticism from nutrition campaigners who have labelled the scheme "obscene".

Dr Aseem Malhotra, cardiologist and science director for the Action on Sugar campaign group, told The Daily Telegraph: "I think this is a really disingenuous stunt. They are trying to deflect attention from their own part in creating an obesity epidemic, which has been fuelled almost entirely by rising calorie consumption."

Dr Malhotra added the programme was "obscene" because it encouraged such companies to associate themselves with active lifestyles.

Critics have cited warnings from Public Health England that **soft drinks** and fruit juices packed with **sugar** are creating an obesity epidemic, especially among young people.

A report commissioned by the National Diet and Nutrition Survey earlier this month found that those aged between four and 18 months are consuming around 40 per cent more sugar than is recommended.

The findings also suggested that **soft drinks** like Coca-Cola contributed to 30 per cent of **sugar** intake of those aged between 11 and 18.

But the company has insisted that is playing a part in tackling obesity in Britain, arguing that 40 per cent of sales now come from 'zero calorie' versions of the drink.

Coca-Cola's general manager Jon Woods stressed the company was refusing to shy away from obesity and refused to accept blame for the epidemic.

He said: "We have set out in the last two years that we want to play a more productive role in finding solutions to obesity; historically we would have shied away from this but we are taking a more proactive approach; this is about calories in and calories out and getting the energy balance right."

Mr Woods added that the project's aim was to encourage young people who wouldn't normally take part in sport to be more active.

The new Coca-Cola programme is to be overseen by health quango UK Active, and has received the backing of former Olympic athlete, Sebastian Coe.

LOAD-DATE: July 15, 2014

LANGUAGE: ENGLISH

PUBLICATION-TYPE: Newspaper; Web Publication

JOURNAL-CODE: WEBI

Copyright 2014 Independent Digital News and Media Limited
All Rights Reserved

MailOnline

MailOnline

May 26, 2014 Monday 10:23 PM GMT

Critics slam Coca-Cola's £20m anti-obesity 'stunt': Experts say firm's plan to offer families free sports sessions is attempt to distract attention from role in health crisis

BYLINE: SIAN BOYLE

SECTION: NEWS

LENGTH: 513 words

- Coca-Cola Zero ParkLives project will offer free sessions from Friday
- But one authority on nutrition dubs it a 'really disingenuous stunt'
- Project is offshoot of self-regulatory 'Public Health Responsibility Deal'
- Food industry lobby has enjoyed preferential access to Ministers

Coca-Cola has come under fire over a £20million anti-obesity campaign it is running in 70 parks this summer.

Thousands of families will be urged to take part in free sports sessions such as tennis, rounders and Zumba from Friday as part of the Coca-Cola Zero ParkLives.

But nutrition campaigners claim it is an 'obscene' attempt to distract attention from the company's part in fuelling Britain's obesity epidemic.

Earlier this month, public health officials warned that children are consuming around 40 per cent more sugar than is recommended.

Public Health England said **soft drinks** were the chief culprit, providing the largest source of **sugar** in the diets of those aged four to 18.

Drinks such as Coca-Cola, with nine teaspoons of sugar per can, accounted for 30 per cent of added sugar intake for those aged 11 to 18, the National Diet and Nutrition Survey found.

Yesterday Dr Aseem Malhotra, cardiologist and science director for campaign group Action on Sugar, branded Coca-Cola's parks scheme 'a really disingenuous stunt'.

Dr Malhotra said: 'They are trying to deflect attention from their own part in creating an obesity epidemic, which has been fuelled almost entirely by rising calorie consumption.'

He added that 'obscene' attempts by firms to associate themselves with active lifestyles could encourage the public to consume more unhealthy products, wrongly assuming that small amounts of activity would be enough to balance it out.

Wayne and Coleen Rooney helped to launch Coca-Cola Zero in 2006. The parks project bearing its name will see activities such as basketball, hula-hooping and t'ai chi in London, Birmingham and Newcastle, with plans to expand it to more cities in the future.

The Coca-Cola Zero ParkLives project is the latest offshoot of the controversial 'Public Health Responsibility Deal', under which the food and drink industries can effectively regulate themselves.

The Daily Mail revealed in February how the food industry lobby had been granted dozens of meetings with ministers since 2010, while health campaigners have been kept at arm's length.

Coca-Cola has stated that in accordance with its marketing code, the fitness programme will not be marketed at children under 12. It will offer free sports sessions and coaching for families and young adults.

Jon Woods, general manager of Coca-Cola Great Britain, said: 'We know that companies get to grow in a market if they stay close to their customers. Obesity is a great concern for our consumers - 61 per cent of adults are overweight and obese, as are 33 per cent of children.

'We want to play a more productive role in finding solutions to obesity.

'Historically, we would have shied away from this but we are taking a more proactive approach. This is about calories in and calories out and getting the energy balance right.'

LOAD-DATE: May 26, 2014

LANGUAGE: ENGLISH

PUBLICATION-TYPE: Web Publication

JOURNAL-CODE: WEBDM

Copyright 2014 Associated Newspapers Ltd.
All Rights Reserved

The Daily Telegraph (London)

May 22, 2014 Thursday
Edition 1;
National Edition

Fruit Shoot deal adds more juice to Britvic's expansion into US market; Questor

BYLINE: John Ficenec

SECTION: BUSINESS; Pg. 6

LENGTH: 604 words

Britvic

749p +54½.

Questor says HOLD

BRITVIC has expanded its US ambitions with a nationwide distribution deal for its Fruit Shoot children's soft drinks.

The maker of Robinsons juice yesterday said it had signed an agreement with PepsiCo Americas Beverages to sell drinks in all US mainland states, up from 41 states it could access previously. The news, along with the announcement of first-half profits rising by more than a fifth, sent shares up 7pc.

The deal wraps up a strong six months for Britvic, during which sales of **fizzy drinks** such as Tango, Pepsi and 7UP sent group pre-tax profits bubbling 20.8pc higher to £45.3m. Revenue was up by 4.9pc to £670.7m for the 28 weeks ended April 13, and investors were rewarded with a 13pc increase in the interim dividend to 6.1p, with the shares going ex-dividend on May 28 and payable on July 11.

Simon Litherland, chief executive, said the company remains on track to achieve £30m of annual cost savings by 2016.

He said: "Whilst we anticipate that the consumer environment is likely to remain challenging across our core markets, we remain confident of delivering earnings before interest and tax in the range of £148m to £156m for the full year."

Investec expects that will translate into full year pre-tax profits of £123.7m, giving 39.5p in earnings per share.

Underneath the increase in half-year profits there are encouraging signs of strength in the retail market as Britvic is making price increases stick. The strongest performance came from the **fizzy drinks** division that increased sales by 35.8m litres to 616.7m, and increased the average selling price from 45.9p to 46.1p. These factors combined increased gross profits in the **fizzy drinks** division by £4.6m, to £104.8m, on revenues up 6.8pc to £284.6m.

Still drinks reported a more mixed picture. Sales of products such as Robinsons fruit squash and J20 were down by 3.3m litres to 189.6m, but Britvic managed to increase average prices from 84.9p, to 88.3p. This ensured the unit's gross profits edged ahead by £1.5m, to £82.9m, on revenue up 2.3pc to £167.4m.

Remember these gross profits do not account for costs such as marketing spend and packaging the drinks.

The strong start marks a remarkable turnaround for Britvic. The FTSE 250 company was forced to recall Fruit Shoot in 2012 because of faulty caps. The recall sent shares crashing to lows of 260p.

The slump in the share price left the company open to a reverse takeover bid from rival AG Barr, the maker of Irn-Bru.

The deal did complete and Britvic's shares have not looked back since.

The merger attempt marked a nadir in Britvic's fortunes. The drinks group has fought back under former Diageo executive Simon Litherland, who joined as chief executive in February last year.

Britvic is moving into the most crucial period of sales for the year. The company enjoyed one of the best British summers in the last decade last year, with trading further boosted by uninterrupted sales of Fruit Shoot. This means sales will have to be strong to beat last year.

The shares are trading on 16.7 times 2014 forecast earnings, falling to 14.6 times next year. Britvic has nearly completed eight months of its financial year and management are confident the company can reach market expectations.

The strong pricing hints that Britvic isn't having to strongly market or discount products to reach sales targets and this bodes well for summer. The company remains at risk from increased regulation such as a sugar tax or a washout summer. However, these are impossible to predict, and Britvic has proven its long-term quality and the shares remain a solid hold.

LOAD-DATE: May 22, 2014

LANGUAGE: ENGLISH

PUBLICATION-TYPE: Newspaper

JOURNAL-CODE: DTL

Copyright 2014 Telegraph Media Group Limited
All Rights Reserved

86 of 215 DOCUMENTS

FINANCIAL TIMES

Financial Times (London, England)

May 22, 2014 Thursday
London Edition 1

Tesco removes sweets from all its store checkouts; Supermarkets

BYLINE: Duncan Robinson

SECTION: NATIONAL NEWS; Pg. 4

LENGTH: 485 words

HIGHLIGHT: The company is under pressure from shareholders to turn round its business, writes Duncan Robinson

Tesco has become the first of the "big four" supermarkets to ban sweets and chocolates from checkouts in all of its stores as health campaigners and regulators crack down on sugary food.

The UK's biggest grocer will stop selling confectionery next to the tills from December as it tries to nudge its customers into healthier eating .

Most supermarkets have already removed goods rich in sugar and carbohydrates from the checkout areas in larger stores, but have kept them on display in smaller convenience outlets.

Scientists and public health authorities have put pressure on food and drink companies to reduce the amount of added sugar in products - which may boost sales but is also thought to increase risks of cancer, diabetes and heart disease.

Last year, the Department of Health called on supermarkets to stop encouraging impulse purchases of unhealthy food.

Almost two-thirds of Tesco's shoppers said that removing sweets and chocolates from checkouts would encourage them to eat more healthily. Meanwhile 67 per cent said taking chocolate away from tills would help them resist their children's pester power.

"If you've made it to the checkout in one piece it can be really frustrating to then be faced with an unhealthy array of sweets designed to tempt your child," said Katie O'Donovan, head of communications at Mumsnet, the popular parenting messaging board for mothers.

Tesco has made several attempts to improve its reputation including a focus on reducing waste, as well as improving "traceability" of its supply chain after the horsemeat scare last year.

The emphasis on good corporate deeds comes as the company comes under pressure from shareholders to turn round its struggling UK business, which has been losing market share to cheaper no-frills rivals such as Aldi and Lidl.

"It's a nice PR piece but there is a lot more that retailers could do," said Steven Dresser of Grocery Insight, a retail consultancy.

Historically, fruit and veg have been much more expensive at large supermarkets such as Tesco, although the gap between the "big four" grocers and the discounters is decreasing, adds Mr Dresser.

Sugar has become the latest target of health campaigners as waistlines across the globe have expanded. Food manufacturers have rushed to cut sugar from many of their products as scientists have cranked up the rhetoric, in some cases labelling sugar "the new tobacco".

Nestlé, the world's biggest food group by sales, says it has reduced the sugar in its products by a third, while Mars has reduced the size of its Snickers and Mars bars to get them below 250 calories. Tesco is doing likewise for its own-brand products.

"We've already removed billions of calories from our **soft drinks**, sandwiches and ready-meal ranges by changing the recipes to reduce their **sugar**, salt and fat content," said Philip Clarke, Tesco chief executive. "And we will continue to look for opportunities to take out more."

LOAD-DATE: May 21, 2014

LANGUAGE: ENGLISH

PUBLICATION-TYPE: Newspaper

Copyright 2014 The Financial Times Ltd.
All Rights Reserved
Please do not cut and paste FT articles and redistribute by email or post to the web.

theguardian

The Guardian - Final Edition

May 22, 2014 Thursday

Tesco bans sweets from checkouts in all stores: Two-thirds of customers back move, survey shows: Health and parent groups welcome the decision

BYLINE: Rebecca Smithers, Consumer affairs correspondent

SECTION: GUARDIAN HOME PAGES; Pg. 7

LENGTH: 443 words

The UK's largest retailer, Tesco, is to ban sweets and chocolates from its checkouts after a survey of customers showed overwhelming support for the move.

Research for Tesco found that 65% of shoppers wanted confectionery removed from checkouts to help them make healthier choices when shopping. Even more (67%) said it would help them choose healthier options for their children.

Tesco removed sweets and chocolates from checkouts at its 740 larger Tesco stores 20 years ago, but they will now be removed from checkouts at all stores, including 1,800 Tesco Metro and Express convenience stores in Britain and Ireland. The retailer committed to removing them by the end of December 2014, but has brought forward the move after surveying its customers.

Its chief executive, Philip Clarke, said the decision followed a commitment to make **soft drinks,** sandwiches and ready meals healthier by changing the recipes to reduce their **sugar,** salt and fat content. "We all know how easy it is to be tempted by **sugary** snacks at the checkout, and we want to help our customers lead healthier lives," Clarke said.

Tesco was the first British supermarket to remove sweets and chocolates from checkouts at larger stores, in 1994. The retailer will now test a variety of healthier products at checkouts before implementing the full change across all stores.

In January, Lidl banned confectionary from the checkout at all 600 of its British stores after surveying parents. It replaced racks of sweets with dried and fresh fruit, oatcakes and juices.

Health campaigners and parents' groups welcomed Tesco's move. Katie O'Donovan of Mumsnet said: "Popping into a shop with a small child in tow can sometimes feel like navigating an assault course. If you've made it to the checkout in one piece it can be really frustrating to then be faced with an unhealthy array of sweets designed to tempt your child. It's really positive to see a supermarket responding to the views of their customers and trying to make life that little bit easier."

Ben Reynolds of the food and health charity Sustain agreed. "Parents will be delighted to hear that they will no longer be pestered for fatty, salty and sugary snacks while queuing at the checkout in their local Tesco. We hope that other supermarkets will now follow Lidl and Tesco's lead, and realise that taking action to improve children's health is not something to fear."

Tesco was among those criticised in research published in January which found that convenience stores run by big supermarket chains involved in the government's responsibility deal on nutrition were exposing children to displays of sweets and snack food at the checkout.

LOAD-DATE: May 22, 2014

LANGUAGE: ENGLISH

PUBLICATION-TYPE: Newspaper

Copyright 2014 Guardian Newspapers Limited
All Rights Reserved

THE TIMES

The Times (London)

May 22, 2014 Thursday
Edition 2;
National Edition

Tesco chooses the healthy option and removes sweets at checkouts

BYLINE: John Simpson

SECTION: NEWS; Pg. 4

LENGTH: 443 words

Sweets and chocolate will be removed from checkouts across all Tesco stores by the end of the year in what the supermarket claims is an industry first.

Britain's largest supermarket chain conducted research that found that 65 per cent of customers said that removing confectionery from checkouts would help them to make healthier choices.

Though other supermarkets have similar policies, if the plans are fully implemented it would put them among the most far-reaching, as they include removing confectionery from areas near to the tills, for example racks of sweets at children's eye level.

Tesco changed its policy to keep sweets away from the areas around checkouts at its larger stores 20 years ago, but the most recent move will apply to all stores in Britain and the Republic of Ireland.

It will test the placement of a variety of healthier products at checkouts before implementing the full change.

A report by Which?, the consumer watchdog, a year ago found that only the Co-operative, Sainsbury's and Tesco had policies of prohibiting sweets and other unhealthy foods from being on display at checkouts.

In January Lidl joined their ranks and went one step farther, making it policy at all 600 of its stores to replace any confectionery on display near checkouts with fresh and dried fruit, oatcakes and juices.

Marks & Spencer removes children's sweets and confectionery from checkouts that have conveyer belts. At the time of the Which? report, Morrisons was reviewing its policy.

Sainsbury's has a policy of no sweets or chocolates at checkouts in supermarkets. This does not apply to smaller convenience stores.

Only Waitrose and Aldi had no policy at the time, though a spokeswoman for the former said last night: "We don't sell single-serve confectionery on checkouts in supermarkets." Katie O'Donovan, head of communications at Mumsnet, the parenting website, said: "If you've made it to the checkout in one piece it can be really frustrating to then be faced with an unhealthy array of sweets designed to tempt your child. It's really positive to see a supermarket responding to the views of customers."

Tam Fry, of the National Obesity Forum, welcomed the move but said: "We will be looking at it very carefully to make sure that it's a real move in the right direction and not just a cheap marketing gimmick."

Philip Clarke, Tesco's chief executive, said: "We all know how easy it is to be tempted by **sugary** snacks at the checkout, and we want to help our customers lead healthier lives. We've already removed billions of calories from our **soft drinks,** sandwiches and ready meal ranges by changing the recipes to reduce their **sugar,** salt and fat content."

LOAD-DATE: May 22, 2014

LANGUAGE: ENGLISH

PUBLICATION-TYPE: Newspaper

JOURNAL-CODE: TIM

Copyright 2014 Times Newspapers Limited
All Rights Reserved

FINANCIAL TIMES

Financial Times (London, England)

May 17, 2014 Saturday
London Edition 1

Appetite for capsules spurs sector to diversify

BYLINE: Shannon Bond in New York and Scheherazade Daneshkhu in London

SECTION: COMPANIES; Pg. 16

LENGTH: 295 words

Hermetically sealed capsules have opened up a huge range of possibilities in the food industry, **write Shannon Bond andScheherazade Daneshkhu** .

Sales of coffee sold in capsule form have trebled in the past five years to $10.8bn, according to Euromonitor, against a rise of 35 per cent for the total coffee market.

Now that success has spurred interest in other drinks and food.

Keurig, which Euromonitor estimates has 85 per cent of the $2.7bn US coffee pod market, is teaming up with Campbell's to sell soup capsules that fit its hot drinks machine.

Nestlé, which dominates the coffee capsule market in Europe with Nespresso, has also used the technology for tea. It rolled out its Special T capsules to Japan in 2013, a year after its Europe launch.

Coca-Cola announced this week it would increase its stake in Keurig to 16 per cent from 10 per cent. Coke invested $1.25bn in February and signed a 10-year deal to put its brands in Keurig's cold drinks maker, which is expected to launch this year.

Rival PepsiCo has a 7 per cent stake in Bevyz Global, a European maker of at-home drinks machines that is also set to launch in the US this year, although it is not clear if Pepsi's brands will be available on the appliance. Keurig also holds a minority stake in Bevyz.

The deal with Coke is in line with Keurig's strategy of offering a vast range of drinks. It has built a dominant position among at-home brewers by brands from Starbucks and Folgers coffee to Snapple iced teas and Ghirardelli cocoa.

The next market for capsules is likely to be baby formula, says Lawrence Hutter of Alvarez & Marsal, the professional services firm.

Nestlé launched its BabyNes system in 2011 using capsules to make bottles of babymilk, but has so far not expanded it beyond Switzerland and France.

LOAD-DATE: May 16, 2014

LANGUAGE: ENGLISH

PUBLICATION-TYPE: Newspaper

Copyright 2014 The Financial Times Ltd.
All Rights Reserved
Please do not cut and paste FT articles and redistribute by email or post to the web.

May 15, 2014 Thursday
Edition 2;
National Edition

Warning: no more than one glass of juice a day

BYLINE: Laura Donnelly

SECTION: NEWS; FRONT PAGE; Pg. 1

LENGTH: 623 words

A SINGLE glass of fruit juice a day is the most anyone should drink, new guidelines say, as a report warns that families are consuming unsafe levels of sugar.

There is rising concern that sugar is one of the greatest threats to health, creating an obesity time bomb and contributing to spiralling levels of diabetes. Health officials issued the warning as a national study found that children and teenagers are consuming around 40 per cent more added sugar than the recommended daily allowance.

Fruit juices and **fizzy drinks** are the chief culprit, providing the largest source of sugar for children aged between four and 18, the National Diet and Nutrition Survey by Public Health England found.

The country's most senior nutritionist yesterday advised limiting children and adults to 150ml of fruit juice per day, and always accompanied by a meal. It is the first time health officials have outlined such a limit.

Dr Alison Tedstone, the chief nutritionist at the body, part of the NHS, said: "The best drinks for school-aged children are water and low-fat milk.

"Fruit juice is also a good choice as it can be included as one of your five portions of fruit and vegetables per day.

"However it should only be drunk once a day and with a meal because it can be high in sugar and can cause tooth decay." Some fruit juices and smoothies contain four times as much sugar as is recommended by the World Health Organisation.

Dr Tedstone said the survey demonstrated the need for a change in habits, particularly for children and teenagers.

It found that every age group exceeded the recommendation that added sugar should be no more than 11 per cent of daily calorie intake.

Among children under 10, added sugar made up an average of 14.7 per cent of their intake, while for those aged 11 to 18 it constituted 15.6 per cent. Among adults, the figure was 12.1 per cent. For boys under 10, fruit juice accounted for 15 per cent of their daily added sugar and other drinks a further 17 per cent.

For girls the same age, fruit juice accounted for 12 per cent and other drinks 16 per cent of added sugar.

As children got older, the proportion from soft drinks including juice rose, to 42 per cent for boys, and 38 per cent for girls.

Cereals and cereal bars were the next biggest contributor.

Health officials said they were particularly concerned about fruit juice, because many people believed it to be healthier than it is.

Nearly two thirds of adults are overweight or obese, as are more than a fifth of children by the time they start primary school. It is forecast that five million people will develop diabetes by 2025, twice the number recorded five years ago.

The national survey found little change in eating habits between 2008 and 2012, with most people failing to meet any of the national guidelines on a healthy diet.

On average, boys ate three portions of fruit and vegetables a day rather than the recommended five, and girls 2.7 portions. Adults under 64 managed an average of 4.1 portions while over-65s averaged 4.6 portions. One recent study suggested people should eat seven or even 10 portions a day.

Kawther Hashem, a nutritionist with Action on **Sugar**, a campaign group, said: "It is highly concerning that many parents are still buying fruit juices, **soft drinks** and cereal products for their children thinking they are choosing healthier products, only to find these items are laden with excess **sugar** and calories."

Catherine Collins, from the British Dietetic Association, said: "I think advice to rein back fruit juice is really sensible - there is no benefit to having more than one glass a day, but if it came to a choice between a second fruit juice or having a can of **fizzy drink**, say, the juice still wins hands down."

LOAD-DATE: May 15, 2014

LANGUAGE: ENGLISH

PUBLICATION-TYPE: Newspaper

JOURNAL-CODE: DTL

Copyright 2014 Telegraph Media Group Limited
All Rights Reserved

May 15, 2014 Thursday
Edition 3;
National Edition

No more than one glass of juice a day, say experts

BYLINE: Louise Sassoon

SECTION: NEWS; Pg. 7

LENGTH: 368 words

CHILDREN and adults should limit fruit juice to just one glass a day after health chiefs warned that families are consuming unsafe levels of sugar.

As many as 40 per cent of children and teenagers have more than the recommended daily allowance of hidden sugars - blamed for soaring rates of diabetes and obesity.

Fruit juices and **fizzy drinks** were flagged as the main sugar source for youngsters aged between four and 18, revealed the National Diet and Nutrition Survey by Public Health England.

Cereals and cereal products were the second major contributor to sugar in children's diets, the study claimed.

Dr Alison Tedstone, chief nutritionist at Public Health England, said the intake of fruit juices needed to be curbed.

She explained: "The data highlights that children are drinking too many **sugary drinks**. The best drinks for school-aged children are water and low fat milk.

"Fruit juice is also a good choice as it can be included as one of your five portions of fruit and vegetables per day.

"However, it should be drunk only once a day and with a meal because it can be high in sugar and can cause tooth decay."

The survey, which tracked the eating habits of 1,000 people over four years from 2008, also highlighted that millions are putting their health at risk by consuming far too much saturated fat and salt and not enough fruit and vegetables.

Just three out of 10 adults have been meeting the "five-a-day" target - and only 10 per cent of boys and seven per cent of girls aged 11 to 18 were shown to be eating the correct portions.

Consumption of oily fish was also well below the recommended 140g portion per week in all age groups - while older adults and the majority of children ate more than the daily maximum 6g salt limit.

And all age groups were shown to exceed the optimum average saturated fat intake of 11 per cent of food energy.

Dr Tedstone added: "The data provides compelling evidence that we all need to make changes to our diet to improve our health.

"It is clear that we need to work together to help people improve their diets."

A spokesman for the Department of Health insisted last night that work was being done "to make sure ingredients are clearly identified" through food labelling.

LOAD-DATE: May 15, 2014

LANGUAGE: ENGLISH

GRAPHIC: Fruit juice contains hidden sugar

PUBLICATION-TYPE: Newspaper

JOURNAL-CODE: DXP

Copyright 2014 Express Newspapers
All Rights Reserved

MailOnline

MailOnline

May 15, 2014 Thursday 8:36 AM GMT

Fruit juice timebomb: Health experts say stick to one glass a day as teenagers' poor diets are blamed for increased diabetes risk

BYLINE: SOPHIE BORLAND

SECTION: HEALTH

LENGTH: 683 words

- Fruit juice should be limited as it contains a lot of sugar, experts warn
- Those aged 11 to 19 are eating 42 per cent more sugar than recommended
- Age group also eating 14 per cent too much fat, risking diabetes and stroke
- Only one third of adults get recommended five-a-day survey reveals
- Medics say government Change4Life advertising is having little impact

The appalling diets of the nation's teenagers have been exposed by a report which shows that many are already putting themselves at risk of diabetes, obesity and heart disease.

And last night health experts warned that fruit juice - seen by many as a healthy option - should be drunk no more than once a day because of its high sugar content.

Girls and boys aged 11 to 19 typically eat 42 per cent too much sugar and 14 per cent too much saturated fat.

Only 10 per cent of teenage boys and 7 per cent of teenage girls manage to get their five portions of fruit and veg a day.

Adults do not fare a great deal better. Only a third get their five-a-day and the diet of the average adult exceeds recommended sugar limits by 10 per cent.

The report, the Government's National Diet and Nutrition Survey, also shows that children aged ten and under typically exceed the recommended daily limit of sugar by 34 per cent.

Their main sources of **sugar** are fruit juice, **soft drinks,** cereal bars, biscuits and cakes.

It reveals that adults are eating half the recommended weekly amount of oily fish - which protects against heart disease, cancer and dementia - while teenagers and children only manage a fifth of this amount.

The survey, which involved 4,000 adults and children between 2008 and 2012, says 48 per cent of men and women have above-normal levels of cholesterol, putting them at higher risk of heart disease and strokes.

Simon Gillespie, chief executive of the British Heart Foundation, said: 'This study paints a clear picture that too many people, especially children, are not eating healthily enough.

'This puts them at greater risk of coronary heart disease, type 2 diabetes and obesity now or in the future. There is no magic bullet to solve this problem. Parents, schools, restaurants, retailers and the food industry all have a role to play.

'But the Government can fire the first shot by implementing a 9pm watershed ban on junk food marketing to stop children being bombarded with advertising about products high in fats, salts and sugars. We also need stringent regulation to protect children from online marketing tactics.'

There is also concern that policies such as the NHS's Change4Life programme are having little effect because only healthy adults and children pay any attention.

The initiative, which has cost taxpayers £75million since its launch in January 2009, consists of television adverts, a website, a helpline and locally-run sports clubs all aimed at curbing the obesity epidemic.

The scheme also produces posters for schools, community clubs, GP surgeries and hospitals urging the public to eat their five- a-day, take regular exercise and cut portion sizes.

Dr Ian Campbell, of the National Obesity Forum, said: 'In spite of a raft of measures designed to encourage us to eat a healthier diet we are, as a nation, failing miserably.

'If we really care about the health of our children we need to take far more decisive action.

'We need to regulate the food industry to make healthy choices easier, more attractive and cheaper.'

Dr Alison Tedstone, the chief nutritionist at Public Health England, the Government agency that released the report, said fruit juice was a good option as one of the recommended five fruit portions a day.

But she warned: 'It should only be drunk once a day and with a meal because it can be high in sugar.'

In March, Dame Sally Davies, the Chief Medical Officer, said the Government may have to introduce a sugar tax to help make the nation's diet more healthy.

Later that month the World Health Organisation urged the public to cut their sugar intake by half to six teaspoons a day.

Yesterday Labour MP Keith Vaz called for food labels to include the numbers of teaspoons of sugar in all products.

LOAD-DATE: May 15, 2014

LANGUAGE: ENGLISH

PUBLICATION-TYPE: Web Publication

JOURNAL-CODE: WEBDM

Copyright 2014 Associated Newspapers Ltd.
All Rights Reserved

The Telegraph

telegraph.co.uk

May 15, 2014 Thursday 8:39 AM GMT

Fruit juice and cereals push children over sugar limits; One third of children have too much sugar - and fruit juice and soft drinks are the main culprits, health offiicals warn

BYLINE: By Laura Donnelly Health Editor

SECTION: NEWS

LENGTH: 621 words

A single glass of fruit juice a day is the most anyone should drink, new guidelines say, as a report warns that families are consuming unsafe levels of sugar.

There is rising concern that sugar is one of the greatest threats to health, creating an obesity time bomb and contributing to spiralling levels of diabetes.

Health officials issued the warning as a national study found that children and teenagers are consuming around 40 per cent more added sugar than the recommended daily allowance.

Fruit juices and **fizzy drinks** are the chief culprit, providing the largest source of sugar for children aged between four and 18, the National Diet and Nutrition Survey by Public Health England found.

The country's most senior nutritionist yesterday advised limiting children and adults to 150ml of fruit juice per day, and always accompanied by a meal. It is the first time health officials have outlined such a limit.

Dr Alison Tedstone, the chief nutritionist at the body, part of the NHS, said: "The best drinks for school-aged children are water and low-fat milk.

"Fruit juice is also a good choice as it can be included as one of your five portions of fruit and vegetables per day.

"However it should only be drunk once a day and with a meal because it can be high in sugar and can cause tooth decay." Some fruit juices and smoothies contain four times as much sugar as is recommended by the World Health Organisation.

Dr Tedstone said the survey demonstrated the need for a change in habits, particularly for children and teenagers.

It found that every age group exceeded the recommendation that added sugar should be no more than 11 per cent of daily calorie intake.

Among children under 10, added sugar made up an average of 14.7 per cent of their intake, while for those aged 11 to 18 it constituted 15.6 per cent. Among adults, the figure was 12.1 per cent. For boys under 10, fruit juice accounted for 15 per cent of their daily added sugar and other drinks a further 17 per cent.

For girls the same age, fruit juice accounted for 12 per cent and other drinks 16 per cent of added sugar.

As children got older, the proportion from soft drinks including juice rose, to 42 per cent for boys, and 38 per cent for girls.

Cereals and cereal bars were the next biggest contributor.

Health officials said they were particularly concerned about fruit juice, because many people believed it to be healthier than it is.

Nearly two thirds of adults are overweight or obese, as are more than a fifth of children by the time they start primary school. It is forecast that five million people will develop diabetes by 2025, twice the number recorded five years ago.

The national survey found little change in eating habits between 2008 and 2012, with most people failing to meet any of the national guidelines on a healthy diet.

On average, boys ate three portions of fruit and vegetables a day rather than the recommended five, and girls 2.7 portions. Adults under 64 managed an average of 4.1 portions while over-65s averaged 4.6 portions. One recent study suggested people should eat seven or even 10 portions a day.

Kawther Hashem, a nutritionist with Action on **Sugar**, a campaign group, said: "It is highly concerning that many parents are still buying fruit juices, **soft drinks** and cereal products for their children thinking they are choosing healthier products, only to find these items are laden with excess **sugar** and calories."

Catherine Collins, from the British Dietetic Association, said: "I think advice to rein back fruit juice is really sensible - there is no benefit to having more than one glass a day, but if it came to a choice between a second fruit juice or having a can of **fizzy drink**, say, the juice still wins hands down."

LOAD-DATE: May 15, 2014

LANGUAGE: ENGLISH

PUBLICATION-TYPE: Newspaper; Web Publication

JOURNAL-CODE: WEBDTNS

Copyright 2014 Telegraph Media Group Limited
All Rights Reserved

theguardian
Guardian.com.

May 14, 2014 Wednesday

Too much sugar, salt and fat: healthy eating still eluding many Britons

BYLINE: Sarah Boseleytheguardian.com

LENGTH: 620 words

ABSTRACT

Public Health England highlights sugary teen diet and national struggle with fruit and vegetables revealed in nutrition survey

FULL TEXT

The UK population is still eating far too much sugar, fat and salt, with people falling short of the five-a-day fruit and vegetable portions that have been recommended by health experts.

Public Health England, releasing data for all of the UK, said it was clear that a lot more needed to be done to improve the British diet.

"The data released today provides compelling evidence that we all need to make changes to our diet to improve our health, especially for teenagers," said Alison Tedstone, chief nutritionist at PHE.

The sugar load of children and young people was particularly high, she said.

The UK guidance is that **sugar** should not exceed 11% of our total energy intake, but for children aged four to 10, it is 14.7%, rising to 15.6% for 11- to 18-year-olds. A third of that comes from **sugary soft drinks** and fruit juice.

The high sugar levels will concern those who believe sugar is the biggest contributor to the obesity problem. The World Health Organisation has recently urged countries to reduce sugar consumption to 10% of total energy, with an ambition to bring it down to 5%. The UK's recommended limit is 11%, a figure that does not include sugars from milk products.

The data comes from the latest edition of the National Diet and Nutrition Survey. Every year a 1,000-strong sample representative of the population of the entire UK is surveyed. The latest publication incorporates findings from 2008/9 to 2011/12.

The problem with diet surveys is that many people either forget what they have eaten or tailor their answers. The average calorie intake recorded by men in the survey was 2,111 calories, while women said they ate an average of 1,613 calories a day. That would suggest they were eating less than they needed.

But, notes the report, tests have shown evidence of under-reporting; people do not reveal everything they have eaten.

Cereals and cereal products contributed more calories to the daily diet than anything else, followed by meat, then dairy products.

The UK recommends a saturated fat limit of 11% of all food energy, but every age group eats more than that. Mean saturated fat consumption in men aged 19 to 64 was 12.6%. The saturated fat tends to come mostly from dairy, cereal and meat products, although in younger children it is mostly from dairy items.

Trans-fat levels are now low, following campaigns to expose the food products that contain them and put pressure on the industry to get rid of them. They amount to 0.6-0.7% of energy intake, well below the recommended 2% maximum. Salt intakes were above the guidance of 6g a day, at 7.2g for adults.

Everybody was judged to be failing to get the recommended fruit and vegetable portions, the survey showed. Adults up to the age of 65 were found to be eating just over four portions a day, and older adults 4.6 portions.

Teenage girls did worst, eating an average of 2.7 portions a day, while boys ate three. In the 11-18 age group, 10% of boys and 7% of girls managed five-a day, while 30% of adults and 41% of older adults achieved that level.

"Eating a healthy, balanced, diet that is high in fruit, vegetables and fibre and low in saturated fat, sugar and salt, alongside being more active, will help you to maintain a healthy weight and lower your risk of developing heart disease, type 2 diabetes and some cancers," said Tedstone. "The findings, from the four years covered by the survey, confirm that eating habits do not change quickly. It is clear that we all need to work together to help people improve their diets. This data will help PHE to target its work in the most effective way."

LOAD-DATE: May 14, 2014

LANGUAGE: ENGLISH

PUBLICATION-TYPE: Newspaper

Copyright 2014 Guardian News and Media Limited or its affiliated companies
All Rights Reserved
Registered in England and Wales. No. 908396
Registered office: PO Box 68164, Kings Place, 90 York Way, London N1P 2AP

The Observer (England)

May 11, 2014

Sugar, not fat, is the enemy, says film challenging obesity myths: Acclaimed documentary's science expert calls for health warnings on soft drink cans

BYLINE: Edward Helmore New York

SECTION: OBSERVER HOME NEWS PAGES; Pg. 23

LENGTH: 808 words

First came An Inconvenient Truth. Then Fast Food Nation. Then Blackfish. Each showed the power of critically acclaimed, successful documentaries to alter perceptions about controversial issues ranging from global warming to mistreatment of animals in captivity and the behaviour of food industry giants.

Now comes Fed Up, a film that looks at the global problem of surging human obesity rates and obesity-related diseases. The film, produced by Laurie David, former wife of Seinfeld creator Larry David, and narrated by TV journalist Katie Couric, seeks to challenge decades of misconception and food industry-

sponsored misinformation about diet and exercise, good and bad calories, fat genes and lifestyle. When it comes to obesity, fat may not be our friend but it's not the enemy that sugar is, says the film's scientific consultant Robert Lustig, a neuroendocrinologist, author and president of the Institute for Responsible Nutrition. It is a view that is gathering support from doctors.

A US government study recently found that 17% of children and young people aged between two and 19 are considered obese. Another predicted that today's American children will lead shorter lives than their parents. Laurie David, who made the climate change film An Inconvenient Truth, calls that statistic "sobering and tragic".

According to Lustig, however, neither obesity nor fat is the issue. "The food industry wants you to focus on three falsehoods that keep it from facing issues of culpability. One, it's about obesity. Two, a calorie is a calorie. Three, it's about personal responsibility."

If obesity was the issue, metabolic illnesses that typically show up in the obese would not be showing up at rates found in the normal-weight population. More than half the populations of the US and UK are experiencing effects normally associated with obesity. If more than half the population has problems, it can't be a behaviour issue. It must be an exposure problem. And that exposure is to sugar."

The film claims that fast-food chains and the makers of processed foods have added more sugar to "low fat" foods to make them more palatable.

The sugar surge adds up to a problem not only for low-income groups that are often associated with diet-related health issues, but for all levels of society, say the film-makers. The film says big business is poisoning us with food marketed under the guise of health benefits.

Early-onset diabetes, a condition associated with exposure to cane sugar and corn syrup, was virtually unknown a few years ago. If current rates continue, one in three Americans will have diabetes by 2050. "Obesity costs very little and is not dangerous in and of itself," says Lustig, who works with the UK's Action on Sugar campaign. "But diabetes costs a whole lot in terms of social evolution, decreased productivity, medical and pharmaceutical costs, and death."

But while the fight against obesity is championed by first lady Michelle Obama, efforts to curb the sugar industry have largely failed. In 2003 the Bush administration threatened to withhold US funding to the World Health Organisation if it published nutritional guidelines advocating that no more than 10% of calories in a daily diet should come from sugar. Moreover, Washington has sweetened the profits of the manufacturers of corn-based sweeteners by awarding billions of dollars in trade subsidies.

The film-makers say it is not in the interest of food, **beverage** or pharmaceutical companies to reduce **sugar** content. "It's too profitable," says Lustig. The pharmaceutical industry talks of diabetes treatment, not prevention. "The food industry makes a disease and the pharmaceutical industry treats it. They make out like bandits while the rest of us are being taken to the cleaners."

Lustig says laws are needed. The model for regulation is alcohol since alcohol metabolises as sugar and produces many of the same chronic diseases while fat metabolises differently.

But Lustig believes that education or government guidelines alone are inadequate to address substance abuse problems. "What's necessary is to limit availability to reduce consumption and reduce alcohol-related health problems," he says.

Proposals include putting health warnings on **soft-drinks** cans, giving equal advertising time to marketing fresh fruit and vegetables and voluntary agreements to reduce **sugar** content.

Lustig says: "If the food industry continues to obfuscate, we will never solve this and by 2026 we will not have healthcare because we will be broke. Food producers are going to have to be forced. There's only one group that can force them, and that's the government. There's one group that can force the government, and that's the people."

Captions:

Research found 17% of Americans under 19 are obese. Photograph by Pat Doyle/Corbis

The TV journalist Katie Couric narrates the film Fed Up.

LOAD-DATE: May 10, 2014

LANGUAGE: ENGLISH

PUBLICATION-TYPE: Newspaper

Copyright 2014 Guardian Newspapers Limited
All Rights Reserved

Daily Mirror

May 6, 2014 Tuesday
Edition 1;
National Edition

Sugar can damage your health;
60% say smoking-style warnings should be on all sweets and cakes

BYLINE: JAMES LYONS

SECTION: NEWS; Pg. 28

LENGTH: 202 words

MOST Britons want cigarette-style warnings on sweets, drinks and treats alerting us to sugar's health risks.

The warnings won 59% support in a poll while 45% believed sugar should be taxed in a bid to improve the nation's health.

Extending a ban on sweet **fizzy drinks** to all schools was backed by 66% of the 1,000 people quizzed in the BBC poll.

There were also calls for limits on sugar in some items, bans on supermarket promotions on unhealthy food and free adult cookery classes to improve diets.

Eating too much sugar is linked to type 2 diabetes, tooth decay and obesity but Dr Julian Cooper, of food giant AB Sugar, said: "It's quite simplistic just to demonise one ingredient to the exclusion of all others.

"We would say we're probably consuming too many calories and probably doing too little exercise and activity. There is probably an over consumption of all calories." But Prof Graham MacGregor, of pressure group Action on Sugar, said: "Sugar is an unnecessary source of calories. There's a huge amount hidden in our food.

"There is an easy way to cut back, as has happened with salt. If they do not gradually reduce it we need to tax it so it becomes too expense for them to use."

james.lyons@mirror.co.uk

LOAD-DATE: May 6, 2014

LANGUAGE: ENGLISH

GRAPHIC: CAKE HOLE Sweet treats are now under fire

PUBLICATION-TYPE: Newspaper

JOURNAL-CODE: DMR

Copyright 2014 MGN Ltd.
All Rights Reserved

i-Independent Print Ltd

May 5, 2014
First Edition

Half of Britons say sugary drinks should cost more; HEALTH

BYLINE: Peter Apps

SECTION: NEWS; Pg. 6

LENGTH: 176 words

A government tax on **sugary drinks** would be supported by 45 per cent of people, a survey has revealed.

A poll of 1,000 adults, carried out by BBC Radio 5 Live, showed nearly half the respondents would back the levy on soft drinks.

The survey also saw 73 per cent voice support for fixed limits on the amount of sugar allowed in certain foods. Two thirds would support a ban on **sugary drinks** in schools.

The research, to be debated on the Richard Bacon programme at 2pm today, also revealed 42 per cent would support an NHS surcharge for people with serious health problems as a result of a high-sugar diet. A spokesperson from the Department of Health said it was keeping all evidence under review.

Graham MacGregor, a professor of cardiovascular medicine at St Barts London Hospital and chairman of the campaign group Action on Sugar, said reducing the content of sugar in foods would be a "brilliant public health policy".

In 2012 the "pasty tax", which saw VAT on hot takeaway food raised to 20 per cent, led to widespread opposition from the catering industry.

LOAD-DATE: May 4, 2014

LANGUAGE: ENGLISH

PUBLICATION-TYPE: Newspaper

JOURNAL-CODE: III

Copyright 2014 Independent Print Limited
All Rights Reserved

MailOnline

May 5, 2014 Monday 1:05 PM GMT

Two in three Brits want school ban on fizzy drinks amid growing public concern over child obesity epidemic

BYLINE: TOM MCTAGUE, MAIL ONLINE DEPUTY POLITICAL EDITOR

SECTION: NEWS

LENGTH: 664 words

- Public support for classroom crackdown on **sugary drinks** revealed in poll
- Three quarters want to see a cap on the amount of sugar in food
- Labour claim government reforms to tackle child obesity don't go far enough
- But sugar industry claims it is being unfairly targeted

Fizzy drinks should be banned in all schools, an overwhelming majority of the public believes.

Two thirds of Brits want Coca Cola and other **sugary drinks** barred by headteachers - including those in academies and free schools.

Four out of 10 favour a **fizzy drinks** tax and six in 10 say cigarette-style warnings on junk food would encourage them to eat more healthily.

The Populus poll findings for the BBC come after it emerged Labour were looking at secret plans to crack down on drinking, smoking and junk food.

Leaked documents revealed that Ed Miliband is considering new laws to force people to live healthier lives if he wins next year's General Election. They include banning cheap beer and wine.

New laws to curb the amount of sugar, fat and salt in food aimed at children are also under consideration by the Labour leader.

Today's BBC 5Live poll, suggesting a majority of Brits support draconian measures to make people healthier, will be welcomed on by Labour as evidence that their 'nanny state' proposals have public backing.

The Coalition has already taken a hard line on sugary foods and drinks in schools, but Labour say they have not gone far enough.

Under new government rules, the sale of **fizzy drinks**, crisps and sweets in state schools is banned. But children can bring junk food into schools in packed lunches if head teachers are happy to let them.

Ordinary state schools have also been ordered to 'remove drinks which have no nutritional value and can cause tooth decay' and should 'encourage children to drink water or drinks such as milk and fruit or vegetable juices, which provide important nutrients like calcium, vitamin C and carotenoids'.

These food standards do not apply to academies or free schools - which now make up more than half of state secondary schools - although they will do from later on this year.

The sugar industry claims that is being unfairly targeted.

Dr Julian Cooper, head of food science at AB Sugar, told the BBC: 'It's quite simplistic just to demonise one ingredient to the exclusion of all others.

'We would say that we're probably consuming too many calories and probably doing too little exercise and activity.

'There is probably an over consumption of all calories; not sugar per se.'

He added: 'There are some products that potentially you can reduce sugar, there's no doubt about it.

'So say for example a tomato sauce that you use for making pasta: if you look at the total sugars in that product, over 90% of the sugars come from the tomatoes.

'So, yes you may have a potential to say "let's take the 10% out" - once you go below that figure you're starting to remove the tomatoes and personally I actually like tomatoes in my tomato sauce on my pasta.'

But three quarters of the public support a cap on the amount of sugar in food, according to the poll.

A further 60 per cent say they wanted supermarkets to stop promotions on unhealthy food products.

Dr Alison Tedstone, a nutritionist at Public Health England, said the government needed to consider the public support 'carefully'.

A spokesman for industry body the Food and Drink Federation said: 'The food and drink industry is committed to supporting improvements in public health.

'Whether through labelling, education, reformulating old favourites or creating new healthier options, food and drink manufacturers have invested in a wide range of measures to empower and enable consumers to make food choices appropriate to their lifestyle.

'Having eliminated artificial transfats and substantially reduced levels of saturated fat and salt in a wide range of products, many manufacturers are now looking at how they can help consumers to reduce their calorie intake through a wide range of innovative approaches.'

LOAD-DATE: May 5, 2014

LANGUAGE: ENGLISH

PUBLICATION-TYPE: Web Publication

JOURNAL-CODE: WEBDM

Copyright 2014 Associated Newspapers Ltd.
All Rights Reserved

FINANCIAL TIMES

Financial Times (London, England)

May 2, 2014 Friday
London Edition 1

Marketing sugar less aggressively

SECTION: LETTERS TO THE EDITOR; Pg. 10

LENGTH: 133 words

From Dr Geof Rayner.

Sir, Mark Carr, the chief executive of AB Sugar, is absolutely right in saying that obesity has complex causation , and that consumers should be given "the right tools to make informed decisions about the food and drink they consume" (Letters, April 29), and I especially welcome the fact that he is committed to playing a role in helping them. In which case, could he please make the case to his colleagues in the sugar-using industry to stop marketing their products so aggressively and expensively.

The combined marketing firepower of just two purveyors of **sugary drinks,** Coca-Cola and Pepsi, was $5.7bn in 2013. (The entire budget of the World Health Organisation that year was $3.96bn.)

Geof Rayner,

London SW17, UK

Former member, Dept of Health expert advisory group on obesity

LOAD-DATE: May 1, 2014

LANGUAGE: ENGLISH

PUBLICATION-TYPE: Newspaper

Copyright 2014 The Financial Times Ltd.
All Rights Reserved
Please do not cut and paste FT articles and redistribute by email or post to the web.

Mirror
mirror.co.uk

May 2, 2014 Friday 4:39 PM GMT

Imogen Thomas tries to emulate Cindy Crawford with a sexy coke break Less fizz - more pop: Imogen Thomas tries to emulate Cindy Crawford with a sexy coke break; But former Big Brother star Imogen doesn't quite have Cindy's sex appeal as she sips on her fizzy drink

BYLINE: By Kerry Harden

SECTION: 3AM,CELEBRITY NEWS

LENGTH: 168 words

Swigging on a can of Diet Coke with her dark wavy hair tumbling over her shoulders, glamour model Imogen Thomas brings back one of the sexiest images ever.

Imogen Thomas tries to emulate Cindy Crawford with a sexy coke break Less fizz - more pop: Imogen Thomas tries to emulate Cindy Crawford with a sexy coke break; But former Big Brother star Imogen doesn't quite have Cindy's sex appeal as she sips on her fizzy drink mirror.co.uk May 2, 2014 Friday 4:39 PM GMT

Supermodel Cindy Crawford left us gasping back in 1992 when she appeared in a Pepsi ad plucking a can from a vending machine and taking a sexy slug.

Gorgeous Cindy sizzled in a tight white vest and cut-off denim shorts, flaunting her trademark pout.

Wearing a clinging black vest over her 34E assets, and aviator sunglasses, Welsh hottie Imogen is giving the supermodel a run for her money.

But, despite dropping a dress size recently - from a size 12 to 10 - we're sorry to say Cindy just about pips her to the post in the saucy stakes.

Perhaps Imogen, 31, wouldn't be too bothered, as she has given up modelling for lads' mags now that she's a mum to daughter Ariana, one.

The Pepsi advert helped launch Cindy's career and she went on to recreate it in 2002 for Diet Pepsi.

Have a look at how it should be done in the original video below.

Cindy Crawford pepsi

LOAD-DATE: September 1, 2014

LANGUAGE: ENGLISH

PUBLICATION-TYPE: Newspaper; Web Publication

JOURNAL-CODE: WEBDMI

Copyright 2014 Trinity Mirror, Plc.
All Rights Reserved

The Express

July 1, 2014 Tuesday
Edition 1;
National Edition

Four sugary drinks each week raises breast cancer risk

BYLINE: Cyril Dixon

SECTION: NEWS; Pg. 29

LENGTH: 493 words

WOMEN who have more than three **sugary drinks** a week suffer a greater risk of developing breast cancer, a study suggests.

Academics found a woman's "breast density" - a major risk factor - increased with the quantity of sweetened fruit juice and **fizzy drinks** she downed.

The researchers are now calling for further investigation to see if their results could help cut the hundreds of thousands of breast cancer deaths each year.

Team leader Dr Caroline Diorio said: "We know that worldwide sugar consumption has increased.

"Our findings show what effect that type of diet could have on breast density, one of the strongest indicators for breast cancer risk."

Dr Diorio, of Laval University, in Quebec, Canada, added: "It is important to continue research and begin to inform the public about the adverse effects of sugar consumption."

Intake The findings, published in London-based journal BMC Public Health, were based on a survey of 1,555 women - half of whom had undergone the menopause - who were asked how often they drank a 355ml portion of sweetened or fizzy fruit juice.

Dr Diorio added: "Among all women, those who had a **sugary drink** intake of more than three servings per week had a mean of 29.6 per cent in breast density.

"But those who did not drink this type of drink had a mean of 26.2 per cent in breast density.

"An increase of about three per cent in breast density is not negligible in terms of breast cancer risk."

She went on to speak about the cancer drug tamoxifen.

Dr Diorio said: "By comparison it has been shown that healthy women at high risk of developing breast cancer who received tamoxifen for four-and-a-half years had a reduction of 6.4 per cent in breast density and it has been observed that tamoxifen can reduce the risk of breast cancer by 30-50 per cent in high-risk women. Dr Diorio concluded: "Considering the worldwide increase in sugar consumption and all the health problems it is related to, it is important to continue research.

"The positive impact of reducing sugar intake in the diet might not only be a breast cancer risk reduction but could also have beneficial effects on several other health problems.

"Daily sugar intake reduction can be part of a global public health plan to promote health in general, reduce obesity and reduce the risk of several chronic diseases including breast cancer."

Experts believe it is possible that consuming more sugar increases density in breast tissue by stimulating cell growth.

Women with high breast density have a higher risk of getting cancer because there are more cells to develop the disease. Their tumours are also more likely to be missed because any areas of abnormal tissue are harder to spot during mammogram screening.

But while experts agree high density increases the risk of cancer, some believe the danger is caused by the subject's increased weight.

A Breast Cancer Care charity spokeswoman said it would need to see more research on the subject before commenting in detail.

LOAD-DATE: July 1, 2014

LANGUAGE: ENGLISH

GRAPHIC: Sweetened fruit juice and **fizzy drinks** have been linked by experts to a risk of breast cancer

PUBLICATION-TYPE: Newspaper

JOURNAL-CODE: DXP

Copyright 2014 Express Newspapers
All Rights Reserved

The Sun (England)

July 1, 2014 Tuesday
Edition 1;
Ireland

The healthometer

SECTION: ME;FEATURES; Pg. 7

LENGTH: 219 words

BLOOD TESTS - A new blood test could work out if a woman's lifestyle puts her at risk of developing breast cancer in ten years' time before symptoms even appear.

SHADE - Nine in ten people know UV radiation can increase the risk of skin cancer, yet more than a third of those polled by Nivea get sunburnt - so make sure you're protected.

MIDDLE-AGED SPERM - Donors up to the age of 45 are just as likely to conceive children as those in their 20s, a study by Newcastle Fertility Centre found.

WATER - Parents are being advised by health chiefs to replace **fizzy drinks** with jugs of water at meal times to reduce children's risk of obesity, high blood pressure and diabetes.

SUGAR - Officials are urging people to cut their daily sugar intake from an average of 15 teaspoons to as little as five teaspoons to prevent obesity levels soaring further.

MARRIAGE - Those in unhappy unions have thicker arteries and are more likely to have heart disease, a study in the journal Psychosomatic Medicine, www.found.TV - Watching three hours or more a day could lead you to an early grave. Those who do have bigger waists and higher cholesterol, say Spanish scientists.

ANTIOXIDANTS - Resveratrol, thought to be the anti-ageing ingredient in red wine, could damage the foetal pancreas in unborn babies, a study by US scientists found.

LOAD-DATE: July 1, 2014

LANGUAGE: ENGLISH

PUBLICATION-TYPE: Newspaper

JOURNAL-CODE: SUN

Copyright 2014 NEWS GROUP NEWSPAPERS LTD
All Rights Reserved

MailOnline

MailOnline

June 30, 2014 Monday 3:01 PM GMT

Just three **fizzy drinks** a WEEK increases the risk of breast cancer, study claims

BYLINE: SOPHIE FREEMAN

SECTION: HEALTH

LENGTH: 871 words

- Researchers at Laval University in Quebec found the more **sugary drinks** women consumed the greater the density of their breasts
- Sugar is thought to enhance cell growth therefore increasing breast density
- Dense breasts have less fatty tissue and more gland tissue increasing the number of cells that have the potential to become cancerous
- Study found cancers are more likely to be missed because abnormal tissue is then harder to spot on a mammogram
- Separate study found IVF drugs don't cause breast or gynecological cancers

Women who have more than three **sugary drinks** a week may have an increased risk of developing breast cancer, a study has found.

Researchers found that the more **sugary drinks** consumed by the women in their study, the greater the density of their breasts - a known risk factor for cancer.

Dense breasts have less fatty tissue and more of the gland tissue that makes and drains milk as well as supportive tissue that surrounds the gland.

Women with dense breasts have a higher risk of developing cancer because there are more cells that can become cancerous.

Their cancers are also more likely to be missed because any areas of abnormal tissue are harder to spot during mammogram screening.

Lead author of the study, Dr Caroline Diorio from Laval University in Quebec, said: 'We know that worldwide consumption of sugar has increased and the findings of this study show what effect that type of diet could have on breast density, one of the strongest indicators for breast cancer risk.'

For the study, 1,555 women - half of whom were pre-menopausal and half of whom were postmenopausal - answered a questionnaire about how often they drank sugar-sweetened fruit juice and **fizzy drinks**. A serving was 355ml, about the size of a normal can of **fizzy drink**.

Their breast density was then measured through mammogram screening.

Dr Diorio said: 'Among all women, those who had a **sugary drink** intake of more than three servings per week had a mean of 29.6 per cent in breast density but those who did not drink this type of drink had a mean of 26.2 per cent in breast density.

'An increase of about three per cent in breast density is not negligible in terms of breast cancer risk.

'By comparison it has been shown that healthy women at high risk of developing breast cancer who received (the breast cancer drug) tamoxifen for four-and-a-half years had a reduction of 6.4 per cent in breast density, and it has been observed that tamoxifen can reduce the risk of breast cancer by 30-50 per cent in high-risk women.'

It's thought that sugar can enhance cell growth, and therefore, density in breast tissue.

Dr Diorio concluded: 'Considering the worldwide increase in sugar consumption and all the health problems it is related to, it is important to continue research on this subject and begin to inform the public about the adverse effects of sugar consumption.'

The study was published in the journal BMC Public Health.

BUT... IVF DRUGS DO NOT CAUSE BREAST OR GYNECOLOGICAL CANCERS

Fertility drugs do not trigger cancer in women trying to get pregnant, says new research.

A US study of almost 10,000 women suggests hormonal drugs and other medication do not raise the overall risk of breast, ovarian and womb cancers.

The exception is women who remained childless despite having drugs called gonadotrophins as part of IVF treatment.

But experts believe the extra risk is caused by underlying health problems responsible for the infertility.

Debate has raged about whether IVF drugs may cause cancer because they stimulate hormones linked to the disease, especially breast cancer.

A new study looked back at 9,892 women treated for infertility between 1965 and 1988 at five US centres, and followed them for 30 years for data on cancer.

Altogether 749 breast cancers, 119 womb cancers and 85 ovarian cancers were found.

Using a popular fertility drug called clomiphene was not linked with breast, womb or ovarian cancers.

A small number of women treated with the drug for at least a year had a 69 per cent higher risk of breast cancer, but only 31 women fell into this category.

Experts said current guidelines recommended just three to six months of treatment.

Dr Bert Scoccia from the University of Illinois, Chicago, presented the data today at the Annual Meeting of the European Society of Human Reproduction and Embryology (ESHRE) in Munich.

He said the results were 'generally reassuring'.

There was a higher cancer risk for women treated with newer fertility hormones known as gonadotrophins who remained childless.

But, said Dr Scoccia, only 13 women were in this category and it was likely the increased risk was caused by their severe infertility 'rather than that of drug usage.'

Richard Kennedy general secretary of the International Federation of Fertility Societies, said 'Cancer risks of drugs used to stimulate egg production have been evaluated in a number of national studies and not found to significantly increase the risk, other than that which relates to the underlying risks in this patient group.

'This study does not change the current recommendation to patients that the risks are low but that clomiphene should generally not be used for more than 12 cycles.'

LOAD-DATE: June 30, 2014

LANGUAGE: ENGLISH

PUBLICATION-TYPE: Web Publication

JOURNAL-CODE: WEBDM

Copyright 2014 Associated Newspapers Ltd.
All Rights Reserved

The Sun (England)

June 30, 2014 Monday
Edition 1;
National Edition

Think before you drink;
DIET CLUB

BYLINE: LYNSEY CLARKE

SECTION: FEATURES; Pg. 36

LENGTH: 232 words

BRITAIN'S war on obesity must start by **sugary drinks** being banned at the dinner table, according to the Government's chief obesity adviser.

As well as **fizzy drinks**, juices should be replaced by water according to Professor Susan Jebb. Experts say fruit juices are adding to the rise in obesity rates, type two diabetes and heart disease.

Orange juice, for example, is crammed with almost as many calories as pop, despite the common belief that it is healthy. Diabetes expert Professor Tom Sanders of King's College London, said: "We need to reintroduce the habit of putting water on the table."

Here LYNSEY CLARKE shows the calorie equivalents between food and drinks.

TWO 10oz glasses of orange juice: 130 cals in each, or 260 in total.

Equivalent to: Six McDonald's McNuggets (250 cals). Tuna salad sandwich (251 cals).

Grilled salmon teriyaki (261 cals).

TWO 12oz cans of Coca-Cola: 140 cals each, 280 in total.

Equivalent to: KFC Hot Shot Bites (290 cals).

Vegetable lasagne (280 cals).

Marks & Spencer Macho Chicken Wrap (275 cals).

TWO 12oz banana and mango smoothies: 196 cals each, or 392 in total.

Equivalent to: Nachos, cheese and sour cream (346 cals). Spag bol (344 cals). Subway Tuscan Melt (380 cals).

TWO 250ml glasses Sainsbury's Cloudy Lemonade: 118 cals each, or 236 in total.

Equivalent to: McDonald's hamburger (250 cals). Two poached eggs on toast (221 cals).

Egg salad sandwich (228 cals).

LOAD-DATE: June 30, 2014

LANGUAGE: ENGLISH

PUBLICATION-TYPE: Newspaper

JOURNAL-CODE: SUN

The Telegraph

telegraph.co.uk

June 30, 2014 Monday 11:01 AM GMT

What To Eat Now: fruit juice, the sweet truth; Our weekly columnist, expert nutritionist and author Ian Marber asks: is it time to stop thinking of fruit juice as a healthy option?

BYLINE: By Ian Marber

SECTION: FASHION

LENGTH: 584 words

Fruit is a strange thing. Eating fruit is a healthy choice, or at least that's what most of us have been brought up to think. The best-known health message - get your five-a-day - includes eating fresh fruit, so how could anything that's derived from fruit be something that needs to be limited, or even avoided altogether?

READ - What to Eat Now: some dietary truths

When eaten whole, fruit contains fibre, which has many roles including providing bulk that slows the speed at which it passes through the digestive system. In doing so the inherent fructose, 'fruit sugar', is released at a reduced rate. Fructose is not broken down in the same way that a refined sugar would be as it is passed to the liver for processing. When eating fruit, even sweet fruit, the fibre goes some way to offset fructose, but when fructose is ingested in larger amount the liver increases output of triglycerides, a type of fat found in your blood. In time this contributes to an enlarged liver as well as raised blood cholesterol.

Taking away the fibre, which is essentially what juicing is, simply means that the fructose is concentrated and in a dose that can't be managed without consequence. In society it is common to elevate something that has an inferior alternative, just as the fruit juice has been when compared to the sugar laden-colas and pops. But should fruit juice be seen as a healthy choice, when it is in fact just marginally better than the lesser, sweeter, choices?

When comparing fruit juice to a squash, cordial or **fizzy drink**, fruit juice has the advantage of containing nutrients such as beta-carotene and vitamin C. The latter was considered so important that drinking orange juice when you had a cold was standard practice (largely debunked now). More than that, freshly squeezed orange juice seemed to denote health and vitality. But cordials, squashes and the like don't contain vitamin C, unless it's added. All they have is refined sugar in various forms and so of course fruit juice was a better option. But being slightly better than something we know to be nutritionally lacking isn't good enough as fruit juice delivers significant amount of fructose and as such should be limited.

READ - What To Eat Now: an apple a day

A draft report by the Scientific Advisory Committee on Nutrition (SACN) made available last week suggests that the maximum amount of sugar in our diet should be reduced to five per cent of energy intake, down from 10 per cent. This is in line with recommendations made by the World Health Organisation made in March of this year. But this includes honey and fruit juices and so despite the healthy aura of juice, even the powers that be are recommending that we drink less of it. The report, which can be seen here covers carbohydrates as a group and I can't help thinking that the current focus on sugar and sugars will widen in time as we continue to examine the amount of carbohydrates in our diet.

READ - What To Eat Now: more fluids, now the sun is out

The problem with fruit juice is that it has retained its healthy aura long past the best-before date. By the time the sugars it contains have been ingested and processed, the human body doesn't distinguish whether the results are from fruit juice or a Mars Bar.

LOAD-DATE: June 30, 2014

LANGUAGE: ENGLISH

PUBLICATION-TYPE: Newspaper; Web Publication

JOURNAL-CODE: WEBDTNS

Copyright 2014 Telegraph Media Group Limited
All Rights Reserved

Copyright 2014 NEWS GROUP NEWSPAPERS LTD
All Rights Reserved

THE TIMES
The Times (London)

June 30, 2014 Monday
Edition 1;
National Edition

Experts have been feeding us a big fat myth; A new book shows that the low-fat craze was based on flimsy www.evidence.Be wary of today's advice from the diet police

BYLINE: Matt Ridley

SECTION: FEATURES; OPINION COLUMN; Pg. 25

LENGTH: 1073 words

The diet police are on the prowl: if you hear a knock on the door, hide the **sugar** bowl, the butter dish and the salt. A draft report from the scientific advisory committee on nutrition said last week that we should halve our intake of **sugar**. The campaign group Action on **Sugar** wants "a total ban on advertising of ultra-processed foods that are high in saturated fats, **sugar** and salt, and sweetened **soft drinks,** to protect children".

I have been curious about this new demonisation of sugar. I now realise that it conceals a grudging admission that fat is not bad for you after all, but the experts cannot bring themselves to say so. There is a strong possibility that the "diabesity" epidemic has been caused largely by the diet police themselves.

So argues a devastating new book: The Big Fat Surprise by Nina Teicholz, an experienced journalist who spent eight years tracking down all the evidence for and against the advice to eat low-fat diets. She finds that it was based on flimsy evidence, supported by an intolerant consensus backed by vested interests and amplified by a docile press. And it made us fatter.

In the 1950s heart disease had come from nowhere to be a big killer in America, especially of men in middle age. Although we now know that cigarettes were a huge cause - and the sharp recent decline of deaths from heart disease is mainly due to people smoking less, plus better treatments - scientists quickly decided that eating fat was the cause. Cholesterol clogs arteries, so eating highsaturated-fat food such as meat, eggs and dairy products must cause high cholesterol in the blood.

Plus, eating fat makes you fat. Obviously, no? The chief source of the antisaturated-fat message was a politically astute scientist named Ancel Keys. In 1961 he persuaded the American Heart Association to issue guidelines on saturated fat intake. The main evidence came from his study of heart disease in six countries in Europe plus Japan, from which he concluded that low-fat diets led to less heart disease.

Yet the data in the study were awful, Teicholz says. Keys left out countries that he knew produced inconvenient results, most of his low-fat countries were ones still recovering from wartime starvation, his dietary evidence came from a tiny subset of the men in his clinical sample, and his lowest-fat diet was from Crete during Lent, when meateating all but ceased. He published results in obscure German journals. Teicholz told me these were huge methodological problems, which should have called the entire study into question.

Even so, the fat effect was weak: an order of magnitude less than the effect of cigarettes on cancer, for example. Yet it was on this feeble and dodgy dossier that an entire edifice of advice was built. Sceptics kept pointing out inconvenient facts, but were ignored. How come native Americans, Inuit and Masai ate mostly meat and fat but had almost no heart disease or obesity, while they immediately got both when they started eating bread and potatoes? How come controlled trials of veterans and prisoners found that substituting

vegetable oils for animal fats caused no change of overall mortality rates? Anyway, we now know it just is not true that eating fat is what makes you fat. The body does not shunt butter directly to your thighs; it processes all food and adds to or draws down from fat reserves based on hormonal signals. Fat has more calories per unit of weight, but it's also more satiating. All the best evidence now suggests that it's easier to gain weight on a high-carb than a high-fat diet because the latter is more filling.

The sceptics were silenced by Keys and his allies and howled down by obedient journalists, a profession in love with conventional wisdom. Teicholz documents how the fat folk reviewed each other's papers, funded each other's projects and kept the doubters out, so that they gradually left the field. (Reminiscent of modern debates on climate change?) The American Heart Association, built up into a major force with funding from the vegetable-oil industry, relentlessly pushed the message that animal fat was bad. The US government issued guidelines in 1978. We in Europe followed suit, as we tend to do. And the message was driven home in the culture. Low-fat became a craze. It still is: look at supermarket shelves.

In the past ten years, study after rigorous study has found that animal fat per se is not harmful, does not cause obesity, does not raise the kinds of cholesterol that predict heart attacks, does not increase death rate and is healthier than carbohydrates. For instance, one two-year trial in Israel found that a fat-and-meat "Atkins" diet lowered weight more than either a low-fat or a Mediterranean diet. As Teicholz puts it in her book: "Every plank in the case against saturated fat has, upon rigorous examination, crumbled away."

Such findings remain too heretical for most diet experts. Those who make them struggle for years to get published and have to couch their findings in cautious language. Those such as Teicholz and Gary Taubes who write books pointing out that this fat emperor had no clothes are treated as pariahs. If anything, the official committees of the diet police are doubling down, demanding that we eat ever less saturated fat.

However, they are also now shifting the emphasis of their disapproval to sugar. In fact, while the evidence against carbohydrates in general as the cause of obesity and diabetes is good, the evidence against refined sugar being peculiarly evil is not. And there's a real problem developing. If we are to condemn carbs and sugar (and therefore fruit), and still condemn fat and red meat (as Action on Sugar does), then there's not much left to eat except sea bass and spinach. Which is not practical.

The message is all stick and no carrot, which is no way to win people round. So here's a suggestion for the diet police: put out a poster saying "We now think you should eat less sugar and bread, but that you should feel free to eat more eggs, meat and cheese again (but we might be wrong)".

The subtitle of Teicholz's book is: "Why butter, meat and cheese belong in a healthy diet." Yesterday I cooked bacon and eggs for my breakfast. And by the way, I don't have a vested interest: my farm has a dairy herd, but then it also grows wheat and vegetable oil.

Native Americans ate meat and fat but had little heart disease

We know the body does not shunt butter directly to your thighs

LOAD-DATE: June 30, 2014

LANGUAGE: ENGLISH

GRAPHIC: Ancel Keys convinced Americans that low-fat diets led to less heart disease

PUBLICATION-TYPE: Newspaper

JOURNAL-CODE: TIM

Copyright 2014 Times Newspapers Limited
All Rights Reserved

THE SUNDAY TIMES
The Sunday Times (London)

June 29, 2014 Sunday
Edition 1;
National Edition

Children's sugar diet linked to heart disease

BYLINE: Kate Mansey; Jon Ungoed-Thomas

SECTION: NEWS; Pg. 10

LENGTH: 767 words

Sweet excess

Sugar consumption of young children (aged 4 to 10)

Actual sugar consumption

15 teaspoons a day

Current recommended limit

10 teaspoons a day

Proposed new limit

5 teaspoons a day

*1 teaspoon equals 4g of added sugar Source: National Diet and Nutrition Survey. 2011-12

CHILDREN who have large amounts of sugar in their diet may be at risk of developing high blood pressure and heart disease, a new study reveals.

Researchers looked at the calorie intake of more than 300 children and found those with high-sugar diets were more likely to have higher blood pressure. The report, published in the American Journal of Clinical Nutrition, says the results support a hypothesis that excessive sugar consumption among children "may contribute to the development of poor cardiovascular health before maturity".

The food industry has argued that sugar is not "implicated" in any serious diseases, including diabetes, heart disease and cancer. However, this is just the latest research that suggests sugar may have a specific and adverse metabolic effect on the body over and above the effects of consuming too many calories.

Kenneth Kell, one of the report's authors, said: "Added sugars in our study were associated with risk factors for the development of cardiovascular disease. However, more research is needed to determine causality and physiological mechanisms."

Dr Aseem Malhotra, science director of the campaign group Action on Sugar, said: "The sooner the medical profession and the public accept that all calories are not metabolised in the same way, the sooner we will be able to tackle the increasing burden of chronic disease." The report has emerged after a government advisory group recommended that sugar consumption be halved to just five teaspoons a day. The group concluded that people who ate too much sugar were also likely to consume more calories overall, so there would be clear benefit in cutting sugar consumption.

Professor Ian Macdonald, who chaired the carbohydrate working group that reviewed sugar in the diet and who has advised Coca -Cola and Mars, said he had warned the two food giants that the long-term prospects for sales of **sugary drinks** and treats were "pretty poor".

France has imposed a tax on soft drinks and Macdonald believes other countries are likely to introduce laws to curb the obesity epidemic.

Campaigners in Britain are calling for a sugar tax and Labour is considering a cap on sugar in children's cereal.

Macdonald said: "Unless something positive happens to get people to change, then some governments will be tempted to try to use legislation. I am keen that this will be based on evidence rather than political expediency."

On Thursday, the carbohydrate working group of the scientific advisory committee on nutrition proposed limiting intake of added sugar to 5% of an individual's energy intake. It was one of the boldest public health initiatives in years and follows a similar recommendation from the World Health Organisation this year.

The new target is a blow to the sugar industry but will be hard to achieve. Some children already consume on average more than three times the new recommended amount.

Statistics from the UK's national diet and nutrition survey show that 11 to 18-yearolds consume 15.6% of their calories from sugar, mostly from **fizzy drinks**, squash and fruit juice. Younger children, aged 4-10, have the secondhighest sugar consumption, gaining 14.7% of their daily calories from sugar.

Food manufacturers have taken steps to reformulate products. Under the responsibility deal - a joint industry and Department of Health initiative to improve the nation's health - companies have removed billions of calories from products.

Manufacturers, supermarkets and caterers - including Britvic, Tesco and the caterer Sodexo - say they have removed more than 10bn calories from products in a year.

Some of the biggest companies - including Coca-Cola and Mondelez International, which makes Cadbury chocolate - have reformulated products but not disclosed the number of calories that have been removed.

The total number of calories removed under the reformulation initiative is likely to be well over 100bn calories a year, but this is less than 6% of a government target to cut 5bn calories a day from the nation's diet, or 1.8 trillion a year. Ministers say this cannot be achieved by the food industry alone and individuals must also change their diet.

The reformulation follows an initiative in America, championed by Michelle Obama, where Kellogg's, Nestlé, Pepsi-Co and other companies cut 6.4 trillion calories from products between 2007 and 2012.

LOAD-DATE: June 29, 2014

LANGUAGE: ENGLISH

GRAPHIC: ADRIAN WEINBRECHT

PUBLICATION-TYPE: Newspaper

JOURNAL-CODE: STS

Copyright 2014 Times Newspapers Limited
All Rights Reserved

THE SUNDAY TIMES
The Sunday Times (London)

June 29, 2014 Sunday
Edition 1;
Scotland

Soft drinks Games row still fizzing

BYLINE: Mark Macaskill

SECTION: NEWS; Pg. 7

LENGTH: 552 words

Allan McGee carries the Queen's Baton as part of the launch of Glasgow2014, but there is anger the Games are being sponsored by the makers of Irn-Bru THE organisers of the Commonwealth Games are facing renewed criticism for their relationship with Scotland's largest **soft drinks** manufacturer after two government reports raised serious concerns over the health impacts of **sugary drinks**.

AG Barr, which makes Irn-Bru, often referred to as Scotland's "other national drink", is one of the main sponsors of this summer's Games - a decision derided by health campaigners when it was announced in 2012.

Its links with the event provoked fresh anger last week after UK government scientists warned that drinking one can of **fizzy drink** a day is unhealthy.

A separate report by Public Health England outlined measures that it will consider recommending to minsters to tackle excessive sugar consumption, which it said is fuelling obesity and poor dental health in children and teenagers. Obesity could cost up to £50bn a year by 2050 if trends continue, the report said.

AG Barr has pledged to reduce the average calorific content per 100ml of their portfolio of drinks by 5% by 2016 but Malcolm Clark, of the Children's Food Campaign, which is backed by dozens of organisations including the British Medical Association, criticised its sponsorship of one of the world's largest sporting events.

In addition to providing undisclosed financial support to the Games, the company will also provide Irn-Bru; Rubicon, an exotic fruit juice drinks range; Strathmore Scottish spring water; and Barr, a range of soft drinks.

The Strathmore water and other beverages will be supplied to the athletes' village while Irn Bru, Strathmore, Rubicon and Barr will be made available across Glasgow 2014 venues.

Clark said: "Irn-Bru's position as a flagship brand for Glasgow 2014 looks even more absurd than when AG Barr was first announced as a major sponsor of the Games two years ago. The Games should be a celebration of healthy lifestyles, not 'an unrivalled opportunity to drive soft drink sales before, during and after', as AG Barr proudly proclaims."

There was similar outrage during the London's Olympics when sponsors including Coca-Cola and McDonald's monopolised food branding in the Olympic park and athletes' village. It contributed £612m to the London Olympiad.

Amid growing concern over the health toll of **sugary drinks**, the Scientific Advisory Committee on Nutrition halved the amount of sugar considered acceptable in adult diets last week. It warned that free sugars, found in sweetened drinks, fruit juice and confectionery and added to many processed foods, should make up just

5% of our daily energy intake. Previous advice was 10%. Public health doctors have suggested **sugary drinks** should carry health warnings.

A Glasgow 2014 spokeswoman said it did not enter into sponsorship agreements "lightly". She said: "People consume many different foods and beverages as part of their daily life and will have the same freedom to choose from a diverse range of options as part of an active, healthy lifestyle when attending the Games."

An AG Barr spokeswoman, said it was proud to support the Games and was making good progress in reducing calorie content across its portfolio.

Children's sugar diet linked to heart disease, page 10

LOAD-DATE: June 29, 2014

LANGUAGE: ENGLISH

PUBLICATION-TYPE: Newspaper

JOURNAL-CODE: STSscot

Copyright 2014 Times Newspapers Limited
All Rights Reserved

Sunday Express

June 29, 2014
Edition 1;
National Edition

This idea lacks fizz;
LETTERS

SECTION: LETTERS; Pg. 28

LENGTH: 83 words

I was interested to read your article about putting a tax on **fizzy drinks** in an effort to tackle obesity.

Fizzy drinks and chocolate biscuits for that matter are already subject to VAT at the standard rate of 20 per cent (check your till receipt). So why add another tax or duty, as with alcoholic drinks? The only **fizzy drink** that I consume is a sugar-free cola, as I find other drinks so sweet that I feel nauseous.

Would this be exempt from a sugar tax, I wonder? Granville Grey, Loughborough, Leics

LOAD-DATE: June 29, 2014

LANGUAGE: ENGLISH

PUBLICATION-TYPE: Newspaper

JOURNAL-CODE: SXP

Copyright 2014 Express Newspapers
All Rights Reserved

i-Independent Print Ltd

June 28, 2014
First Edition

@i Your View;
TEXTS, TWEETS AND EMAILS

SECTION: NEWS; Pg. 16

LENGTH: 960 words

I'm sorry, it's just not funny

Just as some women of my vintage move towards the right in their political views, perhaps my own senior moment has been to cease to find the fictional scorekeeper with the invisible assets, Samantha, amusing in any sense. (Simon Usborne, 27 June). I'm Sorry I Haven't a Clue was, in Humphrey

Lyttelton's hands, a marvel of understated, ironic comedy but has become a nostalgic revisiting of what we once found funny. Long ago, a blonde may well have walked into a bar, asked for an innuendo and the bar man gave her one, but the other stories in yesterday's i of the NHS Savile investigation and the murder of human-rights activist Salwa Bugaighis show us exactly why our propensity to laugh at old men, however enhanced their stature as national treasures, wheeling out convoluted jokes relying on sexual puns should be consigned to the BBC archive.

SANDRA SAMWELL

PETERBOROUGH

After your initial item on the BBC contemplating acceding to complaints about the Samantha jokes on I'm Sorry I Haven't a Clue, I went to a performance of a Shakespeare play at The Globe. It opened with a series of sexual doubleentendres. I would therefore like to suggest to the Beeb that it bans that disgusting writer, Shakespeare, in case some narrow-minded, uncomprehending and prudish ignoramus objects to this shameless filth.

VALERIE WEBER

LONDON SE13

Who will build the UK's homes?

Thomas Bowden claims that it is developers' responsibility to provide affordable housing (Letters, 27 June). This is complete nonsense. If developers are forced to provide the levels of affordable housing that Bowden advocates, they simply will not build any houses at all. Where will that get us? Requiring developers to provide social housing via local planning authority policies and legal agreements is simply a stealthy way for the Government to provide council houses via another route.

The two key issues this country has to face are: 1) Does the Government want to encourage home ownership or not? 2) If yes, how is that best achieved without encouraging rocketing house prices which is really in no one's interest in the long term?

As a planning and development consultant I am coming to the conclusion that there has to be a massive nationwide return to council-house building for rent or purchase on a non-profit basis.

STEVE CHAPMAN

LIVERPOOL

Building regulations require new houses to have a high level of insulation in order to save energy and builders must comply. I therefore cannot see why new buildings (domestic or commercial) should not be required by building regs to be fitted with the maximum possible solar panels in relation to roof size. Owners of these new buildings would not need to be included in the "feed-in tariff" as they will benefit from reduced electricity bills without the need to pay separately for a photovoltaic installation.

BEN BENTLEY

ROWDE, WILTS

Faking it, Wonga style

Much as I agree with the letter (i, 27 June), criticising Wonga's voluntarily stopping sending fake solicitor's letters, I do have some sympathy with it. After all, perhaps Wonga was simply following the example of some MPs who, when their wrongdoings on expenses claims were discovered, thought all they had to do was pay the money back to avoid further retribution.

NEVILLE DENSON

ST BEES, CUMBRIA

The "victims" here received letters from fake lawyers demanding repayment of money they had borrowed from Wonga on the basis that they would pay it back ... which they proceeded not to. They should be grateful they were from fake lawyers.

BEN WHITFORD

ST AUSTELL facing police action. These letters amount to not only fraud but the institutional bullying of some of the most vulnerable in society. The industry is already notoriously controversial and these letters are indicative of a management team who are either incredibly incompetent or assured of their ability to act with impunity.

JORDAN PHILIP

DUDLEY

Sugar unfairly demonised

As an employee of a **fizzy-drinks** company I feel disheartened by the recent reporting on the sugar and obesity issue (report, 26 June).

Fruit juice has the same or more **sugar** than a sugared **fizzy drink**, and with regard to obesity and type-2 diabetes, what is the difference between cake, which we seem to celebrate and promote on TV (Bake Off), **soft drinks**, chocolate, ice-cream, beer and wine, etc? They all have calories. Removing **fizzy drinks** entirely from our diet would not solve obesity or type-2 diabetes. A sedentary lifestyle has a much greater negative impact on health than calories.

JAKE BACKUS

OXFORD

This drug is dangerous

The use of marijuana has been proved to be a common precursor to the taking of stronger illegal drugs and to be a trigger for the around 5 per cent of the population suffering from schizophrenia or bipolar conditions to dangerous psychotic episodes. Messrs Nutini and the two members of One Direction you mention (People, 27 June) are not thinking clearly about the full implications of legalisation.

WINSTON MOLL

ELY, CAMBS

Strangers on the train

In response to PJ Stewart (Letters, 27 June) enjoying talking to strangers on public transport: usually if anyone approaches me on public transport and tries to strike up a conversation I know that they are a nutter.

AC DADDOW

BRISTOL

Cameron's instincts

If David Cameron didn't get it right about Andy Coulson, how can we feel assured that he's got it wrong about Jean-Claude Juncker?

SHEILA SIROTKIN

LONDON SE13

MORE COMMENT on independentvoices.com

i Quiz ANSWERS

Questions on page 2

1 Robin Hood

2 Australia

3 Nurse Edith Cavell

4 Ghost: author of The Ghost in The Machine; recorded "Ghost Town"; maker of Ghost series of cars.

5 JCB diggers

6 "It was a dark and stormy night..."

7 M6

8 Ohio

9 Admiral, from 'Amir'

10 Inside Llewyn Davis

It's an absolute outrage that Wonga is not

LOAD-DATE: June 27, 2014

LANGUAGE: ENGLISH

PUBLICATION-TYPE: Newspaper

JOURNAL-CODE: III

Copyright 2014 Independent Print Limited
All Rights Reserved

Daily Record & Sunday Mail

June 27, 2014 Friday
Edition 1;
National Edition

The scary truth about fruit juice;
Give kids water or milk, say experts, as research shows drinks we thought were healthy contain as much sugar as cola

BYLINE: Melanie Harvey

SECTION: RECORD WOMAN;FEATURES; Pg. 1

LENGTH: 631 words

SUGAR is the danger in our diet and parents are being warned about the ticking time bomb it is creating for children's health.

And it isn't just sweets, chocolate and **fizzy drinks** we need to watch.

Rather than being the healthy option mums and dads once thought it was, fruit juice has been revealed to contain almost as much of the white stuff as a can of cola.

Smoothies are also a bad choice, particularly shop-bought options, which often contain as much sugar as **fizzy drinks**.

The experts say we should give kids milk and water to drink instead.

They point out that fruit juices are helping fuel sharp rises in obesity, type 2 diabetes and heart disease.

A typical teenager consumes 40 per cent more sugar than they should. Adults take in 13 per cent too much.

The advisers say that, while most parents understand **fizzy drinks** are harmful, many wrongly believe that fruit juice is healthy.

They want them removed from the list of recommended five a day.

In a study published in the Lancet Diabetes and Endocrinology journal, researchers warned that orange juice is potentially as bad as sugary, sweetened drinks. A 250ml serving contains 115 calories - or seven teaspoons of sugar. A can of regular Coke has 139 calories.

Susan Jebb, an expert in diet and population health at Oxford University, said that parents should ban all sweetened drinks in favour of water and milk.

The professor, who is the Government's chief adviser on obesity, added: "I'd prefer to get sugar out of drinks altogether - a shift to low or no-calorie drinks and preferably water."

Meanwhile, Professor Tom Sanders, head of diabetes and nutritional sciences at King's College London, said smoothies should be only a treat.

"We need to reintroduce the habit of people putting a jug of water on the table and drinking water with their food instead of some sort of fruity beverage," he said.

Registered nutritionist Dr Carina Norris accepts that it is difficult for parents to wean their children off sugar.

She said that humans have a naturally sweet tooth, developed through years of evolution, but warned it is vital to make the changes to ensure better health in the long run.

Dunfermline-based Dr Norris added: "There are benefits to be had from the vitamins but I would much rather children were getting these vitamins from a piece of fruit rather than juice because they are then getting the benefit of the fibre as well.

"One piece of fruit has less sugar than a glass of juice and will actually fill you up. I would rather see children drink a glass of water but if they refuse point blank then try to water down the juice.

"Make the water more interesting - add ice, use a pretty straw."

When it comes to smoothies, Dr Norris was also cautious. She said: "They do contain some fibre but can be very high in sugar. Again, I would rather people were eating the whole fruit."

On so-called hidden sugars, she warned: "You will find it in so many foods, sauces for instance. Why would you know there was sugar in mayonnaise? You wouldn't but if it wasn't there you wouldn't think it tastes as nice.

"When you cook at home you wouldn't add sugar, you would add flavours through herbs and spices. Some manufacturers are starting to clean up their act and reduce sugar and make food how it should taste."

Hidden sugar foods It isn't just fruit juice that is packed with hidden sugar, many everyday foods contain much more than you would imagine. more than you would imagine.

Innocent Pure Fruit Smoothie Strawberries & Bananas (250ml): 7tsp Yeo Valley Yeo Valley Farm Zero Fat Farm Zero Fat Farm Zero Fat Vanilla Yoghurt Vanilla Yoghurt (150g): 5tsp Glaceau Glaceau Vitamin Water, Defence (500ml): 4tsp Kellogg's Kellogg's Special K Special K (30 ¦ g): 3tsp Loyd Loyd Grossman's Tikka Grossman's Tikka Masala Curry Sauce (175g): 4tsp

LOAD-DATE: June 27, 2014

LANGUAGE: ENGLISH

GRAPHIC: CHANGE IS VITAL TAL T Dr Carina Norris
WA W RNING Juice can contain as much sugar as fizzy pop

PUBLICATION-TYPE: Newspaper

JOURNAL-CODE: DRC

Copyright 2014 Scottish Daily Record & Sunday Mail Ltd
All Rights Reserved

DAILY MAIL (London)

June 27, 2014 Friday

HOW FRUIT JUICE HAS TURNED INTO JUNK FOOD

BYLINE: BY DAVID DERBYSHIRE

LENGTH: 1042 words

THERE was a time when it was marketed as the ultimate health drink, a glass of sunshine packed with vitamins and energy.

Generations were raised to believe orange juice fights off colds, boosts the immune system, tones the skin and protects against cancer. Yet in the topsy-turvy world of health advice, what's good for you one day, turns out to be bad for you the next.

This week an influential body of Government scientists blamed Britain's love affair with orange juice and other **sugary drinks** for fuelling a crisis of obesity and ill health.

The warning follows calls to remove fruit juice as one of the recommended five a day' portions of fruit or vegetables, and for parents to ban it from the meal table.

So if fruit juice turns out to be such a devil in disguise, why have we all been led to believe it was so healthy for so long?

The idea goes back to the 1920s, when American nutritionist Elmer McCollum blamed a condition called acidosis, an excess of acid in the blood, on diets rich in bread and meat. His bizarre solution was lots of lettuce and paradoxically acidic citrus fruits.

At the time orange juice was not hugely popular, but fruit growers leapt on the acidosis panic and sales rose.

Juice got an even bigger boost thanks to World War II when the U.S. Government wanted a new way to get a product rich in vitamin C to troops overseas. It poured money into research.

In 1947 just in time for the post-war consumer boom scientists invented a way to remove water from juice and freeze the concentrate into a palatable product.

The blocks of this concentrate could be sold to the new fridge-owning U.S. consumers or stored by manufacturers for months at a time, and sales exploded.

Meanwhile in the UK, war babies had been given rose hip, blackcurrant and concentrated orange juice by the Government as a cheap nutrition supplement in the 1940s. This continued into the 1950s, seeding the idea in a generation of baby boomers that juice is healthy.

By the 1980s orange juice was being marketed not just as a health drink, but also as the key to a stylish, modern life a status it enjoys today.

But while the juice in the supermarket is often sold as natural' or fresh', it is usually anything but.

Concentrating juice doesn't just remove water, it also removes the flavour. After it has been reconstituted, manufacturers add flavour packs' cocktails of chemicals which restore natural' oranginess.

You may think not from concentrate' juice means a more authentic product. You'd be wrong.

Juice made that way is heated and stored in air-free tanks for up to a year. Again, the process strips the juice of flavour, which has to be added afterwards. But the flavour packs contain orange essence and orange oil so don't have to appear separately on the ingredients list.

Manufacturers say they help give their product a consistent flavour. They also explain why juice in cartons doesn't taste like fresh juice.

Naturalness' isn't the only dubious claim made for juice. For decades, health gurus, and some doctors, have claimed the vitamin C in juice fights common colds.

But while the immune system needs vitamin C, there's little evidence that regularly taking the stuff prevents colds.

Research shows that the best that can be claimed for vitamin C is that it might shorten colds by a day or two. Given that most of us get two colds a year, worrying about vitamin C every day seems an over-reaction.

There's another myth about vitamin C, that citrus fruits are the best source. Yet plenty of vegetables, including broccoli, potatoes, kale and peppers, have doses comparable to those in fruit.

What you will get from juice is sugar. Lots and lots of it.

The new advice this week from the Scientific Advisory Committee on Nutrition is that men should have a maximum of 35g of sugar a day seven to eight teaspoons while women should not exceed 25g five to six teaspoons. A single 330ml glass of orange juice has eight teaspoons.

Helen Bond, spokeswoman for the British Dietetic Association, says: People have lost sight of how much sugar is in food and portion sizes have got bigger. A 150ml glass provides one of your five a day and anything more than that doesn't count. But measure people's glasses and they are often 250ml.

Juice provides a lot of vitamins and minerals, but unlike fresh fruit you don't get the healthy fibre.'

Doctors say the huge volume of sugar in our diet is contributing to the obesity epidemic, causing heart disease, cancer and diabetes.

There is growing concern that not all sugars are the same and that fructose, the type found in fruit, may be more harmful than table sugar. American hormone scientist Robert Lustig argues that it does more damage to the liver and cells than glucose or sucrose. He says excessive fructose intake is key to rising obesity and diabetes levels.

There is even evidence that fructose may contribute to higher uric acid levels in the blood and increase the risk of gout, an excruciatingly painful condition that is becoming ever more common.

Orange juice can also rot your teeth. Around half of five-year-olds have signs of damage to their tooth enamel, and too much fruit juice is thought to be a key cause.

There have been signs this year, however, that the tide is turning.

Schools have been leading the way. In January, Elizabeth Chaplin, the head of Valence Primary School in Dagenham, London, told parents that pupils would not be allowed juice in their lunch boxes. Instead, they had to drink water.

Around that time, Professor Susan Jebb, the Government's obesity tsar, said juice shouldn't count towards your five a day.

Fruit juice is absorbed very fast,' she said, so by the time it gets to your stomach, your body doesn't know whether it's Coca-Cola or orange juice, frankly. I have to say it is a relatively easy thing to give up. If you are going to drink it, you should dilute it.'

Weaning Britain off fruit juice may be difficult. Market research firm Mintel says 83 per cent of us drink fruit juice or a smoothie at least once a week, while 76 per cent believe fruit juice to be healthy.

But if you need motivation when you sit down to breakfast, remember this: there is more sugar in a 250ml glass of fruit juice than in a large bowl of Frosties with milk.

And that's food for thought.

© Daily Mail

LOAD-DATE: June 26, 2014

LANGUAGE: ENGLISH

PUBLICATION-TYPE: Papers

Copyright 2014 Associated Newspapers Ltd.
All Rights Reserved

DAILY MAIL (London)

June 27, 2014 Friday

TOUGH NEW GUIDELINES?... BUT NO HELP TO MEET THEM

BYLINE: BY SOPHIE BORLAND AND EMILY DAVIES

LENGTH: 694 words

OFFICIALS are urging people to halve their sugar intake to as little as five teaspoons a day to prevent obesity levels continuing to soar.

But they have been accused of failing to take proper action against the food industry to help the public meet the strict targets.

Guidelines from scientists advising Public Health England, the agency given the job of tackling obesity, yesterday stated that women should have no more than five to six teaspoons of sugar a day, and men seven to eight.

A 330ml can of **fizzy drink** contains around seven teaspoonfuls, so would meet this limit on its own.

Currently, Britons consume an average of 15 teaspoons daily, mainly due to the high volumes of sugar hidden in everyday items such as fruit juice, muesli, yoghurts, sandwiches and ready meals.

As reported in yesterday's Mail, experts are advising parents to ban fruit juice, squash and soft drinks from the dinner table and give their children only water and milk.

Senior doctors and academics want the Government to force food manufacturers to cut **sugar** levels, ensure products are clearly labelled and impose a tax on **soft drinks**.

But Public Health England merely said it would consider' these ideas as part of a nine-month consultation process that will eventually report to ministers next spring.

PHE simply issued a document suggesting a number of ideas the Government could consider to help the public meet these targets.

These included fruit juice and smoothies being banned from the five-a-day fruit and vegetable guidelines because they are so high in **sugar**. The organisation - set up last year to tackle public health issues - also said it would explore further approaches' such as adding a tax to **soft drinks** and banning junk food adverts before the 9pm watershed.

Other plans include discouraging supersize portions at coffee chains, fast food outlets and cinemas.

But ministers last night remained non-committal and said the PHE document would help inform' the debate about sugar.

Around one in four adults are now considered obese although this is predicted to rise to half the population by 2050, driving up levels of diabetes and heart disease.

Campaigners accused the officials of not doing enough to tackle the obesity epidemic.

Tam Fry, of the National Obesity Forum, said: This is all delaying tactics. We've had delaying tactics from this government for years. They're not going far enough.

We need tough, firm action with the industry. They need to take immediate action rather than sitting on the fence yet again.'

Dr Aseem Malhotra, a cardiologist at Croydon Hospitals, South London, said: This doesn't address the availability of sugar everywhere. The food industry have not made any promises to reduce sugar.

We want this to be translated into something that's meaningful for the public. We want them to slash their sugar levels by 40 per cent within four years.'

The Government has been accused of cosying-up' to the food industry and in so doing, failing to address the obesity epidemic.

Its flagship obesity policy, the responsibility deal, allows food firms to set their own targets for cutting sugar, salt and fat and for clearly stating the number of calories that products contain.

It has already emerged that five of the seven members of the Scientific Advisory Committee on Nutrition, which drew up yesterday's guidelines on sugar have financial ties to the food industry.

They include the chairman, Ian MacDonald, professor of metabolic physiology at Nottingham University, who has worked as a paid adviser for Coca-Cola and Mars and whose research has been funded by Unilever, the world's biggest producer of ice cream.

Others include a consultant for the world's largest chocolate producer, Barry Callebaut, and scientists whose research has been funded by Unilever.

Public Health Minister Jane Ellison said: We want to help people make healthier choices and get the nation into healthy habits. This report will inform the important debate taking place about sugar.'

Dr Alison Tedstone, chief nutritionist at Public Health England, said: We are very concerned about sugar intakes.'

How fruit juice has turned into junk food - Page 22

From yesterday's Mail

© Daily Mail

LOAD-DATE: June 26, 2014

LANGUAGE: ENGLISH

PUBLICATION-TYPE: Papers

Copyright 2014 Associated Newspapers Ltd.
All Rights Reserved

Daily Mirror

June 27, 2014 Friday
Edition 2;
National Edition

Cut sugar to stop obesity

SECTION: NEWS; Pg. 15

LENGTH: 80 words

DAILY sugar consumption should be halved to 5% of a person's energy intake to help tackle the obesity crisis, experts warned yesterday.

The advice came as figures showed almost threequarters of 45 to 74-yearolds in England are obese or overweight, increasing the chances of heart disease, cancer and stroke, as well as Type 2 diabetes.

An adult's recommended daily sugar limit would be reached with just one 330ml can of **fizzy drink,** said the Scientific Advisory Committee on Nutrition.

LOAD-DATE: June 27, 2014

LANGUAGE: ENGLISH

PUBLICATION-TYPE: Newspaper

JOURNAL-CODE: DMR

Copyright 2014 MGN Ltd.
All Rights Reserved

The Daily Telegraph

The Daily Telegraph (London)

June 27, 2014 Friday
Edition 1;
National Edition

Children must be taught that eating is a joy;
We all consume too much sugar - but meals and food are about more than calories

BYLINE: PAUL LEVY

SECTION: EDITORIAL; OPINION, COLUMNS; Pg. 22

LENGTH: 828 words

We all know that our children have too many sugary, **fizzy drinks**, and common sense, as well as science, tells us that this is a cause of the current obesity crisis. It's not the only one - I'm overweight and I never touch sugary pop, not even tonic with my gin.

Fruit juice is another dietary villain, and Professor Susan Jebb, the Government's chief obesity adviser, says families should be stricter about what their children drink, limiting them to a small glass with breakfast. More interesting and more helpful is the advice she's given about other meals: "Drink water, that's the very simple advice to parents." There has also been talk of banning carbonated, sweetened drinks and even fruit juice in schools. But what's really needed is a change of habits.

At home we follow the French (and American, believe it or not) custom; there is always a jug of water on the table at mealtimes - especially when the principal drink is beer or wine and there are guests. (We live in the country, and our guests have usually driven. It's simply uncivilised, and dangerous, to fail to provide an

alternative to alcohol to anyone who is driving.) Before there were such huge profits to be made from selling bottled water, every restaurant in America used to bring water to the table, even before presenting the menu. Good restaurants here in Britain too still do this, and all good eateries now offer patrons the choice of tap or bottled water.

Progress on the home front, though, is slower, the sad statistics show. As Professor Jebb says, "the biggest source of sugar across all age groups is **sugar**-sweetened **beverages**". Youngsters, and plenty of grown-ups, derive pleasure from these obesity time-bomb bevvies. Instead of thinking of bans, we ought to be thinking about providing alternative, less damaging forms of gratification.

Of course, the first thing to get right is to bring children up to regard the act of eating as an enjoyment, not a chore, in other words to recognise and cherish the pleasures of the table - and not merely regard eating as a nutritional necessity. Our motto should be "food, not fuel", and this means, above all, having structured meals, which are family and social occasions.

This is not only important in the fight against flab, but it is (and should be regarded as) the chief way we civilise our children. It is at table that we teach them, not only that eating is fun, but that it is done with a certain amount of grace and concern for the feelings of others, and accompanied by conversation. What children learn at the family table is as important as what they learn at school; unhappily, this is also true if what they learn at table is to be unsociable or aggressive.

For years we have been told that structured meals - meaning meals that take place at about the same time every day, and with attendance more or less mandatory - are fundamental to good family relations. It has not been a very British attitude, but surely we can admit to ourselves that this also affects society; that children brought up this way make better citizens. Even takeaway and ready meals can be consumed at a table with some amount of ceremony and fellowship.

A bottle of wine, too, does no harm and in my view, a lot of good. In our non-religious culture we are trying, after all, to teach our children that meals are mostly about delight, not about sacred ritual, duty or digestion. While the scientific details are not yet incontrovertible, it is clear that a glass, or even two, of wine with dinner, is on the whole beneficial to health.

What is unarguable, though, is that wine (or beer or cider) enhances the pleasure we take in eating, and, providing it's of decent quality, is itself a source of enjoyment. It is, also, the very opposite of the binge drinking that has become such a problem in contemporary Britain.

Just as we want to teach our children a modicum of table manners, we should be instilling good drinking habits in our young - at home. Like the French, we should allow children in the 11 to 18 age group (the National Diet & Nutrition Survey 2008-12 shows this cohort gets a whopping 15.6 per cent of its daily food energy from added sugar) to have a couple of drops of wine to colour their water at mealtimes. As they get older we should give them watered-down wine, and by this means teach them to love and appreciate a proper glass of wine with their food as they become adult.

It's all about pleasure. If we forget this, and think of food only in functional terms, as mere calories, we might as well glug Soylent, "the magic food replacement milkshake" now available in the US. It is said to be palatable, but consuming it is a solitary act, devoid of pleasure. That is where our current attitude to food may lead us. Who wants to sit down at a table with friends over a beige mixture of vegetable oils and nutrients?

Paul Levy is chairman of the Oxford Food Symposium

Comment on Paul Levy's view at www.telegraph.co.uk/personalview

LOAD-DATE: June 27, 2014

LANGUAGE: ENGLISH

PUBLICATION-TYPE: Newspaper

JOURNAL-CODE: DTL

Copyright 2014 Telegraph Media Group Limited
All Rights Reserved

The Daily Telegraph
The Daily Telegraph (London)

June 27, 2014 Friday
Edition 1;
National Edition

No use keeping sugary drinks off the table if children eat meals elsewhere; Letters to the Editor

SECTION: LETTERS; Pg. 23

LENGTH: 407 words

SIR - Professor Susan Jebb, the Government's chief obesity adviser, has said that parents should ban all drinks but water from the dinner table (report, June 25). Perhaps children's digestive systems would benefit if they were to eat at the dinner table in the first place.

Robin Whiting Castle Rising, Norfolk

SIR - By all means, let's encourage families to drink more water and make sugary drinks an occasional treat for children.

Drinking fountains in public places and school corridors were commonplace into the Fifties and Sixties. Since the water supply was privatised, these have all but disappeared.

The first water fountains were a public health measure, introduced by the Victorians. We need to reinstate the humble drinking fountain as part of our battle plan against obesity.

Surely the water companies could fund this as part of their corporate social responsibilities. Now that would be a "Responsibility Deal".

Professor John R Ashton President, Faculty of Public Health London NW1

SIR - The costs of the NHS are unsustainable and obesity adds £15 billion annually. How to solve both problems? Levy VAT at the full rate on all drinks and foods containing added sugar. Nannystatish perhaps, but this sugar is not required nutritionally.

The Conservatives might introduce this after the election, not before, or they risk an attack by Labour on "fashionably slim wealthy rulers targeting fat proles". Richard Bethell Horley, Surrey

SIR - Tax on sugar may have been removed in 1874 (Letters, June 21) but excise duty was imposed on it in 1901. This tax was easily and cheaply collected, being paid by the sugar producers. The tax covered sugar, invert, molasses, glucose syrups and saccharin. The duty was repealed in 1962. Peter Hull Hoo, Kent

SIR - Rather than resuscitating a sugar tax, the Government should be looking at positive ways to encourage reduced calorific consumption. This could be by subsidising the consumption of stevia. This is a zero-calorie

sweetener now being used in carbonated drinks, among other things. The level of subsidy might be related to the estimated amount of NHS savings in its costs of treating obesity.

Nicholas Sibley La Colle sur Loup, Alpes-Maritimes, France

SIR - I still have a box of sugar cubes bought in France at the time of the 1975 sugar crisis. Since I gave up sugar in tea 30 years ago, it will be left to one of my descendants to deal with.

Harry Chamberlain Lichfield, Staffordshire

LOAD-DATE: June 27, 2014

LANGUAGE: ENGLISH

PUBLICATION-TYPE: Newspaper

JOURNAL-CODE: DTL

Copyright 2014 Telegraph Media Group Limited
All Rights Reserved

The Daily Telegraph

The Daily Telegraph (London)

June 27, 2014 Friday
Edition 1;
National Edition

Juices may be dropped from 'five a day'

BYLINE: Laura Donnelly

SECTION: NEWS; Pg. 4

LENGTH: 310 words

FRUIT juice could be removed from offi-cial "five a day" recommendations in an attempt to reduce the nation's sugar intake, health officials have said.

Recommended daily limits on sugar will be halved under new advice to the Government which says no more than 5 per cent of calories should come from sugar.

It follows concern that consumption of fruit juices, smoothies and soft drinks is adding to Britain's obesity crisis.

They currently account for the greatest share of sugar intake among children and teenagers.

Public health officials last night said they will "reconsider" the current advice that fruit juice counts as a maximum of one portion a day, while a smoothie can count as more than one portion, depending on its contents.

The report by Public Health England states: "Fruit juices can be major providers of sugar for some people, particularly children aged under 11 years. Smoothies are also high in sugar from fruit and are popular with consumers as they may count as more than one portion of your five a day."

Officials will publish their recommendations early next year.

Earlier this year, Professor Susan Jebb, the Government's chief obesity adviser, said she would support removing fruit juices from "five a day" guidelines and encourage people to eat a piece of fruit instead.

Earlier this week she recommended that parents should serve only water with meals and ban fruit juice and **fizzy drinks** from the dining table to reduce children's sugar intake.

An investigation by The Daily Telegraph identified some smoothies as having at least three times the new recommended limit, which equates to around six to seven teaspoons of sugar for women, and seven to eight teaspoons for men.

Many fruit juices sold by major brands provide six to seven teaspoons per serving - meaning the daily limits are reached without any other sugar in the diet.

LOAD-DATE: June 27, 2014

LANGUAGE: ENGLISH

PUBLICATION-TYPE: Newspaper

JOURNAL-CODE: DTL

Copyright 2014 Telegraph Media Group Limited
All Rights Reserved

The Daily Telegraph

The Daily Telegraph (London)

June 27, 2014 Friday
Edition 1;
National Edition

One bar of chocolate is enough to push you over government experts' new sugar limits

BYLINE: Laura Donnelly

SECTION: NEWS; Pg. 4

LENGTH: 848 words

A SINGLE bar of chocolate, three yogurts or a **fizzy drink** would take the average person over proposed daily sugar limits announced by scientific advisers to the Government.

The recommendations halve current limits, meaning that the average woman should have no more than five to six teaspoons (25g) of sugar a day, and seven to eight teaspoons (35g) for men.

Scientific advisers to the Government yesterday signalled a war on sugar with draft recommendations that would require radical changes to the UK diet.

The new proposed limits mean that many food products that fit within current daily allowances will no longer do so.

A 330ml can of Coca-Cola contains 35g (1.2oz) of sugar, while a Mars bar has 33g (1.16oz) - both well in excess of the average 25g (0.88oz) limit for women and using up the full allowance for men. Three Waitrose low-fat black cherry yogurts provide 27g (0.95oz) in added sugars, exceeding the women's allowance.

The advice from the Scientific Advisory Committee on Nutrition (SACN) says that no more than five per cent of daily calories should come from sugar - compared with the current limit of 10 per cent.

Health officials say all age groups are exceeding current recommendations, with teenagers on average consuming 50 per cent more sugar than they should. The average adolescent drinks one 330ml can of **fizzy drink** every day.

Yesterday public health officials said families should think carefully about whether children should be allowed **fizzy drinks** at all, and said the public needed to be encouraged to swap such beverages for low-calorie versions, or water.

Dietitians said **fizzy drinks** had "no place" in a healthy diet.

On Wednesday the Government's chief obesity adviser said parents should serve water with meals and ban **fizzy drinks** and juices from the dining table in order to reduce their children's sugar intake. The new proposed guidelines also say Britain needs to boost its intake of fibre from 24 grams (0.8oz) to 30 grams (1.05oz) a day. The recommendations follow a six-year review of 600 scientific studies on consumption of carbohydrates, including sugar. Two thirds of adults in Britain are overweight or obese, and British girls below the age of 20 are now the most overweight in Western Europe.

A separate report by Public Health England outlines a range of measures that it will consider recommending to minsters in an attempt to reduce sugar intake, including taxes on sugary foods, restrictions on advertising and a clampdown on retail promotions relating to sugary foods.

Dr Alison Tedstone, the chief nutritionist at Public Health England, said: "Eating too much sugar is harming our health; excess sugar and calorie intake leads to being overweight and obese, and consequently having a higher risk of developing type 2 diabetes, heart disease and breast and colon cancer."

The report warns that obesity could cost up to £50 billion a year by 2050 if current trends continue.

Experts warned of a health crisis with new figures showing that seven out of 10 people aged 45 and over are overweight or obese. Those below the age of 25 are the only group in which the majority are not overweight, the new statistics from the Health and Social Care Information Centre show.

Tam Fry, of the National Obesity Forum, said: "These figures show that there is a looming health crisis for the nation. There needs to be a clear message that everybody needs to take control and watch what they are eating."

Age UK, the charity, said not enough effort was made to encourage older people to adopt healthier behaviours. Ruthe Isden, from the charity, said: "Historically, public health approaches have been very focused on children, and it's all about getting them while they are young and if you are old then 'it's a bit late to bother'. There is a really important need to turn some of that around and actually start talking to people about how it's never too late."

Paul Levy: Page 22

Big problem Our overweight population

People are deemed to be of normal weight if they have a body mass index (BMI) score of 18.5 to 24.9 and overweight with a score of over 25.

Those with a BMI over 30 are deemed to be obese.

BMI is calculated by dividing a person's weight in kilograms by their height in metres squared.

36% of people aged 16 to 24 are either overweight or obese.

People in this age group have an average BMI score of 24.5.

51% of people aged 25 to 34 are either overweight or obese. People in this age group have an average BMI score of 26.1.

64% of people aged 35 to 44 are either overweight or obese. People in this age group have an average BMI score of 27.3.

72% of people aged 45 to 54 are either overweight or obese. People in this age group have an average BMI score of 28.3.

73% of people aged 55 to 64 are either overweight or obese. People in this age group have an average BMI score of 28.3.

73% of people aged 65 to 74 are either overweight or obese. People in this age group have an average BMI score of 28.3.

70% of people aged 75 and over are either overweight or obese. People in this age group have an average BMI score of 27.6.

LOAD-DATE: June 27, 2014

LANGUAGE: ENGLISH

PUBLICATION-TYPE: Newspaper

JOURNAL-CODE: DTL

Copyright 2014 Telegraph Media Group Limited
All Rights Reserved57 of 371 DOCUMENTS

The Daily Telegraph
The Daily Telegraph (London)

June 27, 2014 Friday
Edition 1;
Scotland

Fruit juices could be dropped from your 5 a day

BYLINE: Laura Donnelly

SECTION: NEWS; Pg. 2

LENGTH: 297 words

FRUIT juice could be removed from official "five a day" recommendations in an attempt to reduce the nation's sugar intake, health officials have said.

Recommended daily limits on sugar will be halved under new advice to the Government which says no more than 5 per cent of calories should come from sugar.

It follows concern that consumption of fruit juices, smoothies and soft drinks is adding to Britain's obesity crisis.

They currently account for the greatest share of sugar intake among children and teenagers.

Public health officials last night said they will "reconsider" the current advice that fruit juice counts as a maximum of one portion a day, while a smoothie can count as more than one portion, depending on its contents.

The report by Public Health England states: "Fruit juices can be major providers of sugar for some people, particularly children aged under 11 years. Smoothies are also high in sugar from fruit and are popular with consumers as they may count as more than one portion of your five a day."

Officials will publish their recommendations early next year.

Earlier this year, Professor Susan Jebb, the Government's chief obesity adviser, said she would support removing fruit juices from "five a day" guidelines and encourage people to eat a piece of fruit instead.

Earlier this week she recommended that parents should serve only water with meals and ban fruit juice and **fizzy drinks** from the dining table to reduce children's sugar intake.

An investigation by The Daily Telegraph identified some smoothies as having at least three times the new recommended limit, which equates to around six to seven teaspoons of sugar for women, and seven to eight teaspoons for men. Many fruit juices sold by major brands provide six to seven teaspoons per serving.

LOAD-DATE: June 27, 2014

LANGUAGE: ENGLISH

PUBLICATION-TYPE: Newspaper

JOURNAL-CODE: DTLscot

Copyright 2014 Telegraph Media Group Limited
All Rights Reserved

The Express

June 27, 2014 Friday
Edition 1;
National Edition

Britons must halve sugar intake say experts

BYLINE: Nathan Rao

SECTION: NEWS; Pg. 3

LENGTH: 321 words

MILLIONS of Britons will have to halve their sugar intake to meet new guidelines to be considered by the Government.

In a bid to fight obesity, scientists advising the Department of Health say one can of **fizzy drink** will contain a person's proposed entire daily allowance.

The Scientific Advisory Committee on Nutrition (SACN) says exceeding current guides is a major reason for obesity which is fuelling a rise in Type 2 diabetes. It wants the recommendation that no more than 10 per cent of daily energy intake should come from "free" or added sugar to be cut to five per cent.

Currently men should eat no more than 35grams - or eight teaspoons of sugar - a day, with women limited to 25 grams. The draft report calls on people to cut down on cakes, biscuits and sweets, but **sugary drinks** are considered the worst menace. One 330ml can of **fizzy drink** can contain a typical adult's proposed five per cent daily energy intake allowance.

Dr Alison Tedstone, chief nutritionist at Public Health England, said: "There is something about sugar-sweetened drinks that will make you more likely to over-consume calories. Adolescents are having **fizzy drinks** every single day."

Professor Ian Macdonald of the SACN said: "High sugar intake in adults is associated with increased energy intake and risk of obesity.

"There is an association between **sugar**-sweetened **beverage** intake and Type 2 diabetes in children. There is a clear demonstration that **sugar**-sweetened **beverage** intake is associated with weight gain."

The report follows a call earlier this week from Professor Tom Sanders of King's College London for fruit juice and **fizzy drinks** to be banished from the family dinner table and replaced by water.

The British Soft Drinks Association said the industry has "dramatically increased" the range of low and no calorie options.

The report has been put out for a public consultation with new government guidelines possibly being set next spring.

LOAD-DATE: June 27, 2014

LANGUAGE: ENGLISH

PUBLICATION-TYPE: Newspaper

JOURNAL-CODE: DXP

Copyright 2014 Express Newspapers
All Rights Reserved

The Express

June 27, 2014 Friday
Edition 1;
National Edition

New WARNING over sugar iN fizzy DRINKS

SECTION: NEWS; FRONT PAGE, TEASERS; Pg. 1

LENGTH: 16 words

New WARNING over sugar iN **fizzy DRINKS** (Just one can contains your daily ration) full story Page 3

LOAD-DATE: June 27, 2014

LANGUAGE: ENGLISH

PUBLICATION-TYPE: Newspaper

JOURNAL-CODE: DXP

Copyright 2014 Express Newspapers
All Rights Reserved

FT
FINANCIAL TIMES
Financial Times (London, England)

June 27, 2014 Friday
London Edition 1

Health agency targets fizzy drinks

BYLINE: Scheherazade Daneshkhu, Consumer Industries Editor

SECTION: NATIONAL NEWS; Pg. 4

LENGTH: 447 words

A tax on **fizzy drinks** and an advertising crackdown are among measures being considered by the government 's health agency to tackle the mounting problem of obesity in the UK.

Public Health England, the government agency, said the country's sugar-rich, unhealthy diet threatened to double obesityrelated healthcare costs to £9.7bn by 2050.

The health agency was speaking yesterday after the UK's top scientific nutrition committee published a 366-page report recommending people slash their daily sugar intake by half or risk rising levels of obesity, type 2 diabetes and heart disease.

If the government backs the proposal, this would mean limiting daily intake of sugar added to food and natural sugars in honey and fruit juices to the equivalent of less than one 330ml can of a sugary carbonated drink.

AB Sugar, part of Associated British Foods, said it was "surprised" at the recommendation sugar not exceed 5 per cent of daily calorie intake, saying: "This sets a level that will be very difficult for most people to meet."

This would bring the UK in line with the recent World Health Organisation proposal that 5 per cent would be "ideal" target, although it stuck to its 10 per cent recommendation as more realistic. All age groups exceed the 10 per cent recommendation in the UK, especially children and teenagers consuming 50 per cent more than this amount. "Obesity is a risk factor that is contributing to an epidemic of chronic diseases in this

country," said Professor Kevin Fenton, director of health at Public Health England. "We're living longer but spending more of those extra years with illnesses."

Some measures being considered, such as a sugar tax on **fizzy drinks**, are likely to set the government on a collision course with the food and beverage industry, which prefers voluntary measures brokered through its so-called "responsibility deal" with the government.

The British Soft Drinks Association hit back, saying soft drinks comprised only 3 per cent of the average UK diet. The biggest companies were raising spending on advertising low and no-calorie drinks this year by 49 per cent, it added.

But Dr Alison Tedstone, Public Health England's chief nutritionist, said **soft drinks** accounted for 30 per cent of children's **sugar** intake. One-third of 10 to 11-year-olds are overweight or obese in England, as are two-thirds of adults.

She said the agency would ramp up health campaigns, saying **fizzy drinks** sales had fallen 9 per cent after an ad campaign last summer. Nutritionists have urged parents to put water on the table at mealtimes instead of fruit juice, saying people have got out of the habit of drinking water to quench thirst.

Blog at www.ft.com/ftdata

LOAD-DATE: June 26, 2014

LANGUAGE: ENGLISH

PUBLICATION-TYPE: Newspaper

Copyright 2014 The Financial Times Ltd.
All Rights Reserved
Please do not cut and paste FT articles and redistribute by email

theguardian

The Guardian - Final Edition

June 27, 2014 Friday

Leading Article: Obesity crisis: Good food guide

BYLINE: Editorial

SECTION: GUARDIAN LEADER PAGES; Pg. 40

LENGTH: 681 words

Put a jug of water on the table. As a slogan, it has the beauty of simplicity. As a health message, it is clear, affordable and inoffensive. But as a symbol of the state's willingness to get to grips with the damage the food industry does to public health, it is redolent of defeatism. The battle to get us all to eat better, drink more healthily and live well takes more than a smart strap line and - in the same way that ending endemic tooth decay meant putting fluoride in the mains water supply - it takes more than self-help and individual effort.

Sugar is bad for us. It is one of the main drivers of the obesity epidemic, particularly in combination with fat. According to YouGov's poll last weekend, nearly four people in five recognise that too much is harmful and

that children and young people in particular consume too much. Now the government's science advisory committee says the target for sugar consumption should be just 5% of the average calory intake. That's half the current target which - like the five portions of fruit and vegetables - is widely honoured in the breach. That was confirmed in this year's official national diet and nutrition survey, which found that all age groups consume more than they should, with **sugary drinks** and cake and biscuits being the main offenders. The problem for consumers is that there are myriad different targets for sugar consumption, which leaves even the most dedicated label-reader confused. Just how the industry likes it.

Governments are always reluctant to tell people what is good for them, and it is even harder now that food processing is the biggest manufacturing sector in Britain. The power of the industry is the single biggest obstacle to a healthier country: see the 50-year campaign against smoking for confirmation. Overcoming the richly resourced opposition of the tobacco industry and slowly pushing anti-smoking policy up the scale from health warnings to advertising and marketing controls and finally to compulsory plain packaging, which it has just been confirmed should be in force next year, has taken an unlucky smoker's lifetime.

Yet this government thought the food business would volunteer to abandon its existing recipe for success and try to improve what we eat. It negotiated a so-called "responsibility deal". Some of the biggest names in food manufacturing, such as Nestle and Coca-Cola, signed up to help customers cut calories. But it now seems the pledges apply only to lesser-selling brands. The main brands are not significantly changed. In the face of four years of failure, it is time to acknowledge that voluntarism does not work. There are times when the state has to step in, and this is one of them.

One idea is to tax **sugary drinks**, as France, Hungary and Ireland all plan to do. Yesterday research from the north-west's Food Active campaign was published that predicted a 20% tax on **sugary drinks** would mean 600 fewer cases of diabetes and 400 fewer strokes in the region. Lobbyists such as Sustain, as well as the main medical bodies, back such a tax. But price is a tough mechanism to manipulate, as proven by the attempt to introduce minimum pricing on alcohol - finally introduced, but at a level affecting only 1% of sales. What would work is setting a target for sugar content. It is already done to salt levels, and is credited with delivering cuts of up to 50% in some foods in just two years. Campaigners believe sugar targets could be as effective. They have advertising controls and different branding to make sugary foods less appealing to children in their sights too. They might work. But not without state backing.

Yet despite this public health emergency, politicians are nervous, talking of personal responsibility and the nanny state tag. There are real concerns about the impact of taxes on low-income families. They should remember that cheap food is often nutritionally bad food. It is linked to obesity and cancer. But for many people, there is no real alternative. That is an affront to public health, and the guardians of public health must take action against it.

LOAD-DATE: June 27, 2014

LANGUAGE: ENGLISH

PUBLICATION-TYPE: Newspaper

Copyright 2014 Guardian Newspapers Limited
All Rights Reserved

theguardian

The Guardian - Final Edition

June 27, 2014 Friday

Guideline on sugar 'should be cut by half'

BYLINE: Ian Sample, Science editor

SECTION: GUARDIAN HOME PAGES; Pg. 19

LENGTH: 467 words

The guideline level of dietary sugar should be cut by half to the equivalent of one can of **fizzy drink** a day, according to scientists who advise the government.

People should get no more than 5% of their daily calories from so-called "free sugar", a term that includes table sugar, the sugar added to food and drinks, and that found naturally in fruit juices, syrups and honey, they said.

The advice, to be considered by the Department of Health after a three-month consultation, sets an upper limit on free sugar of about 25g or six teaspoons a day for a healthy woman, and 35g or seven teaspoons for a man. Across all ages, people in England consume more than the existing guideline of 10% of calories as sugar, with adolescents consuming far more. On average, adults get 12% of their daily energy from sugar, and for adolescents the figure rises to more than 15%.

Scientists raised particular concerns about **sugary drinks** and urged people to minimise the amount they consumed, where possible swapping to healthier alternatives such as water or low-fat milk. Under the proposal, a single can of **fizzy drink** would contain enough sugar on its own to meet an adult's daily sugar limit.

The recommendation follows a six-year review by the government's scientific advisory committee on nutrition (SACN) of research into the health effects of carbohydrates, which include sugar. The report found that higher sugar intake was associated with tooth decay and weight gain. Being overweight puts people at greater risk of type 2 diabetes, cardiovascular disease and some forms of cancer.

"By reducing to 5% you reduce the risk of all those things to what, from a scientific perspective, would be acceptable.

"The challenge is to get there," said Ian Macdonald, chair of SACN's carbohydrate working group. "You have to reduce the consumption of all foods with free sugars, not just one type."

The proposed 5% limit mirrors draft guidelines published by the World Health Organisation. The report says people should get half their daily energy from carbohydrates. It goes on to recommend high-fibre diets, with the guideline level for dietary fibre rising to 30g for adults.

The report does not recommend changing the advice to eat five portions of fruit and vegetables. Public Health England launched its own paper on how to help people reduce their sugar intake. It said it would reconsider its advice on fruit juice and xs bhbsmoothies in its five-a-day campaign.

Alison Tedstone, chief nutritionist at PHE, said: "Being a little bit overweight, being slightly podgy, is raising your risk of serious diseases including type-2 diabetes, which will have potent devastating effects on you and your family."

Captions:

5%

The proportion of daily calories that should come from sugar according

to guidelines from the Department of Health

LOAD-DATE: June 27, 2014

LANGUAGE: ENGLISH

PUBLICATION-TYPE: Newspaper

Copyright 2014 Guardian Newspapers Limited
All Rights Reserved

i-Independent Print Ltd

June 27, 2014
First Edition

Just one can of fizzy drink each day damages health; NUTRITION

BYLINE: Charlie Cooper HEALTH REPORTER

SECTION: NEWS; Pg. 4

LENGTH: 232 words

Drinking just one can of **fizzy drink** a day is unhealthy, according to new guidance which halves the amount of sugar considered acceptable in adult diets.

In a major shift, government health advisers say that free sugars - found in sweetened drinks, fruit juice and confectionery and added to many processed foods - should make up just 5 per cent of our daily energy intake. Previous advice set the limit at 10 per cent.

Experts say the new guidance is necessary to combat Britain's obesity epidemic and protect children from diabetes, heart disease and tooth decay. For an adult this would mean that a single can of **fizzy drink**, which contains the equivalent of six to seven teaspoons of sugar, is almost the entire allowance for the day.

Government advisers from the Scientific Advisory Committee on Nutrition (SACN) said every population group in England is already exceeding the 10 per cent limit on sugar consumption.

Professor Ian Macdonald, the chairman of the SACN's working group on carbohydrates, which has produced the draft guidance, said there were clear associations between high levels of free-sugar intake and overall increased energy intake and obesity. "There is also an association between **sugar**-sweetened **beverages** and type-2 diabetes," he added. Dr Alison Tedstone, the chief nutritionist at Public Health England, said that obesity was a "very diffi-cult" problem for the UK.

LOAD-DATE: June 26, 2014

LANGUAGE: ENGLISH

PUBLICATION-TYPE: Newspaper

JOURNAL-CODE: III

Copyright 2014 Independent Print Limited
All Rights Reserved

The INDEPENDENT
Independent.co.uk

June 27, 2014 Friday 7:57 AM GMT

Britons told to halve sugar intake to beat obesity;
Under new guidelines an adult could no longer drink a can of Coca Cola or similar drink and stay within their daily allowance

BYLINE: Charlie Cooper

SECTION: FRONTPAGE

LENGTH: 780 words

People should halve their daily intake of sugar to combat Britain's obesity epidemic and protect children from diabetes, heart disease and tooth decay, Government health advisors have said.

In a potentially major shift to official dietary advice, experts said that free sugars - those found in sweetened drinks, fruit juice, confectionery, and added to a wide range of processed foods - should make up just five per cent of our daily energy intake.

For an adult this would mean that a single can of a typical **soft drink** such, which contains the equivalent of six to seven teaspoons of **sugar**, would represent almost their entire healthy allowance for the day.

Given the widespread use of free sugars in everything from ready meals to canned soup, this means that, realistically, an adult could no longer drink a can of Coca Cola or similar drink and stay within their daily allowance, experts said.

Government advisors from the Scientific Advisory Committee on Nutrition (SACN) said that every single population group in England is already exceeding the current 10 per cent limit on sugar consumption.

Professor Ian MacDonald, chairman of SACN's working group on carbohydrates, which has produced the draft guidance, said there were clear associations between high levels of free sugar intake and overall increased energy intake and obesity.

"There is also an association between **sugar**-sweetened **beverages** and type-2 diabetes," he said. "In children there is clear demonstration that **sugar**-sweetened **beverages** are associated with obesity. By reducing it to five per cent you would reduce the risk of all of those things. The challenge will be to get there."

The term "free sugars" does not include those found in fruit and milk - but does encompass sugars added to food by the manufacturer, cook or consumer, and sugars naturally present in syrups and unsweetened fruit juices.

Public health minister Jane Ellison said that the new advice confirmed "eating too much sugar can have a significant impact on health". SACN's report will now be subject to a consultation, and final guidance will be

published in March 2015 - at which time the Government will decide whether to change official dietary guidance.

Along with the new guidance from SACN, officials from Public Health England yesterday launched an investigation into new ways to cut the nation's sugar intake. Experts will consider a range of measures, including curbs on advertising to children, and "fiscal measures", particularly on **sugary drinks** that could reduce consumption.

Other countries have introduced a tax on sugar or sugary products, particularly sweetened drinks. However, the Department of Health said that there were no current plans to introduce a "sugar tax".

Professor MacDonald said that staying within the new five per cent target did not necessarily mean reverting to a "boring, Second World War rationing diet".

Swapping **sugary drinks** for water, chocolate for fruit, sugary cereal for porridge, and puddings for low fat yogurts would help consumers, said Dr Alison Tedstone, chief nutritionist at Public Health England.

She added that there had been little improvement in the population's overall sugar intake in five years, and that obesity was a "very difficult" problem for the UK, with two thirds of adults overweight or obese and one in three children leaving primary school overweight or obese.

"Being a little bit overweight, slightly podgy, is raising your risk of serious diseases including type-2 diabetes which will have potentially devastating effects on you and your families," she said. "It could lead to you going blind and having feet amputated. You also raise your risk of cardiovascular disease and some cancers."

The British Soft Drink Association (BSDA), which represents the majority of manufacturers, including Coca Cola, insisted that soft drinks could be part of a balanced diet.

Gavin Partington, BSDA's director general said: "This year, major companies in the industry are increasing advertising spend on low- and no-calorie drinks by 49 per cent and sales of these products have already increased by five per cent over the last two years."

Healthy eating: Suggested meals

Breakfast

Shreddies with semi-skimmed milk and raisins, two pieces of brown toast with reduced fat polyunsaturated spread and peanut butter, white tea (semi-skimmed milk)

Mid morning

White tea (semi-skimmed milk); apple

Lunch

Jacket potato (reduced fat polyunsaturated spread) baked beans with salad (lettuce, cucumber, tomato) and low fat yoghurt, water

Afternoon snack

White tea (semi-skimmed milk), banana

Evening meal

Fish pie with peas, strawberries and single cream, low calorie fizzy diet drink

Source: Public Health England

LOAD-DATE: June 27, 2014

LANGUAGE: ENGLISH

PUBLICATION-TYPE: Newspaper; Web Publication

JOURNAL-CODE: WEBI

Copyright 2014 Independent Digital News and Media Limited
All Rights Reserved

]

The Independent (London)

June 27, 2014 Friday
First Edition

Away from temptation;
Editorials Private firms cannot be trusted to lead the obesity fight

SECTION: NEWS; Pg. 2

LENGTH: 365 words

There is one British demographic above reproach in its indulgence of a sweet tooth. Children, we accept, lack the kind of self-control to be trusted in front of a fridge full of **fizzy drinks** and juice. Government powers are increasingly invoked to protect them from advertising for the kind of sugary products that lead to tooth decay, hyperactivity, and cheeks a little over-chubby.

The latest advice from health professionals, following a report from the Scientific Advisory Committee on Nutrition (SACN), asks parents to ban fruit juice from the dinner table - replacing it with water. This is a sensible idea, no doubt, but obesity troubles mum and dad too - two-thirds of Britain's population are classed as overweight - and the question remains: should the Government turn "nanny" for an adult population that is glugging and chomping its way towards a condition that places a costly burden on health services, and whittles away at productivity?

In some places, the dividing line between adult and child is no clear binary. All the wisdom that age brings can be useless against the pull of sugary snacks, which stimulate the same area of the brain as drugs and opioids. The basic tenet of the libertarian case against state mollycoddling - that we are in full control of our appetites - has been undermined by recent studies. So it will take more than "better choices" to reach the SACN's target of a 50 per cent reduction in the amount of added sugars consumed by the adult population. One can of **fizzy drink**, under the new guidelines, would hit that limit. So far, the Coalition has trusted to a Responsibility Deal with private manufacturers of sugary and fatty products to lead Britain away from the XL aisle.

The trouble is, many of the worst offenders have not signed up, and even those who have still owe their financial health to food and drink that make us unhealthy. Big Food lobbies intensively for self-regulation and reforms based on personal responsibility. Both of these are important. But the Government should also carefully weigh up the case for greater intervention, up to and including a sugar tax. It's not **fizzy-drink** makers, after all, that have to sustain the NHS.

LOAD-DATE: June 26, 2014

LANGUAGE: ENGLISH

PUBLICATION-TYPE: Newspaper

JOURNAL-CODE: IA

Copyright 2014 Independent Print Ltd
All Rights Reserved

The Independent (London)

June 27, 2014 Friday
First Edition

One can of fizzy drink contains an adult's daily sugar allowance

BYLINE: CHARLIE COOPER HEALTH REPORTER

SECTION: NEWS; Pg. 4

LENGTH: 465 words

Drinking just one can of **fizzy drink** a day is unhealthy, according to new guidance which halves the amount of sugar considered acceptable in adult diets.

In a major shift, government health advisers say that free sugars - found in sweetened drinks, fruit juice and confectionery and added to many processed foods - should make up just 5 per cent of our daily energy intake.

Previous advice set the limit at 10 per cent. Experts say the new guidance is necessary to combat Britain's obesity epidemic and protect children from diabetes, heart disease and tooth decay.

For an adult this would mean a single can of **fizzy drink**, which contains the equivalent of six to seven teaspoons of sugar, is almost the entire allowance for the day.

Government advisers from the Scientific Advisory Committee on Nutrition (SACN) said every population group in England is already exceeding the current 10 per cent limit on sugar consumption.

Professor Ian Macdonald, the chairman of the SACN's working group on cwarbohydrates, which has produced the draft guidance, said there were clear associations between high levels of free sugar intake and overall increased energy intake and obesity. "There is also an association between **sugar**-sweetened **beverages** and type-2 diabetes," he said.

The SACN's report will now be subject to a consultation, and final guidance will be published in March 2015 - at which time the Government will decide whether to change official dietary guidance.

Swapping **sugary drinks** for water, chocolate for fruit and sugary cereal for porridge would help consumers, said Dr Alison Tedstone, the chief nutritionist at Public Health England.

She added that there had been little improvement in the population's overall sugar intake in five years, and that obesity was a "very difficult" problem for the UK, with two-thirds of adults overweight or obese and one in three children leaving primary school overweight or obese.

The British Soft Drinks Association (BSDA), which represents most manufacturers, including Coca-Cola, insisted that soft drinks can be part of a balanced diet.

Gavin Partington, ?the director general of the BSDA, said: "Companies are increasing advertising spend on low- and no-calorie drinks by 49 per cent; sales of these products have increased by 5 per cent over the past two years."

HEALTHY EATING

SUGGESTED MEALS

Breakfast

Shreddies with semi-skimmed milk and raisins, two pieces of brown toast with reduced fat polyunsaturated spread and peanut butter, white tea (semi-skimmed milk)

Mid-morning snack

Tea (semi-skimmed milk); apple

Lunch

Jacket potato (reduced fat polyunsaturated spread) baked beans with salad (lettuce, cucumber, tomato)

Afternoon snack

Tea (semi-skimmed milk), banana

Evening meal

Fish pie with peas; strawberries and single cream

SOURCE: PUBLIC HEALTH ENGLAND

LOAD-DATE: June 26, 2014

LANGUAGE: ENGLISH

PUBLICATION-TYPE: Newspaper

JOURNAL-CODE: IA

Copyright 2014 Independent Print Ltd
All Rights Reserved

MailOnline

June 27, 2014 Friday 2:02 AM GMT

How fruit juice has turned into junk food: Modern methods mean even 'not from concentrate' drinks can be stored for a year

BYLINE: DAVID DERBYSHIRE

SECTION: HEALTH

LENGTH: 1082 words

- Juice was popularised after Second World War as cheap health supplement
- But mass production methods take away much of what makes it healthy
- Juices give many vitamins and minerals - but no fibre and a lot of sugar

There was a time when it was marketed as the ultimate health drink, a glass of sunshine packed with vitamins and energy.

Generations were raised to believe orange juice fights off colds, boosts the immune system, tones the skin and protects against cancer. Yet in the topsy-turvy world of health advice, what's good for you one day, turns out to be bad for you the next.

This week an influential body of Government scientists blamed Britain's love affair with orange juice and other **sugary drinks** for fuelling a crisis of obesity and ill health.

The warning follows calls to remove fruit juice as one of the recommended 'five a day' portions of fruit or vegetables, and for parents to ban it from the meal table.

So if fruit juice turns out to be such a devil in disguise, why have we all been led to believe it was so healthy for so long?

The idea goes back to the 1920s, when American nutritionist Elmer McCollum blamed a condition called acidosis, an excess of acid in the blood, on diets rich in bread and meat. His bizarre solution was lots of lettuce and - paradoxically - acidic citrus fruits.

At the time orange juice was not hugely popular, but fruit growers leapt on the acidosis panic and sales rose.

Juice got an even bigger boost thanks to World War II when the U.S. Government wanted a new way to get a product rich in vitamin C to troops overseas. It poured money into research.

In 1947 - just in time for the post-war consumer boom - scientists invented a way to remove water from juice and freeze the concentrate into a palatable product.

The blocks of this concentrate could be sold to the new fridge-owning U.S. consumers or stored by manufacturers for months at a time, and sales exploded.

Meanwhile in the UK, war babies had been given rose hip, blackcurrant and concentrated orange juice by the Government as a cheap nutrition supplement in the 1940s. This continued into the 1950s, seeding the idea in a generation of baby boomers that juice is healthy.

By the 1980s orange juice was being marketed not just as a health drink, but also as the key to a stylish, modern life - a status it enjoys today.

But while the juice in the supermarket is often sold as 'natural' or 'fresh', it is usually anything but.

Concentrating juice doesn't just remove water, it also removes the flavour. After it has been reconstituted, manufacturers add 'flavour packs' - cocktails of chemicals which restore 'natural' oranginess.

You may think 'not from concentrate' juice means a more authentic product. You'd be wrong.

Juice made that way is heated and stored in air-free tanks for up to a year. Again, the process strips the juice of flavour, which has to be added afterwards. But the flavour packs contain orange essence and orange oil so don't have to appear separately on the ingredients list.

Manufacturers say they help give their product a consistent flavour. They also explain why juice in cartons doesn't taste like fresh juice.

'Naturalness' isn't the only dubious claim made for juice. For decades, health gurus, and some doctors, have claimed the vitamin C in juice fights common colds.

But while the immune system needs vitamin C, there's little evidence that regularly taking the stuff prevents colds.

Research shows that the best that can be claimed for vitamin C is that it might shorten colds by a day or two. Given that most of us get two colds a year, worrying about vitamin C every day seems an over-reaction.

There's another myth about vitamin C, that citrus fruits are the best source. Yet plenty of vegetables, including broccoli, potatoes, kale and peppers, have doses comparable to those in fruit.

What you will get from juice is sugar. Lots and lots of it.

The new advice this week from the Scientific Advisory Committee on Nutrition is that men should have a maximum of 35g of sugar a day - seven to eight teaspoons - while women should not exceed 25g - five to six teaspoons. A single 330ml glass of orange juice has eight teaspoons.

Helen Bond, spokeswoman for the British Dietetic Association, says: 'People have lost sight of how much sugar is in food and portion sizes have got bigger. A 150ml glass provides one of your five a day and anything more than that doesn't count. But measure people's glasses and they are often 250ml.

'Juice provides a lot of vitamins and minerals, but unlike fresh fruit you don't get the healthy fibre.'

Doctors say the huge volume of sugar in our diet is contributing to the obesity epidemic, causing heart disease, cancer and diabetes.

There is growing concern that not all sugars are the same - and that fructose, the type found in fruit, may be more harmful than table sugar. American hormone scientist Robert Lustig argues that it does more damage to the liver and cells than glucose or sucrose. He says excessive fructose intake is key to rising obesity and diabetes levels.

There is even evidence that fructose may contribute to higher uric acid levels in the blood - and increase the risk of gout, an excruciatingly painful condition that is becoming ever more common.

Orange juice can also rot your teeth. Around half of five-year-olds have signs of damage to their tooth enamel, and too much fruit juice is thought to be a key cause.

There have been signs this year, however, that the tide is turning.

Schools have been leading the way. In January, Elizabeth Chaplin, the head of Valence Primary School in Dagenham, London, told parents that pupils would not be allowed juice in their lunch boxes. Instead, they had to drink water.

Around that time, Professor Susan Jebb, the Government's obesity tsar, said juice shouldn't count towards your five a day.

'Fruit juice is absorbed very fast,' she said, 'so by the time it gets to your stomach, your body doesn't know whether it's Coca-Cola or orange juice, frankly. I have to say it is a relatively easy thing to give up. If you are going to drink it, you should dilute it.'

Weaning Britain off fruit juice may be difficult. Market research firm Mintel says 83 per cent of us drink fruit juice or a smoothie at least once a week, while 76 per cent believe fruit juice to be healthy.

But if you need motivation when you sit down to breakfast, remember this: there is more sugar in a 250ml glass of fruit juice than in a large bowl of Frosties with milk.

And that's food for thought.

LOAD-DATE: June 27, 2014

LANGUAGE: ENGLISH

PUBLICATION-TYPE: Web Publication

JOURNAL-CODE: WEBDM

Copyright 2014 Associated Newspapers Ltd.
All Rights Reserved

MailOnline

June 27, 2014 Friday 1:46 AM GMT

Cut sugar to just 5 teaspoons each day: Tough new guidelines... but no help to meet them

BYLINE: SOPHIE BORLAND and EMILY DAVIES

SECTION: HEALTH

LENGTH: 731 words

- Officials urging people to have sugar intake to prevent soaring obesity levels
- Britons average 15 teaspoons per day - 330ml **fizzy drinks** contain seven
- Specialists want Government to force food producers to cut sugar levels
- But Public Health England said it would merely 'consider' these ideas

Officials are urging people to halve their sugar intake to as little as five teaspoons a day to prevent obesity levels continuing to soar.

But they have been accused of failing to take proper action against the food industry to help the public meet the strict targets.

Guidelines from scientists advising Public Health England, the agency given the job of tackling obesity, yesterday stated that women should have no more than five to six teaspoons of sugar a day, and men seven to eight.

A 330ml can of **fizzy drink** contains around seven teaspoonfuls, so would meet this limit on its own.

Currently, Britons consume an average of 15 teaspoons daily, mainly due to the high volumes of sugar hidden in everyday items such as fruit juice, muesli, yoghurts, sandwiches and ready meals.

As reported in yesterday's Mail, experts are advising parents to ban fruit juice, squash and soft drinks from the dinner table and give their children only water and milk.

Senior doctors and academics want the Government to force food manufacturers to cut **sugar** levels, ensure products are clearly labelled and impose a tax on **soft drinks**.

But Public Health England merely said it would 'consider' these ideas as part of a nine-month consultation process that will eventually report to ministers next spring.

PHE simply issued a document suggesting a number of ideas the Government could consider to help the public meet these targets.

These included fruit juice and smoothies being banned from the five-a-day fruit and vegetable guidelines because they are so high in sugar.

The organisation - set up last year to tackle public health issues - also said it would 'explore further approaches' such as adding a tax to soft drinks and banning junk food adverts before the 9pm watershed.

Other plans include discouraging supersize portions at coffee chains, fast food outlets and cinemas.

But ministers last night remained non-committal and said the PHE document would help 'inform' the debate about sugar.

Around one in four adults are considered obese although this is predicted to rise to half the population by 2050, driving up levels of diabetes and heart disease.

Campaigners accused the officials of not doing enough to tackle the obesity epidemic.

Tam Fry, of the National Obesity Forum, said: 'This is all delaying tactics. We've had delaying tactics from this government for years. They're not going far enough.

'We need tough, firm action with the industry. They need to take immediate action rather than sitting on the fence yet again.'

Dr Aseem Malhotra, a cardiologist at Croydon Hospitals, South London, said: 'This doesn't address the availability of sugar everywhere. The food industry have not made any promises to reduce sugar.

'We want this to be translated into something that's meaningful for the public. We want them to slash their sugar levels by 40 per cent within four years.'

The Government has been accused of 'cosying-up' to the food industry and in so doing, failing to address the obesity epidemic.

Its flagship obesity policy, the responsibility deal, allows food firms to set their own targets for cutting sugar, salt and fat and for clearly stating the number of calories that products contain.

It has already emerged that five of the seven members of the Scientific Advisory Committee on Nutrition, which drew up yesterday's guidelines on sugar, have financial ties to the food industry.

They include the chairman, Ian MacDonald, professor of metabolic physiology at Nottingham University, who has worked as a paid adviser for Coca-Cola and Mars and whose research has been funded by Unilever, the world's biggest producer of ice cream.

Others include a consultant for the world's largest chocolate producer, Barry Callebaut, and scientists whose research has been funded by Unilever.

Public Health Minister Jane Ellison said: 'We want to help people make healthier choices and get the nation into healthy habits. This report will inform the important debate taking place about sugar.'

Dr Alison Tedstone, chief nutritionist at Public Health England, said: 'We are very concerned about sugar intakes.'

LOAD-DATE: June 27, 2014

LANGUAGE: ENGLISH

PUBLICATION-TYPE: Web Publication

JOURNAL-CODE: WEBDM

Copyright 2014 Associated Newspapers Ltd.
All Rights Reserved

The Telegraph
telegraph.co.uk

June 27, 2014 Friday 10:31 AM GMT

Children must be taught that eating is a joy;
We all consume too much sugar - but meals and food are about more than calories

BYLINE: By Paul Levy

SECTION: LIFESTYLE

LENGTH: 822 words

We all know that our children have too many sugary, **fizzy drinks**, and common sense, as well as science, tells us that this is a cause of the current obesity crisis. It's not the only one - I'm overweight and I never touch sugary pop, not even tonic with my gin.

Fruit juice is another dietary villain, and Professor Susan Jebb, the Government's chief obesity adviser, says families should be stricter about what their children drink, limiting them to a small glass with breakfast. More interesting and more helpful is the advice she's given about other meals: "Drink water, that's the very simple advice to parents." There has also been talk of banning carbonated, sweetened drinks and even fruit juice in schools. But what's really needed is a change of habits.

At home we follow the French (and American, believe it or not) custom; there is always a jug of water on the table at mealtimes - especially when the principal drink is beer or wine and there are guests. (We live in the country, and our guests have usually driven. It's simply uncivilised, and dangerous, to fail to provide an alternative to alcohol to anyone who is driving.)

Before there were such huge profits to be made from selling bottled water, every restaurant in America used to bring water to the table, even before presenting the menu. Good restaurants here in Britain too still do this, and all good eateries now offer patrons the choice of tap or bottled water.

Progress on the home front, though, is slower, the sad statistics show. As Professor Jebb says, "the biggest source of sugar across all age groups is **sugar**-sweetened **beverages**". Youngsters, and plenty of grown-ups, derive pleasure from these obesity time-bomb bevvies. Instead of thinking of bans, we ought to be thinking about providing alternative, less damaging forms of gratification.

Of course, the first thing to get right is to bring children up to regard the act of eating as an enjoyment, not a chore, in other words to recognise and cherish the pleasures of the table - and not merely regard eating as a nutritional necessity. Our motto should be "food, not fuel", and this means, above all, having structured meals, which are family and social occasions.

This is not only important in the fight against flab, but it is (and should be regarded as) the chief way we civilise our children. It is at table that we teach them, not only that eating is fun, but that it is done with a certain amount of grace and concern for the feelings of others, and accompanied by conversation. What children learn at the family table is as important as what they learn at school; unhappily, this is also true if what they learn at table is to be unsociable or aggressive.

For years we have been told that structured meals - meaning meals that take place at about the same time every day, and with attendance more or less mandatory - are fundamental to good family relations. It has not

been a very British attitude, but surely we can admit to ourselves that this also affects society; that children brought up this way make better citizens. Even takeaway and ready meals can be consumed at a table with some amount of ceremony and fellowship.

A bottle of wine, too, does no harm and in my view, a lot of good. In our non-religious culture we are trying, after all, to teach our children that meals are mostly about delight, not about sacred ritual, duty or digestion. While the scientific details are not yet incontrovertible, it is clear that a glass, or even two, of wine with dinner, is on the whole beneficial to health.

What is unarguable, though, is that wine (or beer or cider) enhances the pleasure we take in eating, and, providing it's of decent quality, is itself a source of enjoyment. It is, also, the very opposite of the binge drinking that has become such a problem in contemporary Britain.

Just as we want to teach our children a modicum of table manners, we should be instilling good drinking habits in our young - at home. Like the French, we should allow children in the 11 to 18 age group (the National Diet & Nutrition Survey 2008-12 shows this cohort gets a whopping 15.6 per cent of its daily food energy from added sugar) to have a couple of drops of wine to colour their water at mealtimes. As they get older we should give them watered-down wine, and by this means teach them to love and appreciate a proper glass of wine with their food as they become adult.

It's all about pleasure. If we forget this, and think of food only in functional terms, as mere calories, we might as well glug Soylent, "the magic food replacement milkshake" now available in the US. It is said to be palatable, but consuming it is a solitary act, devoid of pleasure. That is where our current attitude to food may lead us. Who wants to sit down at a table with friends over a beige mixture of vegetable oils and nutrients?

Paul Levy is chairman of the Oxford Food Symposium

LOAD-DATE: June 27, 2014

LANGUAGE: ENGLISH

PUBLICATION-TYPE: Newspaper; Web Publication

JOURNAL-CODE: WEBDTNS

Copyright 2014 Telegraph Media Group Limited
All Rights Reserved

The Telegraph
telegraph.co.uk

June 27, 2014 Friday 6:56 AM GMT

New sugar limits can be breached by a bar of chocolate;
New proposed recommendations to tackle Britain's love of sugar will mean one bar of chocolate will take many people over their daily limit

BYLINE: By Laura Donnelly Health Editor

New sugar limits can be breached by a bar of chocolate; New proposed recommendations to tackle Britain's love of sugar will mean one bar of chocolate will take many people over their daily limit telegraph.co.uk June 27, 2014 Friday 6:56 AM GMT

SECTION: NEWS

LENGTH: 649 words

A single bar of chocolate, three yogurts or a **fizzy drink** would take the average person over proposed daily sugar limits announced by scientific advisers to the Government.

The recommendations halve current limits, meaning that the average woman should have no more than five to six teaspoons (25g) of sugar a day, and seven to eight teaspoons (35g) for men.

Scientific advisers to Government signalled a war on sugar with draft recommendations that would require radical changes to the British diet.

The new proposed limits mean that many food products that fit within current daily allowances no longer will.

A 330ml can of Coca Cola contains 35g (1.2oz) of sugar, while a Mars bar has 33g (1.16oz) - both well in excess of the average 25g (0.88oz) limit for women and using up the full allowance for men. Three Waitrose low-fat black cherry yogurts provide 27g (0.95oz) in added sugars, exceeding the women's' allowance.

The advice from the Scientific Advisory Committee on Nutrition (SACN) says that no more than five per cent of daily calories should come from sugar - compared with the current limit of 10 per cent.

Health officials say all age groups are exceeding current recommendations, with teenagers on average consuming 50 per cent more sugar than they should. The average adolescent drinks one 330ml can of **fizzy drink** every day.

On Thursday public health officials said families should think carefully about whether children should be allowed **fizzy drinks** at all, and said the public needed to be encouraged to swap such beverages for low-calorie versions, or water.

Dietitians said **fizzy drinks** had "no place" in a healthy diet.

On Wednesday the Government's chief obesity adviser said parents should serve water with meals and ban **fizzy drinks** and juices from the dining table in order to reduce their children's sugar intake. The new proposed guidelines also say Britain needs to boost its intake of fibre from 24 grams (0.8oz) to 30 grams (1.05oz) a day. The recommendations follow a six-year review of 600 scientific studies on consumption of carbohydrates, including sugar. Two thirds of adults in Britain are overweight or obese, and British girls below the age of 20 are now the most overweight in Western Europe.

A separate report by Public Health England outlines a range of measures that it will consider recommending to minsters in an attempt to reduce sugar intake, including taxes on sugary foods, restrictions on advertising and a clampdown on retail promotions relating to sugary foods.

Dr Alison Tedstone, the chief nutritionist at Public Health England, said: "Eating too much sugar is harming our health; excess sugar and calorie intake leads to being overweight and obese, and consequently having a higher risk of developing type 2 diabetes, heart disease and breast and colon cancer."

The report warns that obesity could cost up to £50 billion a year by 2050 if current trends continue.

Experts warned of a health crisis with new figures showing that seven out of 10 people aged 45 and over are overweight or obese. Those below the age of 25 are the only group in which the majority are not overweight, the new statistics from the Health and Social Care Information Centre show.

Tam Fry, of the National Obesity Forum, said: "These figures show that there is a looming health crisis for the nation. There needs to be a clear message that everybody needs to take control and watch what they are eating."

Age UK, the charity, said not enough effort was made to encourage older people to adopt healthier behaviours. Ruthe Isden, from the charity, said: "Historically, public health approaches have been very focused on children, and it's all about getting them while they are young and if you are old then 'it's a bit late

to bother'. There is a really important need to turn some of that around and actually start talking to people about how it's never too late."

LOAD-DATE: June 27, 2014

LANGUAGE: ENGLISH

PUBLICATION-TYPE: Newspaper; Web Publication

JOURNAL-CODE: WEBDTNS

Copyright 2014 Telegraph Media Group Limited
All Rights Reserved

THE TIMES

The Times (London)

June 27, 2014 Friday
Edition 1;
National Edition

Fizzy drinks are target in new war on **sugar**; Shun **soft drinks** to beat obesity

BYLINE: Chris Smyth

SECTION: NEWS; Pg. 1,2

LENGTH: 739 words

Fizzy drinks would be effectively banished from the nation's diets under tough new public health guidelines.

Just one can of a **sugary soft drink** contains an adult's entire daily **sugar** allowance under new limits from government health advisers, who say families need to think carefully about whether children should have **fizzy drinks** at all.

Fruit juice, which unlike whole fruit counts towards the sugar limit, could be removed from "five-a-day" guidance on eating fruit and vegetables.

People should also eat more fibre to protect against heart disease, diabetes and bowel cancer, the Scientific Advisory Committee on Nutrition said after an exhaustive review of 600 scientific studies.

In draft guidance released yesterday, the scientists said we should get no more than 5 per cent of calories from sugar, half the current target. Most people already miss this, getting an average of 11 per cent of their energy from sugar. The new limit is equivalent to five to six teaspoons daily of sugar for a woman (25g) and seven to eight teaspoons (35g) for a man - the same as one 330ml **fizzy drink**.

Professor Ian Macdonald, a member of the advisory committee, said that it was "just not realistic" to meet the targets by having a daily **soft drink**. "If you have that, you can't have any cakes or biscuits, you can't have anything with **sugar** in it," he said.

"One can will use up your allowance, but the message has got to be to reduce consumption of all sorts of foods and drinks. It is possible without it being a boring Second World War rationing diet."

Children will also be targeted by an advertising blitz and a school programme will urge them to shun **soft drinks** and chocolate in favour of water and fruit, as public health chiefs said yesterday there was mounting evidence that **sugar** was one of the key causes of Britain's obesity crisis.

A **sugar** tax, limits on portion sizes, restrictions on advertising of **soft drinks** and bans on discounted chocolates are all being considered by Public Health England in a review that will pile pressure on ministers to drop their opposition to new laws. One in three children is too fat by the time they leave primary school and experts say that too much **sugar** is one of the main reasons, with 15 per cent of children's calories coming from **sugar**. More than a quarter also have tooth decay.

Fizzy drinks were singled out, with teenagers getting a third of their sugar from them. While experts said that sugar was no more likely to make us fat than other calories, Alison Tedstone, chief nutritionist at PHE, said that "there seems to be something about sweetened drinks that encourages overconsumption", perhaps because people did not feel full.

"We are very concerned around sugar intake in England. Every single population group is exceeding the current guideline on sugar. We tend to think of it as a cosmetic problem, only to do with images of grossly overweight people. It's not. Being a little bit overweight or slightly podgy is raising your risk of serious disease."

A slice of cake or a ready meal will also take women over their added sugar limits and Dr Tedstone urged: "Instead of having a **fizzy drink** having water, instead of having a chocolate bar having a piece of fruit, those kinds of things."

Jeremy Hunt, the health secretary, is opposed to a sugar tax, but is coming under sustained pressure to do more from doctors and charities.

John Middleton, vice-president of the Faculty of Public Health said: "We need government to do what only it can and take steps like trialling a duty on **sugary drinks** that have no nutritional value. The public health community is united behind this."

Ministers have relied on efforts to persuade food companies to make healthier products. However, Professor Susan Jebb, who has led those efforts, said that voluntary measures "can only do so much and as a public health researcher I know that the scale of the challenge means we need to consider a much broader range of policies".

Gavin Partington, director general of the British Soft Drinks Association, said: "The soft drinks industry has a strong track record in reformulation and innovation which has dramatically increased the range of low and no calorie options now available to consumers ... All soft drinks can be part of a balanced diet and lifestyle and while they contribute just 3 per cent of calories to the average UK diet, we do recognise that some people are consuming too many calories from them."

LOAD-DATE: June 27, 2014

LANGUAGE: ENGLISH

PUBLICATION-TYPE: Newspaper

JOURNAL-CODE: TIM

Copyright 2014 Times Newspapers Limited
All Rights Reserved

THE TIMES
The Times (London)

June 27, 2014 Friday
Edition 2;
National Edition

Super-size drinks back on the menu New York's

BYLINE: AP

SECTION: NEWS; Pg. 36

LENGTH: 47 words

Albany New York's highest court refused to reinstate the city's ban on large cups of **sugary drinks.** It ruled that the health department overstepped its authority in imposing a size limit of 16oz (453g) on beverages sold at restaurants, delis, cinemas, stadiums and street vendors. (AP)

LOAD-DATE: June 27, 2014

LANGUAGE: ENGLISH

PUBLICATION-TYPE: Newspaper

JOURNAL-CODE: TIM

Copyright 2014 Times Newspapers Limited
All Rights Reserved

THE TIMES
The Times (London)

June 27, 2014 Friday
Edition 1;
Scotland

Analysis

BYLINE: Dr Mark Porter

SECTION: NEWS; Pg. 2

LENGTH: 176 words

There have been numerous studies suggesting that a fondness for **fizzy drinks** can increase your chances of developing a number of serious health problems. But the evidence is often conflicting and hard to interpret - is it the **fizzy drinks** doing the damage, or is it something about the type of person who drinks a lot of them that puts them at risk? Researchers and soft drinks manufacturers are still arguing that point but no sensible person can dispute that most **fizzy drinks** are packed with calories; are of little or no nutritional value; are terrible for your teeth, and that it is children and young people most likely to be harmed.

Even Juices and smoothies can cause problems as they are also full of acids and sugar. The best bet is to substitute water. But children like **fizzy drinks,** and occasional treats - as cola was when I was a child - are fine.

If your children do have **fizzy drinks** regularly, try to persuade them to use straws as research suggests these reduce damage to teeth because liquid is less likely to be swilled around in mouths.

LOAD-DATE: June 27, 2014

LANGUAGE: ENGLISH

PUBLICATION-TYPE: Newspaper

JOURNAL-CODE: TIMscot

Copyright 2014 Times Newspapers Limited
All Rights Reserved

THE TIMES

The Times (London)

June 27, 2014 Friday
Edition 1;
Scotland

Can the sugary fizz to beat obesity, health experts urge

BYLINE: Chris Smyth

SECTION: NEWS; Pg. 2

LENGTH: 455 words

Fizzy drinks would be effectively banished from the nation's diets under tough new public health guidelines.

Just one can of a **sugary soft drink** contains an adult's entire daily **sugar** allowance and government health advisers say families need to think carefully about whether children should have **fizzy drinks** at all.

Fruit juice, which unlike whole fruit counts towards the sugar limit, could be removed from "five-a-day" guidance on eating fruit and vegetables. People should also eat more fibre to protect against heart disease,

diabetes and bowel cancer, the Scientific Advisory Committee on Nutrition said after a review of 600 scientific studies.

In draft guidance released yesterday, the scientists said we should get no more than 5 per cent of calories from sugar, half the current target. Most people get an average of 11 per cent of their energy from sugar. The new limit is equivalent to five to six teaspoons daily of sugar for a woman (25g) and seven to eight teaspoons (35g) for a man - the same as one 330ml **fizzy drink**.

Professor Ian Macdonald, an advisory committee member, said: "One can will use up your allowance."

Children will be targeted by an advertising blitz and a school programme will urge them to shun **soft drinks** and chocolate in favour of water and fruit, as public health chiefs said there was mounting evidence that **sugar** was a key causes of the obesity crisis.

A **sugar** tax, limits on portion sizes, restrictions on advertising of **soft drinks** and bans on discounted chocolates are all being considered by Public Health England. One in three children is too fat by the time they leave primary school and experts say that too much **sugar** is one of the main reasons. A quarter also have tooth decay.

Teenagers get a third of their sugar from **fizzy drinks**. While experts said that sugar was no more likely to make us fat than other calories, Alison Tedstone, chief nutritionist at PHE, said that "there seems to be something about sweetened drinks that encourages overconsumption". "Every single population group is exceeding the current guideline on sugar. We tend to think of it as only to do with grossly overweight people. It's not. Being a little overweight or slightly podgy is raising your risk of serious disease.

She urged: "Instead of having a **fizzy drink** having water, instead of having a chocolate bar having a piece of fruit."

Jeremy Hunt, the health secretary, is opposed to a sugar tax, but is coming under sustained pressure to do more.

Gavin Partington of the British Soft Drinks Association, said: "Soft drinks can be part of a balanced diet and while they contribute just 3 per cent of calories to the average UK diet, we do recognise that some people are consuming too many calories from them."

LOAD-DATE: June 27, 2014

LANGUAGE: ENGLISH

PUBLICATION-TYPE: Newspaper

JOURNAL-CODE: TIMscot

Copyright 2014 Times Newspapers Limited
All Rights Reserved

DAILY MAIL (London)

June 26, 2014 Thursday

BAN FRUIT JUICE AT DINNER TIME

LENGTH: 580 words

HEALTH EXPERTS' WARNING TO PARENTS OVER DANGER OF SWEET DRINKS

BY SOPHIE BORLAND HEALTH CORRESPONDENT

PARENTS should ban juice from the dinner table, experts warned yesterday.

Children should be given only water or milk and should not expect sweet drinks all the time, according to senior government advisers.

They point out that fruit juices are helping fuel sharp rises in obesity rates, type 2 diabetes and heart disease.

A typical teenager consumes 40 per cent more sugar than they should. Adults take in 13 per cent too much.

The advisers say that, while most parents understand **fizzy drinks** are harmful, many wrongly believe that fruit juice is healthy.

Professor Tom Sanders, head of diabetes and nutritional sciences at King's College London, said smoothies should be given only as a treat.

It's not a good idea to wean people on the habit of expecting sweet beverages all the time,' he said. Kids should be getting their fluid from drinking water.

We need to reintroduce the habit of people putting a jug of water on the table and drinking water with their food instead of some sort of fruity beverage.Don't put pop on the table.'

In a study published in the Lancet Diabetes and Endocrinology journal, researchers warned that orange juice is potentially as bad as sugary, sweetened drinks. A 250ml serving contains 115 calories - or seven teaspoons of sugar. A can of full-fat Coke has 139 calories.

Some experts want the Government to stop classing juice as one of our five-a-day' portions of fruit and vegetables.

Susan Jebb, an expert in diet and population health at Oxford University, said that parents should ban all sweetened

drinks in favour of water and milk. The professor, who is the Government's chief adviser on obesity, added: I'd prefer to get sugar out of drinks altogether; a shift to low or no calorie drinks, and preferably water.

The main sources of **sugar** in the diet are **sugar**-sweetened **beverages**.

Milk is fine, low fat milk is fine - but that should be the mainstay of our advice.

The very simple advice for parents is to encourage your children to drink water.

Once they've been weaned, children ought to be drinking water.'

The warnings come ahead of the publication tomorrow of a report by Public Health England that is expected to call for a tax on soft drinks.

The Government body responsible for tackling obesity is likely to recommend a levy of up to 20 per cent - raising the price of a 40p can of Coke to 48p and a 2.5litre carton of fruit juice from £2.50 to £3.

The Government is expected to reject this advice and last week the Health Secretary insisted there were no plans for a sugar tax. Jeremy Hunt said the food industry was successfully lowering sugar levels in products voluntarily under the Government's responsibility deal with firms. But experts have criticised the scheme, saying it is not doing enough to combat rising obesity rates.

Nearly a quarter of adults are considered obese - a proportion that is expected to rise to a half by 2050.

Also tomorrow, the Government's scientific advisory committee on nutrition will publish guidelines on sugar limits. Current recommendations state it should be no more than 12 and 13 teaspoons of sugar a day although the experts may conclude we should aim for much less.

Only last week the Government announced that school milk would be provided for all pupils in the hope of slashing consumption of **fizzy drinks** and fruit juice. It will be free for the under-fives but older pupils will be charged.

© Daily Mail

LOAD-DATE: June 25, 2014

LANGUAGE: ENGLISH

PUBLICATION-TYPE: Papers

Copyright 2014 Associated Newspapers Ltd.
All Rights Reserved

Daily Mirror

June 26, 2014 Thursday
Edition 2;
National Edition

DOCS CALL FOR FRUIT JUICE BAN FOR KIDS

BYLINE: JOSH LAYTON

SECTION: NEWS; Pg. 6

LENGTH: 132 words

FRUIT juice should be banned from the family dinner table to protect children, experts said yesterday.

Parents should stick to water or milk and avoid regularly dishing out sweet drinks, the advice claims.

Government advisors blame fruit juices for rises in obesity, type-2 diabetes and heart disease.

An average teenager consumes 40% more sugar than is healthy, research shows. Experts now want parents to treat fruit juices as they would **fizzy drinks**.

Diabetes expert Prof Tom Sanders, of King's College London, said: "Kids should get their fluid from drinking water.

We need to reintroduce the habit of putting water on the table instead of a fruity beverage.

"Don't put pop on the table."

His advice came after a study in journal The Lancet said orange juice is as unhealthy as sweetened drinks.

LOAD-DATE: June 26, 2014

LANGUAGE: ENGLISH

GRAPHIC: RISKY Child with fruit juice

PUBLICATION-TYPE: Newspaper

JOURNAL-CODE: DMR

Copyright 2014 MGN Ltd.
All Rights Reserved

The Daily Telegraph

The Daily Telegraph (London)

June 26, 2014 Thursday
Edition 1;
Scotland

Official: only water for children

BYLINE: Laura Donnelly

SECTION: NEWS; FRONT PAGE; Pg. 1

LENGTH: 260 words

PARENTS should serve only water with meals and ban fruit juice and **fizzy drinks** from the dining table to reduce children's sugar intake, the Government's chief obesity adviser has said.

The recommendation came ahead of guidance, to be published today, about how much sugar people should consume and proposals to cut consumption.

Prof Susan Jebb, the chairman of the Government's "responsibility deal" with the food and drinks industry, also said that doctors must be less reticent about telling overweight people to diet. She said families should introduce strict rules about **sugary drinks**, limiting juice intake to one small glass a day with breakfast.

"Drink water, that's the very simple advice to parents," she said. "Low-fat milk is fine but water should be the mainstay."

Sugary drinks had a particularly big impact on obesity as children and adults tended to consume them on top of their normal calorie intake from food.

Prof Tom Sanders, the head of diabetes and nutritional sciences at King's College London said families should return to the days of having a jug of water on the table at meal times. "The problem is a lot of people don't drink water any more," he said. "At the dinner table keep it simple; just have water on the table, not pop, not juice, not squash."

Last month a league table of 22 nations found British girls and women under the age of 20 were the most overweight in western Europe, with 29.2 per cent overweight or obese.

An estimated 26 per cent of British men and boys of the same age were overweight and obese, placing them 10th.

LOAD-DATE: June 26, 2014

LANGUAGE: ENGLISH

PUBLICATION-TYPE: Newspaper

JOURNAL-CODE: DTLscot

Copyright 2014 Telegraph Media Group Limited
All Rights Reserved

The Express

June 26, 2014 Thursday
Edition 3;
National Edition

Parents told to ban all juices and give water

BYLINE: Sally Guyoncourt

SECTION: NEWS; Pg. 26

LENGTH: 259 words

FRUIT juice should be banished from the dinner table, health experts announced yesterday.

In a bid to beat the rising tide of obesity and Type 2 diabetes among the young, Government health advisers called for fruit juices, smoothies and fizzy pop to be replaced with plain water.

Professor Tom Sanders, of King's College London, called for all **sugary drinks** to be removed from children's diets.

He said: "Kids should be getting their fluid from drinking water. We need to reintroduce the habit of people putting a jug of water on the table and drinking water with their food instead of some sort of fruity beverage."

He was backed by fellow nutritionist and Government adviser Professor Susan Jebb, from Oxford University, who said: "I'd prefer to get sugar out of drinks altogether; a shift to low or no calorie drinks, and preferably water. The main sources of **sugar** in the diet are **sugar**-sweetened **beverages**. The very simple advice for parents is to encourage your children to drink water."

The typical teenager now consumes 40 per cent more sugar than they should while an adult takes in around 13 per cent too much.

A study published in the Lancet warned that orange juice is potentially as bad as sugary, sweetened drinks. A 250ml serving contains 115 calories while a can of Coke has 139 calories.

Prof Sanders also attacked schools for not providing enough break time for children to eat proper meals, and the bad example shown by celebrity chefs. He said: "You watch the master chefs and they do everything that makes a nutritionist's hair stand on end."

LOAD-DATE: June 26, 2014

LANGUAGE: ENGLISH

PUBLICATION-TYPE: Newspaper

JOURNAL-CODE: DXP

Copyright 2014 Express Newspapers
All Rights Reserved

The Guardian - Final Edition

June 26, 2014 Thursday

Fat? Face up to a life of diets, says obesity tsar: Aim to lose 5% of weight every five years - expert: Give water to children, not sweet drinks, parents told

BYLINE: Sarah Boseley, Health editor

SECTION: GUARDIAN HOME PAGES; Pg. 11

LENGTH: 537 words

People who are overweight may have to resign themselves to a lifetime of strategic dieting with the aim of once every five years losing at least 5% of their bodyweight, according to the government's chief obesity adviser.

Susan Jebb, professor of diet and population health at Oxford University and chair of the government's responsibility deal with the food and drinks industry, says more needs to be done to help people by changing the food environment, including removing fattening foods from prominent positions in supermarkets.

On an individual level, the two-thirds of people who are overweight need to be encouraged by GPs to try to lose weight for the sake of their health, Jebb said.

"We have no qualms in telling people who are smokers that they ought to stop smoking, but we are reticent abut telling people to go on a diet," said Jebb in an interview for a book, The Shape We're In.

The book is published today by Guardian Faber, a day after nutrition experts held a briefing on the impact of **sugar** on health and obesity at the Science Media Centre in London, where they said families should put a jug of water on the table at mealtimes and stop buying **soft drinks** for their children. Jebb said the best approach is for parents to keep **sugary drinks** off the meal table. "Choose something else - drink water. Once they have been weaned, they should be drinking water," she said.

According to Jebb, people who lose weight during a 12-week diet do not put it all back on in the following 12 weeks. It is a gradual process over some years.

She said: "If you think of obesity as a chronic relapsing condition, you could say well maybe every five years you have to diet for 12 weeks - I'm not sure that feels so untenable a position when you think of what we ask people with other chronic diseases to do . . . Say, every five years you have to have a really concerted effort to lose 5% of your bodyweight.

"Maybe that's the price we've got to pay, which is not to say I condone fasting, bingeing, dieting, regain - of course I'm not. Nobody sets out to lose weight and intends to put it back on. I'm just trying to manage people's expectations."

Jebb co-authored a study for Weight Watchers which was influential in a government decision to allow GPs to refer overweight patients to slimming clubs on the NHS. She is also paid for articles and appearances on Rosemary Conley TV.

The responsibility deal, which encourages food and drink companies to pledge to reduce the salt, fat and calories in products, has been much criticised because it is voluntary. Companies are selective in their pledges and some - particularly smaller ones - do not sign up at all.

Jebb recognises the weaknesses of the deal. But she believes it is better than most initiatives elsewhere. "The responsibility deal probably isn't enough, but it is a great deal more than in other countries."

New scientific guidance on carbohydrates in the diet, including sugar, is to be issued by the government's advisory committee on nutrition today.

The Shape We're In: How Junk Food and Diets Are Shortening Our Lives, by Sarah Boseley is published today by Guardian Faber at £12.99. To order a copy for £8.99, visit theguardian.com/bookshop or call 0330 333 6846

LOAD-DATE: June 26, 2014

LANGUAGE: ENGLISH

PUBLICATION-TYPE: Newspaper

Copyright 2014 Guardian Newspapers Limited
All Rights Reserved

theguardian

Guardian.com.

June 26, 2014 Thursday

Limit sugar intake to equivalent of one can of soft drink a day, say advisers

BYLINE: Ian Sampletheguardian.com

LENGTH: 801 words

ABSTRACT

Committee's advice to government recommends that proportion of daily calories from 'free sugar' should be halved to 5%

FULL TEXT

The guideline level of dietary sugar should be slashed by half to the equivalent of one can of **fizzy drink** a day, according to scientists who advise the government.

People should get no more than 5% of their daily calories from so-called "free sugar", a term that includes table sugar, the sugar added to food and drinks, and that found naturally in fruit juices, syrups and honey, they said.

The advice, to be considered by the Department of Health after a three-month consultation, sets an upper limit on free sugar of around 25g or six teaspoons a day for a healthy woman, and around 35g or seven teaspoons for a man.

Across all ages, people in England consume more than the existing guideline of 10% of calories as sugar, with adolescents consuming far more. On average, adults get around 12% of their daily energy from sugar, and for adolescents the figure rises to more than 15%.

Scientists raised particular concerns about **sugary drinks** and urged people to minimise the amount they consumed, where possible swapping to healthier alternatives such as water or low-fat milk. Under the proposed guideline, a single can of **fizzy drink** would contain enough sugar on its own to meet an adult's daily sugar limit.

The recommendation follows a six-year review by the government's scientific advisory committee on nutrition (SACN) of research into the health effects of carbohydrates, which include sugar. The report found that a higher sugar intake was associated with tooth decay and weight gain in children and adults. Being overweight puts people at greater risk of type 2 diabetes, cardiovascular disease and some forms of cancer.

"By reducing to 5% you reduce the risk of all those things to what, from a scientific perspective, would be acceptable. "The challenge is to get there," said Ian Macdonald, chair of SACN's carbohydrate working group. "You have to reduce the consumption of all foods with free sugars, not just one type. It is possible without it being a boring second world war rationing diet."

The proposed 5% limit mirrors draft guidelines published by the World Health Organisation in March.

According to the report, people should get half their daily energy from carbohydrates - often starchy foods, such as potatoes and rice. It goes on to recommend high-fibre diets, with the guideline level for dietary fibre rising to 30g for adults. High-fibre diets seem to reduce the risk of cardiovascular disease, type-2 diabetes and bowel cancer. The report does not recommend changing the well-established advice to eat five portions of fruit and vegetables.

To coincide with the SACN report, Public Health England (PHE) launched its own paper on how to help people reduce their sugar intake. It said it would reconsider its advice on fruit juice and smoothies in its five-a-day campaign.

Alison Tedstone, chief nutritionist at PHE, said: "One reason we're worried about sugar is obesity. We tend to think of obesity as being a cosmetic problem, as only to do with those images we see in the media of grossly obese people, but it's not. Being a little bit overweight, being slightly podgy, is raising your risk of serious diseases including type-2 diabetes, which will have potent devastating effects on you and your family. Also it raises the risk of cardiovascular disease and some cancers.

She said PHE would look across the board at ways to reduce the public's sugar intake, from the possible introduction of a sugar tax to tackling advertising to children.

But changing the nation's eating habits will be a formidable task. Two-thirds of adults in the UK are overweight or obese, as are one in five children at reception age and one in three primary school-leavers. Children from poorer backgrounds are almost twice as likely to be overweight or obese as those from an affluent area.

According to Tedstone, **sugary drinks** are responsible for 30% of children's sugar intake, making them the single largest source. There is some evidence that **sugary drinks** encourage over-consumption, because they do not fill people up in the same way as other foods, she said.

"Our data show that adolescents are on average having a can of **fizzy drink** every single day, and that is part and parcel of the reason why they are exceeding their sugar recommendations . It's certainly an area for families and individuals to think about."

Catherine Collins, principal dietitian at St George's hospital NHS trust said: "Unless you're an elite athlete, **sugary soft drinks** really have no place in your diet. They contribute unnecessary calories to daily intake, and for all age groups, but particularly in the young, they contribute to tooth decay - unless of course you already wear dentures."

LOAD-DATE: June 26, 2014

LANGUAGE: ENGLISH

PUBLICATION-TYPE: Newspaper

Copyright 2014 Guardian News and Media Limited or its affiliated companies
All Rights Reserved
Registered in England and Wales. No. 908396
Registered office: PO Box 68164, Kings Place, 90 York Way, London N1P 2AP

i-Independent Print Ltd

June 26, 2014
First Edition

Children should drink only water at mealtimes; HEALTH

BYLINE: Charlie Cooper HEALTH REPORTER

SECTION: NEWS; Pg. 20

LENGTH: 150 words

Parents should replace juice and **fizzy drinks** with jugs of water at mealtimes to reduce children's sugar intake and cut their risk of obesity, high blood pressure and diabetes, experts say.

Sugar-sweetened drinks were the biggest source of sugar intake across all ages, but were a particular problem in children and teenagers, senior health scientists said.

Squash and pop should be considered treats, they said, adding that, along with milk for young children, "just a jug of water" was all families should be serving at dinner.

Despite recent calls for a tax on **sugary drinks** and cuts to sugar content in everyday foods, Susan Jebb, a professor of diet and population health at Oxford University, said parents could play an even bigger part in improving children's diets. "Drink water - that's the very simple advice to parents," she said.

"Once they've been weaned, children ought to be drinking water."

LOAD-DATE: June 25, 2014

LANGUAGE: ENGLISH

PUBLICATION-TYPE: Newspaper

JOURNAL-CODE: III

Copyright 2014 Independent Print Limited
All Rights Reserved

The INDEPENDENT
Independent.co.uk

June 26, 2014 Thursday 7:20 PM GMT

Britons told to halve their sugar intake to be obesity

BYLINE: Charlie Cooper

SECTION: FRONTPAGE

LENGTH: 780 words

People should halve their daily intake of sugar to combat Britain's obesity epidemic and protect children from diabetes, heart disease and tooth decay, Government health advisors have said.

In a potentially major shift to official dietary advice, experts said that free sugars - those found in sweetened drinks, fruit juice, confectionery, and added to a wide range of processed foods - should make up just five per cent of our daily energy intake.

For an adult this would mean that a single can of a typical **soft drink** such, which contains the equivalent of six to seven teaspoons of **sugar**, would represent almost their entire healthy allowance for the day.

Given the widespread use of free sugars in everything from ready meals to canned soup, this means that, realistically, an adult could no longer drink a can of Coca Cola or similar drink and stay within their daily allowance, experts said.

Government advisors from the Scientific Advisory Committee on Nutrition (SACN) said that every single population group in England is already exceeding the current 10 per cent limit on sugar consumption.

Professor Ian MacDonald, chairman of SACN's working group on carbohydrates, which has produced the draft guidance, said there were clear associations between high levels of free sugar intake and overall increased energy intake and obesity.

"There is also an association between **sugar**-sweetened **beverages** and type-2 diabetes," he said. "In children there is clear demonstration that **sugar**-sweetened **beverages** are associated with obesity. By reducing it to five per cent you would reduce the risk of all of those things. The challenge will be to get there."

The term "free sugars" does not include those found in fruit and milk - but does encompass sugars added to food by the manufacturer, cook or consumer, and sugars naturally present in syrups and unsweetened fruit juices.

Public health minister Jane Ellison said that the new advice confirmed "eating too much sugar can have a significant impact on health". SACN's report will now be subject to a consultation, and final guidance will be published in March 2015 - at which time the Government will decide whether to change official dietary guidance.

Along with the new guidance from SACN, officials from Public Health England yesterday launched an investigation into new ways to cut the nation's sugar intake. Experts will consider a range of measures, including curbs on advertising to children, and "fiscal measures", particularly on **sugary drinks** that could reduce consumption.

Other countries have introduced a tax on sugar or sugary products, particularly sweetened drinks. However, the Department of Health said that there were no current plans to introduce a "sugar tax".

Professor MacDonald said that staying within the new five per cent target did not necessarily mean reverting to a "boring, Second World War rationing diet".

Swapping **sugary drinks** for water, chocolate for fruit, sugary cereal for porridge, and puddings for low fat yogurts would help consumers, said Dr Alison Tedstone, chief nutritionist at Public Health England.

She added that there had been little improvement in the population's overall sugar intake in five years, and that obesity was a "very difficult" problem for the UK, with two thirds of adults overweight or obese and one in three children leaving primary school overweight or obese.

"Being a little bit overweight, slightly podgy, is raising your risk of serious diseases including type-2 diabetes which will have potentially devastating effects on you and your families," she said. "It could lead to you going blind and having feet amputated. You also raise your risk of cardiovascular disease and some cancers."

The British Soft Drink Association (BSDA), which represents the majority of manufacturers, including Coca Cola, insisted that soft drinks could be part of a balanced diet.

Gavin Partington, BSDA's director general said: "This year, major companies in the industry are increasing advertising spend on low- and no-calorie drinks by 49 per cent and sales of these products have already increased by five per cent over the last two years."

Healthy eating: Suggested meals

Breakfast

Shreddies with semi-skimmed milk and raisins, two pieces of brown toast with reduced fat polyunsaturated spread and peanut butter, white tea (semi-skimmed milk)

Mid morning

White tea (semi-skimmed milk); apple

Lunch

Jacket potato (reduced fat polyunsaturated spread) baked beans with salad (lettuce, cucumber, tomato) and low fat yoghurt, water

Afternoon snack

White tea (semi-skimmed milk), banana

Evening meal

Fish pie with peas, strawberries and single cream, low calorie fizzy diet drink

Source: Public Health England

LOAD-DATE: June 27, 2014

LANGUAGE: ENGLISH

PUBLICATION-TYPE: Newspaper; Web Publication

JOURNAL-CODE: WEBI

Copyright 2014 Independent Digital News and Media Limited
All Rights Reserved

The INDEPENDENT
Independent.co.uk

June 26, 2014 Thursday 12:50 PM GMT

Drink water to cut obesity, health experts say

BYLINE: Charlie Cooper

SECTION: FRONTPAGE

LENGTH: 549 words

Parents should replace juice and **fizzy drinks** with jugs of water at mealtimes, diet experts have advised, to reduce their children's sugar intake and cut their risk of obesity, high blood pressure and diabetes.

Senior health scientists said today that sugar-sweetened drinks were the biggest source of sugar intake across all ages, but were a particular problem among children and teenagers.

Despite recent calls for a tax on **sugary drinks** and cuts to sugar content in everyday foods, Susan Jebb, professor of diet and population health at the University of Oxford, said that "very simple advice" to parents could play an even bigger part in improving children's diets.

"Drink water, that's the very simple advice to parents," she said. "Encourage your children to drink water. Once they've been weaned, children ought to be drinking water...There is a whole range of drinks out there, [but] I don't need to encourage people to be drinking any of the others. I can firmly stick to encouraging people to getting their fluid from water."

Around one in five children in the UK are obese, while roughly one third are overweight.

Sugary drinks were a particularly important "target" for combating the problem, Professor Jebb said, with clear links between their consumption and the risk of weight gain.

"We also have a plausible mechanism for that effect because liquid calories are known to be less satiating than solid calories - they fill you up less," she said.

Public health officials will issue new advice on sugar reduction tomorrow, as well as new guidance on foods high in carbohydrates such as bread and rice.

Professor Jebb said that "action" needed to be taken on **sugary drinks**, both "classic carbonated **beverages**", and also energy drinks and sports drinks. She added that a tax on **sugary drinks** was a feasible option but insisted that more focus should go on encouraging people to change their eating and drinking behaviour.

Professor Tom Sanders, head of diabetes and nutritional sciences at Kings College London, said that there was a particular problem with the marketing of **sugary drinks** - particularly during sporting events. Among sponsors for the World Cup in Brazil for instance, Coca Cola is an official corporate partner of FIFA, while McDonalds is an official sponsor of the World Cup.

He said such marketing gave the impression that drinks high in sugar could support an active, sporty lifestyle and also warned over the sugar content of many drinks marketed as sports or energy drinks.

"We have the World Cup going on at the moment. Some of these energy drinks are extremely high in sugar containing between 60 and 70 grams of sugar in a single bottle," he said. "That is a problem we are going to have to deal with - how these foods are marketed and how they are presented."

He highlighted Lucozade's Melonade, which contains 47 grams of sugar per 380ml serving - more than half an adult's daily guideline allowance - and is also available in 500ml and 1l bottles.

Professor Sanders agreed that parents should be "weaning" children off squash and pop which he said should be considered a treat and not an alternative to water.

"A glass of orange juice at breakfast is okay, but on the dinner table, [families should have] just a jug of water and maybe milk for younger children," he said.

LOAD-DATE: June 27, 2014

LANGUAGE: ENGLISH

PUBLICATION-TYPE: Newspaper; Web Publication

JOURNAL-CODE: WEBI

Copyright 2014 Independent Digital News and Media Limited
All Rights Reserved

The INDEPENDENT

The Independent (London)

June 26, 2014 Thursday
First Edition

Serve water at dinner to keep children healthy, parents told

BYLINE: CHARLIE COOPER HEALTH REPORTER

SECTION: NEWS; Pg. 16

LENGTH: 230 words

Parents should replace juice and **fizzy drinks** with jugs of water at mealtimes to reduce children's sugar intake and cut their risk of obesity, high blood pressure and diabetes, diet experts have advised.

Senior health scientists said yesterday that sugar-sweetened drinks were the biggest source of sugar intake across all ages, but were a particular problem among children and teenagers. Drinks such as squash and pop should be considered treats, they said, adding that, along with milk for young children, "just a jug of water" was all families should be serving at dinner.

Despite recent calls for a tax on **sugary drinks** and cuts to sugar content in everyday foods, Susan Jebb, a professor of diet and population health at Oxford University, said that "very simple advice" to parents could play an even bigger part in improving children's diets.

"Drink water - that's the very simple advice to parents," she said. "Encourage your children to drink water. Once they've been weaned, children ought to be drinking water??? There is a whole range of drinks out there, [but] I don't need to encourage people to be drinking any of the others."

Around one in five children in the UK is obese, while roughly one-third are overweight. **Sugary drinks** were an important "target" for combating the problem, Professor Jebb said, with clear links between their consumption and the risk of weight gain.

LOAD-DATE: June 25, 2014

LANGUAGE: ENGLISH

PUBLICATION-TYPE: Newspaper

JOURNAL-CODE: IA

Copyright 2014 Independent Print Ltd
All Rights Reserved

MailOnline

June 26, 2014 Thursday 7:19 PM GMT

Court won't reinstate New York City's big-soda ban

BYLINE: ASSOCIATED PRESS

SECTION: AP

LENGTH: 682 words

NEW YORK (AP) - Big sodas can stay on the menu in the Big Apple after New York state's highest court refused Thursday to reinstate the city's first-of-its-kind size limit on **sugary drinks**. But city officials suggested they might be willing to revisit the supersize-soda ban.

The Court of Appeals found that the city Board of Health overstepped its bounds by imposing a 16-ounce cap on **sugary beverages** sold in restaurants, delis, movie theaters, stadiums and street carts. The appointed board tread on the policy-making turf of the elected City Council, the court said.

"By choosing among competing policy goals, without any legislative delegation or guidance, the board engaged in lawmaking," the court wrote in a majority opinion. "... Its choices raise difficult, intricate and controversial issues of social policy."

Indeed, debate over the soda size cap pitted health officials who called it an innovative anti-obesity tool against critics who considered it unfair to businesses and paternalistic toward consumers. Even a Court of Appeals judge, during arguments earlier this month, wondered aloud whether regulators would target triple-decker burgers next.

The American Beverage Association, which led the legal fight against the measure, welcomed Thursday's ruling against a measure it said would have "limited New Yorkers' freedom of choice." Curbing obesity should start "with education - not laws and regulation," spokesman Christopher Gindlesperger said.

But city leaders signaled they might not give up the fight. Mayor Bill de Blasio said the city was "actively reviewing all of its options to protect the health and well-being of our communities"; officials wouldn't immediately specify what those might be. The city hasn't said whether it plans to try to appeal, but the case doesn't raise federal issues that would make it a likely choice for the Supreme Court.

City Council Speaker Melissa Mark-Viverito said in a statement that the big-soda ban would get a hearing if it were brought up in the council. It's unclear how any such measure might fare in a vote, as she and numerous other council members oppose it.

New Yorkers, meanwhile, greeted Thursday's ruling with mixed feelings.

"I think it's up to the individual" what size of soft drink to buy, said Constance Jong, a cashier in her 20s.

But Hazel Plunkett, a 51-year-old fundraiser for a public health group, was disappointed that the regulation remains blocked.

"I don't mind the controversy over it. It's got to get people's attention," she said.

Soda has been under fire for years from health advocates, who say the beverages are uniquely harmful because people don't realize how much sugar they're guzzling. A 21-ounce Coke, McDonald's medium size, has 200 calories and 55 grams of sugar, for instance.

Amid the publicity, U.S. soda sales have dropped for nearly a decade. But consumption of other **sugary beverages**, such as sports drinks and energy drinks, has grown.

Former Mayor Michael Bloomberg made the soda ban a signal piece of his assertive public-health agenda.

"Due to today's unfortunate ruling, more people in New York City will die from obesity-related impacts," he said in a statement Thursday.

A trial court struck down the measure days before it was to take effect last year. Some eateries had already swapped out cups and adjusted menus to comply, and some proprietors decided to stick with the changes. But others were glad Thursday that they hadn't.

"I thought it was ridiculous" - and unfair, said midtown Manhattan cafe manager Young Shin, 30. The measure would have applied to his workplace but not to bars and groceries under state regulation, including 7-Eleven, the home of the Big Gulp.

Lawmakers and health advocates around the country have proposed soda taxes in recent years, but none has succeeded. A California measure that would have slapped warning labels on sodas was recently defeated.

Meanwhile, Coke and Pepsi have rolled out smaller cans and bottles, some as small as 7.5 ounces.

___

Klepper reported from Albany. Associated Press writers Candice Choi and Rachelle Blidner contributed to this report.

LOAD-DATE: June 26, 2014

LANGUAGE: ENGLISH

PUBLICATION-TYPE: Web Publication

JOURNAL-CODE: WEBDM

Copyright 2014 Associated Newspapers Ltd.

All Rights Reserved

MailOnline

June 26, 2014 Thursday 5:42 PM GMT

Just ONE can of fizzy drink should breach new daily sugar limit, experts say - in report already attacked for being 'toothless'

BYLINE: ANNA HODGEKISS

SECTION: HEALTH

LENGTH: 981 words

- Amount that added sugar contributes to calorie intake should be h alved
- Under new guidelines proposed today, maximum intake would be 25g (5 teaspoons) of sugar for women and 35g (seven teaspoons) for men
- Single 330ml can of fizzy drink contains around seven teaspoonfuls
- But there are fears the Government will be pressured by sugar lobby - especially given the recent backing down over minimal alcohol pricing

Just one can of fizzy drink will contain an adult's entire daily ration of sugar, under new guidelines to be considered by the Department of Health.

The amount that added sugar contributes to calorie intake should be slashed by half, according to scientists advising the Government.

Free sugars are those added to food or contained in fruit juices, honey, syrups and sweetened drinks. They do not include the sugars locked inside fresh fruits and vegetables, and milk.

Too much sugar in the diet is known to be one of the primary reasons for the obesity epidemic and rising rates of Type 2 diabetes. Free sugar also poses a serious threat to dental health.

A draft report from the Scientific Advisory Committee on Nutrition (SACN) urges a reduction from the current recommendation of 10 per cent of calorie intake to five per cent.

Under the new sugar guidelines proposed today, the maximum intake would be 25g (5 teaspoons) of sugar for women and 35g (seven teaspoons) for men.

A single 330ml can of fizzy drink contains around seven teaspoonfuls, and so would meet this limit on its own.

Professor Ian Macdonald, who chaired the SACN carbohydrates working group, said: 'One can will use up your 5 per cent - but the message has got to be to reduce consumption of all sorts of foods and drinks.

'It is possible without it being a boring Second World War type of diet.'

But the report has already been attacked for having no clout - and there are fears it will simply be ignored by food and drink manufacturers - and not enforced by the Government.

It faced huge criticism earlier this year for 'dancing to the tune of drinks industry' over alcohol pricing after a BMJ investigation revealed 130 meetings had been held between health officials, ministers and supermarket lobbyists.

David Cameron had backed plans to impose a minimum price per unit of alcohol, and then dropped the policy in July last year over a lack of 'concrete evidence' it would reduce harmful drinking.

Commenting on today's report, Professor Graham MacGregor, chairman of the group Action on Sugar, said: '[We have] recently provided (Health Secretary) Jeremy Hunt with a simple seven-step programme to tackle childhood obesity.

'He must start by setting targets for reducing **sugar** in **soft drinks** this summer and move responsibility for nutrition to an independent body such as the Food Standards Agency.

'This will mean the soft drinks and food industry are given a level-playing field, with the threat of regulation to ensure the whole of the food industry comply.'

SLASHING SUGAR INTAKE

Under the new sugar guidelines proposed today, the maximum intake would be 25g (5 teaspoons) of sugar for women and 35g (seven teaspoons) for men.

A single 330ml can of **fizzy drink** contains around seven teaspoonfuls, and so would meet this limit on its own.

The nutrition experts who compiled the report came to their conclusions after reviewing thousands of research papers from scientists investigating the health effects of sugar.

The guidance will go out for public consultation before a final report due to be published in March 2015.

The scientists also recommended an increase in daily total fibre intake from around 18 grams to 30 grams for adults.

Evidence shows that fibre improves bowel health and can help prevent heart disease.

Carbohydrates should make up half the population's daily calorie intake, as recommended at present, said the committee.

Public Health England (PHE) said it was rising to the sugar challenge by ramping up its health campaigns and calling on consumers, food companies, retailers and other stakeholders to collaborate in tackling the issue.

Dr Alison Tedstone, chief nutritionist at Public Health England, stressed that for the time being, the current recommendation that free sugar should make up 10 per cent of daily calories would remain in force.

She said: "We are very concerned about sugar intakes. If you look at the dietary data you see that every single population group in England is exceeding the recommended intake for sugar.

"One of the reasons we're worried is because of obesity. We tend to think of obesity as being a cosmetic problem, only to do with those images we see of grossly obese people. It's not. Being a little bit overweight or podgy increases your risk of metabolic diseases.

'The situation in the UK is very difficult. We have over two thirds of the adult population being overweight or obese. One in five four to five-year-olds are obese or overweight. By the time they leave primary school, one in three are obese or overweight.'

She added that 28 per cent of five-year-olds suffered from tooth decay in England and **soft drinks** account for about 30 per cent of children's **sugar** intake.

Manufacturers' claims that sweetened drinks only make up some 3 per cent of sugar consumption were based on the whole population, not just children.

Dr Ann Prentice, chair of the SACN and director of human nutrition research at the Medical Research Council, said: "There is strong evidence in the report to show that if people were to have less free sugars

and more fibre in their diet they would lower their risk of cardiovascular disease, Type 2 diabetes and bowel cancer.

'For their health, people need to consume a balanced diet which includes carbohydrate-rich foods that are low in free sugars and high in fibre.'

LOAD-DATE: June 26, 2014

LANGUAGE: ENGLISH

PUBLICATION-TYPE: Web Publication

JOURNAL-CODE: WEBDM

Copyright 2014 Associated Newspapers Ltd.
All Rights Reserved

MailOnline

June 26, 2014 Thursday 12:16 PM GMT

SUGAR GUIDELINE 'SHOULD BE HALVED'

BYLINE: PRESS ASSOCIATION

SECTION: PA

LENGTH: 171 words

One can of **fizzy drink** will contain an adult's entire daily ration of sugar under new guidelines to be considered by the Department of Health.

The amount "free sugar" contributes to dietary calories should be slashed by half, according to scientists advising the Government.

A draft report from the Scientific Advisory Committee on Nutrition (SACN) urges a reduction from the current recommendation of 10% of dietary energy intake to 5%.

This is the equivalent of 25 grams of sugar for women and 35 grams, or seven to eight teaspoonfuls, for men.

A single 330ml can of **fizzy drink** contains around seven teaspoonfuls, and so would meet this limit on its own.

Professor Ian Macdonald, who chaired the SACN carbohydrates working group, said: "One can will use up your 5%, but the message has got to be to reduce consumption of all sorts of foods and drinks.

"It is possible without it being a boring Second World War type of diet."

The guidance will go out for public consultation before a final report due to be published in March 2015.

LOAD-DATE: June 26, 2014

LANGUAGE: ENGLISH

PUBLICATION-TYPE: Web Publication

JOURNAL-CODE: WEBDM

Copyright 2014 Associated Newspapers Ltd.

All Rights R

MailOnline

June 26, 2014 Thursday 9:07 AM GMT

Ban fruit juice at dinner time: Health experts' warning to parents over danger of sweet drinks

BYLINE: SOPHIE BORLAND

SECTION: HEALTH

LENGTH: 597 words

- Children should be given only water or milk, government advisers say
- Many parents wrongly believe that fruit juice is healthy, they say
- A typical teenager consumes 40 per cent more sugar than they should

Parents should ban juice from the dinner table, experts warned yesterday.

Children should be given only water or milk and should not expect sweet drinks all the time, according to senior government advisers.

They point out that fruit juices are helping fuel sharp rises in obesity rates, type 2 diabetes and heart disease.

A typical teenager consumes 40 per cent more sugar than they should. Adults take in 13 per cent too much.

The advisers say that, while most parents understand **fizzy drinks** are harmful, many wrongly believe that fruit juice is healthy.

Professor Tom Sanders, head of diabetes and nutritional sciences at King's College London, said smoothies should be given only as a treat.

'It's not a good idea to wean people on the habit of expecting sweet beverages all the time,' he said. 'Kids should be getting their fluid from drinking water.

'We need to reintroduce the habit of people putting a jug of water on the table and drinking water with their food instead of some sort of fruity beverage. Don't put pop on the table.'

In a study published in the Lancet Diabetes and Endocrinology journal, researchers warned that orange juice is potentially as bad as sugary, sweetened drinks.

A 250ml serving contains 115 calories - or seven teaspoons of sugar.

A can of regular Coke has 139 calories.

Some experts want the Government to stop classing juice as one of our 'five-a-day' portions of fruit and vegetables.

Susan Jebb, an expert in diet and population health at Oxford University, said that parents should ban all sweetened drinks in favour of water and milk.

The professor, who is the Government's chief adviser on obesity, added: 'I'd prefer to get sugar out of drinks altogether; a shift to low or no calorie drinks, and preferably water.

'The main sources of **sugar** in the diet are **sugar**-sweetened **beverages**.

'Milk is fine, low-fat milk is fine - but that should be the mainstay of our advice.

'The very simple advice for parents is to encourage your children to drink water.

'Once they've been weaned, children ought to be drinking water.'

The warnings come ahead of the publication tomorrow of a report by Public Health England that is expected to call for a tax on soft drinks.

The Government body responsible for tackling obesity is likely to recommend a levy of up to 20 per cent - raising the price of a 40p can of Coke to 48p and a 2.5litre carton of fruit juice from £2.50 to £3.

The Government is expected to reject this advice and last week the Health Secretary insisted there were no plans for a sugar tax.

Jeremy Hunt said the food industry was successfully lowering sugar levels in products voluntarily under the Government's responsibility deal with firms.

But experts have criticised the scheme, saying it is not doing enough to combat rising obesity rates.

Nearly a quarter of adults are considered obese - a proportion that is expected to rise to a half by 2050.

Also tomorrow, the Government's scientific advisory committee on nutrition will publish guidelines on sugar limits.

Current recommendations state it should be no more than 12 and 13 teaspoons of sugar a day although the experts may conclude we should aim for much less.

Only last week the Government announced that school milk would be provided for all pupils in the hope of slashing consumption of **fizzy drinks** and fruit juice. It will be free for the under-fives but older pupils will be charged.

LOAD-DATE: June 26, 2014

LANGUAGE: ENGLISH

PUBLICATION-TYPE: Web Publication

JOURNAL-CODE: WEBDM

Copyright 2014 Associated Newspapers Ltd.
All Rights Reserved

Mirror
mirror.co.uk

June 26, 2014 Thursday 9:07 AM GMT

Fruit juice 'should be banned from family dinner table and replaced with water to protect kids'; Parents should stick to water or milk and avoid regularly dishing out sweet drinks, the advice claims

BYLINE: By Josh Layton

SECTION: NEWS,UK NEWS

LENGTH: 131 words

Fruit juice should be banned from the family dinner table to protect children, experts have said.

Parents should stick to water or milk and avoid regularly dishing out sweet drinks, the advice claims.

Government advisors blame fruit juices for rises in obesity, type-2 diabetes and heart disease.

An average teenager consumes 40% more sugar than is healthy, research shows.

Experts now want parents to treat fruit juices as they would **fizzy drinks**.

Diabetes expert Prof Tom Sanders, of King's College London, said: "Kids should get their fluid from drinking water.

"We need to reintroduce the habit of putting water on the table instead of a fruity beverage.

"Don't put pop on the table."

His advice came after a study in journal The Lancet said orange juice is as unhealthy as sweetened drinks.

LOAD-DATE: September 1, 2014

LANGUAGE: ENGLISH

PUBLICATION-TYPE: Newspaper; Web Publication

JOURNAL-CODE: WEBDMI

Copyright 2014 Trinity Mirror, Plc.
All Rights Reserved

The Telegraph
telegraph.co.uk

June 26, 2014 Thursday 10:00 PM GMT

Juice could no longer count towards 'five a day'; Health officials are considering changes so fruit juice no longer counts towards 'five a day' amid concerns that it is fuelling Britain's obesity epidemic

BYLINE: By Laura Donnelly Health Editor

SECTION: NEWS

LENGTH: 404 words

Fruit juice could be removed from official "five a day" recommendations in a bid to reduce the nation's sugar intake, health officials have said.

Recommended daily limits on sugar intake will be halved under new advice to Government which says no more than five per cent of calories should come from sugar.

It follows concern that Britain's obesity crisis is being fuelled by consumption of fruit juices, smoothies and **soft drinks**, which between them account for the greatest share of **sugar** intake among children and teenagers.

Public health officials said on Thursday that they will "reconsider" the current advice that fruit juice counts as a maximum of one portion a day, while a smoothie can count as more than one portion, depending on its contents.

The report by Public Health England states: "Fruit juices can be major providers of sugar for some people, particularly children aged under 11 years. Smoothies are also high in sugar from fruit and are popular with consumers as they may count as more than one portion of your "five a day".

Officials will publish their new recommendations early next year.

Earlier this year, Prof Susan Jebb, the Government's chief obesity advisor, said she would support removing fruit juices from "five a day" and encouraging people to eat a piece of fruit instead.

Investigations by the Telegraph have identified some smoothies with at least three times the new recommended limit, which equates to around 6 to 7 teaspoons of sugar for women, and 7 to 8 teaspoons for men.

Many fruit juices sold by major brands provide 6 to 7 teaspoons per serving - meaning the daily limits would be reached without any other sugar in the diet.

Health officials also pledged to take on a "supersize culture" which has seen portion sizes soar over the years.

Public Health England said it would seek changes from manufacturers so that smaller portions of chocolate bars and **fizzy drinks** become the "default" on offer, and will draw up measures to restrict promotion of unhealthy foods.

Officials are considering a raft of measures, including taxes on sugary foods, restrictions on advertising, including online, and bans on "upselling" of products - so that customers are asked whether they want to "supersize" their purchase, or add an additional product, such as chocolate or cake.

Local authorities could introduce bans on vending machines selling unhealthy snacks in leisure centres, under the proposals.

LOAD-DATE: June 27, 2014

LANGUAGE: ENGLISH

PUBLICATION-TYPE: Newspaper; Web Publication

JOURNAL-CODE: WEBDTNS

Copyright 2014 Telegraph Media Group Limited
All Rights Reserved

The Telegraph
telegraph.co.uk

June 26, 2014 Thursday 9:12 PM GMT

Ban all drinks but water from dinner table, parents told; Britain's war on obesity must start by limiting **sugary drinks** to special occasions, says Government's chief obesity adviser

BYLINE: By Laura Donnelly Health Editor

SECTION: NEWS

LENGTH: 877 words

Latest: Proposed recommendations will mean one bar of chocolate will take many people over their daily sugar limit

Parents should only serve water with meals and ban **fizzy drinks** and juices from the dining table in order to reduce their children's intake of sugar, the Government's chief obesity adviser has said.

The recommendation was made ahead of scientific advice being published about how much sugar people should consume and proposed measures to reduce public levels of consumption.

Read: How to encourage your children to drink water

Prof Susan Jebb, chairman of the Government's responsibility deal with the food and drinks industry, also said doctors needed to be less reticent about telling overweight people to diet.

She said families should introduce strict rules about such drinks, limiting juice intake to one small glass a day with breakfast.

"Drink water, that's the very simple advice to parents," she said. "Encourage your children to stick with water. Low-fat milk is fine but water should be the mainstay," she added.

Prof Jebb said **sugary drinks** had a particularly significant impact on obesity because children and adults tended to consume them in addition to their calorie intake from food, not in place of solid foods.

"The biggest source of sugar across all age groups is **sugar**-sweetened **beverages** and those are an obvious target for action," she added.

The professor of diet and population health at Oxford University made the comments amid rising concern that sugar has become one of the greatest threats to health, fuelling an obesity time bomb and contributing to spiralling levels of diabetes.

Her remarks were backed by Prof Tom Sanders, the head of the Diabetes and Nutritional Sciences Division at King's College London, who said families needed to return to the days of having a jug of water on the table at all meal times.

He said: "The problem is a lot of people don't drink water any more. At the dinner table keep it simple; just have water on the table - not pop, not juice, not squash."

He said parents should stop buying soft drinks and squash cordials, and instead see them as an occasional treat.

Last month a national study found that children and teenagers are consuming around 40 per cent more added sugar than the recommended daily allowance - with fruit juices and **fizzy drinks** the chief culprit.

Two thirds of adults in Britain are overweight or obese, and British girls below the age of 20 are now the most overweight in western Europe.

In an interview, Prof Jebb said GPs needed to do more to raise the issue with overweight patients.

"I think maybe we are a bit too reticent about telling them that maybe they should go on a diet," she said. "We have no qualms in telling people who are smokers that they ought to stop smoking, but we are reticent about telling people to go on a diet.

"Part of the reticence comes out of the sense that many diets fail or, more accurately, many people who try to diet fail. But many people who try to give up smoking fail. It doesn't mean they shouldn't have another go," Prof Jebb said.

She suggested those who are overweight should set themselves an aim of once every five years losing at least 5 per cent of their bodyweight - accepting that some of the weight lost was likely to creep back on.

Prof Sanders also said Britain could learn from the French, by restoring more structured meal times, instead of snacks on the go.

"We need to come to terms with more structured eating and rethink the way we eat," he said.

"The French talk about food far more and spend far more time eating but actually have far less obesity so you can actually enjoy your food .. but its about eating less."

He said Britain's culture of "24/7 snacking" and "eating on the hoof" was fuelling an obesity epidemic, which has left British girls the most overweight in Europe.

On Thursday two reports will be published, setting out new advice from the Government's Scientific Advisory Committee on Nutrition's on recommended sugar limits, alongside a raft of proposals by Public Health England (PHE) about how to help people reduce their sugar intake.

The quango is considering options including targets to reduce sugar content in some foods, as was introduced with salt, increased restrictions on advertisements for processed foods and a tax on sugary products.

Ministers have already indicated that they would rule out such taxes until there is more evidence from other countries that they make a significant impact. A draft version of the PHE paper said **sugary drinks** have

been described as a "low hanging fruit" for a sugar tax, highlighting research showing that a 20 per cent tax on sugar-sweetened drinks "could reduce consumption and prevalence of obesity in adults by 1.3 per cent".

Last month a league table of 22 nations found British girls and women under the age of 20 are the most overweight in Western Europe, with 29.2 per cent overweight or obese.

Of those, 8 per cent of the girls meet the clinical definition of obesity, having a Body Mass Index of 30 or above. An estimated 26 per cent of British boys under 20 are overweight and obese - placing them 10th.

LOAD-DATE: June 27, 2014

LANGUAGE: ENGLISH

PUBLICATION-TYPE: Newspaper; Web Publication

JOURNAL-CODE: WEBDTNS

Copyright 2014 Telegraph Media Group Limited
All Rights Reserved

The Telegraph
telegraph.co.uk

June 26, 2014 Thursday 3:40 PM GMT

One can of fizzy drink will hit new sugar limits;
New limits halve recommended sugar intake and leave little room for fizzy drinks

BYLINE: By Laura Donnelly Health Editor

SECTION: NEWS

LENGTH: 726 words

Recommended daily limits on sugar intake will be halved under new advice to Government - which means just one can of fizzy drink a day would use the whole allowance.

New proposals from the Scientific Advisory Committee on Nutrition (SACN) say that no more than five per cent of calories should come from sugar - compared with current limits of 10 per cent.

Public health officials said families should think carefully about whether children should be allowed fizzy drinks at all, and said the public needed to be encouraged to swap such beverages for low calorie versions, or water.

Read: How to encourage your children to drink water

The proposed recommendations mean women should have no more than five to six teaspoons of sugar a day, and seven to eight teaspoons for men.

Health officials say all age groups are exceeding current recommendations, with teenagers on average having 50 per cent more sugar than they should, on average drinking one 330 ml can of fizzy drink each day.

The guidelines also say Britain needs to boost its intake of fibre from 24 grams to 30 grams a day.

A report by Public Health England suggests the Government should consider a range of measures to reduce public consumption of sugar including taxes on sugary foods, restrictions on advertising and a clampdown on promotions of sugary foods.

Dr Alison Tedstone, Chief Nutritionist at Public Health England, said: "Eating too much sugar is harming our health; excess sugar and calorie intake leads to being overweight and obese and consequently having a higher risk of developing type 2 diabetes, heart disease and breast and colon cancer."

Currently a third of our 10-11 year olds are overweight or obese with the majority coming from the most deprived communities which is unacceptable.

Dr Ann Prentice, chair of SACN, said: "There is strong evidence in the report to show that if people were to have less free sugars and more fibre in their diet they would lower their risk of cardiovascular disease, type 2 diabetes and bowel cancer.

"For their health, people need to consume a balanced diet which includes carbohydrate-rich foods that are low in free sugars and high in fibre."

On Wednesday the Government's chief obesity adviser said parents should only serve water with meals and ban **fizzy drinks** and juices from the dining table in order to reduce their children's intake of sugar.

Prof Susan Jebb, chairman of the Government's responsibility deal with the food and drinks industry, also said doctors needed to be less reticent about telling overweight people to diet.

She said families should introduce strict rules about such drinks, limiting juice intake to one small glass a day with breakfast.

"Drink water, that's the very simple advice to parents," she said. "Encourage your children to stick with water. Low-fat milk is fine but water should be the mainstay," she added.

Prof Jebb said **sugary drinks** had a particularly significant impact on obesity because children and adults tended to consume them in addition to their calorie intake from food, not in place of solid foods.

"The biggest source of sugar across all age groups is **sugar**-sweetened **beverages** and those are an obvious target for action," she added.

The professor of diet and population health at Oxford University made the comments amid rising concern that sugar has become one of the greatest threats to health, fuelling an obesity time bomb and contributing to spiralling levels of diabetes.

Her remarks were backed by Prof Tom Sanders, the head of the Diabetes and Nutritional Sciences Division at King's College London, who said families needed to return to the days of having a jug of water on the table at all meal times.

He said: "The problem is a lot of people don't drink water any more. At the dinner table keep it simple; just have water on the table - not pop, not juice, not squash."

He said parents should stop buying soft drinks and squash cordials, and instead see them as an occasional treat.

Last month a national study found that children and teenagers are consuming around 40 per cent more added sugar than the recommended daily allowance - with fruit juices and **fizzy drinks** the chief culprit.

Two thirds of adults in Britain are overweight or obese, and British girls below the age of 20 are now the most overweight in western Europe.

LOAD-DATE: June 27, 2014

LANGUAGE: ENGLISH

PUBLICATION-TYPE: Newspaper; Web Publication

JOURNAL-CODE: WEBDTNS

Copyright 2014 Telegraph Media Group Limited
All Rights Reser

The Telegraph
telegraph.co.uk

June 26, 2014 Thursday 11:14 AM GMT

How to encourage your children to drink water; The Government's chief obesity advisor has said that parents should ban fruit juice and sugary drinks. But how can you persuade your children to drink water instead?

SECTION: LIFESTYLE

LENGTH: 630 words

The Government's chief obesity advisor, Professor Susan Jebb, has issued guidance saying that parents should introduce strict rules about **sugary drinks** - limiting juice to one small glass a day with breakfast, and making water the mainstay.

But how can you persuade your children to kick the sugar habit? Here are eight top tips.

Make it fun

Put water in an attractive cup (children love having cups with brightly coloured animal pictures or favourite TV characters on), add a novelty straw, use amusingly-shaped ice cubes, or add pretty pieces of fruit like strawberries. This might not persuade cynical adults to up their hl intake, but is thankfully an easy to way to make water appeal to younger children.

Limit their options

Don't stock your fridge with with fizzy sodas and colourful fruit juices - if you want them, but don't want your kids to drink them, put the bottles somewhere they can't see them. But it's probably best to clear the offending items from the house altogether: children are rather good at tracking down hidden treats, and it's best to be a be a role model. If they see you drinking water, they're more likely to follow.

Water first, treats after

If you do want to let your children have **fizzy drinks** on occasion, encourage them to drink a big glass of water beforehand - once they've quenched their thirst, they are much less likely to binge on the sugary stuff.

Explain the benefits

Preaching the virtues of water might sound like a sure way to put your children off it forever, but kids are often genuinely interested in the human body. Take them to the library and get some books on how the body works and nutrition. Teach them how important it is to stay hydrated - even a grumpy teenager might decide to dump the cola if they realise it's giving them acne.

Take small steps to improve the taste

Depending on where you live, water straight from the tap can be an unappealing prospect. Cold drinks are often more attractive to kids, especially in summer, so freeze your children's water bottles before school (they'll have defrosted by lunch) and stick a jug in the fridge that they can access. Filtered water might also taste a little better, while adding lemon and lime slices can add a fruity flavour with far less sugar than squash or juice.

Make it available

The best thing you to can do to encourage your children to drink water is to make it accessible. If they're playing outside, give them bottles; at dinnertime, put a big jug on the table. If everyone in the family is drinking water constantly, they'll get used to topping their water levels up.

Keep it positive

Resist the temptation to nag, or to focus on the fact that you want the kids to have water instead of **sugary drinks**. Encourage them to think about the health benefits, and treat drinking water as a normal part of the day, rather than as a chore that has to be got through,

Make changes gradual

It's probably a bad idea to throw all the **sugary drinks** into the bin overnight and dramatically announce the next day it's a water-only house. Start by making **sugary drinks** an occasional or weekend treat, rather than an everyday habit, serve them in slightly smaller glasses and offer your children the less-bad options, like weak squash instead of **fizzy drinks**. At the same time, introduce jugs of water to the house. If your child is really fussy, start watering down their juice or squash a little more every day, until eventually they are drinking straight water.

LOAD-DATE: June 26, 2014

LANGUAGE: ENGLISH

PUBLICATION-TYPE: Newspaper; Web Publication

JOURNAL-CODE: WEBDTNS

Copyright 2014 Telegraph Media Group Limited
All Rights Reserved

The Telegraph
telegraph.co.uk

June 26, 2014 Thursday 6:00 AM GMT

Coca Cola in controversy over £20m 'anti-obesity' drive; A new programme by Coca Cola to bring free fitness classes to 70 parks in Britain has come under fire from health campaigners

SECTION: NEWS

LENGTH: 712 words

Fizzy drinks giant Coca-Cola has sparked controversy with new plans to fund a £20 million anti-obesity fitness drive in 70 of the country's parks.

The global company says the new programme will mean thousands of free sessions and coaching for families, in activities such as tennis, basketball, hula-hooping, Zumba, rounders and archery.

But the plans last night triggered immediate criticism, with nutrition campaigners accusing the company of an "obscene" attempt to distract from its own part in fuelling Britain's obesity epidemic.

Earlier this month, public health officials warned that children and teenagers are consuming around 40 per cent more added sugar than is recommended.

The report by Public Health England said **soft drinks** and fruit juices were the chief culprit, providing the largest source of **sugar** in the diet among those aged between four and 18.

Soft drinks such as Coca Cola, which has nine teaspoons of **sugar** per can, amounted for 30 per cent of added **sugar** intake for those aged between 11 and 18, the National Diet and Nutrition Survey found.

Dr Aseem Malhotra, cardiologist and science director for campaign group Action on Sugar, said: "I think this is a really disingenuous stunt. They are trying to deflect attention from their own part in creating an obesity epidemic, which has been fuelled almost entirely by rising calorie consumption."

Dr Malhotra said "obscene" attempts by companies to associate themselves with active lifestyles could encourage the public to consume more unhealthy fare, wrongly assuming that small amounts of activity would be enough to balance it out.

Under the scheme, which starts on Friday, called Coca Cola Zero ParkLives, 70 parks in London, Birmingham and Newcastle will offer free activities to local communities, in particular targeting families and young adults.

The scheme will be marketed by Coca Cola Zero - one of its zero calorie products, and will run this summer, then for six months next year, with plans to expand it to more cities between now and 2020.

The company said it was making major strides to help tackle obesity in Britain, with 40 per cent of its sales now involving "zero calorie" versions.

Jon Woods, general manager of Coca Cola Great Britain said: "We know that companies get to grow in a market if they stay close to their customers; obesity is a great concern for our consumers; 61 per cent of adults are overweight and obese, as are 33 per cent of children."

"We have set out in the last two years that we want to play a more productive role in finding solutions to obesity; historically we would have shied away from this but we are taking a more proactive approach; this is about calories in and calories out and getting the energy balance right."

He said the project was an attempt to encourage those who tended not to take part in sport to be more activity, with fun activities, so that children and teenagers would encourage parents to join in.

In accordance with the company's marketing code, the programme will not be marketed at the under 12s, and those who wish to take part in sessions must be accompanied by a parent or guardian.

Mr Woods said the company was trying to play its part in tackling obesity levels, and should not be blamed for the current levels in Britain.

He said: "We don't think all the concerns about obesity can be laid at our door; we want healthy happy consumers; this is something that should be welcomed, not frowned upon."

The initiative comes alongside a controversial "Public Health Responsibility Deal" under which the food and drink industry has pledged to promote a healthier diet and make changes to their own products.

Some campaign groups have criticised the deal, suggesting that it has meant junk food manufacturers have been too close to Government policy.

The new Coca Cola programme will be evaluated by health quango UK Active, and has the backing of former Olympic athlete, Sebastian Coe

He said: "The next 10 years must be about capturing the spirit of the extraordinary summer of 2012 and converting it into lasting change. I am delighted to support Coca-Cola Zero ParkLives to bring business, local authorities and communities together to help people to move more, in local parks which are loved by communities and open to all."

LOAD-DATE: June 26, 2014

LANGUAGE: ENGLISH

PUBLICATION-TYPE: Newspaper; Web Publication

JOURNAL-CODE: WEBDTNS

Copyright 2014 Telegraph Media Group Limited
All Rights Reserved
d

The INDEPENDENT
Independent.co.uk

June 25, 2014 Wednesday 5:01 PM GMT

Drink water to cut obesity, health experts say

BYLINE: Charlie Cooper

SECTION: FRONTPAGE

LENGTH: 549 words

Parents should replace juice and **fizzy drinks** with jugs of water at mealtimes, diet experts have advised, to reduce their children's sugar intake and cut their risk of obesity, high blood pressure and diabetes.

Senior health scientists said today that sugar-sweetened drinks were the biggest source of sugar intake across all ages, but were a particular problem among children and teenagers.

Despite recent calls for a tax on **sugary drinks** and cuts to sugar content in everyday foods, Susan Jebb, professor of diet and population health at the University of Oxford, said that "very simple advice" to parents could play an even bigger part in improving children's diets.

"Drink water, that's the very simple advice to parents," she said. "Encourage your children to drink water. Once they've been weaned, children ought to be drinking water...There is a whole range of drinks out there, [but] I don't need to encourage people to be drinking any of the others. I can firmly stick to encouraging people to getting their fluid from water."

Around one in five children in the UK are obese, while roughly one third are overweight.

Sugary drinks were a particularly important "target" for combating the problem, Professor Jebb said, with clear links between their consumption and the risk of weight gain.

"We also have a plausible mechanism for that effect because liquid calories are known to be less satiating than solid calories - they fill you up less," she said.

Public health officials will issue new advice on sugar reduction tomorrow, as well as new guidance on foods high in carbohydrates such as bread and rice.

Professor Jebb said that "action" needed to be taken on **sugary drinks**, both "classic carbonated **beverages**", and also energy drinks and sports drinks. She added that a tax on **sugary drinks** was a feasible option but insisted that more focus should go on encouraging people to change their eating and drinking behaviour.

Professor Tom Sanders, head of diabetes and nutritional sciences at Kings College London, said that there was a particular problem with the marketing of **sugary drinks** - particularly during sporting events. Among sponsors for the World Cup in Brazil for instance, Coca Cola is an official corporate partner of FIFA, while McDonalds is an official sponsor of the World Cup.

He said such marketing gave the impression that drinks high in sugar could support an active, sporty lifestyle and also warned over the sugar content of many drinks marketed as sports or energy drinks.

"We have the World Cup going on at the moment. Some of these energy drinks are extremely high in sugar containing between 60 and 70 grams of sugar in a single bottle," he said. "That is a problem we are going to have to deal with - how these foods are marketed and how they are presented."

He highlighted Lucozade's Melonade, which contains 47 grams of sugar per 380ml serving - more than half an adult's daily guideline allowance - and is also available in 500ml and 1l bottles.

Professor Sanders agreed that parents should be "weaning" children off squash and pop which he said should be considered a treat and not an alternative to water.

"A glass of orange juice at breakfast is okay, but on the dinner table, [families should have] just a jug of water and maybe milk for younger children," he said.

LOAD-DATE: June 25, 2014

LANGUAGE: ENGLISH

PUBLICATION-TYPE: Newspaper; Web Publication

JOURNAL-CODE: WEBI

Copyright 2014 Independent Digital News and Media Limited
All Rights Reserved

144 of 371 DOCUMENTS

MailOnline

MailOnline

June 24, 2014 Tuesday 4:35 PM GMT

Starbucks enters the $400million soda business with three 'handcrafted' fizzy drinks that will be priced at the usual premium

BYLINE: BRYAN KEOGH

SECTION: NEWS

LENGTH: 566 words

- Fizzio Handcrafted Sodas will be made the old-fashioned way, by combining the flavors and spices in a cheesecloth and steeping them in hot water, just like tea
- A 'tall' will set you back $2.45, a bit pricier than the $1.80 it costs for a similar portion of coffee
- The beverages will go on sale at select locations in the U.S. South on Wednesday

Starbucks is entering the soda business, hoping to do to carbonated drinks what it did to coffee: crafting a designer beverage that people will fork over a premium to quaff down on their way to work.

The coffee giant said today that it is launching three flavors of Fizzio Handcrafted Sodas to be concocted by baristas in about a minute and a half at its more than 3,000 locations in 16 states across the southern U.S.

The strategy is timed to coincide with the rising summer temperatures so the cool drinks make up for any decline in hot coffee sales. They will be available beginning on Wednesday.

'We are changing the game in terms of how to get a carbonated drink,' Josh Fine, brand manager for Fizzio, told USA Today. 'Like what Starbucks did to coffee 40 years ago, we think we can do in the carbonation space.'

The Fizzio sodas will be carbonated fresh, as opposed to combining syrup with carbonated water like in most commercial machines. Instead, the flavors and spices are combined in a large cheesecloth and steeped in hot water, akin to how tea is made. Buyers can choose from light, standard or extra carbonation.

Starbucks went out of its way in a press release to emphasize that no high fructose corn syrup or preservatives or artificial flavors will be used. A 16 fluid ounce cup ('grande' to a barista) will pack 100 calories or less, it said.

The sodas start out at about $2.45 for a 'tall', compared with $1.80 for the same size coffee, according to USA Today.

The three flavors are:

- Spiced root beer: 'The nostalgic taste of classic root beer with a deliciously unexpected twist - cinnamon, nutmeg, clove and star anise add a flavorful kick to this soda.'
- Golden ginger ale: 'A refreshing blend of real ginger, citrus and brown sugar, the Golden Ginger Ale has a unique golden color and a complex, delicious taste that harkens back to the original Ginger Ale introduced at the turn of the 20th century.'
- And Lemon ale: 'A refreshing, citrus-forward blend of real lemon juice with hints of apricot and ginger.'

For those counting grams of sugar, a 'tall' lemon ale contains 21 grams, or about five packets worth, according to The Washington Post. A 'grande' contains about seven packets, while the 'trenta' equals 13, or about 52 grams.

For Starbucks, it's probably most about snaring a share of the $400 million global carbonated beverage market with a premium product the company hopes appeals to nutrition-conscious Millennials. Homemade soft drinks were called the top beverage trend of 2013 by the National Restaurant Association's annual survey of top chefs.

'Adding new drinking occasions is the key to (Starbucks') growth,' Michael Silverstein, senior partner at the Boston Consulting Group, told USA Today.

About a year ago, Starbucks began testing the sodas in Austin and Atlanta, Fine said.

The sodas will only be available at select locations in the following states: Hawaii, California, Arizona, New Mexico, Texas, Oklahoma, Louisiana, Mississippi, Alabama, Georgia, Florida, South Carolina, North Carolina, Virginia, Nevada and Utah.

LOAD-DATE: June 24, 2014

LANGUAGE: ENGLISH

PUBLICATION-TYPE: Web Publication

JOURNAL-CODE: WEBDM

Copyright 2014 Associated Newspapers Ltd.
All Rights Reserved

The Daily Telegraph

The Daily Telegraph (London)

June 23, 2014 Monday
Edition 1;
National Edition

Opponents of a sugar tax may just have to lump it

BYLINE: CRISTINA ODONE

SECTION: NEWS; Pg. 22

LENGTH: 612 words

Jeremy Hunt has said no to a **sugar** tax. The Secretary of State for Health does not want to impose any duties on **soft drinks** and sweets - despite pressure from health experts concerned with Britain's new status as the fat man of Europe.

I hate the nanny state as much as I love nursery food, dripping with butter and drowning in double cream. I don't want to be told how to eat, drink or bake by the Coalition. We have grown casual about our food, with few regarding meal times as a sacred gathering of family and friends, where we sit, talk, and consume the staff of life. Yet food still brings out the sentimental in us, and ministers meddle at their peril.

We associate what we eat with nurturing, Mother, and domesticity. Delia, Nigella, Mary Berry and other celebrity cooks won a special place in our hearts that no talking head or pundit could hope to attain - simply by kneading, frying, blending and sifting for our delectation. We speak of comfort food because that is what it provides: gooey, delicious reassurance in a harsh world. Even estate agents know this. They advise sellers to bake bread when a prospective buyer comes to scope the house: the warm, evocative scent will rob them of all resistance, as the love of baking is more powerful than the fear of bankruptcy.

I appreciate, then, that Mr Hunt is loath to stray into oven-glove territory. But he is wrong to indulge the nation's sweet tooth. Sugar, research shows, is as bad for us as tobacco, alcohol and marijuana. It causes diabetes, heart attacks, strokes, and now research suggests it is linked to cancer too. If this were a drug, Mr Hunt would ban it.

Sugar, instead, is everywhere, enticingly exposed at the till in most supermarkets, for the benefit of children; hidden in the ready-made meals we buy when too tired to cook. Sugar is neither "e" nor "skunk" and comes with no warning label. It is, instead, the money-spinner of a food industry that generates thousands of jobs and millions in income for the Treasury; so the Health Secretary won't chase the sugar dealers down dark alleys, barking "hands up!"

He should draw courage from a poll, conducted yesterday by BBC1, that for the first time shows an appetite for a sugar tax among ordinary people and not just health watchdogs. It's by no means a conclusive poll, but viewers of Sunday Morning Live yesterday were asked to vote on the issue, and 55 per cent said they'd favour a tax on sugar.

I wonder if those respondents had had the same chat with their NHS GP that I recently had with mine. She told me that she fears for the future of our health service because obesity now affects one in five Britons. They tie up a disproportionate amount of doctors' and nurses' time. But worse, obese patients require custom-made hospital beds, ambulance stretchers and all kinds of other specially tailored equipment. In this way, the obese are gobbling up limited NHS services and costing taxpayers more than £55 million a year.

How can we change this worrying trend? I suspect a tax will not work on its own. Our food culture has made couch potatoes as well as sugar highs, and a "holistic" approach that teaches the health benefits of cooking a meal, as well as better and more physical exercise programmes in schools, is called for. But a punitive tax on sugar will send out a clear, sharp message that this stuff is bad. The Government could promise to redistribute the income earned through the tax to subsidise healthier food. I know such interventionist measures sound indigestible. But if we don't do something quick we stand to lose lives, not just our appetites.

Comment on Cristina Odone's view at www.telegraph.co.uk/personalview

LOAD-DATE: June 23, 2014

LANGUAGE: ENGLISH

PUBLICATION-TYPE: Newspaper

JOURNAL-CODE: DTL

Copyright 2014 Telegraph Media Group Limited
All Rights Reserved

Daily Record & Sunday Mail

June 22, 2014 Sunday
Edition 1;
National Edition

Experts Junk star food ads

SECTION: NEWS; Pg. 28

LENGTH: 110 words

TV adverts that use stars to promote junk food should be banned, says a new report .

Experts claim ads for crisps and **sugary drinks,** such as Gary Lineker's Walkers commercials and Beyonce's Pepsi promos, help fuel childhood obesity.

Now Action on Sugar has recommended new laws to stop the "targeted marketing" of youngsters.

Health Secretary Jeremy Hunt asked the pressure group for ideas to tackle a crisis that has left one in three youngsters overweight.

Cardiologist Dr Aseem Malhotra said: "It is quite shameful that the food industry continues to spend billions on junk food ads targeting children.

"They even manage to associate sugary products with sport."

LOAD-DATE: June 22, 2014

LANGUAGE: ENGLISH

PUBLICATION-TYPE: Newspaper

JOURNAL-CODE: SML

Copyright 2014 Scottish Daily Record & Sunday Mail Ltd
All Rights Reserved

Daily Mirror

June 22, 2014 Sunday
Edition 1;
Northern Ireland

junk the celeb junk food ads;
Stars selling crisps and cola 'fuel kids obesity'

BYLINE: BEN GLAZE

SECTION: NEWS; Pg. 20

LENGTH: 347 words

TV adverts that use stars to promote junk food should be banned, says a report requested by the Health Secretary.

Experts claim crisps and **sugary drinks** commercials like Gary Lineker's Walkers campaign and Beyonce's Pepsi promos help fuel childhood obesity.

Now Action on Sugar has recommended new laws to stop the "targeted marketing" of Britain's youngsters.

Health Secretary Jeremy Hunt asked the pressure group for ideas to tackle a crisis that has left one in three youngsters overweight.

The report, seen by the Sunday Mirror ahead of today's release, says: "There must be a total ban on advertising of ultra-processed foods high in saturated fats, **sugars** and salt, and sweetened **soft drinks**."

That would signal the end for Pepsi, Coca Cola and fatty crisp ads.

And the report says stars should not be used in these ads stating: "Celebrity endorsement is wrong and gives the wrong message, particularly to children."

Action on Sugar's proposals also include banning junk food firms sponsoring football teams and sports fixtures.

SHAMEFUL The group's science director, cardiologist Dr Aseem Malhotra, said: "It is quite shameful that the food industry continues to spend billions in junk food advertising targeting children.

"They even manage to associate sugary products with sport.

"A child doing an hour of PE a day would put it all to waste if he or she ended up gorging on burger and chips and a packet of crisps washed down with a **sugary drink**. One has to run half a marathon to burn off those calories. It's time to dissociate junk food and sport."

Wayne Rooney had a £600,000-a-year deal with Coca Cola until 2011 while David Beckham was the face of Pepsi from 1998 to 2008.

Action on **Sugar** also wants a **sugar** tax to limit sweetened **soft drinks**, added **sugars** cut by 40 per cent and reduced fat in ultra-processed foods.

Group chairman Prof Graham MacGregor, said: "Obesity in children leads to premature development of cardiovascular disease, stroke and heart attacks." He said sugar reduction targets should be set this summer with "no delays, no excuses".

ben.glaze@sundaymirror.co.uk

LOAD-DATE: June 22, 2014

LANGUAGE: ENGLISH

GRAPHIC: Fault 'n Lineker: TV ads like Gary's help fuel childhood obesity, says report

PUBLICATION-TYPE: Newspaper

JOURNAL-CODE: SMR

Copyright 2014 MGN Ltd.
All Rights Reserved

The INDEPENDENT

Independent.co.uk

June 22, 2014 Sunday 12:04 AM GMT

Calls for 'sugar tax' to reduce rise in child obesity

BYLINE: Zander Swinburne

SECTION: FRONTPAGE

LENGTH: 381 words

The Government has been urged to introduce a "sugar tax" to curb the child obesity crisis.

Action on Sugar, a group of specialists concerned with sugar and its effects on health, has developed a seven-point plan to tackle the country's growing weight problems at the request of the Health Secretary, Jeremy Hunt. One in five 10 to 11-year-olds is obese and one in three is overweight.

As well as introducing a "**sugar** tax" to discourage children from drinking sweetened **soft drinks**, other measures include reducing fat in ultra-processed foods and "banning junk-food sports sponsorships".

The group's chairman, Professor Graham MacGregor, said: "Obesity in children leads to the premature development of cardiovascular disease, stroke, heart attacks and heart failure, which are the commonest causes of death and disability in the UK.

"Obesity is preventable if the food environment is changed, yet the current policies are not working. The UK requires the implementation of this coherent strategy, starting by setting incremental **sugar** reduction targets for **soft drinks** this summer."

With the health costs of obesity and type-2 diabetes estimated to be about £29bn a year, the group believes that measures to counter this must be taken immediately.

Katharine Jenner, public health nutritionist for Action on Sugar, said: "While individuals do what they can to look after their own welfare, the Government must also act in our best interests, rather than those of big business.

"This means using all the evidence-based tools at our disposal including taxation, reformulation, limiting unhealthy food at checkouts and stopping the marketing of unhealthy foods to children."

Sweet success

Since we were told the truth about sugar - that it is far more of a danger to our health than fat - publishers have been swift to educate us (or cash in). In January we had Fat Chance: The Bitter Truth About Sugar by Dr Robert Lustig; I Quit Sugar: Your Complete 8-Week Detox Program and Cookbook by Sarah Wilson; Sugar Addicts' Diet: See the Pounds Drop Off! by Nicki Waterman and Martha Roberts; and The Sugar Detox: Lose Weight, Feel Great and Look Years Younger by Brooke Alpert and Patricia Farris. Now, to keep us on the straight and narrow, comes the readable and sensible Sweet Nothing by Nicole Mowbray.

LOAD-DATE: June 22, 2014

LANGUAGE: ENGLISH

PUBLICATION-TYPE: Newspaper; Web Publication

JOURNAL-CODE: WEBI

Copyright 2014 Independent Digital News and Media Limited
All Rights Reserved

THE INDEPENDENT ON SUNDAY

The Independent on Sunday

June 22, 2014
Second Edition

Calls for 'sugar tax' to reduce rise in child obesity

BYLINE: Zander Swinburne

SECTION: NEWS; Pg. 4

LENGTH: 381 words

The Government has been urged to introduce a "sugar tax" to curb the child obesity crisis.

Action on Sugar, a group of specialists concerned with sugar and its effects on health, has developed a seven-point plan to tackle the country's growing weight problems at the request of the Health Secretary, Jeremy Hunt. One in five 10 to 11-year-olds is obese and one in three is overweight.

As well as introducing a "**sugar** tax" to discourage children from drinking sweetened **soft drinks**, other measures include reducing fat in ultra-processed foods and "banning junk-food sports sponsorships".

The group's chairman, Professor Graham MacGregor, said: "Obesity in children leads to the premature development of cardiovascular disease, stroke, heart attacks and heart failure, which are the commonest causes of death and disability in the UK.

"Obesity is preventable if the food environment is changed, yet the current policies are not working. The UK requires the implementation of this coherent strategy, starting by setting incremental **sugar** reduction targets for **soft drinks** this summer."

With the health costs of obesity and type-2 diabetes estimated to be about £29bn a year, the group believes that measures to counter this must be taken immediately.

Katharine Jenner, public health nutritionist for Action on Sugar, said: "While individuals do what they can to look after their own welfare, the Government must also act in our best interests, rather than those of big business.

"This means using all the evidence-based tools at our disposal including taxation, reformulation, limiting unhealthy food at checkouts and stopping the marketing of unhealthy foods to children."

SWEET SUCCESS

Since we were told the truth about sugar - that it is far more of a danger to our health than fat - publishers have been swift to educate us (or cash in). In January we had Fat Chance: The Bitter Truth About Sugar by Dr Robert Lustig; I Quit Sugar: Your Complete 8-Week Detox Program and Cookbook by Sarah Wilson; Sugar Addicts' Diet: See the Pounds Drop Off! by Nicki Waterman and Martha Roberts; and The Sugar Detox: Lose Weight, Feel Great and Look Years Younger by Brooke Alpert and Patricia Farris. Now, to keep us on the straight and narrow, comes the readable and sensible Sweet Nothing by Nicole Mowbray.

LOAD-DATE: June 22, 2014

LANGUAGE: ENGLISH

PUBLICATION-TYPE: Newspaper

JOURNAL-CODE: SU

Copyright 2014 Independent Print Limited
All Rights Reserved

Mirror

mirror.co.uk

June 22, 2014 Sunday 1:26 AM GMT

Celebs should not be allowed to sell junk food says new report; Top stars eating unhealthy food encourages childhood obesity says report ordered by the Health Secretary

BYLINE: By Simon Keegan

SECTION: NEWS,UK NEWS

LENGTH: 343 words

TV adverts that use stars to promote junk food should be banned, says a report requested by the Health Secretary.

Experts claim crisps and **sugary drinks** commercials like Gary Lineker'sWalkers campaign and Beyonce's Pepsi promos help fuel childhood obesity.

Now Action on Sugar has recommended new laws to stop the "targeted marketing" of Britain's youngsters.

Health SecretaryJeremy Hunt asked the pressure group for ideas to tackle a crisis that has left one in three youngsters overweight.

The report, seen by the Sunday Mirror ahead of today's release, says: "There must be a total ban on advertising of ultra-processed foods high in saturated fats, **sugars** and salt, and sweetened **soft drinks**." That would signal the end for Pepsi, Coca Cola and fatty crisp ads.

And the report says stars should not be used in these ads stating: "Celebrity endorsement is wrong and gives the wrong message, particularly to children."

Action on Sugar's proposals also include banning junk food firms sponsoring football teams and sports fixtures.

The group's science director, cardiologist Dr Aseem Malhotra, said: "It is quite shameful that the food industry continues to spend billions in junk food advertising targeting children.

"They even manage to associate sugary products with sport.

"A child doing an hour of PE a day would put it all to waste if he or she ended up gorging on burger and chips and a packet of crisps washed down with a **sugary drink**. One has to run half a marathon to burn off those calories. It's time to dissociate junk food and sport."

Wayne Rooney had a £600,000-a-year deal with Coca Cola until 2011 while David Beckham was the face of Pepsi from 1998 to 2008.

Action on **Sugar** also wants a **sugar** tax to limit sweetened **soft drinks**, added **sugars** cut by 40 per cent and reduced fat in ultra-processed foods.

Group chairman Prof Graham MacGregor, said: "Obesity in children leads to premature development of cardiovascular disease, stroke and heart attacks." He said sugar reduction targets should be set this summer with "no delays, no excuses".

LOAD-DATE: September 5, 2014

LANGUAGE: ENGLISH

PUBLICATION-TYPE: Newspaper; Web Publication

JOURNAL-CODE: WEBDMI

Copyright 2014 Trinity Mirror, Plc.
All Rights Reserved

165 of 371 DOCUMENTS

The Observer (England)

June 22, 2014

HEALTH Call for sugar tax to reduce obesity among children

SECTION: OBSERVER HOME NEWS PAGES; Pg. 23

LENGTH: 94 words

A campaign group has called on the government to introduce a "**sugar** tax" to discourage consumption of sweetened **soft drinks.** Action on **Sugar** has developed a seven-point plan to curb childhood obesity following a request for its views from the health secretary. The plan includes introducing a **sugar** tax, limiting the availability of highly processed foods and sweetened drinks, and banning "junk food sports sponsorships". The group's chairman, professor Graham MacGregor, said obesity in children led to cardiovascular disease, strokes, heart attacks and diabetes. PA

LOAD-DATE: June 21, 2014

LANGUAGE: ENGLISH

PUBLICATION-TYPE: Newspaper

Copyright 2014 Guardian Newspapers Limited
All Rights Reserved

169 of 371 DOCUMENTS

The Sunday Telegraph (London)

June 22, 2014

Edition 2;
National Edition

Tax sugar to cut obesity in children, ministers told

BYLINE: EDWARD MALNICK

SECTION: NEWS; Pg. 17

LENGTH: 122 words

A CAMPAIGN group has insisted the Government should introduce a "**sugar** tax" to discourage consumption of sweetened **soft drinks.**

Action on Sugar said its plan to curb childhood obesity followed a request from Jeremy Hunt, the Health Secretary, for its views. Its chairman, Prof Graham MacGregor, said: "Obesity in children leads to heart disease and strokes."

The measures include:

Reducing added sugars by reformulating food;

Halting marketing of ultraprocessed foods to children, cutting fat in these foods by 15 per cent and reducing supply;

Banning sponsorships of sports by junk food firms;

Discouraging drinking of **soft drinks** with a **sugar** tax;

Switching responsibility for nutrition from the Department of Health to an independent agency.

LOAD-DATE: June 22, 2014

LANGUAGE: ENGLISH

PUBLICATION-TYPE: Newspaper

JOURNAL-CODE: STL

Copyright 2014 Telegraph Media Group Limited
All Rights Reserved

The Sun (England)

June 22, 2014 Sunday

Edition 2;
National Edition

Lefties demand new 'sin taxes'

BYLINE: CRAIG WOODHOUSE

SECTION: NEWS; Pg. 25

LENGTH: 100 words

NANNY STATE 2 Political Correspondent A LABOUR-backed group has come up with a wide-ranging manifesto demanding "sin taxes" on fatty, salty and sugary foods.

It also seeks minimum booze prices to force up the cost of alcohol, and warnings on **fizzy drinks.** And in a blitz on motorists, the Socialist Health Alliance wants fuel tax and vehicle levies to be hiked alongside more 20mph zones so walkers can "reclaim our streets".

The group has been endorsed by Shadow Health Secretary Andy Burnham.

But Tory MP Andrew Percy blasted: "These nutjobs wouldn't be out of place in a dictatorship like North Korea."

LOAD-DATE: June 22, 2014

LANGUAGE: ENGLISH

GRAPHIC: Backing ... Burnham

PUBLICATION-TYPE: Newspaper

JOURNAL-CODE: SUN

Copyright 2014 NEWS GROUP NEWSPAPERS LTD
All Rights Reserved

The Sun (England)

June 22, 2014 Sunday
Edition 1;
Scotland

Tory plan to outlaw junk food sponsors

BYLINE: NICK McDERMOTT

SECTION: NEWS; Pg. 19

LENGTH: 116 words

JUNK food giants may be banned from sponsoring sporting events. The Conservatives are considering plans that could end high-profile tieups like Coca Cola's support of the Olympics. A childhood obesity report says bad diet is almost entirely to blame rather than a lack of exercise.

Experts want less sugar and fat added to food, a tax on **fizzy drinks** and a limit on fatty food ads.

Heart expert Aseem Malhotra - who co-authored the Action on Sugar report - said the fact McDonald's and Coca-Cola sponsored the Olympics was "obscene".

He said: "It tells kids that as long as you're active, you can eat high sugar foods."

But Richard Pike, of British Sugar, said that singling out sugar was "misleading".

LOAD-DATE: June 22, 2014

LANGUAGE: ENGLISH

GRAPHIC: Olympic sponsor . . Coke

PUBLICATION-TYPE: Newspaper

JOURNAL-CODE: SUNscot

Copyright 2014 NEWS GROUP NEWSPAPERS LTD
All Rights Reserved

Sunday Sun

June 22, 2014
Edition 1;
National Edition

Campaign calls for a 'sugar tax'

SECTION: NEWS; Pg. 45

LENGTH: 128 words

A CAMPAIGN group has called on the Government to introduce a "**sugar** tax" to discourage consumption of sweetened **soft drinks.**

Action on Sugar said it had developed a sevenpoint plan to curb childhood obesity following a request for its views from Health Secretary Jeremy Hunt.

The measures include bringing in a **sugar** tax, limiting the availability of ultra-processed foods and sweetened **soft drinks,** and banning "junk food sports sponsorships".

The group's chairman, Professor Graham MacGregor, said: "Obesity is preventable if the food environment is changed, yet the current policies are not working.

"The UK requires the implementation of this coherent strategy, starting by setting incremental **sugar** reduction targets for **soft drinks** this summer.

"No delays, no excuses."

LOAD-DATE: June 22, 2014

LANGUAGE: ENGLISH

PUBLICATION-TYPE: Newspaper

JOURNAL-CODE: SSN

Copyright 2014 MGN Limited
All Rights Reserved

Sunday Express

June 22, 2014
Edition 1;
National Edition

'Hit fizzy pop with sugar tax'

BYLINE: Jon Coates

SECTION: NEWS; Pg. 29

LENGTH: 280 words

A SUGAR tax on **fizzy drinks** is being proposed in a bid to stem the epidemic of childhood obesity.

With one in five 10 and 11-yearolds classed as obese and one in three overweight, desperate measures are needed, say experts.

These include banning junk food companies from sponsoring sporting events to stop young people thinking a diet packed with sugar and fat will not harm their health if they do some form of physical activity.

Campaigners also want to see added sugar in foods reduced by 40 per cent and a 15 per cent cut in fat, particularly saturated fat, in ultra-processed foods.

The group Action for Sugar has presented these proposals to Health Secretary Jeremy Hunt.

The Department of Health says one of the measures it is considering is the sugar tax on **fizzy drinks**.

However, Mr Hunt told an event in Westminster last week: "We have no plans for a sugar tax but we do recognise we have got to do a lot better on obesity as an issue."

Professor Graham MacGregor, of Action for Sugar, said: "Obesity in children leads to the premature development of cardiovascular disease, stroke, heart attacks and heart failure, which are the most common cause of death and disability in the UK."

He said obesity could also lead to Type 2 diabetes but could be prevented by a change of diet. However, he warned: "The current policies are not working." The burden placed on the NHS by obesity and Type 2 diabetes is estimated at around £29billion a year. This is expected to rise rapidly given the number of youngsters now overweight.

Action for Sugar claims that if the Government implements its seven-point action plan "the UK will be the first country in the world to halt the obesity epidemic".

LOAD-DATE: June 22, 2014

LANGUAGE: ENGLISH

PUBLICATION-TYPE: Newspaper

JOURNAL-CODE: SXP

Copyright 2014 Express Newspapers
All Rights Reserved

The Daily Telegraph

The Daily Telegraph (London)

June 21, 2014 Saturday
Edition 2;
National Edition

Hunt rules out sugar tax to tackle obesity

BYLINE: Laura Donnelly

SECTION: NEWS; Pg. 2

LENGTH: 271 words

MINISTERS have ruled out introducing a "sugar tax" on unhealthy snacks ahead of a series of reports on the issue.

Jeremy Hunt, the Health Secretary, said yesterday that the Coalition intended to embark on fresh measures to tackle Britain's obesity crisis, but that taxes on **fizzy drinks** and foods loaded with sugar were not part of the strategy.

There have been growing calls for greater government intervention in bringing down levels of sugar consumption.

In a report published today, council leaders call for a ban on "misleading" marketing of food products. The Local Government Association wants the European Commission to strengthen the rules governing claims on health and nutrition, so that products can only boast that they are "low fat" or "low calorie" if they are healthy in other ways.

Earlier this year The Daily Telegraph disclosed how many low-fat foods promoted as healthy options contained up to five times as much sugar as their "full fat" equivalents.

Next week the Government's scientific advisory committee on nutrition will publish recommendations on how to encourage the public to cut levels of sugar in everyday food. It will coincide with the release of a report by Public Health England on the same issue which will set out a range of options, including taxes on sugary foods and greater restrictions on advertising processed foods.

Neither report is expected to endorse one single recommendation, leaving it for ministers to determine the strategy.

Mr Hunt told an event in Westminster: "We have no plans for a sugar tax, but we do recognise that we have got to do a lot better on obesity as an issue."

LOAD-DATE: June 21, 2014

LANGUAGE: ENGLISH

PUBLICATION-TYPE: Newspaper

JOURNAL-CODE: DTL

Copyright 2014 Telegraph Media Group Limited
All Rights Reserved

theguardian

The Guardian

June 21, 2014 Saturday

As more of us become obese, the food manufacturers want us to think it's our own fault, the politicians try to avoid the issue and the diet industry gets rich on the misery it creates. So where do we go from here? Sarah Boseley investigates. Photographs by Johanna Parkin: The British Nutrition Foundation published a 'facts behind the headlines' paper to debunk allegations that sugar is toxic. It is funded extensively by the world's largest food corporations: 'Over the years I dieted myself up from a sensible size 10 to the 20 I am now, with enormous amounts of self-loathing. Now I think of the years of my life I put on hold'

BYLINE: Sarah Boseley

SECTION: GUARDIAN WEEKEND PAGES; Pg. 42

LENGTH: 2764 words

In May 2012, fire engines, police and an ambulance were called to the family home of a teenager called Georgia Davis in Aberdare, south Wales, in order to get her out of it. Nobody could dream up a more horrifying and humiliating nightmare for a girl of her age. A team of more than 40 people was involved in demolishing an upstairs wall of the semi-detached house and constructing a wooden bridge to get a specially reinforced stretcher into her bedroom. Georgia weighed 400kg (63 stone), said some reports. Nobody really knew - she was too heavy to get on the scales. Georgia's rescuers put up tarpaulins to shield her from the

camera lenses as they extracted her through a 10ft square hole in the brickwork and took her to hospital. She was covered by a sheet, because she could no longer get into any of her clothes.

The 19-year-old was morbidly obese and her organs were failing. Her mother, Lesley, called the ambulance because Georgia could no longer stand. For months she had not moved from her bedroom, where she spent her days on her laptop and watching TV. Eventually, like Alice In Wonderland inside the little house after drinking something she shouldn't, she grew too big to get out of the door.

Georgia is the extreme marker of a massive problem that has its roots in the way we live today and affects all of us. Two-thirds of us are overweight. A quarter of us are obese and in real danger of damaging our health and dying prematurely. But we are in denial. Obesity looks like Georgia, we think. It doesn't look like us. Her troubles were served up as entertainment in the tabloids. In photographs, her face, above the mountain of flesh, is curiously passive.

Obesity is not something to gawp at, and it is not a problem just for other people. It affects most of us. It's not about the way we look, or the size of dress or trousers we wear. This is about a very real threat to our health. Obesity is a life-shortening condition. Life expectancy in the UK, which has risen steadily since records began, may for the first time be about to fall. Moderate obesity cuts life expectancy by two to four years, and severe obesity could wipe an entire decade off your life, said the Lancet in 2009. The costs to health services and to the world's economies of vast numbers of people becoming sick and unable to work are already huge and increasing. The National Heath Service is spending £5bn a year treating heart attacks, strokes, diabetes, cancers, liver failure, hip and knee joint problems, and other consequences of obesity, and the bill is expected to reach £15bn within a few decades.

Experiments have shown that what we now think is normal is actually overweight and even obese. In 1999, researchers asked nearly 1,000 men and 1,000 women their weight, height and how they would describe themselves on a scale from "very underweight" to "obese". They repeated the exercise eight years later, and those surveyed weighed dramatically more, but fewer realised it. Only 75% correctly considered themselves overweight, compared with 81% eight years earlier. We just don't want to see it.

Georgia was 15 when she first hit the front page of the Sun, weighing more than 200kg (32 stone) and branded Britain's fattest teen. The question everybody eagerly asked was what had she been eating - how big a mountain of food? How many cakes at one sitting? Why she should want to was very much of subsidiary interest. Georgia and her mother, who is also obese, spoke of comfort eating after Georgia's dad died when she was five. In later stories it emerged that before she was 10, she had become the carer of both Lesley, who had heart disease, and her stepfather Arthur Treloar. Social services discussed removing her from the family, but she resisted. By any stretch of the imagination, Georgia had a tough childhood.

We can avoid being overweight by exercising personal responsibility, politicians say, voicing the script written by the food industry. We choose what we put into our mouths. We ought to know what will make us fat and have the self-restraint to stop eating. But can you really make that judgment of Georgia at the age of seven, who even then weighed 70kg (11 stone) and whom Lesley admits she fed with condensed milk as a baby, weaning her on to tinned potatoes and later filling her up with fried eggs and chips?

Georgia sold her story to the tabloids and TV to get the money to go to a weight-loss camp in North Carolina, where she lost half her body weight. She came back to find nothing had changed at home - her mother bought fish and chips because there was nothing to eat in the house. Georgia's own account, as told to a tabloid newspaper: "Around eight weeks after returning from camp, I drifted off the plan. I felt really alone. My parents weren't doing it with me at home and my friends weren't doing it at college, so there was no motivation to continue. I started reverting to my old ways. I wouldn't eat for half a day, then start bingeing into the night. I knew things were getting out of control, but I didn't want to return to the US because I missed my family too much and I was desperate to go to college and be a normal teenager."

There has been no comprehensive plan from any political party to tackle the obesogenic environment. The unwillingness to talk about fatness allows politicians to avoid the issues or offer half-hearted responses;

As more of us become obese, the food manufacturers want us to think it's our own fault, the politicians try to avoid the issue and the diet industry gets rich on the misery it creates. So where do we go from here? Sarah Boseley investigates. Photographs by Johanna Parkin: The British Nutrition Foundation published a 'facts behind the headlines' paper to debunk allegations that sugar is toxic. It is funded extensively by the world's largest food corporations: 'Over the years I dieted myself up from a sensible size 10 to the 20 I am now, with enormous amounts of self-loathing. Now I think of the years of my life I put on hold' The Guardian June 21, 2014 Saturday

above all else, it enables them to avoid what they fear would be a damaging confrontation with the powerful economic players within the food and drink industry. Politicians are also afraid they will be accused of taxing the poor if they hike the prices of cheap foods - an argument often put forward by their industry friends. One government after another has opted for talks and voluntary agreements on food labelling and marketing to children. The deals that have been struck have been partial and ineffective.

We are ill-served by many of the bodies held up as experts in food and nutrition, because they are not impartial. They take money from big corporations, often with a clear conscience, because they don't see harm in processed foods and drinks. The impressively named British Nutrition Foundation published a "facts behind the headlines" paper to debunk allegations that sugar is toxic. The foundation claims independence, but is full of scientists who believe in working with industry, and it is funded extensively by the world's largest food corporations. Its "sustaining members" include Coca-Cola, Danone Waters and Dairies, DuPont, Kellogg, Nestle, PepsiCo, Tate & Lyle, Associated British Foods (which includes British Sugar) and Unilever.

The paper said that "overall, evidence does not support the claims made that sugar increases the risk of obesity, diabetes and heart disease". The foundation preferred the views of the European Food Safety Authority (EFSA), which suggested the data on the effects of consuming large amounts of sugar were "limited and mainly short-term". The EFSA has been embroiled in controversy over conflicts of interest.

And then there is the UK government's own advisory body, SACN, the scientific advisory committee on nutrition. On this are a number of scientists who work with big food companies and see nothing wrong with it. Chairing the SACN working group reviewing carbohydrates in our diet - including sugar - is Professor Ian Macdonald from Nottingham University. He is a paid adviser to Coca-Cola and Mars, although he stepped down for the duration of SACN's sugar inquiry. He sees no reason he should not work for industry. In fact, he thinks it's a good thing. He believes it gives him perspective. "I have explained my associations with industry to the Department of Health, and they are quite happy with the relationships," he told me. He has advised Coca-Cola that they have an issue, he said. "I told the chief executive of Coca-Cola in Atlanta that he has a real problem, because his company is perceived as being a cause of the problem and they need to do something about their promotion of full-strength Coca-Cola and its status within the business. His senior colleagues winced when I said that to him in the board that I sat on, but he took it on the chin and they are beginning to do something about it."

The trade bodies Sugar Nutrition in the UK and its global counterpart, the London-based World Sugar Research Organisation (WSRO), both argue that there is no good scientific evidence to blame sugar for obesity. They cite one study after another, most of them industry-funded, on the benefits of sugar. For all their bullish stance, they look to be on the back foot. The WSRO's director general, Richard Cottrell, who was previously director of Sugar Nutrition, no longer seems inclined to share his objective views of the science. "I don't speak to the press," he said when I called him, and put the phone down.

The food industry and its friends and collaborators would like us to think it is all our fault. You can eat whatever you like, they insist - there is no such thing as bad food. It is your fault if you overeat and get fat. They are partly responsible for a blame culture that delivers the overweight, burdened with shame and self-loathing, into the hands of the diet gurus.

Yet the diet industry, with its gimmicks, motivational books and celebrity endorsements, is one of the biggest frauds of our time. In the UK, it is estimated to be worth £2bn. There are vast amounts of money to be made from the raising and dashing of people's hopes. When you look at the scientific studies carried out on people trying to lose weight, it's hard not to think that all the blockbuster diet gurus are charlatans - if not, one can only assume that they are incredibly hopeful and optimistic people. They must be wonderful at parties. Because all the research points the same way: it tells us that our bodies, having got fat, will do everything possible to keep that weight on. It's the fear of starvation. We are like hibernating animals that store up food so they can survive a hard winter. Except that, these days, winter never comes. Restaurants and takeaways are full of food. The pizza delivery is on its way.

As more of us become obese, the food manufacturers want us to think it's our own fault, the politicians try to avoid the issue and the diet industry gets rich on the misery it creates. So where do we go from here? Sarah Boseley investigates. Photographs by Johanna Parkin: The British Nutrition Foundation published a 'facts behind the headlines' paper to debunk allegations that sugar is toxic. It is funded extensively by the world's largest food corporations: 'Over the years I dieted myself up from a sensible size 10 to the 20 I am now, with enormous amounts of self-loathing. Now I think of the years of my life I put on hold' The Guardian June 21, 2014 Saturday

Kelly Brownell at the Yale Rudd Center in the US invented the term yo-yo dieting, also known as weight cycling. People embark on very low-calorie diets and all goes well for a number of weeks or months. But then they get the plateau effect. The excess weight stubbornly refuses to shift. Depression and fatigue set in, making it impossible to continue. Weight goes back on and disproportionately what used to be muscle is replaced by fat. It is bad for health and harder to lose next time. So could diets actually shorten your life? Brownell and colleagues looked at this back in 1991, in a study published in the New England Journal of Medicine. Brownell's team looked at the data produced by a large and very well-known long-term trial in the US called the Framingham Heart Study, which followed the lives and monitored the changing health of more than 5,000 people in a town of that name in Massachusetts from 1948. They found that people whose weight fluctuated a lot had a greater risk of heart disease and early death than those whose weight was steady. And this was most marked among the youngest - aged 30 to 44 - who are both the most diet prone and the least likely to fall ill for other reasons. While they point out how hard it is to get the evidence to prove that diets shorten lives, they warn of the possibility that yo-yo dieting may actually cause chronic disease.

Angela Meadows, from Birmingham University's psychology department, believes that diets, not the excess of junk food around us, are actually the root cause of the obesity epidemic. It is going on diets that causes people to gain weight, she says. "I started dieting when I was probably 12, 13 years old, when I wasn't fat. I dieted myself out like most people - if they are dieting, they tend to get very big, and they get bigger and bigger with every diet, and there is more self-loathing, more blaming, more comfort eating, more withdrawal, so you've got that avoiding, coping behaviour.

"The worse you feel about yourself, the less likely you are to go out and actually exercise, and people do sit at home and comfort eat, and then they get bigger and their problems get worse." Meadows' view, and that of the Health at Every Size movement, is that it is possible to be fat and fit - if you are active enough, your weight does not matter, because it is no longer a health issue.

"So over the years I dieted myself up from probably a sensible size 10 or whatever to the 20 I am now with enormous amounts of self-loathing. Now that I've stopped doing that, it's like I've got my life back. I think of the 30-odd years of my life that I put on hold and wasted. My mother's 72 and still won't leave the house unless her arms are covered. She still doesn't eat properly. She's dieted all her life as well. She was thin when she got married and got bigger. Generations of women have been wasting their lives on this, and for what?"

It ended, Meadows says, when she began "eating intuitively", which she describes as listening to her body's signals. She says she does not now respond to the food environment around her - she is not at the mercy of advertisers. "Now that I no longer have that sense of deprivation, I no longer think I should do this and I shouldn't eat that, because then, when things are stressful or when that's just in front of you, that's what you turn to. 'I've had a hard day, I've been good, I deserve it.' If you're quite attuned to your body, you don't respond in that way."

Psychology professor Jane Ogden, who treats people with such morbid obesity that they cannot get out of a wheelchair, thinks we are getting to a point where the personal responsibility argument will not hold. "With obesity, perhaps the tipping point will come when it starts to be seen that the next generation is just following in their parents' footsteps and that child obesity is a form of child abuse. Somebody can have a BMI of 40, but if they have a kid, then they also are fat before the age of 10. That isn't acceptable any longer."

And the huge financial burden on the NHS may eventually force ministers into more action. "The government has to be much more nanny state in terms of policing the food industry, taxing snack food, taxing **fizzy drinks**, banning **fizzy drinks**, banning sugary foods, and not just in school dinners but also in work canteens and hospital food. Every kind of food provision has to be much more controlled by the government. Then they have to put money into cycle paths and street lighting and redesign their cities so that it is much more easy for people to be physically active."

But Ogden says she can see the other side of her own argument. "I do believe all that, but then there is the libertarian in me who says, 'Does that mean they ban Cheesy Wotsits?'

As more of us become obese, the food manufacturers want us to think it's our own fault, the politicians try to avoid the issue and the diet industry gets rich on the misery it creates. So where do we go from here? Sarah Boseley investigates. Photographs by Johanna Parkin: The British Nutrition Foundation published a 'facts behind the headlines' paper to debunk allegations that sugar is toxic. It is funded extensively by the world's largest food corporations: 'Over the years I dieted myself up from a sensible size 10 to the 20 I am now, with enormous amounts of self-loathing. Now I think of the years of my life I put on hold' The Guardian June 21, 2014 Saturday

"I mean, my children sometimes have Cheesy Wotsits, but my children are perfectly thin because they don't have them all the time. Foods are bad only if you have too many of them."

There have been few changes to the environment where Georgia Davis grew up that facilitate healthy living. Aberdare has a covered market near the station, where fruit and vegetables and meat and fish are for sale, alongside knitting wool and clothes and gimmicks and gadgets. But the main shopping streets offer every kind of cheap takeaway, from pizzas to pies to chips to curry. The Pop-In Cafe will do you homemade chicken curry, with both rice and chips - as well as a can of **fizzy drink**, tea or coffee - for £4.60.

To reach the housing estate where Georgia was brought up, you have to climb a steep road that runs up the far side of the valley. A lot of cars are passing, where once adults and children would have had no option but to walk. There is nobody on the pavement, but it is a wet day.

Georgia is not in the yellowish-cream semi-detached house, one of many identical homes on the estate. The shape of the hole made in the upstairs outside wall is still faintly visible under new brickwork and a coat of paint. Her mother appears at the door. "She doesn't live here any more," she said. "But she won't speak to you. She has a contract with the Sun." *

This is an edited extract from The Shape We're In, by Sarah Boseley, published by Guardian Faber at £12.99. To order a copy for £8.99, including free UK mainland p&p, call 0330 333 6846 or go to theguardian.com/bookshop.

Captions:

Georgia Davis was 15, weighing over 200kg, when she was branded Britain's fattest teen by the Sun

LOAD-DATE: June 20, 2014

LANGUAGE: ENGLISH

PUBLICATION-TYPE: Newspaper

Copyright 2014 Guardian Newspapers Limited
All Rights Reserved

MailOnline

June 21, 2014 Saturday 10:07 PM GMT

Sugary beverages 'a trigger for gout': Soft drinks, fructose and beer to blame for rise in patients with 'kings' disease'

BYLINE: ROGER DOBSON

SECTION: HEALTH

LENGTH: 366 words

- Number of gout patients has nearly doubled since 1970s
- Modern causes are sweet drinks, fructose, beer and medication

Gout, once the disease of kings, is on the increase, and **sugary soft drinks** as much as whisky and wine may be to blame, research claims.

The number of patients has nearly doubled since the 1970s and the painful disease is the most common form of inflammatory arthritis in men.

One person in 35 is now affected, according to the research into the prevalence and causes of gout.

Historic sufferers such as King Henry VIII may have developed the condition from fine dining on meat and port, but today's victims may be more likely to get it from **sugar**-sweetened **soft drinks**, fructose and beer, or as a side effect of medications.

Gout is linked to a build-up in the blood of uric acid, a waste product made in the body and excreted through the kidneys. It forms when the body breaks down chemicals in the cells known as purines.

When too much uric acid is produced, or too little is excreted through the bladder, tiny crystals may form in and around joints. These hard, needle-shaped crystals are responsible for the inflammation and pain.

Just why some people get gout and not others is unclear, although genes are thought to play a part. It is also not clear why there are more cases. But several other countries, including the US, Australia and China, show similar rises, starting in the second half of the 20th Century.

'The incidence of gout increased in conjunction with what are likely to be related increases in fructose consumption and obesity,' says Dr Peter Simkin, who led a University of Washington study into the growth of the disease.

Dietary fructose is sugar found naturally in tiny amounts in fruit, but extracted and used to sweeten pre-prepared foods.

'Our diet advice for the gout patient is to limit meat intake, stay away from beer, be wary of hard liquor, drink wine in moderation, do not fast, embrace coffee and dairy products and cut down on fructose,' adds Dr Simkin.

Use of low-dose aspirin and some high blood pressure drugs are also linked to increased risk, including beta-blockers. Eating cherries, however, was found to reduce the risk of attacks by 35 per cent.

LOAD-DATE: June 21, 2014

LANGUAGE: ENGLISH

PUBLICATION-TYPE: Web Publication

JOURNAL-CODE: WEBDM

Copyright 2014 Associated Newspapers Ltd.
All Rights Reserved

The Telegraph
telegraph.co.uk

June 21, 2014 Saturday 7:00 AM GMT

No sugar tax says Jeremy Hunt;
Sugar tax is ruled out by Health Secretary, ahead of major reports on Britain's sweet tooth

BYLINE: By Laura Donnelly Health Editor

SECTION: NEWS

LENGTH: 577 words

Ministers have ruled out introducing a "sugar tax" on unhealthy snacks ahead of two major Government reports on the issue next week.

Jeremy Hunt, the Health Secretary, said the Coalition intends to embark on further measures to tackle Britain's obesity crisis - but said taxes on **fizzy drinks** and foods which are loaded with sugar are not part of their strategy.

His remarks came amid growing calls for greater government intervention to help bring down rising levels of sugar consumption.

In a report published today, council leaders call for a ban on "misleading" marketing of food products.

The Local Government Association wants the European Commission to strengthen the rules governing claims on health and nutrition, so that products can only boast they are "low fat" or "low calorie" if they healthy in other ways.

Under current rules, products labelled as "low fat" may have high sugar or salt content.

Earlier this year the Telegraph disclosed how many low fat foods promoted as healthy-eating options contain more sugar than their "full fat" equivalents - in some cases more than five times as much.

Next week the Government's Scientific Advisory Committee on Nutrition (SACN) will publish recommendations on how to encourage the public to cut levels of sugar in everyday food.

It will coincide with the release of a report by Public Health England on the same issue, which will set out a range of options, including taxes on sugary foods, government targets to reduce sugar and increased restrictions on advertisements for processed foods.

Neither report is expected to endorse one single recommendation - leaving it for ministers to ultimately determine the strategy.

The publications follow rising concern that sugar is one of the greatest threats to health, creating an obesity time bomb and contributing to spiralling levels of diabetes.

Mr Hunt said new measures are needed to tackle childhood obesity, which he said remains "much too high" - but ruled out introducing taxation.

He told an event in Westminster: "We have no plans for a sugar tax, but we do recognise that we have got to do a lot better on obesity as an issue."

The health secretary said latest evidence suggested the tide is turning on childhood obesity, with rates showing a small fall in recent years, and progress being achieved through working with the food industry.

Mr Hunt said: "We are very proud of the progress we made this year on introducing traffic light labelling in supermarkets, calorie counts in more than half of all fast food outlets, progress in reducing the sale of sweets at checkouts - but, there is a lot more that we need to do and this will engage us this Parliament and the next government as well."

On Thursday, a paper by quango Public Health England is expected to warn that coronary heart disease, strokes and cancer are the UK's "leading killers", partly driven by high blood pressure and excess weight - both of which have been linked to high sugar consumption.

In draft versions, two of the main proposals are a tax to increase prices of sugar products and to introduce targets to cut sugar levels.

In March, the World Health Organisation (WHO) suggested that the daily allowance for a person's sugar intake should be halved to six teaspoons for the average adult.

The Government scientific advisory will publish its own recommendations on sugar intake on Thursday, but it has already indicated that it will not follow the WHO advice, but come to its own conclusions.

LOAD-DATE: June 21, 2014

LANGUAGE: ENGLISH

PUBLICATION-TYPE: Newspaper; Web Publication

JOURNAL-CODE: WEBDTNS

Copyright 2014 Telegraph Media Group Limited
All Rights Reserved

theguardian

Guardian.com.

June 20, 2014 Friday

Co-op to remove 100m teaspoons of sugar with own brand squash range

BYLINE: Rebecca Smithers theguardian.com

LENGTH: 554 words

ABSTRACT

Supermarket becomes first in UK to launch no added sugar fruit squash range in response to rising obesity and diabetes levels

FULL TEXT

The Co-operative supermarket is to remove the equivalent of almost 100m teaspoons of sugar from its shelves as it becomes the first UK retailer to launch an own brand fruit squash range with no added sugar.

The move is part of a new pledge - under a voluntary industry-led drive under the government's so-called public health responsibility deal - to help improve the nation's diet in response to rising obesity and diabetes levels.

The new own brand High Juice range - which will appeal to children - will help cut 1.5bn calories from the Co-op's supermarket shelves, while a five-point pledge aims to offer all customers healthier soft drinks without compromising on taste.

By the end of July, the entire range of Co-operative branded squashes will contain no added **sugar**, while more than 90% of all its own brand **soft drinks** (squash, fruit juice, carbonated drinks and water) will be either **sugar** free, have no added **sugar** or will be low in **sugar**.

The government welcomed the move and said other retailers should be make bigger efforts to reformulate own brand products. The health minister Jane Ellison said: "As a nation we are consuming too many calories, and sugar plays a substantial role in this. That's why I am delighted that the Co-op has re-formulated its entire squash range to ensure the range contains 'no added sugar'. I urge all others to consider what more they can do."

Health campaigners have been scrutinising retailers' moves to reduce **sugar** in **soft drinks** and criticised many for acting too slowly. The Co-op move comes as the UK's scientific advisory committee on nutrition (SACN) prepares to publish the results of a long inquiry into carbohydrates, including **sugar**, in the diet and which may recommend lower levels of consumption.

The World Health Organisation has recently set a guideline limit of 10% of daily calories from **sugar**, with a recommendation that countries should aim to get it down as low as 5%. But the recent official national diet and nutrition survey showed people in England were exceeding the 11% current target - with children consuming about 15% of their calories as **sugars**, while a third came from **soft drinks** and fruit juice.

The Children's Food Campaign said: "The new school food standards provide the benchmark for reducing the amount of **sugar** children consume. Industry still has a long way to go to match that. But we are now finally starting to see companies act on public health concerns about the **sugar** levels in their **soft drinks** and the sheer volume of **sugary** products they promote."

It welcomed the Co-op's moves "to significantly reduce the **sugar** in its **soft drinks**, and champion no **sugar** added drinks for ranges, like squashes, popular with children. This approach is far preferable to introducing slightly-less **sugary** versions alongside existing ones, which is the route Coca-Cola has chosen with Coca-Cola Life."

At the UK's largest supermarket, Tesco, David Beardmore, category buying manager for soft drinks, said: "We have already removed 3bn calories from our soft drinks and identified ten key areas where we can go further, working with the soft drinks industry to ensure our customers have a range of choices available."

LOAD-DATE: June 20, 2014

LANGUAGE: ENGLISH

PUBLICATION-TYPE: Newspaper

Copyright 2014 Guardian News and Media Limited or its affiliated companies
All Rights Reserved
Registered in England and Wales. No. 908396
Registered office: PO Box 68164, Kings Place, 90 York Way, London N1P 2AP

DAILY MAIL (London)

June 18, 2014 Wednesday

DENTISTS: CUT SUGAR TO JUST 4 TEASPOONS PER DAY

BYLINE: BY NO BYLINE AVAILABLE

LENGTH: 199 words

WE must slash our sugar intake to just four teaspoons a day to save our teeth, dentists have warned.

They are calling on the Government to force the food industry to lower the amount of sugar in everything from sweets to cakes, chocolates and **fizzy drinks.**

Limiting sugar to this level would equate to less than half a can of Coca-Cola, which has nine teaspoons, two digestive biscuits or one bowl of Kellogg's Frosties cereal.

Tooth decay accounts for 6 to 10 per cent of total health costs. Only a significant cut in sugar - particularly when it is added to processed food and drinks - will make a difference, says a study published today in the Public Health Nutrition Journal. Co-author Professor Aubrey Sheiham of University College London said: The Government must stop acting in the best interests of the food and drink industry and take action on sugar now.'

On average, adults consume 14 and a half teaspoons of sugar every day while teenagers have 19 teaspoons. Recently, the World Health Organisation warned we must cut our daily intake to six teaspoons.

But this latest study warns we must curb it to four teaspoons a day, which for adults works out as a 74 per cent reduction.

© Daily Mail

LOAD-DATE: June 17, 2014

LANGUAGE: ENGLISH

PUBLICATION-TYPE: Papers

Copyright 2014 Associated Newspapers Ltd.
All Rights Reserved

MailOnline

June 18, 2014 Wednesday 2:19 PM GMT

California lawmakers reject putting 'obesity' labels on sugary drinks

California lawmakers reject putting 'obesity' labels on sugary drinks MailOnline June 18, 2014 Wednesday 2:19 PM GMT

BYLINE: ASSOCIATED PRESS

SECTION: NEWS

LENGTH: 535 words

- The bill would have required certain sodas, energy drinks and fruit drinks to warn consumers that added sugar 'contributes to obesity, diabetes and tooth decay'
- It failed on a 7-8 vote, short of the 10 needed, as Democrats doubted the labels would change consumer behavior
- Leaders in San Francisco and Berkeley are considering putting similar measures before voters in November

A bill that would have made California the first state in the nation to require warning labels on sodas and other **sugary drinks** was effectively killed on Tuesday.

Senator Bill Monning's SB1000 failed on a 7-8 vote as his fellow Democratic lawmakers doubted whether a label would change consumer behavior. It needed 10 votes to pass.

Certain sodas, energy drinks and fruit drinks would have included a label reading, 'STATE OF CALIFORNIA SAFETY WARNING: Drinking **beverages** with added **sugar**(s) contributes to obesity, diabetes, and tooth decay.'

It was developed by public health advocates using cigarette and alcohol warnings as a model. Representatives of the beverage industry argued that the bill was unfair by not applying to other foods and drinks, including lattes and chocolate milk.

Mr Monning, of Carmel, said warning labels would be the most efficacious tool for educating people about the dangers of **sugary drinks**.

'Changing behavior is the hardest challenge in the world of medicine,' Monning told lawmakers before the vote. 'But you can't start to even make a commitment to make behavior change if you don't have the information.'

His bill had support from the California Medical Association, the California Center for Public Health Advocacy and groups devoted to improving the health of minorities. A similar bill introduced in Vermont stalled this year.

Democratic Assemblyman Jimmy Gomez noted that cigarette warning labels were accompanied by taxes and prohibitions on smoking in public places before tobacco use plunged.

'It wasn't necessarily the labels that changed peoples' habits, but it was the other requirements,' said Gomez, who represents Los Angeles.

CalBev, the California arm of the American Beverage Association, says it posts calorie counts on the front of many beverage containers as part of a voluntary campaign that started in 2010.

Industry groups also say warning labels may conflict with an upcoming overhaul of the nutritional information labels regulated by the U.S. Food and Drug Administration.

Sugary drinks have been a target of public health advocates who see them as one of the biggest drivers of preventable diseases. Former New York City Mayor Michael Bloomberg pushed a ban on large servings of **soft drinks** in 2012. A court later struck down the ban after it prompted lawsuits and an aggressive campaign from businesses.

Leaders in San Francisco and Berkeley are considering sending measures imposing a **sugary drink** tax to voters in November after nearby Richmond rejected such a tax in 2012.

A children's health group recently launched a 'Sugar Bites' ad campaign in the east San Francisco Bay Area and state capital depicting **sugary drinks** as snarling monsters with sharp teeth held by anxious children.

Mr Monning, who previously called for a soda tax, said he would keep pushing for warning labels.

LOAD-DATE: June 18, 2014

LANGUAGE: ENGLISH

PUBLICATION-TYPE: Web Publication

JOURNAL-CODE: WEBDM

Copyright 2014 Associated Newspapers Ltd.
All Rights Reserved

MailOnline

June 18, 2014 Wednesday 1:22 PM GMT

California lawmakers reject sugary drink warnings

BYLINE: ASSOCIATED PRESS

SECTION: NEWS

LENGTH: 472 words

A bill that would have made California the first state in the nation to require warning labels on sodas and other **sugary drinks** was effectively killed Tuesday.

Senator Bill Monning's SB1000 failed on a 7-8 vote as his fellow Democratic lawmakers doubted whether a label would change consumer behavior. It needed 10 votes to pass.

Certain sodas, energy drinks and fruit drinks would have included a label reading, "STATE OF CALIFORNIA SAFETY WARNING: Drinking **beverages** with added **sugar**(s) contributes to obesity, diabetes, and tooth decay."

It was developed by public health advocates using cigarette and alcohol warnings as a model. Representatives of the beverage industry argued that the bill was unfair by not applying to other foods and drinks, including lattes and chocolate milk.

Mr Monning, of Carmel, said warning labels would be the most efficacious tool for educating people about the dangers of **sugary drinks**.

'Changing behavior is the hardest challenge in the world of medicine,' Monning told lawmakers before the vote. 'But you can't start to even make a commitment to make behavior change if you don't have the information.'

His bill had support from the California Medical Association, the California Center for Public Health Advocacy and groups devoted to improving the health of minorities. A similar bill introduced in Vermont stalled this year.

Democratic Assemblyman Jimmy Gomez noted that cigarette warning labels were accompanied by taxes and prohibitions on smoking in public places before tobacco use plunged.

'It wasn't necessarily the labels that changed peoples' habits, but it was the other requirements,' said Gomez, who represents Los Angeles.

CalBev, the California arm of the American Beverage Association, says it posts calorie counts on the front of many beverage containers as part of a voluntary campaign that started in 2010.

Industry groups also say warning labels may conflict with an upcoming overhaul of the nutritional information labels regulated by the U.S. Food and Drug Administration.

Sugary drinks have been a target of public health advocates who see them as one of the biggest drivers of preventable diseases. Former New York City Mayor Michael Bloomberg pushed a ban on large servings of **soft drinks** in 2012. A court later struck down the ban after it prompted lawsuits and an aggressive campaign from businesses.

Leaders in San Francisco and Berkeley are considering sending measures imposing a **sugary drink** tax to voters in November after nearby Richmond rejected such a tax in 2012.

A children's health group recently launched a 'Sugar Bites' ad campaign in the east San Francisco Bay Area and state capital depicting **sugary drinks** as snarling monsters with sharp teeth held by anxious children.

Mr Monning, who previously called for a soda tax, said he would keep pushing for warning labels.

LOAD-DATE: June 18, 2014

LANGUAGE: ENGLISH

PUBLICATION-TYPE: Web Publication

JOURNAL-CODE: WEBDM

Copyright 2014 Associated Newspapers Ltd.
All Rights Reserved

MailOnline

MailOnline

June 18, 2014 Wednesday 2:42 AM GMT

California lawmakers reject **sugary drink** warnings

BYLINE: ASSOCIATED PRESS

SECTION: AP

LENGTH: 484 words

SACRAMENTO, Calif. (AP) - A bill that would have made California the first state in the nation to require warning labels on sodas and other **sugary drinks** was effectively killed Tuesday.

Sen. Bill Monning's SB1000 failed on a 7-8 vote as his fellow Democratic lawmakers doubted whether a label would change consumer behavior. It needed 10 votes to pass.

Certain sodas, energy drinks and fruit drinks would have included a label reading, "STATE OF CALIFORNIA SAFETY WARNING: Drinking **beverages** with added **sugar**(s) contributes to obesity, diabetes, and tooth decay."

It was developed by public health advocates using cigarette and alcohol warnings as a model. Representatives of the beverage industry argued that the bill was unfair by not applying to other foods and drinks, including lattes and chocolate milk.

Monning, of Carmel, says warning labels would be the most efficacious tool for educating people about the dangers of **sugary drinks**.

"Changing behavior is the hardest challenge in the world of medicine," Monning told lawmakers before the vote. "But you can't start to even make a commitment to make behavior change if you don't have the information."

His bill had support from the California Medical Association, the California Center for Public Health Advocacy and groups devoted to improving the health of minorities. A similar bill introduced in Vermont stalled this year.

Democratic Assemblyman Jimmy Gomez noted that cigarette warning labels were accompanied by taxes and prohibitions on smoking in public places before tobacco use plunged.

"It wasn't necessarily the labels that changed peoples' habits, but it was the other requirements," said Gomez, who represents Los Angeles.

CalBev, the California arm of the American Beverage Association, says it posts calorie counts on the front of many beverage containers as part of a voluntary campaign that started in 2010.

Industry groups also say warning labels may conflict with an upcoming overhaul of the nutritional information labels regulated by the U.S. Food and Drug Administration.

Sugary drinks have been a target of public health advocates who see them as one of the biggest drivers of preventable diseases. Former New York City Mayor Michael Bloomberg pushed a ban on large servings of **soft drinks** in 2012. A court later struck down the ban after it prompted lawsuits and an aggressive campaign from businesses.

Leaders in San Francisco and Berkeley are considering sending measures imposing a **sugary drink** tax to voters in November after nearby Richmond rejected such a tax in 2012.

A children's health group recently launched a "Sugar Bites" ad campaign in the east San Francisco Bay Area and state capital depicting **sugary drinks** as snarling monsters with sharp teeth held by anxious children.

Monning, who previously called for a soda tax, said he would keep pushing for warning labels.

Follow Fenit Nirappil at http://www.twitter.com/FenitN.

LOAD-DATE: June 18, 2014

LANGUAGE: ENGLISH

PUBLICATION-TYPE: Web Publication

JOURNAL-CODE: WEBDM

Copyright 2014 Associated Newspapers Ltd.
All Rights Reserved

MailOnline

June 18, 2014 Wednesday 1:48 AM GMT

California lawmakers reject sugary drink warnings

BYLINE: ASSOCIATED PRESS

SECTION: AP

LENGTH: 255 words

SACRAMENTO, Calif. (AP) - A bill that would have made California the first state in the nation to require warning labels on sodas and other **sugary drinks** was effectively killed on Tuesday.

SB1000 by Sen. Bill Monning, D-Carmel, failed on a 7-8 vote as his fellow Democratic lawmakers doubted whether a label would change consumer behavior. It needed 10 votes to pass.

Certain sodas, energy drinks and fruit drinks would have included a label reading, "STATE OF CALIFORNIA SAFETY WARNING: Drinking **beverages** with added **sugar(**s) contributes to obesity, diabetes, and tooth decay."

It was developed by public health advocates using cigarette and alcohol warnings as a model. Representatives of the beverage industry argued that the bill was unfair by not applying to other foods and drinks, including lattes and chocolate milk.

Monning says warning labels would be the most efficacious tool for educating people about the dangers of **sugary drinks**, including increased risk for obesity and diabetes.

"Changing behavior is the hardest challenge in the world of medicine," Monning told lawmakers before the vote. "But you can't start to even make a commitment to make behavior change if you don't have the information."

Sugary drinks have been a target of public health advocates who see them as one of the biggest drivers of preventable disease. New York City banned large drinks in 2012, prompting lawsuits and an aggressive campaign from businesses.

Monning, who previously pushed for a soda tax, said he would keep pursuing the warning labels.

LOAD-DATE: June 18, 2014

LANGUAGE: ENGLISH

PUBLICATION-TYPE: Web Publication

JOURNAL-CODE: WEBDM

Copyright 2014 Associated Newspapers Ltd.
All Rights Reserved

202 of 371 DOCUMENTS

MailOnline

June 18, 2014 Wednesday 1:37 AM GMT

California sugary drink warning label effort fails

BYLINE: ASSOCIATED PRESS

SECTION: AP

LENGTH: 138 words

SACRAMENTO, Calif. (AP) - A legislative committee has rejected a bill that would have made California the first state in the nation to require warning labels on sodas and other **sugary drinks,** effectively killing the legislation.

The Assembly Committee on Health rejected SB1000 on a 7-8 vote Tuesday as Democratic lawmakers doubted whether a label would change consumer behavior. It needed 10 votes to pass.

Democratic Sen. Bill Monning says warning labels would be among the most effective tools for educating people about the dangers of **sugary drinks,** including increased risk for obesity and diabetes.

Representatives of the beverage industry argued that his bill was unfair by not applying to other foods and drinks.

New York City banned large **sugary drinks** in 2012, prompting lawsuits and an aggressive campaign from businesses.

LOAD-DATE: June 18, 2014

LANGUAGE: ENGLISH

PUBLICATION-TYPE: Web Publication

JOURNAL-CODE: WEBDM

Copyright 2014 Associated Newspapers Ltd.
All Rights Reserved

MailOnline

June 18, 2014 Wednesday 1:14 AM GMT

Cut sugar to just four teaspoons a day, say dentists: Call on Government to force food industry to lower amounts in all products to tackle tooth decay

BYLINE: SEAN POULTER

SECTION: HEALTH

LENGTH: 751 words

- 4tsps is equivalent to less than half a normal can of Coca-Cola, two digestives or one bowl of Frosties
- Tooth decay treatment still accounts for 6-10 per cent of total health costs
- Experts estimate a 20 per cent tax on **fizzy drinks** would cut the number of overweight Britons by more than a quarter of a million.

Dentists are calling for a dramatic reduction of around 75 per cent in daily sugar consumption to save the nation's teeth.

Experts at University College London say consumption of added sugar in treats like sweets, cakes, chocolate and **fizzy drinks** should be cut to around four teaspoons a day.

A cap at that level would equate to less than half a normal can of Coca-Cola, which contains nine teaspoons of sugar, two digestives or just one bowl of Kellogg's Frosties cereal.

The availability of advanced toothpastes and the addition of fluoride to the water supply in some countries and regions has reduced tooth decay, however treatment still accounts for 6-10 per cent of total health costs.

A study published today argues that only a significant cut in the use and consumption of sugar - particularly when it is added to processed food and drink - will make a difference.

The findings will fuel demands for the introduction of some kind of sugar tax on **fizzy drinks** and other foods - similar to those applied to cigarettes - in order to drive up the cost and drive down consumption.

Any such measure would be fiercely fought by the food industry, which insists sugar is natural source of calories and can be eaten safely as part of a normal healthy diet.

The warnings about sugar consumption come from Professor Aubrey Sheiham, who is Emeritus Professor of Dental Public Health at UCL and co-author of the study.

Prof Sheiham said: 'Tooth decay is one of the most widespread health problems and it is thought around a third of UK children aged 12 have visible tooth decay.

The World Health Organisation (WHO) has recently called for a reduction in consumption to around 5 per cent of daily calories, which equates to 25g or six teaspoons.

'Added sugar has found its way into almost all food, and the use of sugar as a means to calm, entertain, or reward children has become normalised, whereas sugar should be an occasional treat.

'The government must stop acting in the best interests of the food and drink industry rather than individuals, and take action on sugar now.'

The government's chief medical officer, Dame Sally Davies, has expressed sympathy for the idea of a sugar tax, while an expert group set up by the official watchdog Public Health England has signalled it should be considered.

An 'options for action' document prepared by the organisations identifies six possible ways of reducing sugar intake: a tax on **sugary drinks**; foods being reformulated to contain less sugar; a cut in portion sizes; advertising rules being tightened; health warnings on sugary products; and encouraging farmers to grow fruit and vegetables instead of sugar beet.

The report estimates that a 20 per cent tax on **fizzy drinks**, which would raise the price of a can from 70p to 84p, would cut the number of overweight Britons by more than a quarter of a million.

The average sugar consumption per day is around 58g or 14.5 teaspoons for adults and 76g or around 19 teaspoons for teenagers.

The World Health Organisation (WHO) has recently called for a reduction in consumption to around 5 per cent of daily calories, which equates to 25g or six teaspoons.

The new study suggests even lower levels are necessary to protect teeth from decay or caries, bringing the figure down to 2-3 per cent of calories, which equates to 15g a day or four teaspoons. That would represent a reduction of 74 per cent against the current average for adults.

The research paper, co-authored by Prof Sheiham and obesity expert Professor Philip James, of the London School of Hygiene and Tropical Medicine, is published in the Public Health Nutrition Journal.

It concludes: 'Previous analyses based on children have misled public health analyses on sugars....The much greater adult burden of dental caries highlights the need for very low sugar intakes throughout life, e.g. 2-3 per cent energy intake, whether or not fluoride intake is optimum.'

Nutritionist and Campaign Director of Action on Sugar, Katharine Jenner, backed the study findings, saying: 'Added sugars are completely unnecessary in our diets and are strongly linked to dental decay as well as to obesity and Type II Diabetes.'

LOAD-DATE: June 18, 2014

LANGUAGE: ENGLISH

PUBLICATION-TYPE: Web Publication

JOURNAL-CODE: WEBDM

Copyright 2014 Associated Newspapers Ltd.
All Rights Reserved

Copyright 2014 Telegraph Media Group Limited
All Rights Reserved

DAILY MAIL (London)

June 16, 2014 Monday

SLAP A 20% 'HEALTH TAX' ON FIZZY DRINKS

LENGTH: 373 words

EXPERTS CALL FOR ACTION TO SLASH UK SUGAR INTAKE

BY FIONA MACRAE SCIENCE CORRESPONDENT

A TAX could be put on **fizzy drinks** and biscuits could be emblazoned with health warnings in a new strategy to slash the nation's sugar intake.

A report commissioned by a Government agency says that targeting **soft drinks** would be an easy option in the war on **sugar**.

It estimates that a 20 per cent tax on **fizzy drinks** - which would raise the price of a can from 70p to 84p - would cut the number of overweight Britons by more than a quarter of a million.

The leaked paper, which was written by heart doctors and health researchers, comes just weeks after the Chief Medical Officer Dame Sally Davies warned that a sugar tax' may be necessary to tackle the country's obesity epidemic.

Figures show that British girls are the fattest in Europe, with almost a third overweight or obese. Around two thirds of adults are overweight or obese.

The options for action' document, which was prepared for Public Health England, identifies six possible ways of reducing sugar intake: a tax on **sugary drinks**; foods being reformulated to contain less sugar; a cut in portion sizes; advertising rules being tightened; health warnings on sugary products; and encouraging farmers to grow fruit and vegetables instead of sugar beet.

The draft report describes **sugary soft drinks** as low-hanging fruit' - or an easy target - and says a 20 per cent **sugar** tax would reduce obesity rates by 1.3 per cent.

It argues that the move would have public support, especially when health benefits are emphasised'.

The paper, obtained by The Grocer magazine, is expected to form the basis of a report to be published on June 26.

Public Health England said the Government had asked for its advice but any decision would lie with ministers.

The Department of Health said it currently had no plans for a sugar tax.

* Our diets could be improved by listening to the right kind of music, scientists claim. Researchers from Oxford University claim high-pitched tones trick the brain into thinking a food is sweeter than it really is, while low notes enhance bitter tastes. They said a dish could be made to taste up to 10 per cent sweeter through sonic seasoning', meaning less sugar was needed.

© Daily Mail

LOAD-DATE: June 15, 2014

LANGUAGE: ENGLISH

PUBLICATION-TYPE: Papers

Copyright 2014 Associated Newspapers Ltd.
All Rights Reserved

227 of 371 DOCUMENTS

Daily Mirror

June 16, 2014 Monday
Edition 1;
National Edition

Sugary drink taxes 'would curb obesity'

SECTION: NEWS; Pg. 2

LENGTH: 130 words

A SUGAR tax should be slapped on **fizzy drinks** to tackle adult obesity, a health watchdog has demanded.

Public Health England wants ministers to bring in a 20% tax rate which adds 14p to a 70p can of Coke.

It also wants a restriction on junk food ads and targets for the drinks industry to cut sugar levels in products.

The body is behind a discussion document out next week that says the UK's biggest killers - heart disease, strokes and cancer - are driven by obesity and high blood pressure.

Prof Graham MacGregor, of campaign group Action on Sugar, said: "If Jeremy Hunt is a real Health Secretary, he has to do something. This is a sensible plan."

The British **Soft Drinks** Association said: "These campaigners appear to have missed the 60% of **soft drinks** that contain no added **sugar.**"

LOAD-DATE: June 16, 2014

LANGUAGE: ENGLISH

PUBLICATION-TYPE: Newspaper

JOURNAL-CODE: DMR

Copyright 2014 MGN Ltd.
All Rights Reserved

MailOnline

June 16, 2014 Monday 12:17 AM GMT

Slap a 20% 'health tax' on fizzy drinks: Experts call for action to slash UK sugar intake

BYLINE: FIONA MACRAE, SCIENCE CORRESPONDENT

SECTION: NEWS

LENGTH: 427 words

- Health Secretary Jeremy Hunt told to introduce 20% '**fizzy drinks** tax'
- Health campaigners say Brits are drinking sugar 'by the spoonful'
- Sugar intake from **fizzy drinks** blamed for obesity and diabetes crisis
- But **fizzy drinks** industry accuse campaigners of being 'blinder by zeal'

A tax could be put on **fizzy drinks** and biscuits could be emblazoned with health warnings in a new strategy to slash the nation's sugar intake.

A report commissioned by a Government agency says that targeting **soft drinks** would be an easy option in the war on **sugar**.

It estimates that a 20 per cent tax on **fizzy drinks** - which would raise the price of a can from 70p to 84p - would cut the number of overweight Britons by more than a quarter of a million.

The leaked paper, which was written by heart doctors and health researchers, comes just weeks after the Chief Medical Officer Dame Sally Davies warned that a 'sugar tax' may be necessary to tackle the country's obesity epidemic.

Figures show that British girls are the fattest in Europe, with almost a third overweight or obese. Around two thirds of adults are overweight or obese.

[IMG 9907823 noborder /]

The 'options for action' document, which was prepared for Public Health England, identifies six possible ways of reducing sugar intake: a tax on **sugary drinks**; foods being reformulated to contain less sugar; a cut in portion sizes; advertising rules being tightened; health warnings on sugary products; and encouraging farmers to grow fruit and vegetables instead of sugar beet.

The draft report describes **sugary soft drinks** as 'low-hanging fruit' - or an easy target - and says a 20 per cent **sugar** tax would reduce obesity rates by 1.3 per cent.

It argues that the move would have public support, 'especially when health benefits are emphasised'.

The paper, obtained by The Grocer magazine, is expected to form the basis of a report to be published on June 26.

Public Health England said the Government had asked for its advice but any decision would lie with ministers.

The Department of Health said it currently had no plans for a sugar tax.

High-pitched music may not help your headache... but it could improve your diet

Our diets could be improved by listening to the right kind of music, scientists claim.

Researchers from Oxford University claim high-pitched tones trick the brain into thinking a food is sweeter than it really is, while low notes enhance bitter tastes.

They said a dish could be made to taste up to 10 per cent sweeter through 'sonic seasoning', meaning less sugar was needed.

LOAD-DATE: June 16, 2014

LANGUAGE: ENGLISH

PUBLICATION-TYPE: Web Publication

JOURNAL-CODE: WEBDM

Copyright 2014 Associated Newspapers Ltd.
All Rights Reserved

The Sun (England)

June 16, 2014 Monday
Edition 1;
National Edition

THE Sun SAYS No fizz tax

SECTION: EDITORIAL; OPINION; LEADING ARTICLES; Pg. 10

LENGTH: 123 words

WHEN authorities want to curb activities they disapprove of, they always turn to the same old blunt weapon. Tax rises.

The latest depressing brainwave from Public Health England would see a huge new levy on **fizzy drinks.**

Thus penalising everyone, from those who enjoy one can of Coke a month to those glugging a litre a day.

Not to mention singling out soft drinks while giving a free ride to countless other products which also make you fat in excess.

Tackling the obesity crisis should be about endlessly repeating the message that being unhealthily overweight can destroy or end your life and that sugar is a major cause.

There are more creative ways to wean the nation off junk food and **fizzy drinks** than clobbering us with yet another new tax.

LOAD-DATE: June 16, 2014

LANGUAGE: ENGLISH

PUBLICATION-TYPE: Newspaper

JOURNAL-CODE: SUN

Copyright 2014 NEWS GROUP NEWSPAPERS LTD
All Rights Reserved

The Sunday Telegraph

The Sunday Telegraph (London)

June 15, 2014
Edition 2;
National Edition

Experts call for tax on fizzy drinks

BYLINE: EDWARD MALNICK

SECTION: NEWS; Pg. 4

LENGTH: 817 words

A TAX on **fizzy drinks**, government targets to reduce sugar and advertising restrictions on processed foods are among a series of measures being suggested by the public health watchdog to help tackle rising levels of sugar consumption.

The Sunday Telegraph has seen a document commissioned by Public Health England that contains several possible actions to reduce sugar in food and drink.

The paper, which was discussed at a meeting between the quango and industry representatives this month, warns that coronary heart disease, strokes and cancer are the UK's "leading killers", partly driven by high blood pressure and excess weight - both of which have been linked to high sugar consumption.

It will form the basis of a draft "options for action" paper to be published by Public Health England later this month.

The document could pave the way for a series of tougher measures to bring down sugar consumption in Britain, including a tax on sugary items. Campaigners said the step was "extremely positive" and a sign that the quango had acknowledged the need for "emergency action" to combat obesity and type 2 diabetes.

However, the food and drink industry is alarmed by the severity of some of the options in the document. It insists the causes of obesity are "far wider" than sugar and warns that a tax would hit the poorest families hardest.

The publication of Public Health England's document on June 26 will coincide with the release of a long-awaited report on carbohydrates by the Scientific Advisory Committee on Nutrition, which will include recommendations on sugar intake in Britain.

The World Health Organisation halved its recommended daily limit of sugar in March.

The Sunday Telegraph has seen an early draft of Public Health England's paper, produced by the UK Health Forum, a coalition of heart disease specialists, which was discussed at two summits hosted by the quango earlier this month - one for experts, charities and campaigners, and the second for industry representatives.

Two of the main proposals are a tax to increase prices of sugar products and targets similar to those in place for salt, which would require manufacturers to reduce levels of the ingredient in their products gradually.

Six "themes" are set out in the draft, each containing a number of possible actions.

Under the heading "use less", the paper suggests "reformulating" food and drink products to reduce sugar content, either with government-set targets or voluntary standards. Such a move would help to monitor sugar levels and create a "level playing field", it says.

Sugar targets have been backed by campaigners and MPs including Keith Vaz, who has type 2 diabetes. The paper states that salt reduction targets have been a success. Under "weaknesses", the paper states that a voluntary approach may lead manufacturers to focus on "niche" products. Last month, The Sunday Telegraph disclosed how companies that signed up to the Government's "healthy eating" pledge have failed to reduce the amount of sugar in some of their main brands.

Under a second theme, "sell less", the paper suggests a "tax on **sugar** or products high in **sugar**" and points to a similar move in France through which **soft drink** sales declined.

The paper says that **sugary drinks** have been described as a "low hanging fruit", highlighting research showing that a 20 per cent tax on sugarsweetened drinks "could reduce consumption and prevalence of obesity in adults by 1.3 per cent". Such a move has public support, the paper states, "especially when health benefits are emphasised".

However, the document also warns that such a tax risks having a "regressive impact on income" and harming the competitiveness of food and drink companies. It says that the cost of the tax may be absorbed by the industry.

It is understood that the "favoured options" among experts and campaigners at the Public Health England summit were for a triple approach of a tax, targets, and banning or severely limiting advertisements of ultra-processed foods. Campaigners say the threat of a tax could force the industry to accept targets.

The Department of Health, which oversees Public Health England, has insisted that it is "not considering a sugar tax". However, Graham MacGregor, the chairman of Action on Sugar, said: "If Jeremy Hunt is a real Health Secretary he has got to do something now - and this is a sensible plan."

Dr Alison Tedstone, the chief nutritionist at Public Health England, said: "PHE is actively exploring ways to reduce the amount of sugar people consume." She confirmed that two events held by Public Health England with stakeholders, including academics, consumer groups and industry figures earlier this month explored ways of reducing the intakes of sugar.

Gavin Partington, the director general of the British Soft Drinks Association, said it was "misguided" to suggest obesity could be "attributed to one ingredient".

Sound of sweetness: Page 21

LOAD-DATE: June 15, 2014

LANGUAGE: ENGLISH

GRAPHIC: The Sunday Telegraph on June 1

PUBLICATION-TYPE: Newspaper

JOURNAL-CODE: STL

Copyright 2014 Telegraph Media Group Limited
All Rights Reserved

THE SUNDAY TIMES

The Sunday Times (London)

June 15, 2014 Sunday
Edition 3;
National Edition

Leaked report calls for tax onslaught on sugary drinks

BYLINE: Kate Mansey ; Jon Ungoed-Thomas

SECTION: NEWS; Pg. 7

LENGTH: 507 words

A LEAKED document commissioned by a Department of Health agency sets out proposals for a tax on **sugary drinks** and health warnings on foods with a high sugar content.

The draft report for Public Health England includes a six-point plan to reduce the production, sale, marketing and consumption of sugar.

The proposals include the new tax on sugary foods, the placing of controls on food advertising, encouraging farmers to grow fruit instead of sugarbeet and placing health warnings on goods high in sugar.

The document, leaked to The Grocer magazine, describes **sugary drinks** as "low-hanging fruit" for greater taxation, arguing that such a move would be supported by the public, "especially when health benefits are emphasised".

It says there is "evidence from modelling studies that a 20% tax on sugar-sweetened drinks in the UK could reduce consumption and prevalence of obesity in adults by 1.3%".

The advertising of products was also criticised. The report suggested controls should be put on marketing high-sugar foods to "vulnerable groups like children".

Another strategy suggests urging manufacturers to reformulate food products to reduce the sugar content, strategy that was used successfully in the past to reduce salt. It also proposes that the UK, one of the biggest producers of sugarbeet in Europe, could use sugar for biofuels instead of food. "[Britain] could implement alternatives to sugar production, eg growing fruits and vegetables. Or non-food uses for sugarbeet, eg biofuels," says, while admitting that would be "challenging to get public health considerations prioritised".

The document was produced by the UK Health Forum, a group of about 80 scientists and non-governmental bodies. A final version is expected to be published later this month when the Scientific Advisory Committee on Nutrition is to announce recommendations on sugar intake.

The Department of Health said the government "remained committed" to cutting sugar intake but had no plans to introduce a sugar tax.

Professor Graham Mac-Gregor, who heads Action on Sugar, a group that campaigns against high-sugar diets, said: "The government has to do something about sugar. If they don't act now it will be a national scandal."

The sugar industry is fighting back, warning that proposals to cut sugar intake to just 5% of calories could make people even fatter.

Producers claim reducing people's sugar consumption could result in a higher intake of fatty and higher-calorie foods. They claim this "might lead to an increase, rather than a decrease, in the prevalence of obesity".

The World Sugar Research Organisation - funded by sugar producers - said a draft proposal by the World Health Organisation to cut sugar consumption is based on flawed evidence.

Robert Lustig, a paediatric endocrinologist at the University of California and the author of Fat Chance: The Bitter Truth about Sugar, rejected the claim.

"We used to have a lowadded-sugar consumption and we didn't have obesity," he said. "We have only got obesity since we increased our added-sugar consumption."

LOAD-DATE: June 15, 2014

LANGUAGE: ENGLISH

PUBLICATION-TYPE: Newspaper

JOURNAL-CODE: STS

Copyright 2014 Times Newspapers Limited
All Rights Reserved

The Telegraph
telegraph.co.uk

June 14, 2014 Saturday 5:16 PM GMT

Health watchdog weighs up sugar tax and reduction targets; Public Health England, the health watchdog, is considering the idea of a tax on fizzy drinks as part of moves to reduce sugar consumption

BYLINE: By Edward Malnick

SECTION: NEWS

LENGTH: 989 words

A tax on **fizzy drinks**, government targets to reduce sugar and advertising restrictions on processed foods are among a series of measures being suggested by the public health watchdog to help tackle rising levels of sugar consumption.

The Telegraph has seen a document commissioned by Public Health England that contains several possible actions to reduce sugar in food and drink.

The paper, which was discussed at a meeting between the quango and industry representatives this month, warns that coronary heart disease, strokes and cancer are the UK's "leading killers", partly driven by high blood pressure and excess weight - both of which have been linked to high sugar consumption.

It will form the basis of a draft "options for action" paper to be published by Public Health England next week.

The document could pave the way for a series of tougher measures to bring down sugar consumption in Britain, including a tax on sugary items.

Campaigners said the step was "extremely positive" and a sign that the quango had acknowledged the need for "emergency action" to combat obesity and Type 2 diabetes.

However, the food and drink industry is alarmed by the severity of some of the options in the document. It insists the causes of obesity are "far wider" than sugar and warns that a tax would hit the poorest families hardest.

The publication of Public Health England's document on June 26 will coincide with the release of a long-awaited report on carbohydrates by the Scientific Advisory Committee on Nutrition, which will include

recommendations on sugar intake in Britain. The World Health Organisation halved its recommended daily limit of sugar in March.

The Telegraph has seen an early draft of Public Health England's paper, produced by the UK Health Forum, a coalition of heart disease specialists, which was discussed at two summits hosted by the quango earlier this month - one for experts, charities and campaigners, and the second for industry representatives.

Two of the main proposals are a tax to increase prices of sugar products and targets similar to those in place for salt, which would require manufacturers to gradually reduce levels of the ingredient in their products.

Six "themes" are set out in the draft, each containing a number of possible actions.

Under the heading "use less", the paper suggests "reformulating" food and drink products to reduce sugar content, either with government-set targets or voluntary standards. Such a move would help to monitor sugar levels and create a "level playing field", it says.

Sugar targets have been backed by campaigners and MPs including Keith Vaz, who has Type 2 diabetes. The paper states that salt reduction targets have been a success.

Under "weaknesses", the paper states that a voluntary approach may lead manufacturers to focus on "niche" products. Last month, The Telegraph disclosed how companies that signed up to the Government's "healthy eating" pledge have failed to reduce the amount of sugar in some of their main brands .

Under a second theme, "sell less", the paper suggests a "tax on **sugar** or products high in **sugar**" and points to a similar move in France through which **soft drink** sales declined.

The paper says that **sugary drinks** have been described as a "low hanging fruit", highlighting research showing that a 20 per cent tax on sugar-sweetened drinks "could reduce consumption and prevalence of obesity in adults by 1.3 per cent". Such a move has public support, the paper states, "especially when health benefits are emphasised".

However, the document also warns that such a tax risks having a "regressive impact on income" and harming the competitiveness of food and drink companies. It says that the cost of the tax may be absorbed by the industry.

It is understood that the "favoured options" among experts and campaigners at the Public Health England summit were for a triple approach of a tax, targets, and banning or severely limiting advertisements of ultra-processed foods.

Campaigners say the threat of a tax could force the industry to accept targets.

Dr Aseem Malhotra, a cardiologist and science director of Action on Sugar, a campaign group, said: "It is extremely positive that Public Health England has acknowledged the necessity for emergency action to combat the increasing prevalence of Type 2 diabetes and obesity that is spiralling out of control."

The Department of Health, which oversees Public Health England, has insisted that it is "not considering a sugar tax".

However, Graham MacGregor, professor of cardiovascular medicine at the Wolfson Institute of Preventive Medicine and chairman of Action on Sugar, said: "If Jeremy Hunt is a real Health Secretary he has got to do something now - and this is a sensible plan."

Gavin Partington, director general of the British Soft Drinks Association, said it was "misguided" to suggest obesity could be "attributed to one ingredient".

An industry source suggested the quango was attempting to "mitigate" against possible criticism of the SACN report for not going far enough, and to show that the government was "doing something" to address the issue.

Dr Alison Tedstone, chief nutritionist at Public Health England, sits on the secretariat of the SACN committee.

She said: "Public Health England is actively exploring ways to reduce the amount of sugar people consume, to inform a wider strategy to improve dietary health and reduce levels of obesity in the population.

"Earlier this month, Public Health England held two events to explore ways of reducing the intakes of sugar with a range of stakeholders including academics, consumers groups and industry.

"We are now preparing a paper for discussion on reducing sugars intake that will be published on the 26th June."

Last week a study by Action of Sugar of **fizzy drinks** concluded that people were drinking **sugar** "by the spoonful" and warned that artisan **beverages** hold more of the ingredient than cheaper blends.

LOAD-DATE: June 15, 2014

LANGUAGE: ENGLISH

PUBLICATION-TYPE: Newspaper; Web Publication

JOURNAL-CODE: WEBDTNS

Copyright 2014 Telegraph Media Group Limited
All Rights Reserved

Daily Record & Sunday Mail

June 12, 2014 Thursday
Edition 1;
National Edition

9 tsps sugar in can of fizz

SECTION: NEWS; Pg. 27

LENGTH: 67 words

CAMPAIGNERS are calling for a limit on sugar in **fizzy drinks** after a study found it can make up a third of a teen's recommended daily intake.

Of 232 **soft drinks,** Fentimans Traditional Curiosity Cola had the most **sugar** at 9.3 teaspoons. Sainsbury's Classic Cola had 8.9, and Coke and Pepsi had 8.7.

Action on **Sugar** are demanding limits but the British **Soft Drinks** Association accused them of "political zeal".

LOAD-DATE: June 12, 2014

LANGUAGE: ENGLISH

PUBLICATION-TYPE: Newspaper

JOURNAL-CODE: DRC

Copyright 2014 Scottish Daily Record & Sunday Mail Ltd DAILY MAIL (London)

June 12, 2014 Thursday

UPMARKET' FIZZY ?DRINKS WITH MORE SUGAR THAN COKE

BYLINE: BY SEAN POULTER CONSUMER AFFAIRS EDITOR

LENGTH: 436 words

UPMARKET **soft drinks** may seem like healthy alternatives to big names like Coca-Cola but they contain far more **sugar**, researchers warn today.

Ginger beer, prestige lemonade, dandelion and burdock, and sparkling elderflower are some of the options now on the shelves.

But they can come with as much as 13 teaspoons of sugar in a bottle.

Big brand and cheaper supermarket versions of Coca-Cola and Pepsi weigh in at around nine teaspoons.

That compares to six or more teaspoons typically found in eight out of ten other high street **fizzy drinks**.

Health campaigners who compiled the figures warn that adults and children are consuming huge quantities of hidden sugar in processed food and drink, which is fuelling obesity and poor health.

The World Health Organisation recommends a drastic cut to a maximum of about 25g a day, which equates to six teaspoons.

But Action on Sugar analysed 232 popular drinks and found that 79 per cent contained this amount of sugar in a single 330ml serving.

The group is urging Health Secretary Jeremy Hunt to put pressure on manufacturers and supermarkets to make their drinks less sweet by cutting sugar levels.

Chairman Professor Graham MacGregor said: Added sugars are completely unnecessary in our diets and are strongly linked to obesity and type 2 diabetes.'

Prof MacGregor, of the Wolfson Institute, London, also warned that sugar was adding to the major problem of dental decay.

He said: Replacing sugar with sweeteners is not the answer, we need to reduce overall sweetness so people's tastes can adjust to having less sweet drinks.'

A similar approach has led to a 15 per cent drop in salt intake over ten years, saving 9,000 lives a year plus reducing annual health spending by £1.5billion, added the professor. He said: It is time to do the same for sugar.'

The research found that sugar levels in apparently similar products showed wide variations, suggesting the amount could be cut.

Kawther Hashem, nutritionist at Action on Sugar, advised: Look on the label for "sugar per 100g" and switch to a lower or no added sugar variety, or even better, don't drink them, they contain nothing of any nutritional value.'

The British Soft Drinks Association claimed the campaigners were blinded by political zeal'.

Director general Gavin Partington said manufacturers have led the way in providing an increasing range of low and no-calorie drinks'.

Dr Glenys Jones, nutritionist at Sugar Nutrition UK, a body financed by manufacturers, said: Like all sources of calories, sugars and sugar-containing food and drinks can be consumed within a healthy, balanced diet and active lifestyle.'

© Daily Mail

LOAD-DATE: June 11, 2014

LANGUAGE: ENGLISH

PUBLICATION-TYPE: Papers

Copyright 2014 Associated Newspapers Ltd.
All Rights Reserved

Daily Mirror

June 12, 2014 Thursday
Edition 1;

Northern Ireland

9 tsps sugar in can of fizz

SECTION: NEWS; Pg. 11

LENGTH: 77 words

CAMPAIGNERS are calling for a limit on sugar in **fizzy drinks** after a study found it can be a third of a teen's recommended daily intake.

Of 232 **soft drinks,** Fentimans Traditional Curiosity Cola had most **sugar** at 9.3 teaspoons. Sainsbury's Classic Cola had 8.9, and Coke and Pepsi had 8.7.

Action on **Sugar's** Prof Graham MacGregor called for "targets for **sugar** reduction". Gavin Partington of the British **Soft Drinks** Association accused campaigners of "political zeal".

LOAD-DATE: June 12, 2014

LANGUAGE: ENGLISH

GRAPHIC: TOO SWEET Sainsbury's cola

PUBLICATION-TYPE: Newspaper

JOURNAL-CODE: DMR

Copyright 2014 MGN Ltd.
All Rights Reserved

257 of 371 DOCUMENTS

The Daily Telegraph
The Daily Telegraph (London)

June 12, 2014 Thursday
Edition 1;
National Edition

San Pellegrino has more sugar than Coke

BYLINE: Sarah Knapton

SECTION: NEWS; Pg. 13

LENGTH: 587 words

UPMARKETfizzy drinks such as San Pellegrino and Fentimans can contain more sugar than a can of Coca-Cola, research has found.

Although cheaper drinks are often blamed for using sugar as a substitute for more expensive ingredients, the research showed that artisan **beverages** hold more **sugar** than cheaper blends.

The campaign group Action for Sugar warned that people were unwittingly drinking spoonfuls of sugar and called for new targets to reduce the content.

The World Health Organisation advises that adults have no more than 12 teaspoons of sugar a day but is considering dropping the level to six, because it is so harmful.

A 330ml can of San Pellegrino Pompelmo contains 10 teaspoons of sugar compared with nine in a can of Coca-Cola, researchers found.

Fentimans Traditional Curiosity Cola has 11.3g of sugar per 100ml, the equivalent of nine teaspoons in a 330ml serving. Its Cherrytree Cola has 10 teaspoons of sugar. **Fizzy drinks** from Waitrose, Sainsbury's and Marks & Spencer were also found to have unhealthy levels of sugar, according to health experts.

Sainsbury's cloudy lemonade contains 11 teaspoons of sugar per 330ml; Waitrose cream soda nine teaspoons and Marks & Spencer ginger beer 11 teaspoons.

"Added sugars are completely unnec-essary in our diets and are strongly linked to obesity and type 2 diabetes as well as to dental caries, which remains a major problem for children and adults," said Graham MacGregor, professor of cardiovascular medicine at the Wolfson Institute, Queen Mary University of London. "We urge the Health Secretary to set incremental targets for sugar reduction now and to start with these **sugary drinks**.

Replacing sugar with sweeteners is not the answer. We need to reduce overall sweetness so people's tastes can adjust to having less sweet drinks."

The survey of 232 drinks from leading supermarkets also found that Asda, Lidl and Tesco cola contained more sugar than Coca-Cola. Elderflower drinks were surprisingly high in sugar. Shloer white grape and elderflower had nine teaspoons per 330ml.

Kawther Hashem, a nutritionist at Action on Sugar said: "People are drinking spoonfuls of sugar in their **fizzy drinks**; even seemingly healthier options such as elderflower can be loaded with sugar. Look on the label for 'sugar per 100g' and switch to a lower or no added sugar variety of your favourite drink, or even better, don't drink them, they contain nothing of any nutritional value. Drink water and save money too!" Fentimans said it was committed to bringing down the sugar content of its **fizzy drinks**.

A spokesman for Marks and Spencer said: "We provide detailed nutritional information on all our products including carbonated drinks, so customers can make an informed choice."

Waitrose said it had removed 7.1 tons of sugar from its chilled juices and would turn its attention to carbonated drinks.

A spokesman for Sainsbury said: "We have a very strong record of sugar reduction across our product range and have tripled our range of no-added-sugar carbonated drinks, from four to 12."

Tsp per 300ml serving Waitrose ginger beer 11 M&S Fiery ginger beer 11 Sainsbury's cloudy lemonade 11 San Pellegrino Pompelmo 10 San Pellegrino Aranciata Rosso 10 Fentimans dandelion and burdock 10 Fentimans Cherrytree Cola 10 Sainsbury's lemonade 9 Fentimans Traditional Curiosity Cola 9 Shloer white grape, apple and cranberry 9 Waitrose sparkling lemonade 9 Sainsbury's classic cola 9 Shloer white grapeand elderflower 9 Essential Waitrose cola 9 M&S cola 9 Coca-Cola 9 Waitrose cream soda 9

LOAD-DATE: June 12, 2014

LANGUAGE: ENGLISH

PUBLICATION-TYPE: Newspaper

JOURNAL-CODE: DTL

Copyright 2014 Telegraph Media Group Limited
All Rights Reserved

The Daily Telegraph

The Daily Telegraph (London)

June 12, 2014 Thursday
Edition 1;
Scotland

San Pellegrino contains more sugar than Coke, study finds

BYLINE: Sarah Knapton

SECTION: NEWS; Pg. 14

LENGTH: 425 words

UPMARKETfizzy drinks such as San Pellegrino and Fentimans can contain more sugar than a can of Coca-Cola, research has found.

Although cheaper drinks are often blamed for using sugar as a substitute for more expensive ingredients, the research showed that artisan **beverages** hold more **sugar** than cheaper blends.

The campaign group Action for Sugar called for new targets to reduce the content. The World Health Organisation advises that adults have no more than 12 teaspoons of sugar a day but is considering dropping the level to six, because it is so harmful. A 330ml can of San Pellegrino Pompelmo contains 10 teaspoons of sugar compared with nine in a can of Coca-Cola, researchers found.

Fentimans Traditional Curiosity Cola has 11.3g of sugar per 100ml, the equivalent of nine teaspoons in a 330ml serving. Its Cherrytree Cola has 10 teaspoons of sugar. **Fizzy drinks** from Waitrose, Sainsbury's and Marks & Spencer were also found to have unhealthy levels of sugar, according to health experts.

Sainsbury's cloudy lemonade contains 11 teaspoons of sugar per 330ml; Waitrose cream soda nine teaspoons and Marks & Spencer ginger beer 11 teaspoons.

"Added sugars are completely unnecessary in our diets and are strongly linked to obesity and type 2 diabetes as well as to dental caries, which remains a major problem for children and adults," said Graham MacGregor, professor of cardiovascular medicine at the Wolfson Institute, Queen Mary University of London .

"We urge the Health Secretary to set incremental targets for sugar reduction now and to start with these **sugary drinks**. Replacing sugar with sweeteners is not the answer. We need to reduce overall sweetness so people's tastes can adjust to having less sweet drinks."

The survey of 232 drinks from leading supermarkets also found that Asda, Lidl and Tesco cola contained more sugar than Coca-Cola. Elderflower drinks were surprisingly high in sugar. Shloer white grape and elderflower had nine teaspoons per 330ml.

Fentimans said it was committed to bringing down the sugar content of its **fizzy drinks**.

A spokesman for Marks and Spencer said: "We provide detailed nutritional information on all our products including carbonated drinks, so customers can make an informed choice."

Waitrose said it had removed 7.1 tons of sugar from its chilled juices and would turn its attention to carbonated drinks.

A spokesman for Sainsbury said: "We have a very strong record of sugar reduction across our product range and have tripled our range of no-added-sugar carbonated drinks, from four to 12."

LOAD-DATE: June 12, 2014

LANGUAGE: ENGLISH

PUBLICATION-TYPE: Newspaper

JOURNAL-CODE: DTLscot

Copyright 2014 Telegraph Media Group Limited
All Rights Reserved

The Express

June 12, 2014 Thursday
Edition 1;
National Edition

'Set a limit on sugar in fizzy drinks'

BYLINE: Jo Willey

SECTION: NEWS; Pg. 8

LENGTH: 160 words

CAMPAIGNERS are demanding action to slash the "shockingly high and unnecessary" levels of sugar in cans of **fizzy drinks**.

Analysis of 232 sugar-sweetened drinks found that 79 per cent contain six or more teaspoons of sugar per can - more than the World Health Organisation's recommended maximum daily limit for all sugar consumption.

The study, conducted by Action On Sugar, found a typical can of cola contains as much sugar as three-and-a-half doughnuts.

Professor Graham MacGregor, chairman of Action On Sugar, said: "Added sugar is completely unnecessary in our diets and is strongly linked to obesity and Type 2 diabetes, as well as to dental decay. We urge the Secretary of State for Health Jeremy Hunt to set incremental targets for sugar reduction now - and to start with these **sugary drinks**."

Gavin Partington of the British Soft Drinks Association said: "Government figures show soft drinks contribute just three per cent of calories to the average diet."

LOAD-DATE: June 12, 2014

LANGUAGE: ENGLISH

PUBLICATION-TYPE: Newspaper

JOURNAL-CODE: DXP

Copyright 2014 Express Newspapers
All Rights Reserved

The Express

June 12, 2014 Thursday

'Set a limit on sugar in **fizzy drinks**'

BYLINE: Jo Willey

SECTION: NEWS; Pg. 23

LENGTH: 196 words

CAMPAIGNERS are demanding action to slash the "shockingly high and unnecessary" levels of sugar in cans of **fizzy drinks**.

Analysis of 232 sugar-sweetened drinks found that 79 per cent contain six or more teaspoons of sugar per can - more than the World Health Organisation's recommended maximum daily limit for all sugar consumption.

The study, conducted by Action On Sugar, found a typical can of cola contains as much sugar as threeand-a-half doughnuts. Professor Graham MacGregor, chairman of Action On Sugar, said: "Added sugar is completely unnecessary in our diets and is strongly linked to obesity and Type 2 diabetes, as well as to dental decay. We urge the Secretary of State for Health Jeremy Hunt to set incremental targets for sugar reduction now - and to start with these **sugary drinks**.

"A similar approach has reduced salt intake; people are consuming 15 per cent less salt than they were 10 years ago. This policy is estimated to be saving 9,000 lives a year, plus healthcare savings of £1.5billion a year."

Gavin Partington of the British Soft Drinks Association said: "Government figures show soft drinks contribute just three per cent of calories to the average diet."

LOAD-DATE: June 12, 2014

LANGUAGE: ENGLISH

PUBLICATION-TYPE: Newspaper

JOURNAL-CODE: DXP

Copyright 2014 Express Newspapers
All Rights Reserved

The Guardian - Final Edition

June 12, 2014 Thursday

Ginger beers found to have more sugar than colas

BYLINE: Sarah Boseley, Health editor

SECTION: GUARDIAN HOME PAGES; Pg. 13

LENGTH: 325 words

Traditional and upmarket **fizzy drinks** such as ginger beer and cloudy lemonade contain more sugar than Coca-Cola and Pepsi, according to a new analysis.

Nearly four out of five (79%) 330ml cans of **fizzy drinks** contain more than six teaspoons of sugar, according to Action on Sugar, which has analysed 232 drinks.

The worst offenders are ginger beers, such as Old Jamaica ginger beer and Jammin sparkling ginger beer flavour drink, which have the equivalent of 13 tsp of sugar per 330ml. Ginger beers by Waitrose, Marks & Spencer and Sainsbury's have 11. Coca-Cola and Pepsi have nine. Also high in sugar are Club Orange (12 tsp), Sainsbury's cloudy lemonade (11 tsp) and Fanta grape-flavoured drink (11 tsp).

Action on Sugar says almost all the sweetened drinks have far too much sugar. Coca-Cola, Pepsi, Sprite and Dr Pepper are particularly concerning because of the high number of cans drunk. Coca-Cola has just announced a new version, with lower sugar, but campaigners say it will still contain the equivalent of 4 tsp.

The survey has been published before a report on carbohydrates in the national diet from the government's nutrition advisory body, which may recommend lower levels. The World Health Organisation recently set a guideline limit of 10% of calories from **sugar**, with a recommended target of 5%. A recent official national diet survey showed children in England were consuming about 15% of their calories as **sugars**, and a third of those came from **soft drinks** and fruit juices.

Professor Graham MacGregor of Action on Sugar urged the health secretary, Jeremy Hunt, to set incremental targets for sugar reduction now - and to start with drinks. He added: "Replacing sugar with sweeteners is not the answer: we need to reduce overall sweetness so people's tastes can adjust to having less sweet drinks. A similar approach has reduced salt intake: people are consuming 15% less salt than they were 10 years ago, and now prefer less salty foods."

LOAD-DATE: June 12, 2014

LANGUAGE: ENGLISH

PUBLICATION-TYPE: Newspaper

Copyright 2014 Guardian Newspapers Limited
All Rights Reserved

theguardian
Guardian.com.

June 12, 2014 Thursday

Traditional fizzy drinks 'contain more sugar than Coca-Cola'

BYLINE: Sarah Boseleytheguardian.com

LENGTH: 501 words

ABSTRACT

Campaign group warns of risks of sugar consumption leading to obesity and diabetes

FULL TEXT

Traditional and upmarket **fizzy drinks** such as ginger beer and cloudy lemonade contain more sugar than Coca-Cola and Pepsi, according to a new analysis.

Nearly four out of five (79%) 330ml cans of **fizzy drinks** contain more than six teaspoons of sugar, according to the campaign Action on Sugar, which has analysed 232 drinks sold in leading supermarkets.

The worst offenders are ginger beers, such as Old Jamaica ginger beer and Jammin sparkling ginger beer flavour drink, which have the equivalent of 13 teaspoons of sugar per 330ml serving. Waitrose, Marks & Spencer and Sainsbury's ginger beers have 11. Coca-Cola and Pepsi have nine.

Also very high in sugar are Club Orange (12 teaspoons), Sainsbury's cloudy lemonade (11 teaspoons) and Fanta grape-flavoured drink (11 teaspoons). Other drinks which some people may chose out of an assumption that they are healthier, such as elderflower and grape juice, also exceed the sugar levels in colas.

Action on Sugar says almost all the sweetened drinks have far too much sugar for our health. Coca-Cola, Pepsi and other popular drinks like Sprite and Dr Pepper are particularly concerning because of the numbers of people who drink them and the frequency with which they do so. Coca-Cola has just announced a new version, with lower sugar, but campaigners protest that it will still contain the equivalent of four teaspooons.

The survey is published before a report, expected later this month, on carbohydrates including **sugar** in the national diet from the government's nutrition advisory body, SACN, which may recommend lower levels. The World Health Organisation has recently set a guideline limit of 10% of daily calories from **sugar**, with a recommendation that countries should aim to get it down as low as 5%. However, the recent official national diet and nutrition survey showed adults and children in England were exceeding the 11% current target - children were consuming around 15% of their calories as **sugars**, and a third of those came from **soft drinks** and fruit juices.

"Added sugars are completely unnecessary in our diets and [are] strongly linked to obesity and type 2 diabetes, as well as to dental caries which remains a major problem for children and adults," said Professor Graham MacGregor of Action on Sugar, who also runs a campaign to lower salt intake.

"We urge the secretary of state for health, Jeremy Hunt MP, to set incremental targets for sugar reduction now - and to start with these **sugary drinks**. Replacing sugar with sweeteners is not the answer: we need to reduce overall sweetness so people's tastes can adjust to having less sweet drinks.

"A similar approach has successfully reduced salt intake; people are consuming 15% less salt than they were 10 years ago, and now prefer less salty foods. This policy is estimated to be saving 9,000 lives a year, plus healthcare savings of £1.5bn a year. It is now time to do the same for sugar."

LOAD-DATE: June 11, 2014

LANGUAGE: ENGLISH

PUBLICATION-TYPE: Newspaper

Copyright 2014 Guardian News and Media Limited or its affiliated companies
All Rights Reserved
Registered in England and Wales. No. 908396
Registered office: PO Box 68164, Kings Place, 90 York Way, London N1P 2AP

The INDEPENDENT
Independent.co.uk

June 12, 2014 Thursday 6:38 AM GMT

Two-thirds of ginger beer contains more sugar that Coca-Cola, research warns; Perhaps it explains why the Famous Five always had so much energy for adventures

BYLINE: Chris Green

SECTION: FRONTPAGE

LENGTH: 599 words

The UK's most popular brand of ginger beer has more than 13 teaspoons of sugar in a single can, according to research published today.

Old Jamaica Ginger Beer, which has been sold in the UK since 1988, was found to contain 52.8g of sugar in a standard 330ml can. Overall, almost two thirds of ginger beer drinks tested had more sugar in them than a can of Coca-Cola.

The research was conducted by Action on Sugar, a campaign group made up of scientists and other specialists concerned about the effect of too much sugar on people's health. They analysed 232 sugar-sweetened drinks taken from the shelves of leading supermarkets last month.

The results showed that 79 per cent contained six or more teaspoons of sugar per can - more than the World Health Organization's recommended daily maximum - and that a typical can of cola had as much sugar as three and a half original glazed Krispy Kreme donuts.

Researchers warned that some products that might be associated by consumers with healthy ingredients or a wholesome image - such as ginger beer, which is knocked back in great quantities by the children in Enid Blyton's Famous Five series - may in fact contain lots of added sugar. Shloer White Grape and Elderflower contained 8.8 teaspoons of sugar in a 330ml serving, the survey showed.

"They shouldn't assume that just because it says ginger or another fruit that all the sugar content comes from that specific fruit," said nutritionist Kawther Hashem, who helped conduct the research. "We'd encourage them to look at the labelling and the list of ingredients. If sugar's the first or second ingredient ... that means that the product contains more."

Action on Sugar said the research also highlighted large variations in the sugar content of very similar products, demonstrating that sugar levels could be reduced significantly without dramatically affecting taste. Old Jamaica Ginger Beer contained 16g of sugar per 100ml, whereas Asda's Chosen by You variety contained just 7g.

A spokesperson for Old Jamaica Ginger Beer said the drink was "produced using a traditional recipe, which includes authentic Jamaican root ginger, to create its trademark fiery taste and is designed to be a treat. We also offer a light version of Old Jamaica Ginger Beer which delivers the same flavour intensity but with no sugar."

Professor Graham MacGregor, a specialist in cardiovascular medicine at the Wolfson Institute, Queen Mary University of London and the group's chairman, said added sugars were a "completely unnecessary" part of human diets and contributed to high levels of obesity, diabetes and tooth decay.

"We urge the Secretary of State for Health, Jeremy Hunt, to set incremental targets for sugar reduction now - and to start with these **sugary drinks**," he said. "Replacing sugar with sweeteners is not the answer: we need to reduce overall sweetness so people's tastes can adjust to having fewer sweet drinks."

But Gavin Partington, director general of the British **Soft Drinks** Association, said Action of **Sugar** had been "blinded by political zeal", adding: "These campaigners appear to have missed the 60 per cent of **soft drinks** on the market which contain no added **sugar**. Perhaps unsurprisingly, they have also ignored the evidence that shows obesity arises from an imbalance of calories consumed and calories expended and is not caused by one particular ingredient."

A spokeswoman for the Department of Health said: "As a nation, we need to consume less sugar. We are working with the food and drinks industry to reduce the amount of sugar in products and make healthier alternatives available."

LOAD-DATE: June 12, 2014

LANGUAGE: ENGLISH

PUBLICATION-TYPE: Newspaper; Web Publication

JOURNAL-CODE: WEBI

Copyright 2014 Independent Digital News and Media Limited
All Rights Reserved

The INDEPENDENT

Independent.co.uk

June 12, 2014 Thursday 12:28 AM GMT

Sweet drinks increasingly contain dangerous amounts of sugar, according to research;

Perhaps it explains why the Famous Five always had so much energy for adventures.

BYLINE: Chris Green

SECTION: FRONTPAGE

LENGTH: 598 words

The UK's most popular brand of ginger beer has more than 13 teaspoons of sugar in a single can, according to research published today.

Old Jamaica Ginger Beer, which has been sold in the UK since 1988, was found to contain 52.8g of sugar in a standard 330ml can. Overall, almost two thirds of ginger beer drinks tested had more sugar in them than a can of Coca-Cola.

The research was conducted by Action on Sugar, a campaign group made up of scientists and other specialists concerned about the effect of too much sugar on people's health. They analysed 232 sugar-sweetened drinks taken from the shelves of leading supermarkets last month.

The results showed that 79 per cent contained six or more teaspoons of sugar per can - more than the World Health Organization's recommended daily maximum - and that a typical can of cola had as much sugar as three and half original glazed Krispy Kreme donuts.

Researchers warned that some products that might be associated by consumers with healthy ingredients or a wholesome image - such as ginger beer, which is knocked back in great quantities by the children in Enid Blyton's Famous Five series - may in fact contain lots of added sugar. Shloer White Grape and Elderflower contained 8.8 teaspoons of sugar in a 330ml serving, the survey showed.

"They shouldn't assume that just because it says ginger or another fruit that all the sugar content comes from that specific fruit," said nutritionist Kawther Hashem, who helped conduct the research. "We'd encourage them to look at the labelling and the list of ingredients. If sugar's the first or second ingredient ... that means that the product contains more."

Action on Sugar said the research also highlighted large variations in the sugar content of very similar products, demonstrating that sugar levels could be reduced significantly without dramatically affecting taste. Old Jamaica Ginger Beer contained 16g of sugar per 100ml, whereas Asda's Chosen by You variety contained just 7g.

A spokesperson for Old Jamaica Ginger Beer said the drink was "produced using a traditional recipe, which includes authentic Jamaican root ginger, to create its trademark fiery taste and is designed to be a treat. We also offer a light version of Old Jamaica Ginger Beer which delivers the same flavour intensity but with no sugar."

Professor Graham MacGregor, a specialist in cardiovascular medicine at the Wolfson Institute, Queen Mary University of London and the group's chairman, said added sugars were a "completely unnecessary" part of human diets and contributed to high levels of obesity, diabetes and tooth decay.

"We urge the Secretary of State for Health, Jeremy Hunt, to set incremental targets for sugar reduction now - and to start with these **sugary drinks**," he said. "Replacing sugar with sweeteners is not the answer: we need to reduce overall sweetness so people's tastes can adjust to having fewer sweet drinks."

But Gavin Partington, director general of the British **Soft Drinks** Association, said Action of **Sugar** had been "blinded by political zeal", adding: "These campaigners appear to have missed the 60 per cent of **soft drinks** on the market which contain no added **sugar**. Perhaps unsurprisingly, they have also ignored the evidence that shows obesity arises from an imbalance of calories consumed and calories expended and is not caused by one particular ingredient."

A spokeswoman for the Department of Health said: "As a nation, we need to consume less sugar. We are working with the food and drinks industry to reduce the amount of sugar in products and make healthier alternatives available."

LOAD-DATE: June 12, 2014

LANGUAGE: ENGLISH

PUBLICATION-TYPE: Newspaper; Web Publication

JOURNAL-CODE: WEBI

Copyright 2014 Independent Digital News and Media Limited
All Rights Reserved

The INDEPENDENT

The Independent (London)

June 12, 2014 Thursday
First Edition

'Lashings of ginger beer' could lead to a sticky end

BYLINE: CHRIS GREEN SENIOR REPORTER

SECTION: NEWS; Pg. 22

LENGTH: 491 words

Perhaps it explains why the Famous Five always had so much energy for adventures. Britain's most popular brand of ginger beer has more than 13 teaspoons of sugar in a single can, according to research published today.

Old Jamaica Ginger Beer, sold in the UK since 1988, was found to contain 52.8g of sugar in a 330ml can. Overall, almost two-thirds of ginger beer drinks tested had more sugar in them than a can of Coca-Cola.

The research was conducted by Action on Sugar, a group of specialists concerned about the effect of too much sugar on people's health. They analysed 232 sugar-sweetened drinks taken from the shelves of leading supermarkets last month, and found that 79 per cent contained six or more teaspoons of sugar per can - more than the World Health Organisation's recommended daily maximum.

Researchers warned that some products that might be associated by consumers with healthy ingredients or a wholesome image - such as ginger beer, which is knocked back in great quantities by the children in Enid Blyton's Famous Five series - may in fact contain lots of added sugar. Shloer White Grape and Elderflower contained 8.8 teaspoons of sugar in a 330ml serving, the survey showed.

"They should not assume that just because it says 'ginger' or another fruit that all the sugar content comes from that specific fruit," said Kawther Hashem, a nutritionist who helped to conduct the research. "We would encourage them to look at the labelling and the list of ingredients. If sugar's the first or second ingredient??? that means that the product contains more."

Action on Sugar said the findings also highlighted large variations in the sugar content of similar products, demonstrating that sugar levels could be reduced significantly without dramatically affecting taste. Old Jamaica Ginger Beer contained 16g of sugar per 100ml, whereas Asda's Chosen by You variety contained just 7g.

A spokesperson for Old Jamaica Ginger Beer said: "[It is] produced using a traditional recipe, which includes authentic Jamaican root ginger to create its trademark fiery taste, and is designed to be a treat. We also offer a light version of Old Jamaica Ginger Beer which delivers the same flavour intensity but with no sugar."

Professor Graham MacGregor, a cardiovascular specialist at the Wolfson Institute, Queen Mary University of London, and the group's chairman, said added sugars were a "completely unnecessary" part of diets and contributed to high levels of obesity, diabetes and tooth decay.

But Gavin Partington, director general of the British **Soft Drinks** Association, said Action on **Sugar** had been "blinded by political zeal", adding: "These campaigners appear to have missed the 60 per cent of **soft drinks** on the market which contain no added **sugar**. Perhaps unsurprisingly, they have also ignored the evidence that shows obesity arises from an imbalance of calories consumed and calories expended and is not caused by one particular ingredient."

LOAD-DATE: June 11, 2014

LANGUAGE: ENGLISH

PUBLICATION-TYPE: Newspaper

JOURNAL-CODE: IA

Copyright 2014 Independent Print Ltd
All Rights Reserved

Mirror
mirror.co.uk

June 12, 2014 Thursday 5:19 PM GMT

Health pressure group urges sugar limit in **fizzy drinks** to help curb obesity epidemic; Call for urgent Government action as study shows one third of the average British teenager's sugar intake is from pop alone

BYLINE: By Ruki Sayid

SECTION: NEWS,UK NEWS

LENGTH: 518 words

Health pressure group urges sugar limit in fizzy drinks to help curb obesity epidemic; Call for urgent Government action as study shows one third of the average British teenager's sugar intake is from pop alone

mirror.co.uk June 12, 2014 Thursday 5:19 PM GMT

Health campaigners are calling for restrictions on the amount of sugar **fizzy drinks** makers can use, after research showed a third of the average teenager's sugar intake was from pop.

An analysis of 232 drinks found more than nine teaspoons of sugar in supermarket cola. And 15 leading big brands and own labels were packed with at least eight teaspoons in a 330ml serving.

Action on Sugar is urging the Government to crack down on permitted levels in a bid to tackle the nation's obesity crisis.

While cola was one of the worst culprits for sugar overload, research revealed added sugar was just as high in alternative drinks like ginger beer and sparkling elderflower. Six out of ten brands of ginger beer were found to contain more sugar than a regular can of Coke with up to 13.2 teaspoons compared to 8.7 in classic Coke.

Elderflower drinks had as much sugar as three and a half Krispy Kreme donuts. Cloudy lemonade had 11.1 teaspoons of sugar, dandelion and burdock 9.7 teaspoons and ginger ale 7.7 per 330mls.

Action on Sugar examined drinks from nine supermarkets and discount stores and found Fentimans Traditional Curiosity Cola topped the sugar table with 9.3 teaspoons per 330ml glass. Lidl Freeway Cola and Tesco Classic Cola contained nine teaspoons, Sainsbury's Classic Cola had 8.9, while colas from Marks & Spencer, Waitrose and Asda were also high with 8.8 teaspoons.

Big brands Coke and Pepsi contained 8.7 teaspoons - the same as Morrisons and Aldi Vive Original, the study found.

Action group chairman, Professor Graham MacGregor, said: "Added sugars are completely unnecessary in our diets and are strongly linked to obesity and type 2 diabetes, as well as tooth decay - which remains a major problem for children and adults.

"We urge the Secretary of State for Health, Jeremy Hunt, to set incremental targets for sugar reduction now - and to start with these **sugary drinks**. Replacing sugar with sweeteners is not the answer, we need to reduce overall sweetness so people's tastes can adjust to having less sweet drinks.

"A similar approach has successfully reduced salt intake. People are consuming 15% less salt than they were 10 years ago, and now prefer less salty foods. This policy is estimated to be saving 9,000 lives a year, plus healthcare savings of £1.5billion a year. It is time to do the same for sugar."

The World Health Organisation recently lowered its recommendation for sugar from 10% of our energy intake to 5% - but Brits are consuming three times the suggested daily level.

Action on Sugar's Dr Aseem Malhora said: "One **sugary drink** per day is associated with an increased risk of 22% for type 2 diabetes - even in the non-obese."

But the British **Soft Drinks** Association accused health campaigners of "being blinded by political zeal". Director general Gavin Partington said: "These campaigners appear to have missed the 60% of **soft drinks** on the market which contain no added **sugar**. Perhaps, unsurprisingly, they have also ignored the evidence that shows obesity arises from an imbalance of calories consumed and calories expended and is not caused by one particular ingredient."

LOAD-DATE: September 4, 2014

LANGUAGE: ENGLISH

PUBLICATION-TYPE: Newspaper; Web Publication

JOURNAL-CODE: WEBDMI

Copyright 2014 Trinity Mirror, Plc.

Health pressure group urges sugar limit in fizzy drinks to help curb obesity epidemic; Call for urgent Government action as study shows one third of the average British teenager's sugar intake is from pop alone mirror.co.uk June 12, 2014 Thursday 5:19 PM GMT

All Rights Reserved

The Sun (England)

June 12, 2014 Thursday
Edition 1;
Northern Ireland

Fizzy pop yer clogs

SECTION: NEWS; Pg. 9

LENGTH: 68 words

FIZZY drinks containing as many as 13 teaspoons of sugar may be "killing us", experts warn.

Forty-two types of shop pop have the sugar of four Krispy Kreme donuts.

Old Jamaica Ginger Beer has 13 teaspoons in a 330ml can, while Sainsbury's cloudy lemonade has 11. The daily recommended limit is six.

Prof Graham MacGregor, of Action on Sugar, said: "Added sugar is strongly linked to obesity and type 2 diabetes."

LOAD-DATE: June 12, 2014

LANGUAGE: ENGLISH

PUBLICATION-TYPE: Newspaper

JOURNAL-CODE: SUN

Copyright 2014 NEWS GROUP NEWSPAPERS LTD
All Rights Reserved

The Times (London)

June 12, 2014 Thursday
Edition 2;
National Edition

'Middle-class' soft drinks contain most sugar

BYLINE: Oliver Moody

SECTION: NEWS; Pg. 13

LENGTH: 548 words

Staples of the middle-class picnic including many types of ginger beer and sparkling elderflower drinks contain more sugar than Coca-Cola.

Several brands of cloudy lemonade, including Sainsbury's, Waitrose and Fentimans, were also found to have more than ten teaspoons of sugar in each 330ml serving, according to a study by anti-sugar campaigners.

The worst offender of 232 **fizzy drinks** analysed by Action on Sugar was Old Jamaica "extra fiery" ginger beer, with 52g, or 13 teaspoons, of sugar in every 330ml - more than twice the World Health Organisation's recommended maximum daily intake and four teaspoons more than a can of Coke.

Club Orange, a carbonated drink produced by Britvic, was found to contain 47g of sugar per 330ml, while Sainsbury's cloudy lemonade was said to have 45g, just under 12 teaspoons and just over 11 respectively.

In all, four out of five sweetened drinks had more sugar per can than the WHO's daily limit of six teaspoons.

The research follows a law passed in California last week that forces some drinks manufacturers to put health warnings on their products. There have been calls for similar measures to be introduced in the UK.

Yesterday Coca-Cola announced that it would launch a new reducedcalorie drink to help in the struggle against obesity. Coca-Cola Life, piloted last year in South America, is sweetened with a mixture of sugar and stevia leaf extract. A 330ml can will contain 89 calories, compared with 139 calories in a can of "full-fat" Coke and less than one calorie in a Diet Coke.

However, campaigners said that people needed to learn not to crave sweet drinks, and that simply substituting sweeteners for sugar would not be enough.

Graham MacGregor, professor of cardiovascular medicine at Queen Mary, University of London, called on the health secretary to set national targets for sugar reduction as a matter of urgency. "Added sugars are completely unnecessary in our diets and are strongly linked to obesity and type 2 diabetes, as well as to dental caries, which remain a major problem for children and adults," he said.

"We urge the secretary of state for health, Jeremy Hunt, to set incremental targets for sugar reduction now - and to start with these **sugary drinks**.

"Replacing sugar with sweeteners is not the answer: we need to reduce overall sweetness so people's tastes can adjust to having less sweet drinks."

A department of health spokeswoman said that the government had made "real progress" in persuading big food and drinks companies to cut the sugar in their products.

"As a nation we need to consume less sugar," she said.

"We are working with the food and drinks industry to reduce the amount of sugar in products and make healthier alternatives available, and we are the first country in Europe to recommend simple, voluntary front-ofpack labelling.

"This will make it easier for us to know how much sugar we are consuming," she added.

Amount of sugar per 100ml Colas Fentimans Curiosity Cola: 11.3g Lidl Freeway Cola: 10.9g Sainsbury's Classic Cola: 10.8g Essential Waitrose Cola: 10.7g Coca Cola: 10.6g Sparkling elderflower drinks Shloer White Grape and Elderflower: 10.7g Pure Pret Grape and Elderflower: 10.6g Ginger beers Old Jamaica: 15.7g Barr's Originals: 14.1g Waitrose: 13.9g Marks and Spencer Fiery: 13.8g Fentimans: 9.4g

LOAD-DATE: June 12, 2014

LANGUAGE: ENGLISH

PUBLICATION-TYPE: Newspaper

JOURNAL-CODE: TIM

Copyright 2014 Times Newspapers Limited
All Rights Reserved

277 of 371 DOCUMENTS

DAILY MAIL (London)

June 11, 2014 Wednesday

NEW COKE WITH LEAF SWEETENER

BYLINE: BY NO BYLINE AVAILABLE

LENGTH: 161 words

A NEW type of Coca-Cola made with an extract from the leaves of a South American plant is to be launched later this year.

The company says it contains a third less sugar and calories than its standard cola, as part of an effort to tackle obesity.

Coca-Cola Life will go on sale in Britain in September after a pilot in Argentina and Chile. It is sweetened with a blend of sugar and stevia leaf extract, and a 330ml can will contain 89 calories. A standard 330ml can of Coca-Cola contains 35g of sugar, or 39 per cent of an adult's guideline daily amount (GDA). The new drink will contain 22.1g, or 25 per cent of the GDA.

The sweetener is sourced from the stevia plant, which is native to South America.

Coca-Cola said the new drink was part of its commitment to offer consumers reduced, low and no-calorie options.

The company is a signatory to the Government's Responsibility Deal, under which the food and drink industry has pledged to promote a healthier diet.

© Daily Mail

LOAD-DATE: June 10, 2014

LANGUAGE: ENGLISH

PUBLICATION-TYPE: Papers

Copyright 2014 Associated Newspapers Ltd.
All Rights Reserved

278 of 371 DOCUMENTS

The Daily Telegraph
The Daily Telegraph (London)

June 11, 2014 Wednesday
Edition 2;
National Edition

Coke turns over new leaf for latest cola

BYLINE: Graham Ruddick

SECTION: BUSINESS; Pg. 1

LENGTH: 182 words

COCA-COLA is to launch its first new cola in Britain for eight years with a naturally sweetened drink that it claims will contain a third less calories and sugar.

Coca-Cola Life, which is sweetened with a blend of sugar and stevia leaf extract, will go on sale in Britain in September following a pilot in Argentina and Chile.

The drink will be launched amid government efforts to tackle an obesity crisis in the UK.

A 330ml can of Coca-Cola Life will contain 89 calories and 22.1 grams of sugar, or 25pc of an adult's guideline daily amount. Regular Coca-Cola contains 35 grams of sugar.

Stevia leaf extract is naturally sourced from the stevia plant, which is native to South America.

There has been controversy in the US around stevia, which some claim poses a health risk. However, purified stevia extracts - the type which is used by Coca-Cola - have been cleared.

Coca-Cola Life is the first cola drink in the UK since the arrival of Coca-Cola Zero in 2006. However, Coca-Cola Great Britain already uses stevia leaf extract as a sweetener in its Sprite and Glaceau vitamin water brands.

LOAD-DATE: June 11, 2014

LANGUAGE: ENGLISH

PUBLICATION-TYPE: Newspaper

JOURNAL-CODE: DTL

Copyright 2014 Telegraph Media Group Limited
All Rights Reserved

FINANCIAL TIMES
Financial Times (London, England)

June 11, 2014 Wednesday
London Edition 1

Europe launch for Coke's natural soda; BEVERAGES

BYLINE: Scheherazade Daneshkhu, Consumer Industries Editor

SECTION: COMPANIES; Pg. 22

LENGTH: 387 words

Coca-Cola aims to battle falling **fizzy-drinks** sales in mature markets with a naturally sweetened soda that will have its European launch in the UK.

The September rollout date comes amid inten-sifying efforts by the British government and -public-health officials to tackle the growing problem of obesity.

Coca-Cola Life was piloted in Argentina and Chile last year. Muhtar Kent, chief executive, said a few months ago that the drink would be extended to more countries this year because "it has shown great promise in recruiting new and lapsed consumers into the sparkling category".

The soda, which abandons Coke's traditional and familiar red packaging in favour of -natural-looking green, is -flavoured with stevia leaf extract, a natural sweetener.

Its sugar and calorie content are two-thirds that of regular Coke - a 330ml can contains 89 calories, against 139 for regular Coke.

Its launch is the first new Coke in the UK since the arrival of Coca-Cola Zero in 2006. More than 40 per cent of the Coke sold in the UK is zero-calorie.

Consumers in the US and Europe are turning away from **sugary drinks** in the face of government and public-health campaigns against obesity.

In the UK, a campaigning group of medics and academics launched Action on Sugar in January with the aim of putting pressure on the food and **beverage** industry to cut the amount of **sugar** added to products by up to 30 per cent over five years.

A stronger preference among consumers for healthier, natural products has also hit sales of zero-calorie carbonated colas because of concerns about artificial sweeteners.

For the first time in nearly 15 years, Coca-Cola sold fewer **fizzy drinks** globally during the first three months of this year, and quarterly profit fell 7.5 per cent from a year ago.

Coke's **fizzy-drink** sales in the UK fell by double-digits in the first quarter - bigger than the overall 1 per cent global fall - which it said had been partly because of the introduction of smaller bottle and can sizes.

Finding natural sweeteners that match the taste of sugar has been a challenge, but Coke has had some success with -stevia-flavoured Sprite in the UK.

James Quincey, president of Coca-Cola Europe, said of Coca-Cola Life: "We have innovated to provide consumers with a new option with fewer calories."

Additional reporting by Shannon Bond in New York

LOAD-DATE: June 10, 2014

LANGUAGE: ENGLISH

PUBLICATION-TYPE: Newspaper

Copyright 2014 The Financial Times Ltd.
All Rights Reserved
Please do not cut and paste FT articles and redistribute by email or post to the web.

theguardian

The Guardian - Final Edition

June 11, 2014 Wednesday

Coca-Cola goes green but campaigners see red

BYLINE: Rebecca Smithers, Consumer affairs correspondent

SECTION: GUARDIAN HOME PAGES; Pg. 16

LENGTH: 238 words

Coca-Cola has announced plans to launch a new version of its bestselling **soft drink** in the UK this autumn with a third less **sugar** and a third fewer calories as part of government and industry efforts to tackle obesity.

Coca-Cola Life, first piloted in Argentina and Chile last year, would, the company said, help meet its pledges made under the UK government's voluntary anti-obesity drive - the responsibility deal - and would offer consumers a greater choice.

But health campaigners said the company was misleading shoppers. The Children's Food Campaign said the new product "still contains over four teaspoons of sugar per 330ml can, which equates to one-quarter of a child's daily recommended maximum intake of sugar".

Sweetened with a blend of sugar and naturally sourced stevia leaf extract, a 330ml can of Coca-Cola Life, which will come with striking green branding, contains 89 calories.

An extract from the stevia plant, which is native to South America, is already used in the company's Sprite and Glaceau vitamin water, as well as by other manufacturers.

More than 40% of the cola sold by Coca-Cola in the UK is already no-calorie, it says, including two zero-calorie options - Coca-Cola Zero and Diet Coke. James Quincey, president of Coca-Cola Europe, said: "As we work with others across society to address the public health challenge of obesity, we will continue to take actions that help people balance their lifestyles."

LOAD-DATE: June 11, 2014

LANGUAGE: ENGLISH

PUBLICATION-TYPE: Newspaper

Copyright 2014 Guardian Newspapers Limited
All Rights Reserved

283 of 371 DOCUMENTS

i-Independent Print Ltd

June 11, 2014
First Edition

New Life for Coca-Cola in UK; The Business Matrix The day at a glance BEVERAGES

SECTION: BUSINESS; Pg. 40

LENGTH: 42 words

Coca-Cola is to launch its first Coke drink in the UK in more than eight years. The low-calorie Coca-Cola Life is sweetened with sugar and the plant extract stevia. The drink was trialled in Chile and Argentina and will be on sale in the UK in September.

LOAD-DATE: June 10, 2014

LANGUAGE: ENGLISH

PUBLICATION-TYPE: Newspaper

JOURNAL-CODE: III

Copyright 2014 Independent Print Limited
All Rights Reserved

284 of 371 DOCUMENTS

The INDEPENDENT
Independent.co.uk

June 11, 2014 Wednesday 11:02 AM GMT

Coca Cola Life: 'Fewer calories' soft drink to launch in Britain; Coca Cola Life has a third less sugar and fewer calories than normal Coke

BYLINE: Laura Chesters

Coca Cola Life: 'Fewer calories' soft drink to launch in Britain; Coca Cola Life has a third less sugar and fewer calories than normal Coke Independent.co.uk June 11, 2014 Wednesday 11:02 AM GMT

SECTION: FRONTPAGE

LENGTH: 233 words

US drinks giant Coca Cola is launching its first coke drink in the UK in more than eight years and has picked Britain ahead of its home nation.

Coca Cola Life, a drink sweetened with stevia which has a third less sugar and a third fewer calories than normal Coca Cola, will launch in September. It trialled the drink in Chile and Argentina and the UK launch is the first in Europe.

Its last big launch was back in 2005/2006 with the Coke Zero brand - which was its largest product launch in 22 years.

The stevia sweetener, extracted from a South American leaf, gained European approval in 2011 and is being used in a variety of products to reduce calories. Coca Cola said it already uses it in Sprite and its Glacéau vitaminwater.

The group said more than 40 per cent of the coke it sells in the UK is already no-calorie. It sells 21 brands and over 100 products across Britain.

James Quincey, president Coca-Cola Europe, said: "With Coca-Cola Life, we have innovated to provide consumers with a new option with fewer calories. We were early signatories to the UK Government's Responsibility Deal."

Most of Coca Cola's UK products are made in Britain in one of its factories in East Kilbride, Edmonton, Wakefield and Sidcup.

Last month the group revealed it will sell a new bottled water - glacéau smartwater - a decade after its disastrous Dasani purified water brand had to be pulled following poor sales.

LOAD-DATE: June 11, 2014

LANGUAGE: ENGLISH

PUBLICATION-TYPE: Newspaper; Web Publication

JOURNAL-CODE: WEBI

Copyright 2014 Independent Digital News and Media Limited
All Rights Reserved

The INDEPENDENT

The Independent (London)

June 11, 2014 Wednesday
First Edition

Coca-Cola catches the green mood

SECTION: BUSINESS; Pg. 47

LENGTH: 62 words

Coca-Cola is to launch its first Coke drink in the UK in more than eight years. The low-calorie Coca-Cola Life is sweetened with a mix of sugar and the plant extract stevia. Its recycleable bottle has a green label. The drink was trialled in Chile and Argentina and will be on sale in the UK in September, ahead of the US and the rest of Europe.

AXEL INDIK/POLARIS /EYEVINE

LOAD-DATE: June 10, 2014

LANGUAGE: ENGLISH

PUBLICATION-TYPE: Newspaper

JOURNAL-CODE: IA

Copyright 2014 Independent Print Ltd
All Rights Reserved

MailOnline

June 11, 2014 Wednesday 2:07 PM GMT

New 'green' Coke with leaf sweetener from South America: Company says latest drink has a THIRD less sugar than standard version

BYLINE: PAUL DONNELLEY

SECTION: NEWS

LENGTH: 558 words

- Coca-Cola Life will go on sale in September after pilot in Argentina and Chile
- The new cola is sweetened with a blend of sugar and stevia leaf extract
- A 330ml can of Coca-Cola contains 35g of sugar, Coca-Cola Life 22.1g

Coca-Cola is to launch a naturally sweetened drink with a third less sugar and calories than its regular cola as part of government and industry-wide efforts to tackle obesity.

Coca-Cola Life will go on sale in Britain in September following a pilot in Argentina and Chile, making it the first new Coca-Cola since Coke Zero was launched in 2006.

It will also be sold in a green can.

The new cola is sweetened with a blend of sugar and stevia leaf extract, and a 330ml can will contain 89 calories.

A regular 330ml can of Coca-Cola contains 35 grams of sugar, or 39% of an adult's GDA (guideline daily amount).

Coca-Cola Life will contain 22.1 grams or 25% of an adult's GDA.

The two other Coca-Cola drinks, Coke Zero and Diet Coke, contain no calories.

Stevia leaf extract is naturally sourced from the stevia plant, which is native to South America.

Currently, Coca-Cola Great Britain uses stevia leaf extract as a sweetener in its Sprite and Glaceau vitamin water brands.

Coca-Cola said that the new drink was part of its commitments to offer consumers reduced, low and no-calorie options - adding that it was the 'most recent example in a series of initiatives by the company to inspire happier, healthier lives'.

The company is a signatory to the Government's Responsibility Deal, under which the food and drink industry has pledged to promote and healthier diet and make changes to their products, and Coca-Cola has committed to reduce the average calories per litre in its range of sparkling drinks by 5 per cent by the end of 2014.

Other than introducing stevia extract to Sprite, it has launched smaller 250ml cans across the Coca-Cola range and recently announced a £20 million anti-obesity fitness drive in 70 parks in Britain.

Early this year, a group of health experts launched a campaign to reduce the amount of **sugar** added to food and **soft drinks** as part of an effort to reverse the UK's obesity and diabetes crisis.

Action on Sugar said that the food industry would easily achieve a 20 to 30 per cent reduction in the amount of sugar added to products, which it said would result in a reduction of approximately 100 calories per day or more in those who are particularly prone to obesity.

https://www.youtube.com/watch?v=7M-WHEKJwmg]

It said the reduction could reverse or halt the obesity epidemic and would also have a significant impact in reducing chronic disease.

Coca-Cola Europe president James Quincey said: 'We are pleased to add Coca-Cola Life to the Coca-Cola portfolio in the UK.

'It complements our existing brands and is well-positioned to meet changing lifestyle trends, providing people with a great-tasting, lower calorie cola sweetened from natural sources.

'With Coca-Cola Life, we have innovated to provide consumers with a new option with fewer calories. We were early signatories to the UK Government's Responsibility Deal and as we work with others across society to address the public health challenge of obesity in the UK and across Europe, we will continue to take actions that help people balance their lifestyles.'

Coca-Cola Life will be available in a 330ml can, 500ml bottle and 1.75L bottle.

LOAD-DATE: June 11, 2014

LANGUAGE: ENGLISH

PUBLICATION-TYPE: Web Publication

JOURNAL-CODE: WEBDM

Copyright 2014 Associated Newspapers Ltd.
All Rights Reserved

Metro (UK)

June 11, 2014 Wednesday
Edition 1;
Scotland

COCA-COLA is launching a naturally [...]; BUSINESS BITES

SECTION: BUSINESS; Pg. 35

LENGTH: 70 words

COCA-COLA is launching a naturally sweetened drink with a third less sugar than regular cola. Coca-Cola Life goes on sale in September. A 330ml can contains 89 calories and is sweetened with a blend of sugar and stevia leaf extract. The move is in response to government efforts to reduce obesity. A regular can of Coca-Cola contains 139 calories and has 35g of sugar - 39 per cent of an adult's recommended daily amount.

LOAD-DATE: June 11, 2014

LANGUAGE: ENGLISH

PUBLICATION-TYPE: Newspaper

JOURNAL-CODE: MTRscot

Copyright 2014 Associated Newspapers Ltd.
All Rights Reserved

The Telegraph
telegraph.co.uk

June 11, 2014 Wednesday 6:00 AM GMT

Coca-Cola to launch new stevia-flavoured drink; Soft drink brand looks to cut sugar and calories in cola

BYLINE: By Graham Ruddick

SECTION: FINANCE

LENGTH: 294 words

Coca-Cola is to launch its first new cola drink in Britain for eight years with a naturally-sweetened drink that it claims will contain a third less calories and sugar.

Coca-Cola Life, which is sweetened with a blend of sugar and stevia leaf extract, will go on sale in Britain in September following a pilot in Argentina and Chile.

The drink will be launched amid Government efforts to tackle an obesity crisis in the UK.

A 330ml can of Coca-Cola Life will contain 89 calories and 22.1 grams of sugar, or 25pc of an adult's guideline daily amount. Regular Coca-Cola contains 35 grams of sugar.

Stevia leaf extract is naturally sourced from the stevia plant, which is native to South America.

There has been controversy in the United States around the health risks surrounding stevia, but purified stevia extracts, which is the type used by Coca-Cola, have been cleared.

Coca-Cola Life is the first cola drink in the UK since the arrival of Coca-Cola Zero in 2006. However, Coca-Cola Great Britain already uses stevia leaf extract as a sweetener in its Sprite and Glaceau vitamin water brands.

James Quincey, president of Coca-Cola Europe, said: "We are pleased to add Coca-Cola Life to the Coca-Cola portfolio in the UK. It complements our existing brands and is well-positioned to meet changing lifestyle trends, providing people with a great-tasting, lower calorie cola sweetened from natural sources.

"With Coca-Cola Life, we have innovated to provide consumers with a new option with fewer calories.

"We were early signatories to the UK Government's Responsibility Deal and as we work with others across society to address the public health challenge of obesity in the UK and across Europe, we will continue to take actions that help people balance their lifestyles."

LOAD-DATE: June 11, 2014

LANGUAGE: ENGLISH

PUBLICATION-TYPE: Newspaper; Web Publication

JOURNAL-CODE: WEBDTNS

Copyright 2014 Telegraph Media Group Limited
All Rights Reserved

All Rights Reserved

mirror.co.uk

June 10, 2014 Tuesday 11:29 PM GMT

Coca Cola Life to launch in UK as new low calorie alternative to classic brand; Distinctive green packaging of new Cola revealed and it contains a third less sugar than the red tinned variety

BYLINE: By Ruki Sayid

SECTION: NEWS,UK NEWS

LENGTH: 384 words

Fizzy drinks giant Coke is launching a new, lower calorie **soft drink**, following calls from health campaigners for big brands to slash **sugar** in their products.

It has ripped up the recipe book to create Coca Cola Life which has a third less sugar than classic Coke and is blended with sweet leaf extract stevia.

New green cans and bottle labels will feature alongside its range of drinks from the iconic 330ml red tins with 139 calories to Coke Light and Zero with no calories.

Coke Life, which has 89 calories per 330ml can, marks the first new **fizzy drinks** launch in the UK by the global brand for eight years.

Following successful trials last year in South America, it will now be made here in factories at Sidcup, Kent, Edmonton, north east London, Wakefield, Yorkshire and East Kilbride, Scotland.

But health experts said Coke would have made more impact by slashing sugar in its classic brand.

Pressure group Action on Sugar has been calling for a dramatic reduction in sugar in our diets so it makes up no more than 5% of our daily energy intake in a bid to combat obesity.

And campaign director and nutritionist Katharine Jenner said: Coke Life is a step in the right direction but we would much rather have seen Coca Cola reduce sugar in its main line as that would have had much more effect on people's sugar intake.

"A small sugar reduction instead of a new brand would have allowed consumers' taste buds to adjust and as the big seller, could have seriously reduced the nation's sugar intake."

Health experts have called for a tax on **sugary drinks** as well as health warnings on tins and bottles to shock families into cutting down on pop.

Reports suggest Brits consume three times more sugar than the World Health Organisation recommends with a daily intake of 15% instead of 5%.

Coke said its new drink would give Brits greater choice and added 40% of its range already contained no calories.

James Quincey. president Coca Cola Europe added: "With Coca Cola Life, we have innovated to provide consumers with a new option with fewer calories.

"We were early signatories to the UK Government's Responsibility Deal and as we work with others across society to address the public health challenge of obesity in the UK and across Europe, we will continue to take actions that help people balance their lifestyles."

LOAD-DATE: September 4, 2014

LANGUAGE: ENGLISH

PUBLICATION-TYPE: Newspaper; Web Publication

JOURNAL-CODE: WEBDMI

Copyright 2014 Trinity Mirror, Plc.
All Rights Reserved

MailOnline

June 9, 2014 Monday 4:47 PM GMT

It takes just 23 minutes to burn off a can of Coke, Coca-Cola advert claims

BYLINE: ASSOCIATED PRESS

SECTION: AP

LENGTH: 635 words

It takes just 23 minutes to burn off the 140 calories in a can of Coke, Coca-Cola claims.

In a bid to help tackle obesity, the company has posted an online video suggesting fun ways to burn the calories.

In the advert, the world's biggest beverage maker asks what would happen if people paid for a can of Coke by first working off the calories it contained.

The advert, which notes that it typically takes 23 minutes of cycling to achieve that, shows a montage of people on a giant stationary bicycle happily trying to earn a can of its cola, with carnival music playing in the background.

The video is unusual because it so frankly addresses how many calories are in its drink. But it also takes a frequent criticism used by health advocates and spins it in a happy light.

"It's so clever on so many levels, but it's twisted too," said Michele Simon, a public health lawyer and author of "Appetite for Profit: How the Food Industry Undermines Our Health and How to Fight Back."

Simon said she thought the video was a response to the recently released "Fed Up" movie, which is critical of the food industry's marketing tactics. In the movie, a health advocate states that a child would have to bike for an hour and 15 minutes to burn off the calories in a 20-ounce Coke.

Coca-Cola's video comes as **soft drinks** have faced growing criticism from health advocates who say they fuel obesity and chronic diseases related to diet. Numerous cities have tried to impose special taxes on **sugary drinks**, although none have succeeded, in large part because of heavy lobbying from the beverage industry.

In New York City, the Board of Health this week asked the state's highest court to reinstate a 16-ounce limit on **sugary drinks** sold in restaurants, stadiums and other venues. The measure, championed by former mayor Michael Bloomberg, was knocked down by a judge after a lawsuit led by the beverage industry.

Coca-Cola, based in Atlanta, began addressing obesity for the first time in a TV ad last year. That ad took a far more serious tone, with a voiceover stating that weight gain is the result of consuming too many calories of any kind, not just soda.

It's an argument frequently used by food companies, which tend to stress the need for physical activity and moderation when addressing criticism about the nutritional content of their products. But health advocates say that glosses over the reality that many people are simply consuming too many calories and that it would

be unrealistic for them to try and offset that with exercise, especially given people's increasingly sedentary lifestyles.

In the meantime, the soda industry is fighting to stop declining sales even without the impact of any special taxes or measures. Just last year, U.S. sales volume declined 3 percent, faster than the 1.2 percent drop the previous year, according to industry tracker Beverage Digest. And critics are increasingly taking aim at other **sugary beverages** sold by Coca-Cola and PepsiCo, such as sports drinks and juices.

Judith Snyder, a Coca-Cola spokeswoman, said the latest video is part of a series that show "moments of delight and surprise" with Coke. She said it's intended to address the theme of energy balance in a lighthearted way.

She said it will be promoted on Facebook and Twitter, but won't run on TV.

At the end of the video, which runs for 1 minute and 40 seconds, the phrase "Movement is happiness" appears on the screen, followed by: "Where will happiness strike next?"

Laura Ries, president of the brand consulting firm Ries & Ries, said that the video could backfire because people might be turned off by the idea that they would need to cycle for 23 minutes just to burn off a Coke.

"They're showing exactly why you wouldn't want to drink a Coke. Twenty-three minutes on a bike is not fun for most people," she said.

LOAD-DATE: June 10, 2014

LANGUAGE: ENGLISH

PUBLICATION-TYPE: Web Publication

JOURNAL-CODE: WEBDM

Copyright 2014 Associated Newspapers Ltd.
All Rights Reserved

MailOnline

MailOnline

June 9, 2014 Monday 1:25 AM GMT

Greens back **sugary drinks** tax

BYLINE: AUSTRALIAN ASSOCIATED PRESS

SECTION: AAP

LENGTH: 276 words

The Greens say they're committed to implementing a tax on **sugary drinks** if they're part of the next government.

The recommendation comes from the New Zealand Medical Association (NZMA), which says obesity may become New Zealand's biggest public health threat over the next decade.

It's calling on the government to adopt 10 changes, including a tax on **sugary drinks**, nutrition guidelines in school canteens and hospitals and teaching kids about nutrition as part of the curriculum.

The NZMA report, released on Monday, says obesity is imposing a huge burden on the health system.

Green Party health spokesman Kevin Hague says the report is ground-breaking and all its recommendations must be implemented.

"Two-thirds of New Zealanders are now obese or overweight and this is a life and death issue that is overwhelming our hospitals," he said.

"The government must accept that doctors are the experts and take their advice on tackling obesity."

Mr Hague says controls on price, availability and marketing have worked to reduce smoking "so why wouldn't we use everything we've got?"

New Zealand is now the fourth most obese country in the world, with 34 per cent of adults overweight and 31 per cent obese.

NZMA chair Mark Peterson says obesity and its related complications could tie up many of the resources of the public health system over the next 10 years.

Existing efforts to reverse the statistics don't appear to be working and international studies show taxation on unhealthy foods is the single most cost-effective approach to tackling obesity, the report says.

It also recommends a food labelling system - possibly the traffic-light system - on food packaging.

LOAD-DATE: June 9, 2014

LANGUAGE: ENGLISH

PUBLICATION-TYPE: Web Publication

JOURNAL-CODE: WEBDM

Copyright 2014 Associated Newspapers Ltd.
All Rights Reserved

Mail Online

MailOnline

June 8, 2014 Sunday 8:52 PM GMT

Doctors back soft drink tax

BYLINE: AUSTRALIAN ASSOCIATED PRESS

SECTION: AAP

LENGTH: 309 words

A tax on **sugary drinks** could be used to help address obesity, which doctors fear may be our biggest public health threat over the next decade.

A report released by the New Zealand Medical Association on Monday says obesity rates keep rising and is creating a huge financial burden to our healthcare system.

New Zealand is now the fourth most obese country in the world, with 34 per cent of adults overweight, and 31 per cent obese.

The association's chair Mark Peterson says obesity and its related complications could tie up many of the resources of the public health system over the next 10 years.

But existing efforts to reverse the statistics don't appear to be working, the report says.

It calls on the government to adopt its 10 policy change recommendations, including encouraging people to cut their sweet intake by taxing **sugar**-sweetened **soft drinks**.

The report says international studies shows taxation on unhealthy foods is the single most cost-effective approach to tackling obesity.

It also recommends a food labelling system - possibly the traffic-light system - on food packaging, nutrition guidelines in school canteens and hospitals, and teaching kids about nutrition as part of the curriculum.

"There are few better examples of the old adage `knowledge is power' than when it comes to front-of-pack food labelling," says the report.

Obesity is also an equity issue, says Dr Peterson.

"Children living in the most depressed areas (are) three times more likely to be obese than children who live in the least deprived areas."

And the report says everyone's responsible for tackling obesity - not just doctors.

"Obesity is not the fault of any one sector, organisation or individual," the report says.

"Given the complexity of the obesity epidemic, successfully halting and reversing it is likely to require many sustained interventions at several levels."

LOAD-DATE: June 8, 2014

LANGUAGE: ENGLISH

PUBLICATION-TYPE: Web Publication

JOURNAL-CODE: WEBDM

Copyright 2014 Associated Newspapers Ltd.
All Rights Reserved

The Sunday Telegraph

The Sunday Telegraph (London)

June 8, 2014
Edition 1;
National Edition

Ban sugary food ads for health and wealth

SECTION: LETTERS; Pg. 25

LENGTH: 149 words

SIR - It was depressingly predictable that a voluntary agreement has led to food manufacturers doing very little to reduce the sugar content in their best-selling items ("Food firms sidestep pledge on sugar", report, June 1). We need the Government to take firm action.

There is no point in only blaming individuals for their unhealthy diets, when food companies spend billions of pounds trying to persuade us to eat and drink things that are bad for us. And with diabetes alone costing the NHS £10 billion a year, sugar-related diseases affect us all.

People have the right to make their own choices, but why do we allow firms to pressure us into making the wrong ones? For the sake of our physical health and the nation's financial health, I believe that it is now time to ban the advertising of **sugary drinks,** processed food, takeaways, alcohol and confectionery. Richard Mountford Tonbridge, Kent

LOAD-DATE: June 8, 2014

LANGUAGE: ENGLISH

PUBLICATION-TYPE: Newspaper

JOURNAL-CODE: STL

Copyright 2014 Telegraph Media Group Limited
All Rights Reserved

Sunday Express

June 8, 2014
Edition 3;
National Edition

Call for fizzy drinks sugar tax in fight to stem obesity

BYLINE: Jon Coates

SECTION: NEWS; Pg. 10

LENGTH: 267 words

A SUGAR TAX on **fizzy drinks** has moved a step closer as health chiefs battle to stem the obesity epidemic plaguing the nation.

With a third of all children and two-thirds of adults now classed as overweight or obese, there are growing fears that tough measures will be the only way to reduce consumption of highly addictive sugar.

Leading doctors have called for **sugary drinks** to be labelled with health warnings to make them as socially unacceptable as cigarettes.

Studies have shown all age groups, especially teenagers, are consuming too much sugar, putting them at risk of diabetes, heart disease, cancer and tooth decay, as well as bumping up health care costs.

health care costs.

A tax on **fizzy drinks** packed with sugar is n a real possibility following two high-level meetings last week hosted by Public Health England.

A proposed sugar reduction strategy will be unveiled by PHE on June 26. A spokeswoman said a tax "will be considered alongside other measures that have the potential to be effective".

Cardiologist Dr Aseem Malhotra said: "For too long we have allowed an unregulated food industry to peddle nutritionally poor, high calorie products with impunity. Inaction is no longer an option."

Last night Prof Dame Sally Davies, the Chief Medical Officer for England, warned that spiralling obesity means as many as one in 10 deaths are now caused by excess weight.

She said many people did not realise they weigh too much because of the "normalisation" of extra weight.

Research by the University of Cambridge suggests up to 53,000 deaths in England and Wales will be attributable to obesity next year.

LOAD-DATE: June 8, 2014

LANGUAGE: ENGLISH

PUBLICATION-TYPE: Newspaper

JOURNAL-CODE: SXP

Copyright 2014 Express Newspapers

All Rights Reserv

The Sun (England)

June 7, 2014 Saturday
Edition 1;
Scotland

SUGAR GOAL

SECTION: FEATURES; Pg. 41

LENGTH: 100 words

WE should consume no more than 25 grams of sugar a day - about the same as a standard Dairy Milk bar and ten grams less than in a can of cola.

Sugary drinks have been around for some time and I love this Lemonade recipe from Eliza Acton's Modern Cookery, first published in 1845.

"Rasp (thinly grate) rind of a juicy lemon with 4oz of sugar and pour on the strained juice. Press mixture into a jar and when wanted dissolve a tablespoon of it in a glass of water. It will keep for a considerable time."

If my calculations are correct the average can of cola contains more than three tablespoons of sugar.

LOAD-DATE: June 7, 2014

LANGUAGE: ENGLISH

PUBLICATION-TYPE: Newspaper

JOURNAL-CODE: SUNscot

Copyright 2014 NEWS GROUP NEWSPAPERS LTD
All Rights Reserved

ed

MailOnline

June 6, 2014 Friday 4:59 PM GMT

Coke ad: It takes 23 minutes to burn off a soda

BYLINE: ASSOCIATED PRESS

SECTION: AP

LENGTH: 635 words

NEW YORK (AP) - Coca-Cola is taking on obesity, this time with an online video showing how fun it could be to burn off the 140 calories in a can of its soda.

In the ad, the world's biggest beverage maker asks what would happen if people paid for a can of Coke by first working off the calories it contained. The ad, which notes that it typically takes 23 minutes of cycling to achieve that, shows a montage of people on a giant stationary bicycle happily trying to earn a can of its cola, with carnival music playing in the background.

The video is unusual because it so frankly addresses how many calories are in its drink. But it also takes a frequent criticism used by health advocates and spins it in a happy light.

"It's so clever on so many levels, but it's twisted too," said Michele Simon, a public health lawyer and author of "Appetite for Profit: How the Food Industry Undermines Our Health and How to Fight Back."

Simon said she thought the video was a response to the recently released "Fed Up" movie, which is critical of the food industry's marketing tactics. In the movie, a health advocate states that a child would have to bike for an hour and 15 minutes to burn off the calories in a 20-ounce Coke.

Coca-Cola's video comes as **soft drinks** have faced growing criticism from health advocates who say they fuel obesity and chronic diseases related to diet. Numerous cities have tried to impose special taxes on **sugary drinks**, although none have succeeded, in large part because of heavy lobbying from the beverage industry.

In New York City, the Board of Health this week asked the state's highest court to reinstate a 16-ounce limit on **sugary drinks** sold in restaurants, stadiums and other venues. The measure, championed by former mayor Michael Bloomberg, was knocked down by a judge after a lawsuit led by the beverage industry.

Coca-Cola, based in Atlanta, began addressing obesity for the first time in a TV ad last year. That ad took a far more serious tone, with a voiceover stating that weight gain is the result of consuming too many calories of any kind, not just soda.

It's an argument frequently used by food companies, which tend to stress the need for physical activity and moderation when addressing criticism about the nutritional content of their products. But health advocates say that glosses over the reality that many people are simply consuming too many calories and that it would be unrealistic for them to try and offset that with exercise, especially given people's increasingly sedentary lifestyles.

In the meantime, the soda industry is fighting to stop declining sales even without the impact of any special taxes or measures. Just last year, U.S. sales volume declined 3 percent, faster than the 1.2 percent drop the previous year, according to industry tracker Beverage Digest. And critics are increasingly taking aim at other **sugary beverages** sold by Coca-Cola and PepsiCo, such as sports drinks and juices.

Judith Snyder, a Coca-Cola spokeswoman, said the latest video is part of a series that show "moments of delight and surprise" with Coke. She said it's intended to address the theme of energy balance in a lighthearted way.

She said it will be promoted on Facebook and Twitter, but won't run on TV.

At the end of the video, which runs for 1 minute and 40 seconds, the phrase "Movement is happiness" appears on the screen, followed by: "Where will happiness strike next?"

Laura Ries, president of the brand consulting firm Ries & Ries, said that the video could backfire because people might be turned off by the idea that they would need to cycle for 23 minutes just to burn off a Coke.

"They're showing exactly why you wouldn't want to drink a Coke. Twenty-three minutes on a bike is not fun for most people," she said.

____

Follow Candice Choi at www.twitter.com/candicechoi

LOAD-DATE: June 6, 2014

LANGUAGE: ENGLISH

PUBLICATION-TYPE: Web Publication

JOURNAL-CODE: WEBDM

Copyright 2014 Associated Newspapers Ltd.
All Rights Reserved

The Daily Telegraph

The Daily Telegraph (London)

June 5, 2014 Thursday
Edition 2;
National Edition

The 'hidden risk' sugar in fizzy drinks and juice

SECTION: NEWS; Pg. 14

LENGTH: 153 words

Fruit juices and **fizzy drinks** such as Coca-Cola contain high levels of a harmful sugar known as fructose, but this is not always clear on the labels, researchers have said.

Analysis of 34 drinks including Coca-Cola, Pepsi, Dr Pepper, Sprite, Minute Maid and Juicy Juice showed they contained high fructose levels.

The University of Southern California study, published in the journal Nutrition, found many popular drinks have a sugar content of 60 per cent fructose and 40 per cent glucose. Fructose is mostly broken down into fat in the liver, which is linked to a number of diseases, including heart disease and stroke, while glucose is largely burnt by the body for energy.

Dr Michael Goran, lead author of the study, said: "The human body isn't designed to process this form of sugar at such high levels."

Many products have labels saying they are made with sugar but do not state which type, the researchers said.

LOAD-DATE: June 5, 2014

LANGUAGE: ENGLISH

PUBLICATION-TYPE: Newspaper

JOURNAL-CODE: DTL

Copyright 2014 Telegraph Media Group Limited
All Rights Reserved

MailOnline

MailOnline

June 5, 2014 Thursday 5:04 PM GMT

Fizzy drinks 'loaded with harmful sugars could increase the risk of developing diabetes, cardiovascular and liver disease'

BYLINE: EMMA INNES

SECTION: HEALTH

LENGTH: 538 words

- The drinks, including Coca Cola, contain more fructose than the labels say
- So, they could contribute to the development of diabetes and heart disease
- This is because fructose, unlike glucose, is processed by the liver and turned into fat - glucose is converted into energy so is less harmful

Fizzy drinks contain far more harmful sugars than the labels say, a new study has today warned.

The drinks, including Coca Cola, Sprite, Dr Pepper and Mountain Dew, contain more fructose than people realise, the research revealed.

They contain as much as 50 per cent more fructose than glucose, researchers say.

The U.S. team behind the study say the human body was not designed to process this combination of sugars.

As a result, they say the drinks can contribute to the development of diabetes, cardiovascular disease and liver disease.

This is because unlike glucose, that is converted into energy, fructose is processed by the liver into fat.

The study, by the Childhood Obesity Research Centre at Keck School of Medicine, University of Southern California, examined the ratio of fructose to glucose in 34 popular brands by analysing their chemical composition.

The research, published online in the journal Nutrition, found that beverages and juices made with high fructose corn syrup (HFCS) all contain 50 per cent more fructose than glucose.

Dr Michael Goran said the blend called into question claims that sugar and HFCS are essentially the same.

He said: 'We found what ends up being consumed in these **beverages** is neither natural **sugar** nor HFCS, but instead a fructose-intense concoction that could increase one's risk for diabetes, cardiovascular disease and liver disease.

'The human body isn't designed to process this form of sugar at such high levels. Unlike glucose, which serves as fuel for the body, fructose is processed almost entirely in the liver where it is converted to fat.'

The trade body responsible for HFCS manufacturers, the Corn Refiners Association, argued HFCS is only negligibly different than natural sugar, sucrose, which is made up of equal parts of fructose and glucose.

However Dr Goran's analysis of beverages made with HFCS showed a fructose to glucose ratio of 60:40 - considerably higher than the equal proportions found in sucrose and challenging the industry's claim that 'sugar is sugar'.

And he argued labelling was inadequate to warn consumers of the fructose content.

The research team purchased beverages based on product popularity and had them analysed for sugar composition in three different laboratories using three different methods.

The results were consistent across the different methods and yielded an average sugar composition of 60 per cent fructose and 40 per cent glucose in beverages made with HFCS.

Dr Goran pointed out Americans consume more HFCS per capita than any other nation and consumption has doubled over the last three decades.

Diabetes rates have tripled in the same period with much of this increase directly linked to sodas, sports drinks and energy drinks.

He said: 'Given that Americans drink 45 gallons of soda a year, it's important for us to have a more accurate understanding of what we're actually drinking, including specific label information on the types of sugars.'

LOAD-DATE: June 5, 2014

LANGUAGE: ENGLISH

PUBLICATION-TYPE: Web Publication

JOURNAL-CODE: WEBDM

Copyright 2014 Associated Newspapers Ltd.
All Rights Reserved
Guardian.com.

June 4, 2014 Wednesday

New evidence on fructose in Coca Cola, Pepsi and other sodas in the US

BYLINE: Sarah Boseleytheguardian.com

LENGTH: 534 words

ABSTRACT

A new analysis from the University of Southern California finds that sodas in the US are potentially a bigger risk for obesity than assumed, because of the high fructose levels they contain

FULL TEXT

The battle over high fructose corn syrup (HFCS) in sodas in the US is likely to intensify following the publication today of a new study from the Childhood Obesity Research Center at the Keck School of Medicine of the University of Southern California. A team led by Michael Goran, its director, has found that hugely popular soft drinks sweetened with HFCS, including Coca Cola, Pepsi, Dr Pepper, Mountain Dew and Sprite, contain more fructose than most people know.

Goran's paper, published in the journal Nutrition, shows that the drinks - as sold on the streets - contain 50% more fructose than glucose. The ratio, it says, is 60:40 and not 50:50 as it is in regular sugar.

Why does this matter? Because, says Goran, other scientific studies have suggested that fructose is part of the cause - and if you read Robert Lustig's book, Fat Chance; the bitter truth about sugar, you may think it is the main cause - of the obesity epidemic.

This is not the first time that Goran's team has analysed the fructose levels in US sodas. They published an earlier paper in the journal Obesity in 2010, which was "completely trashed", he said, by the corn refining industry, which makes HFCS. The Corn Refiners Association maintains that HFCS has essentially the same fructose/glucose proportions as sugar. It also says that fructose does not have a different effect on the body from glucose.

This time, Goran commissioned a more extensive analysis of 34 popular **soft drinks**, using three different techniques in three separate laboratories. The findings were essentially the same. On average, the **sugar** composition in the drinks was 60% fructose and 40% glucose.

Goran told me that this is cause for concern.

Other studies have shown - and this is relatively new science that is emerging - that fructose is much more harmful than glucose. Glucose is used for energy in the body. Fructose is taken up much more readily by the liver, where it is used as fat. That conversion causes a lot of metabolic problems. We now know that fructose has more negative effects. The $6 billion a year corn refining industry repeats the mantra that sugar is sugar.

Why did the corn refining industry not do its own studies to prove Goran wrong? Apparently they at least thought about it. Internal documents revealed during legal proceedings between the sugar industry and the corn refiners and published by the New York Times suggest re-running "the USC study". This is from an internal memo on page 11:

If the results contradict USC, we can publish them...If for any reason the results confirm USC, we can just bury the data.

Goran also takes issue with the labelling of sodas, which sometimes contain higher levels of fructose than specified. Sierra Mist, Gatorade and Mexican Coca-Cola had higher concentrations of fructose in the analyses than the labels suggested, which he says may mean they contain HFCS.

There are, of course, high fructose levels in fruit juices as well as sodas. We should eat fruit - not drink it, says Goran.

LOAD-DATE: June 4, 2014

LANGUAGE: ENGLISH

PUBLICATION-TYPE: Newspaper

Copyright 2014 Guardian News and Media Limited or its affiliated companies
All Rights Reserved
Registered in England and Wales. No. 908396
Registered office: PO Box 68164, Kings Place, 90 York Way, London N1P 2AP

DAILY MAIL (London)

June 2, 2014 Monday

COCA-COLA AND NESTLE DODGE VOW TO CUT SUGAR FROM THEIR TOP BRANDS

BYLINE: BY BEN SPENCER SCIENCE REPORTER

LENGTH: 540 words

FOOD giants that promised to make products healthier have simply side-stepped their pledges.

Some of the world's biggest firms failed to cut the calories or sugar in their most popular brands - despite signing up to the Government's much trumpeted responsibility deal'.

They include Coca-Cola, Magnum ice cream and Kit Kat chocolate bars.

The Public Health Responsibility Deal, introduced in 2011, has been accused of giving the food and drink industries carte blanche to regulate themselves.

As partners' in the scheme, companies have made a variety of pledges covering marketing, labelling and workplace practices.

Thirty seven of them, including Coca-Cola, Mars, Nestle and PepsiCo, signed up to a calorie reduction' pledge. It included supporting and enabling' customers to eat and drink fewer calories, for example by altering the products, reducing portion sizes or offering healthier options. But it has emerged that some of the firms have not touched their main brands - instead focusing on reducing sugar in lesser-known products or altering labelling.

Campaigners called on ministers to abandon the voluntary system and instead introduce sugar reduction targets similar to those in place for salt.

Coca-Cola has reduced the calories in its Sprite drink - but refused to change its flagship product. Unilever, which makes Wall's and Ben & Jerry's, has altered its children's ice creams but not its normal range. Nestle has cut the calories in Aero and white Kit Kat Chunky bars by 5 per cent but not changed ordinary Kit Kats.

Keith Vaz, chairman of the Commons home affairs select committee, said: The problem with the Responsibility Deal is that it is all voluntary.

As far as Coke is concerned, it remains the same product it was ten years ago - there doesn't seem to have been any change at all.'

He told the Sunday Telegraph: We need targets. Unless we get the target we have no benchmark.'

Health experts have become increasingly concerned at the nation's intake of sugar.

Simon Capewell, professor of clinical epidemiology at Liverpool University, said in January: Sugar is the new tobacco. Everywhere, **sugary drinks** and junk foods are pressed on unsuspecting parents and children by a cynical industry focused on profit not health.' To meet the calorie pledge, Coca-Cola has cut the sugar and calories in Glaceau vitamin water and Sprite by 30 per cent. But a 330ml can of Coca-Cola still contains 35g of sugar - equivalent to nine teaspoons.

A spokesman said: We have no plans to change Coca-Cola. We know that people love it and we provide two great-tasting sugar-free, no calorie options in Diet Coke and Coca-Cola Zero.'

Unilever pledged to cut the number of calories in children's ice creams, which now contain 110 or fewer per serving. But ordinary Magnum bars and Ben & Jerry's ice cream tubs have not been changed. A spokesman said: Sugar plays an important role in ice cream, not only for taste but also for texture and structure.'

The Department of Health said: Many manufacturers and retailers are already taking sugar, fat and salt out of their products as a result of our Responsibility Deal, which is based on working collaboratively with industry rather than imposing unrealistic targets.' Daily Mail, January 9

© Daily Mail

LOAD-DATE: June 1, 2014

LANGUAGE: ENGLISH

PUBLICATION-TYPE: Papers

Copyright 2014 Associated Newspapers Ltd.

All Rights Reserved

MailOnline

June 1, 2014 Sunday 9:14 PM GMT

The bitter truth about pledges to cut sugar: Food giants including Coca-Cola and Nestle accused of side-stepping promises to make products less fattening

BYLINE: BEN SPENCER

SECTION: SCIENCE

LENGTH: 814 words

- Producers pledged to reduce sugar following a 2011 deal with Government
- However, some of the world's biggest food producers have failed to act
- 37 firm signed the 2011 pledge including Coca Cola, Mars, Nestle and PepsiCo
- Coca Cola has reduced sugar in Sprite but refused to change the recipe of Coke
- A 330ml can of Coke contains 35g or nine teaspoons of sugar

Food giants which promised to make products healthier under the Government's trumpeted 'responsibility deal' have simply side-stepped their pledges, it has emerged.

Some of the world's biggest firms have failed to reduce calories or sugar content in their most popular brands - including Coca Cola, Magnum ice cream and Kit Kat chocolate.

The Public Health Responsibility Deal, introduced by the Government in 2011, has been much criticised by health campaigners, who say it gives the food and drink industries carte blanche to regulate themselves.

Some 663 organisations are 'partners' in the responsibility deal, under which they have made variety of pledges covering marketing, labelling and workplace practices.

Thirty seven firms, including Coca Cola, Mars, Nestlé and PepsiCo, signed up to a 'calorie reduction' pledge.

Their promise included 'supporting and enabling' customers to eat and drink fewer calories through actions such as product/menu reformulation, reviewing portion sizes, education and information, and actions to shift the marketing mix towards lower calorie options'.

But it has emerged that some of the companies have not touched their main brands - instead focusing on reduced sugar in lesser-known products or altering labelling.

Campaigners called on ministers to abandon the voluntary system and instead introduce sugar reduction targets similar to those in place for salt.

Coca Cola has reduced the calories in its Sprite drink - but has refused to change its flagship product.

Unilever, which makes Wall's and Ben & Jerry's ice creams has reduced the number of calories in children's ice creams but not in its normal range.

Nestlé has cut the calories in Aero and white Kit Kat Chunky bars but not in ordinary milk chocolate Kit Kats.

Keith Vaz MP, chairman of the Commons home affairs select committee, told the Sunday Telegraph: 'The problem with the Responsibility Deal is that it is all voluntary.

'As far as Coke is concerned it remains the same product it was 10 years ago - there doesn't seem to have been any change at all.

'We need targets. Unless we get the target we have no benchmark.'

Health experts have become increasingly concerned at the population's intake of sugar - comparing it to tobacco in terms of its public health impact.

Simon Capewell, professor of clinical epidemiology at the University of Liverpool, said in January: 'Sugar is the new tobacco.

'Everywhere, **sugary drinks** and junk foods are now pressed on unsuspecting parents and children by a cynical industry focused on profit not health.

'Obesity and diabetes already cost the UK over £5billion a year. Without regulation, these costs will exceed £50billion by 2050.'

As part of its commitment to the calorie pledge, Coca Cola has cut the amount of sugar and calories in Glacéau vitaminwater and Sprite by 30 per cent.

But a 330ml can of Coca Cola still contains 35g - or nine teaspoons - of sugar.

A spokesman for Coca Cola GB said: 'We have no plans to change Coca Cola. We know that people love it and we provide two great-tasting sugar-free, no calorie options in Diet Coke and Coca-Cola Zero, which together comprise more than 40 per cent of the cola we sell in the UK.'

Nestlé has reduced the number of calories in Aero and white KitKat Chunky bars by 5 per cent.

However, it has not reduced the number of calories in milk chocolate KitKat bars and other popular products such as Lions and Rolos.

Unilever, which owns Wall's and Ben & Jerry's, pledged to cut the number of calories in children's ice creams, which now contain 110 or fewer calories per serving.

It is also offering 'smaller and lower calorie alternative choices' such as mini Magnums. But ordinary Magnum bars and ice cream tubs from Ben & Jerry's have not been changed.

A Unilever spokesman said: 'Sugar plays an important role in ice cream, not only for taste but also for texture and structure. However, we have a programme in place that aims to gradually reduce sugar in our products whilst maintaining our high standards of product quality.'

A Department of Health spokesman said: 'Many manufacturers and retailers are already taking sugar, fat and salt out of their products as a result of our Responsibility Deal, which is based on working collaboratively with industry rather than imposing unrealistic targets.

'The Scientific Advisory Committee on Nutrition is currently undertaking a review of carbohydrates, and they are looking at sugar as part of this. Their draft report which is due to be published later this year will inform our future thinking.'

LOAD-DATE: June 1, 2014

LANGUAGE: ENGLISH

PUBLICATION-TYPE: Web Publication

JOURNAL-CODE: WEBDM

Copyright 2014 Associated Newspapers Ltd.
All Rights Reserved

DAILY MAIL (London)

July 30, 2014 Wednesday

SUGAR FEARS TAKE FIZZ OUT OF DRINK SALES

BYLINE: BY NO BYLINE AVAILABLE

LENGTH: 221 words

CONSUMERS are cutting down on their **fizzy drink** intake because of fears that the high sugar content is damaging their health.

Research shows that one in four people consumes fewer **fizzy drinks** than six months ago - rising to 34 per cent among those aged 16-24 - with half saying it's because they contain too much sugar.

Earlier this year, campaigners claimed sugar was the new tobacco', with hidden quantities in everyday foods contributing to a huge burden of disease and death'.

Market analysts Mintel said consumption of carbonated soft drinks is this year expected to fall to its lowest point since 2010

.

Britons consumed 5.96billion litres in 2010

, rising to 6.17billion litres in 2011, but this is expected to dip to just 5.95billion litres in 2014.

Sales are expected to reach £7.5billion this year, down almost £1billion since 2011

Katharine Jenner, campaign director for Action on Sugar, said: If drink-makers want to stay in business they need to listen to what their customers want - less sugar, now.'

The slump comes as a study suggested that too many **sugary drinks** can harm the memory of teenagers. Researchers from the University of Southern California found that, after feeding the drinks to rats for a month, adolescent rats performed worse on tests of learning and memory. There was no change in adult rats.

© Daily Mail

LOAD-DATE: July 30, 2014

LANGUAGE: ENGLISH

PUBLICATION-TYPE: Papers

Copyright 2014 Associated Newspapers Ltd.
All Rights Reserved

Daily Mirror

July 30, 2014 Wednesday
Edition 1;
Northern Ireland

Sugar alerts hit pop sales

SECTION: NEWS; Pg. 14

LENGTH: 82 words

SALES of **fizzy drinks** are falling after health warnings about sugar intake and soaring obesity levels.

Analyst Mintel says one in four people, and one in three 16- to 24-year-olds, have cut intake in the past six months. Half said they had done so because of high sugar levels.

Consumption is set to fall from 6.2 billion litres in 2011 to six billion litres this year, which Mintel said would knock £800million off sales.

In January a Liverpool University professor branded sugar "the new tobacco".

LOAD-DATE: July 30, 2014

LANGUAGE: ENGLISH

GRAPHIC: WARNINGS **Fizzy drinks**

PUBLICATION-TYPE: Newspaper

JOURNAL-CODE: DMR

Copyright 2014 MGN Ltd.
All Rights Reserved18 of 205 DOCUMENTS

The Sun (England)

July 30, 2014 Wednesday
Edition 1;
Northern Ireland

Fizz kids go fuzzy

SECTION: NEWS; Pg. 24

LENGTH: 59 words

FIZZY drinks can impair teenagers' memory skills, a study reveals.

Just one can of pop a day may affect youngsters' ability to learn. Tests on rats showed adults were unaffected by sweetened drinks. But adolescent rodents performed poorly in tests. Scientists in California, US, said the drinks made the brain fuzzy "when consumed in excess before adulthood".

LOAD-DATE: July 30, 2014

LANGUAGE: ENGLISH

PUBLICATION-TYPE: Newspaper

JOURNAL-CODE: SUN

Copyright 2014 NEWS GROUP NEWSPAPERS LTD
All Rights Reserved

THE TIMES

The Times (London)

July 30, 2014 Wednesday
Edition 1;
National Edition

Sugar fears cause drop in fizzy pop sales

BYLINE: Rosemary Bennett; Oliver Moody

SECTION: NEWS; Pg. 19

LENGTH: 330 words

One in four consumers is buying fewer fizzy **soft drinks** than six months ago, largely because of recent health warnings about **sugar**.

Overall, a quarter of adults and a third of 16 to 24-year-olds have cut down, the study by Mintel, the market analysts, found.

As a result, consumption of **fizzy drinks** has fallen to its lowest point for six years at 5.95 billion litres annually.

Richard Ford, senior food and drink analyst at Mintel, said health warnings appeared to be hitting home and the industry needed to raise its game. "The findings of our research come as the debate over **sugar's** contribution towards the nation's growing obesity continues to be played out in the media, with carbonated **soft drinks** being highlighted as one area for improvement," he said.

Manufacturers are coming up with lower-**sugar and sugar**-free variants of their standard **soft drinks** to try and match demand for healthier options, he said, such as Coca-Cola Life, which contains a blend of **sugar** and the sweetener stevia leaf extract.

Mr Ford said: "While the industry has been proactive in tackling concerns around the high sugar content of some of the drinks by introducing lowersugar or lower-calorie variants, more work is needed."

Soft drinks are the latest target of health campaigners who have warned that one can of a **sugary soft drink** contains an adult's entire daily **sugar** allowance, under new draft guidelines.

The latest study, published in the US yesterday, warned that teenagers who consume **sugary drinks** daily might risk severely impairing their memories. Researchers from the University of Southern California found that giving adolescent rats **soft drinks** every day for a month dramatically cut their performance in learning and memory tests. In the UK some experts have called for a **sugar** tax to try and discourage consumption. Dame Sally Davies, the chief medical officer, has said research is likely to prove **sugar** is an addictive substance, which might make some form of **sugar** tax necessary.

LOAD-DATE: July 30, 2014

LANGUAGE: ENGLISH

PUBLICATION-TYPE: Newspaper

JOURNAL-CODE: TIM

Copyright 2014 Times Newspapers Limited
All Rights Reserved

MailOnline

MailOnline

July 29, 2014 Tuesday 10:00 PM GMT

Just one **fizzy drink** a day 'can damage teenagers' memory'

BYLINE: LIZZIE PARRY

SECTION: HEALTH

LENGTH: 443 words

- Study at University of Southern California found sugar-laden drinks can affect young people's ability to learn
- Experiments found brain activity and aptitude to remember information was affected by one **fizzy drink** a day

Just one **fizzy drink** a day can damage teenagers' memory, a new study has found.

Researchers at the University of Southern California found the sugar-laden drinks can affect people's ability to learn.

As well as causing obesity, the drinks have also been blamed for making youngsters more violent.

Now, experiments have found their impair the brain's activity, and aptitude for remembering information.

Dr Scott Kanoski, of the University of Southern California, said: 'It is no secret refined carbohydrates, particularly when consumed in soft drinks and other beverages, can lead to metabolic disturbances.

'However, our findings reveal that consuming sugar-sweetened drinks is also interfering with our brain's ability to function normally and remember critical information about our environment, at least when consumed in excess before adulthood.'

In the study, both adult and adolescent rats were given daily access to sweetened beverages that mirrored high fructose corn syrup or sucrose **sugar** concentrations found in common **soft drinks**.

The adults performed normally in tests of cognitive function after a month.

But when consumption occurred during adolescence, the rats performed poorly in tests of learning and memory capability.

In addition to causing memory impairment, adolescent **sugar** sweetened **beverage** consumption also produced inflammation in the hippocampus, an area of the brain that controls many learning and memory functions.

Dr Kanoski, who presented his findings at the annual meeting of the Society for the Study of Ingestive Behaviour in Seattle, added: 'The hippocampus is such a critical brain region for memory function.

'In many ways this region is a canary in the coal mine, as it is particularly sensitive to insult by various environmental factors, including eating foods that are high in saturated fat and processed sugar.'

There is no recommended number of soft and **fizzy drinks** that children can have.

But the NHS advises, as part of a healthy balanced diet, it is important they do not have too many, especially between meals.

The National Diet and Nutrition Survey found **soft drinks** were the largest contributor to **sugar** intake for children aged four to 18 years, with four to 10-year-olds getting 16 per cent of their's from them, with the figure rising to 29 per cent for older children.

In the U.S., research has revealed children and teenagers drink three times more soft drinks than their parents did 20 years ago.

LOAD-DATE: July 29, 2014

LANGUAGE: ENGLISH

PUBLICATION-TYPE: Web Publication

JOURNAL-CODE: WEBDM

Copyright 2014 Associated Newspapers Ltd.
All Rights Reserved

The Daily Telegraph
The Daily Telegraph (London)

July 26, 2014 Saturday
Edition 2;
National Edition

Ban supersize food and drinks to tackle obesity, says Tory health chief

BYLINE: Laura Donnelly

SECTION: NEWS; Pg. 14

LENGTH: 547 words

"SUPERSIZE" food and drinks should be banned in an attempt to combat the obesity epidemic, the new head of the Commons health committee has said.

Sarah Wollaston, a Conservative MP and former GP, said the state had a "duty to intervene" to protect current and future generations from unhealthy habits that could shorten their lives.

In her first major interview since being appointed as chairman of the committee, she said drastic measures were required to help Britons to reduce their bulging waistlines, and in particular, to prevent spiralling obesity among children.

"The scale of the marketing towards children of unhealthy foods is wholly unacceptable, in my view, given the scale of the problem," Dr Wollaston said.

"There is far more we could do. We are going to have to get tough with some of these very big vested interests in unhealthy foods."

She called for a direct ban on "supersized" foods and drinks, so that manufacturers would be restricted to producing chocolate bars, junk food meals and **fizzy drinks** in standard sizes. Dr Wollaston said: "Why aren't we taking more direct steps around supersizing? You go into the cinema and someone will ask if you want to supersize for an extra 20p - we don't need that."

She urged government action to introduce standard sizes, plus price differentials obliging retailers to place a lower price on low-sugar versions of foods and drinks. "You have a size of packaging and you say this is the standard," she said. "I don't personally think this is such a huge deal. I would do it.

"There is nothing to stop people buying two packets of something if you really want that volume of extra calories but most of us wouldn't choose to. Most of us would go for the default size."

The MP for Totnes, Devon, suggested the Government was too fearful of being accused of "nanny state" tactics, and said the public would be likely to adjust quickly to changes to protect their children's health.

"I don't believe it has to feel like the nanny state," she said. "I think we can put in place proportionate, tough measures, just as we've seen with seatbelt legislation and with stopping people smoking indoors." NHS data show one in three children is obese or overweight by the time they leave primary school, while two thirds of adults weigh too much.

Last month a study suggested around one in three adults in England have borderline diabetes, on top of the 4 million diagnosed with the disease.

NHS officials said they were considering measures to reduce standard portions as part of efforts to encourage Britons to eat less sugar.

Public Health England said it was seeking changes from food manufacturers so that smaller sizes of chocolate bars and **fizzy drinks** became the "default".

Officials also promised to explore ways to tackle "upselling" sales techniques - where customers are encouraged to purchase extra products or larger portions - but ideas floated so far have fallen short of a ban on large sizes.

Last month the National Institute of Health and Care Excellence called for thousands more people in England to be offered weight-loss surgery, to reduce obesity levels and cases of diabetes.

But Dr Wollaston said: "My view is that as an NHS we should be focusing on how to stop people getting to that point in the first place."

LOAD-DATE: July 26, 2014

LANGUAGE: ENGLISH

PUBLICATION-TYPE: Newspaper

JOURNAL-CODE: DTL

Copyright 2014 Telegraph Media Group Limited
All Rights Reserved

DAILY MAIL (London)

July 25, 2014 Friday

FIZZY DRINKS BOOST BRITVIC

BYLINE: BY DAILY MAIL REPORTER

LENGTH: 108 words

ROBINSON'S Barley Water-owner Britvic refreshed investors with an upbeat profit forecast, after enjoying a 4.1pc rise in sales during the past quarter, but saw its shares fall.

It now expects full-year underlying profit to come in at the upper end of an earlier forecast of between £148m and £156m.

The best performance came in its **fizzy drinks** division, which makes Pepsi and 7-Up under licence, with UK sales up 10.4pc.

By contrast still drinks such as Fruit Shoot declined in the quarter, with revenue down 3.9pc.

Irish revenues fell 2.5pc, France was up 5.6pc and its other international markets grew 3.8pc.

Britvic shares fell 12p to 724p.

© Daily Mail

LOAD-DATE: July 24, 2014

LANGUAGE: ENGLISH

PUBLICATION-TYPE: Papers

Copyright 2014 Associated Newspapers Ltd.
All Rights Reserved

i-Independent Print Ltd

July 25, 2014
First Edition

Tour puts the fizz into Britvic sales; The Business Matrix The day at a glance BEVERAGES

SECTION: BUSINESS; Pg. 50

LENGTH: 50 words

Britvic, the drinks giant whose brands include Pepsi and Robinsons squash, said its sponsorship of the Tour de France and demand for **fizzy drinks** had helped its thirdquarter sales to rise by 4.1 per cent to £329.5m. Full-year operating profit is expected to be at the top end of guidance of about £156m.

LOAD-DATE: July 24, 2014

LANGUAGE: ENGLISH

The Independent (London)

July 25, 2014 Friday
First Edition

Britvic riding crest of wave

SECTION: BUSINESS; Pg. 56

LENGTH: 28 words

The soft drinks giant Britvic said sponsorship of the Tour de France and demand for **fizzy drinks** have pepped up sales, prompting it to raise full-year profit forecasts

AP

LOAD-DATE: July 24, 2014

LANGUAGE: ENGLISH

PUBLICATION-TYPE: Newspaper

JOURNAL-CODE: IA

Copyright 2014 Independent Print Ltd
All Rights Reserved

The Telegraph
telegraph.co.uk

July 25, 2014 Friday 11:15 PM GMT

Ban supersized foods, says health committee chairman; The Government has a 'duty to intervene' and outlaw supersized portions to protect children from Britain's obesity epidemic, says Dr Sarah Wollaston

SECTION: NEWS

LENGTH: 929 words

"Supersized" food and drinks should be banned by law in a bid to combat Britain's obesity epidemic, the new head of the Commons health select committee has said.

Dr Sarah Wollaston, a Conservative MP and former GP, said the state had a "duty to intervene" to protect current and future generations from unhealthy habits threatening to shorten their lives.

In her first major interview since being appointed as chairman of the influential committee, she said drastic measures were required to help Britons to reduce their bulging waistlines, and in particular, to prevent spiralling obesity among children.

Dr Wollaston told The Daily Telegraph: "The scale of the marketing towards children of unhealthy foods is wholly unacceptable in my view given the scale of the problem.

"There is far more we could do; we are going to have to get tough with some of these very big vested interests in unhealthy foods."

The former GP called for a direct ban on "supersized" foods and drinks, so that manufacturers would be restricted to producing chocolate bars, junk food meals and **fizzy drinks** in standard sizes.

She said: "Why aren't we taking more direct steps around supersizing? You go into the cinema and someone will ask if you want to supersize for an extra 20p - we don't need that."

Dr Wollaston called for Government action to introduce standard sizes, plus price differentials obliging retailers to place a lower price on low-sugar versions of foods and drinks.

"You have a size of packaging and you say this is the standard," she said. "I don't personally think this is such a huge deal. I would do it."

"There is nothing to stop people buying two packets of something if you really want that volume of extra calories but most of us wouldn't choose to; most of us would go for the default size," she said.

The Conservative member for Totnes suggested the Government was too fearful of being accused of "nanny state" tactics, and said the public would be likely to adjust quickly to changes to protect their children's health.

"I don't believe it has to feel like the nanny state," she said. "I think we can put in place proportionate tough measures, just as we've seen with seatbelt legislation and with stopping people smoking indoors. They can be controversial at first but who now would want to go back to having people smoking in restaurants? In the same way, would we really miss being able to buy monster sizes of carbonated drinks?"

Latest NHS data shows one in three children are obese or overweight by the time they leave primary school, while two thirds of adults weigh too much.

Last month a study suggested that around one in three adults in England have borderline diabetes, on top of the four million people diagnosed with the disease.

Two years ago, New York health officials proposed a 16oz limit on soft drink sizes served in restaurants, cinemas and sports stadiums. However, it was subject to legal challenge after being opposed by drinks manufacturers and last month the state's highest court ruled that the board of health had exceeded its legal powers.

Last month NHS officials said they are considering measures to reduce standard portions as part of efforts to encourage Britons to eat less sugar.

Public Health England said it was seeking changes from food manufacturers so that smaller sizes of chocolate bars and **fizzy drinks** become the "default" on offer.

Officials also promised to explore ways to tackle "upselling" sales techniques - where customers are encouraged to purchase extra products or larger portions - but ideas floated so far have fallen short of a ban on large sizes.

Dr Wollaston said no one measure would be sufficient to combat the obesity crisis, but called for a range of interventions to tackle "marketing, price and availability" of unhealthy foods.

Last month the National Institute of Health and Care Excellence (Nice) called for thousands more people in England to be offered weight-loss surgery, in a bid to reduce obesity levels and rising cases of diabetes.

Since then the EU has ruled that obesity should be classed as a "disability" meaning employers have a duty to make adjustments for employees who are hindered by their weight.

The former GP suggested the combined impact of such rulings send out "completely the wrong message" to the public and to those trying to prevent obesity.

Describing the EU decision as "extraordinary" she said: "Obesity is not a disability; it causes disability. This is the trouble, they end up normalising it instead of putting the focus where it should be - prevention and early intervention helping people before they get to the point where morbid obesity causes serious joint problems and heart disease and diabetes."

She said that while Nice could argue that weight-loss surgery was cost effective for those who received it, their decision shifted the emphasis away from prevention of obesity.

"My view is that an NHS we should be focussing on how to stop people getting to that point in the first place," she said.

"It's not just where we are now, it's the tsunami coming towards us," she said, highlighting figures that show that among the most deprived groups, 12 per cent of children are obese when they start primary school.

Dr Wollaston also said she wanted to see clearer labelling of foods in supermarkets, so that parents had better information on which to base quick decisions.

"Very few people on a supermarket shop are going to be reading detailed labels; I think we need to move to a straightforward traffic light system taking account of sugar as well as fat," she said.

LOAD-DATE: July 26, 2014

LANGUAGE: ENGLISH

PUBLICATION-TYPE: Newspaper; Web Publication

JOURNAL-CODE: WEBDTNS

Copyright 2014 Telegraph Media Group Limited
All Rights Reserved

THE TIMES

The Times (London)

July 24, 2014 Thursday
Edition 2;
National Edition

Snack sales boost PepsiCo profits

BYLINE: Dominic Walsh

SECTION: BUSINESS; Pg. 43

LENGTH: 380 words

PepsiCo went some way to rebutting criticism from one of its investors after the soft drinks and snacks manufacturer increased its earnings guidance. The group yesterday reported a 0.5 per cent increase in second-quarter revenues to $16.9 billion, slightly ahead of Wall Street forecasts, while earnings were also better than expected, despite a 2 per cent fall in net income to $1.98 billion.

Its snacks division lifted organic revenue by 5 per cent on the back of strong sales of Lay's, Doritos and Cheetos, and its beverages division, which includes the Tropicana and Gatorade brands, was up 2 per cent. The increases were partly the result of price increases, which some analysts had suggested might be difficult to maintain. However, Indra Nooyi, PepsiCo's chief executive, said: "We feel comfortable that we can sustain our pricing."

Ms Nooyi's raising of its full-year earnings growth guidance from 7 per cent to 8 per cent may give her ammunition as she continues to resist calls from Nelson Peltz's Trian Fund Management, an investor, to spin off its faster-growth snacks division from soft drinks.

Jack Russo, an Edward Jones analyst, agreed that the second-quarter numbers would "take some of the heat off", although he said that it was "always good to have an activist shareholder in there stirring things up".

Some institutional investors have indicated that they would like PepsiCo to give Trian a boardroom seat, although support for a demerger looks thin on the ground. In an increasingly bitter battle, Mr Peltz has accused the PepsiCo chief executive of presiding over "a culture of sycophants".

At an investment conference this month, Mr Peltz said that he had met with more than 100 Pepsi investors and that he had not ruled out a proxy fight.

Hugh Johnston, the consumer group's chief financial officer, refused to be drawn on the battle with Trian, although he insisted that the company's performance benefited from keeping the two divisions under the same roof.

Pepsi's 2 per cent fall in secondquarter volumes of **fizzy drinks** in its core North American region compared with the flat performance reported by Coca-Cola a day earlier.

Shares of PepsiCo, which have been boosted by Mr Peltz's campaign, closed $1.65 higher at $90.82, a rise of 1.8 per cent, on Wall Street.

LOAD-DATE: July 24, 2014

LANGUAGE: ENGLISH

PUBLICATION-TYPE: Newspaper

JOURNAL-CODE: TIM

Copyright 2014 Times Newspapers Limited
All Rights Reserved

THE TIMES

The Times (London)

July 23, 2014 Wednesday
Edition 1;
National Edition

Coca-Cola disappoints, despite World Cup fizz

BYLINE: Dominic Walsh

SECTION: BUSINESS; Pg. 43

LENGTH: 285 words

Coca-Cola bounced back from its first decline in volumes since 1999 yesterday as it reported a 2 per cent increase in the amount of **fizzy drinks** sold in the second quarter on the back of its sponsorship of the World Cup.

However, profits and revenues at the world's biggest soft drinks maker fell amid higher marketing and restructuring costs and increased commodity prices. The slightly below-par performance came as total volumes, including juice and other non-carbonated drinks, grew by 3 per cent, despite a flat performance in North America.

Volumes of Coca-Cola branded drinks, which include Coke Zero and Diet Coke, rose by 1 per cent, globally and in its core North American region.Net revenues in the three months to June 27 fell by 1 per cent to $12.57 billion, with operating income down 2 per cent to $3.17 billion. In Europe, revenues rose by 7 per cent to $1.57 billion, while operating income rose by a similar level to $892 million.

Although the company does not split out its UK performance, the business is hoping the launch of Coca-Cola Life, a low-calorie version of its core brand that is sold in green rather than red packaging, and the introduction of glacéau smartwater will boost trading.

Coca-Cola, which has been attacked over its share plan by David Winters, the activist investor, reported its first volume decline in 15 years in the first quarter as consumers became more health-conscious.

Muhtar Kent, the chairman and chief executive, said he remained focused on "the work required to return our business to the level of sustainable growth we and our share owners expect". The company spent heavily on a revamped social media strategy and the personalised Share a Coke marketing campaign.

LOAD-DATE: July 23, 2014

LANGUAGE: ENGLISH

GRAPHIC: Profits and revenues fell at the world's biggest soft drinks maker amid higher marketing and restructuring costs
REX FEATURES

PUBLICATION-TYPE: Newspaper

JOURNAL-CODE: TIM

Metro (UK)

July 21, 2014 Monday
Edition 1;
Scotland

Liver disease rises by 20% among the under-tens

BYLINE: NICOLE LE MARIE

SECTION: NEWS; Pg. 33

LENGTH: 234 words

MORE children under the age of ten are being treated for liver disease, latest figures show.

A total of 346 were admitted to hospital after being diagnosed, a 20 per cent rise on the 289 figure 15 years before.

Among older children, aged ten to 17, the figure increased 112 per cent in the same period, from 197 to 417, according to figures from the Health and Social Care Information Centre.

The main causes of liver disease are obesity, undiagnosed illness and heavy consumption of alcohol.

Prof Martin Lombard, national clinical director for liver disease at the department of health, said 500,000 children aged four to 14 were at risk of liver disease and, by 2050, 63 per cent would be obese.

Alison Taylor, chief executive of the Children's Liver Foundation, said it wanted 'improved education of midwives and health visitors in spotting the early signs of liver disease in newborns'.

Labour's Luciana Berger, shadow public health minister, said: 'These figures reflect the growing national crisis in liver disease.

'Liver disease is being shamefully overlooked by this government.' Last year, 61 health groups called for a 7p-per-can 'fat tax' on **sugary drinks.**

In 2011, the department of health branded childhood liver disease a 'time bomb' that would explode unless parents drastically changed their children's lifestyles. It said: 'Reducing deaths from liver disease is a priority for this government.'

LOAD-DATE: July 21, 2014

LANGUAGE: ENGLISH

PUBLICATION-TYPE: Newspaper

JOURNAL-CODE: MTRscot

Copyright 2014 Associated Newspapers Ltd.
All Rights Reserved

The Telegraph
telegraph.co.uk

July 21, 2014 Monday 8:08 AM GMT

How to do cycling nutrition on the cheap; Five easy tips to cut the cost of your weekend rides, with supermarket alternatives for expensive carbohydrate bars, energy gels and sports drinks that deliver exactly the same results

BYLINE: By Oliver Duggan

LENGTH: 955 words

Cycling is an expensive hobby. Fork out the ludicrous cover price for a dedicated road cycling magazine and you can see in all its glossy glory the kind of money people are willing to spend on two wheels. I'm not sure how it happened, but " entry level bikes for under £1,000 " is considered bargain hunting. And that's before you've taken things like cages, bottles, shoes, bibs, jerseys, gloves, and helmets into account.

The high-carb cherry on top of this debt-denting sundae? Nutrition.

As the Tour de France gets underway, cyclists across the land are likely to be suckered into the glamour of four hour rides through the country. Given that any endurance athlete needs to refuel every 60 minutes or so, that's going to mean taking on a substantial amount of food during the ride. Over the course of a season, you can spend hundreds on speciality carbohydrate bars, caffeine gels, isotonic drinks, protein recovery and more.

For the budget-conscious, here are five cost-cutting alternatives.

1. Dioralyte

Even the shortest rides require you keep hydrated, and there is no end to sports drinks and powder mixes on offer for the cash rich cyclist. But if you're trying to save money on a long sportive, use Dioralyte.

The sachets of powder are designed to replace salts and nutrients lost through illness, but they're packed full of exactly the same goodness (glucose and minerals) that you sweat away while cycling. Six sachets will cost you a little over £3.50 at Boots, but an even better option is the pharmacy's own brand, which is almost identical and costs £2.99.

By comparrison, a single packet of Torq or Highl energy mix will cost you more than £1. Nuun hydration tablets are coming down in price all the time, but they will still cost you more than the Boots mix in most cycling shops.

A bonus tip: Dioralyte have introduced a new product called Relief, which combines the rehydration qualities of the original with rice starch. That starch adds about about 6 grams of carbohydrate per sachet, and carbs are always welcome (see point three). They're slightly more expensive at £4 for six, however.

The worst thing about using Dioralyte instead of your usual electrolyte-heavy sports powder? The taste, obviously. Their 'blackcurrant' flavour, for example, brings to mind memories of diarrhea rather than blackcurrent. Add a drop of cordial into the mix to expunge.

2. Coca Cola

If you can't help but buy a premade sports drink like Gatorade or Lucozade, there's a suprising and cheaper alternative. Coca-Cola, which is high in sugar, salts, carbohydrates and caffeine, basically offers the go-to mix for long rides. A Lucozade Sport costs around £1.20 and a Gotorade is £1.75, but a similar sized bottle of Coke is £1.15. The real saving comes with the bulkier buys, though. You can get nearly 2 litres of Coca-Cola for less than £2.

Fizzy drinks don't sit well while you're exercising, so the experts suggest you leave it to go flat - in the fridge with the lid off - before taking it on the road. Alternatively, buy a can during a drinks stop, pour it into a glass, and swirl with a spoon until the fizz leaves.

I've had mixed results with this. It works for a quick hit but, even more than the Dioralyte, taste is a significant issue. The sugary mixture can also gum up your water bottle.

3. Marzipan

Carbohydrates are the lifeblood of any cyclist. The main sports nutrition companies offer a myriad of carb bars that vary in quality and price.

My favourite, the SiS GO bar, is £1.20 for each 65g hit (on long rides, I find I can easily put away two or more). Each bar boasts more than 40g of carbohydrates - but there are plenty of supermarket alternatives at a fraction of the price.

Marzipan may be better associated with Christmas cakes, but the almond treat is also surprisingly high in carbohydrate. One 40g bar has 26 grams of carbs, which easily competes with the top-tier alternatives. And you can get a pack of five, chocolate-covered, from Aldi for £1.30.

They have the added bonus of being delicious.

4. Potato farls

If sweetness isn't your thing, Irish potato farls are another good and cheap source of carbohydrates while out on long rides.

Otherwise known as potato cakes, you can pick up a pack of six from Tesco for 50p and each one contains around 20g of carbs. Toast two before your ride, spread on some butter and sandwich them in foil.

They're quite dense so can be broken up without too many crumbs and eaten without stopping, and they can be salty, which makes a nice change from the fructose overload associated with most sports nutrition.

5. Honey

Energy gels are the in vogue sports nutrition, and for good reason. They are easy to consume and deliver results quickly, offering many a rider last-minute salvation from the dreaded bonk . But they are also expensive. SiS, Torq and Highl gels can cost up to £2.30 each.

Enter honey. According to a decade old University of Memphis study, which has started to resurface on sports blogs, honey is a natural sports gel. The double-blind test gave groups of cyclists a placebo, a manufactured carbohydrate gel or honey, and the results were staggering.

The riders who used the honey finished the 40-mile time trial on average three minutes quicker than those who took the placebo, and just seconds behind those on the tailor-made gel, and they did it with a lower heart rate. The reason is that honey contains a mix of easily absorbed sugars and - in every teaspoon - about 17g of carbohydrate.

The main problem? Figuring out how to transport it.

LOAD-DATE: July 21, 2014

LANGUAGE: ENGLISH

PUBLICATION-TYPE: Newspaper; Web Publication

JOURNAL-CODE: WEBDTNS

Copyright 2014 Telegraph Media Group Limited
All Rights Reserved

theguardian

The Guardian - Final Edition

July 18, 2014 Friday

Comment: Food is a drug, and we have to learn to say no: Our entire relationship with food has to change if we are to tackle the obesity crisis. Time to go back to school

BYLINE: Rosie Boycott

SECTION: GUARDIAN LEADER PAGES; Pg. 34

LENGTH: 1024 words

Food is one of the most crucial issues of our time. In America, 13 people die every hour from food-related illnesses, but we have no real solution to the obesity problem - the issues are myriad, and too ingrained in all

corners of our life and our profit-loving world for any one idea to work. And politicians like simple ideas. So while there should indeed be a tax on sweetened **fizzy drinks**, that won't solve the problem on its own. We need a full-scale culture shift, something no government can achieve.

The facts are chilling: one in seven hospital patients in the UK are diabetic; 3.8 million of us have diabetes; one in three is overweight; one in four is clinically obese; and 37% of 11-year-old children are overweight or obese. We are one of the most unhealthy countries in the world. And living with diet-related diseases means heart trouble, cancers, strokes, liver failure, wobbly knees, bad skin and amputation of limbs. It means hospitals spending fortunes to enlarge beds, operating tables, doorways and wheelchairs. Food-related illnesses now kill more people a year than smoking does, and disable an unknown number.

If I were talking about cancer or HIV, there would be a redoubling of efforts to "find a cure". But unlike many cancers, there is a complete, lifetime, free cure available. Just change what and how we eat.

So why don't we? Where food is concerned, we're complicated. We aspire to extreme thinness as advocated by fashion and reinforced by the cult of celebrity, but in reality we nearly all struggle with the pounds. We see people who are grossly fat, their wobbling, sad bodies being winched out of windows, and class that as "obesity", distancing ourselves from the term. As a recovering alcoholic it's a syndrome I'm familiar with - I might be getting drunk but I still have a roof over my head, unlike a "real" alcoholic, who sleeps on a park bench. Are we seriously so weak-willed that we can't say "no" to that extra cake? Go back just 30 years and very few people were obese. Go back 50 years and virtually no one was. For women, size 10 and 12 was the norm, rather than 14 and 16 today - and we ate three meals a day, with tea thrown in for special occasions. Most of us didn't eat unless we were sat at a table at a regular time of day.

That simple fact represented a problem for the food industry, which its army of chemists solved by designing products that override the "full" button, working like any other addictive drug to convince you that you really, really want - even need - that extra slice. Combine sugars, salts and fats, substances once so scarce we never evolved any need to limit their consumption, and you create a sensation as powerful as many banned substances. Then destroy the concept that eating takes place just at mealtimes. Enter any large supermarket today and you'll find whole aisles stocked with snacks.

The odds are stacked against us. The food industry spends over £1bn a year on marketing in the UK, compared with the paltry £14m spent on the government's anti-obesity campaign. Food companies can afford to pay for premium sites on supermarket shelves, so the most tempting products are always within reach. I have been addicted to smoking, drinking and briefly to drugs, and recognise the same "hit" from sugary, salty foods, and the same "cravings" for more. I haven't touched a drink or a drug in many years and I quit the fags almost four years ago, but food, because we have to eat, is far more complex.

Despite all the evidence against sugars and processed foods, governments worldwide do little. But inactivity is no longer excusable. That's why, this week, we are launching an initiative in two London boroughs, Lambeth and Croydon, to transform the way we eat. We're starting with the schools. Free meals for kids between five and seven years old start in September, and cooking goes back on the curriculum. Schools will use vegetable gardens not just for fun but also for teaching. Parents will be invited in to learn to cook too.

But it doesn't stop there: we're going to lobby fast food outlets, local restaurants, supermarkets and food manufacturers to change how and what they sell. Fast food outlets, for instance, can massively cut the calories in their meals by simply changing oil, or changing the size of the box the food is sold in. Small food producers will be funded and encouraged. We take inspiration from Finland where, in the 1970s, they reduced annual mortality from heart disease by 80% by changing the food culture. Like us, they began in the schools and spread the healthy culture outwards to parents.

Right now, the environment in which we make food choices, especially in poor areas, is extremely unhealthy. Food is on sale everywhere: in garages, cinemas, newsagents, pharmacies. And less than a pound will buy you chicken and chips, sugar-laden cakes, a big bag of crisps. Anna Soubry, when she was health minister, was shouted down for saying poor people were fatter than rich people, but she was right. Good food costs more.

A major problem when trying to treat obesity is precisely that. Those making the rules tend to be better off and able to buy better food. Deborah Cohen, an American physician and author of A Big Fat Crisis,

advocates drastic measures: restaurants should serve single portions and food should be banned from shops which aren't dedicated to food.

There are no signs of her measures being adopted anywhere yet, but if food-related illnesses continue to be responsible for more deaths worldwide than any other cause, do we have a choice? Dr Margaret Chan, director general of the World Health Organisation, sees obesity as the tip of the iceberg, pointing towards a catastrophe in which the world has to manage millions suffering from long-term chronic illness.

Like Cohen, Chan does not believe obesity to be a failure of personal will, rather of political will at the highest level. Our flagship project may seem small, but if we pull it off, maybe we will give our government sufficient ammunition to take on the power of the food industry. We have to start somewhere.

Rosie Boycott is the mayor of London's food adviser

LOAD-DATE: July 18, 2014

LANGUAGE: ENGLISH

PUBLICATION-TYPE: Newspaper

Copyright 2014 Guardian Newspapers Limited
All Rights Reserved

The Daily Telegraph

The Daily Telegraph (London)

July 15, 2014 Tuesday
Edition 2;
National Edition

Why dentists are pointing the toothbrush at Mum and Dad; Victoria Lambert drills down into the tooth decay crisis afflicting British children

BYLINE: Victoria Lambert

SECTION: FEATURES; Pg. 21

LENGTH: 718 words

Remind me, what century are we in? The news that 500 British children are admitted to hospital every week on account of rotten teeth made me think we'd slipped back to Tudor times, when you rubbed your teeth with your finger and hoped for the best.

But no, here we are in the 21st century, in which children have access to free, twice-yearly dental check-ups, school visits from grown-ups dressed as comedy molars, and a choice of more electric toothbrushes than flavours of sherbet bon-bon. Our children have been exposed to YouTube tutorials on dental hygiene featuring Elsa from Frozen, and Spongebob Square Pants toothpaste. And yet still their choppers are rotting like old vegetables in the fridge.

"How could you let your child be in so much pain?" asks dental surgeon Nigel Carter. He recalls looking inside the mouth of one young patient and seeing that out of 20 possible deciduous (baby or milk) teeth, 12 were rotted to the stumps.

The figures from the Health and Social Care Information Centre (HSCIC) are alarming: 25,812 children aged five to nine were admitted to hospital for dental problems in 2013/14 - 14 per cent up on 2010/11.

Dr Carter, chief executive of the British Dental Health Foundation charity, says: "The figures are a real representation of what is going on across the country - especially in the North West and parts of Yorkshire. Dental decay is a disease of poverty and disadvantage, very much on North-South lines."

But he doesn't accept it is a new problem: "If you look back 20 or 30 years, the number of children having extractions would have been far higher - the difference is that this would have been carried out in dental surgeries rather than in hospital.

"By the late Eighties, most extractions under general anaesthetic were carried out in community clinics, and since the late Nineties, all have been performed in hospitals for safety reasons. If something goes wrong, you want emergency facilities on site." None the less, Dr Carter finds the figures "horrendous - because this is a preventable condition".

So why are our bambinos turning into real-life gummy bears? Is it the fault of the NHS? Politicians? The food industry? Worse, says Dr Carter. "It's pure parental neglect."

Firstly, there is diet. "Everyone grazes constantly - we've gone from three meals a day to between seven and 10, plus sugary snacks, and all washed down with high-**sugar drinks**. There is a connection with the obesity crisis, but from the dental perspective, sugar is the prime culprit in dental decay."

Decay happens when sugars in food and drinks react with the bacteria in plaque, forming acids. Every time you eat or drink anything containing sugars, acids start to soften and dissolve the enamel. Attacks can last for an hour after eating or drinking, before natural salts in saliva cause the enamel to "remineralise" and harden again.

It's not just brightly coloured, **sugary drinks** that are to blame but fruit juices, too. "An awful lot have added sugars," he warns. "Parents must learn to check by reading labels: anything ending in '-ose', such as sucrose, maltose or fructose is a sugar. And try to ensure children are drinking all the drink in one go - not sipping, as that constantly bathes teeth in sweetness."

Be careful what you pack in their lunch boxes, too. "Things parents think of as healthy can be risky, such as sultanas or dried fruit, which are not only high in sugar but also sticky, so sugar stays in the mouth longer."

Dr Carter is also concerned that children are not taught a proper care regime at home. "Some parents don't even provide a toothbrush or fluoride toothpaste, and don't supervise their children's cleaning regime. We run Aladdin-style schemes - new toothbrushes for old. Kids turn up with a fresh brush, so it's clear that they have been bought a brush so they can join in, which means they don't have one at home or are simply using the one family toothbrush."

Lastly, parents are not taking their children to the dentist every six months for a check-up, where early signs of decay are picked up. "If a child's first experience at the dentist is pain, and being shipped to hospital for multiple extractions, you are probably setting that child up to be dental-phobic for life."

"Foods we think of as healthy can be riskysuch as dried fruit

LOAD-DATE: July 15, 2014

LANGUAGE: ENGLISH

GRAPHIC: Why are our bambinos turning into real-life gummy bears? Pure parental neglect, say experts GETTY IMAGES/BRAND X

PUBLICATION-TYPE: Newspaper

JOURNAL-CODE: DTL

Copyright 2014 Telegraph Media Group Limited
All Rights Reserved

theguardian

Guardian.com.

July 15, 2014 Tuesday

Junk food taxes should go to fight obesity, say councils

BYLINE: Sarah Boseleytheguardian.com

LENGTH: 509 words

ABSTRACT

Soft drinks, sweets and fast food are hugely profitable and doing our health serious damage as we all get fatter. The Local Government Association, now responsible for tackling overweight, wants a £1 billion chunk of the VAT collected to go to an obesity action fund

FULL TEXT

The soft drinks market in the UK is worth £14,955 million, chocolate confectionery £3,976 million and takeaway food £6,200 million. That's a big pile of money spent, thanks to the persuasive marketing of the food and drink industry, on getting fat. The Local Government Association, which has now been handed the job of helping us combat obesity, thinks it should have a slice for its fighting fund.

The big idea, which is now in its manifesto ahead of the general election, is that a fifth of the current VAT on fast food and **sugary drinks** should be diverted into an "obesity action fund". Over £5 billion is raised in taxes on chocolates, **soft drinks** and takeways. If the fund could have 20% of that, says the association representing nearly 400 local councils, it would have £1 billion to spend on tackling one of the biggest health problems of our time.

David Sparks, LGA chairman said:

Our pioneering plans represent a bold, radical and innovative approach to tackling the obesity crisis, which is costing the country £5 billion a year.

By taking a slice of existing VAT we would raise millions which would help transform the lives and futures of obese and overweight people - including the country's three and half million children - rather than simply swelling the Treasury's coffers.

This extra money would be a massive boost in the battle to combat obesity. It would help tackle head-on the crippling problems often associated with the condition, like diabetes.

Councils are doing everything they can to curb obesity at a local level. This involves ground-breaking health and fitness programmes and schemes. This extra money would enable them to ramp up their efforts and really make a major impact on tackling this condition.

There is an element of electioneering here, of course. This is part of a report from the LGA called "Investing In Our Nation's Future, The First 100 Days of The Next Government". Local authorities have been given responsibility for public health, together with a ring-fenced budget, so some may argue they already have money to spend. But they say it's not enough - and when you look at the amount spent by the food and drink industry on marketing, that's not hard to understand. If the LGA were able to raise £1 billion from a share of VAT, that would just match the marketing spend of the food industry in the UK.

I would not begrudge local councils more cash - generated, after all, by our fast food habits - to try to repair the damage, but I think we need to go further. The prices of fast food, sweets and soft drinks, which are far too cheap, need a hike - and real food, such as fruit and vegetables, needs to be subsidised. That would be using the tax system for a real health benefit - and it is only national governments that can do it.

LOAD-DATE: July 15, 2014

LANGUAGE: ENGLISH

PUBLICATION-TYPE: Newspaper

Copyright 2014 Guardian News and Media Limited or its affiliated companies
All Rights Reserved
Registered in England and Wales. No. 908396

THE TIMES

The Times (London)

July 15, 2014 Tuesday
Edition 1;
National Edition

We all need to brush up on 'healthier' teeth care

BYLINE: Dr Mark Porter

SECTION: T2;FEATURES; OPINION COLUMN; Pg. 6

LENGTH: 702 words

Dental health has advanced since I was a child when fillings were widely accepted as an inevitable part of growing up, but the latest statistic suggests the trend towards healthier teeth may be reversing - at least in younger children.

Last year nearly 26,000 primary school children in England were admitted to hospital to have rotting teeth removed (all of their teeth in some cases), 3,000 more than in 2010. And recent surveys suggest that about a quarter of five-year-olds now have some degree of dental decay, increasing to more than half in the more deprived parts of the UK.

There has always been a strong correlation between social deprivation and dental decay, thanks mainly to a poor diet, but there is now growing concern that parents from more affluent backgrounds are also endangering their children's smiles by offering them "healthier" options.

Most people are fully aware of the hazards posed by sugar-laden products but carbohydrates and acids hidden in other foods and drinks often sneak in under the radar. Our teeth are constantly undergoing a cycle of damage and repair. Acids in fruit juices and **fizzy drinks**, and those released by bacteria digesting carbohydrate such as sugar and starch in our diet, eat away at dental enamel. But once diluted (within 30 minutes in most cases) minerals found in saliva, and drinks such as milk and water, replace those the acids have dissolved.

One of the main determinants of dental decay is the frequency with which these cycles of damage and repair occur. It is not about how much carbohydrate and acid you eat or drink, so much as how often, and it pays to limit the number of attacks by encouraging proper meals and avoiding frequent snacking or grazing. From a purely dental point of view it is better to eat a bag of sugar in one go than a teaspoonful ten times a day. But what else can parents do to protect their children? Step one in any good dental regime is to brush teeth at least twice a day. This should be first thing in the morning before breakfast, and last thing at night before going to bed. Brushing immediately after a meal removes food debris but can accentuate enamel damage, particularly if that meal has been high in sugars or acids.

Always use a fluoride-based toothpaste (children under three should use a weaker version - ask your dentist) and make sure they don't swallow it. The latest advice is to spit out the toothpaste but not to rinse the mouth with water afterwards as this increases the take-up of fluoride "hardening" the enamel.

If you are giving your child a fruit juice or smoothie, limit them to no more than 250ml once a day, and provide a straw (research suggests this limits acid attack as children are less likely to swill the drinks around the mouth) - and this straw trick applies to any sugary or **fizzy drink**, including diet versions that still contain damaging acids. Better still, get into the habit of offering water as the beverage of choice.

Most decay in young children occurs in crevices on the biting surface of teeth, particularly at the back of the mouth, so make sure this area is brushed carefully. Your dentist or hygienist will be able to demonstrate the correct technique and ensure your child isn't brushing too hard as this can cause permanent gum recession. Dentists also now advocate a golden hour before bed when a child should not eat or drink anything other than water.

Follow the steps above, and ensure your children visit the dentist regularly, and they should enter early adult life with a full set of intact teeth. After that it is up to them, but at least you will have given them the best possible foundation and their smile has the potential to last a lifetime.

Accidents will happen ? Dental decay isn't the only hazard to our children's teeth - INJURIEs sustained during sport and falling off bikes and trampolines also take their toll. Ask your dentist about a correctly fitted mouthguard if your child takes part in contact sports such as rugby and hockey. ? If they do lose a tooth, pick it up, pop it in a glass of milk and call your dentist immediately. Reimplanting missing adult (as opposed to baby) teeth is one of the few real dental emergencies and success rates are high.

LOAD-DATE: July 15, 2014

LANGUAGE: ENGLISH

GRAPHIC: JOE MCLAREN

PUBLICATION-TYPE: Newspaper

JOURNAL-CODE: TIM

Copyright 2014 Times Newspapers Limited
All Rights Reserved

DAILY MAIL (London)

July 14, 2014 Monday

500 CHILDREN IN HOSPITAL A WEEK WITH ROTTEN TEETH

BYLINE: BY SOPHIE BORLAND HEALTH CORRESPONDENT

LENGTH: 419 words

NEARLY 500 children a week are being admitted to hospital with rotten teeth, NHS figures show.

It is now the main reason for youngsters needing hospital treatment and dentists say the culprits are fruit juice and **fizzy drinks**.

Most children need between four and eight of their baby teeth extracted, although some are having all 20 taken out.

Figures also show that more than a quarter of five-year-olds have some degree of tooth decay and in some areas of England it is well over a third.

Only last month new NHS guidelines urged the public to slash sugar intake to as little as five teaspoons a day over concerns that it is to blame for rising rates of obesity and type 2 diabetes.

Experts are also worried that sugar is behind an increase in tooth decay in children and could affect their ability to learn.

Figures from the Health and Social Care Information Centre show that 25,812 children aged five to nine were admitted to hospital for dental problems for the year 2013/14. This is a 14 per cent rise in three years; in 2010

/11 there were 22,574 such admissions.

Kathryn Harley, a consultant in paediatric dentistry at the Edinburgh Dental Institute, said: We have children who require all 20 of their baby teeth to be extracted. It beggars belief that their diets could produce such a drastic effect.

They are going into hospital because they are either presenting with acute problems with pain or because the stage of dental disease, the number of teeth with decay, is such that they need a general anaesthetic.'

She said most children will require maybe four or eight teeth to be extracted, quite a few will require ten, 12 or 14'.

Graham Barnby, honorary vice-president of the British Dental Health Foundation, said: It all relates to the consumption of sugary, **fizzy drinks**.'

The NHS says women should limit themselves to between five and six teaspoons of sugar a day and men seven to eight.

Currently, Britons consume an average of 15 teaspoons daily.

The Government's chief advisor on obesity, Professor Susan Jebb, also urged parents to ban fruit juice and **fizzy drinks** from the dinner table and stick to water.

A glass of fruit juice has five teaspoons of sugar while Coca-Cola has eight. This would take a person over the limit before even eating. Separate figures from Public Health England show that 27 per cent of five-year-olds have tooth decay - rising to 35 per cent in the North West.

A spokesman for NHS England said: Parents of young children should discourage them from drinking **fizzy drinks**.'

© Daily Mail

LOAD-DATE: July 13, 2014

LANGUAGE: ENGLISH

PUBLICATION-TYPE: Papers

Copyright 2014 Associated Newspapers Ltd.
All Rights Reserved

Daily Mirror

July 14, 2014 Monday
Edition 1;
Northern Ireland

Kids sent to hospital with rotten teeth

SECTION: NEWS; Pg. 20

LENGTH: 133 words

SOME 500 primary school children a week are admitted to hospital in England with rotten teeth, figures reveal.

Almost 26,000 children aged between five and nine, were hospitalised in England to have teeth taken out in 2013-14. Tooth decay is the No1 reason why primary school children are admitted to hospital.

Dental leaders say the disease would be avoided if parents stopped their children guzzling **fizzy drinks.** British Dental Health Foundation chief Dr Nigel Carter said: "It's down to parental care. They also need to ensure children use fluoride toothpaste."

He said despite a decline in cases in the past 30 years, the figures are worrying.

Dr Carter added: "Parents need to brush children's teeth well. If they aren't they're neglecting the child's health. Tooth decay is a preventable disease."

LOAD-DATE: July 14, 2014

LANGUAGE: ENGLISH

PUBLICATION-TYPE: Newspaper

JOURNAL-CODE: DMR

Copyright 2014 MGN Ltd.
All Rights Reserved

The Daily Telegraph

The Daily Telegraph (London)

July 14, 2014 Monday
Edition 1;
National Edition

Bad teeth is top cause of children's hospital admissions

BYLINE: Miranda Prynne

SECTION: NEWS; Pg. 8

LENGTH: 392 words

ROTTEN teeth was the number one cause of hospital admissions among young children, new figures show.

Almost 26,000 primary-school age pupils had multiple teeth removed in the past year and nearly 500 five to nine-yearolds had hospital dental treatment each week in 2013-14.

In some cases dentists removed all young patients's first teeth, according to statistics analysis by the Health and Social Care Information Centre analysis published in The Sunday Times.

The number of hospital admissions for five to nine-year-olds with dental problems increased by more than 3,000 in just three years, from 22,574 in 2010-11 to 25,812 in 2013-14, according to The Health and Social Care Information Centre.

The figures have led to calls to limit the amount of **sugary drinks** and fruit juice drunk by children. Graham Barnby, honorary vice-president of the British Dental Health Foundation, said: "It all relates to the consumption of sugary, **fizzy drinks**."

Kathryn Harley, a consultant in paediatric dentistry, said: "We have children who require all 20 of their baby teeth to be extracted. It beggars belief that their diets could produce such a drastic effect."

She added: "They are going into hospital because they are either presenting with acute problems with pain or because the stage of dental disease, the number of teeth with decay, is such that they need a general anaesthetic."

Ms Harley said most children needed four to eight teeth removed but up to 14 extractions were not uncommon.

She claimed fruit juice should be banned in schools to prevent the problem worsening and believed parents were "inadvertently responsible".

NHS England urged parents to protect their children's teeth. "We have some of the lowest rates of tooth decay in the world but these statistics are of course worrying," it said. "Parents of young children should discourage them from drinking **fizzy drinks** as this can lead to tooth decay."

The rising number of hospital admissions has led to questions about whether dentists should carry out more fillings.

Professor Jimmy Steele, the head of Newcastle University's dentistry school, claimed some dentists, worried about fillings' effectiveness, preferred to monitor decay in the baby teeth instead.

Tonsillitis is the second most common reason for five to nine-year-olds being admitted to hospital, with 11,522 cases in 2012-13.

LOAD-DATE: July 14, 2014

LANGUAGE: ENGLISH

PUBLICATION-TYPE: Newspaper

JOURNAL-CODE: DTL

Copyright 2014 Telegraph Media Group Limited
All Rights Reserved

The Sun (England)

July 14, 2014 Monday
Edition 2;
National Edition

GNASHNAL EPIDEMIC; PERIL OF KIDS' ROTTEN TEETH

BYLINE: HOLLY CHRISTODOULOU ;BEN PERRIN

SECTION: NEWS; Pg. 11

LENGTH: 522 words

HEALTH experts warned of a tooth decay "epidemic" last night after it was revealed rotten teeth are the main reason for young kids being hospitalised.

Leading medics slammed parents who let youngsters guzzle **sugary drinks** with no thought for their gnashers.

More than a quarter of four-yearolds suffer from tooth decay, and increasing numbers of kids are going to hospital to get several crumbling teeth pulled out at once under general anaesthetic.

Urgent action is needed to tackle the nation's addiction to sugar, experts warned.

Tam Fry, chairman of the Child Growth Foundation, said shocking figures from Public Health England last week showed 28 per cent of our fouryear-olds have some form of tooth decay. He told The Sun: "It's an epidemic, hugely embarrassing, dangerous and unhygienic. Sugar is absolutely disastrous to children's teeth and has a residue into adult problems as well."

Separate figures show 25,812 children aged five to nine have needed hospital treatment for dental problems - up by more than 3,000 on three years ago.

And 8,758 toddlers aged four and under have been hospitalised for tooth issues, up from 8,060 in 2010-11, according to a Health and Social Care Information Centre study for NHS England. It is thought some children were having as many as 14 teeth removed.

British Dental Health Foundation chief Dr Nigel Carter said: "It's an absolutely shocking figure and should be virtually zero.

"This sets children up with bad dental health for life.

"The blame for these appalling figures falls squarely in parents' laps. They should try to limit **sugary drinks** and make sure children have a good oral hygiene routine."

Campaigns highlighting the amount of sugar in our most popular drinks have gone viral on social media. One called Think Before You Drink shows sodas and fruit juices with their true sugar content in see-through bags underneath.

A standard 330ml can of Coke contains 35g of sugar - a massive SIX teaspoons - while a 500ml bottle of Lucozade Energy has 43.5g and a Frijj strawberry milkshake 471ml bottle has 46.1g.

Last night experts increased their calls for Health Secretary Jeremy Hunt to act. Cardiologist Aseem Malhotra from Action on Sugar said: "We know tooth decay is the commonest cause of chronic pain in children worldwide. The single and only dietary factor is sugar.

"We need Jeremy Hunt to pull his finger out and start regulating the food industry.

"Parents need to understand there is no requirement for children to have added sugar for energy.

"This is something we've been led to believe by the food industry who are only interested in selling food and not your health."

He said the average four to eightyear-old should have no more than three teaspoons of sugar a day - equal to that in half a can of coke or a third of a regular choc bar.

NHS England spokesman insisted Britain has some of the lowest rates of tooth decay in the world, but admitted: "These statistics are of course worrying. Parents of young children should discourage them from drinking **fizzy drinks**."

Health experts last month called for a tax on **fizzy drinks** to tackle obesity.

holly.christodoulou@the-sun.co.uk

LOAD-DATE: July 14, 2014

LANGUAGE: ENGLISH

GRAPHIC: Bitter sweet ... drinks and their sugar content

PUBLICATION-TYPE: Newspaper

JOURNAL-CODE: SUN

Copyright 2014 NEWS GROUP NEWSPAPERS LTD
All Rights Reserved

The Sun (England)

July 14, 2014 Monday
Edition 1;
Scotland

THE SCOTTISH Sun SAYSDitch the fizz

SECTION: EDITORIAL; OPINION; LEADING ARTICLES; Pg. 8

LENGTH: 124 words

WE'VE known for generations that **fizzy drinks** rot your teeth. It's time today's parents took the warnings seriously.

Most kids would guzzle sugary stuff all day long if they could.

Mums and dads must not let them.

Sweetened drinks and fruit juices are already blamed for Britain's obesity epidemic.

Dentists now say they are the prime cause of the alarming rise in primary-age kids needing operations to remove rotten teeth. Some have had all 20 baby teeth taken out.

We accept that Britain still has low rates of decay. But there is plenty of evidence that too many **fizzy drinks** are damaging our children, often for life.

Parents should wean kids off the pop and on to water.

Yes, it's dull and they may squeal for a while.

But it's healthier. And cheap.

LOAD-DATE: July 14, 2014

LANGUAGE: ENGLISH

PUBLICATION-TYPE: Newspaper

JOURNAL-CODE: SUNscot

Copyright 2014 NEWS GROUP NEWSPAPERS LTD
All Rights Reserved

The Sun (England)

July 14, 2014 Monday
Edition 2;
Scotland

Epidemic fears as kid teeth rot rises

BYLINE: HOLLY CHRISTODOULOU ; BEN PERRIN

SECTION: NEWS; Pg. 6

LENGTH: 110 words

HEALTH experts warned of a tooth decay "epidemic" as it emerged rotten teeth are the main reason for young kids being hospitalised.

Leading medics slammed parents who let youngsters guzzle **sugary drinks** with no thought for their gnashers.

More than a quarter of four-year-olds suffer from tooth decay, official stats show.

And 25,812 children aged five to nine have needed hospital treatment for dental problems - up by more than 3,000 on three years ago.

Urgent action is needed to tackle the nation's addiction to sugar, experts warned.

Tam Fry, of the Child Growth Foundation, warned: "It's an epidemic. Sugar is disastrous to children's teeth."

Sun Says - Page Eight

LOAD-DATE: July 14, 2014

LANGUAGE: ENGLISH

GRAPHIC: Sugar risk ... gnashers

PUBLICATION-TYPE: Newspaper

JOURNAL-CODE: SUNscot

Copyright 2014 NEWS GROUP NEWSPAPERS LTD
All Rights Reserved

MailOnline

July 13, 2014 Sunday 11:56 AM GMT

500 children suffering from tooth decay are hospitalised every WEEK as sugary drinks and fruit juice take their toll

BYLINE: DARREN BOYLE

SECTION: NEWS

LENGTH: 645 words

- Researchers found that more than 25,000 children needed teeth extracted

- Some children had all 20 of their milk teeth removed because of decay
- More than half of children in Leicester are suffering from tooth decay
- Bad oral health linked to deprivation with a sharp north/south divide

Almost 500 children are being hospitalised each week to have teeth removed new figures have revealed.

It is estimated that one in four primary school children are suffering tooth decay as a result of drinking too much fruit juice or soft drinks.

The research by the Health and Social Care Information Centre for NHS England found the level of admissions had increased over the past 12 months.

In 2010/11, 22,752 were hospitalised in order to have one or more teeth removed. That figure increased to to 25,812.

In some extreme cases, dental surgeons were forced to remove all of their milk teeth because of excessive decay.

Kathryn Harley, consultant in paediatric dentistry at the Royal College told The Sunday Times : 'We have children who require all 20 of their baby teeth extracted.

'It beggars belief that their diets could produce such a drastic effect.

'They are going into hospital because they are either presenting with acute problems with pain, or because the stage of dental disease, the number of teeth with decay, is such that they need a general anaesthetic.

A major survey of five-year-old children discovered that more than a quarter were suffering from tooth decay.

According to the survey: 'The results reveal wide variation in the prevalence and severity of dental decay. The areas with poorer oral health tend to be in the north and in the more deprived local authority.'

The figures show that more than half of children in Leicester, 53 per cent, compared with just 12.5 per cent in Brighton and Hove.

Children in Rochdale have on average five decayed teeth compared with 1.88 in South Gloucestershire.

More than eight per cent of children aged five in Blackpool have been hospitalised at least once to have a tooth removed.

The researchers claimed: 'This report highlights the wide variation in the levels of dental decay experienced by five-year-old children living in different parts of the country and in different life circumstances.

'The cause of dental decay is well understood and is related to the frequent exposure of teeth to fermentable carbohydrates.

Provisional figures for the the period 2013-14 show that 25,812 children aged between five and nine have been admitted to hospital to have multiple tooth extractions, up from 22,574 three years previously.

Some dentists observe how decay progresses in baby teeth because there is uncertainty about the effectiveness of fillings, said Professor Jimmy Steele of Newcastle University.

We have children who require all 20 of their baby teeth extracted. 'It beggars belief that their diets could produce such a drastic effect. Paediatric dentist Kathryn Harley

'A lot of dentists are unhappy about taking out teeth generally. They certainly don't like to take kids' teeth out.'

The number of children aged from zero to four admitted to hospital to have teeth out has also increased, from 8,060 in 2010-11 to a provisional figure of 8,758 in 2013-2014.

Other key findings for children being hospitalised show that one in 20 of girls aged from 15 to 19 being treated by a consultant was as a result of intentional self-harm, while boys were more likely than girls to have been injured in an assault, two per cent..

Similar differences were also apparent for 10 to 14-year-olds, but they were more pronounced for the older age group. There were more similarities in children up to the age of nine.

There were a total of 2.5 million Finished Consultant Episodes (FCEs) in the 12 month period from July 2012 to June last year for children aged up to 19, a very small increase of 0.1 per cent on the previous 12 months.

LOAD-DATE: July 13, 2014

LANGUAGE: ENGLISH

PUBLICATION-TYPE: Web Publication

JOURNAL-CODE: WEBDM

Copyright 2014 Associated Newspapers Ltd.
All Rights Reserved

THE SUNDAY TIMES
The Sunday Times (London)

July 13, 2014 Sunday
Edition 3;
National Edition

Rotten teeth put 26,000 children in hospital

BYLINE: Sarah-Kate

SECTION: NEWS; FRONT PAGE; Pg. 1

LENGTH: 403 words

TOOTH decay has become by far the biggest reason why primary school children are admitted to hospital, new research has found.

Almost 26,000 children in England aged between 5 and 9 have been hospitalised to have multiple tooth extractions in 2013-14 - the equivalent of nearly 500 a week. Dental leaders described the figures as shocking and said they were proof of the damage being done to children's teeth by the overconsumption of **sugary drinks** and fruit juice.

Kathryn Harley, a consul- tant in paediatric dentistry, said: "We have children who require all 20 of their baby teeth to be extracted. It beggars belief that their diets could produce such a drastic effect."

Graham Barnby, honorary vice-president of the British Dental Health Foundation, said: "It all relates to the consumption of sugary, **fizzy drinks**."

According to the Health and Social Care Information Centre, which analyses data for NHS England, the number of admissions for dental problems among 5 to 9-yearolds rose from 22,574 in 2010-11 to 25,812 in 2013-14.

Harley said: "They are going into hospital because they are either presenting with acute problems with pain or because the stage of dental disease, the number of teeth with decay, is such that they need a general anaesthetic."

She said most children will require "maybe four or eight teeth to be extracted, quite a few will require 10, 12 or 14". In her view the consumption of fruit juice is so damaging to children's teeth that it should be banned at school.

Parents were "inadvertently responsible and they care dreadfully that their child is in this position", she said.

There is also concern that children are ending up in hospital because dentists do not carry out fillings. Professor Jimmy Steele, head of the dentistry school at Newcastle University, said some dentists prefer to observe how decay in baby teeth progressed because of uncertainty over the effectiveness of fillings.

Steele said: "Dentists are much less likely nowadays than they used to be to try to fill teeth using conventional measures."

The second most common reason for children of 5 to 9 being hospitalised is tonsillitis, with 11,522 cases in 2012-13, the latest figures available.

NHS England said: "We have some of the lowest rates of tooth decay in the world but these statistics are of course worrying. Parents of young children should discourage them from drinking **fizzy drinks** as this can lead to tooth decay."

LOAD-DATE: July 13, 2014

LANGUAGE: ENGLISH

PUBLICATION-TYPE: Newspaper

JOURNAL-CODE: STS

Copyright 2014 Times Newspapers Limited
All Rights Reserved

The Telegraph

telegraph.co.uk

July 13, 2014 Sunday 9:16 AM GMT

Tooth decay is the biggest cause of primary school children being hospitalised; Rotting teeth is the most common cause of primary school aged children being admitted to hospital, new figures show

BYLINE: By Miranda Prynne News Reporter

SECTION: NEWS

LENGTH: 419 words

Almost 26,000 primary school children were treated for tooth decay in the past year, making it the most common reason youngsters are admitted to hospital, research shows.

Nearly 500 children aged five to nine were hospitalised due to rotten teeth each week in 2013-14.

In some cases dentists are forced to remove all 20 baby teeth from their young patients.

The figures sparked further calls for a crackdown on **sugary drinks** and fruit juice, The Sunday Times reported.

The number of hospital admissions for five to nine-year-olds with dental problems increased by more than 3000 in the just three years, from 22,574 in 2010-11 to 25,812 in 2013-14, according to the Health and Social Care Information Centre.

Graham Barnby, honorary vice-president of the British Dental Health Foundation, said: "It all relates to the consumption of sugary, **fizzy drinks**."

Kathryn Harley, a consultant in paediatric dentistry, said: "We have children who require all 20 of their baby teeth to be extracted. It beggars belief that their diets could produce such a drastic effect."

She added: "They are going into hospital because they are either presenting with acute problems with pain or because the stage of dental disease, the number of teeth with decay, is such that they need a general anaesthetic."

Ms Harley said most children need four to eight teeth removed but that having ten to 14 extracted is not uncommon.

She claimed fruit juice should be banned in schools to prevent the problem worsening and pointed the finger at parents who were "inadvertently responsible".

NHS England also urged parents to take action to protect their childrens' dental health.

"We have some of the lowest rates of tooth decay in the world but these statistics are of course worrying," the health body said in a statement.

"Parents of young children should discourage them from drinking **fizzy drinks** as this can lead to tooth decay."

The rising number of young tooth decay patients has also raised questions about whether dentists should carry out more childhood fillings.

Professor Jimmy Steele, head of the dentistry school at Newcastle University, said some dentists are unwilling to carry out filling due to uncertainty about their effectiveness.

They prefer to monitor decay in the baby teeth, he claimed.

"Dentists are much less likely nowadays than they used to be to try to fill teeth using conventional measures," he said.

Tonsillitis is the second most common reason for children of 5 to 9 being admitted to hospital, with 11,522 cases in 2012-13.

LOAD-DATE: July 13, 2014

LANGUAGE: ENGLISH

PUBLICATION-TYPE: Newspaper; Web Publication

JOURNAL-CODE: WEBDTNS

Copyright 2014 Telegraph Media Group Limited
All Rights Reserved

The Daily Telegraph

The Daily Telegraph (London)

July 12, 2014 Saturday
Edition 1;
National Edition

Markets recover as Portugal bank crisis fears recede; Market report

BYLINE: Rebecca Clancy

SECTION: BUSINESS; Pg. 28

LENGTH: 394 words

STOCKS traded in London recovered their poise yesterday after fears of a banking crisis unfolding in Portugal eased.

After trading sideways for most of the day, the FTSE 100 closed up 4.71, or 0.1pc, to 6,677.08.

"Investors were recuperating from Thursday's Portuguese reminder of the risks still inherent in the continent's banking system," said Jasper Lawlor, market analyst at CMC Markets.

It was a similar picture on the FTSE 250, which finished the day up 14.33, or 0.1pc, at 16,725.68.

Lower sugar prices prompted Goldman Sachs to upgrade the company that bottles **fizzy drinks** for Coca-Cola.

Coca-Cola Hellenic (CCH) rose 39, or 3pc, to £13.51 after Goldman raised the drinks bottle maker from "neutral" to "buy". The bank is predicting that sugar prices could fall more than 30pc over the next 10 years.

"We believe lower sugar prices are here to stay as companies become more competitive up to and beyond the end of quotas in 2017," Goldman said.

CCH, which used to be Greece's largest company until it moved its headquarters to Switzerland and listed in London in 2012, will not be the only winner from a potential sugar price slump.

Britvic, the drinks company behind Robinsons and Tango, rose more than 1pc during yesterday's trading following the note from Goldman but couldn't hold on to the gains and closed down 2.5, or 0.4pc, to 698.5p.

"While the retail environment remains tough, we believe CCH and Britvic will be able to hold on to at least some of the benefit from lower costs," Goldman added.

At the other end of the main index was Randgold Resources. The gold miner had risen nearly 3pc on both Wednesday and Thursday, going against the grain of the market as investors looked for a safe haven as the threat of a eurozone crisis reared its head. But as markets stabilised yesterday it fell back and finished the day down 115p, or 2.2pc, to £51.20. It was a similar story at Mexican precious metal miner Fresnillo, which fell 17, or 1.8pc, to 928p.

On the mid-cap index, something was off at Dairy Crest, which dropped 15.3 to 440.1p, after analysts at Societe General cut the rating on one of the big three milk suppliers in the country from "hold" to "sell".

"The removal of production quotas for EU milk in 2015 is likely to have negative consequences for Dairy Crest's key profit engine (UK cheese) in our view," the note from SocGen said.

LOAD-DATE: July 12, 2014

LANGUAGE: ENGLISH

PUBLICATION-TYPE: Newspaper

JOURNAL-CODE: DTL

Copyright 2014 Telegraph Media Group Limited
All Rights Reserved

The Daily Telegraph
The Daily Telegraph (London)

July 12, 2014 Saturday
Edition 1;
National Edition

How to put the squeeze on gastric bands; Education is the key in the fight against obesity - and perhaps, for once, nanny does know best

BYLINE: A N WILSON

SECTION: EDITORIAL; OPINION, COLUMNS; Pg. 21

LENGTH: 951 words

We live in strange times. In my youth we worried ineffectually about the starving millions. Now, in Britain, two thirds of the population are deemed to be overweight, and a quarter actually www.obese.No wonder that Nice, the health watchdog, says we must offer what is called weight-loss surgery - gastric bands or gastric bypass - on the NHS to those whose obesity appears to have swollen out of control.

In the past, my reaction to this would have been simple. Nice must be mad! All that is required, I would have believed, would be to tell these fatties to stop guzzling burgers and chips. But of course, such a reaction is woefully simplistic. One of the reasons why so many people are fat is that the food on offer in all supermarkets - as well as the fast-food chains - is fi lled with sugar. There are one and a half teaspoonfuls of sugar in every slice of sliced bread. Every dollop of ketchup contains a cube of sugar. Go out to Pizza Express for a Margherita and you will be consuming three cubes of sugar. Those who eat this muck are not necessarily any greedier than those who eat lentils, raw carrots and yoghurt. But by failing to read the label, they are adding to their waistline, and hugely increasing the likelihood that they will develop type 2 diabetes - with the risk of blindness, strokes, heart disease and years of immobility as they sit, like beached whales, in their specially constructed giant armchairs.

It is in order to help people caught in this spiral that Nice has decided that it would be cheaper to fix gastric bands - an operation that costs an average of £6,000 - than to live with the consequences of millions of diabetic, obese, blind people who are scarcely able to waddle to the lavatory, and who will need round-the-clock expensive care for the rest of their depressed lives.

You can see the logic of it, but it sounds like defeatism. Surely we should be concentrating our resources upon changing our ways. In political terms, this means that governments should be prepared, not only to tax junk food, but also to ask themselves seriously whether some of the larger fastfood outlets should be given a franchise to operate in Britain at all. We do not allow the open sale of heroin on the high street - yet fried chicken, and burgers, and chips soaked in salt and sugar arguably do far more damage to a far greater number of people, and yet are for sale on every station concourse.

And then again, we ought surely to accept that the NHS has a moral obligation to prioritise. I am 63 years old. I have had very little illness, so it is easy for me to speak - I know that. But I really believe that people my age should be placed lower down the pecking order than younger people, when it comes to choosing how much any hospital can actually afford.

The first raft of money should be reserved for the very young - sick babies and children. Thereafter, there should be money put aside for treating grave illnesses in the middle-aged - the early detection of cancer in the middle-aged is crucial, as we know, and Britain has lagged behind other developed nations in its care of treatable cancer patients.

When the time and money have been expended on these urgent categories of patient, then - and only then - should the hospital authorities see how much money there was in the kitty for performing operations on the elderly.

One can see that fitting gastric bands into the guts of the obese will reduce their danger of continuing to swell - though, as many sadly grotesque examples in America have shown, this is not always the case - real addicts of burgers and fries being prepared, even with excruciating pain, to go on guzzling unwholesome food even if it means literally bursting their guts.

But the moral responsibility does not rest entirely with the NHS or the taxpayer. It is patronising to the poor or the ill-educated to imagine that they can not see what is happening to their own bodies. Those of them who start to burst out of their clothes cannot be unaware of what is happening. Yes, there should be more help available - analagous to family-planning vans or narcotic therapy or counselling - to enable people to eat healthy food. We have all, to some extent, been tricked by the supermarkets into eating sugar, even when we thought we were doing no more than eating a slice of toast. But once we know that this is the case, we can surely - regardless of our income level or our standard of education - do something about it. You don't have to buy sliced bread.

Nobody forces you to slurp sweetened drinks. A cheap cut of meat at your local butcher - scrag end of lamb, say - boiled with pearl barley and some vegetables bought at your local market stall will be far cheaper than any pizza, and will last you for several days, if kept on the boil. You do not have to be posh or clever to realise this. So - by all means bind up a few guts in extreme cases. But for the most part, would we not all prefer public money to be spent on cancer care - and health education?

There is no single solution to the problem of obesity. We need a combination of solutions. Health education is the key - with children from an early age being taught that a love of **fizzy drinks** and junk food is not merely foolish but lethal. But then, again, at the other end of the chain, we should lose our fear of "nanny state". If we expect the state to pay for our health care, out of our taxes, then we should not merely tolerate, but demand a nanny state, which taxes sugar and outlaws those tremendously powerful moneyed chains - of supermarkets and fast-food joints who are, quite simply, the purveyors of poison.

Comment on AN Wilson's view at www.telegraph.co.uk/personalview

LOAD-DATE: July 12, 2014

LANGUAGE: ENGLISH

PUBLICATION-TYPE: Newspaper

JOURNAL-CODE: DTL

Copyright 2014 Telegraph Media Group Limited
All Rights Reserved

i-Independent Print Ltd

July 12, 2014
First Edition

£1bn junk food tax 'should be used to fight obesity'; HEALTH

BYLINE: James Cusick POLITICAL CORRESPONDENT

SECTION: NEWS; Pg. 6

LENGTH: 472 words

The Local Government Association (LGA) has called on the Treasury to divert £1bn from the annual £5bn it raises from VAT on junk foods, confectionery and unhealthy takeaways in order to provide local government authorities with the cash to tackle the causes of Britain's obesity crisis.

The LGA - which was given responsibility for improving public health last year - claims it can make the money "work harder" at preventing the rise of obesity and the related epidemic of type 2 diabetes.

The cost of tackling obesity for the National Health Service is estimated at £5bn. Although there has been a renewed focus on interventions, such as weight-loss surgery, the LGA says not enough is being done on early prevention and creating healthier lifestyles.

The NHS is spending £1m an hour on diabetes, a tenth of its annual budget. The LGA wants the Treasury to top up its current £2.8bn public health budget for England and Wales by diverting 20 per cent of the VAT raised from so-called "sin tax" foodstuffs, such as confectionery and takeaways.

An LGA spokesman said "radical action was needed and an obesity action fund would deliver extra money for councils already commissioning weight-management services, exercise referral schemes and extending the offer of reducedcost or free sport".

David Sparks, the chairman of the LGA, added: "By taking a slice of VAT we could help transform the lives and futures of obese and overweight people."

New draft guidelines from the National Institute of Health and Care Excellence (Nice) are aiming to reduce the debilitating complications of type 2 diabetes by expanding those eligible for weightloss surgery. Currently only those with life-threatening obesity qualify for surgery on the NHS. However, by lowering the qualification level, there are fears that the NHS may struggle to fund the additional cases.

The LGA says that although the new Nice guidelines may be necessary, the focus on obesity is in the wrong place.

In 2010, Denmark levied additional taxation for chocolate, ice-cream and **soft drinks** with a high **sugar** content. But in 2012 after a year of taxing foods containing saturated fat, the government announced it was abolishing the scheme.

The Treasury was contacted last night and asked for its reaction to the LGA's proposal. No comment was received.

An expansion of weight loss surgery in England is being proposed by the national Institute of Health and Care Excellence (NICE) to tackle an epidemic of type 2 diabetes.

In numbers

£5bn The current estimated cost to the National Health Service of tackling obesity.

10% Proportion of its budget the NHS spends on treating diabetes..

3.2m The number of people in the UK with diabetes as of February this year.

163,000 The number of new cases of diabetes diagnosed in 2013.

40% Percentage of people in the UK who have a normal Body Mass Index.

LOAD-DATE: July 11, 2014

LANGUAGE: ENGLISH

PUBLICATION-TYPE: Newspaper

JOURNAL-CODE: III

Copyright 2014 Independent Print Limited
All Rights Reserved

theguardian
Guardian.com.

July 11, 2014 Friday

Drink (tap) water - not sports drinks, unless you really are a football star

BYLINE: Sarah Boseleytheguardian.com

LENGTH: 419 words

ABSTRACT

Sports drinks are fashionable and considered healthy among teenagers, who drink them while watching TV, but they contain useless added sugar. Water is the better option

FULL TEXT

Sports drinks - if they have a justification at all - are for people playing serious sport. According to the European Food Safety Authority (EFSA), they are useful only for "active individuals performing endurance exercise". That is not even a half-hearted kick-about of a ball in a park, let alone the spectator sport of watching the World Cup from the sofa.

But according to a survey, a third of teenagers drink Lucozade, Powerade or other brands while being completely unphysical - at the cinema, watching TV or playing computer games. The survey was of only just over 1000 young people aged 13 to 17, but it fits with what we know - that sports drinks are marketed to young people and widely drunk by them on all sorts of non-sporting occasions. Only 16% of the young people said they consumed sports drinks during or after intense physical exercise.

Sports drinks contain 16 to 18 grams of sugar in every 500ml, which is at least four teaspoons, as well as salt. Coca Cola, Pepsi, Sprite and the rest of the sugar-sweetened **fizzy drinks** are still worse - there are 53 grams of sugar in the same sized bottle of Coca Cola, for instance, or 13 teaspoons. Either is a really bad idea if we are to take thelatest draft recommendation from SACN, the government's scientific advisory committee on nutrition, seriously. They said we should aim to restrict our sugar intake to around 5 grams a day or less.

This survey is a shot across the bows of the sports drinks industry by the Natural Hydration Council, which is funded by the major bottled water companies. They are: Brecon Carreg Natural Mineral Water, Danone Waters (UK & Ireland) Ltd, Highland Spring Group, Iceni Waters, Nestlé Waters UK, Ty Nant, Water Brands Group and Wenlock Spring. They make up 50% of the UK bottled water industry.

So there is a sense in which this is a skirmish in the bottled drinks wars, but they have a point - water is far better for you. And although they promote bottled water on their website, they are happy, when asked, to say that tap water will do just as well.

It has to be a good thing that anybody is agitating for drinking water, even with vested interests behind them. Surprisingly, theEatwell plate promoted by NHS Choices does not specify water of any description as the first choice to drink, although I understand that is under review.

LOAD-DATE: July 10, 2014

LANGUAGE: ENGLISH

PUBLICATION-TYPE: Newspaper

Copyright 2014 Guardian News and Media Limited or its affiliated companies
All Rights Reserved
Registered in England and Wales. No. 908396
Registered office: PO Box 68164, Kings Place, 90 York Way, London N1P 2AP

i-Independent Print Ltd

July 11, 2014
First Edition

Teens warned over high-sugar sports drinks; HEALTH

BYLINE: Ian Johnston

SECTION: NEWS; Pg. 21

LENGTH: 318 words

More than a quarter of teenagers consume sports drinks designed for strenuous exercise while watching TV and playing computer games, according to a new study.

The National Hydration Council (NHC) found that a similar number of young people thought the drinks were healthy enough to be consumed at any time - even though they have large amounts of sugar and salt.

They are designed for use during intense exercise lasting more than an hour.

A survey of 1,000 13 to 17-year-olds found 27 per cent of teenagers drank sports drinks at the cinema, while watching TV or gaming; 25 per cent per cent thought they were healthy enough to be drunk anytime; and just 16 per cent of teenagers used sports drinks for the reason they were designed.

Nearly half said they often feel thirsty and 40 per cent said they were not sure what their body needs to keep hydrated. Professor Paul Gately, an expert on exercise and obesity at Leeds Metropolitan University and an NHC advisor, said: "Sports drinks have a clear purpose for athletes participating in high-intensity exercise - otherwise people are just consuming water, salt and on average 16 grams of sugar in each 500ml bottle.

"Teenagers often perceive these drinks to be more healthy than other soft drinks, when they really only have a purpose to help those who are being vigorously active.

"Knowing which drinks to choose for the amount of physical or recreational exercise you do is important. Drinking water will keep most people well hydrated."

Kinvara Carey, NHC general manager, said the survey's findings showed there was "still some confusion about the role of sports drinks". "The recently updated school food standards establish water as the first choice to hydrate with," she added.

Women should drink 1.4l of fluid and men should have 2l every day, says the NHC. Take "plenty" of water, have milk "regularly" and have "small amounts" of **soft drinks** containing **sugar**.

LOAD-DATE: July 10, 2014

LANGUAGE: ENGLISH

PUBLICATION-TYPE: Newspaper

JOURNAL-CODE: III

Copyright 2014 Independent Print Limited
All Rights Reserved

The INDEPENDENT
Independent.co.uk

July 11, 2014 Friday 8:07 PM GMT

Market Report: The FTSE 100's worst week since early March

BYLINE: Oscar Williams-Grut

SECTION: SHAREWATCH

LENGTH: 351 words

The final bell rang out the FTSE 100's worst week since early March, despite turning higher in its final session.

Reassuring words from Portugal's troubled Banco Espirito Santo sparked a relief rally, with the Footsie climbing 17.80 points to 6,690.17. But it was still 188.35 points, or 2.74 per cent, lower than it had been a week earlier, when the blue-chip index looked as if it could reach record levels.

Jasper Lawler, an analyst at CMC Markets, said: "The Footsie's been banging on the door of 6,900 but it's notable that it can't get through it. Part of it is the way it's made up - miners are constantly struggling because of weak performance out of China and the financials have been hit."

The Greek bottling firm Coca-Cola Hellenic climbed 39p to 1,351p thanks to an upgrade from Goldman Sachs. The investment bank thinks falling **sugar** prices are a boon for the **soft drinks** industry, and the world's second largest bottler of Coca-Cola is well placed to benefit.

Traders piled into gold miners on Thursday as the eurozone crisis threatened to rear its head again, but the calming of the markets yesterday meant a sell-off for the precious metal producers. Randgold Resources fell 115p to 5,120p, Fresnillo lost 17p to 928p, African Barrick Gold slipped 7.2p to 225.3p and Centamin was 2.3p lighter at 67p.

The chemical supplier Synthomer slipped 6.5p to 212p on the FTSE 250, as the maker of products from paint emulsion to mattress foam warned that the strong pound and a slowdown at its Asian business mean profits are likely to be flat this year.

Kofax fell 50.5p to 423p after the small-cap software provider released unaudited full-year results below expectations. The chief executive, Reynolds Bish, said he was "disappointed", with the US company's performance.

GCM Resources leapt 19.25p to 38.75p on AIM as rumours circulated that the Bangladeshi coal operator could be close to getting a mining permit at long last.

Alba Mineral Resources ended the day 0.35p better at 0.62p after snapping up a 5 per cent stake in Horse Hill Development, the operator of the UK Weald oil basin south of London.

LOAD-DATE: July 11, 2014

LANGUAGE: ENGLISH

PUBLICATION-TYPE: Newspaper; Web Publication

JOURNAL-CODE: WEBI

Copyright 2014 Independent Digital News and Media Limited
All Rights Reserved

MailOnline

July 11, 2014 Friday 10:02 PM GMT

MARKET REPORT: Bottling group Coca-Cola HBC adds some fizz to Footsie as Goldman Sachs upgrades rating driven by falls sugar prices

BYLINE: JONATHON HOPKINS

SECTION: MARKETS

LENGTH: 823 words

As stock markets stabilised following sharp sell-offs in the previous session, bottling group Coca-Cola HBC (CCH) added some fizz to the Footsie as a broker upgraded its rating driven by falls in **sugar** prices, a big expense for **soft drinks** firms.

Goldman Sachs predicts a fall in European sugar prices will happen sooner than expected, with the slide set to extend to over 30 per cent over the next two years.

The European Union agreed last year to dismantle the bloc's system of sugar production quotas from October 2017, as part of wider reforms to its common agricultural policy.

'We believe lower sugar prices are here to stay as companies become more competitive up to and beyond the end of quotas in 2017,' Goldman analysts said in a note to clients.

The broker noted that CCH shares have underperformed their European consumer staple peers by 20pc over the last three months, largely due to concerns about the situations in big markets like Russia, Ukraine and Nigeria, and currency exchange rate factors.

But Goldman said the extent of the CCH share price weakness leads it to think that such factors are largely priced in to the stock. The broker set a new 12-month price target for CCH of 1550p, implying 18 per cent upside potential. CCH's blue-chip shares notched up a 39p gain at 1351p.

Goldman stayed neutral on mid cap soft drinks peer Britvic but increased its 12-month price target slightly to 778p from 764p. Britvic shares were down 2.5p at 698.5p.

Aside from the fizzy pop boost, the Footsie also enjoyed a rush from gains by cigarette makers on news that US firms Reynolds and Lorillard plan to merge. Imperial Tobacco was the second top blue-chip gainer, up 81p at 2740p, while British American Tobacco added 7p at 3528p. Signs of deal activity elsewhere in the market also helped support the blue chips. Consumer goods giant Unilever added 24p at 2622p after selling its Slim-Fast brand to private equity firm Kainos Capital.

The FTSE 100 index closed 17.8 points higher at 6690.17, albeit dragged back from its best levels as Wall Street started cautiously and with the index recording a drop of over 2.5 per cent for the week, taking it back to its lowest point since April. The FTSE 250 index ended 11.55 points higher at 15451.42.

Among the fallers, banks were lower with Lloyds Banking Group down 0.44p to 72.67p and Royal Bank of Scotland off 0.8p to 314.1p as financial stocks remained subdued over questions about the stability of Portugal's Banco Espirito Santo The Portuguese bank, which saw its shares suspended by the country's regulator on Thursday, tried to reassure investors by saying it had sufficient reserves to absorb losses but concerns over its survival continue.

Mining stocks were also under pressure as the banking sector's troubles persuaded investors to steer clear of riskier stocks, with Randgold Resources a big blue chip faller, down 115p to 5120p.

Housebuilder Persimmon also fell back, losing 12p to 1242p as data showed British construction output fell in May, suggesting the sector has lost momentum in the second quarter of the year.

Among the small caps, software firm Kofax dropped 50.5p to 423p as it said it expects to report results below its previous guidance for the year ended June 30, due to delays in software licence revenues.

But on the up, Ilika added 5.5p at 67.5p on news it has taken a step forward in its plans to launch a 'world-first' solid-state battery.

The group said it has secured a contract with an existing European customer for solid-state batteries in wireless sensor network applications which will take its revenue level for the current financial year to £0.6m.

In the resources sector, Alba Minerals soared nearly 130 per cent higher, up 0.35p to 0.62p after signing a deal with Horse Hill Developments to invest in a UK onshore oil and gas project.

The well, which is scheduled to be completed by the end of August 2014, is planned to test targets in the proven productive Horse Hill prospect in the Weald Basin situated in Surrey, England.

Regency Mines, which has a 14.9 per cent stake in Alba and has agreed to acquire a 5 per cent stake in Horse Hill Developments, gained nearly 4 per cent, up 0.01p to 0.26p.

And Tertiary Minerals gained 0.38p at 6.38p after the fluorspar explorer raised £420,000 via a placing of new shares with a small number of institutional investors at 5.75p each.

Ceres Power got a boost as intellectual property developer IP Group raised its stake in the firm to 23.2 per cent following a round of funding with new and existing institutional shareholders.

Ceres, which provides low cost fuel cell technology for use in small scale power generation and other applications said it would raise around £20m via a share placing in which IP Group (up 1.5p to 195p) will subscribe for 47.06m shares, representing around a fifth of the total offering.

Ceres Power shares slipped 0.34p lower to 9.29p with the placing priced at 8.5p a share.

LOAD-DATE: July 11, 2014

LANGUAGE: ENGLISH

PUBLICATION-TYPE: Web Publication

JOURNAL-CODE: WEBDM

Copyright 2014 Associated Newspapers Ltd.
All Rights Reserved132 of 205 DOCUMENTS

The Telegraph
telegraph.co.uk

July 11, 2014 Friday 8:27 PM GMT

How to put the squeeze on gastric bands; Education is the key in the fight against obesity - and perhaps, for once, nanny does know best

BYLINE: By AN WIlson

LENGTH: 938 words

We live in strange times. In my youth we worried ineffectually about the starving millions. Now, in Britain, two thirds of the population are deemed to be overweight, and a quarter actually obese. No wonder that Nice, the health watchdog, says we must offer what is called weight-loss surgery - gastric bands or gastric bypass - on the NHS to those whose obesity appears to have swollen out of control.

In the past, my reaction to this would have been simple. Nice must be mad! All that is required, I would have believed, would be to tell these fatties to stop guzzling burgers and chips. But of course, such a reaction is woefully simplistic. One of the reasons why so many people are fat is that the food on offer in all supermarkets - as well as the fast-food chains - is filled with sugar. There are one and a half teaspoonfuls of sugar in every slice of sliced bread. Every dollop of ketchup contains a cube of sugar. Go out to Pizza Express for a Margherita and you will be consuming three cubes of sugar. Those who eat this muck are not necessarily any greedier than those who eat lentils, raw carrots and yoghurt. But by failing to read the label, they are adding to their waistline, and hugely increasing the likelihood that they will develop type 2 diabetes - with the risk of blindness, strokes, heart disease and years of immobility as they sit, like beached whales, in their specially constructed giant armchairs.

It is in order to help people caught in this spiral that Nice has decided that it would be cheaper to fix gastric bands - an operation that costs an average of £6,000 - than to live with the consequences of millions of diabetic, obese, blind people who are scarcely able to waddle to the lavatory, and who will need round-the-clock expensive care for the rest of their depressed lives.

You can see the logic of it, but it sounds like defeatism. Surely we should be concentrating our resources upon changing our ways. In political terms, this means that governments should be prepared, not only to tax junk food, but also to ask themselves seriously whether some of the larger fast-food outlets should be given a franchise to operate in Britain at all. We do not allow the open sale of heroin on the high street - yet fried chicken, and burgers, and chips soaked in salt and sugar arguably do far more damage to a far greater number of people, and yet are for sale on every station concourse.

And then again, we ought surely to accept that the NHS has a moral obligation to prioritise. I am 63 years old. I have had very little illness, so it is easy for me to speak - I know that. But I really believe that people my age should be placed lower down the pecking order than younger people, when it comes to choosing how much any hospital can actually afford.

The first raft of money should be reserved for the very young - sick babies and children. Thereafter, there should be money put aside for treating grave illnesses in the middle-aged - the early detection of cancer in the middle-aged is crucial, as we know, and Britain has lagged behind other developed nations in its care of treatable cancer patients.

When the time and money have been expended on these urgent categories of patient, then - and only then - should the hospital authorities see how much money there was in the kitty for performing operations on the elderly.

One can see that fitting gastric bands into the guts of the obese will reduce their danger of continuing to swell - though, as many sadly grotesque examples in America have shown, this is not always the case - real addicts of burgers and fries being prepared, even with excruciating pain, to go on guzzling unwholesome food even if it means literally bursting their guts.

But the moral responsibility does not rest entirely with the NHS or the taxpayer. It is patronising to the poor or the ill-educated to imagine that they can not see what is happening to their own bodies. Those of them who start to burst out of their clothes cannot be unaware of what is happening. Yes, there should be more help available - analagous to family-planning vans or narcotic therapy or counselling - to enable people to eat healthy food. We have all, to some extent, been tricked by the supermarkets into eating sugar, even when we thought we were doing no more than eating a slice of toast. But once we know that this is the case, we can surely - regardless of our income level or our standard of education - do something about it. You don't have to buy sliced bread. Nobody forces you to slurp sweetened drinks. A cheap cut of meat at your local butcher - scrag end of lamb, say - boiled with pearl barley and some vegetables bought at your local market stall will be far cheaper than any pizza, and will last you for several days, if kept on the boil. You do not have to be posh or clever to realise this. So - by all means bind up a few guts in extreme cases. But for the most part, would we not all prefer public money to be spent on cancer care - and health education?

There is no single solution to the problem of obesity. We need a combination of solutions. Health education is the key - with children from an early age being taught that a love of **fizzy drinks** and junk food is not merely foolish but lethal. But then, again, at the other end of the chain, we should lose our fear of "nanny state". If we expect the state to pay for our health care, out of our taxes, then we should not merely tolerate, but demand a nanny state, which taxes sugar and outlaws those tremendously powerful moneyed chains - of supermarkets and fast-food joints who are, quite simply, the purveyors of poison.

LOAD-DATE: July 11, 2014

LANGUAGE: ENGLISH

PUBLICATION-TYPE: Newspaper; Web Publication

JOURNAL-CODE: WEBDTNS

Copyright 2014 Telegraph Media Group Limited
All Rights Reserved

The Telegraph
telegraph.co.uk

July 11, 2014 Friday 6:16 PM GMT

Market report: Sugar highs boost Coca-Cola Hellenic; A note from Goldman Sachs upgrading the drinks bottle maker put fizz in the shares, while the FTSE sideways as Portugal banking fears ease

BYLINE: By Rebecca Clancy

SECTION: FINANCE

LENGTH: 408 words

Investors were enjoying a sugar rush yesterday, after Goldman Sachs upgraded the company that makes the bottles for **fizzy drinks** giant Coca-Cola.

Coca-Cola Hellenic (CCH) rose 39, or 3pc, to £13.51 after Goldman raised the drinks bottle maker from 'neutral' to 'buy' as part of a note in which it said sugar priced would fall more than 30pc over the next two years.

"We believe lower sugar prices are here to stay as companies become more competitive up to and beyond the end of quotas in 2017," Goldman said.

CCH, which used to be Greece's largest company until it moved its headquarters to Switzerland and listed in London in 2012, will not be the only winner from a potential sugar crash.

Britvic, the drinks company behind Robinsons and Tango, rose more than 1pc during yesterday's trading following the note, but couldn't hold on to the gains and closed down 2.5, or 0.4pc, to 698.5p.

"While the retail environment remains tough, we believe CCH and Britvic will be able to hold onto at least some of the benefit from lower costs," Goldman added.

At the other end of the main index was Randgold Resources. The gold miner had risen nearly 3pc on both Wednesday and Thursday, going against the grain of the market as investors looked for a safe haven as the threat of a eurozone crisis reared its head. But as markets stabilised yesterday it fell back and finished the day down 115, or 2.2pc, to £51.20.

Overall the FTSE 100 spent yesterday trading sideways and closed up 4.71, or 0.1pc, to 6,677.08.

"Investors were recuperating from Thursday's Portuguese reminder of the risks still inherent in the continent's banking system," said Jasper Lawlor, market analyst at CMC Markets.

It was a similar picture on the FTSE 250, which finished the day up 14.33, or 0.1pc, to 16,725.68.

On the mid-cap index, something was off at Dairy Crest, which dropped 15.3 to 440.1p, after analysts at Societe General cut the rating on one of the big three milk suppliers in the country from 'hold' to 'sell'.

"The removal of production quotas for EU milk in 2015 is likely to have negative consequences for Dairy Crest's key profit engine (UK cheese) in our view," the note from SocGen said.

"We expect that a significant increase in low-priced, private label cheese would negatively impact its Cathedral City branded cheese margins as it is forced to further elevate promotional intensity to maintain volume share especially in the current challenging UK retail backdrop."

LOAD-DATE: July 11, 2014

LANGUAGE: ENGLISH

PUBLICATION-TYPE: Newspaper; Web Publication

JOURNAL-CODE: WEBDTNS

Copyright 2014 Telegraph Media Group Limited
All Rights Reserved

The Independent
Independent.co.uk

July 8, 2014 Tuesday 10:38 AM GMT

The sweetest quiz on Earth; Experts say we need to drastically reduce the amount of sugar in our daily diets. Just how much are we hoovering up?

SECTION: LIFE AND STYLE

LENGTH: 212 words

It's official, we're sweet enough. Experts have announced that we should be halving our sugar intake.

In order to tackle Britain's obesity epidemic and protect children from diabetes, tooth decay and heart disease, Government health advisors have said free sugars - those found in sweetened drinks, fruit juice, confectionery, and added to many processed foods - should make up just five per cent of our daily energy intake. And that's far less than most of us are consuming at the moment - many people already exceed the current 10 per cent limit.

But many of us don't realise just how much **sugar** is in the foods we eat. Under the new guidelines, a can of a typical **soft drink** (which contains the equivalent of six to seven teaspoons of **sugar)** would make up nearly the entire healthy allowance for the day. And that's just one drink.

So do you know whether a Krispy Kreme original glazed doughnut has more sugar than a bowl of Coco Pops?

Take our quiz below. We won't sugar-coat the truth.

Quizzes by Quibblo.com

When viewing the results, the answers in green show which is the correct answer, red is incorrect, and the percentage shows how many respondents selected that answer

Measurements are based purely on sugar content, and do not take other nutritional value into account

LOAD-DATE: July 8, 2014

LANGUAGE: ENGLISH

PUBLICATION-TYPE: Newspaper; Web Publication

JOURNAL-CODE: WEBI

Copyright 2014 Independent Digital News and Media Limited
All Rights Reserved

MailOnline

July 8, 2014 Tuesday 4:16 PM GMT

The great tax sugar rush: San Francisco could become the first U.S. city to tax sugary drinks

BYLINE: JILL REILLY

SECTION: NEWS

LENGTH: 338 words

- Could impose penny-per-ounce tax on soda and other sweetened beverages
- Measure could also include energy drinks - not diet sodas and juice

The San Francisco Bay Area city of Berkeley could become the first U.S. city to tax **sugary drinks**.

The Berkeley City Council voted last week to put a measure on the ballot that would impose a penny-per-ounce tax on soda and other **sugar**-sweetened **beverages** such as energy drinks.

Diet sodas and juice would be exempted.

San Francisco is also pursuing a soda tax ballot measure.

Scroll down for video

'When we pass this measure in November, Berkeley will be the first in the country where such a measure has been passed,' said Vicki Alexander, a co-chairwoman of a local campaign in Berkeley to put the measure on the ballot.

'We are very excited to see that day happen.'

Public health advocates across the country have clamored for ways to reduce consumption of **sugary drinks** and junk food, but lawmakers and voters have generally opposed enacting taxes or other regulations.

Former New York City Mayor Michael Bloomberg's plan to limit the sale of large **sugary drinks** was rejected on June 26 by the state's highest court.

A California bill to require **sugary soft drinks** to carry labels warning of obesity, diabetes and tooth decay died in the California Legislature on June 17.

Two California cities, Richmond and El Monte, failed two years ago in their attempts to become the first in the country to impose taxes of a penny per ounce on businesses that sell **sugary drinks**.

Revenues from Berkeley's tax, if it passes, would go toward the city's general fund.

'It's disingenuous because there's nothing in this measure that's going to education.

'This goes right into the general account. It's a money grab,' said Ted Mundorff, CEO of Landmark Theatres, which has movie theaters in Berkeley.

A Berkeley vending machine executive said her company would be forced to pass the tax on to consumers in order to stay in business.

A city survey of 500 likely voters showed majority support for the tax in April.

LOAD-DATE: July 8, 2014

LANGUAGE: ENGLISH

PUBLICATION-TYPE: Web Publication

JOURNAL-CODE: WEBDM

Copyright 2014 Associated Newspapers Ltd.
All Rights Reserved

MailOnline

July 8, 2014 Tuesday 3:52 PM GMT

Soda tax's last stand? Bay Area preps for showdown

BYLINE: ASSOCIATED PRESS

SECTION: AP

LENGTH: 1076 words

WASHINGTON (AP) - If two of the most progressive U.S. cities don't pass a tax on **sugary drinks**, will the idea finally fizzle out?

Sugary drinks have been under fire for years, with many blaming them for rising rates of obesity and chronic diseases. Yet efforts to curb consumption by imposing taxes and other measures have failed, in part because the beverage industry has spent millions to defeat the efforts.

Now, the question of whether a bottle of Dr Pepper with 64 grams of sugar should be treated like a pack of cigarettes is being considered in San Francisco and Berkeley, with the two California cities aiming to become the country's first to pass per-ounce taxes on **sugary drinks**.

The stakes are high, especially given the Bay Area's reputation for liberal politics. If approved, Coca-Cola, PepsiCo and other companies fear it could galvanize health advocates elsewhere. If defeated, the idea of a soda tax could be dead.

"The industry is really motivated to beat us here. If they can beat us in San Francisco and Berkeley, nobody is going to take them on," said Larry Tramutola, the political consultant handling the campaign in support of the tax in Berkeley.

The odds aren't in favor of taxes. Since 2009, about 30 special taxes on **sugary drinks** have been introduced around the country. Few have gained traction and none have prevailed. Chris Gindlesperger, a spokesman for the American Beverage Association, the lobbying group for Coke and Pepsi, says the failures show people don't support the idea.

Others say the industry uses unfair tactics to defeat measures, such as setting up groups with names like "Citizens Against Beverage Taxes," which sound like they are community-driven but aren't. They are nevertheless influential in shaping people's attitudes.

In San Francisco and Berkeley, supporters of the tax say they're better organized to battle such tactics. They're hitting the streets to educate voters and plan to run TV ads, work phone banks and mail fliers.

"In other places, bless their hearts, but they were ill-prepared for what was coming at them" said Maggie Muir, a consultant who was hired by San Francisco lawmakers to lead the political committee in support of the soda tax.

The San Francisco proposal is for a two-cent-per-ounce tax on **sugary drinks** and would not apply to milk or natural fruit juices without any added sugars. It needs a two-thirds vote to pass in the November election.

The tax in Berkeley is for a penny per ounce and needs a simple majority of the vote.

It's a high bar either way. Just two years ago, similar measures were soundly defeated in other California cities. Part of the reason is that even some who think drinking **sugary drinks** can be harmful don't believe taxing is the solution.

Barbara Cassidy, 50, fears a soda tax could lead to similar taxes on other foods.

"It's a slippery slope," said Cassidy, a lifelong San Francisco resident.

SUGARY DRINKS UNDER FIRE

Overall, Americans have been cutting back on soda for years, with sales volume down about 13 percent over the past decade, according to the industry tracker **Beverage** Digest. But other **sugary beverages** with healthier images have climbed; sports drinks, for instance, are up about 35 percent.

Taxing a product to discourage use has proven effective with cigarettes. According to the American Cancer Society, there's a 4 percent decline in overall smoking rates for every 10 percent hike in cost. Still, at least one study has questioned how effective a tax on **sugary drinks** could be since people might just spend their money on other high-calorie foods.

France and other countries have imposed taxes on **sugary drinks** in the meantime. Mexico, which has one of the highest obesity and soda consumption rates in the world, passed a tax on **sugary drinks** last year.

Back in the U.S., critics have persisted. California lawmakers considered a measure that would have slapped a warning label on sodas before it was ultimately defeated last month.

In New York City, former mayor Michael Bloomberg pushed to cap the size of **sugary drinks** sold in restaurants and other venues at 16 ounces. The move was shot down after legal challenges spearheaded by the beverage industry, with the state's highest court ruling last month that the city's Board of Health overstepped its authorities by imposing the cap.

The beverage industry also waged a heavy marketing campaign to win over the public. Some ads featured the Statue of Liberty holding up a giant soda instead of a torch.

"Overall, we lost the PR war," said Thomas Farley, the city's health commissioner at the time.

If given another chance, Farley said he would frame the cap as government giving customers the option to get a drink in a more reasonable size. Right now, a small drink in many places is 16 ounces. At McDonald's, that translates to 39 grams of sugar and 140 calories.

A DIFFERENT KIND OF FIGHT

In San Francisco, a small group dressed in red t-shirts that say "Coalition for an Affordable City" has been posting signs in opposition to the tax on **sugary drinks**.

The coalition was created by a public affairs firm hired by the American Beverage Association. It bears close resemblance to groups the industry's lobbying association has set up to fight soda measures in other cities. Gindlesperger, the spokesman for the ABA, said the name isn't intended to mislead people about the group's origin.

"We've had to build coalitions in places with our partners like retailers and restaurants and grocers and others to voice our opposition," he said.

Scott Wiener, one of the city supervisors who introduced the tax in San Francisco, thinks otherwise.

"They come in with this fake organization, trying to convince our residents that this coalition is really looking out for their best interests," he said.

Before introducing the tax, Wiener worked with his political consultants, Erwin & Muir, which established a group called "Choose Health SF" and enlisted the support of organizations including the California Nurses Association and parent groups.

Like the beverage industry, Choose Health SF is already in campaign mode, hitting farmers markets and neighborhood festivals to recruit supporters. It also set up a website and created a satirical account on Twitter called "Big Soda Crybaby" to mock the industry's talking points.

Muir said the idea is to "inoculate and educate" the public against the coming campaigning blitz by the beverage industry.

______

Follow Candice Choi at www.twitter.com/candicechoi

LOAD-DATE: July 8, 2014

LANGUAGE: ENGLISH

PUBLICATION-TYPE: Web Publication

JOURNAL-CODE: WEBDM

Copyright 2014 Associated Newspapers Ltd.
All Rights Reserved

JOURNAL-CODE: SUN

Copyright 2014 NEWS GROUP NEWSPAPERS LTD
All Rights Reserved

The Sun (England)

July 8, 2014 Tuesday
Edition 1;
Scotland

10 reasons why fruit juice IS good for you; JUICY FACTS BEHIND YOUR FAVOURITE DRINKS

SECTION: ME;FEATURES; Pg. 6,7

LENGTH: 1481 words

FRUIT juices went from health food to junk food after a headteacher banned them from lunchboxes.

Kids were told to stick to water at Valence Primary School, Dagenham, Essex, amid fears over the sugar and calories in juices.

But fruit drinks CAN be a healthy part of your "five a day" and balanced diet if you do not drink too much.

Here, Sun nutritionist AMANDA URSELL shares ten juicy facts and her verdicts on favourite drinks, with health marks out of five.

! A RECOMMENDED 150ml serving of 100 per cent pure orange juice has just 54 calories and 13g of sugar. Compare this to a 150g medium-sized orange with 40 calories and 13g of sugar and it's not the calorific disaster you may have believed. If you glug down a whole litre of fruit juice you get 360 calories which is the same as you find in a Starbucks grande coffee mocha. Very few people can afford to drink this many calories every day whatever their source, so stick to 150ml to keep energy intakes under control.

$ Fruit juices don't give you a quick sugarrush after drinking. This is because they almost all have a low glycaemic index. The glycaemic index measures the speed at which blood sugar levels rise after eating or drinking a carbohydrate compared with pure glucose. A GI score of over 70 is high GI (such as croissants), medium GI is from 56-69 (such as digestive biscuits) and low GI is 55 or less. Apple juice has a GI of 44, orange juice is 50, pineapple juice is 46 and grapefruit juice is 48.

% A 150ml glass of fruit juice counts as one of your "five a day" of vegetables and fruit. A smoothie that gives you 150ml of juice plus 80g of fruit pulp counts as two towards your five a day.

& Fruit juices do not lose all their nutrients during processing. A 150ml glass of orange juice has 47mg of vitamin C. This is 7mg more than our daily target for this vital vitamin. A 150g orange gives us 81mg.

(Fruit juice does not make you fat. In fact, results from our National Dietary and Nutritional Survey 2008-2010 revealed that fruit juice consumers have a lower body weight, body mass index and waist circumference than people who didn't drink juice.

) Adults who guzzled fruit juice were also found to eat more portions of fruit and vegetables (5.1 portions a day) compared to non-juice consumers (3.7 portions daily). The same was true for children with juice drinkers eating 3.5 servings of fruit and veg a day and non-juice drinkers having a serving less with just 2.5.

* Drinking fruit juice doesn't mean that you automatically have a bad, junkfilled diet. The same survey revealed that intakes of vitamins C (needed for goodquality skin) and folate (for healthy nerves) along with minerals like iron (for energy), zinc (for immunity) and selenium (a vital component of critical antioxidants) are higher in people who drink fruit juice regularly. Not all of these nutrients, including iron and zinc, are found in juices, which indicates that if anything, the general diets of juice drinkers may be "better" overall than non-juice drinkers. + It doesn't wreck your teeth if you play by the rules and restrict drinking fruit juice to 150ml at meal times. Do this and it won't erode your enamel or by itself cause tooth decay. If you have juices between meals - just as if you ate dried fruits and sweets between meals - it will almost certainly up your chances of tooth damage.

, Fruit juice may help to lower blood pressure. It is packed with the mineral potassium and also contains a special supernutrient called hesperidin and research has shown that a small daily drink of citrus juice can actually help to lower diastolic blood pressure.

- "Fruit juice drinks" are not the same as "100 per cent fruit juice". The former contain fruit juice concentrates plus added water and added sugar and sometime artificial sweeteners too so a 150ml serving will typically have 18g of sugar (four and a half teaspoons worth) and fewer vitamins and minerals than its "real" juice counterpart.

ASDA EXOTIC JUICE Per 100ml: 48 cals, 11.3g sugar AMANDA'S VERDICT: Higher in sugars than 100 per cent fruit juice, this does at least contain half juice and half water, a higher proportion than many other juice drinks. 2 out of 5 ASDA CHOSEN BY KIDS APPLE & MANGO JUICE Per 100ml: 6 cals, 1.1g sugar AMANDA'S VERDICT: When judged on the calorie and sugar front, this juice drink is a better choice. But if you don't like the idea of artificial sweeteners, you won't be keen on this one. 4 SUN EXOTIC CITRUS TWIST EXOTIC JUICE Per 100ml: 48 cals, 11.5g sugar AMANDA'S VERDICT: A mix of less than a fifth fruit juice plus water, sugar and flavourings. Added vitamin C gives it an undeserved healthy image. 2 CAPRI-SUN Per 100ml: 43.9 cals, 10.4g sugar AMANDA'S VERDICT: Although this has a higher proportion of juice to water, it is still way less than 100 per cent and needs flavourings and added sugar. 2 ASDA APPLE & MANGO JUICE DRINK Per 100ml: 46 cals, 11g sugar AMANDA'S VERDICT: This is a mix of fruit juice, water, sugar and flavourings. Children and adults would be better getting their vitamin C from real fruit. 2 ASDA LEMON & LIME JUICE DRINK Per 100ml: 46 cals, 11.4g sugar AMANDA'S VERDICT: Another example of a **sugary drink**, which provides little goodness or nutritional value from the small proportion of real fruit juice. 2 V8 VEGETABLE JUICE Per 100ml: 20 cals, 3.2g sugar AMANDA'S VERDICT: A better choice on the calorie front and with no added sugar, the only downside is that this has added salt - an unexpected contribution towards your 6g max per day. 3 TROPICANA ORANGE JUICE Per 100ml: 48 cals, 10g sugar AMANDA'S VERDICT: A good all-rounder, complete with a range of vitamins from C to the B vitamin folic acid, plus a good supply of flavonoids. 4 SUNSWEET PRUNE JUICE Per 100ml: 62 cals, 8.5g sugar AMANDA'S VERDICT: Prune juice is naturally higher in calories than orange or apple, so stick with the 150ml message on portions. Prunes contain sugar alcohols and speed up bowel movement so keep you regular. 4 WELCH'S PURPLE GRAPE JUICE Per 100ml: 68 cals, 16.5g sugar AMANDA'S VERDICT: Grape juice is higher in sugars than citrus juices but also rich in antioxidant flavonoids, which may be useful for heart health. 4 PRINCES 100 PER CENT PURE APPLE JUICE Per 100ml: 44 cals, 10.4g sugar AMANDA'S VERDICT: You would be better off with a cloudy apple juice, which will give you some fibre and also is higher in supernutrients. 3 SAINSBURY'S RED GRAPE JUICE Per 100ml: 68 cals, 16.3g sugar AMANDA'S VERDICT: Red grape juices have more antioxidants than white grape juice. But grape juice is higher in sugar than other fruit juices so stick with a 100ml glass to keep your intake down. 4 SPRING VALLEY MANGO JUICE Per 100ml: 43 cals, 10.5g sugar AMANDA'S VERDICT: Another fruit juice drink that sounds healthier than it is. With 25 per cent fruit juice, why not save your money and get the real thing with a lot more nutrients? 2 SAINSBURY'S FRUIT COCKTAIL JUICE, REDUCED SUGAR Per 100ml: 29 cals, 6.2g sugar AMANDA'S VERDICT: With fruit juices, look for reduced-sugar versions, which cut sugars from 10g per 100g to more like 6g. 4 TESCO CRANBERRY & RASPBERRY JUICE DRINK Per 100ml: 49 cals, 11.8g sugar AMANDA'S VERDICT: You would be better off with a low-sugar, 100 per cent cranberry juice. Having a daily glass of this may help to reduce the risk of cystitis. 2 COPELLA APPLE JUICE Per 100ml: 46 cals, 10.3g sugar AMANDA'S VERDICT: A good cloudy apple juice with around 0.7g of fibre per 100ml and more antioxidants than clear apple juice.

You still need to stick with 150ml a day though.

4 ! Unlike milk or soup, fruit juices do not appear to have any effect on helping you to feel fuller after you have drunk them.

If you are watching your weight and want to feel full, a glass of skimmed milk is a better choice. $ If you drink juices between meals, it will increase your risk of tooth erosion and possibly tooth decay as well. This is because the acids and natural sugars in fruit juices are a perfect combination for raising acidity and encouraging the activity of bacteria. % Whichever way you look at it, you get more fibre if you eat the whole fruit rather than drinking juice made from it.

A 150ml glass of apple juice has no fibre, for example, while a 100g apple has 1.8g.

A 100g orange has 1.7g of fibre while a 150ml glass of juice, even with bits, still only has 0.15g. & If you are thirsty and turn to fruit juice to solve the problem, then you could find yourself gulping down a 300ml serving without realising.

Doing this means you not only consume 135 calories but are also taking in 30g of sugars. This is unnecessary when a glass of water will do the trick. (It is easy to be fooled into thinking that "juice drinks" are 100 per cent fruit juices.

In fact, some are only 12 per cent juice.

This means that you get fewer vitamins.

The volume of the drink is then made up with water and added sugar.

LOAD-DATE: July 8, 2014

LANGUAGE: ENGLISH

PUBLICATION-TYPE: Newspaper

JOURNAL-CODE: SUNscot

Copyright 2014 NEWS GROUP NEWSPAPERS LTD

All Rights Reserved

MailOnline

July 7, 2014 Monday 2:17 PM GMT

Is SUGAR responsible for rising dementia cases? High levels of glucose can cause memory loss, study finds

BYLINE: SARAH GRIFFITHS

SECTION: HEALTH

LENGTH: 891 words

- Scientists at the Charité University Medical Centre in Berlin claim sugar and carbohydrates harm the brain's structure and function
- High levels of glucose are associated with worse memory in healthy adults
- It's been found that a sugary diet is linked to an increased risk of dementia

A diet high in sugar could be damaging your brain as well as inflating your waistline, scientists have warned.

In the past research has linked sugary foods with obesity but a new study has now found the risks associated with a sugar laden diet could have more widespread health implications, eating away at a person's brain power.

A team at Charité University Medical Centre in Berlin found eating lots of sugar and other carbohydrates harms both the brain's physical structure as well as its function.

They discovered high levels of glucose are associated with worse memory in healthy adults and could poetntially lead to more people suffering dementia.

The new research backs up previous studies, which have shown that diabetes - characterised by high levels of blood glucose - has been linked to an increased risk of dementia, Scientific American reported.

Experts have also previously found that high blood sugar levels are linked to a smaller hippocampus, which is an area of the brain used for remembering events and facts.

AS part of the new study, scientists wanted to find out whether glucose has a negative effect on memory in healthy people without diabetes.

They looked at short-term and long-term glucose markers in 141 healthy adults without any signs of diabetes, who took a memory test.

The participants also underwent an MRI scan to examine the structure of their hippocampus.

The experiment revealed that higher glucose blood levels were linked with bad memory as well as a smaller hippocampus.

Agnes Flöel, a neurologist at Charité and co-author of the study, said the results 'provide further evidence that glucose might directly contribute to hippocampal atrophy'.

However, she said the study cannot confirm a causal relation between brain health and eating lots of sugar.

Scientists will explore whether dietary and lifestyle changes might reverse changes in the brain.

Experts recently urged people to halve their sugar intake to just five teaspoons a day, in order to tackle soaring obesity levels.

Guidelines from scientists advising Public Health England - the agency given the job of tackling obesity - said that women should have no more than five to six teaspoons of sugar a day, and men seven to eight.

Currently, Britons consume an average of 15 teaspoons daily, mainly due to the high volumes of sugar hidden in everyday items such as fruit juice, muesli, yoghurts, sandwiches and ready meals.

Senior doctors and academics want the Government to force food manufacturers to cut **sugar** levels, ensure products are clearly labelled and impose a tax on **soft drinks**, but so far, options are only being considered.

In the meantime, experts are advising parents to ban fruit juice, squash and soft drinks from the dinner table and give their children only water and milk.

While some parents think that fruit juice is healthy, nutritionists have pointed out that it has played a part in fuelling sharp rises in obesity rates, type 2 diabetes and heart disease.

A typical teenager consumes 40 per cent more sugar than they should and Adults take in 13 per cent too much.

A study published in the British Medical Journal last month, claims that more than one in three adults have 'pre-diabetes,' a term used to indicate that a person has raised sugar levels and is therefore at a greater risk of developing type 2 diabetes.

Type 2 diabetes, which develops in adulthood, can shave six years off a person's life.

'FRIGHTENING RISE IN DIABETES CASES: MORE THAN 750 PEOPLE ARE BEING DIAGNOSED WITH THE CONDITION EVERY DAY

New figures show hundreds of people are diagnosed with diabetes every day in Britain.

Diabetes UK said that more than 280,000 people a year are diagnosed with diabetes - the equivalent of the population of Newcastle upon Tyne.

Each day 738 people are told that they have type 2 diabetes - which is linked to being overweight - and 30 are diagnosed with type 1 diabetes, which is not linked to weight.

About 3.8million people in the UK now have the condition and about 35 per cent of the population have borderline diabetes.

Diabetes UK has called for more focus on preventing type 2 diabetes, saying that if the rate of people developing the condition continues the consequences could be 'disastrous'.

The charity's chief executive, Barbara Young, said: 'It is deeply worrying that more than 700 people a day are being diagnosed with diabetes and this clearly shows the frightening scale of what is fast becoming a national health emergency.

'If we continue to see people being diagnosed at this rate then the consequences will be disastrous.

'As the number of people with diabetes grows, we are likely to see even more people endure devastating health complications such as amputation and kidney failure and more people die tragically young.

'It would also lead to an increase in NHS costs that would be simply unsustainable. As a country, we are still not giving diabetes healthcare the priority it needs and we also need to get much better at preventing type 2 diabetes before it is too late.'

LOAD-DATE: July 7, 2014

LANGUAGE: ENGLISH

PUBLICATION-TYPE: Web Publication

JOURNAL-CODE: WEBDM

i-Independent Print Ltd

July 4, 2014
First Edition

Children exposed to poor eating by TV; HEALTH

BYLINE: Charlie Cooper HEALTH REPORTER

SECTION: NEWS; Pg. 19

LENGTH: 162 words

Children are being bombarded with scenes of unhealthy eating in television programmes which "unrealistically" portray slim, healthy characters consuming high levels of fatty, sugary and salty foods, health experts warn.

Laws were passed in 2007 to curb the advertising of foods high in fat and sugar to under-16s, but no such restrictions are in place for portrayals of food in the programmes children watch.

However, a new study has revealed what Professor Clodagh O'Gorman, from University Hospital Limerick's department of paediatrics, called "very poor" representation of food and diet in TV shows aimed at children.

Unhealthy foods accounted for nearly half of the food shown, with sweet snacks the most commonly shown item. **Sugary drinks** represented a quarter of all the drinks displayed on screen.

The study, which is published in the journal Archives of Disease in Childhood today, is the first to analyse in detail the levels of exposure to unhealthy eating habits on TV.

LOAD-DATE: July 3, 2014

LANGUAGE: ENGLISH

PUBLICATION-TYPE: Newspaper

JOURNAL-CODE: III

Copyright 2014 Independent Print Limited
All Rights Reserved

The INDEPENDENT
The Independent (London)

July 4, 2014 Friday
First Edition

Children are force-fed unhealthy diet by TV shows, warn doctors

BYLINE: CHARLIE COOPER HEALTH REPORTER

SECTION: NEWS; Pg. 13

LENGTH: 518 words

Children are being bombarded with scenes of unhealthy eating in television programmes which "unrealistically" portray slim, healthy characters consuming high levels of fatty, sugary and salty foods, health experts have warned.

While laws were passed in 2007 to curb the advertising of foods high in fat and sugar to under-16s, no such restrictions are in place for portrayals of food in the programmes children watch.

However, a new study has revealed what one expert called "very poor" representation of food and diet in TV shows aimed at children.

Unhealthy foods accounted for nearly half of the food shown, with sweet snacks the most commonly shown item. **Sugary drinks** represented a quarter of all the drinks displayed on screen.

Researchers analysed more than 82 hours of children's television broadcast between 6am and 11am between July and October 2010, on the BBC and on Ireland's public service broadcaster RT??.

The study, which is published in the journal Archives of Disease in Childhood today, is the first to analyse in detail the levels of exposure to unhealthy eating habits on TV.

While the study did not name any particular shows, Professor Clodagh O'Gorman, from University Hospital Limerick's department of paediatrics, who took part in the study, said that the majority of children's programming from the US, Britain and Europe presented an unhealthy representation of eating and diet.

Programmes aimed at teenagers were particularly poor, she said. "The impression [given by the shows] was: 'It's a regular day, school's out, let's go and have fast food'," she said. "The programmes were not showing the effects of eating all of these high-fat and high-sugar foods that we would expect children to show in real life. That's unrealistic.

"Many shows were largely based at coffee shops or meeting places for kids where they all eat fast food and have high-calorie drinks. And yet they are all happy, they are all thin, certainly not reflective of the obesity seen in kids today."

The crisis in childhood eating habits and its effect on obesity levels has come into sharp focus in recent weeks, with health officials in the UK announcing new draft dietary guidelines which would halve daily recommended sugar intakes. The measures were largely aimed at curbing the consumption of sugar-sweetened drinks among children and teenagers.

Experts are concerned that high levels of obesity among the young could exact a heavy toll on society as the current generation grows up with an elevated risk of heart disease, diabetes and some cancers. In England, one-third of children in year six are overweight or obese, with similar levels in Scotland, Wales and Ireland.

Professor O'Gorman said that, while it was too soon to consider new laws to regulate the portrayal of eating on TV, public service broadcasters had a responsibility to consider the impact of their programmes of children's health. "Public space broadcasters could be making choices of one programme that represents a healthy lifestyle over another programme that does not," she said. "Society has a role to play in this and parents have a role."

LOAD-DATE: July 3, 2014

LANGUAGE: ENGLISH

PUBLICATION-TYPE: Newspaper

JOURNAL-CODE: IA

Copyright 2014 Independent Print Ltd
All Rights Reserved

The Telegraph
telegraph.co.uk

July 4, 2014 Friday 11:52 AM GMT

Top tips for reducing your sugar intake; Experts are going to war with sugar. Here, health coach Laura Thomas shares advice for cutting down your intake of the white stuff

BYLINE: By Laura Thomas

LENGTH: 668 words

With the recent draft report from the Scientific Advisory Committee (SCAN) urging people to halve their sugar intake, it's clear the Government wants us to embrace a lower-sugar lifestyle.

Here are my top tips for cutting back without feeling like the joy is being taken out eating, and addressing some of the the trickier social and emotional challenges when it comes to long-term change.

Look at drinks first

Drinking **sugar** is often a habit rather than necessity: the **sugar** in the tea, the fruity drink with lunch or the **soft drink** when socialising. If going without is particularly tough at first, look to cut down the amount of **sugar** gradually to allow a natural shift in your taste preference for it. For example, put just half the teaspoon of **sugar** into your tea or use a smaller juice glass in the morning.

Read: Sweet poison: why sugar is ruining our health

Find lower sugar substitutes for your favourite foods

Quite often there can be a big difference in sugar content between brands of the same food. Spending a little time researching and finding the ones that don't have as much added sugar shaves off a few grams that can make a difference if you're eating these products daily.

Be mindful of total sugar (especially fructose)

Although fruits and natural sources of sugar have nutritional benefit and are healthy in many respects, they are still sweet and you're likely to still experience powerful cravings when excessively consuming them. Fructose, which is found in refined sugar and fruit sources, is the addictive part of sugar that we desire the most. To start managing your cravings, be mindful of the total amount of sweet food in your diet (including fruit), the quantities you're consuming and how often you're eating it.

Avoid hidden sugar

It goes without saying, avoid sugar that you don't know you're eating. Double check sauces, dressings, cereals, soups etc. Look to make your own where you can and cut back on processed foods as much as possible.

One thing at a time

When you first start looking to cut back on sugar and you realise you're eating a lot of it, the whole prospect can be somewhat depressing. Start by making one change at a time, like having a lower-sugar topping for your porridge or making your own salad dressing. Small changes over time are more sustainable than a drastic all-or-nothing approach.

Consider your use of sugar

It's very common for people to use sugar as a stress-coping mechanism. It's accessible, cheap, quick and easy. Take note if you're consuming sugar in response to emotional hunger. Seek to build in other coping mechanisms that don't involve sweet food e.g yoga or a run, something relaxing like a walk or a simple breathing exercise.

Embrace the savoury foods you love

To avoid the doom and gloom feeling of eating less sugar, embrace your favourite savoury foods in all their forms. Try new combinations of them, be more experimental with foods and try to enjoy the process of finding savoury alternatives that you really get excited about.

Work through social challenges

Often it's the social side of sugar that be particularly challenging. Birthdays, weddings and numerous annual occasions are closely associated with excessively sugary food. Seek to understand if you're eating sugar because everyone else is, or if it's closely tied to the joy you feel at these events and celebrations. Building awareness of your sugar habits in social situations is the first best step to adjusting them.

Laura Thomas is the founder of Happy Sugar Habits which helps individuals get control over their sugar cravings. Visit for more tips, recipes and advice

LOAD-DATE: July 4, 2014

LANGUAGE: ENGLISH

PUBLICATION-TYPE: Newspaper; Web Publication

JOURNAL-CODE: WEBDTNS

Copyright 2014 Telegraph Media Group Limited

All Rights Reserved

The Telegraph
telegraph.co.uk

July 4, 2014 Friday 7:16 AM GMT

Coca Cola investigate after can explodes in car; Top of a can of Coca Cola was blown off when it exploded after being left in the back of a car

BYLINE: By Keith Perry

SECTION: NEWS

LENGTH: 281 words

Drinks giant Coca Cola is investigating after a can of the **fizzy drink** exploded in a customer's car.

Pensioner Michael Knight said he left the drink on the rear seat of his Citroen C5 car and discovered that the top had blown off, spilling the contents over the seat.

He said the can had been left under a towel so was not in direct sunlight although he concedes that the car could have become hot while parked.

Mr Knight is concerned that their grandchildren could have been in the car at the time, which is fitted with a child seat.

He will also need have the car valeted as the sticky residue from the exploding can has spilled over the carpets and seats.

Mr Knight, from Taunton, Somerset, said: "We left the Coca Cola can in the car inadvertently and were shocked to find the thing had exploded.

"It appears to have given way at the weak spot on the tin and just blown apart.

"The contents are all over the car. We have never heard of anything like this happening before and Coca-Cola is investigating.

"We are concerned that our grandchildren could have been with us at the time and it was left next to a child seat for our youngest granddaughter who is eight.

"What would happen if someone was driving along a busy motorway and the thing exploded and caused the driver to swerve?"

A spokesman for Coca Cola said: "Thank you for bringing this matter to our attention. We take great pride in the quality of our products and were very concerned to hear of your reader's recent experience.

"We have been in touch with Mr Knight to apologise for any distress and to arrange to collect the can so that we can conduct a thorough investigation to understand exactly what may have happened."

LOAD-DATE: July 4, 2014

LANGUAGE: ENGLISH

PUBLICATION-TYPE: Newspaper; Web Publication

JOURNAL-CODE: WEBDTNS

Copyright 2014 Telegraph Media Group Limited
All Rights Reserved

The INDEPENDENT
Independent.co.uk

July 3, 2014 Thursday 11:30 PM GMT

Children bombarded with unhealthy eating messages on TV, experts warn; Unhealthy foods accounted for nearly half of the food shown, with sweet snacks the most commonly shown item

BYLINE: Charlie Cooper

SECTION: FRONTPAGE

LENGTH: 646 words

Children are being bombarded with scenes of unhealthy eating on TV, in programmes which "unrealistically" portray slim, healthy characters consuming high levels of fatty, sugary and salty foods, health experts have warned.

While laws were passed in 2007 to curb the advertising of foods high in fat and sugar to under-16s, no such restrictions are in place for portrayals of food in the programmes children watch.

However, a new study has revealed what one expert called "very poor" representation of food and diet in TV shows aimed at children.

Unhealthy foods accounted for nearly half of the food shown, with sweet snacks the most commonly shown item. **Sugary drinks** represented a quarter of all the drinks displayed on screen.

Researchers analysed more than 82 hours of children's television broadcast between 6am and 11am between July and October 2010, on the BBC and on Ireland's public service broadcaster RTÉ.

The study, which is published in the journal Archives of Disease in Childhood today, is the first to analyse in detail the levels of exposure to unhealthy eating habits on TV.

While the study did not name any particularly shows, Professor Clodagh O'Gorman, from University Hospital Limerick's department of paediatrics, who took part in the study, said that the majority of children's programming from the USA, Britain and Europe presented a unhealthy representation of eating and diet.

Programmes aimed at teenagers was particularly poor, she said.

"The impression [given by the shows] was: 'It's a regular day, school's out, let's go and have fast food'," she said. "The programmes were not showing the effects of eating all of these high fat and high sugar foods that we would expect children to show in real life. That's unrealistic.

"Many shows were largely based at coffee shops or meeting places for kids where they all eat fast food and have high calorie drinks. And yet they are all happy, they are all thin, certainly not reflective of the overweight and obesity seen in kids today."

The crisis in childhood eating habits and its effect on obesity levels has come into sharp focus in recent weeks, with health officials in the UK announcing new draft dietary guidelines which would halve daily recommended sugar intakes. The measures were largely aimed at curbing the consumption of sugar-sweetened drinks among children and teenagers.

Health experts are increasingly concerned that high levels of overweight and obesity among the young could exact a heavy toll on society as the current generation grows up with an elevated risk of heart disease, diabetes and some cancers. In England, one third of children in year six are overweight or obese, with similarly high levels in Scotland, Wales and Ireland.

Professor O'Gorman said that, while it was too soon to consider new laws to regulate the portrayal of eating on TV, public service broadcasters had a responsibility to consider the impact of their programmes of children's health.

"Public space broadcasters whose mission is to protect, inform and empower the public could be making choices of one programme that represents a healthy lifestyle over another programme that does not," she said. "Society has a role to play in this and parents have a role to play. But if the BBC and the RTÉ, had parameters for choosing good health food shows over poor health food shows, I think the programmers and broadcasters who make these shows would ultimately start to improve the way the portray food."

There is, as of yet, no established link between the unhealthy eating that children see in TV programmes, and their own eating habits, but Professor O'Gorman said this was an area that now warranted research.

A BBC spokesperson said: "We broadcast lots of programmes to promote healthy eating to children and to help them understand where food comes from, with series like I Can Cook, Incredible Edibles and Blue Peter."

LOAD-DATE: July 4, 2014

LANGUAGE: ENGLISH

PUBLICATION-TYPE: Newspaper; Web Publication

JOURNAL-CODE: WEBI

Copyright 2014 Independent Digital News and Media Limited
All Rights Reserved

MailOnline

MailOnline

July 3, 2014 Thursday 10:07 AM GMT

Kids consuming more than two serves of fatty food and at least one sugary drink a day, according to study

BYLINE: LOUISE CHEER and AAP

SECTION: NEWS

LENGTH: 405 words

- It is the first time unhealthy eating has been linked directly to weight gain
- The Victorian university study looked at 4,000 kids between four and 10
- Data was collected from them over four time periods every two years
- It found for each unhealthy item consumed a child's BMI score increased

Australian kids are consuming more than two serves of fatty food and at least one **sugary drink** each day, which is directly having an impact on their weight gain.

A long-term Deakin University study also found for each unhealthy item consumed by children, their BMI score increased.

The new study looked at 4,164 children from ages four to 10, with data collected over four time periods every two years. It classed fatty foods as hamburgers, hot chips, potato chips, doughnuts, cake or chocolate. **Sugary drinks** included cordial, **soft drinks** and fruit juice.

It found six-years-olds consumed 1.4 serves of **sugary drinks** a day and 1.9 serves of fatty foods. At 10 years, this increased to 1.5 serves per day for **sugary drinks**.

Deakin University WHO Collaborating Centre for Obesity Prevention's Dr Lynne Millar said many would say 'yes we already know this', but studies to date had been inconclusive.

'Some [found] a connection between **sugary drinks** and fatty foods and children's weight and others not,' she said.

'We have conducted a very robust study, and for the first time found that these foods and drinks independently contribute to childhood obesity.'

Dr Millar said the study showed that consuming snack foods had become 'normalised' for children and she was shocked by the results.

'I didn't think kids on average would eat that much unhealthy food,' she said.

'I thought they may have days when they'd just have fruit, vegetables and meat and other healthy food, rather than snack food each day.'

For each drink or high fat food consumed, the children's BMI-z score - a standardised BMI measure used specifically with children - increased by 0.015 and 0.014 units respectively.

'While the increases may seem low, they are cause for concern,' Dr Millar said.

Dr Millar said a normal BMI-z score would be zero, so a score above one was considered overweight and above two was obese.

One in four Australian children are overweight or obese and this issue is more than an individual's responsibility, she said.

'It's about providing a healthy environment for a child and making it easier for them to eat healthy food and be healthy people,' Dr Millar said.

LOAD-DATE: July 3, 2014

LANGUAGE: ENGLISH

PUBLICATION-TYPE: Web Publication

JOURNAL-CODE: WEBDM

Copyright 2014 Associated Newspapers Ltd.
All Rights Reserved

The Express

July 2, 2014 Wednesday
Edition 1;
National Edition

Sweet taste of success in battle to cut out sugar; Letters

SECTION: LETTERS; Pg. 28

LENGTH: 86 words

AFTER reading your article on **fizzy drinks** I thought I would share my experience to help inspire others ("Britons must halve sugar intake say experts", June 27).

I've had a sweet tooth since I was a kid but this year my New Year's resolution was to cut down on sugar. It's taken me six months but I've cut right down and now have only one sugar in tea and coffee.

Now **fizzy drinks** taste sickly to me, they are so sweet. It can take time to get off sugar but come on, if I can do it, so can you.

John Tucker, Luton, Beds

LOAD-DATE: July 2, 2014

LANGUAGE: ENGLISH

PUBLICATION-TYPE: Newspaper

JOURNAL-CODE: DXP

Copyright 2014 Express Newspapers
All Rights Reserved

The Sun (England)

July 2, 2014 Wednesday
Edition 1;
National Edition

THE Sun SAYS Weigh to go

BYLINE: Sun SAYS

SECTION: EDITORIAL; OPINION; LEADING ARTICLES; Pg. 8

LENGTH: 126 words

OUR childhood obesity figures are shocking.

When they reach primary school just over a fifth of kids are overweight or obese. By the time they leave that has shot up to a third.

Yet pupils aren't routinely weighed at school in those six intervening years. As Tory MP and ex-GP Sarah Wollaston says, they should be, so that problems can be caught early.

Schools must also teach them more about nutrition and the risks of piling on the pounds.

We don't agree with singling out **fizzy drinks** for an extra tax to deter buyers. That's unfair all round.

Dr Wollaston's idea, to insist drink firms cut the price of low-sugar products, is far better.

Obesity is a crisis we must resolve. For the millions whose lives it wrecks - and for the NHS which is overwhelmed by it.

LOAD-DATE: July 2, 2014

LANGUAGE: ENGLISH

PUBLICATION-TYPE: Newspaper

JOURNAL-CODE: SUN

Copyright 2014 NEWS GROUP NEWSPAPERS LTD
All Rights Reserved

Daily Mirror

August 31, 2014 Sunday
Edition 1;
Northern Ireland

docs' plea for our obese kids; They want emergency taskforce and sugary drinks tax

BYLINE: VINCENT MOSS

SECTION: NEWS; Pg. 2

LENGTH: 417 words

DOCTORS are calling for an emergency taskforce to stop obesity destroying children's health.

GPs say the group should be like the Government's Cobra panel, which deals with terrorism and national disasters.

They warn the NHS will be overwhelmed without action to cut the consumption of junk food, including a tax on **sugary drinks**. Obesity now costs the health service £4.2billion a year.

Medics outline their fears about Britain's youngsters - among Europe's fattest - in a letter to Chief Medical Officer Professor Dame Sally Davies sent at the start of the new school term.

The Royal College of GPs and 11 other organisations call for a Child Obesity Action Group to be set up urgently.

It would bring together doctors, nurses, midwives, dieticians, dentists and schools to help children avoid the worst food and drinks. The letter calls for more measuring of children's weight, more training on the issue for GPs and health workers, and better education for parents about obesity risks.

Dr Rachel Pryke, the Royal College's clinical lead for nutrition, said: "Overweight children are being set up for a lifetime of sickness.

"We are in danger of destroying the health of a whole generation. As parents and health professionals, we need to take responsibility and ensure every child has a healthy and varied diet and regular exercise.

malnourished "We cannot allow our young people to become malnourished, squandering their childhood and vitality hunched over computer consoles and gorging on junk food. We have reached a state of emergency with childhood obesity and the current threat to public health is most definitely severe."

The doctors point out that obesity, now affecting 31 per cent of children, can lead to cancer and many other diseases, including diabetes. Dr Richard Roope, the Royal College's clinical lead for cancer, warned that today's children could be the first generation to die before their parents.

He said: "For the first time we have a generation who may predecease their parents. After smoking, obesity is the biggest reversible factor in cancers.

"Radical steps need to be taken - at the very least levying tax on **sugary drinks**. We've seen this approach work with smoking.

"We have a huge problem when seven-year-olds present with type 2 diabetes, previously associated with the weight gain of middle age. Our children are among the most overweight in Europe. This is something we should all be ashamed of. We have a responsibility to reverse the trend."

vincent.moss@sundaymirror.co.uk

LOAD-DATE: September 10, 2014

LANGUAGE: ENGLISH

PUBLICATION-TYPE: Newspaper

JOURNAL-CODE: SMR

Copyright 2014 MGN Ltd.
All Rights Reserved

MailOnline

MailOnline

August 31, 2014 Sunday 11:55 PM GMT

Fat children have up to six times more high blood pressure risk: Figures lead to doctors calling for taskforce to tackle child obesity

BYLINE: JENNY HOPE, DAILY MAIL MEDICAL CORRESPONDENT

SECTION: HEALTH

LENGTH: 911 words

- Body representing GPs warns an entire generation will be 'destroyed' by junk food and **sugary drinks**
- Young obese girls have the biggest problem of getting heart disease
- One in three children is obese by time they leave primary school in the UK

Children who are obese are up to six times more at risk of high blood pressure - an adult condition commonly linked to heart disease, warn specialists.

Young obese girls have the biggest problem, with a 5.9-fold chance of developing it compared with those of healthy weight, while the risk is four times greater for obese boys.

The study from Germany also shows that children and teenagers who are overweight are more likely to have high blood pressure readings.

Experts say the findings are 'alarming', given that one in three children is overweight and obese by the time they leave primary school in the UK.

They come as doctors called for the creation of an emergency taskforce to tackle 'the rising epidemic' of childhood obesity, similar to the Government's Cobra panel which deals with terrorism and national disasters.

The professional body representing GPs has warned that an entire generation will be 'destroyed' by a diet of junk food and **sugary drinks** unless urgent action is taken.

In an open letter to the Chief Medical Officer for England, Professor Dame Sally Davies, the Royal College of General Practitioners (RCGP) and 11 linked organisations said a national Child Obesity Action Group (COAG) should be formed as 'a matter of urgency'.

Doctors, nurses, midwives, dieticians, dentists and schools would collaborate to try to prevent obesity and improve treatment services to stop children developing health problems in later life.

Dr Richard Roope, RCGP clinical lead for cancer, said: 'For the first time, we have a generation of patients who may predecease their parents. Only three per cent of the public associate weight with cancer, yet, after smoking, obesity is the biggest reversible factor in cancers.'

Stark evidence of the link between body fat and ill-health, even in young children, was released yesterday (sun) by researchers running a Family Heart Study in Nuremberg, Germany.

It included 22,051 children and adolescents aged three to 18 years from 'health-conscious' families whose blood pressure, body mass index (BMI), waist circumference and body fat percentage was measured.

They found that compared with normal weight children and adolescents, the risk of prehypertension (which precedes a condition needing medical treatment) was significantly higher in youngsters with high BMI scores.

The prevalence of high blood pressure increased in boys and girls as body weight went up, based on averages among the groups at the age of seven years and again at 14 years.

Hypertension in normal weight boys was 5.7 per cent, rising to 10.4 per cent for the overweight and 18.6 per cent in obese boys.

The researchers found the prevalence of hypertension was highest in obese girls at 24.4 per cent, and 9 per cent in

the overweight compared with five per cent in those of normal weight.

Prof Schwandt said 'The risk of having hypertension is 5.9 time higher for an obese girls and 4.3 times higher for an obese boy than for normal-weight young people.'

He released the data yesterday (sun) at the European Society of Cardiology (ESC) meeting in Barcelona.

Hypertension was a blood pressure reading over the 95th percentile of the blood pressure curve for children and adolescents. The diagnosis was based on several measurements on separate days and on repeated estimations with the child sitting quietly for five minutes.

Professor Empar Lurbe, professor of paediatrics at the University of Valencia, Spain, told the meeting: 'Blood pressure in children increases with age and body size.

Our study clearly shows that the fatter young people are, the greater their risk of prehypertension and hypertension. Any weight loss they can achieve will help reduce their risk

'This makes it impossible to use a single blood pressure level to define hypertension, as we do in adults.'

Professor Schwandt said 'Our study clearly shows that the fatter young people are, the greater their risk of prehypertension and hypertension. Any weight loss they can achieve will help reduce their risk.

'This is of great importance because of the ongoing rise in the prevalence of hypertension and overweight/obesity in young people and the tracking of childhood overweight into adulthood.'

Tam Fry, from the UK's National Obesity Forum, said 'It's alarming. This demonstrates that obesity is no longer a timebomb; it is a crisis which is escalating every day.

'We need to stop children getting fat in the first place, because fat children become fat adults and as this study shows, even before then they are suffering from major health risks.

'When it comes to high blood pressure, which vastly increases the risk of heart attacks and strokes, it is likely that the foods being eaten by obese children are not helping; crisps piled high in salt, and so forth.

'This has to be wake-up call. We are looking at a situation in which children are buried before their parents, the obesity crisis in Britain is horrifying.'

Professor Pedro Marques-Vidal, head of the ESC's preventive health group, said doctors were 'definitely not' advocating that blood pressure drugs be given to children.

He said there was evidence that losing weight could reverse high blood pressure and it was vital that parents and schools worked together to combat the problem.

LOAD-DATE: August 31, 2014

LANGUAGE: ENGLISH

PUBLICATION-TYPE: Web Publication

JOURNAL-CODE: WEBDM

Copyright 2014 Associated Newspapers Ltd.

MAIL ON SUNDAY (London)

August 31, 2014 Sunday

COKE ISN T UNHEALTHY, WE VE SPENT £15M CUTTING CALORIES

BYLINE: BY VICKI OWEN

LENGTH: 804 words

I DON T think any of our products are unhealthy, says Jon Woods, the UK and Ireland boss of Coca-Cola. It s a comment that is sure to raise eyebrows, as there are many people for whom the bright red cans of **fizzy drink** epitomise junk food.

Soft drinks have recently been cited as a key offender in Britain s obesity problem and a tax on **sugary** food is one proposed solution to a concern that Woods is more than willing to tackle head on.

You can t get away from the fact that there is an issue with people becoming more and more overweight in the UK. Sixty per cent of adults and 30 per cent of children are overweight or obese, he says in his gentle, Belfast accent.

If you re in the food and drink business like me then you have a responsibility to play a part in dealing with it. And at Coke we re playing a very, very active role in the conversation around obesity.

The latest step that Coca-Cola has taken to offer more choice is, of course, a new product - Coca-Cola Life. The cans look exactly like a classic Coke, but instead of bright red they are an almost luminous green - the obligatory colour these days for products claiming a life-enhancing quality.

Coca-Cola Life is intended to capture the middle ground between regular Coca-Cola and the no-calorie Coca-Cola Zero. It has 30 per cent fewer calories than regular Coke and uses a plant-based sweetener, stevia, hailed by its proponents as a natural alternative to sugar.

However, it still contains 89 calories and has come under attack, with campaign group Action on Sugar s scientific director Dr Aseem Malhotra describing it as a product with a high sugar content that would encourage people to have a sweet tooth .

For Woods this is a familiar refrain. He admits: Look, not everybody thinks we re doing all the best things, but I think most health campaigners would accept that we re making very meaningful strides.

He reels off a series of facts about Coca-Cola s range, which nets £1.5[0/00]billion of sales every year in the UK[0/00].

We re reformulating a lot of our products. Sprite has 30 per cent fewer calories than it had 18 months ago. Fanta s got 30 per cent fewer calories. Oasis has got fewer calories. We are very actively reformulating our products - for instance Dr Pepper, Woods says.

This is not easy to do. If it were easy to do we d have done it a long time ago, but creating really great tasting products with fewer calories is not that straightforward and so we ve invested £15[0/00]million in reformulating our brands over the last couple of years.

One of the commitments we made to the Government is that we d reduce the average calories per litre of our sparkling products by 5 per cent. And we re on track to deliver that.

Whatever the public concern about health and obesity, and however rightly or wrongly brands like Coca-Cola are blamed, the business is doing well. UK sales have risen and the company employs 4,500 staff here.

Woods, 46, commutes to the London HQ from the family home in Oxfordshire where he lives with his wife and two children.

Like so many children, they enjoy Coke. So does Woods keep an eye on how much they consume?

I m the same as any other parent. You watch what your kids eat and drink. As they get older they get to make more of the choices themselves.

My kids are very good, they make good choices. They love a Coke, they love Oasis.

Those are a few of the brands that are in the fridge at home. But they drink a lot of other things as well and they are very physically active so they are burning a lot of calories.

Woods has been in the soft drinks business almost his entire career. He studied banking and finance at Loughborough University, but says he got lucky and went into sales and marketing in the food and drink industry at Cadbury Schweppes, eventually arriving at Coca-Cola in 2005 and taking charge of the UK and Ireland operations in 2010

.

He says: Believe it or not I wanted to be a bank manager like my dad. But it was really not for me.

I m very, very fortunate that I ve ended up spending 20 years in the food and drink business. It s a great business and I think I chose well and got lucky.

Indeed the job does seem to have its upsides. When we meet, Woods has just returned from launching another new brand, glaceau smartwater, with Kylie Minogue.

It is already the number one premium water brand in America, and the UK will be the beachhead for a European launch.

It s sourced water from our spring in Northumberland. And then we add electrolytes to give it crispness, so it tastes slightly different.

We launched this ten days ago and I saw pictures of some Tesco stores with glaceau smartwater appearing this week.

Kylie was helping us launch it. She is one of our ambassadors, to help us launch. She was really fun - she was great this morning.

It does sound rather more fun than banking.

LOAD-DATE: August 30, 2014

LANGUAGE: ENGLISH

PUBLICATION-TYPE: Papers

Copyright 2014 Associated Newspapers Ltd.
All Rights Reserved

MAIL ON SUNDAY (London)

August 31, 2014 Sunday

STARTING TO WORRY ABOUT YOUR WEIGHT? MEET STEVIA¦

LENGTH: 221 words

COCA-COLA Life will reach full distribution in Britain in a fortnight.

The drink contains a third less sugar and a third fewer calories than a regular Coca-Cola - 89 compared to 139.

Like 97 per cent of Coca-Cola products sold in Britain it is made in East Kilbride, in Scotland, Wakefield in West Yorkshire, Sidcup, in South-East London, Edmonton, in North London, or Milton Keynes, in Buckinghamshire. The key ingredient is a sweetener extracted from the Paraguayan plant stevia rebaudiana, known as stevia. It is a relative of the chrysanthemum, and has been grown and harvested for two centuries. It is now available in 75 countries.

Products using stevia have received approval from food regulators in most major markets in recent years and a number of leading food and drink makers have been producing stevia-based products.

Coca-Cola s arch-rival Pepsi has its own stevia based product called Pepsi next.

Coca-Cola Life was launched in Argentina and Chile last year and in America earlier this year.

After launching in Britain it will go on sale in Sweden.

Jon Woods, UK and Ireland general manager of Coca-Cola, says: Coca-Cola Life is for a slightly older age group. It s for people who are just starting to think about calories and concerns over weight management but love the taste of Coke and love the idea of naturalness.

LOAD-DATE: August 30, 2014

LANGUAGE: ENGLISH

PUBLICATION-TYPE: Papers

Copyright 2014 Associated Newspapers Ltd.
All Rights Reserved

Mirror

mirror.co.uk

August 31, 2014 Sunday 12:15 AM GMT

Childhood obesity must be tackled by "emergency taskforce" to stop destroying kids' health; Medics have outlined their fears about Britain's youngsters - among Europe's fattest - in a letter to Chief Medical Officer Professor Dame Sally Davies

BYLINE: By Vincent Moss

SECTION: NEW ARTICLES

LENGTH: 424 words

Doctors are calling for an emergency taskforce to stop obesity destroying children's health.

GPs say the group should be like the Government's Cobra panel, which deals with terrorism and national disasters.

They warn the NHS will be overwhelmed without action to cut the consumption of junk food, including a tax on **sugary drinks**.

Obesity now costs the health service £4.2billion a year.

Medics outline their fears about Britain's youngsters - among Europe's fattest - in a letter to Chief Medical Officer Professor Dame Sally Davies sent at the start of the new school term.

The Royal College of GPs and 11 other organisations call for a Child Obesity Action Group to be set up urgently.

British kids overweight or obese

It would bring together doctors, nurses, midwives, dieticians, dentists and schools to help children avoid the worst food and drinks.

The letter calls for more measuring of children's weight, more training on the issue for GPs and health workers, and better education for parents about obesity risks.

Dr Rachel Pryke, the Royal College's clinical lead for nutrition, said: "Overweight children are being set up for a lifetime of sickness.

"We are in danger of destroying the health of a whole generation.

UK Obesity Epidemic

"As parents and health professionals, we need to take responsibility and ensure every child has a healthy and varied diet and regular exercise.

"We cannot allow our young people to become malnourished, squandering their childhood and vitality hunched over computer consoles and gorging on junk food.

"We have reached a state of emergency with childhood obesity and the current threat to public health is most definitely severe."

The doctors point out that obesity, now affecting 31 per cent of children, can lead to cancer and many other diseases, including diabetes.

Direct cost to the NHS of obesity

Dr Richard Roope, the Royal College's clinical lead for cancer, warned that today's children could be the first generation to die before their parents.

He said: "For the first time we have a generation who may predecease their parents.

"After smoking, obesity is the biggest reversible factor in cancers.

"Radical steps need to be taken - at the very least levying tax on **sugary drinks**. We've seen this approach work with smoking.

"We have a huge problem when seven-year-olds present with type 2 diabetes, previously associated with the weight gain of middle age.

"Our children are among the most overweight in Europe.

"This is something we should all be ashamed of. We have a responsibility to reverse the trend."

LOAD-DATE: August 31, 2014

LANGUAGE: ENGLISH

PUBLICATION-TYPE: Newspaper; Web Publication

JOURNAL-CODE: WEBDMI

Copyright 2014 Trinity Mirror, Plc.
All Rights Reserved

THE SUNDAY TIMES

The Sunday Times (London)

August 31, 2014 Sunday
Edition 3;
National Edition

GPs declare state of emergency over childhood obesity threat

BYLINE: Sarah-Kate

SECTION: NEWS; Pg. 7

LENGTH: 402 words

THE professional body representing GPs has declared a state of emergency on childhood obesity and urged the government to set up a taskforce to tackle the problem.

The Royal College of General Practitioners (RCGP) said urgent action was required because obesity was in danger of "destroying the health of a whole generation of children". It said the threat posed by childhood obesity was "most definitely severe" and that the National Health Service would be overwhelmed.

GPs were prompted to issue the alert after seeing children as young as seven in their surgeries with type 2 diabetes caused by obesity. They said the country should be "ashamed" of the damage to children who could face "a lifetime of sickness".

Dr Rachel Pryke, RCGP clinical spokeswoman for nutrition, said:"We are in danger of destroying the health of a whole generation of children. We cannot allow our young people to become malnourished, squandering their childhood and vitality hunched over computer consoles and gorging on junk food.

"We have reached a state of emergency with childhood obesity and the current threat to public health is most definitely 'severe'."

In an open letter to Dame Sally Davies, the chief medical officer for England, timed to coincide with the start of the new school year this week, the RCGP and 11 partner organisations have recommended that a national child obesity action group be established.

Dr Richard Roope, RCGP clinical spokesman for cancer, said: "GPs and other healthcare professionals are now seeing a range of health problems in children that, in many cases, will develop into serious lifelong illnesses. We have a huge problem on our hands when seven-year-olds present in our surgeries with type 2 diabetes - something that was previously only ever associated with the weight gain of middle-age."

He added:"For the first time we have a generation of patients who may predecease their parents. Only 3% of the public associate weight with cancer yet, after smoking, obesity is the biggest reversible factor in cancers."

The GPs want a tax on **sugary drinks,** arguing that there was a notable fall in the number of smokers when the price of cigarettes was increased.

By the time children start primary school, 9% are obese. The proportion rises to 19% The government is not considering a sugar tax. The chief medical officer will formally respond to the letter addressing all the points raised in due course."

LOAD-DATE: September 3, 2014

LANGUAGE: ENGLISH

GRAPHIC: "We know that obesity is at its lowest Doctors blame video games and junk food for the obesity crisis

PUBLICATION-TYPE: Newspaper

JOURNAL-CODE: STS

Copyright 2014 Times Newspapers Limited
All Rights Reserved

Mail Online

MailOnline

August 30, 2014 Saturday 10:01 PM GMT

'Coke ISN'T unhealthy, we've spent £15m cutting calories': Boss insists Coca-Cola is leading the fight against obesity

BYLINE: VICKI OWEN, FINANCIAL MAIL ON SUNDAY

SECTION: NEWS

LENGTH: 1049 words

I don't think any of our products are unhealthy,' says Jon Woods, the UK and Ireland boss of Coca-Cola.

It's a comment that is sure to raise eyebrows, as there are many people for whom the bright red cans of **fizzy drink** epitomise junk food.

Soft drinks have recently been cited as a key offender in Britain's obesity problem and a tax on **sugary** food is one proposed solution to a concern that Woods is more than willing to tackle head on.

'You can't get away from the fact that there is an issue with people becoming more and more overweight in the UK. Sixty per cent of adults and 30 per cent of children are overweight or obese,' he says in his gentle, Belfast accent.

'If you're in the food and drink business like me then you have a responsibility to play a part in dealing with it. And at Coke we're playing a very, very active role in the conversation around obesity.'

The latest step that Coca-Cola has taken to offer more choice is, of course, a new product - Coca-Cola Life. The cans look exactly like a classic Coke, but instead of bright red they are an almost luminous green - the obligatory colour these days for products claiming a life-enhancing quality.

Coca-Cola Life is intended to capture the middle ground between regular Coca-Cola and the no-calorie Coca-Cola Zero. It has 30 per cent fewer calories than regular Coke and uses a plant-based sweetener, stevia, hailed by its proponents as a natural alternative to sugar.

However, it still contains 89 calories and has come under attack, with campaign group Action on Sugar's scientific director Dr Aseem Malhotra describing it as 'a product with a high sugar content' that would 'encourage people to have a sweet tooth'.

For Woods this is a familiar refrain. He admits: 'Look, not everybody thinks we're doing all the best things, but I think most health campaigners would accept that we're making very meaningful strides.'

He reels off a series of facts about Coca-Cola's range, which nets £1.5billion of sales every year in the UK .

'We're reformulating a lot of our products. Sprite has 30 per cent fewer calories than it had 18 months ago. Fanta's got 30 per cent fewer calories. Oasis has got fewer calories. We are very actively reformulating our products - for instance Dr Pepper,' Woods says.

'This is not easy to do. If it were easy to do we'd have done it a long time ago, but creating really great tasting products with fewer calories is not that straightforward and so we've invested £15million in reformulating our brands over the last couple of years.

'One of the commitments we made to the Government is that we'd reduce the average calories per litre of our sparkling products by 5 per cent. And we're on track to deliver that.'

Whatever the public concern about health and obesity, and however rightly or wrongly brands like Coca-Cola are blamed, the business is doing well. UK sales have risen and the company employs 4,500 staff here.

Woods, 46, commutes to the London HQ from the family home in Oxfordshire where he lives with his wife and two children. Like so many children, they enjoy Coke. So does Woods keep an eye on how much they consume?

'I'm the same as any other parent. You watch what your kids eat and drink. As they get older they get to make more of the choices themselves.

'My kids are very good, they make good choices. They love a Coke, they love Oasis.

'Those are a few of the brands that are in the fridge at home. But they drink a lot of other things as well and they are very physically active so they are burning a lot of calories.'

Woods has been in the soft drinks business almost his entire career. He studied banking and finance at Loughborough University, but says he 'got lucky' and went into sales and marketing in the food and drink industry at Cadbury Schweppes, eventually arriving at Coca-Cola in 2005 and taking charge of the UK and Ireland operations in 2010.

He says: 'Believe it or not I wanted to be a bank manager like my dad. But it was really not for me.

'I'm very, very fortunate that I've ended up spending 20 years in the food and drink business. It's a great business and I think I chose well and got lucky.'

Indeed the job does seem to have its upsides. When we meet, Woods has just returned from launching another new brand, glaceau smartwater, with Kylie Minogue. It is already the number one premium water brand in America, and the UK will be the beachhead for a European launch.

'It's sourced water from our spring in Northumberland. And then we add electrolytes to give it crispness, so it tastes slightly different.

'We launched this ten days ago and I saw pictures of some Tesco stores with glaceau smartwater appearing this week.

'Kylie was helping us launch it. She is one of our ambassadors, to help us launch. She was really fun - she was great this morning.'

It does sound rather more fun than banking.

Starting to worry about your weight? Meet stevia...

Coca-Cola Life will reach full distribution in Britain in a fortnight.

The drink contains a third less sugar and a third fewer calories than a regular Coca-Cola - 89 compared to 139.

Like 97 per cent of Coca-Cola products sold in Britain it is made in East Kilbride, in Scotland, Wakefield in West Yorkshire, Sidcup, in South-East London, Edmonton, in North London, or Milton Keynes, in Buckinghamshire. The key ingredient is a sweetener extracted from the Paraguayan plant stevia rebaudiana, known as stevia. It is a relative of the chrysanthemum, and has been grown and harvested for two centuries. It is now available in 75 countries.

Products using stevia have received approval from food regulators in most major markets in recent years and a number of leading food and drink makers have been producing stevia-based products.

Coca-Cola's arch-rival Pepsi has its own stevia based product called Pepsi next.

Coca-Cola Life was launched in Argentina and Chile last year and in America earlier this year.

After launching in Britain it will go on sale in Sweden.

Jon Woods, UK and Ireland general manager of Coca-Cola, says: 'Coca-Cola Life is for a slightly older age group. It's for people who are just starting to think about calories and concerns over weight management but love the taste of Coke and love the idea of naturalness.'

LOAD-DATE: August 30, 2014

LANGUAGE: ENGLISH

PUBLICATION-TYPE: Web Publication

JOURNAL-CODE: WEBDM

Copyright 2014 Associated Newspapers Ltd.
All Rights Reserved

Daily Mirror

Daily Mirror

August 29, 2014 Friday
Edition 1;
Northern Ireland

'Beat obesity with fizz tax'

SECTION: FEATURES; Pg. 27

LENGTH: 82 words

A TAX on **fizzy drinks** is the best way to tackle the childhood obesity epidemic, a study has concluded.

It would cut consumption and also raise vast sums to pay for obesity prevention.

Other options that could also help are after-school PE and a ban on TV advertising aimed at youngsters.

Experts in Washington said a US tax of one cent per ounce would have generated nearly £8billion in 2010.

They said: "Unfortunately, implementation of any of these policies in the near term is extremely unlikely."

LOAD-DATE: September 5, 2014

LANGUAGE: ENGLISH

PUBLICATION-TYPE: Newspaper

JOURNAL-CODE: DMR

Copyright 2014 MGN Ltd.
All Rights Reserved

Mail Online

MailOnline

August 23, 2014 Saturday 9:00 AM GMT

Give children smaller plates at mealtimes to restrict their food and cut obesity, says top health official

BYLINE: PRESS ASSOCIATION and DAMIEN GAYLE FOR MAILONLINE

SECTION: NEWS

LENGTH: 448 words

- Public Health England chief Duncan Selbie gives stark warning to parents
- The number of people with diabetes could treble in 20 years, he says
- He also hints at measures to tackle smoking in the home in front of children

Give your children their dinner on smaller plates to stop them from getting too fat, a top public official has said.

Duncan Selbie, head of Public Health England, warned that unless young people ate less and exercised more millions would suffer from poor health adults.

More than a third of schoolchildren in year six now rank as overweight, according to official statistics, and the number of people with diabetes in Britain could treble in the next 20 years, warned Mr Selbie.

He told the Daily Telegraph he learned a simple example of how to stop children getting fat from a Manchester mother who had been raised on the idea that one should always clear one's plate.

'The child (her son) was overweight at school, and it was a definite problem,' he said.

'The prescription was that they brought them smaller plates. The child was back on track: he went on to be ahead of the class.'

Just last month official figures showed nearly one in 10 four to five-year-olds starting school in England were obese, rising to almost one in five among youngsters getting ready to start secondary.

The growing obesity crisis among the nation's children has led to school uniform suppliers stocking XXXXXXXXL trousers with 50 inch waistlines to meet demand.

Tam Fry, from the National Obesity Forum, said this week that their research shows that in the next 15-20 years 50 per cent of the entire UK population will be overweight.

He said: 'It really is an an appalling situation we find ourselves in and very disturbing that we are seeing school uniforms going up to waist sizes of 50 inches.

'Unfortunately, a significant amount of adults do not eat properly and this is passed down to their children.

'Something drastic needs to be done to combat this.'

The Health and Social Care Information Centre said 45.1million prescription items - including insulin, anti-diabetic drugs and monitoring devices for the condition - were handed out to patients in England last year.

That is a more than 50 per cent rise on the number prescribed in 2005/6.

In a wide-ranging interview Mr Selbie also told the Telegraph minimum pricing for alcohol should be introduced and more **fizzy drinks** should be made sugar-free.

He praised the bans introduced on smoking in public places and in cars with children on board, but added that it is likely smoking in the home in front of children will be looked at next.

Referring to the rise in cases of tuberculosis, Mr Selbie said it was because the UK is such an 'open nation'.

LOAD-DATE: August 23, 2014

LANGUAGE: ENGLISH

PUBLICATION-TYPE: Web Publication

JOURNAL-CODE: WEBDM

Copyright 2014 Associated Newspapers Ltd.
All Rights Reserved

The Telegraph
telegraph.co.uk

August 22, 2014 Friday 10:00 PM GMT

Give children smaller plates to help them lose weight; Duncan Selbie, the head of Public Health England, says it is a national 'tragedy' that a third of 11-year-olds are overweight

BYLINE: By Steven Swinford Senior Political Correspondent

SECTION: NEWS

LENGTH: 967 words

Parents should consider giving children smaller plates at meal times to help them lose weight, the country's most senior public health official has said.

Duncan Selbie, the head of Public Health England, said it was a national "tragedy" that a third of 11-year-olds were overweight and warned that unless they ate less and exercised more, millions would suffer in later life.

In an interview with The Telegraph, Mr Selbie said that in many cases families could improve their child's health with simple, practical steps such as exercising together or giving them smaller plates to reduce the amount of food they ate.

He said he came across the idea after meeting the mother of an overweight child in Manchester, who grew up on the philosophy that "you have to clear your plate" and so always encouraged her child to finish their meal.

"The child was weighed at school, it was a definite problem," Mr Selbie said. "The mum was reasonably educated, but at a time where you had to clear your plate.

"The prescription was that they bought them smaller plate. The child was back on track, he went on to be ahead of the class. It's a small example of common sense."

Mr Selbie made a series of other suggestions to improve public health, including:

* Encouraging **fizzy drinks** manufacturers to make the majority of their products sugar-free;

* Discouraging parents from smoking at home in front of their children;

* Introducing minimum alcohol pricing and giving councillors the power to close bars and pubs if they damaged public heatlh

He said that according to new research, Britain's obesity crisis would result in the number of people with type 2 diabetes trebling over the next 20 years - eventiually reaching 6.2million by 2034.

"We know that the fatter you are, the more likely you are to get diabetes," Mr Selbie said. "The numbers are staggering. I remember in my class there was one very overweight child.

"I think that many of us would recognise that if you watch snakes of children on the underground you can see the weight change that has happened."

Mr Selbie, who has attracted controversy for his public health interventions in the past, said he was not trying to be an "evangelist" and tell people what to do, but wanted to help them make better choices.

He said that Britain had become "obsessed" with hospitals and the NHS, when in fact it should be more concerned with preventing illness.

"We conflate good health with the NHS," he said. "The Prime Minister does it, the secretary of state does it. The front line isn't the NHS, it's stopping it in the first place."

Sugar

Mr Selbie warned that for decades public health experts had created a "sense in the public's mind that it's all about fat", while failing to spell out the problems of eating too much sugar.

He said that as a result many low fat foods promoted as healthy options routinely contained high levels of sugar.

"We have seen a shift in how food manufacturing has replaced fat with sugar," he said. "Where in the public's mind low fat is good, they have not made the connection with what's in their product beyond that."

He called for food manufacturers to reformulate foods to reduce the levels of sugar, and suggested that they could make virtue out of marketing sugar-free products.

He highlighted how more than half of Pepsi's products sold in the UK were sugar free, and suggested that its rivals should follow suit.

Tobacco

Mr Selbie said that his own father "smoked and drank like a fish", adding that he had "no interest" in being evangelical about "what adults choose to do".

However, he argued that young people "who do not have a choice" needed greater protection. He said that he was "delighted" with the smoking ban in public places, cars with children present and the moves towards standardised packaging.

The "next step" was likely to be tackling smoking at home, he said.

Alcohol

Mr Selbie said he was "disappointed" by the Coalition's decision to put plans for minimum alcohol pricing on hold last year.

In 2011, the Coalition said it would enforce a ban on alcohol sales "below the rate of duty plus VAT".

The government went further in 2012, announcing that a minimum price of at least 40p per unit of alcohol should be introduced and David Cameron indicated his own support for the policy.

However, ministers put the plans on hold after saying there was not sufficient evidence that it would reduce excessive drinking.

Mr Selbie said: "Our issue is about children. Price drives the decision making of the young, and if you go to Blackpool you can buy a cider for less than a bottle of water. It is such an obvious harm that the government will in time have to address this."

Councils should be given new powers to shut down bars and pubs if they damage people's health, Mr Selbie said.

He said that premises selling alcohol should have to pass a "public health" test, enabling councils to shut them down if they were selling alcohol too cheaply.

Every local council in Britain had signed up to the proposal, which is currently with the Home Office. "It would be a big, big step forward," he said.

Tuberculosis

Mr Selbie also highlighted the rise in the number of cases of tuberculosis in Britain, which has been attributed to immigration.

He said that the number of cases of tuberculosis will soon exceed the number in the US for the first time, and take on an increasingly antibiotitc-resistant form.

"We're just about to cross over with the United States in having more cases of TB as a nation than the whole of the US. This is to do with Britain being the most open nation in the world. There has been a rise in resistant strains which take two years rather than six months to treat."

LOAD-DATE: August 23, 2014

LANGUAGE: ENGLISH

PUBLICATION-TYPE: Newspaper; Web Publication

JOURNAL-CODE: WEBDTNS

Copyright 2014 Telegraph Media Group Limited
All Rights Reserved

Daily Mirror

August 21, 2014 Thursday
Edition 1;
Northern Ireland

The sugar you can eat and still be healthy!

BYLINE: ANGELA DOWDEN

SECTION: FEATURES; Pg. 42,43

LENGTH: 1030 words

Unless you've been living under a rock lately, you can't have missed the stories about how we're eating too much sugar. Experts warn that sugary foods slip down all too easily, giving us extra, empty calories we don't need and contributing to the UK's obesity crisis. That's not to mention **sugary drinks** giving us all diabetes - and rotting our teeth.

In June, a draft report from the Government's Scientific Advisory Committee on Nutrition (SACN) proposed that our target maximum intake of 'free' sugars - defined as sugar added to food by the manufacturer or naturally present in honey, syrups, and juices - should be cut to 25g a day, or about half the currently recommended limit of 50g (12 level teaspoons of sugar).

In practical terms, you could reach 25g of free sugars with just two or three Jaffa Cakes and a cheese and pickle sarnie - not great if you have a sweet tooth. But there's brighter news, because other experts say there's no need to be forensic about removing all sugar from your family's diet.

Registered dietitian Penny Hunking says: "I am concerned that the report's recommendations are simply not achievable for many people.

"You can still be healthy if your free or added sugar intake is up to around 50g a day, which is the recommendation in place currently. Sugar at this intake is not associated with diabetes or obesity.

"It's about context and good everyday food choices can include a small amount of sugary foods that people enjoy."

With that in mind, here are some higher sugar foods it's OK to carry on eating...

Frosted Wheats

If you're not ready to give up on sweet cereals, pour a bowl of Frosted Wheats or Honey Nut Shredded Wheat, which are only sweetened on the outside and are still healthy high fibre options. Both our a bowl of Froste ney Nut Shredded re only sweetened on the outside and are still healthy high fibre options. Both provide around 6.5g free sugar in a 40g bowl, which is approximately an eighth of the current recommended daily maximum m and around 20 per cent less than in plain Cheerios.

Juice and smoothies

With juice containing as much free sugar as a full sugar cola, it's no wonder that it has come under scrutiny recently. But a small orange juice is an excellent supplier of vitamin C and will increase the amount of iron you absorb from your breakfast. Smoothies are healthier still, as they contain crushed whole fruits that add good amounts of fibre. The bottom line? While whole fruit is best, juice and smoothies are very convenient options and if you stick to just one small glass a day the benefits should generally outweigh the downsides.

Fruit yogurt

A study by Cambridge University scientists, published earlier this year, linked a weekly consumption of four and a half standard pots of low fat yogurt, including sweetened varieties like strawberry, with a 28 per cent lower risk of developing type two diabetes. Any kind of yogurt provides a good source of calcium, protein and the B vitamin riboflavin, important for energy release, so eating some regularly is more important than worrying about whether it contains a bit too much added sugar or not. Don't think it's healthier to sweeten yogurt yourself, either - 125g natural yogurt with two teaspoons of honey is more sugary than a pot of the ready sweetened fruit variety.

Milkshake

Similarly, adding sugar and flavouring to milk doesn't detract from the fact that it's a great source of dairy nutrients. Obviously, it's healthier to have milk straight, or to whizz up your own banana or berry milkshake, but if only commercially flavoured milk hits the spot, that's ber fla oka bra sug te's okay. A 200ml glass of supermarket own brand strawberry milk averages 19-20g sugar, of which around half (two teaspoons) is added, representing about a fifth of your daily free sugar limit. Studies show that chocolate milk can be really beneficial for recovery after a gruelling workout.

Dried fruit

Fruit that's dried has a much more concentrated sugar content than fresh, but it's still a healthy provider of fibre and minerals (vitamin C levels are reduced, though). Half the sugar in dried fruit is considered unhealthy 'free' sugar, but this definition is rather arbitrary and is under review. "A practical rule of thumb with dried fruit is to think of how much you are having in terms of its fresh fruit equivalence," says Penny Hunking. "A miniature pack of raisins is like being equal to a small bunch of grapes and is plenty enough!'

Tomato soup

Tomatoes whizzed and pureed into soup can contain quite high amounts of sugars regardless of whether any is added on top. A 300g bowl of Heinz Cream of Tomato contains 14.8g and the same serving of New Covent Garden Slow Roast Tomato has 13.8g. However, it's still good to tuck in as all types of tomato soup (fresh, canned or homemade) count as one of your five a day and contain loads of lycopene, which has been linked with lower risk of strokes.

Ketchup

A favourite target of the sugar police, tomato ketchup is another useful source of healthy lycopene. If you stick to one tablespoon (providing 4g of sugar) and don't plaster absolutely everything you eat with it you'll be fine. Or go for the reduced sugar and salt version with a much more modest total of 2.3g sugar per tablespoon.

Digestives

The UK's favourite biscuit actually contains only a middling amount of sugar, scoring amber on the traffic light system of labelling. If you stick to just one you'll be adding half a teaspoon of free sugar to your diet (a 20th of your daily limit) along with 71 calories and 1.5g saturated fat. As part of a balanced diet, that's very manageable.

Lemonade

"Full sugar" lemonades are often quite low in sugar compared with other non-diet **fizzy drinks**. For example, big brands R Whites and Schweppes contain 7.9g and 13.9g of sugar per 330ml can respectively, compared with a whopping 52g in a can of Old Jamaica Ginger beer. Lilt is another lower sugar option with 15g in a can. If you really aren't ready to swap straight over to water or a diet drink, these options with more modest amounts of sugar are a better option.

BY NUTRITIONIST ANGELA DOWDEN

There's no need to be forensic about removing all sugar from family diets

LOAD-DATE: August 21, 2014

LANGUAGE: ENGLISH

PUBLICATION-TYPE: Newspaper

JOURNAL-CODE: DMR

Copyright 2014 MGN Ltd.

All Rights Reserved

MailOnline

MailOnline

August 21, 2014 Thursday 4:03 PM GMT

Could a 'sugar tax' on soft drinks help tackle the obesity epidemic? Scientists find 20% price rise deters people and helps battle the bulge

BYLINE: LIZZIE PARRY FOR MAILONLINE

SECTION: HEALTH

LENGTH: 396 words

- South African study measured effect of a tax on the prevalence of obesity
- Researchers found 20% price rise resulted in 3.8% fall in obesity in men and 2.4% in women - equating to a fall of 220,000 obese adults

A tax raising the price of sugar sweetened drinks could help tackle the obesity epidemic, scientists have claimed.

Scientists at the University of Witwatersrand in Johannesburg, South Africa, measured the effect of a 20 per cent tax on soft drinks, on the prevalence of obesity in adults in the country.

The team found the effective price rise could act as one initiative, in a multi-faceted approach to preventing obesity, Medical Daily reported.

Researchers found that implementing the tax resulted in a 3.8 per cent reduction in obesity in men and a 2.4 per cent fall in women, equating to a fall of 220,000 obese adults in South Africa.

Author Mercy Manyema, said the onus is on governments to help improve the health of the population.

'It is the responsibility of the government to protect the health of its population,' she said.

'One way of doing so is through "nudging" people to make healthier and more sustainable choices.

'An SSB (**sugar** sweetened **beverage)** tax has the potential to do this in addressing obesity-related diseases.'

Senior researcher, Professor Karen Hofman, added: 'While SSBs alone may not be the only reason for an increase in body fat, these **fizzy drinks** do not contain any essential nutrients, have a high sugar content and a strong link to weight gain.

'Drinking just one SSB a day increases the likelihood of being overweight by 27 per cent for adults and 55 per cent for children.'

The report follows a recommendation by the South African Minister of Health, Dr Aaron Motsoaledi, on the need to regulate foods high in sugar, in order to tackle the overweight population, and the diseases related to obesity.

Author Aviva Tugendhaft, added: 'This is not surprising considering that one 330ml serving of a fizzy sweetened drink contains an average of eight teaspoons of sugar and the same size fruit juice contains an average of nine teaspoons of sugar.'

In the last 30 years, the number of obese adults in South Africa has soared, making the nation the fattest in sub-Saharan Africa.

More than half of adults are now considered to be overweight or obese - 42 per cent of women and 13 per cent of men.

The paper was published in the PLOS ONE journal.

LOAD-DATE: August 21, 2014

LANGUAGE: ENGLISH

PUBLICATION-TYPE: Web Publication

JOURNAL-CODE: WEBDM

Copyright 2014 Associated Newspapers Ltd.
All Rights Reserved

DAILY MAIL (London)

August 19, 2014 Tuesday

SUGAR SHOCKERS

LENGTH: 148 words

Which has more?

BY DAILY MAIL REPORTER

SUGAR is the new tobacco', and has been linked to obesity, type 2 diabetes and heart disease. The advice is to have no more than 90g a day, and only 50g should be added' sugar (equivalent to 10 tsp). But which of these has the most sugar?

ANSWER: The Volvic Juiced it looks like it might be just water and lemon juice, but actually has 35g sugar in a bottle, which is twice the amount in the same-sized bottle of Lucozade Sport (17.5g).

The sugar levels in most sports drinks tend to be kept down, as high sugar concentration can slow the absorption of fluid from the drink, which is a problem if you're trying to rehydrate during a workout.

But the Volvic product is just another sugared **soft drink**, far removed from plain water. The **sugar** in both drinks is largely added **sugar**.

Volvic Juiced Cloudy Lemon (500ml)

Lucozade Sport Orange (500ml)

© Daily Mail

LOAD-DATE: August 18, 2014

LANGUAGE: ENGLISH

PUBLICATION-TYPE: Papers

Daily Mirror

August 17, 2014 Sunday
Edition 1;
Northern Ireland

FAT CHANCERS; Supermarkets cut 5bn calories from ranges but experts say they STILL push unhealthy foods

BYLINE: STEPHEN HAYWARD

SECTION: NEWS; Pg. 26

LENGTH: 462 words

SUPERMARKETS have cut more than five billion calories from food products in the past four years.

They have reduced the fat and salt content of thousands of items - revamping ready meals, meat, fruit juices, sandwiches and sauces - according to a report by the Department of Health.

But campaigners say they have not done enough and want tough new rules to force stores to make food healthier. They are calling for an end to cheap deals - such as buy one get one free (BOGOF) - on unhealthy products.

TOUGH

The big stores promised to cut salt and sugar levels under the Government's "responsibility deal" on food and drink.

But Tam Fry, of the National Obesity Forum, said: "The responsibility deal will never work unless the Government makes everybody sign it. We need to get tough. Without any kind of compulsory action we are going to see the industry dragging its feet.

"These companies should commit to permanently reducing across all products." Britain's obesity crisis already costs the NHS over £5billion a year to treat. More than 60 per cent of adults and a third of children aged 10 and 11 are now said to be overweight or obese.

Under the responsibility deal, launched in 2011, supermarkets are also committed to cutting out unhealthy processed trans fats and spending a bigger slice of their marketing budgets on lowercalorie options.

According to a report seen by the Sunday Mirror, Asda has already removed nine tonnes of **sugar** from table sauces, cutting 36 million calories, while Tesco has reduced the number of calories ries from its juices and **soft drinks** by three billion. Sainsbury's has cut 244 tonnes of fat from minced beef, saving two million calories.

Morrisons has cut 21,776 tonnes of fat from products, and reduced its calorie count by one billion. As well as an end to BOGOF deals on unhealthy items, campaigners want supermarkets to ban sweets and chocolate from checkouts. So far only Tesco and Lidl have agreed to do this.

Linda Hindle, of the British Dietetic Association, said: "Supermarkets could be doing a lot more to tackle obesity. They are in a position to make a really big impact."

stephen.hayward@sundaymirror.co.uk

Calorie counters ALDI - 4.2m calories cut in a year by swapping chicken tikka meal for new healthier version.

ASDA - 36m fewer calories after removing 9 tonnes of sugar from sauces.

CO-OP - removing added **sugar** from its **soft drinks** has saved 1.5bn calories.

MORRISONS - reducing salt and fat in own-label products has cut calories by 1bn.

SAINSBURY'S - 244 tonnes of fat cut from minced-beef products means 2 million fewer calories.

TESCO - 3bn calories stripped from own-brand juices and soft drinks since 2010.

WAITROSE - 7.1 tonnes of sugar cut from chilled fruit juices, resulting in 28m fewer calories over the past three years.

LOAD-DATE: August 17, 2014

LANGUAGE: ENGLISH

GRAPHIC: Choice cuts: stores reduce calorie counts

PUBLICATION-TYPE: Newspaper

JOURNAL-CODE: SMR

Copyright 2014 MGN Ltd.

All Rights Reserved

The Express

August 16, 2014 Saturday

Edition 1;
National Edition

Monster deal for Coca-Cola; City & Business Edited by PETER CUNLIFFE email: city@express.co.uk Visit City & Business pages online at www.express.co.uk/city Tel: 020 8612 7162

BYLINE: PETER CUNLIFFE

SECTION: BUSINESS; Pg. 67

LENGTH: 92 words

COCA-COLA has paid £1.3billion to buy a stake in energy drinks company Monster as it looks to further diversify its portfolio from Coke and other **fizzy drinks.**

Coke has bought the 16.7 per cent holding in its fellow US company as it seeks to grow its market share in the energy drinks market, which is valued at £16billion globally according to EuroMonitor International.

As part of the deal, Coke will transfer ownership of its energy drinks brands, which includes Relentless, to Monster. In return Monster will transfer its non-energy drinks to Coke.

LOAD-DATE: August 16, 2014

LANGUAGE: ENGLISH

PUBLICATION-TYPE: Newspaper

JOURNAL-CODE: DXP

Copyright 2014 Express Newspapers
All Rights Reserved

Financial Times (London, England)

August 15, 2014 Friday
London Edition 3

Coke to pay $2.2bn for stake in energy drink maker Monster

BYLINE: Elizabeth Paton in New York

SECTION: FRONT PAGE - COMPANIES & MARKETS; Pg. 13

LENGTH: 302 words

Coca-Cola is paying $2.15bn for a 16.7 per cent stake in Monster, sending shares in the world's leading energy drink maker up 22 per cent in after-hours trading in New York.

Coke said yesterday that it would make a cash payment for the stake, with rights to two seats on the board. Analysts believe the move could eventually lead to a full buyout.

As part of the deal, Monster, which has long been a subject of takeover speculation, will absorb Coke's worldwide energy drinks business into its operations. Monster will then move its non-energy business to Coke and the companies will enter expanded distribution agreements. Monster is issuing new shares to accommodate Coke's investment.

Analysts have estimated that energy drink sales could hit $21.5bn by 2017, up from $12.5bn in 2012. Monster is the dominant player in the sector, with a 42 per cent market share.

"Coca-Cola continues to identify innovative approaches to partnerships that enable us to stay at the forefront of consumer trends," said Muhtar Kent, chief executive.

"Our equity investment in Monster is a capital-efficient way to bolster our participation in the fast-growing . . . global energy drinks category."

As customers in mature markets become more healthconscious, Coke, the world's largest drinks maker, has grappled with a decline in **fizzy drink** sales , its core revenue driver. It has turned its attentions towards expanding its beverage brand portfolio , with stakebuilding in and takeovers of small and popular alternative drinks brands.

"This is a very smart move by Coca-Cola, giving it critical exposure in the energy drinks sector," said Ali Dibadj, analyst at Sanford Bernstein. Speculation about a deal had been rife on Wall Street for months, he added.

Shares in Coca-Cola rose 1.3 per cent to $40.70 in after-hours trading in New York.

LOAD-DATE: August 14, 2014

LANGUAGE: ENGLISH

PUBLICATION-TYPE: Newspaper

Copyright 2014 The Financial Times Ltd.
All Rights Reserved
Please do not cut and paste FT articles and redistribute by email or post to the web

MailOnline

August 15, 2014 Friday 2:56 PM GMT

'Thanks Michelle!': Disgusted teens across the country pose alongside new healthy vending machines in their schools to protest the removal of their favorite treats

BYLINE: JAMES GORDON

SECTION: NEWS

LENGTH: 873 words

- First Lady has introduced new rules that saw companies limited in what products they could advertise on signs, vending machines, cups and menu boards on school grounds
- Food industry spent nearly $150 million in 2009 on marketing in schools, 93 percent was promoting drinks
- Schools now require drink companies to promote their diet sodas or water on signs and scoreboards, rather than full-calorie options like Coca-Cola and Pepsi
- Michelle Obama's Let's Move program is now in its fourth year

Students returning back to school after the summer vacation are finding out what First Lady Michelle Obama's new rules for healthy eating in schools, truly mean.

Gone are the the signs marketing high-sugar sodas to thirsty kids, so too are the fattening chips and chocolate.

Instead, those having cravings for all-things-fattening will have to make do with protein bars, vegetable snacks, bottles of mineral water and diet soda.

Mrs Obama's new rules which were approved by her husbands administration now see vending machines across the country featuring healthier options in an overall effort to fight obesity.

Scroll down for video

With many schools back in session, teens have taken to Twitter to vent their frustration at the lack of options that the new vending machines provide.

'Michelle Obama is single-handedly ruining my life by changing school lunch and the vending machines,' tweeted one student who was craving calories.

'How about Michelle Obama quit worrying about the vending machines and worry about how terrible school lunches are. Like that's cardboard,' tweeted another angry student.

Some students seem to have taken matters into their own hands, however, and are starting to bring in their favorite sugary snacks from outside of school.

'Smuggling junk food in my purse to school because there's only healthy food in the vending machines,' wrote one Twitter user.

The new rules phase out the advertising of **sugary drinks** and junk foods around campuses during the school day and ensure that other promotions in schools are in line with health standards that already apply to school foods.

That means a scoreboard at a high school football or basketball game eventually is no longer allowed to advertise Coca-Cola, for example, but it could advertise Diet Coke or Dasani water, which is also owned by Coca-Cola Co.

The same rules apply to the front of vending machines. Cups, posters and menu boards which promote foods that don't meet the standards have been phased out.

Ninety percent of such marketing in schools is related to beverages, and in advance of the new rules coming into effect, many soda companies already began to transition their sales and advertising in schools from sugary sodas and sports drinks to their own healthier products.

The rules are part of first lady Michelle Obama's Let's Move initiative to combat child obesity, which is now in its fourth year.

'The idea here is simple - our classrooms should be healthy places where kids aren't bombarded with ads for junk food,' the first lady said in a statement released before the announcement.

"Because when parents are working hard to teach their kids healthy habits at home, their work shouldn't be undone by unhealthy messages at school.'

The rules also would allow more children access to free lunches and ensure that schools have wellness policies in place.

The rules come on the heels of USDA regulations that are now requiring foods in the school lunch line to be healthier.

Calorie, fat, sugar and sodium limits have to be met on almost every food and beverage sold during the school day at 100,000 schools. Concessions sold at afterschool sports games are exempt.

The healthier food rules have come under fire from conservatives who think the government shouldn't dictate what kids eat - and from some students who don't like the healthier foods.

Aware of the backlash, the USDA is allowing schools to make some of their own decisions on what constitutes marketing and asking for comments on some options.

Rules for other school fundraisers, like bake sales and marketing for those events is still up to individual schools or states.

Off-campus fundraisers, like an event at a local fast-food outlet that benefits a school, are still permitted.

But posters advertising the fast food are no longer allowed in school hallways.

The rules also makes allowances for major infrastructure costs - that scoreboard advertising Coca-Cola, for example, won't have to be immediately be torn down, but school has to get one with a healthier message the next time its replaced.

The beverage industry - led by Coca-Cola Co., Dr. Pepper Snapple Group and PepsiCo - is on board with the move. American Beverage Association President and CEO Susan Neely said in a statement that aligning signage with the healthier drinks that will be offered in schools is the 'logical next step.'

'Mrs. Obama's efforts to continue to strengthen school wellness make sense for the well-being of our schoolchildren,' Neely said.

Although Mrs. Obama lobbied Congress to pass the school nutrition bill in 2010, most of her efforts in recent years have been focused on the private sector, building partnerships with food companies and retailers to sell healthier foods.

LOAD-DATE: August 15, 2014

LANGUAGE: ENGLISH

PUBLICATION-TYPE: Web Publication

JOURNAL-CODE: WEBDM

Copyright 2014 Associated Newspapers Ltd.
All Rights Reserved

The Daily Telegraph

The Daily Telegraph (London)

August 14, 2014 Thursday
Edition 1;
National Edition

How putting on weight can lead to cancer; Calls for a tax on unhealthy food to help prevent cancer

BYLINE: Sarah Knapton

SECTION: NEWS; Pg. 1,2

LENGTH: 815 words

BRITAIN'S obesity epidemic is causing more than 12,000 cases of cancer every year, according to the largest study of its kind that found gaining weight can increase the risk of the disease by up to two thirds.

The study, which monitored more than 5.2 million Britons over seven years, shows that just a few pounds of extra weight can increase a person's risk of developing 10 common cancers.

The findings have prompted calls for a tax on calorific foods and campaigners warn that the benefits of recent medical breakthroughs and screenings could be wiped out in a decade as thousands more people are diagnosed with cancer because of unhealthy lifestyles.

The study found that for every extra two stone women gained, their risk of womb cancer rose by 62 per cent, while the risk of cervical cancer rose by 10 per cent and ovarian cancer by nine per cent. For every two and a half stone gained by men, and two stone by women, the risk of kidney or gall bladder cancer rose by 31 per cent, while liver cancer rose by 19 per cent and leukaemeia by nine per cent.

The researchers estimate that about 12,000 cancers each year are a direct result of weight gain or obesity, but they predict that diagnoses will rise to more than 15,700 within 12 years. "People are generally quite aware that being overweight increases the risk of diabetes and cardiovascular disease but they still don't know about the important relationship to cancer," said study leader Dr Krishnan Bhaskaran of the London School of Hygiene & Tropical Medicine.

"The number of people who are overweight or obese is rapidly increasing both in the UK and worldwide.

"Our results show that if these trends continue, we can expect to see substantially more cancers as a result."

The study looked at records from the UK's Clinical Practice Research Datalink, a database of anonymous records from clinics, GPs surgeries and laboratories.

More than 5.2 million people over 16, who were cancer-free, were followed for about seven and a half years. A total of 166,955 people developed cancer in that period and changes in Body Mass Index (BMI), a measure of obesity, were found to be linked to 17 types of disease, after adjusting for individual factors such as age, sex, smoking status, and socioeconomic status.

"There was a lot of variation in the effects of BMI on different cancers," added Dr Bhaskaran. "For example, risk of cancer of the uterus increased substantially at higher body mass index; for other cancers, we saw more modest increases in risk, or no effect at all."

The researchers estimate that excess weight could account for 41 per cent of uterine and 10 per cent of gall bladder, kidney, liver, and colon cancers in the UK.

They predict that within 12 years the population will have gained on average nine pounds per adult, which will result in 3,790 more cancer cases each year.

Health experts and campaigners said the Government must to do more to help people become healthier and penalise companies who sell unhealthy foods.

Last year a report by Oxford University found that a 20 per cent tax on **sugary drinks** would reduce the number of obese adults in the UK by 180,000.

"We have to ditch this love-in with the food industry and start penalising those who continue to make unhealthy food." said Tam Fry of the National Obesity Forum. "We are now starting to reap the rewards of decades of inaction and unwillingness to do anything about obesity and its consequences.

"The public is still not aware of the harm that being overweight or obese does and we have become too worried with making fat people thin rather than preventing people getting that way in the first place. From the earliest age, children should be educated at school about the dangers."

One in four adults in England is obese and this is likely to rise to 60 per cent of men, and 50 per cent of women, by 2050. Three in every 10 children between two and 15 are currently overweight or obese.

Obesity and diabetes already cost the UK more than £5billion every year, and the figure is likely to rise to £50 billion in the next 36 years.

"We have sufficient evidence that obesity is an important cause of unnecessary suffering and death from many forms of cancer," said Dr Peter Campbell from the American Cancer Society, Atlanta, USA, who wrote an accompanying commentary in The Lancet. "More research is not needed to justify, or even demand, policy changes."

Dr Campbell recommended taxing calorific foods and subsidising healthier food. "We need a political environment, and politicians with sufficient courage, to implement such policies," he added.

However, the Department of Health yesterday ruled out a tax on high-calorie foods, or **sugary drinks**: "We are not considering a new tax on particular foods, instead we are helping families to eat healthily and exercise more. We are also working with industry to reduce calories in food and drinks."

LOAD-DATE: August 14, 2014

LANGUAGE: ENGLISH

PUBLICATION-TYPE: Newspaper

JOURNAL-CODE: DTL

Copyright 2014 Telegraph Media Group Limited
All Rights Reserved

The Telegraph
telegraph.co.uk

August 14, 2014 Thursday 12:00 AM GMT

Obesity epidemic fuelling 12,000 cancer cases a year; A study of more than five million Britons has found that the excess weight increases the risk of developing ten common cancers by up to 62 per cent,

BYLINE: By Sarah Knapton Science Correspondent

SECTION: NEWS

LENGTH: 813 words

The obesity epidemic is fuelling more than 12,000 cases of cancer every year and diagnoses will soar unless urgent action is taken, health experts have warned.

Excess weight increases the risk of developing ten common cancers by up to 62 per cent, the biggest ever study has shown, prompting calls for a tax on calorific foods.

Campaigners have warned that the benefits of recent medical breakthroughs and screenings could be wiped out in a decade as thousands more people are diagnosed with cancer because of unhealthy lifestyles.

The landmark study followed more than five million Britons over more than seven years, monitoring their Body Mass Index (BMI) and health.

It found that for every extra two stone that women gained or two and a half stone for men, the risk of kidney or gall bladder cancer rose by 31 per cent, liver by 19 per cent and leukaemia by nine per cent.

For women the risk of womb cancer rose by 62 per cent, ovarian cancer by nine per cent and cervical cancer by 10 per cent.

"People are generally quite aware that being overweight increases the risk of diabetes and cardiovascular disease but they still don't know about the important relationship to cancer," said study leader Dr Krishnan Bhaskaran of the London School of Hygiene & Tropical Medicine.

"The number of people who are overweight or obese is rapidly increasing both in the UK and worldwide.

"Our results show that if these trends continue, we can expect to see substantially more cancers as a result."

The researchers estimate that around 12,000 cancers each year are a direct result of weight gain or obesity. But they predict that diagnoses will rise by more than 3,700 within 12 years.

The study looked at records from the UKs Clinical Practice Research Datalink, a database of anonymous records from clinics, GPs surgeries and labs.

More than 5.2 million over 16s, who were cancer-free, were followed for around 7.5 years.

A total of 166 955 people developed cancer in that period and BMI was found to be linked to 17 types of disease, after adjusting for individual factors such as age, sex, smoking status, and socioeconomic status.

"There was a lot of variation in the effects of BMI on different cancers," added Dr Bhaskaran.

"For example, risk of cancer of the uterus increased substantially at higher body mass index; for other cancers, we saw more modest increases in risk, or no effect at all."

The researchers estimate that excess weight could account for 41 per cent of uterine and 10 per cent of gallbladder, kidney, liver, and colon cancers in the UK.

They predict that within 12 years the population will have gained on average nine pounds per adult which will result in an additional 3790 cancer cases each year.

Health experts and campaigners said the government must to do more to help people adopt healthier lifestyles and penalise companies who sell unhealthy foods.

Last year a report by Oxford University found that a 20 per cent tax on **sugary drinks** would reduce the number of obese adults in the UK by 180,000.

"We have to ditch this love-in with the food industry and start penalising those who continue to make unhealthy food." said Tam Fry of the National Obesity Forum.

"We are now starting to reap the rewards of decades of inaction and unwillingness to do anything about obesity and its consequences.

"The public is still not aware of the harm that being overweight or obese does and we have become too worried with making fat people thin rather than preventing people getting that way in the first place.

"From the earliest age, children should be educated at school about the dangers, otherwise we are just mopping up the water without turning off the tap."

One in four adults in England is obese and these figures are set to climb to 60 per cent of men, 50 per cent of women, by 2050.

Three in every 10 children aged between two and 15 are currently overweight or obese.

Obesity and diabetes already costs the UK over £5billion every year which is likely to rise to £50 billion in the next 36 years.

"We have sufficient evidence that obesity is an important cause of unnecessary suffering and death from many forms of cancer," said Dr Peter Campbell from the American Cancer Society, Atlanta, USA, who wrote an accompanying commentary in The Lancet.

"More research is not needed to justify, or even demand, policy changes aimed at curbing overweight and obesity.

Dr Campbell recommended taxing calorific foods and providing subsidies for healthier food.

"We need a political environment, and politicians with sufficient courage, to implement such policies effectively," he added.

However the Department of Health yesterday ruled out a tax on high calorie foods, or **sugary drinks**.

"We are not considering a new tax on particular foods, instead we are helping families to eat healthily and exercise more.

"We are also working with industry to reduce calories in food and drinks."

LOAD-DATE: August 14, 2014

LANGUAGE: ENGLISH

PUBLICATION-TYPE: Newspaper; Web Publication

JOURNAL-CODE: WEBDTNS

Copyright 2014 Telegraph Media Group Limited
All Rights Reserved

MailOnline

August 12, 2014 Tuesday 11:54 AM GMT

Tropical treat we've all gone nuts for: Coconut water is low calorie and full of minerals. No wonder sales are up 120% in a year

BYLINE: ANNE SHOOTER

SECTION: FOOD

LENGTH: 727 words

- Drink tastes like slightly sweet, slightly salty water and is high in minerals
- Minerals are depleted after exercise and is often sold as post-workout drink
- It has become Britain's fastest-growing soft drink with 12.5 million sold

Coconut water has become Britain's fastest-growing **soft drink**. It's made by extracting the milky fluid inside unripe green coconuts (this turns to milk when the coconut is ripe) and can be drunk as is or flavoured with **sugar** and fruit juice.

The drink, which tastes like slightly sweet, slightly salty water, is low in calories and said to be high in minerals such as potassium and magnesium, which are vital for muscle movement, nerve transmission and brain operation.

These minerals are depleted after exercise, so coconut water is often sold as a post-workout drink.

Scroll down for video

Sales are up 114 per cent since last year, according to internet shopping experts mySupermarket, and there are 36 brands available in Britain.

The market leader, Vita Coco, has grown 120 per cent over the past 12 months, with growth doubling every year since it launched four years ago.

Last year, 12.5 million Vita Coco drinks were sold in Britain.

The company was launched by two childhood friends from New York. They had met two Brazilian women in a bar, who told them they really missed drinking coconut water, which is hugely popular in Rio de Janeiro.

Soon afterwards, the pair launched Vita Coco, which is now available in 75,000 stores in 13 countries around the world.

While its popularity continues to grow, critics say that the benefits are hugely hyped, as the drink is 95 per cent water. They also say it is only slightly more effective than water as a drink after exercise, with many sports drinks higher in minerals. However, with only a small amount of sugar, it is lower in calories than most sports drinks.

Coke and Pepsi each own coconut water brands - Zico and O.N.E, respectively - after jumping on the bandwagon on the back of Vita Coco's huge success.

In the past year, sales of coconut water rocketed by 71 per cent at Ocado and 55 per cent at Tesco.

Matt Clark, Tesco's world foods buying manager, says: 'We started selling coconut water in 2008 when it was still very much a niche drink.

'But in the past year sales have soared. Demand tends to rise whenever a celebrity such as Rihanna is photographed holding a coconut drink.' M&S has launched its own range this year, at £2.99 a litre, and their most popular variety, the unflavoured M&S coconut water, has been selling 17,000 litres a week during the summer.

They use coconut water from Indonesia and spent a year perfecting their recipes.

Alex Beckett, of retail analysts Mintel, says: 'Coconut water has been a runaway success story in the UK since 2010. Heightened awareness of the nutritional benefits of coconut water has driven interest in this drink.'

Introducing sneeze and stain-free lilies

We all know that being given lilies can be a mixed blessing. Yes, they look beautiful, but they make many of us sneeze. And those yellow pollen stains!

Despite this, they are one of the nation's favourite flowers, with nearly 19 million bunches sold every year.

But their high pollen content makes them one of the worst culprits for hay fever.

Now Marks & Spencer has a solution: a lily bouquet which is stain free and sneeze free. In a High Street first, the retailer has launched the Autograph Lily Bouquet, comprising of special Vendrome lilies, which have pollen that doesn't fall from the flower.

More than 12 million people in Britain suffer from the allergy, and the recent warm and wet weather has seen symptoms worsen as pollen levels soar to the worst seen for a decade.

M&S flower expert Simon Richards said: 'Lilies are one of the most sneeze-inducing flowers, and hay fever sufferers tend to steer clear of them.

'The benefits of our new bouquet are two-fold. The blooms won't trigger hay fever, and customers can enjoy the lilies without pollen shedding and staining.'

Vendrome lilies have been grown over eight years after the bulbs were found by chance. They are now available exclusively at M&S.

This autumn, the retailer also plans to launch Autograph Double Lilies: hybrid blooms which have a double blossom and don't contain stamens, so are completely pollen free.

The Autograph Lily Bouquet is priced at £35 and available exclusively online until the end of August.

LOAD-DATE: August 12, 2014

LANGUAGE: ENGLISH

PUBLICATION-TYPE: Web Publication

JOURNAL-CODE: WEBDM

Copyright 2014 Associated Newspapers Ltd.
All Rights Reserved

i-Independent Print Ltd

August 11, 2014
First Edition

on marketing and branding; How could Coca Cola get it so wrong?

BYLINE: Claire Beale

SECTION: FEATURES; Pg. 41

LENGTH: 314 words

Next week: Danny Rogers on PR and advertising There was a time when big companies could do almost anything they liked to the brands we love - change the name, the size, the packaging - and all we could do was like it, or write to complain.

No longer, as Coca Cola found out last week. The drinks giant has been meddling with the ingredients of its Vitaminwater brand in the US, but when consumers started detecting a strange taste to their favourite drink, battle commenced.

Coca Cola has been coming under fire for peddling products that are making us obese, so a few months ago it replaced the crystallised fructose in Vitaminwater with natural sweetener Stevia. But the firm didn't explain the switch to consumers, who quickly took to social media to make it clear they wouldn't be buying the brand again. By last week Coca Cola had been forced into an embarrassing U-turn, saying: "The fans have spoken. We're changing back to the taste you know and love."

You'd think Coca Cola might have learnt its lesson from the New Coke farrago back in the '80s, when the formula for the iconic drink was re-engineered and met with such a consumer backlash that within a couple of months the company had to admit it got it wrong and New Coke was replaced with Classic Coke.

For all the money that big brands spend on researching consumers, it's astounding how it can go so badly wrong. The marketing history books are full of examples, from the shortlived £2m rebranding of the Post Office Group to Consignia in 2001 to British Airways' unpopular decision to colour its tail fins with vibrant ethnic designs.

These days social media speeds up and globalises consumer backlash; and U-turns tend to be swifter. But in an age when brands have a real-time dialogue with their consumers, there's really no excuse for getting it wrong in the first place.

Claire Beale is brand editor of Marketing magazine

LOAD-DATE: August 10, 2014

LANGUAGE: ENGLISH

PUBLICATION-TYPE: Newspaper

JOURNAL-CODE: III

Copyright 2014 Independent Print Limited
All Rights Reserved

MailOnline

August 11, 2014 Monday 11:31 PM GMT

Tropical treat we've all gone nuts for: Coconut water is low calorie and full of minerals. No wonder sales are up 120% in a year

BYLINE: ANNE SHOOTER

SECTION: FOOD

LENGTH: 723 words

- Drink tastes like slightly sweet, slightly salty water and is high in minerals
- Minerals are depleted after exercise and is often sold as post-workout drink
- It has become Britain's fastest-growing soft drink with 12.5 million sold

Coconut water has become Britain's fastest-growing **soft drink**. It's made by extracting the milky fluid inside unripe green coconuts (this turns to milk when the coconut is ripe) and can be drunk as is or flavoured with **sugar** and fruit juice.

The drink, which tastes like slightly sweet, slightly salty water, is low in calories and said to be high in minerals such as potassium and magnesium, which are vital for muscle movement, nerve transmission and brain operation.

These minerals are depleted after exercise, so coconut water is often sold as a post-workout drink.

Sales are up 114 per cent since last year, according to internet shopping experts mySupermarket, and there are 36 brands available in Britain.

The market leader, Vita Coco, has grown 120 per cent over the past 12 months, with growth doubling every year since it launched four years ago.

Last year, 12.5 million Vita Coco drinks were sold in Britain.

The company was launched by two childhood friends from New York. They had met two Brazilian women in a bar, who told them they really missed drinking coconut water, which is hugely popular in Rio de Janeiro.

Soon afterwards, the pair launched Vita Coco, which is now available in 75,000 stores in 13 countries around the world.

While its popularity continues to grow, critics say that the benefits are hugely hyped, as the drink is 95 per cent water. They also say it is only slightly more effective than water as a drink after exercise, with many sports drinks higher in minerals. However, with only a small amount of sugar, it is lower in calories than most sports drinks.

Coke and Pepsi each own coconut water brands - Zico and O.N.E, respectively - after jumping on the bandwagon on the back of Vita Coco's huge success.

In the past year, sales of coconut water rocketed by 71 per cent at Ocado and 55 per cent at Tesco.

Matt Clark, Tesco's world foods buying manager, says: 'We started selling coconut water in 2008 when it was still very much a niche drink.

'But in the past year sales have soared. Demand tends to rise whenever a celebrity such as Rihanna is photographed holding a coconut drink.' M&S has launched its own range this year, at £2.99 a litre, and their most popular variety, the unflavoured M&S coconut water, has been selling 17,000 litres a week during the summer.

They use coconut water from Indonesia and spent a year perfecting their recipes.

Alex Beckett, of retail analysts Mintel, says: 'Coconut water has been a runaway success story in the UK since 2010. Heightened awareness of the nutritional benefits of coconut water has driven interest in this drink.'

Introducing sneeze and stain-free lilies

We all know that being given lilies can be a mixed blessing. Yes, they look beautiful, but they make many of us sneeze. And those yellow pollen stains!

Despite this, they are one of the nation's favourite flowers, with nearly 19 million bunches sold every year.

But their high pollen content makes them one of the worst culprits for hay fever.

Now Marks & Spencer has a solution: a lily bouquet which is stain free and sneeze free. In a High Street first, the retailer has launched the Autograph Lily Bouquet, comprising of special Vendrome lilies, which have pollen that doesn't fall from the flower.

More than 12 million people in Britain suffer from the allergy, and the recent warm and wet weather has seen symptoms worsen as pollen levels soar to the worst seen for a decade.

M&S flower expert Simon Richards said: 'Lilies are one of the most sneeze-inducing flowers, and hay fever sufferers tend to steer clear of them.

'The benefits of our new bouquet are two-fold. The blooms won't trigger hay fever, and customers can enjoy the lilies without pollen shedding and staining.'

Vendrome lilies have been grown over eight years after the bulbs were found by chance. They are now available exclusively at M&S.

This autumn, the retailer also plans to launch Autograph Double Lilies: hybrid blooms which have a double blossom and don't contain stamens, so are completely pollen free.

The Autograph Lily Bouquet is priced at £35 and available exclusively online until the end of August.

LOAD-DATE: August 11, 2014

LANGUAGE: ENGLISH

PUBLICATION-TYPE: Web Publication

JOURNAL-CODE: WEBDM

Copyright 2014 Associated Newspapers Ltd.
All Rights Reserved

THE TIMES
The Times (London)

August 6, 2014 Wednesday
Edition 2;
National Edition

That wasn't it, admits Coke in recipe U-turn

SECTION: NEWS; Pg. 37

LENGTH: 458 words

Dear Coca-Cola, next time you think of changing the ingredients in one of your most popular drinks, you might want to taste it first."

Customer reaction to a new recipe for Coke's Vitaminwater has been so bad the company has bowed to demand to switch back (Alexandra Frean writes).

The U-turn came Just months after the Atlanta-based company changed the sweetener in the drink to a mix of sugar and stevia, a lowcalorie sweetener with a metallic aftertaste, from the original sugar and crystalline fructose.

Customers reacted as bitterly as stevia is alleged to taste.

Recipes for disaster ? Coca-Cola backtracked after the introduction of New Coke in 1985, which had a change in formula and flopped. The reintroduction of the original formula, rebranded Coca-Cola Classic, led to a surge in sales.

? Heinz introduced green ketchup in 2000. That lasted six years. ? In 1993, Miller stopped brewing Miller Clear, the first colourless beer. Pepsi tried something similar in 1992, with Crystal Pepsi.

? Flora withdrew a new recipe for its margarine in 2003 after complaints. ? Twinings changed its Earl Grey to add more lemon and "an extra hint of bergamot" in 2011.

"Terrible," said many. "I am miserable and cannot remove the disgusting chemical taste of stevia from my mouth" a former fan complained.

Coke admitted "our fans haven't had the greatest things to say about it. So we're changing back to the taste you know and love." The original version will start reappearing on shelves this autumn.

Coke's decision to introduce stevia into Vitaminwat er came after its sales fell by 18 per cent in 2013, says Beverage Digest, an industry tracker.

Having to backtrack is an embarrassment for Coke, which has announced plans to introduce a stevia-sweetened version of its flagship drink, to be called Coca-Cola Life, in the US and Britain in September. The product has already been made available in Argentina.

"With Coca-Cola Life, we have innovated to provide consumers with a new option with fewer calories," James Quincey, president of Coca-Cola Europe, said.

Its launch was the latest in a series of initiatives to promote wellbeing and address obesity in the UK "by providing consumers with beverages for every lifestyle and occasion".

While US sales have been falling for more than ten years, Coke has always been able to rely on overseas sales for growth.

Now, however, the pace of global soda sales has been in decline for three years in a row, according to The Wall Street Journal. This is adding to the pressure for the company to refocus on North America, where it has shaken up management and boosted marketing.

Rob Frankel, an independent branding expert, said Coke's response to complaints meant that the episode was unlikely to do serious long-term harm.

LOAD-DATE: August 6, 2014

LANGUAGE: ENGLISH

GRAPHIC: Paris Hilton promoted Vitaminwater; below, rapper 50 Cent was given a stake in the brand in a deal with Coca-Cola
RICHARD YOUNG/REX FEATURES ; RICK DIAMOND/GETTY IMAGES FOR BET

PUBLICATION-TYPE: Newspaper

JOURNAL-CODE: TIM

Copyright 2014 Times Newspapers Limited
All Rights Reserved

The INDEPENDENT
Independent.co.uk

October 1, 2014 Wednesday 6:07 PM GMT

Pepsi Cola launches new stevia-based soft drink 'Pepsi True'; Pepsi True has 30% less calories than normal Pepsi and follows the release of Coca-Cola Life, also made using stevia

BYLINE: Maria Tadeo

SECTION: FRONTPAGE

LENGTH: 210 words

Pepsi has announced it is to go head-to-head with Coca Cola with the launch of a new stevia-based soft drink marketed as a healthy alternative.

The new Pepsi True is sweetened using the naturally occurring sweetener extracted from a South American plant, and will be sold exclusively on Amazon.com in a major departure from the typical distribution chain in the beverage sector.

"It's a brand-new product proposition with a brand-new media platform, and we want to make sure the launch reflects a right-size approach," Simon Lowden, chief marketing officer at Pepsi Beverages North America.

The American beverage giant added the market for mid-calorie drinks - those containing more calories than its "diet" counterpart - remains small, and that Pepsie True would be a niche product as part of the wider portfolio that includes Pepsi Max and Diet Pepsi.

Business news in pictures

According to Pepsi, the new drink contains 30 per cent less calories than normal Pepsi and it does not use artificial sweeteners or high-fructose corn syrup, unlike most soft drinks in the market.

Pepsi True comes shortly after Cola-Cola launched its own stevia-based, healthier drink, Coca-Cola Life, in what's already being dubbed "the stevia wars" as consumers look for healthier options.

LOAD-DATE: October 1, 2014

LANGUAGE: ENGLISH

PUBLICATION-TYPE: Newspaper; Web Publication

JOURNAL-CODE: WEBI

Copyright 2014 Independent Digital News and Media Limited
All Rights Reserved

The INDEPENDENT
Independent.co.uk

October 1, 2014 Wednesday 4:27 PM GMT

How fruit drinks are causing tooth decay in toddlers; Juice drinks aimed at infants are simply not necessary and only contribute to children developing a sweet tooth

BYLINE: Melanie Wakeman

SECTION: FEATURES

LENGTH: 730 words

More than one in ten children under the age of three have tooth decay according to statistics published by Public Health England. The figures show that an average of three teeth in these children were decayed, missing or filled. This is shocking, although not surprising considering children of all age groups exceed the the current recommendations for added sugar.

The World Health Organisation recommends we limit our sugar intake to 10 per cent of our total calorie intake a day because of the negative effect that sugar has on our health. Boys and girls around the age of three require around 1,100 calories a day, which means no more than 5.5 teaspoons of added sugar to their daily diet. This may seem a lot but the figure is likely to soon be halved by the Scientific Advisory Committee on Nutrition (which advises on public health in England), limiting intake of added sugar to just five per cent of calorie intake.

According to the latest survey of national diet and nutrition, children and teenagers consume around 40 per cent more added **sugar** than the recommended daily allowance. And fruit juice and **soft drinks** are the main culprits.

Why fruit juice isn't so innocent

Fruit juice has had to bear the brunt of a large amount of negative press recently. Although fruit juice is rich in "natural sugar", sugar is sugar as far as the body is concerned. The sugar content of a glass of apple juice is not much different to that in a glass of cola and the damage it can cause to teeth is massive.

We are constantly reminded that fruit juice contributes to one of our "five-a-day" of fruit and vegetables and clever marketing means it looks like one of the healthiest options - but unfortunately it isn't. Current advice is to consume no more than 150ml of fruit juice per day. This is equivalent to around two and a half oranges. I doubt a young child could eat this many oranges so they should not drink that amount of juice (or sugar) instead.

Fussy eating phases commonly start around the age of two so drinks are often seen as an easy way to get at least some fruit into children. Of course fruit juice tastes nice and sweet so it will appeal to a child, which makes it difficult for children to accept water instead. Whole fruit is much better in general, not just for teeth, because it contains fibre which slows down the absorption of sugar in the body and has added vitamins. Freshly pressed juice or juice with "bits" in is also preferable. If children do have fruit juice, dilute it by 50 per cent with water and offer it at meal times only.

Juice drinks aimed at infants and toddlers are simply not necessary and only contribute to children developing a "sweet tooth". Water and milk for the under threes are the best choice.

Drinking vessels

Drinking juice from bottles and sippy-cups, particularly those with valves, increases damage to the front teeth because children often suck the drink and it gets washed over the teeth rather than going to the back of the throat. Some children also suck on bottles for comfort which prolongs exposure to the sugar.

Once a child reaches a year old, they should be encouraged to start drinking from a cup. Introducing a non-spill cup at meal times when you begin weaning can make life a lot easier when making the transition to an open cup later on. Many children can find this transition difficult (as can parents) because the bottle is often a great source of comfort and less messy but the later you leave it the more resistance you may experience.

Although breastfeeding may continue well beyond one year old, moving away from a bottle will also help children develop important co-ordination skills. They will eventually learn how to control the cup without spilling the contents and of course this will help protect their teeth too. It can be useful to teach your child to sip drinks through a straw. This limits the exposure of any sugars onto the teeth surfaces as the drink is drawn towards the back of the mouth and children often enjoy drinking this way.

Children are attracted to bright, **sugary drinks** but as the evidence and the public health figures show, too many are suffering damage. Tooth decay before the age of three is avoidable.

Melanie Wakeman is a Senior Lecturer in Nutrition and Applied Physiology at Birmingham City University

This article was originally published on The Conversation. Read the original article.

LOAD-DATE: October 1, 2014

LANGUAGE: ENGLISH

PUBLICATION-TYPE: Newspaper; Web Publication

JOURNAL-CODE: WEBI

Copyright 2014 Independent Digital News and Media Limited
All Rights Reserved

The Telegraph
telegraph.co.uk

October 1, 2014 Wednesday 8:33 AM GMT

Fruit drinks fuelling tooth decay among under 3s; Parents warned to stop filling baby bottles with juice and sugary drinks as figures disclose 'rampant' tooth decay among three year-olds

BYLINE: By Laura Donnelly Health Editor

SECTION: NEWS

LENGTH: 899 words

Up to one third of children are suffering from tooth decay by the age of three because parents are giving them too much fruit juice and squash, health officials have warned.

Senior dentists said the country should be ashamed of itself after the first ever national breakdown of infant health showed "rampant" levels of aggressive decay in some parts of the country.

Officials from Public Health England (PHE) said busy parents were dropping infants off at nurseries with teeth un-brushed, and doling out baby bottles and sipping cups filled with **sugary drinks**.

Dr Sandra White, PHE director of dental public health, said many parents were giving babies and toddlers fruit juices without realising they were packed with sugar.

She advised parents to only give milk or water to children until the age of three, and avoid using bottles or sipping cups for any drinks which contain sugar.

Dr White said fruit juice was now "the biggest culprit" behind shocking levels of decay in many parts of the country.

The senior dentist said: "Fruit juices appear healthy but actually have a lot of sugar - parents are just trying to do their best for children but don't realise how much sugar there is in the fruit drinks."

Many parents who prided themselves on only buying organic were unaware that the sugar content was just as high, she said.

In other cases, infants were being given squash drinks at a dangerously young age, she said.

"You find parents putting squashes into baby bottles - children who are hardly out of nappies with a bottle of **sugary drink** in their mouth," she said.

Such practices have led to "rampant" levels of early childhood caries in some parts of the country, the official warned.

Some parents were not brushing children's teeth in the mornings at all, she said, leaving the job to nurseries and carers.

"With mothers and fathers often at work all day, you get busy parents dropping kids off at nursery and running, and they haven't brushed their teeth, so this isn't just a job for parents - it's for nurseries and for grandparents, and carers," she said.

The new PHE data comparing all local authorities in England shows that in Leicester, 34 per cent of three-year-olds are suffering from tooth decay, along with more than one quarter of those living in Oldham, Slough, Manchester, Hillingdon and Salford.

In South Gloucestershire, the figure is less than 2 per cent, with low levels of decay also found in East Riding of Yorkshire, Staffordshire and North Tyneside, the data shows.

Dr White said Britain needed to return to the sensible eating habits learned during war-time rationing.

"Look at war time, when sugar was rationed; we had really good health then, including dental health. We need to get back to that time when we ate less sugar and more importantly drank less sugar."

She said too many children were forced to go through "harrowing" dental extractions involving a general anaesthetic, leaving parents feeling guilty.

"This is preventable, it doesn't need to happen - we should be ashamed of ourselves as a nation," the senior dentist said.

On average, 12 per cent of three-year-olds in England had suffered from obvious decay, dentists found - with an average of three teeth decayed, missing or filled.

The investigation also examined levels of "early childhood caries" - a particularly aggressive form of decay starting in the upper front teeth, which is related to consumption of **sugary drinks** from bottles and sipping cups. The highest levels were found in London, with 16.1 per cent of three-year olds in Hillingdon and 14 per cent of those in Newham found to be suffering such damage.

Health officials said that in rare cases, three-year-olds had suffered such delay that all their teeth were removed.

Separate figures show that among infants aged 1 ½ year to 3 years old, fruit juice is the greatest single source of **sugar** intake, making up 14 per cent of intake, with 12 per cent from **soft drinks** and 12 per cent from sweets.

Dr White said she sympathised with parents who gave infants sipping cups, in a bid to reduce spillages.

"I know it is difficult with toddlers, I had it with my own kids, you give them a drink in an open cup and with one shake it is everywhere," she said.

"The worst thing is going to bed with any kind of **sugary drink** in a bottle," the dentist advised.

Officials said the data on three-year-olds suggested widespread problems across society, with problems in affluent as well as deprived areas.

Dr White suggested many families who prided themselves on only buying organic fruit juices did not realise the sugar content was still dangerously high.

"Organic apple juice sounds really healthy on the packet but actually it's packed with sugar," she said. "Posh sugar is no better than any other sugar."

Local authorities with the highest percentage of three year olds with decay

Leicester 34

Oldham 30.4

Slough 25.7

Manchester 25.6

Hillingdon 25.3

Salford 24.8

Newham 23.1

Herefordshire 22.3

Luton 21.8

Blackburn with Darwen 20.6

Local authorities with the lowest percentage of three year olds with decay

South Gloucestershire 1.9

East Riding of Yorkshire 3.9

Staffordshire 4

North Tyneside 4

Hampshire 4.6

Hartlepool 4.7

Cambridgeshire 4.8

Surrey 5.1

South Tyneside 5.1

Wokingham 5.5

Source: Public Health England

LOAD-DATE: October 1, 2014

LANGUAGE: ENGLISH

PUBLICATION-TYPE: Newspaper; Web Publication

JOURNAL-CODE: WEBDTNS

Copyright 2014 Telegraph Media Group Limited
All Rights Reserved

DAILY MAIL (London)

September 30, 2014 Tuesday

DRINKING COLA COULD CUT VITAMIN D LEVELS

BYLINE: BY NO BYLINE AVAILABLE

LENGTH: 123 words

CAN drinking cola lead to a lack of vitamin D? That's the suggestion from scientists at Laval University, in Canada, who asked more than 700 women about their intake of **sugary drinks**, including cola and sweet fruit juice.

Blood samples showed that women who drank cola three times a week had 12 per cent less vitamin D than women who never drank it. Other fizzy soft drinks and fruit drinks didn't appear to have an effect.

Writing in the journal Nutrients, the team identified higher levels of phosphoric acid in cola as a possible culprit. Previous research has linked phosphoric acid to lower levels of vitamin D and calcium. Vitamin D helps regulate levels of calcium and phosphate in the body, and keeps bones and teeth healthy.

© Daily Mail

LOAD-DATE: September 29, 2014

LANGUAGE: ENGLISH

PUBLICATION-TYPE: Papers

Copyright 2014 Associated Newspapers Ltd.
All Rights Reserved

DAILY MAIL (London)

September 30, 2014 Tuesday

ONE IN 8 THREE-YEAR-OLDS HAS ROTTING TEETH... AND FRUIT JUICE IS TO BLAME

BYLINE: BY SOPHIE BORLAND HEALTH CORRESPONDENT

LENGTH: 557 words

TENS of thousands of young children are suffering from rotting teeth caused by drinking fruit juice and squash, a major study has revealed.

As many as one in eight children have suffered tooth decay by the age of three - although in some parts of England the rates are as high as a third.

Senior dentists say the problem is often caused by well-meaning parents giving toddlers sugar-laden drinks in bottles and beakers. They are now urging families to restrict children to milk and water.

Middle-class parents who buy expensive organic juices in the belief thay are healthier have been warned they can contain as much sugar as a glass of coke. One health official observed: Posh sugar is no better than any other sugar.'

Earlier this year health officials urged the public to cut their sugar intake to between five and seven teaspoons a day to prevent rising levels of obesity and rotting teeth.

A 200ml glass of organic apple juice contains 20 grams of sugar - nearly five teaspoons - only slightly less than the same amount of coke, which has 22 grams.

Experts, including the chief medical officer Dame Sally Davies, have called for a tax to be slapped on **sugary drinks** to deter the public from buying them.

In the first study of its kind, officials at Public Health England - a Government agency - examined the teeth of a sample of 53,640 three-year-olds at nursery schools. They found an average of 12 per cent - one in eight - had tooth decay ranging from small holes, needing fillings or having teeth extracted. If the trend is repeated across England, then nearly 85,000 three-year-olds have rotten teeth.

Leicester has the highest rates, with 34 per cent of three-year-olds having rotten teeth. Others included the relatively affluent boroughs of Hillingdon, West London, at 25 per cent, and Charnwood in Leicestershire at 29 per cent.

Sandra White, director of dental health for Public Health England, said: The biggest culprit is fruit juice. Organic apple juice sounds healthy on the packet, but actually it's packed with sugar.

Posh sugar is no better than any other sugar.

Parents think they are doing good, but fruit juice needs to be restricted to one small glass a day.

Our key advice for under three is just have water and milk, that way they won't get a taste for the sweeter liquids that cause decay.'

Many parents give children fruit juice or squash in bottles and beakers to comfort them or before bed. They may suck them for hours on end - with the sugar slowly eroding their teeth. Officials say if children must have juice it should be from a cup which is drunk quickly, causing minimal harm to their teeth.

Although the decay affects children's milk teeth, which fall out naturally, experts say it also harms their gums making them more prone to infection in adulthood.

This is the first time such a survey has been carried out so it is impossible to know if the problem is getting worse. But NHS figures published in July showed the number of children being admitted to hospital with tooth decay had risen by 14 per cent in three years.

Dr Christopher Allen, chairman of the British Dental Association's dental public health committee, said: Parents may feel that giving sugar-sweetened drinks is comforting, but in reality it's more likely to cause pain and suffering as it is the major cause of tooth decay in toddlers.'

© Daily Mail

LOAD-DATE: September 29, 2014

LANGUAGE: ENGLISH

PUBLICATION-TYPE: Papers

Copyright 2014 Associated Newspapers Ltd.
All Rights Reserved

DAILY MAIL (London)

September 30, 2014 Tuesday

WHICH LOW-CAL COLA IS TOP OF THE POPS?

BYLINE: BY ANNE SHOOTER

LENGTH: 483 words

NEW, bright green Coca-Cola Life bottles are in stores now with the natural sweetener stevia replacing some of the sugar. So what does it taste like and how does it compare to other, low-calorie colas on the flavour front?

Coca-Cola Life

27 calories per 100ml, £1.85 for 1.75 litres

This is very sweet but doesn't leave me feeling like there's a sugary coating on my teeth, as I do with normal Coca-Cola.

Sugar is still the second ingredient on the list, but there are steviol glycosides, too, which come from the leaf of the tropical stevia plant. Stevia has a slight aniseed flavour but I can't detect it here. However, it is too sweet for me and my children. They'll normally do anything for a Diet Coke, but they didn't like it at all. VERDICT: 2/5

Diet Pepsi

0.6 cals per 100ml,

£1.98 for 2 litres

To me, Pepsi has always had a strong vanilla flavour: it reminds me of cola cube sweets. Although it's full of chemical sweeteners and preservatives, it doesn't, in all honesty, taste too artificial. In fact, I rather like it.

VERDICT: 4/5

Fentimans Curiosity Cola

46 cals per 100ml, £1.19 for 275ml

ALL right, so this one isn't low calorie and loses a point for that but it doesn't have any nonsense in it, being made mainly from ginger root extract. It's cola, but not as you know it. It's definitely a grown-up drink, with all sorts of botanicals offering different flavours.

I am a big fan though frankly, I'd like it with a shot of rum.

VERDICT: 4/5

Simply M&S Diet Cola

0 cals per 100ml, 89p for 2 litres

This doesn't taste anything like cola to me. In fact, it tastes like some kind of foreign fruit drink and a quick scan of the ingredients tells me why. It contains apple, carrot and hibiscus extracts, along with barley malt. Not unpleasant. Just not cola.

VERDICT: 3/5

Tesco 30pC less sugar Cola

31 cals per 100ml, 89p for 2 litres

THIS is like a cross between the M&S cola (it has apple, carrot and hibiscus, plus molasses) and Coca-Cola Life, as it contains stevia extract. It is also very sweet, with an artificial aftertaste. Not for me. VERDICT: 1/5

Asda Diet Cola

0 cals, 42p for 2 litres

This is horrid. It has very similar ingredients to the M&S Cola but it has an overwhelming flavour of artificial sweetener. Contains sucralose and the artificial sweetener Acesulfame K, whereas the M&S one uses just sucralose. VERDICT: 0/5

The Co-operative Diet Cola

2 cals per 250ml, 69p for 2 litres

The bottle for this one is so flimsy I could barely hold it, and I spilled the drink all over the kitchen. I don't like the taste of this one either it's just too artificial for me. And guess what? It contains that nasty Acesulfame K again.

VERDICT: 0/5

Lidl Freeway

Cola Light

0.4 cals per 100ml, 39p for 2 litres

This is lovely and fizzy. And even though it contains three artificial sweeteners (including the dreaded Acesulfame K), I love it! With ice and a slice of lemon, it's my favourite. VERDICT: 5/5

© Daily Mail

LOAD-DATE: September 29, 2014

LANGUAGE: ENGLISH

PUBLICATION-TYPE: Papers

Copyright 2014 Associated Newspapers Ltd.
All Rights Reserved

The Daily Telegraph

The Daily Telegraph (London)

September 30, 2014 Tuesday
Edition 1;
National Edition

Fruit juice blamed for rampant tooth decay in toddlers

BYLINE: Laura Donnelly

SECTION: NEWS; Pg. 6

LENGTH: 382 words

UP TO one third of children suffer from tooth decay by the age of three because parents give them too much fruit juice and squash, health officials have said.

Analysis by Public Health England (PHE) found "rampant" levels of tooth decay in some parts of the country.

Officials said parents were taking children to nursery without brushing their teeth, and giving babies and toddlers **sugary drinks** in bottles or sipping cups.

Dr Sandra White, PHE's director of dental public health, advised parents to give children under three only milk or water, and avoid using bottles or sipping cups for any drinks that contain sugar.

She said fruit juice was "the biggest culprit" behind severe levels of decay in many parts of the country. "Fruit juices appear healthy but actually have a lot of sugar. Parents are just trying to do their best for children, but don't realise how much sugar there is in the fruit drinks."

Many parents who prided themselves on only buying organic were unaware that the sugar content was just as high, Dr White added.

In other cases, infants were being given squash at a dangerously young age, she said. "You find parents putting squash into baby bottles - children who are hardly out of nappies with a bottle of **sugary drink** in their mouth."

Some parents were also failing to brush their children's teeth in the mornings, she said. "You get busy parents dropping kids off at nursery and running, and they haven't brushed their teeth, so this isn't just a job for parents - it's for nurseries and for grandparents, and carers," Dr White said.

The data, which compared all local authorities in England, showed that in Leicester, 34 per cent of three-year-olds suffered from tooth decay, as did more than a quarter in Oldham; Slough; Manchester; Hillingdon, north-west London; and Salford. However, in South Gloucestershire the figure was less than 2 per cent. There were also low levels of decay in the East Riding of Yorkshire, Staffordshire and North Tyneside.

Dr White said Britain needed to return to the sensible eating habits learnt during wartime rationing. "Look at wartime, when sugar was rationed - we had really good health then, including dental health. We need to get back to that time when we ate less sugar and, more importantly, drank less sugar," she said.

LOAD-DATE: September 30, 2014

LANGUAGE: ENGLISH

PUBLICATION-TYPE: Newspaper

JOURNAL-CODE: DTL

Copyright 2014 Telegraph Media Group Limited
All Rights Reserved

theguardian

The Guardian

September 30, 2014 Tuesday

Fruit juice and squash blamed after survey reveals toddler tooth decay: 12% of under-threes need treatment across England Dentists call for rethink on fluoridation of water

BYLINE: Sarah Boseley Health editor

SECTION: GUARDIAN HOME PAGES; Pg. 2

LENGTH: 687 words

Small children are having large numbers of teeth extracted because of decay caused by drinking fruit juice and squash from bottles and feeding cups, the health watchdog for England has warned.

Public Health England is calling for under-threes to be given only milk and water to drink after the first nationwide survey into their dental health.

Overall, 12% of three-year-olds have some tooth decay, but in some parts of the UK the proportion rises to a third, the study shows. "As a country, we should be ashamed," said Dr Sandra White, PHE's director of dental health, who described the findings as "shocking".

Filling bottles and sipping cups with juice or squash, which tend to contain large quantities of sugar, is a particular problem because the sweetened liquid coats the back of the front teeth, causing them to rot. The decay can then spread to the child's other teeth.

Some children have had most of their teeth removed by the dentist, which is often stigmatising and can affect the growth of their adult teeth, the PHE found. "People still put sugar in milk and (many) children have sugared drinks as well," White said. "They sometimes fall asleep with **sugary drinks** in a bottle in their mouth."

By the age of five, dental decay and fillings are more common in those from more deprived areas, but at age three, there is a greater spread, PHE found.

More affluent parents tended to think fruit juice is good for their small children, according to the study.

"People have thought they were doing the right thing - fruit juice is part of the five-a-day and has vitamins. But it is stacked with sugar. Parents are trying to do the best, but sometimes they don't know the best thing to do. In some more affluent areas, there is almost a feeling of reassurance that if something is organic, it is fine. But it is still sugar and causing decay," White said.

PHE is advising parents to reduce the amount of sugar they give their children for the sake of their teeth and to avoid them putting on excess weight. They should not add sugar to weaning foods or liquids.

Babies and small children should drink only water and milk and should stop using a bottle at 12 months. PHE also recommends giving children sugar-free medicines.

The survey shows the highest rates of dental decay in three-year-olds are in the East Midlands, followed by the north-west, London, and then Yorkshire and the Humber. Three-year-olds with tooth decay have an average of three missing, decayed or filled teeth.

In Gloucestershire 2% of the children have tooth decay; Leicester has the highest rate at 34%.

Fluoridated water helps prevent decay, but only 10% of the country has the chemical added to its supply. Parents should use fluoride toothpaste for their children, PHE says. A smear is sufficient for under-threes, with a pea-sized amount for older children.

Parents should also help and supervise their children while they brush their teeth twice a day, including before bed.

Public health is also the responsibility of local authorities, so PHE suggests they prioritise dental health, particularly in areas shown to have serious problems.

Professor Nigel Hunt, dean of the faculty of dental surgery at the Royal College of Surgeons, expressed concern about the significant variations in decay around the country.

"Tooth decay is almost entirely preventable so action must be taken to inform parents of the risks and ensure that all children are able to visit a dentist regularly so that any problems are diagnosed and referred to a specialist dentist when appropriate, to be treated as quickly as possible," he said.

"Steps have already been taken to increase levels of fluoride in everyday toothpaste but it is essential that parents and carers understand the need to brush children's teeth twice a day and to reduce sugary foods and drinks.

"As well as ensuring all children are able to visit an NHS dentist we would like to see the government further explore with local authorities the introduction of water fluoridation in order to reduce these significant inequalities in children's oral health in England."

Captions:

In some areas, one in three have decay

LOAD-DATE: September 29, 2014

LANGUAGE: ENGLISH

PUBLICATION-TYPE: Newspaper

Copyright 2014 Guardian Newspapers Limited
All Rights Reserved

The Sun (England)

September 30, 2014 Tuesday
Edition 1;
National Edition

Kids' bad gnashers

SECTION: NEWS; Pg. 21

LENGTH: 125 words

A THIRD of toddlers in some parts of England have rotten teeth, shocking figures reveal.

On average, one in eight kids aged three suffer from tooth decay - with dentists calling it a national scandal.

They blamed parents for giving kids sweets, **sugary drinks** and fruit juice and advised giving only water or milk to drink.

Researchers from Public Health England looked at more than 50,000 threeyear-olds.

Kids in Leicester had the worst teeth, with 34 per cent showing signs of decay, while Oldham had 30 per cent. In South Gloucestershire, just two per cent had rotten teeth.

The British Dental Association's Dr Christopher Allen said: "Until we see the political will to end this scandal, governments will keep footing the bill for preventable treatment."

LOAD-DATE: September 30, 2014

LANGUAGE: ENGLISH

PUBLICATION-TYPE: Newspaper

JOURNAL-CODE: SUN

Copyright 2014 NEWS GROUP NEWSPAPERS LTD
All Rights Reserved

The Guardian - Final Edition

September 29, 2014 Monday

G2: Health: Dr Dillner's dilemma: Should I use sports drinks?

BYLINE: Luisa Dillner

SECTION: GUARDIAN FEATURES PAGES; Pg. 13

LENGTH: 487 words

When did water stop being the best drink for quenching your thirst? It's cheap, has no calories and a US independent Beverage Guidance panel ranked it top for its "contribution to intake of energy and essential nutrients" and for "its positive effects on health". In comparison, sports drinks were way down the list, just above "sweetened, nutrient-poor drinks" such as Coca-Cola. Yet sports beverages - flavoured, often brightly coloured drinks containing carbohydrates, minerals, electrolytes and sometimes vitamins, are widely promoted at sporting events and by sports personalities. But do they really improve your sporting performance? Last week, the Australian authors of a paper in the Journal of Medical Ethics argued that "sponsorship could mislead the public into thinking these products work well and/or are good for health - for which there is no strong scientific evidence".

An advert earlier this year claiming Lucozade Sport "hydrated you and fuels you better than water" was banned by the Advertising Standards Authority, which ruled it was misleading. Meanwhile, pediatric journals warn that the acidity in sports drinks erodes teeth enamel and encourages obesity - a 500ml bottle of Lucozade Sport contains 17.5g of sugar (a teaspoon is 4g). An analysis by Carl Heneghan's team at Oxford's Centre for Evidence-Based Medicine of 1,035 web pages with 431 performance-enhancing claims for 104 different products (a mixture of sports drinks, supplements, trainers and clothing) found only three studies out of 146 were high quality and at low risk of bias. The team concluded there was insufficient evidence for the health benefits of any of the claims. Meanwhile, an investigation into sports drinks by the BMJ two years ago concluded that people who avoided sports drinks could well get thinner and run faster.

The solution

Sports drinks do not provide any benefit when compared with water if you are exercising for 90 minutes or less. Water will rehydrate you just fine, without the added sugar. You also don't need to stock up on fluids "just in case". While dehydration is a risk in endurance exercise, the body's thirst mechanism can be trusted to prevent it. A study of cyclists in time trials found that relying on thirst (drinking to thirst, as it's called) to replace fluid produced a better performance than either over or under-drinking.

While it's clear that non-elite athletes don't need sports drinks, it is not obvious they are essential to anyone at all. A paper in the journal Sports Medicine in 2013 said that while experts had created lots of guidelines, there wasn't much evidence on how they were used in the real world. The few studies that exist vary enormously and journals often don't publish negative studies - those that don't show a benefit - because they're considered boring. So until there is evidence, you don't need to feel less of an athlete for not clutching a branded bottle.

LOAD-DATE: September 29, 2014

LANGUAGE: ENGLISH

PUBLICATION-TYPE: Newspaper

Copyright 2014 Guardian Newspapers Limited
All Rights Reserved

MailOnline

MailOnline

September 29, 2014 Monday 10:22 PM GMT

Which low-cal cola is top of the pops? As Coca-Cola launch a new 'natural' drink, ANNE SHOOTER the savvy shopper tests which is best

BYLINE: ANNE SHOOTER FOR THE DAILY MAIL

SECTION: FOOD

LENGTH: 493 words

New, bright green Coca-Cola Life bottles are in stores now - with the natural sweetener stevia replacing some of the sugar.

So what does it taste like and how does it compare to other, low-calorie colas on the flavour front?

Coca-Cola Life

27 calories per 100ml, £1.85 for 1.75 litres.

This is very sweet but doesn't leave me feeling like there's a sugary coating on my teeth, as I do with normal Coca-Cola.

Sugar is still the second ingredient on the list, but there are steviol glycosides, too, which come from the leaf of the tropical stevia plant.

Stevia has a slight aniseed flavour but I can't detect it here. However, it is too sweet for me and my children. They'll normally do anything for a Diet Coke, but they didn't like it at all.

VERDICT: 2/5

Diet Pepsi

0.6 cals per 100ml, £1.98 for 2 litres.

To me, Pepsi has always had a strong vanilla flavour: it reminds me of cola cube sweets.

Although it's full of chemical sweeteners and preservatives, it doesn't, in all honesty, taste too artificial. In fact, I rather like it.

VERDICT: 4/5

Fentimans Curiosity Cola

46 cals per 100ml, £1.19 for 275ml.

All right, so this one isn't low calorie - and loses a point for that - but it doesn't have any nonsense in it, being made mainly from ginger root extract.

It's cola, but not as you know it. It's definitely a grown-up drink, with all sorts of botanicals offering different flavours.

I am a big fan - though frankly, I'd like it with a shot of rum.

VERDICT: 4/5

Simply M&S Diet Cola

0 cals per 100ml, 89p for 2 litres.

This doesn't taste anything like cola to me. In fact, it tastes like some kind of foreign fruit drink - and a quick scan of the ingredients tells me why.

It contains apple, carrot and hibiscus extracts, along with barley malt.

Not unpleasant. Just not cola.

VERDICT: 3/5

Tesco 30% Less Sugar Cola

31 cals per 100ml, 89p for 2 litres.

This is like a cross between the M&S cola (it has apple, carrot and hibiscus, plus molasses) and Coca-Cola Life, as it contains stevia extract.

It is also very sweet, with an artificial aftertaste. Not for me.

VERDICT: 1/5

Asda Diet Cola

0 cals, 42p for 2 litres.

This is horrid. It has very similar ingredients to the M&S Cola but it has an overwhelming flavour of artificial sweetener.

Contains sucralose and the artificial sweetener Acesulfame K, whereas the M&S one uses just sucralose.

VERDICT: 0/5

The Co-operative Diet Cola

2 cals per 250ml, 69p for 2 litres.

The bottle for this one is so flimsy I could barely hold it, and I spilled the drink all over the kitchen.

I don't like the taste of this one either - it's just too artificial for me.

And guess what? It contains that nasty Acesulfame K again.

VERDICT: 0/5

Lidl Freeway Cola Light

0.4 cals per 100ml, 39p for 2 litres.

This is lovely and fizzy.

And even though it contains three artificial sweeteners (including the dreaded Acesulfame K), I love it!

With ice and a slice of lemon, it's my favourite.

VERDICT: 5/5

To save while you shop visit mysupermarket.co.uk

LOAD-DATE: September 29, 2014

LANGUAGE: ENGLISH

PUBLICATION-TYPE: Web Publication

JOURNAL-CODE: WEBDM

Copyright 2014 Associated Newspapers Ltd.
All Rights Reserved

MailOnline

September 26, 2014 Friday 3:25 AM GMT

Coke launches smaller 250ML 'handbag-size' can for just $2 - but will it REALLY tackle obesity?

BYLINE: MAY SLATER FOR DAILY MAIL AUSTRALIA

SECTION: NEWS

LENGTH: 535 words

- Soft drink giant launches new slimline cans for $2 in Australia
- The small cans will be available at convenience stores and petrol stations
- Coca-cola profits fell 16 per cent last financial year
- Smaller sizes are part of Coca-Cola's commitment to a 'healthier Australia'

In a move to boost sales and promote a healthier image, beverage giant Coca-Cola Amatil has launched a new 250ml slimline can for Australian consumers on the run.

In what Coca Cola says is its biggest product launch since Coke Zero, the 'perfectly small' cans will cost no more than $2 and will be sold at convenience stores and petrol outlets Australia-wide.

The company believes the availability of smaller cans of its Coca-Cola, Sprite and Fanta drinks will increase sales by appealing to consumers watching their hip-pockets and their waistlines.

'We've listened to what consumers want and we're getting serious about smaller portion sizes,' a Coca-Cola Spokesperson told Daily Mail Australia. 'This is really about providing consumers with more choice and the point of difference is that you'll be able to buy these small cans from more convenient outlets, giving people the option to grab a taste of their favourite drink on the run.'

Coca Cola says the smaller cans are one part of the company's commitment to a 'healthier Australia'.

'Last year we launched a series of commitments across our food and beverage products to help address the problem of obesity in Australia and portion sizes was certainly a part of that plan,' said a spokesperson for the company.'

Changes also include offering a wider selection of low-kilojoule beverage options and providing transparent nutritional information in more places, including vending machines.

'We believe Coca-Cola has an important role in providing more beverage choices, including more choice in serve sizes and low-kilojoule options, clearly communicating the kilojoule content of our products and supporting community-based physical activity programs,' said Phil Roberts, Commercial and Franchise Director of Coca-Cola South Pacific last year.

Coca-Cola Amatil is Australia's largest beverage company, with 60 per cent of the **soft drink** market. But last year, the company posted a 16 per cent fall in profits as consumers moved away from **sugary** fizzies towards healthier options like juices and coconut water.

Coca-Cola, who are launching a multimillion dollar 'small' marketing campaign say the new 250ml cans have been popular in other markets.

'People still love Coke, it's been around for 128 years and there's nothing that will replace it,' said a Spokesperson for the company.

Coca-Cola already sells 200ml 'mini-cans' in multi-packs in supermarkets for about $1.09 a can. The average price of a regular can of coke is about $3.

Jeff Rogut from the Australasian Association of Convenience Stores says the new 250ml cans may help smaller retailers compete by being able to offer a cheaper, more convenient product but he doesn't believe they will 'revolutionize' sales.

'The product is the same. The price may change and the value equation may change but consumers are interested in having something really new and different,'' he told Fairfax. 'How many ways can you package Coca-Cola?.'

LOAD-DATE: September 26, 2014

LANGUAGE: ENGLISH

PUBLICATION-TYPE: Web Publication

JOURNAL-CODE: WEBDM

Copyright 2014 Associated Newspapers Ltd.
All Rights Reserved

The Telegraph
telegraph.co.uk

September 25, 2014 Thursday 11:50 AM GMT

Coke and Pepsi promise 20 per cent product calorie slim down; Bloomberg's Olivia Sterns reports on a pledge by Coca-Cola and Pepsi to cut calories by 20 per cent in their product offerings by 2025

BYLINE: By Telegraph Video and AFP, video source Bloomberg

SECTION: FINANCE

LENGTH: 449 words

US soft-drinks giants Tuesday promised to work to reduce the country's beverage calorie consumption by 20 per cent by 2025 in a campaign to counter obesity trends.

Coca-Cola, PepsiCo and Dr. Pepper Snapple pledged to provide smaller-sized bottles, and more water and other low- or no-calorie beverages, to the market to help bring down per-person consumption of their high-**sugar drinks**.

They also agreed to better publicise calorie counts on vending machines, retail coolers and all drink-vending equipment controlled by the companies.

Appearing at an event organised by the Clinton Global Initiative in New York, representatives from each signed a voluntary initiative to employ marketing and consumer outreach to prod consumers to drink fewer **sugary drinks** and promote calorie awareness.

The goal is to cut calories from drinks by 20 per cent per person within a decade.

The initiative comes as Coca-Cola, PepsiCo and others struggle with flagging sales in their home market. Coca-Cola reported flat sales in North America in the most recent quarter.

Regulators in New York City, California and other venues have proposed measures to cut drink size or enhance labelling requirements.

The companies also said they would intensify awareness campaigns and promotion of healthier beverages in communities where there have been fewer options to often **sugar**-laden **soft drinks**.

They will retain an independent evaluator to track progress, in conjunction with an advocacy group set up by the Clinton Foundation, founded by former president Bill Clinton, and the American Heart Association.

Public health advocates said the measures did not go far enough.

The Centre for Science in the Public Interest, a public-interest advocacy group in Washington, said the initiative was "welcome news," but called on the companies to drop their opposition to taxes and warning labels on **sugary drinks**.

"We applaud President Clinton for his efforts," the group said in a statement. "But we need much bigger and faster reductions to adequately protect the public's health."

Measures to tax **sugary drinks** "could further reduce calories in America's beverage mix even more quickly, and would raise needed revenue for the prevention and treatment of soda-related diseases," the group said in a statement.

Marion Nestle, a nutrition and public health professor at New York University, said the companies would have no problem reaching the 20 per cent target in light of consumption trends that are already happening.

"If they really want to promote public health, they should stop fighting soda taxes and lobbying against this and other public health measures," she said in an email message. "This is pure public relations."

LOAD-DATE: September 25, 2014

LANGUAGE: ENGLISH

PUBLICATION-TYPE: Newspaper; Web Publication

JOURNAL-CODE: WEBDTNS

Copyright 2014 Telegraph Media Group Limited
All Rights Reserved

FT
FINANCIAL
TIMES
Financial Times (London, England)

September 24, 2014 Wednesday

Golden cola makes for an improbable source of national pride; Peru

BYLINE: Andres Schipani

SECTION: FT REPORT - LATIN AMERICAN BRANDS; Pg. 3

LENGTH: 425 words

HIGHLIGHT: Inca Kola is among a few privileged beverages more popular in their home markets than Coca-Cola. Andres Schipani reports

Reina Huamán, a 52-year-old housewife and occasional cook, returned to her home town of Trujillo this year a heroine, after a trip to Lima.

She had won a television cooking contest sponsored by Inca Kola, the country's favourite soft drink. "This is our national drink, it is something we share at every table in Peru," she says. Despite the whiff of product placement, she is not exaggerating the brand's appeal.

Inca Kola is consumed across all social classes in Peru. It is very sweet, with flavours hard to discern. First-timers might find its colour and taste unpleasant. Jorge Luis Borges, the Argentine writer, was supposed to have described it as "an implausible drink" .

And since the yellow-gold beverage was introduced 80 years ago by the drinks company of a British family called Lindley, who had settled in Peru, it has been a source of national pride.

The brand's success has even been highlighted by a leading business school. Branding Lessons from Inca Kola, the Peruvian Soda That Bested Coca-Cola , an essay published by the Wharton School of Management at the University of Pennsylvania, says: "The success of Inca Kola also reflects the uniqueness of Peruvian consumers, who tend to have very strong ties to products that they associate with personal and national identity."

Inca Kola is, experts point out, among a few privileged beverages more popular at home than Coca-Cola. Others include Irn-Bru, the best-selling **fizzy drink** in Scotland.

In 1999, Coca-Cola purchased 38.5 per cent of Inca Kola shares with voting rights from the Lindley Corporation for an undisclosed sum, reported by Wharton to have been $200m, and creating what Coca-Cola's executives now call a "strategic alliance".

The US group has subsequently bought a further 10 per cent of shares without voting rights.

But 15 years on, Inca Kola still outsells Coca-Cola in Peru, with 30 per cent of the market against just over 20 per cent for Coke, says Alfredo Quiñones, Inca Kola brand manager at Coca-Cola in Lima.

Peru's decade-long surge in domestic demand has helped the business grow. About 90 per cent of Inca Kola is sold at small, independent shops and economists believe the continuing success of the beverage lies largely in the hands of housewives, such as Ms Huamán

But Hajime Kasuga, a Peruvian chef who teaches at Le Cordon Bleu school in Lima and was a juror on The Inca Kola Seasoning , the television cooking contest that elevated Ms Huamán to a heroine, goes further.

"Inca Kola runs through the veins of Peruvian babies - and that is not an exaggeration."

LOAD-DATE: September 23, 2014

LANGUAGE: ENGLISH

PUBLICATION-TYPE: Newspaper

Copyright 2014 The Financial Times Ltd.
All Rights Reserved
Please do not cut and paste FT articles and redistribute by email or post to the web.

i-Independent Print Ltd

September 24, 2014
First Edition

Glasgow Games a boon for A G Barr; The Business Matrix The day at a glance DRINKS

SECTION: NEWS; Pg. 46

LENGTH: 47 words

The Commonwealth Games in Glasgow this summer helped A G Barr, the Scottish **fizzy drinks** maker, report a near 15 per cent rise in half-yearly profits to £19m as sales rose 5.4 per cent to £135.7m. It also unveiled a new 10-year contract to sell Snapple in the UK and parts of Europe.

LOAD-DATE: September 23, 2014

LANGUAGE: ENGLISH

PUBLICATION-TYPE: Newspaper

JOURNAL-CODE: III

Copyright 2014 Independent Print Limited
All Rights Reserved

i-Independent Print Ltd

September 23, 2014
First Edition

TV-free days advised in bid to tackle obesity crisis; HEALTH

BYLINE: Charlie Cooper HEALTH REPORTER

SECTION: NEWS; Pg. 4

LENGTH: 177 words

People should cut the time they spend watching television to two hours per day, or even consider "TV-free days", to cut their risk of becoming overweight, according to new health guidance.

To maintain a healthy weight, people should also be encouraged to walk or cycle to work or school, and should avoid **fizzy drinks**, sports drinks and even squash, the draft guidance from the National Institute for Health and Care Excellence (Nice) says. Nice already has a range of recommendations for people who are already overweight or obese, but the new guidelines are also aimed at helping people to maintain a healthy weight.

They recommend that people eat fewer energy-dense foods, such as biscuits, confectionary, full-fat cheese and fried food. Instead a "Mediterranean diet" based on vegetables, fruit, beans, pulses, fish and olive oil is recommended.

People are also advised to cut their alcohol consumption to avoid weight gain, with warnings that a man who drinks the upper weekly limit of 21 units of alcohol is consuming around 1,400 to 1,800 calories each week.

LOAD-DATE: September 22, 2014

LANGUAGE: ENGLISH

PUBLICATION-TYPE: Newspaper

JOURNAL-CODE: III

Copyright 2014 Independent Print Limited
All Rights Reserved

MailOnline

September 23, 2014 Tuesday 11:34 PM GMT

Coca-Cola, Pepsi and Dr Pepper promise to cut 20 per cent of calories Americans get from their drinks - by shrinking soda sizes and plugging low-sugar alternatives

BYLINE: KIERAN CORCORAN FOR MAILONLINE and REUTERS

SECTION: NEWS

LENGTH: 512 words

- Companies made pledge - to take effect by 2025 - today in New York
- Recipe for core sodas will be the same - but sales tactics will change
- Companies will promote low-calorie options and water instead
- Backed by former President Bill Clinton, who confessed to past soda habit

The biggest soda companies in the country promised to cut the calories Americans get from their drinks by a fifth - but will do it by clever marketing rather than changing the sodas themselves.

The Coca-Cola Company, PepsiCo Inc. and Dr Pepper Snapple Group Inc. today gave a joint pledge to cut the calories consumed via their products by 20 per cent before 2025.

However, the companies' plan won't involve changing what's in their **sugary drinks** - but by promoting their lower-calorie alternatives, cutting portion sizes and making people more aware of the amount of sugar in regular sodas.

The companies - whose full-calorie products are already in decline - made the promise today alongside the rest of the American Beverage Association at a New York summit arranged by the Clinton Foundation and supported by former President Bill Clinton.

They plan to reach their 20 percent goal through education targeted at communities with less interest in and access to lower calorie beverages.

They will promote products like bottled water and introduce and expand their offerings of zero calorie or lower-calorie products.

Initially, those efforts will be rolled out in Little Rock, Arkansas and Los Angeles.

The foundation was also linked to a separate initiative to reduce beverage calories in schools in 2006.

Speaking about the pledge to the New York Times, President Clinton said: 'This is huge - I've heard it could mean a couple of pounds of weight lost each year in some cases.'

He added that he could sympathize with those who drink a lot of soda, because it is a cheap way to keep back hunger.

He said: 'When I was in my freshman year in college trying to live on a dollar a day, I drank at least one and sometimes two 16-ounce bottles of Royal Crown Cola a day because they cost 15 cents.'

Now he prefers water and iced tea.

Susan K. Neely, the president of the American Beverage Association, said the plan will build on earlier success.

She said: 'It'd be hard to sustain the progress that has been made so far without this commitment,'

In 2010, 16 of the biggest U.S. food and beverage companies, including Coca-Cola and PepsiCo, pledged to remove 1 trillion calories from the U.S. marketplace by 2012 and 1.5 trillion by 2015.

The companies are part of the CEO-led Healthy Weight Commitment Foundation formed in 2009 to reduce obesity.

In January, companies in the foundation said they had collectively sold 6.4 trillion fewer calories in the United States in 2012 than in 2007.

ut an analysis by University of North Carolina researchers, published in the American Journal of Preventive Medicine last week, showed that total calories from packaged goods sold to households with children by those companies did not change from 2011 to 2012.

Government data show that 34 percent of adults were obese in 2007-2008.

LOAD-DATE: September 23, 2014

LANGUAGE: ENGLISH

PUBLICATION-TYPE: Web Publication

JOURNAL-CODE: WEBDM

Copyright 2014 Associated Newspapers Ltd.
All Rights Reserved

The Telegraph

telegraph.co.uk

September 23, 2014 Tuesday 7:00 AM GMT

TV free days' would cut obesity and promote healthier lifestyle, says Nice;
TV free days should become routine to encourage people to be more active and reduce obesity, the National Institute for Health and Clinical Excellence has said

BYLINE: By Rebecca Smith Medical Editor

SECTION: NEWS

LENGTH: 493 words

Everyone should have routine TV free days, limit computer use and take up active hobbies to encourage a healthier lifestyle and prevent obesity, the National Institute for Health and Clinical Excellence has said.

A raft of recommendations have been included in draft guidance in an attempt to improve the health of the nation but will be seen by many as 'nanny state' advice and restating obvious measures.

The guidance said everyone should get enough sleep, eat breakfast, follow a Mediterranean diet, increase their activity levels by walking to cycling to school or work, taking up active hobbies such as dancing or swimming and use the stairs where possible.

In addition people should stop eating in front of the television, avoid **sugary drinks**, reduce screen time by instituting TV free days or limiting viewing to no more than two hours a day, and eat fewer biscuits, cakes and sweets and takeaways.

The guidance also said that using pedometers or other wearable activity monitors can help motivate people to take part in activities.

Professor Mike Kelly, director of the Centre for Public Health at NICE, said: "Obesity rates have nearly doubled over the last 10 years and continue to be a huge concern for local authorities and the health service in England. NICE has already published a range of guidelines to help prevent and manage obesity, but this draft update focuses on the changes individuals can make that might help them reduce their risk of overweight and obesity. Following a healthier diet and being more physically active is important for everyone, not just if you are already overweight or obese.

"The general rule for maintaining a healthy weight is that energy intake through food and drink should not exceed energy output from daily activity. We all know we should probably take the stairs rather than the lift, cut down on TV time, eat more healthily and drink less alcohol. But it can be difficult to know the most useful changes that we can make in terms of our weight.

"This updated guideline makes a number of recommendations which aim to ensure that the advice people are given about maintaining a healthy weight is more specific and based on real evidence."

Dr Alison Tedstone, Chief Nutritionist at Public Health England, said: "PHE welcome the NICE draft recommendations on physical activity and diet. Even people who are a healthy weight should review their diet and lifestyle as eating a balanced diet based on the eatwell plate, being regularly active, drinking alcohol within the lower level guidelines and not smoking helps to protect your health and wellbeing.

"Making sustained changes - like walking or cycling to work instead of taking the bus or driving and swapping sugary foods and drinks to those with no added sugar - can make an enormous difference to a person's health. It is also important to keep an eye on your weight as being overweight increases your risk of type 2 diabetes, heart disease and some cancers."

LOAD-DATE: September 27, 2014

LANGUAGE: ENGLISH

PUBLICATION-TYPE: Newspaper; Web Publication

JOURNAL-CODE: WEBDTNS

Copyright 2014 Telegraph Media Group Limited
All Rights Reserved

Mail Online

MailOnline

September 22, 2014 Monday 3:23 AM GMT

Scotland's other national drink... now made in Milton Keynes: Irn-Bru plant in Bucks helps soft drink expand south of the border

BYLINE: ALAN SIMPSON FOR THE SCOTTISH DAILY MAIL

SECTION: NEWS

LENGTH: 428 words

- Maker AG Barr expected to reveal 5.6 per cent rise in sales in market update
- England and Wales now account for more than half the group's sales
- Fall in price of sugar should also help the company grow more, say analysts

According to the hugely successful advertising campaign, it is a drink made in Scotland from girders.

But Irn-Bru maker AG Barr has seen sales soar in the past year, mainly thanks to a new production plant in England which has helped it crack the UK market.

The facility opened in Milton Keynes, Buckinghamshire, last year to help accelerate a sales drive south of the Border.

Now the company is expected to reveal that sales have risen by 5.6 per cent to around £135million after seeing a big increase in popularity among consumers in England.

Barr, which saw rival Britvic pull the plug on a proposed £1.9billion merger last year following a Competition Commission inquiry, is expected to cite the growth as a platform for further expansion in southern England.

The factory in Milton Keynes was not open in the first half of last year and the group said in a summer trading update that sales were likely to have grown 5.6 per cent to about £135million.

AG Barr has expanded beyond its core Scottish customer base in the past four years, with England and Wales now chipping in more than half of group sales.

The drinks maker said in May it was to invest a further £4million in the Milton Keynes site and confirmed plans to close its Tredegar carton-making factory in Wales, with the loss of 67 jobs.

Barr's other brands, including Rubicon, Tizer and Strathmore Water, are also expected to have achieved resilient performances when chief executive Roger White reports the firm's trading figures tomorrow.

City analysts believe falling commodity prices should help underpin the company's further growth, with input prices expected to be lower next year, especially for the crucial component, sugar.

Charles Pick, drinks analyst at Numis Securities, forecasts interim pre-tax profits of £17.8million at Barr compared with £16.6million last time.

The City consensus for annual profits at the group to January 2015 is £41.6million.

Chris Wickham, analyst at Oriel Securities, said: 'The group remains on track to deliver organic growth.

'In particular, the group's flagship Irn-Bru brand continues to grow share by expanding southwards.'

Scotland's 'other national drink' was invented in 1901 by Andrew Greig Barr.

Only two people know the 32 secret ingredients used to make the drink, including Robin Barr, 74, who stood down as chairman of the firm in 2009 after 31 years.

LOAD-DATE: September 22, 2014

LANGUAGE: ENGLISH

PUBLICATION-TYPE: Web Publication

JOURNAL-CODE: WEBDM

Copyright 2014 Associated Newspapers Ltd.

All Rights Reserved

The Telegraph
telegraph.co.uk

September 22, 2014 Monday 5:02 PM GMT

Coca-Cola Life, the new drink fronted by Rosie Huntington-Whiteley; With a third less sugar and calories, Coca Cola has choosen British model Rosie Huntington-Whiteley to front its new slimline offering, Coca-Cola Life

BYLINE: By Katy Young

SECTION: FASHION

LENGTH: 969 words

A drink by Coca-Cola isn't an obvious choice for a model picture of health, such as Rosie Huntington-Whiteley, to front. We were confused: what's this new green can got that regular Coca-Cola doesn't, and what's with a model agreeing to be the face of it?

It turns out that the hype is more about what Coca-Cola Life doesn't have (a third less sugar, and a third less calories thanks to stevia leaf extract and other natural sweeteners) than what it does, and it also turns out Huntington-Whiteley comes with a similar approach to staying healthy; "I'm a great believer in not denying yourself when it comes to what you eat and drink, but like everything in life it's about finding a healthy and happy balance," she tells us.

READ - Fitness Truths: not all calories are made equal

We needed to know about said balancing act - does she work out? Does she juice? Does she drink coffee, or is she a green tea believer? Here are her answers, and we like them.

On juicing...

"I'm a big advocate of juicing I think it's a great way for us to get our nutrients and vitamins. I also think they taste delicious! I will make my own at home in the morning or if I don't have time I'll pick one up at a juice place. There's lots of places popping up now so I'm happy to see it's catching on."

On Coca-Cola...

"I have always enjoyed the great taste of Diet Coke. I always enjoy a Diet Coke poured over ice with lemon on a hot summer day!"

Any guilty pleasures...

"Bacon!!"

On healthy food...

"I always try to cook cleanly and simply at home so that when I go out to eat at a restaurant I can indulge. I find it gives me a good balance. I'll grill a piece of chicken or fish and have a big plate of fresh organic vegetables and salad for lunch and dinner."

READ - Rosie Huntington-Whiteley: 'Looks go and you fade'

On healthy restaurants...

"In L.A. there's this great vegan restaurant called Crossroads. I had never tried vegan food until a friend took me there. I was blown away by how delicious and creative the food was. It's also a beautiful setting so it doesn't feel like a crazy health place. I take my girlfriends now and they all have the same reaction I did when I first went. It proves that healthy eating can be fun and delicious.

When I'm in London I go to Fernandez Wells in Soho, although it's not necessarily the healthiest of places but I love the Spanish style tapas. I'll go with a girlfriend and catch up over a glass of wine and some cheese! "

On her morning fix...

"I start my day by always chugging a litre of water. Overnight we get dehydrated so that immediately hydrates me and gets me going in the morning. Then I'll make myself a latte with soya milk, I'll check my emails and feed my dogs. Then I'll make myself some breakfast which is usually a green juice, a couple soft boiled or scrambled eggs and some steamed spinach.

On her healthy mantra...

"It's all about balance. I never deprive myself of anything. I listen to my body and give it what it needs. I exercise as often as possible. For me a healthy life starts with exercise. I always feel my best when I'm working out on a regular basis. It releases endorphins and keeps me strong and in shape."

Three tips for staying clean and lean...

"1. As much as I can, I will choose organic foods, especially meat, fruit and vegetables, and I opt for foods and drinks that use natural ingredients. 2. I try to never skip meals, I find when I do I will over eat and reach for all the bad things because I'm over hungry. 3. Drink at least three to four litres of water a day! It makes such a difference for my body when I stay hydrated. My energy levels are up, my skin and eyes are clearer, my stomach is flatter and I rarely get run down or sick."

Exercise tips from her brother, Toby Huntington-Whiteley, personal trainer...

"Try to get your heart rate up at least once a day. Maybe you get off the bus or train a stop early and walk at faster pace than normal to your destination. Maybe you skip with a rope for 10-15 mins in the morning, maybe you go for a run in the local park or take a fun exercise class with your friends. Exercise should be fun. Find ways that work for you so you can enjoy yourself."

On staying motivated...

"I believe we should try to always look our best for ourselves at any age. Find a way of exercising that's fun, recently I have started taking tennis lessons and I love it! Looking and feeling good isn't just a luxury or for vanity reasons, our bodies are our most precious possessions and we should look after ourselves and be the best we can be!"

On winding down...

"I love being at home with my loved ones and my dogs. Listening to good music, enjoying great food, sharing funny stories and jokes. I also love to be outdoors like the beach or the countryside. I always feel my most relaxed when I'm surrounded by nature."

READ - Fitness Truths: fat-loss myths debunked

Rosie Huntington-Whiteley was speaking at the launch of Coca-Cola Life - the new addition to the Coca-Cola family, sweetened from natural sources. For more information visit coca-cola.co.uk/coca-cola-life/

LOAD-DATE: September 29, 2014

LANGUAGE: ENGLISH

PUBLICATION-TYPE: Newspaper; Web Publication

JOURNAL-CODE: WEBDTNS

Copyright 2014 Telegraph Media Group Limited
All Rights Reserved

MailOnline

September 20, 2014 Saturday 12:44 AM GMT

Farhan Akhtar launches zero sugar drink

BYLINE: RADHIKA BHALLA

SECTION: INDIANEWS

LENGTH: 324 words

Actor and film-maker Farhan Akhtar was in the Capital to launch the new Coke Zero, a venture by **soft drink** brand Coca-Cola. The product has no **sugar** added and has the tagline 'Great Coke Taste, with Zero **sugar'**.

The event was held at The Oberoi on Friday afternoon. Dressed in brown trousers, a black T-shirt and blue blazer with matching slip-ons, the actor presented a new avatar with a moustache that he's growing for an upcoming flick, along with a more buffed physique.

Talking to MAIL TODAY about endorsing the brands he said: "I endorse brands that I personally like. As for the health aspect of the aerated drink, I think that if you exercise, eat well and stay fit, you can drink or eat what you want and still be healthy."

Regarding the 'zero' title, he further said: "I'd like to see zero violence against women, which is a cause that I strongly support. I'd like to see zero pollution, zero child trafficking - the list is endless."

About his upcoming movie Do with Vidhu Vinod Chopra and Bejoy Nambiar, the actor said he was excited to be working with Bollywood megastar Amitabh Bachchan 14 years after Lakshya which Akhtar directed in 2004.

The movie also stars Aditi Rao Hydari, and the story reportedly revolves around a bedridden chess master to be essayed by Big B.

Akhtar also spoke about his debut in Marathi cinema with Por Bazaar which addresses the issue of child trafficking. Others at the do included Olympian luger, Shiva Keshavan, fashion designer Sabyasachi Mukherjee and fashion photographer Atul Kasbekar.

They were joined on stage by Manpreet Gulri, country head, Subway; Alok Tandon, CEO, INOX Leisure; Venkatesh Kini, president, Coca-Cola India and South West Asia and Debabrata Mukherjee, VP Marketing, Coca-Cola India.

As part of inaugurating the black coloured can, four out of the 40,000 fans of Coca-Cola Zero on social media website Facebook, were presented the first packs of the product by Akhtar and Kini.

LOAD-DATE: September 20, 2014

LANGUAGE: ENGLISH

PUBLICATION-TYPE: Web Publication

JOURNAL-CODE: WEBDM

Copyright 2014 Associated Newspapers Ltd.
All Rights Reserved

DAILY MAIL (London)

September 19, 2014 Friday

WE MUST TAX SUGAR BEFORE BRITAIN EATS ITSELF TO DEATH

BYLINE: BY IAN BIRRELL

LENGTH: 1370 words

Mr Cube played a big role in my childhood. Not only did he help fund my schooling and summer holidays, but he also filled my house with all manner of sweet delights, syrupy treats and sickly treacles.

For my father was an executive with Tate & Lyle, the country's leading sugar manufacturer, whose packets were decorated with the famous cartoon cube figure.

Every now and then, arriving home from his office, he would deliver the firm's latest innovation from his briefcase for our testing. As a young boy, I devoured these sugary creations, which I imagined came from some Willy Wonka-style factory overseen by the friendly-looking Mr Cube.

Back in the Sixties and early Seventies, of course, most of the rest of my diet was prepared by my mother or school dinner ladies and, like other children, I spent long hours running around parks and playing fields.

In those days, fast food was a soggy cheese and tomato sandwich.

Today, my son laughs when I tell him I can recall my first McDonald's, savouring those famous burgers as if they were haute cuisine.

How innocent such times seem compared with now, when most children spend hours each day staring at screens, fast-food outlets litter high streets and advertising constantly beseeches us to buy sweet treats.

The result? Obesity has become a national crisis threatening to overwhelm the Health Service, which is being forced to treat ever-growing numbers of patients with weight problems and associated health problems so severe they end up in hospital.

No one should be under any illusion about the scale of the gargantuan challenge this crisis presents.

Just listen to Simon Stevens, the smart new chief executive of the National Health Service, who this week warned that obesity is a slow-motion car crash' that threatens to cripple an already overburdened service.

He said: If as a nation we keep piling on the pounds around the waistline, we'll be piling on the pounds in terms of future taxes needed just to keep the NHS afloat.'

As politicians talk of raising taxes to further fund the NHS, and hospitals fall into debt, diabetes alone is already draining £1 in every ten spent on healthcare, and excess weight causes almost one in four deaths from heart disease. Meanwhile, **fizzy drinks** and sweets are causing a surge in tooth decay, with 500 children admitted to hospital each week with rotting teeth.

Health trusts have to splash out on super-size ambulances and £8,000 beds to cope with patients weighing up to 78st.

A larmingly, this could be just the beginning, as a tidal wave of avoidable lifestyle' diseases swamps the NHS.

Shockingly, in clinical terms a quarter of British adults and a fifth of children are obese numbers which have doubled in the past two decades.

In total, two-thirds of British men and more than half the nation's women are considered to be overweight, which presents a greater threat to public health than smoking.

As a result, we have a generation of children who, because of their diet, their weight and their inactivity, could live shorter lives than their parents and grandparents which would turn around historic trends of longer life expectancy.

Britain's obesity problems are among the worst in Europe but this is a global concern, as is all too evident on streets from Baltimore to Beijing and Cape Town to Cairo.

Forget the tired old cliches pushed by the foreign aid industry: two-thirds of the world's overweight people are now found in poorer countries, where more people now go to bed having consumed too many calories than go to bed hungry.

The question, of course, is how to turn the tide something that defeated every one of the 188 countries studied in a recent Lancet investigation of global obesity over the past three decades.

I used to think the solution was simple when I started to see more and more unsightly mountains of fat and flesh wobbling through British towns and cities: they should eat less and exercise more.

And so they should this remains the best dietary advice available. Yet blaming gluttony and slothfulness alone will not solve a problem of such severity.

Public warnings from the Government and health experts have had some impact. Sales of **fizzy drinks**, for instance, have fallen over the past six months, while shoppers are buying fewer bags of sugar and more fresh fruit.

Yet, thanks to the addition of so much sugar in processed food and drink, the amount of invisible' sugar ending up in the bellies of Britons has risen by almost a third over the past two decades.

This has made doctors increasingly desperate in their calls for action. Some even argue sugar is addictive, having a similar impact on the brain's reward circuits as drugs such as cocaine. Certainly, it's clear many obese patients get locked into a tragic and ever-worsening spiral of depression, low self-esteem and compulsive over-eating.

This is why bariatric surgery the fitting of gastric bands may prove ultimately to be the most cost-efficient treatment.

One obese woman told me how she would binge in fast-food joints then pretend not to be hungry at home, while her soaring weight mystified her family. Her growing size made her more miserable, which led her to gorge even more on burgers, chips and fried chicken.

So what is to be done?

Instinctively, I recoil at nanny-state diktats, lectures from politicians (of all people) over how to live our lives, and the idea of government intervention unless absolutely necessary.

But the health crisis unfolding before our eyes demands urgent action even if it has to be forced on us through legislation.

There is a recent precedent which convinces me there is a solution: the war on smoking has shown that social habits can be changed. The imposition of hefty taxes on the cost of cigarettes, along with a smoking ban in public places, has saved thousands of lives along with millions of pounds of taxpayers' money that might have been wasted on treating smoking-related diseases.

Smoking peaked in 1974, but since then has slumped. Last year alone, sales of manufactured cigarettes fell in this country by another 11 per cent.

Now we need to do something similar to save the nation and its health service from drowning in a sea of fat.

T his means taking on the food industry and imposing significant taxes on all that lethal sugar they stuff into their wares, even those such as dried fruit, cereals and yoghurts which are promoted as healthy products.

We should also enforce tobacco-style warnings on food and drink packets, as well as wide-ranging advertising restrictions.

Manufacturers and retailers will howl about hitting the poor hardest with the rising prices that would probably result from such a tax, but the truth is that this is the very social group who are the biggest victims of obesity.

No doubt, politicians will prevaricate, fearing they will be blamed for putting up the cost of living.

Naturally, they will also calculate that the cost of political inaction to the health of the nation not to mention the NHS will be felt long after they have left office.

But the introduction of a tax on sugar cannot wait. Ask yourself why Asda yesterday announced it was introducing a women's size 32 in its shops, and what that says about the shape of our nation.

It pains me to argue for higher taxes on anything, let alone something so interwoven into my own life. But sometimes statistics do not lie.

One government agency suggested that a 20 per cent tax on soft drinks alone could cut the number of overweight Britons by 250,000.

The curious thing about Tate & Lyle's Mr Cube is that he was a very political figure, created in 1949 at a time when sugar was still rationed and Labour was threatening to nationalise the sugar industry.

When the sword-wielding character first appeared, he angrily attacked state control and promoted private enterprise in speech bubbles.

He symbolised a public mood seeking more freedom to enjoy the sweet things in life, even appearing in political cartoons alongside Clement Attlee and Winston Churchill.

But we live in very different times today and we need to stop seeing sugar as something benign and cuddly. With apologies to Mr Cube and all that he stood for, the state must take action to confront something that has become a modern scourge.

© Daily Mail

LOAD-DATE: September 18, 2014

LANGUAGE: ENGLISH

PUBLICATION-TYPE: Papers

Copyright 2014 Associated Newspapers Ltd.
All Rights Reserved

i-Independent Print Ltd

September 19, 2014
First Edition

Obesity is none of your boss's business; A new NHS plan for office slimming clubs and jogging groups is wrongheaded

BYLINE: My View Chris Blackhurst

SECTION: NEWS; Pg. 17

LENGTH: 863 words

When the going gets tough, blame business. There's more than a whiff of that in the call from the new head of the NHS, Simon Stevens, that employers should do more to tackle obesity among their workers.

In his first speech about public health since taking charge in April, Stevens said that "obesity is the new smoking, and it represents a slow-motion car crash in terms of avoidable illness and rising healthcare costs".

He suggested a system in which organisations were rewarded with tax breaks for setting up slimming clubs, team weigh-ins and jogging groups. Stevens pointed to his own experience in the US, where he lost three stone in weight while working for an employer that received tax incentives for hitting healthy living targets, including weight loss.

Stevens' intentions are sound. As he said, with the explosion in the number of cases of type 2 diabetes, fuelled by obesity, the health service faces a financial crisis. Nevertheless, his solution leaves an unfortunate taste.

Companies are put upon enough as it is, without feeling obliged to start weighing their employees and putting them on diets. Stevens is erecting yet one more burden, an extra mound of red tape that they could well do without.

Doubtless, the argument will be made that they will enjoy tax advantages - but in all probability that financial gain will not match the sheer hassle, not to mention cost, of establishing running clubs, weighing sessions and the rest. As it is, our taxation system is already the most complex in the world - more complicated, incredibly, than that of India.

Next, to go with their rafts of health and safety officers, compliance officers, and community officers, firms will be advertising for "obesity officers". The nation's army of back-office staff bearing clipboards and not adding directly to the bottom line is bound to grow still further.

Then there's the relationship between employer and employee. They get a tax break if you lose weight. Either you don't try or you do try. In any event you fail to reach the weight loss target. What then? Does it form part of your annual review, another box to be appraised and ticked? In other countries, employers do pay more attention to their workers' health. But that is because they cover the healthcare costs and staff understand where they're coming from. Here, what's being proposed is a fudge, reflecting the fact that private health is not so widely available or commonly accepted. Enabling your employer to enjoy a tax break is not the same as directly bringing down the cost of a private health membership.

Stevens should avoid throwing stones. Before he tackles employers he ought to put his own house in order. He could target obesity among hospital staff, and move to scrap fast-food outlets and vending machines selling chocolate and sweets and fattening **fizzy drinks** in reception areas and corridors.

Until the likes of Burger King, and Costa Coffee with its choice of cakes, pastries and juices carrying four times the recommended daily sugar limit, are excised from hospitals, it is hard to take Stevens' implorations seriously.

Rather than attack people in their workplaces, he should take a good look around - at the snack and burger bars that line, not just our hospital foyers but our high streets. Councils should be financially rewarded for not allowing the opening or expansion of yet more burger joints.

By tackling the suppliers of much of the nation's obesity, and embarking on a mass public information campaign, he begins to get to the heart of the problem. Meal deals offering chocolate, sweets and crisps but not fruit, should be purged by councils armed with new powers.

In attempting to put the onus on employers he's choosing a soft target - one that is also selective, since it does not take in the unemployed, and their drinking and eating habits. Ignoring them and only going after those in work is misguided.

Stevens makes the comparison with smoking. At no time have employers been offered tax incentives to get their workers to kick the habit.

Smoking has been reduced though a combination of unavoidable publicity about the dangers, and attacking cigarettes at source, at supply. That's where, with obesity also, the problem really lies.

People require educating on the harm being overweight can cause, in the same way they were told about smoking. He should look at ways of taxing fatty foods, making his own hospitals healthier, and empowering councils to be able to look at what is being sold on their streets.

Exhorting employers to slim down their workforces is not the answer. It's a task that should rest with government, that they could without. Stevens' buck-passing will not achieve the dramatic turnaround he seeks.

Proof of that, surely, comes from his own past. There he was, losing three stone and making his bosses happy by helping them to acquire tax breaks. But elsewhere, the US went on its sweet, stomachpopping way - regardless of inducements, it continues to have a severe obesity problem.

Stevens is correct to raise the issues. But he needs to rethink the solution.

i@independent.co.uk

People still need educating on the harm being overweight can cause, like with smoking

LOAD-DATE: September 18, 2014

LANGUAGE: ENGLISH

PUBLICATION-TYPE: Newspaper

JOURNAL-CODE: III

Copyright 2014 Independent Print Limited
All Rights Reserved

The INDEPENDENT
Independent.co.uk

September 19, 2014 Friday 3:48 PM GMT

Coca Cola and Rosie Huntington-Whiteley launch London pop-up shop

BYLINE: Laura Chesters

SECTION: BUSINESS NEWS

LENGTH: 203 words

Coca Cola obviously thinks the launch of its new drink Coca-Cola Life did not get enough media coverage because it has drafted in model Rosie Huntington-Whiteley to unveil its new shop.

The temporary shop - known as a pop up - has opened in South Molton Street in London. The drinks giant launched the new drink in the UK this month. It is sweetened with stevia and has a third less sugar and a third fewer calories than normal Coca Cola.

Ms Huntington-Whiteley said: "I'm a great believer in not denying yourself when it comes to what you eat and drink but, like everything in life, it's about finding a healthy and happy balance. With the Coca-Cola Life pop-up boutique we are inviting people to recognise and celebrate those moments as well as create new personal experiences."

Coca Cola trialled the drink in Chile and Argentina and the UK launch is the first in Europe. Its last big launch was back in 2005/2006 with the Coke Zero brand - which was its largest product launch in 22 years.

The stevia sweetener, extracted from a South American leaf, gained European approval in 2011 and is being used in a variety of products to reduce calories. Coca Cola said it already uses it in Sprite and its Glacéau vitaminwater.

LOAD-DATE: September 19, 2014

LANGUAGE: ENGLISH

PUBLICATION-TYPE: Newspaper; Web Publication

JOURNAL-CODE: WEBI

Copyright 2014 Independent Digital News and Media Limited
All Rights Reserved

MailOnline

MailOnline

September 19, 2014 Friday 10:59 PM GMT

Farhan Akhtar launches zero sugar drink

BYLINE: RADHIKA BHALLA

SECTION: INDIANEWS

LENGTH: 324 words

Actor and film-maker Farhan Akhtar was in the Capital to launch the new Coke Zero, a venture by **soft drink** brand Coca-Cola. The product has no **sugar** added and has the tagline 'Great Coke Taste, with Zero **sugar'**.

The event was hosted at The Oberoi on Friday afternoon. Dressed in brown trousers, a black T-shirt and blue blazer with matching slip-ons, the actor presented a new avatar with a moustache that he's growing for an upcoming flick, along with a more buffed physique.

Talking to MAIL TODAY about endorsing the brands he said: "I endorse brands that I personally like. As for the health aspect of the aerated drink, I think that if you exercise, eat well and stay fit, you can drink or eat what you want and still be healthy."

Regarding the 'zero' title, he further said: "I'd like to see zero violence against women, which is a cause that I strongly support. I'd like to see zero pollution, zero child trafficking - the list is endless."

About his upcoming movie Do with Vidhu Vinod Chopra and Bejoy Nambiar, the actor said he was excited to be working with Bollywood megastar Amitabh Bachchan 14 years after Lakshya which Akhtar directed in 2004.

The movie also stars Aditi Rao Hydari, and the story reportedly revolves around a bedridden chess master to be essayed by Big B.

Akhtar also spoke about his debut in Marathi cinema with Por Bazaar which addresses the issue of child trafficking. Others at the do included Olympian luger, Shiva Keshavan, fashion designer Sabyasachi Mukherjee and fashion photographer Atul Kasbekar.

They were joined on stage by Manpreet Gulri, country head, Subway; Alok Tandon, CEO, INOX Leisure; Venkatesh Kini, president, Coca-Cola India and South West Asia and Debabrata Mukherjee, VP Marketing, Coca-Cola India.

As part of inaugurating the black coloured can, four out of the 40,000 fans of Coca-Cola Zero on social media website Facebook, were presented the first packs of the product by Akhtar and Kini.

LOAD-DATE: September 19, 2014

LANGUAGE: ENGLISH

PUBLICATION-TYPE: Web Publication

JOURNAL-CODE: WEBDM

Copyright 2014 Associated Newspapers Ltd.
All Rights Reserved

MailOnline

MailOnline

September 19, 2014 Friday 2:31 AM GMT

We MUST tax sugar before Britain eats itself to death: His father was a Tate & Lyle executive, but IAN BIRRELL believes the food industry needs to be taken on

BYLINE: IAN BIRRELL FOR THE DAILY MAIL

SECTION: NEWS

LENGTH: 1597 words

- Ian Birrell believes tax on sugar should be introduced to halt obesity crisis
- He also suggested tobacco-style warnings and advertising restrictions
- Gastric bands could prove to be most cost-effective treatment, says Birrell
- A quarter of British adults and a fifth of children are now obese

Mr Cube played a big role in my childhood. Not only did he help fund my schooling and summer holidays, but he also filled my house with all manner of sweet delights, syrupy treats and sickly treacles.

For my father was an executive with Tate & Lyle, the country's leading sugar manufacturer, whose packets were decorated with the famous cartoon cube figure.

Every now and then, arriving home from his office, he would deliver the firm's latest innovation from his briefcase for our testing.

As a young boy, I devoured these sugary creations, which I imagined came from some Willy Wonka-style factory overseen by the friendly-looking Mr Cube.

Back in the Sixties and early Seventies, of course, most of the rest of my diet was prepared by my mother or school dinner ladies and, like other children, I spent long hours running around parks and playing fields.

In those days, fast food was a soggy cheese and tomato sandwich.

Today, my son laughs when I tell him I can recall my first McDonald's, savouring those famous burgers as if they were haute cuisine.

How innocent such times seem compared with now, when most children spend hours each day staring at screens, fast-food outlets litter high streets and advertising constantly beseeches us to buy sweet treats.

The result? Obesity has become a national crisis threatening to overwhelm the Health Service, which is being forced to treat ever-growing numbers of patients with weight problems and associated health problems so severe they end up in hospital.

No one should be under any illusion about the scale of the gargantuan challenge this crisis presents.

Just listen to Simon Stevens, the smart new chief executive of the National Health Service, who this week warned that obesity is 'a slow-motion car crash' that threatens to cripple an already overburdened service.

He said: 'If as a nation we keep piling on the pounds around the waistline, we'll be piling on the pounds in terms of future taxes needed just to keep the NHS afloat.'

As politicians talk of raising taxes to further fund the NHS, and hospitals fall into debt, diabetes alone is already draining £1 in every ten spent on healthcare, and excess weight causes almost one in four deaths from heart disease.

Meanwhile, **fizzy drinks** and sweets are causing a surge in tooth decay, with 500 children admitted to hospital each week with rotting teeth.

Health trusts have to splash out on super-size ambulances and £8,000 beds to cope with patients weighing up to 78st.

Alarmingly, this could be just the beginning, as a tidal wave of avoidable 'lifestyle' diseases swamps the NHS.

Shockingly, in clinical terms a quarter of British adults and a fifth of children are obese - numbers which have doubled in the past two decades.

In total, two-thirds of British men and more than half the nation's women are considered to be overweight, which presents a greater threat to public health than smoking.

As a result, we have a generation of children who, because of their diet, their weight and their inactivity, could live shorter lives than their parents and grandparents - which would turn around historic trends of longer life expectancy.

Britain's obesity problems are among the worst in Europe - but this is a global concern, as is all too evident on streets from Baltimore to Beijing and Cape Town to Cairo.

Forget the tired old cliches pushed by the foreign aid industry: two-thirds of the world's overweight people are now found in poorer countries, where more people now go to bed having consumed too many calories than go to bed hungry.

The question, of course, is how to turn the tide - something that defeated every one of the 188 countries studied in a recent Lancet investigation of global obesity over the past three decades.

I used to think the solution was simple when I started to see more and more unsightly mountains of fat and flesh wobbling through British towns and cities: they should eat less and exercise more.

And so they should - this remains the best dietary advice available. Yet blaming gluttony and slothfulness alone will not solve a problem of such severity.

Public warnings from the Government and health experts have had some impact. Sales of **fizzy drinks**, for instance, have fallen over the past six months, while shoppers are buying fewer bags of sugar and more fresh fruit.

Yet, thanks to the addition of so much sugar in processed food and drink, the amount of 'invisible' sugar ending up in the bellies of Britons has risen by almost a third over the past two decades.

This has made doctors increasingly desperate in their calls for action. Some even argue sugar is addictive, having a similar impact on the brain's reward circuits as drugs such as cocaine. Certainly, it's clear many obese patients get locked into a tragic and ever-worsening spiral of depression, low self-esteem and compulsive over-eating.

This is why bariatric surgery - the fitting of gastric bands - may prove ultimately to be the most cost-efficient treatment.

One obese woman told me how she would binge in fast-food joints then pretend not to be hungry at home, while her soaring weight mystified her family. Her growing size made her more miserable, which led her to gorge even more on burgers, chips and fried chicken.

So what is to be done?

Instinctively, I recoil at nanny-state diktats, lectures from politicians (of all people) over how to live our lives, and the idea of government intervention unless absolutely necessary.

But the health crisis unfolding before our eyes demands urgent action - even if it has to be forced on us through legislation.

THERE'S LESS THAN 100 DAYS LEFT: BUY NOW, OR YOU'LL RUIN CHRISTMAS

Put away the suncream and the beach towels - there's fewer than 100 shopping days to go before Christmas.

Last week, online grocer Ocado sent its first email advising customers to book their delivery slot for Christmas, Waitrose has followed suit and Sainsbury's will do so in mid-October.

Selfridges, the smart department store, opened their Xmas shop in the first week of August, while Harrods started taking 'priority bookings' for its Santa's grotto only a week ago. Not to be outdone, catalogue outlet Argos last month published its predictions for the most wanted Christmas toys, with Loom Bands, foam-firing Nerf guns and a customise-your-own Monopoly set expected to sell out.

And this week, 'Christmas' has appeared as one of the most commonly typed words or phrases on Twitter.

But don't panic, website Xmasclock.com has a ticking timer showing the days, hours, minutes and seconds until Christmas Eve, should you forget.

There is a recent precedent which convinces me there is a solution: the war on smoking has shown that social habits can be changed. The imposition of hefty taxes on the cost of cigarettes, along with a smoking ban in public places, has saved thousands of lives - along with millions of pounds of taxpayers' money that might have been wasted on treating smoking-related diseases.

Smoking peaked in 1974, but since then has slumped. Last year alone, sales of manufactured cigarettes fell in this country by another 11 per cent.

Now we need to do something similar to save the nation - and its health service - from drowning in a sea of fat.

This means taking on the food industry and imposing significant taxes on all that lethal sugar they stuff into their wares, even those such as dried fruit, cereals and yoghurts which are promoted as healthy products.

We should also enforce tobacco-style warnings on food and drink packets, as well as wide-ranging advertising restrictions.

Manufacturers and retailers will howl about hitting the poor hardest with the rising prices that would probably result from such a tax, but the truth is that this is the very social group who are the biggest victims of obesity.

No doubt, politicians will prevaricate, fearing they will be blamed for putting up the cost of living.

Naturally, they will also calculate that the cost of political inaction to the health of the nation - not to mention the NHS - will be felt long after they have left office.

But the introduction of a tax on sugar cannot wait. Ask yourself why Asda yesterday announced it was introducing a women's size 32 in its shops, and what that says about the shape of our nation.

It pains me to argue for higher taxes on anything, let alone something so interwoven into my own life. But sometimes statistics do not lie.

One government agency suggested that a 20 per cent tax on soft drinks alone could cut the number of overweight Britons by 250,000.

The curious thing about Tate & Lyle's Mr Cube is that he was a very political figure, created in 1949 at a time when sugar was still rationed and Labour was threatening to nationalise the sugar industry.

When the sword-wielding character first appeared, he angrily attacked state control and promoted private enterprise in speech bubbles.

He symbolised a public mood seeking more freedom to enjoy the sweet things in life, even appearing in political cartoons alongside Clement Attlee and Winston Churchill.

But we live in very different times today - and we need to stop seeing sugar as something benign and cuddly. With apologies to Mr Cube and all that he stood for, the state must take action to confront something that has become a modern scourge.

LOAD-DATE: September 19, 2014

LANGUAGE: ENGLISH

PUBLICATION-TYPE: Web Publication

JOURNAL-CODE: WEBDM

Copyright 2014 Associated Newspapers Ltd.
All Rights Reserved

MailOnline

September 17, 2014 Wednesday 9:30 AM GMT

Just HALF a can of Coke exceeds the new daily sugar guidelines backed by scientists - who recommend just three cubes a day

BYLINE: LIZZIE PARRY FOR MAILONLINE

SECTION: HEALTH

LENGTH: 2062 words

- Study by scientists at University College London has called for recommended daily sugar intake to be slashed to 14g - three cubes a day
- World Health Organisation currently advises a maximum of 10% of our total energy intake from free - or added - sugars, with 5% as a 'target'
- This equates to 50g of free sugars (10 cubes), with 25g (5 cubes) as the target each day - but 14g would be just 3% of energy from added sugars
- One 330ml can of Coca Cola contains 35g of sugars - or 7 cubes
- Bar of Dairy Milk has 7 cubes, while McDonald's Strawberry Milkshake has 12
- Diet Coke and Coke Zero alternatives are both sugar-free and low-calorie
- Calls for sugary food and drinks to be banned from schools and vending machines to be removed from public places
- Experts back a 'sugar tax' to increase the retail price of sugar-rich products

Just half a can of Coca Cola will exceed daily recommended sugar levels if new guidelines - backed by experts - are introduced.

The new move would see the daily recommended sugar intake slashed to just 14g a day - the equivalent of three sugar cubes.

It comes amid calls to ban sugary foods from schools as part of a radical new plan to combat obesity.

Leading academics have said vending machines selling sweets and **fizzy drinks** should also be removed from public places.

They have also called for a 'sugar tax' to increase the retail price of **sugary drinks** and sugar-rich foods by at least 20 per cent.

Current guidelines from the World Health Organisation set a maximum of 10 per cent of total energy intake from free sugars, with five per cent as a 'target'.

Free sugars are defined as those added artificially to foods such as **fizzy drinks** and confectionery, rather than naturally occurring sugars found in fruit.

This equates to around 50g of free sugars (10 cubes) per day as the maximum, with 25g (five cubes) as the target.

However new research from University College London and the London School of Hygiene and Tropical Medicine has advised sugars in a person's diet should make up no more than three per cent of total energy intake.

The latest research suggests that five per cent should be the absolute maximum, say the scientists behind the report.

A 330ml can of Coca Cola contains 35g of sugar, although the Diet Coke and Coke Zero alternatives are sugar-free.

Scientists claim the drastic step is needed to prevent soaring tooth decay and spiraling obesity levels.

Their findings, published in the journal BMC Public Health, analysed the effect of sugars on tooth decay - and found it was the only cause of such damage.

Current guidelines in the UK recommend that 'added' sugars - those used to sweeten food, **fizzy drinks**, honeys, syrups and fruit juices - should not make up more than 10 per cent of the total energy we get from food.

This equates to around 50g of sugar a day - the equivalent to 10 cubes of sugar for adults and older children, and nine for five to 10-year-olds.

But it is just those sugars added as a sweetener that count.

The sugars in milk, vegetables and fruit (not fruit juice), including dried fruit, do not wreak as much havoc on a person's body.

HOW DO OTHER POPULAR SNACKS COMPARE?

- Cadbury's Dairy Milk (45g bar) - 25g of sugar, the equivalent to five cubes
- Two McVitie's Digestive Biscuits (31g) - 5g of sugar, or one cube
- Muller Light yoghurt (175g) - 12.4g of sugar, or just over two cubes
- McDonald's Strawberry Milkshake - 62g of sugar, or 12 cubes
- Galaxy Minstrels (42g bag) - 28.9 of sugar, or six cubes
- Cadbury Twirl (two finger bar) - 24g of sugar, or five cubes
- Kit Kat Chunky - 23.7g of sugar, or four cubes
- Fruit Pastilles (seven sweets) - 15g of sugar, or three cubes

BANISH THE CAKE AND WATCH WHAT YOU DRINK: HOW TO CUT SUGAR FROM YOUR DIET

To maintain a healthy, balanced diet, it is important to cut down on foods and drinks containing added sugars.

These tips can help cut sugar from your diet:

- Choose water or unsweetened fruit juice instead of sugary **fizzy drinks** and juice drinks. Remember to dilute these for children
- If you like **fizzy drinks**, try diluting fruit juice with sparking water
- Swap cakes or biscuits for a currant bun, scone or some malt loaf with low-fat spread
- If you take sugar in hot drinks or add sugar to breakfast cereal, gradually reduce the amount until you can cut it out altogether

- Rather than spreading jam, marmalade, syrup, treacle or honey on your toast, try a low-fat spread, sliced banana or low-fat cream cheese
- Check nutrition labels to help pick the foods with less added sugar, or opt for low-sugar versions
- Try halving the sugar you use in recipes, it works for most things, except jam, meringues and ice cream
- Choose tins of fruit in juice rather than syrup
- Choose wholegrain breakfast cereals, but not those coated with sugar or honey

Source: NHS Choices

The two most common forms of unhealthy added sugars, are table sugar (sucrose) and high-fructose corn-syrup - a liquid sweetener made from maize.

Both are added to countless foods, from **fizzy drinks** to ready meals.

In addition, the sugars found in fruit juices and honeys are also the unhealthy 'added' type.

The NHS Choices website suggests that food or drink containing less than 5g of sugar per 100g is classified as low.

More than 15g per 100g is high.

However, sugar is an important part of a person's daily diet - it is the essential fuel that powers all the cells in the body.

But excess levels have been linked with raised levels of the hormone insulin, which increases the risk of diabetes.

In addition, the body turns excess sugar into fat, which is stored around the major organs, raising the risk of liver and heart disease.

Some experts also fear high-sugar diets may encourage the growth of some cancers. The theory is that glucose, one of the main ingredients in added sugar, creates repeated spikes of insulin.

Many tumours appear to have insulin receptors, hence a rise in this hormone fuels their growth, though studies have failed to explain why.

Study author Aubrey Sheiham, Emeritus Professor of Dental Public Health at UCL, said: 'Tooth decay is a serious problem worldwide and reducing sugars intake makes a huge difference.

'Only two per cent of people at all ages living in Nigeria had tooth decay when their diet contained almost no sugar, around 2g per day.

'This is in stark contrast to the USA, where 92 per cent of adults have experienced tooth decay.'

To tackle tooth decay, the authors recommend a series of radical policy changes to reduce sugar consumption.

Co-author Professor Philip James, honorary professor of nutrition at the London School of Hygiene and Tropical Medicine, said: 'Our top priority is not to allow the idea of a magic single bullet to solve the problem to be developed.

'There now needs to be an explicit revision of population dietary goals as it relates to every aspect of government policy.

'We need to make sure that use of fruit juices and the concept of sugar-containing treats for children are not only no longer promoted, but explicitly seen as unhelpful.

'Food provided at nurseries and schools should have a maximum of free sugars in the complete range of foods amounting to no more than 2.5 per cent of energy.

'Vending machines offering confectionary and **sugary drinks** in areas controlled or supported financially by local or central government should be removed.

'We are not talking draconian policies to 'ban' such sugar-rich products, which are available elsewhere, but no publicly-supported establishment should be contributing to the expensive problems of dental caries, obesity and diabetes.

'The food industry should be told that they should progressively reformulate their products to reduce or preferably remove all the sugars from their products.'

'New food labels should label anything above 2.5 per cent sugars as 'high'.

'Given the politics of big business, the most governments may do is to reduce the limit from 10 per cent to 5 per cent but our paper suggests that it should be 2.5 per cent.

'There is a huge issue about how to curtail the flow of sugars in the food chain and divert sugar.

'If produced at all, it should be converted into alcohol, as in Brazil, to be used as fuel for vehicles.'

He added: 'A sugars tax should be developed to increase the cost of sugar-rich food and drinks. This would be simplest as a tax on sugar as a mass commodity, since taxing individual foods depending on their sugar content is an enormously complex administrative process.

'The retail price of **sugary drinks** and sugar rich foods needs to increase by at least 20 per cent to have a reasonable effect on consumer demand so this means a major tax on sugars as a commodity.

'The level will depend on expert analyses but my guess is that a 100 per cent tax might be required.'

However, some experts have warned such drastic measures will not be feasible, branding plans to reach a 5 per cent target a 'struggle', blaming society's reliance on processed food and drink.

Mel Wakeman, senior lecturer in applied physiology at Birmingham City University, said: 'Although many large food companies have pledged to reduce the amount of sugar in their products, this does not happen overnight and we still consume too much processed food and drink to be able to achieve the new recommended sugar allowance.

'I agree we need to cut our sugar intake but many will struggle to reach a 5 per cent target, never mind 3 per cent.

'It is unrealistic at the moment to ask the population to have negligible sugar in their diet but the public do need help to reduce sugar intake from current levels.'

The British **Soft Drinks** Association said more evidence is needed before a '**sugar** tax' should be introduced.

Gavin Partington, BSDA Director General, said: 'We should rely on more than Professor James' guesswork to imagine the impact of a tax that would push prices up for millions of consumers with no clear impact on public health as politicians in Denmark have found.

'The authors of this report appear to have ignored the strict rules on what drinks are allowed to be sold in schools and drinks containing added sugar are not permitted.

'To suggest that fruit juice should not be promoted, when evidence shows it helps people towards their five fruit and veg a day, suggests they are rather more concerned with their campaign than with delivering public health outcomes.'

MailOnline has contacted Coca Cola for comment.

COCA COLA WILL DISPLAY RED WARNING LOGO ON ITS CANS TO INDICATE HIGH SUGAR CONTENT, IN U-TURN BY BOSSES

Earlier this month Coca Cola agreed to put a red warning logo on its cans, to indicate high sugar content.

It marked a U-turn by bosses, as they decided to adopt the colour coded system of traffic light labels, which is designed to help consumers identify healthy products and improve their diet.

Coca-Cola, like many other international brands, had long fought against the use of colour coded labels, largely because they did not want a red warning logo on cans and bottles.

However, it has now decided to fall into line, not least because sales of its full sugar original versions are actually well below those for its healthier alternatives, Coke Zero and Diet Coke.

The traffic light labelling system uses red, amber and green on a front of pack nutrition label to identify whether products are high medium or low in sugar, fat, and salt.

There are also figures to show how much one portion of the product contributes to the daily recommended maximum.

The company said its adoption of the new labels were consistent with a commitment to provide consumers with transparent nutrition information on the front of its packs.

Jon Woods, general manager of Coca-Cola UK and Ireland, said: 'We carefully considered the new scheme when it was announced last year but decided to continue with the single-colour GDA (guideline daily amounts) system that was tried and tested across Europe.

'We have monitored the labelling scheme since it started to appear in-store and asked shoppers in Great Britain for their views.

'Our UK consumers have told us they want a single, consistent front-of-pack labelling scheme across all food and drink products to help them make the right choices for them and their families.

'We have therefore decided to put the new scheme on our packs here.'

LOAD-DATE: September 17, 2014

LANGUAGE: ENGLISH

PUBLICATION-TYPE: Web Publication

JOURNAL-CODE: WEBDM

Copyright 2014 Associated Newspapers Ltd.
All Rights Reserved

MailOnline

September 19, 2014 Friday 12:52 AM GMT

We MUST tax sugar before Britain eats itself to death: His dad was a Tate & Lyle executive, but IAN BIRRELL believes the food industry needs to be taken on

BYLINE: IAN BIRRELL FOR THE DAILY MAIL

SECTION: NEWS

LENGTH: 1597 words

- Ian Birrell believes tax on sugar should be introduced to halt obesity crisis
- He also suggested tobacco-style warnings and advertising restrictions
- Gastric bands could prove to be most cost-effective treatment, says Birrell
- A quarter of British adults and a fifth of children are now obese

Mr Cube played a big role in my childhood. Not only did he help fund my schooling and summer holidays, but he also filled my house with all manner of sweet delights, syrupy treats and sickly treacles.

For my father was an executive with Tate & Lyle, the country's leading sugar manufacturer, whose packets were decorated with the famous cartoon cube figure.

Every now and then, arriving home from his office, he would deliver the firm's latest innovation from his briefcase for our testing.

As a young boy, I devoured these sugary creations, which I imagined came from some Willy Wonka-style factory overseen by the friendly-looking Mr Cube.

Back in the Sixties and early Seventies, of course, most of the rest of my diet was prepared by my mother or school dinner ladies and, like other children, I spent long hours running around parks and playing fields.

In those days, fast food was a soggy cheese and tomato sandwich.

Today, my son laughs when I tell him I can recall my first McDonald's, savouring those famous burgers as if they were haute cuisine.

How innocent such times seem compared with now, when most children spend hours each day staring at screens, fast-food outlets litter high streets and advertising constantly beseeches us to buy sweet treats.

The result? Obesity has become a national crisis threatening to overwhelm the Health Service, which is being forced to treat ever-growing numbers of patients with weight problems and associated health problems so severe they end up in hospital.

No one should be under any illusion about the scale of the gargantuan challenge this crisis presents.

Just listen to Simon Stevens, the smart new chief executive of the National Health Service, who this week warned that obesity is 'a slow-motion car crash' that threatens to cripple an already overburdened service.

He said: 'If as a nation we keep piling on the pounds around the waistline, we'll be piling on the pounds in terms of future taxes needed just to keep the NHS afloat.'

As politicians talk of raising taxes to further fund the NHS, and hospitals fall into debt, diabetes alone is already draining £1 in every ten spent on healthcare, and excess weight causes almost one in four deaths from heart disease.

Meanwhile, **fizzy drinks** and sweets are causing a surge in tooth decay, with 500 children admitted to hospital each week with rotting teeth.

Health trusts have to splash out on super-size ambulances and £8,000 beds to cope with patients weighing up to 78st.

Alarmingly, this could be just the beginning, as a tidal wave of avoidable 'lifestyle' diseases swamps the NHS.

Shockingly, in clinical terms a quarter of British adults and a fifth of children are obese - numbers which have doubled in the past two decades.

In total, two-thirds of British men and more than half the nation's women are considered to be overweight, which presents a greater threat to public health than smoking.

As a result, we have a generation of children who, because of their diet, their weight and their inactivity, could live shorter lives than their parents and grandparents - which would turn around historic trends of longer life expectancy.

Britain's obesity problems are among the worst in Europe - but this is a global concern, as is all too evident on streets from Baltimore to Beijing and Cape Town to Cairo.

Forget the tired old cliches pushed by the foreign aid industry: two-thirds of the world's overweight people are now found in poorer countries, where more people now go to bed having consumed too many calories than go to bed hungry.

The question, of course, is how to turn the tide - something that defeated every one of the 188 countries studied in a recent Lancet investigation of global obesity over the past three decades.

I used to think the solution was simple when I started to see more and more unsightly mountains of fat and flesh wobbling through British towns and cities: they should eat less and exercise more.

And so they should - this remains the best dietary advice available. Yet blaming gluttony and slothfulness alone will not solve a problem of such severity.

Public warnings from the Government and health experts have had some impact. Sales of **fizzy drinks**, for instance, have fallen over the past six months, while shoppers are buying fewer bags of sugar and more fresh fruit.

Yet, thanks to the addition of so much sugar in processed food and drink, the amount of 'invisible' sugar ending up in the bellies of Britons has risen by almost a third over the past two decades.

This has made doctors increasingly desperate in their calls for action. Some even argue sugar is addictive, having a similar impact on the brain's reward circuits as drugs such as cocaine. Certainly, it's clear many obese patients get locked into a tragic and ever-worsening spiral of depression, low self-esteem and compulsive over-eating.

This is why bariatric surgery - the fitting of gastric bands - may prove ultimately to be the most cost-efficient treatment.

One obese woman told me how she would binge in fast-food joints then pretend not to be hungry at home, while her soaring weight mystified her family. Her growing size made her more miserable, which led her to gorge even more on burgers, chips and fried chicken.

So what is to be done?

Instinctively, I recoil at nanny-state diktats, lectures from politicians (of all people) over how to live our lives, and the idea of government intervention unless absolutely necessary.

But the health crisis unfolding before our eyes demands urgent action - even if it has to be forced on us through legislation.

THERE'S LESS THAN 100 DAYS LEFT: BUY NOW, OR YOU'LL RUIN CHRISTMAS

Put away the suncream and the beach towels - there's fewer than 100 shopping days to go before Christmas.

Last week, online grocer Ocado sent its first email advising customers to book their delivery slot for Christmas, Waitrose has followed suit and Sainsbury's will do so in mid-October.

Selfridges, the smart department store, opened their Xmas shop in the first week of August, while Harrods started taking 'priority bookings' for its Santa's grotto only a week ago. Not to be outdone, catalogue outlet Argos last month published its predictions for the most wanted Christmas toys, with Loom Bands, foam-firing Nerf guns and a customise-your-own Monopoly set expected to sell out.

And this week, 'Christmas' has appeared as one of the most commonly typed words or phrases on Twitter.

But don't panic, website Xmasclock.com has a ticking timer showing the days, hours, minutes and seconds until Christmas Eve, should you forget.

There is a recent precedent which convinces me there is a solution: the war on smoking has shown that social habits can be changed. The imposition of hefty taxes on the cost of cigarettes, along with a smoking ban in public places, has saved thousands of lives - along with millions of pounds of taxpayers' money that might have been wasted on treating smoking-related diseases.

Smoking peaked in 1974, but since then has slumped. Last year alone, sales of manufactured cigarettes fell in this country by another 11 per cent.

Now we need to do something similar to save the nation - and its health service - from drowning in a sea of fat.

This means taking on the food industry and imposing significant taxes on all that lethal sugar they stuff into their wares, even those such as dried fruit, cereals and yoghurts which are promoted as healthy products.

We should also enforce tobacco-style warnings on food and drink packets, as well as wide-ranging advertising restrictions.

Manufacturers and retailers will howl about hitting the poor hardest with the rising prices that would probably result from such a tax, but the truth is that this is the very social group who are the biggest victims of obesity.

No doubt, politicians will prevaricate, fearing they will be blamed for putting up the cost of living.

Naturally, they will also calculate that the cost of political inaction to the health of the nation - not to mention the NHS - will be felt long after they have left office.

But the introduction of a tax on sugar cannot wait. Ask yourself why Asda yesterday announced it was introducing a women's size 32 in its shops, and what that says about the shape of our nation.

It pains me to argue for higher taxes on anything, let alone something so interwoven into my own life. But sometimes statistics do not lie.

One government agency suggested that a 20 per cent tax on soft drinks alone could cut the number of overweight Britons by 250,000.

The curious thing about Tate & Lyle's Mr Cube is that he was a very political figure, created in 1949 at a time when sugar was still rationed and Labour was threatening to nationalise the sugar industry.

When the sword-wielding character first appeared, he angrily attacked state control and promoted private enterprise in speech bubbles.

He symbolised a public mood seeking more freedom to enjoy the sweet things in life, even appearing in political cartoons alongside Clement Attlee and Winston Churchill.

But we live in very different times today - and we need to stop seeing sugar as something benign and cuddly. With apologies to Mr Cube and all that he stood for, the state must take action to confront something that has become a modern scourge.

LOAD-DATE: September 19, 2014

LANGUAGE: ENGLISH

PUBLICATION-TYPE: Web Publication

JOURNAL-CODE: WEBDM

Copyright 2014 Associated Newspapers Ltd.
All Rights Reserved

The Express

September 16, 2014 Tuesday
Edition 1;
Northern Ireland

'Ban sugary junk food from schools'

BYLINE: Jo Willey

SECTION: NEWS; Pg. 11

LENGTH: 156 words

JUNK food loaded with sugar should be taxed and banned from schools in the war on obesity and tooth decay, say food experts.

Vending machines selling sweets and **fizzy drinks** should also be banished from public places, according to leading British academics. Studies show that sugars in the diet should make up no more than three per cent of total energy intake.

Philip James, honorary professor of nutrition at the London School of Hygiene & Tropical Medicine, said: "Vending machines offering confectionary and **sugary drinks** in areas controlled or supported financially by local or central government should be removed."

He added: "A sugars tax should be developed to increase the cost of sugar-rich food and drinks. This would be simplest as a tax on sugar as a mass commodity."

The research, from experts at University College London and the LSHTM, published in the journal BMC Public Health, analysed the effect of sugars on tooth decay.

LOAD-DATE: September 16, 2014

LANGUAGE: ENGLISH

PUBLICATION-TYPE: Newspaper

MailOnline

September 16, 2014 Tuesday 10:05 AM GMT

Put sugar tax on unhealthy products to prevent obesity and tooth decay, say scientists

BYLINE: SOPHIE BORLAND FOR THE DAILY MAIL

SECTION: HEALTH

LENGTH: 539 words

- **Fizzy drinks** and sweets should be doubled in price, researchers say
- They say it will help to prevent an epidemic of tooth decay
- Say the NHS spends more on tooth decay than any other illness, bar cancer

Fizzy drinks and sweet treats should be doubled in price to prevent an epidemic of tooth decay, according to researchers.

They are calling for a 100 per cent **sugar** tax to be slapped on **soft drinks** and confectionary to deter the public from consuming such vast quantities.

Earlier this year the chief medical officer Professor Dame Sally Davies conceded that a sugar tax may be necessary to tackle obesity although ministers say there are no plans for such measures.

But researchers from the London School of Hygiene and Medicine say it is urgently needed to prevent rotting teeth in adults and children.

They say the NHS now spends more treating tooth decay than any other illness apart from cancer and it causes so much pain it severely impacts patients' quality of life.

Figures show that 500 children are admitted to hospital every week with rotting teeth and more than a quarter of youngsters have some degree of decay.

Professor Philip James, lead researcher, said a number of drastic proposals were needed including sugar tax of at least 20 per cent but ideally 100 per cent.

This would raise the price of a can of Coke to 84p, a Mars bar to £1.20 and a Cornetto ice cream to £2.50.

Writing in the journal BMC Public Health, he also called for other drastic measures including schools to stop serving fruit juice and vending machines to be banned from public places.

Professor James said: 'A sugars tax should be developed to increase the cost of sugar-rich food and drinks.

'This would be simplest as a tax on sugar as a mass commodity, since taxing individual foods depending on their sugar content is an enormously complex administrative process.

'The retail price of **sugary drinks** and sugar rich foods needs to increase by at least 20 per cent to have a reasonable effect on consumer demand so this means a major tax on sugars as a commodity.

'The level will depend on expert analyses but my guess is that a 100 per cent tax might be required.

'Vending machines offering confectionery and **sugary drinks** in areas controlled or supported financially by local or central government should be removed.

'We are not talking draconian policies to 'ban' such sugar-rich products, which are available elsewhere, but no publicly-supported establishment should be contributing to the expensive problems of dental caries, obesity and diabetes.'

'We need to make sure that use of fruit juices and the concept of sugar-containing treats for children are not only no longer promoted, but explicitly seen as unhelpful.'

He also said nurseries and schools should stop serving sugar-rich drinks including fruit juice and treats.

The Government says it has no plans for a sugar tax and instead is working with food and drinks manufacturers to make products less sweet.

Only yesterday, American researchers said that sugar - not salt- was to blame for high blood pressure.

Dr James DiNicolantonio, a heart disease specialist at Saint Luke's Mid America Heart Institute in Kansas City, said it affects a key area of the brain that causes the heart rate to quicken.

LOAD-DATE: September 16, 2014

LANGUAGE: ENGLISH

PUBLICATION-TYPE: Web Publication

JOURNAL-CODE: WEBDM

Copyright 2014 Associated Newspapers Ltd.
All Rights Reserved

FREE METRO

Metro (UK)

September 16, 2014 Tuesday
Edition 1;
National Edition

War on sugar 'will cut decay'

SECTION: NEWS; Pg. 23

LENGTH: 64 words

SUGARY food should be banned from schools to cut soaring levels of tooth decay, experts say. Vending machines with sweets and **sugary drinks** must be removed from public areas, they insist. A tax raising the price of sugar-rich food and drinks by 20 per cent would help, according to a study by UCL and the London School of Hygiene and Tropical Medicine, published in BMC Public Health.

LOAD-DATE: September 16, 2014

LANGUAGE: ENGLISH

PUBLICATION-TYPE: Newspaper

JOURNAL-CODE: MTR

Copyright 2014 Associated Newspapers Ltd.
All Rights Reserved

THE TIMES

The Times (London)

September 16, 2014 Tuesday
Edition 2;
National Edition

Call for sugar controls in fight against tooth decay

BYLINE: Kat Lay

SECTION: NEWS; Pg. 11

LENGTH: 454 words

The amount of sugar we eat should be severely restricted, experts said yesterday, as new research revealed the extent of tooth decay caused by our diets. Researchers concluded that sugars should make up no more than 3 per cent of total energy intake - about 15g a day, well under the amount found in one can of cola.

The study, published today in the journal BMC Public Health, looked at health records from countries across the world to compare dental records with diet over time. They found that adults were far more likely to have tooth decay than children, and its prevalence increased in line with sugar consumption.

"Tooth decay is a serious problem worldwide and reducing sugar intake makes a huge difference." said Aubrey Sheiham, emeritus professor of dental public health at UCL and a co-author of the study.

"Data from Japan were particularly revealing, as the population had no access to sugar during or shortly after the Second World War. We found that decay was hugely reduced during this time, but then increased as they began to import sugar again.

"Similarly, only 2 per cent of people living in Nigeria had tooth decay when their diet contained almost no sugar. This is in stark contrast to the USA, where 92 per cent of adults have experienced tooth decay."

In children, an increase from nearzero sugar to 5 per cent of energy doubled the prevalence of tooth decay.

The present government guidelines set a maximum of 10 per cent of total energy intake from sugars, although advisers are looking at plans to reduce that by half.

Philip James, honorary professor of nutrition at the London School of Hygiene & Tropical Medicine and coauthor of the study, said: "We need to make sure that fruit juices and sugarcontaining treats for children are explicitly seen as unhelpful.

"Food provided at nurseries and schools should have a maximum of free sugars amounting to no more than 2.5 per cent of energy.

"Vending machines offering confectionery and **sugary drinks** in areas controlled or supported financially by local or central government should be removed.

"We are not talking draconian policies to ban sugar-rich products, which are available elsewhere, but no publicly supported establishment should be contributing to the expensive problems of dental caries, obesity and diabetes."

He also called for a sugar tax, with the money raised going to fund health services.

Katharine Jenner, a nutritionist and the campaign director for Action on Sugar, said: "Tooth decay, caused by sugars, is one of the most universal and costly health problems, yet is often overlooked . Teenagers are eating around 20 teaspoons of sugars a day - five times the amount recommended in this paper. We need to take serious action on sugar now."

LOAD-DATE: September 16, 2014

LANGUAGE: ENGLISH

PUBLICATION-TYPE: Newspaper

JOURNAL-CODE: TIM

Copyright 2014 Times Newspapers Limited
All Rights Reserved

DAILY MAIL (London)

September 15, 2014 Monday

STUDY'S CONTROVERSIAL CLAIM

BYLINE: BY SOPHIE BORLAND HEALTH CORRESPONDENT

LENGTH: 514 words

SUGAR - not salt - is to blame for high blood pressure, researchers suggest.

They argue that high sugar levels affect a key area of the brain, causing the heart rate to quicken and blood pressure to rise.

The American scientists highlight a recent study of 8,670 French adults which found no link between salt and high blood pressure.

For years the public have been urged to restrict their salt intake to a teaspoon a day, and it is blamed for up to 3million deaths worldwide each year.

But researchers led by Dr James DiNicolantonio, a heart disease specialist at Saint Luke's Mid America Heart Institute in Kansas City, believe sugar may be the real danger.

Writing in the American Journal of Cardiology, they state: It is sugar, not the salt, that may be the actual causative factor for high blood pressure.

This notion is supported by... analyses of randomised control trials (large-scale studies) suggesting that sugar is more strongly related to blood pressure in humans than sodium.

Encouraging consumers to hold the sugar, not the salt, may be the better dietary strategy to achieve blood pressure control.'

Around a quarter of British adults have high blood pressure, which experts say greatly increases their risk of a stroke and makes them vulnerable to heart disease.

The team believes high sugar levels may increase blood pressure by affecting part of the brain linked to hormone production, called the hypothalamus.

Dr DiNicolantonio also contradicts experts who claim that reducing salt consumption will lower levels of obesity. He added: We argue the opposite, a reduction in salt intake may lead to an increased intake in processed foods (and added sugars) and thereby increase the risk of diabetes, obesity, and cardiovascular disease.'

The team's conclusions are further evidence of the risks posed by eating too much sugar.

In June, officials issued new guidelines urging the public to limit themselves to between five and eight teaspoons a day over concerns it is causing obesity and type 2 diabetes.

However the average adult consumes around 15 teaspoons, largely due to the high levels hidden in cereals, yoghurt, sandwiches and ready meals.

Dr Aseem Malhotra, a cardiologist and science director of campaign group Action On Sugar, said the public health risk posed by sugar had been underestimated. He said: We know that sugar does not provide any nutrients and there is growing evidence it is a... risk factor for many diseases.'

But Professor Graham MacGregor, an expert in cardiovascular medicine at Queen Mary, University of London, said evidence blaming sugar for high blood pressure was incredibly weak'.

He added that research spanning several decades had shown a strong link between salt and high blood pressure.

Experts have previously called on the Government to impose a sugar tax on **fizzy drinks** - although ministers have so far rejected the idea.

The Government's chief advisor on obesity, Professor Susan Jebb, has also urged parents to ban **sugary drinks** from the dinner table amid fears over a rise in tooth decay among children.

s.borland@dailymail.co.uk

© Daily Mail

LOAD-DATE: September 14, 2014

LANGUAGE: ENGLISH

PUBLICATION-TYPE: Papers

Copyright 2014 Associated Newspapers Ltd.
All Rights Reserved

JOURNAL-CODE: DXP

Copyright 2014 Express Newspapers
All Rights Reserved

MailOnline

MailOnline

September 15, 2014 Monday 5:01 PM GMT

Obese Australians drink roughly 152ml of soft drink PER DAY... and one in four people regularly sip on fizzy beverages

BYLINE: AAP

SECTION: NEWS

LENGTH: 256 words

- Overweight people more likely to drink **sugary drinks** over healthy **beverages**
- 23% of people drink **soft drink** every day
- Two surveys conducted by Western Australia and South Australia governments found men drink **soft drink** more than women
- Health expert warns young people to be aware of the serious consequences when drinking **sugary beverages**

People who consume **soft drink** are significantly more likely to be overweight and sip more of the **sugary beverage** than their healthy counterparts, research reveals.

An analysis of two government surveys in Western Australia and South Australia has found about 23 per cent of people drink soft drink every day.

In both surveys, men were more likely than women to have a soft drink, and beverage consumption decreased with age.

Curtin University researcher Christina Pollard said people who drank diet refreshments were more likely to be overweight, but overweight and obese people were also more likely to be soft drink consumers.

People of a healthy weight drank about 80ml of soft drink a day, while obese people drank up to 152ml.

Ms Pollard told the Public Health Association of Australia conference in Perth on Monday that interventions to limit soft drink consumption needed to be developed.

She said consumption increased during the warmer months and after a growth in brands' spending on advertising.

Young people in particular were not aware of the consequences of drinking **sugary beverages**, which could cause weight gain, tooth erosion and poor dietary quality when consumed in excess, Ms Pollard said.

LOAD-DATE: September 15, 2014

LANGUAGE: ENGLISH

PUBLICATION-TYPE: Web Publication

JOURNAL-CODE: WEBDM

Copyright 2014 Associated Newspapers Ltd.
All Rights Reserved

The INDEPENDENT

Independent.co.uk

September 14, 2014 Sunday 4:23 PM GMT

French campaigners call for ban on free fizzy drink refills; French nutritionists have called for a ban on unlimited fizzy drink re-fills in restaurants.

BYLINE: Helen Lock

SECTION: FRONTPAGE

LENGTH: 243 words

Campaigners in France have spoken out against the introduction of "drink as much as you like" drinks machines at two popular burger chains.

Two fast-food restaurants, Quick and KFC, have recently installed American-style machines so that customers can consume unlimited amounts of drinks like lemonade and Coke, the Daily Telegraph has reported.

The move has caused outrage among French chefs and nutritionists.

Serge Hercberg, the head of the National Nutrition and Health Programme, a government healthy eating initiative, said: "This must be banned. It is in total contradiction with public health recommendations."

"I oppose all marketing practices that encourage people to consume excessive quantities of unhealthy products," he told the Telegraph.

A French chef and food campaigner, Xavier Denamur, said: "Anything that encourages people to eat as much as they like or drink as much as they like is a catastrophe. It should be outlawed."

Demamur said that the government in France was telling people to eat less fat, sugar and salt and there is a "largely state-owned" chain (Quick) getting kids hooked on **fizzy drinks**. "People get more addicted to sugar than cocaine," he said.

While obesity rates in France remain lower than most Western countries, fast-food is becoming more popular. The French spend more on burgers and sandwiches than on traditional bistros, and McDonalds has 1,285 restaurants in France, making it one of the chain's most profitable markets.

LOAD-DATE: September 15, 2014

LANGUAGE: ENGLISH

PUBLICATION-TYPE: Newspaper; Web Publication

JOURNAL-CODE: WEBI

Copyright 2014 Independent Digital News and Media Limited
All Rights Reserved

MailOnline

September 14, 2014 Sunday 4:25 PM GMT

Sugar could be worse for your blood pressure than salt, shock new research reveals

BYLINE: SOPHIE BORLAND FOR THE DAILY MAIL

SECTION: HEALTH

LENGTH: 676 words

- For years the public have been urged to slash their salt intake
- It had been thought salt increases the risk of strokes by a quarter and it has been blamed on 3 million deaths worldwide annually
- But study claims sugar is more related to blood pressure than sodium

Sugar - not salt - is to blame for high blood pressure, US researchers claim.

They argue that high sugar levels affect a key area of the brain which causes the heart rate to quicken and blood pressure to rise.

The scientists from New York and Kansas also highlight a recent study of 8,670 French adults which found no link between salt and high blood pressure.

Scroll down for video

For years the public have been urged to slash their salt intake and guidelines state it should be restricted to a teaspoon a day.

Experts say it increases the risk of strokes by a quarter and it has been blamed on 3 million deaths worldwide annually.

But in an article in the American Journal of Cardiology, researchers led by Dr James DiNicolantonio state 'It is sugar not the salt that may be the actual causative factor for high blood pressure.

'This notion is supported by meta analyses of randomised control trials (large-scale studies) suggesting that sugar is more strongly related to blood pressure in humans than sodium.

'Encouraging consumers to hold the sugar, not the salt, may be the better dietary strategy to achieve blood pressure control.'

Around a quarter of adults in the UK have high blood pressure and it greatly increases the risk of strokes and heart disease.

This research is further evidence of the health risks posed by eating too much sugar.

In June, officials issued new guidelines urging the public to limit themselves to between five and seven teaspoons a day over concerns it is causing obesity and type 2 diabetes.

The American researchers believe that high sugar levels affect a key area of the brain called the hypothalamus which causes the heart rate to quicken and blood pressure to rise.

They also think it may cause our bodies to produce more insulin, a hormone, which may also speed up the heart rate.

Dr DiNicolantonio, a heart disease research scientist at Saint Luke's Mid America Heart Institute in Kansas City also contradicts experts who claim reducing salt consumptions will lower levels of obesity and heart disease.

He adds: 'We argue the opposite, a reduction in salt intake may lead to an increased intake in processed foods (and added sugars) and, thereby, increase the risk of diabetes, obesity, and cardiovascular disease.'

The reasons are complicated but it is thought that low salt levels increase the amount of certain fats in the blood.

Dr Aseem Malhotra, a cardiologist and science director of Action on Sugar, a campaign group, said the public health risk posed by sugar had been underestimated.

He added: 'We know that sugar does not provide any nutrients and there is growing evidence it is an independent risk factor for many diseases.

But Professor Graham McGregor, an expert in cardiovascular medicine at Queen Mary, the University of London, said the evidence blaming sugar rather than salt for high blood pressure was 'incredibly weak.'

He said that research spanning several decades had shown a strong link between salt and high blood pressure.

Guidelines from Public Health England, the agency responsible for tackling obesity, in June stated that women should have no more than five to six teaspoons of sugar a day and men seven to eight.

But the average adult consumes 15 teaspoons largely due to the high levels of hidden sugar in fruit juice, muesli, yoghurts, sandwiches and ready meals.

Some experts want the Government to impose a sugar tax on **fizzy drinks** although ministers have so far rejected these calls.

The Government's chief advisor on obesity, Professor Susan Jebb, has also urged parents to ban fruit juice and **fizzy drinks** from the dinner table and stick to water.

Experts are also worried that sugar is behind an increase in tooth decay in children and could affect their ability to learn.

LOAD-DATE: September 14, 2014

LANGUAGE: ENGLISH

PUBLICATION-TYPE: Web Publication

JOURNAL-CODE: WEBDM

Copyright 2014 Associated Newspapers Ltd.
All Rights Reserved

The Sunday Telegraph

The Sunday Telegraph (London)

September 14, 2014
Edition 1;
National Edition

France in a fizz over free pop

BYLINE: DAVID CHAZAN

SECTION: FEATURES; Pg. 27

LENGTH: 222 words

THE INTRODUCTION of "drink as much you like" soda fountains by two fast-food chains in France has stirred up an outcry from campaigners determined to stop the "Americanisation" of the country.

The popular French hamburger chain Quick and fried chicken outlet KFC have started offering free refills of fizzy and **sugary drinks** with meals.

"This must be banned," said Serge Hercberg, the head of the National Nutrition and Health Programme, a government initiative to encourage healthy eating. "It is in total contradiction with public health recommendations."

France introduced a tax on **sugary drinks** three years ago in an attempt to slim down the average Frenchman, who has put on about 6.6lb since 1997. **Fizzy drinks** are banned in school canteens.

François Charpy of Quick, which has 400 outlets in France, said that the scheme had not led to overindulgence, adding that the increase in the quantities of **fizzy drinks** dispensed was "no more than 10 per cent". He said the cost to the chain was small, and that the policy attracted customers.

Obesity rates in France remain lower than in most Western countries, but the national appetite for fast food is growing.

The French get though an average of 14 burgers each per year, making them Europe's second biggest burger eaters after the British, who consume 17, according to a recent survey.

LOAD-DATE: September 14, 2014

LANGUAGE: ENGLISH

PUBLICATION-TYPE: Newspaper

JOURNAL-CODE: STL

Copyright 2014 Telegraph Media Group Limited
All Rights Reserved

The Telegraph
telegraph.co.uk

September 13, 2014 Saturday 4:39 PM GMT

French call for ban on 'free refills' of fizzy drinks; French nutritionists want to ban 'drink as much you like' soda fountains in bid to halt the spread of obesity

BYLINE: By David Chazan Paris

SECTION: NEWS

LENGTH: 627 words

It is a cause célèbre for campaigners determined to save the French from turning into a nation of Coke-guzzling hamburger munchers.

The recent introduction of American-style "drink as much you like" soda fountains by two fast-food chains in France has sparked an outcry from nutritionists and doctors fighting the spread of obesity, and chefs struggling to preserve the deep-rooted tradition of Gallic gastronomy.

The popular French hamburger chain Quick and KFC have started offering free refills of fizzy and **sugary drinks** with meals of burgers and fries.

"This must be banned," said Serge Hercberg, the head of the National Nutrition and Health Programme, a government initiative to encourage healthy eating.

"It is in total contradiction with public health recommendations," said Dr Hercberg, who is also professor of nutrition at a leading Paris medical school. "I oppose all marketing practices that encourage people to consume excessive quantities of unhealthy products."

France introduced a tax on **sugary drinks** three years ago in a bid to slim down the average Frenchman, who has put on about 3 kgs since 1997. **Fizzy drinks** are banned in school canteens.

At a Quick outlet in Paris, Nabil, a 23-year-old student at a business school who was serving himself a second beaker of lemonade, shrugged off the health concerns. "I think it's a winning concept," he said. "At my age, you don't worry too much about health, but if I drink more than three I feel bloated."

Nineteen-year-old Francine, a supermarket checkout girl, said: "It's great. I only take a little bit more and it costs nothing."

François Charpy of Quick, which has 400 outlets in France, claims that the scheme has not led to overindulgence, saying the increase in the quantities of **fizzy drinks** dispensed is "no more than 10 per cent". He said the cost to the chain was small and it attracted customers.

However, 14-year-old Eric was gulping down his fourth beaker of his favourite soda. "My parents tell me not to drink too much fizzy stuff, but I really like it," he said.

Xavier Denamur, a leading French chef and one of the country's most prominent campaigners against junk food, said: "Anything that encourages people to eat as much they like or drink as much as they like is a catastrophe. It should be outlawed."

Mr Denamur, who owns four restaurants in Paris, pointed out that the French state owned a large stake in Quick but the chain was "going against government policy".

"The government is telling people to eat less fat, less sugar and less salt, and here we've got a largely state-owned chain that's going to get kids hooked on **fizzy drinks**. People get more addicted to sugar than cocaine," he said.

Obesity rates in France remain lower than in most western countries, but the national appetite for fast food is growing. The French now spend more on burgers and sandwiches than on traditional sit-down bistros. Hamburgers and soft drinks have replaced steak and red wine in the diets of many younger people.

The French get though an average of 14 burgers each per year, making them Europe's second biggest burger eaters after the British, who consume 17, according to a recent survey.

McDonalds has 1,285 restaurants in France, which is one of its most profitable markets, generating sales of nearly (EURO)4.5 billion (£3.6 billion) a year.

The symbol of the American way of eating has also found its way on to the menus of high-end restaurants, with Gallic touches such as foie gras added to the ingredients.

However, Dr Hercberg said low-income people were the main customers of fast-food chains. "It is the most disadvantaged people who are going to suffer from the unlimited supply of fizzy and **sugary drinks**," he said. "We have to fight social inequality in nutrition."

LOAD-DATE: September 15, 2014

LANGUAGE: ENGLISH

PUBLICATION-TYPE: Newspaper; Web Publication

JOURNAL-CODE: WEBDTNS

Copyright 2014 Telegraph Media Group Limited

All Rights Reserved

The INDEPENDENT

The Independent (London)

September 12, 2014 Friday
First Edition

Ecuador to tax fast food in effort to halt soaring obesity rate

BYLINE: SIMEON TEGEL

SECTION: WORLD; Pg. 38

LENGTH: 357 words

Ecuador is set to become the latest Latin American nation to crack down on junk food after President Rafael Correa promised a new tax to slim the South American nation's bulging waistlines.

Details of the levy have yet to be made public but Mr Correa's ruling alliance has a large majority in congress that invariably passes legislation with minimal changes, and the President's antipathy towards foreign fast food chains - especially from the US - is clear.

"People are dying from bad food, not a lack of food," he told local journalists. "People will stop eating so many McDonald's and Burger King hamburgers [with this tax]. This favours the production of our traditional gastronomy.

"If you want to make yourself sick, that is your problem. We are in a free country. But those who deliberately affect your health, they should contribute a little more to the healthcare system to help you once you are ill."

Latin America has some of the fastest rising obesity rates in the world, thanks to strong economic growth and a rapidly urbanising population. According to the Pan American Health Organisation, half the adults in the region are now overweight or obese, rising to three-quarters in some countries. In Ecuador, 63 per cent of people aged 19 to 59 and 30 per cent of children aged five to 11 are overweight, according to the Health Ministry.

Last year, Mexico - the world's fattest nation, after the US - slapped a tax of one peso, or around 5p, per litre on **sugary drinks.** Brazil and Chile are debating similar measures. Costa Rica, Peru and Uruguay have all banned junk food in schools. Ecuador already has a traffic lights food-labelling system, with red indicating high levels of fat, salt or sugar.

Nutritionists, though, may raise an eyebrow at Mr Correa's insistence that only food "chains" will pay the new tax. That stipulation from the leftist, famously anti-American president, who has given asylum to the WikiLeaks founder Julian Assange, would exempt the street stands and family-owned eateries that sell burgers across the country, often without the quality controls in the country's 26 McDonald's and 15 Burger King franchises.

LOAD-DATE: September 11, 2014

LANGUAGE: ENGLISH

PUBLICATION-TYPE: Newspaper

JOURNAL-CODE: IA

The INDEPENDENT

Independent.co.uk

September 11, 2014 Thursday 10:17 PM GMT

Ecuador to tax fast food in effort to halt soaring obesity rate; 'People are dying from bad food, not a lack of food' says President

BYLINE: Simeon Tegel

SECTION: FRONTPAGE

LENGTH: 357 words

Ecuador is set to become the latest Latin American nation to crack down on junk food after President Rafael Correa promised a new tax to slim the South American nation's bulging waistlines.

Details of the levy have yet to be made public but Mr Correa's ruling alliance has a large majority in congress that invariably passes legislation with minimal changes, and the President's antipathy towards foreign fast food chains - especially from the US - is clear.

"People are dying from bad food, not a lack of food," he told local journalists. "People will stop eating so many McDonald's and Burger King hamburgers [with this tax]. This favours the production of our traditional gastronomy.

"If you want to make yourself sick, that is your problem. We are in a free country. But those who deliberately affect your health, they should contribute a little more to the healthcare system to help you once you are ill."

Latin America has some of the fastest rising obesity rates in the world, thanks to strong economic growth and a rapidly urbanising population. According to the Pan American Health Organisation, half the adults in the region are now overweight or obese, rising to three-quarters in some countries. In Ecuador, 63 per cent of people aged 19 to 59 and 30 per cent of children aged five to 11 are overweight, according to the Health Ministry.

Last year, Mexico - the world's fattest nation, after the US - slapped a tax of one peso, or around 5p, per litre on **sugary drinks.** Brazil and Chile are debating similar measures. Costa Rica, Peru and Uruguay have all banned junk food in schools. Ecuador already has a traffic lights food-labelling system, with red indicating high levels of fat, salt or sugar.

Nutritionists, though, may raise an eyebrow at Mr Correa's insistence that only food "chains" will pay the new tax. That stipulation from the leftist, famously anti-American president, who has given asylum to the WikiLeaks founder Julian Assange, would exempt the street stands and family-owned eateries that sell burgers across the country, often without the quality controls in the country's 26 McDonald's and 15 Burger King franchises.

LOAD-DATE: September 12, 2014

LANGUAGE: ENGLISH

PUBLICATION-TYPE: Newspaper; Web Publication

JOURNAL-CODE: WEBI

Copyright 2014 Independent Digital News and Media Limited
All Rights Reserved

The Express

September 10, 2014 Wednesday
Edition 1;
National Edition

Fruit juice 'doubles risk of asthma'

SECTION: NEWS; Pg. 27

LENGTH: 141 words

DRINKING a couple of glasses of pure fruit juice a day could double the risk of asthma in children, a study claims.

Scientists found by the age of 11 youngsters were twice as likely to have the lung disease if they drank more than 10 glasses of 100 per cent juice a week, compared with those drinking less than four glasses a week.

Experts admitted their findings, published in the European Journal of Clinical Nutrition, were "unexpected" and called for more research.

Pure fruit juice contains large amounts of naturally occurring sugar.

Previous studies suggest very high sugar content could make airways more vulnerable to allergic inflammation.

Scientists from the Netherlands looked at how often 2,406 children consumed drinks, which included pure fruit juice, sugar-added drinks and milk, and if they had asthma. Pure fruit juice doubled the risk.

LOAD-DATE: September 10, 2014

LANGUAGE: ENGLISH

PUBLICATION-TYPE: Newspaper

JOURNAL-CODE: DXP

Copyright 2014 Express Newspapers
All Rights Reserved

171 of 249 DOCUMENTS

Financial Times (London, England)

September 10, 2014 Wednesday
London Edition 1

A Big Mac, Coke and side order of decline

BYLINE: Luke Johnson

SECTION: THE ENTREPRENEUR; Pg. 14

LENGTH: 744 words

Have many big consumer brands peaked? I ask this question because I believe a number of the world's biggest products appear to be in structural decline.

Companies such as Interbrand and Brand Finance value brands. But two of their top global brands, Coca-Cola and McDonald's, are facing what may be existential threats. Both are in pronounced decline in their home country, and it appears these slumps are not temporary. Coca-Cola sales have been falling for a decade in the US, according to Euromonitor - supplanted by everything from iced tea and sports drinks to coffee and fruit juices. **Sugary,** carbonated **beverages** are seen by the younger generation as unhealthy. Once such trends become established they are difficult to reverse. And **fizzy drinks** still make up three-quarters of the company's overall sales.

Meanwhile McDonald's restaurants are losing their appeal among millennials , according to recent research. More than 40 per cent of the company's 35,000 restaurants are in the US - but younger customers are visiting McDonald's less, and going instead to rival fast-casual chains. Diners want fresher, healthier food and a more bespoke offering. And what happens in America is likely to be followed in other territories.

It may be that the health issues challenging Coca-Cola and McDonald's are specific to those market leaders. But I suspect other factors are at play too. Customers younger than 30 are more promiscuous in their brand choices and have less product loyalty. The digital revolution has brought almost total price transparency, diminishing the ability of brands to charge more. Retailers are embracing own-label to defend their margins.

Greater variety and the ability to browse from a wider range of goods online mean younger customers are less tolerant of an overwhelming dominance of a few big businesses. Just as media consumption has fragmented considerably - the old TV and radio networks, newspapers, magazines and suchlike gradually losing their power - so 20- and 30- somethings are more inclined to buy from a range of sources than rely on a few vendors. In both alcoholic and soft drinks, for example, almost all the growth is coming from smaller, independent producers.

The difficulty for big corporations such as McDonald's and Coca-Cola is that their core products are vastly profitable. Replacing these enormous cash flows is an almost impossible task. Large organisations find it extremely hard to innovate despite investing large sums in research and development because the strategic emphasis at the board and among shareholders will always remain focused on the bulk of the profits - the traditional, ageing product lines.

For both these companies their business model is essentially that of pure intellectual property owners. This means they need invest only modest amounts in capital expenditure. They license most of the hard work to partners. Coca-Cola relies on its bottlers to produce and sell the actual product; McDonald's depends increasingly upon franchisees to operate its restaurants. That system is fine when the strength of the IP and marketing are undoubted; but when the brands lose their relevance, then they may not be able to charge such high fees and mark-ups to their partners. Burger King has struggled considerably in its franchisee relationships over the years, and is now buying Tim Hortons .

And that is usually the answer from large companies which feel they cannot grow - they make acquisitions. Frequently these fail because the cultures clash. Ironically McDonald's made two excellent purchases of minority stakes years ago - Chipotle and Pret A Manger - but then sold both off. Each has become steadily more successful and is part of the growing band of competitors to McDonald's.

Meanwhile Coca-Cola has a division meant to cultivate and buy drink start-ups with potential. Let's hope they do better than they did with Malvern Water, near where I have a home. Schweppes had bottled water there for more than 150 years: but Coca-Cola felt the operation was too small so shut the plant down in 2010 and sold it off for development. They did not sell the brand to any local entrepreneurs to revive - the sort of behaviour that gets big business a bad name.

All of these are reasons why I prefer smaller, dynamic concerns to the behemoths.

lukej@riskcapitalpartners.co.uk Twitter: @LukeJohnsonRCP The writer is chairman of Risk Capital Partners, a private equity firm, and The Centre for Entrepreneurs

LOAD-DATE: September 9, 2014

LANGUAGE: ENGLISH

PUBLICATION-TYPE: Newspaper

Copyright 2014 The Financial Times Ltd.
All Rights Reserved
Please do not cut and paste FT articles and redistribute by email or post to the web.

Daily Record & Sunday Mail

September 8, 2014 Monday
Edition 1;
National Edition

Scots' sugar fix blamed on retailers; Government urged to take sweet revenge to curb the obesity crisis

BYLINE: Heather Greenaway

SECTION: FEATURES; Pg. 30

LENGTH: 346 words

SCOTLAND'S sweet tooth is getting sweeter, thanks to supermarkets offering deals on chocolate, sweets and **fizzy drinks**.

A new study has revealed Scots families are in the grip of a powerful sugar addiction, fuelled by in-store price cuts.

The research, carried out by Scotland's Rural College (SRUC), analysed the shopping habits of more than 3000 Scottish households between 2006 and 2011.

Sales of bargain confectionery have rocketed by almost 10 per cent, from 33.5 per cent in 2006 to 43 per cent in 2011, while deals on **fizzy drinks** drove sales up from 47 per cent to 54 per cent .

Researchers put the steep rise in the purchase of sugary treats entirely down to supermarkets' temporary price cuts, as sales did not rise between 2008 and 2011 when there were no promotions.

The study also noted stores in Scotland had increased their use of deals as a way of boosting sales during the recession when customers could not afford luxuries.

Dr Cesar Revoredo-Giha from the SRUC, who carried out the research, believes the results have important implications for public health and supermarkets should be forced to curb the trend.

He said: "Policies tending to increase the retailers' awareness or forbidding the use of such marketing tools by retailers for specific products due to their implication for health are certainly justified."

Developed countries are facing an obesity '' epidemic, which is increasingly affecting children. Scotland has one of the worst records of the 34 industrialised countries belonging to the Organisation of Economic co-operation and Development, with 68 per cent of men and 62 per cent of women overweight or obese.

Studies also suggest that more than 15 per cent of boys and 13 per cent of girls under the age of 16 are obese and 30 per cent of children are overweight.

In Scotland, ministers have said they will "carefully consider" recommendations put forward to the UK Government that an adult's entire daily sugar ration should be limited to the equivalent of one **fizzy drink** - that's one can of Irn-Bru.

An entire daily sugar ration 'is one Irn-Bru'

LOAD-DATE: September 8, 2014

LANGUAGE: ENGLISH

GRAPHIC: ADDICTED Sales of sweets have soared

PUBLICATION-TYPE: Newspaper

JOURNAL-CODE: DRC

Copyright 2014 Scottish Daily Record & Sunday Mail Ltd
All Rights Reserved
The Sunday Times (London)

September 7, 2014 Sunday
Edition 1;
Scotland

Supermarket deals 'fuel sugar habit'

BYLINE: Mark Macaskill

SECTION: NEWS; Pg. 15

LENGTH: 531 words

FAMILIES in Scotland are in the grip of a powerful sugar addiction driven by supermarket deals on sweets, crisps and **fizzy drinks**, a study warns.

Scientists found that households with children had "significantly" increased their purchases of sugary products in recent years, fuelled by in-store price cuts.

One theory is that the big supermarkets used price cuts to shift more goods during the recession, when household budgets were tight.

It is well known that many of us eat too much sugar - which is helping to drive up obesity - but the findings lay bare the full extent of the nation's sweet tooth.

The study, carried out by Scotland's Rural College (SRUC), analysed data on the shopping habits of more than 3,000 households between 2006 and 2011.

It included the price paid for products, whether they were part of a promotion, and how much was bought. It emerged that, over the five years, an increasing proportion of sales of sugary snacks came from cut-price offers.

Bargain confectionery sales rocketed by almost 10 percentage points, from 33.5% in 2006 to 43% in 2011, while deals on frozen products drove sales up from 44.5% to 49%, and for **fizzy drinks** from 47% to 54%. Researchers concluded that increased consumption of take-home confectionery and soft drinks in 2011 was fuelled entirely by supermarkets' temporary price cuts.

Sales of sugary products between 2008 and 2011 did not rise when there were no promotions.

"This finding highlights the importance of promotions in stimulation [of] the purchases of sugary products," said the researchers. It was also noted that supermarkets in Scotland had increased their use of promotions, "reinforcing the idea that retailers have been using promotions to keep the expenditure growing during the recession times".

The study, by Dr Cesar Revoredo-Giha from the SRUC and presented last month at the European Association of Agricultural Economists congress in Ljubljana, Slovenia, concluded: "Overall, the results indicate that young households and families with children have increased substantively their purchases of sugary products, helped by different types of retail promotions."

Revoredo-Giha goes further by suggesting the results "have important implications for public health".

He argues that evidence that retailers' promotions encourage hoseholds to buy more sugary products could be used to curb the trend. "Policies tending to increase the retailers' awareness or forbidding the use of such marketing tools by retailers for specific products due to implication for health are certainly justified."

Developed countries are facing an obesity epidemic, which is increasingly affecting children. Scotland has one of the worst records of the 34 industrialised countries belonging to the Organisation of Economic Co-operation and Development, with 68% of males and 62% of females overweight or obese. Studies suggest that more than 15% of boys and 13% of girls under the age of 16 are obese and 30% of children are overweight.

In Scotland, ministers have said they will "carefully consider" recommendations put forward to the UK government that an adult's entire daily sugar ration should be limited to the equivalent of one **fizzy drink**.

LOAD-DATE: September 7, 2014

LANGUAGE: ENGLISH

GRAPHIC: The obesity epidemic sweeping Scotland is increasingly affecting children

PUBLICATION-TYPE: Newspaper

JOURNAL-CODE: STSscot

Copyright 2014 Times Newspapers Limited
All Rights Reserved

Daily Star

September 1, 2014 Monday
Edition 1;
National Edition

FAT KIDS OUT OF CONTROL; Docs call for obesity squads

BYLINE: ED RILEY

SECTION: NEWS; Pg. 20

LENGTH: 260 words

DOCTORS are calling for a task-force to crackdown on the rise of childhood obesity.

GP's want to form a team similar to the government's Cobra panel which deals with responding to terror threats.

They say the UK has reached a "state of emergency" with children aged just seven developing diabetes caused by junk food and **sugary drinks**.

Medics warn an entire generation will be "destroyed" unless action is taken and have penned a letter to chief medical officer Professor Dame Sally Davies.

They say the NHS will be overwhelmed without urgent action to cut the number of youngsters "hunched over computer consoles and gorging on junk food".

Shock figures show a third of Brit kids are overweight or obese.

The Royal College of GPs and 11 other organisations want a Child Obesity Action Group to be set up immediately. Dr Rachel Pryke, RCGP clinical lead for nutrition, said: "The stark fact is that overweight children are being set up for a lifetime of sickness and health problems.

"We are in danger of destroying the health of a whole generation of children.

"As parents and health professionals, we need to take responsibility and ensure that every child has a healthy and varied diet and regular exercise."

Docs want more training in malnutrition and projects to educate families about the dangers of being overweight.

Dr Richard Roope, RCGP clinical lead for cancer, called for a tax on **sugary drinks**.

He added: "GPs aren't killjoys, we want all our patients to have healthy and fulfilling lives, but this crisis is happening and it's real."

ed.riley@dailystar.co.uk

LOAD-DATE: September 1, 2014

LANGUAGE: ENGLISH

PUBLICATION-TYPE: Newspaper

JOURNAL-CODE: DST

Copyright 2014 Express Newspapers
All Rights Reserved

i-Independent Print Ltd

September 1, 2014
First Edition

GPs demand task force to tackle childhood obesity; SOCIETY

BYLINE: David Mercer

SECTION: NEWS; Pg. 6

LENGTH: 170 words

Doctors have called for an emergency task force to be set up to tackle childhood obesity. The professional body representing GPs has warned that an entire generation will be "destroyed" by a diet of junk food and **sugary drinks** unless urgent action is taken.

In an open letter to the Chief Medical Officer for England, Professor Dame Sally Davies, the Royal College of General Practitioners (RCGP) and 11 partner organisations said a national Child Obesity Action Group should be formed as "a matter of urgency".

The task force would tackle "the rising epidemic of childhood obesity", a RCGP spokesman said. Doctors, nurses, midwives, dieticians, dentists and schools would collaborate to try to prevent obesity and improve treatment services.

Dr Rachel Pryke, RCGP clinical lead for nutrition said: "We are in danger of destroying the health of a whole generation of children.As parents and health professionals, we need to take responsibility and ensure that every child has a healthy and varied diet and regular exercise."

LOAD-DATE: August 31, 2014

LANGUAGE: ENGLISH

PUBLICATION-TYPE: Newspaper

JOURNAL-CODE: III

MailOnline

MailOnline

October 31, 2014 Friday 7:44 PM GMT

So is this new 'Green' Coke all it's cracked to be? Critics warn low-sugar Coca-Cola Life is simply a marketing gimmick

BYLINE: MADLEN DAVIES FOR MAILONLINE

SECTION: HEALTH

LENGTH: 867 words

- Coca-Cola launched Coke Life this year in U.K., U.S., Chile and Argentina
- Sold in a green can, its also set to hit Australian shelves in April next year
- It has a third less sugar than regular Coke and 89 calories compared to 139
- Sweetened with extract of stevia leaf, a plant native to South America
- Stevia leaf extract is 200 - 300 times sweeter than table sugar
- Already used in Sprite products in the UK, but critics say has an aftertaste
- Experts voiced concerns about the marketing of the products as healthy
- Warn it still contains colouring, caffeine and phosphoric acid
- Also still contains 19 per cent of our recommended daily sugar intake

It's the latest member of the Coca-Cola family designed to ease the consumer conscience.

Called Coca-Cola Life, it's marketed as having less sugar than regular Coke but none of those artificial sweeteners that increasingly plague Diet Coke drinkers.

The new naturally sweetened drink - which contains a third less sugar and calories than regular cola - has been created amid calls for the company to do more to tackle the global obesity epidemic.

Sold in a green can or a recyclable bottle, it is already available in the UK, U.S., Chile, Argentina and is due to hit Australian shelves in April.

But critics say the product - marketed as 'healthy' - may do more to improve the company's finances than the health of its consumers.

Scroll down for video

Coke Life is sweetened with a blend of sugar and stevia leaf extract - a calorie-free sweetener which is 200-300 times sweeter than table sugar.

A plant used for centuries by Paraguay's Guarani Indians, it has shot from relative obscurity to being used as a key sweetener by large companies such as Coca-Cola and Danone in just a few years.

Currently, Coca-Cola Great Britain uses stevia leaf extract as a sweetener in its Sprite and Glaceau vitamin water brands, though critics have complained it has a 'bitter aftertaste', similar to liquorice.

A 330ml can of Coke Life contains 89 calories, compared to 139 calories in a regular can of Coca- Cola.

Coca-Cola's other products, Coke Zero and Diet Coke - sweetened with the artificial sweetener aspartame - contain around one calorie each.

But the company is facing a growing backlash against such artificial sweeteners such as aspartame and an increased demand for natural products.

The solution? Bring out Coke Life for the slightly more health conscious consumers out there known as 'balance-seekers'.

Coke's UK marketing director, Bobby Brittain, has said: 'We know exactly who our Coke Life will appeal to.

'It's 20 and 30-somethings who have begun to realise they're not completely immortal and that they do have a sense of responsibility about what they consume.'

Some health experts are sceptical about Coca-Cola's motives and worry about the health implications of a **fizzy drink** marketed in 'healthy' green packaging.

Writing for The Conversation, Professor Sandra Jones, director of the Centre for Health and Social Research at Australian Catholic University, said: 'While stevia is safe to consume, nutritionists have noted that simply removing some sugar and replacing it with stevia doesn't make a drink (or food) healthy.

'Coca-Cola Life still contains colouring, caffeine, phosphoric acid and 19 per cent of our recommended daily sugar intake.'

'In fact, a cola drink with a few less calories may be part of the problem rather than the answer to reducing our waistlines. People tend to consume greater quantities of foods they believe to be healthy, and seeing a food promoted as healthy can lead people to eat more calories.'

The colour green is associated with healthiness, and so there may be a subtle reason why Coca- Cola decided to stray away from its iconic red-labelling with this product.

Professor Jones said: 'A recent European study found people drink less soft drink from a red-labelled cup than a blue-labelled cup. At a subconscious level, the colour red operates as a stop signal.'

When it was launched, Coca-Cola said that the new drink was part of its commitments to offer consumers reduced, low and no-calorie options - adding that it was the 'most recent example in a series of initiatives by the company to inspire happier, healthier lives'.

The company is a signatory to the Government's Responsibility Deal, under which the food and drink industry has pledged to promote and healthier diet and make changes to their products.

Coca-Cola has committed to reduce the average calories per litre in its range of sparkling drinks by 5 per cent by the end of 2014.

But Professor Jones is sceptical about whether introducing Coke Life is consistent with the promise to promote healthier lifestyles.

She said: 'Ultimately, many of these decisions are likely being made to increase the health of the companies' profits rather than the health of their customers.'

A look at The Coca Cola Company's 2013 financial results shows profits from regular soft drinks are declining and sales of "healthier" options are increasing, she added.

She continued: 'Perhaps the most genuine option for this and other such companies would be to cut back on the production and marketing of sweetened soft drinks and focus on selling products that are actually good for consumers.

LOAD-DATE: October 31, 2014

LANGUAGE: ENGLISH

PUBLICATION-TYPE: Web Publication

JOURNAL-CODE: WEBDM

Copyright 2014 Associated Newspapers Ltd.
All Rights Reserved

MailOnline

October 31, 2014 Friday 3:49 PM GMT

Did YOU know one glass of apple juice has around SEVEN teaspoons of sugar in it... and a 'healthy' fat-free yogurt contains five?

BYLINE: MADLEN DAVIES FOR MAILONLINE

SECTION: HEALTH

LENGTH: 1070 words

- Glass of apple juice contains as much sugar as three Krispy Kreme donuts
- A fat-free yogurt contains as much as three scoops of ice cream
- World Health Organisation recommends no more than five teaspoons a day
- Survey reveals 90% of mothers are unaware of these high levels of sugar
- A fifth confess their child doesn't eat a balanced diet

Most of us have no idea a glass of seemingly healthy apple juice contains almost as much sugar as three Krispy Kreme donuts or a bar of Dairy Milk, a survey has found.

With almost seven teaspoons of sugar in a single serving, apple juice contains more than the recommended daily amount for adults who want to stay healthy.

According to the World Health Organisation, this should be six teaspoons of 'added' sugar - i.e. not that found naturally in products such as milk.

Scroll down for video

Similarly, a fat-free yogurt - a food also often marketed as a natural, healthy choice - contains five teaspoons of sugar - the same as in three scoops of ice cream.

The high levels of sugar found in everyday - often so-called 'healthy items' - were revealed as part of a survey by Bupa.

This asked 2,000 mothers if they were aware of the amount of sugar found in foods they may be giving their children.

In the poll, more than 90 per cent admitted they had no idea about the high levels of sugar in apple juice and fat-free yogurt.

Bupa states the average glass of concentrated apple juice contains nearly seven teaspoons on sugar.

One Krispy Kreme Original glazed donut, on the other hand, contains 10g of sugar - which equates to two teaspoons.

Nearly half of mothers (46 per cent) admitted they were worried their child might be addicted to sugar.

But despite their concerns, a fifth confessed their child does not eat a balanced diet.

And 60 per cent admitted they do not regularly look at the nutritional information on food.

Too much sugar in a child's diet can cause permanent damage to their health, including increased risk of heart disease and Type 2 diabetes. It can also cause tooth decay.

Despite this, 40 per cent of mothers said their children have sweets, **fizzy drinks** or chocolate at least once a day.

SO HOW MUCH SUGAR SHOULD WE BE EATING?

Earlier this year, the World Health Organisation published draft guidelines urging adults to eat no more than 12 teaspoons of sugar a day and to aim for six.

The guideline amount slashed was amid fears sugar poses same threat as tobacco.

The number of obese British adults is expected to double from one in four to one in two by 2050 - at a cost to the economy of £50billion a year.

The WHO said the crisis was being fuelled by hidden sugar in processed food and drink such as yogurts, muesli, sauces, **fizzy drinks**, juice and smoothies.

However, low-fat foods have also come under fire from experts, after it was revealed they often contain more sugar than the full-fat alternative.

WHO also said children should try for less than six teaspoons and avoid cans of **fizzy drink** such as Coke, which contains seven spoons.

Instead five or six teaspoons is the ideal figure that people should aim for.

Doctors say keeping to a maximum of six teaspoons of sugar a day is key to avoiding obesity, heart disease and other serious illnesses because they fear sugar is as dangerous as tobacco.

Chief medical officer Sally Davies has already said a tax may be put on calorie-laden food and drink to curb soaring levels of obesity.

Until recently, it was thought that 'bad drinks' were those such as Coke and Pepsi, and fruit juice was a healthy alternative helping us get our 'five a day'.

But increasingly experts are warning that fruit juices and are fuelling the obesity epidemic.

Low-fat foods have also come under fire from experts, after it was revealed they often contain more sugar than the full-fat alternative.

Earlier this year a study found manufacturers are making their 'healthy' options more palatable by replacing fat with sugar.

While most low-fat supermarket products contain a third fewer calories than their regular fat version, 10 per cent actually have more or the same calories, mainly due to added sugars.

Obesity specialist Dr Matthew Capehorn said weight-conscious shoppers should realise that choosing low-fat products made by brands including Weight Watchers could hamper their efforts to cut calories.

Nearly a quarter of mothers said they like putting sugary treats in their child's lunchbox, with the top culprits being cakes, chocolate and fruit juice.

On top of this, nearly a third of mothers think that as they give their children healthy food, sugar isn't an issue.

The news comes as NICE announce that schools and nurseries should run tooth brushing schemes to improve children's teeth.

As many as one in eight children now suffer tooth decay by the age of three - although in some parts of England the rates are as high as a third.

SUGAR PUFFS TO BE RENAMED 'HONEY MONSTER PUFFS' BECAUSE PARENTS ARE SO PARANOID ABOUT SUGAR

Sugar Puffs are being given a makeover in an attempt to address parents' concerns about the amount of sugar in the cereal - and revive plunging sales.

Rebranded as 'Honey Monster Puffs' after the yellow furry character featured in previous adverts, they will be made from a new recipe with less sugar and 20 per cent more honey.

The cereal will also feature traffic light nutritional labelling on the front of the pack, in a move manufacturers Halo Foods said would allow customers to make 'informed decisions' about what they eat for breakfast.

Overall, manufacturers say the sugar content has been reduced by a third in a decade.

Honey Monster Puffs will contain 8.6g of sugar - a cube and a half - in a 30g portion, down from the previous recipe which contained almost two cubes per portion.

However critics say the move is largely a marketing ploy - as once broken down, honey and added sugar become the same thing - glucose.

Dietitian Helen Bond told MailOnline: 'Added sugar and honey are grouped in the same category by the UK Scientific Advisory Committee on Nutrition.

'Although honey is seen as more "natural" than added sugar, it is still counted as a "free sugar" - i.e. one that has been added to a product rather than say lactose, a sugar found naturally in milk.

'Once broken down in the body, honey will do exactly the same thing as the added sugar would have.'

LOAD-DATE: October 31, 2014

LANGUAGE: ENGLISH

PUBLICATION-TYPE: Web Publication

JOURNAL-CODE: WEBDM

Copyright 2014 Associated Newspapers Ltd.
All Rights Reserved

The Telegraph

telegraph.co.uk

October 30, 2014 Thursday 4:48 PM GMT

Is your child addicted to sugar?; Nearly half of mothers believe their children are addicted to sugar - but most are unaware quite how much they're really consuming

BYLINE: By Olivia Walmsley

LENGTH: 719 words

Nearly half of mothers (46 per cent) believe their children may be addicted to sugar, according to a new poll conducted by Bupa.

Yet many of them were unaware of the true scale of the problem, with the majority uninformed about the high sugar content of everyday foods such as yoghurts and juice.

Even though 43 per cent of the 2,000 mothers questioned claimed to know how much sugar was in the food their children eat, when asked how much was contained in everyday lunchbox items, most had no real idea.

While 88 per cent of children have at least one yoghurt a day, 93 per cent of the mothers questioned weren't aware that a typical fat-free yoghurt contains five teaspoons of sugar

The research found that 77 per cent of children consumed at least one glass of fruit juice each day - but 94 per cent of mothers didn't know that the average amount of sugar in a typical glass of apple juice is 6.5 teaspoons.

Some 60 per cent admitted that they fail to regularly check the nutritional information on food products for their children.

According to Mumsnet editor Sarah Crowne, sugar consumption is a continual source of anxiety among parents: "There's a great deal of discussion on Mumsnet about how to feed your kids well - from coping with fussy toddlers to tips for cajoling refusenik teens into managing a sensible breakfast before school.

"Excessive sugar consumption is a perennial concern amongst our users - particularly with 'hidden' sugar popping up in everyday products such as pasta sauce and bread, not to mention the large quantities in some of the most popular children's yoghurts and fruit snacks.

"In an ideal world, obviously, we'd all shun jars and packets and cook perfectly-balanced meals from scratch every day - but the fact is that time and money constraints often make this impossible.

"As with most aspects of child-rearing, parents simply do their best: to be clued-up about nutrition, avoid the obvious sugar-laden pitfalls and try to strike a healthy balance when feeding their kids."

Yet, in spite of these concerns and efforts, 40 per cent of parents questioned in the new poll said they gave their children sweets, **fizzy drinks** or chocolate at least once a day.

Nearly 25 per cent surveyed said they placed sugary treats in their child's lunchbox, with the top culprits being cakes, chocolate, and fruit juice. Nearly a third of mothers believed that, as they gave their children healthy food, too much sugar wasn't an issue.

Nicole Mowbray, author of Sweet Nothing, an account of giving up sugar, says: 'While these figures are shocking, they're not surprising."

"Even though parents try their best, sugar lurks in so many of the foods we give our children, from the addition of fruit juice to savoury baby foods to make it more palatable, to the high quantities of sugar lurking in everyday sauces such as tomato ketchup, or this new idea that honey is in some way healthier, when it isn't.

"That's before you've even considered the amount of sugar given to youngsters in the form of 'treats'. Parents should use their pressure power to force manufacturers to start reducing the amount of sugars they add to food and drinks aimed at children.'

Too much sugar in a child's diet can cause permanent damage to health, including increased risk of heart disease and Type 2 diabetes. as well as severe damage to dental health.

Andrew Wallis, of Cornwall Council, said earlier in the year that tooth decay among children had reached epidemic proportions. In fact, the research was commissioned by Bupa to mark the launch of its Tooth Fairy campaign - parents can create a personalised video for their children at toothfairy.bupa.co.uk.

Bupa's Clinical Director of Dentistry, Dr Steve Preddy commented: "With 26,000 primary school children admitted to hospital for tooth decay in the past year, there is a need now more than ever, for parents to be paying attention to their child's sugar consumption.

"The recent announcement from NICE has caused much debate amongst health and teaching authorities, however the most important thing to remember is tooth decay is preventable.

Parents need to be regularly looking at the nutritional information of food products; it is often what are thought of as healthy foods or unexpected ones."

Do you find it hard to limit your child's sugar intake?

LOAD-DATE: November 5, 2014

LANGUAGE: ENGLISH

PUBLICATION-TYPE: Newspaper; Web Publication

JOURNAL-CODE: WEBDTNS

Copyright 2014 Telegraph Media Group Limited
All Rights Reserved

MailOnline

October 29, 2014 Wednesday 10:31 PM GMT

Better than Coke Zero and close to the real thing - but not quite as good for the waistline: An exclusive first taste of 'Coke Life'

BYLINE: LEESA SMITH FOR DAILY MAIL AUSTRALIA

SECTION: NEWS

LENGTH: 662 words

- Coca Cole Life is being launched in Australia in April next year
- The gleaming green can has 35 per cent less sugar than Classic coke
- Natural sweetener, Stevia, is used to replace the sugar
- Daily Mail Australia was given an exclusive taste of the new product

When I was asked to try the new product from Coca-Cola - I knew I would be instantly transported back down nostalgia lane to my childhood.

Daily Mail Australia was offered an exclusive taste test of the soft drink giant's new beverage for the slightly more health conscious consumers out there known as 'balance-seekers'.

A can of Coca-Cola Life boasts 35 per cent less sugar thanks to the introduction of the natural sweetener Stevia - meaning it only contains 290 calories compared to the classic can's 450 calories.

Scroll down for video

'Coke Life is absolutely about not only keeping pace with consumer needs and demands but hopefully trying to out-pace them,' Coca-Cola Group Marketing Manager Dianne Everett said.

The gleaming green can almost looks like an impersonator next to the traditional colours of the red, white, black and silver branding across the three others kinds of Coke.

Coca-Cola Group Marketing Manager Dianne Everett said the colour was chosen to stand out above its carbonated competitors.

'It's really about making sure we stand out on shelf with something that's vibrant so that we are clearly positioning ourself as yet another vibrant addition to the Coke family - and it also compliments the rest of the family,' she said.

Trying the new Coke was a serious blast from the past for me as it was designed to taste like the classic product which I haven't had for probably for the best part of three decades.

Never having been much of a soft drink fan, I compared the can of Life to the Zero product which is marketed towards men who aren't enticed by Diet version.

As subtle as it may be - I felt that Coke Life tasted more like the real deal than Zero - which has no sugar but contains the controversial artificial sweetener Aspartame - as does Diet Coke.

Just before tasting Coke Life, I wrote a story on buses needing to increase the load limit by two tonnes to cater for the fact that Australian's waistlines have expanded almost 20 per cent between 1989 and 2012.

Aloysa Hourigan from Nutrition Australia mentioned to Daily Mail Australia that although the statistics were not surprising - she hoped that a downward trend of junk food consumption in America would be picked up Down Under.

'There are little glimmers of hope around such as in the U.S where the consumption of McDonald's and Coca Cola has been decreasing and their sales are dropping,' she said.

'Perhaps it will be a bit of a trend that might reflect back eventually on weight loss but it takes time to see those changes.'

Ms Everett expressed that the multi-million dollar international company had never marketed its products as a healthy range.

'We don't attempt to market Coca Cola or any of our products - and certainly not even our latest in the line with Coke Life - as a health drink,' she said.

'We are under no illusions we are operating in that space.'

'We are a fun great tasting drink that people consume on occasions that are relevant to them.'

'We truly believe obesity is a very complex issue and the causes of which are multi-factorial and our responsibility in this current environment is we want to continue to provide range and choice,' Ms Everett said.

'Consumers ultimately decide the kind of portions, sizes and flavours that they are seeking which is all part of what we think we need to do to have that responsible social footprint.'

Set to be launched in New Zealand and Australia in April next year, Coca Cola Life has already been launched in America, the U.K. and Argentina - where the product actually has an astounding 60 per cent less sugar to cater to that South American particular market.

Whether the die-hard Aussie Coke fans will embrace the new Coca Cola Life remains to be green.... I mean seen.

LOAD-DATE: October 31, 2014

LANGUAGE: ENGLISH

PUBLICATION-TYPE: Web Publication

JOURNAL-CODE: WEBDM

Copyright 2014 Associated Newspapers Ltd.
All Rights Reserved

MailOnline

MailOnline

October 29, 2014 Wednesday 5:49 PM GMT

MailOnline

October 28, 2014 Tuesday 4:41 AM GMT

Why does Australia need another Coke? Drinks giant's new green 'natural' drink cuts calories by 60 per cent compared to the classic red variety... and doesn't have the chemicals of Diet and Zero

BYLINE: SARAH DEAN FOR DAILY MAIL AUSTRALIA

SECTION: NEWS

LENGTH: 901 words

- Coca-Cola Life comes in a green can and has 27 calories
- It has 60 per cent less calories than classic Coke
- Unlike Diet Coke and Coke Zero it still contains sugar
- But it uses the plant Stevia to sweeten it as well, instead of the man-made sweetener Aspartame used in Diet Coke
- Nutrition Australia says the drink still encourages people to want sweet things and is still likely to cause dental erosion
- Coca-Cola are expected to launch the product in Australia

Coca-Cola is expected to launch a new green product in Australia, made from a naturally sweet plant, in a bid to target health conscious soft drink lovers.

The new carbonated beverage provides a lower-calorie soft drink than Coca-Cola's traditional red can and has more natural ingredients than Diet Coke.

But unlike Diet Coke, which is completely sugar free, Coke Life still has sugar.

The new addition to Coke's range of 3,500 beverages, should also not be mistaken for Coke Zero, which is a low-calorie drink that is marketed at men who don't like the word 'diet' and is meant to taste exactly the same as classic Coca-Cola.

Scroll down for video

Coke Life is roughly inbetween Diet Coke and classic Coca-Cola when it comes to calories - you will still find 27 in a can.

However, it has 60 per cent less sugar than classic Coke because it uses the natural plant Stevia and sugar as sweeteners, rather than just sugar.

Nutritionist Aloysa Hourigan from Nutrition Australia said that Stevia is not known to have any health risks but that doesn't necessarily mean it is healthy to drink Coke Life.

'Nutrition Australia would still say that while it's safe it's still encouraging people to want to eat sweets.

'The problem with all these artificial drinks is they still have high acidity levels and dental erosion.

IT'S FOUND IN DIET COKE - BUT WHAT IS ASPARTAME?

Aspartame is a man-made sweetener used as a **sugar** substitute in some foods and **beverages**.

The safety of aspartame has been the subject of several political and medical controversies.

However, the European Food Safety Authority concluded in its 2013 re-evaluation that aspartame and its breakdown products are safe for human consumption at current levels of exposure.

People with the genetic condition phenylketonuria are however told to avoid it.

'The other thing is there is some research that suggests that cola based colour drinks, whether sweetened naturally or artificially, are potentially impact negatively on insulin resistance meaning you are more likely to gain weight,' she told Daily Mail Australia.

'It is not proven or definite but there is some concern around it. No matter what, the best drink is water and that's we'd still agree.'

If you're wondering what the need for another low-calorie Coke drink is, the global brand which is worth an estimated $74 billion US dollars reportedly wanted a product for the growing number of people who are worried about about artificial sweeteners like Aspartame, one of the most intensively scrutinised food additives.

Aspartame is ingested every day by millions of people around the world in more than 6,000 well-known brands of food, drink and medicine. However, it has been the subject of a number of studies that appear to show harmful effects on human health.

One study linked diet drinks containing Aspartame to premature births, while another suggested it could cause cancer.

However, in December 2013, the European Food Safety Authority (EFSA) reviewed aspartame and confirmed it as safe, so Coca-Cola have continued to use it.

WHAT IS THE SWEETENER STEVIA?

Stevia is the name of the plant from which stevia sweetener (steviol glycoside) is obtained.

You can buy stevia in a powdered form as a **sugar** substitute that you can add to food and **beverages** at home - or you may find it in icecream, water-based beverages, brewed **soft drinks**, plain and flavoured soy beverages.

It can be used in cooking and baking according to the Stevia Australia website, excepting that it does not caramelise like sugar.

The INDEPENDENT

Independent.co.uk

October 27, 2014 Monday 5:37 PM GMT

Why the UK's official healthy food guide needs changing after 20 years; Health concerns have changed significantly - not to mention other food issues such as environmental sustainability, animal welfare and Fair Trade

BYLINE: Mike Rayner, Kremlin Wickramasinghe

SECTION: FEATURES

LENGTH: 865 words

The Eatwell plate is the UK government's official food guide about which foods we should eat to achieve a healthy diet. It is essentially a pie-chart depicting the recommended intakes of five specified food groups: fruit and vegetables, dairy products, cereals, meat and processed foods. It was first published 20 years ago - and despite some two decades of nutritional research has not been changed since.

In some countries - notably Australia, the US and Brazil - the official food guide is revised on a regular basis. Some two decades since it was first published, Public Health England has announced that it will revise the Eatwell plate in the light of proposed new recommendations on sugar from the government's Scientific Advisory Committee on Nutrition.

Then and now

The types of food we eat and the challenges for a healthy diet have changed significantly over the past 20 years. In particular we now know that added **sugar** is much more harmful to our health than we thought back then. We are now much less convinced that fruit juice - consumption of which is 15 times that in the 1970s - is much healthier than **sugary soft drinks.** And the need to cut down on red meat in our diets has become clearer, as has our need to reduce saturated fat intake rather than total fat intake.

Many people have rightly argued that the Eatwell plate is now out of date and some recent government publications, such as the new standards for school food published earlier this year, have not referred to it at all. Now is the opportunity to undertake a fully comprehensive review and here are some issues for Public Health England to consider.

What to take account ofWe need to redefine the food groups on the Eatwell plate. As Susan Jebb, the government's adviser on obesity, also agrees: fruit juice should not be included in the fruit and vegetable group (and potatoes should be).Healthier and less healthy foods within food groups should be identified. At the moment the cereals group includes salty and sugary breakfast cereals. We think that the guide should make it clear that, while current levels of cereal intake should be maintained, there are some cereals that are healthier than others.The angles of the segments of the plate - showing how much of the five food groups we should eat - also needs to change. Over the last 20 years a reduction in red meat consumption has been recommended by most public health experts. But the guide doesn't reflect this. The angle for the meat group needs to be made smaller.A perennial and growing criticism of the guide is that - unlike its US equivalent - it gives a place to unhealthy processed foods and even depicts a can of cola. Cola (or other **sugary drinks)** should be replaced with a glass of water.Some of the foods shown in the old guide have a greater impact on the environment than others. In particular, most of the foods in the meat group have higher associated greenhouse gas emissions than foods in other groups. Then there are less obvious issues such as air miles. Asparagus is shown in the guide but asparagus air freighted to the UK from South America, for example, will have a higher environmental impact than British carrots. One idea might be to design the plate in such a way that more sustainable food items can be found closer to the centre and unsustainable food items closer to the outer layer of the plate. So as you go from centre to outer circle within a group, the environmental burden of the food item increases.

Health news: in pictures

Today we are not just concerned about our diets for health reasons; there are various other considerations that shape our choices, such as environmental sustainability in food production, animal welfare and Fair Trade - and the Eatwell plate needs to take account of these wider concerns. The guide already takes account of practicalities, for example it sensibly only shows foods that are readily available. But we now know that the ways in which we consume our food not only affects our health but that of the planet and therefore the health of future generations. It is unforgivable to ignore this fact when providing dietary advice.

All of these things need to be taken into account when revising things such as the angles of the segments and how and where in the guide foods are depicted. The World Wildlife Fund has produced its own version of the Eatwell plate called the Livewell plate, which shows how this can be done.

Much has been discovered and published during the past 20 years which has changed our understanding of food. Revision of the Eatwell plate - merely to take account of one government report - seems illogical. This

rare opportunity should be used to develop a systematic and transparent approach to reviewing current evidence and to undertake a comprehensive review of the Eatwell plate to reflect all of the dietary challenges we face today.

Read more:The effects of too much fast foodWhat alcohol does to your body

By Kremlin Wickramasinghe, University of Oxford and Mike Rayner, University of Oxford

This article was originally published on The Conversation. Read the original article.

LOAD-DATE: October 27, 2014

LANGUAGE: ENGLISH

PUBLICATION-TYPE: Newspaper; Web Publication

JOURNAL-CODE: WEBI

Copyright 2014 Independent Digital News and Media Limited
All Rights Reserved

It has been used in Japan as the main sweetener for over 30 years and safety reviewed by both FSANZ and many other national food standard agencies in other countries e.g the EFSA and USFDA.

FSANZ has concluded that the use of steviol glycosides at proposed levels does not raise any public health and safety concerns.

Coca-Cola Life does not contain Aspartame so may be seen as healthier - but it does contain more sugar than Diet Coke.

'I guess with the other artificial sweeteners there is not a lot of evidence unless they are in very high doses that they are harmful. But because research hasn't been done checking Stevia it seems to be quiet safe in those respects,' Ms Hourigan said.

Coke Life is meant to taste similar to classic Coca-Cola but some have noted a slightly aniseed taste to the beverage.

The drink is already available in the UK, US, Mexico, Argentina, Chile and Sweden.

Rival soft drink brand Pepsi have also brought out a drink that uses Stevia as a sweetener in some markets. They have named it Pepsi Next.

Meanwhile, The Sydney Morning Herald has reported Coca-Cola Amatil will soon announce major job cuts to combat a drop in revenues in the Australian beverages business.

Chief executive Alison Watkins is expected to unveil a new profit-sharing structure with The Coca Cola Co in Indonesia.

LOAD-DATE: October 28, 2014

LANGUAGE: ENGLISH

PUBLICATION-TYPE: Web Publication

JOURNAL-CODE: WEBDM

Copyright 2014 Associated Newspapers Ltd.
All Rights Reserved MailOnline

October 23, 2014 Thursday 3:21 PM GMT

'Of course fat people CAN'T take responsibility for their weight - there's too much temptation everywhere,' says obesity doctor

BYLINE: DR SALLY NORTON FOR MAILONLINE

SECTION: HEALTH

LENGTH: 1210 words

- Dr Sally Norton is an NHS consultant specialising in weight loss
- Says rather than blame, overweight people need as much help as possible
- Argues busy lifestyles and constant temptation to eat junk are the culprits

- Says many of us have no time or energy to work on our weight, health and fitness in the remaining hours after finishing work
- Backs new plans unveiled today for more weight loss schemes in workplace

Obese people will be paid to lose weight through schemes in the workplace, under radical plans unveiled today.

Under the NHS-backed scheme, slimmers would be given rewards such as cash or shopping vouchers.

Employers will also be urged to offer incentives to staff who shed pounds.

Firms will be given some funding to set up slimming or exercise classes and also receive tax breaks from the Government.

Critics argue that obese people should take responsibility for their own health.

But Dr Sally Norton, an NHS consultant specialising in weight loss, says our busy lifestyles and constant temptation to eat fatty foods means this is simply unrealistic.

Dr Norton - who works at Bristol Royal Infirmary and Southmead Hospital - argues we should give overweight people as much help as possible, to try and halt the obesity crisis.

Here, she gives her view...

I am getting more and more cross.

Every time I discuss weight loss and health issues, someone will say, in a rather accusatory tone, 'surely it is down to the individual to take responsibility for their own weight and well-being'.

Perhaps, in an ideal world - but this is the real world and we need to face facts.

How can we be stuck in a sedentary job, eating poor quality food, hunched over a computer, under stress from 9-5, five days a week, and expect to have the time or energy to work on our weight, health and fitness in the remaining hours?

As an NHS weight loss surgeon, the person in front of me is seeking help.

Should my response then be: 'Well, of course you do understand I can't help you - it was your personal choice, your weak will, your lack of ability to resist temptation.

'And now you are going to die early from a weight related disease.

'Oh, and by the way, there's a Caramel Latte for you as you leave the premises - in one of the many coffee shops springing up in hospitals - to help with the shock?'

Of course not - because I don't believe that is true.

Instead, I will provide weight loss surgery, where appropriate.

But I think we should be insisting that employers, manufactures, retail outlets, governments and especially our hospitals are doing all they can to help us keep weight off in a healthy way.

Two thirds of us are overweight or obese and we are making sure that our kids are following in our footsteps.

The bottom line is, that however much we want to eat more healthily and take more exercise, we are surrounded by temptation wherever we turn.

Portions are getting bigger and bigger. Manufacturers are busy dreaming up increasingly calorie-packed offerings that they market to us with well-researched psychological manipulation.

We seem busier than ever, but in a sedentary way.

We are often trapped in offices with limited opportunity for activity, and everything is geared towards convenience: drive-throughs, escalators, home deliveries, gadgets to reduce physical effort.

So, yes, personal responsibility is important - but we need help.

It is blindingly obvious that we can't beat this on our own.

We need manufacturers to do their bit by providing us with easy access to healthier food choices.

We don't need tricks such as coercing us into buying double sized chocolate bars that we all know won't be shared - but instead will change our perception of a normal serving.

We also need to make health and well-being part of our daily lives, and that, for many of us, involves the workplace.

Simon Stevens has today suggested that companies should be encouraged to help us tackle our weight and health.

'BAN COFFEE SHOPS IN HOSPITALS SELLING SUGAR-LADEN DRINKS AND CAKES'

Dr Norton recently spoke out about the rise of coffee outlets in hospitals and says she no longer shakes the hands of her patients - more often than not because they arrive clutching a Costa take-out.

In the place of these popular coffee shops and fast food outlets, she has called for restaurants and cafes championing local food producers and offering healthy and tasty snacks.

'We read every week, in The BMJ and other leading medical journals, of research detailing the perils of sugar and **fizzy drinks**,' she said.

'We frequently hear laments about the cost to the NHS of the epidemic of obesity and type 2 diabetes, which is threatening to engulf us.

'And yet, the NHS, which I understood to be an organisation that promotes and supports health (rather than just treating disease), is actually contributing to the problem.

'As a weight loss surgeon, I find it frustrating and, frankly, embarrassing to spend time in clinic, explaining to my patients how **sugary drinks** and snacks are one of the biggest drivers of obesity, when I know that just outside in our hospital foyer are not one, but two Costa coffee shops, as well as vending machines stocked full of coke and chocolate.'

This has provoked all sorts of derisory comments about how we would be affronted to have our employers forcing us on to the scales every Monday for an office weigh-in.

Of course, there are good and bad ways to do this, but the crux is that many of us spend a vast proportion of our lives at work.

So if our work environment doesn't encourage us to be healthy then we just don't stand a chance.

Simon Stevens says the NHS is in financial crisis - but can we expect individuals to take responsibility for their health? Well it hasn't worked so far.

Simon Stevens is right.

If we don't challenge the causes of illness then the NHS is at risk of becoming bankrupt.

When providing a cure is financially not viable any more then the only choice is to identify the cause of the imbalance and tackle it at the roots.

It was never enough to look for a cure for cancer - it was always the aim to stop smoking through education; through warning on packaging; through removing temptation.

How is it any different for obesity?

Simon Stevens is asking government to get involved - to offer corporate rewards for health in the workplace - and I agree.

Let's make the most of this opportunity to improve our health, weight and well-being at our employer's expense - and we may even find work more enjoyable as a result.

Any employer shuddering at the thought of introducing this initiative can take note of the boom in workplace wellness programmes in the U.S., where companies have seen the benefit of caring for their staff.

UK employers lose over £20 billion a year due to absenteeism, but the costs of presenteeism - reduced

productivity at work due to ill-health or poor fitness - may be three times higher.

Being overweight or obese increases the number of sickness days taken by 50 per cent.

This equates to around £14 billion a year in lost revenue and may increase presenteeism rates 15-fold.

As doctors, we need to give you the holistic care you expect to help you lead a longer, happier life but, at the same time, we must try to preserve the NHS from bankruptcy.

LOAD-DATE: October 23, 2014

LANGUAGE: ENGLISH The Express

October 22, 2014 Wednesday
Edition 1;
National Edition

Schools should help children brush teeth to fight decay

BYLINE: Jo Willey

SECTION: NEWS; Pg. 6

LENGTH: 333 words

SCHOOLS and nurseries should help children brush their teeth in a bid to halt a growing epidemic of decay, health experts urge today.

Children as young as three are suffering from oral health problems including severe tooth decay.

In new recommendations, the National Institute for Health and Care Excellence urges local authorities, nurseries and schools to help tackle the crisis in disadvantaged areas of England.

Tooth decay and gum disease are the two most common and largely preventable dental problems.

Condemned Nice says those at risk include the most vulnerable in society and those who are dependent on others to care for them, such as young children and frail, older people.

Professor Mike Kelly, director of the centre for public health at Nice, said: "Children as young as three are being condemned to a life with rotten teeth, gum disease and poor health going into adulthood.

"Many children have poor diets and poor mouth hygiene because there is a misunderstanding about the importance of looking after children's early milk teeth and gums.

"They eat too much sugar and don't clean their teeth with fluoride toothpaste. As a society we should help parents and carers give their children the best start in life and act now to stop the rot before it starts."

Dr Sandra White, director of dental public health at Public Health England, said: "While children's oral health has improved over the past 40 years, one in eight three-year-olds has suffered from tooth decay."

She said oral health is "everyone's responsibility" and that nurseries, children's centres and primary schools all have a role to play.

It was revealed in July that rotten teeth were the top cause of hospital admissions among young children in the previous year.

Statistics also revealed that 500 children aged between five and nine had hospital dental treatment each week in 2013-14, with dentists removing all young patients' first teeth in some cases. The figures led to calls to limit the **sugary drinks** given to children.

LOAD-DATE: October 22, 2014

LANGUAGE: ENGLISH

PUBLICATION-TYPE: Newspaper

JOURNAL-CODE: DXP

Copyright 2014 Express Newspapers
All Rights Reserved

FINANCIAL TIMES
Financial Times (London, England)

October 22, 2014 Wednesday
London Edition 1

Strong dollar and cost of restructuring hit Coca-Cola; Beverages

BYLINE: Elizabeth Paton in New York

SECTION: COMPANIES; Pg. 20

LENGTH: 495 words

HIGHLIGHT: Quarterly revenues fall in Europe and US amid 'challenging' backdrop

Shares in Coca-Cola fell as much as 7 per cent yesterday after flatlining sales, a stronger dollar and heavy restructuring costs led to a weaker-than-expected third quarter for the world's largest beverage company.

Profit fell to $2.1bn for the three months to September 26, from $2.4bn a year before. Revenue totalled $11.9bn, falling short of the $12.1bn forecast by Wall Street.

Coke outlined plans to cut as much as $3bn in costs by 2019, and warned it expected profit for 2014 to be "below its long-term EPS [earnings per share] growth targets." The company added that currency effects would squeeze operating income for the full-year by 6 percentage points.

Chief executive Muhtar Kent , who has come under activist investor pressure this year over a controversial pay plan for senior executives, stressed that Coke was facing currency pressures and volatile economic environments in developed and emerging markets.

Sales volume rose 2 per cent in Latin America and Asia, but fell 5 per cent in Europe and 1 per cent in North America.

"We continue to face a challenging macro environment - far more challenging than was expected at the start of the year," he said, adding that geopolitical issues were weighing on travel and customer confidence.

"There just is a lot of apprehension and so . . . mobility is down. When traffic is down that impacts our immediate consumption business," he said.

The explanation contrasts with the confidence of rival PepsiCo earlier this month. Buoyed by sales from its more robust snacks business, it raised its full-year forecast after posting better than expected profit in the third quarter, saying it had not felt the brunt of a summer of political and economic global volatility.

"One of the nice things about our portfolio is that we power through geopolitical challenges pretty well. In good times and bad, people tend to want the simple pleasures we are able to offer them," Pepsi's chief financial officer, Hugh Johnston , told the Financial Times.

Both companies have been grappling with a long-term decline in carbonated drinks sales in developed

markets such as the US and Europe, where consumers have cut back on sugar.

A bid to diversify its drinks portfolio away from **fizzy drinks** has led to a number of high-profile acquisitions for Coke. In August Coke said it would pay $2.15bn for a 16.7 per cent stake in energy drink brand Monster, following its buyout of Zinco Coconut water and stakebuilding in Keurig Green Mountain.

Mr Kent said yesterday that Coke would sell back most of its company-owned North American bottling operations to independent bottlers by the end of 2017, and a substantial portion of the remaining territories no later than 2020 as part of a bid to streamline costs.

Shares in Coke have risen 4.8 per cent this year, lagging behind PepsiCo's 12.8 per cent increase but outperforming the S&P 500, which is up 3 per cent.

The shares were down 6.4 per cent to $40.52 in afternoon trading in New York.

See Lex

LOAD-DATE: October 21, 2014

LANGUAGE: ENGLISH

PUBLICATION-TYPE: Newspaper

Copyright 2014 The Financial Times Ltd.
All Rights Reserved
Please do not cut and paste FT articles and redistribute by email or post to the web.

PUBLICATION-TYPE: Web Publication

JOURNAL-CODE: WEBDM

Copyright 2014 Associated Newspapers Ltd.
All Rights Reserved

The INDEPENDENT

Independent.co.uk

October 22, 2014 Wednesday 7:42 PM GMT

Warren Buffett loses $2.5 billion in three days on Coca-Cola and IBM; Warren Buffett's investment firm holds large positions in IBM and Coca Cola

BYLINE: Maria Tadeo

SECTION: FRONTPAGE

LENGTH: 236 words

It's not been a good week for billionaire investor Warren Buffett.

His bet on Coca-Cola and American tech giant IBM have cost him more than $2 billion in just three days.

On Monday, Buffett lost nearly $1 billion after shares in IBM plummeted to a three-year low after it badly missed third-quarter earnings estimates and announced it would pay $1.5 billion to ditch its loss-making chip division.

That's bad news for Buffett considering his investment firm, Berkshire Hathaway, is the largest investor in IBM.

Yesterday, Buffett's week of hell continued after Coca-Cola shares plummeted six per cent in New York trading after it reported flat sales and lowered its guidance for the year.

Business news in pictures

Coca-Cola is battling an on-going shift in consumer taste as customers look for healthier options containing less **sugar** and fewer calories. Yesterday, it confirmed **soft drinks** sales fell 1 per cent in its flagship North American market.

And that is a problem for Coca Cola, which recently introduced a stevia-based drink containing naturally occurring sugars and splashed $2 billion to secure a 16 per cent in energy drinks company, Monster, in a bid a broaden its portfolio.

The set of disappointing results from both companies come after Buffett admitted he made a "huge mistake" investing in Tesco, which has seen its share price more than halve in the past 12 months following a series of profit warnings.

LOAD-DATE: October 22, 2014

LANGUAGE: ENGLISH

PUBLICATION-TYPE: Newspaper; Web Publication

JOURNAL-CODE: WEBI

Copyright 2014 Independent Digital News and Media Limited
All Rights Reserved

The Telegraph
telegraph.co.uk

October 22, 2014 Wednesday 10:03 AM GMT

McDonalds and Coca-Cola suffer as Americans lose taste for junk food; Profits tumble 30pc at McDonalds and Coca-Cola warns it will miss forecasts as younger customers seek out healthy alternatives

BYLINE: By Katherine Rushton US Business Editor

SECTION: FINANCE

LENGTH: 508 words

America may be the spiritual home of junk food, but profits at McDonalds and Coca-Cola are plunging amid signs that US customers want healthier fare.

McDonalds, the fast food chain, saw profits drop 30pc to $1.07bn in the last quarter, as customers turned their backs on its cheap burgers and fries. Trade at its American restaurants dropped 3pc on a like-for-like basis, whilst sales in its European branches dropped by 1pc.

The move was most pronounced amongst so-called "millennials", sparking anxiety amongst analysts and investors who fear that the recent decline in demand for fast food is the start of a more fundamental shift.

Many of them are gravitating towards so-called "fast casual" restaurants, such as Chipotle, the burrito chain, which are slightly more expensive than McDonalds, but are perceived as offering better quality fare.

Don Thompson, McDonalds' chief executive, said that the three months to October fell short of expectations "by all measures" and that McDonalds' challenges are "more formidable than expected".

McDonalds has already tried to combat America's drift away from junk food, by offering more salads and healthier dishes alongside traditional staples like chicken nuggets or Big Macs. However, the complex menu has slowed service, exacerbating the problem.

The restaurant chain has also suffered from a hike in its tax bill, and difficulties in Russia where some of its busiest restaurants have been shut down in a symbolic attack against the US. The business is also battling to regain consumer confidence in Asia, after it emerged that a meat supplier had sold out of date produce to McDonalds and other fast food businesses.

However, it was the underlying weakness in McDonalds' US business which has investors most concerned. Shares in the company fell 0.6pc to $91.05 in morning trading in New York.

Meanwhile, also appears to be losing its fizz. Shares in the drinks giant tumbled nearly 6pc to $40.76, as it warned investors that it will fall short of its profit forecasts for 2014.

The company was already part way through a cost-cutting plan, but said it would go even futher than planned, stripping out $3bn in annual costs between now and 2019.

"We have taken a hard look at our progress to date and realise that while the strategies we laid out at the beginning of the year are on the right track, the scope and pace of our actions must increase," said Muhtar Kent, chief executive.

Like McDonalds, Coca-Cola has been struggling to adapt to customers' changing tastes. Americans have

fallen out of love with high-sugar, high-calorie **fizzy drinks**, and are seeking out healthier alternatives instead.

Coca-Cola has dealt with this by introducing a raft of low-sugar alternatives to classic Coca-Cola, including the zero-calorie Coke Zero. However, customers are growing increasingly concerned about the potential negative effects of the artificial sweeteners those drinks use.

The drinks giant saw sales rise 1pc worldwide in the third quarter, as measured by volume, but in North America they fell 1pc.

LOAD-DATE: October 22, 2014

LANGUAGE: ENGLISH

PUBLICATION-TYPE: Newspaper; Web Publication

JOURNAL-CODE: WEBDTNS

Copyright 2014 Telegraph Media Group Limited
All Rights Reserved

The INDEPENDENT

Independent.co.uk

October 21, 2014 Tuesday 5:21 PM GMT

Coca Cola shares plunge as soft drinks market loses fizz; Coca Cola vows to streamline operations in an effort to slash costs faster

BYLINE: Maria Tadeo

SECTION: BUSINESS NEWS

LENGTH: 266 words

Shares in Coca Cola plunged in New York trading after the world's largest beverage company reported disappointing **fizzy drink** sales as consumers look for healthier options.

Coca Cola said net income for the third quarter ended September fell to $2.1 billion, or 48 cents a share, from $2.4 billion, or 54 cents a share, a year earlier.

While earnings per share came in line with analyst expectations, Coke said it no longer expects to meet its long-term target of high-single-digit growth.

Coca Cola also unveiled a new cost-cutting plan, which it said will save company $3 billion per year by 2019, stepping up efforts to slash costs from a previous target of $1 billion.

Business news in pictures

"We have taken a hard look at our progress to date and realise that while the strategies we laid out at the beginning of the year are on the right track, the scope and pace of our actions must increase," chief

executive Muhtar Kent added.

Coca-Cola said global beverage volume rose 1 per cent, as an increase in non-carbonated drinks lifted **fizzy drink** volume. In its flagship North American market, the company said soda and non-carbonated drinks each fell by 1 per cent. Overall revenue was flat at $11.97 billion.

Consumers are increasingly ditching **sugary drinks** in favour of healthier options. Diet drinks have also come under scrutiny with medical research linking artificial sweeteners with a rise in obesity.

In an effort to capture health-conscious customers, Coca Cola recently launched a new stevia-based **soft drink** marketed as mid-calorie alternative containing naturally occurring **sugar.**

LOAD-DATE: October 21, 2014

LANGUAGE: ENGLISH

PUBLICATION-TYPE: Newspaper; Web Publication

JOURNAL-CODE: WEBI

Copyright 2014 Independent Digital News and Media Limited
All Rights Reserved

Mail Online

MailOnline

October 20, 2014 Monday 4:35 PM GMT

Sugary drinks could age the body as much as SMOKING, scientists warn

BYLINE: SARAH GRIFFITHS FOR MAILONLINE

SECTION: HEALTH

LENGTH: 705 words

- **Fizzy drinks** appear to speed up the rate at which cells age
- People drinking two cans daily had DNA changes of cells 4.6 years older
- They measured telomeres - caps found at the ends of chromosomes
- Shorter than average telomeres are seen as a sign of premature death
- This is the first study to link soft drinks to premature ageing

Sugary soft drinks may accelerate ageing as much as smoking, new research shows.

Experts found the damage **fizzy drinks** cause to health goes beyond making people fat - they also appear to speed up the rate at which cells age.

The research showed that people who drank the equivalent of two cans of cola a day had DNA changes of cells 4.6 years older.

Campaigners have blamed **sugary drinks** for contributing to the rise in obesity and the number of people with type-2 diabetes, but this is the first piece to research to link **soft drinks** with premature ageing.

Scientists analysed thousands of DNA samples to find that people who regularly reached for a **fizzy drink** had shorter telomeres,

These are tiny structures that protect DNA from damage and are an indicator of health.

Found at the ends of chromosomes, they protect the DNA in them from damage, much like the caps on the ends of shoelaces prevent fraying.

As we get older, our telomeres get shorter and shorter, leading to DNA becoming damaged and raising the odds of age-related illnesses such as Alzheimer's, diabetes and heart disease.

Shorter than average telomeres are seen as a sign of ill health and premature death.

Studying telomeres, the scientists found people who regularly drank sugar-sweetened **fizzy drinks** had 'significantly' shorter telomeres than those who did not.

Professor Elissa Epel, from the University of California at San Francisco, said: 'Regular consumption of sugar-sweetened sodas might influence disease development, not only by straining the body's metabolic control of sugars, but also through accelerated cellular ageing of tissues.

'This is the first demonstration that soda is associated with telomere shortness.

'This finding held regardless of age, race, income and education level.

'Telomere shortening starts long before disease onset.'

While she only studied adults, Professor Epel warned it is possible that drinking **fizzy drinks** is linked to telomere shortening in children, too.

However, she stressed the study shows a link between soft drinks and ageing, but doesn't prove drinking **fizzy drinks** causes the ageing of cells.

HOW WORK CAN MAKE YOU STAY YOUNG FOR LONGER

Working keeps you young, according to a study published last summer.

The research linked unemployment with premature ageing. It is thought that the financial and emotional stress of being jobless makes its mark on the body's DNA.

Men who had been out of work for at least two of the three years before their blood was taken were more than twice as likely to have short telomeres as those who had been in continuous employment.

Researcher Jessica Buxton, of Imperial College London, said this suggests that the financial and emotional stress associated with being out of work was to blame.

She said: 'Stressful life experiences in childhood and adulthood have previously been linked to accelerated telomere shortening.'

In the study, the scientists measured telomeres in the white blood cells of 5,309 participants aged 20 to 65, with no history of diabetes or heart disease.

They found drinking two cans of cola a day - 20 fluid ounces - was linked to 4.6 years of ageing, based on telomere shortening and that one in five of the study's participants fell into this category.

Drinking a small soft drink daily, equivalent to eight fluid ounces, was associated with telomere shortening equivalent to 1.9 additional years of ageing.

The effect on telomere length was similar to that of smoking, the researchers wrote in the American Journal of Public Health.

Dr Cindy Leung, also of the university, said: 'It is critical to understand both dietary factors that may shorten telomeres, as well as dietary factors that may lengthen telomeres.

'Here it appeared that the only beverage consumption that had a measurable negative association with

telomere length was consumption of sugared soda.'

LOAD-DATE: October 20, 2014

LANGUAGE: ENGLISH

PUBLICATION-TYPE: Web Publication

JOURNAL-CODE: WEBDM

Copyright 2014 Associated Newspapers Ltd.
All Rights Reserved

The Daily Telegraph

The Daily Telegraph (London)

October 17, 2014 Friday
Edition 1;
National Edition

Two **fizzy drinks** a day could make you look four years older

SECTION: NEWS; Pg. 13

LENGTH: 329 words

DRINKING **sugary soft drinks** may accelerate biological ageing as much as smoking does, a study has found.

The findings, from an analysis of thousands of DNA samples, suggested that, as well as promoting obesity, sweet **fizzy drinks** may actually speed up the rate at which cells age.

The study focused on telomeres, protective caps on the ends of the chromosomes that provide a measure of biological ageing. Telomeres shorten with age, and short telomeres are associated with chronic problems of ageing such as heart disease, diabetes and some cancers.

The researchers found that people who regularly drank sugar-sweetened pop had significantly shorter telomeres than those who did not.

Prof Elissa Epel, a member of the US team from the University of California at San Francisco, said: "Regular consumption of sugar-sweetened sodas might influence disease development, not only by straining the body's metabolic control of sugars, but also through accelerated cellular ageing of tissues.

"This is the first demonstration that soda is associated with telomere shortness."

She said age, race, income and education level did not influence the findings. However she cautioned that the results did not prove a casual link. Prof Epel added: "Telomere shortening starts long before disease onset.

"Further, although we only studied adults here, it is possible that soda consumption is associated with telomere shortening in children, as well."

The scientists measured telomeres in the white blood cells of 5,309 participants aged 20 to 65 with no history of diabetes or heart disease. Consumption of 20 fluid ounces of pop a day - equivalent to about two cans of cola - was associated with 4.6 years of additional biological ageing, based on telomere shortening.

More than a fifth of the participants fell into this category of consumption.

The effect on telomere length was similar to that of smoking, the researchers said.

The findings appear in the American Journal of Public Health.

LOAD-DATE: October 17, 2014

LANGUAGE: ENGLISH

PUBLICATION-TYPE: Newspaper

JOURNAL-CODE: DTL

Copyright 2014 Telegraph Media Group Limited
All Rights Reserved

MailOnline

October 17, 2014 Friday 10:42 AM GMT

Run FOUR miles to burn off just one bottle of coke: Scientists call for exercise data to be printed on packaging instead of calories

BYLINE: BEN SPENCER FOR THE DAILY MAIL

SECTION: HEALTH

LENGTH: 572 words

- Scientists discover most people ignore calories printed on food and drink
- Calls for data to be show in terms of how long it would take to burn off
- Calories in a double cheeseburger are the equivalent of a 5.6 mile hike

If you knew you would need to go for a four-mile run to burn off the calories you have consumed, would you drink a bottle of Coca-Cola?

Scientists are calling for exercise data to be printed on packaging - because they think calorific information is meaningless to most people.

A 500ml bottle of Coke, for example, contains 210 calories, more than a 10th of the daily recommended intake for a woman.

But US scientists think that statistic is ignored by most people and does not work as a health message.

Instead, telling them that it would take a 4.2 mile run or 42-minute walk to burn off the calories is far more effective.

The researchers, from the Johns Hopkins Bloomberg School of Public Health in Baltimore, found that teenagers given the information chose healthier drinks or smaller bottles.

Other scientists have called for the same approach to be taken in fast food restaurants.

They say that if a menu tells you a double cheeseburger will take a 5.6-mile hike before the calories are burned off, most people would rather choose a smaller hamburger which would require a walk of 2.6 miles.

The findings, published in the American Journal of Public Health, adds to growing evidence suggesting that calorific information is ignored by most people.

Study leader Professor Sara Bleich said: 'People don't really understand what it means to say a typical soda has 250 calories.

'If you're going to give people calorie information, there's probably a better way to do it.

'What our research found is that when you explain calories in an easily understandable way such as how many miles of walking needed to burn them off, you can encourage behaviour change.'

The research team displayed signs in six corner shops in Baltimore, presenting facts about a 590ml bottles of **fizzy drinks**.

Scroll down for video

The signs said that to burn off the 250 calories in the drinks would require 50 minutes of running or a five-miles walk. The scientists found that customers bought far more healthy drinks once the signs went up.

The average calories of the drinks they purchased dropped from 203 calories to 179. Water purchases, meanwhile, increased from 1 per cent to 4 per cent.

Prof Bleich said the findings were especially relevant for young people.

'This is a very low-cost way to get children old enough to make their own purchases to drink fewer **sugar**-sweetened **beverages** and they appear to be effective even after they are removed.

'There is a strong scientific link between consumption of **sugary beverages** and obesity. Using these easy-to-understand and easy-to-install signs may help promote obesity prevention or weight loss.'

Separate research found that the same idea worked in fast food restaurants.

Scientists at the University of North Carolina found that exercise information persuaded people to choose more healthy food on menus.

Professor Anthony Viera at the University of North Carolina said: 'We believe that labels displaying information about physical activity will allow people to better appreciate the trade-offs of high-calorie foods, and thereby influence them to make choices for foods with lower calories.

'And we think that labelling foods like this may even have the extra benefit of promoting physical activity.'

LOAD-DATE: October 17, 2014

LANGUAGE: ENGLISH

PUBLICATION-TYPE: Web Publication

JOURNAL-CODE: WEBDM

Copyright 2014 Associated Newspapers Ltd.
All Rights Reserved

MailOnline

October 15, 2014 Wednesday 1:55 AM GMT

Making everyone green with envy! Rosie Huntington-Whiteley looks

sensational in one shoulder cut-out gown for new Coca-Cola Life campaign

BYLINE: SHARNAZ SHAHID FOR MAIL ONLINE

SECTION: TV&SHOWBIZ

LENGTH: 324 words

She is one of the most famous supermodels to have graced this planet, so it's hardly Rosie Huntington-Whiteley knows how to make the world green with envy.

The 27-year-old star - who is currently dating Hollywood hunk Jason Statham - looked sensational in a gorgeous jade coloured gown for her latest campaign with Coca-Cola Life.

The eye-catching floor-length dress showed off the model's slim figure to the maximum, with the frock featuring a cut-out detail to one side, teamed with a long sleeved shoulder.

Scroll down for video

In another shot, she is seen sipping on the new beverage dressed in a sparkly sequinned vest top.

Wearing her glossy blonde locks down in loose natural waves, Rosie certainly oozed heaps of elegance as she helped promote the new drink, which boasts a third less sugar and calories.

Speaking about the **fizzy drink**, Rosie said: 'I'm a great believer in not denying yourself when it comes to what you eat and drink but, like everything in life, it's about finding a healthy and happy balance.

'My favourite life moments are when I'm with hanging out with my friends and family, cooking and eating good food, sharing funny stories and dancing and singing to great music.

'I love to relax at home with my dogs, go swimming in the sea and take long walks in the countryside. Sometimes it's the simplest of things that can make you happy.'

Rosie, who has been dating The Expendables actor since 2010, is currently settling back into life in Los Angeles, after strutting her assets along the runways at Paris Fashion Week.

Although the British beauty leads an urban lifestyle since relocating to the States, Rosie still appears to be a country girl at heart.

'I miss my family and friends of course,' she explained. 'Whenever I go home to Devon I'm always reminded of how beautiful the countryside is in the UK.

'I always feel so peaceful and far away from it all when I'm back home. I'm very grateful and proud of where I come from.'

LOAD-DATE: October 15, 2014

LANGUAGE: ENGLISH

PUBLICATION-TYPE: Web Publication

JOURNAL-CODE: WEBDM

Copyright 2014 Associated Newspapers Ltd.
All Rights Reserved

MailOnline

October 14, 2014 Tuesday 9:11 AM GMT

Never mind sore muscles, SUGARY DRINKS may cost athletes a medal at the Olympics

BYLINE: MADLEN DAVIES FOR MAILONLINE

SECTION: HEALTH

LENGTH: 767 words

- Oral problems affected a fifth of athletes performances at the 2012 Olympics
- Nearly half reported they had not visited a dentist in the past year
- Nagging pains in the mouth putting athletes off could cost them medals
- Experts said sugary energy drinks and high-carb diets may be to blame
- Dehydration means less protective saliva is produced, causing tooth decay
- Flossing, brushing teeth properly could boost athlete's performance
- Using fluoride toothpaste, mouthwash and switching to water also helps
- Oral health should be prioritised to make slight gains, experts advised

Sugary energy drinks may have cost athletes a gold medal at the Olympics, health experts have today claimed.

Toothache and bleeding gums affected nearly a fifth of athletes' performances at the London 2012 Games, a new study has found.

Nagging pains in the mouth could have made the difference between medals by putting athletes off their stride, it said.

The survey found 18 per cent of top sports men and women complained the state of their teeth had a negative impact on their ability to perform.

Nearly half (46.5 per cent) reported they had not visited a dentist in the past year.

Saliva helps to protect teeth from decay and erosion, but becoming dehydrated from intense training can lead to a dry mouth, which increases the risk of oral health problems.

The amount of energy that athletes need for training often means they have high-carbohydrate diets and regularly use sugary, acidic energy drinks which may also contribute to tooth decay and erosion.

But simply flossing, knowing how to brush teeth properly, using fluoride toothpaste and mouthwash could provide the same marginal performance gains as expensive physical therapies, dentists advised.

The news comes after a study by German researchers published last month, which found the longer athletes exercised, the less saliva they produced and the more alkaline it became.

Alkaline saliva works to encourage the growth of plaque bacteria which causes tooth decay, the team explained.

For every extra hour of training each week, the study found an increased risk of a person needing fillings, or having decayed or missing teeth.

HOW EXERCISE ROTS YOUR TEETH

Hang up your trainers - because exercising could ruin your smile.

German researchers found the longer an athlete trains, the more damage they cause to their teeth.

They found that the longer athletes exercised, the less saliva they produced and the more alkaline it became.

Alkaline saliva works to encourage the growth of plaque bacteria, which causes tooth decay, the team explained.

They suggested that running might reduce the enamel-protecting protein found in saliva, meaning even drinking water could lead to tooth erosion.

For every extra hour of training each week, the study found an increased risk of a person needing fillings, or having decayed or missing teeth.

As part of the study a team of dentists at the University Hospital Heidelberg in Germany examined the teeth of 35 triathletes and 35 non-athletes.

Commenting on the results of the new study, health experts at University College London said dentistry must be given the same priority as other sports sciences to help boost their prowess on the track, field or in the pool.

Professor Ian Needleman of the UCL Eastman Dental Institute said: 'Oral health could be an easy win for athletes, as the oral conditions that can affect performance are all easily preventable.

'Professional athletes and their teams spend a lot of time and money on ways to marginally improving performance, as this can make all the difference in elite sports.

'Simple strategies to prevent oral health problems can offer marginal performance gains that require little to no additional time or money.

'Things like better tooth brushing techniques and higher fluoride toothpastes could prevent the toothache and associated sleeping and training difficulties that can make the crucial difference between gold and silver.

'We do not want to demonise energy drinks and are not saying that athletes shouldn't be using them.

'However, people should be aware of the risks to oral health and can take simple measures to mitigate these.'

He said that water or hypotonic drinks are more suitable for hydration, and told athletes to spit out toothpaste rather than rinsing their mouths out after brushing their teeth.

He added: 'For sports where athletes need a lot of energy drinks, high fluoride toothpastes and mouthrinses should be seriously considered.'

The advice was published in the British Journal of Sports Medicine.

LOAD-DATE: October 14, 2014

LANGUAGE: ENGLISH

PUBLICATION-TYPE: Web Publication

JOURNAL-CODE: WEBDM

Copyright 2014 Associated Newspapers Ltd.
All Rights Reserved

i-Independent Print Ltd

October 10, 2014
First Edition

Junk food tax hits soft drinks giants Coke and Pepsi; Panorama Around the world in 10 stories MEXICO

BYLINE: Candice Choi IN NEW YORK

SECTION: NEWS; Pg. 34

LENGTH: 216 words

Recent declines in sales suffered by Pepsi and Coke in Mexico underscore why the beverage industry is fighting tax proposals on **sugary drinks** in San Francisco and Berkeley, California.

PepsiCo said snacks sales volumes in Mexico have declined by 3 per cent as a consequence of a new tax on junk foods. PepsiCo - which makes Frito-Lay chips, Gatorade and Tropicana - reported similar declines in its snacks business for the first half of the year, when the tax went into effect.

Coca-Cola has also reported beverage volume declines in Mexico for the first half of the year, citing a similar tax on drinks. Mexico has the world's highest per capita consumption of Coca-Cola drinks.

In the US, San Francisco and Berkeley are seeking to become the first cities to pass taxes on **sugary drinks** in the upcoming November election. The measures are being closely watched because many say defeats in the Bay Area, which is known for its liberal politics, would be a major blow to advocates of such taxes as a way to improve nutrition. Similar measures in other US cities have failed.

Health advocates have pushed taxes as a tool to cut consumption of calorie-laden junk food, similar to tactics that have successfully been used against cigarettes. Makers of such products say they are being unfairly singled out. AP

LOAD-DATE: October 9, 2014

LANGUAGE: ENGLISH

PUBLICATION-TYPE: Newspaper

JOURNAL-CODE: III

Copyright 2014 Independent Print Limited
All Rights Reserved

mirror.co.uk

October 9, 2014 Thursday 2:53 PM GMT

Coca Cola Life, and why you can't have it both ways; If the lower sugar version of Coke is called Coca Cola Life, Mirror columnist Gary Bainbridge asks what that makes the higher sugar version?

BYLINE: By Gary Bainbridge

SECTION: NEWS,UK NEWS

LENGTH: 689 words

I was wandering through a branch of struggling retailer Tesco the other day, trying to find soap suitable for a man's face, and thinking that it would just be simpler if my employers paid Tesco directly every month.

And then I stumbled upon them - literally, long-time readers will be unsurprised to hear. It was a display of bottles of Coca-Cola in green packaging.

I expect most of you will already know about this development. Sadly I am way behind the curve when it comes to soft drink innovations and am constantly playing catch-up.

This is because I am no longer in the target market for pop manufacturers. The only time I see somebody roughly of my age in a Coca-Cola commercial these days is when Father Christmas appears on the side of a lorry.

But I am not yet dead, and so I picked up one of the bottles in an attempt to figure out what was going on. Perhaps Coca-Cola had come out on the Irish republican side, or at least was making a show of neutrality in the face of its continued production of orange Fanta. Maybe it had decided to start flavouring a variety of Coke with pesto. It couldn't taste any worse than Cherry Coke.

I read the label. The product was called Coca-Cola Life, and its unique selling point was that it didn't have quite as much sugar in it as normal Coke. But to prevent it from just tasting like mildly spiced brown water it was blinged up with a natural sweetener. A little light Googling tells me this is extracted from the stevia leaf, native to South America.

For a moment, imagine the marketing meeting which must have taken place when they decided on the branding of this new product. Wasn't there anybody in that room who thought that naming it Coca-Cola Life was a terrible idea? What does that make normal Coca-Cola with its normal amount of sugar - Coca-Cola Death?

And who is going to be taken in by this blatant pseudo-eco-nonsensical attempt to brand a carbonated soft drink with vegetable extract made in vast vats so that it appears as natural as sucking the dew off a blade of grass? The usual suspects, that's who, the worst people in the world.

The people who think you can have it both ways.

These are the people who are ethical right up to the point at which it affects their everyday lives. The socialists who are converted to selective or private education when it's apparent their own children are earmarked for Hellmouth Comp a mile away. The conservatives who rail against soft sentences for law breakers and then complain about the nanny state when they're fined for doing 34mph in a 30mph area.

We're all like that to some extent. The human race has a boundless capacity for hypocrisy. Being able to hold two conflicting points of view at the same time is sometimes even considered to be a sign of intelligence.

But the problem with Britain at the moment is that the major political parties are all targeting that sort of voter - the sort of voter who wants easy answers. The sort of voter who thinks you don't have to spend money to have a decent national health service. The sort of voter who thinks you can opt out of all the responsibilities of being part of the European Union, but still have all the benefits.

Because you can't. You cannot have it both ways. You can either spend £1 on a four-pint carton of milk, or

you can give small dairy farmers a decent living. You can either have low taxes, or you can have a fully resourced state with swept, pothole-free streets, an NHS which isn't falling apart, a safety net if you lose your job, and decent pensions.

At some point we have to make a choice. It's not necessarily as extreme as full-fat Coke from a supermarket or water from a stream, but currently our parliamentary political parties are not offering that choice.

And that is why our politicians are struggling to regain our respect. It's not because of expenses scandals - though that doesn't help - or yah-boo Prime Minister's Question Times. It's because they pretend that we can have it all, and deep down even the Coca-Cola Life drinkers they are courting know that is not true.

It's because they talk about hard choices, but refuse to put those choices in our hands.

LOAD-DATE: October 13, 2014

LANGUAGE: ENGLISH

PUBLICATION-TYPE: Newspaper; Web Publication

JOURNAL-CODE: WEBDMI

Copyright 2014 Trinity Mirror, Plc.
All Rights Reserved

THE TIMES

The Times (London)

October 9, 2014 Thursday
Edition 1;
National Edition

Sugary drinks can damage teenage brains

SECTION: NEWS; Pg. 25

LENGTH: 224 words

Soft drinks laden with **sugar** could damage teenagers' memory. Experiments on adolescent rats fed sweetened beverages showed that they suffered brain inflammation which affected their recall as well making them pre-diabetic. Researchers believe that their findings, published online in Hippocampus, will also apply to humans.

Professor Scott Kanoski, of Southern California University, said: "The brain is especially vulnerable to dietary influences during critical periods of development -like adolescence."

The lab animals were given water laced with large quantities of **sugar** or high-fructose corn syrup (HFCS) in concentrations comparable to popular **soft drinks**. Neither adult rats fed the same drinks nor adolescents who did not consume **sugar** had the same issues.

About 35 to 40 per cent of the rats' caloric intake was from sugar or HFCS. In comparison, added sugars

make up about 17 per cent of the total caloric intake of teens in the US on average, said the researchers.

The rats were then tested in mazes that probe their spatial memory ability. Adolescents that had consumed the **sugary beverages** - particularly HFCS - performed worse than any other group.

The hippocampus is a region of the temporal lobe located deep within the brain that controls memory formation. People with Alzheimer's disease and other dementias often suffer damage here.

LOAD-DATE: October 9, 2014

LANGUAGE: ENGLISH

PUBLICATION-TYPE: Newspaper

JOURNAL-CODE: TIM

Copyright 2014 Times Newspapers Limited
All Rights Reserved

MailOnline

MailOnline

October 7, 2014 Tuesday 11:54 PM GMT

'Sports drinks' contain NINE teaspoons of sugar and are no better for you than a can of Coke, study finds

BYLINE: LILLIAN RADULOVA FOR DAILY MAIL AUSTRALIA

SECTION: NEWS

LENGTH: 505 words

- Food Standards Australia New Zealand issued a proposal for electrolyte drinks 'to carry health claims related to their respective purposes'
- Consumer group Choice has opposed the proposal claiming it would mislead consumers
- Currently, products are required to meet a strict set of criteria including a certain level of sugar before they can claim they 'improve hydration'

A bottle of sports drink has nine teaspoons of **sugar** and should be on the shelves next to **soft drinks** such as Coke and Lemonade rather than being allowed to carry claims of health benefits on their bottles, a consumer advocacy group has warned.

Consumer group Choice claims most people receive no health benefits from drinking sports drinks, which contained a similar amount of sugar to a can of coke.

Choice has also revealed that a 600mL bottle of Gatorade has 36 grams of sugar - or nine teaspoons - while a standard 375ml can of Coca Cola has 40 grams, or one extra teaspoon.

The group said the drinks should be in soft drink isles of supermarkets rather then next to health foods.

The comments follows a submission by the Food Standards Australia New Zealand asking for electrolyte drinks 'to carry health claims related to their respective purposes'.

Drinks are currently required to have a certain level of energy, sugar and sodium before they can say that they 'improve hydration'.

'Rules about health claims were introduced last year to stop situations where you had clearly unhealthy products like sweets and chocolates making claims that they were 99 per cent fat free,' a spokesperson for CHOICE, Tom Godfrey, said.

'There's extensive research showing that any specific health claim gives a product a "halo effect" and people believe the product is healthier overall.'

'Sports drinks can help elite athlete but they aren't designed for everyday use,' Mr Godfrey said

'Yet drinks like Gatorade and Powerade are marketed and sold to everyone.

'Most people will receive no health benefit from a bright blue **sugar drink**. Sports drinks belong on the shelves next to Coke and Lemonade, not in the health food aisle and the claims on the label need to reflect this.'

The advocacy group claims that the drinks are already well overused by consumers who drink the product when playing sport, when they are simply spending time outdoors or as hangover cures.

However, Geoff Parker from the Australian Beverages Council said the labelling would allow product supplier to share research about the benefits of hydrating through sports drinks with consumers, rather than misleading them.

'What manufacturers wanted to do was to tap into a whole lot of internationally recognised research and scientific studies which are out there, which start to flesh out some of the more detailed, functional benefits that electrolytes and sports drinks can actually make,' he told the ABC.

'So some of their claims might be... that sports drinks hydrate better than water for intensive exercise, for example, or [that] sports drinks hydrate twice as fast as water for intensive exercise.'

LOAD-DATE: October 7, 2014

LANGUAGE: ENGLISH

PUBLICATION-TYPE: Web Publication

JOURNAL-CODE: WEBDM

Copyright 2014 Associated Newspapers Ltd.
All Rights Reserved

The INDEPENDENT

Independent.co.uk

October 6, 2014 Monday 5:12 PM GMT

Pepsi Cola launches new stevia-based soft drink 'Pepsi True'; Pepsi True has 30% less calories than normal Pepsi and follows the release of Coca-Cola Life, also made using stevia

BYLINE: Maria Tadeo

SECTION: FRONTPAGE

LENGTH: 210 words

Pepsi has announced it is to go head-to-head with Coca Cola with the launch of a new stevia-based soft drink marketed as a healthy alternative.

The new Pepsi True is sweetened using the naturally occurring sweetener extracted from a South American plant, and will be sold exclusively on Amazon.com in a major departure from the typical distribution chain in the beverage sector.

"It's a brand-new product proposition with a brand-new media platform, and we want to make sure the launch reflects a right-size approach," Simon Lowden, chief marketing officer at Pepsi Beverages North America.

The American beverage giant added the market for mid-calorie drinks - those containing more calories than its "diet" counterpart - remains small, and that Pepsi True would be a niche product as part of the wider portfolio that includes Pepsi Max and Diet Pepsi.

Business news in pictures

According to Pepsi, the new drink contains 30 per cent less calories than normal Pepsi and it does not use artificial sweeteners or high-fructose corn syrup, unlike most soft drinks in the market.

Pepsi True comes shortly after Cola-Cola launched its own stevia-based, healthier drink, Coca-Cola Life, in what's already being dubbed "the stevia wars" as consumers look for healthier options.

LOAD-DATE: October 6, 2014

LANGUAGE: ENGLISH

PUBLICATION-TYPE: Newspaper; Web Publication

JOURNAL-CODE: WEBI

Copyright 2014 Independent Digital News and Media Limited
All Rights Reserved

The Telegraph
telegraph.co.uk

October 24, 2014 Friday 5:48 PM GMT

Is your child addicted to sugar?;
Nearly half of mothers believe their children are addicted to sugar - but most are unaware quite how much they're really consuming

BYLINE: By Olivia Walmsley

LENGTH: 720 words

Nearly half of mothers (46 per cent) believe their children may be addicted to sugar, according to a new poll conducted by Bupa.

Yet many of them were unaware of the true scale of the problem, with the majority uninformed about the high sugar content of everyday foods such as yoghurts and juice.

Even though 43 per cent of the 2,000 mothers questioned claimed to know how much sugar was in the food their children eat, when asked how much was contained in everyday lunchbox items, most had no real idea.

While 88 per cent of children have at least one yoghurt a day, 93 per cent of the mothers questioned weren't aware that a typical fat-free yoghurt contains five teaspoons of sugar

The research found that 77 per cent of children consumed at least one glass of fruit juice each day - but 94 per cent of mothers didn't know that the average amount of sugar in a typical glass of apple juice is 6.5 teaspoons.

Some 60 per cent admitted that they fail to regularly check the nutritional information on food products for their children.

According to Mumsnet editor Sarah Crowne, sugar consumption is a continual source of anxiety among parents: "There's a great deal of discussion on Mumsnet about how to feed your kids well - from coping with fussy toddlers to tips for cajoling refusenik teens into managing a sensible breakfast before school.

"Excessive sugar consumption is a perennial concern amongst our users - particularly with 'hidden' sugar popping up in everyday products such as pasta sauce and bread, not to mention the large quantities in some of the most popular children's yoghurts and fruit snacks.

"In an ideal world, obviously, we'd all shun jars and packets and cook perfectly-balanced meals from scratch every day - but the fact is that time and money constraints often make this impossible.

"As with most aspects of child-rearing, parents simply do their best: to be clued-up about nutrition, avoid the obvious sugar-laden pitfalls and try to strike a healthy balance when feeding their kids."

Yet, in spite of these concerns and efforts, 40 per cent of parents questioned in the new poll said they gave

their children sweets, **fizzy drinks** or chocolate at least once a day.

Nearly 25 per cent surveyed said they placed sugary treats in their child's lunchbox, with the top culprits being cakes, chocolate, crisps and fruit juice. Nearly a third of mothers believed that, as they gave their children healthy food, too much sugar wasn't an issue.

Nicole Mowbray, author of Sweet Nothing, an account of giving up sugar, says: 'While these figures are shocking, they're not surprising."

"Even though parents try their best, sugar lurks in so many of the foods we give our children, from the addition of fruit juice to savoury baby foods to make it more palatable, to the high quantities of sugar lurking in everyday sauces such as tomato ketchup, or this new idea that honey is in some way healthier, when it isn't.

"That's before you've even considered the amount of sugar given to youngsters in the form of 'treats'. Parents should use their pressure power to force manufacturers to start reducing the amount of sugars they add to food and drinks aimed at children.'

Too much sugar in a child's diet can cause permanent damage to health, including increased risk of heart disease and Type 2 diabetes. as well as severe damage to dental health.

Andrew Wallis, of Cornwall Council, said earlier in the year that tooth decay among children had reached epidemic proportions. In fact, the research was commissioned by Bupa to mark the launch of its Tooth Fairy campaign - parents can create a personalised video for their children at toothfairy.bupa.co.uk.

Bupa's Clinical Director of Dentistry, Dr Steve Preddy commented: "With 26,000 primary school children admitted to hospital for tooth decay in the past year, there is a need now more than ever, for parents to be paying attention to their child's sugar consumption.

"The recent announcement from NICE has caused much debate amongst health and teaching authorities, however the most important thing to remember is tooth decay is preventable.

Parents need to be regularly looking at the nutritional information of food products; it is often what are thought of as healthy foods or unexpected ones."

Do you find it hard to limit your child's sugar intake?

LOAD-DATE: October 24, 2014

LANGUAGE: ENGLISH

PUBLICATION-TYPE: Newspaper; Web Publication

JOURNAL-CODE: WEBDTNS

Copyright 2014 Telegraph Media Group Limited
All Rights Reserved

MailOnline

December 1, 2014 Monday 12:22 PM GMT

The experiment that proves sugar really is poison: Man who drank 10 cans of Coke a day for a month gains TWO STONE and sees his blood pressure soar

BYLINE: MADLEN DAVIES FOR MAILONLINE

SECTION: HEALTH

LENGTH: 1058 words

- George Prior, 50, from Los Angeles, had a fit and healthy physique before
- Decided to drink 10 cans of Coca-Cola a day for an experiment
- Says he took on the challenge to highlight the harmful effects of sugar
- He gained 2st in weight, lost muscle and developed a protruding belly
- Also saw his blood pressure soar to an unhealthy 145/96
- Reported feeling intense sugar cravings and being so full he couldn't eat
- One can of Coke contains more sugar than the recommended daily intake

A protruding belly, high blood pressure and intense sugar cravings.

This is what happens to the body after drinking 10 cans of Coca-Cola a day, according to one man who took up the challenge.

George Prior, 50, decided to embark on a 'Coke diet' in order to show the harmful effects of the high levels of sugar found in the world's most popular drink.

In just 30 days he saw drastic changes to his formerly healthy and muscular physique.

He developed a protruding stomach and waistline and his weight ballooned from 12 stone (168lb) to 14 stone (192 lb).

His blood pressure soared from 129/77 to 145/96. This is way above the ideal level of 120/80 - and above this level, the risk of heart disease or stroke is greatly increased.

Scroll down for video

As well as the fast weight gain, Mr Prior, a father-of-two, said he feared he was becoming addicted to Coca-Cola after experiencing intense cravings.

While he tried to stick to his normal Paleo diet - a low-carbohydrate diet which focuses on eating lean meats, vegetables and berries - he began experiencing sugar cravings that were difficult to ignore.

'The most dramatic change was in weight: 23lb of gain over 30 days,' Mr Prior, who lives in L.A., said.

'I also seemed to develop a craving for Cokes, or other sugars, during the time I was drinking Cokes.'

'[As a result], I'm urging people to examine the amount of sugar in their diets.

'People need to be aware of the real and powerful damaging effects of sugar on their health.

Mr Prior's experiment comes shortly after New York assemblyman Karim Camara said **fizzy drinks** 'are the new smoking' and called for **sugar**-laden **beverages** to carry warning labels similar to those found on cigarette packets.

Mr Camara said the aim of his bill, which would introduce warning labels on drinks, is to educate the public, and lower the consumption of the drinks.

SO HOW MUCH SUGAR SHOULD WE BE EATING?

Earlier this year, the World Health Organisation published draft guidelines urging adults to eat no more than five cubes of sugar a day.

The guideline amount slashed was amid fears sugar poses same threat as tobacco.

The number of obese British adults is expected to double from one in four to one in two by 2050 - at a cost to the economy of £50 billion a year.

The WHO said the crisis was being fuelled by hidden sugar in processed food and drink such as yogurts, muesli, sauces, **fizzy drinks**, juice and smoothies.

However, low-fat foods have also come under fire from experts, after it was revealed they often contain more sugar than the full-fat alternative.

WHO also said children should try for less than five cubes of sugar a day and avoid cans of **fizzy drink** such as Coke, which contains seven cubes.

Instead five cubes is the ideal figure that people should aim for.

In March, the World Health Organization (WHO) called for the recommended daily intake of sugar to be slashed from 50g to 25g - or from 10 to five cubes a day.

A regular can of Coke contains 35g of sugar, the equivalent of seven cubes, so that alone would exceed this limit.

As part of his challenge, Mr Prior drank 10 cans of Coke a day - a total of 350g of sugar - the equivalent of around 70 cubes.

Experts warn **fizzy drinks** contain sugar but have no other nutritional value, and Mr Prior said he found it hard to eat as much food because the Coke left him full at lunch and dinner times.

The BBQ accessory entrepreneur said he wanted to stir up debate: 'I did the experiment to get people thinking and talking about how much sugar they eat and how unhealthy it is.

'I would prefer not to do it again.

'I don't like being this heavy.

'The actual drinking of the 10 Cokes got to be an irritating chore every day.

'There were a lot of visits to the restroom, a feeling of constant fullness, and a clutter of cans everywhere.'

After Mr Prior stopped drinking the fizzy soft drink he lost 5lb in four days.

He now concerned about the effect of sugar can have on children.

He said: 'Kids shouldn't drink Cokes.

'But then kids shouldn't drink juices, either, and that's going to be a very hard sell to parents who believe that juice is "natural", or even "organic".

'It's sugar, and not only do kids not need it, it's bad for them.

Indeed, past research has found **sugary drinks** are a primary contributor to type 2 diabetes and tooth decay, especially in children.

And experts warn that **fizzy drinks** are also fuelling the obesity epidemic - and, in turn, other serious illnesses, including cancer, heart disease and stroke.

Mr Prior added: 'I think there are a lot of people suffering health problems like diabetes and heart disease, who aren't aware they could help themselves by just stopping sugar.'

He also blasted Coca-Cola for their marketing techniques to make consumers believe 'they care' when they are just creating more products to sell.'

The latest member of the Coca-Cola family, called Coca-Cola Life, is marketed as having less sugar than regular Coke but none of the artificial sweeteners that increasingly worry Diet Coke drinkers.

The new naturally sweetened drink - which contains a third less sugar and calories than regular cola - has been created amid calls for the company to do more to tackle the global obesity epidemic.

Sold in a green can or a recyclable bottle, it is already available in the UK, U.S., Chile, Argentina and is due to hit Australian shelves in April.

Bur Mr Prior said: '**Sugar's** legal, **soft drinks** are legal,' he said. 'The responsibility is with consumers.

'Coke's genius plan is "reduce" the amount of sugar in their drinks.

'It's genius because it makes them look like they care, but still continue to sell sugar.

'I'd like to see controls on the lobbying and spending of big corporations who sell sugar and don't want the government to tell people how bad it is.'

MailOnline has approached Coca-Cola for comment.

LOAD-DATE: December 1, 2014

LANGUAGE: ENGLISH

PUBLICATION-TYPE: Web Publication

JOURNAL-CODE: WEBDM

Copyright 2014 Associated Newspapers Ltd.
All Rights Reserved

MailOnline

December 1, 2014 Monday 1:13 AM GMT

Labour 'ban on mince pies' in child obesity war: Party attacked over plans to put legal limits on sugar, fat and salt in food specifically aimed at youngsters

BYLINE: JACK DOYLE FOR THE DAILY MAIL

SECTION: NEWS

LENGTH: 618 words

- Labour's health spokesman wants to put legal limits on sugar, fat and salt
- Andy Burnham also said GPs should send obese for exercise classes
- Tory MP David Morris accused him of turning into the Grinch
- He said Mr Burnham wants to ban mince pies for children at Christmas

Labour was accused last night of trying to 'ban mince pies at Christmas' over plans to put draconian legal limits on sugar, fat and salt in food.

The party's health spokesman Andy Burnham said he was looking at mandatory maximum levels of the amount of fat, salt and sugar in foods specifically designed for youngsters.

He also said GPs should be sending obese patients for exercise classes to help them lose weight.

But last night one Tory MP accused Mr Burnham of behaving like 'the Grinch who wants to ban mince pies at Christmas.'

Conservative MP David Morris said: 'Conservatives are taking common sense steps to help people lose weight, but Labour just want to ban things.

'Andy Burnham is turning into the Grinch who wants to stop mince pies at Christmas. It's the same old we know best approach from Labour.'

The plan was welcomed by anti-obesity campaigners but was also branded 'ridiculous' and 'interfering'.

Shadow health secretary Mr Burnham told Sky News preventing health problems occurring in the first place was needed to help make the NHS sustainable.

Currently there are some referral schemes allowing patients to be sent to subsidised gym sessions. But Mr Burnham said physical exercise should be available more widely.

'Personally I would like to look more at exercise and physical activity - making referrals for physical activity from all GP surgeries,' he said.

'If people become more physically active, then they get more control over what they're eating and what they're drinking and all the other things.'

'The time has come to look at the food industry. I've said many times, I cannot defend the amount of sugar that children are eating.

'We've seen more and more sugar built into our food over time. I'm looking there at a mandatory maximum limit on fat salt and sugar in children's food. The status quo simply isn't working. We need to consider more radical action.'

The proposal was welcomed by Tam Fry from the National Obesity Forum, who said: ' Mr Burnham is absolutely right. It's probably the most important thing for the next government. He said 'runinous' levels of sugar, especially in **fizzy drinks,** were doing huge damage to children's teeth, as well as causing obesity.'

But Tory MP Philip Davies said: 'It's completely ridiculous. It is absolutely nanny state and basically the Labour Party want to use anything as an excuse to interfere in everybody's lives, and ban them from doing anything they don't personally approve of.

'It's a pretty good taste of what we could expect to see if we had the misfortune of a Labour government.'

The proposal would be an effort to combat obesity levels among children. A report last month found childhood obesity in the UK is at 'alarming' levels with up to one in three being dangerously fat.

Doctors said Britain is 'the fat man of Europe' and problems have set in among some children by the age of nine.

The Royal College of Paediatrics and Child Health (RCPCH), which represents doctors specialising in children and young people, said 26 per cent of boys and 29 per cent of girls are overweight and obese 'storing up serious health problems for the future'.

Mr Fry speculated that the food industry could be given several years to reduce the sugar and fat content in food voluntarily. If they did not comply then the law could be changed, he said.

In March UN health chiefs warned that children should not be give **fizzy drinks** because they contain dangerous amounts of sugar.

LOAD-DATE: December 1, 2014

LANGUAGE: ENGLISH

PUBLICATION-TYPE: Web Publication

JOURNAL-CODE: WEBDM

Copyright 2014 Associated Newspapers Ltd.
All Rights Reserved

MailOnline

December 1, 2014 Monday 11:57 AM GMT

The experiment that proves sugar really is poison: Man who drank 10 cans of Coke a day for a month gains TWO STONE and sees his blood pressure soar

BYLINE: MADLEN DAVIES FOR MAILONLINE

SECTION: HEALTH

LENGTH: 956 words

- George Prior, 50, from Los Angeles, had a fit and healthy physique before
- Decided to drink 10 cans of Coca-Cola a day for an experiment
- Says he took on the challenge to highlight the harmful effects of sugar
- He gained 2st in weight, lost muscle and developed a protruding belly
- Also saw his blood pressure soar to an unhealthy 145/96
- Reported feeling intense sugar cravings and being so full he couldn't eat
- One can of Coke contains more sugar than the recommended daily intake

A protruding belly, high blood pressure and intense sugar cravings.

This is what happens to the body after drinking 10 cans of Coca-Cola a day, according to one man who took up the challenge.

George Prior, 50, decided to embark on a 'Coke diet' in order to show the harmful effects of the high levels of sugar found in the world's most popular drink.

In just 30 days he saw drastic changes to his formerly healthy and muscular physique.

He developed a protruding stomach and waistline and his weight ballooned from 12 stone (168lb) to 14 stone (192 lb).

His blood pressure soared from 129/77 to 145/96. This is way above the ideal level of 120/80 - and above this level, the risk of heart disease or stroke is greatly increased.

As well as the fast weight gain, Mr Prior, a father-of-two, said he feared he was becoming addicted to Coca-Cola after experiencing intense cravings.

While he tried to stick to his normal Paleo diet - a low-carbohydrate diet which focuses on eating lean meats, vegetables and berries - he began experiencing sugar cravings that were difficult to ignore.

'The most dramatic change was in weight: 23lb of gain over 30 days,' Mr Prior, who lives in L.A., said.

'I also seemed to develop a craving for Cokes, or other sugars, during the time I was drinking Cokes.'

'[As a result], I'm urging people to examine the amount of sugar in their diets.

'People need to be aware of the real and powerful damaging effects of sugar on their health.

Mr Prior's experiment comes shortly after New York assemblyman Karim Camara said **fizzy drinks** 'are the new smoking' and called for **sugar**-laden **beverages** to carry warning labels similar to those found on cigarette packets.

Mr Camara said the aim of his bill, which would introduce warning labels on drinks, is to educate the public, and lower the consumption of the drinks.

SO HOW MUCH SUGAR SHOULD WE BE EATING?

Earlier this year, the World Health Organisation published draft guidelines urging adults to eat no more than five cubes of sugar a day.

The guideline amount slashed was amid fears sugar poses same threat as tobacco.

The number of obese British adults is expected to double from one in four to one in two by 2050 - at a cost to the economy of £50 billion a year.

The WHO said the crisis was being fuelled by hidden sugar in processed food and drink such as yogurts, muesli, sauces, **fizzy drinks**, juice and smoothies.

However, low-fat foods have also come under fire from experts, after it was revealed they often contain more sugar than the full-fat alternative.

WHO also said children should try for less than five cubes of sugar a day and avoid cans of **fizzy drink** such as Coke, which contains seven cubes.

Instead five cubes is the ideal figure that people should aim for.

In March, the World Health Organization (WHO) called for the recommended daily intake of sugar to be slashed from 50g to 25g - or from 10 to five cubes a day.

A regular can of Coke contains 35g of sugar, the equivalent of seven cubes, so that alone would exceed this limit.

Past research has found **sugary drinks** are a primary contributor to type 2 diabetes and tooth decay, especially in children.

And experts warn that **fizzy drinks** are also fuelling the obesity epidemic - and, in turn, other serious illnesses, including cancer, heart disease and stroke.

As part of his challenge, Mr Prior drank 10 cans of Coke a day - a total of 350g of sugar - the equivalent of around 70 cubes.

Experts warn **fizzy drinks** contain sugar but have no other nutritional value, and Mr Prior said he found it hard to eat as much food because the Coke left him full at lunch and dinner times.

The BBQ accessory entrepreneur said he wanted to stir up debate: 'I did it to get people thinking and talking about how much sugar they eat and how unhealthy it is.

'I would prefer not to do it again.

'I don't like being this heavy.

'The actual drinking of the 10 Cokes got to be an irritating chore every day.

'There were a lot of visits to the restroom, a feeling of constant fullness, and a clutter of cans everywhere.'

Since Mr Prior stopped drinking the fizzy soft drink he lost five pounds in four days.

He now concerned about the effect of sugar can have on children.

He said: 'Kids shouldn't drink Cokes.

'But then kids shouldn't drink juices, either, and that's going to be a very hard sell to parents who believe that juice is 'natural,' or even 'organic.

'It's sugar, and not only do kids not need it, it's bad for them.

'I think there are a lot of people suffering health problems like diabetes and heart disease, who aren't aware they could help themselves by just stopping sugar.'

He also blasted Coca-Cola for their marketing techniques to make consumers believe 'they care' when they are just creating more products to sell.

'**Sugar's** legal, **soft drinks** are legal,' he said. 'The responsibility is with consumers.

'Coke's genius plan is 'reduce' the amount of sugar in their drinks.

'It's genius because it makes them look like they care, but still continue to sell sugar.

'I'd like to see controls on the lobbying and spending of big corporations who sell sugar and don't want the government to tell people how bad it is.'

MailOnline has approached Coca-Cola for comment.

LOAD-DATE: December 1, 2014

LANGUAGE: ENGLISH

PUBLICATION-TYPE: Web Publication

JOURNAL-CODE: WEBDM

Copyright 2014 Associated Newspapers Ltd.
All Rights Reserved

The Express

November 28, 2014 Friday
Edition 1;
National Edition

SAB and Coke pair up;
City & Business Edited by PETER CUNLIFFE email: city@express.co.uk Visit City & Business pages online at www.express.co.uk/city Tel: 020 8612 7162

BYLINE: PETER CUNLIFFE

SECTION: BUSINESS; Pg. 71

LENGTH: 187 words

BREWING giant SABMiller is joining forces with Coca-Cola in a £1.8billion deal to create Africa's biggest bottler for the **fizzy drink**.

The FTSE 100 Grolsch and Peroni maker is thirsty for expansion in the continent's growing market for soft drinks and has agreed with Coke to combine the bottling operations of their non-alcoholic beverage businesses in southern and East Africa.

The new bottler, Coca-Cola Beverages Africa, will serve a dozen high-growth countries accounting for about 40 per cent of all Coke's beverage volumes in Africa. Its operations will have an annual revenue of

£1.8billion. SABMiller will have a 57 per cent stake. Soft drinks make up about 20 per cent of SAB's volumes but are growing faster than its beer operations.

SABMiller chief executive Alan Clark said: "Soft drinks are an important element of our growth strategy. This transaction increases our exposure to the total beverage market in Africa.

"This is significant with favourable demographics and economic development pointing to excellent growth prospects. This also signifies a strengthening of our strategic relationship with Coca-Cola."

LOAD-DATE: November 28, 2014

LANGUAGE: ENGLISH

PUBLICATION-TYPE: Newspaper

JOURNAL-CODE: DXP

Copyright 2014 Express Newspapers
All Rights Reserved

Daily Record & Sunday Mail

November 27, 2014 Thursday
Edition 1;
National Edition

Profits fizz at Britvic; HEALTH KICK FOR DRINKS FIRM

SECTION: FEATURES; OPINION, COLUMN; Pg. 58

LENGTH: 298 words

ROBINSONS squash firm Britvic have seen annual profits jump 23 per cent on the back of sales of more health conscious **fizzy drinks** such as sugar-free Pepsi Max.

The company said earnings lifted to £132.9million in the year to September 28 as their UK **fizzy drinks** brands grew volumes by 4.4 per cent in a market that declined overall.

Group sales rose 1.7 per cent to £1.3billion.

Britvic, whose other brands include Tango, J2O and Fruit Shoot, produce Pepsi, 7UP and Mountain Dew Energy under an agreement with PepsiCo.

They said Pepsi Max was a key driver of sales after a marketing campaign featuring football stars Lionel Messi and Sergio Aguero.

But Britvic - who rejected a merger with Scots rivals AG Barr last July - said this year had begun slowly, reflecting increasingly challenging trading conditions. Shares fell more than two per cent.

During the period, their still drink volumes, excluding water, fell five per cent, with Robinsons impacted by own-label competition.

They added that their premium juice drink, J20, saw their market share fall as customers focused on value.

During the year, Hertfordshirebased Britvic stopped selling the full sugar version of children's drink Fruit Shoot as part of "a commitment to address public health". In their international business, they signed a 15-

year franchise with US partners PepisCo to make Fruit Shoot in the US, and also launched the drink in India during the summer.

Britvic said cost savings would total £25million next year, and they were on track to deliver £30million of savings in 2016.

Chief executive Simon Litherland said: "We have delivered revenue and margin growth, and profit significantly ahead of last year, despite challenging trading conditions. However, we are confident of further improving our profitability in 2015."

LOAD-DATE: December 2, 2014

LANGUAGE: ENGLISH

PUBLICATION-TYPE: Newspaper

JOURNAL-CODE: DRC

Copyright 2014 Scottish Daily Record & Sunday Mail Ltd
All Rights Reserved

DAILY MAIL (London)

November 27, 2014 Thursday

BRITVIC SALES FIZZ

BYLINE: BY NO BYLINE AVAILABLE

LENGTH: 102 words

BRITVIC posted a 23pc leap in profits thanks to sales of more health-conscious **fizzy drinks** such as sugar-free Pepsi Max.

The firm said earnings lifted to £132.9m in the year to September 28 as its UK **fizzy drinks** brands grew volumes by 4.4pc in a market which declined overall. Group sales rose 1.7pc to £1.3bn. It said Pepsi Max was a key driver of sales following a marketing campaign featuring football stars.

But Britvic, which rejected a merger with smaller rival AG Barr last July, said this year had begun slowly reflecting increasingly challenging trading conditions.

Shares slid 41.5p to 655p.

© Daily Mail

LOAD-DATE: November 26, 2014

LANGUAGE: ENGLISH

PUBLICATION-TYPE: Papers

Copyright 2014 Associated Newspapers Ltd.
All Rights Reserved

DAILY MAIL (London)

November 27, 2014 Thursday

HALF OF CHILDREN ARE OVERWEIGHT BEFORE THEY EVEN REACH 11

BYLINE: BY LAURA CLARK EDUCATION CORRESPONDENT

LENGTH: 656 words

HALF of children have been classed as overweight before they leave primary school at 11, figures reveal.

Major research tracking 13,000 youngsters found more than a third - 35 per cent - were overweight at the age of 11, including 20 per cent who were classed as obese.

Researchers also identified a sharp rise in obesity rates between the ages of seven and 11 as parents begin to give children control over their food choices and sedentary activities, such as playing computer games, become more appealing to youngsters.

Academics behind the study said it should be a warning to parents of how easy it was for healthy children to become overweight or obese.

Overweight children face an increased risk of serious illnesses such as cardiovascular disease and type 2 diabetes, as well as psychological problems such as depression.

The research found that overweight youngsters were more likely to start puberty at an early age and were less likely to feel happy about their appearance. Heavier girls were also less likely to be happy with their lives in general.

In further findings, a clear link' emerged between children's weight at age 11 and their parents' level of education. Youngsters were more likely to be slim if their parents had degrees, the study said.

And children with overweight mothers were more likely to be overweight themselves.

The so-called Millennium Cohort Study - which is following British children born between 2000 and 2002 - weighed and measured participants at the ages of three, five, seven and 11.

Some 49 per cent were classed as overweight in at least one of the four surveys.

And 20 per cent of children were obese at the age of 11 and a further 15 per cent classed as overweight.

It meant that 35 per cent were either overweight or obese - up from 25 per cent at the age of seven. At that age, 13 per cent were considered obese and 12 per cent overweight.

The figures also showed that 12 per cent of children who were a healthy weight at seven had become overweight by 11 and a further six per cent had become obese.

Dr Roxanne Connelly, who led the research for London's Institute of Education, said: What this is showing is that children with a healthy weight can easily drop into the overweight category as they grow older. Parents should be cautious about that.'

She added: A major take-home message is that over time children tend to be tipping over into that overweight and obese category. It seems to be a pattern of moving in that direction rather than children becoming healthier.

One thing we did find was that a lot of children who were overweight at age seven were slowly becoming obese so it could be a creeping problem.'

The reasons for the big jump in obesity rates between the ages of seven and 11 needed further exploration, she said.

But she added: This is maybe the age where parents are letting go of the reins in terms of deciding what children are eating and having that sort of control over their diet. Also perhaps it is the age where children are moving away from active play towards more indoor computer game-type play.'

But Dr Connelly insisted the study showed it was by no means inevitable' that children will stay overweight. Some 4 per cent of children who were obese at seven had slimmed down to a healthy weight by 11.

Dr Ann Hoskins, of Public Health England, the agency responsible for tackling obesity said a virtual doubling' of overweight and obesity rates was deeply concerning'.

She said: Parents and carers can help their children maintain a healthy weight by following a balanced diet, ensuring portion sizes are not too large and avoiding **sugary drinks** and sugary or fatty snacks.

Children also need a minimum of 60 minutes of moderate to vigorous exercise each day, which can be achieved in one session or through shorter bursts of ten-minute activity.

PHE is working to help local authorities tackle the environmental causes of obesity. We are also working with schools.'

© Daily Mail

LOAD-DATE: November 26, 2014

LANGUAGE: ENGLISH

PUBLICATION-TYPE: Papers

Copyright 2014 Associated Newspapers Ltd.
All Rights Reserved

theguardian

The Guardian

November 27, 2014 Thursday 12:43 PM GMT

How to avoid more gastric surgery? Five ways we can change our health culture; Prevention is key to limiting the impact of widespread obesity on the NHS, which is why we need a wholesale change in attitude

BYLINE: Sarah Boseley

SECTION: COMMENT IS FREE

LENGTH: 661 words

Redesigning the human gut is a pretty extreme measure to cope with the junk food and all-day eating culture of the 21st century. But Nice, the National Institute for Health and Care Excellence, just gave its blessing to weight-loss stomach surgery for thousands more people than are currently getting it.

About 6,500 people went under the knife in 2009-10, undergoing one of several gastric operations that will prevent them eating too much food - or make them feel very ill trying. That could rise to 15,000 a year under Nice's proposals, and possibly many more. An astonishing 2 million people would be eligible.

This is a pragmatic answer to misery and ill-health, and a way to reduce soaring NHS bills. Around 10% of the entire NHS budget is spent on type 2 diabetes and its complications, which is usually caused by obesity: people go blind, their kidneys fail, they have heart attacks and strokes, and there are 100 amputations every week as a result of this disease.

The smug and skinny who say it is all fat people's own fault are plain ignorant. Trying to lose weight is more than hard. Our biology is fighting us. Diets work for a few weeks and then the weight all goes back on. Our hormones tell our metabolism to slow down because we appear to be in danger of starving to death.

Gastric surgery can press the reset button. Gastric bands - which account for only 8% of weight-loss operations - just reduce the size of the stomach pouch, leaving some people with the same food cravings as before, but the gastric bypass and the sleeve appear to change people's appetite so that they want less food, including less fatty and sweet food. And within weeks most people with type 2 diabetes are off their medication. For the obesity experts, it's a kind of magic.

But it is not the answer for everyone. Surgery is always risky and there are occasional deaths. It has to be reserved for those people who have no other option - and even 25% of those say no. And it is absolutely not the solution to the obesity epidemic. The NHS's chief executive, Simon Stevens, has already said that prevention is the key, and he is right.

To tackle this massive and growing issue at source, we have to change attitudes and confront some vested interests head-on. We are long past the point where it could be argued that it is up to the individual to take a look at themselves in the mirror, get a set of bathroom scales and cut down on the chips. Here are some of the things that need to happen:

1. Government needs to get real and get tough about changing the food environment. Voluntary agreements with industry to list calories and reduce fat in processed foods are a half-hearted business. The public health responsibility deal needs to be enforced with a big stick. Companies that do not use traffic light labelling need to be named and shamed. The marketing of sweets and fatty foods and **sugary drinks** to children has to be stopped.

2. Food taxes need to be introduced, alongside subsidies for fruit and vegetables. People buy food on price, particularly in the less affluent areas of the country where obesity tends to be higher. A tax on sugar-sweetened drinks might not even be a vote loser in the current anti-sugar climate.

3. Towns need to be designed to encourage physical activity and safe cycling. This should be a default for planners.

4. Children need healthy meals in school - all credit to Jamie Oliver for making waves - and also food education. They need to know what is good to eat and what is not so good. And they need to learn to cook it.

5. Perhaps most difficult of all, we need to get over the blame and shame culture. Obesity is not just about gargantuan people. Being overweight technically begins at a BMI of 25 and obesity at 30. People may not look obviously huge, but their health is at risk. Two-thirds of adults and a quarter of children are now in the overweight and obese category. It's not a dirty little individual secret any more. We need to talk.

LOAD-DATE: November 27, 2014

LANGUAGE: ENGLISH

PUBLICATION-TYPE: Newspaper

JOURNAL-CODE: WEBGNS

Copyright 2014 The Guardian, a division of Transcontinental Media Group Inc.
All Rights Reserved
The Express

November 27, 2014 Thursday
Edition 1;
National Edition

Rivals dilute Britvic;
City & Business Edited by PETER CUNLIFFE email: city@express.co.uk Visit City & Business pages online at www.express.co.uk/city Tel: 020 8612 7162

SECTION: BUSINESS; Pg. 63

LENGTH: 183 words

A GROWING thirst for health conscious **fizzy drinks** put some sparkle into Britvic's annual profit but shares fell flat amid fears that fierce competition will hit sales.

The Robinsons and Tango maker scored a better-than-expected 17.6 per cent rise in full-year pretax earnings to £158.1million as revenue rose 2.4 per cent to £1.34billion.

Britvic, which last year rejected a merger with smaller rival AG Barr, scored with sales of drinks such as sugar-free Pepsi Max, following a marketing campaign featuring football stars such as Lionel Messi, Jack Wilshere and Sergio Aguero. Achieving cost savings sooner than expected helped the bottom line but sales of premium juice drinks such as J2O dried up as consumers sought cheaper alternatives.

Chief executive Simon Litherland said: "We have delivered revenue and margin growth despite challenging trading conditions in each of our markets. We are confident of improving our profitability in 2015 as we bring to market our strong innovation and marketing plans and bene-fit from the delivery of the cost-savings programme."

Shares fell 41½p to 655p.

LOAD-DATE: November 27, 2014

LANGUAGE: ENGLISH

PUBLICATION-TYPE: Newspaper

JOURNAL-CODE: DXP

The Independent
Independent.co.uk

November 27, 2014 Thursday 4:45 PM GMT

The Fairlife 'Coke Milk' adverts: Do we really need pin-up girls to sell us drinks?; The 'racy' advertisment is patronising for their female and male audiences

BYLINE: Ylva Johannesson

SECTION: COMMENT

LENGTH: 504 words

Coca-Cola are investing in a new processed milk drink, developed by Fairlife, which claims to contain 50 per cent more protein and half the **sugar** of normal milk, with naturally enhanced Calcium levels. According to Coke, this new high protein drink will make it "rain money" for the **soft drinks** company. Health and nutrition claims aside, with the drink selling at twice the price of normal milk, this seems likely.

Nevertheless, that's not the most disconcerting aspect about the drink, which has already been branded "Milka-Cola". What worries me is the accompanying advertisement campaign for this "good-for-you milk". A series of mainly white, young women with wide open mouths and long flowing hair, wearing high heels and skimpy white 'milky' dresses are portrayed as pin-up girls. Even if this is an ironic joke (which sadly, it seems not to be) the female stereotyping is almost too obvious.

Similar to previous gendered advertisements of every day products, used to sell everything from beer to soap, Fairlife milk is being marketed with an undertone of wholesomeness and cleanliness. While the woman in the classic Lux soap ad became edible through her clean skin, the Fairlife woman becomes drinkable through her thin waist.

In one of the images a blond girl is pictured standing on a scale, slightly bent over revealing to reveal her a sliver of bottom. The caption reads: "better milk looks good on you". Another image shows a brunette woman posing in a milk-splashed dress, which barely covers her crotch, with the words "drink what she's wearing". As if the images themselves weren't insulting enough, these captions enhance the sexist undertones of a message supposedly intended to focus on health and nutrition.

10 Of The Most Controversial Fashion Adverts

Previous attempts to promote cow's milk in America include the long standing Got Milk? campaign, which among other things featured animated cows and athletes. In the UK, the "Make Mine Milk" campaign in 2010 targeted teenagers using a variety of celebrities of different ages, backgrounds and gender. Overall, pretty mainstream, but effective.

At what point did milk, a humble drink traditionally marketed for children and teenagers, become associated with pin-up girls? Unlike "Make Mine Milk", Coke's new campaign does not seem to be about awareness, but promotion for a product with the sole purpose of profit. But, Fairlife's 'racy' advertisment is patronising for their female and male audiences. Have the creators of this campaign thought this through at all? After all, it is now 2014, not 1940.

"Classics never go out of style" Fairlife states on their website. Sadly, objectifying representations of women have become 'classics', and this campaign is yet another example of the ways in which sexism operates:

through the repetition of language and image, whereby degrading depictions of women gradually become normalised and made invisible. These problems will not be solved over night, but taking these ads off the market is a good start.

LOAD-DATE: November 27, 2014

LANGUAGE: ENGLISH

PUBLICATION-TYPE: Newspaper; Web Publication

JOURNAL-CODE: WEBI

Copyright 2014 Independent Digital News and Media Limited
All Rights Reserved

The INDEPENDENT
Independent.co.uk

November 26, 2014 Wednesday 9:36 AM GMT

Coke milk? Coca-Cola to launch premium milk brand called Fairlife; The racy marketing to entice consumers to buy Fairlife, which launches in the US next month

BYLINE: Jamie Merrill

SECTION: FRONTPAGE

LENGTH: 602 words

When Coke tried to muscle in on the mineral water market, it was hounded out for selling bottled tap water.

Now the company has got its commercial eyes on milk. But rather than just repackaging another part of our everyday diet, the firm boasts its drink will come souped up in a high-protein, high-calcium, low-sugar form - and sold at twice the price of a normal pint.

Fairlife will go on sale in the US next month and according to Coca-Cola, a major investor in the product, it will be more "nutritious" with 50 per cent more natural protein and calcium and less sugar than ordinary milk.

Heralded by breathless promotional materials that claim the new drink will take "milk where it's never been before", the drink is seen as the "premiumisation of milk" by the company.

Speaking at Morgan Stanley's Global Consumer Conference last week, Coca-Cola's North American chief, Sandy Douglas, said: "It's basically the premiumisation of milk... We'll charge twice as much for it as the milk we're used to buying in a jug."

Mr Douglas claimed that the milk "tastes better" than regular milk and is made on sustainable dairy farms with "high-care processes" and a "proprietary milk-filtering process".

Much of America's milk is made in controversial mega-dairies where up to 30,000 cows are kept indoors all year round. But Mr Douglas said its milk will come from 92 family-owned farms, and Fairlife boasts that it will be "pursuing the highest standards of milk quality, agricultural sustainability and animal comfort".

A Fairlife spokesman said: "In response to consumer demand for better, wholesome nutrition from safe, responsible sources, Fairlife, a joint venture between Coca-Cola and the Select Milk Producers dairy co-op, is excited to soon be introducing an innovative ultra-filtered milk that... offers consumers a dairy option that is sourced from sustainable family farms and provides strong market potential to redefine the category."

The move is a major long-term investment for Coca-Cola, which has traditionally focused on carbonated drinks and owns nearly 1,000 drinks brands worldwide.

More recently it has branched out into still orange juice in America and low-**sugar drinks** elsewhere in the world. Soda stream: Failed drinks

Mr Douglas added: "We're going to be investing in the milk business for a while to build the brand so it won't rain money in the early couple of years. But like Simply [orange juice], when you do it well it rains money later."

Not all Coca-Cola brand adventures have been profitable though. In 2004 the firm was forced to take its Dasani bottled water off British shelf after just four weeks.

The firm was unable to convince British consumers to buy the drink, which was reported to be Sidcup tap water that had been filtered and pumped full of minerals. It was found to contain illegal levels of the chemical bromate.

Soda stream: Failed drinks

Surge

The idea was to knock Mountain Dew off the top spot in the lucrative extreme sports drinks market, but Surge was discontinued in 2002 after six years on sale.

Sprite Remix

This clear drink emerged in 2003 and quickly developed a strong fan base. Drinkers soon got bored with its sickly taste though.

OK Soda

Coca-Cola killed off OK Soda within seven months, in 1993. Marketing focused on the caffeinated drink's transparent appearance, ignoring its overly sweet taste.

Tab Clear

The soda did well enough to go global, but customers got bored and Coca-Cola pulled the drink by 1994.

LOAD-DATE: November 26, 2014

LANGUAGE: ENGLISH

PUBLICATION-TYPE: Newspaper; Web Publication

JOURNAL-CODE: WEBI

Copyright 2014 Independent Digital News and Media Limited
All Rights Reserved

The INDEPENDENT
The Independent (London)

November 26, 2014 Wednesday
First Edition

It's milk but not as we know it - Coke 'premiumises' the white stuff

BYLINE: JAMIE MERRILL

SECTION: NEWS; Pg. 19

LENGTH: 553 words

When Coke tried to muscle in on the mineral water market, it was hounded out for selling bottled tap water.

Now the company has got its commercial eyes on milk. But rather than just repackaging another part of our everyday diet, the firm boasts its drink will come souped up in a high-protein, high-calcium, low-sugar form - and sold at twice the price of a normal pint.

Fairlife will go on sale in the US next month and according to Coca-Cola, a major investor in the product, it will be more "nutritious" with 50 per cent more natural protein and calcium and less sugar than ordinary milk.

Heralded by breathless promotional materials that claim the new drink will take "milk where it's never been before", the drink is seen as the "premiumisation of milk" by the company.

Speaking at Morgan Stanley's Global Consumer Conference last week, Coca-Cola's North American chief, Sandy Douglas, said: "It's basically the premiumisation of milk??? We'll charge twice as much for it as the milk we're used to buying in a jug."

Mr Douglas claimed that the milk "tastes better" than regular milk and is made on sustainable dairy farms with "high-care processes" and a "proprietary milk-filtering process".

Much of America's milk is made in controversial mega-dairies where up to 30,000 cows are kept indoors all year round. But Mr Douglas said its milk will come from 92 family-owned farms, and Fairlife boasts that it will be "pursuing the highest standards of milk quality, agricultural sustainability and animal comfort".

A Fairlife spokesman said: "In response to consumer demand for better, wholesome nutrition from safe, responsible sources, Fairlife, a joint venture between Coca-Cola and the Select Milk Producers dairy co-op, is excited to soon be introducing an innovative ultra-filtered milk that??? offers consumers a dairy option that is sourced from sustainable family farms and provides strong market potential to redefine the category."

The move is a major long-term investment for Coca-Cola, which has traditionally focused on carbonated drinks and owns nearly 1,000 drinks brands worldwide. More recently it has branched out into still orange juice in America and low-**sugar drinks** elsewhere in the world.

Mr Douglas added: "We're going to be investing in the milk business for a while to build the brand so it won't rain money in the early couple of years. But like Simply [orange juice], when you do it well it rains money later."

Not all Coca-Cola brand adventures have been profitable though. In 2004 the firm was forced to take its Dasani bottled water off British shelf after just four weeks.

The firm was unable to convince British consumers to buy the drink, which was reported to be Sidcup tap water that had been filtered and pumped full of minerals. It was found to contain illegal levels of the chemical bromate.

GOING FLAT

COCA-COLA'S FAILURES

Surge

The idea was to knock Mountain Dew off the top spot in the lucrative extreme sports drinks market, but Surge was discontinued in 2002 after six years on sale.

Sprite Remix

This clear drink emerged in 2003 and quickly developed a strong fan base. Drinkers soon got bored with its sickly taste though.

OK Soda

Coca-Cola killed off OK Soda within seven months, in 1993. Marketing focused on the caffeinated drink's transparent appearance, ignoring its overly sweet taste.

LOAD-DATE: November 25, 2014

LANGUAGE: ENGLISH

PUBLICATION-TYPE: Newspaper

JOURNAL-CODE: IA

Copyright 2014 Independent Print Ltd
All Rights Reserved

MailOnline

MailOnline

November 26, 2014 Wednesday 12:47 AM GMT

Coca-Cola to sell milk... at twice the normal price: Firm says lactose-free product with 30% more calcium will 'rain money' for the company

BYLINE: RUPERT STEINER, CHIEF CITY CORRESPONDENT FOR THE DAILY MAIL

SECTION: HEALTH

LENGTH: 403 words

- Company to produce brand of milk as it moves away from **sugary drinks**

- Bosses predict new milk product Fairlife will 'rain money' for the firm
- Drink will cost twice as much as normal milk but boasts 50% more protein

Coca Cola is to to produce its own brand of milk as it expands from carbonated **sugary drinks**.

The American drinks giant is making a daily pint called Fairlife which it predicts will 'rain money' for the firm.

The new product will cost double the price of traditional versions containing higher protein, less sugar and it will be lactose free.

Fairlife will contain 50 per cent more protein, 50 per cent less sugar than normal milk, and 30 per cent more calcium.

Coke's move into the dairy business follows a shift into water and its Simply juices as profits fall from its main range of **fizzy drinks**.

The milk claims it has a longer shelf life than ordinary pasteurised milk - as 'we pasteurise our milk at an even higher temperature for less time'.

Consumers are increasingly drinking healthier products containing less fat and sugar.

Speaking at a consumer conference Coke's global chief customer officer Sandy Douglas said: 'We're going to be investing in the milk business for a while to build the brand so it won't rain money in the early couple of years. But like Simply, when you do it well it rains money later.'

Douglas said that the milk which has been tested in Minnesota in America will 'taste better and we'll charge twice as much for it as the milk we are used to buying in a jug.'

The product is specially filtered to extract elements of fat and sugar and Coke hopes that it will compete in the rapid growth sports and energy drinks market.

It comes at a time when milk sales in America have been tumbling as consumers moved to orange juice and other drinks.

A spokesman said Fairlife is only available in America at present.

The firm has a chequered history in terms of launching new products in the UK.

In 2004 it was discovered that the main ingredient to its Dasani water brand which was priced at 95p a bottle was tap water.

Bottled in south-east London, wags pointed out that Coke's venture into water was remarkably similar to Del Boy's attempts to sell Peckham water in Only Fools and Horses.

Later 500,000 of its bottles in the UK eventually had to be withdrawn after it was discovered the drink had been potentially contaminated with carcinogenic chemicals.

The brand was never reintroduced to the UK.

LOAD-DATE: November 26, 2014

LANGUAGE: ENGLISH

PUBLICATION-TYPE: Web Publication

JOURNAL-CODE: WEBDM

Copyright 2014 Associated Newspapers Ltd.
All Rights Reserved

The Guardian

November 25, 2014 Tuesday 8:08 PM GMT

Coca-Cola enters dairy market with 'Milka-Cola'; Fairlife will cost twice as much as regular milk, contain 30% less sugar, and 'make it rain money' for the soft drinks firm

BYLINE: Rupert Neate

SECTION: BUSINESS

LENGTH: 240 words

Coca-Cola is launching its own brand of milk, which it claims could become so popular that it will "rain money" for the company.

Fairlife, which will launch in the US next month, will cost twice as much as regular milk and will have 50% more protein and 30% less sugar.

Sandy Douglas, Coke's global chief customer officer, said Fairlife was "a milk that's premiumised and tastes better and we'll charge twice as much for it as the milk we're used to buying".

He told a conference: "We're going to be investing in the milk business for a while to build the brand, so it won't rain money in the early couple of years. But like Simply [Coke's premium fruit juice line], when you do it well, it rains money later."

Douglas said Fairlife, a joint venture with US dairy farmers, used "a proprietary milk filtering process that allows you to increase protein by 50%, take sugar down by 30%, and have no lactose."

He made the comments at a Morgan Stanley investment conference last week, but a transcript has only recently been released.

Milk sales in the US are down 8% over the past decade, and half of American adults do not drink milk, according to the trade journal Dairy Today.

A Fairlife spokeswoman said there were no plans to launch the product, which has already been branded "Milka-Cola" on Twitter, outside the US. Coke is still smarting from a failed attempt to sell bottled tap water in the UK under the brand Dasani in 2004.

LOAD-DATE: November 25, 2014

LANGUAGE: ENGLISH

PUBLICATION-TYPE: Newspaper

JOURNAL-CODE: WEBGNS

Copyright 2014 The Guardian, a division of Transcontinental Media Group Inc.

All Rights Reserved

The INDEPENDENT
Independent.co.uk

November 25, 2014 Tuesday 6:48 PM GMT

Coke milk? Coca-Cola to launch premium milk brand called Fairlife; The racy marketing to entice consumers to buy Fairlife, which launches in the US next month

BYLINE: Jamie Merrill

SECTION: FRONTPAGE

LENGTH: 597 words

When Coke tried to muscle in on the mineral water market, it was hounded out for selling bottled tap water.

Now the company has got its commercial eyes on milk. But rather than just repackaging another part of our everyday diet, the firm boasts its drink will come souped up in a high-protein, high-calcium, low-sugar form - and sold at twice the price of a normal pint.

Fairlife will go on sale in the US next month and according to Coca-Cola, a major investor in the product, it will be more "nutritious" with 50 per cent more natural protein and calcium and less sugar than ordinary milk.

Heralded by breathless promotional materials that claim the new drink will take "milk where it's never been before", the drink is seen as the "premiumisation of milk" by the company.

Speaking at Morgan Stanley's Global Consumer Conference last week, Coca-Cola's North American chief, Sandy Douglas, said: "It's basically the premiumisation of milk... We'll charge twice as much for it as the milk we're used to buying in a jug."

Mr Douglas claimed that the milk "tastes better" than regular milk and is made on sustainable dairy farms with "high-care processes" and a "proprietary milk-filtering process".

Much of America's milk is made in controversial mega-dairies where up to 30,000 cows are kept indoors all year round. But Mr Douglas said its milk will come from 92 family-owned farms, and Fairlife boasts that it will be "pursuing the highest standards of milk quality, agricultural sustainability and animal comfort".

A Fairlife spokesman said: "In response to consumer demand for better, wholesome nutrition from safe, responsible sources, Fairlife, a joint venture between Coca-Cola and the Select Milk Producers dairy co-op, is excited to soon be introducing an innovative ultra-filtered milk that... offers consumers a dairy option that is sourced from sustainable family farms and provides strong market potential to redefine the category."

The move is a major long-term investment for Coca-Cola, which has traditionally focused on carbonated drinks and owns nearly 1,000 drinks brands worldwide.

More recently it has branched out into still orange juice in America and low-**sugar drinks** elsewhere in the world.

Mr Douglas added: "We're going to be investing in the milk business for a while to build the brand so it won't rain money in the early couple of years. But like Simply [orange juice], when you do it well it rains money later."

Not all Coca-Cola brand adventures have been profitable though. In 2004 the firm was forced to take its Dasani bottled water off British shelf after just four weeks.

The firm was unable to convince British consumers to buy the drink, which was reported to be Sidcup tap water that had been filtered and pumped full of minerals. It was found to contain illegal levels of the chemical bromate.

Soda stream: Failed drinks

Surge

The idea was to knock Mountain Dew off the top spot in the lucrative extreme sports drinks market, but Surge was discontinued in 2002 after six years on sale.

Sprite Remix

This clear drink emerged in 2003 and quickly developed a strong fan base. Drinkers soon got bored with its sickly taste though.

OK Soda

Coca-Cola killed off OK Soda within seven months, in 1993. Marketing focused on the caffeinated drink's transparent appearance, ignoring its overly sweet taste.

Tab Clear

The soda did well enough to go global, but customers got bored and Coca-Cola pulled the drink by 1994.

LOAD-DATE: November 25, 2014

LANGUAGE: ENGLISH

PUBLICATION-TYPE: Newspaper; Web Publication

JOURNAL-CODE: WEBI

Copyright 2014 Independent Digital News and Media Limited
All Rights Reserved

MailOnline

November 25, 2014 Tuesday 5:03 PM GMT

Dairy you to try it! Coca-Cola plans to unveil new kind of MILK to boost profits as Americans cut down on sugary sodas

BYLINE: ALEXANDRA KLAUSNER FOR MAILONLINE

SECTION: NEWS

LENGTH: 551 words

- As Americans cut down on unhealthy **soft drinks**, Coca-Cola has turned to creating a premium type of milk called Fairlife to boost dwindling profits
- Less Americans are buying **sugary** sodas and more are turning towards protein products
- Fairlife will be available in stores in late December and boast 50 per cent more protein and 50 per cent less **sugar** than regular milk
- Fairlife will cost around double the amount of regular milk

Coca-Cola plans to unveil a classic beverage nationwide this December that is a far cry from the sugary soda that made the brand famous.

As Americans cut down on unhealthy soft drinks, Coca-Cola has turned to creating a premium type of milk to boost their dwindling profits.

Fairlife will be available in stores in late December and boasts 50 per cent more protein and 50 per cent less sugar than regular milk. It also contains 30 per cent more calcium.

The added nutritional benefits fatten the price tag and it will cost about double the amount of regular milk.

Fairlife is also lactose free.

'It's basically the premiumization of milk,' Sandy Douglas, a senior vice president at Coca-Cola's North American operation at the Morgan Stanley Global Consumer Conference last week.

The company has had success in advertising healthier beverages with their Simply juice line which has done well despite the fruit juice industry starting to slump.

Fruit juices are also packed with **sugars** and are a major contributor along with **soft drinks** to childhood obesity.

CBS reports that Coca-Cola advertises Simlpy juices as being healthier, unsweetened, and never frozen.

'Our ambition there is to create the Simply of milk,' Douglas said of the new product.

American shoppers have flocked towards higher protein products like Yogurt and less are buying items with high sugar contents.

'Protein is the fastest-growing segment of the beverage category,' Mike Saint John, president of Coca-Cola North America's Minute Maid business unit, told the Dairy Today.

Despite the growing popularity of high protein products, Dairy Today reports that one of every two adults doesn't drink milk at all.

Milk sales have also declined by 8 per cent over the last ten years.

What Coca-Cola aims to do is advertise how Fairlife is healthier than traditional milk because it's cold-filtered to concentrate protein and remove fat and sugar.

Coca-Cola already sells a protein shake called Core Power which goes through the same filtration process.

In order to build hype around the new milk, Coca-Cola has developed provocative ads which they launched earlier this year in Minnesota of women wearing dresses made from milk, reports Business Insider.

'Swing into something better,' says one advertisement picturing a woman in a dress made of milk hanging from a swing.

'Milk with flair,' reads another ad picturing a woman in a dress made from milk that resembles an iconic image of Marilyn Monroe in a white dress.

Despite the Milk industry being on the decline, Coca-Cola believes that their premium product equipped with a premium price tag will reel in the cash flow.

'Now to be clear, we're going to be investing in the milk business for a while to build the brand so it won't rain money in the early couple of years,' Douglas said at last week's conference.

'But like Simply, when you do it well, it rains money later.'

LOAD-DATE: November 25, 2014

LANGUAGE: ENGLISH

PUBLICATION-TYPE: Web Publication

JOURNAL-CODE: WEBDM

Copyright 2014 Associated Newspapers Ltd.
All Rights Reserved

The Telegraph
telegraph.co.uk

November 25, 2014 Tuesday 9:49 AM GMT

Coca-Cola to release its own brand of expensive milk; Soft drinks giant Coca-Cola is branching out into milk, and believes its low sugar version will 'rain money'

BYLINE: By Andrew Trotman

SECTION: FINANCE

LENGTH: 323 words

Coca-Cola is to release its own brand of milk that will become so popular it will "rain money", the drinks giant has said.

Fairlife, which will hit US shelves next month, will cost twice as much as standard milk currently sold in shops, the company admitted.

However, Coca-Cola's milk will contain higher protein, lower sugar and no lactose.

"Our vision for the nutrition beverage business and the milk product ... has a proprietary milk filtering process that allows you to increase protein by 50pc, take sugar down by 30pc, and have no lactose, and a milk that's premiumised and taste better and we'll charge twice as much for it as the milk we used to buying in a jug," Sandy Douglas, senior vice-president and global chief customer officer, told the Morgan Stanley Global Consumer Conference .

Mr Douglas said he expects Coca-Cola's milk to build popularity slowly but maintained that the Fairlife business had "tremendous growth potential".

"We're going to be investing in the milk business for a while to build the brand so it won't rain money in the early couple of years. But ... when you do it well it rains money later," he added.

Coca-Cola is entering the dairy market at a turnbulent time. Sales of conventional milk are falling in the US, while revenues of flavoured and higher-protein milk are climbing.

Dean Foods, the largest milk processor in the US, has closed plants and cut its full-year adjusted profit forecast for this year.

Milk prices have been rising in the US since 2008, when China started sourcing foreign-made milk powder and infant formula after six babies died and more than 300,000 children fell ill due to local milk products contaminated with melamine.

Dean Foods said raw milk prices rose 22pc in the first quarter ended March 31. Prices continue to rise, increasing 4pc between March and May, the company said.

US milk production rose just 1pc between January and March, Department of Agriculture said.

LOAD-DATE: November 25, 2014

LANGUAGE: ENGLISH

PUBLICATION-TYPE: Newspaper; Web Publication

JOURNAL-CODE: WEBDTNS

Copyright 2014 Telegraph Media Group Limited
All Rights Reserved

THE SUNDAY TIMES
The Sunday Times (London)

November 23, 2014 Sunday
Edition 1;
Northern Ireland

I choke on the words but our bulging world needs laws to curb Big Food

BYLINE: CAMILLA CAVENDISH

SECTION: NEWS; OPINION; COLUMNS; Pg. 27

LENGTH: 1182 words

Thirty per cent of the world's population is overweight or obese, according to a new study. The implications are, well, massive. Who would have thought prosperity would prove so selfdefeating? The study, by McKinsey (where I used to work), says flab is costing us as much as smoking or war, because of an explosion of ill health. Today's children could be the first generation to die earlier than their parents, from diseases such as type 2 diabetes.

Aside from the militants of the "fat pride" community, none of us actually wants to be fat. So why are we so powerless to control ourselves? Our brains are still wired for primeval scarcity, not modern abundance. At least mine is. I have been quite capable of eating an entire tub of chocolate ice cream and pretending I haven't (until my husband has found the empty tub in the bin).

McKinsey's report is the first robust, global analysis to bust the myth that willpower and education will somehow win the battle against the bulge. It examines 74 policies in different countries, ranging from cycling campaigns to food taxes. And it finds, essentially, that humans are not good at digesting health advice. Even people who shed pounds through diet programmes or surgery often pile it on again later.

My favourite demonstration of food delusion is a series of experiments led by Brian Wansink, a professor at Cornell University. He invited students to eat tomato soup, and kept surreptitiously refilling their bowls through holes drilled in the table. Unaware, they happily slurped down vast quantities. They judged whether they were full by looking at how much was left in the bowl, rather than what their stomachs were telling them.

In another Wansink experiment, he gave stale popcorn to filmgoers in Chicago. Some of the popcorn was in big buckets and some in buckets so giant that it was assumed no one could finish it. At the end of the film, those with the giant buckets had eaten 50% more than those with big buckets. Confronted with this ugly truth, the scoffers mostly denied they had eaten so much.

What Wansink calls "mindless eating" has nothing to do with hunger. It is driven much more by social cues: the size of the portion and what others around us are doing.

So it is telling that McKinsey concludes that the single most effective policy to combat world obesity would be to reduce portion sizes in packaged foods, fast-food restaurants and canteens. That's one-up to Wansink. But industry doesn't like it. When the mayor of New York tried to ban jumbosize **sugary drinks** three years ago- a liquid version of the giant popcorn buckets - American **soft-drinks** companies took him to court after court until they defeated him.

Smaller portions, McKinsey says, are just step one. You also need to change the ingredients in processed and junk food. Whoa. This is dynamite. A respected consulting firm with multinational clients is saying that we consumers are incapable of resisting Big Food, just as we got trapped by Big Tobacco. And that the only way to beat Big Food, as with Big Tobacco, is to legislate.

McKinsey doesn't put it quite like that, of course. It praises voluntary partnerships, especially the one the British government has been building. My concern with voluntary partnerships is this. Whenever my children escape from the muesli gulag that is our home and stay the night somewhere that offers Coco Pops for breakfast, they will gorge on Coco Pops until the box is empty. Voluntary pledges have made no discernible difference to Coco Pops.

How could they? What company willingly loses money by making healthier food? Waitrose and Marks & Spencer have acted responsibly by removing trans fats from own-brand products, but their upmarket customers appreciate that. The big American manufacturers, petrol forecourts and fast-food outlets would simply lose competitive advantage if any one of them alone made its food less tasty or used more expensive ingredients.

The food and drink industry has grown rich on the profits from degraded food: the high-fructose corn syrup that lurks in **fizzy drinks**; the trans fats that have been declared a toxin by the World Health Organisation. To protect its profits, the industry uses similar tactics to the old tobacco firms. It argues that the health effects are unproven - and sponsors research to bolster that claim. It suggests there is no such thing as bad food; only bad diet and not enough exercise. It says governments should not tell people what to eat.

That final point is a strong one. But governments have a duty to protect people, and this problem is now overwhelming. The food industry has tried to assuage health concerns by offering "low-fat" options, rather as tobacco firms offered "low-tar" cigarettes.

But "low-fat" processed foods are a con. Most contain refined carbohydrates and sugar, which create spikes in blood sugar levels and addictive cravings. Doctors and researchers such as the endocrinologist Robert Lustig are now arguing that sugar switches on the same hormonal pathways as nicotine. That is probably why people can't wean themselves off it.

It's not all the fault of companies: dieticians have sown confusion at every turn. Apple juice was one of our five a day; now it's evil incarnate. Margarine was better than butter; now it's the other way round. Food companies can exploit this confusion to go on pushing the same old stuff. "There, there," they say. "Everything in moderation." But we humans are lousy at moderation. So is the industry. Try to buy a newspaper at the station, as David Cameron once memorably said, and "as you queue to pay, you're surrounded by cut-price offers for giant chocolate bars". Mr Cameron was in opposition then: he now has other things on his plate. But his government is so worried about obesity that one minister is urging gyms to get rid of mirrors. Rather than tackling "Lycra shame", surely a hopeless task, it would be better to tackle what goes into our food.

Ed Miliband, the Labour leader, has said little about fast food, despite his attacks on "predatory" capitalists. Perhaps he fears that if you take on the pushers, you take on the addicts too, including the parents who are indignant about schools getting rid of vending machines. But this only makes it more important to break the cycle.

I've met thoughtful people in the food industry who would welcome a chance to make food healthier, but only if there were a level playing field. They need regulations that ban international competitors from using certain ingredients - as Denmark has done with trans fats - or that cap the energy content in foods.

The story that convinced me legislation is needed came from someone who has followed shoppers round supermarkets. Far from using the new "traffic light" labels to choose the healthy option, people are actively seeking out pizzas with red labels, assuming they will taste better.

So Big Food wins again; and we need protection from ourselves.

camilla.cavendish@sunday-times.co.uk

WHEN MY CHILDREN ESCAPE FROM THE MUESLI GULAG THAT IS OUR HOME AND STAY SOMEWHERE THAT OFFERS COCO POPS, THEY WILL GORGE ON THEM

LOAD-DATE: November 23, 2014

LANGUAGE: ENGLISH

PUBLICATION-TYPE: Newspaper

JOURNAL-CODE: STS

Copyright 2014 Times Newspapers Limited
All Rights Reserved

MailOnline

MailOnline

November 21, 2014 Friday 10:32 PM GMT

Navajo Nation president approves junk-food tax for tribe where obesity rates have reached 60 per cent

BYLINE: ASSOCIATED PRESS

SECTION: NEWS

LENGTH: 478 words

- Navajo Nation President Ben Shelly signed legislation to increase by 2 per cent the sales tax on food with little to no nutritional value next year
- The obesity rate for some Navajo age groups is as high as 60 percent

The sales tax on cookies, chips, sodas and other junk food sold on the country's largest American Indian reservation is going up.

Navajo Nation President Ben Shelly signed legislation on Friday to increase by 2 per cent the sales tax on food with little to no nutritional value, starting next year.

No other sales tax on the Navajo Nation specifically targets the spending habits of consumers. It will remain in effect until 2020, but it can be extended by the Navajo Nation Council.

Navajos advocating for a junk-food tax said they wanted to pass a bill that could serve as a model for Indian Country to improve the rates of diabetes and obesity among tribal members.

Proposals targeting **sugary drinks** with proposed bans, size limits, tax hikes and warning labels haven't gained widespread traction across the country.

'We want them to think twice about buying healthy foods instead of soda pop, potato chips and the junk food,' said Gloria Begay of the Dine Community Advocacy Alliance.

'The effort is really much more in the message of Navajo people making better choices for quality foods.'

The bill cited statistics from the Navajo-area Indian Health Service that said about one-third of Navajos are diabetic or pre-diabetic, and the obesity rate for some age groups is as high as 60 percent.

Diabetes was the fourth-leading cause of death in the Navajo area from 2003 to 2005, the health service said.

The $1 million-a-year that the additional tax is expected to generate will pay for projects including farmer's markets, vegetable gardens and wellness and exercise equipment in the tribe's 110 communities.

Begay said her group is meeting with tribal officials to figure out exactly how the money will be disbursed.

Another bill to eliminate the tribe's 5 percent sales tax on fresh fruit and vegetables sold on the Navajo Nation went into effect October 1.

Shelly vetoed another version of the junk food tax earlier this year. His spokesman, Deswood Tome, said Friday that the latest version is clearer on how it will be implemented.

Representatives of the beverage industry had lobbied the tribe to reject the tax, saying it would create problems for retailers and doesn't solve health problems. It also applies to sports drinks, fruit juice and pita chips.

The tax won't add significantly to the price of junk food, but buying food on the reservation presents obstacles that don't exist in most of urban America.

The reservation is a vast 27,000 square miles with few grocery stores and a population with an unemployment rate of around 50 percent.

Thousands of people live without electricity and have no way of storing perishable food items for too long.

LOAD-DATE: November 21, 2014

LANGUAGE: ENGLISH

PUBLICATION-TYPE: Web Publication

JOURNAL-CODE: WEBDM

THE TIMES

The Times (London)

November 18, 2014 Tuesday
Edition 1;
Scotland

Hospitals obliged by law to serve good food; 'Raise standard of NHS food'

BYLINE: Hamish Macdonell

SECTION: NEWS; Pg. 1,4

LENGTH: 522 words

Hospitals in Scotland could become the first in the UK to be legally obliged to serve nutritional meals to patients.

Under plans being considered by the Scottish government, NHS boards would be placed under a statutory duty to provide nutritious food.

Any hospitals which failed to reach acceptable standards would be forced to make improvments or face penalties. In extreme cases, "hit squads" of nutrition specialists could be sent in to ensure patients are fed properly.

A consultation on the plans was announced yesterday as part of a package of measures aimed at improving hospital food standards after reports that some patients were being fed for as little as 89p per day.

The British Medical Association (BMA) welcomed the announcement and said a "significant" number of patients admitted to Scottish hospitals are undernourished and need the right foods to aid their recovery.

They also urged health boards to go further and stop patients having easy access to sweets, **fizzy drinks** and crisps being sold from hospital trolleys.

Ministers want to model the new regime for hospital food on the existing practice in Scotland's schools, which are required by law to provide nutritious meals for pupils. The government has the power to send in expert teams to any schools failing to meet the standard expected.

Alex Neil, the health secretary, also promised £300,000 for health boards to improve meals and pledged more regular and frequent inspections.

"This is a package of measures that will help to ensure that hospital food continues to improve, that NHS boards have the appropriate amount of support and advice that they need to deliver these improvements and that we have an even stronger inspection process," Mr Neil said.

"We already have rigorous standards in place, and clear guidance about how these standards can be met. Catering teams, dieticians, specialist consultants and nursing staff are doing great work to ensure that patients are getting nutritious and balanced diets in hospital. "But I recognise there is always more that can be done to drive up standards."

Scotland was the first part of the UK to introduce national standards for food, fluid and nutrition in hospitals. Ministers also claim that, since 2008, more than £1.75 million has been invested to improve those standards.

Jackson Carlaw, the Scottish Conservative health spokesman, said: "Any indication that the Scottish government is willing to improve the standard of hospital meals is welcome.

"It's inconceivable that having top quality hospital food should be anything other than a priority."

Alison Johnstone, a Green MSP, said: "We should take this opportunity to focus on using more local and fresh ingredients, and we should continually involve patients in a review of menus."

The BMA said that there should be more focus on what is sold to patients in hospital and their relatives. Dr Lewis Morrison, a member of the BMA's Scottish Council, said: "Hospital corridors are littered with vending

machines selling high-sugar, high-fat food and drink to patients, visitors and staff. These unhealthy foods are supplementing the poor quality of hospital food."

LOAD-DATE: November 18, 2014

LANGUAGE: ENGLISH

PUBLICATION-TYPE: Newspaper

JOURNAL-CODE: TIMscot

Copyright 2014 Times Newspapers Limited
All Rights Reserved

MailOnline

November 17, 2014 Monday 2:10 PM GMT

'Fizzy drinks are the new smoking': All cans and bottles should carry labels warning that sugar causes obesity, diabetes and tooth decay, politician claims

BYLINE: LIZZIE PARRY FOR MAILONLINE

SECTION: HEALTH

LENGTH: 1232 words

- New York politician Karim Camara has introduced a bill that will require health warning labels on certain drinks with added sugar
- He said the size of the warning should be dicatated by the size of the drink
- They should read: 'DRINKING **BEVERAGES** WITH ADDED **SUGAR** CONTRIBUTES TO OBESITY, DIABETES AND TOOTH DECAY'

Experts have called for sugar-laden **fizzy drinks** to carry warning labels similar to those found on cigarette packets, in a bid to combat the adverse health effects.

New York assemblyman Karim Camara has introduced a bill that will require health warning labels on certain drinks with added sugar.

It comes after the World Health Organisation recommended slashing a person's daily intake of added sugar from 50g to 25g - or from 10 to five cubes a day.

A regular can of Coke contains 35g of sugar, so that alone would exceed this limit.

The size of the warning, said Mr Camara, should be dictated by the size of the drink.

'We can't sit back and pretend that **sugary drinks** aren't harmful to people,' he said.

'We have a public health crisis, which is a direct result of people consuming too much sugar.

'The research is clear: too much sugar leads to health problems such as obesity and diabetes.

'As a society, we have a moral obligation to educate people so they can make healthier choices.'

The Brooklyn democrat said the labels should read: 'SAFETY WARNING: DRINKING **BEVERAGES** WITH ADDED **SUGAR** CONTRIBUTES TO OBESITY, DIABETES AND TOOTH DECAY.'

Mr Camara was joined on the steps of City Hall by representatives from the American Heart Association, the American Diabetes Association and the Center for Science in the Public Interest.

Past research has found **sugary drinks** are a primary contributor to type 2 diabetes, especially in children.

In August, the Centers for Disease Control and Prevention (CDC) reported that 40 per cent of children born between 2000 and 2011 would develop type 2 diabetes in their lifetime.

Experts at the CDC estimate 35 per cent of Americans are obese, and 69 per cent are overweight.

Obesity is known to cause other serious illnesses, including cancer, heart disease and stroke.

Mr Camara said the aim of introducing warning labels on drinks is to educate the public, and lower the consumption of the drinks.

He likened their goal to that of health campaigners who fought to include warning labels on cigarette packages.

'This is about pragmatic legislation,' he said. 'This is not about an attack on an industry for the sake of the industry.

'This is about doing what's best for children and adults in our society.

'We cannot afford for our children to continue to be overweight, to continue to have learning problems, to have health problems that may lead to difficult lives and at times illnesses that can lead to the loss of life.'

Mr Camara urged the beverage industry to support the bill after they spent $13million in New York State in 2010 to oppose a proposed penny-per-ounce **sugary drink** tax, arguing that education is what was really needed.

Assemblyman Jeffrery Dinowitz, consumer affairs and protection chair, said: 'Studies have established a direct link between consumption of **sugar**-sweetened **beverages** and rising rates of obesity, diabetes, and other major health problems in New York and across the United States, which cause thousands of deaths and cost billions of dollars in health care spending annually.

'We must do everything we can to make the public aware of the serious risks incurred by the over-consumption of sugar.

'Many years ago, when faced with a similarly devastating public health crisis, we chose to place warning labels on cigarettes and other tobacco products to allow citizens to make more informed choices about what they purchase.

'Given the scientific evidence of sugar's negative consequences for our society, is not only appropriate, but incumbent, upon us to use similar warning labels with **sugar**-sweetened **beverages**.'

Nutrition policy consultant said warning labels are a 'smart way to educate consumers about the negative health impacts' of their favourite **fizzy drinks**.

She said: 'Right now, the beverage industry bombards our kids with its own messages encouraging constant consumption.

'In 2010, for example, preschoolers viewed an average of 213 ads for **sugary drinks** and energy drinks, while children and teens watched an average of 277 and 406 ads, respectively.'

Stephen Habbe, from the American Diabetes Association, said the type 2 epidemic in the U.S. has been 'in part, fuelled by the increased consumption of calorie-dense **sugar**-sweetened **beverages'**.

He said nearly 30 million children and adults in the U.S. have diabetes, with more than 1.3million in New York diagnosed with the condition.

Another 86 million Americans have pre-diabetes, putting them at risk of developing type 2 diabetes later in life.

Mr Habbe said: 'Diabetes kills tens of thousands of Americans every year and results in disabling complications for thousands of others.

SO HOW MUCH SUGAR SHOULD WE BE EATING?

Earlier this year, the World Health Organisation published draft guidelines urging adults to eat no more than 12 teaspoons of sugar a day and to aim for six.

The guideline amount slashed was amid fears sugar poses same threat as tobacco.

The number of obese British adults is expected to double from one in four to one in two by 2050 - at a cost to the economy of £50billion a year.

The WHO said the crisis was being fuelled by hidden sugar in processed food and drink such as yogurts, muesli, sauces, **fizzy drinks**, juice and smoothies.

However, low-fat foods have also come under fire from experts, after it was revealed they often contain more sugar than the full-fat alternative.

WHO also said children should try for less than six teaspoons and avoid cans of **fizzy drink** such as Coke, which contains seven spoons.

Instead five or six teaspoons is the ideal figure that people should aim for.

'The American Diabetes Association supports reducing the consumption of **sugar**-sweetened **beverages** through public information, education, and health policy, and applauds Assemblyman Camara for his interest in warning labels for **sugar**-sweetened **beverages**.'

And the American Heart Association echoed Mr Habbe's backing of the bill.

Robin Vitale said: 'There is no doubt that consumption of **sugary drinks** has increased dramatically in recent decades.

'It is important for our government leaders to work with the public health community to identify, test and implement effective evidence-based strategies to help reverse this dangerous trend.

'The American Heart Association supports a comprehensive, multi-pronged approach to combatting obesity which is a leading cause of heart disease and stroke.

'We are very interested in the proposed legislation by Assembly Member Camara as part of a broad range of public policy approaches addressing the consumption of **sugary drinks**.'

The American **Beverage** Association told the New York Observer, warning labels will not change consumer behaviour.

Spokesman Christopher Grindlesperger, said: 'Obesity is a serious and complex issue, but a misleading warning label on certain beverages will not change behaviors or teach people about healthy lifestyles.

'There's a better way to empower consumers to make the choices that are right for them.

'If we want to get serious about obesity, it starts with education - not laws and regulation.'

LOAD-DATE: November 17, 2014

LANGUAGE: ENGLISH

PUBLICATION-TYPE: Web Publication

JOURNAL-CODE: WEBDM

Copyright 2014 Associated Newspapers Ltd.
All Rights Reserved

The Telegraph
telegraph.co.uk

November 17, 2014 Monday 8:02 PM GMT

America's sugar addiction: Just how bad is it?; As the United States' first tax on sugary drinks goes into effect in California, here's a look at the facts and figures for America's love affair with sugar

BYLINE: By David Lawler Jack Gurdon, Washington

SECTION: NEWS

LENGTH: 631 words

The numbers are stark:

· More than one-third of Americans are currently obese, including 17 per cent of children.

· Type 2 diabetes diagnoses have increased tenfold in the last thirty years.

· America now spends over $190bn (£121bn) annually treating diseases which are tied directly to obesity.

While a number of factors have contributed to these worrying trends, health advocates and policymakers are now focusing on one culprit: sugar.

Berkeley, California recently became the first US city to enact a tax on sugary drinks, while larger cities such as New York and San Francisco have debated similar measures.

Americans don't like the government dictating their diets, and the US surely won't come down from its sugar high overnight, but there are significant costs associated with America's addiction to sugar.

And Britain isn't far behind.

Fizzy drink fanatics...

Americans consume more fizzy drinks per capita than any other country on earth, and super-size sodas are contributing to America's equally enlarged rates of diabetes and other diseases.

· A typical American child has, on average, 8 ounces of fizzy drinks per day. Those rates increase significantly among adolescents. Having one sugar sweetened beverage per day increases an individual's risk of both diabetes and heart disease by upwards of 25 per cent.

· The average American purchases 170 litres of fizzy drinks per year, more than twice as much as a typical Brit.

· Drinking more sugar calories does not significantly reduce sugar consumption elsewhere in the diet. Sugar dissolved into fizzy drinks reduces an individual's appetite only 1/7 as much as solid sugar.

Whole wheat toast, hold the sugar...

It's not just **fizzy drinks** that have led to skyrocketing sugar consumption rates.

· Beginning in the 1970's America waged a "war on fat", which led to food producers cutting fat content and - you guessed it- sprinkling in some sugar.

· From whole wheat bread to soy milk and nearly everything in between, over 80 per cent of American processed foods now contain added sugar.

· According to the American Journal of Clinical Nutrition, a typical American now consumes roughly 120lbs of sugar per year. That's approximately 50 per cent more than in 1950.

Photo:Alamy

A costly habit...

· The obesity rate in America has more than doubled since the 1980's amongst adults, and tripled amongst children.

· Seven per cent of Americans now have some form of diabetes, while an American is ten times more likely today to be diagnosed with type 2 diabetes than ten years ago.

· According to one estimate, America now spends approximately $190 million (£121million) annually to treat diseases directly linked with obesity. That's more than the entire gross domestic product of New Zealand.

And Britain's addicted too...

· Two-thirds of Brits are considered overweight, with one in four classified as obese. Among Western European countries, only Iceland and Malta have higher proportions of overweight individuals.

· One in five children aged 4-5 and one in three children aged 10-11 are overweight or obese

· British teens get 15.6 per cent of their food energy from sugar, that's three times the recommended amount.

Photo:PA

Despite these staggering statistics, we continue to consume more sugar than ever.

At this point, nutrition activists like Michelle Obama will try just about anything to convince kids to choose vegetables over sugary snacks.

So far it doesn't seem to be working, but if Berkeley's "soda tax" experiment catches on in the US, it may just come to Britain next.

LOAD-DATE: November 17, 2014

LANGUAGE: ENGLISH

PUBLICATION-TYPE: Newspaper; Web Publication

JOURNAL-CODE: WEBDTNS

Copyright 2014 Telegraph Media Group Limited
All Rights Reserved

The Sunday Telegraph
The Sunday Telegraph (London)

November 16, 2014
Edition 1;
National Edition

Time to curb the American sugar rush; Californian vote to tax sugary drinks may be start of a trend to fight the nation fat

BYLINE: PETER FOSTER

SECTION: NEWS; Pg. 29

LENGTH: 938 words

THE REPUBLICAN recapture of the Senate hogged all the headlines at this month's midterm elections, even though in an era of perpetual gridlock in Washington the change was unlikely to make much practical difference to the lives of ordinary Americans.

There was, however, another little-reported vote on Nov 5 which really could have profound consequences for the future of the US. It took place in the über-liberal enclave of Berkeley, California (pop 80,000), which agreed to impose a penny-an-ounce tax on **sugary drinks**.

At first blush, that hardly sounds like a social earthquake, especially when you consider that in recent years at least 30 similar ballot initiatives have been rejected by US voters. Indeed, on that same day, just across the water in San Francisco, voters declined to impose a so-called "soda tax" of their own. However, this was only because it didn't get the required two-thirds majority - 55 per cent voted in favour, a hefty proportion.

Like many pundits, the American Beverage Association which represents the $76 billion-a-year (£48.5 billion) soft-drink industry rushed to dismiss the Berkeley vote. "Berkeley is very eclectic," said a spokesman airily, "It doesn't look like Anytown USA."

That is certainly true, but what the "Big Soda" lobbyist is not telling - and well knows - is that Berkeley has an uncanny track record of predicting the future when it comes to social trends.

Many things that seemed outlandish at the time, but later became mainstream, started in Berkeley: from pavement recycling and banning plastic bags to granting rights for same-sex couples and outlawing smoking indoors, Berkeley was 20 years ahead of its time.

That's why public health experts such as Yale University's Kelly Brownell, who have been warning for 20 years of the need to come up with ways to curb America's sugar habit, believe the vote could signal momentous change.

"Berkeley is much more of a forecast than an outlier," he says, predicting that Berkeley will again set a trend. "Once some place goes first, it's a lot easier for other places to go second, third and fourth."

Time will tell, but it is perhaps an indication of how deeply the "Big Food" lobby fears a public change of heart on sugar that it spent a combined $11million fighting the soda tax referendums.

And that change is surely coming. Those numbers would suggest that America is waking up to the cost of a sugar addiction that has seen childhood obesity rates triple in a generation and precipitated an epidemic of Type 2 diabetes that now afflicts more than 20million Americans, compared with twomillion in 1973.

The social costs are huge and so are the financial ones. Depending on where you draw the line, the US spends around $190 billion a year on additional medical costs caused by obesity, about 20 per cent of its total health care costs.

It will not be long before a growing number of people demand that the food industry picks up the tab for at least some of the damage caused by the products that, in all senses, are putting a massive strain on America.

The common argument against the nanny state - "getting fat is just a matter of personal choice" - won't wash, just as it didn't for tobacco or laws that mandated the wearing of seatbelts. The cost to overall public good is simply too high to ignore.

America cannot afford to get fatter. Conservatives love liberty, but they also hate the spiralling cost of Medicaid, the government health insurance programme for those on low incomes, which disproportionately picks up the costs of the obesity epidemic. And right now, obesity rates are predicted to double in the US by 2030.

This won't happen by chance. Lifestyles are more sedentary now, but that fact on its own cannot explain why childhood obesity rates have shot up so fast. Only the relentless pushing of sugar to children by food companies explains that.

Go to a child's sporting event and it is astonishing how many children come waddling off the pitch and immediately guzzle a bottle of Gatorade or similar. And even worse, their parents seem oblivious to the self-destructive folly of it all.

That is why, far-fetched as it seems today, it is also possible to envisage 20 years from now restrictions being placed on the way sweets and chocolates are advertised to children, just as tobacco adverts are banned today.

Because in truth there is no escape from the marketing machine: one recent US study of walk-to-school children found they were healthier than those who were driven there, but they were also fatter.

Baffled scientists only explained that paradox when they discovered that those who walked went past the local store where they picked up absurdly cheap drinks and sweets en route.

Sugary drinks are particularly pernicious since you can consume them without spoiling your appetite. One study found that a **fizzy drink** consumed before a meal reduced a child's appetite by just 9 per cent, compared with 63 per cent if the equivalent sugar was eaten in a solid form.

High fructose corn syrup and alcohol are metabolised in the liver. This is why children who drink a lot of **fizzy drinks** and are obese are at risk of fatty liver disease just like alcoholics are. The fact is, America did not get fat by accident, but by the multibillion dollar design of an industry that until now has done everything to avoid paying its fair share of the costs imposed on society by its products.

How long before the public and government decide the polluter must pay? If the Berkeley precedent holds, we could look back on Nov 5, 2014 as the moment when, on the contentious issue of sugar taxes, the fat lady finally started to sing.

LOAD-DATE: November 16, 2014

LANGUAGE: ENGLISH

PUBLICATION-TYPE: Newspaper

JOURNAL-CODE: STL

Copyright 2014 Telegraph Media Group Limited

Mirror

mirror.co.uk

November 15, 2014 Saturday 5:57 PM GMT

Have a flabby Christmas! Health experts warn festive foods are packed with hidden fat and sugar; Regular Christmas sandwiches can contain up to 7 teaspoons of sugar - while some hot drinks have even more

BYLINE: By Stephen Hayward

SECTION: NEWS,UK NEWS

LENGTH: 346 words

Christmas bad food

Seasonal drinks and snacks have been launched by high street chains in the run-up to Christmas - and they're packed with fat and sugar.

Regular-sized Christmas drinks sold by Costa Coffee and Pret a Manger contain more than 10 teaspoons of sugar - half the recommended daily intake.

Other festive treats are Eat's Merry Berry Mocha with 28.9g of sugar - equivalent to seven teaspoons - while its Festive Full Works Bloomer contains 583 calories and 15.7g of sugar.

The recommended daily sugar intake is 90g. Experts advise women not to exceed 2,000 calories a day and men to limit themselves to 2,500, while the recommended daily saturated fat allowance is 20g.

Pret's Christmas lunch sandwich packs in 540 calories and 11.1g of sugar while its orange spiced hot chocolate has 42.4g of sugar - over 10 teaspoons. Another belly buster is a small Costa Coffee's Sticky Toffee Latte with 11.9g of saturated fat and 40.7g of sugar.

Critics believe high **sugar drinks** are fuelling obesity - and tooth decay is the most common reason for children in England being admitted to hospital.

All the chains publish nutritional contents on their websites. But health experts believe the information should be clearly displayed in branches too.

The British Dietetic Association said: "If the information was clearly visible in shops, it might make people think twice about ordering one of these drinks."

Its spokesman Azmina Govindji said: "You might not expect sugar from a sandwich, but a Christmas sandwich could clock up over two teaspoons - most of it probably coming from the cranberry sauce."

Costa said: "While some customers will want to indulge in a Costa festive treat, we also provide customers with the option of selecting lower-calorie drinks from our standard range or the option to customise their drinks if they choose."

Eat said: "As well as the Festive Full works Bloomer we also serve healthier and lighter options from our Christmas range; such as our Turkey and Cranberry hinge which contains less that 5% fat."

Will you be eating healthy this Christmas?

LOAD-DATE: November 16, 2014

LANGUAGE: ENGLISH

PUBLICATION-TYPE: Newspaper; Web Publication

JOURNAL-CODE: WEBDMI

Copyright 2014 Trinity Mirror, Plc.
All Rights Reserved

The Observer

The Observer

November 15, 2014 Saturday 1:55 PM GMT

How Berkeley took on the might of Big Soda and won; Californian city known for its liberal causes defeated powerful opposition over its campaign

BYLINE: Andrew Gumbel

SECTION: US NEWS

LENGTH: 999 words

In Berkeley, America's unofficial capital of progressive politics, they're calling it the battle against Big Soda.

A coalition of public health advocates, educators, environmentalists and local politicians believes that the time has come to push back against the great foaming tide of Coca-Cola, Pepsi and other sugar-clogged **fizzy drinks** that have contributed to an epidemic of childhood obesity and Type 2 diabetes.

It's a nationwide movement, inspired by Michelle Obama's campaigns to get underprivileged children to eat fresh food and by Michael Bloomberg, the former mayor of New York, who tried and failed to ban supersized sodas within the five boroughs.

However, in Berkeley - home to the University of California's flagship campus, birthplace of the 1960s counterculture and crucible of the organic food movement - the campaigners have just notched up their first clear victory. Almost lost in the Republican sweep in this month's midterm elections was a referendum in Berkeley in which a proposed tax of one cent for every ounce of soda won 75% voter approval.

That may seem unsurprising in a small city with an affluent, well-educated, liberal electorate where soda is widely dismissed as cheap and nasty. But 31 other cities have tried to pass a similar tax in the past - including the neighbouring city of Richmond and Berkeley's big brother across the bay, San Francisco - and

31 times the initiative has been defeated by a beverage industry willing to sink more than $100m into opposition campaigning.

The American Beverage Association spent $2.4m (£1.53m) - $30 per eligible voter - in Berkeley on TV ads, fliers and "push polls", in which voters were ostensibly asked about their preferences but in fact made to think about whether the tax wasn't a restriction on personal freedom imposed by unaccountable bureaucrats.

In the past, similar strategies have been sufficient to turn just enough voters, especially those at the lower end of the income scale, who tend to be the biggest soda drinkers- Coke being significantly cheaper than, say, organic carrot juice. Berkeley, however, had a particularly determined local coalition and enjoyed support from Bloomberg's private foundation, which pumped hundreds of thousands of dollars into TV ads aired during the World Series, the climax of the baseball season, which happened to be won by the San Francisco Giants.

The campaigners hope that Berkeley will now set the national standard - as it has in the past with restricting smoking in bars and restaurants and creating city pavements with easy access for wheelchairs. "We fully expect other communities to take on the soda industry and succeed," the campaign's co-chair, Vicki Alexander, said on election night.

That optimism is supported by a recent poll showing more than two-thirds of Californians supporting a modest soda tax. A 2012 study led by researchers from the San Francisco campus of the University of California found that a nationwide tax of one cent per ounce of soda could prevent 240,000 cases of diabetes per year and save 26,000 lives over a decade.

The soda tax campaigners even found qualified good news out of San Francisco, whose own measure on this month's ballot - twice as big, at two cents per ounce - won a solid majority (55%). It failed only because of a strategic decision by the measure's backers to specify how they intended to spend the revenue on public health, which in turn necessitated a two-thirds super-majority under California's voting rules.

The beverage industry sees Berkeley as a political freak of nature and does not expect other municipalities to follow suit. "They say this is the first domino, but as far as I'm concerned it's the only domino - the last domino," said Roger Salazar, spokesman for the "no" campaign. "These types of taxes are ones that historically and predominantly have been rejected by voters. We don't see that changing."

It remains to be seen how much difference the Berkeley vote will actually make. Since it is levied on distributors, not retailers, Coca-Cola and the other drinks giants may simply choose to absorb the cost themselves. There are questions about collecting the tax efficiently, about a possible exemption for the University of California campus - which is owned by the state - and other logistical problems. Some researchers and food writers say that the underlying problem will never be solved as long as the prices of fruit and vegetables rise faster than those of processed foods - an imbalance caused principally by government subsidies for big agribusiness interests whose cheap corn syrup is a major source of the sugar in Coke, Pepsi and thousands of other common grocery-store products.

However, the soda tax campaigners and Bloomberg say they are happy even if the tax is strictly symbolic because the key battle is over consumer habits, not price tags.

Howard Wolfson, Bloomberg's top political strategist, said: "There's a greater understanding among voters and consumers that soft drinks are bad for you in a way that wasn't true 10 or even five years ago."

For Kent Sims, a Bay Area economist who has worked closely with the food industry, the tobacco industry's drastic change in fortune shows what might happen. "If you smoke in California now, you're a pariah," he said. "People look at you like you're some kind of scumbag."The goal here is to have sugary sodas permanently tagged as bad, bad, bad, just like cigarettes. We've already taken the first step. San Francisco is likely to put the issue back on the ballot next year or in 2016 at the latest - this time with no specific direction for the tax revenues, so that a simple majority would suffice.

Larry Tramutola, a consultant to the Measure D campaign, said other municipalities are likely to follow suit because the publicity surrounding the initiatives alone makes them worthwhile. "We touched a broad, broad population about the impact of **sugary drinks**. Win or lose, it was a big educational campaign. That's going to continue."

LOAD-DATE: November 15, 2014

LANGUAGE: ENGLISH

PUBLICATION-TYPE: Newspaper

JOURNAL-CODE: WEBONS

Copyright 2014 Guardian Newspapers Limited
All Rights Reserved

The Telegraph
telegraph.co.uk

November 15, 2014 Saturday 2:01 PM GMT

American Way: It is time to curb the great American sugar rush; As the costs of America's obesity epidemic continues to soar, a vote to tax fizzy drinks could herald a change in attitudes to a nation's sugar addiction, writes Peter Foster in Washington

BYLINE: By Peter Foster Washington

SECTION: NEWS

LENGTH: 1011 words

The Republican recapture of the US Senate hogged all the headlines at this month's midterm elections, even though in an era of perpetual gridlock in Washington the change was unlikely to make much practical difference to the lives of ordinary Americans.

There was, however, another little-reported vote on November 5 which really could have profound consequences for the future of America. It took place in the uber-liberal enclave of Berkeley, California (pop 80,000) which agreed overwhelmingly to impose a penny-an-ounce tax on **sugary drinks**.

At first blush, that hardly sounds like a social earthquake, especially when you consider that in recent years at least 30 similar ballot initiatives have been rejected by US voters. Indeed on that same day, just across the water in San Francisco, voters declined to impose a so-called "soda tax" of their own.

Like many pundits, the American Beverage Association which represents the $76 billion-a-year (£48.5bn) soft-drink industry rushed to dismiss the Berkeley vote.

"Berkeley is very eclectic," said a spokesman airily, "It doesn't look like Anytown USA."

That is certainly true, but what the 'Big Soda' lobbyist is not telling - and well knows - is that Berkeley has an uncanny track record of predicting the future when it comes to social trends.

Many things that seemed outlandish at the time, but later became mainstream, started in Berkeley: from pavement recycling and banning plastic bags, to granting rights for same-sex couples and outlawing smoking indoors, Berkeley was 20 years ahead of its time.

That's why public health experts like Yale University's Kelly Brownell who have been warning for 20 years of the need to come up with ways to curb America's sugar habit, believe the vote could signal momentous change.

"Berkeley is much more of a forecast than an outlier," he says, predicting that as in the past, Berkeley will again set a trend. "Once someplace goes first, it's a lot easier for other places to go second, third and fourth."

Time will tell, but it is perhaps an indication of how deeply the "Big Food" lobby fears a public change of heart on sugar, that it spent a combined $11m fighting the soda tax referendum in San Francisco and Berkeley.

And that change is surely coming. It is instructive that although the San Francisco measure failed to pass, 55 per cent of residents still voted in favour of a tax despite all those campaign dollars - not the required two-thirds majority needed to pass the motion, but a hefty proportion nonetheless.

Those numbers would suggest that America is waking up to the cost of a sugar addiction that has seen childhood obesity rates triple in a generation and precipitated an epidemic of Type 2 diabetes that now afflicts more than 20 million Americans - compared with just two million in 1973.

The social costs are huge and so are the financial ones. Depending on where you draw the line, America spends around $190bn a year on additional medical costs caused by obesity, or about 20 per cent of its total healthcare costs.

It will not be long before a growing number of people demand that the food industry - like the tobacco industry before it - picks up the tab for at least some of the damage caused by the products that, in all senses, are putting a massive strain on America.

The common argument against the nanny state - "getting fat is just a matter of personal choice" - won't wash, just as it didn't for tobacco or laws that mandated the wearing of seatbelts. The cost to overall public good is simply too high to ignore.

America cannot afford to get fatter. Conservatives love liberty but they also hate the spiralling cost of Medicaid, the government health insurance programme for those on low incomes, which disproportionately picks up the costs of the obesity epidemic. And right now, obesity rates are predicted to double in the US by 2030.

And this won't happen by chance. Lifestyles are more sedentary now, but that fact on its own cannot explain why childhood obesity rates have shot up so fast. Only the relentless pushing of sugar to children by food companies explains that.

Go to a child's sporting event in America and it is astonishing how many children come waddling off the baseball diamond or the soccer pitch and immediately guzzle on a bottle of Gatorade or similar. And even worse, their parents seem oblivious to the self-destructive folly of it all.

That is why, far-fetched as it seems today, it is also possible to envisage 20 years from now restrictions being placed on the way sweets and chocolates are advertised to children, just as tobacco adverts are banned on television and at sporting events today.

Because in truth there is no escape from the marketing machine: one recent US study of walk-to-school children found they were healthier than those who were driven to school, but they were also fatter.

Baffled scientists only explained that paradox when they discovered that the children who walked went past the local store where they picked up absurdly cheap sodas and candies en route. The sugar-pushers are everywhere.

Sugary drinks are particularly pernicious since you can drink a lot of them without spoiling your appetite - one study found that a **fizzy drink** consumed before a meal reduced a child's appetite by just 9 per cent, compared with 63 per cent if the equivalent sugar was eaten in a solid form like a candy bar.

High fructose corn syrup and alcohol are the two forms of calories that are metabolised in the liver, which is why children that drink a lot of **fizzy drinks** and are obese are at risk of fatty liver disease just like alcoholics are.

The fact is America did not get fat by accident, but by the multi-billion dollar design of an industry that until now has done everything in its power to avoid paying its fair share of the costs imposed on society by its products.

How long before the public and government decide the polluter must pay? If the Berkeley precedent holds, we could look back on November 5 2014 as the moment when, on the contentious issue of sugar taxes, the fat lady finally started to sing.

LOAD-DATE: November 15, 2014

LANGUAGE: ENGLISH

PUBLICATION-TYPE: Newspaper; Web Publication

JOURNAL-CODE: WEBDTNS

Copyright 2014 Telegraph Media Group Limited
All Rights Reserved

FT
FINANCIAL TIMES
Financial Times (London, England)

November 14, 2014 Friday

Spread of city life destroys myth of western illness

BYLINE: Andrew Ward

SECTION: FT REPORT - FT HEALTH: COMBATING DIABETES; Pg. 1

LENGTH: 953 words

HIGHLIGHT: Obesity as a result of changed lifestyles is helping to generate a global epidemic, reports Andrew Ward

Which country has the most diabetics? The US seems the obvious candidate given its high rate of obesity - the main cause of the disease.

In fact, the answer is China, with 96.3m, according to the latest estimates from the International Diabetes Federation (IDF) published today. Next up is India's 66.8m, followed by the US with 25.8m.

Of course, these numbers are in large part simply a function of size. But they give the lie to an oft-repeated myth that diabetes is a mainly western phenomenon. More than three-quarters of the 387m diabetics worldwide live in low and middle income countries, according to the IDF's 2014 Diabetes Atlas.

This proportion will only rise as the developing world increasingly adopts the junk food culture and more sedentary lifestyles that have fuelled the diabetes epidemic in the US and Europe.

Lars Sorensen, chief executive of Novo Nordisk, the world's biggest maker of insulin for diabetics, says one of the chief culprits is the global trend towards urbanisation.

"When people move to cities . . . we see a tremendous rise in diabetes because of long commutes, access to junk food, stressful lives [and] little possibility for exercise . . . So it's a huge problem for emerging markets."

Between now and 2035, China is forecast by the IDF to see its number of diabetics rise by almost half to 142.6m, while India's will climb by nearly two-thirds to 109m. But the biggest increase is expected in Africa, where cases are projected to rise by 92 per cent to 41.5m.

For developing world governments, this threatens to impose a heavy burden on still nascent public health systems. If left untreated, as is still often the case in many countries, diabetes causes damage to the heart, blood vessels, eyes, kidneys and nerves. Strokes, blindness, amputations and kidney failure are among the potential consequences of a disease that robs the body of its ability to control blood sugar.

Whether through the cost of treating these complications or by widening access to the medicines that can prevent them, the economic burden will be great. At an estimated $612bn this year, spending on diabetes-related treatments already accounts for 11 per cent of total global healthcare costs, the IDF says. Yet, more than 80 per cent of this money is used to treat 17 per cent of diabetes sufferers - those in the richest countries.

A study published last month by the US Centers for Disease Control and Prevention suggested that, after more than doubling between 1990 and 2008, the growth of diabetes in the US may be starting to slow. This could signal that rising public awareness of the health risks from obesity and high sugar intake is starting to change behaviour. The rate of obesity in the US has plateaued at about a third of adults for a decade.

But even if lifestyles do become healthier - still a very big if - the incidence of diabetes will keep rising in the US and Europe because people are living longer and old age is itself a risk factor. The IDF projects a rise of about 30 per cent in both regions between now and 2035. Globally, the number of diabetics is forecast to rise by just over half during that period to 591.9m.

Petra Wilson, IDF chief executive, wants the G20 group of leading economies to help craft a co-ordinated global response. "It is not just a problem for health ministers," she says. "It's for finance ministers too. If we're going to meet this challenge it will take a comprehensive effort across all areas of government and policy."

Many health advocates believe public education is not enough. They point to Mexico, which last year introduced a "fat tax" on fast food and **sugary drinks.** Berkeley this month became the first US community to impose a 1-cent-an-ounce levy on **soft drinks.**

High sugar consumption has been strongly linked with type 2 diabetes - the largely preventable form of the disease which occurs when the pancreas does not produce enough insulin or the body has stopped responding to it. This accounts for 90 per cent of global cases, with the rest involving type 1, which develops most often among young people and causes the destruction of insulin-producing cells in the pancreas.

Novo Nordisk and rival drugmakers such as Sanofi and Eli Lilly are developing more advanced treatments, including once-weekly injections and oral insulins, while medical device makers are working on new high-tech glucose monitoring devices and insulin pumps. All these could improve the lives of patients and increase compliance with treatment regimes.

David Cavan, IDF policy director, says the top priority must be prevention. "Science has come a long way in developing treatments to control the disease. Where we haven't moved as far forward is in changing lifestyles and getting the political and public health communities to take this challenge seriously enough."

Drug companies are often accused of riding the epidemic rather than trying to stop it. But the industry is increasingly recognising it has an interest in helping societies keep the cost of diabetes sustainable. Novo Nordisk, for example, has launched a partnership with Mexico City's government to develop policies to combat diabetes and is extending the scheme to other big cities around the world.

Mads Krogsgaard Thomsen, chief science officer at Novo Nordisk, says talks with Chinese leaders have reassured him that the problem is being taken seriously. But he worries whether US and European governments are capable of a sufficiently long-term approach.

"Politicians are rarely thinking about how they can save money 20 years from now by reducing kidney failure. They are thinking about what money they have to spend on schools next year. This is a very big dilemma that policy makers need to tackle."

LOAD-DATE: November 13, 2014

LANGUAGE: ENGLISH

PUBLICATION-TYPE: Newspaper

Copyright 2014 The Financial Times Ltd.
All Rights Reserved
Please do not cut and paste FT articles and redistribute by email or post to the web.

Financial Times (London, England)

November 14, 2014 Friday

Reliable facts in short supply for advice on what to eat; Diet

BYLINE: Scheherazade Daneshkhu

SECTION: FT REPORT - FT HEALTH: COMBATING DIABETES; Pg. 2

LENGTH: 753 words

HIGHLIGHT: As obesity rates have risen faster than expected so has incidence of the disease, says Scheherazade Daneshkhu

Almost every day, some new piece of dietary advice is issued to the public - usually implausible enough to grab the attention. Diabetes sufferers will have been told that grapefruit could protect against the illness; that dairy foods lower the risk of diabetes; and that zero-calorie sweeteners increase it.

Yet, as with similar claims about food and cancer , the relationship between illness and diet is hard to pin down. One reason is the lack of robust long-term experiments, as well as the complexity of diets and chronic illnesses.

However, nutritionists and scientists agree on one thing when it comes to food and diabetes: overeating leads to weight gain and overweight people are more likely to develop type 2 diabetes than lean people.

In addition, some people have a genetic disposition that leads them to develop diabetes without necessarily being overweight.

Dr Gojka Roglic, technical officer of the World Health Organisation's diabetes programme, says: "Obesity is the strongest risk factor for type 2 diabetes. Not all obese people develop type 2, only those that have an innate genetic susceptibility - but even they are less likely to get it if they are not overweight."

Obesity rates worldwide have climbed far more rapidly than expected - by 67 per cent over the past decade to 500m people, which in turn has led to rates of diabetes escalating far beyond the Geneva-based WHO's forecasts.

Why does being overweight trigger the risk of getting diabetes?

Broadly-speaking, excess weight is stored as fat under the skin. But once these safe fat stores are exhausted, the fat spills over into organs such as the liver and round the pancreas, interfering with the pancreas's production of insulin and the liver's function in regulating sugar levels. This results in higher blood sugar levels, which can lead to type 2 diabetes.

As long as people do not overeat, whether what they eat makes a difference in terms of the risk of developing type 2 diabetes, is a matter for debate.

Recent controversy has swirled around high fructose corn syrup (HFCS) - a byproduct of corn used to sweeten processed foods and drinks.

One study published two years ago by Oxford university and the University of Southern California, suggested that HFCS consumption could increase the risk of diabetes. Basing its evidence on countries such as the US, which has the highest consumption of HFCS, the research found the prevalence of diabetes in such nations was 20 per cent higher. "This study doesn't prove that HFCS causes diabetes," warned Diabetes UK, a charity that funds research. "For example, it does not show that individuals with diabetes consumed higher levels of HFCS or that this consumption was the key factor leading to their condition."

At the same time, the International Journal of Obesity published a study - part-funded by the Corn Refiners Association - suggesting there was no evidence to pin the US obesity epidemic on HFCS.

Stephanie Dunbar, director of nutrition at the American Diabetes Association, says: "Our position is that people with, and at risk of diabetes, should limit or avoid intake of **sugar**-sweetened **beverages** (from any caloric sweetener including high-fructose corn syrup and sucrose) to reduce the risk for weight gain and worsening of cardiometabolic risk profile."

Clearly, further research is needed, which is why Diabetes UK commissioned more work to assess the link between fructose and type 2.

The research, carried out by Loranne Agius, professor of metabolic biochemistry at Newcastle University, shows that dietary fructose is a much more powerful agent than glucose in causing fatty liver and changes in liver function, including the insulin resistance that increases the risk of developing type 2 diabetes.

Since the starch in carbohydrate breaks down into glucose in the body, she concludes there is no evidence from long-term clinical studies to support the assumption that low-fat, high-carbohydrate diets are healthier than ones low in carbohydrate and high in fat. This view has contributed to a rise in consumption of pasta, rice, and potatoes.

In the meantime, experts see great scope for preventive measures: from labelling foods to taxation.

The World Health Organisation cites studies showing that in China, increases in the price of unhealthy foods reduced intake, while in the US, cheaper healthy foods led to a 68 per cent rise in consumption.

The WHO adds that exercise plays an important role: moderate exercise of 150 minutes a week cuts the risk of diabetes by 27 per cent.

LOAD-DATE: November 13, 2014

LANGUAGE: ENGLISH

PUBLICATION-TYPE: Newspaper

Copyright 2014 The Financial Times Ltd.
All Rights Reserved
Please do not cut and paste FT articles and redistribute by email or post to the web.

FINANCIAL TIMES
Financial Times (London, England)

November 14, 2014 Friday

A tax on sugary drinks helps - a little; Case study: Mexico

BYLINE: Jude Webber

SECTION: FT REPORT - FT HEALTH: COMBATING DIABETES; Pg. 4

LENGTH: 361 words

HIGHLIGHT: Children are getting the pathology for the disease 30 years early, reports Jude Webber

Soon after Mexico approved a tax on sugary fizzy drinks and junk food last year to fight an obesity epidemic, Miguel Barbosa, a leftist senator, slipped into a diabetic coma. He later admitted that it was his failure to manage his illness that caused an infection that put his life in danger for two weeks and led to the amputation of his foot.

When it comes to diabetes, Mexico is a ticking time bomb. Seven out of 10 adults and a third of children are obese or overweight - a principal cause of type 2 diabetes, which has risen sharply in Latin America's second-largest economy - and 9.2 per cent of Mexicans have been given a preliminary diagnosis of diabetes. But the Mexican Diabetes Federation warns that the number of sufferers could in fact be twice as high.

"Diabetes used to be something we only saw in adults, but now we're starting to see it in children too. It's very worrying. These children are getting this pathology 30 years early," says Valeria Szymanski, a nutritionist at the Mexican Association of Diabetes.

"Right now, not everyone who is overweight has diabetes, but they very probably will develop it. It's a huge problem - the health system could collapse," she says.

Is the 10 per cent tax on fizzy drinks, plus another levy on junk food, which have been in place since the start of this year, working?

Mexicans guzzled some 163 litres of fizzy drinks per head a year before the tax and frittered away 24 times more on junk food than on the 10 most important basic foodstuffs.

Fernando Zárate, a leftist legislator who has been pushing in vain for the soda tax to be doubled, says a study by the National Institute of Public Health showed that consumption of taxed sugary drinks had fallen 10 per cent while consumption of untaxed beverages such as water and milk had risen 7 per cent.

Mr Zárate says a 20 per cent tax on **fizzy drinks** would have saved 13bn pesos in the medical cost of treating conditions directly related to being overweight or suffering diabetes in the next decade, and help prevent as many as 1.3m cases of diabetes by 2030.

At the current 10 per cent soda tax rate, the number of cases prevented is 400,000 to 630,000, he estimates.

LOAD-DATE: November 13, 2014

LANGUAGE: ENGLISH

PUBLICATION-TYPE: Newspaper

Copyright 2014 The Financial Times Ltd.
All Rights Reserved
Please do not cut and paste FT articles and redistribute by email or post to the web.

DAILY MAIL (London)

November 11, 2014 Tuesday

THE FRUIT SMOOTHIES FOR CHILDREN WITH MORE SUGAR THAN COCA-COLA

BYLINE: BY SEAN POULTER CONSUMER AFFAIRS EDITOR

LENGTH: 356 words

FRUIT smoothies and juices sold as healthy drinks for children can contain 60 per cent more sugar than Coca-Cola, a survey has found.

One leading cardiologist said the drinks are so unhealthy that they should be seen as a treat rather than a normal part of a daily diet.

A 200ml serving of Coca-Cola contains the equivalent of five teaspoons of sugar. The same amount of some fruit smoothies can contain up to eight.

These include Tesco's Goodness Slurper smoothies for kids in apple and banana flavour, and Ella's Kitchen's The Yellow One Squished Smoothie Fruits, which contains apple, banana, mango and apricots.

The products are sold in servings smaller than 200ml, so the sugar hit is reduced. However, health campaigners say the survey results give a good indication of just how much sugar they contain. The research by Action on Sugar found that more than a quarter of drinks - 57 of the 203 - contain at least as much sugar as Coca-Cola.

And a quarter of all the drinks contained sugar or glucose-fructose syrup as an added ingredient. The campaigning group warned that high-**sugar drinks** are fuelling obesity.

At the same time tooth decay is the most common reason for children in England being admitted to hospital.

When a whole fruit is processed into a drink, the sugars in the cell walls are released as free sugars' which damage the teeth, while the absence of fibre means you take in more calories.

Chairman of Action on Sugar, Professor Graham MacGregor, said: It is a complete scandal that these drinks are marketed to children and parents as if they are "healthy". This has to stop.'

Dr Aseem Malhotra, a cardiologist and science director of the group, added: Fruit juice and smoothies should not be part of a healthy balanced diet.

There is increasing scientific evidence that regular **sugary drink** consumption is associated with an increased risk of type 2 diabetes and heart disease, independent of body weight.'

Gavin Partington, of the British Soft Drinks Association, said: It is unfortunate this survey omits to mention the established health benefits of fruit juice, such as vitamin C.'

s.poulter@dailymail.co.uk

© Daily Mail

LOAD-DATE: November 10, 2014

LANGUAGE: ENGLISH

PUBLICATION-TYPE: Papers

Copyright 2014 Associated Newspapers Ltd.
All Rights Reserved

DAILY MAIL (London)

November 11, 2014 Tuesday

20 RULES THAT WILL HELP STOP YOUR CHILDREN BEING OBESE

BYLINE: BY SOPHIE BORLAND HEALTH CORRESPONDENT

LENGTH: 167 words

childhood obesity can be cured by getting youngsters to follow a set of up to 20 basic rules, a doctor claims.

For instance, they must walk or bike to school, while snacks and sweets, fruit juice and **fizzy drinks** are rationed to once a week.

Children are banned from having second helpings at dinner, can watch television for only two hours a day - with none before 5pm - and are not allowed white bread for lunch.

The rules have been drawn up by Danish paediatrician Dr Jens Christian Holm, who claims to have helped 1,300 overweight and obese children. He says they have been so successful across Denmark that other countries such as Britain should follow suit.

Dr Holm's plan hinges on sticking to between 15 to 20 rules - from a list of about 90 - individually tailored to a child to improve their diet, boost exercise levels and slash time spent sitting.

NHS figures due next month are expected to show that one UK child in three is overweight or obese by the time they leave primary school.

© Daily Mail

LOAD-DATE: November 10, 2014

LANGUAGE: ENGLISH

PUBLICATION-TYPE: Papers

Copyright 2014 Associated Newspapers Ltd.
All Rights Reserved

Daily Mirror

November 11, 2014 Tuesday
Edition 1;
Scotland

WARNING ON SUGAR IN LUNCHBOX JUICES

BYLINE: RUKI SAYID

SECTION: NEWS; Pg. 22

LENGTH: 176 words

MORE than a quarter of children's fruit juices have more sugar than a glass of Coke, and should carry a red health warning, campaigners said yesterday.

A study of 203 lunchbox juices, including own brands from Tesco, Waitrose and Sainsbury's, found 57 had the equivalent of six teaspoons of sugar - one more than a 200ml glass of Coca Cola.

The "from concentrate" favourites are seen as a healthy alternative to **fizzy drinks**.

But research by Action on Sugar also found 24 of the drinks contained more sugar than twoand-a-half Krispy Kreme donuts.

The health campaign's Prof Graham MacGregor said: "It is a complete scandal these drinks are marketed to children and parents as if they are healthy. We need to stop Britain's childhood obesity epidemic spiralling out of control."

He wants juice makers to cut sugar and the Government to stop recommending a 150ml glass of unsweetened fruit juice as one of the recommended five a day.

Sainsbury's and Waitrose insisted their juices had no "added" **sugar**. Tesco said: "We have cut the number of calories in our **soft drink**

LOAD-DATE: November 11, 2014

LANGUAGE: ENGLISH

GRAPHIC: RESEARCH Prof MacGregor

PUBLICATION-TYPE: Newspaper

JOURNAL-CODE: DMR

Copyright 2014 MGN Ltd.
All Rights Reserved

The Daily Telegraph
The Daily Telegraph (London)

November 11, 2014 Tuesday
Edition 1;
National Edition

The children's fruit juices that are sweeter than Coca-Cola; Parents warned against buying sugary drinks that are marketed as healthy

BYLINE: Sarah Knapton

SECTION: NEWS; Pg. 10

LENGTH: 819 words

WITH bright, child-friendly packaging and reassuring names such as "goodness slurper" and "chosen by kids", supermarket fruit drinks have all the appearance of being healthy and nutritious.

But more than a quarter contain as much sugar as Coca-Cola, and some considerably more, a study found.

Own brands from Waitrose, Tesco, Sainsbury's and Asda are among the worst culprits.

Asda's Chosen by Kids tropical juice drink contains the equivalent of seven teaspoons per 200ml, or 26g, which is over a teaspoon more than "full-fat" Coke. Tesco's Goodness Slurper Apple & Banana Fruit Smoothie Snacks are even worse, with eight teaspoons per 200ml. Essential Waitrose Pure Pineapple Juice contains six teaspoons.

Other sugary fruit drinks include the "Yellow One" smoothie by Ella's Kitchen, with eight teaspoons per 200ml, and the same brand's "Orange One", with seven. Innocent's apple and blackcurrant Smoothie for Kids also has seven. Capri-Sun blackcurrant juice drink and Eager Cloudy Pressed Apple and Mango Juice both contain six.

Although juice contains the natural sugars of fruit, when they are processed the sugars in the cell walls are released as "free sugars", which damage teeth and provide unnecessary calories.

A quarter of drinks surveyed contained added sugar or glucosefructose syrup. The "no-added sugar" version of Asda's Chosen by Kids drink contained less than one spoonful in total, giving some indication of how much sugar is added.

Action on Sugar, which carried out the study, warned parents to avoid fruit juices unless it was with meals. They also advised watering down drinks or giving children water instead.

"Our advice is to eat the fruit, don't drink the juice. Juice should be an occasional treat, not an everyday drink," said Katharine Jenner, campaign director of Action on Sugar. "These processed drinks are laden with sugar and calories and do not have the nutritional benefits of fresh fruit and vegetables."

The World Health Organisation advises that people get a maximum of 10 per cent of total energy from added sugars - about 50g or 10 teaspoons. A study6tsp/per by University College London suggested that this figure should be cut to 14g, or three teaspoons, meaning the daily limit would be reached from half a can of Coke.

Sugary drinks do not just cause tooth decay. One in five children aged between four and five is clinically overweight or obese, which becomes one in three between the ages of 10 and 11.

"There is increasing scientific evidence that regular **sugary drink** consumption is associated with an increased risk of Type-2 diabetes and heart disease, independent of body weight, suggesting we are all vulnerable," said Dr Aseem Malhotra, a cardiologist with Action on Sugar.

The report authors found that some drinks were not so sweet. Innocent 100% Apple Juice for Kids contains 15.6g of sugar per 200ml, about a third less than Morrison's Apple Juice From Concentrate which has 22.8g.

Campaigners surveyed 203 products and found 57 had the same amount of sugar or more than Coke.

Many drinks tested were targeted at parents as healthy options for their children. "It is a complete scandal that these drinks are marketed to children and parents as if they are healthy," said Prof Graham MacGregor of the Wolfson Institute, Queen Mary University of London. "We need to stop Britain's childhood obesity epidemic spiralling out of control."

Current UK guidelines state that a small (150ml) glass of unsweetened 100 per cent fruit juice can count as one of your "Five a Day", but Action on Sugar said this advice should be withdrawn.

Kawther Hashem, a nutritionist, added: "What is more concerning are the products with added sugar and glucose-fructose syrup. We call on all manufacturers to stop adding more sugars to already sweet juices, particularly in children's products."

A spokesman for Tesco said: "We have cut the number of calories in our soft drink ranges by over three billion and we will be the first major retailer to remove sweets and chocolate from checkouts across all stores."

A Sainsbury's spokesman said: "We are unhappy with these comparisons as our smoothies come in 90ml pouches and the data refer to a 200ml portion. Juices and smoothies are naturally sweet and we've done a lot of work to make our labelling clear and to reduce sugar levels in our ranges."

Asda said: "Our customers wouldn't be surprised to learn that fruit juice contains natural sugars. Our product is naturally sweet and has no added sugar."

Fiona Hunter, an independent nutritionist and member of the British Dietetic Association, said: "Most nutritionists and dietitians agree that juice can be part of a varied, healthy diet for the whole family. 100 per cent pure fruit juice is nutrient rich, so whilst it contains only natural sugar from the fruit, it also adds real nutritional value to the diet with vitamins and minerals."

Detailed list: www.telegraph.co.uk/news

LOAD-DATE: November 11, 2014

LANGUAGE: ENGLISH

GRAPHIC: 203 the more It to theyInnocent Smoothies for Kids 7tsp/28.4g sugar per 200mlMorrisons pineapple juice 6tsp/25.2g sugar per 200mlEager apple and mango juice 6tsp/24g sugar per 200mlElla's Kitchen: the Orange One smoothie 7tsp/28.4g sugar per 200mlEssential Waitrose pineapple juice 6tsp/23.8g sugar per 200ml

PUBLICATION-TYPE: Newspaper

JOURNAL-CODE: DTL

Copyright 2014 Telegraph Media Group Limited
All Rights Reserved

i-Independent Print Ltd

November 11, 2014
First Edition

Fruit juices 'have dangerous levels of sugar'; HEALTH

BYLINE: Katie Grant

SECTION: NEWS; Pg. 23

LENGTH: 515 words

Fruit juices for children marketed as being healthy can contain as much sugar as a glass of Coca-Cola, according to a new study.

An analysis of over 200 juices, smoothies and fruit drinks by the health group Action on **Sugar** revealed more than a quarter of the **beverages** tested contained at least as much sugar as a glass of Coca-Cola, with up to six teaspoons per 250ml glass.

Research carried out by the campaign group found that the worst offenders included Tesco Goodness Slurper Apple & Banana Fruit Smoothie Snack for Kids, which contains 16.1g of sugar per 100ml

Also on the list was Asda's Chosen by Kids Tropical Juice From Concentrate, which contains 13g of sugar per 100ml.

The survey looked specifically at juices that were aimed at children or marketed as lunchbox-friendly.

"It is a complete scandal that these drinks are marketed to children and parents as if they are 'healthy'; this has to stop," said Professor Graham MacGregor, the chairman of Action on Sugar. "We need to stop Britain's childhood obesity epidemic spiralling out of control."

Katharine Jenner, Campaign Director of Action on Sugar, said: "Juice should be an occasional treat, not an 'everyday' drink."

And cardiologist Dr Aseem Malhotra said: "Fruit juice and smoothies should not be part of a healthy balanced diet."

Action on Sugar has called for manufacturers to reduce the amount of sugar they add to their products, and for the Government to withdraw its advice that a small glass of ruit juice can count towards fruit and vegetable intake recommendations.

But the British Soft Drinks Association argued fruit juice consumption in the UK equated to an average of just 45ml per person per day - accounting for just one per cent of the calories in the average British diet.

"Fruit juice is a useful contribution towards our five-a-day," said Public Health England's chief nutritionist Alison Tedstone.

However, Dr Tedstone added: "Because the process of juicing releases sugars from the fruit we recommend that you try to limit your fruit juice to 150mls a day, including that from smoothies."

Dr Malhotra warned that there is increasing evidence that regular **sugary drink** consumption could be associated with an increased risk of type 2 diabetes and heart disease

High-sugar horrors

Juice: Asda Chosen By Kids Tropical Juice From Concentrate Sugars (g) per 200 ml serving: 26.0
Teaspoons sugar per 200ml: 7

Juice: Morrisons Pineapple Juice Sugars (g) per 200 ml serving: 25.2 Teaspoons sugar per 200ml: 6

Juice: Eager Cloudy Pressed Apple & Mango Juice Sugars (g) per 200 ml serving: 24 Teaspoons sugar per 200ml serving: 6

Juice: Essential Waitrose Pure Pineapple Juice (left) Sugars (g) per 200 ml serving: 23.8 Teaspoons sugar per 200ml serving: 6

Juice: Eager 100% Cloudy Pressed Pineapple Juice (left) Sugars (g) per 200 ml serving: 23 Teaspoons sugar per 200ml serving: 6

Juice: Morrisons Apple Juice From Concentrate (right) Sugars (g) per 200 ml serving: 22.8 Teaspoons sugar per 200ml serving: 6

Juice: Calypso Juice Shots 100% Pure Apple Juice Sugars (g) per 200 ml serving: 22.8 Teaspoons sugar per 200ml serving: 6

LOAD-DATE: November 10, 2014

LANGUAGE: ENGLISH

PUBLICATION-TYPE: Newspaper

JOURNAL-CODE: III

Copyright 2014 Independent Print Limited
All Rights Reserved

The INDEPENDENT
Independent.co.uk

November 11, 2014 Tuesday 9:01 AM GMT

'Healthy' fruit juices have dangerous levels of sugar, study shows; A quarter of juices tested had as much sugar as Coca-Cola

BYLINE: Katie Grant

SECTION: FRONTPAGE

LENGTH: 343 words

Fruit juices marketed at children as being healthy can contain as much sugar as a glass of Coca-Cola, according to a new study.

An analysis of over 200 juices, smoothies and fruit drinks by the health group Action on **Sugar** revealed more than a quarter of the **beverages** tested contained at least as much sugar as a glass of Coca-Cola, with up to six teaspoons per 250ml glass.

Research carried out by the campaign group found that the worst offenders included Asda's Chosen by Kids Tropical Juice From Concentrate, which contains 13g of sugar per 100ml, and Tesco Goodness Slurper Apple & Banana Fruit Smoothie Snack for Kids, which contains 16.1g of sugar per 100ml.

The survey looked specifically at juices that were aimed at children or marketed as lunchbox-friendly. "It is a complete scandal that these drinks are marketed to children and parents as if they are 'healthy'; this has to stop," said Professor Graham MacGregor, chairman of Action on Sugar. In pictures: 10 food and drinks with hidden sugar highs

"We need to stop Britain's childhood obesity epidemic spiralling out of control," Professor MacGregor added.

Cardiologist Aseem Malhotra warned that in addition to tooth decay, there is increasing scientific evidence that regular **sugary drink** consumption could be associated with an increased risk of type 2 diabetes and heart disease. "Fruit juice and smoothies should not be part of a healthy balanced diet," he said.

Action on Sugar has called for manufacturers to reduce the amount of sugar they add to their products, and for the Government to withdraw its advice that a small glass of unsweetened fruit juice can count towards fruit and vegetable intake recommendations.

But the British Soft Drinks Association argued fruit juice consumption in the UK equated to an average of just 45ml per day per person - accounting for just one per cent of the calories in the average British diet.

"Fruit juice is a useful contribution towards our five-a-day," said Public Health England's chief nutritionist Alison Tedstone. But she recommended just 150ml a day.

LOAD-DATE: November 11, 2014

LANGUAGE: ENGLISH

PUBLICATION-TYPE: Newspaper; Web Publication

JOURNAL-CODE: WEBI

Copyright 2014 Independent Digital News and Media Limited
All Rights Reserved

MailOnline

MailOnline

November 11, 2014 Tuesday 2:50 AM GMT

The fruit smoothies for children with more sugar than Coca-Cola: drinks sold as healthy can contain up to 60% more

BYLINE: SEAN POULTER, CONSUMER AFFAIRS EDITOR

SECTION: HEALTH

LENGTH: 399 words

- A 200ml serving of Coca-Cola contains equivalent of five teaspoons of sugar
- But the same amount of some fruit smoothies can contain up to eight
- Research found more than a quarter of drinks contain as much sugar as cola
- Expert says smoothies and juices should be a treat - not part of a daily diet

Fruit smoothies and juices sold as healthy drinks for children can contain 60 per cent more sugar than Coca-Cola, a survey has found.

One leading cardiologist said the drinks are so unhealthy that they should be seen as a treat rather than a normal part of a daily diet.

A 200ml serving of Coca-Cola contains the equivalent of five teaspoons of sugar. The same amount of some fruit smoothies can contain up to eight.

These include Tesco's Goodness Slurper smoothies for kids in apple and banana flavour, and Ella's Kitchen's The Yellow One Squished Smoothie Fruits, which contains apple, banana, mango and apricots.

The products are sold in servings smaller than 200ml, so the sugar hit is reduced.

However, health campaigners say the survey results give a good indication of just how much sugar they contain.

The research by Action on Sugar found that more than a quarter of drinks - 57 of the 203 - contain at least as much sugar as Coca-Cola.

And a quarter of all the drinks contained sugar or glucose-fructose syrup as an added ingredient. The campaigning group warned that high-**sugar drinks** are fuelling obesity.

At the same time tooth decay is the most common reason for children in England being admitted to hospital.

When a whole fruit is processed into a drink, the sugars in the cell walls are released as 'free sugars' which damage the teeth, while the absence of fibre means you take in more calories.

Chairman of Action on Sugar, Professor Graham MacGregor, said: 'It is a complete scandal that these drinks are marketed to children and parents as if they are "healthy". This has to stop.'

Dr Aseem Malhotra, a cardiologist and science director of the group, added: 'Fruit juice and smoothies should not be part of a healthy, balanced diet.

'There is increasing scientific evidence that regular **sugary drink** consumption is associated with an increased risk of type 2 diabetes and heart disease, independent of body weight.'

Gavin Partington, of the British Soft Drinks Association, said: 'It is unfortunate this survey omits to mention the established health benefits of fruit juice, such as vitamin C.'

LOAD-DATE: November 11, 2014

LANGUAGE: ENGLISH

PUBLICATION-TYPE: Web Publication

JOURNAL-CODE: WEBDM

Copyright 2014 Associated Newspapers Ltd.
All Rights Reserved

212 of 288 DOCUMENTS

mirror.co.uk

November 10, 2014 Monday 9:20 PM GMT

Quarter of fruit juices and smoothies have MORE sugar than Coca-Cola; Well-meaning parents trying to avoid fizzy pop might instead be packing their children's lunchboxes with drinks that are even more sugary

BYLINE: By Sophie Tarr

SECTION: NEWS,UK NEWS

LENGTH: 528 words

Well-meaning parents who would not dream of giving their children Coca-Cola might instead be packing their lunchboxes with juices that are even more sugary, a health group has warned.

Action on Sugar surveyed more than 200 juices, smoothies and fruit drinks to find that more than a quarter contained the same level of sugar or more as Coca-Cola,which has 10.6g for every 100ml.

Among the worst offenders identified by the Queen Mary University of London-based campaign group were Asda'sChosen by Kids Tropical Juice From Concentrate, which contains 13g of sugar per 100ml, and TescoGoodness Slurper Apple & Banana Fruit Smoothie Snack for Kids, which contains 16.1g of sugar for 100ml.

Action on Sugar nutritionist Kawther Hashem said it was a concerning finding, given rates of childhood obesity and tooth decay.

She said the survey looked specifically at juices that were aimed at children or marketed as lunchbox-friendly.

Fresh fruit juices - which cannot contain additives like extra sugar - tended to do better than fruit drinks or juices made from concentrate, she said.

But she warned parents against seeing the word 'juice' on a label as a green light.

"It wasn't clear-cut, but I do think the ones at the top of the sugar list are usually from concentrate," she said.

She said parents were better off giving their children diluted juice or - better still - water and a piece of fruit.

She also recommended parents steer clear of artificially sweetened drinks, "because you're still encouraging the sweet consumption ... These are young children.

"You're training their taste buds - we would prefer that you give that child a piece of orange than something that tastes like orange".

Action on Sugar has called for manufacturers to reduce the amount of sugar they add to their products, and for the Government to withdraw its advice that a small glass of unsweetened fruit juice can count towards fruit-and-vegetable intake recommendations.

But the British Soft Drinks Association (BSDA) said fruit juice consumption in the UK equated to an average of just 45ml per person per day - accounting for 1% of the calories in the average British diet.

"Given Government figures show that the vast majority of adults and children are not getting their recommended five fruit and veg a day it is unfortunate this survey omits to mention the established health benefits of fruit juice, such as vitamin C," BSDA Director-General Gavin Partington said.

"Then again, one should not be surprised that politically motivated campaigners are prepared to ignore the evidence in pursuit of their goal."

Public Health England's chief nutritionist Alison Tedstone said families needed to be conscious of their sugar intake.

But she did not back calls to rethink the inclusion of juice in the five-a-day guidelines.

"Fruit juice is a useful contribution towards our five a day, however, because the process of juicing releases sugars from the fruit we recommend that you try to limit your fruit juice to 150mls a day, including that from smoothies and only consume these and other **sugary drinks** with meals to reduce the risk of tooth decay," Dr Tedstone said.

LOAD-DATE: November 13, 2014

LANGUAGE: ENGLISH

PUBLICATION-TYPE: Newspaper; Web Publication

JOURNAL-CODE: WEBDMI

Copyright 2014 Trinity Mirror, Plc.
All Rights Reserved

The Telegraph

telegraph.co.uk

November 10, 2014 Monday 11:57 PM GMT

Fruit drinks aimed at children contain more sugar than Coca-Cola; More than quarter of fruit juices, drinks and smoothies aimed at children contain as much sugar as Coca-Cola

BYLINE: By Sarah Knapton Science Editor

SECTION: NEWS

LENGTH: 931 words

With bright, child-friendly packaging and reassuring names like 'goodness slurper' and 'chosen by kids' parents could be forgiven for thinking that supermarket fruit drinks are healthy and nutritious.

But more than quarter of fruit juices, drinks and smoothies aimed at children contain as much sugar as Coca-Cola, and some considerably more.

Own brands from Waitrose, Tesco, Sainsbury's and ASDA were among the worst culprits.

ASDA's 'Chosen by Kids' tropical juice drink had the equivalent of seven teaspoons per 200 ml, which is more than one teaspoon more than full fat Coke. Tesco Goodness Slurper Apple & Banana Fruit Smoothie Snack for kids was even worse, with eight teaspoons per 200 ml.

Essential Waitrose Pure Pineapple Juice contains six teaspoons, as does Capri-Sun blackcurrant juice drink.

Although fruit juice contains natural sugars like fruit, in processing the sugars in the cell walls are released as 'free sugars' which damage teeth further and provide unnecessary calories.

And a quarter of drinks surveyed even contained added sugar or glucose-fructose syrup. The 'no-added sugar' version of ASDA's Chosen by Kids Tropical Juice Drinks contained less than one spoonful, giving some indication of how much is added to the

Health experts from Action on Sugar warned parents to avoid fruit juices altogether unless it was at mealtimes. They also advised watering down drinks or swapping them for plain water.

"Our advice is to eat the fruit, don't drink the juice. Juice should be an occasional treat, not an 'everyday' drink," said Katharine Jenner, Campaign Director of Action on Sugar.

"These processed drinks are laden with sugar and calories and do not have the nutritional benefits of fresh fruit and vegetables."

The World Health Organisation currently advises that people get a maximum of 10 per cent of total energy from added sugars - around 50g or 10 teaspoons. However a recent study by University College London suggested that figure should be slashed to 14g, or just three teaspoons, meaning the daily limit would be reached from just half a can of Coke.

Too much sugar doesn't just lead to tooth decay. It can also cause obesity and Type 2 diabetes.

One in five children aged between four and five are already clinically overweight or obese and one in three between the ages of 10 and 11.

"It is not just tooth decay but there is increasing scientific evidence that regular **sugary drink** consumption is associated with an increased risk of type 2 diabetes and heart disease, independent of body weight, suggesting we are all vulnerable," said Dr Aseem Malhotra, Cardiologist, Action on Sugar.

"Fruit juice and smoothies should not be part of a healthy balanced diet."

The report authors found that drinks did not need to be so sweet. Innocent 100% Apple Juice for Kids contains 15.6g of sugars per 200ml, a third (32%) less than Morrison's Apple Juice From Concentrate which has 22.8g of sugars per 200ml.

Campaigners looked at 2O3 products and found over one-in-four (57) had the same amount or more than Coke. If they used a traffic light system, 117 would have the red warning.

And many are targeted at adults who think they are buying healthy options for their children.

"It is a complete scandal that these drinks are marketed to children and parents as if they are 'healthy': this has to stop," said Professor Graham MacGregor of the Wolfson Institute, Queen Mary University of London.

"We need to stop Britain's childhood obesity epidemic spiralling out of control."

Current UK guidelines state a small (15Oml) glass of unsweetened 1OO per cent fruit juice can count as one of your '5 a Day'. But Action on Sugar said this recommendation should be withdrawn. And only six products are actually sold in 150ml portion size packaging, making it harder for parents to make a healthy choice for their children.

Nutritionist Kawther Hashem said: "It is highly concerning that many parents are still buying fruit juices and juice drinks for their children thinking they are choosing healthy products; children should be given as little juice as possible.

"What is more concerning are the products with added sugar and glucose-fructose syrup. We call on all manufacturers to stop adding more sugars to already sweet juices, particularly in children's products."

A spokesman for Tesco said: "We have cut the number of calories in our soft drink ranges by over three billion and we will be the first major retailer to remove sweets and chocolate from checkouts across all stores.

"We'll continue to take action like this because we know it's what our customers want us to do."

A spokesman for Sainsbury's added: "We are unhappy with these comparisons as our smoothies come in 90ml pouches and the data refer to a 200ml portion. Juices and smoothies are naturally sweet and we've done a lot of work to make our labelling clear and to reduce sugar levels in our ranges."

Asda said:"Our customers wouldn't be surprised to learn that fruit juice contains natural sugars. Our product is naturally sweet and has no added sugar."

Fiona Hunter, independent nutritionist and member of the British Dietetic Association said: "Most nutritionists and dietitians agree that juice can be part of a varied, healthy diet for the whole family. In fact the Government's healthy eating advice, the Eatwell Plate, includes juice as part of a balanced diet.

"100 per cent pure fruit juice is nutrient rich, so whilst it contains only natural sugar from the fruit, it also adds real nutritional value to the diet with vitamins and minerals including vitamin C, potassium and folate."

LOAD-DATE: November 18, 2014

LANGUAGE: ENGLISH

PUBLICATION-TYPE: Newspaper; Web Publication

JOURNAL-CODE: WEBDTNS

Copyright 2014 Telegraph Media Group Limited
All Rights Reserved

The Telegraph
telegraph.co.uk

November 10, 2014 Monday 11:19 AM GMT

Christmas adverts 2014 - which are the best?; Television campaigns from John Lewis, Marks & Spencer and other big brands are now as much a part of Christmas as turkey and baubles. Harry Wallop rounds up the major Christmas 2014 adverts, and gives his verdict

BYLINE: By Harry Wallop

LENGTH: 1736 words

John Lewis

In recent years, John Lewis has kickstarted the Christmas advert frenzy, when all the retailers and big consumer brands begin their assault on consumers' pockets. This year, 2014, is no different, with its advert staring a cute boy Sam, and his best friend, Monty the penguin, being viewed on YouTube more than 4 million times within the first 24 hours.

It ticked many tried-and-tested John Lewis boxes. There is an old song covered by an upcoming pop star. This time, however, the singer is not a breathy young woman, but a breathy young man: Tom Odell, who squeezes every drop of emotion out of the slightly ropey John Lennon offcut, 'Real Love'.

No one speaks, the story is told just through the pictures and the song: "Just like little girls and boys / playing with their little toys / seems that all we really were doing / was waiting for love."

The advert, which cost £1 million to shoot, was filmed mostly in east London.

Verdict: Cute - but can't match the simplicity of 2011, with the boy waiting to give his parents a gift .

Marks and Spencer

Britain's biggest clothes retailer has, for the first time in almost a decade, ditched celebritries from its Christmas advert . It recycles last year's slogan - "Magic and Sparkle" - though this time the magic and sparkle does not refer to Rosie Huntington-Whiteley but instead to two fairies, played by two very attractive models, Claire Chust and Chloé François, who fly around the nightime sky, doing good. This includes turning off children's electronic devices and making it snow outside.

The music is Fly Me to the Moon, sung by Julie London.

The advert will debut in the biggest slot of the week: the first advert break of ITV's X Factor on Saturday, November 8.

The advert was filmed in Prague and London. The company will not say how much it cost to make, but admitted that the lack of celebrities freed up some budget. It is spending this money on a social media campaign, using the hashtag #FollowTheFairies . M&S intends to undertake a "random act of kindness" around the country every day between now and Christmas.

Verdict: We still miss Noémie Lenoir.

Coca-Cola

The **fizzy drinks** company claimed, possibly ambitiously, that one in five people in Britain thought Christmas started with the traditional Coca-Cola truck advert. Call me old-fashioned, but I thought it was the first Sunday in Advent.

But Coca-Cola, to give it credit, has been running Christmas adverts since 1931, when it featured a red-suited Father Christmas. This, famously, helped cement the idea that Santa Claus should be dressed in red, rather than green.

The 2014 advert is remarkably similar to Marks and Spencer -- various people are seen undertaking kind acts: a policeman gives a homeless man a meal, a businessman hands over his umbrella to a mother in the street. The music is Andy Williams singing Make Someone Happy, a guarantee tear-jerker.

Verdict: More tooth-rottingly sugary than a can of Coke, but suprisingly touching and effective.

Boots

The opening shot of the advert is an alarm clock going off at midnight, as Christmas Day turns into Boxing Day. Curious. Various people, all looking tired and a bit fed up, then are shown travelling across the country in the dead of night. Curiouser and curiouser.

All the while, the rather lovely but distinctly downbeat Song For You by Alexi Murdoch is playing. The song is all about aching limbs, sore throats and wanting to cry.

Of course, it turns out these people are all travelling so they can help their mother celebrate a late Christmas. She is a hospital nurse who has spent all of Christmas night on duty. She returns home early on Boxing Day morning to find them all waiting to surprise her. The catchline is: "Because she's special".

Verdict: It perfectly captures the mood of post-recession Britain without being too preachy or sentimental. A gem.

Waitrose

The supermarket has history when it comes to worthy adverts. Let's not forget its 2012 offering with Delia Smith and Heston Blumenthal telling us that instead of spending "money on a fancy television advert" the supermarket was giving money to charity. Pass the organic, hessian sick bucket.

This one is less preachy, but virtue seeps out of every frame. The music is a cover of Dolly Parton's Try, about overcoming adversity. It sung by a "choir" of customers who "donated their voices". The effect is a thousand pink-ribbonned kittens catterwauling into your eardrums.

The story is about a school girl so shy she looks as if she could be suffering from locked-in syndrome. She doesn't want to be involved in the school gingerbread stall. But, who should be there to help: an inspirational teacher? her mother? No, silly, a Waitrose employee. Sorry, a 'partner'. And because partners own the business, they really care about helping. And the girl, after many disasters, makes fantastic biscuits.

The partner in the advert is a real-life Waitrose worker: 25-year-old Adejumoke Sanusi, who works at the Canary Wharf Waitrose.

Verdict: Your heartstrings are tugged, yanked and pulled until they snap. But the music is dire.

Tesco

Britain's biggest supermarket is under severe pressure. Sales are slipping, customers are leaving and it has mislaid £263 million of profits. The Serious Fraud Office is investigating.

Has it come up with an advert to remind us why this behemoth was once one of the world's most admired companies? Er, no.

The music is jolly (Irene Cara's What a Feeling), the voice over is done by Julia Davis (masterful) and there is a zip to the ad. But the story is very underwhelming. It features people getting out their Christmas decorations, including Tesco workers in their hi-viz jackets (ooh, festive). It culminates with hundreds of people gathering in a windswept Tesco carpark to watch a big illumination above the shop-front.

Verdict: As dazzling as a energy-saving 40w light bulb.

Sky

For many, a sofa-bound stupor on Christmas afternoon is a key part of the festive season. And why stick with tired, old BBC One, when you can have the fun and excitement of Sky Movies? Well, that's the clear message of this advert.

It's slick, it's fun and it's clever. A family, and their dog, get sucked into the telly and find themselves turned into Muppets, and then into Spiderman heroes, then into Lego minifigures and finally into animated Frozen characters. Michael Gambon does the voice over -- a classy touch.

Verdict: Makes you smile. And you can't ask much more than that.

Debenhams

In the past, Debenhams has gone for all-out glamour. Its 2012 advert with the model Morven MacSween wearing a red dress in the snow won plaudits. But this year the department store has gone for winsome children. A whole army of them.

The advert features about 30 pyjama-clad tots running around a Debenhams department in the dead of night. Unlike normal children -- who would just find the chocolate and scoff it -- this lot are looking for gifts for their parents and themselves, and casually perusing the Christmas decorations on offer.

The music is Paul McCartney's We All Stand Together, ususally known as The Frog Chorus. If in doubt, cover a track by a former Beatle.

Verdict: The least imaginative of the adverts so far.

Halfords

Yes, I know, hub caps and roof boxes are hardly typical Christmas fare. But the retailer is trying to remind the parents of Britain of their Raleigh Chopper-filled childhoods.

The advert features no adults and edgy music (Zoraide, performed by Cairobi). Indeed, it is a vision of some dystopian future, with hooded youths running riot on their bikes in a 1970s frost-covered housing estate. Their dull post-Christmas lunch lull is rescued by whizzing around on two wheels.

The catchline is: "Does anything beat a bike?"

Verdict: As sparkly as Christmas tree on the curbside in January. But it works.

Lidl

The German discount chain is desperate to remind consumers that as well as being dirt cheap, it also sells some quite nice food. This advert reeks of desperation as much as the supermarket's X-Bolt aftershave .

A large group of consumers are all treated to a luxury Christmas lunch. Lobster? Tick. Succulent turkey? Tick. Fine wines? Tick. They ooh and they aah and speculate that the food must come from Waitrose or M&S. But - shock, horror - they discover at the end it's all from Lidl.

Verdict: Plodding.

Aldi

Lidl's arch rival. And a company known for its witty advertising.

This is not very witty, sadly. Though it does contain a fun dig at how passé onsies are. It also contains a celebrity in the shape of Jools Holland, tinkling away at a piano, which makes the advert feel more like something Iceland would produce.

A variety of different British families are pictured tucking into Christmas lunch around the globe, including a group of sailors at sea. You only find out which supermarket the advert is promoting in the final frame. Everyone is having fun, everyone is shopping at Aldi. Apparently.

Verdict: Glossy, a bit dull, but proof that Aldi is now very much part of the establishment.

Harrods

Harrods always used to be known for its January sale advert, not its Christmas advert. But the shop is trying to keep up with the times.

This advert, using stop-frame animation, is reminiscent of Bagpuss or the 1970s Paddington Bear version. It features a little cloth mouse called Pumpernickel, trying to help Father Christmas.

Though, rather than delivering toys to children, the mouse turns on the light bulbs that adorn the outside of Harrods shop in Knightsbridge. Which seems to miss the point, slightly.

The ad, voiced by Jane Horrocks, will only air in London cinemas (and online), not on the television.

Verdict: Charming in a very understated way.

LOAD-DATE: November 10, 2014

LANGUAGE: ENGLISH

PUBLICATION-TYPE: Newspaper; Web Publication

JOURNAL-CODE: WEBDTNS

DAILY MAIL (London)

November 7, 2014 Friday

US TOWN TO TAX FIZZY DRINKS AND FAT' COFFEES

BYLINE: BY NO BYLINE AVAILABLE

LENGTH: 249 words

VOTERS in California have approved the first US tax on **fizzy drinks** in a watershed moment for public health campaigners.

Residents of Berkeley, a left-wing university town, voted to put a premium on unhealthy beverages by the likes of Coca-Cola, as well as energy drinks and fat-laden coffees from chains such as Starbucks.

Campaigners said anything with extra sugar in it led to increased diabetes and obesity and should be discouraged. They called the battle their Waterloo' and launched a well-organised campaign on social media and at grass-roots level to win 75 per cent of the vote.

The decision has sparked a debate in America where brands such as Coca-Cola have been marketed as part of the fabric of American life for decades.

The Berkeley tax rate is a penny per fluid ounce, meaning a typical can of **fizzy drink**, which has 12oz in it, will go up by 12 cents. In the US, most cans cost $1, meaning the tax will be the equivalent of 12 per cent.

Under the law, drinks distributors have to foot the bill, but they will almost certainly pass it on to consumers.

Berkeley Mayor Tom Bates said: We're saying no to big soda. We're saying that Berkeley and the rest of the country need to pay attention that soda is such a destructive product.'

The drinks industry reportedly spent £1.5?million trying to defeat the measure.

Drinks industry spokesman Chris Gindlesperger said Berkeley doesn't look like mainstream America' and warned other politicians not to follow in the town's footsteps.

© Daily Mail

LOAD-DATE: November 6, 2014

LANGUAGE: ENGLISH

PUBLICATION-TYPE: Papers

Copyright 2014 Associated Newspapers Ltd.
All Rights Reserved

MailOnline

November 7, 2014 Friday 1:23 PM GMT

Rates of bowel cancer in young people expected to soar up to 90% in the next 15 years - with junk food and inactivity to blame

BYLINE: MADLEN DAVIES FOR MAILONLINE

SECTION: HEALTH

LENGTH: 750 words

- Bowel cancer rates in young people expected to rocket by 38% by 2020
- Expected to soar by up to 90% by 2030 in people aged 20 to 34 years old
- Data from 1975 to 2010 shows cases decreased among the elderly
- Bowel cancer is more common in people over 50 so they are screened
- Cases in over 50s will decrease by 23% by 2020 and 41% by 2030
- Previous research has linked disease with junk food and **fizzy drinks**

Soaring numbers of young people will develop bowel cancer within the next 20 years, an alarming study has warned.

While cases in the over 50s have declined, numbers for younger adults aged 20 to 49 are expected to rocket.

Data from 1975 to 2010 showed cases among the elderly - who are screened for the disease - have reduced.

However, rates increased for patients 20 to 49 years old, with the biggest increase of 1.99 per cent in patients aged 20 to 34 years old.

And worryingly, by 2020, the incidence rate of bowel cancer in people aged 20-34 will increase by 37.8 per cent. By 2030, this will be 90 per cent.

But for patients older than 50, it will decrease by 23.2 per cent by 2020 and 41.1 per cent by 2030.

The findings are surprising as until now, bowel cancer - often called colorectal cancer as it starts in the colon or the rectum - has been mainly a disease of the elderly.

In the UK, almost nine out of 10 people with bowel cancer are over 60 years old.

Dr Christina Bailey of the University of Texas, said: 'The increasing incidence of colorectal cancer among young adults is concerning and highlights the need to investigate potential causes and external influences such as lack of screening and behavioural factors.'

Previous research found that snacking on chocolate, biscuits and cakes could increase the risk of the disease - as could drinking **fizzy drinks**.

WHAT ARE THE CAUSES OF BOWEL CANCER?

It's not known exactly what causes bowel cancer, but there are a number of things that can increase the risk. These include:

Age - almost nine in 10 cases of bowel cancer occur in people aged 60 or over

Diet - a diet high in red or processed meats and low in fibre can increase your risk

Weight - bowel cancer is more common in people who are overweight or obese

Exercise - being inactive increases the risk of getting bowel cancer

Alcohol and smoking - a high alcohol intake and smoking may increase your chances of getting bowel cancer

Family history - having a close relative who developed bowel cancer below 50 years of age puts you at a greater lifetime risk of developing the condition

It's already known that red or processed meat, for example bacon and sausages, is linked with bowel cancer.

The three main symptoms are blood in the stools, changes in bowel habit (such as to more frequent, looser stools) and abdominal pain.

The study found from 1998 through 2006, the incidence of bowel cancer declined 3 per cent per year in men and 2.4 per cent in women.

This was mainly because better screening was recommended for all adults over 50 years old.

However screening is not routine for those under 50 - meaning many patients are diagnosed late.

The study analysed age disparities in trends of the cancer in the U.S. from the Surveillance, Epidemiology and End Results (SEER) CRC registry.

This included all patients diagnosed with colon or rectal cancer from 1975 through 2010.

The study results indicate that overall, the rate of diagnosis declined 0.92 per cent between 1975 and 2010.

The most pronounced decline was 1.15 per cent in patients 75 years or older, while the rate for patients 50 to 74 years dropped 0.97 per cent.

Commenting on the findings, Dr Kiran Turaga, of the Medical College of Wisconsin, Milwaukee, said the findings were 'rather unsettling'.

He added: 'Nevertheless, assuming that this increasing incidence of colorectal cancer in young adults is a real phenomenon, it begs the question of why this is occurring and what one should do about it.'

Widely screening people of all ages for the cancer might add significant cost and risk without any benefits to society, he added.

'However, this report should stimulate opportunities for development of better risk-prediction tools that might help us identify these individuals early and initiate better screening/prevention strategies,' he continued.

In the future, cancer experts could diagnose bowel cancer using DNA tests of stools, genomic profiling and mathematical modelling.

The study published online in the Journal of the American Medical Association.

LOAD-DATE: November 7, 2014

LANGUAGE: ENGLISH

PUBLICATION-TYPE: Web Publication

JOURNAL-CODE: WEBDM

Copyright 2014 Associated Newspapers Ltd.

All Rights Reserved

The Telegraph
telegraph.co.uk

November 7, 2014 Friday 3:45 PM GMT

Christmas adverts 2014 - which are the best?; Television campaigns from John Lewis, Marks & Spencer and other big brands are now as much a part of Christmas as turkey and baubles. Harry Wallop rounds up the major Christmas 2014 adverts, and gives his verdict

BYLINE: By Harry Wallop

LENGTH: 1271 words

John Lewis

In recent years, John Lewis has kickstarted the Christmas advert frenzy, when all the retailers and big consumer brands begin their assault on consumers' pockets. This year, 2014, is no different, with its advert staring a cute boy Sam, and his best friend, Monty the penguin, being viewed on YouTube more than 4 million times within the first 24 hours.

It ticked many tried-and-tested John Lewis boxes. There is an old song covered by an upcoming pop star. This time, however, the singer is not a breathy young woman, but a breathy young man: Tom Odell, who squeezes every drop of emotion out of the slightly ropey John Lennon offcut, 'Real Love'.

No one speaks, the story is told just through the pictures and the song: "Just like little girls and boys / playing with their little toys / seems that all we really were doing / was waiting for love."

The advert, which cost £1 million to shoot, was filmed mostly in east London.

Verdict: Cute - but can't match the simplicity of 2011, with the boy waiting to give his parents a gift .

Marks and Spencer

Britain's biggest clothes retailer has, for the first time in almost a decade, ditched celebritries from its Christmas advert . It recycles last year's slogan - "Magic and Sparkle" - though this time the magic and sparkle does not refer to Rosie Huntington-Whiteley but instead to two fairies, played by two very attractive models, Claire Chust and Chloé François, who fly around the nightime sky, doing good. This includes turning off children's electronic devices and making it snow outside.

The music is Fly Me to the Moon, sung by Julie London.

The advert will debut in the biggest slot of the week: the first advert break of ITV's X Factor on Saturday, November 8.

The advert was filmed in Prague and London. The company will not say how much it cost to make, but admitted that the lack of celebrities freed up some budget. It is spending this money on a social media campaign, using the hashtag #FollowTheFairies . M&S intends to undertake a "random act of kindness" around the country every day between now and Christmas.

Verdict: We still miss Noémie Lenoir.

Coca-Cola

The **fizzy drinks** company claimed, possibly ambitiously, that one in five people in Britain thought Christmas started with the traditional Coca-Cola truck advert. Call me old-fashioned, but I thought it was the first Sunday in Advent.

But Coca-Cola, to give it credit, has been running Christmas adverts since 1931, when it featured a red-suited Father Christmas. This, famously, helped cement the idea that Santa Claus should be dressed in red, rather than green.

The 2014 advert is remarkably similar to Marks and Spencer -- various people are seen undertaking kind acts: a policeman gives a homeless man a meal, a businessman hands over his umbrella to a mother in the street. The music is Andy Williams singing Make Someone Happy, a guarantee tear-jerker.

Verdict: More tooth-rottingly sugary than a can of Coke, but suprisingly touching and effective.

Boots

The opening shot of the advert is an alarm clock going off at midnight, as Christmas Day turns into Boxing Day. Curious. Various people, all looking tired and a bit fed up, then are shown travelling across the country in the dead of night. Curiouser and curiouser.

All the while, the rather lovely but distinctly downbeat Song For You by Alexi Murdoch is playing. The song is all about aching limbs, sore throats and wanting to cry.

Of course, it turns out these people are all travelling so they can help their mother celebrate a late Christmas. She is a hospital nurse who has spent all of Christmas night on duty. She returns home early on Boxing Day morning to find them all waiting to surprise her. The catchline is: "Because she's special".

Verdict: It perfectly captures the mood of post-recession Britain without being too preachy or sentimental. A gem.

Debenhams

In the past, Debenhams has gone for all-out glamour. Its 2012 advert with the model Morven MacSween wearing a red dress in the snow won plaudits. But this year the department store has gone for winsome children. A whole army of them.

The advert features about 30 pyjama-clad tots running around a Debenhams department in the dead of night. Unlike normal children -- who would just find the chocolate and scoff it -- this lot are looking for gifts for their parents and themselves, and casually perusing the Christmas decorations on offer.

The music is Paul McCartney's We All Stand Together, ususally known as The Frog Chorus. If in doubt, cover a track by a former Beatle.

Verdict: The least imaginative of the adverts so far.

Halfords

Yes, I know, hub caps and roof boxes are hardly typical Christmas fare. But the retailer is trying to remind the parents of Britain of their Raleigh Chopper-filled childhoods.

The advert features no adults and edgy music (Zoraide, performed by Cairobi). Indeed, it is a vision of some dystopian future, with hooded youths running riot on their bikes in a 1970s frost-covered housing estate. Their dull post-Christmas lunch lull is rescued by whizzing around on two wheels.

The catchline is: "Does anything beat a bike?"

Verdict: As sparkly as Christmas tree on the curbside in January. But it works.

Lidl

The German discount chain is desperate to remind consumers that as well as being dirt cheap, it also sells some quite nice food. This advert reeks of desperation as much as the supermarket's X-Bolt aftershave .

A large group of consumers are all treated to a luxury Christmas lunch. Lobster? Tick. Succulent turkey? Tick. Fine wines? Tick. They ooh and they aah and speculate that the food must come from Waitrose or M&S. But - shock, horror - they discover at the end it's all from Lidl.

Verdict: Plodding.

Aldi

Lidl's arch rival. And a company known for its witty advertising.

This is not very witty, sadly. Though it does contain a fun dig at how passé onsies are. It also contains a celebrity in the shape of Jools Holland, tinkling away at a piano, which makes the advert feel more like something Iceland would produce.

A variety of different British families are pictured tucking into Christmas lunch around the globe, including a group of sailors at sea. You only find out which supermarket the advert is promoting in the final frame. Everyone is having fun, everyone is shopping at Aldi. Apparently.

Verdict: Glossy, a bit dull, but proof that Aldi is now very much part of the establishment.

Harrods

Harrods always used to be known for its January sale advert, not its Christmas advert. But the shop is trying to keep up with the times.

This advert, using stop-frame animation, is reminiscent of Bagpuss or the 1970s Paddington Bear version. It features a little cloth mouse called Pumpernickel, trying to help Father Christmas.

Though, rather than delivering toys to children, the mouse turns on the light bulbs that adorn the outside of Harrods shop in Knightsbridge. Which seems to miss the point, slightly.

The ad, voiced by Jane Horrocks, will only air in London cinemas (and online), not on the television.

Verdict: Charming in a very understated way.

LOAD-DATE: November 7, 2014

LANGUAGE: ENGLISH

PUBLICATION-TYPE: Newspaper; Web Publication

JOURNAL-CODE: WEBDTNS

Copyright 2014 Telegraph Media Group Limited
All Rights Reserved

241 of 288 DOCUMENTS

The Daily Telegraph

The Daily Telegraph (London)

November 6, 2014 Thursday
Edition 1;
Scotland

Europe negotiations? Ill do it my Milky Way;
Sketch David Cameron assured Commons he has 'a plan', but the sweet-talking must end soon

BYLINE: Michael Deacon

SECTION: NEWS; Pg. 16

LENGTH: 481 words

AT Prime Minister's Questions, Keith Vaz - Labour MP for Keith Vaz, chairman of the Keith Vaz select committee and shadow minister for Keith Vaz - told David Cameron to reduce the amount of sugar in the public's diet.

The Prime Minister said Mr Vaz was "absolutely right to raise the importance of this issue", which is an exquisitely polite way of saying no. Then he added: "I gather he also wants me to ban sugar and **fizzy drinks** in No10 Downing Street for 24 hours. I'll try and negotiate that with my children."

Mr Cameron did not set out the negotiation strategy he plans to pursue with his children, but Government sources say it will be much like his negotiation strategy for EU reform. By 2017, the sources reveal, the Prime Minister is hoping to win a series of as yet unspecified concessions from his children, rumoured to include a reduction in their intake of Fanta and Milky Ways. The Prime Minister's children, however, are said to be standing firm, maintaining that free consumption of sugar and **fizzy drinks** is a fundamental principle of childhood.

As a result of this impasse, the Prime Minister's negotiation strategy has come in for criticism, with both Ukip and Labour calling into question his prospects of success. Cynics suspect that, should negotiations with his children fail, Mr Cameron will attempt to fudge the issue by reclassifying cabbage as a form of boiled sweet, thus allowing him to claim that his children have bowed to his demands after all.

"My children have agreed never to touch sugary, toothrotting cabbage again," the Prime Minister is expected to announce. "This is precisely the kind of hard-won, vital reform the British people have been crying out for."

For the moment, however, the EU is seen as the more pressing issue in the Commons, and yesterday Ed Miliband devoted all six of his questions to it. "The Prime Minister is nearly two years into his renegotiation with the European Union," said the Labour leader. "He has to get 27 countries to agree with him. How many has he got so far?"

Mr Cameron seemed unable to put his finger on the exact number, perhaps because it was so large, but he assured the Commons that he had "a plan". This, he said, put him in sharp contrast to Mr Miliband, who had "no plan". Mr Miliband retaliated by squawking that Mr Cameron was a "don't know Prime Minister", a putdown that probably sounded snappier in the prep session.

"John Prescott has said Labour has a problem communicating," retorted Mr Cameron. "When you get a lecture from John Prescott on the English language, you really are in trouble!"

Given that Lord Prescott was not present, I feel it incumbent upon me to defend his honour. Frankfully, to be so irrespective of a former deputy prize milliner was unsusceptible. Let's not mince around the bush: it was below the bolt.

'When you get a lecture from John Prescott on English you're in trouble'

LOAD-DATE: November 6, 2014

LANGUAGE: ENGLISH

PUBLICATION-TYPE: Newspaper

JOURNAL-CODE: DTLscot

Copyright 2014 Telegraph Media Group Limited
All Rights Reserved

The Guardian

November 6, 2014 Thursday 6:46 PM GMT

Could Berkeley's soda tax be a model for tackling obesity across America?; Supporters of 1¢ per ounce tax on sugary drinks say their ballot victory on Tuesday proves that Americans are ready to fight drinks industry lobbying to create a healthier society

BYLINE: Peter Moskowitz in New York

SECTION: US NEWS

LENGTH: 1018 words

When backers of a new soda tax in Berkeley, California, realized their ballot initiative would pass on Tuesday, making the city the first in the nation to tax **sugary beverages**, they were ecstatic.

They say their victory - the initiative won by a three-to-one margin - is proof that Americans are ready to begin regulating the causes of the country's obesity and diabetes epidemics.

"We've been getting congratulations all over the country," said Xavier Morales, one of the lead campaigners for the tax, and the director of the Latino Coalition for a Healthy California. "This is just the beginning."

Activists hope Berkeley can become a model for addressing the influence of soda and other unhealthy foods on American health, in a similar way New York City kicked off an era of steep tax increases for tobacco, reducing sales.

Berkeley's new law will add a 1¢ per ounce tax to soda and most other **beverages** with added **sugar**. That means a 2l bottle of Coca-Cola could cost 64¢ more than it did before the ballot initiative. Assuming a bottle costs around $2, that's about a 30% increase. The revenue from the tax will go to a general fund for the city that advocates say will partially be used for health and health education programs.

Proponents of soda taxes say they're an effective way to dissuade Americans, especially children, from consuming too many **sugary beverages**, and to gently push them to other choices, such as bottled water. Opponents, namely the soda industry, have painted the taxes ineffective at fighting obesity, and as an unfair burden to low-income Americans.

"It's just the lowest-hanging fruit of discriminatory taxes," said Chris Gindlesperger, a spokesman for the American Beverage Association (ABA). "What happened in Berkeley was more about making a political statement than about making public policy."

But a growing body of research seems to support the idea of soda taxes. A 2012 study by researchers at the University of California San Francisco found that if a penny-per-ounce tax was levied nationally, 100,000 cases of heart disease, 8,000 strokes, and 240,000 cases of diabetes could be prevented annually. Over 9% of Americans have diabetes, according to the Centers for Disease Control, and an additional 86 million adult Americans have elevated blood sugar levels that could lead to diabetes.

Mexico passed a soda tax late last year, and there's already evidence there that soda consumption rates are falling. Those statistics, and the overwhelming vote in support of a tax in Berkeley, have some calling it the first sign of a national movement.

"This is a demonstration that when you put the idea to voters, instead of legislators who receive campaign contributions from soda companies, that it can pass," said Tom Farley, the former commissioner of New York City's health department under Mayor Michael Bloomberg and a professor of public health at Hunter College. Farley lobbied New York state legislators for a similar tax in 2010, but the effort failed.

"This idea is now far more likely to pass in other places," he said.

But if that's what proponents of Berkeley's soda tax want, they have a long road ahead. Soda taxes had failed to pass in approximately 30 municipalities before the vote in Berkeley, according to the ABA. On Tuesday, a similar measure in San Francisco garnered a majority of the votes but didn't reach the two-thirds threshold required for passage.

Soda companies have spent big trying to thwart soda taxes, including about $2.4m in Berkeley, according to the Berkeley vs. Big Soda Campaign. That works out to about $30 per voter. Pro-tax campaigners spent about $400,000 in Berkeley according to Morales, and were boosted by several TV ad buys by groups backed by Michael Bloomberg, which could bring their total closer to $1m, several media outlets reported. The ABA spent about $9m to help defeat a similar measure in San Francisco, according to activists from San Francisco's Choose Health SF campaign.

Ads against the tax were plastered all over Berkeley's buses and subway stations, including one huge ad on the floor of the station. In San Francisco, the ABA funded protest groups that took to the city's streets in the days leading up to the election.

The soda industry has tried to paint Berkeley's win as an anomaly caused more by the demographics of the city than any national trend. "Berkeley is not like the rest of the country - it's a largely homogenous, very progressive, wealthy community," Gindlesperger said. "So you've got a small number of people voting for this in a country of 310 million."

Gindlesperger has a point. Berkeley prides itself of its unique brand of quirky progressivism. It's the kind of place that hosts a juggling and unicycle festival every year. Alameda county, which Berkeley is in, voted for Barack Obama by a 4-to-1 margin in the 2012 presidential race. And it's one of the birthplaces of the local food movement, thanks to famed chef Alice Water's restaurant Chez Panisse.

But to activists, Berkeley's liberalness doesn't change the fact that the prospect of the tax passing - and a precedent being set - worried soda companies enough to inspire millions in spending.

"If they thought it was an anomaly, the American Beverage Association wouldn't have spent millions fighting it," said Robert Proctor, a professor of the history of science at Stanford University who has written about tobacco advertising and testified against tobacco companies in front of Congress.

Proctor said the methods being used by soda companies to discredit the soda tax - especially calling it harmful to poor consumers, and portraying Berkeley as a city not in-line with American ideals - are reminiscent of strategies used by tobacco companies when cigarettes began to be heavily regulated in the 1960s.

To proponents of the soda tax, that's a sign the tax is a winning idea. "The beverage industry has done such a great job to make themselves ubiquitous that if we attack them it's like we're attacking apple pie," said Morales. "But we are in touch with America. We just touched an electric third rail. We touched it and we survived."

LOAD-DATE: November 6, 2014

LANGUAGE: ENGLISH

PUBLICATION-TYPE: Newspaper

JOURNAL-CODE: WEBGNS

Copyright 2014 The Guardian, a division of Transcontinental Media Group Inc.
All Rights Reserved

The Telegraph
telegraph.co.uk

November 6, 2014 Thursday 8:23 PM GMT

Six in ten drink just one glass of water a day;
The NHS would rather people drank pure water, but we consume very little, preferring tea, coffee and sugary drinks, research shows

BYLINE: By Dan Hyde Consumer Affairs Editor

SECTION: NEWS

LENGTH: 323 words

Most people drink just one glass of pure water a day despite Government attempts to boost consumption, research has found.

Kantar Worldpanel, a retail analyst, studied the drinking habits of 30,000 people as health officials try to move people away from drinking tea, coffee and **sugary drinks** .

It found less than one per cent drank eight glasses of pure water each day - the amount recommended by the NHS .

Six in ten people drank just one glass tap water or bottled water, two in ten drank two, while one in ten said they drank three.

Giles Quick, director of Kantar Worldpanel, said: "The Government is not saying that you should drink just pure water, but it would prefer people to drink more of it as the amount of fat and sugar we take in from food increases every year."

Water is necessary to avoid dehydration, which leads to headaches, a lack of energy and health issues such as kidney stones.

While the NHS says "all drinks count" as part of its eight a day quota, it encourages people to avoid "sugary, soft and **fizzy" drinks** that are high in calories.

"Water is the healthiest choice for quenching your thirst at any time," the NHS said. "If you don't like the taste of plain water, try sparkling water or add a slice of lemon or lime ... it's important that tea, coffee or other drinks containing caffeine are not your only source of fluid."

The research indicated that Britons drank 12.4 billion glasses of tap water at home every year, compared to 29.4 billion cups of tea and 13.1 billion cups of coffee. Squash, juice and carbonated drinks counted for 16.4 billion glasses each day, Kantar Worldpanel said.

It concluded that fewer than 250,000 people drank eight glass of pure water every day, which left "a lot of work to do".

"We need to raise awareness of the need to drink water, as most people are not counting the amount they consume in the same way as with the five-a-day for fruit and vegetables," Mr Quick said.

LOAD-DATE: November 6, 2014

LANGUAGE: ENGLISH

PUBLICATION-TYPE: Newspaper; Web Publication

JOURNAL-CODE: WEBDTNS

Copyright 2014 Telegraph Media Group Limited

MailOnline

MailOnline

November 3, 2014 Monday 4:12 PM GMT

More than 10,000 people being paid sickness benefits... because they are too FAT to work

BYLINE: IAN DRURY FOR THE DAILY MAIL

SECTION: NEWS

LENGTH: 699 words

- Benefits for overweight illnesses have more than doubled in five years
- Thousands receive up to £138 in Disability Living Allowance for obesity
- Total welfare bill for obesity-related illnesses has hit £54million a year
- Obesity costs the national health service more than £9billion a year

Thousands of people are being paid sickness benefits because they are too fat to work - at a cost to the taxpayer of £54million.

Shocking figures show welfare payments for claimants with obesity-related illnesses have more than doubled in five years - highlighting the crisis blighting Britain.

Almost 12,000 people received Disability Living Allowance last year because they have metabolic disease - the medical term for a combination of obesity, diabetes and high blood pressure.

Scroll down for video

The Department for Work and Pensions statistics show that the number of claimants with the condition has more than doubled from around 5,500 five years ago.

Obesity is also a massive burden on the NHS and costs the health service more than £9billion a year.

Ministers have been accused of failing to take proper action against the food industry to help the public by cutting calories in food and drink, and to help people make healthier choices.

Some DLA claimants have jobs but the vast majority are out of work. Recipients can receive up to £138 a week.

Tam Fry, spokesman for the National Obesity Forum, said: 'Successive governments have made life too easy for too many obese people.

'If the obese have a legitimate cause for their fatness - and there may be medical or genetic reasons - benefits should not be denied to them. But getting long-term benefits simply for over-eating is an insult to society.'

Andy Silvester, from the Taxpayers' Alliance campaign group, said: 'It's crucial that every one of these claims is investigated to ensure it's necessary.'

Julia Manning, from the think tank 2020health, said: 'This obesity crisis in the West is a far worse health catastrophe than Ebola but there has been no government urgent action, even though we see the benefits bill spiralling.'

But Jill Tipping, of the obesity support charity HOOP UK, said: 'Obese people have an addiction to food. It's an illness.'

Nearly one in five British secondary school pupils and a quarter of adults are obese, according to officials figures.

Health experts predict that by 2050 the annual bill for obestity-related illnessed will have risen to £50billion a year, with almost two-thirds of the population obese.

In one extreme case, Paul Mason, 51, who was once known as the fattest man in the world, had to give up his job as a postman when he became too huge to complete his delivery rounds.

Mr Mason, of Ipswich, used to weigh 70 stone but shed a whopping 48 stone after gastric band surgery and now tips the scales at 22 stone

At the height of his weight problems he ate about 20,000 calories a day, weighed 70 stone, and needed a specially-built bungalow provided for him by the council.

When Mr Mason became ill, firefighters had to knock down a wall in his house and use a forklift truck to get him to hospital.

The Government has vowed to cut the welfare bill and began phasing out Disability Living Allowance in April and replacing it with the new Personal Independence Payment.

Ministers also launched a Call to Action campaign in October 2011 in a bid to tackle obesity and reduce the nation's calorie intake by 2020.

The drive has called on the food and drink industry to play a key role in slashing the nation's calorie consumption and encouraging families to eat healthily and enjoy active lifestyles.

But senior doctors and academics want the Government to force food manufacturers to cut **sugar** levels, ensure products are clearly labelled and impose a tax on **soft drinks.**

Public Health England, the Department of Health agency responsible for tackling obesity, has said it would 'consider' the ideas as part of a nine-month consultation process that will eventually report to ministers next spring.

A Department for Work and Pensions spokesman said: 'Just having a metabolic disease in itself is unlikely to qualify for DLA. But they could qualify if they have a physical or mental disability caused or made worse by it.'

LOAD-DATE: November 3, 2014

LANGUAGE: ENGLISH

PUBLICATION-TYPE: Web Publication

JOURNAL-CODE: WEBDM

Copyright 2014 Associated Newspapers Ltd.
All Rights Reserved

MailOnline

November 3, 2014 Monday 12:04 PM GMT

More than 10,000 people being paid sickness benefits... because they are too FAT to work

BYLINE: IAN DRURY FOR THE DAILY MAIL

SECTION: NEWS

LENGTH: 695 words

- Benefits for overweight illnesses have more than doubled in five years
- Thousands receive up to £138 in Disability Living Allowance for obesity
- Total welfare bill for obesity-related illnesses has hit £54million a year
- Obesity costs the national health service more than £9billion a year

Thousands of people are being paid sickness benefits because they are too fat to work - at a cost to the taxpayer of £54million.

Shocking figures show welfare payments for claimants with obesity-related illnesses have more than doubled in five years - highlighting the crisis blighting Britain.

Almost 12,000 people received Disability Living Allowance last year because they have metabolic disease - the medical term for a combination of obesity, diabetes and high blood pressure.

The Department for Work and Pensions statistics show that the number of claimants with the condition has more than doubled from around 5,500 five years ago.

Obesity is also a massive burden on the NHS and costs the health service more than £9billion a year.

Ministers have been accused of failing to take proper action against the food industry to help the public by cutting calories in food and drink, and to help people make healthier choices.

Some DLA claimants have jobs but the vast majority are out of work. Recipients can receive up to £138 a week.

Tam Fry, spokesman for the National Obesity Forum, said: 'Successive governments have made life too easy for too many obese people.

'If the obese have a legitimate cause for their fatness - and there may be medical or genetic reasons - benefits should not be denied to them. But getting long-term benefits simply for over-eating is an insult to society.'

Andy Silvester, from the Taxpayers' Alliance campaign group, said: 'It's crucial that every one of these claims is investigated to ensure it's necessary.'

Julia Manning, from the think tank 2020health, said: 'This obesity crisis in the West is a far worse health catastrophe than Ebola but there has been no government urgent action, even though we see the benefits bill spiralling.'

But Jill Tipping, of the obesity support charity HOOP UK, said: 'Obese people have an addiction to food. It's an illness.'

Nearly one in five British secondary school pupils and a quarter of adults are obese, according to officials figures.

Health experts predict that by 2050 the annual bill for obestity-related illnessed will have risen to £50billion a year, with almost two-thirds of the population obese.

In one extreme case, Paul Mason, 51, who was once known as the fattest man in the world, had to give up his job as a postman when he became too huge to complete his delivery rounds.

Mr Mason, of Ipswich, used to weigh 70 stone but shed a whopping 48 stone after gastric band surgery and now tips the scales at 22 stone

At the height of his weight problems he ate about 20,000 calories a day, weighed 70 stone, and needed a specially-built bungalow provided for him by the council.

When Mr Mason became ill, firefighters had to knock down a wall in his house and use a forklift truck to get him to hospital.

The Government has vowed to cut the welfare bill and began phasing out Disability Living Allowance in April and replacing it with the new Personal Independence Payment.

Ministers also launched a Call to Action campaign in October 2011 in a bid to tackle obesity and reduce the nation's calorie intake by 2020.

The drive has called on the food and drink industry to play a key role in slashing the nation's calorie consumption and encouraging families to eat healthily and enjoy active lifestyles.

But senior doctors and academics want the Government to force food manufacturers to cut **sugar** levels, ensure products are clearly labelled and impose a tax on **soft drinks.**

Public Health England, the Department of Health agency responsible for tackling obesity, has said it would 'consider' the ideas as part of a nine-month consultation process that will eventually report to ministers next spring.

A Department for Work and Pensions spokesman said: 'Just having a metabolic disease in itself is unlikely to qualify for DLA. But they could qualify if they have a physical or mental disability caused or made worse by it.'

LOAD-DATE: November 3, 2014

LANGUAGE: ENGLISH

PUBLICATION-TYPE: Web Publication

JOURNAL-CODE: WEBDM

Copyright 2014 Associated Newspapers Ltd.

All Rights Reserved

MailOnline

November 3, 2014 Monday 12:35 AM GMT

Almost 12,000 deemed too fat to work are given £54m benefits: Number of claimants with obesity-related illnesses more than doubles in five years

BYLINE: IAN DRURY FOR THE DAILY MAIL

SECTION: NEWS

LENGTH: 680 words

- Benefits claimants who are 'too fat to work' more than double in five years
- Almost 12,000 receive disability benefits because of metabolic disease
- Obesity costs the NHS more than £9billion a year

Thousands of people are being paid sickness benefits because they are too fat to work - at a cost to the taxpayer of £54million.

Shocking figures show welfare payments for claimants with obesity-related illnesses have more than doubled in five years - highlighting the crisis blighting Britain.

Almost 12,000 people received Disability Living Allowance last year because they have metabolic disease - the medical term for a combination of obesity, diabetes and high blood pressure.

The Department for Work and Pensions statistics show that the number of claimants with the condition has more than doubled from ariound 5,500 five years ago.

Obesity is also a massive burden on the NHS and costs the health service more than £9billion a year.

Ministers have been accused of failing to take proper action against the food industry to help the public by cutting calories in food and drink, and to help people make healthier choices.

Some DLA claimants have jobs but the vast majority are out of work. Recipients can receive up to £138 a week.

Tam Fry, spokesman for the National Obesity Forum, said: 'Successive governments have made life too easy for too many obese people.

'If the obese have a legitimate cause for their fatness - and there may be medical or genetic reasons - benefits should not be denied to them. But getting long-term benefits simply for over-eating is an insult to society.'

Andy Silvester, from the Taxpayers' Alliance campaign group, said: 'It's crucial that every one of these claims is investigated to ensure it's necessary.'

Julia Manning, from the think tank 2020health, said: 'This obesity crisis in the West is a far worse health catastrophe than Ebola but there has been no government urgent action, even though we see the benefits bill spiralling.'

But Jill Tipping, of the obesity support charity HOOP UK, said: 'Obese people have an addiction to food. It's an illness.'

Nearly one in five British secondary school pupils and a quarter of adults are obese, according to officials figures.

Health experts predict that by 2050 the annual bill for obestity-related illnessed will have risen to £50billion a year, with almost two-thirds of the population obese.

In one extreme case, Paul Mason, 51, who was once known as the fattest man in the world, had to give up his job as a postman when he became too huge to complete his delivery rounds.

Mr Mason, of Ipswich, used to weigh 70 stone but shed a whopping 48 stone after gastric band surgery and now tips the scales at 22 stone

At the height of his weight problems he ate about 20,000 calories a day, weighed 70 stone, and needed a specially-built bungalow provided for him by the council.

When Mr Mason became ill, firefighters had to knock down a wall in his house and use a forklift truck to get him to hospital.

The Government has vowed to cut the welfare bill and began phasing out Disability Living Allowance in April and replacing it with the new Personal Independence Payment.

Ministers also launched a Call to Action campaign in October 2011 in a bid to tackle obesity and reduce the nation's calorie intake by 2020.

The drive has called on the food and drink industry to play a key role in slashing the nation's calorie consumption and encouraging families to eat healthily and enjoy active lifestyles.

But senior doctors and academics want the Government to force food manufacturers to cut **sugar** levels, ensure products are clearly labelled and impose a tax on **soft drinks.**

Public Health England, the Department of Health agency responsible for tackling obesity, has said it would 'consider' the ideas as part of a nine-month consultation process that will eventually report to ministers next spring.

A Department for Work and Pensions spokesman said: 'Just having a metabolic disease in itself is unlikely to qualify for DLA. But they could qualify if they have a physical or mental disability caused or made worse by it.'

LOAD-DATE: November 3, 2014

LANGUAGE: ENGLISH

PUBLICATION-TYPE: Web Publication

JOURNAL-CODE: WEBDM

Copyright 2014 Associated Newspapers Ltd.
All Rights Reserved
Rights Reserved
